# Screening Host Antiviral Proteins under the Enhanced Immune Responses Induced by a Variant Strain of Porcine Epidemic Diarrhea Virus

**DOI:** 10.1128/spectrum.00661-22

**Published:** 2022-06-28

**Authors:** Min Sun, Zeyanqiu Yu, Miao Luo, Bin Li, Zihao Pan, Jiale Ma, Huochun Yao

**Affiliations:** a Institute of Veterinary Medicine, Jiangsu Academy of Agricultural Sciences, Nanjing, China; b College of Veterinary Medicine, Nanjing Agricultural University, Nanjing, China; c OIE Reference Lab for Swine Streptococcosis, Nanjing, China; d Key Laboratory of Animal Bacteriology, Ministry of Agriculture, Nanjing Agricultural University, Nanjing, China; e School of Life Sciences, Jiangsu University, Zhenjiang, China; Thomas Jefferson University

**Keywords:** porcine epidemic diarrhea virus, transcriptomics, immune-enhanced variant, IFN, IFI44, OASL

## Abstract

While discussing the ideal candidates of viral restriction factor, the interferon (IFN) and interferon-stimulated genes (ISGs) could be considered potential targets. However, numerous viruses have evolved multiple strategies to modulate the host innate immune signaling for optimal infection, including the porcine epidemic diarrhea virus (PEDV), a coronavirus spreading widely around the world with high morbidity and mortality in piglets. The immunosuppression mediated by PEDV infection creates an impediment for studying the host-virus interactions and screening the antiviral ISGs. Here, the PEDV variant strain 85-7^C40^ was screened using the continuous passaging, which showed significantly attenuated viral replication compared with its parent on MARC-145 cells. The comparative transcriptome analysis (accession nos. SRR13154018 to SRR13154026) indicated that 85-7^C40^ infection led to enhanced immune response on MARC-145 cells, particularly to the IFN antiviral signaling, which mediated the stronger activation of numerous ISGs. Numerous ISGs activated by 85-7^C40^ showed antiviral effects against the wild-type strain infection, particularly the IFI44 (an ISG upregulated specifically by the 85-7^C40^ infection) and OASL (upregulated higher in 85-7^C40^ than 85-7-infected cells), exhibited powerful antiviral activity. IFI44 promoted the production of RIG-I, while the OASL interacted directly with RIG-I, and then they both activated the phosphorylation of STAT1, indicating that they restricted PEDV replication by positively regulating the type I IFN response. Our results provided insight into the ISGs with antiviral activity against PEDV infection and also expanded our understanding of the innate immune response to PEDV infection, which may promote the development of novel therapeutics.

**IMPORTANCE** Host innate immune responses, particularly interferon (IFN) antiviral signaling, can activate diverse downstream ISGs to exert antiviral effects. However, porcine epidemic diarrhea virus (PEDV) infection has evolved multiple strategies to escape from this immune clearance. The immunosuppression mediated by PEDV infection creates an impediment for studying the host-virus interactions. We screened a PEDV variant strain, 85-7^C40^, which induced enhanced immune responses on MARC-145 cells and thus mediated the stronger activation of numerous ISGs. The laboratory-generated variant might induce inconsistent immune responses with a natural wild-type strain during infection, while numerous ISGs activated by 85-7^C40^ showed antiviral effects against the wild-type strain infection, particularly the IFI44 and OASL, restricted PEDV replication by positively regulating the type I IFN response. These findings were suggestive of the immune-enhanced variant being capable of using as an ideal viral model for screening the efficient antiviral proteins and elucidating the underlying mechanisms between PEDV and host innate immune responses.

## INTRODUCTION

Porcine epidemic diarrhea virus (PEDV), the etiological agent of porcine epidemic diarrhea (PED), induced watery diarrhea and dehydration in piglets, which was first recognized in Belgium in 1977 (1–3). Later, PEDV spread to numerous countries in Europe, Asia, Australia, and America, creating serious economic loss in the swine industry worldwide (4–7). PEDV is a single-strand, positive sense RNA virus from the family Coronaviridae. The genome is organized as 5′-untranslated region (UTR)-open reading frame (ORF) 1-S-ORF3-E-M-N-3′-UTR, and the ORF1 encodes 16 nonstructural proteins (nsp1 to nsp16) in order (3, 8, 9).

Virus infection can trigger an interferon (IFN) synthesis quickly as the pattern-recognition receptors (PRRs) detecting the pathogen-associated molecular patterns (PAMPs) (8, 10). Certainly, PEDV can regulate the host innate immune response, like antagonizing the transcription of the IFN and the IFN-stimulated genes (ISGs), and disrupting the inflammatory response (8, 11). Further research discovers that PEDV encodes several immune antagonist proteins, including the structural proteins and nonstructural proteins, targeting the relevant signaling nodes with special mechanisms (8, 11–13). Presently, it has been confirmed that genetic variations occur frequently in the clinical evolution and the cell cultural process of PEDV, which influences the viral pathogenicity and replication during infection (5, 14–16). Nonetheless, there is very little information to indicate its potential relevance to the host immune response.

The type I (IFN-α and IFN-β) and type III (IFN-λ1 and IFN-λ3) IFNs can activate diverse downstream ISGs to exert antiviral effect in inhibiting the PEDV replication (17–20). At present, hundreds of ISGs have been identified, and most of them display direct antiviral functions, while several others have been confirmed to play important roles by providing positive or negative feedback to the IFN antiviral response (21). Mostly, antiviral ISGs inhibit several specific steps of the life cycle adopted by the virus within the infected cells, whereas some other ISGs were inhibitory only against the special virus or only in specific cell types (22, 23). The oligoadenylate synthetase (OAS) family is a set of ISGs, with the 2′–5′-linked oligoadenylates (2-5A) catalysis domain, or tandem ubiquitin-like domains (24). These proteins have illustrated powerful antiviral activities to several viruses by activating the RNaseL or enhancing RIG-I-mediated signaling and others (25, 26), while their antiviral effects could be evaded by diverse viruses (27). IFI44 is a type I IFN-inducible protein; it is involved in virus infection and then elicits distinct regulatory effects on virus replication (28–30). Previous studies have indicated that IFI44 is a candidate of human immunodeficiency virus type 1 (HIV-1) restriction factor and also reduces the replication of the Bunyamwera orthobunyavirus (BUNV) (31) and respiratory syncytial virus (RSV) (32), while it is reported to negatively modulate the IFN responses, and its silencing decreased the replication of the influenza A virus (IAV) and the lymphocytic choriomeningitis virus (LCMV) (33). To date, several ISGs have been identified that associate with PEDV infection (34), while the regulatory networks of more ISGs still need to be further explored.

PEDV infection antagonizes the host innate immune responses (such as the IFN antiviral response) effectively (8, 11–13), which creates an impediment for screening the antiviral proteins and then exploring the underlying mechanism. The supplementation of the IFN *in vitro* can significantly activate the antiviral response to inhibit the PEDV replication on the host cells. However, within the heavy barrage of the upregulated downstream ISGs, it is difficult to identify the highly effective inhibitors against the PEDV infection specifically. Screening the immune-enhancing or IFN-inducible PEDV variants is supposed to be the potential approach to overcome the above challenges. The IFN-inducible PEDV would be an especially ideal viral model to study the interaction between PEDV and the host innate immune responses. Evidently, the IFN-sensitive influenza virus NS1 R38A/K41A strain has been reported to induce higher levels of IFN production and neutralizing antibodies, which has been the alternatives to the traditional vaccines (35). The PEDV is also IFN-sensitive; screening the IFN-inducible PEDV candidate may turn out to be a revolutionary step in the PEDV vaccinology.

The naturally occurring PEDV variant 85-7^C40^ showed significantly attenuated viral replications compared with its parent strain 85-7 on MARC-145 cells. Comparative transcriptome analysis confirmed that the strain 85-7^C40^ induced higher levels of type I and III IFN, thereby leading to the stronger activation of numerous ISGs. Based on these findings, the immune-enhancing variant strain 85-7^C40^ was employed to identify the efficient antiviral proteins and elucidate the underlying mechanisms in the interactions between PEDV and host innate immune responses.

## RESULTS

### The variant strain 85-7^C40^ showed significantly attenuated viral replication on MARC-145 cells.

**FIG 1:**
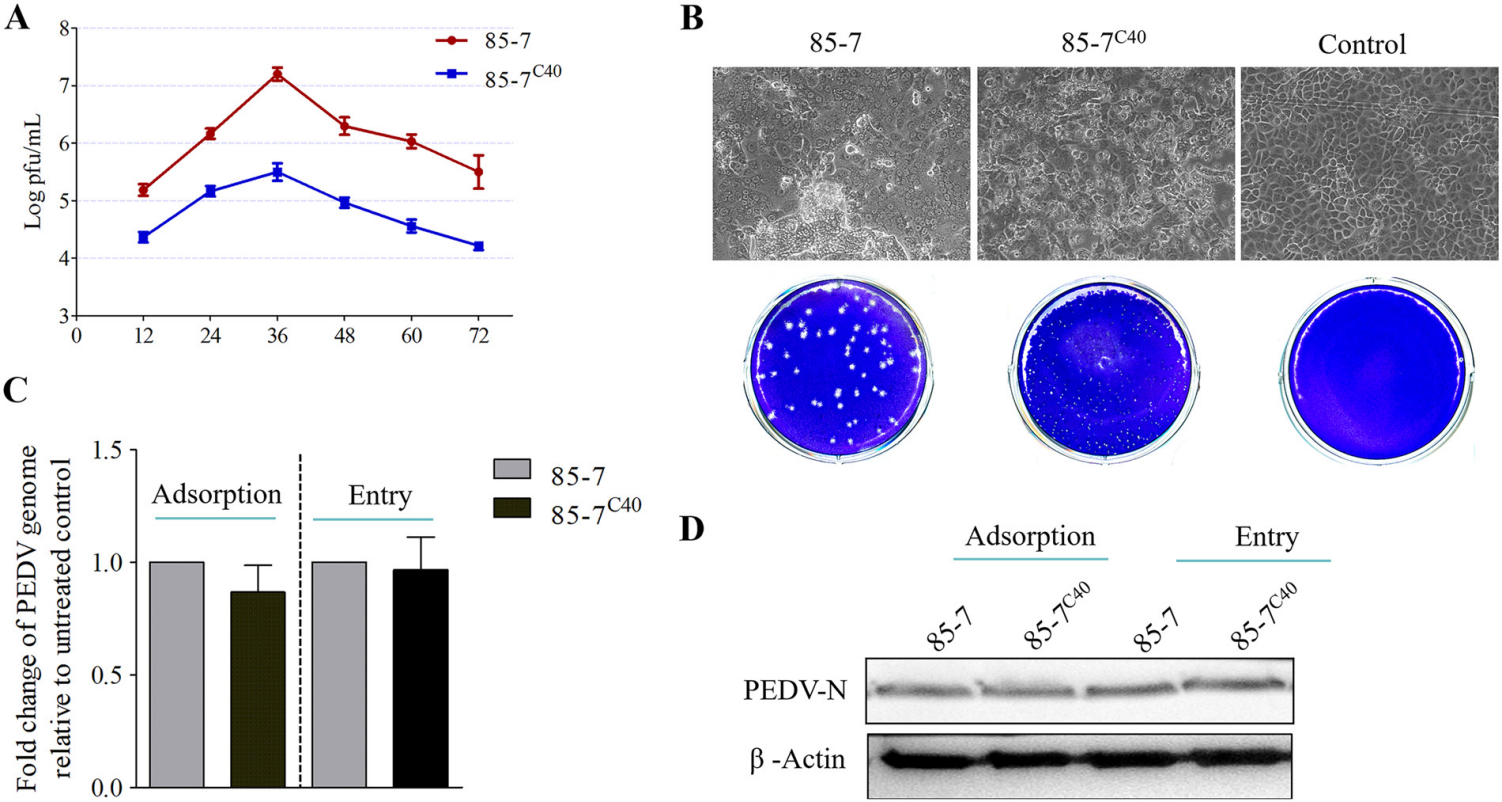
Comparison of the proliferation characteristics between the 85-7 and 85-7^C40^ strains on MARC-145 cells. (A) Virus growth curve of the 85-7 and 85-7^C40^ strains. MARC-145 cells were infected with the indicated strain at an multiplicity of infection (MOI) of 0.1, and then the supernatant was collected at the indicated time. Virus titer was determined by the plaque assay. (B) Comparison of the cytopathic effect (CPE) and plaque phenotype between the 85-7 and variant 85-7^C40^ strains. The 85-7^C40^ strain showed weaker cell-cell fusion capacity and distinctly smaller plaques than the 85-7 strain. (C, D) There was no difference in the adsorption and entry process between the 85-7 and 85-7^C40^ strains. The viral binding and entry assays were performed as described in Materials and Methods. The porcine epidemic diarrhea virus (PEDV) genome level of 85-7-infected group was set as the baseline. (C)The viral RNA was extracted, and the PEDV genome was detected with the quantitative reverse transcription-PCR (RT-qPCR) method. (D) Meanwhile, the cell proteins were collected, and then the same amounts of proteins were subjected to Western blot analysis using the monoclonal antibody (MAb) for PEDV protein N. β-Actin was used as the internal control. Error bars indicate the standard error of the mean (SEM) of three independent experiments.

Previous study had revealed that the PEDV 85-7 strain underwent frequent variations within the cell culture process, and the variant C40 strain (85-7^C40^) exhibited smaller plaque and higher titer on Vero cells (14). As Vero cell is an interferon defect model, we explored whether the variant 85-7^C40^ also exhibited similarly excellent replication capacity on an immune robust model, such as MARC-145 cells. Growth kinetic analysis of the two strains displayed a similar growth pattern, and both of them reached the peak viral titer at 36 h postinfection (hpi) on MARC-145 cells. However, the 85-7^C40^ strain yielded significantly lower titers than the 85-7 strain during the entire infection process (Fig. 1A), which was opposite to the results with Vero cells (14). Compared to the 85-7 strain, the 85-7^C40^ strain had weaker cell-cell fusion capacity and smaller plaque (Fig. 1B), with a similar phenotype on Vero cells (14). Nonetheless, both the quantitative reverse transcription-PCR (RT-qPCR) and the immunoblot analyses indicated that there was no significant difference in the adsorption and entry process between the 85-7 and 85-7^C40^ strains (Fig. 1C and D). When considered together, in view of the different IFN secretion characteristics between the two cell models, the IFN production and its downstream signaling might be regulated specifically in the infection process of 85-7^C40^ on MARC-145 cells.

### Transcriptome analysis illustrated that the 85-7^C40^ induced enhanced antiviral response.

**FIG 2:**
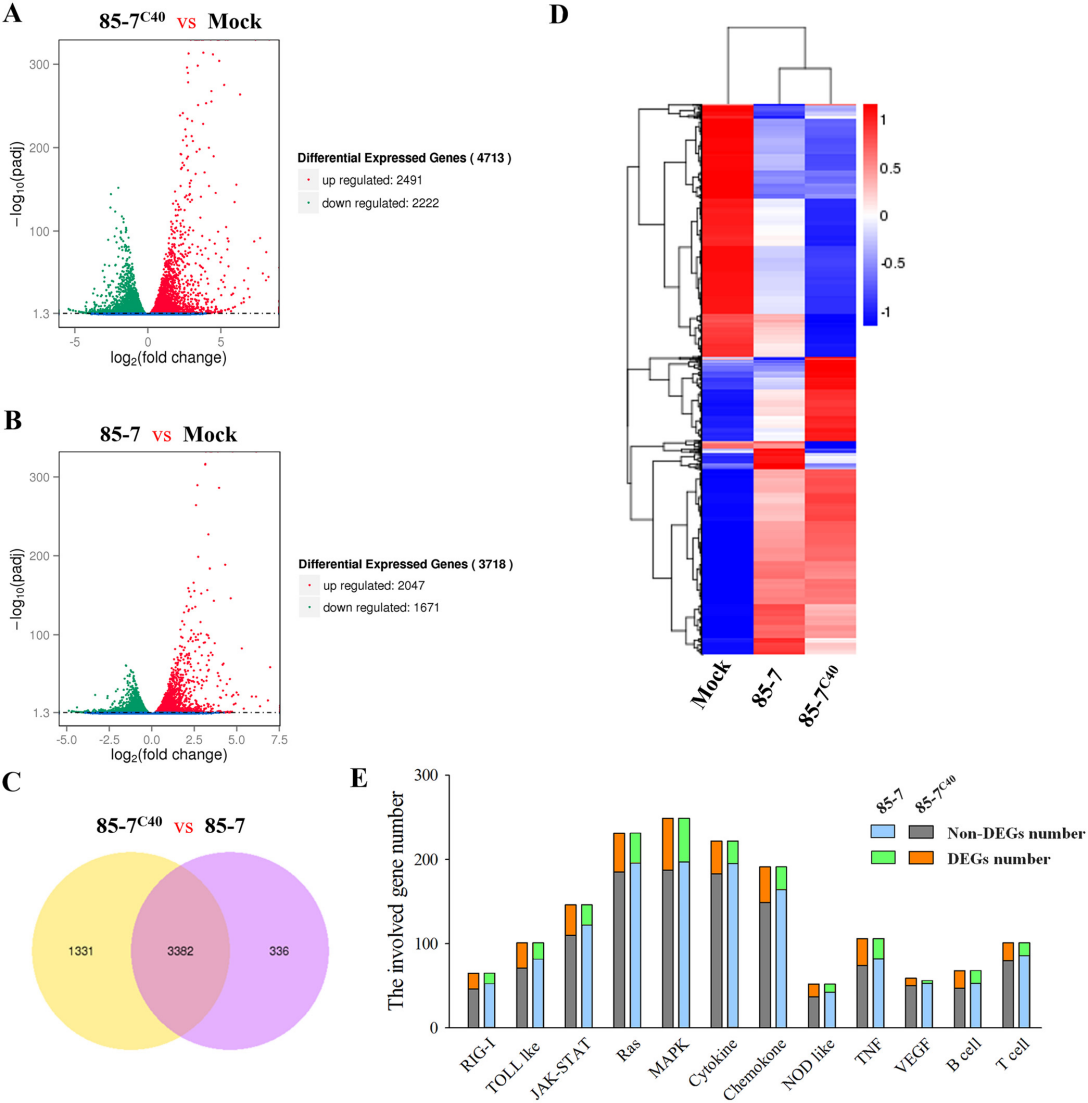
Comparative analysis of the mRNA expression on MARC-145 cells infected with the 85-7 and 85-7^C40^ strains. (A) Volcano plot analysis of the differentially expressed genes (DEGs) in the PEDV variant 85-7^C40^-infected MARC-145 cells. The green dots represent the downregulated DEGs, and the red dots represent the upregulated DEGs. The uninfected MARC-145 cells were used as the mock control. (B) Volcano plot analysis of the DEGs in 85-7-infected MARC-145 cells. (C) Venn diagram of the differential expression analysis. The two groups are represented by different colors. The numbers of specific or common DEGs are labeled in black. The overlapping areas represent the DEG number shared by the two groups; the nonoverlapping areas represent the DEG number that was unique to the other group. (D) Heat map analysis of DEGs. Each row represents a specific DEG, and each column represents a comparison group. Red indicates upregulated expression, and blue indicates downregulated expression. (E) Involved DEG number distributed in each host innate immune related KEGG pathway. Twelve pathways (RIG-I like, Toll-like, JAK/STAT, Ras, MAPK, cytokine, chemokine, NOD-like, tumor necrosis factor [TNF], vascular endothelial growth factor [VEGF], B-cell, and T-cell signaling) were regulated by the PEDV 85-7 or 85-7^C40^ infection on MARC-145 cells. The DEG number and the non-DEG number from the two groups were represented with different colors.

To obtain a global view of the potential host proteins involved in the 85-7^C40^ infection process, comparative transcriptome analysis was performed at 24 hpi. The volcano plots of the differentially expressed genes exhibited that the 85-7^C40^ strain infection induced 4,713 differentially expressed genes (DEGs) (*P* < 0.05) totally, compared to those in the mock-infected MARC-145 cells, with 2,491 upregulated and 2,222 downregulated genes, respectively (Fig. 2A; Table S1). Moreover, compared to the 85-7 strain-infected cells (with 2,047 upregulated and 1,671 downregulated genes) (Fig. 2B; Table S2), it included 3,382 common DEGs and 1,331 special genes (Fig. 2C). Both the upregulated and the downregulated genes were categorized as biological process (BP), molecular function (MF), and cellular component (CC) by the gene ontology (GO) enrichment analysis, while the enriched GO terms of the 85-7^C40^ strain were observed to be slightly different from the 85-7 strain, especially the MF and the CC modules (Fig. S1). In addition, the DEG expression patterns between the PEDV-infected cells and mock-infected cells were classified by heat cluster analysis. The coregulated genes were clustered (Fig. 2D), which exhibited more significant changes in the cells infected by the variant 85-7^C40^, especially in the genes related with the immune response process (Fig. 2E).

The mRNA changes of the cytokines between the 85-7 and the variant 85-7^C40^-infected MARC-145 cells were compared. As shown in Table 1, compared with the mock-infected cells, the infection of the 85-7 and the 85-7^C40^ strains regulated the expression of 26 and 37 cytokines, respectively. For these DEGs, 18 genes were upregulated, like *CXCL3*, *IL-7*, *CXCL8*, and *CXL10*, while 7 genes were downregulated, like *CX3CL1* and *TGFB1*, in both groups. It should be noted that the 85-7^C40^ specifically modulated the expression of 12 cytokines (10 upregulation and 2 downregulation), whereas only 85-7 specifically downregulated *BMP8B*. Remarkably, only the variant 85-7^C40^ activated the transcription of the type I IFN (*IFNB1*) and the type III IFN (*IFNL1* and *IFNL3*) significantly (Table 1). The IFN-λ1 and IFN-λ3 displayed significant anti-PEDV effects (17, 36), and the 85-7 strain was susceptible to the IFN-β (19), implying the potential reason for the lower viral titer of variant 85-7^C40^ on MARC-145 cells. These data revealed that a higher level of antiviral response was induced during the variant 85-7^C40^ infection process, which could be a suitable viral model to screen the host antiviral proteins as candidates for PEDV prevention and control.

**TABLE 1:** Transcription level change of cytokines in 85-7- and 85-7^C40^-infected cells

Genes encoding cytokines	Fold change	
	85-7/mock	85-7^C40^/mock
Upregulation in both strains		
IL7	2.4	3.5
FGF18	2.9	2.20
TNF15	6.1	5.1
TNF18	2.2	2.8
LIF	3.1	4.5
VEGFC	3.5	5.3
BMP2	9.74	10.7
CNTF	2.1	3.1
INHBE	7.8	5.8
GDF15	10.1	9.4
INHBA	26.9	66.7 ^*a*^
CXCL11	5.3/0 ^*b*^	25.2/0^*a*^,^*b*^
CXCL3	11.1	35.7 ^*a*^
CXCL8	16.2	61.4 ^*a*^
CXCL10	42. 6	307.8 ^*a*^
FGF21	18.2	43.6 ^*a*^
CTGF	7.7	18.2 ^*a*^
HBEGF	10.7	29.6 ^*a*^
Downregulation in both strains		
CX3CL1	−2.8	−1.6
C1QT6	−2.2	−3.8
BMP1	−1.9	−2.4
TGFB1	−1.9	−3.2
MIF	−3.2	−2.3
HDGF	−1.8	−2.9
GDF11	−1.7	−2.6
Upregulation only in 85-7^C40^		
IFNB1	/	34.3/0 ^*a*^ ^,^ ^*b*^
IFNL1	/	5.8/0 ^*a*^ ^,^ ^*b*^
IFNL3	/	7.2/0 ^*a*^ ^,^ ^*b*^
CXCL1	/	6.3/0 ^*a*^ ^,^ ^*b*^
IL6	/	8.4/0 ^*a*^ ^,^ ^*b*^
CXCL9	/	3.8/0 ^*a*^ ^,^ ^*b*^
IL12A	/	10.5^*a*^
NGF	/	7.5^*a*^
FGF9	/	4.6^*a*^
CLCF1	/	6.7^*a*^
Downregulation only in 85-7^C40^		
TSG6	/	−4.0^*a*^
NFIL3	/	0/4.6 ^*a*^ ^,^ ^*b*^
Downregulation only in 85-7		
BMP8B	−2.7	/

a The regulated values induced by 85-7^C40^ were significantly higher or lower than that of 85-7.

b The values mean the readcount_85-7 or readcount^C40^ to readcount_mock, and the readcount of the corresponding genes in uninfected MARC-145 cells or 85-7^C40^-infected MARC-145 cells were zero. /, indicates the transcription of corresponding genes was undetected in the transcriptome data. The underline is responsible for emphatically pointing out the significant transcription level change of corresponding genes in 85-7- and 85-7^C40^-infected cells.

### Classification and verification of upregulated ISGs during PEDV 85-7 and 85-7^C40^ infection.

**FIG 3:**
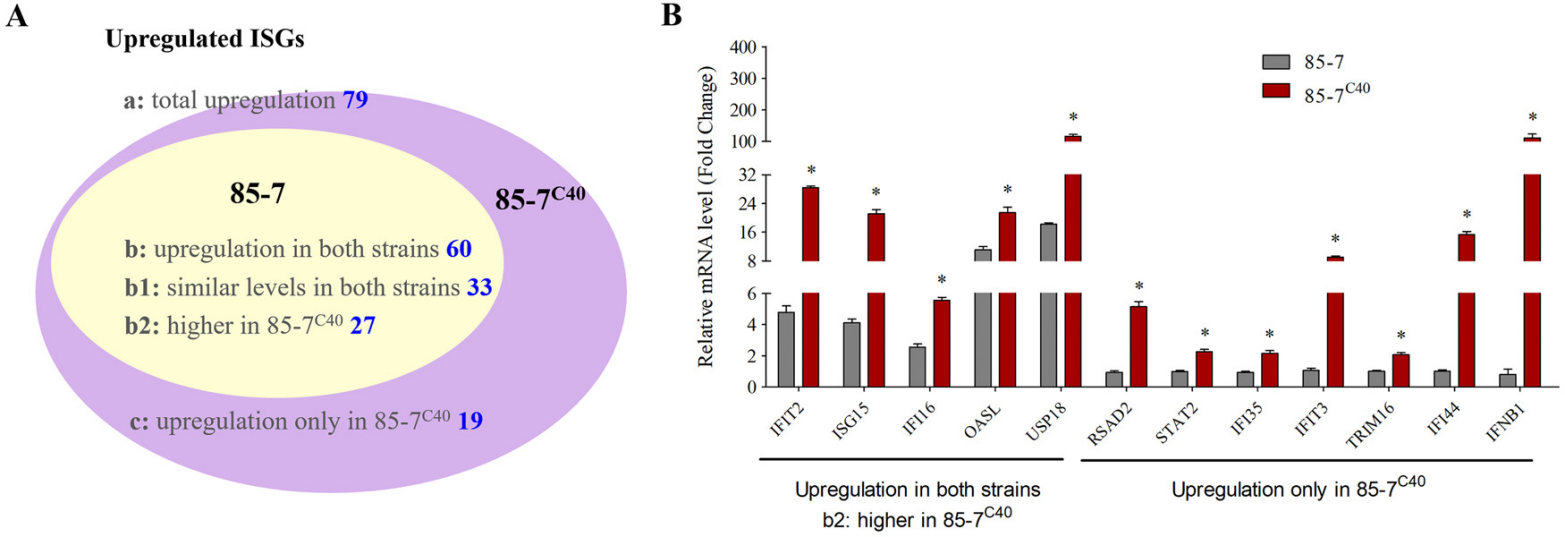
Classification and verification of differentially expressing interferon-stimulated genes (ISGs) during 85-7 and 85-7^C40^ infection on MARC-145 cells. (A) Classification of the upregulated ISGs during 85-7 and 85-7^C40^ infection. The two groups are represented by different colors. The 85-7 infection induced ISGs are all included in the 85-7^C40^-infected group, and the nonoverlapping areas represent the ISG number that was unique in the 85-7^C40^ infection group. (B) Confirmation of the different expression genes by RT-qPCR. The 85-7- or 85-7^C40^-infected MARC-145 cells were collected, respectively. Then, the RNA was extracted, and the genes were detected with corresponding primers. Here, five ISGs (*IFIT2*, *ISG15*, *IFI16*, *OASL*, and *USP18*) upregulated in both infection groups, and the other seven genes (*RSAD2*, *IFIT3*, *STAT2*, *IFI35*, *TRIM16*, *IFI44*, *IFNB*1) upregulated only in the 85-7^C40^ infection process were selected. The qPCR verification results were consistent with the trends in the sequencing results. Error bars indicate the standard error of the mean (SEM) of three independent experiments.

ISGs were the potential candidates to screen the antiviral proteins, as numerous ISGs owned the direct antiviral activities by targeting different viral cycle stages (21, 22). Though PEDV evolved numerous strategies to evade the type I IFN production and inhibit the JAK/STAT signaling (8, 11, 19), the 85-7 strain still regulated the expression of around 78 ISGs, while the IFN-inducible strain 85-7^C40^ infection regulated about 100 ISGs. Among these ISGs, 60 genes were upregulated in both groups, while the 85-7^C40^ infection led to the higher upregulation of 27 ISGs (Table 2, Fig. 3A). There were 19 specific upregulated ISGs within the 85-7^C40^-infected group; especially *RSAD2*, *IFI44*, *IFIT3*, *IFI35*, *TRIM16*, and *CXCL9* were totally silenced in the mock process and 85-7 strain-infected groups, while *IFIT3* and *IFI44* displayed the highest upregulation in the 85-7^C40^-infected group (Table 2). Moreover, 14 ISGs were downregulated in both groups (e.g., *TRIM7*, *IFITM10*, *IFI30*), and 7 ISGs were downregulated just in the 85-7^C40^-infected group (e.g., *TNFAIP6* and *PMM2*).

**TABLE 2:** Transcription level change of interferon-stimulated genes (ISGs) in 85-7- or 85-7^C40^-infected MARC-145 cells

ISGs	Fold change	
	85-7/mock	85-7^C40^/mock
Similar upregulation in both strains		
TRIM4	2.8	3.5
TRIM47	2.1	1.8
TRIM56	2.2	3.2
SOCS4	2.2	3.0
SOCS5	9.1	9.5
AQP	2.9	3.8
PDGFRL	2.3	3.1
JUNB	5.0	4.2
VEGFC	3.5	5.3
CEBPD	2.7	1.8
FUT4	2.4	2.9
NUP50	1.5	2.2
MYD88	1.6	3.2
EPAS1	2.2	2.3
MTHFD2	3.9	3.6
SPTLC2	2.2	2.8
ABTB2	3.2	3.5
TNFRSF10A	2.8	3.3
DCP1A	2.1	2.5
SMAD3	2.4	2.1
PIM3	6.0	7.0
SPSB1	3.4	3.4
BAG1	2.0	2.8
RNF19B	2.5	3.2
ODC1	2.2	3.2
TXNIP	2.5	2.0
C4orf32	3.3	4.0
EHD4	2.9	3.8
AQP9	6.6	8.4
SAMD4A	5.0	6.9
CRY1	3.0	4.1
BLZF1	2.1	3.3
N4BP1	3.2	3.7
Higher upregulation in 85-7^C40^		
IFI6	4.1	16.8 ^*a*^
IFIT2	4.4	25.3 ^*a*^
IFIT5	2.3	8.7 ^*a*^
TRIM25	1.5	2.9 ^*a*^
ISG15	4.1	17.5 ^*a*^
IFI16	2.2	5.8 ^*a*^
USP18	34.1	156.2 ^*a*^
GBP2	1.8	5.6 ^*a*^
DDX58	7.0	32.7 ^*a*^
APOL2	6.1	23.2 ^*a*^
EIF2AK2	1.8	4.2 ^*a*^
XAF1	7.2	17.1 ^*a*^
PARP12	1.8	3.6 ^*a*^
PMAIP1	6.3	12.9 ^*a*^
SOCS1	3.8	11.9 ^*a*^
OAS1	5.0/0 ^*b*^	14.4/0 ^*a*^ ^,^ ^*b*^
OAS2	20.5/0 ^*b*^	102.6/0 ^*a*^ ^,^ ^*b*^
OASL	16.7/0 ^*b*^	30.7/0 ^*a*^ ^,^ ^*b*^
IFI44L	10.7/0 ^*b*^	28.2/0 ^*a*^ ^,^ ^*b*^
CXCL11	5.3/0 ^*b*^	25.2/0 ^*a*^ ^,^ ^*b*^
CXCL10	42.6	307.8 ^*a*^
MAFF	6.8	14.8 ^*a*^
ATF3	70.5	318.2 ^*a*^
TMEM140	4.1	8.0 ^*a*^
RIPK2	4.7	7.8 ^*a*^
DUSP5	3.8	7.9 ^*a*^
PLEKHA4	14.9	27.2 ^*a*^
Upregulation only in 85-7^C40^		
RSAD2	/	3.9/0 ^*a*^ ^,^ ^*b*^
IFI44	/	12.3/0 ^*a*^ ^,^ ^*b*^
IFIT3	/	30.8/0 ^*a*^ ^,^ ^*b*^
IFI35	/	2.1/0 ^*a*^ ^,^ ^*b*^
TRIM16	/	2.1/0 ^*a*^ ^,^ ^*b*^
OAS3	/	8.9^*a*^
STAT1	/	3.6^*a*^
STAT2	/	3.1^*a*^
IRF8	/	5.7^*a*^
MOV10	/	3.0^*a*^
CCL2	/	2.4^*a*^
BATF2	/	2.0^*a*^
CXCL9	/	3.8/0 ^*a*^ ^,^ ^*b*^
CCL5	/	11.7^*a*^
DTX3L	/	4.0^*a*^
MCL1	/	3.2^*a*^
PLSCR1	/	8.7^*a*^
BST2	/	2.1^*a*^
PSMB9	/	5.7^*a*^
Downregulation in both strains		
TRIM7	−2.5	−8.2^*a*^
TRIM17	−2.7	−3.5
TRIM6	−1.9	−2.2
IFITM10	−2.3	−3.3
IGFBP2	−2.7	−1.9
FNDC4	−1.8	−2.9
ENPP1	−1.7	−2.1
IFI30	−2.0	−2.5
SCARB2	−1.6	−2.1
GPX2	−3.2	−3.5
AHNAK2	−3.4	−2.8
CX3CL1	−1.6	−2.8
UNC93B1	−1.6	−2.1
IMPA2	−2.0	−3.7
Downregulation only in 85-7^C40^		
TNFAIP6	/	−4.0^*a*^
PMM2	/	−3.1^*a*^
NFIL3	/	0/4.6 ^*a*^ ^,^ ^*b*^
RNASE4	/	−3.2^*a*^
HEG1	/	−6.2^*a*^
Opposite changes between the 85-7 and 85-7^C40^		
IL6ST	1.2	0/3.92 ^*a*^ ^,^ ^*b*^
GALNT2	1.3	−11.4^*a*^

a The regulated values induced by 85-7^C40^ were significantly higher or lower than that of parent 85-7.

b The values mean the readcount_85-7 or readcount_85-7^C40^ to readcount_mock, and the readcount of the corresponding genes in uninfected MARC-145 or 85-7^C40^-infected MARC-145 cells were zero. /, indicates the transcription of corresponding genes was undetected in the transcriptome data. The underline is responsible for emphatically pointing out the significant transcription level change of corresponding genes in 85-7- and 85-7^C40^-infected cells.

To verify the transcriptomic results, 12 genes were selected for validation through the RT-qPCR analysis. Among the selected ISGs, 5 genes (*IFIT2*, *ISG15*, *IFI16*, *OASL*, and *USP18*) were upregulated to a higher level in PEDV 85-7^C40^-infected group, while the other 7 genes (*RSAD2*, *IFIT3*, *STAT2*, *IFI35*, *TRIM16*, *IFI44*, and *IFNB1*) were induced only in the PEDV 85-7^C40^-infected group. The RT-qPCR results were basically consistent with the transcriptomic results (Fig. 3B), indicating that the PEDV 85-7 strain infection induced the activation of several ISGs independent of the IFN production, while the PEDV variant 85-7^C40^ strain infection stimulated a stronger immune response with higher and broader expression of the ISGs.

### Anti-PEDV effect of the ISGs specifically stimulated by the variant 85-7^C40^ infection.

**FIG 4:**
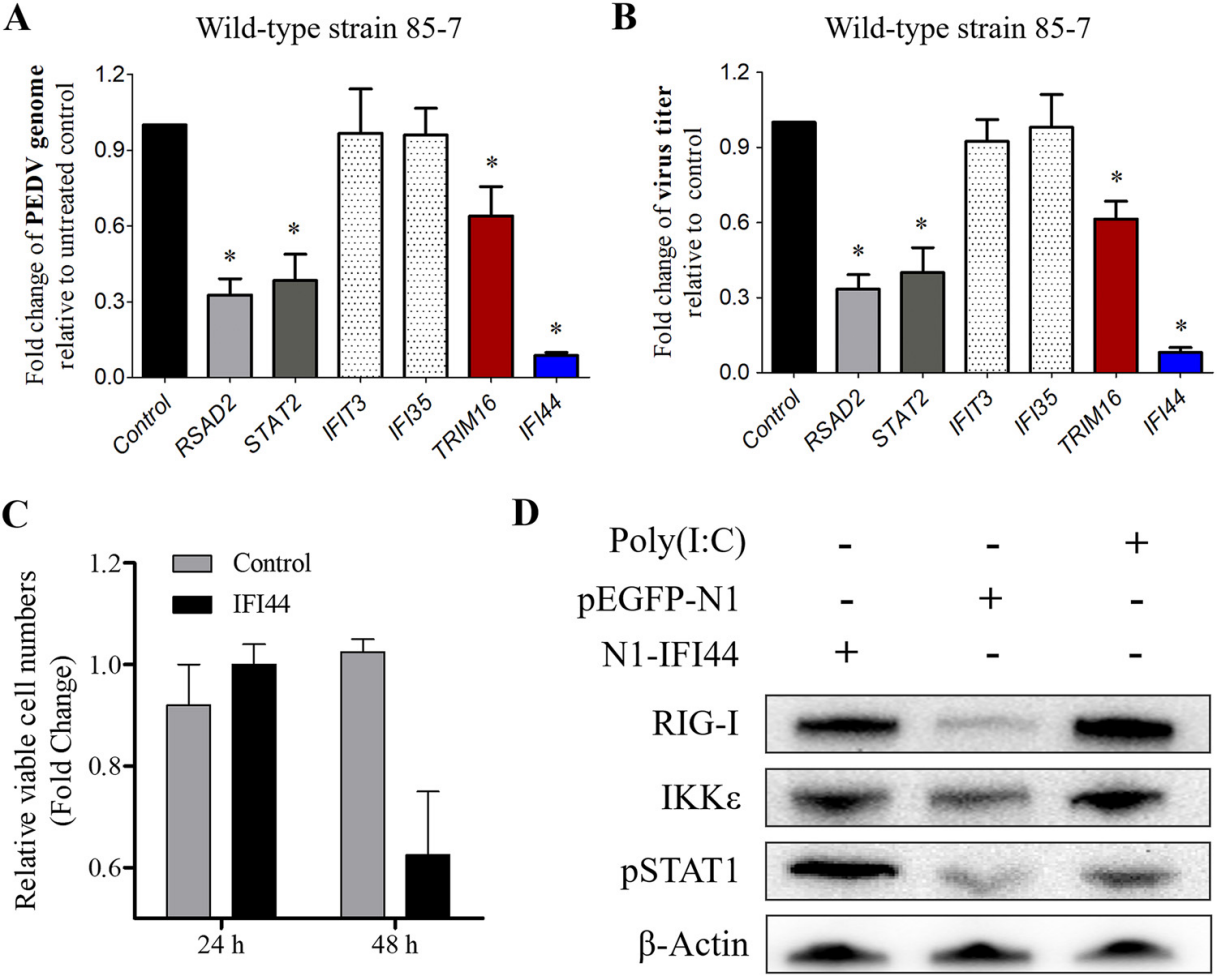
ISG protein IFI44 was stimulated by the variant 85-7^C40^ strain infection specifically and showed powerful anti-PEDV activity. (A) Effect of the overexpressed ISG proteins on the fold change of PEDV genome. The ISG proteins overexpressed MARC-145 cells were infected with PEDV at an MOI of 0.1. The viral genome was extracted and analyzed with RT-qPCR method. The PEDV genome in the MARC-145 cells transfected with vector pEGFP-N1 was used as the control. (B) Effect of the overexpressed ISG proteins on the fold change of viral titer. The ISG proteins overexpressed MARC-145 cells were infected with the PEDV at an MOI of 0.1. The cellular supernatant was collected, and then the viral yield was determined by plaque assay. The viral titer in the MARC-145 cells transfected with vector pEGFP-N1 was used as the control. (C) Relative viable cell numbers after IFI44 overexpression. MARC-145 cells were seeded at equal densities and then transfected with empty vector (pEGFP-N1, control) or N1-IFI44; viable cell numbers were quantified after 24 or 48 h by trypan blue exclusion. Error bars indicate the standard error of the mean (SEM) of three independent experiments. (D) IFI44 activated RIG-I signaling. MARC-145 cells were seeded at equal densities and then transfected with empty vector (pEGFP-N1, control) or N1-IFI44 for 24 h; then cell lysates were immunoblotted with the indicated antibodies. The poly(I:C) transfected cells were used as the positive control.

The ISGs specifically produced in the 85-7^C40^-infected MARC-145 cells were focused on first. The MARC-145 cells were transfected with the recombinant plasmids (N1-RSAD2, N1-IFIT3, N1-IFI44, N1-IFI35, N1-TRIM16, or N1-STAT2). Among the six ISGs selected here, *RSAD2*, *IFIT3*, *IFI44*, *IFI35*, and *TRIM16* were totally silenced in the mock process and the 85-7 strain-infected group, and *STAT2* was the important adaptor molecule in JAK/STAT signaling. Both the fluorescence and Western blot analyses indicated that RSAD2, IFIT3, IFI44, IFI35, TRIM16, and STAT2 were expressed effectively (Fig. S2A and B), although the expression levels of RSAD2 and TRIM16 were relatively low until 36 and 48 h, respectively. With the overexpression of the corresponding ISG proteins, IFI35 and IFIT3 displayed no anti-PEDV effect, TRIM16 reduced the PEDV genome and titer by about 40%, while RSAD2 and STAT2 were at about 60% (Fig. 4A and B). In addition, both the RT-qPCR and Western blot results illustrated that all of them had no effect on PEDV binding and entry efficiency (Fig. S3). Moreover, IFI44 had the strongest anti-PEDV effect, with the PEDV genome being reduced powerfully, and the viral titer reduced from 10^6.1^ to 10^5.0^ PFU/mL (Fig. 4A and B). These results suggested that the variant 85-7^C40^ induced specific ISG proteins and then exhibited a significant anti-PEDV activity, particularly the protein IFI44.

### Overexpression of IFI44 did not reduce cellular proliferation but activated the RIG-I production on MARC-145 cells.

Several previous studies have confirmed the antiviral effect of IFI44 in human original cells (31, 32), while the underlying mechanisms in the original animal cells need to be further explored. Reduced cellular proliferation is a common feature of the IFN response, which is mediated by canonical ISGs, such as IFI44 (32). Viable cell numbers were quantified manually by Trypan blue exclusion, and the results indicated that overexpression of IFI44 did not reduce the cellular proliferation after 24 h but led to a little reduction in proliferation after 48 h without significance (Fig. 4C). The antiviral activity of IFI44 was evaluated at 24 h, suggesting that the observed antiviral activity was not associated with the reduction in viable cell number and proliferation. Furthermore, the activation of RIG-I signaling with the overexpression of IFI44 was detected, and Western blot assay showed that IFI44 could induce the production of RIG-I and IKKε (Fig. 4D). In addition, the phosphorylation of STAT1 (pSTAT1) was also upregulated with the overexpression of IFI44. These results suggested that IFI44 might restrict PEDV replication by positively regulating the production of type I IFN and activating its downstream signaling.

### Anti-PEDV effect of the ISGs higher upregulated by the PEDV variant 85-7^C40^.

**FIG 5:**
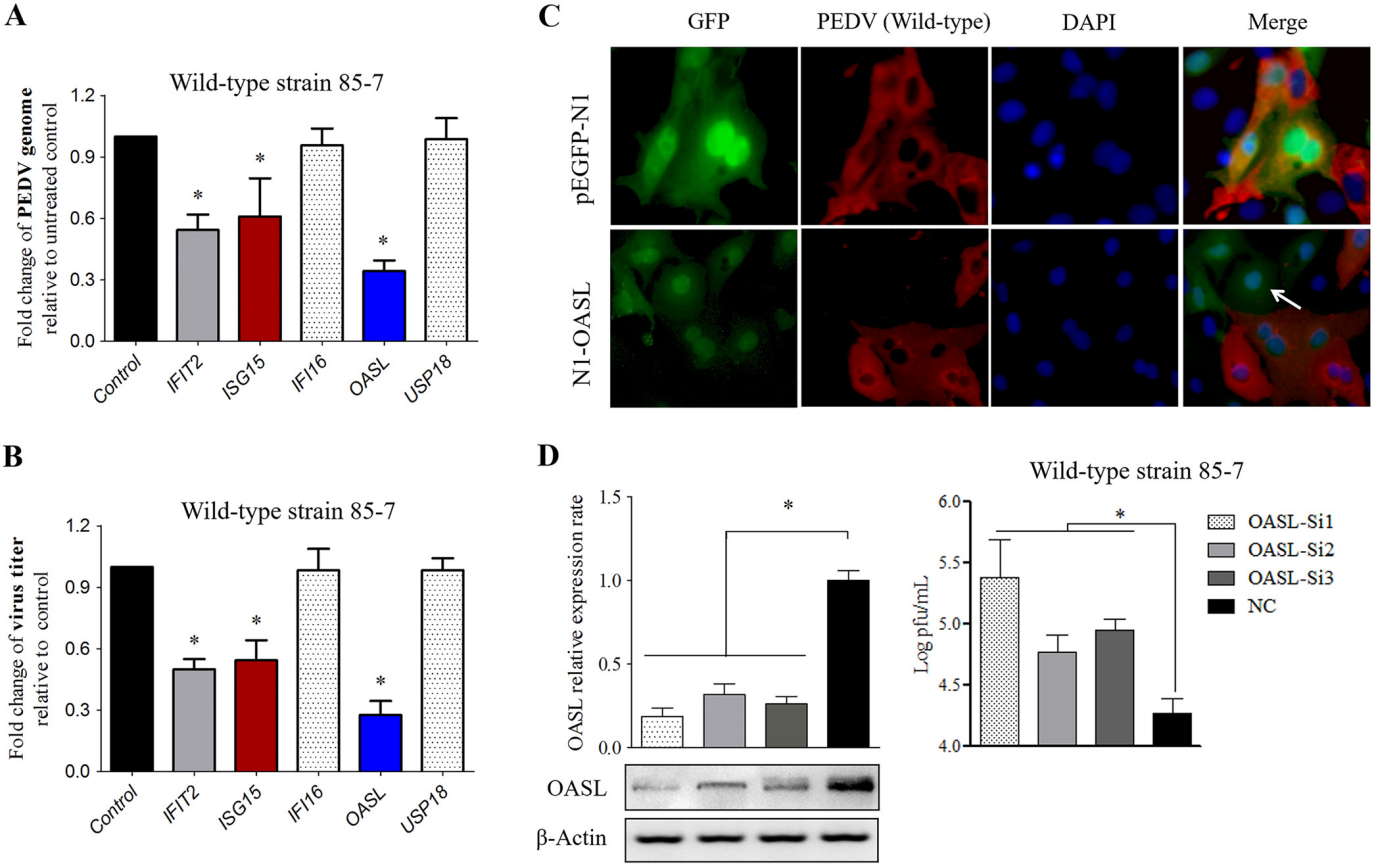
Antiviral activity of the ISGs induced by both the 85-7 and 85-7^C40^ strains on MARC-145 cells. (A) Effect of the overexpressed ISGs on the fold change of PEDV genome. The ISGs overexpressed MARC-145 cells were infected with PEDV at an MOI of 0.1. The viral genome was extracted and analyzed with the RT-qPCR method. The PEDV genome in the MARC-145 cells transfected with vector pEGFP-N1 was used as the control. (B) Effect of the overexpressed ISGs on the fold change of viral titer. The ISGs overexpressed MARC-145 cells were infected with PEDV at an MOI of 0.1. The cellular supernatant was collected, and then the viral yield was determined by plaque assay. The viral titer in the MARC-145 cells transfected with vector pEGFP-N1 was used as the control. (C) OASL-overexpressed cells were partly free of PEDV infection. MARC-145 cells transfected with pEGFP-N1 or N1-OASL plasmids were infected with PEDV at an MOI of 0.1 for 24 h. Then, the cells were fixed for IFA assay with PEDV-specific MAb. (D) Knockdown of OASL enhanced PEDV infection on MARC-145 cells. Three pairs of OASL-specific short interfering RNAs (siRNAs) (Si1, Si2, and Si3) were transfected into the MARC-145 cells, the mRNA and protein levels of OASL were detected by RT-qPCR and Western blot, respectively. The irrelevant siRNA was used as negative control (NC). β-Actin was used as the internal reference. Then, the OASL knockdown MARC-145 cells were infected with PEDV at an MOI of 0.1. The cellular supernatant was collected at 24 hpi, and the virus titer was detected as PFU/mL. Error bars indicate the standard error of the mean (SEM) of three independent experiments. DAPI, 4′,6-diamidino-2-phenylindole; GFP, green fluorescent protein.

Numerous ISGs were upregulated to higher levels (generally more than 20-fold) under the enhanced immune responses, suggesting that the robust antiviral effects might be driven by them. The five selected ISGs (*IFIT2*, *ISG15*, *IFI16*, *USP18*, and *OASL*) were all upregulated to a greater extent in 85-7^C40^-infected MARC-145 cells, and *OASL* was totally silenced in the mock process. Fluorescence observation and Western blot analysis verified that these five proteins were overexpressed effectively (Fig. S4A and B). In comparison with the control group, OASL, ISG15, and IFIT2 reduced the PEDV genome copies significantly, while the other two ISG proteins displayed no effects on PEDV replication (Fig. 5A). A similar effect was also found on the fold changes of the PEDV titers (Fig. 5B), thereby implying that only OASL, ISG15, and IFIT2 exhibited significant anti-PEDV activity. In addition, the RT-qPCR and Western blot results indicated that the OASL, ISG15, and IFIT2 exhibited no effect on PEDV adsorption (Fig. S3A and C) and cell entry process (Fig. S3B and D). Of the above three anti-PEDV proteins, OASL presented the highest antiviral effect, and the OASL-overexpressed cells were partly free of viral infection (Fig. 5C). As the expression level of OASL was upregulated significantly during PEDV infection process, the native OASL expression was silenced with the specific short interfering RNA (siRNA), and subsequently the PEDV yield was enhanced (Fig. 5D).

### OASL interacted with RIG-I to regulate the antiviral response of the interferon on MARC-145 cells.

**FIG 6:**
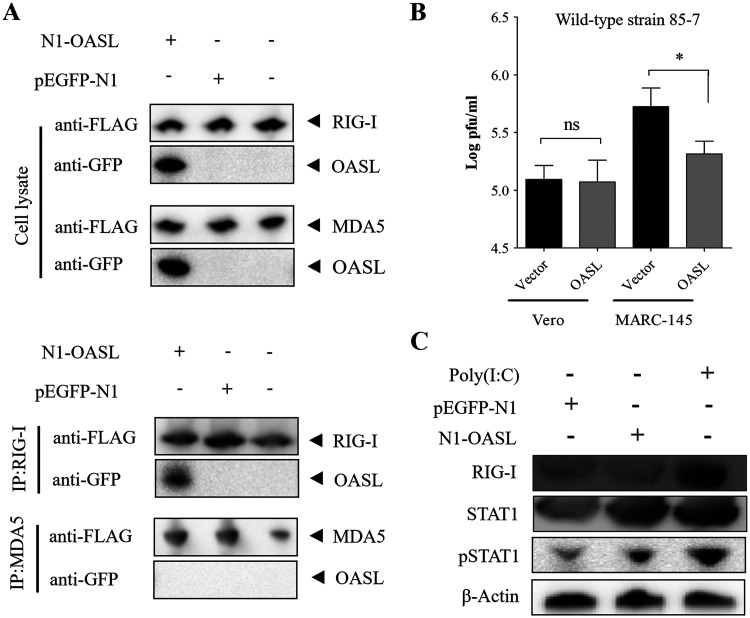
OASL interacted with RIG-I to regulate antiviral response of interferon on MARC-145 cells. (A) OASL interacted directly with RIG-I on MARC-145 cells. The MARC-145 cells were transfected with N1-OASL and pFLAG-RIG-I or pFLAG-MDA5; then the cell lysate was collected to verify the corresponding proteins’ expression and immunoprecipitated (IP) with FLAG antibody, followed by immunoblotting with GFP antibody. (B) Anti-PEDV effect of OASL on MARC-145 and Vero cells. MARC-145 or Vero cells were transfected with N1-OASL or vector pEGFP-N1 for 24 h and then infected with PEDV at an MOI of 0.1. Then, the cellular supernatant was collected, and the viral yield was compared to the pEGFP-N1 transfected group, respectively. Error bars indicate the standard error of the mean (SEM) of three independent experiments. (C) OASL activated phosphorylation of STAT1. MARC-145 cells were seeded at equal densities and then transfected with empty vector (pEGFP-N1, control) or N1-OASL for 24 h; then cell lysates were immunoblotted with the indicated antibodies. The poly(I:C)-transfected cells were used as the positive control. ns, not significant.

The earlier reports indicated that the human OASL owned the antiviral effect by enhancing the RIG-I pathway and interacting with the RIG-I directly (25). To confirm whether the OASL interacted with the RIG-I to perform a similar function on MARC-145 cells, a co-IP assay was used to detect the interaction effect. The results illustrated that OASL, RIG-I, and MDA5 were expressed efficiently, and the co-IP assay exhibited that OASL directly interacted with RIG-I, but not with the MDA5 (Fig. 6A). Moreover, OASL exhibited the anti-PEDV effect on MARC-145 cells but not on Vero cells (Fig. 6B), which might due to the different characteristics of type I IFN production on these two cell lines. Evidently, the silence of the native RNaseL had no effect on OASL anti-PEDV activity (Fig. S5), which further verified that the anti-PEDV effect of OASL was not dependent on the RNaseL. In addition, overexpression of OASL did not induce the production of RIG-I but activated the expression of STAT1 and phosphorylation of STAT1 (Fig. 6C), which implied that it could activate JAK/STAT signaling. All the above results implied that OASL inhibited the PEDV replication depending on the OASL/RIG-I signaling and then activated its downstream signaling on MARC-145 cells.

## DISCUSSION

Presently the inactivated, live-attenuated, and subunit (S protein) vaccines are the important measures to control PEDV infection, although their prevention efficiency needs to be improved further (37). One reason for this is the frequent genetic mutations of PEDV (5–7). Another more vital reason is that PEDV infection antagonizes the host innate immune responses mediated by the type I and III IFN effectively (10, 12, 38), which creates an impediment to screen the host antiviral proteins. PEDV can antagonize the JAK/STAT signaling by the targeted degradation of STAT1 (20), which significantly inhibits the IFN production and following activation of the downstream ISGs (18). In this study, several ISGs were activated at relatively low levels through an IFN-independent mode on PEDV wild-type strain 85-7-infected MARC-145 cells and then displayed little anti-PEDV activity. These findings suggested that the ISGs with significant antiviral effects seemed to be hard to screen out using an immunosuppressive PEDV strain. Further works need to overcome the current challenge caused by the immune escape of PEDV.

Several studies reported that PEDV underwent frequent variations in the infection process *in vivo* and *in vitro*, and many functional sites were identified to promote the virus to regulate the host microenvironment (5, 14–16). We sought to screen out a PEDV variant to activate the host antiviral response and then identified the effective anti-PEDV ISGs. The IFN-inducible strain 85-7^C40^ was screened out unwittingly by the continuous proliferation on the IFN-deficient Vero cells, thereby implying that the continuous proliferation on the immune-deficient cells might be beneficial for the virus to evolve into an immune-enhancing strain. The comparative transcriptome analysis indicated that the variant 85-7^C40^ activated type I and III IFN production and significantly stimulated the upregulation of 19 peculiar ISGs, and several of them exerted effective anti-PEDV activity, thus partially revealing why the 85-7^C40^ strain presented lower titer than the 85-7 strain on MARC-145 cells.

Even so, one concern was raised that the strain 85-7^C40^ is a laboratory-generated variant, which might be significant different from the natural variants generated in the host animal. Therefore, the innate immune responses induced by strain 85-7^C40^ might be inconsistent with the situations of host animal infected by a wild-type strain. Our study selected 11 upregulated ISGs to recertify their antiviral effects on the wild-type PEDV strain 85-7. Although four ISGs, IFIT3, IFI35, IFI16 and USP18, really had no antiviral effects, seven other ISGs significantly decreased the replications of the wild-type strain 85-7. PEDV was reported to escape from the host immune response by interfering with RIG-I signaling (39), whereas OASL robustly interacted with RIG-I directly, activated the expression of phosphorylation of STAT1, could counteract the antagonism, and then played anti-PEDV activity on MARC-145 cells. Moreover, IFI44 played a crucial part in regulating the replication of the various viruses, and overexpressed IFI44 significantly contributes to the restriction of PEDV replication on MARC-145 cells. In addition, IFI44 was totally silenced upon 85-7 strain infection, but it was extremely activated in the variant 85-7^C40^ infection process, further illustrating that the PEDV 85-7 strain might shield the expression of numerous anti-PEDV ISGs to create an enabling environment in a specific manner. Although DeDiego et al. (33) reported that human IFI44 negatively regulated the IFN responses and supported IAV and LCMV replication on A549 cells, our study identified that IFI44 could activate RIG-I signaling. Two other studies from different laboratories verified that IFI44 uses antiviral activity to reduce the replication of BUNV and RSV on HeLa and A549 cells, which is consistent with our results for PEDV on MARC-145 cells. The sequence identity between the IFI44 protein derived from MARC-145 cells and human IFI44 protein was just 86.3%, with a 61-amino-acid mutation (Fig. S6), while the underlying mechanism for the diverging roles of IFI44 protein derived from different hosts in antiviral action need to be further explored.

Previous studies also have identified several host proteins shown the anti-PEDV activity, such as BST2, STAT1, FBXW7, S100A11, and hnRNPA1 (20, 34, 40). In the transcriptome results of this study, the encoding genes of BST2, STAT1, and hnRNPA1 were only upregulated in the PEDV 85-7^C40^ infection process. *S100A11* was downregulated in both the PEDV 85-7 and 85-7^C40^ infection processes, and no significant difference was observed between them. *FBXW7* was upregulated in the PEDV infection process for both 85-7 and 85-7^C40^, while it showed a higher level in 85-7^C40^-infected MARC-145 cells. Furthermore, the transcription factor IRF8, significantly associated with the pathogenic infection and host response (41), was determined to mediate the upregulation of numerous genes relevant to PEDV infections (42). In our data, IRF8 was upregulated only in the PEDV 85-7^C40^ infection process and thus was speculated to mediate the immune regulatory process between PEDV strain 85-7^C40^ and host cells. The interaction between PEDV and host cells during infection was a complex process; the comprehensive regulation of diverse ISGs and other host factors determined the proliferation efficiency of the infected virus.

In particular, the PEDV variant 85-7^C40^, with the characteristics of activating the IFN production, provided the ideal model to study the interaction mechanism between PEDV infection and the ISGs. The IFN defense against the viral infection was mediated by activation of the ISGs, whereas the PEDV-mediated IFN antagonistic effect could limit the screening of the anti-PEDV ISGs (20). Moreover, earlier studies reported that the PEDV encoded diverse proteins, including nsp1, PLP2, nsp5, nsp14, nsp15, ORF3, E, M, N, and nsp16, to antagonize the IFN antiviral response (8, 11, 43). The comparative genomic analysis of our two strains (85-7 and 85-7^C40^) indicated the sequence differences only in the nsp16, S, and E proteins (Table S3), suggesting that further exploring the key sites involved in the variant 85-7^C40^ inducing IFN production process could be helpful to clarify the PEDV infection mechanism. Otherwise, the PEDV variant 85-7^C40^, with variation in the TM domain of the protein E (Table S3), also activated higher expression levels of inflammatory factors (interleukin [IL]-6 and IL-8, etc.) on Vero and MARC-145 cells, indicating that the 85-7^C40^ strain was an appropriate model to explore the characteristic of inflammatory response regulated by PEDV infection.

In summary, we obtained a naturally occurring PEDV variant 85-7^C40^ strain inducing type I and III IFN production and proposed as an ideal viral model to study the interaction mechanism between PEDV and the host innate immune responses. Using this immune-enhanced strain, we identified numerous specific proteins involved PEDV infection on MARC-145 cells. Thus, IFI44 and OASL were determined to positively regulate the RIG-I signaling, activate the phosphorylation of STAT1 on MARC-145 cells, and show effective anti-PEDV activities. These findings would be beneficial for elucidating the PEDV immune escape mechanism and in the development of an improved vaccine.

## MATERIALS AND METHODS

### Viruses and cells.

MARC-145 cells were maintained in Dulbecco’s modified Eagle’s medium (DMEM; HyClone, USA) with 10% fetal bovine serum (TCB, USA). PEDV 85-7 strain and its variant 85-7^C40^ were saved in our lab and propagated on MARC-145 cells at a multiplicity of infection (MOI) of 0.1 in the absence of trypsin (14). The cells and viruses were cultured at 37°C with 5% CO_2_ for the indicated time. Viable cell numbers were quantified manually by trypan blue exclusion.

### Virus proliferation characteristic analysis.

MARC-145 cells were infected with the same amount of the PEDV 85-7 or 85-7^C40^ strain at an MOI of 0.1, and the plaque morphology and cytopathic effect (CPE) were recorded and compared. Then, the viral binding and entry assays were performed as previously described (44). The binding and entry efficiency were evaluated with the viral genome level and protein N expression level by the RT-qPCR and Western blot assay, respectively. As to the virus growth curve assay, MARC-145 cells were infected with the 85-7 or 85-7^C40^ strain at an MOI of 0.1, and the culture supernatants were collected at the indicated time points (12, 24, 36, 48, 60, and 72 hpi). Then, the virus titer was determined by plaque assay and quantified as PFU/mL.

### Comparative transcriptome analysis.

MARC-145 cells were infected with the PEDV 85-7 or 85-7^C40^ strain at an MOI of 0.1 in 6-well plates, and then the cells were collected with 1 mL/well RNAiso Plus reagent (TaKaRa, Dalian, China). Each sample was set for three repeats, and the mock-infected cells were used as a negative control. The mock samples were labeled A1 to A3, the 85-7-infected cells were labeled B1 to B3, and the 85-7^C40^-infected cells were labeled C1 to C3, respectively. Then, the sample preparation (RNA quantification and qualification, library preparation for transcriptome sequencing, clustering, sequencing, etc.) and the data analysis (quality control, reads mapping to the reference genome, quantification of gene expression level, differential expression analysis, etc.) were performed by the Novogene Company (Beijing, China). The results were further verified by RT-qPCR assay as described below.

### Overexpression of ISGs proteins.

The genes of the relevant ISGs (*IFIT2*, *IFIT3*, *ISG15*, *IFI16*, *USP18*, *OASL*, *RSAD2*, *IFI44*, *IFI35*, *TRIM16*, and *STAT2*) were amplified from the PEDV variant 85-7^C40^-infected MARC-145 cells by RT-PCR, and the corresponding primers (GenScript, Nanjing, China) are shown in Table S4. Then, the gene fragments were cloned into the eukaryotic expression plasmid pEGFP-N1 with the ClonExpress Ultra one-step cloning kit (Vazyme, Nanjing, China), and the recombinant vectors were designated N1-IFIT2, N1-IFIT3, N1-ISG15, N1-IFI16, N1-USP18, N1-OASL, N1-RSAD2, N1-IFI44, N1-IFI35, N1-TRIM16, and N1-STAT2, respectively. All the proteins were labeled with the green fluorescent protein (GFP) tag. The recombinant plasmids were extracted with the E.Z.N.A. Endo-Free plasmid minikit I (OMEGA, USA), and the same amounts of plasmids were transfected into MARC-145 cells for the indicated time (24, 36, or 48 h). The overexpression of the proteins was verified by the fluorescence observation and Western blot assay with the GFP antibody. The plasmid pEGFP-N1 was used as the negative control. The recombinant vectors pFLAG-RIG-I and pFLAG-MDA5, expressing RIG-I and MDA5, respectively, were saved in our lab.

### Western blot analysis.

The total proteins of the MARC-145 cells infected with PEDV, transfected with recombinant plasmids or poly(I:C), were harvested with the radioimmunoprecipitation assay (RIPA) lysis and extraction buffer (ThermoFisher, USA) with the protease inhibitor phenylmethylsulfonyl fluoride (PMSF). The same amount of proteins were analyzed by SDS-PAGE and then prepared for Western blot analysis. The PEDV anti-N monoclonal antibody, anti-GFP antibody, anti-FLAG antibody, anti-RIG-I, anti-IKKε, anti-STAT1, anti-pSTAT1 (phosphorylated STAT1), or anti-β-actin was used as the primary antibody, and then the horseradish peroxidase (HRP) goat anti-rabbit or goat anti-mouse IgG antibody acted as the second antibody. The chemiluminescence was excited with the ECL regent (Thermo, USA) and detected with the ChemiDoc touch imaging system (Bio-Rad, USA).

### RT-qPCR.

Briefly, total RNA from the MARC-145 cells infected with PEDV or the gene silencing cells was extracted using the RNAiso Plus reagent (TaKaRa, Dalian, China), according to the manufacturer’s protocols. The 1 μg total RNA (NanoDrop 2000c, Thermo, USA) was performed the reverse transcription reaction to synthesize cDNA (PrimeScript RT reagent kit, TaKaRa, Dalian, China). Then, the SYBR green real-time qPCR was carried out (SYBR Premix Ex Taq II, TaKaRa, Dalian, China) on an ABI 7300 real-time PCR system. The primers are listed in Table S4, and the β-actin gene was served as the endogenous control. The 2^−ΔΔCt^ method was used to calculate the relative transcript levels of the target gene.

### siRNA knockdown assay.

The siRNA-mediated gene silence was conducted to knockdown the endogenous *OASL* and *RNaseL* genes on MARC-145 cells, and the procedures were prepared as the previous described (19). Each gene was designed three pairs of siRNAs (GenePharma, Shanghai, China) targeting the different sites, and a scrambled siRNA (SiNC) was used as the control. The sequences against the *OASL* gene were as follows: Si1-F, GCAGAGAAAUUUCGUGAAATT; Si1-R, UUUCACGAAAUUUCUCUGCTT; Si2-F, GGAAAUGGGUACUGAAGAATT; Si2-R, UUCUUCAGUACCCAUUUCCTT; Si3-F, GCAGCAGCUAGAAUUCCAATT; and Si3-R, UUGGAAUUCUAGCUGCUGCTT. The sequences against the *RNaseL* gene were as follows: Si1-F, GCAAGAGCACAUAGAGAUUTT; Si1-R, AAUCUCUAUGUGCUCUUGCTT; Si2-F, GGAAGUCUCUUGUCUGCAATT; Si2-R, UUGCAGACAAGAGACUUCCTT; Si3-F, GCCCGAAAUGUCCUGUCAUTT; and Si3-R, AUGACAGGACAUUUCGGGCTT. The knockdown efficiency was evaluated by RT-qPCR or Western blot analysis, and then the cells were treated under the corresponding experiments.

### Co-IP assay.

The MARC-145 cells were cultured to 70 to 90% confluence, and then the cells were cotransfected with the N1-OASL and pFLAG-RIG-I or pFLAG-MDA5 recombinant plasmids using Lipofectamine 2000 (Invitrogen, USA). The total cell proteins were collected 24 h later, and the interaction of OASL with RIG-I or MDA5 was checked with a co-immunoprecipitation (Co-IP) kit (Thermo, USA). The FLAG antibody was used to incubate the protein A/G-agarose beads and the cell proteins complex, and then the anti-GFP antibody was used as the primary antibody in the subsequent Western blot assay.

### Statistical analysis.

Statistical analyses of all the data were implemented with GraphPad Prism5 software (La Jolla, CA, USA). A *P* value < 0.05 was considered statistically significant and is labeled with an asterisk in the figures.

### Data availability.

All data sets generated from the transcriptome analysis in this study can be found in the GenBank database under the accession number from SRR13154018 to SRR13154026 of BioProject PRJNA681069.
