# Supplementary material for: Screening Host Antiviral Proteins under the Enhanced Immune Responses Induced by a Variant Strain of Porcine Epidemic Diarrhea Virus
Source: Microbiol Spectr. 2022 Jun 28;10(4):e00661-22. doi: 10.1128/spectrum.00661-22 (PMC9430966; doi:10.1128/spectrum.00661-22)
Supplement: Supplemental file 1 — Fig. S1-S6 and Tables S1-S4. Download spectrum.00661-22-s0001.pdf, PDF file, 4.7 MB [file spectrum.00661-22-s0001.pdf]

Screening host antiviral proteins under the enhanced immune responses induced by a variant strain of porcine epidemic diarrhea virus

Min Sun[1,2, 5]; Zeyanqiu Yu[2,3]; Miao Luo[2,3]; Bin Li[1]; Zihao Pan[2,3,4]; Jiale Ma[2,3,4,*]; Huochun Yao[2,3,4]

[1] Institute of Veterinary Medicine, Jiangsu Academy of Agricultural Sciences, Nanjing 210014, China

[2] College of Veterinary Medicine, Nanjing Agricultural University, Nanjing 210095, China

[3] OIE Reference Lab for Swine Streptococcosis, Nanjing 210095, China

[4] Key Laboratory of Animal Bacteriology, Ministry of Agriculture, Nanjing Agricultural University, Nanjing 210095, China

[5] School of Life Sciences, Jiangsu University, Zhenjiang 212013, China

* Correspondence: Jiale Ma, jialema@njau.edu.cn

Supplementary information

Figure S1 GO analysis of the DEGs in 85-7 or the variant 85-7^{C40} infected MARC-145 cells.

Figure S2 Fluorescence and western blot analyses indicated that the RSAD2, IFIT3, IFI44, IFI35, TRIM16, and the STAT2 were expressed effectively. (A) Fluorescence observation of the recombinant plasmids transfected MARC-145 cells. The MARC-145 cells were transfected with the recombinant plasmids N1-RSAD2, N1-IFIT3, N1-STAT2, N1-IFI35, N1-TRIM16 and N1-IFI44, respectively. At 24h post transfection, the cells were determined for the fluorescence intensity. (B) Western blot analysis of the overexpressed RSAD2, IFIT3, STAT2, IFI35, TRIM16 or IFI44 proteins on MARC-145 cells. The cells were transfected with recombinant plasmids for indicated

time (24h, 36h or 48h) respectively, then the total cell proteins were harvested and subjected to western blot analysis with the anti-GFP antibody. The β-actin was as the internal reference.

Figure S3 The selected ISGs showed no effect on PEDV binding and cell entry.

Figure S4 The expression efficiencies of IFIT2, ISG15, IFI16, USP18 and OASL were verified by fluorescence observation and the western blot analysis. (A) Fluorescence observation of the recombinant plasmids transfected MARC-145 cells. The MARC-145 cells were transfected with the recombinant plasmids N1-IFIT2, N1-ISG15, N1-IFI16, N1-OASL and N1-USP18, respectively. At 24h post transfection, the cells were determined for the fluorescence intensity. (B) Western blot analysis of the overexpressed IFIT2, ISG15, IFI16, OASL or USP18 proteins on MARC-145 cells. The cells were transfected with recombinant plasmids for indicated time (24h, 36h or 48h) respectively, then the total cell proteins were harvested and subjected to western blot analysis with the anti-GFP antibody. The β-actin was as the internal reference.

Figure S5 The knockdown of RNaseL had no effect on the OASL anti-PEDV activity.

Figure S6 The sequence alignment between the human IFI44 protein and the IFI44 protein derived from MARC-145 cells.

Table S1 Lists of the significantly regulated DEGs in 85-7^{C40} infected MARC-145 cells.

Table S2 Lists of the significantly regulated DEGs in 85-7 infected MARC-145 cells.

Table S3 The difference of the genome and encoding proteins between the PEDV 85-7 and variant 85-7^{C40} strain.

Table S4 Primers used in this study.

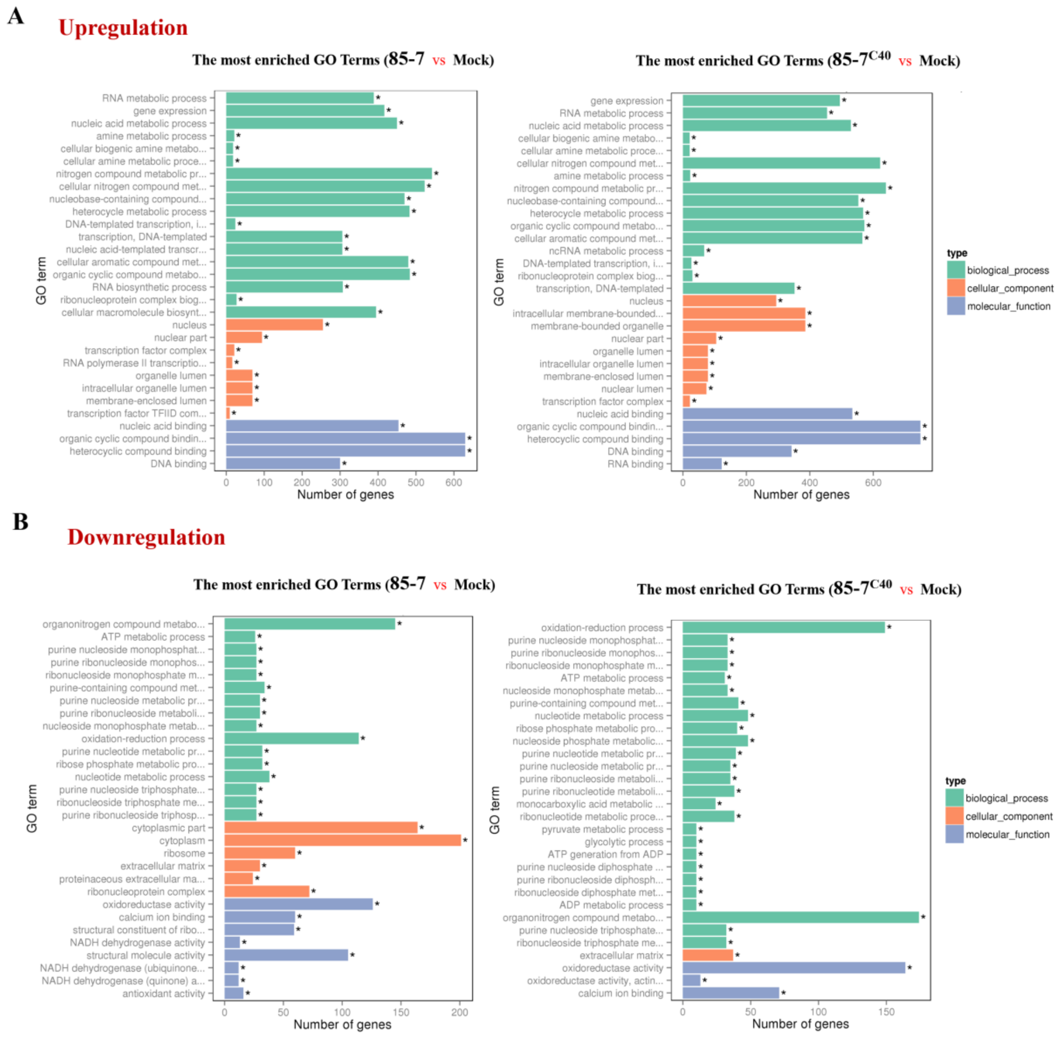

Figure S1 GO analysis of the DEGs in 85-7 or the variant 85-7^{C40} infected MARC-145 cells. The uninfected cells were as the mock control. The green column indicated the biological process (BP), the yellow column indicated the cellular component (CC), and the blue column indicated the molecular function (MF), plotted by number of genes on the horizontal axis and GO term on the vertical. (A) The most enriched GO terms of the up-regulated DEGs in 85-7 (left) or 85-7^{C40} (right) infected cells. The uninfected cells were as the mock control. (B) The most enriched GO terms of the down-regulated DEGs in 85-7 (left) or 85-7^{C40} (right) infected cells. The uninfected cells were as the mock control.

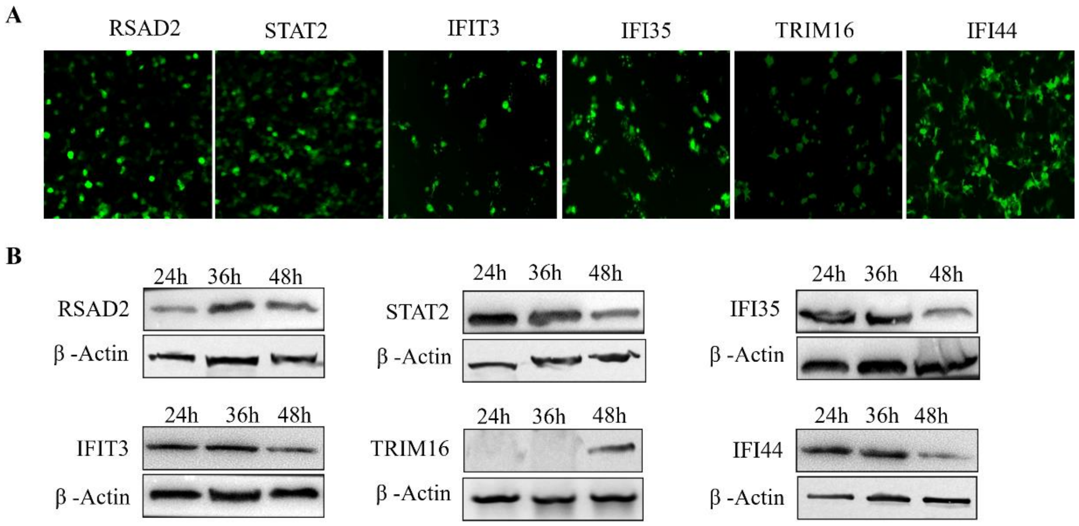

Figure S2 Fluorescence and western blot analyses indicated that the RSAD2, IFIT3, IFI44, IFI35, TRIM16, and the STAT2 were expressed effectively. (A) Fluorescence observation of the recombinant plasmids transfected MARC-145 cells. The MARC-145 cells were transfected with the recombinant plasmids N1-RSAD2, N1-IFIT3, N1-STAT2, N1-IFI35, N1-TRIM16 and N1-IFI44, respectively. At 24h post transfection, the cells were determined for the fluorescence intensity. (B) Western blot analysis of the overexpressed RSAD2, IFIT3, STAT2, IFI35, TRIM16 or IFI44 proteins on MARC-145 cells. The cells were transfected with recombinant plasmids for indicated time (24h, 36h or 48h) respectively, then the total cell proteins were harvested and subjected to western blot analysis with the anti-GFP antibody. The β-actin was as the internal reference.

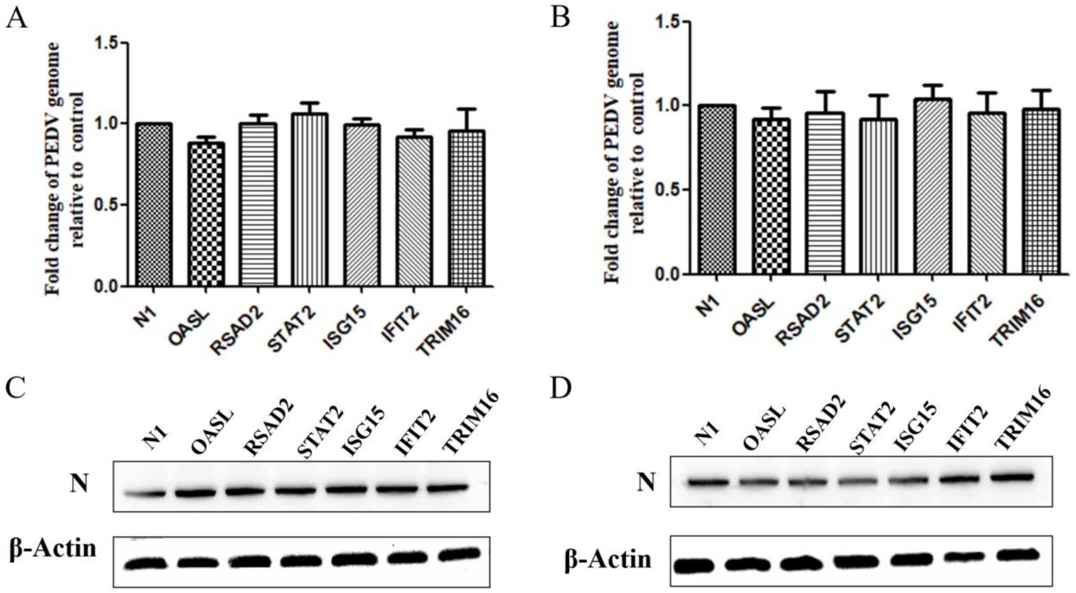

Figure S3 The selected ISGs showed no effect on PEDV binding and cell entry. The recombinant plasmids N1-OASL, N1-RSAD2, N1-STAT2, N1-ISG15, N1-IFIT2 or N1-TRIM16 were transfected into MARC-145 cells, and then infected with PEDV at an MOI of 0.1. The pEGFP-N1 vector was as the control. The PEDV binding and entry analysis were performed as described in the materials and methods section. Then the cells were collected for RT-qPCR and Western blot analysis. (A) The effect of the overexpressed ISG proteins on PEDV binding by detecting the fold change of PEDV genome. (B) The effect of the overexpressed ISG proteins on PEDV entry by detecting the fold change of PEDV genome. (C) The effect of the overexpressed ISG proteins on PEDV binding by detecting the PEDV N protein. (D) The effect of the overexpressed ISG proteins on PEDV entry by detecting the PEDV N protein.

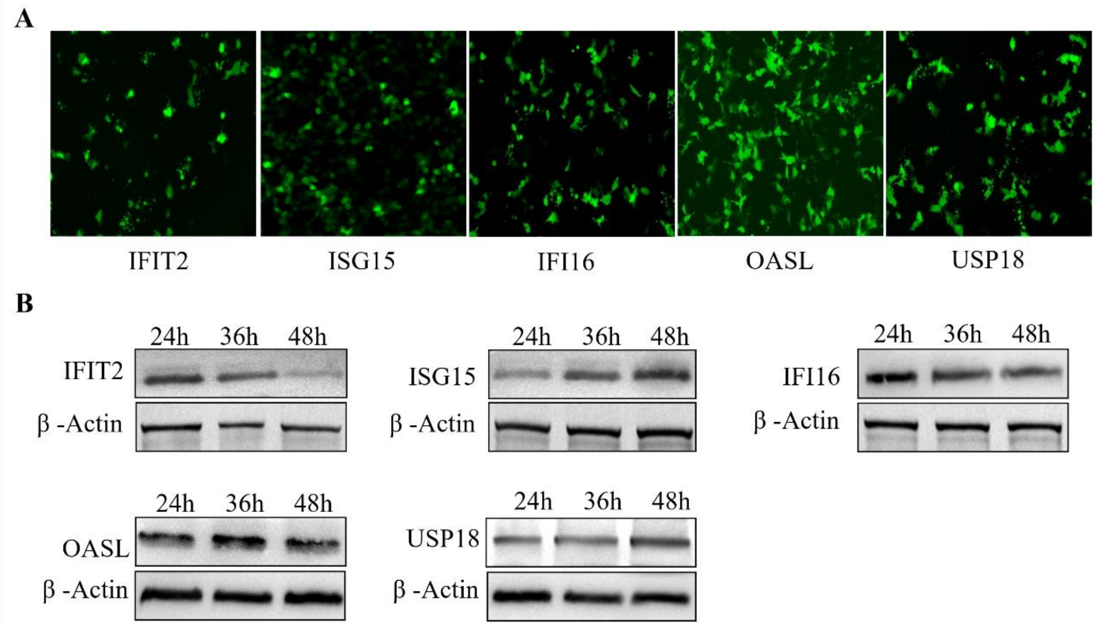

Figure S4 The expression efficiencies of IFIT2, ISG15, IFI16, USP18 and OASL were verified by fluorescence observation and the western blot analysis. (A) Fluorescence observation of the recombinant plasmids transfected MARC-145 cells. The MARC-145 cells were transfected with the recombinant plasmids N1-IFIT2, N1-ISG15, N1-IFI16, N1-OASL and N1-USP18, respectively. At 24h post transfection, the cells were determined for the fluorescence intensity. (B) Western blot analysis of the overexpressed IFIT2, ISG15, IFI16, OASL or USP18 proteins on MARC-145 cells. The cells were transfected with recombinant plasmids for indicated time (24h, 36h or 48h) respectively, then the total cell proteins were harvested and subjected to western blot analysis with the anti-GFP antibody. The β-actin was as the internal reference.

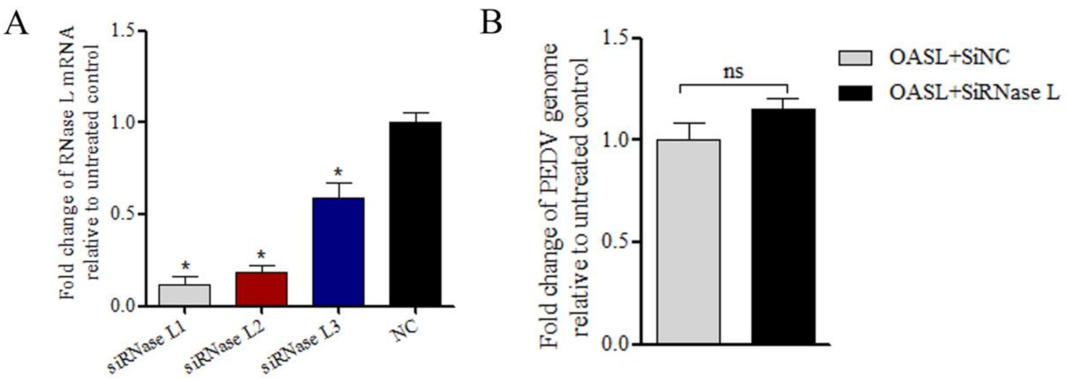

Figure S5 The knockdown of RNaseL had no effect on the OASL anti-PEDV activity. (A) Knockdown of the endogenous RNaseL with the siRNAs. Three pairs of RNaseL-specific siRNAs (Si1, Si2 and Si3) were transfected into the MARC-145 cells, then the mRNA levels of RNaseL were detected by RT-qPCR. The irrelevant siRNA (NC) was used as negative control. (B) Knockdown of RNaseL had no effect on OASL anti-PEDV activity. The N1-OASL and the RNaseL-specific siRNA were co-transfected into MARC-145 cells, and then infected with PEDV at an MOI of 0.1. The cellular supernatant was collected at 24 hpi, and the PEDV genome was detected with RT-qPCR method. The level of PEDV genome in N1-OASL and irrelevant siRNA (NC) co-transfected MARC-145 cells was as the control.

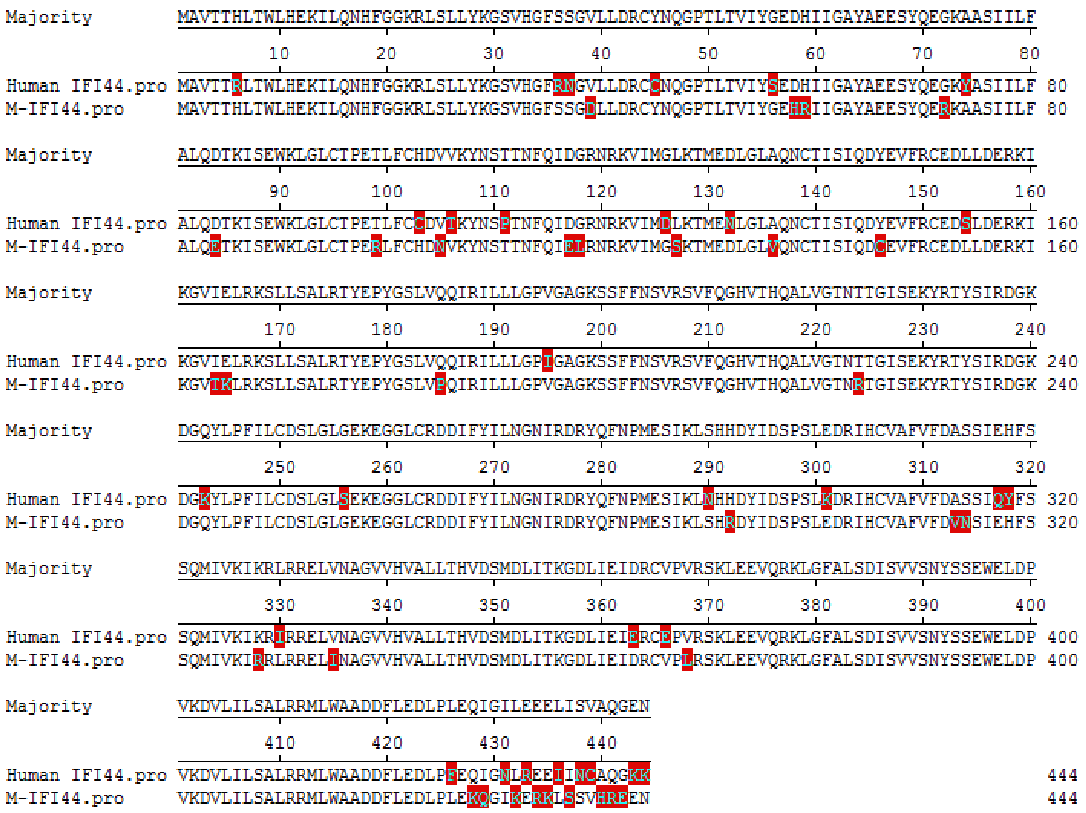

Figure S6 The sequence alignment between the human IFI44 protein and the IFI44 protein derived from MARC-145 cells (M-IFI44). The red mark indicated the mutation site, and the red horizontal lines indicated the deletion region.

Table S1 Lists of the significantly regulated DEGs in 85-7C40 infected MARC-145 cells

Gene_id	log2FoldChange	pval	padj	Blast swiss prot
XM_007960048.1	1.0292	1.90E-37	7.61E-36	sp|O95164|UBL3_HUMAN Ubiquitin-like protein 3 OS=Homo sapiens GN=UBL3 PE=1 SV=1//8.38869e-74
XM_007960058.1	-2.2347	0.0057649	0.028364	sp|Q5W0Q7|USPL1_HUMAN SUMO-specific isopeptidase USPL1 OS=Homo sapiens GN=USPL1 PE=1 SV=1//0
XM_007960060.1	3.1193	6.75E-09	6.67E-08	-//-
XM_007960089.1	3.4714	0.0048804	0.024395	sp|Q9Y3M8|STA13_HUMAN StAR-related lipid transfer protein 13 OS=Homo sapiens GN=STARD13 PE=1 SV=2//0
XM_007960106.1	0.39996	0.0081183	0.038784	sp|P40938|RFC3_HUMAN Replication factor C subunit 3 OS=Homo sapiens GN=RFC3 PE=1 SV=2//0
XM_007960123.1	-1.0817	5.53E-06	4.21E-05	sp|Q9JLM8|DCLK1_MOUSE Serine/threonine-protein kinase DCLK1 OS=Mus musculus GN=Dclk1 PE=1 SV=1//0
XM_007960150.1	0.71717	4.83E-06	3.70E-05	sp|O00287|RFXAP_HUMAN Regulatory factor X-associated protein OS=Homo sapiens GN=RFXAP PE=1 SV=1//6.34309e-93
XM_007960171.1	0.59914	1.43E-05	0.0001034	sp|Q96B26|EXOS8_HUMAN Exosome complex component RRP43 OS=Homo sapiens GN=EXOSC8 PE=1 SV=1//0
XM_007960193.1	0.92837	1.04E-17	1.84E-16	sp|Q5R4N5|UFM1_PONAB Ubiquitin-fold modifier 1 OS=Pongo abelii GN=UFM1 PE=3 SV=1//2.058e-49
XM_007960203.1	0.98875	5.35E-06	4.08E-05	sp|Q9Y693|LHFP_HUMAN Lipoma HMGIC fusion partner OS=Homo sapiens GN=LHFP PE=2 SV=1//8.87905e-93
XM_007960206.1	2.0139	7.75E-62	6.45E-60	sp|Q12778|FOXO1_HUMAN Forkhead box protein O1 OS=Homo sapiens GN=FOXO1 PE=1 SV=2//0
XM_007960218.1	1.5347	5.83E-34	2.08E-32	sp|O75554|WBP4_HUMAN WW domain-binding protein 4 OS=Homo sapiens GN=WBP4 PE=1 SV=1//0
XM_007960222.1	0.54764	5.71E-05	0.0003854	sp|Q86V97|KBTB6_HUMAN Kelch repeat and BTB domain-containing protein 6 OS=Homo sapiens GN=KBTBD6 PE=1 SV=1//0
XM_007960234.1	-3.1435	0.006716	0.032604	sp|Q6N069|NAA16_HUMAN N-alpha-acetyltransferase 16, NatA auxiliary subunit OS=Homo sapiens GN=NAA16 PE=1 SV=2//0
XM_007960317.1	2.8495	9.37E-83	1.26E-80	sp|Q9UHK0|NUFP1_HUMAN Nuclear fragile X mental retardation-interacting protein 1 OS=Homo sapiens GN=NUFIP1 PE=1 SV=2//0
XM_007960319.1	-2.3178	0.0004095	0.002475	sp|Q8IXQ4|GPAM1_HUMAN GPALPP motifs-containing protein 1 OS=Homo sapiens GN=GPALPP1 PE=1 SV=1//0
XM_007960326.1	-0.20289	0.0023753	0.012699	sp|P61288|TCTP_PIG Translationally-controlled tumor protein OS=Sus scrofa GN=TPT1 PE=2 SV=1//1.93386e-121
XM_007960331.1	1.51	1.26E-45	6.69E-44	sp|Q96JB2|COG3_HUMAN Conserved oligomeric Golgi complex subunit 3 OS=Homo sapiens GN=COG3 PE=1 SV=3//0
XM_007960379.1	-0.89572	5.81E-34	2.08E-32	sp|Q5R876|ITM2B_PONAB Integral membrane protein 2B OS=Pongo abelii GN=ITM2B PE=2 SV=1//3.23778e-155
XM_007960389.1	1.2612	3.70E-06	2.87E-05	sp|Q4R683|CDAC1_MACFA Cytidine and dCMP deaminase domain-containing protein 1 OS=Macaca fascicularis GN=CDADC1 PE=2 SV=1//0
XM_007960415.1	-0.60464	9.24E-05	0.0006096	sp|Q9BY08|EBPL_HUMAN Emopamil-binding protein-like OS=Homo sapiens GN=EBPL PE=1 SV=1//4.0047e-110
XM_007960420.1	-1.3891	2.96E-06	2.32E-05	sp|Q8N5I3|KCNRG_HUMAN Potassium channel regulatory protein OS=Homo sapiens GN=KCNRG PE=1 SV=1//3.2983e-161
XM_007960440.1	1.4809	4.46E-17	7.59E-16	sp|Q9UL03|INT6_HUMAN Integrator complex subunit 6 OS=Homo sapiens GN=INTS6 PE=1 SV=1//0
XM_007960444.1	1.1495	0.0012397	0.0069964	sp|Q5RA50|F124A_PONAB Protein FAM124A OS=Pongo abelii GN=FAM124A PE=2 SV=1//0
XM_007960449.1	1.4289	1.38E-35	5.22E-34	sp|Q9H501|ESF1_HUMAN ESF1 homolog OS=Homo sapiens GN=ESF1 PE=1 SV=1//0
XM_007960511.1	0.56588	0.0016277	0.008978	sp|Q9NZU0|FLRT3_HUMAN Leucine-rich repeat transmembrane protein FLRT3 OS=Homo sapiens GN=FLRT3 PE=1 SV=1//0
XM_007960515.1	1.334	7.27E-28	2.07E-26	sp|O14917|PCD17_HUMAN Protocadherin-17 OS=Homo sapiens GN=PCDH17 PE=2 SV=2//0
XM_007960530.1	0.47053	1.09E-05	8.02E-05	sp|Q8WXF1|PSPC1_HUMAN Paraspeckle component 1 OS=Homo sapiens GN=PSPC1 PE=1 SV=1//4.28468e-115
XM_007960538.1	-1.4216	1.15E-07	1.03E-06	sp|P63159|HMGB1_RAT High mobility group protein B1 OS=Rattus norvegicus GN=Hmgb1 PE=1 SV=2//8.18615e-82
XM_007960539.1	-0.88111	2.57E-27	7.16E-26	sp|P07156|HMGB1_CRIGR High mobility group protein B1 (Fragment) OS=Cricetulus griseus GN=HMGB1 PE=1 SV=1//3.87754e-27
XM_007960540.1	0.8822	2.27E-10	2.54E-09	sp|Q92665|RT31_HUMAN 28S ribosomal protein S31, mitochondrial OS=Homo sapiens GN=MRPS31 PE=1 SV=3//1.03497e-155
XM_007960553.1	1.2582	5.66E-05	0.0003818	sp|Q9HC56|PCDH9_HUMAN Protocadherin-9 OS=Homo sapiens GN=PCDH9 PE=1 SV=2//0
XM_007960563.1	0.91448	2.46E-12	3.17E-11	sp|Q6PGQ7|BORA_HUMAN Protein aurora borealis OS=Homo sapiens GN=BORA PE=1 SV=2//0
XM_007960564.1	1.818	3.68E-16	5.97E-15	sp|Q08AG7|MZT1_HUMAN Mitotic-spindle organizing protein 1 OS=Homo sapiens GN=MZT1 PE=1 SV=2//3.75449e-32

XM_007960571.1	3.6002	3.49E-119	8.60E-117	sp|Q13887|KLF5_HUMAN Krueppel-like factor 5 OS=Homo sapiens GN=KLF5 PE=1 SV=2//0
XM_007960578.1	-1.1155	4.56E-09	4.57E-08	sp|Q7Z4G1|COMD6_HUMAN COMM domain-containing protein 6 OS=Homo sapiens GN=COMMD6 PE=1 SV=1//8.57457e-56
XM_007960601.1	-0.36672	3.63E-07	3.09E-06	sp|Q96CX2|KCD12_HUMAN BTB/POZ domain-containing protein KCTD12 OS=Homo sapiens GN=KCTD12 PE=1 SV=1//0
XM_007960608.1	-0.6255	1.82E-05	0.0001296	sp|O75503|CLN5_HUMAN Ceroid-lipofuscinosis neuronal protein 5 OS=Homo sapiens GN=CLN5 PE=1 SV=2//0
XM_007960664.1	0.63428	3.18E-15	4.84E-14	sp|Q9NV92|NFIP2_HUMAN NEDD4 family-interacting protein 2 OS=Homo sapiens GN=NDFIP2 PE=1 SV=2//9.73781e-155
XM_007960665.1	2.9527	2.76E-17	4.75E-16	sp|Q866R9|SPY2_CHLAE Protein sprouty homolog 2 OS=Chlorocebus aethiops GN=SPRY2 PE=2 SV=1//0
XM_007960669.1	3.2629	0.0070075	0.033892	sp|Q866R9|SPY2_CHLAE Protein sprouty homolog 2 OS=Chlorocebus aethiops GN=SPRY2 PE=2 SV=1//0
XM_007960691.1	1.0419	6.18E-13	8.24E-12	sp|Q86V85|GP180_HUMAN Integral membrane protein GPR180 OS=Homo sapiens GN=GPR180 PE=2 SV=1//0
XM_007960703.1	-1.49	2.47E-05	0.0001733	sp|Q86YF9|DZIP1_HUMAN Zinc finger protein DZIP1 OS=Homo sapiens GN=DZIP1 PE=1 SV=1//0
XM_007960704.1	1.6434	2.01E-46	1.11E-44	sp|Q13217|DNJC3_HUMAN DnaJ homolog subfamily C member 3 OS=Homo sapiens GN=DNAJC3 PE=1 SV=1//0
XM_007960721.1	0.22987	0.0036791	0.018891	sp|O00410|IPO5_HUMAN Importin-5 OS=Homo sapiens GN=IPO5 PE=1 SV=4//0
XM_007960755.1	-0.6825	2.63E-19	5.07E-18	sp|P60982|DEST_PIG Destrin OS=Sus scrofa GN=DSTN PE=1 SV=3
XM_007960779.1	1.5345	1.25E-05	9.10E-05	sp|Q12934|BFSP1_HUMAN Filensin OS=Homo sapiens GN=BFSP1 PE=1 SV=3//0
XM_007960854.1	1.5339	3.81E-80	4.89E-78	sp|P52799|EFNB2_HUMAN Ephrin-B2 OS=Homo sapiens GN=EFNB2 PE=1 SV=1//0
XM_007960867.1	1.1866	7.60E-20	1.50E-18	sp|Q7L211|ABHDD_HUMAN Alpha/beta hydrolase domain-containing protein 13 OS=Homo sapiens GN=ABHD13 PE=2 SV=1//0
XM_007960879.1	2.7453	1.55E-192	7.06E-190	sp|Q9Y4H2|IRS2_HUMAN Insulin receptor substrate 2 OS=Homo sapiens GN=IRS2 PE=1 SV=2//0
XM_007960880.1	2.0093	1.69E-27	4.76E-26	sp|Q9NX57|RAB20_HUMAN Ras-related protein Rab-20 OS=Homo sapiens GN=RAB20 PE=1 SV=1//1.19818e-164
XM_007960882.1	-0.45023	4.32E-10	4.74E-09	sp|P08572|CO4A2_HUMAN Collagen alpha-2(IV) chain OS=Homo sapiens GN=COL4A2 PE=1 SV=4//8.79501e-06
XM_007960883.1	-1.4956	3.15E-51	1.99E-49	sp|Q7SIB2|CO4A1_BOVIN Collagen alpha-1(IV) chain (Fragment) OS=Bos taurus GN=COL4A1 PE=1 SV=1//1.71235e-151
XM_007960891.1	0.9318	2.46E-09	2.53E-08	sp|Q9HA77|SYCM_HUMAN Probable cysteine--tRNA ligase, mitochondrial OS=Homo sapiens GN=CARS2 PE=1 SV=1//0
XM_007960902.1	-2.3629	4.17E-07	3.53E-06	sp|Q9NXR5|ANR10_HUMAN Ankyrin repeat domain-containing protein 10 OS=Homo sapiens GN=ANKRD10 PE=1 SV=2//4.02628e-104
XM_007960903.1	Inf	1.72E-05	0.0001231	sp|Q8IVM7|CM029_HUMAN Putative uncharacterized protein encoded by LINC00346 OS=Homo sapiens GN=LINC00346 PE=5 SV=1//2.56787e-87
XM_007960922.1	-1.2993	3.73E-61	2.99E-59	sp|O75197|LRP5_HUMAN Low-density lipoprotein receptor-related protein 5 OS=Homo sapiens GN=LRP5 PE=1 SV=2//7.2502e-117
XM_007960942.1	-2.6792	4.64E-05	0.0003164	sp|P98196|AT11A_HUMAN Probable phospholipid-transporting ATPase IH OS=Homo sapiens GN=ATP11A PE=1 SV=3//0
XM_007960950.1	Inf	0.0019825	0.010741	sp|O15068|MCF2L_HUMAN Guanine nucleotide exchange factor DBS OS=Homo sapiens GN=MCF2L PE=1 SV=2//0
XM_007960989.1	-0.53122	2.42E-12	3.11E-11	sp|P11279|LAMP1_HUMAN Lysosome-associated membrane glycoprotein 1 OS=Homo sapiens GN=LAMP1 PE=1 SV=3//0
XM_007961011.1	-1.2902	6.65E-24	1.60E-22	sp|Q14393|GAS6_HUMAN Growth arrest-specific protein 6 OS=Homo sapiens GN=GAS6 PE=1 SV=2//0
XM_007961039.1	-1.0083	0.0033616	0.017423	sp|O60343|TBCD4_HUMAN TBC1 domain family member 4 OS=Homo sapiens GN=TBC1D4 PE=1 SV=2//0
XM_007961040.1	-1.2641	1.28E-07	1.13E-06	sp|Q15319|PO4F3_HUMAN POU domain, class 4, transcription factor 3 OS=Homo sapiens GN=POU4F3 PE=1 SV=1//1.11342e-122
XM_007961045.1	-1.2107	1.53E-05	0.0001101	sp|Q8WV15|T255B_HUMAN Transmembrane protein 255B OS=Homo sapiens GN=TMEM255B PE=2 SV=1//3.67686e-142
XM_007961077.1	-3.4008	0.0001062	0.0006945	sp|P48764|SL9A3_HUMAN Sodium/hydrogen exchanger 3 OS=Homo sapiens GN=SLC9A3 PE=1 SV=2//1.31531e-72
XM_007961092.1	0.5353	0.0001171	0.000761	sp|Q15645|PCH2_HUMAN Pachytene checkpoint protein 2 homolog OS=Homo sapiens GN=TRIP13 PE=1 SV=2//0
XM_007961098.1	1.2357	2.66E-07	2.28E-06	sp|Q9H1D9|RPC6_HUMAN DNA-directed RNA polymerase III subunit RPC6 OS=Homo sapiens GN=POLR3F PE=1 SV=1//0
XM_007961146.1	-1.3294	1.48E-07	1.31E-06	sp|Q4R5X8|NDUS6_MACFA NADH dehydrogenase [ubiquinone] iron-sulfur protein 6, mitochondrial OS=Macaca fascicularis GN=NDUFS6 PE=2 SV=1//6.99203e-87
XM_007961159.1	-0.56988	2.21E-06	1.75E-05	sp|O75884|RBBP9_HUMAN Putative hydrolase RBBP9 OS=Homo sapiens GN=RBBP9 PE=1 SV=2//9.25207e-115
XM_007961166.1	0.60754	4.24E-06	3.27E-05	sp|Q4R6W4|MED10_MACFA Mediator of RNA polymerase II transcription subunit 10 OS=Macaca fascicularis GN=MED10 PE=2 SV=1//2.86225e-91
XM_007961171.1	-1.8252	0.0002798	0.0017235	sp|Q28891|S5A1_MACFA 3-oxo-5-alpha-steroid 4-dehydrogenase 1 OS=Macaca

				fascicularis GN=SRD5A1 PE=1 SV=1//9.0037e-164
XM_007961215.1	1.176	1.31E-18	2.44E-17	sp|A6NCL7|AN33B_HUMAN Ankyrin repeat domain-containing protein 33B OS=Homo sapiens GN=ANKRD33B PE=3 SV=1//0
XM_007961216.1	-0.93154	2.84E-15	4.33E-14	sp|P51397|DAP1_HUMAN Death-associated protein 1 OS=Homo sapiens GN=DAP PE=1 SV=3//4.15896e-54
XM_007961243.1	1.4201	1.50E-61	1.24E-59	sp|Q96BN8|OTUL_HUMAN Ubiquitin thioesterase otulin OS=Homo sapiens GN=OTULIN PE=1 SV=3//0
XM_007961245.1	-0.6482	5.80E-10	6.26E-09	sp|Q9UJT9|FBXL7_HUMAN F-box/LRR-repeat protein 7 OS=Homo sapiens GN=FBXL7 PE=2 SV=1//0
XM_007961248.1	1.3822	1.49E-60	1.18E-58	sp|Q9HD67|MYO10_HUMAN Unconventional myosin-X OS=Homo sapiens GN=MYO10 PE=1 SV=3//0
XM_007961251.1	0.88358	2.17E-18	3.98E-17	sp|Q969S3|ZN622_HUMAN Zinc finger protein 622 OS=Homo sapiens GN=ZNF622 PE=1 SV=1//0
XM_007961271.1	-1.1429	4.69E-55	3.24E-53	sp|P55285|CADH6_HUMAN Cadherin-6 OS=Homo sapiens GN=CDH6 PE=1 SV=1//0
XM_007961272.1	-1.2498	2.00E-10	2.25E-09	sp|P55285|CADH6_HUMAN Cadherin-6 OS=Homo sapiens GN=CDH6 PE=1 SV=1//0
XM_007961302.1	-0.32391	0.0002681	0.0016552	sp|Q5R6D0|TCP4_PONAB Activated RNA polymerase II transcriptional coactivator p15 OS=Pongo abelii GN=SUB1 PE=2 SV=1//4.69895e-50
XM_007961333.1	1.304	3.79E-08	3.52E-07	sp|Q8TDN6|BRX1_HUMAN Ribosome biogenesis protein BRX1 homolog OS=Homo sapiens GN=BRIX1 PE=1 SV=2//0
XM_007961334.1	0.80674	6.19E-08	5.64E-07	sp|Q5R7X9|RAD1_PONAB Cell cycle checkpoint protein RAD1 OS=Pongo abelii GN=RAD1 PE=2 SV=1//0
XM_007961351.1	-0.32863	0.0026772	0.014171	sp|P61600|NAA20_MOUSE N-alpha-acetyltransferase 20 OS=Mus musculus GN=Naa20 PE=2 SV=1//6.71712e-127
XM_007961378.1	0.41262	0.0034839	0.017981	sp|Q68DH5|LMBD2_HUMAN LMBR1 domain-containing protein 2 OS=Homo sapiens GN=LMBRD2 PE=2 SV=1//0
XM_007961392.1	1.7097	1.10E-31	3.58E-30	sp|P43003|EAA1_HUMAN Excitatory amino acid transporter 1 OS=Homo sapiens GN=SLC1A3 PE=1 SV=1//0
XM_007961398.1	1.8322	0.0001784	0.00113	sp|Q6KC79|NIPBL_HUMAN Nipped-B-like protein OS=Homo sapiens GN=NIPBL PE=1 SV=2//1.32289e-08
XM_007961415.1	0.88645	4.59E-23	1.07E-21	sp|O75694|NU155_HUMAN Nuclear pore complex protein Nup155 OS=Homo sapiens GN=NUP155 PE=1 SV=1//0
XM_007961444.1	0.99579	6.84E-10	7.33E-09	sp|Q99650|OSMR_HUMAN Oncostatin-M-specific receptor subunit beta OS=Homo sapiens GN=OSMR PE=1 SV=1//0
XM_007961451.1	2.1465	0.007054	0.034094	sp|Q01101|INSM1_HUMAN Insulinoma-associated protein 1 OS=Homo sapiens GN=INSM1 PE=1 SV=1//0
XM_007961467.1	0.53923	5.39E-06	4.11E-05	sp|Q95KZ0|PE2R4_PANTR Prostaglandin E2 receptor EP4 subtype OS=Pan troglodytes GN=PTGER4 PE=2 SV=1//0
XM_007961482.1	0.49599	9.70E-07	7.96E-06	sp|A6NDU8|CE051_HUMAN UPF0600 protein C5orf51 OS=Homo sapiens GN=C5orf51 PE=1 SV=1//1.95727e-168
XM_007961534.1	1.0093	1.40E-19	2.72E-18	sp|Q9NP92|RT30_HUMAN 28S ribosomal protein S30, mitochondrial OS=Homo sapiens GN=MRPS30 PE=1 SV=2//0
XM_007961537.1	-0.28475	0.003686	0.018921	sp|P31040|SDHA_HUMAN Succinate dehydrogenase [ubiquinone] flavoprotein subunit, mitochondrial OS=Homo sapiens GN=SDHA PE=1 SV=2//2.04686e-150
XM_007961543.1	-0.54095	1.41E-09	1.48E-08	sp|Q8TE57|ATS16_HUMAN A disintegrin and metalloproteinase with thrombospondin motifs 16 OS=Homo sapiens GN=ADAMTS16 PE=2 SV=3//9.63584e-20
XM_007961547.1	0.79122	0.0002606	0.001612	-//-
XM_007961556.1	-1.1322	0.0031769	0.016564	sp|Q86YL5|TDRP_HUMAN Testis development-related protein OS=Homo sapiens GN=TDRP PE=1 SV=2//7.2165e-69
XM_007961592.1	-0.70899	0.0055478	0.027444	sp|O94819|KBTBB_HUMAN Kelch repeat and BTB domain-containing protein 11 OS=Homo sapiens GN=KBTBD11 PE=1 SV=1//0
XM_007961625.1	-2.4465	0.0003288	0.0020096	sp|A8MXJ8|F90A5_HUMAN Putative protein FAM90A5P OS=Homo sapiens GN=FAM90A5P PE=5 SV=1//4.63236e-148
XM_007961633.1	-0.90167	7.73E-18	1.38E-16	sp|Q4R5M2|CATB_MACFA Cathepsin B OS=Macaca fascicularis GN=CTSB PE=2 SV=1//0
XM_007961651.1	-2.0068	0.010045	0.047044	sp|Q96KS9|F167A_HUMAN Protein FAM167A OS=Homo sapiens GN=FAM167A PE=1 SV=1//1.22997e-118
XM_007961656.1	0.40642	0.0036591	0.018792	sp|Q96QG7|MTMR9_HUMAN Myotubularin-related protein 9 OS=Homo sapiens GN=MTMR9 PE=1 SV=1//0
XM_007961657.1	1.7454	5.65E-12	7.12E-11	sp|Q96BK5|PINX1_HUMAN PIN2/TERF1-interacting telomerase inhibitor 1 OS=Homo sapiens GN=PINX1 PE=1 SV=2//0
XM_007961676.1	0.45774	9.61E-06	7.10E-05	sp|O95271|TNKS1_HUMAN Tankyrase-1 OS=Homo sapiens GN=TNKS PE=1 SV=2//0
XM_007961691.1	1.5468	4.56E-12	5.78E-11	sp|Q86YV5|SG223_HUMAN Tyrosine-protein kinase SgK223 OS=Homo sapiens GN=SGK223 PE=1 SV=4//0
XM_007961732.1	1.0508	5.87E-23	1.36E-21	sp|Q8NEZ2|VP37A_HUMAN Vacuolar protein sorting-associated protein 37A OS=Homo sapiens GN=VPS37A PE=1 SV=1//0
XM_007961733.1	0.44359	1.27E-06	1.03E-05	sp|Q9UIV1|CNOT7_HUMAN CCR4-NOT transcription complex subunit 7 OS=Homo sapiens GN=CNOT7 PE=1 SV=3//0
XM_007961748.1	1.6102	1.78E-21	3.81E-20	sp|Q2PFX1|PGFRL_MACFA Platelet-derived growth factor receptor-like protein OS=Macaca fascicularis GN=PDGFRL PE=2 SV=1//0

XM_007961775.1	1.3338	2.88E-15	4.38E-14	sp|Q9UKK6|NXT1_HUMAN NTF2-related export protein 1 OS=Homo sapiens GN=NXT1 PE=1 SV=1//7.58403e-98
XM_007961814.1	1.1076	2.22E-43	1.08E-41	sp|P21281|VATB2_HUMAN V-type proton ATPase subunit B, brain isoform OS=Homo sapiens GN=ATP6V1B2 PE=1 SV=3//0
XM_007961873.1	-1.2511	1.68E-11	2.05E-10	sp|P13497|BMP1_HUMAN Bone morphogenetic protein 1 OS=Homo sapiens GN=BMP1 PE=1 SV=2//0
XM_007961900.1	1.4204	7.65E-39	3.21E-37	sp|P48454|PP2BC_HUMAN Serine/threonine-protein phosphatase 2B catalytic subunit gamma isoform OS=Homo sapiens GN=PPP3CC PE=1 SV=3//0
XM_007961907.1	0.45317	0.0017749	0.0097227	sp|Q9NQY0|BIN3_HUMAN Bridging integrator 3 OS=Homo sapiens GN=BIN3 PE=1 SV=1//4.50806e-178
XM_007961908.1	-1.4781	4.62E-104	9.38E-102	sp|O19092|CYTC_MACMU Cystatin-C OS=Macaca mulatta GN=CST3 PE=2 SV=1//1.26632e-78
XM_007961930.1	2.3991	1.95E-28	5.68E-27	sp|Q9UBN6|TR10D_HUMAN Tumor necrosis factor receptor superfamily member 10D OS=Homo sapiens GN=TNFRSF10D PE=1 SV=1//3.73838e-23
XM_007961931.1	2.5079	3.49E-34	1.26E-32	sp|Q9UBN6|TR10D_HUMAN Tumor necrosis factor receptor superfamily member 10D OS=Homo sapiens GN=TNFRSF10D PE=1 SV=1//7.12558e-10
XM_007961932.1	1.7317	5.33E-13	7.12E-12	sp|O00220|TR10A_HUMAN Tumor necrosis factor receptor superfamily member 10A OS=Homo sapiens GN=TNFRSF10A PE=1 SV=3//0
XM_007961936.1	-0.93189	1.42E-37	5.74E-36	sp|Q9Y4K0|LOXL2_HUMAN Lysyl oxidase homolog 2 OS=Homo sapiens GN=LOXL2 PE=1 SV=1//0
XM_007961944.1	1.1844	3.02E-09	3.07E-08	sp|A6NCS4|NKX26_HUMAN Homeobox protein Nkx-2.6 OS=Homo sapiens GN=NKX2-6 PE=1 SV=1//3.2728e-153
XM_007961968.1	1.1589	6.56E-28	1.87E-26	sp|Q7L273|KCTD9_HUMAN BTB/POZ domain-containing protein KCTD9 OS=Homo sapiens GN=KCTD9 PE=1 SV=1//0
XM_007961971.1	1.7695	2.72E-48	1.57E-46	sp|P63150|2ABA_RABIT Serine/threonine-protein phosphatase 2A 55 kDa regulatory subunit B alpha isoform OS=Oryctolagus cuniculus GN=PPP2R2A PE=2 SV=1//0
XM_007961978.1	-2.7438	1.29E-05	9.42E-05	sp|Q5R9Y6|DPYL2_PONAB Dihydropyrimidinase-related protein 2 OS=Pongo abelii GN=DPYSL2 PE=2 SV=1//0
XM_007961987.1	0.90004	3.04E-08	2.85E-07	sp|Q9UPQ4|TRI35_HUMAN Tripartite motif-containing protein 35 OS=Homo sapiens GN=TRIM35 PE=1 SV=2//0
XM_007962035.1	-1.3262	6.81E-21	1.42E-19	sp|Q9H8N7|ZN395_HUMAN Zinc finger protein 395 OS=Homo sapiens GN=ZNF395 PE=1 SV=2//0
XM_007962075.1	3.7127	2.39E-74	2.64E-72	sp|Q13115|DUS4_HUMAN Dual specificity protein phosphatase 4 OS=Homo sapiens GN=DUSP4 PE=1 SV=1//0
XM_007962079.1	0.91385	2.29E-11	2.78E-10	sp|Q5RDE9|LERL1_PONAB Leptin receptor overlapping transcript-like 1 OS=Pongo abelii GN=LEPROTL1 PE=2 SV=1//4.26377e-81
XM_007962088.1	-0.88651	0.0031192	0.016298	sp|Q93062|RBPMS_HUMAN RNA-binding protein with multiple splicing OS=Homo sapiens GN=RBPMS PE=1 SV=1//5.05022e-109
XM_007962099.1	-0.87527	7.50E-22	1.64E-20	sp|P00390|GSHR_HUMAN Glutathione reductase, mitochondrial OS=Homo sapiens GN=GSR PE=1 SV=2//0
XM_007962122.1	-1.8783	4.77E-06	3.66E-05	sp|Q9UJV8|PURG_HUMAN Purine-rich element-binding protein gamma OS=Homo sapiens GN=PURG PE=2 SV=1//0
XM_007962123.1	#NAME?	0.0031353	0.016374	sp|Q9UJV8|PURG_HUMAN Purine-rich element-binding protein gamma OS=Homo sapiens GN=PURG PE=2 SV=1//9.88136e-174
XM_007962138.1	-1.7147	6.41E-70	6.53E-68	sp|Q96SM3|CPXM1_HUMAN Probable carboxypeptidase X1 OS=Homo sapiens GN=CPXM1 PE=2 SV=2//0
XM_007962173.1	2.5514	2.06E-48	1.20E-46	sp|Q9HAW0|BRF2_HUMAN Transcription factor IIIB 50 kDa subunit OS=Homo sapiens GN=BRF2 PE=1 SV=1//0
XM_007962176.1	1.2759	3.63E-10	4.00E-09	sp|Q6WKZ4|RFIP1_HUMAN Rab11 family-interacting protein 1 OS=Homo sapiens GN=RAB11FIP1 PE=1 SV=3//0
XM_007962183.1	0.6904	3.16E-09	3.20E-08	sp|Q9UBL3|ASH2L_HUMAN Set1/Ash2 histone methyltransferase complex subunit ASH2 OS=Homo sapiens GN=ASH2L PE=1 SV=1//0
XM_007962194.1	0.58867	0.0006632	0.0038859	sp|O15116|LSM1_HUMAN U6 snRNA-associated Sm-like protein LSm1 OS=Homo sapiens GN=LSM1 PE=1 SV=1//3.18254e-87
XM_007962195.1	0.40842	0.0031645	0.016511	sp|Q8NEB5|PPC1B_HUMAN Phosphatidate phosphatase PPAPDC1B OS=Homo sapiens GN=PPAPDC1B PE=1 SV=2//2.59202e-144
XM_007962231.1	1.802	0.00441	0.022306	sp|O75410|TACC1_HUMAN Transforming acidic coiled-coil-containing protein 1 OS=Homo sapiens GN=TACC1 PE=1 SV=2//0
XM_007962242.1	0.92266	3.09E-38	1.26E-36	sp|Q13443|ADAM9_HUMAN Disintegrin and metalloproteinase domain-containing protein 9 OS=Homo sapiens GN=ADAM9 PE=1 SV=1//0
XM_007962277.1	-1.9588	4.48E-19	8.50E-18	sp|Q8N474|SFRP1_HUMAN Secreted frizzled-related protein 1 OS=Homo sapiens GN=SFRP1 PE=1 SV=1//1.05807e-157
XM_007962367.1	-0.52118	1.73E-06	1.39E-05	sp|Q68CP4|HGNAT_HUMAN Heparan-alpha-glucosaminide N-acetyltransferase OS=Homo sapiens GN=HGSNAT PE=1 SV=2//0
XM_007962370.1	-0.38205	7.66E-06	5.74E-05	sp|Q8TDR2|STK35_HUMAN Serine/threonine-protein kinase 35 OS=Homo sapiens GN=STK35 PE=1 SV=2//0
XM_007962371.1	2.6739	3.10E-190	1.39E-187	sp|Q96QB1|RHG07_HUMAN Rho GTPase-activating protein 7 OS=Homo sapiens GN=DLC1 PE=1 SV=4//1.70896e-164
XM_007962376.1	1.9648	5.42E-27	1.49E-25	sp|Q9BXY0|MAK16_HUMAN Protein MAK16 homolog OS=Homo sapiens GN=MAK16 PE=1 SV=2//1.66831e-13

XM_007962381.1	5.7	1.79E-05	0.0001276	sp|Q9BXX3|AN30A_HUMAN Ankyrin repeat domain-containing protein 30A OS=Homo sapiens GN=ANKRD30A PE=2 SV=3//1.32121e-31
XM_007962413.1	0.74782	0.0001187	0.0007705	sp|P17030|ZNF25_HUMAN Zinc finger protein 25 OS=Homo sapiens GN=ZNF25 PE=2 SV=2//0
XM_007962434.1	-1.7363	0.0044147	0.022322	sp|P07949|RET_HUMAN Proto-oncogene tyrosine-protein kinase receptor Ret OS=Homo sapiens GN=RET PE=1 SV=3//0
XM_007962479.1	-2.8188	0.0002942	0.0018057	sp|P17041|ZNF32_HUMAN Zinc finger protein 32 OS=Homo sapiens GN=ZNF32 PE=1 SV=2//1.52308e-06
XM_007962483.1	-0.66828	0.000436	0.0026239	sp|P48061|SDF1_HUMAN Stromal cell-derived factor 1 OS=Homo sapiens GN=CXCL12 PE=1 SV=1//2.24113e-40
XM_007962485.1	0.47093	6.49E-05	0.0004358	sp|P48061|SDF1_HUMAN Stromal cell-derived factor 1 OS=Homo sapiens GN=CXCL12 PE=1 SV=1//6.44916e-43
XM_007962486.1	-2.3082	1.72E-09	1.80E-08	sp|A0PK05|TMM72_HUMAN Transmembrane protein 72 OS=Homo sapiens GN=TMEM72 PE=2 SV=1//8.16914e-164
XM_007962493.1	3.6599	9.79E-60	7.55E-58	sp|Q5RBE4|DEPP_PONAB Protein DEPP OS=Pongo abelii GN=DEPP PE=2 SV=1//7.1583e-109
XM_007962499.1	5.9704	2.29E-34	8.32E-33	sp|Q9BXX2|AN30B_HUMAN Ankyrin repeat domain-containing protein 30B OS=Homo sapiens GN=ANKRD30B PE=2 SV=3//5.0165e-08
XM_007962542.1	-2.4329	0.010149	0.047432	sp|A6NF34|ANTRL_HUMAN Anthrax toxin receptor-like OS=Homo sapiens GN=ANTXRL PE=3 SV=3//0
XM_007962559.1	0.56284	2.27E-09	2.34E-08	sp|O14925|TIM23_HUMAN Mitochondrial import inner membrane translocase subunit Tim23 OS=Homo sapiens GN=TIMM23 PE=1 SV=1//2.67714e-108
XM_007962667.1	2.0143	2.87E-09	2.92E-08	sp|P00367|DHE3_HUMAN Glutamate dehydrogenase 1, mitochondrial OS=Homo sapiens GN=GLUD1 PE=1 SV=2//4.73513e-175
XM_007962669.1	1.1105	1.54E-05	0.0001107	sp|A6NIR3|AGAP5_HUMAN Arf-GAP with GTPase, ANK repeat and PH domain-containing protein 5 OS=Homo sapiens GN=AGAP5 PE=2 SV=2//0
XM_007962672.1	-0.96857	3.40E-20	6.82E-19	sp|P00367|DHE3_HUMAN Glutamate dehydrogenase 1, mitochondrial OS=Homo sapiens GN=GLUD1 PE=1 SV=2//0
XM_007962701.1	0.34331	0.0017897	0.0098002	sp|P36894|BMR1A_HUMAN Bone morphogenetic protein receptor type-1A OS=Homo sapiens GN=BMPR1A PE=1 SV=2//0
XM_007962779.1	-2.5388	0.0076429	0.036665	sp|Q9BRX8|F213A_HUMAN Redox-regulatory protein FAM213A OS=Homo sapiens GN=FAM213A PE=1 SV=3//2.58225e-143
XM_007962818.1	-1.0432	1.20E-11	1.48E-10	sp|Q8N2G6|ZCH24_HUMAN Zinc finger CCHC domain-containing protein 24 OS=Homo sapiens GN=ZCCHC24 PE=1 SV=1//3.57453e-165
XM_007962819.1	1.8371	1.50E-68	1.48E-66	sp|P30405|PPIF_HUMAN Peptidyl-prolyl cis-trans isomerase F, mitochondrial OS=Homo sapiens GN=PPIF PE=1 SV=1//5.67137e-119
XM_007962847.1	1.5217	4.71E-37	1.84E-35	sp|O14802|RPC1_HUMAN DNA-directed RNA polymerase III subunit RPC1 OS=Homo sapiens GN=POLR3A PE=1 SV=2//0
XM_007962905.1	1.8245	1.21E-86	1.76E-84	sp|Q96F45|ZN503_HUMAN Zinc finger protein 503 OS=Homo sapiens GN=ZNF503 PE=1 SV=1//0
XM_007962927.1	0.49282	0.0015558	0.0086267	sp|Q9DA37|SAMD8_MOUSE Sphingomyelin synthase-related protein 1 OS=Mus musculus GN=Samd8 PE=2 SV=1//0
XM_007962987.1	-2.4539	3.22E-06	2.50E-05	sp|Q13555|KCC2G_HUMAN Calcium/calmodulin-dependent protein kinase type II subunit gamma OS=Homo sapiens GN=CAMK2G PE=1 SV=3//0
XM_007963014.1	-0.75162	9.88E-06	7.29E-05	sp|Q96BP2|CHCH1_HUMAN Coiled-coil-helix-coiled-coil-helix domain-containing protein 1 OS=Homo sapiens GN=CHCHD1 PE=1 SV=1//1.97474e-62
XM_007963021.1	-2.2227	0.0004359	0.0026239	sp|Q495W5|FUT11_HUMAN Alpha-(1,3)-fucosyltransferase 11 OS=Homo sapiens GN=FUT11 PE=1 SV=1//0
XM_007963072.1	0.65899	6.14E-12	7.70E-11	sp|Q8WXX5|DNJC9_HUMAN DnaJ homolog subfamily C member 9 OS=Homo sapiens GN=DNAJC9 PE=1 SV=1//7.7745e-152
XM_007963133.1	-1.406	2.46E-06	1.94E-05	sp|Q9H251|CAD23_HUMAN Cadherin-23 OS=Homo sapiens GN=CDH23 PE=1 SV=2//0
XM_007963136.1	0.86309	3.78E-11	4.49E-10	sp|Q9H7M9|GI24_HUMAN Platelet receptor Gi24 OS=Homo sapiens GN=C10orf54 PE=1 SV=3//0
XM_007963148.1	-0.84753	1.66E-09	1.73E-08	sp|O95470|SGPL1_HUMAN Sphingosine-1-phosphate lyase 1 OS=Homo sapiens GN=SGPL1 PE=1 SV=3//0
XM_007963167.1	-1.8481	1.79E-102	3.43E-100	sp|Q13542|4EBP2_HUMAN Eukaryotic translation initiation factor 4E-binding protein 2 OS=Homo sapiens GN=EIF4EBP2 PE=1 SV=1//2.44207e-67
XM_007963178.1	0.83749	8.69E-22	1.89E-20	sp|Q4R543|IPYR_MACFA Inorganic pyrophosphatase OS=Macaca fascicularis GN=PPA1 PE=2 SV=2//0
XM_007963189.1	-0.58981	3.41E-08	3.19E-07	sp|Q8CCK0|H2AW_MOUSE Core histone macro-H2A.2 OS=Mus musculus GN=H2afy2 PE=1 SV=3//0
XM_007963218.1	-0.98499	8.10E-15	1.21E-13	sp|O95858|TSN15_HUMAN Tetraspanin-15 OS=Homo sapiens GN=TSPAN15 PE=1 SV=1//0
XM_007963229.1	1.8762	3.10E-106	6.47E-104	sp|Q8IYB8|SUV3_HUMAN ATP-dependent RNA helicase SUPV3L1, mitochondrial OS=Homo sapiens GN=SUPV3L1 PE=1 SV=1//0
XM_007963230.1	0.49232	1.19E-09	1.26E-08	sp|O75436|VP26A_HUMAN Vacuolar protein sorting-associated protein 26A OS=Homo sapiens GN=VPS26A PE=1 SV=2//0
XM_007963235.1	2.5678	2.42E-225	1.47E-222	sp|Q9NR30|DDX21_HUMAN Nucleolar RNA helicase 2 OS=Homo sapiens GN=DDX21 PE=1 SV=5//0
XM_007963255.1	-2.1509	3.69E-24	8.94E-23	sp|Q8NFU7|TET1_HUMAN Methylcytosine dioxygenase TET1 OS=Homo sapiens GN=TET1 PE=1 SV=2//0

XM_007963275.1	-0.37573	0.0018766	0.010215	sp|P30039|PBLD_HUMAN Phenazine biosynthesis-like domain-containing protein OS=Homo sapiens GN=PBLD PE=1 SV=2//0
XM_007963281.1	-3.0744	0.0097102	0.045643	sp|Q8N100|ATOH7_HUMAN Protein atonal homolog 7 OS=Homo sapiens GN=ATOH7 PE=1 SV=1//1.43872e-60
XM_007963295.1	1.3502	6.33E-45	3.26E-43	sp|Q96EB6|SIR1_HUMAN NAD-dependent protein deacetylase sirtuin-1 OS=Homo sapiens GN=SIRT1 PE=1 SV=2//0
XM_007963312.1	-1.056	1.96E-23	4.63E-22	sp|Q6NUK4|REEP3_HUMAN Receptor expression-enhancing protein 3 OS=Homo sapiens GN=REEP3 PE=1 SV=1//7.16123e-147
XM_007963314.1	1.3319	3.69E-33	1.27E-31	sp|Q15652|JHD2C_HUMAN Probable JmjC domain-containing histone demethylation protein 2C OS=Homo sapiens GN=JMJD1C PE=1 SV=2//0
XM_007963324.1	1.6426	5.22E-57	3.76E-55	sp|Q96SZ5|AEDO_HUMAN 2-aminoethanethiol dioxygenase OS=Homo sapiens GN=ADO PE=1 SV=2//6.75486e-143
XM_007963325.1	0.9399	0.0001807	0.0011434	sp|Q5R9L2|ZN365_PONAB Protein ZNF365 OS=Pongo abelii GN=ZNF365 PE=2 SV=1//0
XM_007963380.1	0.88062	0.0028361	0.014954	sp|Q96FC7|PHIPL_HUMAN Phytanoyl-CoA hydroxylase-interacting protein-like OS=Homo sapiens GN=PHYHIPL PE=1 SV=3//0
XM_007963400.1	0.82141	1.30E-16	2.17E-15	sp|Q4H0T5|TFAM_TRACR Transcription factor A, mitochondrial OS=Trachypithecus cristatus GN=TFAM PE=2 SV=1//8.35155e-146
XM_007963444.1	2.969	6.82E-49	4.05E-47	sp|O94907|DKK1_HUMAN Dickkopf-related protein 1 OS=Homo sapiens GN=DKK1 PE=1 SV=1//9.39e-148
XM_007963445.1	-1.3166	0.010717	0.049873	sp|P40617|ARL4A_HUMAN ADP-ribosylation factor-like protein 4A OS=Homo sapiens GN=ARL4A PE=1 SV=2//1.89065e-136
XM_007963447.1	0.82728	2.92E-11	3.51E-10	sp|Q9H0L4|CSTFT_HUMAN Cleavage stimulation factor subunit 2 tau variant OS=Homo sapiens GN=CSTF2T PE=1 SV=1//0
XM_007963486.1	-1.0755	5.31E-44	2.61E-42	sp|Q9H1C4|UN93B_HUMAN Protein unc-93 homolog B1 OS=Homo sapiens GN=UNC93B1 PE=1 SV=2//0
XM_007963496.1	4.6614	8.93E-76	1.04E-73	sp|P09913|IFIT2_HUMAN Interferon-induced protein with tetratricopeptide repeats 2 OS=Homo sapiens GN=IFIT2 PE=1 SV=1//0
XM_007963497.1	4.9466	7.36E-05	0.000491	sp|A5A6J9|IFIT3_PANTR Interferon-induced protein with tetratricopeptide repeats 3 OS=Pan troglodytes GN=IFIT3 PE=2 SV=1//0
XM_007963498.1	4.466	0.0003541	0.0021573	sp|A5A6J9|IFIT3_PANTR Interferon-induced protein with tetratricopeptide repeats 3 OS=Pan troglodytes GN=IFIT3 PE=2 SV=1//0
XM_007963503.1	3.1166	1.40E-38	5.78E-37	sp|Q13325|IFIT5_HUMAN Interferon-induced protein with tetratricopeptide repeats 5 OS=Homo sapiens GN=IFIT5 PE=1 SV=1//0
XM_007963523.1	4.8334	0	0	sp|Q15327|ANKR1_HUMAN Ankyrin repeat domain-containing protein 1 OS=Homo sapiens GN=ANKRD1 PE=1 SV=2//0
XM_007963529.1	1.4562	1.10E-09	1.16E-08	sp|Q5RD78|HECD2_PONAB Probable E3 ubiquitin-protein ligase HECTD2 OS=Pongo abelii GN=HECTD2 PE=2 SV=1//0
XM_007963533.1	4.3518	6.35E-259	4.97E-256	sp|Q9UQK1|PPR3C_HUMAN Protein phosphatase 1 regulatory subunit 3C OS=Homo sapiens GN=PPP1R3C PE=1 SV=2//0
XM_007963534.1	0.93087	3.93E-35	1.46E-33	sp|Q9H2K2|TNKS2_HUMAN Tankyrase-2 OS=Homo sapiens GN=TNKS2 PE=1 SV=1//0
XM_007963544.1	0.8925	4.22E-19	8.03E-18	sp|Q3KNM2|MARH5_MOUSE E3 ubiquitin-protein ligase MARCH5 OS=Mus musculus GN=March5 PE=2 SV=1//0
XM_007963564.1	0.38957	7.70E-07	6.38E-06	sp|P52732|KIF11_HUMAN Kinesin-like protein KIF11 OS=Homo sapiens GN=KIF11 PE=1 SV=2//0
XM_007963586.1	0.51896	1.97E-15	3.03E-14	sp|Q9NZM1|MYOF_HUMAN Myoferlin OS=Homo sapiens GN=MYOF PE=1 SV=1//0
XM_007963611.1	1.9878	2.48E-07	2.14E-06	sp|Q8WTT2|NOC3L_HUMAN Nucleolar complex protein 3 homolog OS=Homo sapiens GN=NOC3L PE=1 SV=1//0
XM_007963695.1	-0.19241	0.007258	0.034964	sp|Q9HD45|TM9S3_HUMAN Transmembrane 9 superfamily member 3 OS=Homo sapiens GN=TM9SF3 PE=1 SV=2//0
XM_007963697.1	-0.95163	1.27E-16	2.11E-15	sp|A2T7M0|SCND1_PONPY SCAN domain-containing protein 1 OS=Pongo pygmaeus GN=SCAND1 PE=3 SV=1//2.48117e-67
XM_007963747.1	0.64606	6.23E-07	5.20E-06	sp|Q9Y3B2|EXOS1_HUMAN Exosome complex component CSL4 OS=Homo sapiens GN=EXOSC1 PE=1 SV=1//2.57887e-140
XM_007963748.1	-1.1054	6.19E-18	1.10E-16	sp|P18669|PGAM1_HUMAN Phosphoglycerate mutase 1 OS=Homo sapiens GN=PGAM1 PE=1 SV=2//1.22234e-87
XM_007963753.1	-1.4047	3.78E-42	1.76E-40	sp|Q86XE5|HOGA1_HUMAN 4-hydroxy-2-oxoglutarate aldolase, mitochondrial OS=Homo sapiens GN=HOGA1 PE=1 SV=1//0
XM_007963758.1	1.8435	4.72E-43	2.23E-41	sp|Q5R8D4|AVPI1_PONAB Arginine vasopressin-induced protein 1 OS=Pongo abelii GN=AVPI1 PE=2 SV=1//8.10411e-82
XM_007963762.1	-1.4923	5.10E-40	2.23E-38	sp|Q9BSK0|MALD1_HUMAN MARVEL domain-containing protein 1 OS=Homo sapiens GN=MARVELD1 PE=1 SV=1//1.34911e-72
XM_007963813.1	0.68659	1.35E-05	9.83E-05	sp|Q96A46|MFRN2_HUMAN Mitoferrin-2 OS=Homo sapiens GN=SLC25A28 PE=2 SV=1//0
XM_007963821.1	0.88217	6.70E-09	6.63E-08	sp|Q9NQZ7|ENTP7_HUMAN Ectonucleoside triphosphate diphosphohydrolase 7 OS=Homo sapiens GN=ENTPD7 PE=2 SV=1//0
XM_007963822.1	1.3018	1.02E-09	1.09E-08	sp|Q6XZF7|DNMBP_HUMAN Dynamin-binding protein OS=Homo sapiens GN=DNMBP PE=1 SV=1//0
XM_007963823.1	1.8511	5.26E-53	3.43E-51	sp|Q6XZF7|DNMBP_HUMAN Dynamin-binding protein OS=Homo sapiens GN=DNMBP PE=1 SV=1//0

XM_007963830.1	1.0612	7.17E-07	5.96E-06	sp|O15111|IKKA_HUMAN Inhibitor of nuclear factor kappa-B kinase subunit alpha OS=Homo sapiens GN=CHUK PE=1 SV=2//0
XM_007963838.1	0.99238	5.53E-18	9.90E-17	sp|Q6QNY1|BL1S2_HUMAN Biogenesis of lysosome-related organelles complex 1 subunit 2 OS=Homo sapiens GN=BLOC1S2 PE=1 SV=1//4.86722e-59
XM_007963839.1	-1.0789	1.82E-63	1.59E-61	sp|O00767|ACOD_HUMAN Acyl-CoA desaturase OS=Homo sapiens GN=SCD PE=1 SV=2//0
XM_007963849.1	-0.98631	1.20E-21	2.59E-20	sp|O95169|NDUB8_HUMAN NADH dehydrogenase [ubiquinone] 1 beta subcomplex subunit 8, mitochondrial OS=Homo sapiens GN=NDUFB8 PE=1 SV=1//3.72823e-124
XM_007963850.1	0.50314	1.85E-06	1.48E-05	sp|Q9NWT6|HIF1N_HUMAN Hypoxia-inducible factor 1-alpha inhibitor OS=Homo sapiens GN=HIF1AN PE=1 SV=2//0
XM_007963860.1	1.5332	3.51E-09	3.55E-08	sp|Q96RR1|PEO1_HUMAN Twinkle protein, mitochondrial OS=Homo sapiens GN=PEO1 PE=1 SV=1//0
XM_007963863.1	-0.49921	5.21E-06	3.98E-05	sp|Q8N983|RM43_HUMAN 39S ribosomal protein L43, mitochondrial OS=Homo sapiens GN=MRPL43 PE=1 SV=1//4.83302e-101
XM_007963876.1	-0.83053	8.10E-12	1.01E-10	sp|Q9BWM7|SFXN3_HUMAN Sideroflexin-3 OS=Homo sapiens GN=SFXN3 PE=1 SV=2//0
XM_007963877.1	-0.88947	0.0003083	0.0018874	sp|Q9H5P4|PDZD7_HUMAN PDZ domain-containing protein 7 OS=Homo sapiens GN=PDZD7 PE=1 SV=1//0
XM_007963878.1	-2.3663	0.0001479	0.0009478	sp|Q9BRK4|LZTS2_HUMAN Leucine zipper putative tumor suppressor 2 OS=Homo sapiens GN=LZTS2 PE=1 SV=2//0
XM_007963884.1	-2.5084	2.78E-05	0.0001942	-//-
XM_007963934.1	-2.7637	0.0064949	0.031622	sp|Q8WN03|KCIP2_MUSPF Kv channel-interacting protein 2 OS=Mustela putorius furo GN=Kcnip2 PE=2 SV=1//3.97147e-131
XM_007963935.1	-3.5134	0.00257	0.013647	sp|Q8WN03|KCIP2_MUSPF Kv channel-interacting protein 2 OS=Mustela putorius furo GN=Kcnip2 PE=2 SV=1//2.36957e-130
XM_007963939.1	0.77767	5.08E-09	5.08E-08	sp|Q86YV9|HPS6_HUMAN Hermansky-Pudlak syndrome 6 protein OS=Homo sapiens GN=HPS6 PE=1 SV=1//0
XM_007963942.1	-1.4994	1.62E-08	1.56E-07	sp|Q86U70|LDB1_HUMAN LIM domain-binding protein 1 OS=Homo sapiens GN=LDB1 PE=1 SV=2//0
XM_007963974.1	-1.7556	0.0097252	0.045704	sp|Q9H7T3|CJ095_HUMAN Uncharacterized protein C10orf95 OS=Homo sapiens GN=C10orf95 PE=1 SV=1//1.76294e-22
XM_007963975.1	0.34521	0.0001808	0.0011437	sp|P85515|ACTZ_RAT Alpha-centractin OS=Rattus norvegicus GN=Actr1a PE=1 SV=1//0
XM_007964001.1	-1.0871	0.0044106	0.022306	sp|Q9ESN1|DOC2G_MOUSE Double C2-like domain-containing protein gamma OS=Mus musculus GN=Doc2g PE=2 SV=1//0
XM_007964007.1	1.3395	5.21E-37	2.04E-35	sp|Q9BYE7|PCGF6_HUMAN Polycomb group RING finger protein 6 OS=Homo sapiens GN=PCGF6 PE=1 SV=2//0
XM_007964010.1	0.62901	0.0006431	0.0037783	sp|Q15542|TAF5_HUMAN Transcription initiation factor TFIID subunit 5 OS=Homo sapiens GN=TAF5 PE=1 SV=3//0
XM_007964025.1	-0.95622	0.0002382	0.0014824	sp|Q5TCZ1|SPD2A_HUMAN SH3 and PX domain-containing protein 2A OS=Homo sapiens GN=SH3PXD2A PE=1 SV=1//0
XM_007964038.1	-1.1219	3.57E-06	2.77E-05	sp|Q8NDM7|CFA43_HUMAN Cilia- and flagella-associated protein 43 OS=Homo sapiens GN=CFAP43 PE=2 SV=3//0
XM_007964039.1	-1.1248	2.88E-08	2.71E-07	sp|P78417|GSTO1_HUMAN Glutathione S-transferase omega-1 OS=Homo sapiens GN=GSTO1 PE=1 SV=2//1.24102e-169
XM_007964043.1	1.0333	2.88E-06	2.25E-05	sp|Q8IWB1|IPRI_HUMAN Inositol 1,4,5-trisphosphate receptor-interacting protein OS=Homo sapiens GN=ITPRIP PE=1 SV=1//0
XM_007964056.1	-1.0746	3.97E-12	5.06E-11	sp|Q5RBA4|MYL9_PONAB Myosin regulatory light polypeptide 9 OS=Pongo abelii GN=MYL9 PE=2 SV=3//1.71856e-106
XM_007964057.1	0.5835	1.21E-05	8.84E-05	sp|Q9NQW7|XPP1_HUMAN Xaa-Pro aminopeptidase 1 OS=Homo sapiens GN=XPNPEP1 PE=1 SV=3//0
XM_007964058.1	0.54894	3.85E-05	0.0002646	sp|Q9NQW7|XPP1_HUMAN Xaa-Pro aminopeptidase 1 OS=Homo sapiens GN=XPNPEP1 PE=1 SV=3//0
XM_007964059.1	-0.8074	9.54E-26	2.51E-24	sp|Q6PDV7|RL10_RAT 60S ribosomal protein L10 OS=Rattus norvegicus GN=Rpl10 PE=1 SV=3//3.07116e-157
XM_007964077.1	2.9895	1.75E-32	5.84E-31	sp|Q16690|DUS5_HUMAN Dual specificity protein phosphatase 5 OS=Homo sapiens GN=DUSP5 PE=1 SV=2//0
XM_007964078.1	0.40272	1.94E-08	1.85E-07	sp|Q5R4K5|SMC3_PONAB Structural maintenance of chromosomes protein 3 OS=Pongo abelii GN=SMC3 PE=2 SV=1//0
XM_007964094.1	-1.018	1.27E-09	1.33E-08	sp|Q8WV74|NUDT8_HUMAN Nucleoside diphosphate-linked moiety X motif 8, mitochondrial OS=Homo sapiens GN=NUDT8 PE=2 SV=2//3.99056e-143
XM_007964096.1	0.68279	2.08E-18	3.81E-17	sp|Q9HCL2|GPAT1_HUMAN Glycerol-3-phosphate acyltransferase 1, mitochondrial OS=Homo sapiens GN=GPAM PE=1 SV=3//0
XM_007964114.1	-1.0888	7.57E-21	1.57E-19	sp|Q86VF7|NRAP_HUMAN Nebulin-related-anchoring protein OS=Homo sapiens GN=NRAP PE=2 SV=2//0
XM_007964115.1	-0.85746	2.95E-20	5.93E-19	sp|Q14520|HABP2_HUMAN Hyaluronan-binding protein 2 OS=Homo sapiens GN=HABP2 PE=1 SV=1//0
XM_007964116.1	-1.6806	1.99E-12	2.58E-11	sp|Q14520|HABP2_HUMAN Hyaluronan-binding protein 2 OS=Homo sapiens GN=HABP2 PE=1 SV=1//0

XM_007964121.1	-0.73375	3.69E-14	5.31E-13	sp|O94964|SOGA1_HUMAN Protein SOGA1 OS=Homo sapiens GN=SOGA1 PE=1 SV=2//0
XM_007964123.1	0.88764	1.21E-08	1.18E-07	sp|Q8NBF2|NHLC2_HUMAN NHL repeat-containing protein 2 OS=Homo sapiens GN=NHLRC2 PE=1 SV=1//0
XM_007964128.1	1.7423	0.0005564	0.0033033	sp|P47899|ADRB1_MACMU Beta-1 adrenergic receptor OS=Macaca mulatta GN=ADRB1 PE=3 SV=1//0
XM_007964129.1	0.88536	4.60E-08	4.25E-07	sp|Q7Z3E2|CC186_HUMAN Coiled-coil domain-containing protein 186 OS=Homo sapiens GN=CCDC186 PE=1 SV=2//0
XM_007964169.1	1.4191	1.17E-43	5.69E-42	sp|Q5W0V3|F16B1_HUMAN Protein FAM160B1 OS=Homo sapiens GN=FAM160B1 PE=1 SV=1//0
XM_007964170.1	0.65925	3.33E-14	4.81E-13	sp|Q8WWH5|TRUB1_HUMAN Probable tRNA pseudouridine synthase 1 OS=Homo sapiens GN=TRUB1 PE=1 SV=1//0
XM_007964205.1	-0.73696	2.16E-16	3.54E-15	sp|Q8HXQ9|NDUV1_MACFA NADH dehydrogenase [ubiquinone] flavoprotein 1, mitochondrial OS=Macaca fascicularis GN=NDUFV1 PE=2 SV=1//0
XM_007964222.1	1.0477	1.33E-23	3.17E-22	sp|Q05940|VMAT2_HUMAN Synaptic vesicular amine transporter OS=Homo sapiens GN=SLC18A2 PE=1 SV=2//0
XM_007964223.1	1.4598	2.65E-94	4.41E-92	sp|Q8NEN9|PDZD8_HUMAN PDZ domain-containing protein 8 OS=Homo sapiens GN=PDZD8 PE=1 SV=1//0
XM_007964225.1	-2.0015	0.0049359	0.024645	sp|Q04743|EMX2_HUMAN Homeobox protein EMX2 OS=Homo sapiens GN=EMX2 PE=1 SV=2//4.52797e-141
XM_007964228.1	-0.35242	0.0033842	0.017516	sp|Q7L804|RFIP2_HUMAN Rab11 family-interacting protein 2 OS=Homo sapiens GN=RAB11FIP2 PE=1 SV=1//0
XM_007964236.1	0.42017	5.38E-09	5.36E-08	sp|Q86Y37|CACL1_HUMAN CDK2-associated and cullin domain-containing protein 1 OS=Homo sapiens GN=CACUL1 PE=1 SV=1//0
XM_007964237.1	-0.55665	0.0060443	0.029638	sp|Q8WY41|NANO1_HUMAN Nanos homolog 1 OS=Homo sapiens GN=NANOS1 PE=1 SV=2//1.33267e-18
XM_007964241.1	-1.7533	0.0086042	0.040901	sp|Q6P4A7|SFXN4_HUMAN Sideroflexin-4 OS=Homo sapiens GN=SFXN4 PE=1 SV=1//3.8655e-107
XM_007964245.1	-1.4542	1.45E-63	1.27E-61	sp|Q5REY3|PRDX3_PONAB Thioredoxin-dependent peroxide reductase, mitochondrial OS=Pongo abelii GN=PRDX3 PE=2 SV=1//0
XM_007964272.1	0.38267	1.74E-05	0.0001244	sp|Q9BZH6|WDR11_HUMAN WD repeat-containing protein 11 OS=Homo sapiens GN=WDR11 PE=1 SV=1//0
XM_007964287.1	0.36196	0.0006158	0.0036285	sp|O95359|TACC2_HUMAN Transforming acidic coiled-coil-containing protein 2 OS=Homo sapiens GN=TACC2 PE=1 SV=3//0
XM_007964297.1	-0.51892	3.81E-13	5.14E-12	sp|Q92743|HTRA1_HUMAN Serine protease HTRA1 OS=Homo sapiens GN=HTRA1 PE=1 SV=1//0
XM_007964306.1	-1.2571	4.74E-42	2.20E-40	sp|P48047|ATPO_HUMAN ATP synthase subunit O, mitochondrial OS=Homo sapiens GN=ATP5O PE=1 SV=1//6.19186e-149
XM_007964315.1	4.6302	0.0001732	0.0011005	sp|Q9H5V7|IKZF5_HUMAN Zinc finger protein Pegasus OS=Homo sapiens GN=IKZF5 PE=1 SV=1//0
XM_007964318.1	2.414	1.04E-87	1.57E-85	sp|Q9H8K7|CJ088_HUMAN Uncharacterized protein C10orf88 OS=Homo sapiens GN=C10orf88 PE=1 SV=2//0
XM_007964320.1	-0.89777	5.90E-20	1.17E-18	sp|Q5RF40|ACDSB_PONAB Short/branched chain specific acyl-CoA dehydrogenase, mitochondrial OS=Pongo abelii GN=ACADSB PE=2 SV=1//0
XM_007964322.1	-0.46422	1.81E-05	0.0001296	sp|Q9WVA3|BUB3_MOUSE Mitotic checkpoint protein BUB3 OS=Mus musculus GN=Bub3 PE=2 SV=2//0
XM_007964340.1	-2.1152	1.45E-29	4.38E-28	sp|Q9H008|LHPP_HUMAN Phospholysine phosphohistidine inorganic pyrophosphate phosphatase OS=Homo sapiens GN=LHPP PE=1 SV=2//2.11948e-170
XM_007964345.1	0.53918	0.0002186	0.0013662	sp|Q5JPI9|MET10_HUMAN Protein-lysine N-methyltransferase METTL10 OS=Homo sapiens GN=METTL10 PE=1 SV=2//8.5214e-176
XM_007964386.1	-2.3178	0.0067804	0.032887	sp|Q9P287|BCCIP_HUMAN BRCA2 and CDKN1A-interacting protein OS=Homo sapiens GN=BCCIP PE=1 SV=1//1.68298e-109
XM_007964392.1	2.5528	8.75E-61	6.94E-59	sp|O43184|ADA12_HUMAN Disintegrin and metalloproteinase domain-containing protein 12 OS=Homo sapiens GN=ADAM12 PE=1 SV=3//0
XM_007964397.1	-2.6368	0.0054109	0.026821	-//-
XM_007964416.1	-3.9099	0.0094483	0.044488	sp|Q28514|GSTP1_MACMU Glutathione S-transferase P OS=Macaca mulatta GN=GSTP1 PE=2 SV=3//1.17433e-147
XM_007964422.1	0.73389	7.53E-08	6.81E-07	sp|P18583|SON_HUMAN Protein SON OS=Homo sapiens GN=SON PE=1 SV=4//3.69309e-07
XM_007964444.1	0.35	1.75E-05	0.0001255	sp|O76003|GLRX3_HUMAN Glutaredoxin-3 OS=Homo sapiens GN=GLRX3 PE=1 SV=2//0
XM_007964452.1	-2.0844	7.93E-15	1.19E-13	sp|Q12983|BNIP3_HUMAN BCL2/adenovirus E1B 19 kDa protein-interacting protein 3 OS=Homo sapiens GN=BNIP3 PE=1 SV=2//4.40385e-95
XM_007964496.1	-1.4935	6.81E-13	9.07E-12	sp|Q8IYW2|CFA46_HUMAN Cilia- and flagella-associated protein 46 OS=Homo sapiens GN=CFAP46 PE=2 SV=3//0
XM_007964500.1	7.8707	1.34E-51	8.54E-50	sp|Q5JTH9|RRP12_HUMAN RRP12-like protein OS=Homo sapiens GN=RRP12 PE=1 SV=2//1.4013e-46
XM_007964514.1	0.90259	0.0004571	0.0027438	sp|P16260|GDC_HUMAN Graves disease carrier protein OS=Homo sapiens GN=SLC25A16 PE=1 SV=3//1.57818e-08

XM_007964519.1	0.63608	5.17E-16	8.29E-15	sp|Q53EZ4|CEP55_HUMAN Centrosomal protein of 55 kDa OS=Homo sapiens GN=CEP55 PE=1 SV=3//0
XM_007964523.1	-0.78261	2.56E-16	4.17E-15	sp|P18669|PGAM1_HUMAN Phosphoglycerate mutase 1 OS=Homo sapiens GN=PGAM1 PE=1 SV=2//2.58919e-73
XM_007964525.1	0.54871	1.27E-05	9.29E-05	sp|Q5T2E6|CJ076_HUMAN UPF0668 protein C10orf76 OS=Homo sapiens GN=C10orf76 PE=2 SV=1//0
XM_007964527.1	-0.96175	0.0002844	0.00175	sp|Q52NJ4|ARL3_PIG ADP-ribosylation factor-like protein 3 OS=Sus scrofa GN=ARL3 PE=2 SV=1//3.08695e-103
XM_007964533.1	0.91313	1.23E-16	2.06E-15	sp|Q9NQB0|TF7L2_HUMAN Transcription factor 7-like 2 OS=Homo sapiens GN=TCF7L2 PE=1 SV=2//4.1998e-20
XM_007964535.1	-0.32771	0.0014143	0.0079022	sp|Q8TCE6|FA45A_HUMAN Protein FAM45A OS=Homo sapiens GN=FAM45A PE=2 SV=1//0
XM_007964536.1	0.4229	0.0030638	0.016039	sp|Q15811|ITSN1_HUMAN Intersectin-1 OS=Homo sapiens GN=ITSN1 PE=1 SV=3//0
XM_007964537.1	0.40289	1.41E-05	0.0001023	sp|Q2PFX0|ATE1_MACFA Arginyl-tRNA--protein transferase 1 OS=Macaca fascicularis GN=ATE1 PE=2 SV=1//0
XM_007964539.1	3.8243	2.11E-09	2.19E-08	sp|P0C7Q2|ARMS2_HUMAN Age-related maculopathy susceptibility protein 2 OS=Homo sapiens GN=ARMS2 PE=2 SV=1//9.58638e-33
XM_007964542.1	1.408	2.33E-39	9.95E-38	sp|Q15018|F175B_HUMAN BRISC complex subunit Abro1 OS=Homo sapiens GN=FAM175B PE=1 SV=2//0
XM_007964543.1	-0.85591	0.0033799	0.017498	-//-
XM_007964553.1	-0.80299	1.13E-17	1.99E-16	sp|Q96NT0|CC115_HUMAN Coiled-coil domain-containing protein 115 OS=Homo sapiens GN=CCDC115 PE=2 SV=1//4.84147e-98
XM_007964559.1	0.40849	1.07E-06	8.77E-06	sp|Q9NXE4|NSMA3_HUMAN Sphingomyelin phosphodiesterase 4 OS=Homo sapiens GN=SMPD4 PE=1 SV=2//0
XM_007964571.1	0.71639	1.55E-25	4.06E-24	sp|O60243|H6ST1_HUMAN Heparan-sulfate 6-O-sulfotransferase 1 OS=Homo sapiens GN=HS6ST1 PE=1 SV=5//0
XM_007964593.1	1.0306	4.48E-25	1.15E-23	sp|Q9D7M8|RPB4_MOUSE DNA-directed RNA polymerase II subunit RPB4 OS=Mus musculus GN=Polr2d PE=2 SV=2//7.30188e-91
XM_007964595.1	-2.0113	0.0001523	0.0009733	sp|P38484|INGR2_HUMAN Interferon gamma receptor 2 OS=Homo sapiens GN=IFNGR2 PE=1 SV=2//0
XM_007964598.1	1.2531	1.07E-15	1.68E-14	sp|Q587I9|SFT2C_HUMAN Vesicle transport protein SFT2C OS=Homo sapiens GN=SFT2D3 PE=2 SV=1//2.03802e-20
XM_007964615.1	0.78513	1.83E-26	4.91E-25	sp|Q9Y2U5|M3K2_HUMAN Mitogen-activated protein kinase kinase kinase 2 OS=Homo sapiens GN=MAP3K2 PE=1 SV=2//0
XM_007964616.1	0.59143	4.25E-11	5.04E-10	sp|Q60HG1|ERCC3_MACFA TFIIH basal transcription factor complex helicase XPB subunit OS=Macaca fascicularis GN=ERCC3 PE=2 SV=1//0
XM_007964650.1	1.7164	1.46E-20	2.98E-19	sp|Q9BYG3|MK67I_HUMAN MKI67 FHA domain-interacting nucleolar phosphoprotein OS=Homo sapiens GN=NIFK PE=1 SV=1//2.22376e-169
XM_007964687.1	-1.1487	1.20E-14	1.79E-13	sp|Q9NZI6|TF2L1_HUMAN Transcription factor CP2-like protein 1 OS=Homo sapiens GN=TFCP2L1 PE=2 SV=1//0
XM_007964700.1	1.17	2.07E-24	5.07E-23	sp|Q9H7F4|T185B_HUMAN Transmembrane protein 185B OS=Homo sapiens GN=TMEM185B PE=1 SV=2//0
XM_007964707.1	3.3215	3.62E-18	6.56E-17	sp|Q13516|OLIG2_HUMAN Oligodendrocyte transcription factor 2 OS=Homo sapiens GN=OLIG2 PE=2 SV=2//8.71027e-18
XM_007964708.1	1.7865	1.03E-07	9.20E-07	sp|Q9MYU8|E41L5_CANFA Band 4.1-like protein 5 OS=Canis familiaris GN=EPB41L5 PE=2 SV=1//0
XM_007964731.1	-2.3915	0.0050887	0.025373	sp|P07108|ACBP_HUMAN Acyl-CoA-binding protein OS=Homo sapiens GN=DBI PE=1 SV=2//3.56402e-35
XM_007964751.1	0.99931	9.46E-32	3.10E-30	sp|Q9NVP1|DDX18_HUMAN ATP-dependent RNA helicase DDX18 OS=Homo sapiens GN=DDX18 PE=1 SV=2//0
XM_007964756.1	1.0543	1.26E-16	2.10E-15	sp|Q5R8D1|EIF3J_PONAB Eukaryotic translation initiation factor 3 subunit J OS=Pongo abelii GN=EIF3J PE=2 SV=1//1.87164e-49
XM_007964757.1	0.4617	0.0054501	0.026991	sp|Q96JI7|SPTCS_HUMAN Spatacsin OS=Homo sapiens GN=SPG11 PE=1 SV=3//1.57961e-177
XM_007964758.1	-1.5534	0.0005096	0.0030371	sp|Q9Y5B6|PAXB1_HUMAN PAX3- and PAX7-binding protein 1 OS=Homo sapiens GN=PAXBP1 PE=1 SV=2//0
XM_007964784.1	1.7023	9.04E-06	6.70E-05	sp|O14513|NCKP5_HUMAN Nck-associated protein 5 OS=Homo sapiens GN=NCKAP5 PE=1 SV=2//0
XM_007964809.1	-0.62766	0.010656	0.049615	sp|O60583|CCNT2_HUMAN Cyclin-T2 OS=Homo sapiens GN=CCNT2 PE=1 SV=2//0
XM_007964853.1	0.86271	2.39E-31	7.69E-30	sp|Q92575|UBXN4_HUMAN UBX domain-containing protein 4 OS=Homo sapiens GN=UBXN4 PE=1 SV=2//0
XM_007964863.1	-1.2657	4.74E-71	4.92E-69	sp|P14618|KPYM_HUMAN Pyruvate kinase PKM OS=Homo sapiens GN=PKM PE=1 SV=4//0
XM_007964949.1	-1.1553	0.0026995	0.014279	sp|Q86Y78|LYPD6_HUMAN Ly6/PLAUR domain-containing protein 6 OS=Homo sapiens GN=LYPD6 PE=1 SV=1//1.91789e-90
XM_007964950.1	-1.5379	2.84E-24	6.90E-23	sp|Q9BE24|LDHA_MACFA L-lactate dehydrogenase A chain OS=Macaca fascicularis GN=LDHA PE=2 SV=4//0
XM_007964951.1	0.49038	1.09E-05	8.00E-05	sp|Q9H3L0|MMAD_HUMAN Methylmalonic aciduria and homocystinuria type D protein, mitochondrial OS=Homo sapiens GN=MMADHC PE=1 SV=2//0

XM_007964953.1	3.4121	0	0	sp|Q6SA80|RND3_RAT Rho-related GTP-binding protein RhoE OS=Rattus norvegicus GN=Rnd3 PE=2 SV=1//1.32127e-162
XM_007964956.1	0.90649	1.13E-12	1.49E-11	sp|Q13287|NMI_HUMAN N-myc-interactor OS=Homo sapiens GN=NMI PE=1 SV=2//0
XM_007964957.1	-2.0012	6.67E-05	0.0004473	sp|P98066|TSG6_HUMAN Tumor necrosis factor-inducible gene 6 protein OS=Homo sapiens GN=TNFAIP6 PE=1 SV=2//1.74738e-177
XM_007965011.1	0.7941	1.59E-07	1.40E-06	sp|Q9NYP9|MS18A_HUMAN Protein Mis18-alpha OS=Homo sapiens GN=MIS18A PE=1 SV=1//1.99579e-133
XM_007965027.1	1.1391	8.56E-11	9.87E-10	sp|Q68UT7|HUNK_PANTR Hormonally up-regulated neu tumor-associated kinase OS=Pan troglodytes GN=HUNK PE=3 SV=1//0
XM_007965029.1	2.5422	0.0014831	0.0082448	sp|Q99569|PKP4_HUMAN Plakophilin-4 OS=Homo sapiens GN=PKP4 PE=1 SV=2//0
XM_007965040.1	1.3564	1.18E-05	8.66E-05	-//-
XM_007965134.1	-1.335	6.37E-40	2.76E-38	sp|Q8HXQ0|SODC_MACMU Superoxide dismutase [Cu-Zn] OS=Macaca mulatta GN=SOD1 PE=2 SV=3//2.94506e-105
XM_007965162.1	1.045	0.0029971	0.015716	sp|Q12884|SEPR_HUMAN Prolyl endopeptidase FAP OS=Homo sapiens GN=FAP PE=1 SV=5//0
XM_007965214.1	-1.5402	3.74E-06	2.89E-05	sp|Q99250|SCN2A_HUMAN Sodium channel protein type 2 subunit alpha OS=Homo sapiens GN=SCN2A PE=1 SV=3//0
XM_007965224.1	-0.71325	1.57E-15	2.44E-14	sp|Q7Z4L5|TT21B_HUMAN Tetratricopeptide repeat protein 21B OS=Homo sapiens GN=TTC21B PE=1 SV=2//0
XM_007965236.1	-1.6957	4.31E-21	9.03E-20	sp|Q15858|SCN9A_HUMAN Sodium channel protein type 9 subunit alpha OS=Homo sapiens GN=SCN9A PE=1 SV=3//0
XM_007965302.1	1.8701	7.68E-22	1.68E-20	sp|Q0VFZ6|CC173_HUMAN Coiled-coil domain-containing protein 173 OS=Homo sapiens GN=CCDC173 PE=2 SV=2//0
XM_007965315.1	0.24337	0.0009095	0.0052254	sp|P05455|LA_HUMAN Lupus La protein OS=Homo sapiens GN=SSB PE=1 SV=2//0
XM_007965316.1	0.60477	1.17E-12	1.54E-11	sp|Q6ZT12|UBR3_HUMAN E3 ubiquitin-protein ligase UBR3 OS=Homo sapiens GN=UBR3 PE=2 SV=2//0
XM_007965327.1	0.91091	7.69E-33	2.60E-31	sp|Q9H8Y8|GORS2_HUMAN Golgi reassembly-stacking protein 2 OS=Homo sapiens GN=GORASP2 PE=1 SV=3//0
XM_007965335.1	-1.9426	1.51E-13	2.11E-12	sp|Q53TN4|CYBR1_HUMAN Cytochrome b reductase 1 OS=Homo sapiens GN=CYBRD1 PE=1 SV=1//1.13681e-162
XM_007965360.1	1.131	1.18E-06	9.58E-06	sp|Q07687|DLX2_HUMAN Homeobox protein DLX-2 OS=Homo sapiens GN=DLX2 PE=1 SV=2//2.63312e-145
XM_007965370.1	0.71284	0.0003749	0.0022757	sp|P23229|ITA6_HUMAN Integrin alpha-6 OS=Homo sapiens GN=ITGA6 PE=1 SV=5//0
XM_007965380.1	1.3295	5.57E-06	4.23E-05	sp|Q9NYL2|MLTK_HUMAN Mitogen-activated protein kinase kinase kinase MLT OS=Homo sapiens GN=ZAK PE=1 SV=3//0
XM_007965400.1	0.26679	0.0043282	0.021932	sp|Q86X95|CIR1_HUMAN Corepressor interacting with RBPJ 1 OS=Homo sapiens GN=CIR1 PE=1 SV=1//3.27864e-30
XM_007965420.1	-0.84355	4.81E-23	1.12E-21	sp|Q71S46|AT5G3_RAT ATP synthase F(0) complex subunit C3, mitochondrial OS=Rattus norvegicus GN=Atp5g3 PE=2 SV=1//5.87815e-74
XM_007965425.1	1.3405	8.73E-39	3.65E-37	sp|P35453|HXD13_HUMAN Homeobox protein Hox-D13 OS=Homo sapiens GN=HOXD13 PE=1 SV=3//7.16944e-162
XM_007965426.1	-1.8213	9.72E-10	1.03E-08	sp|P28358|HXD10_HUMAN Homeobox protein Hox-D10 OS=Homo sapiens GN=HOXD10 PE=1 SV=2//0
XM_007965427.1	-1.9137	0.0010246	0.005844	sp|P31277|HXD11_HUMAN Homeobox protein Hox-D11 OS=Homo sapiens GN=HOXD11 PE=3 SV=3//5.46615e-125
XM_007965432.1	-1.7984	0.0055583	0.027489	sp|P31249|HXD3_HUMAN Homeobox protein Hox-D3 OS=Homo sapiens GN=HOXD3 PE=1 SV=3//0
XM_007965434.1	-1.3501	8.25E-11	9.53E-10	sp|A2D5I1|HXD4_LAGLA Homeobox protein Hox-D4 OS=Lagothrix lagotricha GN=HOXD4 PE=3 SV=1//2.14997e-110
XM_007965440.1	-0.38789	1.15E-08	1.11E-07	sp|P51991|ROA3_HUMAN Heterogeneous nuclear ribonucleoprotein A3 OS=Homo sapiens GN=HNRNPA3 PE=1 SV=2//9.89502e-123
XM_007965449.1	-1.4686	0.0027644	0.014601	sp|Q8N4P2|TT30B_HUMAN Tetratricopeptide repeat protein 30B OS=Homo sapiens GN=TTC30B PE=1 SV=2//0
XM_007965471.1	1.2594	2.67E-28	7.74E-27	sp|Q9HB20|PKHA3_HUMAN Pleckstrin homology domain-containing family A member 3 OS=Homo sapiens GN=PLEKHA3 PE=1 SV=2//6.93045e-178
XM_007965529.1	1.105	0.0004371	0.0026302	sp|Q5RA93|CWC22_PONAB Pre-mRNA-splicing factor CWC22 homolog OS=Pongo abelii GN=CWC22 PE=2 SV=1//0
XM_007965569.1	1.6773	3.94E-44	1.94E-42	sp|Q8NFH5|NUP53_HUMAN Nucleoporin NUP53 OS=Homo sapiens GN=NUP35 PE=1 SV=1//0
XM_007965570.1	Inf	0.0004736	0.0028332	sp|Q7Z570|Z804A_HUMAN Zinc finger protein 804A OS=Homo sapiens GN=ZNF804A PE=1 SV=3//0
XM_007965572.1	1.0019	1.71E-39	7.38E-38	sp|Q8WU90|ZC3HF_HUMAN Zinc finger CCCH domain-containing protein 15 OS=Homo sapiens GN=ZC3H15 PE=1 SV=1//0
XM_007965576.1	-0.39842	0.0002344	0.0014601	sp|Q6P995|F171B_HUMAN Protein FAM171B OS=Homo sapiens GN=FAM171B PE=2 SV=3//0
XM_007965594.1	-1.5968	0.0023083	0.012365	sp|Q9UBP9|GULP1_HUMAN PTB domain-containing engulfment adapter protein 1 OS=Homo sapiens GN=GULP1 PE=1 SV=1//0
XM_007965604.1	-0.70478	4.77E-22	1.06E-20	sp|P05997|CO5A2_HUMAN Collagen alpha-2(V) chain OS=Homo sapiens

				GN=COL5A2 PE=1 SV=3//4.87664e-175
XM_007965606.1	1.2569	1.34E-29	4.05E-28	sp|Q8IWA0|WDR75_HUMAN WD repeat-containing protein 75 OS=Homo sapiens GN=WDR75 PE=1 SV=1//0
XM_007965656.1	1.7493	1.33E-13	1.86E-12	sp|A6NFY4|NEMP2_HUMAN Nuclear envelope integral membrane protein 2 OS=Homo sapiens GN=NEMP2 PE=2 SV=3//0
XM_007965666.1	0.28439	3.24E-05	0.0002246	sp|Q4R5J0|TCPQ_MACFA T-complex protein 1 subunit theta OS=Macaca fascicularis GN=CCT8 PE=2 SV=1//0
XM_007965668.1	-0.97242	0.001802	0.0098535	sp|O94925|GLSK_HUMAN Glutaminase kidney isoform, mitochondrial OS=Homo sapiens GN=GLS PE=1 SV=1//0
XM_007965669.1	1.856	0.0001349	0.0008695	sp|P42224|STAT1_HUMAN Signal transducer and activator of transcription 1-alpha/beta OS=Homo sapiens GN=STAT1 PE=1 SV=2//0
XM_007965691.1	3.6662	5.31E-05	0.0003588	sp|Q96AH0|SOSB2_HUMAN SOSS complex subunit B2 OS=Homo sapiens GN=NABP1 PE=1 SV=1//5.72383e-122
XM_007965692.1	3.1486	0.000212	0.0013294	sp|Q96AH0|SOSB2_HUMAN SOSS complex subunit B2 OS=Homo sapiens GN=NABP1 PE=1 SV=1//6.68628e-99
XM_007965714.1	2.2439	2.73E-157	9.79E-155	sp|O94768|ST17B_HUMAN Serine/threonine-protein kinase 17B OS=Homo sapiens GN=STK17B PE=1 SV=1//0
XM_007965727.1	0.92617	1.30E-19	2.54E-18	sp|Q9Y5Q9|TF3C3_HUMAN General transcription factor 3C polypeptide 3 OS=Homo sapiens GN=GTF3C3 PE=1 SV=1//0
XM_007965729.1	-0.88581	3.27E-13	4.44E-12	sp|Q75T13|PGAP1_HUMAN GPI inositol-deacylase OS=Homo sapiens GN=PGAP1 PE=1 SV=1//0
XM_007965741.1	1.677	2.36E-20	4.76E-19	sp|Q9H8M1|CQ10B_HUMAN Coenzyme Q-binding protein COQ10 homolog B, mitochondrial OS=Homo sapiens GN=COQ10B PE=2 SV=1//4.90297e-160
XM_007965743.1	0.26138	0.0014286	0.0079702	sp|P10809|CH60_HUMAN 60 kDa heat shock protein, mitochondrial OS=Homo sapiens GN=HSPD1 PE=1 SV=2//0
XM_007965748.1	-1.772	5.82E-05	0.0003922	sp|P26772|CH10_RAT 10 kDa heat shock protein, mitochondrial OS=Rattus norvegicus GN=Hspe1 PE=1 SV=3//1.75372e-56
XM_007965753.1	1.0181	1.88E-06	1.50E-05	sp|O94822|LTN1_HUMAN E3 ubiquitin-protein ligase listerin OS=Homo sapiens GN=LTN1 PE=1 SV=6//0
XM_007965770.1	0.99539	8.29E-08	7.47E-07	sp|A2RUC4|TYW5_HUMAN tRNA wybutosine-synthesizing protein 5 OS=Homo sapiens GN=TYW5 PE=1 SV=1//0
XM_007965794.1	5.0216	1.79E-06	1.43E-05	sp|C4NYZ3|AOXB_MACFA Aldehyde oxidase 2 OS=Macaca fascicularis GN=AOX2 PE=2 SV=2//0
XM_007965798.1	2.4745	3.86E-182	1.66E-179	sp|P49759|CLK1_HUMAN Dual specificity protein kinase CLK1 OS=Homo sapiens GN=CLK1 PE=1 SV=2//0
XM_007965813.1	1.0849	5.65E-05	0.0003813	sp|Q5R977|F126B_PONAB Protein FAM126B OS=Pongo abelii GN=FAM126B PE=2 SV=1//0
XM_007965857.1	0.66133	2.90E-05	0.0002018	sp|Q96Q45|TM237_HUMAN Transmembrane protein 237 OS=Homo sapiens GN=TMEM237 PE=1 SV=2//0
XM_007965883.1	-0.82283	2.91E-13	3.97E-12	sp|Q5I0H3|SUMO1_RAT Small ubiquitin-related modifier 1 OS=Rattus norvegicus GN=Sumo1 PE=1 SV=1//3.96523e-64
XM_007965884.1	1.3986	0.0033779	0.017496	sp|Q4R779|NOP58_MACFA Nucleolar protein 58 OS=Macaca fascicularis GN=NOP58 PE=2 SV=1//0
XM_007965899.1	-0.82303	6.26E-08	5.70E-07	sp|Q6P1L5|F117B_HUMAN Protein FAM117B OS=Homo sapiens GN=FAM117B PE=1 SV=2//0
XM_007965911.1	-0.95473	9.63E-06	7.11E-05	sp|Q6UW02|CP20A_HUMAN Cytochrome P450 20A1 OS=Homo sapiens GN=CYP20A1 PE=1 SV=1//0
XM_007965938.1	2.8708	1.04E-160	3.94E-158	sp|Q9UHI8|ATS1_HUMAN A disintegrin and metalloproteinase with thrombospondin motifs 1 OS=Homo sapiens GN=ADAMTS1 PE=1 SV=4//0
XM_007966033.1	0.65191	0.0001764	0.0011194	sp|Q13467|FZD5_HUMAN Frizzled-5 OS=Homo sapiens GN=FZD5 PE=1 SV=2//0
XM_007966037.1	0.81153	0.000133	0.0008581	sp|Q6ZWE6|PKHM3_HUMAN Pleckstrin homology domain-containing family M member 3 OS=Homo sapiens GN=PLEKHM3 PE=2 SV=2//0
XM_007966041.1	-2.1151	0.0046921	0.023605	sp|P07320|CRGD_HUMAN Gamma-crystallin D OS=Homo sapiens GN=CRYGD PE=1 SV=3//9.00174e-118
XM_007966168.1	1.5195	0.0052634	0.026155	sp|Q86UK0|ABCAC_HUMAN ATP-binding cassette sub-family A member 12 OS=Homo sapiens GN=ABCA12 PE=1 SV=3//0
XM_007966192.1	-0.94468	2.30E-09	2.36E-08	sp|Q9BY49|PECR_HUMAN Peroxisomal trans-2-enoyl-CoA reductase OS=Homo sapiens GN=PECR PE=1 SV=2//0
XM_007966212.1	-0.94051	1.45E-10	1.64E-09	sp|P61515|RL37P_RAT Putative 60S ribosomal protein L37a OS=Rattus norvegicus GN=Rpl37a-ps1 PE=5 SV=2//2.86886e-60
XM_007966218.1	-1.4496	2.89E-36	1.11E-34	sp|P18065|IBP2_HUMAN Insulin-like growth factor-binding protein 2 OS=Homo sapiens GN=IGFBP2 PE=1 SV=2//0
XM_007966279.1	0.70338	2.41E-09	2.48E-08	sp|Q5R6Z6|RCD1_PONAB Cell differentiation protein RCD1 homolog OS=Pongo abelii GN=RQCD1 PE=2 SV=2//0
XM_007966307.1	1.1729	5.45E-10	5.91E-09	sp|Q96BH1|RNF25_HUMAN E3 ubiquitin-protein ligase RNF25 OS=Homo sapiens GN=RNF25 PE=1 SV=1//0
XM_007966314.1	-1.3873	4.35E-63	3.75E-61	sp|Q02318|CP27A_HUMAN Sterol 26-hydroxylase, mitochondrial OS=Homo sapiens GN=CYP27A1 PE=1 SV=1//0
XM_007966317.1	-2.4575	0.0012004	0.0067834	sp|Q13319|CD5R2_HUMAN Cyclin-dependent kinase 5 activator 2 OS=Homo sapiens GN=CDK5R2 PE=1 SV=1//2.25393e-151

XM_007966383.1	-1.8036	0.0043545	0.022048	sp|O62654|DESM_BOVIN Desmin OS=Bos taurus GN=DES PE=2 SV=3//0
XM_007966414.1	-0.84497	4.75E-10	5.18E-09	sp|Q8IZ52|CHSS2_HUMAN Chondroitin sulfate synthase 2 OS=Homo sapiens GN=CHPF PE=1 SV=2//0
XM_007966441.1	1.3248	5.25E-14	7.49E-13	sp|Q9NSD9|SYFB_HUMAN Phenylalanine--tRNA ligase beta subunit OS=Homo sapiens GN=FARSB PE=1 SV=3//0
XM_007966443.1	0.57213	4.80E-15	7.26E-14	sp|O95573|ACSL3_HUMAN Long-chain-fatty-acid--CoA ligase 3 OS=Homo sapiens GN=ACSL3 PE=1 SV=3//0
XM_007966452.1	1.2362	1.69E-61	1.38E-59	sp|Q8IWB7|WDFY1_HUMAN WD repeat and FYVE domain-containing protein 1 OS=Homo sapiens GN=WDFY1 PE=1 SV=1//0
XM_007966472.1	2.8761	1.87E-156	6.60E-154	sp|Q28224|IRS1_CHLAE Insulin receptor substrate 1 OS=Chlorocebus aethiops GN=IRS1 PE=2 SV=1//0
XM_007966496.1	-1.7368	0.0010137	0.0057833	sp|P55787|CO4A4_RABIT Collagen alpha-4(IV) chain (Fragment) OS=Oryctolagus cuniculus GN=COL4A4 PE=2 SV=1//5.02191e-06
XM_007966529.1	2.2471	2.14E-98	3.81E-96	sp|Q3UBG2|PCLI1_MOUSE PTB-containing, cubilin and LRP1-interacting protein OS=Mus musculus GN=Pid1 PE=1 SV=2//9.31419e-139
XM_007966566.1	2.4103	1.54E-14	2.27E-13	sp|Q9H930|SP14L_HUMAN Nuclear body protein SP140-like protein OS=Homo sapiens GN=SP140L PE=2 SV=3//0
XM_007966597.1	1.1723	4.32E-49	2.60E-47	sp|Q9Y376|CAB39_HUMAN Calcium-binding protein 39 OS=Homo sapiens GN=CAB39 PE=1 SV=1//0
XM_007966598.1	-1.6848	1.59E-47	8.92E-46	sp|Q9NQX7|ITM2C_HUMAN Integral membrane protein 2C OS=Homo sapiens GN=ITM2C PE=1 SV=1//6.37035e-149
XM_007966603.1	-1.3497	0.0006566	0.0038523	sp|A6NCS6|CB072_HUMAN Uncharacterized protein C2orf72 OS=Homo sapiens GN=C2orf72 PE=1 SV=2//1.83552e-86
XM_007966617.1	0.56589	2.74E-13	3.74E-12	sp|Q4R4J7|NUCL_MACFA Nucleolin OS=Macaca fascicularis GN=NCL PE=2 SV=3//0
XM_007966665.1	-0.72139	2.49E-05	0.0001748	sp|Q9BUP0|EFHD1_HUMAN EF-hand domain-containing protein D1 OS=Homo sapiens GN=EFHD1 PE=1 SV=1//5.78786e-94
XM_007966680.1	-1.7182	3.99E-06	3.08E-05	sp|Q5RDX5|NGEF_PONAB Ephexin-1 OS=Pongo abelii GN=NGEF PE=2 SV=1//0
XM_007966730.1	1.4715	3.31E-30	1.03E-28	sp|P61208|ARL4C_MOUSE ADP-ribosylation factor-like protein 4C OS=Mus musculus GN=Arl4c PE=2 SV=1//3.21315e-118
XM_007966731.1	2.4047	2.17E-212	1.22E-209	sp|Q9P0V3|SH3B4_HUMAN SH3 domain-binding protein 4 OS=Homo sapiens GN=SH3BP4 PE=1 SV=1//0
XM_007966745.1	1.6914	0.0009381	0.0053773	sp|Q14201|BTG3_HUMAN Protein BTG3 OS=Homo sapiens GN=BTG3 PE=1 SV=3//5.0542e-168
XM_007966755.1	Inf	2.46E-06	1.94E-05	sp|P52951|GBX2_HUMAN Homeobox protein GBX-2 OS=Homo sapiens GN=GBX2 PE=2 SV=3//0
XM_007966780.1	0.54069	6.66E-09	6.60E-08	sp|Q5RBX2|BET1L_PONAB BET1-like protein OS=Pongo abelii GN=BET1L PE=3 SV=1//1.6261e-65
XM_007966801.1	0.30212	0.0013174	0.0073985	sp|Q5R764|CXAR_PONAB Coxsackievirus and adenovirus receptor homolog OS=Pongo abelii GN=CXADR PE=2 SV=1//0
XM_007966844.1	1.0663	2.16E-15	3.32E-14	sp|Q9H0C8|ILKAP_HUMAN Integrin-linked kinase-associated serine/threonine phosphatase 2C OS=Homo sapiens GN=ILKAP PE=1 SV=1//0
XM_007966854.1	0.50284	0.000467	0.0027975	sp|Q9Y576|ASB1_HUMAN Ankyrin repeat and SOCS box protein 1 OS=Homo sapiens GN=ASB1 PE=1 SV=1//0
XM_007966885.1	-0.75857	3.67E-11	4.36E-10	sp|Q8WXC6|MYOV2_HUMAN Myeloma-overexpressed gene 2 protein OS=Homo sapiens GN=MYEOV2 PE=1 SV=3//1.5029e-19
XM_007966904.1	-1.6356	1.98E-46	1.09E-44	sp|P35052|GPC1_HUMAN Glypican-1 OS=Homo sapiens GN=GPC1 PE=1 SV=2//0
XM_007966943.1	2.0654	0.009507	0.044736	sp|Q08AI8|CB054_HUMAN Uncharacterized protein C2orf54 OS=Homo sapiens GN=C2orf54 PE=2 SV=2//0
XM_007966946.1	0.82054	5.39E-31	1.71E-29	sp|P48552|NRIP1_HUMAN Nuclear receptor-interacting protein 1 OS=Homo sapiens GN=NRIP1 PE=1 SV=2//0
XM_007966984.1	-1.0509	3.07E-05	0.0002131	sp|Q6IWH7|ANO7_HUMAN Anoctamin-7 OS=Homo sapiens GN=ANO7 PE=1 SV=2//0
XM_007966985.1	-1.3213	0.0005606	0.0033236	-//-
XM_007967000.1	-0.44944	1.69E-07	1.48E-06	sp|Q9UMX3|BOK_HUMAN Bcl-2-related ovarian killer protein OS=Homo sapiens GN=BOK PE=1 SV=1//1.14647e-143
XM_007967047.1	0.49505	0.0014584	0.0081197	sp|Q5H9S7|DCA17_HUMAN DDB1- and CUL4-associated factor 17 OS=Homo sapiens GN=DCAF17 PE=1 SV=1//0
XM_007967048.1	-1.5781	7.63E-05	0.0005079	-//-
XM_007967053.1	0.78853	7.72E-21	1.60E-19	sp|Q5R8D9|HSP13_PONAB Heat shock 70 kDa protein 13 OS=Pongo abelii GN=HSPA13 PE=2 SV=1//0
XM_007967054.1	1.1584	0.0022157	0.011907	sp|E5RQL4|FONG_HUMAN Formiminotransferase N-terminal subdomain-containing protein OS=Homo sapiens GN=FTCDNL1 PE=2 SV=1//1.38094e-70
XM_007967098.1	0.91998	1.20E-35	4.54E-34	sp|P29375|KDM5A_HUMAN Lysine-specific demethylase 5A OS=Homo sapiens GN=KDM5A PE=1 SV=3//0
XM_007967159.1	0.2071	0.0039024	0.019925	sp|Q86V24|ADR2_HUMAN Adiponectin receptor protein 2 OS=Homo sapiens GN=ADIPOR2 PE=1 SV=1//0
XM_007967233.1	0.38344	0.0001076	0.0007025	sp|Q969R8|ITFG2_HUMAN Integrin-alpha FG-GAP repeat-containing protein 2 OS=Homo sapiens GN=ITFG2 PE=1 SV=1//0
XM_007967273.1	0.60449	7.03E-06	5.28E-05	sp|Q9NQ88|TIGAR_HUMAN Fructose-2,6-bisphosphatase TIGAR OS=Homo sapiens

				GN=TIGAR PE=1 SV=1//0
XM_007967276.1	2.0506	4.44E-18	7.99E-17	sp|Q5RES7|RCAN1_PONAB Calcipressin-1 OS=Pongo abelii GN=RCAN1 PE=2 SV=1//2.36554e-90
XM_007967277.1	0.77121	3.24E-17	5.55E-16	sp|Q5RD58|CL004_PONAB Protein C12orf4 homolog OS=Pongo abelii PE=2 SV=1//0
XM_007967299.1	3.5877	1.27E-06	1.03E-05	sp|Q96NY7|CLIC6_HUMAN Chloride intracellular channel protein 6 OS=Homo sapiens GN=CLIC6 PE=2 SV=3//0
XM_007967301.1	-0.68936	3.56E-15	5.40E-14	sp|Q0MQB3|NDUA9_GORGO NADH dehydrogenase [ubiquinone] 1 alpha subcomplex subunit 9, mitochondrial OS=Gorilla gorilla gorilla GN=NDUFA9 PE=2 SV=2//0
XM_007967326.1	-0.40437	1.89E-06	1.51E-05	sp|P30409|CD9_CHLAE CD9 antigen OS=Chlorocebus aethiops GN=CD9 PE=2 SV=2//1.029e-132
XM_007967339.1	-2.6304	2.06E-26	5.53E-25	sp|P23763|VAMP1_HUMAN Vesicle-associated membrane protein 1 OS=Homo sapiens GN=VAMP1 PE=1 SV=1//1.3118e-49
XM_007967342.1	-1.3195	6.43E-95	1.10E-92	sp|P04406|G3P_HUMAN Glyceraldehyde-3-phosphate dehydrogenase OS=Homo sapiens GN=GAPDH PE=1 SV=3//0
XM_007967346.1	-0.53143	2.95E-08	2.78E-07	sp|Q4U2R6|RM51_HUMAN 39S ribosomal protein L51, mitochondrial OS=Homo sapiens GN=MRPL51 PE=1 SV=1//1.3486e-82
XM_007967389.1	3.4508	2.91E-40	1.28E-38	-//-
XM_007967394.1	-3.9211	0.0063374	0.030931	sp|Q95K74|PIANP_MACFA PILR alpha-associated neural protein OS=Macaca fascicularis GN=PIANP PE=2 SV=1//1.44654e-107
XM_007967404.1	-1.6678	6.66E-118	1.62E-115	-//-
XM_007967409.1	-1.6874	0.0036255	0.01865	sp|Q8IVL6|P3H3_HUMAN Prolyl 3-hydroxylase 3 OS=Homo sapiens GN=LEPREL2 PE=1 SV=1//0
XM_007967411.1	-2.593	1.47E-09	1.53E-08	sp|Q16538|GP162_HUMAN Probable G-protein coupled receptor 162 OS=Homo sapiens GN=GPR162 PE=2 SV=1//0
XM_007967423.1	-1.4861	5.21E-105	1.08E-102	sp|P60174|TPIS_HUMAN Triosephosphate isomerase OS=Homo sapiens GN=TPI1 PE=1 SV=3//0
XM_007967439.1	-2.8016	1.23E-18	2.29E-17	sp|Q53EV4|LRC23_HUMAN Leucine-rich repeat-containing protein 23 OS=Homo sapiens GN=LRRC23 PE=2 SV=2//5.4757e-138
XM_007967442.1	-1.6226	2.62E-35	9.80E-34	sp|O35127|C10_MOUSE Protein C10 OS=Mus musculus GN=Grcc10 PE=2 SV=1//5.38351e-67
XM_007967446.1	-1.7281	5.15E-05	0.0003496	sp|P29350|PTN6_HUMAN Tyrosine-protein phosphatase non-receptor type 6 OS=Homo sapiens GN=PTPN6 PE=1 SV=1//0
XM_007967448.1	3.6391	1.76E-44	8.87E-43	sp|Q92979|NEP1_HUMAN Ribosomal RNA small subunit methyltransferase NEP1 OS=Homo sapiens GN=EMG1 PE=1 SV=4//8.94042e-176
XM_007967449.1	-1.2194	5.15E-52	3.31E-50	sp|Q6P1A2|MBOA5_HUMAN Lysophospholipid acyltransferase 5 OS=Homo sapiens GN=LPCAT3 PE=1 SV=1//0
XM_007967451.1	-1.5773	1.20E-14	1.79E-13	sp|Q9NZP8|C1RL_HUMAN Complement C1r subcomponent-like protein OS=Homo sapiens GN=C1RL PE=1 SV=2//0
XM_007967454.1	-1.4801	1.87E-08	1.79E-07	sp|Q9BQT9|CSTN3_HUMAN Calsyntenin-3 OS=Homo sapiens GN=CLSTN3 PE=1 SV=1//0
XM_007967455.1	-2.4564	0.01013	0.047352	sp|Q5RBM7|RET5_PONAB Retinol-binding protein 5 OS=Pongo abelii GN=RBP5 PE=2 SV=3//5.76943e-94
XM_007967486.1	1.3621	3.03E-38	1.24E-36	sp|P11169|GTR3_HUMAN Solute carrier family 2, facilitated glucose transporter member 3 OS=Homo sapiens GN=SLC2A3 PE=2 SV=1//0
XM_007967487.1	0.91327	1.16E-11	1.42E-10	sp|Q9P0K8|FOXJ2_HUMAN Forkhead box protein J2 OS=Homo sapiens GN=FOXJ2 PE=1 SV=1//0
XM_007967489.1	3.4427	3.99E-27	1.10E-25	sp|Q6TAC8|C3AR_MACFA C3a anaphylatoxin chemotactic receptor OS=Macaca fascicularis GN=C3AR1 PE=2 SV=1//0
XM_007967490.1	1.472	2.29E-48	1.33E-46	sp|Q5R630|NECP1_PONAB Adaptin ear-binding coat-associated protein 1 OS=Pongo abelii GN=NECAP1 PE=2 SV=1//1.38771e-147
XM_007967499.1	-1.2599	2.84E-33	9.81E-32	sp|Q8MI29|CBR1_MACFA Carbonyl reductase [NADPH] 1 OS=Macaca fascicularis GN=CBR1 PE=2 SV=1//0
XM_007967527.1	1.9075	0.0054351	0.026922	sp|Q9NVD3|SETD4_HUMAN SET domain-containing protein 4 OS=Homo sapiens GN=SETD4 PE=2 SV=1//0
XM_007967537.1	1.488	0.0005261	0.0031348	sp|Q96E93|KLRG1_HUMAN Killer cell lectin-like receptor subfamily G member 1 OS=Homo sapiens GN=KLRG1 PE=1 SV=1//1.22654e-107
XM_007967604.1	-0.81274	6.29E-10	6.77E-09	sp|Q15370|ELOB_HUMAN Transcription elongation factor B polypeptide 2 OS=Homo sapiens GN=TCEB2 PE=1 SV=1//2.13345e-80
XM_007967608.1	-1.3446	5.31E-08	4.87E-07	sp|Q4KMG9|TM52B_HUMAN Transmembrane protein 52B OS=Homo sapiens GN=TMEM52B PE=2 SV=1//1.6793e-85
XM_007967609.1	1.9339	3.96E-107	8.50E-105	sp|Q5BIZ2|GBRL1_XENTR Gamma-aminobutyric acid receptor-associated protein-like 1 OS=Xenopus tropicalis GN=gabarapl1 PE=3 SV=1//5.79418e-74
XM_007967630.1	0.75614	1.37E-15	2.13E-14	sp|Q13112|CAF1B_HUMAN Chromatin assembly factor 1 subunit B OS=Homo sapiens GN=CHAF1B PE=1 SV=1//0
XM_007967665.1	2.7434	0.000267	0.0016492	sp|Q9UBD9|CLCF1_HUMAN Cardiotrophin-like cytokine factor 1 OS=Homo sapiens GN=CLCF1 PE=1 SV=1//2.31736e-157
XM_007967696.1	-2.6571	9.35E-28	2.65E-26	sp|Q95KG7|MANS1_MACFA MANSC domain-containing protein 1 OS=Macaca

				fascicularis GN=MANSC1 PE=2 SV=1//0
XM_007967699.1	1.6796	4.62E-08	4.26E-07	sp|Q9BY84|DUS16_HUMAN Dual specificity protein phosphatase 16 OS=Homo sapiens GN=DUSP16 PE=1 SV=1//0
XM_007967704.1	-0.81607	0.0022195	0.011924	sp|P46527|CDN1B_HUMAN Cyclin-dependent kinase inhibitor 1B OS=Homo sapiens GN=CDKN1B PE=1 SV=1//4.10182e-133
XM_007967705.1	0.36862	0.0036437	0.018722	sp|P46527|CDN1B_HUMAN Cyclin-dependent kinase inhibitor 1B OS=Homo sapiens GN=CDKN1B PE=1 SV=1//3.14013e-133
XM_007967707.1	-4.5086	0.0003875	0.0023484	sp|Q96LR9|APLD1_HUMAN Apolipoprotein L domain-containing protein 1 OS=Homo sapiens GN=APOLD1 PE=2 SV=2//5.68245e-99
XM_007967709.1	0.53281	7.73E-06	5.78E-05	sp|Q9H0S4|DDX47_HUMAN Probable ATP-dependent RNA helicase DDX47 OS=Homo sapiens GN=DDX47 PE=1 SV=1//0
XM_007967718.1	-1.0284	1.26E-11	1.54E-10	sp|Q9NRV9|HEBP1_HUMAN Heme-binding protein 1 OS=Homo sapiens GN=HEBP1 PE=1 SV=1//1.3155e-133
XM_007967719.1	3.1039	9.61E-104	1.91E-101	sp|A2RU67|K1467_HUMAN Uncharacterized protein KIAA1467 OS=Homo sapiens GN=KIAA1467 PE=1 SV=1//0
XM_007967724.1	0.73849	0.0098078	0.046063	sp|Q14190|SIM2_HUMAN Single-minded homolog 2 OS=Homo sapiens GN=SIM2 PE=1 SV=2//0
XM_007967725.1	6.3164	2.40E-267	1.95E-264	sp|Q5RCY3|EMP1_PONAB Epithelial membrane protein 1 OS=Pongo abelii GN=EMP1 PE=2 SV=1//2.05711e-71
XM_007967740.1	2.1494	6.34E-08	5.78E-07	sp|Q28DR4|H4_XENTR Histone H4 OS=Xenopus tropicalis GN=TGas006m08.1 PE=3 SV=1//4.09329e-46
XM_007967741.1	-1.255	2.00E-44	1.00E-42	sp|Q4R3X5|H2AJ_MACFA Histone H2A.J OS=Macaca fascicularis GN=H2AFJ PE=2 SV=1//6.40341e-85
XM_007967759.1	-3.0949	0.0094672	0.044568	sp|P52566|GDIR2_HUMAN Rho GDP-dissociation inhibitor 2 OS=Homo sapiens GN=ARHGDIB PE=1 SV=3//1.98295e-131
XM_007967772.1	-0.37631	4.46E-06	3.43E-05	sp|P53804|TTC3_HUMAN E3 ubiquitin-protein ligase TTC3 OS=Homo sapiens GN=TTC3 PE=1 SV=2//0
XM_007967773.1	0.76995	2.95E-25	7.66E-24	sp|Q9Y3F4|STRAP_HUMAN Serine-threonine kinase receptor-associated protein OS=Homo sapiens GN=STRAP PE=1 SV=1//0
XM_007967774.1	-0.49599	1.46E-07	1.29E-06	sp|Q9Y315|DEOC_HUMAN Deoxyribose-phosphate aldolase OS=Homo sapiens GN=DERA PE=1 SV=2//0
XM_007967839.1	#NAME?	0.010041	0.047039	sp|Q9HAU0|PKHA5_HUMAN Pleckstrin homology domain-containing family A member 5 OS=Homo sapiens GN=PLEKHA5 PE=1 SV=1//1.87271e-38
XM_007967850.1	-0.45724	0.0057311	0.02821	sp|Q14432|PDE3A_HUMAN cGMP-inhibited 3',5'-cyclic phosphodiesterase A OS=Homo sapiens GN=PDE3A PE=1 SV=3//0
XM_007967869.1	0.27961	0.010471	0.048789	sp|Q9Y3E0|GOT1B_HUMAN Vesicle transport protein GOT1B OS=Homo sapiens GN=GOLT1B PE=1 SV=1//1.23091e-56
XM_007967875.1	-0.63824	6.69E-21	1.39E-19	sp|Q4R5B6|LDHB_MACFA L-lactate dehydrogenase B chain OS=Macaca fascicularis GN=LDHB PE=2 SV=3//0
XM_007967903.1	-2.8098	0.0017669	0.0096826	sp|Q86YS7|C2CD5_HUMAN C2 domain-containing protein 5 OS=Homo sapiens GN=C2CD5 PE=1 SV=1//0
XM_007967905.1	0.58921	1.32E-14	1.95E-13	sp|Q9HBU6|EKI1_HUMAN Ethanolamine kinase 1 OS=Homo sapiens GN=ETNK1 PE=1 SV=1//0
XM_007967990.1	4.0039	3.09E-14	4.47E-13	sp|Q9C0J9|BHE41_HUMAN Class E basic helix-loop-helix protein 41 OS=Homo sapiens GN=BHLHE41 PE=1 SV=1//2.8825e-44
XM_007967994.1	-1.8241	0.0017962	0.0098304	sp|Q9NVK5|FGOP2_HUMAN FGFR1 oncogene partner 2 OS=Homo sapiens GN=FGFR1OP2 PE=1 SV=1//5.41099e-172
XM_007967996.1	1.4158	2.17E-63	1.88E-61	sp|Q9NVM9|ASUN_HUMAN Protein asunder homolog OS=Homo sapiens GN=ASUN PE=1 SV=2//0
XM_007967999.1	0.706	5.31E-12	6.71E-11	sp|Q4R6N3|MED21_MACFA Mediator of RNA polymerase II transcription subunit 21 OS=Macaca fascicularis GN=MED21 PE=2 SV=1//5.90935e-94
XM_007968012.1	2.7243	0.0006971	0.0040716	sp|A6NFE2|SMCO2_HUMAN Single-pass membrane and coiled-coil domain-containing protein 2 OS=Homo sapiens GN=SMCO2 PE=2 SV=2//0
XM_007968037.1	0.62577	1.59E-08	1.54E-07	sp|Q9P2K6|KLH42_HUMAN Kelch-like protein 42 OS=Homo sapiens GN=KLHL42 PE=1 SV=2//0
XM_007968055.1	-0.93238	0.0010332	0.0058884	sp|Q96K12|FACR2_HUMAN Fatty acyl-CoA reductase 2 OS=Homo sapiens GN=FAR2 PE=2 SV=1//0
XM_007968089.1	-0.45452	5.51E-06	4.19E-05	sp|O15397|IPO8_HUMAN Importin-8 OS=Homo sapiens GN=IPO8 PE=1 SV=2//0
XM_007968100.1	1.4474	8.91E-54	5.99E-52	sp|Q6ZUT9|DEN5B_HUMAN DENN domain-containing protein 5B OS=Homo sapiens GN=DENND5B PE=1 SV=2//0
XM_007968118.1	-0.90048	0.00451	0.022746	sp|Q96G01|BICD1_HUMAN Protein bicaudal D homolog 1 OS=Homo sapiens GN=BICD1 PE=1 SV=3//1.17907e-06
XM_007968131.1	-2.2406	0.0008606	0.0049629	sp|O00429|DNM1L_HUMAN Dynamin-1-like protein OS=Homo sapiens GN=DNM1L PE=1 SV=2//0
XM_007968132.1	1.0952	5.12E-41	2.29E-39	sp|Q99959|PKP2_HUMAN Plakophilin-2 OS=Homo sapiens GN=PKP2 PE=1 SV=2//0
XM_007968133.1	3.0856	2.43E-211	1.33E-208	sp|P15036|ETS2_HUMAN Protein C-ets-2 OS=Homo sapiens GN=ETS2 PE=1 SV=1//0
XM_007968134.1	0.62337	5.59E-10	6.05E-09	sp|Q9Y2Z4|SYYM_HUMAN Tyrosine--tRNA ligase, mitochondrial OS=Homo sapiens GN=YARS2 PE=1 SV=2//0
XM_007968140.1	0.27362	0.0032901	0.017118	sp|Q9DC53|CPNE8_MOUSE Copine-8 OS=Mus musculus GN=Cpne8 PE=2 SV=3//0

XM_007968144.1	-1.8654	2.00E-22	4.51E-21	sp|Q4R577|C1R_MACFA Complement C1r subcomponent OS=Macaca fascicularis GN=C1R PE=2 SV=1//0
XM_007968155.1	0.80807	9.27E-22	2.01E-20	sp|Q9NP50|FA60A_HUMAN Protein FAM60A OS=Homo sapiens GN=FAM60A PE=1 SV=1//4.64056e-106
XM_007968156.1	1.3056	1.10E-38	4.58E-37	sp|Q96M96|FGD4_HUMAN FYVE, RhoGEF and PH domain-containing protein 4 OS=Homo sapiens GN=FGD4 PE=1 SV=2//0
XM_007968161.1	2.1962	7.04E-33	2.39E-31	sp|Q5R6W3|RBM18_PONAB Probable RNA-binding protein 18 OS=Pongo abelii GN=RBM18 PE=2 SV=1//1.54264e-130
XM_007968177.1	-0.82601	6.51E-15	9.78E-14	sp|P0CB92|NDUA8_PONPY NADH dehydrogenase [ubiquinone] 1 alpha subcomplex subunit 8 OS=Pongo pygmaeus GN=NDUFA8 PE=2 SV=1//2.89381e-102
XM_007968180.1	1.1248	0.0016916	0.0093091	sp|A4Q9F4|TTL11_MOUSE Tubulin polyglutamylase TTLL11 OS=Mus musculus GN=Ttll11 PE=2 SV=1//0
XM_007968201.1	-1.9587	2.69E-11	3.23E-10	sp|Q9NSI6|BRWD1_HUMAN Bromodomain and WD repeat-containing protein 1 OS=Homo sapiens GN=BRWD1 PE=1 SV=4//0
XM_007968203.1	-0.44928	3.17E-08	2.96E-07	sp|P27105|STOM_HUMAN Erythrocyte band 7 integral membrane protein OS=Homo sapiens GN=STOM PE=1 SV=3//0
XM_007968211.1	0.21097	0.0051768	0.025759	sp|Q5R8Z8|RAB14_PONAB Ras-related protein Rab-14 OS=Pongo abelii GN=RAB14 PE=2 SV=3//7.61083e-143
XM_007968212.1	-0.57548	4.91E-13	6.57E-12	sp|P05114|HMGN1_HUMAN Non-histone chromosomal protein HMG-14 OS=Homo sapiens GN=HMGN1 PE=1 SV=3//7.62395e-31
XM_007968213.1	-1.212	7.96E-13	1.06E-11	sp|O15145|ARPC3_HUMAN Actin-related protein 2/3 complex subunit 3 OS=Homo sapiens GN=ARPC3 PE=1 SV=3//1.32394e-120
XM_007968245.1	-0.99804	1.60E-38	6.62E-37	sp|P62138|PP1A_RAT Serine/threonine-protein phosphatase PP1-alpha catalytic subunit OS=Rattus norvegicus GN=Ppp1ca PE=1 SV=1//0
XM_007968260.1	2.8335	1.37E-11	1.68E-10	sp|Q13219|PAPP1_HUMAN Pappalysin-1 OS=Homo sapiens GN=PAPPA PE=1 SV=3//0
XM_007968261.1	4.035	8.27E-05	0.0005485	sp|Q5QFB9|PAPAS_HUMAN Protein PAPPAS OS=Homo sapiens GN=PAPPA-AS1 PE=5 SV=1//2.24493e-50
XM_007968284.1	2.3592	8.31E-09	8.18E-08	sp|O95150|TNF15_HUMAN Tumor necrosis factor ligand superfamily member 15 OS=Homo sapiens GN=TNFSF15 PE=1 SV=2//1.44153e-176
XM_007968295.1	-0.30312	0.0005078	0.0030269	sp|P79251|VATG1_BOVIN V-type proton ATPase subunit G 1 OS=Bos taurus GN=ATP6V1G1 PE=1 SV=3//2.04624e-56
XM_007968323.1	-1.1969	8.31E-07	6.87E-06	sp|P02760|AMBP_HUMAN Protein AMBP OS=Homo sapiens GN=AMBP PE=1 SV=1//0
XM_007968351.1	0.73605	3.16E-08	2.96E-07	sp|Q5R4W3|DPOE3_PONAB DNA polymerase epsilon subunit 3 OS=Pongo abelii GN=POLE3 PE=2 SV=1//2.32697e-60
XM_007968368.1	-0.31513	0.0031106	0.016265	sp|O00258|WRB_HUMAN Tail-anchored protein insertion receptor WRB OS=Homo sapiens GN=WRB PE=1 SV=2//2.98753e-120
XM_007968393.1	0.7143	7.98E-19	1.50E-17	sp|Q5VWJ9|SNX30_HUMAN Sorting nexin-30 OS=Homo sapiens GN=SNX30 PE=1 SV=1//0
XM_007968416.1	1.8585	1.45E-150	4.97E-148	sp|Q16739|CEGT_HUMAN Ceramide glucosyltransferase OS=Homo sapiens GN=UGCG PE=1 SV=1//0
XM_007968424.1	2.3468	1.35E-74	1.51E-72	sp|Q9H1X3|DJC25_HUMAN DnaJ homolog subfamily C member 25 OS=Homo sapiens GN=DNAJC25 PE=1 SV=1//2.81765e-180
XM_007968433.1	-0.59979	0.0036229	0.018641	sp|Q14914|PTGR1_HUMAN Prostaglandin reductase 1 OS=Homo sapiens GN=PTGR1 PE=1 SV=2//5.11862e-173
XM_007968434.1	-1.7079	2.33E-05	0.0001646	sp|Q8TF39|ZN483_HUMAN Zinc finger protein 483 OS=Homo sapiens GN=ZNF483 PE=1 SV=3//0
XM_007968459.1	-0.96257	1.60E-34	5.86E-33	sp|P29451|THIO_MACMU Thioredoxin OS=Macaca mulatta GN=TXN PE=3 SV=2//8.10551e-69
XM_007968476.1	0.60862	3.82E-08	3.54E-07	sp|B2RYE5|E41LB_RAT Band 4.1-like protein 4B OS=Rattus norvegicus GN=Epb41l4b PE=2 SV=1//0
XM_007968481.1	-0.31257	0.0003854	0.002336	sp|Q9H330|TM245_HUMAN Transmembrane protein 245 OS=Homo sapiens GN=TMEM245 PE=1 SV=2//0
XM_007968490.1	0.42871	2.84E-09	2.89E-08	sp|P54727|RD23B_HUMAN UV excision repair protein RAD23 homolog B OS=Homo sapiens GN=RAD23B PE=1 SV=1//0
XM_007968515.1	1.028	2.64E-11	3.18E-10	sp|Q9NVV0|TM38B_HUMAN Trimeric intracellular cation channel type B OS=Homo sapiens GN=TMEM38B PE=1 SV=1//0
XM_007968516.1	0.52806	7.36E-05	0.0004909	sp|Q9BXM9|FSD1L_HUMAN FSD1-like protein OS=Homo sapiens GN=FSD1L PE=1 SV=2//0
XM_007968531.1	-0.64402	8.16E-06	6.08E-05	sp|Q8TE77|SSH3_HUMAN Protein phosphatase Slingshot homolog 3 OS=Homo sapiens GN=SSH3 PE=1 SV=2//0
XM_007968532.1	-0.8931	2.87E-06	2.25E-05	sp|Q5RAA9|NPS3A_PONAB Protein NipSnap homolog 3A OS=Pongo abelii GN=NIPSNAP3A PE=2 SV=1//1.99594e-165
XM_007968590.1	0.59544	1.54E-09	1.61E-08	sp|Q9BS26|ERP44_HUMAN Endoplasmic reticulum resident protein 44 OS=Homo sapiens GN=ERP44 PE=1 SV=1//0
XM_007968599.1	0.3086	0.0029336	0.015413	sp|Q5RB31|SC61B_PONAB Protein transport protein Sec61 subunit beta OS=Pongo abelii GN=SEC61B PE=3 SV=3//9.9777e-39
XM_007968609.1	-2.9732	4.25E-07	3.60E-06	sp|Q8IXK2|GLT12_HUMAN Polypeptide N-acetylgalactosaminyltransferase 12 OS=Homo sapiens GN=GALNT12 PE=1 SV=3//0

XM_007968612.1	-1.1048	0.0005947	0.0035079	sp|Q68DC2|ANKS6_HUMAN Ankyrin repeat and SAM domain-containing protein 6 OS=Homo sapiens GN=ANKS6 PE=1 SV=2//0
XM_007968622.1	0.79396	1.62E-09	1.69E-08	sp|Q9NR45|SIAS_HUMAN Sialic acid synthase OS=Homo sapiens GN=NANS PE=1 SV=2//0
XM_007968623.1	0.81869	9.36E-06	6.92E-05	sp|Q14142|TRI14_HUMAN Tripartite motif-containing protein 14 OS=Homo sapiens GN=TRIM14 PE=2 SV=2//0
XM_007968653.1	0.92932	1.56E-14	2.29E-13	sp|Q5RCP1|TSTD2_PONAB Thiosulfate sulfurtransferase/rhodanese-like domain-containing protein 2 OS=Pongo abelii GN=TSTD2 PE=2 SV=1//0
XM_007968663.1	0.56114	6.02E-12	7.58E-11	sp|Q15464|SHB_HUMAN SH2 domain-containing adapter protein B OS=Homo sapiens GN=SHB PE=1 SV=2//0
XM_007968704.1	1.1138	4.02E-19	7.67E-18	sp|Q9GZS1|RPA49_HUMAN DNA-directed RNA polymerase I subunit RPA49 OS=Homo sapiens GN=POLR1E PE=1 SV=2//0
XM_007968709.1	-0.71711	0.0023813	0.012722	sp|Q9UBQ7|GRHPR_HUMAN Glyoxylate reductase/hydroxypyruvate reductase OS=Homo sapiens GN=GRHPR PE=1 SV=1//0
XM_007968711.1	0.98362	0.0068892	0.033349	sp|Q8N3Z6|ZCHC7_HUMAN Zinc finger CCHC domain-containing protein 7 OS=Homo sapiens GN=ZCCHC7 PE=1 SV=2//0
XM_007968777.1	0.49556	4.54E-06	3.49E-05	sp|O43889|CREB3_HUMAN Cyclic AMP-responsive element-binding protein 3 OS=Homo sapiens GN=CREB3 PE=1 SV=1//0
XM_007968778.1	0.71438	1.60E-08	1.55E-07	sp|Q9HCG7|GBA2_HUMAN Non-lysosomal glucosylceramidase OS=Homo sapiens GN=GBA2 PE=1 SV=2//0
XM_007968795.1	2.2885	1.08E-68	1.06E-66	sp|P57078|RIPK4_HUMAN Receptor-interacting serine/threonine-protein kinase 4 OS=Homo sapiens GN=RIPK4 PE=1 SV=1//0
XM_007968827.1	2.7398	6.93E-06	5.22E-05	sp|Q8N2Y8|RUSC2_HUMAN Iporin OS=Homo sapiens GN=RUSC2 PE=1 SV=3//0
XM_007968865.1	-0.3606	0.0004148	0.0025047	sp|Q9UJZ1|STML2_HUMAN Stomatin-like protein 2, mitochondrial OS=Homo sapiens GN=STOML2 PE=1 SV=1//0
XM_007968871.1	-0.87813	0.0091882	0.043385	sp|Q01853|TERA_MOUSE Transitional endoplasmic reticulum ATPase OS=Mus musculus GN=Vcp PE=1 SV=4//0
XM_007968912.1	-2.1439	5.91E-06	4.48E-05	sp|A6NKF2|ARI3C_HUMAN AT-rich interactive domain-containing protein 3C OS=Homo sapiens GN=ARID3C PE=3 SV=1//9.7366e-157
XM_007968941.1	-1.5174	1.97E-06	1.57E-05	sp|Q8K1D8|ENHO_MOUSE Adropin OS=Mus musculus GN=Enho PE=2 SV=1//1.86156e-12
XM_007968994.1	1.7266	6.30E-12	7.90E-11	sp|Q12986|NFX1_HUMAN Transcriptional repressor NF-X1 OS=Homo sapiens GN=NFX1 PE=1 SV=2//0
XM_007968996.1	2.4387	5.34E-39	2.26E-37	sp|Q12986|NFX1_HUMAN Transcriptional repressor NF-X1 OS=Homo sapiens GN=NFX1 PE=1 SV=2//0
XM_007969002.1	1.4977	6.03E-35	2.24E-33	sp|Q99933|BAG1_HUMAN BAG family molecular chaperone regulator 1 OS=Homo sapiens GN=BAG1 PE=1 SV=4//0
XM_007969005.1	1.1077	0.003867	0.019776	sp|O95834|EMAL2_HUMAN Echinoderm microtubule-associated protein-like 2 OS=Homo sapiens GN=EML2 PE=1 SV=1//0
XM_007969028.1	-1.3986	5.34E-27	1.47E-25	sp|P0CB94|NDUB6_PONPY NADH dehydrogenase [ubiquinone] 1 beta subcomplex subunit 6 OS=Pongo pygmaeus GN=NDUFB6 PE=2 SV=1//2.87259e-65
XM_007969029.1	1.0678	0.0070125	0.033908	sp|Q9NS56|TOPRS_HUMAN E3 ubiquitin-protein ligase Topors OS=Homo sapiens GN=TOPORS PE=1 SV=1//2.30063e-161
XM_007969031.1	-0.81662	0.0001733	0.0011011	sp|P21399|ACOC_HUMAN Cytoplasmic aconitate hydratase OS=Homo sapiens GN=ACO1 PE=1 SV=3//0
XM_007969032.1	5.0304	1.10E-43	5.39E-42	sp|O95786|DDX58_HUMAN Probable ATP-dependent RNA helicase DDX58 OS=Homo sapiens GN=DDX58 PE=1 SV=2//0
XM_007969034.1	0.76748	0.0015758	0.0087246	sp|Q9Y2K7|KDM2A_HUMAN Lysine-specific demethylase 2A OS=Homo sapiens GN=KDM2A PE=1 SV=3//0
XM_007969038.1	-1.0374	1.35E-56	9.57E-55	sp|P60707|ACTB_TRIVU Actin, cytoplasmic 1 OS=Trichosurus vulpecula GN=ACTB PE=2 SV=1//0
XM_007969043.1	-1.1396	6.50E-06	4.91E-05	sp|Q86TA1|MOB3B_HUMAN MOB kinase activator 3B OS=Homo sapiens GN=MOB3B PE=1 SV=2//5.28267e-146
XM_007969108.1	0.52733	5.06E-10	5.50E-09	sp|Q9P2J3|KLHL9_HUMAN Kelch-like protein 9 OS=Homo sapiens GN=KLHL9 PE=1 SV=2//0
XM_007969115.1	Inf	5.50E-08	5.04E-07	sp|O77812|IFNB_MACFA Interferon beta OS=Macaca fascicularis GN=IFNB1 PE=3 SV=1//5.80499e-119
XM_007969124.1	-0.57389	5.90E-06	4.48E-05	sp|P42568|AF9_HUMAN Protein AF-9 OS=Homo sapiens GN=MLLT3 PE=1 SV=2//0
XM_007969127.1	1.2488	6.14E-21	1.28E-19	sp|Q9UI40|NCKX2_HUMAN Sodium/potassium/calcium exchanger 2 OS=Homo sapiens GN=SLC24A2 PE=1 SV=1//0
XM_007969132.1	-0.36215	3.43E-08	3.20E-07	sp|P62755|RS6_RAT 40S ribosomal protein S6 OS=Rattus norvegicus GN=Rps6 PE=1 SV=1//6.92248e-149
XM_007969147.1	-0.47882	4.57E-07	3.85E-06	sp|Q63486|RRAGA_RAT Ras-related GTP-binding protein A OS=Rattus norvegicus GN=Rraga PE=1 SV=1//0
XM_007969185.1	-3.1303	0.0092536	0.043656	sp|Q8WYR4|RSPH1_HUMAN Radial spoke head 1 homolog OS=Homo sapiens GN=RSPH1 PE=1 SV=1//2.09178e-175
XM_007969195.1	-0.29823	0.0041415	0.021043	sp|O75475|PSIP1_HUMAN PC4 and SFRS1-interacting protein OS=Homo sapiens GN=PSIP1 PE=1 SV=1//0
XM_007969206.1	-0.64898	0.0004265	0.0025724	sp|Q5RB84|ZDH21_PONAB Probable palmitoyltransferase ZDHHC21 OS=Pongo abelii GN=ZDHHC21 PE=2 SV=1//3.36393e-162

XM_007969242.1	3.6551	4.28E-50	2.66E-48	sp|Q8IV03|LUR1L_HUMAN Leucine rich adaptor protein 1-like OS=Homo sapiens GN=LURAP1L PE=1 SV=2//5.07407e-91
XM_007969321.1	-0.2985	0.0047962	0.024024	sp|Q7Z2K6|ERMP1_HUMAN Endoplasmic reticulum metallopeptidase 1 OS=Homo sapiens GN=ERMP1 PE=1 SV=2//0
XM_007969328.1	1.6617	1.79E-07	1.56E-06	sp|Q4ADV7|RIC1_HUMAN RAB6A-GEF complex partner protein 1 OS=Homo sapiens GN=RIC1 PE=1 SV=2//0
XM_007969343.1	1.8793	0.0026796	0.014181	sp|Q9Y2P8|RCL1_HUMAN RNA 3'-terminal phosphate cyclase-like protein OS=Homo sapiens GN=RCL1 PE=1 SV=3//0
XM_007969349.1	1.5275	2.39E-34	8.69E-33	sp|Q7L3B6|CD37L_HUMAN Hsp90 co-chaperone Cdc37-like 1 OS=Homo sapiens GN=CDC37L1 PE=1 SV=1//0
XM_007969350.1	0.6933	6.07E-07	5.07E-06	sp|Q8IY26|PPAC2_HUMAN Presqualene diphosphate phosphatase OS=Homo sapiens GN=PPAPDC2 PE=1 SV=3//4.94127e-154
XM_007969360.1	1.2662	3.71E-61	2.98E-59	sp|Q8NEA6|GLIS3_HUMAN Zinc finger protein GLIS3 OS=Homo sapiens GN=GLIS3 PE=2 SV=5//0
XM_007969370.1	0.92175	0.0001168	0.0007593	sp|P55347|PKNX1_HUMAN Homeobox protein PKNOX1 OS=Homo sapiens GN=PKNOX1 PE=1 SV=3//3.23211e-33
XM_007969373.1	1.4072	1.72E-45	9.05E-44	sp|Q15397|K0020_HUMAN Pumilio domain-containing protein KIAA0020 OS=Homo sapiens GN=KIAA0020 PE=1 SV=3//0
XM_007969408.1	-1.3054	1.16E-23	2.76E-22	sp|P02488|CRYAA_MACMU Alpha-crystallin A chain OS=Macaca mulatta GN=CRYAA PE=1 SV=2//4.76494e-111
XM_007969428.1	1.062	1.04E-14	1.55E-13	sp|Q96E09|F122A_HUMAN Protein FAM122A OS=Homo sapiens GN=FAM122A PE=1 SV=1//3.31632e-148
XM_007969442.1	0.34305	0.002518	0.01341	sp|Q7Z6K3|PTAR1_HUMAN Protein prenyltransferase alpha subunit repeat-containing protein 1 OS=Homo sapiens GN=PTAR1 PE=1 SV=2//0
XM_007969452.1	2.3284	2.96E-179	1.25E-176	sp|P57059|SIK1_HUMAN Serine/threonine-protein kinase SIK1 OS=Homo sapiens GN=SIK1 PE=1 SV=2//0
XM_007969455.1	1.3142	6.43E-10	6.91E-09	sp|Q8IY18|SMC5_HUMAN Structural maintenance of chromosomes protein 5 OS=Homo sapiens GN=SMC5 PE=1 SV=2//0
XM_007969466.1	3.0999	2.36E-37	9.38E-36	sp|Q14684|RRP1B_HUMAN Ribosomal RNA processing protein 1 homolog B OS=Homo sapiens GN=RRP1B PE=1 SV=3//0
XM_007969484.1	0.62085	7.09E-07	5.89E-06	sp|P79288|KLF9_PIG Krueppel-like factor 9 OS=Sus scrofa GN=KLF9 PE=2 SV=3//3.67805e-147
XM_007969485.1	-0.76459	8.19E-27	2.22E-25	sp|Q9UHN6|TMEM2_HUMAN Transmembrane protein 2 OS=Homo sapiens GN=TMEM2 PE=1 SV=1//0
XM_007969487.1	0.92506	8.86E-11	1.02E-09	sp|Q5VST6|AB17B_HUMAN Alpha/beta hydrolase domain-containing protein 17B OS=Homo sapiens GN=ABHD17B PE=2 SV=1//0
XM_007969496.1	1.4244	2.75E-88	4.21E-86	sp|O76080|ZFAN5_HUMAN AN1-type zinc finger protein 5 OS=Homo sapiens GN=ZFAND5 PE=1 SV=1//8.19673e-119
XM_007969498.1	4.0199	4.08E-20	8.15E-19	sp|Q6JZS3|CP1A1_ORYLA Cytochrome P450 1A1 OS=Oryzias latipes GN=cyp1a1 PE=3 SV=1//1.62499e-166
XM_007969503.1	1.4293	3.03E-59	2.29E-57	sp|P04083|ANXA1_HUMAN Annexin A1 OS=Homo sapiens GN=ANXA1 PE=1 SV=2//0
XM_007969505.1	2.6475	3.05E-10	3.38E-09	sp|O75031|HSF2B_HUMAN Heat shock factor 2-binding protein OS=Homo sapiens GN=HSF2BP PE=1 SV=1//0
XM_007969525.1	-0.72561	1.95E-06	1.56E-05	sp|Q92882|OSTF1_HUMAN Osteoclast-stimulating factor 1 OS=Homo sapiens GN=OSTF1 PE=1 SV=2//1.99736e-140
XM_007969536.1	1.826	4.59E-75	5.22E-73	sp|Q969G6|RIFK_HUMAN Riboflavin kinase OS=Homo sapiens GN=RFK PE=1 SV=2//5.50339e-103
XM_007969549.1	0.84912	2.56E-20	5.16E-19	sp|Q2PKF4|GNAQ_PIG Guanine nucleotide-binding protein G(q) subunit alpha OS=Sus scrofa GN=GNAQ PE=2 SV=3//0
XM_007969560.1	0.44156	4.27E-06	3.29E-05	sp|Q9Y617|SERC_HUMAN Phosphoserine aminotransferase OS=Homo sapiens GN=PSAT1 PE=1 SV=2//0
XM_007969562.1	-1.4133	2.02E-11	2.45E-10	sp|Q862Z5|CYTB_MACFU Cystatin-B OS=Macaca fuscata fuscata GN=CSTB PE=3 SV=1//2.68964e-63
XM_007969575.1	0.81793	8.53E-05	0.0005652	sp|Q04724|TLE1_HUMAN Transducin-like enhancer protein 1 OS=Homo sapiens GN=TLE1 PE=1 SV=2//0
XM_007969583.1	2.3089	6.73E-22	1.48E-20	sp|Q8IZ41|RASEF_HUMAN Ras and EF-hand domain-containing protein OS=Homo sapiens GN=RASEF PE=1 SV=1//0
XM_007969593.1	-1.7967	0.0035179	0.018135	sp|Q5T6J7|GNTK_HUMAN Probable gluconokinase OS=Homo sapiens GN=IDNK PE=1 SV=1//3.51585e-113
XM_007969666.1	0.6996	5.78E-08	5.28E-07	sp|P54826|GAS1_HUMAN Growth arrest-specific protein 1 OS=Homo sapiens GN=GAS1 PE=2 SV=2//1.47819e-116
XM_007969698.1	1.5538	1.10E-08	1.07E-07	sp|Q99500|S1PR3_HUMAN Sphingosine 1-phosphate receptor 3 OS=Homo sapiens GN=S1PR3 PE=1 SV=2//0
XM_007969707.1	-1.0647	0.0002611	0.0016146	sp|Q92529|SHC3_HUMAN SHC-transforming protein 3 OS=Homo sapiens GN=SHC3 PE=1 SV=1//0
XM_007969731.1	0.82885	1.85E-14	2.71E-13	sp|Q01974|ROR2_HUMAN Tyrosine-protein kinase transmembrane receptor ROR2 OS=Homo sapiens GN=ROR2 PE=1 SV=2//0
XM_007969734.1	0.73226	1.60E-20	3.25E-19	sp|Q60HD1|SPTC1_MACFA Serine palmitoyltransferase 1 OS=Macaca fascicularis GN=SPTLC1 PE=2 SV=1//0
XM_007969741.1	2.4721	4.02E-108	9.00E-106	sp|Q9H8X2|IPPK_HUMAN Inositol-pentakisphosphate 2-kinase OS=Homo sapiens GN=IPPK PE=1 SV=1//0

XM_007969748.1	-0.94906	7.28E-06	5.47E-05	sp|O75144|ICOSL_HUMAN ICOS ligand OS=Homo sapiens GN=ICOSLG PE=1 SV=2//3.91242e-163
XM_007969795.1	0.41184	0.0001259	0.0008145	sp|Q5T036|F120S_HUMAN Putative FAM120A opposite strand protein OS=Homo sapiens GN=FAM120AOS PE=5 SV=1//2.4357e-31
XM_007969797.1	-2.1239	0.0029887	0.01568	-//-
XM_007969800.1	-1.8517	0.0066366	0.032254	sp|Q9HBU1|BARX1_HUMAN Homeobox protein BarH-like 1 OS=Homo sapiens GN=BARX1 PE=1 SV=2//5.36151e-123
XM_007969812.1	0.744	8.96E-09	8.80E-08	sp|Q9Y520|PRC2C_HUMAN Protein PRRC2C OS=Homo sapiens GN=PRRC2C PE=1 SV=4//0
XM_007969821.1	-0.79279	7.25E-18	1.29E-16	sp|Q5SR56|HIAL1_HUMAN Hippocampus abundant transcript-like protein 1 OS=Homo sapiens GN=HIATL1 PE=2 SV=3//0
XM_007969848.1	-1.4014	2.70E-69	2.68E-67	sp|P17858|PFKAL_HUMAN ATP-dependent 6-phosphofructokinase, liver type OS=Homo sapiens GN=PFKL PE=1 SV=6//0
XM_007969894.1	2.7295	3.49E-141	1.06E-138	sp|Q7RTV3|ZN367_HUMAN Zinc finger protein 367 OS=Homo sapiens GN=ZNF367 PE=1 SV=1//6.53365e-170
XM_007969902.1	0.94925	4.42E-22	9.80E-21	sp|Q5T890|ER6L2_HUMAN DNA excision repair protein ERCC-6-like 2 OS=Homo sapiens GN=ERCC6L2 PE=1 SV=2//0
XM_007969910.1	-0.9351	0.008059	0.038509	sp|Q9BY71|LRRC3_HUMAN Leucine-rich repeat-containing protein 3 OS=Homo sapiens GN=LRRC3 PE=1 SV=1//1.47112e-151
XM_007969921.1	-1.7162	8.25E-12	1.03E-10	sp|Q8N6M6|AMPO_HUMAN Aminopeptidase O OS=Homo sapiens GN=AOPEP PE=1 SV=2//3.89139e-47
XM_007969924.1	-0.97996	0.0033236	0.017252	sp|Q8N6M6|AMPO_HUMAN Aminopeptidase O OS=Homo sapiens GN=AOPEP PE=1 SV=2//0
XM_007969941.1	-3.9386	0.0087366	0.041458	sp|Q8WW24|TEKT4_HUMAN Tektin-4 OS=Homo sapiens GN=TEKT4 PE=1 SV=1//0
XM_007969944.1	1.6579	7.71E-40	3.34E-38	sp|Q5NVD0|PRP4_PONAB U4/U6 small nuclear ribonucleoprotein Prp4 OS=Pongo abelii GN=PRPF4 PE=2 SV=1//0
XM_007969945.1	-0.60071	9.14E-05	0.0006032	-//-
XM_007969947.1	-0.4093	0.0001749	0.0011098	sp|Q6UWL2|SUSD1_HUMAN Sushi domain-containing protein 1 OS=Homo sapiens GN=SUSD1 PE=1 SV=1//8.59588e-07
XM_007969955.1	-0.45822	0.0053311	0.026461	sp|Q5ZME8|SMU1_CHICK WD40 repeat-containing protein SMU1 OS=Gallus gallus GN=SMU1 PE=2 SV=1//2.91822e-58
XM_007969957.1	-1.7037	4.34E-10	4.76E-09	-//-
XM_007969958.1	2.5504	1.22E-13	1.72E-12	-//-
XM_007969969.1	-2.5274	0.0058742	0.028869	sp|Q6ZUB1|S31E1_HUMAN Spermatogenesis-associated protein 31E1 OS=Homo sapiens GN=SPATA31E1 PE=2 SV=2//0
XM_007969976.1	1.4971	2.25E-18	4.11E-17	sp|Q9BZ76|CNTP3_HUMAN Contactin-associated protein-like 3 OS=Homo sapiens GN=CNTNAP3 PE=2 SV=3//0
XM_007969980.1	1.048	5.05E-19	9.55E-18	sp|O00237|RN103_HUMAN E3 ubiquitin-protein ligase RNF103 OS=Homo sapiens GN=RNF103 PE=1 SV=1//0
XM_007969992.1	0.94309	1.05E-29	3.19E-28	sp|O95602|RPA1_HUMAN DNA-directed RNA polymerase I subunit RPA1 OS=Homo sapiens GN=POLR1A PE=1 SV=2//0
XM_007970008.1	-0.32311	0.0028158	0.014858	sp|Q2NKX9|CB068_HUMAN UPF0561 protein C2orf68 OS=Homo sapiens GN=C2orf68 PE=2 SV=1//5.22325e-79
XM_007970019.1	-0.98792	5.26E-08	4.83E-07	sp|O95183|VAMP5_HUMAN Vesicle-associated membrane protein 5 OS=Homo sapiens GN=VAMP5 PE=1 SV=1//4.07092e-40
XM_007970020.1	-1.2485	2.94E-14	4.25E-13	sp|Q5REQ5|VAMP8_PONAB Vesicle-associated membrane protein 8 OS=Pongo abelii GN=VAMP8 PE=3 SV=1//2.46237e-47
XM_007970026.1	-0.90235	2.95E-29	8.80E-28	sp|Q5R5H1|METK2_PONAB S-adenosylmethionine synthase isoform type-2 OS=Pongo abelii GN=MAT2A PE=2 SV=1//0
XM_007970033.1	0.92721	5.98E-12	7.52E-11	sp|Q96FG2|ELMD3_HUMAN ELMO domain-containing protein 3 OS=Homo sapiens GN=ELMOD3 PE=1 SV=2//0
XM_007970038.1	-1.3975	1.07E-06	8.78E-06	sp|P63312|TYB10_RAT Thymosin beta-10 OS=Rattus norvegicus GN=Tmsb10 PE=2 SV=2//7.46476e-22
XM_007970051.1	-0.51296	2.14E-08	2.04E-07	sp|P53597|SUCA_HUMAN Succinyl-CoA ligase [ADP/GDP-forming] subunit alpha, mitochondrial OS=Homo sapiens GN=SUCLG1 PE=1 SV=4//1.06582e-164
XM_007970063.1	0.41	3.24E-06	2.52E-05	sp|Q5RF84|UB2G2_PONAB Ubiquitin-conjugating enzyme E2 G2 OS=Pongo abelii GN=UBE2G2 PE=2 SV=1//5.24071e-111
XM_007970073.1	-0.46713	8.23E-07	6.80E-06	sp|P55854|SUMO3_HUMAN Small ubiquitin-related modifier 3 OS=Homo sapiens GN=SUMO3 PE=1 SV=2//2.0008e-64
XM_007970099.1	0.48984	0.0018959	0.010309	sp|P52789|HXK2_HUMAN Hexokinase-2 OS=Homo sapiens GN=HK2 PE=1 SV=2//0
XM_007970130.1	-2.0734	0.0005064	0.0030196	sp|Q6XYB7|LBX2_HUMAN Transcription factor LBX2 OS=Homo sapiens GN=LBX2 PE=2 SV=1//6.27117e-105
XM_007970131.1	1.2952	1.24E-08	1.20E-07	sp|Q9BSM1|PCGF1_HUMAN Polycomb group RING finger protein 1 OS=Homo sapiens GN=PCGF1 PE=1 SV=2//7.33732e-177
XM_007970137.1	1.1915	0.0048657	0.024333	sp|Q17RM4|CC142_HUMAN Coiled-coil domain-containing protein 142 OS=Homo sapiens GN=CCDC142 PE=2 SV=1//0
XM_007970141.1	-0.50583	2.09E-06	1.66E-05	sp|Q13724|MOGS_HUMAN Mannosyl-oligosaccharide glucosidase OS=Homo sapiens GN=MOGS PE=1 SV=5//0
XM_007970145.1	-1.1214	1.24E-24	3.08E-23	sp|Q96G27|WBP1_HUMAN WW domain-binding protein 1 OS=Homo sapiens

				GN=WBP1 PE=1 SV=1//5.74574e-130
XM_007970177.1	0.61142	0.005128	0.025546	sp|Q9NSI2|F207A_HUMAN Protein FAM207A OS=Homo sapiens GN=FAM207A PE=1 SV=2//2.25164e-90
XM_007970182.1	-2.3706	0.0010032	0.0057311	sp|Q14203|DCTN1_HUMAN Dynactin subunit 1 OS=Homo sapiens GN=DCTN1 PE=1 SV=3//0
XM_007970185.1	1.7969	7.46E-47	4.15E-45	sp|P13995|MTDC_HUMAN Bifunctional methylenetetrahydrofolate dehydrogenase/cyclohydrolase, mitochondrial OS=Homo sapiens GN=MTHFD2 PE=1 SV=2//0
XM_007970198.1	1.6674	0.0001591	0.0010148	sp|P63269|ACTH_RAT Actin, gamma-enteric smooth muscle OS=Rattus norvegicus GN=Actg2 PE=2 SV=1//0
XM_007970204.1	1.0449	1.07E-26	2.89E-25	sp|O75319|DUS11_HUMAN RNA/RNP complex-1-interacting phosphatase OS=Homo sapiens GN=DUSP11 PE=1 SV=1//0
XM_007970229.1	0.58209	2.21E-06	1.75E-05	sp|Q6GMV2|SMYD5_HUMAN SET and MYND domain-containing protein 5 OS=Homo sapiens GN=SMYD5 PE=1 SV=2//0
XM_007970243.1	-3.2891	0.0037484	0.01921	sp|Q04741|EMX1_HUMAN Homeobox protein EMX1 OS=Homo sapiens GN=EMX1 PE=1 SV=2//1.89131e-144
XM_007970253.1	-0.76121	1.29E-12	1.69E-11	sp|P35270|SPRE_HUMAN Sepiapterin reductase OS=Homo sapiens GN=SPR PE=1 SV=1//9.23089e-148
XM_007970290.1	2.0454	4.78E-46	2.59E-44	sp|O00566|MPP10_HUMAN U3 small nucleolar ribonucleoprotein protein MPP10 OS=Homo sapiens GN=MPHOSPH10 PE=1 SV=2//0
XM_007970291.1	-0.75358	6.34E-06	4.80E-05	sp|Q9UJ70|NAGK_HUMAN N-acetyl-D-glucosamine kinase OS=Homo sapiens GN=NAGK PE=1 SV=4//0
XM_007970292.1	-0.35178	0.0047093	0.02367	sp|Q6UWH6|TX261_HUMAN Protein TEX261 OS=Homo sapiens GN=TEX261 PE=2 SV=1//3.02877e-134
XM_007970318.1	0.44997	4.70E-05	0.0003202	sp|Q96C01|F136A_HUMAN Protein FAM136A OS=Homo sapiens GN=FAM136A PE=1 SV=1//5.22334e-88
XM_007970319.1	-1.3137	1.10E-46	6.09E-45	sp|Q95KC9|PCYOX_MACFA Prenylcysteine oxidase OS=Macaca fascicularis GN=PCYOX1 PE=2 SV=1//0
XM_007970343.1	1.6424	2.36E-31	7.63E-30	sp|Q05195|MAD1_HUMAN Max dimerization protein 1 OS=Homo sapiens GN=MXD1 PE=1 SV=1//3.95037e-102
XM_007970346.1	-0.33586	0.0056156	0.027722	sp|Q96IK5|GMCL1_HUMAN Germ cell-less protein-like 1 OS=Homo sapiens GN=GMCL1 PE=1 SV=1//0
XM_007970348.1	-1.6346	2.93E-16	4.77E-15	sp|P09525|ANXA4_HUMAN Annexin A4 OS=Homo sapiens GN=ANXA4 PE=1 SV=4//0
XM_007970366.1	-2.8441	0.0048455	0.024237	sp|Q2M2I8|AAK1_HUMAN AP2-associated protein kinase 1 OS=Homo sapiens GN=AAK1 PE=1 SV=3//5.8265e-91
XM_007970382.1	-0.57915	2.21E-07	1.91E-06	sp|Q9H6X2|ANTR1_HUMAN Anthrax toxin receptor 1 OS=Homo sapiens GN=ANTXR1 PE=1 SV=2//0
XM_007970412.1	2.135	1.63E-69	1.64E-67	sp|Q9NRX1|PNO1_HUMAN RNA-binding protein PNO1 OS=Homo sapiens GN=PNO1 PE=1 SV=1//1.10674e-167
XM_007970415.1	0.6833	0.000558	0.0033101	sp|Q96MX6|WDR92_HUMAN WD repeat-containing protein 92 OS=Homo sapiens GN=WDR92 PE=1 SV=1//0
XM_007970431.1	-0.28236	6.06E-05	0.0004076	sp|P61161|ARP2_MOUSE Actin-related protein 2 OS=Mus musculus GN=Actr2 PE=1 SV=1//0
XM_007970438.1	2.0629	9.54E-70	9.64E-68	sp|P43007|SATT_HUMAN Neutral amino acid transporter A OS=Homo sapiens GN=SLC1A4 PE=1 SV=1//0
XM_007970439.1	0.44112	6.39E-06	4.84E-05	-//-
XM_007970442.1	0.64974	1.93E-05	0.0001375	-//-
XM_007970448.1	0.45184	7.06E-05	0.0004721	sp|Q5R5K6|LEGL_PONAB Galectin-related protein OS=Pongo abelii GN=LGALSL PE=2 SV=1//8.95708e-112
XM_007970450.1	1.6825	1.47E-78	1.81E-76	sp|Q96FA3|PELI1_HUMAN E3 ubiquitin-protein ligase pellino homolog 1 OS=Homo sapiens GN=PELI1 PE=1 SV=2//0
XM_007970472.1	-0.65299	1.23E-14	1.82E-13	sp|P40925|MDHC_HUMAN Malate dehydrogenase, cytoplasmic OS=Homo sapiens GN=MDH1 PE=1 SV=4//0
XM_007970493.1	0.3432	7.98E-06	5.96E-05	sp|P50991|TCPD_HUMAN T-complex protein 1 subunit delta OS=Homo sapiens GN=CCT4 PE=1 SV=4//0
XM_007970496.1	-1.1883	6.36E-10	6.83E-09	-//-
XM_007970499.1	1.0844	2.34E-06	1.85E-05	sp|O14980|XPO1_HUMAN Exportin-1 OS=Homo sapiens GN=XPO1 PE=1 SV=1//0
XM_007970524.1	0.98316	0.0003555	0.0021652	sp|Q3MIT2|PUS10_HUMAN Putative tRNA pseudouridine synthase Pus10 OS=Homo sapiens GN=PUS10 PE=1 SV=1//0
XM_007970527.1	0.71454	5.83E-09	5.80E-08	sp|Q92968|PEX13_HUMAN Peroxisomal membrane protein PEX13 OS=Homo sapiens GN=PEX13 PE=1 SV=2//0
XM_007970528.1	1.8351	7.71E-10	8.23E-09	sp|Q04864|REL_HUMAN Proto-oncogene c-Rel OS=Homo sapiens GN=REL PE=1 SV=1//0
XM_007970557.1	1.6775	0.0011865	0.0067135	sp|Q96PX6|CC85A_HUMAN Coiled-coil domain-containing protein 85A OS=Homo sapiens GN=CCDC85A PE=2 SV=3//0
XM_007970606.1	-0.4198	1.38E-09	1.45E-08	sp|P62979|RS27A_HUMAN Ubiquitin-40S ribosomal protein S27a OS=Homo sapiens GN=RPS27A PE=1 SV=2//2.87543e-83
XM_007970631.1	1.0132	2.34E-23	5.52E-22	sp|Q01082|SPTB2_HUMAN Spectrin beta chain, non-erythrocytic 1 OS=Homo sapiens GN=SPTBN1 PE=1 SV=2//0

XM_007970653.1	-1.6731	1.37E-18	2.53E-17	sp|O95800|GPR75_HUMAN Probable G-protein coupled receptor 75 OS=Homo sapiens GN=GPR75 PE=1 SV=1//0
XM_007970689.1	-1.4906	1.07E-31	3.51E-30	sp|P12110|CO6A2_HUMAN Collagen alpha-2(VI) chain OS=Homo sapiens GN=COL6A2 PE=1 SV=4//0
XM_007970695.1	-1.5027	6.12E-12	7.68E-11	sp|P12110|CO6A2_HUMAN Collagen alpha-2(VI) chain OS=Homo sapiens GN=COL6A2 PE=1 SV=4//1.8553e-152
XM_007970712.1	-0.35396	0.000129	0.0008337	sp|Q5XXB5|MSH2_CHLAE DNA mismatch repair protein Msh2 OS=Chlorocebus aethiops GN=MSH2 PE=2 SV=1//0
XM_007970713.1	-1.4271	1.90E-23	4.50E-22	sp|Q1WER1|EPCAM_MACMU Epithelial cell adhesion molecule OS=Macaca mulatta GN=TACSTD1 PE=2 SV=1//0
XM_007970714.1	0.59024	3.72E-18	6.73E-17	sp|P62155|CALM_XENLA Calmodulin OS=Xenopus laevis GN=calm1 PE=1 SV=2//8.23041e-102
XM_007970719.1	-1.9933	0.010047	0.047044	sp|P48449|ERG7_HUMAN Lanosterol synthase OS=Homo sapiens GN=LSS PE=1 SV=1//0
XM_007970736.1	3.2555	1.89E-08	1.81E-07	sp|O75159|SOCS5_HUMAN Suppressor of cytokine signaling 5 OS=Homo sapiens GN=SOCS5 PE=1 SV=1//0
XM_007970743.1	-0.34279	0.0047186	0.023707	sp|Q9P021|CRIPT_HUMAN Cysteine-rich PDZ-binding protein OS=Homo sapiens GN=CRIPT PE=1 SV=1//6.77684e-64
XM_007970745.1	1.2278	0.0001768	0.0011208	sp|Q99814|EPAS1_HUMAN Endothelial PAS domain-containing protein 1 OS=Homo sapiens GN=EPAS1 PE=1 SV=3//0
XM_007970759.1	3.2801	6.34E-64	5.64E-62	sp|Q02156|KPCE_HUMAN Protein kinase C epsilon type OS=Homo sapiens GN=PRKCE PE=1 SV=1//0
XM_007970760.1	0.68064	2.22E-14	3.23E-13	sp|Q5RDI0|SRBD1_PONAB S1 RNA-binding domain-containing protein 1 OS=Pongo abelii GN=SRBD1 PE=2 SV=1//0
XM_007970779.1	1.1434	7.97E-06	5.95E-05	sp|O75688|PPM1B_HUMAN Protein phosphatase 1B OS=Homo sapiens GN=PPM1B PE=1 SV=1//0
XM_007970782.1	1.6081	1.39E-41	6.28E-40	sp|O75688|PPM1B_HUMAN Protein phosphatase 1B OS=Homo sapiens GN=PPM1B PE=1 SV=1//0
XM_007970783.1	1.0962	1.28E-13	1.79E-12	sp|O60318|GANP_HUMAN Germinal-center associated nuclear protein OS=Homo sapiens GN=MCM3AP PE=1 SV=2//2.05536e-70
XM_007970785.1	0.18178	0.0085636	0.040734	sp|P42704|LPPRC_HUMAN Leucine-rich PPR motif-containing protein, mitochondrial OS=Homo sapiens GN=LRPPRC PE=1 SV=3//0
XM_007970796.1	0.35128	7.29E-06	5.48E-05	sp|Q8IVE3|PKHH2_HUMAN Pleckstrin homology domain-containing family H member 2 OS=Homo sapiens GN=PLEKHH2 PE=1 SV=2//0
XM_007970808.1	-0.84691	3.10E-06	2.42E-05	sp|P47974|TISD_HUMAN Zinc finger protein 36, C3H1 type-like 2 OS=Homo sapiens GN=ZFP36L2 PE=1 SV=3//8.85423e-164
XM_007970812.1	-1.9365	4.15E-05	0.0002839	sp|Q8TDS5|OXER1_HUMAN Oxoeicosanoid receptor 1 OS=Homo sapiens GN=OXER1 PE=2 SV=1//0
XM_007970831.1	-0.51116	0.0008466	0.0048858	sp|Q504Y2|PKDCC_HUMAN Extracellular tyrosine-protein kinase PKDCC OS=Homo sapiens GN=PKDCC PE=2 SV=2//0
XM_007970859.1	1.7347	2.90E-26	7.74E-25	sp|Q9BTF0|THUM2_HUMAN THUMP domain-containing protein 2 OS=Homo sapiens GN=THUMPD2 PE=2 SV=2//0
XM_007970929.1	1.7131	0.0001385	0.0008906	sp|Q9UKI2|BORG2_HUMAN Cdc42 effector protein 3 OS=Homo sapiens GN=CDC42EP3 PE=1 SV=1//1.11192e-118
XM_007970942.1	1.2943	1.17E-49	7.16E-48	sp|Q03701|CEBPZ_HUMAN CCAAT/enhancer-binding protein zeta OS=Homo sapiens GN=CEBPZ PE=1 SV=3//0
XM_007970945.1	2.0774	1.45E-44	7.38E-43	sp|P19525|E2AK2_HUMAN Interferon-induced, double-stranded RNA-activated protein kinase OS=Homo sapiens GN=EIF2AK2 PE=1 SV=2//0
XM_007970951.1	0.60046	0.000219	0.0013679	sp|Q8N954|GPT11_HUMAN G patch domain-containing protein 11 OS=Homo sapiens GN=GPATCH11 PE=1 SV=3//5.27554e-154
XM_007970961.1	0.87547	4.05E-21	8.49E-20	sp|Q9NZV1|CRIM1_HUMAN Cysteine-rich motor neuron 1 protein OS=Homo sapiens GN=CRIM1 PE=1 SV=1//0
XM_007970975.1	1.0332	3.40E-18	6.17E-17	sp|Q6P3X3|TTC27_HUMAN Tetratricopeptide repeat protein 27 OS=Homo sapiens GN=TTC27 PE=1 SV=1//0
XM_007970994.1	2.2469	1.37E-75	1.58E-73	sp|Q8IV61|GRP3_HUMAN Ras guanyl-releasing protein 3 OS=Homo sapiens GN=RASGRP3 PE=1 SV=1//0
XM_007970996.1	0.56603	3.78E-10	4.17E-09	sp|Q5R679|FA98A_PONAB Protein FAM98A OS=Pongo abelii GN=FAM98A PE=2 SV=1//0
XM_007971026.1	0.52637	4.96E-05	0.0003374	sp|Q4R6D9|MEMO1_MACFA Protein MEMO1 OS=Macaca fascicularis GN=MEMO1 PE=2 SV=1//0
XM_007971051.1	-1.4428	0.0021697	0.011679	sp|Q5RD13|LBH_PONAB Protein LBH OS=Pongo abelii GN=LBH PE=3 SV=1//2.44076e-64
XM_007971055.1	-3.666	4.91E-33	1.67E-31	sp|Q9UM73|ALK_HUMAN ALK tyrosine kinase receptor OS=Homo sapiens GN=ALK PE=1 SV=3//0
XM_007971056.1	-0.59882	3.20E-07	2.73E-06	sp|Q5RDU7|YPEL5_PONAB Protein yippee-like 5 OS=Pongo abelii GN=YPEL5 PE=2 SV=1//3.69467e-78
XM_007971059.1	-1.3929	0.0018227	0.0099542	sp|Q8N3C7|CLIP4_HUMAN CAP-Gly domain-containing linker protein 4 OS=Homo sapiens GN=CLIP4 PE=1 SV=1//3.65235e-10
XM_007971062.1	-0.88662	7.01E-07	5.83E-06	sp|Q6ZUX3|F179A_HUMAN Protein FAM179A OS=Homo sapiens GN=FAM179A PE=2 SV=2//0
XM_007971063.1	2.0659	3.81E-135	1.07E-132	sp|Q15061|WDR43_HUMAN WD repeat-containing protein 43 OS=Homo sapiens

				GN=WDR43 PE=1 SV=3//0
XM_007971068.1	0.38764	1.64E-08	1.58E-07	sp|P62142|PP1B_RAT Serine/threonine-protein phosphatase PP1-beta catalytic subunit OS=Rattus norvegicus GN=Ppp1cb PE=1 SV=3//0
XM_007971077.1	3.4144	5.20E-302	5.43E-299	sp|P15408|FOSL2_HUMAN Fos-related antigen 2 OS=Homo sapiens GN=FOSL2 PE=1 SV=1//3.00885e-162
XM_007971107.1	0.31659	0.0025531	0.01357	sp|Q9HCN4|GPN1_HUMAN GPN-loop GTPase 1 OS=Homo sapiens GN=GPN1 PE=1 SV=1//0
XM_007971121.1	-1.5217	1.44E-20	2.94E-19	sp|Q9H6D8|FNDC4_HUMAN Fibronectin type III domain-containing protein 4 OS=Homo sapiens GN=FNDC4 PE=2 SV=1//2.43551e-150
XM_007971125.1	0.49144	8.85E-11	1.02E-09	sp|O15355|PPM1G_HUMAN Protein phosphatase 1G OS=Homo sapiens GN=PPM1G PE=1 SV=1//0
XM_007971138.1	0.26851	0.0025906	0.013739	sp|Q15036|SNX17_HUMAN Sorting nexin-17 OS=Homo sapiens GN=SNX17 PE=1 SV=1//0
XM_007971161.1	-2.1824	0.010729	0.049918	sp|Q6UW56|ARAID_HUMAN All-trans retinoic acid-induced differentiation factor OS=Homo sapiens GN=ATRAID PE=1 SV=2//7.66238e-121
XM_007971162.1	1.1322	5.67E-27	1.56E-25	sp|P27708|PYR1_HUMAN CAD protein OS=Homo sapiens GN=CAD PE=1 SV=3//0
XM_007971186.1	-1.5506	3.37E-06	2.62E-05	sp|B0BLS0|OST4_RAT Dolichyl-diphosphooligosaccharide--protein glycosyltransferase subunit 4 OS=Rattus norvegicus GN=Ost4 PE=3 SV=1//1.30606e-17
XM_007971189.1	0.41143	1.03E-05	7.61E-05	sp|Q6NUQ4|TM214_HUMAN Transmembrane protein 214 OS=Homo sapiens GN=TMEM214 PE=1 SV=2//0
XM_007971196.1	-0.45804	0.00071	0.0041425	sp|P49450|CENPA_HUMAN Histone H3-like centromeric protein A OS=Homo sapiens GN=CENPA PE=1 SV=1//3.2712e-48
XM_007971249.1	-0.24821	0.0008885	0.0051118	sp|P40939|ECHA_HUMAN Trifunctional enzyme subunit alpha, mitochondrial OS=Homo sapiens GN=HADHA PE=1 SV=2//0
XM_007971254.1	0.42022	1.14E-08	1.11E-07	sp|Q5R5U1|RAB10_PONAB Ras-related protein Rab-10 OS=Pongo abelii GN=RAB10 PE=2 SV=1//1.79965e-133
XM_007971271.1	-1.2681	2.79E-07	2.39E-06	sp|Q9Y2G0|EFR3B_HUMAN Protein EFR3 homolog B OS=Homo sapiens GN=EFR3B PE=1 SV=2//0
XM_007971305.1	-0.84169	2.97E-12	3.81E-11	sp|P0C875|F228B_HUMAN Protein FAM228B OS=Homo sapiens GN=FAM228B PE=2 SV=1//2.00639e-169
XM_007971320.1	-0.76692	5.33E-11	6.25E-10	sp|P59708|SF3B6_MOUSE Splicing factor 3B subunit 6 OS=Mus musculus GN=Sf3b6 PE=1 SV=1//4.65163e-75
XM_007971321.1	-0.83754	5.50E-06	4.18E-05	sp|Q53FA7|QORX_HUMAN Quinone oxidoreductase PIG3 OS=Homo sapiens GN=TP53I3 PE=1 SV=2//0
XM_007971326.1	-2.6071	8.58E-08	7.72E-07	sp|P55345|ANM2_HUMAN Protein arginine N-methyltransferase 2 OS=Homo sapiens GN=PRMT2 PE=1 SV=1//7.944e-136
XM_007971334.1	-5.3185	5.21E-07	4.37E-06	sp|P55345|ANM2_HUMAN Protein arginine N-methyltransferase 2 OS=Homo sapiens GN=PRMT2 PE=1 SV=1//4.49522e-137
XM_007971340.1	-3.5748	3.84E-12	4.90E-11	sp|P55345|ANM2_HUMAN Protein arginine N-methyltransferase 2 OS=Homo sapiens GN=PRMT2 PE=1 SV=1//4.46129e-30
XM_007971357.1	0.53875	0.0033396	0.017322	sp|Q53T59|H1BP3_HUMAN HCLS1-binding protein 3 OS=Homo sapiens GN=HS1BP3 PE=1 SV=1//0
XM_007971360.1	3.3599	0	0	sp|P62747|RHOB_RAT Rho-related GTP-binding protein RhoB OS=Rattus norvegicus GN=Rhob PE=1 SV=1//2.34133e-135
XM_007971370.1	-1.5711	5.09E-50	3.14E-48	sp|P18827|SDC1_HUMAN Syndecan-1 OS=Homo sapiens GN=SDC1 PE=1 SV=3//8.29271e-141
XM_007971371.1	-0.557	3.44E-13	4.66E-12	sp|Q4R4I5|LAP4A_MACFA Lysosomal-associated transmembrane protein 4A OS=Macaca fascicularis GN=LAPTM4A PE=2 SV=1//4.25911e-134
XM_007971381.1	0.73822	3.15E-06	2.46E-05	sp|Q9HBH5|RDH14_HUMAN Retinol dehydrogenase 14 OS=Homo sapiens GN=RDH14 PE=1 SV=1//0
XM_007971431.1	-1.5205	2.15E-21	4.58E-20	sp|Q5R669|TRIB2_PONAB Tribbles homolog 2 OS=Pongo abelii GN=TRIB2 PE=2 SV=1//0
XM_007971440.1	-2.5344	0.0057196	0.02816	sp|Q14693|LPIN1_HUMAN Phosphatidate phosphatase LPIN1 OS=Homo sapiens GN=LPIN1 PE=1 SV=2//0
XM_007971447.1	2.2027	7.37E-15	1.11E-13	sp|O75461|E2F6_HUMAN Transcription factor E2F6 OS=Homo sapiens GN=E2F6 PE=1 SV=1//7.25383e-176
XM_007971448.1	1.858	0.0017428	0.0095649	sp|O75461|E2F6_HUMAN Transcription factor E2F6 OS=Homo sapiens GN=E2F6 PE=1 SV=1//3.40612e-176
XM_007971472.1	0.56698	3.02E-12	3.88E-11	sp|O75116|ROCK2_HUMAN Rho-associated protein kinase 2 OS=Homo sapiens GN=ROCK2 PE=1 SV=4//0
XM_007971497.1	1.6968	1.06E-111	2.42E-109	sp|P11926|DCOR_HUMAN Ornithine decarboxylase OS=Homo sapiens GN=ODC1 PE=1 SV=2//0
XM_007971506.1	-2.4739	0.0010595	0.006029	sp|Q717R9|CYS1_HUMAN Cystin-1 OS=Homo sapiens GN=CYS1 PE=1 SV=1//3.25438e-35
XM_007971516.1	-0.89828	4.72E-41	2.11E-39	sp|Q5RFJ2|1433T_PONAB 14-3-3 protein theta OS=Pongo abelii GN=YWHAQ PE=2 SV=2//9.36134e-161
XM_007971529.1	0.40192	1.76E-06	1.41E-05	sp|Q9UKF6|CPSF3_HUMAN Cleavage and polyadenylation specificity factor subunit 3 OS=Homo sapiens GN=CPSF3 PE=1 SV=1//0
XM_007971537.1	2.5352	3.67E-93	5.97E-91	sp|P28663|SNAB_MOUSE Beta-soluble NSF attachment protein OS=Mus

				musculus GN=Napb PE=1 SV=2//3.09162e-93
XM_007971563.1	0.47499	0.0003098	0.0018959	sp|Q5RCH7|ID2_PONAB DNA-binding protein inhibitor ID-2 OS=Pongo abelii GN=ID2 PE=2 SV=1//2.0151e-88
XM_007971580.1	Inf	0.0082526	0.039383	sp|Q8WXG1|RSAD2_HUMAN Radical S-adenosyl methionine domain-containing protein 2 OS=Homo sapiens GN=RSAD2 PE=1 SV=1//0
XM_007971596.1	-0.86789	2.00E-05	0.0001418	sp|Q9NUR3|TM74B_HUMAN Transmembrane protein 74B OS=Homo sapiens GN=TMEM74B PE=2 SV=1//1.18463e-134
XM_007971606.1	-0.2978	0.0068708	0.033267	sp|P62083|RS7_RAT 40S ribosomal protein S7 OS=Rattus norvegicus GN=Rps7 PE=1 SV=1//7.42642e-136
XM_007971607.1	0.68457	3.13E-11	3.74E-10	sp|O60930|RNH1_HUMAN Ribonuclease H1 OS=Homo sapiens GN=RNASEH1 PE=1 SV=2//0
XM_007971608.1	-0.71341	0.0004944	0.0029524	sp|F6QS54|MTND_MACMU 1,2-dihydroxy-3-keto-5-methylthiopentene dioxygenase OS=Macaca mulatta GN=ADI1 PE=3 SV=1//4.82448e-113
XM_007971611.1	-0.40051	2.76E-05	0.0001931	sp|Q9CQE7|ERGI3_MOUSE Endoplasmic reticulum-Golgi intermediate compartment protein 3 OS=Mus musculus GN=Ergic3 PE=2 SV=1//8.99835e-121
XM_007971619.1	1.6213	7.16E-105	1.47E-102	sp|Q5RC80|RBM39_PONAB RNA-binding protein 39 OS=Pongo abelii GN=RBM39 PE=2 SV=1//0
XM_007971623.1	1.0464	1.12E-45	5.96E-44	sp|Q9BVI0|PHF20_HUMAN PHD finger protein 20 OS=Homo sapiens GN=PHF20 PE=1 SV=2//0
XM_007971653.1	0.8124	8.32E-11	9.59E-10	sp|Q9H410|DSN1_HUMAN Kinetochore-associated protein DSN1 homolog OS=Homo sapiens GN=DSN1 PE=1 SV=2//0
XM_007971674.1	1.6584	4.02E-46	2.19E-44	sp|O60287|NPA1P_HUMAN Nucleolar pre-ribosomal-associated protein 1 OS=Homo sapiens GN=URB1 PE=1 SV=4//0
XM_007971685.1	3.206	0.0012055	0.0068106	-//-
XM_007971687.1	0.97483	0.0001896	0.001196	-//-
XM_007971688.1	-3.6398	0.0062873	0.030713	sp|Q14766|LTBP1_HUMAN Latent-transforming growth factor beta-binding protein 1 OS=Homo sapiens GN=LTBP1 PE=1 SV=4//3.37193e-106
XM_007971691.1	1.1573	1.26E-15	1.97E-14	sp|Q76L83|ASXL2_HUMAN Putative Polycomb group protein ASXL2 OS=Homo sapiens GN=ASXL2 PE=1 SV=1//0
XM_007971704.1	1.2284	4.65E-14	6.66E-13	sp|Q96DW6|S2538_HUMAN Solute carrier family 25 member 38 OS=Homo sapiens GN=SLC25A38 PE=1 SV=1//0
XM_007971741.1	-0.93787	0.0025478	0.013545	sp|Q9Y2C4|EXOG_HUMAN Nuclease EXOG, mitochondrial OS=Homo sapiens GN=EXOG PE=1 SV=2//0
XM_007971743.1	-0.48011	1.88E-06	1.50E-05	sp|Q13705|AVR2B_HUMAN Activin receptor type-2B OS=Homo sapiens GN=ACVR2B PE=1 SV=3//0
XM_007971749.1	0.89473	1.42E-05	0.0001026	sp|Q5R495|OXSR1_PONAB Serine/threonine-protein kinase OSR1 OS=Pongo abelii GN=OXSR1 PE=2 SV=1//0
XM_007971751.1	1.6756	2.05E-24	5.04E-23	sp|B3Y681|MYD88_PONPY Myeloid differentiation primary response protein MyD88 OS=Pongo pygmaeus GN=MYD88 PE=2 SV=1//0
XM_007971822.1	1.635	1.21E-62	1.03E-60	sp|Q5RAJ6|DJB11_PONAB DnaJ homolog subfamily B member 11 OS=Pongo abelii GN=DNAJB11 PE=2 SV=1//0
XM_007971836.1	1.9844	3.88E-20	7.74E-19	sp|P41161|ETV5_HUMAN ETS translocation variant 5 OS=Homo sapiens GN=ETV5 PE=1 SV=1//0
XM_007971839.1	0.88068	0.0030901	0.016169	sp|P62997|TRA2B_RAT Transformer-2 protein homolog beta OS=Rattus norvegicus GN=Tra2b PE=1 SV=1//7.79813e-67
XM_007971848.1	0.39493	3.95E-05	0.0002708	sp|Q5R7K7|SENP2_PONAB Sentrin-specific protease 2 OS=Pongo abelii GN=SENP2 PE=2 SV=1//0
XM_007971851.1	1.0762	2.23E-09	2.31E-08	sp|Q96HV5|TM41A_HUMAN Transmembrane protein 41A OS=Homo sapiens GN=TMEM41A PE=1 SV=1//1.02912e-168
XM_007971856.1	1.0542	5.28E-05	0.0003575	sp|O43283|M3K13_HUMAN Mitogen-activated protein kinase kinase kinase 13 OS=Homo sapiens GN=MAP3K13 PE=1 SV=1//0
XM_007971862.1	0.71002	1.21E-10	1.38E-09	sp|Q5R5M8|ECHP_PONAB Peroxisomal bifunctional enzyme OS=Pongo abelii GN=EHHADH PE=2 SV=1//0
XM_007971873.1	-2.3269	1.55E-16	2.57E-15	sp|P54753|EPHB3_HUMAN Ephrin type-B receptor 3 OS=Homo sapiens GN=EPHB3 PE=1 SV=2//0
XM_007971905.1	-0.55916	4.38E-16	7.05E-15	sp|Q13200|PSMD2_HUMAN 26S proteasome non-ATPase regulatory subunit 2 OS=Homo sapiens GN=PSMD2 PE=1 SV=3//0
XM_007971913.1	-1.4209	2.34E-14	3.39E-13	sp|P59089|CU086_HUMAN Putative uncharacterized protein encoded by LINC00205 OS=Homo sapiens GN=LINC00205 PE=5 SV=1//1.06207e-74
XM_007971929.1	0.63516	7.81E-08	7.06E-07	sp|Q9NUQ8|ABCF3_HUMAN ATP-binding cassette sub-family F member 3 OS=Homo sapiens GN=ABCF3 PE=1 SV=2//0
XM_007971932.1	-1.4221	0.000361	0.0021955	sp|Q92997|DVL3_HUMAN Segment polarity protein dishevelled homolog DVL-3 OS=Homo sapiens GN=DVL3 PE=1 SV=2//0
XM_007971937.1	-1.0668	3.17E-59	2.39E-57	sp|P39060|COIA1_HUMAN Collagen alpha-1(XVIII) chain OS=Homo sapiens GN=COL18A1 PE=1 SV=5//0
XM_007971948.1	-2.5141	0.0002488	0.0015435	-//-
XM_007971961.1	-1.5534	3.53E-86	5.12E-84	sp|P12109|CO6A1_HUMAN Collagen alpha-1(VI) chain OS=Homo sapiens GN=COL6A1 PE=1 SV=3//1.41523e-07
XM_007971962.1	0.901	0.0001964	0.001236	sp|Q9BYG0|B3GN5_HUMAN Lactosylceramide 1,3-N-acetyl-beta-D-glucosaminyltransferase OS=Homo sapiens GN=B3GNT5 PE=1 SV=1//0

XM_007971975.1	-4.219	3.88E-06	3.00E-05	sp|Q6TFL4|KLH24_HUMAN Kelch-like protein 24 OS=Homo sapiens GN=KLHL24 PE=2 SV=1//0
XM_007971976.1	0.25463	0.0015946	0.0088177	sp|Q9ULM3|YETS2_HUMAN YEATS domain-containing protein 2 OS=Homo sapiens GN=YEATS2 PE=1 SV=2//0
XM_007971991.1	-1.4277	3.11E-11	3.72E-10	sp|Q6PCB8|EMB_HUMAN Embigin OS=Homo sapiens GN=EMB PE=1 SV=1//0
XM_007972005.1	1.5819	0.0002074	0.0013023	sp|P48431|SOX2_HUMAN Transcription factor SOX-2 OS=Homo sapiens GN=SOX2 PE=1 SV=1//1.83781e-170
XM_007972009.1	-1.6114	9.52E-06	7.04E-05	sp|Q9UFE4|CCD39_HUMAN Coiled-coil domain-containing protein 39 OS=Homo sapiens GN=CCDC39 PE=2 SV=3//0
XM_007972018.1	0.3676	0.000589	0.0034799	sp|Q92995|UBP13_HUMAN Ubiquitin carboxyl-terminal hydrolase 13 OS=Homo sapiens GN=USP13 PE=1 SV=2//0
XM_007972124.1	0.33696	0.0001424	0.0009141	sp|Q8NDX5|PHC3_HUMAN Polyhomeotic-like protein 3 OS=Homo sapiens GN=PHC3 PE=1 SV=1//0
XM_007972134.1	0.51612	1.19E-12	1.56E-11	sp|Q5R4Q3|SEC62_PONAB Translocation protein SEC62 OS=Pongo abelii GN=SEC62 PE=2 SV=1//1.57922e-56
XM_007972137.1	1.6326	3.28E-99	5.99E-97	sp|Q9BRX2|PELO_HUMAN Protein pelota homolog OS=Homo sapiens GN=PELO PE=1 SV=2//0
XM_007972153.1	2.0939	7.39E-07	6.13E-06	sp|Q9NPC7|MYNN_HUMAN Myoneurin OS=Homo sapiens GN=MYNN PE=1 SV=1//0
XM_007972171.1	-1.3939	2.22E-10	2.49E-09	sp|O00461|GOLI4_HUMAN Golgi integral membrane protein 4 OS=Homo sapiens GN=GOLIM4 PE=1 SV=1//0
XM_007972202.1	0.87866	7.06E-07	5.87E-06	sp|O75683|SURF6_HUMAN Surfeit locus protein 6 OS=Homo sapiens GN=SURF6 PE=1 SV=3//4.0834e-128
XM_007972213.1	1.8002	2.49E-62	2.10E-60	sp|Q9UID6|ZN639_HUMAN Zinc finger protein 639 OS=Homo sapiens GN=ZNF639 PE=1 SV=1//0
XM_007972214.1	-0.8304	1.14E-07	1.02E-06	sp|Q4R979|RBM4_MACFA RNA-binding protein 4 OS=Macaca fascicularis GN=RBM4 PE=2 SV=1//1.04838e-10
XM_007972216.1	0.60313	2.57E-13	3.52E-12	sp|Q5R4K9|KPCI_PONAB Protein kinase C iota type OS=Pongo abelii GN=PRKCI PE=2 SV=2//0
XM_007972221.1	0.65966	8.98E-06	6.67E-05	sp|Q9BTT6|LRRC1_HUMAN Leucine-rich repeat-containing protein 1 OS=Homo sapiens GN=LRRC1 PE=1 SV=1//0
XM_007972246.1	-0.94241	0.0091071	0.043039	sp|O15217|GSTA4_HUMAN Glutathione S-transferase A4 OS=Homo sapiens GN=GSTA4 PE=1 SV=1//1.43674e-155
XM_007972250.1	-0.64282	0.000237	0.0014754	sp|P0CB96|NDUS4_PONPY NADH dehydrogenase [ubiquinone] iron-sulfur protein 4, mitochondrial OS=Pongo pygmaeus GN=NDUFS4 PE=2 SV=1//3.31282e-100
XM_007972262.1	-1.2444	0.0048205	0.024129	sp|Q8TEZ7|MPRB_HUMAN Membrane progestin receptor beta OS=Homo sapiens GN=PAQR8 PE=2 SV=1//0
XM_007972274.1	-1.1297	3.26E-13	4.42E-12	sp|P08F94|PKHD1_HUMAN Fibrocystin OS=Homo sapiens GN=PKHD1 PE=1 SV=1//0
XM_007972310.1	2.2379	1.73E-102	3.36E-100	sp|Q91ZR2|SNX18_MOUSE Sorting nexin-18 OS=Mus musculus GN=Snx18 PE=1 SV=1//0
XM_007972312.1	-3.2934	0.0002882	0.0017718	sp|Q8IZF7|AGRF2_HUMAN Adhesion G-protein coupled receptor F2 OS=Homo sapiens GN=ADGRF2 PE=2 SV=1//0
XM_007972323.1	-0.35361	7.76E-06	5.80E-05	sp|O75509|TNR21_HUMAN Tumor necrosis factor receptor superfamily member 21 OS=Homo sapiens GN=TNFRSF21 PE=1 SV=1//0
XM_007972343.1	-1.2005	0.0091523	0.043225	sp|O95847|UCP4_HUMAN Mitochondrial uncoupling protein 4 OS=Homo sapiens GN=SLC25A27 PE=2 SV=1//3.48056e-07
XM_007972357.1	-0.33967	0.0072364	0.03489	sp|Q9Y6X5|ENPP4_HUMAN Bis(5'-adenosyl)-triphosphatase ENPP4 OS=Homo sapiens GN=ENPP4 PE=1 SV=3//0
XM_007972358.1	1.5987	0.0025452	0.01354	sp|Q9EPT8|CLIC5_RAT Chloride intracellular channel protein 5 OS=Rattus norvegicus GN=Clic5 PE=1 SV=1//6.81978e-162
XM_007972363.1	1.1316	8.02E-07	6.64E-06	sp|Q13950|RUNX2_HUMAN Runt-related transcription factor 2 OS=Homo sapiens GN=RUNX2 PE=1 SV=2//0
XM_007972367.1	1.9833	3.48E-19	6.69E-18	-//-
XM_007972376.1	0.51536	3.11E-11	3.72E-10	sp|Q99459|CDC5L_HUMAN Cell division cycle 5-like protein OS=Homo sapiens GN=CDC5L PE=1 SV=2//0
XM_007972394.1	1.6149	9.78E-17	1.65E-15	sp|O00221|IKBE_HUMAN NF-kappa-B inhibitor epsilon OS=Homo sapiens GN=NFKBIE PE=1 SV=3//0
XM_007972435.1	-1.8891	5.89E-05	0.000397	sp|Q9H1X1|RSPH9_HUMAN Radial spoke head protein 9 homolog OS=Homo sapiens GN=RSPH9 PE=1 SV=1//0
XM_007972436.1	0.73978	1.73E-07	1.51E-06	sp|Q15013|MD2BP_HUMAN MAD2L1-binding protein OS=Homo sapiens GN=MAD2L1BP PE=1 SV=1//0
XM_007972478.1	1.4048	6.83E-17	1.15E-15	sp|O15160|RPAC1_HUMAN DNA-directed RNA polymerases I and III subunit RPAC1 OS=Homo sapiens GN=POLR1C PE=1 SV=1//0
XM_007972496.1	0.91956	8.27E-21	1.71E-19	sp|P11831|SRF_HUMAN Serum response factor OS=Homo sapiens GN=SRF PE=1 SV=1//0
XM_007972522.1	4.6508	6.21E-149	2.09E-146	sp|Q96EU6|RRP36_HUMAN Ribosomal RNA processing protein 36 homolog OS=Homo sapiens GN=RRP36 PE=1 SV=1//1.89929e-112
XM_007972523.1	-0.96845	2.36E-28	6.85E-27	sp|Q14738|2A5D_HUMAN Serine/threonine-protein phosphatase 2A 56 kDa regulatory subunit delta isoform OS=Homo sapiens GN=PPP2R5D PE=1 SV=1//0

XM_007972536.1	-1.2186	1.38E-24	3.43E-23	sp|Q9BT09|CNPY3_HUMAN Protein canopy homolog 3 OS=Homo sapiens GN=CNPY3 PE=1 SV=1//5.56997e-155
XM_007972538.1	0.92923	2.22E-15	3.40E-14	sp|Q6DKI1|RL7L_HUMAN 60S ribosomal protein L7-like 1 OS=Homo sapiens GN=RPL7L1 PE=1 SV=1//7.26649e-152
XM_007972540.1	1.6437	1.79E-09	1.86E-08	sp|D6RGH6|MCIN_HUMAN Multicilin OS=Homo sapiens GN=MCIDAS PE=1 SV=1//0
XM_007972544.1	0.77074	1.29E-05	9.41E-05	sp|Q15814|TBCC_HUMAN Tubulin-specific chaperone C OS=Homo sapiens GN=TBCC PE=1 SV=2//0
XM_007972579.1	-1.2978	0.0020952	0.011309	sp|Q5T0Z8|CF132_HUMAN Uncharacterized protein C6orf132 OS=Homo sapiens GN=C6orf132 PE=1 SV=4//8.56122e-78
XM_007972590.1	2.3868	4.50E-99	8.15E-97	sp|Q13895|BYST_HUMAN Bystin OS=Homo sapiens GN=BYSL PE=1 SV=3//0
XM_007972602.1	-0.53738	1.87E-08	1.79E-07	sp|Q96B49|TOM6_HUMAN Mitochondrial import receptor subunit TOM6 homolog OS=Homo sapiens GN=TOMM6 PE=1 SV=1//8.35011e-35
XM_007972631.1	0.69689	2.15E-16	3.54E-15	sp|P42285|SK2L2_HUMAN Superkiller viralicidic activity 2-like 2 OS=Homo sapiens GN=SKIV2L2 PE=1 SV=3//0
XM_007972691.1	-2.7706	5.70E-08	5.21E-07	sp|Q3Y452|TDRG1_HUMAN Testis development-related protein 1 OS=Homo sapiens GN=TDRG1 PE=1 SV=1//1.17139e-45
XM_007972719.1	1.508	5.71E-27	1.56E-25	sp|O95279|KCNK5_HUMAN Potassium channel subfamily K member 5 OS=Homo sapiens GN=KCNK5 PE=1 SV=1//0
XM_007972730.1	-0.77495	1.96E-16	3.23E-15	sp|Q4R5F2|LGUL_MACFA Lactoylglutathione lyase OS=Macaca fascicularis GN=GLO1 PE=2 SV=3//8.30326e-128
XM_007972738.1	0.62124	9.72E-16	1.53E-14	sp|Q9H8U3|ZFAN3_HUMAN AN1-type zinc finger protein 3 OS=Homo sapiens GN=ZFAND3 PE=1 SV=1//3.16195e-121
XM_007972742.1	-0.97165	6.10E-12	7.67E-11	sp|Q9P0B6|CC167_HUMAN Coiled-coil domain-containing protein 167 OS=Homo sapiens GN=CCDC167 PE=1 SV=2//1.01559e-59
XM_007972744.1	1.4565	4.59E-59	3.45E-57	sp|Q5R981|CMTR1_PONAB Cap-specific mRNA (nucleoside-2'-O-)-methyltransferase 1 OS=Pongo abelii GN=CMTR1 PE=2 SV=1//0
XM_007972753.1	1.3784	8.22E-32	2.70E-30	sp|Q95LL3|TB22B_MACFA TBC1 domain family member 22B OS=Macaca fascicularis GN=TBC1D22B PE=2 SV=1//0
XM_007972754.1	3.0321	1.64E-235	1.05E-232	sp|P11309|PIM1_HUMAN Serine/threonine-protein kinase pim-1 OS=Homo sapiens GN=PIM1 PE=1 SV=3//0
XM_007972781.1	1.5973	2.59E-114	6.10E-112	sp|P84104|SRSF3_MOUSE Serine/arginine-rich splicing factor 3 OS=Mus musculus GN=Srsf3 PE=1 SV=1//3.62012e-51
XM_007972798.1	2.8835	8.98E-09	8.81E-08	sp|P0C671|CF222_HUMAN Uncharacterized protein C6orf222 OS=Homo sapiens GN=C6orf222 PE=1 SV=1//0
XM_007972840.1	#NAME?	0.010116	0.047308	sp|P40189|IL6RB_HUMAN Interleukin-6 receptor subunit beta OS=Homo sapiens GN=IL6ST PE=1 SV=2//0
XM_007972852.1	0.67957	9.15E-12	1.14E-10	sp|Q6BDS2|URFB1_HUMAN UHRF1-binding protein 1 OS=Homo sapiens GN=UHRF1BP1 PE=1 SV=1//0
XM_007972883.1	0.43484	0.0033909	0.017546	sp|Q9H6K1|CF106_HUMAN Uncharacterized protein C6orf106 OS=Homo sapiens GN=C6orf106 PE=1 SV=2//0
XM_007972892.1	-0.36733	0.0007016	0.0040966	sp|O95989|NUDT3_HUMAN Diphosphoinositol polyphosphate phosphohydrolase 1 OS=Homo sapiens GN=NUDT3 PE=1 SV=1//3.97217e-96
XM_007972899.1	-1.1998	0.0008202	0.004745	-//-
XM_007972900.1	1.0739	3.72E-06	2.88E-05	sp|Q86T20|CF001_HUMAN Uncharacterized protein C6orf1 OS=Homo sapiens GN=C6orf1 PE=2 SV=2//2.71873e-79
XM_007972913.1	3.7991	1.151599827 52811e-317	1.48449999 982855e-314	sp|Q96PC2|IP6K3_HUMAN Inositol hexakisphosphate kinase 3 OS=Homo sapiens GN=IP6K3 PE=1 SV=2//0
XM_007972914.1	-0.58377	9.34E-06	6.91E-05	sp|Q9BRT2|UQCC2_HUMAN Ubiquinol-cytochrome-c reductase complex assembly factor 2 OS=Homo sapiens GN=UQCC2 PE=1 SV=1//5.3044e-85
XM_007972915.1	0.66729	1.40E-06	1.14E-05	sp|Q16611|BAK_HUMAN Bcl-2 homologous antagonist/killer OS=Homo sapiens GN=BAK1 PE=1 SV=1//1.47138e-110
XM_007972918.1	-0.4826	2.44E-09	2.51E-08	sp|Q14573|ITPR3_HUMAN Inositol 1,4,5-trisphosphate receptor type 3 OS=Homo sapiens GN=ITPR3 PE=1 SV=2//0
XM_007972925.1	2.6928	5.39E-06	4.11E-05	sp|Q96PV0|SYGP1_HUMAN Ras/Rap GTPase-activating protein SynGAP OS=Homo sapiens GN=SYNGAP1 PE=1 SV=4//0
XM_007972937.1	0.66251	1.37E-08	1.33E-07	sp|O43189|PHF1_HUMAN PHD finger protein 1 OS=Homo sapiens GN=PHF1 PE=1 SV=3//0
XM_007972952.1	0.4926	4.66E-07	3.92E-06	sp|O15213|WDR46_HUMAN WD repeat-containing protein 46 OS=Homo sapiens GN=WDR46 PE=1 SV=3//0
XM_007972956.1	-0.79799	0.0002821	0.0017365	sp|O96024|B3GT4_HUMAN Beta-1,3-galactosyltransferase 4 OS=Homo sapiens GN=B3GALT4 PE=2 SV=1//0
XM_007972957.1	-0.53647	4.54E-14	6.50E-13	sp|P62271|RS18_RAT 40S ribosomal protein S18 OS=Rattus norvegicus GN=Rps18 PE=1 SV=3//4.33385e-93
XM_007972986.1	-2.1699	1.91E-07	1.66E-06	sp|P28068|DMB_HUMAN HLA class II histocompatibility antigen, DM beta chain OS=Homo sapiens GN=HLA-DMB PE=1 SV=1//5.91629e-148
XM_007972990.1	2.5105	3.90E-05	0.0002676	sp|P28065|PSB9_HUMAN Proteasome subunit beta type-9 OS=Homo sapiens GN=PSMB9 PE=1 SV=2//1.74687e-119
XM_007973006.1	-1.672	0.001229	0.0069376	sp|Q9Y4H4|GPSM3_HUMAN G-protein-signaling modulator 3 OS=Homo sapiens GN=GPSM3 PE=1 SV=1//3.04211e-30

XM_007973021.1	-1.2036	0.0032437	0.016896	sp|Q99946|PRRT1_HUMAN Proline-rich transmembrane protein 1 OS=Homo sapiens GN=PRRT1 PE=2 SV=2//1.12607e-95
XM_007973048.1	1.5791	2.39E-30	7.46E-29	sp|O77932|DXO_HUMAN Decapping and exoribonuclease protein OS=Homo sapiens GN=DXO PE=2 SV=2//0
XM_007973050.1	0.75809	1.22E-05	8.94E-05	sp|P49842|STK19_HUMAN Serine/threonine-protein kinase 19 OS=Homo sapiens GN=STK19 PE=1 SV=2//0
XM_007973076.1	-1.1991	2.45E-10	2.74E-09	sp|Q9Y330|ZBT12_HUMAN Zinc finger and BTB domain-containing protein 12 OS=Homo sapiens GN=ZBTB12 PE=1 SV=1//0
XM_007973083.1	-1.3394	1.34E-06	1.09E-05	sp|Q9Y334|VWA7_HUMAN von Willebrand factor A domain-containing protein 7 OS=Homo sapiens GN=VWA7 PE=2 SV=4//0
XM_007973086.1	-0.73356	2.67E-10	2.97E-09	sp|O35900|LSM2_MOUSE U6 snRNA-associated Sm-like protein LSm2 OS=Mus musculus GN=Lsm2 PE=3 SV=1//9.5542e-58
XM_007973102.1	0.88176	0.0016632	0.00916	sp|Q5SSQ6|SAPC1_HUMAN Suppressor APC domain-containing protein 1 OS=Homo sapiens GN=SAPCD1 PE=2 SV=2//2.818e-82
XM_007973129.1	-1.089	3.10E-05	0.0002149	sp|Q8NDX9|LY65B_HUMAN Lymphocyte antigen 6 complex locus protein G5b OS=Homo sapiens GN=LY6G5B PE=1 SV=1//3.56555e-117
XM_007973144.1	-0.68873	3.11E-09	3.16E-08	sp|Q31612|1B73_HUMAN HLA class I histocompatibility antigen, B-73 alpha chain OS=Homo sapiens GN=HLA-B PE=1 SV=1//4.38819e-180
XM_007973179.1	1.8088	0.0029078	0.015299	sp|Q3MIW9|DPCR1_HUMAN Diffuse panbronchiolitis critical region protein 1 OS=Homo sapiens GN=DPCR1 PE=2 SV=2//4.82856e-100
XM_007973201.1	2.2754	8.87E-178	3.67E-175	sp|Q7YR42|IEX1_PANTR Radiation-inducible immediate-early gene IEX-1 OS=Pan troglodytes GN=IER3 PE=3 SV=1//1.23464e-83
XM_007973204.1	-1.2062	8.01E-77	9.54E-75	sp|P09244|TBB7_CHICK Tubulin beta-7 chain OS=Gallus gallus PE=2 SV=1//0
XM_007973210.1	2.94	0	0	sp|Q5R4L1|PLK2_PONAB Serine/threonine-protein kinase PLK2 OS=Pongo abelii GN=PLK2 PE=3 SV=1//0
XM_007973227.1	0.31521	0.0019337	0.010496	sp|Q7YR39|DHX16_PANTR Putative pre-mRNA-splicing factor ATP-dependent RNA helicase DHX16 OS=Pan troglodytes GN=DHX16 PE=3 SV=1//0
XM_007973241.1	0.94642	3.54E-12	4.53E-11	sp|Q5TM62|RT18B_MACMU 28S ribosomal protein S18b, mitochondrial OS=Macaca mulatta GN=MRPS18B PE=3 SV=1//5.42683e-167
XM_007973269.1	-1.0138	0.0097911	0.045994	sp|Q5TM52|RNF39_MACMU RING finger protein 39 OS=Macaca mulatta GN=RNF39 PE=3 SV=1//0
XM_007973321.1	1.9442	0.0001062	0.0006945	sp|Q01063|PDE4D_MOUSE cAMP-specific 3',5'-cyclic phosphodiesterase 4D OS=Mus musculus GN=Pde4d PE=1 SV=2//0
XM_007973330.1	3.0702	1.93E-14	2.82E-13	sp|Q08499|PDE4D_HUMAN cAMP-specific 3',5'-cyclic phosphodiesterase 4D OS=Homo sapiens GN=PDE4D PE=1 SV=2//0
XM_007973340.1	1.5102	2.34E-05	0.0001647	sp|Q08499|PDE4D_HUMAN cAMP-specific 3',5'-cyclic phosphodiesterase 4D OS=Homo sapiens GN=PDE4D PE=1 SV=2//0
XM_007973341.1	1.2355	1.33E-09	1.39E-08	sp|Q6R2W3|SCND3_HUMAN SCAN domain-containing protein 3 OS=Homo sapiens GN=ZBED9 PE=2 SV=1//0
XM_007973344.1	-1.4858	0.0018229	0.0099542	sp|P59796|GPX6_HUMAN Glutathione peroxidase 6 OS=Homo sapiens GN=GPX6 PE=2 SV=2//2.16846e-142
XM_007973356.1	1.1381	5.27E-25	1.34E-23	sp|Q16670|ZSC26_HUMAN Zinc finger and SCAN domain-containing protein 26 OS=Homo sapiens GN=ZSCAN26 PE=1 SV=2//0
XM_007973397.1	1.7254	0.0043489	0.02203	sp|P02262|H2A1_RAT Histone H2A type 1 OS=Rattus norvegicus PE=1 SV=2//2.1685e-69
XM_007973398.1	3.7212	0.0004216	0.0025454	sp|Q99878|H2A1J_HUMAN Histone H2A type 1-J OS=Homo sapiens GN=HIST1H2AJ PE=1 SV=3//1.6572e-68
XM_007973401.1	-1.2975	0.0022223	0.011936	sp|Q28DR4|H4_XENTR Histone H4 OS=Xenopus tropicalis GN=TGas006m08.1 PE=3 SV=1//1.21708e-50
XM_007973403.1	3.3319	0.0003772	0.0022885	sp|Q99877|H2B1N_HUMAN Histone H2B type 1-N OS=Homo sapiens GN=HIST1H2BN PE=1 SV=3//2.85355e-59
XM_007973405.1	4.3736	1.61E-10	1.82E-09	sp|P16401|H15_HUMAN Histone H1.5 OS=Homo sapiens GN=HIST1H1B PE=1 SV=3//9.85065e-30
XM_007973411.1	-0.82618	4.65E-06	3.57E-05	sp|Q9UJN7|ZN391_HUMAN Zinc finger protein 391 OS=Homo sapiens GN=ZNF391 PE=2 SV=2//0
XM_007973415.1	2.0452	0.0006039	0.0035612	sp|Q5R5U3|ZN271_PONAB Zinc finger protein 271 OS=Pongo abelii GN=ZNF271 PE=2 SV=1//6.43128e-06
XM_007973425.1	2.95	1.46E-07	1.29E-06	sp|P02262|H2A1_RAT Histone H2A type 1 OS=Rattus norvegicus PE=1 SV=2//7.85131e-67
XM_007973428.1	-0.91293	2.32E-05	0.000164	sp|O14618|CCS_HUMAN Copper chaperone for superoxide dismutase OS=Homo sapiens GN=CCS PE=1 SV=1//0
XM_007973434.1	0.73241	4.00E-08	3.70E-07	sp|Q9ULW3|ABT1_HUMAN Activator of basal transcription 1 OS=Homo sapiens GN=ABT1 PE=1 SV=1//1.46079e-168
XM_007973435.1	-0.39805	1.45E-05	0.0001048	sp|Q86SG4|DPCA2_HUMAN Putative Dresden prostate carcinoma protein 2 OS=Homo sapiens GN=HMGN2P46 PE=5 SV=1//5.17345e-13
XM_007973446.1	0.93152	0.0010126	0.0057803	sp|Q8WVV5|BT2A2_HUMAN Butyrophilin subfamily 2 member A2 OS=Homo sapiens GN=BTN2A2 PE=1 SV=2//0
XM_007973449.1	0.6243	3.94E-12	5.02E-11	sp|Q5R4D8|SIM15_PONAB Small integral membrane protein 15 OS=Pongo abelii GN=SMIM15 PE=3 SV=1//1.49422e-28
XM_007973463.1	2.0584	1.07E-16	1.79E-15	sp|Q28DR4|H4_XENTR Histone H4 OS=Xenopus tropicalis GN=TGas006m08.1 PE=3 SV=1//6.18689e-49

XM_007973470.1	1.2073	0.0002421	0.0015037	sp|Q28DR4|H4_XENTR Histone H4 OS=Xenopus tropicalis GN=TGas006m08.1 PE=3 SV=1//6.97424e-51
XM_007973472.1	1.6099	0.0063847	0.031133	sp|P22752|H2A1_MOUSE Histone H2A type 1 OS=Mus musculus GN=Hist1h2ab PE=1 SV=3//5.13901e-70
XM_007973474.1	4.4058	2.43E-17	4.21E-16	sp|P16402|H13_HUMAN Histone H1.3 OS=Homo sapiens GN=HIST1H1D PE=1 SV=2//4.72919e-28
XM_007973482.1	5.2488	4.73E-49	2.83E-47	sp|P02252|H14_RABIT Histone H1.4 OS=Oryctolagus cuniculus GN=HIST1H1E PE=1 SV=2//5.65308e-32
XM_007973483.1	2.5382	3.62E-60	2.83E-58	sp|P0C169|H2A1C_RAT Histone H2A type 1-C OS=Rattus norvegicus PE=1 SV=2//9.37257e-69
XM_007973487.1	Inf	1.99E-09	2.07E-08	sp|Q6LED0|H31_RAT Histone H3.1 OS=Rattus norvegicus PE=1 SV=3//1.09076e-91
XM_007973488.1	2.1547	1.46E-10	1.66E-09	sp|Q6LED0|H31_RAT Histone H3.1 OS=Rattus norvegicus PE=1 SV=3//6.33056e-71
XM_007973489.1	1.9924	6.93E-05	0.0004635	sp|P33778|H2B1B_HUMAN Histone H2B type 1-B OS=Homo sapiens GN=HIST1H2BB PE=1 SV=2//5.17706e-59
XM_007973490.1	1.3635	1.18E-31	3.85E-30	sp|P16402|H13_HUMAN Histone H1.3 OS=Homo sapiens GN=HIST1H1D PE=1 SV=2//6.08222e-34
XM_007973508.1	-1.6419	5.67E-05	0.0003824	sp|Q9Y2C5|S17A4_HUMAN Probable small intestine urate exporter OS=Homo sapiens GN=SLC17A4 PE=2 SV=1//0
XM_007973543.1	0.36871	0.0001828	0.001155	sp|Q99LU8|CF062_MOUSE Uncharacterized protein C6orf62 homolog OS=Mus musculus PE=2 SV=1//1.64131e-150
XM_007973544.1	0.47642	0.0014734	0.0081929	sp|Q99LU8|CF062_MOUSE Uncharacterized protein C6orf62 homolog OS=Mus musculus PE=2 SV=1//6.6542e-120
XM_007973545.1	0.45605	4.89E-06	3.74E-05	sp|O95551|TYDP2_HUMAN Tyrosyl-DNA phosphodiesterase 2 OS=Homo sapiens GN=TDP2 PE=1 SV=1//0
XM_007973546.1	-0.83337	1.08E-11	1.33E-10	sp|Q5R833|ACO13_PONAB Acyl-coenzyme A thioesterase 13 OS=Pongo abelii GN=ACOT13 PE=2 SV=1//1.01889e-77
XM_007973548.1	1.0402	7.94E-27	2.16E-25	sp|Q9HD23|MRS2_HUMAN Magnesium transporter MRS2 homolog, mitochondrial OS=Homo sapiens GN=MRS2 PE=1 SV=1//0
XM_007973556.1	-0.69854	0.0002545	0.001576	sp|Q3MSM3|SSDH_HYLLA Succinate-semialdehyde dehydrogenase, mitochondrial OS=Hylobates lar GN=ALDH5A1 PE=2 SV=1//0
XM_007973572.1	1.4393	1.33E-49	8.11E-48	sp|Q9HCJ5|ZSWM6_HUMAN Zinc finger SWIM domain-containing protein 6 OS=Homo sapiens GN=ZSWIM6 PE=1 SV=2//4.94718e-35
XM_007973573.1	1.1408	2.09E-69	2.08E-67	sp|Q06945|SOX4_HUMAN Transcription factor SOX-4 OS=Homo sapiens GN=SOX4 PE=1 SV=1//2.01529e-119
XM_007973575.1	0.84618	0.0039625	0.020199	sp|Q5VV42|CDKAL_HUMAN Threonylcarbamoyladenosine tRNA methylthiotransferase OS=Homo sapiens GN=CDKAL1 PE=1 SV=1//0
XM_007973581.1	1.2654	1.33E-18	2.47E-17	sp|Q6ZNC8|MBOA1_HUMAN Lysophospholipid acyltransferase 1 OS=Homo sapiens GN=MBOAT1 PE=1 SV=1//0
XM_007973587.1	2.5784	4.00E-11	4.75E-10	sp|Q7Z419|R144B_HUMAN E3 ubiquitin-protein ligase RNF144B OS=Homo sapiens GN=RNF144B PE=1 SV=1//0
XM_007973590.1	0.57253	1.34E-14	1.98E-13	sp|P35659|DEK_HUMAN Protein DEK OS=Homo sapiens GN=DEK PE=1 SV=1//1.83456e-144
XM_007973595.1	-0.98268	2.12E-05	0.0001502	sp|Q3BCR4|TPMT_CHLAE Thiopurine S-methyltransferase OS=Chlorocebus aethiops GN=TPMT PE=2 SV=1//1.21611e-161
XM_007973607.1	1.6241	1.24E-47	7.04E-46	sp|P49790|NU153_HUMAN Nuclear pore complex protein Nup153 OS=Homo sapiens GN=NUP153 PE=1 SV=2//0
XM_007973621.1	-0.95552	7.83E-07	6.49E-06	sp|P36959|GMPR1_HUMAN GMP reductase 1 OS=Homo sapiens GN=GMPR PE=1 SV=1//0
XM_007973651.1	0.61034	1.20E-07	1.06E-06	sp|Q9UMY1|NOL7_HUMAN Nucleolar protein 7 OS=Homo sapiens GN=NOL7 PE=1 SV=2//4.23163e-89
XM_007973652.1	0.74953	1.55E-17	2.70E-16	sp|Q96S59|RANB9_HUMAN Ran-binding protein 9 OS=Homo sapiens GN=RANBP9 PE=1 SV=1//0
XM_007973653.1	0.4142	0.0007219	0.0042061	sp|Q9UNQ2|DIM1_HUMAN Probable dimethyladenosine transferase OS=Homo sapiens GN=DIMT1 PE=1 SV=1//0
XM_007973660.1	1.9532	0.0014168	0.0079102	sp|Q9NXC2|GFOD1_HUMAN Glucose-fructose oxidoreductase domain-containing protein 1 OS=Homo sapiens GN=GFOD1 PE=1 SV=1//0
XM_007973675.1	1.1297	2.93E-22	6.59E-21	sp|Q9UI26|IPO11_HUMAN Importin-11 OS=Homo sapiens GN=IPO11 PE=1 SV=1//0
XM_007973676.1	2.3932	1.11E-115	2.67E-113	sp|P05305|EDN1_HUMAN Endothelin-1 OS=Homo sapiens GN=EDN1 PE=1 SV=1//6.40237e-115
XM_007973690.1	0.97784	1.09E-12	1.43E-11	sp|P0DJ93|SIM13_HUMAN Small integral membrane protein 13 OS=Homo sapiens GN=SMIM13 PE=3 SV=1//4.71481e-40
XM_007973718.1	1.4293	6.85E-25	1.73E-23	sp|Q8N0V5|GNT2A_HUMAN N-acetyllactosaminide beta-1,6-N-acetylglucosaminyl-transferase, isoform A OS=Homo sapiens GN=GCNT2 PE=2 SV=1//0
XM_007973728.1	-0.35777	0.0014114	0.0078879	sp|P05549|AP2A_HUMAN Transcription factor AP-2-alpha OS=Homo sapiens GN=TFAP2A PE=1 SV=1//0
XM_007973743.1	1.0615	3.81E-08	3.54E-07	sp|O43324|MCA3_HUMAN Eukaryotic translation elongation factor 1 epsilon-1 OS=Homo sapiens GN=EEF1E1 PE=1 SV=1//4.09762e-108

XM_007973752.1	0.62665	2.58E-05	0.0001812	sp|Q6IEG0|SNR48_HUMAN U11/U12 small nuclear ribonucleoprotein 48 kDa protein OS=Homo sapiens GN=SNRNP48 PE=1 SV=2//0
XM_007973753.1	-0.48846	3.18E-05	0.0002203	sp|P15924|DESP_HUMAN Desmoplakin OS=Homo sapiens GN=DSP PE=1 SV=3//0
XM_007973771.1	1.9057	5.13E-16	8.24E-15	sp|Q9BRS2|RIOK1_HUMAN Serine/threonine-protein kinase RIO1 OS=Homo sapiens GN=RIOK1 PE=1 SV=2//0
XM_007973784.1	-2.8448	0.0001611	0.0010265	sp|Q6UX98|ZDH24_HUMAN Probable palmitoyltransferase ZDHHC24 OS=Homo sapiens GN=ZDHHC24 PE=1 SV=1//5.3689e-58
XM_007973802.1	0.8113	3.53E-08	3.29E-07	sp|O75818|RPP40_HUMAN Ribonuclease P protein subunit p40 OS=Homo sapiens GN=RPP40 PE=1 SV=3//0
XM_007973803.1	0.8495	3.58E-17	6.14E-16	sp|Q9Y232|CDYL1_HUMAN Chromodomain Y-like protein OS=Homo sapiens GN=CDYL PE=1 SV=2//0
XM_007973818.1	2.7419	3.21E-176	1.30E-173	sp|Q5TGL8|PXDC1_HUMAN PX domain-containing protein 1 OS=Homo sapiens GN=PXDC1 PE=2 SV=3//4.46806e-161
XM_007973825.1	-0.97765	9.34E-07	7.68E-06	sp|Q6UX98|ZDH24_HUMAN Probable palmitoyltransferase ZDHHC24 OS=Homo sapiens GN=ZDHHC24 PE=1 SV=1//5.86274e-121
XM_007973839.1	0.72314	2.26E-09	2.33E-08	sp|Q4R713|CWC27_MACFA Peptidyl-prolyl cis-trans isomerase CWC27 homolog OS=Macaca fascicularis GN=CWC27 PE=2 SV=1//0
XM_007973849.1	-0.7027	7.25E-09	7.15E-08	sp|P50453|SPB9_HUMAN Serpin B9 OS=Homo sapiens GN=SERPINB9 PE=1 SV=1//0
XM_007973863.1	1.5932	2.38E-22	5.35E-21	sp|Q12948|FOXC1_HUMAN Forkhead box protein C1 OS=Homo sapiens GN=FOXC1 PE=1 SV=3//0
XM_007973869.1	1.3388	0.0054614	0.027034	sp|Q9UKP5|ATS6_HUMAN A disintegrin and metalloproteinase with thrombospondin motifs 6 OS=Homo sapiens GN=ADAMTS6 PE=2 SV=2//0
XM_007973878.1	1.7498	0.0057511	0.028302	sp|Q8NHY5|HUS1B_HUMAN Checkpoint protein HUS1B OS=Homo sapiens GN=HUS1B PE=1 SV=2//3.2484e-158
XM_007973919.1	0.83163	0.0003026	0.0018545	sp|P36406|TRI23_HUMAN E3 ubiquitin-protein ligase TRIM23 OS=Homo sapiens GN=TRIM23 PE=1 SV=1//0
XM_007973920.1	1.1045	1.50E-08	1.45E-07	sp|A2T6E3|ZSC12_MACNE Zinc finger and SCAN domain-containing protein 12 OS=Macaca nemestrina GN=ZSCAN12 PE=3 SV=1//5.0922e-06
XM_007973924.1	1.504	0.0019688	0.010674	sp|Q6LED0|H31_RAT Histone H3.1 OS=Rattus norvegicus PE=1 SV=3//1.41342e-57
XM_007973925.1	3.1226	2.68E-28	7.75E-27	sp|Q6LED0|H31_RAT Histone H3.1 OS=Rattus norvegicus PE=1 SV=3//1.52001e-74
XM_007973929.1	1.8655	6.95E-10	7.44E-09	-//-
XM_007973935.1	-0.91162	8.98E-06	6.67E-05	-//-
XM_007973939.1	-0.9753	5.90E-05	0.0003974	sp|Q9BYT8|NEUL_HUMAN Neurolysin, mitochondrial OS=Homo sapiens GN=NLN PE=1 SV=1//0
XM_007973946.1	0.70518	4.73E-07	3.99E-06	sp|Q96EQ0|SGTB_HUMAN Small glutamine-rich tetratricopeptide repeat-containing protein beta OS=Homo sapiens GN=SGTB PE=1 SV=1//0
XM_007973967.1	0.62383	6.31E-11	7.35E-10	sp|Q9UBB5|MBD2_HUMAN Methyl-CpG-binding domain protein 2 OS=Homo sapiens GN=MBD2 PE=1 SV=1//3.72589e-09
XM_007973982.1	0.29644	0.0024777	0.013207	sp|Q5U5Q3|MEX3C_HUMAN RNA-binding E3 ubiquitin-protein ligase MEX3C OS=Homo sapiens GN=MEX3C PE=1 SV=3//0
XM_007974053.1	-0.76313	3.62E-13	4.89E-12	sp|P42765|THIM_HUMAN 3-ketoacyl-CoA thiolase, mitochondrial OS=Homo sapiens GN=ACAA2 PE=1 SV=2//0
XM_007974059.1	-0.54044	2.60E-13	3.56E-12	sp|P18621|RL17_HUMAN 60S ribosomal protein L17 OS=Homo sapiens GN=RPL17 PE=1 SV=3//3.32433e-123
XM_007974093.1	-1.2061	3.65E-44	1.81E-42	sp|Q9UBX1|CATF_HUMAN Cathepsin F OS=Homo sapiens GN=CTSF PE=1 SV=1//0
XM_007974106.1	0.87808	3.49E-07	2.97E-06	sp|Q5R4B4|HDHD2_PONAB Haloacid dehalogenase-like hydrolase domain-containing protein 2 OS=Pongo abelii GN=HDHD2 PE=2 SV=1//7.22211e-179
XM_007974129.1	#NAME?	0.0062712	0.030648	sp|Q8WXA9|SREK1_HUMAN Splicing regulatory glutamine/lysine-rich protein 1 OS=Homo sapiens GN=SREK1 PE=1 SV=1//6.13583e-102
XM_007974150.1	-1.007	1.88E-44	9.50E-43	sp|A5A6H5|ATPA_PANTR ATP synthase subunit alpha, mitochondrial OS=Pan troglodytes GN=ATP5A1 PE=2 SV=1//0
XM_007974189.1	-0.94797	9.86E-23	2.26E-21	sp|Q9NY33|DPP3_HUMAN Dipeptidyl peptidase 3 OS=Homo sapiens GN=DPP3 PE=1 SV=2//0
XM_007974249.1	-0.41782	0.0007417	0.0043161	sp|Q5R9M9|S39A6_PONAB Zinc transporter ZIP6 OS=Pongo abelii GN=SLC39A6 PE=2 SV=1//0
XM_007974254.1	0.58883	0.0051338	0.025565	sp|Q5R8Y3|RPR1A_PONAB Regulation of nuclear pre-mRNA domain-containing protein 1A OS=Pongo abelii GN=RPRD1A PE=2 SV=1//0
XM_007974345.1	-1.6104	0.0008098	0.0046884	sp|Q9P2G3|KLH14_HUMAN Kelch-like protein 14 OS=Homo sapiens GN=KLHL14 PE=1 SV=2//0
XM_007974346.1	-1.4826	1.56E-24	3.86E-23	sp|Q9P2G3|KLH14_HUMAN Kelch-like protein 14 OS=Homo sapiens GN=KLHL14 PE=1 SV=2//0
XM_007974381.1	-1.8151	6.17E-09	6.12E-08	sp|Q5R9X1|CADH2_PONAB Cadherin-2 OS=Pongo abelii GN=CDH2 PE=2 SV=1//0
XM_007974399.1	1.6713	1.39E-45	7.34E-44	sp|Q92750|TAF4B_HUMAN Transcription initiation factor TFIID subunit 4B OS=Homo sapiens GN=TAF4B PE=1 SV=2//0
XM_007974473.1	1.7435	1.07E-23	2.55E-22	sp|Q92908|GATA6_HUMAN Transcription factor GATA-6 OS=Homo sapiens GN=GATA6 PE=1 SV=2//0
XM_007974489.1	0.8622	1.72E-19	3.35E-18	sp|Q96FV9|THOC1_HUMAN THO complex subunit 1 OS=Homo sapiens GN=THOC1

				PE=1 SV=1//0
XM_007974497.1	-0.61013	0.0064739	0.031534	sp|Q5RAT4|ENOF1_PONAB Mitochondrial enolase superfamily member 1 OS=Pongo abelii GN=ENOSF1 PE=2 SV=1//0
XM_007974506.1	-2.0158	0.0010129	0.0057806	sp|Q8N3J2|METL4_HUMAN Methyltransferase-like protein 4 OS=Homo sapiens GN=METTL4 PE=2 SV=3//0
XM_007974515.1	2.4727	5.48E-05	0.0003707	sp|Q92539|LPIN2_HUMAN Phosphatidate phosphatase LPIN2 OS=Homo sapiens GN=LPIN2 PE=1 SV=1//0
XM_007974520.1	1.2802	7.55E-05	0.0005032	sp|Q9BXX0|EMIL2_HUMAN EMILIN-2 OS=Homo sapiens GN=EMILIN2 PE=1 SV=3//1.63525e-117
XM_007974531.1	-0.28533	0.0001412	0.0009071	sp|Q3THE2|ML12B_MOUSE Myosin regulatory light chain 12B OS=Mus musculus GN=Myl12b PE=1 SV=2//8.65065e-109
XM_007974534.1	1.4114	2.06E-67	1.99E-65	sp|Q5IS58|TGIF1_PANTR Homeobox protein TGIF1 OS=Pan troglodytes GN=TGIF1 PE=2 SV=1//0
XM_007974535.1	1.686	2.46E-16	4.02E-15	-//-
XM_007974559.1	#NAME?	0.0065033	0.031655	sp|O43829|ZBT14_HUMAN Zinc finger and BTB domain-containing protein 14 OS=Homo sapiens GN=ZBTB14 PE=1 SV=2//0
XM_007974602.1	1.9863	0.0015603	0.0086476	sp|A6NKL6|T200C_HUMAN Transmembrane protein 200C OS=Homo sapiens GN=TMEM200C PE=2 SV=2//0
XM_007974636.1	0.74475	3.57E-14	5.15E-13	sp|Q6IQ22|RAB12_HUMAN Ras-related protein Rab-12 OS=Homo sapiens GN=RAB12 PE=1 SV=3//1.45149e-128
XM_007974644.1	1.1473	4.85E-31	1.55E-29	sp|Q16594|TAF9_HUMAN Transcription initiation factor TFIID subunit 9 OS=Homo sapiens GN=TAF9 PE=1 SV=1//1.74173e-176
XM_007974657.1	-0.80384	1.53E-21	3.29E-20	sp|Q9GZX9|TWSG1_HUMAN Twisted gastrulation protein homolog 1 OS=Homo sapiens GN=TWSG1 PE=1 SV=1//1.67903e-150
XM_007974664.1	0.83432	9.08E-29	2.67E-27	sp|Q13636|RAB31_HUMAN Ras-related protein Rab-31 OS=Homo sapiens GN=RAB31 PE=1 SV=1//1.75162e-126
XM_007974676.1	-1.8793	3.54E-95	6.12E-93	sp|O14732|IMPA2_HUMAN Inositol monophosphatase 2 OS=Homo sapiens GN=IMPA2 PE=1 SV=1//0
XM_007974681.1	-1.2618	0.0085328	0.040606	sp|Q9GMS6|MPPE1_MACFA Metallophosphoesterase 1 OS=Macaca fascicularis GN=MPPE1 PE=2 SV=1//0
XM_007974683.1	0.73233	4.13E-23	9.63E-22	sp|Q7LBR1|CHM1B_HUMAN Charged multivesicular body protein 1b OS=Homo sapiens GN=CHMP1B PE=1 SV=1//3.02954e-101
XM_007974684.1	-1.8582	1.77E-05	0.0001265	sp|Q8CGK7|GNAL_MOUSE Guanine nucleotide-binding protein G(olf) subunit alpha OS=Mus musculus GN=Gnal PE=1 SV=1//0
XM_007974712.1	0.48869	4.75E-11	5.59E-10	sp|Q9Y4W6|AFG32_HUMAN AFG3-like protein 2 OS=Homo sapiens GN=AFG3L2 PE=1 SV=2//0
XM_007974714.1	-0.81675	3.37E-13	4.57E-12	sp|Q9BUF5|TBB6_HUMAN Tubulin beta-6 chain OS=Homo sapiens GN=TUBB6 PE=1 SV=1//0
XM_007974715.1	1.4091	1.74E-14	2.55E-13	sp|Q96N28|SLMO1_HUMAN Protein slowmo homolog 1 OS=Homo sapiens GN=SLMO1 PE=2 SV=1//1.55032e-72
XM_007974718.1	1.0244	3.97E-17	6.77E-16	sp|Q8TAP6|CEP76_HUMAN Centrosomal protein of 76 kDa OS=Homo sapiens GN=CEP76 PE=1 SV=1//0
XM_007974721.1	-2.9475	1.09E-05	8.01E-05	sp|Q08AE8|SPIR1_HUMAN Protein spire homolog 1 OS=Homo sapiens GN=SPIRE1 PE=1 SV=3//1.71086e-102
XM_007974762.1	0.65435	8.85E-11	1.02E-09	sp|Q4R7K1|MCES_MACFA mRNA cap guanine-N7 methyltransferase OS=Macaca fascicularis GN=RNMT PE=2 SV=1//0
XM_007974769.1	-1.8905	2.13E-09	2.20E-08	sp|Q5R7L8|BL1S1_PONAB Biogenesis of lysosome-related organelles complex 1 subunit 1 OS=Pongo abelii GN=BLOC1S1 PE=2 SV=2//1.34024e-76
XM_007974772.1	-1.0601	0.0016066	0.0088747	sp|P62752|RL23A_RAT 60S ribosomal protein L23a OS=Rattus norvegicus GN=Rpl23a PE=2 SV=1//2.69018e-49
XM_007974774.1	2.1455	1.28E-85	1.84E-83	sp|O15118|NPC1_HUMAN Niemann-Pick C1 protein OS=Homo sapiens GN=NPC1 PE=1 SV=2//0
XM_007974775.1	-0.39965	0.0051194	0.025509	sp|Q91ZH7|ABHD3_MOUSE Phospholipase ABHD3 OS=Mus musculus GN=Abhd3 PE=2 SV=1//0
XM_007974779.1	-0.7415	1.57E-11	1.92E-10	sp|Q9Y3B7|RM11_HUMAN 39S ribosomal protein L11, mitochondrial OS=Homo sapiens GN=MRPL11 PE=1 SV=1//6.91001e-114
XM_007974781.1	#NAME?	9.11E-07	7.51E-06	-//-
XM_007974830.1	-2.5657	0.0058133	0.028582	sp|Q8N1L1|CV037_HUMAN Putative uncharacterized protein encoded by LINC00528 OS=Homo sapiens GN=LINC00528 PE=5 SV=1//7.59091e-38
XM_007974845.1	2.4373	1.61E-15	2.49E-14	sp|Q7RTP6|MICA3_HUMAN Protein-methionine sulfoxide oxidase MICAL3 OS=Homo sapiens GN=MICAL3 PE=1 SV=2//0
XM_007974860.1	7.2872	2.10E-90	3.27E-88	sp|Q9UMW8|UBP18_HUMAN Ubl carboxyl-terminal hydrolase 18 OS=Homo sapiens GN=USP18 PE=1 SV=1//3.80731e-144
XM_007974862.1	6.8601	4.54E-33	1.55E-31	sp|Q5RE63|UBP18_PONAB Ubl carboxyl-terminal hydrolase 18 OS=Pongo abelii GN=USP18 PE=2 SV=1//9.27867e-22
XM_007974863.1	-1.1756	1.06E-21	2.30E-20	sp|Q9HCC0|MCCB_HUMAN Methylcrotonoyl-CoA carboxylase beta chain, mitochondrial OS=Homo sapiens GN=MCCC2 PE=1 SV=1//0
XM_007974868.1	0.56809	0.0078452	0.037578	sp|Q14129|DGCR6_HUMAN Protein DGCR6 OS=Homo sapiens GN=DGCR6 PE=1 SV=3//9.63848e-125
XM_007974873.1	-0.91605	1.68E-06	1.35E-05	sp|P19086|GNAZ_HUMAN Guanine nucleotide-binding protein G(z) subunit

				alpha OS=Homo sapiens GN=GNAZ PE=2 SV=3//0
XM_007974898.1	4.1284	0.0085949	0.040874	sp|Q9H479|FN3K_HUMAN Fructosamine-3-kinase OS=Homo sapiens GN=FN3K PE=1 SV=1//2.36065e-09
XM_007974910.1	-1.1651	6.91E-30	2.12E-28	sp|P49593|PPM1F_HUMAN Protein phosphatase 1F OS=Homo sapiens GN=PPM1F PE=1 SV=3//0
XM_007974912.1	-3.6749	1.34E-16	2.22E-15	sp|O60688|YPEL1_HUMAN Protein yippee-like 1 OS=Homo sapiens GN=YPEL1 PE=3 SV=1//8.42994e-81
XM_007974916.1	0.9962	6.42E-10	6.90E-09	sp|Q9HCN8|SDF2L_HUMAN Stromal cell-derived factor 2-like protein 1 OS=Homo sapiens GN=SDF2L1 PE=1 SV=2//8.81196e-119
XM_007974921.1	1.6737	9.76E-21	2.01E-19	sp|A8MPS7|YDJC_HUMAN Carbohydrate deacetylase OS=Homo sapiens GN=YDJC PE=1 SV=1//0
XM_007974934.1	0.3535	3.70E-05	0.0002545	sp|P42356|PI4KA_HUMAN Phosphatidylinositol 4-kinase alpha OS=Homo sapiens GN=PI4KA PE=1 SV=3//0
XM_007974935.1	-0.46034	0.0013623	0.007633	sp|O95721|SNP29_HUMAN Synaptosomal-associated protein 29 OS=Homo sapiens GN=SNAP29 PE=1 SV=1//2.98265e-169
XM_007974936.1	0.27896	0.0007081	0.0041325	sp|P46109|CRKL_HUMAN Crk-like protein OS=Homo sapiens GN=CRKL PE=1 SV=1//0
XM_007974957.1	0.47973	0.0002601	0.001609	sp|Q9BT49|THAP7_HUMAN THAP domain-containing protein 7 OS=Homo sapiens GN=THAP7 PE=1 SV=2//0
XM_007974982.1	-0.40234	0.0018856	0.010261	sp|Q53GT1|KLH22_HUMAN Kelch-like protein 22 OS=Homo sapiens GN=KLHL22 PE=1 SV=2//0
XM_007974987.1	1.0255	0.0018753	0.01021	sp|Q16587|ZNF74_HUMAN Zinc finger protein 74 OS=Homo sapiens GN=ZNF74 PE=2 SV=3//0
XM_007974990.1	-1.4459	1.68E-14	2.47E-13	sp|Q9BZR6|RTN4R_HUMAN Reticulon-4 receptor OS=Homo sapiens GN=RTN4R PE=1 SV=1//0
XM_007974993.1	-1.1575	0.004184	0.021244	sp|Q9ULC8|ZDHC8_HUMAN Probable palmitoyltransferase ZDHHC8 OS=Homo sapiens GN=ZDHHC8 PE=1 SV=3//0
XM_007974997.1	0.53538	9.69E-07	7.95E-06	sp|Q8WYQ5|DGCR8_HUMAN Microprocessor complex subunit DGCR8 OS=Homo sapiens GN=DGCR8 PE=1 SV=1//0
XM_007974998.1	0.75227	4.13E-10	4.53E-09	sp|Q8IZ69|TRM2A_HUMAN tRNA (uracil-5-)-methyltransferase homolog A OS=Homo sapiens GN=TRMT2A PE=1 SV=2//0
XM_007975031.1	0.85735	2.32E-09	2.39E-08	sp|Q7L3V2|BOP_HUMAN Protein Bop OS=Homo sapiens GN=BOP PE=1 SV=1//0
XM_007975035.1	-1.4637	1.57E-08	1.51E-07	sp|P13224|GP1BB_HUMAN Platelet glycoprotein Ib beta chain OS=Homo sapiens GN=GP1BB PE=1 SV=1//5.11009e-67
XM_007975038.1	-2.5011	0.0021656	0.01166	sp|Q99719|SEPT5_HUMAN Septin-5 OS=Homo sapiens GN=SEPT5 PE=1 SV=1//0
XM_007975046.1	-0.73685	6.38E-11	7.43E-10	sp|Q9NQ50|RM40_HUMAN 39S ribosomal protein L40, mitochondrial OS=Homo sapiens GN=MRPL40 PE=1 SV=1//9.49874e-96
XM_007975089.1	-1.0124	1.13E-13	1.59E-12	sp|Q8WYQ3|CHC10_HUMAN Coiled-coil-helix-coiled-coil-helix domain-containing protein 10, mitochondrial OS=Homo sapiens GN=CHCHD10 PE=1 SV=1//1.57138e-34
XM_007975099.1	-2.9518	3.93E-62	3.31E-60	sp|P24347|MMP11_HUMAN Stromelysin-3 OS=Homo sapiens GN=MMP11 PE=1 SV=3//0
XM_007975114.1	-1.6693	9.28E-114	2.16E-111	sp|Q6DN04|MIF_MACMU Macrophage migration inhibitory factor OS=Macaca mulatta GN=MIF PE=3 SV=4//2.48942e-80
XM_007975125.1	0.489	0.0047412	0.023797	sp|Q16676|FOXD1_HUMAN Forkhead box protein D1 OS=Homo sapiens GN=FOXD1 PE=2 SV=1//1.83822e-69
XM_007975140.1	-1.7259	4.89E-42	2.27E-40	sp|P30046|DOPD_HUMAN D-dopachrome decarboxylase OS=Homo sapiens GN=DDT PE=1 SV=3//2.30482e-77
XM_007975143.1	-1.7884	8.99E-13	1.19E-11	sp|P30711|GSTT1_HUMAN Glutathione S-transferase theta-1 OS=Homo sapiens GN=GSTT1 PE=1 SV=4//4.79598e-168
XM_007975170.1	-3.9478	5.04E-07	4.23E-06	sp|P19440|GGT1_HUMAN Gamma-glutamyltranspeptidase 1 OS=Homo sapiens GN=GGT1 PE=1 SV=2//0
XM_007975174.1	-2.6484	5.12E-61	4.08E-59	sp|Q2VPJ9|LR75B_HUMAN Leucine-rich repeat-containing protein 75B OS=Homo sapiens GN=LRRC75B PE=2 SV=1//4.60015e-131
XM_007975177.1	2.8826	1.77E-147	5.87E-145	sp|Q8TED0|UTP15_HUMAN U3 small nucleolar RNA-associated protein 15 homolog OS=Homo sapiens GN=UTP15 PE=1 SV=3//0
XM_007975181.1	-2.0446	3.10E-06	2.42E-05	sp|P19440|GGT1_HUMAN Gamma-glutamyltranspeptidase 1 OS=Homo sapiens GN=GGT1 PE=1 SV=2//0
XM_007975184.1	-0.79685	9.99E-09	9.77E-08	sp|P62323|SMD3_XENLA Small nuclear ribonucleoprotein Sm D3 OS=Xenopus laevis GN=snrpd3 PE=2 SV=1//3.7429e-78
XM_007975199.1	0.96016	2.66E-24	6.48E-23	sp|Q69YQ0|CYTSA_HUMAN Cytospin-A OS=Homo sapiens GN=SPECC1L PE=1 SV=2//2.8643e-51
XM_007975200.1	1.0381	3.32E-18	6.03E-17	sp|Q69YQ0|CYTSA_HUMAN Cytospin-A OS=Homo sapiens GN=SPECC1L PE=1 SV=2//2.64069e-150
XM_007975208.1	0.72457	7.06E-08	6.42E-07	sp|Q9BY89|K1671_HUMAN Uncharacterized protein KIAA1671 OS=Homo sapiens GN=KIAA1671 PE=1 SV=2//0
XM_007975223.1	2.86	5.72E-13	7.63E-12	sp|Q8N1W1|ARG28_HUMAN Rho guanine nucleotide exchange factor 28 OS=Homo sapiens GN=ARHGEF28 PE=1 SV=3//0
XM_007975233.1	1.1616	2.67E-06	2.10E-05	sp|Q9UH36|SRR1L_HUMAN SRR1-like protein OS=Homo sapiens GN=SRRD PE=2 SV=1//0

XM_007975254.1	0.65224	1.56E-07	1.37E-06	sp|Q96AY4|TTC28_HUMAN Tetratricopeptide repeat protein 28 OS=Homo sapiens GN=TTC28 PE=1 SV=4//0
XM_007975271.1	3.4436	1.29E-254	9.44E-252	sp|O14682|ENC1_HUMAN Ectoderm-neural cortex protein 1 OS=Homo sapiens GN=ENC1 PE=1 SV=2//0
XM_007975273.1	2.9588	8.55E-164	3.29E-161	sp|P17861|XBP1_HUMAN X-box-binding protein 1 OS=Homo sapiens GN=XBP1 PE=1 SV=2//1.72407e-87
XM_007975306.1	-0.73187	0.0089405	0.04232	sp|Q01844|EWS_HUMAN RNA-binding protein EWS OS=Homo sapiens GN=EWSR1 PE=1 SV=1//1.06888e-11
XM_007975350.1	-3.7513	2.49E-27	6.95E-26	sp|Q66H96|CABP7_RAT Calcium-binding protein 7 OS=Rattus norvegicus GN=Cabp7 PE=1 SV=1//1.54371e-148
XM_007975375.1	-0.39309	1.31E-05	9.52E-05	sp|P07686|HEXB_HUMAN Beta-hexosaminidase subunit beta OS=Homo sapiens GN=HEXB PE=1 SV=3//0
XM_007975381.1	2.1729	2.94E-23	6.91E-22	sp|P15018|LIF_HUMAN Leukemia inhibitory factor OS=Homo sapiens GN=LIF PE=1 SV=1//2.04343e-110
XM_007975387.1	1.7346	1.76E-52	1.14E-50	sp|Q9BXI6|TB10A_HUMAN TBC1 domain family member 10A OS=Homo sapiens GN=TBC1D10A PE=1 SV=1//0
XM_007975394.1	0.78832	5.61E-25	1.42E-23	sp|A2VDN6|SF3A1_BOVIN Splicing factor 3A subunit 1 OS=Bos taurus GN=SF3A1 PE=2 SV=1//0
XM_007975418.1	0.81762	7.72E-22	1.69E-20	sp|O00541|PESC_HUMAN Pescadillo homolog OS=Homo sapiens GN=PES1 PE=1 SV=1//0
XM_007975426.1	-0.82744	4.83E-08	4.44E-07	sp|Q8NEJ0|DUS18_HUMAN Dual specificity protein phosphatase 18 OS=Homo sapiens GN=DUSP18 PE=1 SV=1//9.91058e-128
XM_007975427.1	0.80672	5.01E-20	9.95E-19	sp|Q9Y6X9|MORC2_HUMAN MORC family CW-type zinc finger protein 2 OS=Homo sapiens GN=MORC2 PE=1 SV=2//0
XM_007975443.1	-1.0439	1.31E-05	9.54E-05	sp|Q8WWX9|SELM_HUMAN Selenoprotein M OS=Homo sapiens GN=SELM PE=1 SV=3//2.82296e-80
XM_007975468.1	0.8852	8.55E-05	0.0005657	sp|Q9HBE1|PATZ1_HUMAN POZ-, AT hook-, and zinc finger-containing protein 1 OS=Homo sapiens GN=PATZ1 PE=1 SV=1//1.77224e-07
XM_007975514.1	0.79961	4.08E-15	6.18E-14	sp|Q9Y295|DRG1_HUMAN Developmentally-regulated GTP-binding protein 1 OS=Homo sapiens GN=DRG1 PE=1 SV=1//0
XM_007975576.1	0.57486	5.86E-11	6.85E-10	sp|A9CB42|RTCB_PAPAN tRNA-splicing ligase RtcB homolog OS=Papio anubis GN=RTCB PE=3 SV=1//0
XM_007975578.1	1.2891	9.02E-17	1.52E-15	sp|Q5PXZ9|TIMP3_MACMU Metalloproteinase inhibitor 3 OS=Macaca mulatta GN=TIMP3 PE=2 SV=1//2.1731e-138
XM_007975585.1	-2.2807	0.0033125	0.01721	-//-
XM_007975600.1	-0.36226	0.000351	0.0021388	sp|P09601|HMOX1_HUMAN Heme oxygenase 1 OS=Homo sapiens GN=HMOX1 PE=1 SV=1//0
XM_007975601.1	0.41189	4.24E-07	3.59E-06	sp|P33992|MCM5_HUMAN DNA replication licensing factor MCM5 OS=Homo sapiens GN=MCM5 PE=1 SV=5//0
XM_007975611.1	0.57758	0.0002297	0.0014322	sp|Q9BWW9|APOL5_HUMAN Apolipoprotein L5 OS=Homo sapiens GN=APOL5 PE=2 SV=1//0
XM_007975644.1	4.5389	1.03E-19	2.02E-18	sp|Q9BQE5|APOL2_HUMAN Apolipoprotein L2 OS=Homo sapiens GN=APOL2 PE=1 SV=1//2.52521e-154
XM_007975646.1	0.21178	0.0025619	0.013607	sp|P35579|MYH9_HUMAN Myosin-9 OS=Homo sapiens GN=MYH9 PE=1 SV=4//0
XM_007975651.1	-0.60942	5.79E-10	6.26E-09	sp|Q8IWF2|FXRD2_HUMAN FAD-dependent oxidoreductase domain-containing protein 2 OS=Homo sapiens GN=FOXRED2 PE=1 SV=1//0
XM_007975692.1	-1.9316	4.69E-46	2.54E-44	sp|Q9BXI9|C1QT6_HUMAN Complement C1q tumor necrosis factor-related protein 6 OS=Homo sapiens GN=C1QTNF6 PE=1 SV=3//4.57964e-162
XM_007975700.1	0.68326	9.44E-18	1.67E-16	sp|Q5R3F8|PPR29_HUMAN Protein phosphatase 1 regulatory subunit 29 OS=Homo sapiens GN=ELFN2 PE=1 SV=1//0
XM_007975702.1	2.9984	3.26E-217	1.93E-214	sp|Q00587|BORG5_HUMAN Cdc42 effector protein 1 OS=Homo sapiens GN=CDC42EP1 PE=1 SV=1//6.94607e-33
XM_007975707.1	0.87394	1.57E-07	1.38E-06	sp|Q96GD0|PLPP_HUMAN Pyridoxal phosphate phosphatase OS=Homo sapiens GN=PDXP PE=1 SV=2//8.40915e-155
XM_007975708.1	-1.3712	1.77E-18	3.26E-17	sp|Q9Y3L3|3BP1_HUMAN SH3 domain-binding protein 1 OS=Homo sapiens GN=SH3BP1 PE=1 SV=3//0
XM_007975711.1	-1.5104	9.24E-102	1.75E-99	sp|P09382|LEG1_HUMAN Galectin-1 OS=Homo sapiens GN=LGALS1 PE=1 SV=2//5.66732e-94
XM_007975713.1	0.72244	0.0031127	0.016272	sp|Q9UGY1|NOL12_HUMAN Nucleolar protein 12 OS=Homo sapiens GN=NOL12 PE=1 SV=1//4.19215e-86
XM_007975718.1	2.2404	7.50E-196	3.65E-193	sp|P07305|H10_HUMAN Histone H1.0 OS=Homo sapiens GN=H1F0 PE=1 SV=3//1.08053e-49
XM_007975725.1	-1.241	8.89E-14	1.26E-12	sp|Q5R592|RPAB2_PONAB DNA-directed RNA polymerases I, II, and III subunit RPABC2 OS=Pongo abelii GN=POLR2F PE=2 SV=1//4.44082e-58
XM_007975742.1	3.8852	1.30E-173	5.16E-171	sp|Q9ULX9|MAFF_HUMAN Transcription factor MafF OS=Homo sapiens GN=MAFF PE=1 SV=2//8.71737e-73
XM_007975757.1	1.3541	8.82E-11	1.01E-09	sp|Q9JMK2|KC1E_MOUSE Casein kinase I isoform epsilon OS=Mus musculus GN=Csnk1e PE=1 SV=2//0
XM_007975762.1	-1.2662	2.23E-52	1.44E-50	sp|O43731|ERD23_HUMAN ER lumen protein-retaining receptor 3 OS=Homo sapiens GN=KDELR3 PE=2 SV=1//2.52367e-119

XM_007975766.1	-1.6392	1.45E-30	4.55E-29	sp|Q5R4S2|B4GA1_PONAB Beta-1,4-glucuronyltransferase 1 OS=Pongo abelii GN=B4GAT1 PE=2 SV=1//0
XM_007975776.1	-0.33566	0.0008354	0.0048252	sp|Q4R3C7|TOM22_MACFA Mitochondrial import receptor subunit TOM22 homolog OS=Macaca fascicularis GN=TOMM22 PE=2 SV=3//1.99154e-57
XM_007975786.1	-2.9818	9.34E-42	4.28E-40	sp|O95502|NPTXR_HUMAN Neuronal pentraxin receptor OS=Homo sapiens GN=NPTXR PE=3 SV=2//0
XM_007975790.1	-0.95884	5.83E-07	4.88E-06	sp|Q694C5|ABC3G_ERYPA DNA dC->dU-editing enzyme APOBEC-3G OS=Erythrocebus patas GN=APOBEC3G PE=3 SV=1//7.30418e-30
XM_007975794.1	-1.0996	8.03E-05	0.0005334	sp|Q8IUX4|ABC3F_HUMAN DNA dC->dU-editing enzyme APOBEC-3F OS=Homo sapiens GN=APOBEC3F PE=1 SV=3//0
XM_007975813.1	-1.3954	2.78E-13	3.80E-12	sp|Q9NRW3|ABC3C_HUMAN DNA dC->dU-editing enzyme APOBEC-3C OS=Homo sapiens GN=APOBEC3C PE=1 SV=2//3.07047e-113
XM_007975814.1	-0.47715	1.23E-09	1.30E-08	sp|Q7YR25|ABC3G_CHLAE DNA dC->dU-editing enzyme APOBEC-3G (Fragment) OS=Chlorocebus aethiops GN=APOBEC3G PE=1 SV=1//1.96898e-25
XM_007975827.1	0.48789	0.0007498	0.0043594	sp|P01127|PDGFB_HUMAN Platelet-derived growth factor subunit B OS=Homo sapiens GN=PDGFB PE=1 SV=1//2.61176e-151
XM_007975829.1	-1.9861	2.19E-29	6.61E-28	sp|Q5R703|SNG1_PONAB Synaptogyrin-1 OS=Pongo abelii GN=SYNGR1 PE=2 SV=1//1.89329e-123
XM_007975831.1	-2.3332	0.0025462	0.01354	sp|O43759|SNG1_HUMAN Synaptogyrin-1 OS=Homo sapiens GN=SYNGR1 PE=1 SV=2//1.76375e-80
XM_007975834.1	0.45225	8.47E-05	0.0005612	sp|Q15750|TAB1_HUMAN TGF-beta-activated kinase 1 and MAP3K7-binding protein 1 OS=Homo sapiens GN=TAB1 PE=1 SV=1//0
XM_007975835.1	2.4862	1.07E-71	1.14E-69	sp|O00254|PAR3_HUMAN Proteinase-activated receptor 3 OS=Homo sapiens GN=F2RL2 PE=1 SV=1//0
XM_007975836.1	-2.0985	2.92E-05	0.0002033	sp|Q09327|MGAT3_HUMAN Beta-1,4-mannosyl-glycoprotein 4-beta-N-acetylglucosaminyltransferase OS=Homo sapiens GN=MGAT3 PE=2 SV=3//0
XM_007975840.1	0.5765	9.60E-10	1.02E-08	sp|Q52MA5|MID51_XENTR Mitochondrial dynamics protein MID51 OS=Xenopus tropicalis GN=mief1 PE=2 SV=1//0
XM_007975841.1	2.3995	6.88E-245	4.86E-242	sp|P18848|ATF4_HUMAN Cyclic AMP-dependent transcription factor ATF-4 OS=Homo sapiens GN=ATF4 PE=1 SV=3//0
XM_007975842.1	1.4183	2.75E-24	6.68E-23	sp|Q86WX3|AROS_HUMAN Active regulator of SIRT1 OS=Homo sapiens GN=RPS19BP1 PE=1 SV=1//4.50914e-66
XM_007975883.1	1.3137	5.17E-60	4.02E-58	sp|P56488|PAR1_PAPHA Proteinase-activated receptor 1 OS=Papio hamadryas GN=F2R PE=2 SV=1//0
XM_007975885.1	-0.62196	0.0006381	0.0037538	sp|Q5R9W8|XPP3_PONAB Probable Xaa-Pro aminopeptidase 3 OS=Pongo abelii GN=XPNPEP3 PE=2 SV=1//0
XM_007975887.1	-0.38923	1.14E-08	1.11E-07	sp|P50502|F10A1_HUMAN Hsc70-interacting protein OS=Homo sapiens GN=ST13 PE=1 SV=2//0
XM_007975893.1	1.2094	3.10E-40	1.36E-38	sp|P55085|PAR2_HUMAN Proteinase-activated receptor 2 OS=Homo sapiens GN=F2RL1 PE=1 SV=1//0
XM_007975919.1	-0.58133	1.64E-17	2.86E-16	sp|P12956|XRCC6_HUMAN X-ray repair cross-complementing protein 6 OS=Homo sapiens GN=XRCC6 PE=1 SV=2//0
XM_007975920.1	0.61138	2.58E-09	2.65E-08	sp|Q6ICB0|DESI1_HUMAN Desumoylating isopeptidase 1 OS=Homo sapiens GN=DESI1 PE=1 SV=1//2.0409e-98
XM_007975922.1	3.5958	1.30E-06	1.06E-05	sp|P24387|CRHBP_HUMAN Corticotropin-releasing factor-binding protein OS=Homo sapiens GN=CRHBP PE=1 SV=2//0
XM_007975928.1	1.4272	1.10E-11	1.36E-10	sp|Q9H6E4|CC134_HUMAN Coiled-coil domain-containing protein 134 OS=Homo sapiens GN=CCDC134 PE=1 SV=1//5.28489e-125
XM_007975929.1	-0.23106	0.0005526	0.0032857	sp|Q12772|SRBP2_HUMAN Sterol regulatory element-binding protein 2 OS=Homo sapiens GN=SREBF2 PE=1 SV=2//0
XM_007975932.1	0.72873	1.49E-15	2.32E-14	sp|Q8N302|AGGF1_HUMAN Angiogenic factor with G patch and FHA domains 1 OS=Homo sapiens GN=AGGF1 PE=1 SV=2//0
XM_007975955.1	-0.61231	3.67E-05	0.0002528	sp|Q9H4I9|EMRE_HUMAN Essential MCU regulator, mitochondrial OS=Homo sapiens GN=SMDT1 PE=1 SV=1//8.28066e-53
XM_007975960.1	-1.0583	3.31E-14	4.78E-13	sp|P56556|NDUA6_HUMAN NADH dehydrogenase [ubiquinone] 1 alpha subcomplex subunit 6 OS=Homo sapiens GN=NDUFA6 PE=1 SV=3//1.01382e-100
XM_007975970.1	-2.3962	0.0019476	0.010567	sp|Q8NET5|NFAM1_HUMAN NFAT activation molecule 1 OS=Homo sapiens GN=NFAM1 PE=1 SV=1//4.02996e-126
XM_007975982.1	3.2461	1.88E-08	1.80E-07	sp|Q9Y3A4|RRP7A_HUMAN Ribosomal RNA-processing protein 7 homolog A OS=Homo sapiens GN=RRP7A PE=1 SV=2//1.27013e-34
XM_007975983.1	3.723	4.69E-97	8.30E-95	sp|Q9Y3A4|RRP7A_HUMAN Ribosomal RNA-processing protein 7 homolog A OS=Homo sapiens GN=RRP7A PE=1 SV=2//3.29512e-167
XM_007976000.1	-0.9068	1.94E-08	1.86E-07	sp|O95922|TTLL1_HUMAN Probable tubulin polyglutamylase TTLL1 OS=Homo sapiens GN=TTLL1 PE=2 SV=1//0
XM_007976001.1	1.3656	3.02E-07	2.58E-06	sp|Q8IVS2|FABD_HUMAN Malonyl-CoA-acyl carrier protein transacylase, mitochondrial OS=Homo sapiens GN=MCAT PE=1 SV=2//0
XM_007976008.1	-1.19	5.54E-28	1.59E-26	sp|P30536|TSPOA_HUMAN Translocator protein OS=Homo sapiens GN=TSPO PE=1 SV=3//1.28558e-92
XM_007976038.1	-0.35189	0.0008208	0.0047473	sp|Q9Y512|SAM50_HUMAN Sorting and assembly machinery component 50 homolog OS=Homo sapiens GN=SAMM50 PE=1 SV=3//0
XM_007976045.1	-1.46	0.0011445	0.006486	sp|Q3SXP7|K1644_HUMAN Uncharacterized protein KIAA1644 OS=Homo sapiens

				GN=KIAA1644 PE=2 SV=2//1.52292e-95
XM_007976046.1	0.53945	5.57E-10	6.03E-09	sp|Q6ICC9|LDOCL_HUMAN Protein LDOC1L OS=Homo sapiens GN=LDOC1L PE=2 SV=1//5.30236e-136
XM_007976052.1	-2.9846	0.0025573	0.013589	sp|P85298|RHG08_HUMAN Rho GTPase-activating protein 8 OS=Homo sapiens GN=ARHGAP8 PE=1 SV=1//0
XM_007976057.1	1.1352	3.96E-45	2.06E-43	sp|Q9UKX7|NUP50_HUMAN Nuclear pore complex protein Nup50 OS=Homo sapiens GN=NUP50 PE=1 SV=2//0
XM_007976078.1	-1.4327	1.72E-07	1.51E-06	sp|P23142|FBLN1_HUMAN Fibulin-1 OS=Homo sapiens GN=FBLN1 PE=1 SV=4//0
XM_007976081.1	0.21492	0.010344	0.04826	sp|Q4R4Y2|ATX10_MACFA Ataxin-10 OS=Macaca fascicularis GN=ATXN10 PE=2 SV=1//0
XM_007976086.1	-1.142	0.0004986	0.0029748	-//-
XM_007976095.1	-0.46782	0.0062238	0.030444	sp|O75347|TBCA_HUMAN Tubulin-specific chaperone A OS=Homo sapiens GN=TBCA PE=1 SV=3//4.38572e-43
XM_007976134.1	2.4094	3.11E-06	2.43E-05	sp|O00203|AP3B1_HUMAN AP-3 complex subunit beta-1 OS=Homo sapiens GN=AP3B1 PE=1 SV=3//0
XM_007976152.1	2.8081	6.80E-104	1.37E-101	sp|Q86V86|PIM3_HUMAN Serine/threonine-protein kinase pim-3 OS=Homo sapiens GN=PIM3 PE=1 SV=3//0
XM_007976162.1	2.85	0.0014146	0.0079022	sp|Q9BV10|ALG12_HUMAN Dol-P-Man:Man(7)GlcNAc(2)-PP-Dol alpha-1,6-mannosyltransferase OS=Homo sapiens GN=ALG12 PE=1 SV=1//2.24278e-07
XM_007976164.1	-0.34814	0.0059783	0.029354	sp|O15126|SCAM1_HUMAN Secretory carrier-associated membrane protein 1 OS=Homo sapiens GN=SCAMP1 PE=1 SV=2//0
XM_007976180.1	-1.9902	8.05E-39	3.37E-37	sp|Q96RD6|PANX2_HUMAN Pannexin-2 OS=Homo sapiens GN=PANX2 PE=2 SV=2//0
XM_007976193.1	-0.67677	0.0014574	0.0081163	sp|Q15759|MK11_HUMAN Mitogen-activated protein kinase 11 OS=Homo sapiens GN=MAPK11 PE=1 SV=2//0
XM_007976198.1	0.81005	7.64E-14	1.08E-12	sp|Q6ZUX7|LHPL2_HUMAN Lipoma HMGIC fusion partner-like 2 protein OS=Homo sapiens GN=LHFPL2 PE=2 SV=2//1.46502e-120
XM_007976237.1	-1.5751	2.05E-24	5.04E-23	sp|P15848|ARSB_HUMAN Arylsulfatase B OS=Homo sapiens GN=ARSB PE=1 SV=1//0
XM_007976282.1	1.0968	4.31E-38	1.75E-36	sp|Q8N9B5|JMY_HUMAN Junction-mediating and -regulatory protein OS=Homo sapiens GN=JMY PE=1 SV=2//0
XM_007976292.1	-0.59518	1.62E-05	0.0001161	sp|Q9H2D6|TARA_HUMAN TRIO and F-actin-binding protein OS=Homo sapiens GN=TRIOBP PE=1 SV=3//1.37135e-97
XM_007976293.1	2.6496	0.0001183	0.0007684	sp|Q96MD7|CI085_HUMAN Uncharacterized protein C9orf85 OS=Homo sapiens GN=C9orf85 PE=1 SV=1//3.59061e-22
XM_007976302.1	4.3049	1.54E-05	0.0001108	sp|P13373|VPRE2_MOUSE Immunoglobulin omega chain OS=Mus musculus GN=Vpreb2 PE=2 SV=1//4.62676e-12
XM_007976305.1	0.67355	1.78E-12	2.32E-11	sp|Q13356|PPIL2_HUMAN Peptidyl-prolyl cis-trans isomerase-like 2 OS=Homo sapiens GN=PPIL2 PE=1 SV=1//0
XM_007976322.1	2.4431	3.76E-22	8.35E-21	sp|Q9BPW4|APOL4_HUMAN Apolipoprotein L4 OS=Homo sapiens GN=APOL4 PE=2 SV=3//4.10504e-160
XM_007976324.1	1.311	7.23E-63	6.19E-61	sp|Q9BWT7|CAR10_HUMAN Caspase recruitment domain-containing protein 10 OS=Homo sapiens GN=CARD10 PE=2 SV=2//7.56851e-123
XM_007976327.1	-0.38867	7.35E-08	6.66E-07	sp|Q9Y262|EIF3L_HUMAN Eukaryotic translation initiation factor 3 subunit L OS=Homo sapiens GN=EIF3L PE=1 SV=1//0
XM_007976328.1	-0.57231	4.99E-11	5.87E-10	sp|Q9UPQ9|TNR6B_HUMAN Trinucleotide repeat-containing gene 6B protein OS=Homo sapiens GN=TNRC6B PE=1 SV=4//0
XM_007976329.1	0.85273	1.96E-14	2.86E-13	sp|Q8HXY5|PUR8_MACFA Adenylosuccinate lyase OS=Macaca fascicularis GN=ADSL PE=2 SV=1//6.59884e-81
XM_007976331.1	-3.3016	0.0049171	0.024556	-//-
XM_007976333.1	-1.3983	4.30E-58	3.17E-56	sp|Q60HG4|NB5R3_MACFA NADH-cytochrome b5 reductase 3 OS=Macaca fascicularis GN=CYB5R3 PE=2 SV=3//2.29325e-162
XM_007976334.1	-0.35009	0.0087402	0.041466	sp|Q9HBI1|PARVB_HUMAN Beta-parvin OS=Homo sapiens GN=PARVB PE=1 SV=1//0
XM_007976338.1	-0.33425	0.0004553	0.0027334	sp|Q5RFF7|TTC38_PONAB Tetratricopeptide repeat protein 38 OS=Pongo abelii GN=TTC38 PE=2 SV=1//0
XM_007976339.1	-0.32163	0.0019332	0.010496	sp|Q8TCT0|CERK1_HUMAN Ceramide kinase OS=Homo sapiens GN=CERK PE=1 SV=1//0
XM_007976342.1	-0.88678	6.59E-09	6.53E-08	sp|Q9BYB0|SHAN3_HUMAN SH3 and multiple ankyrin repeat domains protein 3 OS=Homo sapiens GN=SHANK3 PE=1 SV=3//0
XM_007976343.1	-2.8388	0.0010448	0.0059516	sp|P10323|ACRO_HUMAN Acrosin OS=Homo sapiens GN=ACR PE=2 SV=4//7.27748e-88
XM_007976345.1	0.51078	3.10E-05	0.0002149	sp|P56937|DHB7_HUMAN 3-keto-steroid reductase OS=Homo sapiens GN=HSD17B7 PE=1 SV=1//0
XM_007976357.1	0.25935	0.0013289	0.0074553	sp|Q5RCY1|UHMK1_PONAB Serine/threonine-protein kinase Kist OS=Pongo abelii GN=UHMK1 PE=2 SV=1//0
XM_007976376.1	0.77399	1.39E-06	1.13E-05	sp|Q9UNI6|DUS12_HUMAN Dual specificity protein phosphatase 12 OS=Homo sapiens GN=DUSP12 PE=1 SV=1//9.33446e-153
XM_007976395.1	-1.8473	0.0022077	0.011866	sp|Q5VTH2|FLTOP_HUMAN Protein Flattop OS=Homo sapiens GN=CFAP126 PE=2 SV=1//1.92928e-116
XM_007976409.1	#NAME?	0.0094818	0.044627	sp|O75173|ATS4_HUMAN A disintegrin and metalloproteinase with

				thrombospondin motifs 4 OS=Homo sapiens GN=ADAMTS4 PE=1 SV=3//0
XM_007976410.1	-0.55471	2.89E-10	3.21E-09	sp|Q0MQG5|NDUS2_PANTR NADH dehydrogenase [ubiquinone] iron-sulfur protein 2, mitochondrial OS=Pan troglodytes GN=NDUFS2 PE=2 SV=1//0
XM_007976439.1	Inf	0.0007735	0.0044916	sp|Q96NY8|PVRL4_HUMAN Nectin-4 OS=Homo sapiens GN=PVRL4 PE=1 SV=1//0
XM_007976458.1	-1.6606	2.99E-43	1.42E-41	sp|Q9Y624|JAM1_HUMAN Junctional adhesion molecule A OS=Homo sapiens GN=F11R PE=1 SV=1//1.59897e-173
XM_007976474.1	#NAME?	0.0076068	0.036508	sp|Q9HCU0|CD248_HUMAN Endosialin OS=Homo sapiens GN=CD248 PE=1 SV=1//0
XM_007976489.1	-1.9769	1.22E-32	4.10E-31	sp|P84889|VANG2_RAT Vang-like protein 2 OS=Rattus norvegicus GN=Vangl2 PE=2 SV=1//0
XM_007976518.1	2.1348	5.06E-132	1.40E-129	sp|Q5U318|PEA15_RAT Astrocytic phosphoprotein PEA-15 OS=Rattus norvegicus GN=Pea15 PE=1 SV=1//1.35353e-75
XM_007976523.1	-1.6521	2.78E-17	4.78E-16	sp|Q969P0|IGSF8_HUMAN Immunoglobulin superfamily member 8 OS=Homo sapiens GN=IGSF8 PE=1 SV=1//0
XM_007976560.1	-1.5215	1.60E-06	1.28E-05	sp|Q9BVJ7|DUS23_HUMAN Dual specificity protein phosphatase 23 OS=Homo sapiens GN=DUSP23 PE=1 SV=1//1.08195e-91
XM_007976589.1	2.5337	1.92E-59	1.46E-57	sp|Q16666|IF16_HUMAN Gamma-interferon-inducible protein 16 OS=Homo sapiens GN=IFI16 PE=1 SV=3//0
XM_007976640.1	-1.1937	1.05E-22	2.40E-21	sp|Q4R6L9|SERC5_MACFA Serine incorporator 5 OS=Macaca fascicularis GN=SERINC5 PE=2 SV=1//0
XM_007976711.1	0.70931	2.63E-11	3.17E-10	sp|Q92733|PRCC_HUMAN Proline-rich protein PRCC OS=Homo sapiens GN=PRCC PE=1 SV=1//5.30503e-180
XM_007976713.1	-1.511	2.87E-11	3.45E-10	sp|P51858|HDGF_HUMAN Hepatoma-derived growth factor OS=Homo sapiens GN=HDGF PE=1 SV=1//2.51533e-119
XM_007976731.1	-1.7132	0.0013851	0.0077491	sp|P29373|RABP2_HUMAN Cellular retinoic acid-binding protein 2 OS=Homo sapiens GN=CRABP2 PE=1 SV=2//4.94864e-93
XM_007976732.1	-1.3029	2.96E-36	1.13E-34	sp|P48681|NEST_HUMAN Nestin OS=Homo sapiens GN=NES PE=1 SV=2//0
XM_007976739.1	-0.29974	0.002099	0.011324	sp|Q8NCW5|NNRE_HUMAN NAD(P)H-hydrate epimerase OS=Homo sapiens GN=APOA1BP PE=1 SV=2//0
XM_007976740.1	1.6439	0.0015595	0.0086454	sp|Q5T3I0|GPTC4_HUMAN G patch domain-containing protein 4 OS=Homo sapiens GN=GPATCH4 PE=1 SV=2//0
XM_007976742.1	-0.35902	4.07E-08	3.77E-07	sp|P02545|LMNA_HUMAN Prelamin-A/C OS=Homo sapiens GN=LMNA PE=1 SV=1//0
XM_007976749.1	0.76256	2.50E-14	3.62E-13	sp|Q5RD67|S2544_PONAB Solute carrier family 25 member 44 OS=Pongo abelii GN=SLC25A44 PE=2 SV=2//0
XM_007976754.1	-1.6887	0.0054903	0.027171	sp|A2T6K4|OSTCN_MACNE Osteocalcin OS=Macaca nemestrina GN=BGLAP PE=3 SV=1//6.30847e-52
XM_007976757.1	0.41913	6.72E-07	5.59E-06	sp|Q9UPR3|SMG5_HUMAN Protein SMG5 OS=Homo sapiens GN=SMG5 PE=1 SV=3//0
XM_007976795.1	-1.5871	1.09E-24	2.72E-23	sp|Q9Y2Q5|LTOR2_HUMAN Ragulator complex protein LAMTOR2 OS=Homo sapiens GN=LAMTOR2 PE=1 SV=1//1.87719e-85
XM_007976800.1	1.1339	1.52E-13	2.12E-12	sp|Q92974|ARHG2_HUMAN Rho guanine nucleotide exchange factor 2 OS=Homo sapiens GN=ARHGEF2 PE=1 SV=4//0
XM_007976806.1	1.2959	6.22E-06	4.71E-05	sp|Q92963|RIT1_HUMAN GTP-binding protein Rit1 OS=Homo sapiens GN=RIT1 PE=1 SV=1//1.06838e-149
XM_007976844.1	-3.3209	0.0002851	0.0017539	sp|P14324|FPPS_HUMAN Farnesyl pyrophosphate synthase OS=Homo sapiens GN=FDPS PE=1 SV=4//0
XM_007976875.1	-0.37753	0.001986	0.010754	sp|Q13505|MTX1_HUMAN Metaxin-1 OS=Homo sapiens GN=MTX1 PE=1 SV=2//1.64709e-08
XM_007976876.1	-0.55761	1.32E-08	1.28E-07	sp|Q5R8E3|GLCM_PONAB Glucosylceramidase OS=Pongo abelii GN=GBA PE=2 SV=1//0
XM_007976889.1	-0.67384	4.58E-09	4.59E-08	sp|Q8N6L1|KTAP2_HUMAN Keratinocyte-associated protein 2 OS=Homo sapiens GN=KRTCAP2 PE=1 SV=2//2.49303e-63
XM_007976893.1	-1.5352	9.84E-19	1.84E-17	sp|Q9P2X0|DPM3_HUMAN Dolichol-phosphate mannosyltransferase subunit 3 OS=Homo sapiens GN=DPM3 PE=1 SV=2//3.30685e-42
XM_007976898.1	1.8386	1.58E-49	9.58E-48	sp|P20827|EFNA1_HUMAN Ephrin-A1 OS=Homo sapiens GN=EFNA1 PE=1 SV=2//1.44764e-139
XM_007976911.1	-4.0845	0.0044195	0.022341	sp|Q9Y5L5|LENEP_HUMAN Lens epithelial cell protein LEP503 OS=Homo sapiens GN=LENEP PE=2 SV=1//1.01185e-32
XM_007976951.1	-0.93511	0.0001604	0.0010221	sp|Q15126|PMVK_HUMAN Phosphomevalonate kinase OS=Homo sapiens GN=PMVK PE=1 SV=3//5.96784e-122
XM_007976966.1	1.0628	8.48E-13	1.12E-11	sp|P08887|IL6RA_HUMAN Interleukin-6 receptor subunit alpha OS=Homo sapiens GN=IL6R PE=1 SV=1//0
XM_007976976.1	0.77253	3.89E-19	7.44E-18	sp|Q7Z7E8|UB2Q1_HUMAN Ubiquitin-conjugating enzyme E2 Q1 OS=Homo sapiens GN=UBE2Q1 PE=1 SV=1//0
XM_007977016.1	-0.90117	1.77E-21	3.79E-20	sp|P51153|RAB13_HUMAN Ras-related protein Rab-13 OS=Homo sapiens GN=RAB13 PE=1 SV=1//5.70574e-145
XM_007977017.1	-0.50772	5.65E-05	0.0003813	sp|O76095|JTB_HUMAN Protein JTB OS=Homo sapiens GN=JTB PE=1 SV=1//5.77004e-88
XM_007977018.1	-0.52613	0.0003404	0.0020778	sp|Q71TY3|RS27_RAT 40S ribosomal protein S27 OS=Rattus norvegicus GN=Rps27 PE=2 SV=3//4.40983e-45
XM_007977044.1	0.24404	0.0052866	0.026264	sp|Q5RE70|INT3_PONAB Integrator complex subunit 3 OS=Pongo abelii

				GN=INTS3 PE=2 SV=1//0
XM_007977064.1	-1.5862	0.008842	0.041895	sp|Q99584|S10AD_HUMAN Protein S100-A13 OS=Homo sapiens GN=S100A13 PE=1 SV=1//9.77431e-52
XM_007977084.1	-2.1485	2.74E-106	5.76E-104	sp|P26447|S10A4_HUMAN Protein S100-A4 OS=Homo sapiens GN=S100A4 PE=1 SV=1//1.96395e-68
XM_007977085.1	-1.3925	1.80E-49	1.09E-47	sp|P06703|S10A6_HUMAN Protein S100-A6 OS=Homo sapiens GN=S100A6 PE=1 SV=1//1.0985e-46
XM_007977128.1	Inf	1.98E-09	2.06E-08	-//-
XM_007977129.1	3.6555	0.0012101	0.0068343	-//-
XM_007977146.1	-1.6989	3.27E-59	2.46E-57	sp|P31949|S10AB_HUMAN Protein S100-A11 OS=Homo sapiens GN=S100A11 PE=1 SV=2//5.9555e-71
XM_007977147.1	1.3185	0.0013196	0.0074053	sp|P0C2Y1|NBPF7_HUMAN Putative neuroblastoma breakpoint family member 7 OS=Homo sapiens GN=NBPF7 PE=5 SV=1//5.78732e-106
XM_007977182.1	-2.3985	1.81E-05	0.0001296	sp|Q52LC2|VAS1L_HUMAN V-type proton ATPase subunit S1-like protein OS=Homo sapiens GN=ATP6AP1L PE=2 SV=1//5.56713e-103
XM_007977187.1	-1.608	2.50E-05	0.0001754	sp|Q96L92|SNX27_HUMAN Sorting nexin-27 OS=Homo sapiens GN=SNX27 PE=1 SV=2//0
XM_007977193.1	1.6687	9.58E-84	1.32E-81	sp|A9X1A5|CING_PAPAN Cingulin OS=Papio anubis GN=CGN PE=3 SV=1//0
XM_007977194.1	-0.77286	1.55E-09	1.62E-08	sp|P62268|RS23_RAT 40S ribosomal protein S23 OS=Rattus norvegicus GN=Rps23 PE=1 SV=3//4.69482e-99
XM_007977202.1	-1.0061	5.35E-31	1.70E-29	sp|P28070|PSB4_HUMAN Proteasome subunit beta type-4 OS=Homo sapiens GN=PSMB4 PE=1 SV=4//0
XM_007977215.1	1.0223	4.39E-27	1.21E-25	sp|Q8N1G0|ZN687_HUMAN Zinc finger protein 687 OS=Homo sapiens GN=ZNF687 PE=1 SV=1//0
XM_007977226.1	-0.45003	3.87E-07	3.29E-06	sp|P55036|PSMD4_HUMAN 26S proteasome non-ATPase regulatory subunit 4 OS=Homo sapiens GN=PSMD4 PE=1 SV=1//0
XM_007977276.1	-0.98795	0.0010434	0.0059453	sp|Q5R4W2|AF1Q_PONAB Protein AF1q OS=Pongo abelii GN=MLLT11 PE=3 SV=1//1.79069e-40
XM_007977281.1	0.97157	1.52E-45	7.98E-44	sp|Q9H3M7|TXNIP_HUMAN Thioredoxin-interacting protein OS=Homo sapiens GN=TXNIP PE=1 SV=1//0
XM_007977284.1	-1.6391	2.70E-29	8.10E-28	sp|Q8IVB5|LIX1L_HUMAN LIX1-like protein OS=Homo sapiens GN=LIX1L PE=2 SV=1//0
XM_007977286.1	-0.51633	0.0007441	0.0043275	sp|Q9BT43|RPC7L_HUMAN DNA-directed RNA polymerase III subunit RPC7-like OS=Homo sapiens GN=POLR3GL PE=1 SV=1//6.51924e-84
XM_007977333.1	1.3619	2.53E-72	2.72E-70	sp|Q04721|NOTC2_HUMAN Neurogenic locus notch homolog protein 2 OS=Homo sapiens GN=NOTCH2 PE=1 SV=3//0
XM_007977334.1	0.33945	9.90E-05	0.0006505	sp|Q4KM74|SC22B_RAT Vesicle-trafficking protein SEC22b OS=Rattus norvegicus GN=Sec22b PE=1 SV=3//6.12753e-135
XM_007977338.1	0.95399	3.34E-07	2.85E-06	sp|Q9H094|NBPF3_HUMAN Neuroblastoma breakpoint family member 3 OS=Homo sapiens GN=NBPF3 PE=2 SV=1//1.9622e-16
XM_007977354.1	0.86982	0.0045697	0.023021	sp|Q3BBV0|NBPF1_HUMAN Neuroblastoma breakpoint family member 1 OS=Homo sapiens GN=NBPF1 PE=2 SV=1//1.29656e-83
XM_007977361.1	-0.29777	0.000273	0.0016837	sp|Q60HD7|SERA_MACFA D-3-phosphoglycerate dehydrogenase OS=Macaca fascicularis GN=PHGDH PE=2 SV=4//0
XM_007977392.1	-1.9133	6.67E-33	2.27E-31	sp|P13611|CSPG2_HUMAN Versican core protein OS=Homo sapiens GN=VCAN PE=1 SV=3//0
XM_007977416.1	-0.67115	0.0018449	0.010057	sp|Q9H8W5|TRI45_HUMAN Tripartite motif-containing protein 45 OS=Homo sapiens GN=TRIM45 PE=1 SV=2//0
XM_007977424.1	-1.4897	1.44E-63	1.26E-61	sp|Q9P2B2|FPRP_HUMAN Prostaglandin F2 receptor negative regulator OS=Homo sapiens GN=PTGFRN PE=1 SV=2//0
XM_007977438.1	-1.4672	0.0007948	0.0046028	sp|P05023|AT1A1_HUMAN Sodium/potassium-transporting ATPase subunit alpha-1 OS=Homo sapiens GN=ATP1A1 PE=1 SV=1//0
XM_007977454.1	2.8974	0.001349	0.0075628	sp|Q9N2E9|NGF_PONPY Beta-nerve growth factor OS=Pongo pygmaeus GN=NGF PE=3 SV=1//2.78094e-162
XM_007977468.1	-0.4347	2.40E-06	1.89E-05	sp|Q2MJK3|RASN_PIG GTPase NRas OS=Sus scrofa GN=NRAS PE=2 SV=1//4.22162e-117
XM_007977481.1	1.0397	1.56E-22	3.53E-21	sp|Q9D287|SPF27_MOUSE Pre-mRNA-splicing factor SPF27 OS=Mus musculus GN=Bcas2 PE=2 SV=1//1.82666e-156
XM_007977492.1	-1.7382	9.65E-30	2.94E-28	sp|Q9NRN5|OLFL3_HUMAN Olfactomedin-like protein 3 OS=Homo sapiens GN=OLFML3 PE=2 SV=1//0
XM_007977504.1	0.81545	4.55E-08	4.20E-07	sp|Q9H816|DCR1B_HUMAN 5' exonuclease Apollo OS=Homo sapiens GN=DCLRE1B PE=1 SV=1//0
XM_007977505.1	0.84256	1.36E-17	2.39E-16	sp|Q5VWQ0|RSBN1_HUMAN Round spermatid basic protein 1 OS=Homo sapiens GN=RSBN1 PE=1 SV=2//0
XM_007977537.1	1.6059	0.0076692	0.036783	sp|Q9HCE1|MOV10_HUMAN Putative helicase MOV-10 OS=Homo sapiens GN=MOV10 PE=1 SV=2//1.40134e-19
XM_007977557.1	0.44738	3.33E-05	0.0002307	sp|Q5RDH2|CT2NL_PONAB CTTNBP2 N-terminal-like protein OS=Pongo abelii GN=CTTNBP2NL PE=2 SV=1//0
XM_007977585.1	1.4274	3.21E-36	1.22E-34	sp|Q9BQA1|MEP50_HUMAN Methylosome protein 50 OS=Homo sapiens GN=WDR77 PE=1 SV=1//0

XM_007977589.1	-3.5513	0.0013463	0.0075495	sp|P36718|OVGP1_PAPAN Oviduct-specific glycoprotein OS=Papio anubis GN=OVGP1 PE=2 SV=2//0
XM_007977645.1	0.75525	0.0008703	0.0050147	sp|Q9H1V8|S6A17_HUMAN Sodium-dependent neutral amino acid transporter SLC6A17 OS=Homo sapiens GN=SLC6A17 PE=1 SV=3//0
XM_007977679.1	-2.0913	0.0038721	0.019793	sp|Q01433|AMPD2_HUMAN AMP deaminase 2 OS=Homo sapiens GN=AMPD2 PE=1 SV=2//0
XM_007977682.1	-0.26663	0.001578	0.0087305	sp|P08754|GNAI3_HUMAN Guanine nucleotide-binding protein G(k) subunit alpha OS=Homo sapiens GN=GNAI3 PE=1 SV=3//0
XM_007977719.1	-1.0695	1.23E-47	6.99E-46	sp|Q9HCU4|CELR2_HUMAN Cadherin EGF LAG seven-pass G-type receptor 2 OS=Homo sapiens GN=CELSR2 PE=1 SV=1//0
XM_007977722.1	1.1585	3.96E-08	3.67E-07	sp|Q8NDZ6|T161B_HUMAN Transmembrane protein 161B OS=Homo sapiens GN=TMEM161B PE=2 SV=1//0
XM_007977725.1	1.1797	2.50E-35	9.38E-34	sp|Q5RAU6|KISHB_PONAB Protein kish-B OS=Pongo abelii GN=TMEM167B PE=3 SV=1//1.04849e-39
XM_007977728.1	1.2136	1.32E-15	2.07E-14	sp|Q5R9W6|TAF13_PONAB Transcription initiation factor TFIID subunit 13 OS=Pongo abelii GN=TAF13 PE=2 SV=1//6.87922e-65
XM_007977741.1	-2.1838	0.0003733	0.0022668	sp|Q9Y3A0|COQ4_HUMAN Ubiquinone biosynthesis protein COQ4 homolog, mitochondrial OS=Homo sapiens GN=COQ4 PE=1 SV=3//2.28761e-153
XM_007977757.1	2.2865	2.74E-195	1.31E-192	sp|Q5VTL8|PR38B_HUMAN Pre-mRNA-splicing factor 38B OS=Homo sapiens GN=PRPF38B PE=1 SV=1//9.76558e-10
XM_007977764.1	-0.4085	0.0026403	0.013993	sp|Q5T8I3|F102B_HUMAN Protein FAM102B OS=Homo sapiens GN=FAM102B PE=1 SV=2//0
XM_007977769.1	0.65841	4.20E-16	6.79E-15	sp|Q6NUK1|SCMC1_HUMAN Calcium-binding mitochondrial carrier protein SCaMC-1 OS=Homo sapiens GN=SLC25A24 PE=1 SV=2//0
XM_007977770.1	-4.2069	2.00E-16	3.29E-15	sp|Q9UKW4|VAV3_HUMAN Guanine nucleotide exchange factor VAV3 OS=Homo sapiens GN=VAV3 PE=1 SV=1//0
XM_007977771.1	-1.834	5.79E-101	1.07E-98	sp|Q9UKW4|VAV3_HUMAN Guanine nucleotide exchange factor VAV3 OS=Homo sapiens GN=VAV3 PE=1 SV=1//0
XM_007977793.1	-1.6221	0.000101	0.0006628	sp|P12107|COBA1_HUMAN Collagen alpha-1(XI) chain OS=Homo sapiens GN=COL11A1 PE=1 SV=4//4.43011e-06
XM_007977794.1	-2.6185	2.47E-12	3.18E-11	sp|P12107|COBA1_HUMAN Collagen alpha-1(XI) chain OS=Homo sapiens GN=COL11A1 PE=1 SV=4//4.53176e-06
XM_007977823.1	0.8689	1.36E-10	1.55E-09	sp|Q9NUP7|TRM13_HUMAN tRNA:m(4)X modification enzyme TRM13 homolog OS=Homo sapiens GN=TRMT13 PE=1 SV=2//0
XM_007977824.1	1.8026	0.0002527	0.0015663	sp|Q96DD0|LRC39_HUMAN Leucine-rich repeat-containing protein 39 OS=Homo sapiens GN=LRRC39 PE=2 SV=1//0
XM_007977839.1	-0.39668	0.0002381	0.0014817	sp|P70187|HIAT1_MOUSE Hippocampus abundant transcript 1 protein OS=Mus musculus GN=Hiat1 PE=2 SV=3//0
XM_007977882.1	-1.2697	2.27E-15	3.47E-14	sp|Q96MV1|TMM56_HUMAN Transmembrane protein 56 OS=Homo sapiens GN=TMEM56 PE=1 SV=1//1.64554e-164
XM_007977899.1	2.8201	1.42E-62	1.21E-60	sp|P13726|TF_HUMAN Tissue factor OS=Homo sapiens GN=F3 PE=1 SV=1//6.79728e-170
XM_007977910.1	Inf	0.0085053	0.040483	sp|Q5QJE6|TDIF2_HUMAN Deoxynucleotidyltransferase terminal-interacting protein 2 OS=Homo sapiens GN=DNTTIP2 PE=1 SV=2//0
XM_007977912.1	2.0124	0.00602	0.029533	sp|Q4R8R1|BCAR3_MACFA Breast cancer anti-estrogen resistance protein 3 OS=Macaca fascicularis GN=BCAR3 PE=2 SV=2//0
XM_007977925.1	0.53213	5.60E-11	6.55E-10	sp|Q91WV0|NC2B_MOUSE Protein Dr1 OS=Mus musculus GN=Dr1 PE=2 SV=1//2.96231e-71
XM_007977926.1	0.52854	4.38E-10	4.79E-09	sp|Q5R809|TMED5_PONAB Transmembrane emp24 domain-containing protein 5 OS=Pongo abelii GN=TMED5 PE=2 SV=1//8.70143e-125
XM_007977952.1	-0.31963	0.0038659	0.019775	sp|O60447|EVI5_HUMAN Ecotropic viral integration site 5 protein homolog OS=Homo sapiens GN=EVI5 PE=1 SV=3//0
XM_007978019.1	1.5153	0.0025458	0.01354	sp|Q9NY43|BARH2_HUMAN BarH-like 2 homeobox protein OS=Homo sapiens GN=BARHL2 PE=1 SV=2//0
XM_007978020.1	1.2847	3.75E-44	1.85E-42	sp|Q5RCA4|ZN326_PONAB DBIRD complex subunit ZNF326 OS=Pongo abelii GN=ZNF326 PE=2 SV=1//0
XM_007978033.1	1.8672	7.12E-83	9.64E-81	sp|Q7Z3D4|LYSM3_HUMAN LysM and putative peptidoglycan-binding domain-containing protein 3 OS=Homo sapiens GN=LYSMD3 PE=1 SV=2//0
XM_007978040.1	2.4934	1.12E-32	3.78E-31	sp|P32456|GBP2_HUMAN Interferon-induced guanylate-binding protein 2 OS=Homo sapiens GN=GBP2 PE=1 SV=3//0
XM_007978049.1	0.98602	1.30E-24	3.24E-23	sp|Q96E39|RMXL1_HUMAN RNA binding motif protein, X-linked-like-1 OS=Homo sapiens GN=RBMXL1 PE=1 SV=1//4.40365e-115
XM_007978050.1	-0.39622	0.0033154	0.01722	sp|Q5R886|TF2B_PONAB Transcription initiation factor IIB OS=Pongo abelii GN=GTF2B PE=2 SV=1//0
XM_007978058.1	-1.2947	5.02E-08	4.62E-07	-//-
XM_007978062.1	-0.65558	1.78E-09	1.85E-08	sp|Q7LGA3|HS2ST_HUMAN Heparan sulfate 2-O-sulfotransferase 1 OS=Homo sapiens GN=HS2ST1 PE=1 SV=1//0
XM_007978072.1	0.85528	1.12E-06	9.16E-06	sp|Q68D91|MBLC2_HUMAN Metallo-beta-lactamase domain-containing protein 2 OS=Homo sapiens GN=MBLAC2 PE=2 SV=3//1.24894e-178
XM_007978090.1	2.3416	1.97E-44	9.89E-43	sp|Q9NWK9|BCD1_HUMAN Box C/D snoRNA protein 1 OS=Homo sapiens GN=ZNHIT6 PE=1 SV=1//0

XM_007978100.1	3.5215	0	0	sp|O00622|CYR61_HUMAN Protein CYR61 OS=Homo sapiens GN=CYR61 PE=1 SV=1//0
XM_007978101.1	1.8481	1.55E-47	8.71E-46	sp|O95999|BCL10_HUMAN B-cell lymphoma/leukemia 10 OS=Homo sapiens GN=BCL10 PE=1 SV=1//1.65938e-112
XM_007978102.1	1.5817	1.75E-32	5.86E-31	sp|Q8N6N3|CA052_HUMAN UPF0690 protein C1orf52 OS=Homo sapiens GN=C1orf52 PE=1 SV=1//2.83126e-97
XM_007978104.1	1.5397	1.83E-16	3.01E-15	sp|Q5VT97|SYDE2_HUMAN Rho GTPase-activating protein SYDE2 OS=Homo sapiens GN=SYDE2 PE=1 SV=2//0
XM_007978165.1	1.2817	1.51E-17	2.65E-16	sp|Q6ZT98|TTLL7_HUMAN Tubulin polyglutamylase TTLL7 OS=Homo sapiens GN=TTLL7 PE=2 SV=2//0
XM_007978166.1	-0.53247	3.12E-11	3.73E-10	sp|Q6VY07|PACS1_HUMAN Phosphofurin acidic cluster sorting protein 1 OS=Homo sapiens GN=PACS1 PE=1 SV=2//0
XM_007978169.1	-1.0386	0.0018022	0.0098535	sp|O97817|AGRL2_BOVIN Adhesion G protein-coupled receptor L2 OS=Bos taurus GN=ADGRL2 PE=2 SV=1//0
XM_007978196.1	-0.94165	0.0006682	0.003909	-//-
XM_007978198.1	Inf	8.72E-08	7.84E-07	sp|Q8TCB0|IFI44_HUMAN Interferon-induced protein 44 OS=Homo sapiens GN=IFI44 PE=2 SV=2//0
XM_007978199.1	Inf	5.21E-17	8.84E-16	sp|Q53G44|IF44L_HUMAN Interferon-induced protein 44-like OS=Homo sapiens GN=IFI44L PE=2 SV=3//0
XM_007978205.1	1.255	1.16E-38	4.83E-37	sp|Q9UDY4|DNJB4_HUMAN DnaJ homolog subfamily B member 4 OS=Homo sapiens GN=DNAJB4 PE=1 SV=1//0
XM_007978225.1	0.79688	3.94E-08	3.65E-07	sp|Q8NAN2|FA73A_HUMAN Protein FAM73A OS=Homo sapiens GN=FAM73A PE=1 SV=1//0
XM_007978263.1	1.2225	2.58E-25	6.71E-24	sp|Q96B67|ARRD3_HUMAN Arrestin domain-containing protein 3 OS=Homo sapiens GN=ARRDC3 PE=1 SV=1//0
XM_007978289.1	-0.68179	6.63E-07	5.52E-06	sp|O14772|FPGT_HUMAN Fucose-1-phosphate guanylyltransferase OS=Homo sapiens GN=FPGT PE=1 SV=2//0
XM_007978322.1	-1.4799	9.91E-05	0.0006511	sp|Q96NW7|LRRC7_HUMAN Leucine-rich repeat-containing protein 7 OS=Homo sapiens GN=LRRC7 PE=1 SV=1//0
XM_007978345.1	0.40617	3.40E-05	0.0002351	sp|Q5RBQ0|GBG12_PONAB Guanine nucleotide-binding protein G(I)/G(S)/G(O) subunit gamma-12 OS=Pongo abelii GN=GNG12 PE=3 SV=3//1.03202e-39
XM_007978352.1	4.4615	1.380699999 30137e-315	1.59250000 000053e-312	sp|P24522|GA45A_HUMAN Growth arrest and DNA damage-inducible protein GADD45 alpha OS=Homo sapiens GN=GADD45A PE=1 SV=1//1.26262e-113
XM_007978374.1	0.57516	4.55E-06	3.50E-05	sp|Q5RDC9|S35D2_PONAB UDP-N-acetylglucosamine/UDP-glucose/GDP-mannose transporter OS=Pongo abelii GN=SLC35D2 PE=2 SV=1//0
XM_007978453.1	-0.34071	3.60E-05	0.0002483	sp|P23458|JAK1_HUMAN Tyrosine-protein kinase JAK1 OS=Homo sapiens GN=JAK1 PE=1 SV=2//0
XM_007978456.1	-2.4941	1.43E-28	4.20E-27	sp|P10589|COT1_HUMAN COUP transcription factor 1 OS=Homo sapiens GN=NR2F1 PE=1 SV=1//0
XM_007978536.1	1.4883	0.0036204	0.018632	sp|P51589|CP2J2_HUMAN Cytochrome P450 2J2 OS=Homo sapiens GN=CYP2J2 PE=1 SV=2//0
XM_007978538.1	0.93841	1.18E-17	2.07E-16	sp|Q9UJC3|HOOK1_HUMAN Protein Hook homolog 1 OS=Homo sapiens GN=HOOK1 PE=1 SV=2//0
XM_007978554.1	5.3221	0	0	sp|P05627|JUN_MOUSE Transcription factor AP-1 OS=Mus musculus GN=Jun PE=1 SV=3//2.61244e-159
XM_007978593.1	1.3551	1.19E-33	4.17E-32	sp|P54646|AAPK2_HUMAN 5'-AMP-activated protein kinase catalytic subunit alpha-2 OS=Homo sapiens GN=PRKAA2 PE=1 SV=2//0
XM_007978594.1	0.61067	1.16E-11	1.43E-10	sp|O14495|LPP3_HUMAN Lipid phosphate phosphohydrolase 3 OS=Homo sapiens GN=PPAP2B PE=1 SV=1//0
XM_007978596.1	-1.3167	5.33E-84	7.49E-82	sp|Q6B7M7|COF1_SHEEP Cofilin-1 OS=Ovis aries GN=CFL1 PE=2 SV=3//9.43706e-112
XM_007978616.1	-0.3076	2.84E-05	0.0001977	sp|Q60HC5|DHC24_MACFA Delta(24)-sterol reductase OS=Macaca fascicularis GN=DHCR24 PE=2 SV=2//0
XM_007978628.1	1.9547	6.19E-66	5.77E-64	sp|O95801|TTC4_HUMAN Tetratricopeptide repeat protein 4 OS=Homo sapiens GN=TTC4 PE=1 SV=3//0
XM_007978648.1	-2.0981	0.003054	0.015996	sp|Q5RDY9|F151A_PONAB Protein FAM151A OS=Pongo abelii GN=FAM151A PE=2 SV=1//0
XM_007978662.1	-2.3067	0.0032874	0.017107	sp|Q5VXM1|CDCP2_HUMAN CUB domain-containing protein 2 OS=Homo sapiens GN=CDCP2 PE=2 SV=1//0
XM_007978686.1	-1.3518	9.99E-17	1.68E-15	sp|P49895|IOD1_HUMAN Type I iodothyronine deiodinase OS=Homo sapiens GN=DIO1 PE=2 SV=3//9.10533e-152
XM_007978713.1	-0.34602	0.0096074	0.045179	sp|Q60HG9|CPT2_MACFA Carnitine O-palmitoyltransferase 2, mitochondrial OS=Macaca fascicularis GN=CPT2 PE=2 SV=1//0
XM_007978735.1	0.8413	1.60E-13	2.23E-12	sp|Q96BR5|COA7_HUMAN Cytochrome c oxidase assembly factor 7 OS=Homo sapiens GN=COA7 PE=1 SV=2//1.34971e-162
XM_007978751.1	-4.2425	0.0022517	0.012088	sp|Q5TAX3|TUT4_HUMAN Terminal uridylyltransferase 4 OS=Homo sapiens GN=ZCCHC11 PE=1 SV=3//0
XM_007978752.1	0.61893	2.32E-12	3.00E-11	sp|Q5RDD2|PR38A_PONAB Pre-mRNA-splicing factor 38A OS=Pongo abelii GN=PRPF38A PE=2 SV=1//3.22955e-136

XM_007978753.1	0.65604	0.0022658	0.012149	sp|Q5T0F9|C2D1B_HUMAN Coiled-coil and C2 domain-containing protein 1B OS=Homo sapiens GN=CC2D1B PE=1 SV=1//0
XM_007978755.1	1.0264	1.94E-09	2.02E-08	sp|Q13415|ORC1_HUMAN Origin recognition complex subunit 1 OS=Homo sapiens GN=ORC1 PE=1 SV=2//0
XM_007978758.1	1.0992	2.63E-14	3.81E-13	sp|Q96EK9|KTI12_HUMAN Protein KTI12 homolog OS=Homo sapiens GN=KTI12 PE=1 SV=1//0
XM_007978759.1	-0.84509	1.69E-12	2.21E-11	sp|O95881|TXD12_HUMAN Thioredoxin domain-containing protein 12 OS=Homo sapiens GN=TXNDC12 PE=1 SV=1//7.72207e-117
XM_007978763.1	-1.5152	0.0001513	0.0009681	sp|P20337|RAB3B_HUMAN Ras-related protein Rab-3B OS=Homo sapiens GN=RAB3B PE=1 SV=2//3.80658e-159
XM_007978764.1	-3.5801	0.0009514	0.0054494	sp|O43847|NRDC_HUMAN Nardilysin OS=Homo sapiens GN=NRD1 PE=1 SV=2//6.7452e-71
XM_007978781.1	0.88165	5.00E-27	1.38E-25	sp|Q9Y3C5|RNF11_HUMAN RING finger protein 11 OS=Homo sapiens GN=RNF11 PE=1 SV=1//6.45029e-83
XM_007978784.1	-0.97204	2.05E-20	4.14E-19	sp|P42773|CDN2C_HUMAN Cyclin-dependent kinase 4 inhibitor C OS=Homo sapiens GN=CDKN2C PE=1 SV=1//6.39344e-98
XM_007978828.1	-1.2354	0.0014712	0.008186	sp|Q7L4P6|BEND5_HUMAN BEN domain-containing protein 5 OS=Homo sapiens GN=BEND5 PE=1 SV=1//0
XM_007978832.1	-2.586	1.27E-37	5.14E-36	sp|A6NFA1|TIKI2_HUMAN Metalloprotease TIKI2 OS=Homo sapiens GN=TRABD2B PE=1 SV=2//0
XM_007978833.1	-1.233	6.08E-10	6.55E-09	sp|A7XV07|SKIT8_MOUSE Selection and upkeep of intraepithelial T-cells protein 8 OS=Mus musculus GN=Skint8 PE=2 SV=2//3.59903e-75
XM_007978837.1	-0.40261	1.42E-05	0.0001027	sp|P30085|KCY_HUMAN UMP-CMP kinase OS=Homo sapiens GN=CMPK1 PE=1 SV=3//5.44429e-132
XM_007978851.1	-1.1803	1.08E-53	7.21E-52	sp|P62752|RL23A_RAT 60S ribosomal protein L23a OS=Rattus norvegicus GN=Rpl23a PE=2 SV=1//5.39372e-85
XM_007978869.1	0.973	0.0001819	0.0011495	sp|Q70IA8|MOB3C_HUMAN MOB kinase activator 3C OS=Homo sapiens GN=MOB3C PE=1 SV=1//5.11148e-152
XM_007978871.1	0.49648	0.0014039	0.0078502	sp|Q5TC12|ATPF1_HUMAN ATP synthase mitochondrial F1 complex assembly factor 1 OS=Homo sapiens GN=ATPAF1 PE=1 SV=1//0
XM_007978923.1	0.94813	1.60E-44	8.10E-43	sp|O94955|RHBT3_HUMAN Rho-related BTB domain-containing protein 3 OS=Homo sapiens GN=RHOBTB3 PE=1 SV=2//0
XM_007978940.1	0.41245	0.0001646	0.0010485	sp|Q5SWH9|TMM69_HUMAN Transmembrane protein 69 OS=Homo sapiens GN=TMEM69 PE=2 SV=1//8.46759e-160
XM_007978941.1	0.64145	5.97E-15	8.99E-14	sp|Q5R9C3|GPBL1_PONAB Vasculin-like protein 1 OS=Pongo abelii GN=GPBP1L1 PE=2 SV=1//0
XM_007978955.1	-0.98021	2.68E-38	1.10E-36	sp|Q06830|PRDX1_HUMAN Peroxiredoxin-1 OS=Homo sapiens GN=PRDX1 PE=1 SV=1//1.04714e-142
XM_007978973.1	3.1116	1.78E-07	1.56E-06	sp|O00472|ELL2_HUMAN RNA polymerase II elongation factor ELL2 OS=Homo sapiens GN=ELL2 PE=1 SV=2//0
XM_007978981.1	2.6222	2.39E-25	6.23E-24	sp|Q9H4B4|PLK3_HUMAN Serine/threonine-protein kinase PLK3 OS=Homo sapiens GN=PLK3 PE=1 SV=2//0
XM_007979043.1	1.6022	2.88E-05	0.000201	sp|Q5RE82|DPH2_PONAB Diphthamide biosynthesis protein 2 OS=Pongo abelii GN=DPH2 PE=2 SV=1//0
XM_007979050.1	0.89974	0.0023803	0.012719	sp|Q6P179|ERAP2_HUMAN Endoplasmic reticulum aminopeptidase 2 OS=Homo sapiens GN=ERAP2 PE=1 SV=2//0
XM_007979092.1	0.6948	6.18E-08	5.64E-07	sp|Q96G25|MED8_HUMAN Mediator of RNA polymerase II transcription subunit 8 OS=Homo sapiens GN=MED8 PE=1 SV=2//4.97261e-162
XM_007979100.1	-0.37364	2.62E-05	0.0001837	sp|Q12834|CDC20_HUMAN Cell division cycle protein 20 homolog OS=Homo sapiens GN=CDC20 PE=1 SV=2//0
XM_007979143.1	-0.62286	5.22E-06	3.99E-05	sp|Q32P28|P3H1_HUMAN Prolyl 3-hydroxylase 1 OS=Homo sapiens GN=LEPRE1 PE=1 SV=2//0
XM_007979144.1	1.5874	1.33E-14	1.97E-13	sp|Q9BV19|CA050_HUMAN Uncharacterized protein C1orf50 OS=Homo sapiens GN=C1orf50 PE=1 SV=2//3.31945e-130
XM_007979182.1	-2.0424	0.0004359	0.0026239	sp|Q8IXN7|RIMKA_HUMAN N-acetylaspartylglutamate synthase A OS=Homo sapiens GN=RIMKLA PE=2 SV=2//0
XM_007979207.1	1.6645	1.49E-24	3.68E-23	sp|P20800|EDN2_HUMAN Endothelin-2 OS=Homo sapiens GN=EDN2 PE=1 SV=2//4.68184e-105
XM_007979209.1	0.63686	0.0089768	0.042478	sp|Q70KY4|FOXO6_MOUSE Forkhead box protein O6 OS=Mus musculus GN=Foxo6 PE=1 SV=1//5.35517e-18
XM_007979228.1	3.6544	1.13E-10	1.29E-09	sp|P17812|PYRG1_HUMAN CTP synthase 1 OS=Homo sapiens GN=CTPS1 PE=1 SV=2//0
XM_007979229.1	4.1159	9.68E-08	8.66E-07	sp|P17812|PYRG1_HUMAN CTP synthase 1 OS=Homo sapiens GN=CTPS1 PE=1 SV=2//0
XM_007979244.1	-0.25519	0.0008743	0.0050351	sp|Q9UIQ6|LCAP_HUMAN Leucyl-cystinyl aminopeptidase OS=Homo sapiens GN=LNPEP PE=1 SV=3//0
XM_007979249.1	-0.5807	0.0008311	0.0048028	sp|Q9UJD0|RIMS3_HUMAN Regulating synaptic membrane exocytosis protein 3 OS=Homo sapiens GN=RIMS3 PE=1 SV=1//1.4617e-173
XM_007979281.1	-0.88076	3.64E-25	9.39E-24	sp|Q8HXW6|PPT1_MACFA Palmitoyl-protein thioesterase 1 OS=Macaca fascicularis GN=PPT1 PE=2 SV=1//0
XM_007979289.1	2.0985	3.21E-27	8.92E-26	sp|Q8NA29|NLS1_HUMAN Sodium-dependent lysophosphatidylcholine symporter

				1 OS=Homo sapiens GN=MFSD2A PE=1 SV=1//0
XM_007979292.1	1.2391	2.32E-21	4.94E-20	sp|Q9H3H1|MOD5_HUMAN tRNA dimethylallyltransferase, mitochondrial OS=Homo sapiens GN=TRIT1 PE=1 SV=1//0
XM_007979295.1	1.4138	3.59E-11	4.28E-10	sp|Q9BVS4|RIOK2_HUMAN Serine/threonine-protein kinase RIO2 OS=Homo sapiens GN=RIOK2 PE=1 SV=2//0
XM_007979301.1	-0.64573	0.0002421	0.0015037	sp|Q9NQ87|HEYL_HUMAN Hairy/enhancer-of-split related with YRPW motif-like protein OS=Homo sapiens GN=HEYL PE=1 SV=2//3.67632e-151
XM_007979302.1	-2.5858	4.05E-06	3.13E-05	sp|Q9BXI3|5NT1A_HUMAN Cytosolic 5'-nucleotidase 1A OS=Homo sapiens GN=NT5C1A PE=2 SV=1//0
XM_007979310.1	-0.5749	3.23E-12	4.13E-11	sp|O94854|K0754_HUMAN Uncharacterized protein KIAA0754 OS=Homo sapiens GN=KIAA0754 PE=1 SV=4//9.88378e-06
XM_007979312.1	0.86552	8.60E-22	1.88E-20	sp|Q9HB90|RRAGC_HUMAN Ras-related GTP-binding protein C OS=Homo sapiens GN=RRAGC PE=1 SV=1//0
XM_007979313.1	-0.7859	6.92E-09	6.83E-08	sp|Q99417|MYCBP_HUMAN C-Myc-binding protein OS=Homo sapiens GN=MYCBP PE=1 SV=3//9.93098e-55
XM_007979314.1	-2.438	0.003924	0.02003	sp|P57773|CXA9_HUMAN Gap junction alpha-9 protein OS=Homo sapiens GN=GJA9 PE=2 SV=2//0
XM_007979320.1	1.1798	2.88E-10	3.20E-09	sp|Q9Y3A2|UTP11_HUMAN Probable U3 small nucleolar RNA-associated protein 11 OS=Homo sapiens GN=UTP11L PE=1 SV=2//5.64567e-166
XM_007979334.1	1.1584	4.15E-13	5.58E-12	sp|Q14872|MTF1_HUMAN Metal regulatory transcription factor 1 OS=Homo sapiens GN=MTF1 PE=1 SV=2//0
XM_007979361.1	1.0432	2.69E-24	6.55E-23	sp|Q5RBS5|BOREA_PONAB Borealin OS=Pongo abelii GN=CDCA8 PE=2 SV=1//0
XM_007979369.1	0.95894	4.06E-26	1.08E-24	sp|Q13823|NOG2_HUMAN Nucleolar GTP-binding protein 2 OS=Homo sapiens GN=GNL2 PE=1 SV=1//0
XM_007979370.1	1.3446	3.43E-07	2.93E-06	sp|Q8TAD8|SNIP1_HUMAN Smad nuclear-interacting protein 1 OS=Homo sapiens GN=SNIP1 PE=1 SV=1//3.86142e-153
XM_007979382.1	-0.57845	7.40E-11	8.56E-10	sp|P82914|RT15_HUMAN 28S ribosomal protein S15, mitochondrial OS=Homo sapiens GN=MRPS15 PE=1 SV=1//2.53259e-118
XM_007979390.1	-1.5129	3.48E-37	1.37E-35	sp|Q9NVM1|EVA1B_HUMAN Protein eva-1 homolog B OS=Homo sapiens GN=EVA1B PE=1 SV=1//6.31906e-49
XM_007979427.1	2.1261	0.0016809	0.0092552	sp|Q9H9G7|AGO3_HUMAN Protein argonaute-3 OS=Homo sapiens GN=AGO3 PE=1 SV=2//0
XM_007979428.1	-4.3012	0.0016044	0.0088653	sp|Q8CJG1|AGO1_MOUSE Protein argonaute-1 OS=Mus musculus GN=Ago1 PE=1 SV=2//0
XM_007979435.1	0.79281	0.010531	0.049059	sp|Q86Z20|CC125_HUMAN Coiled-coil domain-containing protein 125 OS=Homo sapiens GN=CCDC125 PE=1 SV=2//0
XM_007979483.1	5.9613	6.37E-11	7.42E-10	sp|Q6P1W5|CA094_HUMAN Uncharacterized protein C1orf94 OS=Homo sapiens GN=C1orf94 PE=1 SV=2//0
XM_007979502.1	1.3671	0.0010931	0.0062123	sp|P17040|ZSC20_HUMAN Zinc finger and SCAN domain-containing protein 20 OS=Homo sapiens GN=ZSCAN20 PE=2 SV=3//0
XM_007979540.1	1.7255	8.99E-103	1.76E-100	sp|P54577|SYYC_HUMAN Tyrosine--tRNA ligase, cytoplasmic OS=Homo sapiens GN=YARS PE=1 SV=4//0
XM_007979554.1	-2.5594	0.0001065	0.0006964	sp|Q9P206|K1522_HUMAN Uncharacterized protein KIAA1522 OS=Homo sapiens GN=KIAA1522 PE=1 SV=2//0
XM_007979564.1	-0.66936	2.55E-07	2.19E-06	sp|Q8IWT0|ARCH_HUMAN Protein archease OS=Homo sapiens GN=ZBTB8OS PE=1 SV=2//6.24004e-118
XM_007979602.1	-0.49124	2.52E-09	2.58E-08	sp|Q13347|EIF3I_HUMAN Eukaryotic translation initiation factor 3 subunit I OS=Homo sapiens GN=EIF3I PE=1 SV=1//0
XM_007979612.1	-0.47647	4.68E-11	5.52E-10	sp|Q07666|KHDR1_HUMAN KH domain-containing, RNA-binding, signal transduction-associated protein 1 OS=Homo sapiens GN=KHDRBS1 PE=1 SV=1//5.69318e-175
XM_007979615.1	-1.5404	4.40E-30	1.37E-28	sp|Q9BV90|SNR25_HUMAN U11/U12 small nuclear ribonucleoprotein 25 kDa protein OS=Homo sapiens GN=SNRNP25 PE=1 SV=1//3.3363e-86
XM_007979663.1	-1.2359	1.41E-14	2.08E-13	sp|P05413|FABPH_HUMAN Fatty acid-binding protein, heart OS=Homo sapiens GN=FABP3 PE=1 SV=4//2.11854e-88
XM_007979667.1	0.36884	0.0003368	0.0020564	sp|Q96DI7|SNR40_HUMAN U5 small nuclear ribonucleoprotein 40 kDa protein OS=Homo sapiens GN=SNRNP40 PE=1 SV=1//0
XM_007979715.1	1.1015	7.28E-05	0.0004864	sp|Q9BV79|MECR_HUMAN Trans-2-enoyl-CoA reductase, mitochondrial OS=Homo sapiens GN=MECR PE=1 SV=2//7.23654e-23
XM_007979717.1	0.73329	1.63E-20	3.31E-19	sp|Q8VE97|SRSF4_MOUSE Serine/arginine-rich splicing factor 4 OS=Mus musculus GN=Srsf4 PE=2 SV=1//4.40387e-92
XM_007979750.1	1.2316	1.48E-48	8.72E-47	sp|Q9Y5A9|YTHD2_HUMAN YTH domain-containing family protein 2 OS=Homo sapiens GN=YTHDF2 PE=1 SV=2//0
XM_007979760.1	-0.65612	1.24E-16	2.07E-15	sp|Q9H0X4|ITFG3_HUMAN Protein ITFG3 OS=Homo sapiens GN=ITFG3 PE=1 SV=1//0
XM_007979784.1	2.1059	1.49E-78	1.83E-76	sp|P58004|SESN2_HUMAN Sestrin-2 OS=Homo sapiens GN=SESN2 PE=1 SV=1//0
XM_007979785.1	-1.3117	6.97E-10	7.46E-09	sp|Q5RFJ9|ATIF1_PONAB ATPase inhibitor, mitochondrial OS=Pongo abelii GN=ATPIF1 PE=3 SV=1//3.23986e-46
XM_007979803.1	1.7046	3.20E-56	2.25E-54	sp|P15927|RFA2_HUMAN Replication protein A 32 kDa subunit OS=Homo sapiens GN=RPA2 PE=1 SV=1//5.38929e-177

XM_007979809.1	0.79991	5.51E-11	6.45E-10	sp|Q86Y82|STX12_HUMAN Syntaxin-12 OS=Homo sapiens GN=STX12 PE=1 SV=1//9.77769e-150
XM_007979812.1	4.0728	1.81E-45	9.47E-44	sp|Q28808|IFI6_PANTR Interferon alpha-inducible protein 6 OS=Pan troglodytes GN=IFI6 PE=2 SV=1//1.49884e-54
XM_007979819.1	-1.7734	5.19E-05	0.0003519	sp|Q9NQ29|LUC7L_HUMAN Putative RNA-binding protein Luc7-like 1 OS=Homo sapiens GN=LUC7L PE=1 SV=1//2.7248e-10
XM_007979820.1	-0.256	0.008996	0.042551	sp|Q5TGY3|AHDC1_HUMAN AT-hook DNA-binding motif-containing protein 1 OS=Homo sapiens GN=AHDC1 PE=1 SV=1//0
XM_007979846.1	-0.5479	0.0033744	0.017482	sp|Q8IYJ3|SYTL1_HUMAN Synaptotagmin-like protein 1 OS=Homo sapiens GN=SYTL1 PE=1 SV=1//0
XM_007979853.1	2.164	6.79E-70	6.89E-68	sp|Q96A09|FA46B_HUMAN Protein FAM46B OS=Homo sapiens GN=FAM46B PE=1 SV=2//0
XM_007979863.1	0.39797	0.0047398	0.023797	sp|Q4R579|GPN2_MACFA GPN-loop GTPase 2 OS=Macaca fascicularis GN=GPN2 PE=2 SV=1//0
XM_007979866.1	-1.6668	0.0001052	0.0006887	sp|P31947|1433S_HUMAN 14-3-3 protein sigma OS=Homo sapiens GN=SFN PE=1 SV=1//2.20333e-164
XM_007979880.1	-4.1192	0.0044104	0.022306	sp|Q9NUE0|ZDH18_HUMAN Palmitoyltransferase ZDHHC18 OS=Homo sapiens GN=ZDHHC18 PE=2 SV=2//1.42629e-180
XM_007979902.1	1.279	1.87E-12	2.43E-11	sp|O00488|ZN593_HUMAN Zinc finger protein 593 OS=Homo sapiens GN=ZNF593 PE=1 SV=2//1.1837e-73
XM_007979921.1	-1.7741	3.35E-15	5.09E-14	sp|Q9H299|SH3L3_HUMAN SH3 domain-binding glutamic acid-rich-like protein 3 OS=Homo sapiens GN=SH3BGRL3 PE=1 SV=1//7.02492e-57
XM_007979940.1	-0.65743	2.41E-19	4.65E-18	sp|Q86SG4|DPCA2_HUMAN Putative Dresden prostate carcinoma protein 2 OS=Homo sapiens GN=HMGN2P46 PE=5 SV=1//2.33391e-26
XM_007979946.1	-0.63307	0.0001515	0.000969	sp|P29372|3MG_HUMAN DNA-3-methyladenine glycosylase OS=Homo sapiens GN=MPG PE=1 SV=3//6.10792e-178
XM_007979974.1	-1.8017	8.98E-48	5.13E-46	sp|Q9NZV5|SELN_HUMAN Selenoprotein N OS=Homo sapiens GN=SEPN1 PE=1 SV=5//0
XM_007979976.1	-1.8108	0.0001144	0.0007448	sp|Q9NR34|MA1C1_HUMAN Mannosyl-oligosaccharide 1,2-alpha-mannosidase IC OS=Homo sapiens GN=MAN1C1 PE=1 SV=1//0
XM_007979979.1	-2.7084	1.88E-37	7.54E-36	sp|Q4R626|RSRP1_MACFA Arginine/serine-rich protein 1 OS=Macaca fascicularis GN=RSRP1 PE=2 SV=1//1.38076e-68
XM_007980026.1	-0.68595	0.0006747	0.0039459	sp|Q5RD30|NPAL3_PONAB NIPA-like protein 3 OS=Pongo abelii GN=NIPAL3 PE=2 SV=1//0
XM_007980049.1	1.5979	2.23E-07	1.92E-06	sp|Q9R0U0|SRS10_MOUSE Serine/arginine-rich splicing factor 10 OS=Mus musculus GN=Srsf10 PE=1 SV=2//3.43239e-36
XM_007980051.1	2.1063	2.81E-128	7.42E-126	sp|Q9NPJ4|PNRC2_HUMAN Proline-rich nuclear receptor coactivator 2 OS=Homo sapiens GN=PNRC2 PE=1 SV=1//2.93317e-75
XM_007980070.1	0.69053	3.02E-08	2.83E-07	sp|Q14241|ELOA1_HUMAN Transcription elongation factor B polypeptide 3 OS=Homo sapiens GN=TCEB3 PE=1 SV=2//0
XM_007980071.1	1.8395	6.65E-28	1.89E-26	sp|Q9GZP4|PITH1_HUMAN PITH domain-containing protein 1 OS=Homo sapiens GN=PITHD1 PE=1 SV=1//1.47958e-143
XM_007980073.1	-0.54498	1.51E-07	1.33E-06	sp|P62914|RL11_RAT 60S ribosomal protein L11 OS=Rattus norvegicus GN=Rpl11 PE=1 SV=2//1.86101e-128
XM_007980074.1	1.8133	3.87E-87	5.82E-85	sp|Q02535|ID3_HUMAN DNA-binding protein inhibitor ID-3 OS=Homo sapiens GN=ID3 PE=1 SV=2//2.03109e-65
XM_007980075.1	0.73266	0.0017003	0.0093524	sp|Q14209|E2F2_HUMAN Transcription factor E2F2 OS=Homo sapiens GN=E2F2 PE=1 SV=1//0
XM_007980092.1	0.94957	5.30E-05	0.0003585	sp|P28221|5HT1D_HUMAN 5-hydroxytryptamine receptor 1D OS=Homo sapiens GN=HTR1D PE=1 SV=1//0
XM_007980093.1	-1.4077	2.23E-06	1.76E-05	sp|O00746|NDKM_HUMAN Nucleoside diphosphate kinase, mitochondrial OS=Homo sapiens GN=NME4 PE=1 SV=1//8.27531e-124
XM_007980094.1	1.3861	8.45E-67	8.08E-65	sp|Q86V48|LUZP1_HUMAN Leucine zipper protein 1 OS=Homo sapiens GN=LUZP1 PE=1 SV=2//0
XM_007980113.1	1.1196	1.10E-13	1.54E-12	sp|Q9NUA8|ZBT40_HUMAN Zinc finger and BTB domain-containing protein 40 OS=Homo sapiens GN=ZBTB40 PE=1 SV=4//0
XM_007980206.1	-0.96158	6.79E-12	8.51E-11	sp|Q9BXM7|PINK1_HUMAN Serine/threonine-protein kinase PINK1, mitochondrial OS=Homo sapiens GN=PINK1 PE=1 SV=1//0
XM_007980209.1	1.3584	3.39E-22	7.57E-21	sp|Q969V5|MUL1_HUMAN Mitochondrial ubiquitin ligase activator of NFKB 1 OS=Homo sapiens GN=MUL1 PE=1 SV=1//0
XM_007980211.1	-1.5839	1.14E-05	8.33E-05	sp|Q6ZT52|FA43B_HUMAN Protein FAM43B OS=Homo sapiens GN=FAM43B PE=2 SV=1//9.14206e-163
XM_007980213.1	-1.841	5.45E-39	2.31E-37	sp|Q7Z7J9|CK2N1_HUMAN Calcium/calmodulin-dependent protein kinase II inhibitor 1 OS=Homo sapiens GN=CAMK2N1 PE=1 SV=1//3.26965e-35
XM_007980235.1	0.72702	0.0044566	0.022511	sp|Q5T2D3|OTUD3_HUMAN OTU domain-containing protein 3 OS=Homo sapiens GN=OTUD3 PE=1 SV=1//0
XM_007980258.1	#NAME?	0.0003012	0.0018466	sp|Q96S21|RB40C_HUMAN Ras-related protein Rab-40C OS=Homo sapiens GN=RAB40C PE=1 SV=1//2.60956e-180
XM_007980270.1	2.9358	1.16E-05	8.53E-05	sp|Q8NHP1|ARK74_HUMAN Aflatoxin B1 aldehyde reductase member 4 OS=Homo sapiens GN=AKR7L PE=2 SV=6//0
XM_007980272.1	1.3541	6.09E-24	1.47E-22	sp|Q9UKD2|MRT4_HUMAN mRNA turnover protein 4 homolog OS=Homo sapiens

				GN=MRTO4 PE=1 SV=2//1.08996e-149
XM_007980283.1	-0.98895	1.71E-05	0.0001225	sp|P30038|AL4A1_HUMAN Delta-1-pyrroline-5-carboxylate dehydrogenase, mitochondrial OS=Homo sapiens GN=ALDH4A1 PE=1 SV=3//0
XM_007980292.1	0.39134	0.0093112	0.043909	sp|Q9QYT7|PIGQ_MOUSE Phosphatidylinositol N-acetylglucosaminyltransferase subunit Q OS=Mus musculus GN=Pigq PE=2 SV=3//0
XM_007980323.1	-0.75282	5.06E-10	5.50E-09	sp|P21912|SDHB_HUMAN Succinate dehydrogenase [ubiquinone] iron-sulfur subunit, mitochondrial OS=Homo sapiens GN=SDHB PE=1 SV=3//0
XM_007980358.1	-0.87989	7.07E-20	1.39E-18	sp|Q14919|NC2A_HUMAN Dr1-associated corepressor OS=Homo sapiens GN=DRAP1 PE=1 SV=3//3.11204e-72
XM_007980431.1	2.1313	9.59E-22	2.08E-20	sp|Q92681|RSCA1_HUMAN Regulatory solute carrier protein family 1 member 1 OS=Homo sapiens GN=RSC1A1 PE=2 SV=1//0
XM_007980444.1	1.0094	2.77E-29	8.29E-28	sp|Q96C19|EFHD2_HUMAN EF-hand domain-containing protein D2 OS=Homo sapiens GN=EFHD2 PE=1 SV=1//3.73486e-113
XM_007980498.1	-0.40732	0.0053192	0.026408	sp|O75911|DHRS3_HUMAN Short-chain dehydrogenase/reductase 3 OS=Homo sapiens GN=DHRS3 PE=1 SV=2//0
XM_007980528.1	5.5269	2.51E-60	1.97E-58	sp|P16860|ANFB_HUMAN Natriuretic peptides B OS=Homo sapiens GN=NPPB PE=1 SV=1//6.36805e-71
XM_007980533.1	0.93987	1.93E-12	2.50E-11	sp|P51797|CLCN6_HUMAN Chloride transport protein 6 OS=Homo sapiens GN=CLCN6 PE=1 SV=2//0
XM_007980558.1	-1.8107	1.18E-10	1.35E-09	sp|Q9UK22|FBX2_HUMAN F-box only protein 2 OS=Homo sapiens GN=FBXO2 PE=1 SV=2//7.57174e-155
XM_007980567.1	1.1657	1.90E-13	2.62E-12	sp|Q8CFJ9|WDR24_MOUSE WD repeat-containing protein 24 OS=Mus musculus GN=Wdr24 PE=2 SV=1//0
XM_007980571.1	1.3122	2.02E-06	1.61E-05	sp|Q9Y5Z9|UBIA1_HUMAN UbiA prenyltransferase domain-containing protein 1 OS=Homo sapiens GN=UBIAD1 PE=1 SV=1//0
XM_007980572.1	0.52241	2.96E-06	2.32E-05	sp|Q9UGV2|NDRG3_HUMAN Protein NDRG3 OS=Homo sapiens GN=NDRG3 PE=1 SV=2//0
XM_007980573.1	0.38868	0.0011225	0.0063646	sp|Q01780|EXOSX_HUMAN Exosome component 10 OS=Homo sapiens GN=EXOSC10 PE=1 SV=2//0
XM_007980577.1	0.41499	0.0028987	0.015255	sp|P19623|SPEE_HUMAN Spermidine synthase OS=Homo sapiens GN=SRM PE=1 SV=1//0
XM_007980584.1	-1.7462	3.83E-12	4.88E-11	sp|O00187|MASP2_HUMAN Mannan-binding lectin serine protease 2 OS=Homo sapiens GN=MASP2 PE=1 SV=4//0
XM_007980585.1	-0.65724	4.33E-10	4.75E-09	sp|Q13148|TADBP_HUMAN TAR DNA-binding protein 43 OS=Homo sapiens GN=TARDBP PE=1 SV=1//0
XM_007980615.1	0.68789	5.28E-08	4.85E-07	sp|O43896|KIF1C_HUMAN Kinesin-like protein KIF1C OS=Homo sapiens GN=KIF1C PE=1 SV=3//0
XM_007980639.1	1.77	5.51E-15	8.31E-14	sp|Q96BD6|SPSB1_HUMAN SPRY domain-containing SOCS box protein 1 OS=Homo sapiens GN=SPSB1 PE=1 SV=1//0
XM_007980646.1	-2.6903	5.23E-35	1.95E-33	sp|O95479|G6PE_HUMAN GDH/6PGL endoplasmic bifunctional protein OS=Homo sapiens GN=H6PD PE=1 SV=2//0
XM_007980653.1	0.4777	0.0007179	0.0041842	sp|Q9H6Q4|NARFL_HUMAN Cytosolic Fe-S cluster assembly factor NARFL OS=Homo sapiens GN=NARFL PE=1 SV=1//0
XM_007980661.1	-1.0605	1.77E-59	1.35E-57	sp|P06733|ENOA_HUMAN Alpha-enolase OS=Homo sapiens GN=ENO1 PE=1 SV=2//0
XM_007980677.1	2.4987	2.50E-29	7.52E-28	sp|Q9UJM3|ERRFI_HUMAN ERBB receptor feedback inhibitor 1 OS=Homo sapiens GN=ERRFI1 PE=1 SV=1//0
XM_007980702.1	-0.65228	3.69E-10	4.07E-09	sp|Q15836|VAMP3_HUMAN Vesicle-associated membrane protein 3 OS=Homo sapiens GN=VAMP3 PE=1 SV=3//3.78777e-46
XM_007980733.1	0.59822	1.40E-05	0.0001018	sp|Q5RC70|DJC11_PONAB DnaJ homolog subfamily C member 11 OS=Pongo abelii GN=DNAJC11 PE=2 SV=1//0
XM_007980735.1	1.2618	5.72E-21	1.20E-19	sp|Q9UJP4|KLH21_HUMAN Kelch-like protein 21 OS=Homo sapiens GN=KLHL21 PE=1 SV=4//0
XM_007980737.1	0.7945	4.03E-09	4.06E-08	sp|Q5SY16|NOL9_HUMAN Polynucleotide 5'-hydroxyl-kinase NOL9 OS=Homo sapiens GN=NOL9 PE=1 SV=1//0
XM_007980768.1	1.2681	4.48E-25	1.15E-23	sp|Q15834|CC85B_HUMAN Coiled-coil domain-containing protein 85B OS=Homo sapiens GN=CCDC85B PE=1 SV=2//7.57028e-48
XM_007980769.1	-3.652	0.0004786	0.0028614	sp|Q13421|MSLN_HUMAN Mesothelin OS=Homo sapiens GN=MSLN PE=1 SV=2//0
XM_007980773.1	0.87285	1.70E-06	1.36E-05	sp|Q9Y543|HES2_HUMAN Transcription factor HES-2 OS=Homo sapiens GN=HES2 PE=2 SV=1//4.23579e-78
XM_007980781.1	-2.2719	1.30E-96	2.28E-94	sp|Q6NV75|GP153_HUMAN Probable G-protein coupled receptor 153 OS=Homo sapiens GN=GPR153 PE=2 SV=2//0
XM_007980792.1	-0.44839	3.75E-05	0.0002575	sp|O60725|ICMT_HUMAN Protein-S-isoprenylcysteine O-methyltransferase OS=Homo sapiens GN=ICMT PE=1 SV=1//8.35101e-163
XM_007980797.1	-0.8236	2.23E-10	2.50E-09	sp|Q6ZRF8|RN207_HUMAN RING finger protein 207 OS=Homo sapiens GN=RNF207 PE=2 SV=2//0
XM_007980865.1	1.2198	9.59E-08	8.59E-07	sp|Q9UF72|T73AS_HUMAN Putative TP73 antisense gene protein 1 OS=Homo sapiens GN=TP73-AS1 PE=5 SV=1//3.9173e-76
XM_007980866.1	-0.81957	1.46E-05	0.0001052	sp|Q9P2S5|WRP73_HUMAN WD repeat-containing protein WRAP73 OS=Homo sapiens GN=WRAP73 PE=2 SV=1//0

XM_007980871.1	-1.2641	1.02E-05	7.55E-05	sp|O75095|MEGF6_HUMAN Multiple epidermal growth factor-like domains protein 6 OS=Homo sapiens GN=MEGF6 PE=1 SV=4//0
XM_007980886.1	-0.61893	9.69E-13	1.28E-11	sp|O46431|FIBP_CHLAE Acidic fibroblast growth factor intracellular-binding protein OS=Chlorocebus aethiops GN=FIBP PE=2 SV=2//0
XM_007980931.1	#NAME?	0.0065591	0.031913	-//-
XM_007980987.1	-0.30185	0.0060103	0.029498	sp|Q9NP77|SSU72_HUMAN RNA polymerase II subunit A C-terminal domain phosphatase SSU72 OS=Homo sapiens GN=SSU72 PE=1 SV=1//2.00124e-140
XM_007980991.1	-1.7902	1.33E-47	7.51E-46	sp|Q6PCB0|VWA1_HUMAN von Willebrand factor A domain-containing protein 1 OS=Homo sapiens GN=VWA1 PE=2 SV=1//0
XM_007980992.1	-1.9888	0.0050407	0.025151	sp|A6NKF7|TM88B_HUMAN Transmembrane protein 88B OS=Homo sapiens GN=TMEM88B PE=3 SV=1//8.71983e-16
XM_007981002.1	-0.64335	0.0008955	0.0051477	sp|Q96S94|CCNL2_HUMAN Cyclin-L2 OS=Homo sapiens GN=CCNL2 PE=1 SV=1//1.24115e-128
XM_007981053.1	1.0202	5.49E-13	7.34E-12	sp|Q96L58|B3GT6_HUMAN Beta-1,3-galactosyltransferase 6 OS=Homo sapiens GN=B3GALT6 PE=1 SV=2//8.13911e-152
XM_007981065.1	#NAME?	0.0009618	0.0055046	sp|Q4R7H0|TTL10_MACFA Protein polyglycylase TTLL10 OS=Macaca fascicularis GN=TTLL10 PE=2 SV=1//0
XM_007981089.1	4.1304	3.85E-39	1.64E-37	sp|P05161|ISG15_HUMAN Ubiquitin-like protein ISG15 OS=Homo sapiens GN=ISG15 PE=1 SV=5//9.73105e-88
XM_007981094.1	-1.4868	4.46E-51	2.80E-49	sp|Q96NU1|SAM11_HUMAN Sterile alpha motif domain-containing protein 11 OS=Homo sapiens GN=SAMD11 PE=2 SV=3//0
XM_007981106.1	-3.4176	8.87E-06	6.59E-05	-//-
XM_007981107.1	1.3773	1.26E-20	2.58E-19	sp|Q4R681|MSTO1_MACFA Protein misato homolog 1 OS=Macaca fascicularis GN=MSTO1 PE=2 SV=2//0
XM_007981120.1	-3.4379	0.0025902	0.013739	sp|Q5VU13|VSIG8_HUMAN V-set and immunoglobulin domain-containing protein 8 OS=Homo sapiens GN=VSIG8 PE=2 SV=1//0
XM_007981124.1	-0.3089	0.0001877	0.0011847	sp|Q9UMX0|UBQL1_HUMAN Ubiquilin-1 OS=Homo sapiens GN=UBQLN1 PE=1 SV=2//1.09537e-27
XM_007981125.1	-0.29584	0.0015896	0.0087923	sp|P49760|CLK2_HUMAN Dual specificity protein kinase CLK2 OS=Homo sapiens GN=CLK2 PE=1 SV=1//0
XM_007981136.1	1.133	1.25E-34	4.58E-33	sp|Q4R4U9|SYSC_MACFA Serine--tRNA ligase, cytoplasmic OS=Macaca fascicularis GN=SARS PE=2 SV=3//6.7033e-22
XM_007981137.1	2.4283	1.11E-28	3.25E-27	sp|Q6UXG2|K1324_HUMAN UPF0577 protein KIAA1324 OS=Homo sapiens GN=KIAA1324 PE=2 SV=2//0
XM_007981144.1	-0.71737	0.0018428	0.010048	sp|P70187|HIAT1_MOUSE Hippocampus abundant transcript 1 protein OS=Mus musculus GN=Hiat1 PE=2 SV=3//3.06517e-16
XM_007981145.1	0.37409	0.0018644	0.010158	sp|Q6ZNA5|FRRS1_HUMAN Ferric-chelate reductase 1 OS=Homo sapiens GN=FRRS1 PE=2 SV=2//3.18014e-21
XM_007981150.1	-2.3671	0.0024948	0.013292	-//-
XM_007981156.1	-0.30661	0.00045	0.0027031	sp|Q8NI35|INADL_HUMAN InaD-like protein OS=Homo sapiens GN=INADL PE=1 SV=3//0
XM_007981161.1	0.54444	3.53E-06	2.74E-05	sp|Q15468|STIL_HUMAN SCL-interrupting locus protein OS=Homo sapiens GN=STIL PE=1 SV=2//0
XM_007981173.1	1.0115	1.48E-05	0.000107	sp|Q8TAD8|SNIP1_HUMAN Smad nuclear-interacting protein 1 OS=Homo sapiens GN=SNIP1 PE=1 SV=1//0
XM_007981174.1	1.3277	5.21E-34	1.87E-32	sp|O95789|ZMYM6_HUMAN Zinc finger MYM-type protein 6 OS=Homo sapiens GN=ZMYM6 PE=2 SV=2//0
XM_007981176.1	1.6794	1.92E-39	8.22E-38	sp|Q6ZMZ0|RN19B_HUMAN E3 ubiquitin-protein ligase RNF19B OS=Homo sapiens GN=RNF19B PE=1 SV=2//4.75993e-13
XM_007981177.1	-1.966	4.16E-82	5.53E-80	-//-
XM_007981182.1	0.79164	0.0006296	0.003707	sp|Q96I76|GPTC3_HUMAN G patch domain-containing protein 3 OS=Homo sapiens GN=GPATCH3 PE=2 SV=1//6.32097e-161
XM_007981183.1	0.91462	4.53E-12	5.75E-11	sp|Q86SQ9|DHDDS_HUMAN Dehydrodolichyl diphosphate syntase complex subunit DHDDS OS=Homo sapiens GN=DHDDS PE=1 SV=3//3.48949e-139
XM_007981188.1	-1.0753	8.87E-16	1.40E-14	sp|Q60HF8|FUCO_MACFA Tissue alpha-L-fucosidase OS=Macaca fascicularis GN=FUCA1 PE=2 SV=1//2.84271e-85
XM_007981198.1	1.7187	3.51E-13	4.76E-12	sp|O95154|ARK73_HUMAN Aflatoxin B1 aldehyde reductase member 3 OS=Homo sapiens GN=AKR7A3 PE=1 SV=2//0
XM_007981205.1	-0.85512	5.42E-09	5.39E-08	sp|Q5R9N3|PLOD1_PONAB Procollagen-lysine,2-oxoglutarate 5-dioxygenase 1 OS=Pongo abelii GN=PLOD1 PE=2 SV=1//1.16006e-25
XM_007981206.1	-0.63637	4.80E-13	6.44E-12	sp|P52209|6PGD_HUMAN 6-phosphogluconate dehydrogenase, decarboxylating OS=Homo sapiens GN=PGD PE=1 SV=3//0
XM_007981208.1	1.08	0.0044588	0.022511	sp|Q9HAN9|NMNA1_HUMAN Nicotinamide/nicotinic acid mononucleotide adenylyltransferase 1 OS=Homo sapiens GN=NMNAT1 PE=1 SV=1//3.98731e-132
XM_007981210.1	-0.55191	7.04E-11	8.16E-10	sp|Q28CQ4|UBC9_XENTR SUMO-conjugating enzyme UBC9 OS=Xenopus tropicalis GN=ube2i PE=2 SV=1//3.20078e-105
XM_007981214.1	0.83155	2.39E-11	2.89E-10	sp|Q5T9A4|ATD3B_HUMAN ATPase family AAA domain-containing protein 3B OS=Homo sapiens GN=ATAD3B PE=1 SV=1//0
XM_007981244.1	2.9777	0.0006397	0.0037617	sp|Q5R5N6|BGLR_PONAB Beta-glucuronidase OS=Pongo abelii GN=GUSB PE=2 SV=2//5.90385e-33

XM_007981250.1	-0.44972	8.81E-06	6.55E-05	sp|Q9Y6H1|CHCH2_HUMAN Coiled-coil-helix-coiled-coil-helix domain-containing protein 2 OS=Homo sapiens GN=CHCHD2 PE=1 SV=1//6.99256e-38
XM_007981258.1	0.27814	0.0011171	0.006338	sp|P40227|TCPZ_HUMAN T-complex protein 1 subunit zeta OS=Homo sapiens GN=CCT6A PE=1 SV=3//0
XM_007981280.1	3.543	1.68E-201	8.58E-199	sp|P55245|EGFR_MACMU Epidermal growth factor receptor OS=Macaca mulatta GN=EGFR PE=2 SV=2//0
XM_007981388.1	0.57258	5.62E-12	7.09E-11	sp|P17936|IBP3_HUMAN Insulin-like growth factor-binding protein 3 OS=Homo sapiens GN=IGFBP3 PE=1 SV=2//2.31542e-170
XM_007981413.1	0.3186	0.0009206	0.0052828	sp|Q68A21|PURB_RAT Transcriptional activator protein Pur-beta OS=Rattus norvegicus GN=Purb PE=1 SV=3//3.21382e-125
XM_007981498.1	0.79931	3.90E-13	5.25E-12	sp|Q96EL2|RT24_HUMAN 28S ribosomal protein S24, mitochondrial OS=Homo sapiens GN=MRPS24 PE=1 SV=1//4.19765e-111
XM_007981526.1	-0.51061	6.00E-07	5.01E-06	sp|P25787|PSA2_HUMAN Proteasome subunit alpha type-2 OS=Homo sapiens GN=PSMA2 PE=1 SV=2//6.93194e-173
XM_007981538.1	6.0604	6.44E-159	2.39E-156	sp|P08476|INHBA_HUMAN Inhibin beta A chain OS=Homo sapiens GN=INHBA PE=1 SV=2//0
XM_007981551.1	0.64622	6.12E-09	6.07E-08	sp|P11233|RALA_HUMAN Ras-related protein Ral-A OS=Homo sapiens GN=RALA PE=1 SV=1//9.74395e-127
XM_007981552.1	1.0829	0.0001057	0.0006915	sp|Q9NRH1|YAED1_HUMAN Yae1 domain-containing protein 1 OS=Homo sapiens GN=YAE1D1 PE=2 SV=1//4.24609e-47
XM_007981553.1	-0.89175	5.86E-12	7.39E-11	sp|P02794|FRIH_HUMAN Ferritin heavy chain OS=Homo sapiens GN=FTH1 PE=1 SV=2//6.13994e-125
XM_007981572.1	-0.70161	4.12E-13	5.54E-12	sp|Q9N0C7|EPDR1_MACFA Mammalian ependymin-related protein 1 OS=Macaca fascicularis GN=EPDR1 PE=2 SV=3//2.49682e-157
XM_007981573.1	2.4161	5.76E-06	4.37E-05	sp|Q7YRN1|SFRP4_MACMU Secreted frizzled-related protein 4 OS=Macaca mulatta GN=SFRP4 PE=2 SV=1//0
XM_007981618.1	1.754	1.31E-29	3.97E-28	sp|Q7L9B9|EEPD1_HUMAN Endonuclease/exonuclease/phosphatase family domain-containing protein 1 OS=Homo sapiens GN=EEPD1 PE=1 SV=2//0
XM_007981653.1	0.67379	0.0019311	0.010488	sp|Q8TA86|RP9_HUMAN Retinitis pigmentosa 9 protein OS=Homo sapiens GN=RP9 PE=1 SV=2//8.26701e-90
XM_007981654.1	-2.0858	2.49E-23	5.86E-22	sp|O95302|FKBP9_HUMAN Peptidyl-prolyl cis-trans isomerase FKBP9 OS=Homo sapiens GN=FKBP9 PE=1 SV=2//0
XM_007981657.1	1.314	5.31E-34	1.90E-32	sp|Q8IY47|KBTB2_HUMAN Kelch repeat and BTB domain-containing protein 2 OS=Homo sapiens GN=KBTBD2 PE=1 SV=2//0
XM_007981701.1	-1.6664	7.12E-05	0.0004758	sp|Q9UPT6|JIP3_HUMAN C-Jun-amino-terminal kinase-interacting protein 3 OS=Homo sapiens GN=MAPK8IP3 PE=1 SV=3//0
XM_007981703.1	-1.2241	1.94E-05	0.0001378	sp|Q5R819|AQP1_PONAB Aquaporin-1 OS=Pongo abelii GN=AQP1 PE=2 SV=3//0
XM_007981707.1	-2.521	0.0008309	0.0048028	sp|Q5RF72|F188B_PONAB Protein FAM188B OS=Pongo abelii GN=FAM188B PE=2 SV=1//0
XM_007981709.1	-2.4625	1.37E-06	1.11E-05	sp|Q9UPT6|JIP3_HUMAN C-Jun-amino-terminal kinase-interacting protein 3 OS=Homo sapiens GN=MAPK8IP3 PE=1 SV=3//0
XM_007981718.1	1.4545	5.57E-77	6.67E-75	sp|Q5RBL1|SYG_PONAB Glycine--tRNA ligase OS=Pongo abelii GN=GARS PE=2 SV=1//0
XM_007981734.1	-1.6838	2.10E-17	3.65E-16	sp|Q8N3F0|MTURN_HUMAN Maturin OS=Homo sapiens GN=MTURN PE=2 SV=2//1.11228e-77
XM_007981772.1	6.4074	1.67E-15	2.59E-14	sp|Q02930|CREB5_HUMAN Cyclic AMP-responsive element-binding protein 5 OS=Homo sapiens GN=CREB5 PE=1 SV=3//1.16742e-56
XM_007981790.1	-0.87469	3.31E-21	7.00E-20	sp|P31937|3HIDH_HUMAN 3-hydroxyisobutyrate dehydrogenase, mitochondrial OS=Homo sapiens GN=HIBADH PE=1 SV=2//0
XM_007981805.1	-0.81304	3.02E-08	2.83E-07	sp|Q0VCS4|HXA2_BOVIN Homeobox protein Hox-A2 OS=Bos taurus GN=HOXA2 PE=2 SV=1//0
XM_007981807.1	-1.2198	6.70E-10	7.19E-09	sp|O43365|HXA3_HUMAN Homeobox protein Hox-A3 OS=Homo sapiens GN=HOXA3 PE=1 SV=1//2.87628e-39
XM_007981816.1	-0.44411	0.0001385	0.0008906	sp|P02830|HXA7_MOUSE Homeobox protein Hox-A7 OS=Mus musculus GN=Hoxa7 PE=2 SV=2//6.39075e-116
XM_007981817.1	-0.73893	1.86E-13	2.57E-12	sp|P31269|HXA9_HUMAN Homeobox protein Hox-A9 OS=Homo sapiens GN=HOXA9 PE=1 SV=4//9.69208e-146
XM_007981818.1	-1.4015	0.0001687	0.0010732	-//-
XM_007981819.1	-1.8611	1.86E-50	1.16E-48	sp|P31260|HXA10_HUMAN Homeobox protein Hox-A10 OS=Homo sapiens GN=HOXA10 PE=1 SV=3//2.3236e-161
XM_007981826.1	-0.86582	0.00753	0.036187	-//-
XM_007981827.1	-0.581	1.67E-06	1.34E-05	sp|P82930|RT34_HUMAN 28S ribosomal protein S34, mitochondrial OS=Homo sapiens GN=MRPS34 PE=1 SV=2//5.77405e-132
XM_007981837.1	-0.80487	7.82E-08	7.06E-07	sp|Q9Y4A8|NF2L3_HUMAN Nuclear factor erythroid 2-related factor 3 OS=Homo sapiens GN=NFE2L3 PE=1 SV=1//0
XM_007981880.1	0.82335	0.0007124	0.0041543	sp|Q96EE4|CC126_HUMAN Coiled-coil domain-containing protein 126 OS=Homo sapiens GN=CCDC126 PE=2 SV=2//6.83323e-63
XM_007981889.1	-0.20807	0.0033355	0.017309	sp|Q14956|GPNMB_HUMAN Transmembrane glycoprotein NMB OS=Homo sapiens GN=GPNMB PE=1 SV=2//0
XM_007981890.1	1.9956	2.27E-33	7.90E-32	sp|O15504|NUPL2_HUMAN Nucleoporin-like protein 2 OS=Homo sapiens

				GN=NUPL2 PE=1 SV=1//0
XM_007981900.1	-0.56581	0.0059106	0.029035	sp|A1XQS2|TOM7_PIG Mitochondrial import receptor subunit TOM7 homolog OS=Sus scrofa GN=TOMM7 PE=3 SV=1//1.16842e-31
XM_007981902.1	Inf	1.68E-05	0.0001203	sp|P79341|IL6_MACFA Interleukin-6 OS=Macaca fascicularis GN=IL6 PE=2 SV=1//1.05352e-127
XM_007981920.1	1.1098	0.0001002	0.0006577	sp|Q02446|SP4_HUMAN Transcription factor Sp4 OS=Homo sapiens GN=SP4 PE=1 SV=2//0
XM_007981926.1	0.88048	5.81E-19	1.10E-17	sp|P26012|ITB8_HUMAN Integrin beta-8 OS=Homo sapiens GN=ITGB8 PE=2 SV=1//0
XM_007981930.1	Inf	2.54E-06	2.00E-05	sp|Q6ZN28|MACC1_HUMAN Metastasis-associated in colon cancer protein 1 OS=Homo sapiens GN=MACC1 PE=1 SV=2//0
XM_007981935.1	1.2455	4.81E-25	1.23E-23	sp|Q3B726|RPA43_HUMAN DNA-directed RNA polymerase I subunit RPA43 OS=Homo sapiens GN=TWISTNB PE=1 SV=1//0
XM_007981960.1	-0.86417	4.38E-09	4.40E-08	sp|Q5R869|MSRB1_PONAB Methionine-R-sulfoxide reductase B1 OS=Pongo abelii GN=MSRB1 PE=3 SV=2//3.45901e-73
XM_007981965.1	-1.2676	3.17E-31	1.02E-29	sp|Q02373|NDUBA_BOVIN NADH dehydrogenase [ubiquinone] 1 beta subcomplex subunit 10 OS=Bos taurus GN=NDUFB10 PE=1 SV=2//1.01484e-92
XM_007981966.1	1.7144	7.12E-30	2.18E-28	sp|P35869|AHR_HUMAN Aryl hydrocarbon receptor OS=Homo sapiens GN=AHR PE=1 SV=2//0
XM_007981972.1	-0.46022	0.0055822	0.027576	sp|O95857|TSN13_HUMAN Tetraspanin-13 OS=Homo sapiens GN=TSPAN13 PE=2 SV=1//6.41929e-114
XM_007981979.1	0.52982	9.50E-05	0.0006259	sp|Q8IV38|ANKY2_HUMAN Ankyrin repeat and MYND domain-containing protein 2 OS=Homo sapiens GN=ANKMY2 PE=1 SV=1//0
XM_007981988.1	-2.8221	4.98E-05	0.0003385	sp|Q6X4U4|SOSD1_HUMAN Sclerostin domain-containing protein 1 OS=Homo sapiens GN=SOSTDC1 PE=1 SV=2//9.77051e-137
XM_007982004.1	0.58304	4.06E-08	3.75E-07	sp|Q12788|TBL3_HUMAN Transducin beta-like protein 3 OS=Homo sapiens GN=TBL3 PE=1 SV=2//0
XM_007982014.1	-0.69977	4.00E-06	3.09E-05	sp|Q9Y6U3|ADSV_HUMAN Adseverin OS=Homo sapiens GN=SCIN PE=1 SV=4//0
XM_007982044.1	-1.592	2.18E-60	1.72E-58	sp|Q3YAJ5|NDUA4_MACMU Cytochrome c oxidase subunit NDUFA4 OS=Macaca mulatta GN=NDUFA4 PE=3 SV=1//6.02684e-51
XM_007982062.1	1.3597	4.71E-09	4.71E-08	sp|P55789|ALR_HUMAN FAD-linked sulfhydryl oxidase ALR OS=Homo sapiens GN=GFER PE=1 SV=2//3.63948e-120
XM_007982083.1	1.1511	2.03E-21	4.33E-20	sp|Q9NS00|C1GLT_HUMAN Glycoprotein-N-acetylgalactosamine 3-beta-galactosyltransferase 1 OS=Homo sapiens GN=C1GALT1 PE=1 SV=1//0
XM_007982098.1	-1.1467	5.69E-07	4.76E-06	sp|P60897|DSS1_MOUSE 26S proteasome complex subunit DSS1 OS=Mus musculus GN=Shfm1 PE=3 SV=1//5.00447e-20
XM_007982099.1	1.1449	0.0024772	0.013207	sp|Q9UJS0|CMC2_HUMAN Calcium-binding mitochondrial carrier protein Aralar2 OS=Homo sapiens GN=SLC25A13 PE=1 SV=2//0
XM_007982107.1	4.1155	5.81E-10	6.28E-09	sp|O88485|DC1I1_MOUSE Cytoplasmic dynein 1 intermediate chain 1 OS=Mus musculus GN=Dync1i1 PE=1 SV=2//0
XM_007982113.1	-1.1475	5.78E-28	1.65E-26	sp|Q15165|PON2_HUMAN Serum paraoxonase/arylesterase 2 OS=Homo sapiens GN=PON2 PE=1 SV=3//0
XM_007982115.1	-2.4049	0.0002174	0.0013599	sp|Q15599|NHRF2_HUMAN Na(+)/H(+) exchange regulatory cofactor NHE-RF2 OS=Homo sapiens GN=SLC9A3R2 PE=1 SV=2//2.51066e-28
XM_007982123.1	-2.0247	4.34E-155	1.51E-152	sp|Q86TG7|PEG10_HUMAN Retrotransposon-derived protein PEG10 OS=Homo sapiens GN=PEG10 PE=1 SV=2//0
XM_007982139.1	0.53778	2.61E-08	2.47E-07	sp|O15155|BET1_HUMAN BET1 homolog OS=Homo sapiens GN=BET1 PE=1 SV=1//5.2628e-62
XM_007982141.1	-1.0282	8.44E-11	9.73E-10	sp|P61954|GBG11_RAT Guanine nucleotide-binding protein G(I)/G(S)/G(O) subunit gamma-11 OS=Rattus norvegicus GN=Gng11 PE=3 SV=1//3.67032e-42
XM_007982155.1	4.6633	2.79E-09	2.85E-08	sp|Q5K651|SAMD9_HUMAN Sterile alpha motif domain-containing protein 9 OS=Homo sapiens GN=SAMD9 PE=1 SV=1//0
XM_007982173.1	1.3797	3.66E-17	6.26E-16	sp|Q5RL73|RBM48_HUMAN RNA-binding protein 48 OS=Homo sapiens GN=RBM48 PE=2 SV=1//0
XM_007982175.1	0.42587	0.010657	0.049615	sp|Q8WUU5|GATD1_HUMAN GATA zinc finger domain-containing protein 1 OS=Homo sapiens GN=GATAD1 PE=1 SV=1//1.58699e-155
XM_007982191.1	-0.55329	2.58E-05	0.0001808	sp|Q4R8S6|CP51A_MACFA Lanosterol 14-alpha demethylase OS=Macaca fascicularis GN=CYP51A1 PE=2 SV=2//0
XM_007982216.1	-0.67714	4.81E-11	5.65E-10	sp|Q9UP38|FZD1_HUMAN Frizzled-1 OS=Homo sapiens GN=FZD1 PE=1 SV=2//0
XM_007982234.1	0.58367	0.0015704	0.0086993	sp|A4D1E9|GTPBA_HUMAN GTP-binding protein 10 OS=Homo sapiens GN=GTPBP10 PE=1 SV=1//0
XM_007982286.1	1.5558	0.0012435	0.0070152	sp|Q9UBU7|DBF4A_HUMAN Protein DBF4 homolog A OS=Homo sapiens GN=DBF4 PE=1 SV=1//0
XM_007982290.1	1.53	0.0047027	0.023647	sp|P08183|MDR1_HUMAN Multidrug resistance protein 1 OS=Homo sapiens GN=ABCB1 PE=1 SV=3//0
XM_007982309.1	0.61635	0.0018303	0.0099924	sp|Q9UKG9|OCTC_HUMAN Peroxisomal carnitine O-octanoyltransferase OS=Homo sapiens GN=CROT PE=1 SV=2//0
XM_007982331.1	-0.89885	1.01E-08	9.88E-08	sp|O95025|SEM3D_HUMAN Semaphorin-3D OS=Homo sapiens GN=SEMA3D PE=2 SV=2//0
XM_007982333.1	0.87871	2.18E-09	2.25E-08	sp|Q14563|SEM3A_HUMAN Semaphorin-3A OS=Homo sapiens GN=SEMA3A PE=1

				SV=1//0
XM_007982361.1	1.2437	5.08E-75	5.71E-73	sp|Q5RE75|SEM3C_PONAB Semaphorin-3C OS=Pongo abelii GN=SEMA3C PE=2 SV=1//0
XM_007982374.1	0.73596	0.0004755	0.0028438	sp|B2RSH2|GNAI1_MOUSE Guanine nucleotide-binding protein G(i) subunit alpha-1 OS=Mus musculus GN=Gnai1 PE=2 SV=1//0
XM_007982377.1	0.98637	0.001143	0.006479	sp|Q8N0U4|F185A_HUMAN Protein FAM185A OS=Homo sapiens GN=FAM185A PE=2 SV=3//0
XM_007982397.1	0.42369	0.002078	0.011222	sp|Q6PCB5|RSBNL_HUMAN Round spermatid basic protein 1-like protein OS=Homo sapiens GN=RSBN1L PE=1 SV=2//2.21055e-17
XM_007982459.1	0.54624	0.006239	0.030511	sp|Q6IQ20|NAPEP_HUMAN N-acyl-phosphatidylethanolamine-hydrolyzing phospholipase D OS=Homo sapiens GN=NAPEPLD PE=1 SV=2//0
XM_007982480.1	0.79668	3.48E-07	2.96E-06	sp|O43913|ORC5_HUMAN Origin recognition complex subunit 5 OS=Homo sapiens GN=ORC5 PE=1 SV=1//0
XM_007982485.1	-0.84185	2.21E-07	1.91E-06	sp|A6NDG6|PGP_HUMAN Phosphoglycolate phosphatase OS=Homo sapiens GN=PGP PE=1 SV=1//0
XM_007982502.1	0.98585	1.75E-12	2.28E-11	sp|Q96PZ0|PUS7_HUMAN Pseudouridylate synthase 7 homolog OS=Homo sapiens GN=PUS7 PE=1 SV=2//0
XM_007982513.1	1.2537	0.0040054	0.020403	sp|Q3T136|AT7L1_BOVIN Ataxin-7-like protein 1 OS=Bos taurus GN=ATXN7L1 PE=2 SV=2//6.19348e-90
XM_007982515.1	-0.39398	0.000128	0.000828	sp|Q16563|SYPL1_HUMAN Synaptophysin-like protein 1 OS=Homo sapiens GN=SYPL1 PE=1 SV=1//7.31171e-166
XM_007982516.1	0.78981	5.37E-19	1.01E-17	sp|Q52I78|NAMPT_PIG Nicotinamide phosphoribosyltransferase OS=Sus scrofa GN=NAMPT PE=2 SV=2//0
XM_007982517.1	0.91987	1.38E-07	1.22E-06	sp|Q8N9Z2|CC71L_HUMAN Coiled-coil domain-containing protein 71L OS=Homo sapiens GN=CCDC71L PE=2 SV=2//1.76959e-80
XM_007982525.1	-0.42608	0.0008541	0.004928	sp|P31323|KAP3_HUMAN cAMP-dependent protein kinase type II-beta regulatory subunit OS=Homo sapiens GN=PRKAR2B PE=1 SV=3//0
XM_007982562.1	-0.30922	0.0018339	0.010009	sp|P09622|DLDH_HUMAN Dihydrolipoyl dehydrogenase, mitochondrial OS=Homo sapiens GN=DLD PE=1 SV=2//0
XM_007982593.1	-1.2479	4.02E-18	7.24E-17	sp|P42126|ECI1_HUMAN Enoyl-CoA delta isomerase 1, mitochondrial OS=Homo sapiens GN=ECI1 PE=1 SV=1//1.81272e-179
XM_007982598.1	3.9923	7.72E-257	5.84E-254	sp|Q9QYI6|DNJB9_MOUSE DnaJ homolog subfamily B member 9 OS=Mus musculus GN=Dnajb9 PE=1 SV=2//1.37108e-133
XM_007982626.1	3.015	7.80E-44	3.81E-42	sp|O00458|IFRD1_HUMAN Interferon-related developmental regulator 1 OS=Homo sapiens GN=IFRD1 PE=1 SV=4//0
XM_007982629.1	0.48896	5.09E-05	0.0003454	sp|Q9H0V1|TM168_HUMAN Transmembrane protein 168 OS=Homo sapiens GN=TMEM168 PE=2 SV=2//0
XM_007982660.1	0.76313	2.99E-06	2.33E-05	sp|Q9P1T7|MDFIC_HUMAN MyoD family inhibitor domain-containing protein OS=Homo sapiens GN=MDFIC PE=1 SV=2//7.00653e-139
XM_007982678.1	1.3783	1.62E-40	7.13E-39	sp|Q2IBA6|MET_CHLAE Hepatocyte growth factor receptor OS=Chlorocebus aethiops GN=MET PE=3 SV=1//0
XM_007982703.1	-0.91578	0.0001411	0.0009067	sp|Q5RCP3|LSM8_PONAB U6 snRNA-associated Sm-like protein LSm8 OS=Pongo abelii GN=LSM8 PE=3 SV=3//1.04535e-61
XM_007982790.1	-0.70255	7.55E-16	1.20E-14	sp|Q99758|ABCA3_HUMAN ATP-binding cassette sub-family A member 3 OS=Homo sapiens GN=ABCA3 PE=1 SV=2//0
XM_007982793.1	3.6309	2.44E-144	7.65E-142	sp|O15354|GPR37_HUMAN Prosaposin receptor GPR37 OS=Homo sapiens GN=GPR37 PE=1 SV=2//0
XM_007982811.1	-2.9173	4.50E-06	3.46E-05	sp|O00634|NET3_HUMAN Netrin-3 OS=Homo sapiens GN=NTN3 PE=1 SV=1//0
XM_007982814.1	1.4792	0.007563	0.036322	sp|Q2TB10|ZN800_HUMAN Zinc finger protein 800 OS=Homo sapiens GN=ZNF800 PE=1 SV=1//0
XM_007982815.1	1.2829	6.99E-08	6.35E-07	sp|Q2TB10|ZN800_HUMAN Zinc finger protein 800 OS=Homo sapiens GN=ZNF800 PE=1 SV=1//0
XM_007982816.1	-0.99088	9.23E-20	1.81E-18	sp|P84083|ARF5_RAT ADP-ribosylation factor 5 OS=Rattus norvegicus GN=Arf5 PE=1 SV=2//7.52311e-129
XM_007982817.1	1.5665	9.77E-42	4.46E-40	sp|Q96CN9|GCC1_HUMAN GRIP and coiled-coil domain-containing protein 1 OS=Homo sapiens GN=GCC1 PE=1 SV=1//0
XM_007982820.1	-0.89822	2.66E-28	7.72E-27	sp|P27449|VATL_HUMAN V-type proton ATPase 16 kDa proteolipid subunit OS=Homo sapiens GN=ATP6V0C PE=1 SV=1//1.12101e-79
XM_007982825.1	1.2442	6.49E-33	2.21E-31	sp|Q9NW13|RBM28_HUMAN RNA-binding protein 28 OS=Homo sapiens GN=RBM28 PE=1 SV=3//0
XM_007982826.1	0.43568	0.0004961	0.0029613	sp|Q9Y303|NAGA_HUMAN Putative N-acetylglucosamine-6-phosphate deacetylase OS=Homo sapiens GN=AMDHD2 PE=1 SV=2//0
XM_007982856.1	-0.47601	1.08E-06	8.85E-06	sp|Q16864|VATF_HUMAN V-type proton ATPase subunit F OS=Homo sapiens GN=ATP6V1F PE=1 SV=2//2.33458e-79
XM_007982858.1	-0.75732	0.01013	0.047352	sp|Q6ZWJ8|KCP_HUMAN Kielin/chordin-like protein OS=Homo sapiens GN=KCP PE=2 SV=2//0
XM_007982877.1	-1.1459	2.87E-34	1.04E-32	sp|Q99835|SMO_HUMAN Smoothened homolog OS=Homo sapiens GN=SMO PE=1 SV=1//0
XM_007982923.1	0.57522	2.13E-07	1.85E-06	sp|Q2VPB7|AP5B1_HUMAN AP-5 complex subunit beta-1 OS=Homo sapiens GN=AP5B1 PE=1 SV=4//0

XM_007982958.1	-1.5143	0.0064811	0.031562	sp|Q7RTY9|PRS41_HUMAN Putative serine protease 41 OS=Homo sapiens GN=PRSS41 PE=5 SV=1//2.05898e-91
XM_007982964.1	-1.2012	0.0049018	0.024491	sp|Q7RTY9|PRS41_HUMAN Putative serine protease 41 OS=Homo sapiens GN=PRSS41 PE=5 SV=1//9.82497e-176
XM_007982973.1	-0.40831	8.61E-05	0.0005693	sp|Q95KB4|S35B4_MACFA UDP-xylose and UDP-N-acetylglucosamine transporter OS=Macaca fascicularis GN=SLC35B4 PE=2 SV=1//0
XM_007982975.1	-0.28964	0.0002048	0.0012869	sp|P15121|ALDR_HUMAN Aldose reductase OS=Homo sapiens GN=AKR1B1 PE=1 SV=3//0
XM_007982978.1	-2.3436	6.40E-10	6.88E-09	sp|O60218|AK1BA_HUMAN Aldo-keto reductase family 1 member B10 OS=Homo sapiens GN=AKR1B10 PE=1 SV=2//0
XM_007983008.1	3.0351	1.96E-08	1.87E-07	sp|Q9BWK5|MRI_HUMAN Modulator of retrovirus infection homolog OS=Homo sapiens GN=MRI PE=1 SV=2//4.08379e-42
XM_007983009.1	3.0025	6.25E-48	3.59E-46	sp|Q9NV12|TM140_HUMAN Transmembrane protein 140 OS=Homo sapiens GN=TMEM140 PE=1 SV=2//2.9118e-51
XM_007983012.1	-0.74615	4.35E-10	4.77E-09	sp|A4D1P6|WDR91_HUMAN WD repeat-containing protein 91 OS=Homo sapiens GN=WDR91 PE=1 SV=2//0
XM_007983016.1	-0.91576	0.0014376	0.008014	sp|Q9UQ35|SRRM2_HUMAN Serine/arginine repetitive matrix protein 2 OS=Homo sapiens GN=SRRM2 PE=1 SV=2//1.057e-43
XM_007983032.1	-1.6356	0.0036362	0.018694	sp|Q9UQ35|SRRM2_HUMAN Serine/arginine repetitive matrix protein 2 OS=Homo sapiens GN=SRRM2 PE=1 SV=2//6.27353e-44
XM_007983037.1	-2.3067	8.86E-21	1.83E-19	sp|P68303|MT2_MACFA Metallothionein-2 OS=Macaca fascicularis GN=MT2 PE=3 SV=1//2.14723e-09
XM_007983039.1	-1.2942	2.75E-33	9.52E-32	sp|Q15370|ELOB_HUMAN Transcription elongation factor B polypeptide 2 OS=Homo sapiens GN=TCEB2 PE=1 SV=1//8.26059e-81
XM_007983065.1	1.1091	1.40E-22	3.18E-21	sp|Q70SY1|CR3L2_HUMAN Cyclic AMP-responsive element-binding protein 3-like protein 2 OS=Homo sapiens GN=CREB3L2 PE=1 SV=3//0
XM_007983088.1	-1.8599	7.72E-36	2.93E-34	sp|Q96H79|ZCCHL_HUMAN Zinc finger CCCH-type antiviral protein 1-like OS=Homo sapiens GN=ZC3HAV1L PE=1 SV=2//3.07233e-170
XM_007983090.1	1.9349	0.0012933	0.007271	sp|Q7Z2W4|ZCCHV_HUMAN Zinc finger CCCH-type antiviral protein 1 OS=Homo sapiens GN=ZC3HAV1 PE=1 SV=3//1.55492e-129
XM_007983111.1	-1.4904	9.05E-10	9.64E-09	sp|Q4G012|CG055_RAT UPF0562 protein C7orf55 homolog OS=Rattus norvegicus PE=3 SV=1//6.62146e-74
XM_007983137.1	1.8284	2.72E-70	2.80E-68	sp|Q9H0J9|PAR12_HUMAN Poly [ADP-ribose] polymerase 12 OS=Homo sapiens GN=PARP12 PE=1 SV=1//0
XM_007983158.1	-1.4626	2.04E-10	2.29E-09	sp|Q9ULE3|DEN2A_HUMAN DENN domain-containing protein 2A OS=Homo sapiens GN=DENND2A PE=2 SV=4//0
XM_007983167.1	0.9976	2.79E-08	2.63E-07	sp|P15056|BRAF_HUMAN Serine/threonine-protein kinase B-raf OS=Homo sapiens GN=BRAF PE=1 SV=4//0
XM_007983187.1	0.49361	0.0005802	0.0034346	sp|Q99640|PMYT1_HUMAN Membrane-associated tyrosine- and threonine-specific cdc2-inhibitory kinase OS=Homo sapiens GN=PKMYT1 PE=1 SV=1//0
XM_007983200.1	-1.4918	0.0080511	0.03848	sp|Q8N4S7|PAQR4_HUMAN Progestin and adipoQ receptor family member 4 OS=Homo sapiens GN=PAQR4 PE=2 SV=3//4.64245e-171
XM_007983231.1	3.3103	0	0	sp|Q9NP84|TNR12_HUMAN Tumor necrosis factor receptor superfamily member 12A OS=Homo sapiens GN=TNFRSF12A PE=1 SV=1//1.83127e-65
XM_007983267.1	-1.2014	1.73E-20	3.52E-19	sp|Q9Y2Q3|GSTK1_HUMAN Glutathione S-transferase kappa 1 OS=Homo sapiens GN=GSTK1 PE=1 SV=3//5.52691e-151
XM_007983322.1	0.67641	7.60E-10	8.12E-09	sp|Q12774|ARHG5_HUMAN Rho guanine nucleotide exchange factor 5 OS=Homo sapiens GN=ARHGEF5 PE=1 SV=3//0
XM_007983335.1	0.43273	2.19E-08	2.08E-07	sp|Q9UHC6|CNTP2_HUMAN Contactin-associated protein-like 2 OS=Homo sapiens GN=CNTNAP2 PE=1 SV=1//0
XM_007983344.1	0.75516	1.13E-20	2.32E-19	sp|Q5R4G6|CUL1_PONAB Cullin-1 OS=Pongo abelii GN=CUL1 PE=2 SV=1//0
XM_007983357.1	2.2559	6.39E-129	1.73E-126	sp|Q8TD17|ZN398_HUMAN Zinc finger protein 398 OS=Homo sapiens GN=ZNF398 PE=1 SV=1//0
XM_007983358.1	0.92609	4.46E-19	8.47E-18	sp|Q9UDV7|ZN282_HUMAN Zinc finger protein 282 OS=Homo sapiens GN=ZNF282 PE=2 SV=3//0
XM_007983398.1	0.86678	0.003928	0.020042	sp|Q75MW2|ZN767_HUMAN Protein ZNF767 OS=Homo sapiens GN=ZNF767P PE=5 SV=1//6.62297e-40
XM_007983408.1	-0.90068	3.21E-08	3.00E-07	sp|Q5EB76|VA0E2_RAT V-type proton ATPase subunit e 2 OS=Rattus norvegicus GN=Atp6v0e2 PE=3 SV=1//3.56265e-46
XM_007983412.1	-4.0092	0.0062536	0.030569	sp|Q96FA7|ZB6CL_HUMAN ZBED6 C-terminal-like protein OS=Homo sapiens GN=ZBED6CL PE=2 SV=1//2.74356e-125
XM_007983460.1	-1.2334	6.37E-08	5.79E-07	sp|Q8TDP1|RNH2C_HUMAN Ribonuclease H2 subunit C OS=Homo sapiens GN=RNASEH2C PE=1 SV=1//1.78393e-101
XM_007983508.1	0.74787	2.52E-10	2.81E-09	sp|Q9UG63|ABCF2_HUMAN ATP-binding cassette sub-family F member 2 OS=Homo sapiens GN=ABCF2 PE=1 SV=2//0
XM_007983536.1	6.9651	7.62E-23	1.76E-21	sp|Q9UGJ0|AAKG2_HUMAN 5'-AMP-activated protein kinase subunit gamma-2 OS=Homo sapiens GN=PRKAG2 PE=1 SV=1//0
XM_007983560.1	2.1584	4.85E-30	1.50E-28	sp|O43543|XRCC2_HUMAN DNA repair protein XRCC2 OS=Homo sapiens GN=XRCC2 PE=1 SV=1//4.04143e-173
XM_007983586.1	0.65108	1.01E-08	9.86E-08	sp|O15503|INSI1_HUMAN Insulin-induced gene 1 protein OS=Homo sapiens GN=INSIG1 PE=1 SV=3//2.55543e-133

XM_007983604.1	-0.71695	7.53E-19	1.42E-17	-//-
XM_007983625.1	0.52702	0.0060455	0.029638	sp|O75190|DNJB6_HUMAN DnaJ homolog subfamily B member 6 OS=Homo sapiens GN=DNAJB6 PE=1 SV=2//1.05284e-54
XM_007983655.1	0.66885	3.54E-10	3.90E-09	sp|Q86XI2|CNDG2_HUMAN Condensin-2 complex subunit G2 OS=Homo sapiens GN=NCAPG2 PE=1 SV=1//0
XM_007983679.1	-1.5524	0.0053364	0.026481	sp|Q75L30|YG027_HUMAN Putative uncharacterized protein FLJ92257 OS=Homo sapiens PE=5 SV=1//2.13441e-10
XM_007983686.1	-1.1562	2.28E-10	2.55E-09	sp|Q9UHC9|NPCL1_HUMAN Niemann-Pick C1-like protein 1 OS=Homo sapiens GN=NPC1L1 PE=1 SV=2//5.5276e-55
XM_007983701.1	0.86666	7.25E-16	1.15E-14	sp|Q6NUQ1|RINT1_HUMAN RAD50-interacting protein 1 OS=Homo sapiens GN=RINT1 PE=1 SV=1//0
XM_007983704.1	0.57144	4.55E-07	3.84E-06	sp|A4D0T7|YG055_HUMAN Putative transmembrane protein LINC00998 OS=Homo sapiens GN=LINC00998 PE=5 SV=2//2.19456e-13
XM_007983705.1	0.20895	0.01033	0.048207	sp|Q04206|TF65_HUMAN Transcription factor p65 OS=Homo sapiens GN=RELA PE=1 SV=2//0
XM_007983707.1	0.86488	5.06E-25	1.29E-23	sp|Q92621|NU205_HUMAN Nuclear pore complex protein Nup205 OS=Homo sapiens GN=NUP205 PE=1 SV=3//0
XM_007983712.1	1.6346	2.38E-16	3.91E-15	sp|Q7Z2W4|ZCCHV_HUMAN Zinc finger CCCH-type antiviral protein 1 OS=Homo sapiens GN=ZC3HAV1 PE=1 SV=3//9.32974e-77
XM_007983717.1	0.63817	1.92E-06	1.53E-05	sp|P42575|CASP2_HUMAN Caspase-2 OS=Homo sapiens GN=CASP2 PE=1 SV=2//0
XM_007983800.1	0.54467	0.0038859	0.019859	sp|P30414|NKTR_HUMAN NK-tumor recognition protein OS=Homo sapiens GN=NKTR PE=1 SV=2//0
XM_007983888.1	1.1752	0.0032859	0.017104	sp|Q96LX8|ZN597_HUMAN Zinc finger protein 597 OS=Homo sapiens GN=ZNF597 PE=2 SV=1//0
XM_007983905.1	-0.8438	3.21E-05	0.0002224	sp|Q96AT1|K1143_HUMAN Uncharacterized protein KIAA1143 OS=Homo sapiens GN=KIAA1143 PE=1 SV=2//1.17633e-72
XM_007983926.1	-0.28584	0.00612	0.029983	sp|Q9UGP4|LIMD1_HUMAN LIM domain-containing protein 1 OS=Homo sapiens GN=LIMD1 PE=1 SV=1//0
XM_007983991.1	-2.3889	7.43E-06	5.57E-05	sp|Q7Z5A4|PRS42_HUMAN Serine protease 42 OS=Homo sapiens GN=PRSS42 PE=2 SV=1//6.70191e-143
XM_007984063.1	#NAME?	0.0046233	0.023275	sp|P24855|DNAS1_HUMAN Deoxyribonuclease-1 OS=Homo sapiens GN=DNASE1 PE=1 SV=1//1.10277e-136
XM_007984086.1	-0.67202	8.66E-07	7.15E-06	sp|P27816|MAP4_HUMAN Microtubule-associated protein 4 OS=Homo sapiens GN=MAP4 PE=1 SV=3//2.13609e-142
XM_007984120.1	0.62813	1.29E-08	1.25E-07	sp|Q8N114|SHSA5_HUMAN Protein shisa-5 OS=Homo sapiens GN=SHISA5 PE=1 SV=1//1.44512e-57
XM_007984136.1	-0.59685	2.12E-16	3.48E-15	sp|P31930|QCR1_HUMAN Cytochrome b-c1 complex subunit 1, mitochondrial OS=Homo sapiens GN=UQCRC1 PE=1 SV=3//0
XM_007984178.1	-0.3376	0.0063977	0.031183	sp|Q9BU61|NDUF3_HUMAN NADH dehydrogenase [ubiquinone] 1 alpha subcomplex assembly factor 3 OS=Homo sapiens GN=NDUFAF3 PE=1 SV=1//4.66044e-117
XM_007984203.1	-0.28004	0.0013643	0.0076427	sp|P55268|LAMB2_HUMAN Laminin subunit beta-2 OS=Homo sapiens GN=LAMB2 PE=1 SV=2//0
XM_007984221.1	-0.64384	9.59E-11	1.10E-09	sp|Q865R2|GPX1_MACFU Glutathione peroxidase 1 OS=Macaca fuscata fuscata GN=GPX1 PE=2 SV=3//2.69867e-128
XM_007984222.1	-0.70668	7.79E-25	1.96E-23	sp|Q5REY6|RHOA_PONAB Transforming protein RhoA OS=Pongo abelii GN=RHOA PE=2 SV=2//5.92947e-135
XM_007984226.1	-0.88302	5.35E-10	5.80E-09	sp|Q9BSH3|NICN1_HUMAN Nicolin-1 OS=Homo sapiens GN=NICN1 PE=2 SV=1//1.28449e-149
XM_007984231.1	0.26926	0.0009511	0.005449	sp|Q6EMK4|VASN_HUMAN Vasorin OS=Homo sapiens GN=VASN PE=1 SV=1//0
XM_007984243.1	0.31923	0.0017076	0.0093881	sp|P57737|CORO7_HUMAN Coronin-7 OS=Homo sapiens GN=CORO7 PE=1 SV=2//0
XM_007984245.1	-0.56636	2.61E-06	2.05E-05	sp|P13798|ACPH_HUMAN Acylamino-acid-releasing enzyme OS=Homo sapiens GN=APEH PE=1 SV=4//0
XM_007984248.1	1.2475	0.0005572	0.0033073	sp|Q86WK7|AMGO3_HUMAN Amphoterin-induced protein 3 OS=Homo sapiens GN=AMIGO3 PE=2 SV=1//0
XM_007984296.1	-1.3778	6.06E-09	6.02E-08	sp|Q99624|S38A3_HUMAN Sodium-coupled neutral amino acid transporter 3 OS=Homo sapiens GN=SLC38A3 PE=1 SV=1//0
XM_007984297.1	-1.3879	6.68E-62	5.59E-60	sp|P04899|GNAI2_HUMAN Guanine nucleotide-binding protein G(i) subunit alpha-2 OS=Homo sapiens GN=GNAI2 PE=1 SV=3//0
XM_007984339.1	0.6196	1.18E-06	9.65E-06	sp|Q9WVF8|TUSC2_MOUSE Tumor suppressor candidate 2 OS=Mus musculus GN=Tusc2 PE=1 SV=3//2.56179e-55
XM_007984344.1	2.0575	0.0033069	0.017189	sp|Q9NS23|RASF1_HUMAN Ras association domain-containing protein 1 OS=Homo sapiens GN=RASSF1 PE=1 SV=1//1.037e-131
XM_007984348.1	0.6017	6.19E-09	6.14E-08	sp|Q12893|TM115_HUMAN Transmembrane protein 115 OS=Homo sapiens GN=TMEM115 PE=1 SV=1//3.06062e-173
XM_007984351.1	-0.86704	0.0078944	0.037781	sp|O14569|C56D2_HUMAN Cytochrome b561 domain-containing protein 2 OS=Homo sapiens GN=CYB561D2 PE=1 SV=1//2.75186e-109
XM_007984395.1	0.349	0.0001527	0.0009753	sp|Q8NDT2|RB15B_HUMAN Putative RNA-binding protein 15B OS=Homo sapiens GN=RBM15B PE=1 SV=3//0
XM_007984399.1	1.0934	3.05E-12	3.90E-11	sp|Q9Y4B4|ARIP4_HUMAN Helicase ARIP4 OS=Homo sapiens GN=RAD54L2 PE=1

				SV=4//0
XM_007984429.1	4.8148	3.09E-102	5.90E-100	sp|O43818|U3IP2_HUMAN U3 small nucleolar RNA-interacting protein 2 OS=Homo sapiens GN=RRP9 PE=1 SV=1//0
XM_007984456.1	0.5027	0.0002107	0.0013225	sp|Q16829|DUS7_HUMAN Dual specificity protein phosphatase 7 OS=Homo sapiens GN=DUSP7 PE=1 SV=4//0
XM_007984474.1	-1.281	0.0002243	0.0013996	sp|Q6IBS0|TWF2_HUMAN Twinfilin-2 OS=Homo sapiens GN=TWF2 PE=1 SV=2//0
XM_007984497.1	-1.0117	2.02E-09	2.09E-08	sp|P19123|TNNC1_MOUSE Troponin C, slow skeletal and cardiac muscles OS=Mus musculus GN=Tnnc1 PE=2 SV=1//2.57866e-100
XM_007984500.1	0.66356	0.0019822	0.010741	sp|Q9Y2I1|NISCH_HUMAN Nischarin OS=Homo sapiens GN=NISCH PE=1 SV=3//0
XM_007984501.1	-3.5092	2.56E-13	3.52E-12	sp|Q6QON3|NT5D2_RAT 5'-nucleotidase domain-containing protein 2 OS=Rattus norvegicus GN=Nt5dc2 PE=2 SV=2//0
XM_007984508.1	-1.4768	3.22E-06	2.51E-05	sp|Q8WVI0|SMIM4_HUMAN Small integral membrane protein 4 OS=Homo sapiens GN=SMIM4 PE=1 SV=2//9.32747e-42
XM_007984512.1	2.2066	6.26E-54	4.22E-52	sp|Q9BVP2|GNL3_HUMAN Guanine nucleotide-binding protein-like 3 OS=Homo sapiens GN=GNL3 PE=1 SV=2//0
XM_007984573.1	1.6288	7.16E-13	9.53E-12	sp|O60304|ZN500_HUMAN Zinc finger protein 500 OS=Homo sapiens GN=ZNF500 PE=2 SV=2//0
XM_007984574.1	-1.4915	6.59E-107	1.40E-104	sp|Q60HC7|TKT_MACFA Transketolase OS=Macaca fascicularis GN=TKT PE=2 SV=1//0
XM_007984575.1	1.3058	5.20E-30	1.61E-28	sp|Q9NPI6|DCP1A_HUMAN mRNA-decapping enzyme 1A OS=Homo sapiens GN=DCP1A PE=1 SV=2//0
XM_007984610.1	0.8089	5.88E-25	1.49E-23	sp|Q49A26|GLYR1_HUMAN Putative oxidoreductase GLYR1 OS=Homo sapiens GN=GLYR1 PE=1 SV=3//0
XM_007984621.1	0.74301	6.04E-10	6.52E-09	sp|Q4R8M1|SELK_MACFA Selenoprotein K OS=Macaca fascicularis GN=SELK PE=3 SV=3//1.76035e-36
XM_007984657.1	-0.97909	8.08E-06	6.02E-05	sp|Q9UK61|F208A_HUMAN Protein FAM208A OS=Homo sapiens GN=FAM208A PE=1 SV=3//1.96886e-96
XM_007984706.1	-0.28276	0.0002167	0.001357	sp|O60437|PEPL_HUMAN Periplakin OS=Homo sapiens GN=PPL PE=1 SV=4//0
XM_007984737.1	1.0744	3.49E-28	1.01E-26	sp|Q6L8Q7|PDE12_HUMAN 2',5'-phosphodiesterase 12 OS=Homo sapiens GN=PDE12 PE=1 SV=2//0
XM_007984740.1	1.6245	1.34E-09	1.41E-08	sp|Q5RCF1|ARF4_PONAB ADP-ribosylation factor 4 OS=Pongo abelii GN=ARF4 PE=2 SV=3//3.5617e-106
XM_007984741.1	-0.7856	6.80E-11	7.89E-10	sp|Q8IWF6|DEN6A_HUMAN Protein DENND6A OS=Homo sapiens GN=DENND6A PE=1 SV=1//0
XM_007984772.1	-1.0024	2.76E-08	2.60E-07	sp|Q9UK23|NAGPA_HUMAN N-acetylglucosamine-1-phosphodiester alpha-N-acetylglucosaminidase OS=Homo sapiens GN=NAGPA PE=1 SV=2//0
XM_007984776.1	1.1426	3.60E-13	4.87E-12	sp|P86397|HTD2_HUMAN Hydroxyacyl-thioester dehydratase type 2, mitochondrial OS=Homo sapiens GN=RPP14 PE=1 SV=1//1.49624e-116
XM_007984787.1	-0.71183	5.43E-13	7.25E-12	sp|P11177|ODPB_HUMAN Pyruvate dehydrogenase E1 component subunit beta, mitochondrial OS=Homo sapiens GN=PDHB PE=1 SV=3//0
XM_007984824.1	-1.3428	9.23E-07	7.60E-06	sp|P23470|PTPRG_HUMAN Receptor-type tyrosine-protein phosphatase gamma OS=Homo sapiens GN=PTPRG PE=1 SV=4//0
XM_007984866.1	1.7328	0.0035622	0.01835	sp|A6NMZ2|SNTAN_HUMAN Sentan OS=Homo sapiens GN=SNTN PE=2 SV=1//3.68229e-87
XM_007984890.1	2.1992	1.79E-35	6.73E-34	sp|Q7Z3G6|PRIC2_HUMAN Prickle-like protein 2 OS=Homo sapiens GN=PRICKLE2 PE=1 SV=2//0
XM_007984939.1	-0.3209	3.65E-05	0.0002511	sp|Q4R4R4|PRAF3_MACFA PRA1 family protein 3 OS=Macaca fascicularis GN=ARL6IP5 PE=2 SV=1//7.82827e-118
XM_007984948.1	-1.7726	0.000181	0.001145	sp|Q9Y2L6|FRM4B_HUMAN FERM domain-containing protein 4B OS=Homo sapiens GN=FRMD4B PE=1 SV=4//0
XM_007984949.1	-1.2677	5.34E-06	4.08E-05	-//-
XM_007984982.1	-0.84062	0.0001012	0.0006638	sp|Q9NS67|GPR27_HUMAN Probable G-protein coupled receptor 27 OS=Homo sapiens GN=GPR27 PE=2 SV=1//1.88231e-142
XM_007984997.1	2.6147	5.62E-217	3.24E-214	sp|Q8N488|RYBP_HUMAN RING1 and YY1-binding protein OS=Homo sapiens GN=RYBP PE=1 SV=2//1.96396e-76
XM_007984998.1	0.88853	1.59E-10	1.80E-09	sp|Q6PI26|SHQ1_HUMAN Protein SHQ1 homolog OS=Homo sapiens GN=SHQ1 PE=1 SV=2//0
XM_007984999.1	-2.1053	1.46E-16	2.41E-15	sp|A0PJZ3|GXLT2_HUMAN Glucoside xylosyltransferase 2 OS=Homo sapiens GN=GXYLT2 PE=2 SV=2//0
XM_007985050.1	0.47859	0.0014164	0.00791	sp|Q53H47|SETMR_HUMAN Histone-lysine N-methyltransferase SETMAR OS=Homo sapiens GN=SETMAR PE=1 SV=2//0
XM_007985051.1	-2.4187	1.39E-16	2.30E-15	sp|Q6UXK5|LRRN1_HUMAN Leucine-rich repeat neuronal protein 1 OS=Homo sapiens GN=LRRN1 PE=1 SV=1//0
XM_007985061.1	2.3809	3.77E-185	1.65E-182	sp|O14503|BHE40_HUMAN Class E basic helix-loop-helix protein 40 OS=Homo sapiens GN=BHLHE40 PE=1 SV=1//0
XM_007985075.1	0.74176	1.28E-19	2.50E-18	sp|Q66HA6|ARL8B_RAT ADP-ribosylation factor-like protein 8B OS=Rattus norvegicus GN=Arl8b PE=2 SV=1//9.07202e-121
XM_007985076.1	0.52284	4.55E-07	3.84E-06	sp|Q92611|EDEM1_HUMAN ER degradation-enhancing alpha-mannosidase-like protein 1 OS=Homo sapiens GN=EDEM1 PE=1 SV=1//0

XM_007985091.1	3.27	9.68E-172	3.79E-169	sp|Q9NZU5|LMCD1_HUMAN LIM and cysteine-rich domains protein 1 OS=Homo sapiens GN=LMCD1 PE=1 SV=1//0
XM_007985101.1	1.3638	3.27E-19	6.29E-18	sp|Q9NS91|RAD18_HUMAN E3 ubiquitin-protein ligase RAD18 OS=Homo sapiens GN=RAD18 PE=1 SV=2//0
XM_007985119.1	-1.2978	0.0016115	0.0088967	-//-
XM_007985159.1	-1.1687	2.25E-17	3.90E-16	sp|Q9Y4R7|TTLL3_HUMAN Tubulin monoglycylase TTLL3 OS=Homo sapiens GN=TTLL3 PE=1 SV=2//0
XM_007985164.1	-0.49044	7.16E-08	6.49E-07	sp|O75528|TADA3_HUMAN Transcriptional adapter 3 OS=Homo sapiens GN=TADA3 PE=1 SV=1//0
XM_007985165.1	0.80596	1.35E-10	1.53E-09	sp|Q8N5M9|JAGN1_HUMAN Protein jagunal homolog 1 OS=Homo sapiens GN=JAGN1 PE=1 SV=1//1.94007e-114
XM_007985193.1	1.3313	0.0039274	0.020042	sp|Q5FWE3|PRRT3_HUMAN Proline-rich transmembrane protein 3 OS=Homo sapiens GN=PRRT3 PE=1 SV=3//0
XM_007985205.1	0.93892	0.0014959	0.0083119	sp|Q96AQ7|CIDEC_HUMAN Cell death activator CIDE-3 OS=Homo sapiens GN=CIDEC PE=1 SV=1//2.30165e-23
XM_007985209.1	-0.646	2.46E-10	2.75E-09	sp|Q8WUW1|BRK1_HUMAN Protein BRICK1 OS=Homo sapiens GN=BRK1 PE=1 SV=1//3.008e-46
XM_007985212.1	0.75192	5.20E-06	3.97E-05	sp|Q93075|TATD2_HUMAN Putative deoxyribonuclease TATDN2 OS=Homo sapiens GN=TATDN2 PE=2 SV=2//0
XM_007985224.1	1.5248	1.52E-22	3.45E-21	sp|Q96B77|TM186_HUMAN Transmembrane protein 186 OS=Homo sapiens GN=TMEM186 PE=2 SV=1//2.88403e-133
XM_007985236.1	2.0775	0.00332	0.017241	sp|Q14135|VGLL4_HUMAN Transcription cofactor vestigial-like protein 4 OS=Homo sapiens GN=VGLL4 PE=1 SV=4//0
XM_007985245.1	2.2717	0.0007222	0.0042068	sp|Q9N2B0|HRH1_PONPY Histamine H1 receptor OS=Pongo pygmaeus GN=HRH1 PE=3 SV=1//0
XM_007985261.1	2.1206	8.21E-06	6.12E-05	sp|O18924|PPARG_MACMU Peroxisome proliferator-activated receptor gamma OS=Macaca mulatta GN=PPARG PE=2 SV=1//0
XM_007985263.1	2.0719	1.46E-49	8.89E-48	sp|Q8NCE0|SEN2_HUMAN tRNA-splicing endonuclease subunit Sen2 OS=Homo sapiens GN=TSEN2 PE=1 SV=2//0
XM_007985266.1	0.40682	8.55E-05	0.0005657	sp|Q9H000|MKRN2_HUMAN Probable E3 ubiquitin-protein ligase makorin-2 OS=Homo sapiens GN=MKRN2 PE=1 SV=2//0
XM_007985280.1	-0.7113	1.90E-24	4.68E-23	sp|P62912|RL32_RAT 60S ribosomal protein L32 OS=Rattus norvegicus GN=Rpl32 PE=1 SV=2//8.74144e-85
XM_007985290.1	-0.25943	0.0003501	0.0021348	sp|Q8TEM1|PO210_HUMAN Nuclear pore membrane glycoprotein 210 OS=Homo sapiens GN=NUP210 PE=1 SV=3//0
XM_007985310.1	0.72021	1.59E-13	2.21E-12	sp|Q9BTV4|TMM43_HUMAN Transmembrane protein 43 OS=Homo sapiens GN=TMEM43 PE=1 SV=1//0
XM_007985311.1	-0.33745	0.0083252	0.039694	sp|Q01831|XPC_HUMAN DNA repair protein complementing XP-C cells OS=Homo sapiens GN=XPC PE=1 SV=4//0
XM_007985313.1	-0.9831	2.47E-13	3.40E-12	sp|P62311|LSM3_MOUSE U6 snRNA-associated Sm-like protein LSm3 OS=Mus musculus GN=Lsm3 PE=3 SV=2//9.64876e-49
XM_007985325.1	1.3438	2.06E-33	7.17E-32	sp|Q6PII3|CC174_HUMAN Coiled-coil domain-containing protein 174 OS=Homo sapiens GN=CCDC174 PE=2 SV=3//0
XM_007985334.1	2.0683	6.71E-11	7.79E-10	sp|Q6ZNL6|FGD5_HUMAN FYVE, RhoGEF and PH domain-containing protein 5 OS=Homo sapiens GN=FGD5 PE=1 SV=3//0
XM_007985335.1	1.5633	3.86E-79	4.83E-77	sp|Q28HY5|CP072_XENTR UPF0472 protein C16orf72 homolog OS=Xenopus tropicalis GN=TEgg029f10.1 PE=2 SV=1//1.43904e-144
XM_007985336.1	-0.74662	7.92E-08	7.15E-07	sp|P82663|RT25_HUMAN 28S ribosomal protein S25, mitochondrial OS=Homo sapiens GN=MRPS25 PE=1 SV=1//4.63029e-103
XM_007985371.1	3.3556	3.41E-14	4.92E-13	sp|O94876|TMCC1_HUMAN Transmembrane and coiled-coil domains protein 1 OS=Homo sapiens GN=TMCC1 PE=1 SV=3//0
XM_007985375.1	-1.1692	5.14E-10	5.59E-09	sp|Q9Y4D7|PLXD1_HUMAN Plexin-D1 OS=Homo sapiens GN=PLXND1 PE=1 SV=3//0
XM_007985407.1	-0.76667	1.70E-12	2.22E-11	sp|Q86YS6|RAB43_HUMAN Ras-related protein Rab-43 OS=Homo sapiens GN=RAB43 PE=1 SV=1//9.67522e-124
XM_007985418.1	-1.052	0.0023271	0.012463	sp|Q9H845|ACAD9_HUMAN Acyl-CoA dehydrogenase family member 9, mitochondrial OS=Homo sapiens GN=ACAD9 PE=1 SV=1//4.94077e-24
XM_007985419.1	-0.66497	5.33E-09	5.31E-08	sp|Q9H845|ACAD9_HUMAN Acyl-CoA dehydrogenase family member 9, mitochondrial OS=Homo sapiens GN=ACAD9 PE=1 SV=1//0
XM_007985442.1	-0.26271	9.65E-05	0.0006353	sp|Q4R4T0|RPN1_MACFA Dolichyl-diphosphooligosaccharide--protein glycosyltransferase subunit 1 OS=Macaca fascicularis GN=RPN1 PE=2 SV=1//0
XM_007985457.1	0.21722	0.0016414	0.0090491	sp|P61621|S61A1_RAT Protein transport protein Sec61 subunit alpha isoform 1 OS=Rattus norvegicus GN=Sec61a1 PE=2 SV=2//0
XM_007985512.1	1.2224	2.84E-06	2.22E-05	sp|Q2QGD7|ZXDC_HUMAN Zinc finger protein ZXDC OS=Homo sapiens GN=ZXDC PE=1 SV=2//0
XM_007985522.1	-0.60277	1.28E-05	9.35E-05	sp|Q5RFM9|AL1L1_PONAB Cytosolic 10-formyltetrahydrofolate dehydrogenase OS=Pongo abelii GN=ALDH1L1 PE=2 SV=1//0
XM_007985535.1	0.71797	1.52E-19	2.96E-18	sp|O95219|SNX4_HUMAN Sorting nexin-4 OS=Homo sapiens GN=SNX4 PE=1 SV=1//0
XM_007985536.1	1.8157	5.37E-10	5.82E-09	sp|Q9BXB4|OSB11_HUMAN Oxysterol-binding protein-related protein 11

				OS=Homo sapiens GN=OSBPL11 PE=1 SV=2//0
XM_007985544.1	-2.6331	2.62E-05	0.0001835	sp|Q9ULI3|HEG1_HUMAN Protein HEG homolog 1 OS=Homo sapiens GN=HEG1 PE=1 SV=3//0
XM_007985546.1	-0.58476	4.92E-13	6.58E-12	sp|P18084|ITB5_HUMAN Integrin beta-5 OS=Homo sapiens GN=ITGB5 PE=1 SV=1//0
XM_007985548.1	1.0339	1.15E-17	2.03E-16	sp|P11172|UMPS_HUMAN Uridine 5'-monophosphate synthase OS=Homo sapiens GN=UMPS PE=1 SV=1//0
XM_007985573.1	0.72318	8.24E-19	1.55E-17	sp|Q49A88|CCD14_HUMAN Coiled-coil domain-containing protein 14 OS=Homo sapiens GN=CCDC14 PE=1 SV=3//0
XM_007985588.1	-0.88457	1.45E-07	1.28E-06	sp|O95622|ADCY5_HUMAN Adenylate cyclase type 5 OS=Homo sapiens GN=ADCY5 PE=1 SV=3//0
XM_007985597.1	0.96029	2.29E-13	3.16E-12	sp|Q96SL1|DIRC2_HUMAN Disrupted in renal carcinoma protein 2 OS=Homo sapiens GN=DIRC2 PE=1 SV=1//0
XM_007985598.1	2.3208	1.02E-28	3.00E-27	sp|Q460N5|PAR14_HUMAN Poly [ADP-ribose] polymerase 14 OS=Homo sapiens GN=PARP14 PE=1 SV=3//0
XM_007985604.1	2.0046	2.39E-25	6.23E-24	sp|Q8TDB6|DTX3L_HUMAN E3 ubiquitin-protein ligase DTX3L OS=Homo sapiens GN=DTX3L PE=1 SV=1//0
XM_007985606.1	0.81129	5.60E-22	1.24E-20	sp|Q5R909|IMA5_PONAB Importin subunit alpha-5 OS=Pongo abelii GN=KPNA1 PE=2 SV=1//0
XM_007985612.1	1.2575	2.75E-15	4.20E-14	sp|Q5RE95|WDR5B_PONAB WD repeat-containing protein 5B OS=Pongo abelii PE=2 SV=1//0
XM_007985637.1	1.0701	8.30E-18	1.47E-16	sp|Q15051|IQCB1_HUMAN IQ calmodulin-binding motif-containing protein 1 OS=Homo sapiens GN=IQCB1 PE=1 SV=1//0
XM_007985662.1	1.3784	3.68E-05	0.0002534	sp|Q9Y2K9|STB5L_HUMAN Syntaxin-binding protein 5-like OS=Homo sapiens GN=STXBP5L PE=1 SV=2//0
XM_007985665.1	0.98313	6.41E-28	1.83E-26	sp|Q5R8H5|T2EA_PONAB General transcription factor IIE subunit 1 OS=Pongo abelii GN=GTF2E1 PE=2 SV=1//0
XM_007985667.1	-0.67507	2.81E-05	0.0001964	sp|P0CB71|NDUB4_PONPY NADH dehydrogenase [ubiquinone] 1 beta subcomplex subunit 4 OS=Pongo pygmaeus GN=NDUFB4 PE=2 SV=1//5.35727e-72
XM_007985669.1	-1.6524	0.0086926	0.041276	sp|Q5R9Y1|FSTL1_PONAB Follistatin-related protein 1 OS=Pongo abelii GN=FSTL1 PE=2 SV=2//0
XM_007985672.1	0.71882	1.50E-07	1.32E-06	sp|Q96CX6|LRC58_HUMAN Leucine-rich repeat-containing protein 58 OS=Homo sapiens GN=LRRC58 PE=1 SV=2//7.56676e-173
XM_007985692.1	-0.77457	0.0001682	0.0010701	sp|Q14061|COX17_HUMAN Cytochrome c oxidase copper chaperone OS=Homo sapiens GN=COX17 PE=1 SV=2//1.01055e-25
XM_007985707.1	1.5301	4.24E-37	1.66E-35	sp|Q9NPL8|TIDC1_HUMAN Complex I assembly factor TIMMDC1, mitochondrial OS=Homo sapiens GN=TIMMDC1 PE=1 SV=2//2.31404e-173
XM_007985762.1	2.359	1.32E-66	1.25E-64	sp|Q9H974|QTRD1_HUMAN Queuine tRNA-ribosyltransferase subunit QTRTD1 OS=Homo sapiens GN=QTRTD1 PE=1 SV=1//0
XM_007985778.1	3.575	9.08E-28	2.57E-26	sp|O15524|SOCS1_HUMAN Suppressor of cytokine signaling 1 OS=Homo sapiens GN=SOCS1 PE=1 SV=1//5.19402e-114
XM_007985802.1	1.4065	2.13E-15	3.27E-14	sp|Q6NW34|CC017_HUMAN Uncharacterized protein C3orf17 OS=Homo sapiens GN=C3orf17 PE=1 SV=3//0
XM_007985805.1	0.75249	0.0094045	0.044311	sp|Q8N3Z3|GTPB8_HUMAN GTP-binding protein 8 OS=Homo sapiens GN=GTPBP8 PE=2 SV=1//4.34625e-173
XM_007985819.1	1.2791	3.38E-33	1.17E-31	sp|Q9NT62|ATG3_HUMAN Ubiquitin-like-conjugating enzyme ATG3 OS=Homo sapiens GN=ATG3 PE=1 SV=1//0
XM_007985836.1	0.72007	0.0037058	0.019005	sp|Q5BVD1|TTMP_HUMAN TPA-induced transmembrane protein OS=Homo sapiens GN=TTMP PE=1 SV=2//3.49161e-124
XM_007985845.1	3.9231	5.66E-54	3.83E-52	sp|Q0VAA5|PLCX2_HUMAN PI-PLC X domain-containing protein 2 OS=Homo sapiens GN=PLCXD2 PE=2 SV=1//0
XM_007985937.1	1.4313	9.66E-22	2.09E-20	sp|Q6PKC3|TXD11_HUMAN Thioredoxin domain-containing protein 11 OS=Homo sapiens GN=TXNDC11 PE=1 SV=2//0
XM_007985958.1	1.5331	9.27E-64	8.19E-62	sp|Q5RCE6|RL1D1_PONAB Ribosomal L1 domain-containing protein 1 OS=Pongo abelii GN=RSL1D1 PE=2 SV=2//0
XM_007985959.1	-0.64369	2.77E-06	2.17E-05	sp|Q5RCA5|NXPE3_PONAB NXPE family member 3 OS=Pongo abelii GN=NXPE3 PE=2 SV=1//0
XM_007985964.1	-0.56191	8.30E-11	9.58E-10	sp|P83732|RL24_RAT 60S ribosomal protein L24 OS=Rattus norvegicus GN=Rpl24 PE=2 SV=1//4.6018e-76
XM_007985968.1	1.6382	9.65E-42	4.41E-40	sp|Q7L0Y3|MRRP1_HUMAN Mitochondrial ribonuclease P protein 1 OS=Homo sapiens GN=TRMT10C PE=1 SV=2//0
XM_007985969.1	1.1223	1.15E-16	1.92E-15	sp|Q5RCE6|RL1D1_PONAB Ribosomal L1 domain-containing protein 1 OS=Pongo abelii GN=RSL1D1 PE=2 SV=2//0
XM_007986053.1	1.2659	1.83E-43	8.88E-42	sp|O94826|TOM70_HUMAN Mitochondrial import receptor subunit TOM70 OS=Homo sapiens GN=TOMM70A PE=1 SV=1//0
XM_007986140.1	1.4835	2.08E-10	2.34E-09	sp|Q9H649|NSUN3_HUMAN Putative methyltransferase NSUN3 OS=Homo sapiens GN=NSUN3 PE=2 SV=1//0
XM_007986148.1	-0.9831	9.35E-10	9.94E-09	sp|Q28520|PROS_MACMU Vitamin K-dependent protein S (Fragment) OS=Macaca mulatta GN=PROS1 PE=2 SV=2//0
XM_007986169.1	0.6502	8.88E-08	7.97E-07	sp|Q8BJF9|CHM2B_MOUSE Charged multivesicular body protein 2b OS=Mus musculus GN=Chmp2b PE=1 SV=1//3.01732e-105

XM_007986199.1	1.1122	1.01E-11	1.25E-10	sp|Q92889|XPF_HUMAN DNA repair endonuclease XPF OS=Homo sapiens GN=ERCC4 PE=1 SV=3//0
XM_007986201.1	0.33271	0.0005753	0.0034066	sp|Q9UPV9|TRAK1_HUMAN Trafficking kinesin-binding protein 1 OS=Homo sapiens GN=TRAK1 PE=1 SV=1//0
XM_007986203.1	-1.0968	1.16E-05	8.51E-05	-//-
XM_007986204.1	0.47805	4.39E-07	3.71E-06	sp|Q9NS87|KIF15_HUMAN Kinesin-like protein KIF15 OS=Homo sapiens GN=KIF15 PE=1 SV=1//0
XM_007986205.1	0.25025	0.0004033	0.0024416	sp|Q92922|SMRC1_HUMAN SWI/SNF complex subunit SMARCC1 OS=Homo sapiens GN=SMARCC1 PE=1 SV=3//1.57833e-08
XM_007986207.1	2.6397	1.09E-145	3.46E-143	sp|P30304|MPIP1_HUMAN M-phase inducer phosphatase 1 OS=Homo sapiens GN=CDC25A PE=1 SV=2//0
XM_007986209.1	0.87445	1.10E-21	2.38E-20	sp|P78332|RBM6_HUMAN RNA-binding protein 6 OS=Homo sapiens GN=RBM6 PE=1 SV=5//0
XM_007986210.1	1.4236	4.29E-26	1.14E-24	sp|P55145|MANF_HUMAN Mesencephalic astrocyte-derived neurotrophic factor OS=Homo sapiens GN=MANF PE=1 SV=3//3.24138e-125
XM_007986214.1	1.2899	0.0001239	0.0008024	sp|Q5RB79|RPP14_PONAB Ribonuclease P protein subunit p14 OS=Pongo abelii GN=RPP14 PE=2 SV=3//7.05341e-42
XM_007986217.1	0.54131	4.72E-11	5.56E-10	sp|Q9BXW9|FACD2_HUMAN Fanconi anemia group D2 protein OS=Homo sapiens GN=FANCD2 PE=1 SV=2//0
XM_007986218.1	1.4555	3.55E-23	8.29E-22	sp|O43187|IRAK2_HUMAN Interleukin-1 receptor-associated kinase-like 2 OS=Homo sapiens GN=IRAK2 PE=1 SV=2//2.31674e-86
XM_007986222.1	-0.77383	4.62E-09	4.63E-08	-//-
XM_007986224.1	-1.8082	0.0002762	0.0017019	-//-
XM_007986225.1	-0.41257	9.67E-08	8.65E-07	sp|Q9Y678|COPG1_HUMAN Coatomer subunit gamma-1 OS=Homo sapiens GN=COPG1 PE=1 SV=1//0
XM_007986226.1	-1.3413	8.05E-34	2.86E-32	sp|Q9NZ53|PDXL2_HUMAN Podocalyxin-like protein 2 OS=Homo sapiens GN=PODXL2 PE=1 SV=1//0
XM_007986233.1	1.431	5.05E-53	3.30E-51	sp|O75417|DPOLQ_HUMAN DNA polymerase theta OS=Homo sapiens GN=POLQ PE=1 SV=2//0
XM_007986290.1	-0.90234	7.05E-23	1.63E-21	sp|P58238|PSME1_MACFA Proteasome activator complex subunit 1 OS=Macaca fascicularis GN=PSME1 PE=2 SV=1//1.20644e-154
XM_007986294.1	0.93251	1.42E-23	3.36E-22	sp|Q96EP0|RNF31_HUMAN E3 ubiquitin-protein ligase RNF31 OS=Homo sapiens GN=RNF31 PE=1 SV=1//0
XM_007986295.1	-0.68587	3.31E-10	3.66E-09	sp|Q9UL46|PSME2_HUMAN Proteasome activator complex subunit 2 OS=Homo sapiens GN=PSME2 PE=1 SV=4//1.53974e-157
XM_007986296.1	0.40669	0.0025294	0.013467	sp|Q8TEX9|IPO4_HUMAN Importin-4 OS=Homo sapiens GN=IPO4 PE=1 SV=2//0
XM_007986297.1	1.3066	0.0020621	0.011144	sp|O95072|REC8_HUMAN Meiotic recombination protein REC8 homolog OS=Homo sapiens GN=REC8 PE=1 SV=1//0
XM_007986303.1	-0.71869	3.47E-06	2.69E-05	sp|Q9BY43|CHM4A_HUMAN Charged multivesicular body protein 4a OS=Homo sapiens GN=CHMP4A PE=1 SV=3//1.31554e-72
XM_007986315.1	-1.1463	3.37E-20	6.75E-19	sp|Q8N5H3|FA89B_HUMAN Protein FAM89B OS=Homo sapiens GN=FAM89B PE=1 SV=2//3.96718e-62
XM_007986317.1	-1.0495	2.54E-20	5.13E-19	sp|Q71UE8|NEDD8_RAT NEDD8 OS=Rattus norvegicus GN=Nedd8 PE=1 SV=1//1.47225e-26
XM_007986318.1	-0.47634	0.0001908	0.0012025	sp|Q9BSI4|TINF2_HUMAN TERF1-interacting nuclear factor 2 OS=Homo sapiens GN=TINF2 PE=1 SV=1//0
XM_007986327.1	#NAME?	0.0006398	0.0037617	sp|Q8NFM4|ADCY4_HUMAN Adenylate cyclase type 4 OS=Homo sapiens GN=ADCY4 PE=1 SV=1//0
XM_007986342.1	-1.8503	5.82E-22	1.28E-20	sp|Q9P2P1|NYNRI_HUMAN Protein NYNRIN OS=Homo sapiens GN=NYNRIN PE=2 SV=3//0
XM_007986365.1	1.9552	1.27E-48	7.47E-47	sp|Q9NYV6|RRN3_HUMAN RNA polymerase I-specific transcription initiation factor RRN3 OS=Homo sapiens GN=RRN3 PE=1 SV=1//0
XM_007986402.1	-0.38731	0.0081946	0.03914	sp|Q96FN9|DTD2_HUMAN Probable D-tyrosyl-tRNA(Tyr) deacylase 2 OS=Homo sapiens GN=DTD2 PE=2 SV=1//1.09195e-113
XM_007986456.1	-0.55497	5.99E-09	5.95E-08	sp|Q969W0|SPTSA_HUMAN Serine palmitoyltransferase small subunit A OS=Homo sapiens GN=SPTSSA PE=1 SV=2//2.46156e-33
XM_007986460.1	-0.61029	0.0009675	0.0055345	sp|Q9UNH7|SNX6_HUMAN Sorting nexin-6 OS=Homo sapiens GN=SNX6 PE=1 SV=1//9.56012e-59
XM_007986466.1	-1.5381	0.000142	0.0009118	sp|Q4R3M6|NDUS5_MACFA NADH dehydrogenase [ubiquinone] iron-sulfur protein 5 OS=Macaca fascicularis GN=NDUFS5 PE=3 SV=3//3.09591e-67
XM_007986467.1	1.1328	1.22E-44	6.22E-43	sp|Q9NRL2|BAZ1A_HUMAN Bromodomain adjacent to zinc finger domain protein 1A OS=Homo sapiens GN=BAZ1A PE=1 SV=2//0
XM_007986481.1	1.8028	3.67E-08	3.41E-07	sp|Q969Q6|P2R3C_HUMAN Serine/threonine-protein phosphatase 2A regulatory subunit B'' subunit gamma OS=Homo sapiens GN=PPP2R3C PE=1 SV=1//0
XM_007986483.1	-0.24106	0.0073426	0.035333	sp|P60901|PSA6_RAT Proteasome subunit alpha type-6 OS=Rattus norvegicus GN=Psma6 PE=1 SV=1//0
XM_007986484.1	1.0123	1.12E-16	1.88E-15	sp|P25963|IKBA_HUMAN NF-kappa-B inhibitor alpha OS=Homo sapiens GN=NFKBIA PE=1 SV=1//0
XM_007986515.1	-1.8266	3.93E-05	0.0002698	sp|Q9NXR1|NDE1_HUMAN Nuclear distribution protein nudE homolog 1

				OS=Homo sapiens GN=NDE1 PE=1 SV=2//0
XM_007986523.1	1.4973	1.70E-30	5.33E-29	sp|P55317|FOXA1_HUMAN Hepatocyte nuclear factor 3-alpha OS=Homo sapiens GN=FOXA1 PE=1 SV=2//0
XM_007986545.1	-0.77185	3.18E-06	2.47E-05	sp|Q15436|SC23A_HUMAN Protein transport protein Sec23A OS=Homo sapiens GN=SEC23A PE=1 SV=2//0
XM_007986552.1	-0.42779	1.45E-07	1.28E-06	sp|Q5R5X0|PININ_PONAB Pinin OS=Pongo abelii GN=PNN PE=2 SV=1//9.70803e-22
XM_007986562.1	1.1249	7.03E-14	1.00E-12	sp|Q7Z6M2|FBX33_HUMAN F-box only protein 33 OS=Homo sapiens GN=FBXO33 PE=1 SV=1//0
XM_007986569.1	-1.2509	2.15E-14	3.13E-13	sp|O95255|MRP6_HUMAN Multidrug resistance-associated protein 6 OS=Homo sapiens GN=ABCC6 PE=1 SV=2//0
XM_007986582.1	0.73986	5.14E-05	0.000349	sp|Q9NXS3|KLH28_HUMAN Kelch-like protein 28 OS=Homo sapiens GN=KLHL28 PE=2 SV=2//0
XM_007986593.1	-0.97863	5.12E-26	1.36E-24	sp|Q00688|FKBP3_HUMAN Peptidyl-prolyl cis-trans isomerase FKBP3 OS=Homo sapiens GN=FKBP3 PE=1 SV=1//1.51962e-160
XM_007986612.1	-0.82137	3.90E-19	7.44E-18	sp|P83883|RL36A_RAT 60S ribosomal protein L36a OS=Rattus norvegicus GN=Rpl36a PE=1 SV=2//1.50064e-70
XM_007986613.1	0.81658	8.02E-18	1.42E-16	sp|Q10469|MGAT2_HUMAN Alpha-1,6-mannosyl-glycoprotein 2-beta-N-acetylglucosaminyltransferase OS=Homo sapiens GN=MGAT2 PE=1 SV=1//0
XM_007986624.1	0.33871	1.29E-05	9.41E-05	sp|P62332|ARF6_RAT ADP-ribosylation factor 6 OS=Rattus norvegicus GN=Arf6 PE=1 SV=2//8.9906e-116
XM_007986656.1	1.1386	6.96E-27	1.90E-25	sp|Q9H4B6|SAV1_HUMAN Protein salvador homolog 1 OS=Homo sapiens GN=SAV1 PE=1 SV=2//0
XM_007986658.1	-1.6764	0.0001937	0.0012197	sp|Q8N4C6|NIN_HUMAN Ninein OS=Homo sapiens GN=NIN PE=1 SV=4//0
XM_007986668.1	-1.3623	0.0066466	0.032288	sp|P06737|PYGL_HUMAN Glycogen phosphorylase, liver form OS=Homo sapiens GN=PYGL PE=1 SV=4//0
XM_007986670.1	-0.58884	8.06E-10	8.59E-09	sp|Q9H3N1|TMX1_HUMAN Thioredoxin-related transmembrane protein 1 OS=Homo sapiens GN=TMX1 PE=1 SV=1//1.36946e-156
XM_007986672.1	-1.663	1.10E-53	7.28E-52	sp|Q15041|AR6P1_HUMAN ADP-ribosylation factor-like protein 6-interacting protein 1 OS=Homo sapiens GN=ARL6IP1 PE=1 SV=2//1.24226e-116
XM_007986690.1	-0.61331	3.96E-12	5.05E-11	sp|Q5R8I2|CN166_PONAB UPF0568 protein C14orf166 homolog OS=Pongo abelii PE=2 SV=1//6.06591e-167
XM_007986697.1	-1.4123	2.67E-17	4.62E-16	sp|Q96HE7|ERO1A_HUMAN ERO1-like protein alpha OS=Homo sapiens GN=ERO1L PE=1 SV=2//0
XM_007986725.1	-0.82799	4.83E-10	5.27E-09	sp|Q16667|CDKN3_HUMAN Cyclin-dependent kinase inhibitor 3 OS=Homo sapiens GN=CDKN3 PE=1 SV=1//2.21065e-143
XM_007986736.1	2.7907	0.0007916	0.0045856	sp|Q9UPU9|SMAG1_HUMAN Protein Smaug homolog 1 OS=Homo sapiens GN=SAMD4A PE=1 SV=3//0
XM_007986742.1	1.5884	2.86E-75	3.27E-73	sp|Q8WXH5|SOCS4_HUMAN Suppressor of cytokine signaling 4 OS=Homo sapiens GN=SOCS4 PE=1 SV=1//0
XM_007986747.1	0.5711	8.05E-12	1.00E-10	sp|Q5R623|MISSL_PONAB MAPK-interacting and spindle-stabilizing protein-like OS=Pongo abelii GN=MAPK1IP1L PE=2 SV=3//3.65481e-12
XM_007986749.1	-0.71414	3.91E-12	4.99E-11	sp|P17931|LEG3_HUMAN Galectin-3 OS=Homo sapiens GN=LGALS3 PE=1 SV=5//4.04386e-90
XM_007986769.1	-0.41094	0.0089029	0.042156	sp|Q9HAT8|PELI2_HUMAN E3 ubiquitin-protein ligase pellino homolog 2 OS=Homo sapiens GN=PELI2 PE=1 SV=1//0
XM_007986770.1	#NAME?	0.0004439	0.0026678	sp|Q9HAT8|PELI2_HUMAN E3 ubiquitin-protein ligase pellino homolog 2 OS=Homo sapiens GN=PELI2 PE=1 SV=1//0
XM_007986787.1	0.93591	6.01E-23	1.39E-21	sp|Q147X3|NAA30_HUMAN N-alpha-acetyltransferase 30 OS=Homo sapiens GN=NAA30 PE=1 SV=1//4.96209e-167
XM_007986802.1	-2.1955	3.97E-08	3.67E-07	sp|Q86TY3|CN037_HUMAN Uncharacterized protein C14orf37 OS=Homo sapiens GN=C14orf37 PE=1 SV=1//0
XM_007986803.1	1.5829	1.01E-36	3.90E-35	sp|Q9BVV6|TALD3_HUMAN Protein TALPID3 OS=Homo sapiens GN=KIAA0586 PE=1 SV=4//0
XM_007986825.1	-0.41119	1.05E-06	8.64E-06	sp|Q9BZ67|FRMD8_HUMAN FERM domain-containing protein 8 OS=Homo sapiens GN=FRMD8 PE=1 SV=1//0
XM_007986833.1	-1.0301	2.31E-05	0.000163	sp|Q16799|RTN1_HUMAN Reticulon-1 OS=Homo sapiens GN=RTN1 PE=1 SV=1//0
XM_007986886.1	0.59057	8.45E-08	7.60E-07	sp|P51948|MAT1_HUMAN CDK-activating kinase assembly factor MAT1 OS=Homo sapiens GN=MNAT1 PE=1 SV=1//0
XM_007986893.1	1.3978	8.72E-24	2.09E-22	sp|F7GSQ4|TRM5_MACMU tRNA (guanine(37)-N1)-methyltransferase OS=Macaca mulatta GN=TRMT5 PE=3 SV=1//0
XM_007986908.1	0.97282	4.98E-10	5.44E-09	sp|Q4R6W9|SNPC1_MACFA snRNA-activating protein complex subunit 1 OS=Macaca fascicularis GN=SNAPC1 PE=2 SV=1//0
XM_007986913.1	5.8322	6.02E-10	6.50E-09	sp|Q8NCM2|KCNH5_HUMAN Potassium voltage-gated channel subfamily H member 5 OS=Homo sapiens GN=KCNH5 PE=1 SV=3//0
XM_007986956.1	-0.65032	2.17E-05	0.0001538	sp|P24588|AKAP5_HUMAN A-kinase anchor protein 5 OS=Homo sapiens GN=AKAP5 PE=1 SV=3//0
XM_007986964.1	-2.9574	0.00043	0.00259	sp|Q9Y2K1|ZBTB1_HUMAN Zinc finger and BTB domain-containing protein 1 OS=Homo sapiens GN=ZBTB1 PE=1 SV=3//0

XM_007986966.1	-1.0468	5.49E-12	6.94E-11	sp|P54652|HSP72_HUMAN Heat shock-related 70 kDa protein 2 OS=Homo sapiens GN=HSPA2 PE=1 SV=1//0
XM_007986974.1	1.6071	1.23E-80	1.59E-78	sp|A1L390|PKHG3_HUMAN Pleckstrin homology domain-containing family G member 3 OS=Homo sapiens GN=PLEKHG3 PE=1 SV=1//0
XM_007986976.1	-1.8201	0.0027075	0.014314	sp|Q4AEH9|GPX2_HYLLA Glutathione peroxidase 2 OS=Hylobates lar GN=GPX2 PE=2 SV=2//2.97456e-134
XM_007986987.1	-0.72418	0.0019806	0.010736	sp|P59190|RAB15_HUMAN Ras-related protein Rab-15 OS=Homo sapiens GN=RAB15 PE=1 SV=1//2.22022e-45
XM_007987059.1	-1.8837	0.0003065	0.0018774	sp|Q9NYT0|PLEK2_HUMAN Pleckstrin-2 OS=Homo sapiens GN=PLEK2 PE=1 SV=1//8.12222e-06
XM_007987080.1	0.39517	1.44E-05	0.0001039	sp|Q8TC12|RDH11_HUMAN Retinol dehydrogenase 11 OS=Homo sapiens GN=RDH11 PE=1 SV=2//0
XM_007987084.1	1.4312	0.0056473	0.02786	sp|Q68DK2|ZFY26_HUMAN Zinc finger FYVE domain-containing protein 26 OS=Homo sapiens GN=ZFYVE26 PE=1 SV=3//0
XM_007987093.1	1.0357	2.01E-42	9.44E-41	sp|Q07352|TISB_HUMAN Zinc finger protein 36, C3H1 type-like 1 OS=Homo sapiens GN=ZFP36L1 PE=1 SV=1//3.83506e-174
XM_007987114.1	-0.88671	1.75E-10	1.97E-09	sp|P84089|ERH_MOUSE Enhancer of rudimentary homolog OS=Mus musculus GN=Erh PE=1 SV=1//5.82762e-72
XM_007987118.1	0.36746	0.0033736	0.017481	sp|Q92537|SUSD6_HUMAN Sushi domain-containing protein 6 OS=Homo sapiens GN=SUSD6 PE=1 SV=1//2.12246e-176
XM_007987156.1	0.58671	1.83E-05	0.0001304	sp|P57105|SYJ2B_HUMAN Synaptojanin-2-binding protein OS=Homo sapiens GN=SYNJ2BP PE=1 SV=2//5.70457e-96
XM_007987157.1	0.92709	0.000171	0.0010872	sp|O75586|MED6_HUMAN Mediator of RNA polymerase II transcription subunit 6 OS=Homo sapiens GN=MED6 PE=1 SV=2//9.64666e-166
XM_007987162.1	0.6419	2.63E-18	4.79E-17	sp|Q96RV3|PCX1_HUMAN Pecanex-like protein 1 OS=Homo sapiens GN=PCNX PE=1 SV=2//0
XM_007987193.1	0.47573	0.0078873	0.037755	sp|Q9HBF4|ZFYV1_HUMAN Zinc finger FYVE domain-containing protein 1 OS=Homo sapiens GN=ZFYVE1 PE=1 SV=1//0
XM_007987220.1	-0.68445	1.44E-06	1.17E-05	sp|P49753|ACOT2_HUMAN Acyl-coenzyme A thioesterase 2, mitochondrial OS=Homo sapiens GN=ACOT2 PE=1 SV=6//0
XM_007987230.1	0.57795	4.40E-08	4.07E-07	sp|Q8ND90|PNMA1_HUMAN Paraneoplastic antigen Ma1 OS=Homo sapiens GN=PNMA1 PE=1 SV=2//0
XM_007987247.1	-1.1479	1.06E-14	1.58E-13	sp|Q02252|MMSA_HUMAN Methylmalonate-semialdehyde dehydrogenase [acylating], mitochondrial OS=Homo sapiens GN=ALDH6A1 PE=1 SV=2//0
XM_007987262.1	0.7995	0.0015743	0.0087187	sp|Q96MY7|F161B_HUMAN Protein FAM161B OS=Homo sapiens GN=FAM161B PE=1 SV=2//0
XM_007987272.1	-0.82559	6.97E-20	1.37E-18	sp|P61917|NPC2_PANTR Epididymal secretory protein E1 OS=Pan troglodytes GN=NPC2 PE=2 SV=1//4.5412e-92
XM_007987279.1	-1.4975	9.88E-05	0.0006491	sp|Q3B8N5|PROX2_HUMAN Prospero homeobox protein 2 OS=Homo sapiens GN=PROX2 PE=2 SV=3//0
XM_007987285.1	0.20638	0.0086888	0.041267	sp|P36957|ODO2_HUMAN Dihydrolipoyllysine-residue succinyltransferase component of 2-oxoglutarate dehydrogenase complex, mitochondrial OS=Homo sapiens GN=DLST PE=1 SV=4//0
XM_007987294.1	0.6358	7.10E-08	6.45E-07	sp|P49770|EI2BB_HUMAN Translation initiation factor eIF-2B subunit beta OS=Homo sapiens GN=EIF2B2 PE=1 SV=3//0
XM_007987321.1	6.8732	4.79E-58	3.52E-56	sp|P01100|FOS_HUMAN Proto-oncogene c-Fos OS=Homo sapiens GN=FOS PE=1 SV=1//0
XM_007987322.1	1.3485	4.92E-07	4.14E-06	sp|Q8WYK2|JDP2_HUMAN Jun dimerization protein 2 OS=Homo sapiens GN=JDP2 PE=1 SV=1//7.25098e-75
XM_007987349.1	-0.54183	6.85E-06	5.16E-05	sp|Q5R589|ERG28_PONAB Probable ergosterol biosynthetic protein 28 OS=Pongo abelii PE=2 SV=1//2.89312e-93
XM_007987350.1	0.73511	2.65E-09	2.71E-08	sp|P10600|TGFB3_HUMAN Transforming growth factor beta-3 OS=Homo sapiens GN=TGFB3 PE=1 SV=1//0
XM_007987359.1	0.80665	1.74E-10	1.97E-09	sp|Q7L8A9|VASH1_HUMAN Vasohibin-1 OS=Homo sapiens GN=VASH1 PE=1 SV=1//0
XM_007987378.1	0.9988	1.29E-27	3.62E-26	sp|Q8K3X4|I2BPL_MOUSE Interferon regulatory factor 2-binding protein-like OS=Mus musculus GN=Irf2bpl PE=1 SV=1//1.86156e-71
XM_007987397.1	0.74206	2.90E-13	3.96E-12	sp|Q9UKY4|POMT2_HUMAN Protein O-mannosyl-transferase 2 OS=Homo sapiens GN=POMT2 PE=1 SV=2//0
XM_007987398.1	-1.3985	3.35E-05	0.0002319	sp|Q95JY5|SAM15_MACFA Sterile alpha motif domain-containing protein 15 OS=Macaca fascicularis GN=SAMD15 PE=2 SV=2//0
XM_007987404.1	1.0348	2.69E-24	6.55E-23	sp|Q9NXG2|THUM1_HUMAN THUMP domain-containing protein 1 OS=Homo sapiens GN=THUMPD1 PE=1 SV=2//0
XM_007987406.1	-1.5347	1.29E-32	4.34E-31	sp|O95433|AHSA1_HUMAN Activator of 90 kDa heat shock protein ATPase homolog 1 OS=Homo sapiens GN=AHSA1 PE=1 SV=1//0
XM_007987415.1	-1.5144	1.81E-08	1.74E-07	sp|Q9GZT3|SLIRP_HUMAN SRA stem-loop-interacting RNA-binding protein, mitochondrial OS=Homo sapiens GN=SLIRP PE=1 SV=1//6.96244e-44
XM_007987495.1	-1.4	8.81E-11	1.01E-09	sp|Q8WXE9|STON2_HUMAN Stonin-2 OS=Homo sapiens GN=STON2 PE=1 SV=1//0
XM_007987496.1	-0.5187	9.49E-10	1.01E-08	sp|Q9UBV2|SE1L1_HUMAN Protein sel-1 homolog 1 OS=Homo sapiens GN=SEL1L PE=1 SV=3//0
XM_007987570.1	-0.49082	4.45E-08	4.10E-07	sp|P62193|PRS4_RAT 26S protease regulatory subunit 4 OS=Rattus

				norvegicus GN=Psmc1 PE=2 SV=1//0
XM_007987571.1	0.55998	5.31E-06	4.05E-05	sp|Q9H7Z3|NRDE2_HUMAN Protein NRDE2 homolog OS=Homo sapiens GN=NRDE2 PE=1 SV=3//0
XM_007987572.1	-1.3365	3.21E-58	2.38E-56	sp|P62155|CALM_XENLA Calmodulin OS=Xenopus laevis GN=calm1 PE=1 SV=2//1.78506e-10
XM_007987583.1	1.066	0.0001965	0.0012366	sp|Q6B0B8|TIGD3_HUMAN Tigger transposable element-derived protein 3 OS=Homo sapiens GN=TIGD3 PE=2 SV=1//0
XM_007987603.1	1.0995	1.14E-05	8.38E-05	sp|Q6IN85|P4R3A_HUMAN Serine/threonine-protein phosphatase 4 regulatory subunit 3A OS=Homo sapiens GN=SMEK1 PE=1 SV=1//0
XM_007987625.1	0.70548	0.0002198	0.0013726	sp|Q15643|TRIPB_HUMAN Thyroid receptor-interacting protein 11 OS=Homo sapiens GN=TRIP11 PE=1 SV=3//0
XM_007987629.1	-0.52964	1.36E-11	1.67E-10	sp|P22695|QCR2_HUMAN Cytochrome b-c1 complex subunit 2, mitochondrial OS=Homo sapiens GN=UQCRC2 PE=1 SV=3//0
XM_007987631.1	-1.5885	0.0021746	0.0117	sp|Q0MQC6|NDUB1_PANTR NADH dehydrogenase [ubiquinone] 1 beta subcomplex subunit 1 OS=Pan troglodytes GN=NDUFB1 PE=3 SV=1//8.5776e-33
XM_007987641.1	-0.46498	3.02E-10	3.35E-09	sp|Q4R4T8|LGMN_MACFA Legumain OS=Macaca fascicularis GN=LGMN PE=2 SV=1//0
XM_007987652.1	1.2156	4.79E-19	9.07E-18	sp|Q7SZ07|CP52B_XENLA Uncharacterized protein C16orf52 homolog B OS=Xenopus laevis PE=2 SV=1//3.80311e-104
XM_007987654.1	-0.26578	0.0024823	0.013229	sp|Q9P203|BTBD7_HUMAN BTB/POZ domain-containing protein 7 OS=Homo sapiens GN=BTBD7 PE=1 SV=3//0
XM_007987655.1	0.54272	0.006151	0.030128	sp|Q9P203|BTBD7_HUMAN BTB/POZ domain-containing protein 7 OS=Homo sapiens GN=BTBD7 PE=1 SV=3//0
XM_007987681.1	0.31667	1.73E-05	0.0001237	sp|Q5RDL2|DDX24_PONAB ATP-dependent RNA helicase DDX24 OS=Pongo abelii GN=DDX24 PE=2 SV=1//0
XM_007987696.1	0.84032	1.00E-29	3.06E-28	sp|P58268|REQU_CHICK Zinc finger protein ubi-d4 OS=Gallus gallus GN=REQ PE=2 SV=1//0
XM_007987697.1	-2.5725	6.55E-27	1.79E-25	sp|Q5R9E3|CBG_PONAB Corticosteroid-binding globulin OS=Pongo abelii GN=SERPINA6 PE=2 SV=1//0
XM_007987748.1	0.71572	1.07E-15	1.68E-14	sp|Q01850|CDR2_HUMAN Cerebellar degeneration-related protein 2 OS=Homo sapiens GN=CDR2 PE=1 SV=2//0
XM_007987761.1	4.6672	3.02E-11	3.62E-10	sp|P30411|BKRB2_HUMAN B2 bradykinin receptor OS=Homo sapiens GN=BDKRB2 PE=1 SV=2//0
XM_007987765.1	-5.4284	3.01E-08	2.82E-07	sp|O95976|IGSF6_HUMAN Immunoglobulin superfamily member 6 OS=Homo sapiens GN=IGSF6 PE=2 SV=2//9.20101e-161
XM_007987788.1	-1.8131	1.34E-14	1.98E-13	sp|Q9H1A3|METL9_HUMAN Methyltransferase-like protein 9 OS=Homo sapiens GN=METTL9 PE=2 SV=1//5.88189e-158
XM_007987790.1	1.9032	7.96E-30	2.43E-28	sp|O14613|BORG1_HUMAN Cdc42 effector protein 2 OS=Homo sapiens GN=CDC42EP2 PE=1 SV=1//2.30146e-113
XM_007987798.1	0.64968	7.73E-06	5.78E-05	sp|O75909|CCNK_HUMAN Cyclin-K OS=Homo sapiens GN=CCNK PE=1 SV=2//0
XM_007987815.1	0.83735	2.43E-07	2.10E-06	sp|Q9H9Y2|RPF1_HUMAN Ribosome production factor 1 OS=Homo sapiens GN=RPF1 PE=1 SV=2//0
XM_007987831.1	-2.1539	1.41E-05	0.0001023	sp|Q9UI08|EVL_HUMAN Ena/VASP-like protein OS=Homo sapiens GN=EVL PE=1 SV=2//0
XM_007987832.1	-1.1399	2.83E-05	0.0001977	sp|Q5R896|EVL_PONAB Ena/VASP-like protein OS=Pongo abelii GN=EVL PE=2 SV=1//0
XM_007987836.1	-1.6041	0.0012168	0.0068705	sp|Q8N8R3|MCATL_HUMAN Mitochondrial basic amino acids transporter OS=Homo sapiens GN=SLC25A29 PE=2 SV=2//1.8054e-12
XM_007987837.1	-1.8456	1.37E-10	1.56E-09	sp|Q8N8R3|MCATL_HUMAN Mitochondrial basic amino acids transporter OS=Homo sapiens GN=SLC25A29 PE=2 SV=2//2.08137e-12
XM_007987866.1	0.41039	0.0013841	0.0077454	sp|Q70CQ4|UBP31_HUMAN Ubiquitin carboxyl-terminal hydrolase 31 OS=Homo sapiens GN=USP31 PE=2 SV=2//1.27349e-85
XM_007987877.1	0.48467	2.98E-05	0.0002075	sp|P83436|COG7_HUMAN Conserved oligomeric Golgi complex subunit 7 OS=Homo sapiens GN=COG7 PE=1 SV=1//0
XM_007987883.1	1.5469	2.77E-09	2.83E-08	sp|Q8TBZ3|WDR20_HUMAN WD repeat-containing protein 20 OS=Homo sapiens GN=WDR20 PE=1 SV=2//0
XM_007987937.1	-0.50147	7.79E-07	6.46E-06	sp|Q78JW9|UBFD1_MOUSE Ubiquitin domain-containing protein UBFD1 OS=Mus musculus GN=Ubfd1 PE=1 SV=2//0
XM_007987993.1	-2.2801	0.0002635	0.0016284	sp|Q8N142|PURA1_HUMAN Adenylosuccinate synthetase isozyme 1 OS=Homo sapiens GN=ADSSL1 PE=1 SV=1//0
XM_007987997.1	#NAME?	0.0028648	0.015095	sp|P31749|AKT1_HUMAN RAC-alpha serine/threonine-protein kinase OS=Homo sapiens GN=AKT1 PE=1 SV=2//0
XM_007988015.1	-1.8908	1.97E-36	7.55E-35	sp|Q8IVF2|AHNK2_HUMAN Protein AHNAK2 OS=Homo sapiens GN=AHNAK2 PE=1 SV=2//0
XM_007988016.1	1.0838	0.0020667	0.011166	sp|Q96F83|CN079_HUMAN Uncharacterized protein C14orf79 OS=Homo sapiens GN=C14orf79 PE=2 SV=2//0
XM_007988045.1	0.25664	0.0020146	0.010906	sp|P53350|PLK1_HUMAN Serine/threonine-protein kinase PLK1 OS=Homo sapiens GN=PLK1 PE=1 SV=1//0
XM_007988057.1	0.3415	0.0012541	0.0070683	sp|Q9BTE1|DCTN5_HUMAN Dynactin subunit 5 OS=Homo sapiens GN=DCTN5 PE=1 SV=1//1.2172e-111

XM_007988059.1	-1.5998	2.68E-11	3.23E-10	sp|P50238|CRIP1_HUMAN Cysteine-rich protein 1 OS=Homo sapiens GN=CRIP1 PE=1 SV=3//9.09902e-50
XM_007988093.1	1.2693	0.0013082	0.0073489	sp|Q8N7A1|KLDC1_HUMAN Kelch domain-containing protein 1 OS=Homo sapiens GN=KLHDC1 PE=2 SV=2//0
XM_007988101.1	-1.0755	1.92E-31	6.22E-30	sp|Q15398|DLGP5_HUMAN Disks large-associated protein 5 OS=Homo sapiens GN=DLGAP5 PE=1 SV=2//1.18523e-85
XM_007988105.1	1.5434	1.67E-31	5.43E-30	sp|Q92623|TTC9A_HUMAN Tetratricopeptide repeat protein 9A OS=Homo sapiens GN=TTC9 PE=2 SV=3//2.76421e-105
XM_007988107.1	1.1085	1.38E-06	1.12E-05	sp|Q8CD94|LIN52_MOUSE Protein lin-52 homolog OS=Mus musculus GN=Lin52 PE=3 SV=1//9.15183e-47
XM_007988108.1	-0.57727	1.12E-06	9.12E-06	sp|O15033|AREL1_HUMAN Apoptosis-resistant E3 ubiquitin protein ligase 1 OS=Homo sapiens GN=AREL1 PE=1 SV=3//0
XM_007988109.1	0.95045	3.06E-07	2.61E-06	sp|Q9UHC1|MLH3_HUMAN DNA mismatch repair protein Mlh3 OS=Homo sapiens GN=MLH3 PE=1 SV=3//0
XM_007988111.1	1.5014	2.16E-74	2.40E-72	sp|O15270|SPTC2_HUMAN Serine palmitoyltransferase 2 OS=Homo sapiens GN=SPTLC2 PE=1 SV=1//0
XM_007988112.1	2.7457	2.69E-39	1.15E-37	sp|Q13686|ALKB1_HUMAN Alkylated DNA repair protein alkB homolog 1 OS=Homo sapiens GN=ALKBH1 PE=1 SV=2//0
XM_007988123.1	-0.89564	2.10E-05	0.0001492	sp|P12277|KCRB_HUMAN Creatine kinase B-type OS=Homo sapiens GN=CKB PE=1 SV=1//4.18481e-111
XM_007988124.1	0.72858	3.04E-18	5.53E-17	sp|Q5R581|KLC1_PONAB Kinesin light chain 1 OS=Pongo abelii GN=KLC1 PE=2 SV=3//0
XM_007988129.1	-0.2111	0.0086861	0.041263	sp|Q13330|MTA1_HUMAN Metastasis-associated protein MTA1 OS=Homo sapiens GN=MTA1 PE=1 SV=2//0
XM_007988140.1	0.67426	0.0038263	0.019586	sp|P22752|H2A1_MOUSE Histone H2A type 1 OS=Mus musculus GN=Hist1h2ab PE=1 SV=3//3.50594e-67
XM_007988143.1	-1.8102	0.0005391	0.0032093	sp|Q9Y577|TRI17_HUMAN E3 ubiquitin-protein ligase TRIM17 OS=Homo sapiens GN=TRIM17 PE=1 SV=1//0
XM_007988144.1	-4.6162	0.000259	0.0016026	sp|Q16695|H31T_HUMAN Histone H3.1t OS=Homo sapiens GN=HIST3H3 PE=1 SV=3//1.94661e-83
XM_007988206.1	1.031	1.17E-16	1.96E-15	sp|Q9BU76|MMTA2_HUMAN Multiple myeloma tumor-associated protein 2 OS=Homo sapiens GN=MMTAG2 PE=1 SV=1//5.13316e-109
XM_007988211.1	-2.2199	1.86E-06	1.49E-05	sp|P56704|WNT3A_HUMAN Protein Wnt-3a OS=Homo sapiens GN=WNT3A PE=1 SV=2//3.69413e-164
XM_007988212.1	-4.0268	0.0063882	0.031144	sp|O14904|WNT9A_HUMAN Protein Wnt-9a OS=Homo sapiens GN=WNT9A PE=2 SV=2//0
XM_007988221.1	0.31721	0.0092199	0.043516	sp|Q5SQN1|SNP47_HUMAN Synaptosomal-associated protein 47 OS=Homo sapiens GN=SNAP47 PE=1 SV=3//0
XM_007988265.1	-0.33001	6.79E-06	5.12E-05	sp|P09874|PARP1_HUMAN Poly [ADP-ribose] polymerase 1 OS=Homo sapiens GN=PARP1 PE=1 SV=4//0
XM_007988274.1	1.4158	1.61E-44	8.14E-43	sp|Q6IQ49|SDE2_HUMAN Protein SDE2 homolog OS=Homo sapiens GN=SDE2 PE=1 SV=1//0
XM_007988280.1	-3.2087	1.04E-11	1.28E-10	sp|O00292|LFTY2_HUMAN Left-right determination factor 2 OS=Homo sapiens GN=LEFTY2 PE=1 SV=2//0
XM_007988281.1	-2.522	0.0006356	0.0037401	sp|P07099|HYEP_HUMAN Epoxide hydrolase 1 OS=Homo sapiens GN=EPHX1 PE=1 SV=1//0
XM_007988289.1	-2.5749	0.0055739	0.02756	sp|O94886|CSCL1_HUMAN CSC1-like protein 1 OS=Homo sapiens GN=TMEM63A PE=1 SV=3//0
XM_007988315.1	1.3042	2.42E-17	4.20E-16	sp|Q63HK3|ZKSC2_HUMAN Zinc finger protein with KRAB and SCAN domains 2 OS=Homo sapiens GN=ZKSCAN2 PE=1 SV=2//0
XM_007988341.1	0.30231	0.0008164	0.0047256	sp|O15121|DEGS1_HUMAN Sphingolipid delta(4)-desaturase DES1 OS=Homo sapiens GN=DEGS1 PE=1 SV=1//0
XM_007988342.1	1.7436	8.31E-77	9.85E-75	sp|Q9NVF7|FBX28_HUMAN F-box only protein 28 OS=Homo sapiens GN=FBXO28 PE=1 SV=1//0
XM_007988365.1	-3.4065	0.0025781	0.013683	sp|Q96F81|DISP1_HUMAN Protein dispatched homolog 1 OS=Homo sapiens GN=DISP1 PE=1 SV=3//0
XM_007988369.1	-0.51712	1.51E-06	1.22E-05	sp|Q5RAV3|AIDA_PONAB Axin interactor, dorsalization-associated protein OS=Pongo abelii GN=AIDA PE=2 SV=1//0
XM_007988390.1	2.636	4.00E-41	1.80E-39	sp|Q14774|HLX_HUMAN H2.0-like homeobox protein OS=Homo sapiens GN=HLX PE=1 SV=3//0
XM_007988391.1	-0.43814	0.0036296	0.018667	sp|Q9GKW0|MARC2_MACFA Mitochondrial amidoxime reducing component 2 OS=Macaca fascicularis GN=MARC2 PE=2 SV=1//0
XM_007988392.1	-0.91814	2.29E-13	3.15E-12	sp|Q9H7X2|CA115_HUMAN Uncharacterized protein C1orf115 OS=Homo sapiens GN=C1orf115 PE=2 SV=1//7.61034e-32
XM_007988398.1	1.0125	8.92E-06	6.63E-05	sp|Q8N371|KDM8_HUMAN Lysine-specific demethylase 8 OS=Homo sapiens GN=KDM8 PE=1 SV=1//0
XM_007988420.1	5.5246	1.69E-90	2.65E-88	sp|Q9Y3B9|RRP15_HUMAN RRP15-like protein OS=Homo sapiens GN=RRP15 PE=1 SV=2//8.3571e-158
XM_007988456.1	-0.18214	0.0091294	0.043126	sp|P49454|CENPF_HUMAN Centromere protein F OS=Homo sapiens GN=CENPF PE=1 SV=2//0
XM_007988457.1	0.51789	4.53E-11	5.34E-10	sp|Q15678|PTN14_HUMAN Tyrosine-protein phosphatase non-receptor type 14

				OS=Homo sapiens GN=PTPN14 PE=1 SV=2//0
XM_007988480.1	-1.3035	3.52E-11	4.20E-10	sp|Q86V25|VASH2_HUMAN Vasohibin-2 OS=Homo sapiens GN=VASH2 PE=1 SV=2//0
XM_007988481.1	1.9071	2.37E-15	3.63E-14	sp|Q5VTE6|ANGE2_HUMAN Protein angel homolog 2 OS=Homo sapiens GN=ANGEL2 PE=2 SV=1//0
XM_007988489.1	0.63706	0.0003962	0.002399	sp|Q9Y5Y0|FLVC1_HUMAN Feline leukemia virus subgroup C receptor-related protein 1 OS=Homo sapiens GN=FLVCR1 PE=1 SV=1//0
XM_007988490.1	-0.47982	0.0098596	0.046256	sp|Q96IY1|NSL1_HUMAN Kinetochore-associated protein NSL1 homolog OS=Homo sapiens GN=NSL1 PE=1 SV=3//0
XM_007988499.1	4.822	0.0015657	0.0086754	sp|Q95KZ1|FA71A_MACFA Protein FAM71A OS=Macaca fascicularis GN=FAM71A PE=2 SV=1//0
XM_007988501.1	8.3136	0	0	sp|P18847|ATF3_HUMAN Cyclic AMP-dependent transcription factor ATF-3 OS=Homo sapiens GN=ATF3 PE=1 SV=2//3.77845e-94
XM_007988502.1	-1.2741	1.38E-10	1.57E-09	sp|Q9UMX5|NENF_HUMAN Neudesin OS=Homo sapiens GN=NENF PE=1 SV=1//1.51082e-97
XM_007988506.1	0.85338	5.22E-18	9.36E-17	sp|Q15172|2A5A_HUMAN Serine/threonine-protein phosphatase 2A 56 kDa regulatory subunit alpha isoform OS=Homo sapiens GN=PPP2R5A PE=1 SV=1//0
XM_007988509.1	2.2826	1.13E-86	1.67E-84	sp|Q9NZJ0|DTL_HUMAN Denticleless protein homolog OS=Homo sapiens GN=DTL PE=1 SV=3//0
XM_007988514.1	-0.6236	1.09E-05	8.00E-05	sp|Q92604|LGAT1_HUMAN Acyl-CoA:lysophosphatidylglycerol acyltransferase 1 OS=Homo sapiens GN=LPGAT1 PE=1 SV=1//0
XM_007988520.1	1.6525	4.35E-73	4.75E-71	sp|Q4R6K2|ZNT1_MACFA Zinc transporter 1 OS=Macaca fascicularis GN=SLC30A1 PE=2 SV=1//0
XM_007988578.1	4.1001	3.58E-05	0.0002472	sp|Q68CQ4|DIEXF_HUMAN Digestive organ expansion factor homolog OS=Homo sapiens GN=DIEXF PE=1 SV=2//0
XM_007988590.1	1.6282	3.07E-11	3.67E-10	sp|Q5REJ0|CA074_PONAB UPF0739 protein C1orf74 homolog OS=Pongo abelii PE=2 SV=1//2.61276e-157
XM_007988648.1	0.67473	0.0008726	0.0050266	sp|O60825|F262_HUMAN 6-phosphofructo-2-kinase/fructose-2,6-bisphosphatase 2 OS=Homo sapiens GN=PFKFB2 PE=1 SV=2//0
XM_007988650.1	2.1379	1.87E-52	1.21E-50	sp|Q5VVQ6|OTU1_HUMAN Ubiquitin thioesterase OTU1 OS=Homo sapiens GN=YOD1 PE=1 SV=1//0
XM_007988703.1	3.3902	0.0072168	0.034804	sp|Q7LBE3|S26A9_HUMAN Solute carrier family 26 member 9 OS=Homo sapiens GN=SLC26A9 PE=1 SV=1//0
XM_007988707.1	1.0049	9.66E-23	2.22E-21	sp|Q5R839|S41A1_PONAB Solute carrier family 41 member 1 OS=Pongo abelii GN=SLC41A1 PE=2 SV=1//0
XM_007988717.1	-1.4879	7.24E-85	1.03E-82	sp|Q9H1E3|NUCKS_HUMAN Nuclear ubiquitous casein and cyclin-dependent kinase substrate 1 OS=Homo sapiens GN=NUCKS1 PE=1 SV=1//8.81528e-41
XM_007988729.1	-0.94141	0.0063288	0.030895	sp|Q8N468|MFSD4_HUMAN Major facilitator superfamily domain-containing protein 4 OS=Homo sapiens GN=MFSD4 PE=2 SV=3//0
XM_007988731.1	-1.2054	7.32E-11	8.48E-10	sp|Q5RD01|CDK18_PONAB Cyclin-dependent kinase 18 OS=Pongo abelii GN=CDK18 PE=2 SV=2//0
XM_007988753.1	2.7406	2.60E-293	2.48E-290	sp|Q9H093|NUAK2_HUMAN NUAK family SNF1-like kinase 2 OS=Homo sapiens GN=NUAK2 PE=1 SV=1//0
XM_007988803.1	-1.1302	1.81E-08	1.74E-07	sp|O00750|P3C2B_HUMAN Phosphatidylinositol 4-phosphate 3-kinase C2 domain-containing subunit beta OS=Homo sapiens GN=PIK3C2B PE=1 SV=2//0
XM_007988806.1	1.6967	3.16E-76	3.69E-74	sp|Q5SWA1|PR15B_HUMAN Protein phosphatase 1 regulatory subunit 15B OS=Homo sapiens GN=PPP1R15B PE=1 SV=1//0
XM_007988856.1	-1.6178	0.0030106	0.015783	sp|O75152|ZC11A_HUMAN Zinc finger CCCH domain-containing protein 11A OS=Homo sapiens GN=ZC3H11A PE=1 SV=3//0
XM_007988885.1	1.2732	1.24E-21	2.67E-20	sp|P78543|BTG2_HUMAN Protein BTG2 OS=Homo sapiens GN=BTG2 PE=1 SV=1//1.79864e-90
XM_007988912.1	0.75957	4.16E-15	6.30E-14	sp|Q53G59|KLH12_HUMAN Kelch-like protein 12 OS=Homo sapiens GN=KLHL12 PE=1 SV=2//0
XM_007988917.1	0.74684	1.76E-18	3.23E-17	sp|Q9UGL1|KDM5B_HUMAN Lysine-specific demethylase 5B OS=Homo sapiens GN=KDM5B PE=1 SV=3//0
XM_007988954.1	-1.1685	7.56E-26	2.00E-24	sp|Q9H4A4|AMPB_HUMAN Aminopeptidase B OS=Homo sapiens GN=RNPEP PE=1 SV=2//0
XM_007988956.1	-1.1811	0.0012437	0.0070152	sp|P29536|LMOD1_HUMAN Leiomodin-1 OS=Homo sapiens GN=LMOD1 PE=1 SV=3//0
XM_007988958.1	-1.2876	4.99E-05	0.0003387	sp|Q96DD7|SHSA4_HUMAN Protein shisa-4 OS=Homo sapiens GN=SHISA4 PE=2 SV=3//1.72955e-93
XM_007989035.1	-0.94679	1.30E-34	4.75E-33	sp|P18669|PGAM1_HUMAN Phosphoglycerate mutase 1 OS=Homo sapiens GN=PGAM1 PE=1 SV=2//7.75524e-176
XM_007989042.1	-1.6201	5.15E-12	6.52E-11	sp|Q6P823|H33_XENTR Histone H3.3 OS=Xenopus tropicalis GN=TGas113e22.1 PE=1 SV=3//2.65897e-90
XM_007989127.1	0.44621	0.0055771	0.027564	sp|P41220|RGS2_HUMAN Regulator of G-protein signaling 2 OS=Homo sapiens GN=RGS2 PE=1 SV=1//5.87149e-137
XM_007989140.1	-0.75782	0.0042093	0.021367	sp|Q9R050|SSBP3_RAT Single-stranded DNA-binding protein 3 OS=Rattus norvegicus GN=Ssbp3 PE=2 SV=1//4.08188e-121
XM_007989180.1	0.35559	1.75E-05	0.0001256	sp|P23284|PPIB_HUMAN Peptidyl-prolyl cis-trans isomerase B OS=Homo sapiens GN=PPIB PE=1 SV=2//1.32285e-139

XM_007989207.1	-0.89482	2.52E-06	1.99E-05	sp|Q9H246|CA021_HUMAN Uncharacterized protein C1orf21 OS=Homo sapiens GN=C1orf21 PE=1 SV=1//7.67373e-76
XM_007989213.1	0.31287	0.0078128	0.037439	sp|Q8WW01|SEN15_HUMAN tRNA-splicing endonuclease subunit Sen15 OS=Homo sapiens GN=TSEN15 PE=1 SV=1//1.78594e-101
XM_007989219.1	-0.80665	9.53E-19	1.78E-17	sp|Q9CPW4|ARPC5_MOUSE Actin-related protein 2/3 complex subunit 5 OS=Mus musculus GN=Arpc5 PE=2 SV=3//2.10523e-99
XM_007989224.1	-0.57439	0.0042669	0.02164	sp|Q9H3K6|BOLA2_HUMAN BolA-like protein 2 OS=Homo sapiens GN=BOLA2 PE=1 SV=1//2.47013e-52
XM_007989239.1	-1.6078	3.48E-08	3.24E-07	sp|Q9BQ83|SLX1_HUMAN Structure-specific endonuclease subunit SLX1 OS=Homo sapiens GN=SLX1A PE=1 SV=1//3.26007e-171
XM_007989245.1	-0.22377	0.0012921	0.0072659	sp|P11047|LAMC1_HUMAN Laminin subunit gamma-1 OS=Homo sapiens GN=LAMC1 PE=1 SV=3//0
XM_007989248.1	0.94136	3.65E-23	8.52E-22	sp|Q5R874|DHX9_PONAB ATP-dependent RNA helicase A OS=Pongo abelii GN=DHX9 PE=2 SV=1//0
XM_007989250.1	-1.8004	9.29E-05	0.0006126	sp|P0DMN0|ST1A4_HUMAN Sulfotransferase 1A4 OS=Homo sapiens GN=SULT1A4 PE=1 SV=1//2.24107e-174
XM_007989285.1	-2.0237	0.000422	0.0025471	sp|P15104|GLNA_HUMAN Glutamine synthetase OS=Homo sapiens GN=GLUL PE=1 SV=4//0
XM_007989298.1	1.5929	2.39E-31	7.69E-30	sp|Q5VY09|IER5_HUMAN Immediate early response gene 5 protein OS=Homo sapiens GN=IER5 PE=2 SV=3//4.46601e-101
XM_007989303.1	0.61188	0.0001876	0.0011845	sp|Q5R6Q2|STX6_PONAB Syntaxin-6 OS=Pongo abelii GN=STX6 PE=2 SV=1//1.89076e-149
XM_007989307.1	-0.50304	9.29E-11	1.06E-09	sp|Q9UBH6|XPR1_HUMAN Xenotropic and polytropic retrovirus receptor 1 OS=Homo sapiens GN=XPR1 PE=1 SV=1//0
XM_007989309.1	0.27786	0.0069046	0.033416	sp|Q9BR61|ACBD6_HUMAN Acyl-CoA-binding domain-containing protein 6 OS=Homo sapiens GN=ACBD6 PE=1 SV=1//0
XM_007989311.1	-1.5869	2.69E-05	0.000188	sp|O00391|QSOX1_HUMAN Sulfhydryl oxidase 1 OS=Homo sapiens GN=QSOX1 PE=1 SV=3//0
XM_007989327.1	-0.62479	3.56E-07	3.03E-06	sp|Q8NFQ8|TOIP2_HUMAN Torsin-1A-interacting protein 2 OS=Homo sapiens GN=TOR1AIP2 PE=1 SV=1//0
XM_007989375.1	1.7787	1.34E-53	8.86E-52	-//-
XM_007989419.1	-0.97716	2.60E-11	3.13E-10	sp|O60783|RT14_HUMAN 28S ribosomal protein S14, mitochondrial OS=Homo sapiens GN=MRPS14 PE=1 SV=1//4.07001e-86
XM_007989435.1	0.28989	0.0078608	0.037645	sp|Q5TC82|RC3H1_HUMAN Roquin-1 OS=Homo sapiens GN=RC3H1 PE=1 SV=1//0
XM_007989452.1	-0.34116	0.0015245	0.0084639	sp|Q6PI48|SYDM_HUMAN Aspartate--tRNA ligase, mitochondrial OS=Homo sapiens GN=DARS2 PE=1 SV=1//0
XM_007989487.1	-0.41993	4.63E-07	3.90E-06	sp|Q2PFL9|PRDX6_MACFA Peroxiredoxin-6 OS=Macaca fascicularis GN=PRDX6 PE=2 SV=3//1.26771e-155
XM_007989488.1	1.4672	0.0016111	0.0088967	sp|Q9UNG2|TNF18_HUMAN Tumor necrosis factor ligand superfamily member 18 OS=Homo sapiens GN=TNFSF18 PE=1 SV=2//7.08579e-133
XM_007989504.1	#NAME?	0.0045006	0.022709	sp|P04075|ALDOA_HUMAN Fructose-bisphosphate aldolase A OS=Homo sapiens GN=ALDOA PE=1 SV=2//0
XM_007989514.1	-2.1794	0.0003637	0.0022115	sp|P04075|ALDOA_HUMAN Fructose-bisphosphate aldolase A OS=Homo sapiens GN=ALDOA PE=1 SV=2//0
XM_007989545.1	1.2418	1.92E-15	2.96E-14	sp|Q5T7V8|GORAB_HUMAN RAB6-interacting golgin OS=Homo sapiens GN=GORAB PE=1 SV=1//0
XM_007989585.1	-1.8595	0.0009291	0.0053297	sp|Q4R6Q9|CC181_MACFA Coiled-coil domain-containing protein 181 OS=Macaca fascicularis GN=CCDC181 PE=2 SV=1//0
XM_007989601.1	1.7228	7.86E-25	1.98E-23	sp|Q9H2G9|GO45_HUMAN Golgin-45 OS=Homo sapiens GN=BLZF1 PE=1 SV=2//0
XM_007989617.1	0.43207	6.76E-05	0.0004528	sp|O75663|TIPRL_HUMAN TIP41-like protein OS=Homo sapiens GN=TIPRL PE=1 SV=2//1.59744e-173
XM_007989643.1	-1.4993	2.05E-14	2.99E-13	-//-
XM_007989675.1	1.4072	0.0001383	0.0008895	sp|Q71H61|ILDR2_HUMAN Immunoglobulin-like domain-containing receptor 2 OS=Homo sapiens GN=ILDR2 PE=2 SV=1//0
XM_007989684.1	0.79915	2.42E-12	3.11E-11	sp|Q5RDB9|TADA1_PONAB Transcriptional adapter 1 OS=Pongo abelii GN=TADA1 PE=2 SV=1//0
XM_007989692.1	1.4208	1.15E-65	1.07E-63	sp|Q9BZX2|UCK2_HUMAN Uridine-cytidine kinase 2 OS=Homo sapiens GN=UCK2 PE=1 SV=1//3.42755e-174
XM_007989693.1	-0.3298	0.0005938	0.0035065	sp|P49189|AL9A1_HUMAN 4-trimethylaminobutyraldehyde dehydrogenase OS=Homo sapiens GN=ALDH9A1 PE=1 SV=3//0
XM_007989696.1	-1.8088	1.17E-39	5.06E-38	sp|O14880|MGST3_HUMAN Microsomal glutathione S-transferase 3 OS=Homo sapiens GN=MGST3 PE=1 SV=1//6.28297e-104
XM_007989698.1	-1.7186	0.0054978	0.027202	sp|Q5U4P2|ASPH1_HUMAN Aspartate beta-hydroxylase domain-containing protein 1 OS=Homo sapiens GN=ASPHD1 PE=1 SV=3//1.22255e-177
XM_007989722.1	-1.3991	2.36E-51	1.49E-49	sp|O15539|RGS5_HUMAN Regulator of G-protein signaling 5 OS=Homo sapiens GN=RGS5 PE=1 SV=1//1.28657e-99
XM_007989723.1	1.9252	0.0001229	0.0007971	sp|Q4R525|RGS4_MACFA Regulator of G-protein signaling 4 OS=Macaca fascicularis GN=RGS4 PE=2 SV=1//4.16904e-133
XM_007989735.1	1.7178	2.04E-53	1.34E-51	sp|Q7L0Q8|RHOU_HUMAN Rho-related GTP-binding protein RhoU OS=Homo sapiens GN=RHOU PE=1 SV=1//3.2577e-142

XM_007989740.1	0.98082	1.83E-12	2.38E-11	sp|Q6IQ19|CCSAP_HUMAN Centriole, cilia and spindle-associated protein OS=Homo sapiens GN=CCSAP PE=1 SV=2//5.93844e-138
XM_007989766.1	0.88529	8.47E-14	1.20E-12	sp|Q14746|COG2_HUMAN Conserved oligomeric Golgi complex subunit 2 OS=Homo sapiens GN=COG2 PE=1 SV=1//0
XM_007989767.1	-3.2029	0.0070785	0.03419	sp|Q9GLN8|ANGT_PANTR Angiotensinogen OS=Pan troglodytes GN=AGT PE=2 SV=1//0
XM_007989769.1	1.1048	7.97E-18	1.41E-16	sp|Q9H425|CA198_HUMAN Uncharacterized protein C1orf198 OS=Homo sapiens GN=C1orf198 PE=1 SV=1//0
XM_007989770.1	-0.81859	3.99E-05	0.0002733	sp|Q9H2C2|ARV1_HUMAN Protein ARV1 OS=Homo sapiens GN=ARV1 PE=2 SV=1//9.11548e-173
XM_007989792.1	-0.94225	1.19E-24	2.97E-23	sp|Q14764|MVP_HUMAN Major vault protein OS=Homo sapiens GN=MVP PE=1 SV=4//0
XM_007989794.1	1.0461	5.41E-16	8.67E-15	sp|Q8IYI6|EXOC8_HUMAN Exocyst complex component 8 OS=Homo sapiens GN=EXOC8 PE=1 SV=2//0
XM_007989798.1	0.70201	6.44E-11	7.49E-10	sp|Q4R599|TSNAX_MACFA Translin-associated protein X OS=Macaca fascicularis GN=TSNAX PE=2 SV=1//0
XM_007989818.1	-0.60695	2.75E-18	5.01E-17	sp|P55812|UBIM_PIG Ubiquitin-like protein FUBI OS=Sus scrofa GN=FAU PE=3 SV=1//3.18591e-43
XM_007989849.1	0.47716	1.46E-05	0.0001052	sp|O95749|GGPPS_HUMAN Geranylgeranyl pyrophosphate synthase OS=Homo sapiens GN=GGPS1 PE=1 SV=1//0
XM_007989851.1	-3.514	0.0047191	0.023707	sp|Q8NCR0|B3GL2_HUMAN UDP-GalNAc:beta-1,3-N-acetylgalactosaminyltransferase 2 OS=Homo sapiens GN=B3GALNT2 PE=1 SV=1//8.13175e-164
XM_007989871.1	0.98921	0.0002817	0.0017347	sp|Q99698|LYST_HUMAN Lysosomal-trafficking regulator OS=Homo sapiens GN=LYST PE=1 SV=3//0
XM_007989874.1	-1.7787	1.33E-120	3.30E-118	sp|P14543|NID1_HUMAN Nidogen-1 OS=Homo sapiens GN=NID1 PE=1 SV=3//0
XM_007989876.1	-0.4939	0.0018068	0.0098764	sp|O60478|G137B_HUMAN Integral membrane protein GPR137B OS=Homo sapiens GN=GPR137B PE=2 SV=1//0
XM_007989877.1	0.47021	0.0070832	0.034205	sp|Q86YB8|ERO1B_HUMAN ERO1-like protein beta OS=Homo sapiens GN=ERO1LB PE=1 SV=2//0
XM_007989879.1	-1.5845	1.17E-05	8.59E-05	sp|Q8WWZ3|EDAD_HUMAN Ectodysplasin-A receptor-associated adapter protein OS=Homo sapiens GN=EDARADD PE=1 SV=3//2.40068e-132
XM_007989918.1	0.67319	0.010736	0.049943	sp|Q9UHR6|ZNHI2_HUMAN Zinc finger HIT domain-containing protein 2 OS=Homo sapiens GN=ZNHIT2 PE=1 SV=1//0
XM_007989925.1	2.0647	0.0024506	0.013076	sp|Q9H772|GREM2_HUMAN Gremlin-2 OS=Homo sapiens GN=GREM2 PE=2 SV=1//6.12153e-97
XM_007989935.1	-0.29583	0.003433	0.017739	sp|Q60HF9|FUMH_MACFA Fumarate hydratase, mitochondrial OS=Macaca fascicularis GN=FH PE=2 SV=1//0
XM_007989949.1	-1.2644	1.00E-05	7.38E-05	sp|Q9BXW4|MLP3C_HUMAN Microtubule-associated proteins 1A/1B light chain 3C OS=Homo sapiens GN=MAP1LC3C PE=1 SV=1//6.54901e-94
XM_007989981.1	#NAME?	0.0063443	0.03095	sp|Q5SW79|CE170_HUMAN Centrosomal protein of 170 kDa OS=Homo sapiens GN=CEP170 PE=1 SV=1//0
XM_007989985.1	1.1221	0.0051746	0.025754	sp|Q99592|ZBT18_HUMAN Zinc finger and BTB domain-containing protein 18 OS=Homo sapiens GN=ZBTB18 PE=1 SV=1//0
XM_007990065.1	0.79245	1.29E-13	1.81E-12	sp|Q99611|SPS2_HUMAN Selenide, water dikinase 2 OS=Homo sapiens GN=SEPHS2 PE=1 SV=3//0
XM_007990077.1	0.88164	5.33E-10	5.79E-09	sp|Q9H5H4|ZN768_HUMAN Zinc finger protein 768 OS=Homo sapiens GN=ZNF768 PE=1 SV=2//0
XM_007990107.1	1.6388	3.04E-25	7.86E-24	sp|Q96CS4|ZN689_HUMAN Zinc finger protein 689 OS=Homo sapiens GN=ZNF689 PE=2 SV=1//0
XM_007990155.1	-1.6554	8.96E-37	3.48E-35	sp|Q6P823|H33_XENTR Histone H3.3 OS=Xenopus tropicalis GN=TGas113e22.1 PE=1 SV=3//1.70347e-75
XM_007990157.1	-1.1868	5.91E-22	1.30E-20	sp|Q14739|LBR_HUMAN Lamin-B receptor OS=Homo sapiens GN=LBR PE=1 SV=2//0
XM_007990158.1	0.59268	6.61E-15	9.93E-14	sp|Q9H7D7|WDR26_HUMAN WD repeat-containing protein 26 OS=Homo sapiens GN=WDR26 PE=1 SV=3//0
XM_007990160.1	-0.38039	5.99E-05	0.0004028	sp|Q9NSE4|SYIM_HUMAN Isoleucine--tRNA ligase, mitochondrial OS=Homo sapiens GN=IARS2 PE=1 SV=2//0
XM_007990169.1	-2.1355	7.83E-14	1.11E-12	sp|P70289|PTPRV_MOUSE Receptor-type tyrosine-protein phosphatase V OS=Mus musculus GN=Ptprv PE=2 SV=1//3.40742e-06
XM_007990176.1	0.61299	2.37E-17	4.11E-16	sp|Q9Y520|PRC2C_HUMAN Protein PRRC2C OS=Homo sapiens GN=PRRC2C PE=1 SV=4//3.30855e-36
XM_007990181.1	0.95254	1.02E-40	4.54E-39	sp|Q8WYP5|ELYS_HUMAN Protein ELYS OS=Homo sapiens GN=AHCTF1 PE=1 SV=3//0
XM_007990227.1	0.70267	2.57E-12	3.31E-11	sp|Q9H0C5|BTBD1_HUMAN BTB/POZ domain-containing protein 1 OS=Homo sapiens GN=BTBD1 PE=1 SV=1//0
XM_007990230.1	1.7052	8.33E-16	1.32E-14	sp|Q8WUR7|CO040_HUMAN UPF0235 protein C15orf40 OS=Homo sapiens GN=C15orf40 PE=1 SV=2//6.5578e-76
XM_007990241.1	2.7747	1.96E-98	3.52E-96	sp|Q8TF30|WHAMM_HUMAN WASP homolog-associated protein with actin, membranes and microtubules OS=Homo sapiens GN=WHAMM PE=1 SV=2//0

XM_007990245.1	0.37002	0.0072379	0.03489	sp|Q9UEG4|ZN629_HUMAN Zinc finger protein 629 OS=Homo sapiens GN=ZNF629 PE=1 SV=2//0
XM_007990265.1	0.64111	7.84E-06	5.86E-05	sp|Q92610|ZN592_HUMAN Zinc finger protein 592 OS=Homo sapiens GN=ZNF592 PE=1 SV=2//0
XM_007990268.1	-3.6451	0.000665	0.0038953	sp|Q8WUZ0|BCL7C_HUMAN B-cell CLL/lymphoma 7 protein family member C OS=Homo sapiens GN=BCL7C PE=1 SV=3//3.17329e-72
XM_007990279.1	1.2988	5.55E-17	9.41E-16	sp|Q6P4I2|WDR73_HUMAN WD repeat-containing protein 73 OS=Homo sapiens GN=WDR73 PE=1 SV=1//0
XM_007990296.1	1.8978	0.0053989	0.026771	sp|Q12802|AKP13_HUMAN A-kinase anchor protein 13 OS=Homo sapiens GN=AKAP13 PE=1 SV=2//0
XM_007990297.1	-1.0967	1.28E-18	2.38E-17	sp|P62425|RL7A_RAT 60S ribosomal protein L7a OS=Rattus norvegicus GN=Rpl7a PE=1 SV=2//6.01933e-161
XM_007990326.1	3.9524	3.99E-82	5.33E-80	sp|Q5REE2|AEN_PONAB Apoptosis-enhancing nuclease OS=Pongo abelii GN=AEN PE=2 SV=1//0
XM_007990341.1	-0.89676	3.15E-07	2.70E-06	sp|Q4R2Y9|ABHD2_MACFA Abhydrolase domain-containing protein 2 OS=Macaca fascicularis GN=ABHD2 PE=2 SV=1//0
XM_007990382.1	-1.7502	0.0023619	0.01264	sp|Q9H2F3|3BHS7_HUMAN 3 beta-hydroxysteroid dehydrogenase type 7 OS=Homo sapiens GN=HSD3B7 PE=1 SV=2//0
XM_007990386.1	0.60801	0.000217	0.0013577	sp|Q5RDP9|AP3S2_PONAB AP-3 complex subunit sigma-2 OS=Pongo abelii GN=AP3S2 PE=2 SV=1//3.01873e-130
XM_007990400.1	-0.71466	9.56E-07	7.85E-06	sp|Q99828|CIB1_HUMAN Calcium and integrin-binding protein 1 OS=Homo sapiens GN=CIB1 PE=1 SV=4//6.57534e-115
XM_007990401.1	0.77755	2.41E-12	3.11E-11	sp|Q5R7E7|NGRN_PONAB Neugrin OS=Pongo abelii GN=NGRN PE=2 SV=2//8.54488e-170
XM_007990469.1	-0.19594	0.0052124	0.025913	sp|P46940|IQGA1_HUMAN Ras GTPase-activating-like protein IQGAP1 OS=Homo sapiens GN=IQGAP1 PE=1 SV=1//0
XM_007990473.1	1.2331	3.15E-06	2.45E-05	sp|Q6NX45|ZN774_HUMAN Zinc finger protein 774 OS=Homo sapiens GN=ZNF774 PE=1 SV=2//0
XM_007990493.1	1.1556	1.12E-09	1.18E-08	-//-
XM_007990511.1	-1.5883	0.0044613	0.022516	sp|Q6DN12|MCTP2_HUMAN Multiple C2 and transmembrane domain-containing protein 2 OS=Homo sapiens GN=MCTP2 PE=1 SV=3//3.50167e-30
XM_007990512.1	0.71962	1.88E-12	2.44E-11	sp|Q96FF9|CDCA5_HUMAN Sororin OS=Homo sapiens GN=CDCA5 PE=1 SV=1//3.10624e-134
XM_007990516.1	-0.69732	8.57E-06	6.38E-05	sp|O09018|COT2_RAT COUP transcription factor 2 OS=Rattus norvegicus GN=Nr2f2 PE=1 SV=1//0
XM_007990517.1	-2.4211	0.0001681	0.0010698	sp|P43135|COT2_MOUSE COUP transcription factor 2 OS=Mus musculus GN=Nr2f2 PE=1 SV=2//0
XM_007990524.1	3.051	8.20E-87	1.21E-84	sp|Q8NCT1|ARRD4_HUMAN Arrestin domain-containing protein 4 OS=Homo sapiens GN=ARRDC4 PE=2 SV=3//0
XM_007990538.1	0.81719	5.67E-14	8.09E-13	sp|O15015|ZN646_HUMAN Zinc finger protein 646 OS=Homo sapiens GN=ZNF646 PE=1 SV=1//0
XM_007990571.1	0.82187	5.78E-07	4.84E-06	sp|Q9H672|ASB7_HUMAN Ankyrin repeat and SOCS box protein 7 OS=Homo sapiens GN=ASB7 PE=1 SV=2//0
XM_007990584.1	1.4021	1.08E-19	2.12E-18	sp|Q9BQE4|SELS_HUMAN Selenoprotein S OS=Homo sapiens GN=VIMP PE=1 SV=3//1.33152e-79
XM_007990586.1	-0.81042	6.13E-17	1.04E-15	sp|O14874|BCKD_HUMAN [3-methyl-2-oxobutanoate dehydrogenase [lipoamide]] kinase, mitochondrial OS=Homo sapiens GN=BCKDK PE=1 SV=2//0
XM_007990632.1	0.41643	0.0095565	0.044959	sp|Q8N0Z6|TTC5_HUMAN Tetratricopeptide repeat protein 5 OS=Homo sapiens GN=TTC5 PE=1 SV=2//0
XM_007990665.1	1.2311	1.11E-12	1.46E-11	sp|P00491|PNPH_HUMAN Purine nucleoside phosphorylase OS=Homo sapiens GN=PNP PE=1 SV=2//0
XM_007990683.1	-1.6669	3.09E-05	0.0002145	sp|Q8HZQ0|RNAS4_PANTR Ribonuclease 4 OS=Pan troglodytes GN=RNASE4 PE=3 SV=1//8.79113e-87
XM_007990694.1	0.69508	2.28E-08	2.17E-07	sp|Q9H7H0|MET17_HUMAN Methyltransferase-like protein 17, mitochondrial OS=Homo sapiens GN=METTL17 PE=1 SV=1//0
XM_007990748.1	0.30295	3.40E-05	0.0002351	sp|Q9Y5B9|SP16H_HUMAN FACT complex subunit SPT16 OS=Homo sapiens GN=SUPT16H PE=1 SV=1//0
XM_007990753.1	0.89042	4.19E-20	8.35E-19	sp|Q5R6A9|TOX4_PONAB TOX high mobility group box family member 4 OS=Pongo abelii GN=TOX4 PE=2 SV=2//0
XM_007990771.1	0.41021	1.12E-05	8.20E-05	sp|Q8VD66|ABHD4_MOUSE Abhydrolase domain-containing protein 4 OS=Mus musculus GN=Abhd4 PE=2 SV=1//0
XM_007990777.1	-0.40399	9.85E-05	0.0006477	sp|Q15070|OXA1L_HUMAN Mitochondrial inner membrane protein OXA1L OS=Homo sapiens GN=OXA1L PE=1 SV=3//0
XM_007990785.1	-1.4428	5.53E-39	2.34E-37	sp|P50281|MMP14_HUMAN Matrix metalloproteinase-14 OS=Homo sapiens GN=MMP14 PE=1 SV=3//0
XM_007990786.1	-1.0009	1.03E-22	2.35E-21	sp|Q7Z4F1|LRP10_HUMAN Low-density lipoprotein receptor-related protein 10 OS=Homo sapiens GN=LRP10 PE=1 SV=2//0
XM_007990792.1	0.95582	2.18E-09	2.25E-08	sp|Q86U06|RBM23_HUMAN Probable RNA-binding protein 23 OS=Homo sapiens GN=RBM23 PE=1 SV=1//3.97272e-13

XM_007990794.1	-0.84174	9.30E-05	0.000613	sp|O43294|TGFI1_HUMAN Transforming growth factor beta-1-induced transcript 1 protein OS=Homo sapiens GN=TGFB1I1 PE=1 SV=2//0
XM_007990812.1	1.9021	1.19E-128	3.18E-126	sp|Q96IF1|AJUBA_HUMAN LIM domain-containing protein ajuba OS=Homo sapiens GN=AJUBA PE=1 SV=1//0
XM_007990820.1	0.34567	0.0066404	0.032265	sp|Q96GQ5|RUS1_HUMAN RUS1 family protein C16orf58 OS=Homo sapiens GN=C16orf58 PE=1 SV=2//0
XM_007990846.1	-1.8044	1.89E-05	0.000135	sp|Q9H7J1|PPR3E_HUMAN Protein phosphatase 1 regulatory subunit 3E OS=Homo sapiens GN=PPP1R3E PE=1 SV=2//2.07344e-123
XM_007990849.1	-0.44247	1.51E-07	1.33E-06	sp|Q28165|PABP2_BOVIN Polyadenylate-binding protein 2 OS=Bos taurus GN=PABPN1 PE=1 SV=3//1.22761e-101
XM_007990881.1	0.69269	0.000122	0.0007918	sp|Q8NEJ9|NGDN_HUMAN Neuroguidin OS=Homo sapiens GN=NGDN PE=1 SV=1//8.65552e-151
XM_007990909.1	5.2752	6.07E-35	2.25E-33	sp|Q13268|DHRS2_HUMAN Dehydrogenase/reductase SDR family member 2, mitochondrial OS=Homo sapiens GN=DHRS2 PE=1 SV=4//2.75726e-160
XM_007990919.1	-1.728	1.12E-07	9.95E-07	sp|A6NEY3|GG6L3_HUMAN Putative golgin subfamily A member 6-like protein 3 OS=Homo sapiens GN=GOLGA6L3 PE=5 SV=3//5.09112e-56
XM_007990921.1	1.8245	3.24E-34	1.17E-32	sp|Q7Z2Z1|TICRR_HUMAN Treslin OS=Homo sapiens GN=TICRR PE=1 SV=2//0
XM_007990938.1	-0.69347	9.06E-05	0.0005979	sp|Q8N5I4|DHRSX_HUMAN Dehydrogenase/reductase SDR family member on chromosome X OS=Homo sapiens GN=DHRSX PE=2 SV=2//1.2494e-166
XM_007990940.1	-0.71658	0.0085399	0.040631	-//-
XM_007990969.1	0.42639	0.0068673	0.033265	sp|Q08623|HDHD1_HUMAN Pseudouridine-5'-phosphatase OS=Homo sapiens GN=HDHD1 PE=1 SV=3//1.06211e-153
XM_007990993.1	0.64001	3.62E-14	5.21E-13	sp|Q13796|SHRM2_HUMAN Protein Shroom2 OS=Homo sapiens GN=SHROOM2 PE=1 SV=1//0
XM_007990999.1	0.74755	8.03E-06	5.99E-05	sp|Q9ULE0|WWC3_HUMAN Protein WWC3 OS=Homo sapiens GN=WWC3 PE=1 SV=3//0
XM_007991001.1	1.8061	1.03E-48	6.06E-47	sp|P51793|CLCN4_HUMAN H(+)/Cl(-) exchange transporter 4 OS=Homo sapiens GN=CLCN4 PE=1 SV=2//0
XM_007991023.1	0.55819	2.46E-10	2.75E-09	sp|P80723|BASP1_HUMAN Brain acid soluble protein 1 OS=Homo sapiens GN=BASP1 PE=1 SV=2//7.30213e-09
XM_007991039.1	-1.6494	1.32E-34	4.82E-33	sp|P20065|TYB4_MOUSE Thymosin beta-4 OS=Mus musculus GN=Tmsb4x PE=1 SV=1//4.97794e-22
XM_007991057.1	1.3766	3.35E-42	1.57E-40	sp|O75665|OFD1_HUMAN Oral-facial-digital syndrome 1 protein OS=Homo sapiens GN=OFD1 PE=1 SV=1//0
XM_007991073.1	-0.70478	0.0031364	0.016376	sp|Q9NWZ8|GEMI8_HUMAN Gem-associated protein 8 OS=Homo sapiens GN=GEMIN8 PE=1 SV=1//1.02033e-146
XM_007991116.1	1.544	3.94E-14	5.66E-13	sp|Q9HBJ8|TMM27_HUMAN Collectrin OS=Homo sapiens GN=TMEM27 PE=1 SV=1//7.6521e-127
XM_007991136.1	-0.74259	6.10E-10	6.57E-09	sp|Q96A49|SYAP1_HUMAN Synapse-associated protein 1 OS=Homo sapiens GN=SYAP1 PE=1 SV=1//0
XM_007991256.1	0.39577	9.75E-06	7.19E-05	sp|Q5RA42|IF1AX_PONAB Eukaryotic translation initiation factor 1A, X-chromosomal OS=Pongo abelii GN=EIF1AX PE=2 SV=3//4.98334e-61
XM_007991257.1	-1.406	4.39E-19	8.36E-18	sp|P51812|KS6A3_HUMAN Ribosomal protein S6 kinase alpha-3 OS=Homo sapiens GN=RPS6KA3 PE=1 SV=1//0
XM_007991281.1	0.37353	0.0011886	0.0067227	sp|O43462|MBTP2_HUMAN Membrane-bound transcription factor site-2 protease OS=Homo sapiens GN=MBTPS2 PE=1 SV=1//0
XM_007991284.1	-0.50281	0.0083153	0.039656	sp|Q6PRD7|CEMP1_HUMAN Cementoblastoma-derived protein 1 OS=Homo sapiens GN=CEMP1 PE=2 SV=1//8.3979e-131
XM_007991288.1	0.75694	1.04E-10	1.19E-09	sp|Q9NXV2|KCTD5_HUMAN BTB/POZ domain-containing protein KCTD5 OS=Homo sapiens GN=KCTD5 PE=1 SV=1//4.04663e-27
XM_007991301.1	0.56965	3.25E-15	4.94E-14	sp|P21673|SAT1_HUMAN Diamine acetyltransferase 1 OS=Homo sapiens GN=SAT1 PE=1 SV=1//5.68647e-121
XM_007991318.1	-1.1151	9.35E-11	1.07E-09	sp|A6NKF1|SAC31_HUMAN SAC3 domain-containing protein 1 OS=Homo sapiens GN=SAC3D1 PE=1 SV=2//2.75755e-156
XM_007991322.1	0.37943	0.0001084	0.0007077	sp|P09884|DPOLA_HUMAN DNA polymerase alpha catalytic subunit OS=Homo sapiens GN=POLA1 PE=1 SV=2//0
XM_007991366.1	0.43593	1.32E-07	1.17E-06	sp|Q92793|CBP_HUMAN CREB-binding protein OS=Homo sapiens GN=CREBBP PE=1 SV=3//6.94862e-46
XM_007991374.1	0.99919	1.98E-15	3.04E-14	sp|Q96EY1|DNJA3_HUMAN DnaJ homolog subfamily A member 3, mitochondrial OS=Homo sapiens GN=DNAJA3 PE=1 SV=2//1.24163e-16
XM_007991402.1	-1.2511	4.70E-11	5.54E-10	sp|P41732|TSN7_HUMAN Tetraspanin-7 OS=Homo sapiens GN=TSPAN7 PE=1 SV=2//2.68481e-179
XM_007991403.1	1.1958	1.19E-27	3.37E-26	sp|Q9NPA3|M1IP1_HUMAN Mid1-interacting protein 1 OS=Homo sapiens GN=MID1IP1 PE=1 SV=1//1.92059e-122
XM_007991420.1	-0.92391	6.80E-17	1.15E-15	sp|Q5R563|RENR_PONAB Renin receptor OS=Pongo abelii GN=ATP6AP2 PE=2 SV=1//0
XM_007991421.1	1.5299	1.47E-76	1.73E-74	sp|Q8IWR0|Z3H7A_HUMAN Zinc finger CCCH domain-containing protein 7A OS=Homo sapiens GN=ZC3H7A PE=1 SV=1//0
XM_007991453.1	2.3853	2.29E-06	1.81E-05	sp|Q5RE60|AOFA_PONAB Amine oxidase [flavin-containing] A OS=Pongo abelii GN=MAOA PE=2 SV=1//0
XM_007991495.1	0.94881	2.95E-16	4.79E-15	sp|Q86YC2|PALB2_HUMAN Partner and localizer of BRCA2 OS=Homo sapiens

				GN=PALB2 PE=1 SV=1//0
XM_007991498.1	0.88895	1.29E-13	1.80E-12	sp|O75695|XRP2_HUMAN Protein XRP2 OS=Homo sapiens GN=RP2 PE=1 SV=4//0
XM_007991509.1	-1.0985	1.28E-17	2.25E-16	sp|Q0MQJ3|NDUBB_PONPY NADH dehydrogenase [ubiquinone] 1 beta subcomplex subunit 11, mitochondrial OS=Pongo pygmaeus GN=NDUFB11 PE=2 SV=1//3.91675e-89
XM_007991533.1	-1.7048	8.23E-05	0.0005465	sp|P52846|ST1A1_MACFA Sulfotransferase 1A1 OS=Macaca fascicularis GN=SULT1A1 PE=2 SV=1//3.07225e-32
XM_007991541.1	-0.71455	2.66E-10	2.96E-09	sp|P49061|TIMP1_PAPCY Metalloproteinase inhibitor 1 OS=Papio cynocephalus GN=TIMP1 PE=2 SV=1//1.43394e-136
XM_007991544.1	0.51023	9.47E-07	7.78E-06	sp|P10398|ARAF_HUMAN Serine/threonine-protein kinase A-Raf OS=Homo sapiens GN=ARAF PE=1 SV=2//0
XM_007991549.1	-0.59804	1.05E-08	1.02E-07	sp|Q9UBK9|UXT_HUMAN Protein UXT OS=Homo sapiens GN=UXT PE=1 SV=1//1.37874e-85
XM_007991553.1	0.92298	2.40E-05	0.000169	sp|P17025|ZN182_HUMAN Zinc finger protein 182 OS=Homo sapiens GN=ZNF182 PE=2 SV=2//0
XM_007991559.1	0.69811	7.28E-06	5.47E-05	sp|P51508|ZNF81_HUMAN Zinc finger protein 81 OS=Homo sapiens GN=ZNF81 PE=1 SV=3//0
XM_007991575.1	0.6582	1.25E-07	1.11E-06	-//-
XM_007991578.1	5.4572	4.26E-94	6.96E-92	sp|Q9UET6|TRM7_HUMAN Putative tRNA (cytidine(32)/guanosine(34)-2'-O)-methyltransferase OS=Homo sapiens GN=FTSJ1 PE=1 SV=2//0
XM_007991579.1	-0.95371	4.37E-13	5.87E-12	sp|Q9NRS6|SNX15_HUMAN Sorting nexin-15 OS=Homo sapiens GN=SNX15 PE=1 SV=1//1.43323e-165
XM_007991593.1	0.9103	7.76E-13	1.03E-11	sp|Q3MII6|TBC25_HUMAN TBC1 domain family member 25 OS=Homo sapiens GN=TBC1D25 PE=1 SV=2//0
XM_007991663.1	-1.0812	6.88E-14	9.79E-13	sp|Q4R4I9|PRAF2_MACFA PRA1 family protein 2 OS=Macaca fascicularis GN=PRAF2 PE=2 SV=1//1.19157e-64
XM_007991676.1	-1.0853	4.10E-32	1.36E-30	sp|Q04941|PLP2_HUMAN Proteolipid protein 2 OS=Homo sapiens GN=PLP2 PE=1 SV=1//1.59557e-102
XM_007991708.1	1.0309	2.02E-05	0.0001436	sp|Q8N1L9|BATF2_HUMAN Basic leucine zipper transcriptional factor ATF-like 2 OS=Homo sapiens GN=BATF2 PE=1 SV=1//3.77149e-117
XM_007991739.1	-0.94446	3.71E-05	0.0002548	sp|Q96G61|NUD11_HUMAN Diphosphoinositol polyphosphate phosphohydrolase 3-beta OS=Homo sapiens GN=NUDT11 PE=1 SV=1//7.0679e-82
XM_007991776.1	-2.0103	3.37E-07	2.88E-06	-//-
XM_007991781.1	-0.72956	7.90E-15	1.18E-13	sp|Q99714|HCD2_HUMAN 3-hydroxyacyl-CoA dehydrogenase type-2 OS=Homo sapiens GN=HSD17B10 PE=1 SV=3//6.45257e-180
XM_007991839.1	-1.0912	0.0003324	0.0020301	sp|Q5XKR9|F104B_HUMAN Protein FAM104B OS=Homo sapiens GN=FAM104B PE=3 SV=1//6.03276e-67
XM_007991846.1	-1.0983	0.0006102	0.0035974	sp|Q9H213|MAGH1_HUMAN Melanoma-associated antigen H1 OS=Homo sapiens GN=MAGEH1 PE=1 SV=1//1.0357e-117
XM_007991871.1	-1.3624	1.03E-45	5.48E-44	sp|Q6P823|H33_XENTR Histone H3.3 OS=Xenopus tropicalis GN=TGas113e22.1 PE=1 SV=3//2.05964e-81
XM_007991883.1	-0.95473	2.66E-06	2.09E-05	sp|Q96HE8|TMM80_HUMAN Transmembrane protein 80 OS=Homo sapiens GN=TMEM80 PE=2 SV=3//8.04911e-48
XM_007991884.1	-1.5343	6.10E-11	7.12E-10	sp|Q2TA37|ARL2_BOVIN ADP-ribosylation factor-like protein 2 OS=Bos taurus GN=ARL2 PE=1 SV=1//2.83742e-126
XM_007991957.1	-2.5301	1.98E-147	6.47E-145	sp|P98172|EFNB1_HUMAN Ephrin-B1 OS=Homo sapiens GN=EFNB1 PE=1 SV=1//0
XM_007991986.1	0.35158	5.70E-05	0.0003843	sp|O95239|KIF4A_HUMAN Chromosome-associated kinesin KIF4A OS=Homo sapiens GN=KIF4A PE=1 SV=3//0
XM_007992006.1	-1.0405	4.13E-09	4.16E-08	sp|P98177|FOXO4_HUMAN Forkhead box protein O4 OS=Homo sapiens GN=FOXO4 PE=1 SV=5//0
XM_007992017.1	-1.1488	3.13E-05	0.000217	sp|P31785|IL2RG_HUMAN Cytokine receptor common subunit gamma OS=Homo sapiens GN=IL2RG PE=1 SV=1//0
XM_007992032.1	-0.39167	1.11E-06	9.06E-06	sp|Q9UKP3|ITBP2_HUMAN Integrin beta-1-binding protein 2 OS=Homo sapiens GN=ITGB1BP2 PE=1 SV=1//0
XM_007992033.1	0.96531	8.41E-23	1.94E-21	sp|P21675|TAF1_HUMAN Transcription initiation factor TFIID subunit 1 OS=Homo sapiens GN=TAF1 PE=1 SV=2//0
XM_007992050.1	-0.56884	0.0008172	0.004729	sp|Q5HYW3|RGAG4_HUMAN Retrotransposon gag domain-containing protein 4 OS=Homo sapiens GN=RGAG4 PE=2 SV=1//2.92536e-49
XM_007992066.1	-1.9248	6.65E-12	8.33E-11	sp|Q9Y237|PIN4_HUMAN Peptidyl-prolyl cis-trans isomerase NIMA-interacting 4 OS=Homo sapiens GN=PIN4 PE=1 SV=1//3.77548e-67
XM_007992068.1	-0.57765	2.06E-10	2.32E-09	sp|P79103|RS4_BOVIN 40S ribosomal protein S4 OS=Bos taurus GN=RPS4 PE=2 SV=3//0
XM_007992076.1	-0.76246	0.005629	0.027782	sp|Q9BY41|HDAC8_HUMAN Histone deacetylase 8 OS=Homo sapiens GN=HDAC8 PE=1 SV=2//0
XM_007992094.1	-1.7359	1.82E-12	2.38E-11	sp|P36021|MOT8_HUMAN Monocarboxylate transporter 8 OS=Homo sapiens GN=SLC16A2 PE=1 SV=2//0
XM_007992098.1	0.83217	1.20E-18	2.24E-17	sp|Q9NVW2|RNF12_HUMAN E3 ubiquitin-protein ligase RLIM OS=Homo sapiens GN=RLIM PE=1 SV=3//0
XM_007992119.1	-1.0266	1.73E-07	1.51E-06	sp|Q9BE18|MAGE1_MACFA Melanoma-associated antigen E1 OS=Macaca fascicularis GN=MAGEE1 PE=2 SV=1//0

XM_007992135.1	-1.4515	2.73E-46	1.50E-44	sp|Q5R9K2|COX7B_PONAB Cytochrome c oxidase subunit 7B, mitochondrial OS=Pongo abelii GN=COX7B PE=3 SV=1//5.17038e-53
XM_007992136.1	-1.0805	2.19E-11	2.65E-10	sp|Q04656|ATP7A_HUMAN Copper-transporting ATPase 1 OS=Homo sapiens GN=ATP7A PE=1 SV=3//0
XM_007992139.1	-1.3394	6.51E-31	2.07E-29	sp|A5A6K4|PGK1_PANTR Phosphoglycerate kinase 1 OS=Pan troglodytes GN=PGK1 PE=2 SV=1//0
XM_007992140.1	-0.58374	7.61E-07	6.31E-06	sp|Q5R7P7|TAF9B_PONAB Transcription initiation factor TFIID subunit 9B OS=Pongo abelii GN=TAF9B PE=2 SV=1//4.67875e-172
XM_007992154.1	0.64316	5.76E-14	8.21E-13	sp|Q2HJ94|DNJA2_BOVIN DnaJ homolog subfamily A member 2 OS=Bos taurus GN=DNAJA2 PE=2 SV=1//0
XM_007992160.1	-1.0364	0.001288	0.0072468	sp|Q6RI45|BRWD3_HUMAN Bromodomain and WD repeat-containing protein 3 OS=Homo sapiens GN=BRWD3 PE=1 SV=2//3.92199e-37
XM_007992162.1	-1.1986	1.73E-11	2.11E-10	sp|Q6P823|H33_XENTR Histone H3.3 OS=Xenopus tropicalis GN=TGas113e22.1 PE=1 SV=3//2.76909e-89
XM_007992163.1	-1.6235	1.84E-43	8.94E-42	sp|Q5RFN7|SH3L1_PONAB SH3 domain-binding glutamic acid-rich-like protein OS=Pongo abelii GN=SH3BGRL PE=3 SV=1//2.34435e-70
XM_007992185.1	-1.0994	1.03E-27	2.92E-26	sp|Q8TB96|TIP_HUMAN T-cell immunomodulatory protein OS=Homo sapiens GN=ITFG1 PE=1 SV=1//0
XM_007992190.1	-2.2363	0.0005369	0.0031973	sp|Q86VE3|SATL1_HUMAN Spermidine/spermine N(1)-acetyltransferase-like protein 1 OS=Homo sapiens GN=SATL1 PE=2 SV=3//1.38976e-08
XM_007992195.1	0.6756	1.54E-11	1.89E-10	sp|P24386|RAE1_HUMAN Rab proteins geranylgeranyltransferase component A 1 OS=Homo sapiens GN=CHM PE=1 SV=3//0
XM_007992223.1	3.1782	0.0002091	0.0013126	sp|Q13156|RFA4_HUMAN Replication protein A 30 kDa subunit OS=Homo sapiens GN=RPA4 PE=1 SV=2//2.1501e-142
XM_007992290.1	1.6099	1.93E-09	2.00E-08	sp|O60220|TIM8A_HUMAN Mitochondrial import inner membrane translocase subunit Tim8 A OS=Homo sapiens GN=TIMM8A PE=1 SV=1//4.63742e-56
XM_007992302.1	0.8917	3.02E-13	4.11E-12	sp|Q9P291|ARMX1_HUMAN Armadillo repeat-containing X-linked protein 1 OS=Homo sapiens GN=ARMCX1 PE=1 SV=1//0
XM_007992313.1	-1.5435	0.0023785	0.012713	sp|Q7L311|ARMX2_HUMAN Armadillo repeat-containing X-linked protein 2 OS=Homo sapiens GN=ARMCX2 PE=2 SV=1//0
XM_007992318.1	-0.9422	0.0016954	0.0093281	sp|Q5H9K5|ZMAT1_HUMAN Zinc finger matrin-type protein 1 OS=Homo sapiens GN=ZMAT1 PE=2 SV=1//0
XM_007992331.1	-0.88368	0.0038693	0.019784	sp|Q4R7Y2|RL10_MACFA 60S ribosomal protein L10 OS=Macaca fascicularis GN=RPL10 PE=2 SV=1//1.26979e-140
XM_007992357.1	0.70716	6.61E-06	4.98E-05	sp|Q6P1M9|ARMX5_HUMAN Armadillo repeat-containing X-linked protein 5 OS=Homo sapiens GN=ARMCX5 PE=1 SV=1//0
XM_007992366.1	1.1106	1.75E-27	4.91E-26	sp|Q2PG52|BEX1_MACFA Protein BEX1 OS=Macaca fascicularis GN=BEX1 PE=2 SV=1//1.23873e-63
XM_007992377.1	0.73636	0.0014862	0.00826	sp|Q9NWD9|BEX4_HUMAN Protein BEX4 OS=Homo sapiens GN=BEX4 PE=2 SV=1//4.7449e-67
XM_007992444.1	-0.44931	0.0001078	0.0007041	sp|Q6PEV8|F199X_HUMAN Protein FAM199X OS=Homo sapiens GN=FAM199X PE=1 SV=1//0
XM_007992468.1	-1.7026	0.0029829	0.015657	sp|Q8IUQ4|SIAH1_HUMAN E3 ubiquitin-protein ligase SIAH1 OS=Homo sapiens GN=SIAH1 PE=1 SV=2//0
XM_007992469.1	-1.9496	4.24E-26	1.13E-24	sp|Q5RF74|RN128_PONAB E3 ubiquitin-protein ligase RNF128 OS=Pongo abelii GN=RNF128 PE=2 SV=1//0
XM_007992474.1	-2.1448	2.00E-89	3.09E-87	sp|P57739|CLD2_HUMAN Claudin-2 OS=Homo sapiens GN=CLDN2 PE=1 SV=1//8.22603e-112
XM_007992506.1	0.95539	1.40E-12	1.84E-11	sp|P60892|PRPS1_RAT Ribose-phosphate pyrophosphokinase 1 OS=Rattus norvegicus GN=Prps1 PE=1 SV=2//0
XM_007992590.1	1.8686	3.32E-94	5.47E-92	sp|O75113|N4BP1_HUMAN NEDD4-binding protein 1 OS=Homo sapiens GN=N4BP1 PE=1 SV=4//0
XM_007992622.1	1.0828	0.0005484	0.003262	sp|Q4VCS5|AMOT_HUMAN Angiomotin OS=Homo sapiens GN=AMOT PE=1 SV=1//5.06488e-26
XM_007992640.1	-1.0552	1.88E-11	2.29E-10	sp|Q02819|NUCB1_MOUSE Nucleobindin-1 OS=Mus musculus GN=Nucb1 PE=1 SV=2//1.06024e-55
XM_007992660.1	1.6836	4.68E-09	4.69E-08	sp|Q9BVJ6|UT14A_HUMAN U3 small nucleolar RNA-associated protein 14 homolog A OS=Homo sapiens GN=UTP14A PE=1 SV=1//0
XM_007992678.1	1.7348	1.12E-09	1.18E-08	sp|Q8ND82|Z280C_HUMAN Zinc finger protein 280C OS=Homo sapiens GN=ZNF280C PE=1 SV=1//0
XM_007992686.1	0.77681	4.36E-07	3.69E-06	sp|Q9Y388|RBMX2_HUMAN RNA-binding motif protein, X-linked 2 OS=Homo sapiens GN=RBMX2 PE=1 SV=2//3.55892e-142
XM_007992736.1	0.72874	3.04E-20	6.10E-19	sp|O75487|GPC4_HUMAN Glypican-4 OS=Homo sapiens GN=GPC4 PE=1 SV=4//0
XM_007992776.1	-1.9881	9.86E-08	8.81E-07	sp|A6ZKI3|F127A_HUMAN Protein FAM127A OS=Homo sapiens GN=FAM127A PE=1 SV=1//3.85805e-73
XM_007992777.1	-1.5491	2.34E-19	4.51E-18	sp|Q17RB0|F127C_HUMAN Protein FAM127C OS=Homo sapiens GN=FAM127C PE=1 SV=1//8.28803e-61
XM_007992783.1	-2.8274	0.0003777	0.0022908	sp|P0DMW5|SIL2B_HUMAN Small integral membrane protein 10-like protein 2B OS=Homo sapiens GN=SMIM10L2B PE=4 SV=1//4.2595e-21
XM_007992797.1	0.40776	0.0010269	0.0058558	sp|Q5RA70|MMGT1_PONAB Membrane magnesium transporter 1 OS=Pongo abelii GN=MMGT1 PE=2 SV=1//1.49968e-64

XM_007992822.1	-0.36388	0.0017399	0.0095509	sp|Q5RB63|HTSF1_PONAB HIV Tat-specific factor 1 homolog OS=Pongo abelii GN=HTATSF1 PE=2 SV=1//1.47024e-17
XM_007992827.1	0.74556	6.63E-13	8.83E-12	sp|Q7Z4Q2|HEAT3_HUMAN HEAT repeat-containing protein 3 OS=Homo sapiens GN=HEATR3 PE=1 SV=2//0
XM_007992835.1	1.3515	1.67E-08	1.60E-07	sp|Q6PJ61|FBX46_HUMAN F-box only protein 46 OS=Homo sapiens GN=FBXO46 PE=1 SV=3//0
XM_007992904.1	-0.4556	4.32E-06	3.32E-05	sp|O75398|DEAF1_HUMAN Deformed epidermal autoregulatory factor 1 homolog OS=Homo sapiens GN=DEAF1 PE=1 SV=1//3.62279e-09
XM_007992922.1	-0.52946	2.38E-08	2.26E-07	sp|P22304|IDS_HUMAN Iduronate 2-sulfatase OS=Homo sapiens GN=IDS PE=1 SV=1//0
XM_007992924.1	-1.3396	0.0007433	0.0043241	sp|P22304|IDS_HUMAN Iduronate 2-sulfatase OS=Homo sapiens GN=IDS PE=1 SV=1//2.07223e-48
XM_007992970.1	-0.68248	0.0017944	0.0098232	sp|Q13496|MTM1_HUMAN Myotubularin OS=Homo sapiens GN=MTM1 PE=1 SV=2//0
XM_007993047.1	-1.8144	7.26E-12	9.08E-11	sp|P21810|PGS1_HUMAN Biglycan OS=Homo sapiens GN=BGN PE=1 SV=2//0
XM_007993075.1	-0.45301	1.53E-09	1.60E-08	sp|P28570|SC6A8_RAT Sodium- and chloride-dependent creatine transporter 1 OS=Rattus norvegicus GN=Slc6a8 PE=2 SV=1//0
XM_007993086.1	-0.60745	4.47E-11	5.29E-10	sp|Q5REH6|SSRD_PONAB Translocon-associated protein subunit delta OS=Pongo abelii GN=SSR4 PE=2 SV=2//2.2526e-104
XM_007993125.1	0.25023	0.0012582	0.0070881	sp|P51617|IRAK1_HUMAN Interleukin-1 receptor-associated kinase 1 OS=Homo sapiens GN=IRAK1 PE=1 SV=2//0
XM_007993126.1	-0.91611	6.12E-21	1.28E-19	sp|Q95LG8|MECP2_MACFA Methyl-CpG-binding protein 2 OS=Macaca fascicularis GN=MECP2 PE=2 SV=1//5.80766e-31
XM_007993136.1	0.78251	1.27E-14	1.88E-13	sp|P50402|EMD_HUMAN Emerin OS=Homo sapiens GN=EMD PE=1 SV=1//2.54696e-125
XM_007993146.1	-0.96093	8.99E-16	1.42E-14	sp|Q14657|LAGE3_HUMAN EKC/KEOPS complex subunit LAGE3 OS=Homo sapiens GN=LAGE3 PE=1 SV=2//3.68707e-53
XM_007993180.1	1.4092	4.23E-58	3.13E-56	sp|O60832|DKC1_HUMAN H/ACA ribonucleoprotein complex subunit 4 OS=Homo sapiens GN=DKC1 PE=1 SV=3//0
XM_007993235.1	1.2776	0.0010697	0.0060809	sp|Q8HZJ0|ASMT_MACMU Acetylserotonin O-methyltransferase OS=Macaca mulatta GN=ASMT PE=2 SV=1//2.95279e-95
XM_007993237.1	-0.90816	1.85E-15	2.86E-14	sp|P14209|CD99_HUMAN CD99 antigen OS=Homo sapiens GN=CD99 PE=1 SV=1//1.26536e-07
XM_007993252.1	1.1497	8.61E-24	2.07E-22	sp|P23607|ZFA_MOUSE Zinc finger autosomal protein OS=Mus musculus GN=Zfa PE=3 SV=1//1.36813e-10
XM_007993260.1	-1.9025	0.0042855	0.021729	sp|Q8HXX6|SAP3_MACFA Ganglioside GM2 activator OS=Macaca fascicularis GN=GM2A PE=2 SV=2//2.56658e-89
XM_007993262.1	1.7069	3.04E-12	3.89E-11	sp|Q2M3X9|ZN674_HUMAN Zinc finger protein 674 OS=Homo sapiens GN=ZNF674 PE=2 SV=1//0
XM_007993267.1	0.34288	0.0019854	0.010753	sp|Q4V328|GRAP1_HUMAN GRIP1-associated protein 1 OS=Homo sapiens GN=GRIPAP1 PE=1 SV=1//3.98228e-103
XM_007993278.1	-0.42298	5.91E-10	6.38E-09	sp|Q5RFL9|NONO_PONAB Non-POU domain-containing octamer-binding protein OS=Pongo abelii GN=NONO PE=2 SV=3//4.04231e-175
XM_007993285.1	0.49207	0.0074781	0.035945	sp|Q9BWT1|CDCA7_HUMAN Cell division cycle-associated protein 7 OS=Homo sapiens GN=CDCA7 PE=1 SV=1//9.3448e-106
XM_007993287.1	0.38908	0.0005945	0.0035079	sp|O60488|ACSL4_HUMAN Long-chain-fatty-acid--CoA ligase 4 OS=Homo sapiens GN=ACSL4 PE=1 SV=2//0
XM_007993299.1	-0.52941	1.44E-05	0.0001042	sp|O15347|HMGB3_HUMAN High mobility group protein B3 OS=Homo sapiens GN=HMGB3 PE=1 SV=4//3.02051e-53
XM_007993302.1	-0.59672	5.01E-10	5.46E-09	sp|P51572|BAP31_HUMAN B-cell receptor-associated protein 31 OS=Homo sapiens GN=BCAP31 PE=1 SV=3//4.69752e-112
XM_007993304.1	-0.70424	0.010416	0.048553	sp|P33897|ABCD1_HUMAN ATP-binding cassette sub-family D member 1 OS=Homo sapiens GN=ABCD1 PE=1 SV=2//0
XM_007993330.1	-1.4011	5.18E-10	5.63E-09	sp|Q7L5N7|PCAT2_HUMAN Lysophosphatidylcholine acyltransferase 2 OS=Homo sapiens GN=LPCAT2 PE=1 SV=1//0
XM_007993346.1	-0.35578	5.97E-06	4.53E-05	sp|Q9UKV5|AMFR_HUMAN E3 ubiquitin-protein ligase AMFR OS=Homo sapiens GN=AMFR PE=1 SV=2//0
XM_007993347.1	-0.46582	2.34E-05	0.0001647	sp|Q5RAI8|CPSF5_PONAB Cleavage and polyadenylation specificity factor subunit 5 OS=Pongo abelii GN=NUDT21 PE=2 SV=1//2.90573e-161
XM_007993350.1	-0.83957	8.40E-13	1.11E-11	sp|Q9BXC9|BBS2_HUMAN Bardet-Biedl syndrome 2 protein OS=Homo sapiens GN=BBS2 PE=1 SV=1//0
XM_007993354.1	-2.2279	1.64E-61	1.35E-59	sp|P68303|MT2_MACFA Metallothionein-2 OS=Macaca fascicularis GN=MT2 PE=3 SV=1//3.03936e-09
XM_007993356.1	-1.208	3.40E-46	1.86E-44	-//-
XM_007993357.1	-2.4601	1.36E-42	6.38E-41	-//-
XM_007993398.1	3.3721	1.64E-75	1.88E-73	sp|Q15011|HERP1_HUMAN Homocysteine-responsive endoplasmic reticulum-resident ubiquitin-like domain member 1 protein OS=Homo sapiens GN=HERPUD1 PE=1 SV=1//0
XM_007993429.1	-0.53793	5.24E-07	4.40E-06	sp|Q4R930|AR2BP_MACFA ADP-ribosylation factor-like protein 2-binding protein OS=Macaca fascicularis GN=ARL2BP PE=2 SV=1//9.53626e-96
XM_007993430.1	-1.7386	1.70E-11	2.08E-10	sp|Q9Y342|PLLP_HUMAN Plasmolipin OS=Homo sapiens GN=PLLP PE=1

				SV=1//5.92697e-101
XM_007993433.1	-1.4653	1.66E-14	2.43E-13	sp|P78423|X3CL1_HUMAN Fractalkine OS=Homo sapiens GN=CX3CL1 PE=1 SV=1//0
XM_007993437.1	0.48317	2.72E-06	2.13E-05	sp|Q6FI81|CPIN1_HUMAN Anamorsin OS=Homo sapiens GN=CIAPIN1 PE=1 SV=2//0
XM_007993442.1	0.52076	6.57E-07	5.47E-06	sp|P19387|RPB3_HUMAN DNA-directed RNA polymerase II subunit RPB3 OS=Homo sapiens GN=POLR2C PE=1 SV=2//0
XM_007993443.1	-0.43552	0.000213	0.001335	sp|Q96A19|C102A_HUMAN Coiled-coil domain-containing protein 102A OS=Homo sapiens GN=CCDC102A PE=1 SV=2//0
XM_007993495.1	-2.369	7.03E-42	3.23E-40	sp|P51511|MMP15_HUMAN Matrix metalloproteinase-15 OS=Homo sapiens GN=MMP15 PE=1 SV=1//0
XM_007993543.1	-0.41678	1.28E-07	1.14E-06	sp|Q4R559|AATM_MACFA Aspartate aminotransferase, mitochondrial OS=Macaca fascicularis GN=GOT2 PE=2 SV=1//0
XM_007993562.1	-0.6994	1.24E-18	2.31E-17	sp|P37837|TALDO_HUMAN Transaldolase OS=Homo sapiens GN=TALDO1 PE=1 SV=2//0
XM_007993580.1	-1.7352	0.0013453	0.0075458	sp|Q96MX0|CKLF3_HUMAN CKLF-like MARVEL transmembrane domain-containing protein 3 OS=Homo sapiens GN=CMTM3 PE=1 SV=1//2.55055e-82
XM_007993583.1	#NAME?	9.78E-05	0.0006433	sp|Q8IZR5|CKLF4_HUMAN CKLF-like MARVEL transmembrane domain-containing protein 4 OS=Homo sapiens GN=CMTM4 PE=1 SV=1//2.48088e-122
XM_007993586.1	0.31154	0.0001068	0.0006978	sp|Q6PDL0|DC1L2_MOUSE Cytoplasmic dynein 1 light intermediate chain 2 OS=Mus musculus GN=Dync1li2 PE=1 SV=2//0
XM_007993618.1	1.4458	1.68E-05	0.0001206	sp|O00748|EST2_HUMAN Cocaine esterase OS=Homo sapiens GN=CES2 PE=1 SV=1//0
XM_007993619.1	2.1164	9.89E-40	4.27E-38	sp|O00748|EST2_HUMAN Cocaine esterase OS=Homo sapiens GN=CES2 PE=1 SV=1//0
XM_007993641.1	0.77418	7.55E-21	1.57E-19	sp|Q6PCD5|RFWD3_HUMAN E3 ubiquitin-protein ligase RFWD3 OS=Homo sapiens GN=RFWD3 PE=1 SV=3//0
XM_007993642.1	-1.1904	4.80E-30	1.48E-28	sp|Q92896|GSLG1_HUMAN Golgi apparatus protein 1 OS=Homo sapiens GN=GLG1 PE=1 SV=2//1.80317e-12
XM_007993647.1	1.3175	2.67E-06	2.10E-05	-//-
XM_007993648.1	0.67315	2.31E-05	0.0001632	sp|Q5RKV6|EXOS6_HUMAN Exosome complex component MTR3 OS=Homo sapiens GN=EXOSC6 PE=1 SV=1//4.26302e-87
XM_007993649.1	1.4031	7.53E-57	5.39E-55	sp|P49588|SYAC_HUMAN Alanine--tRNA ligase, cytoplasmic OS=Homo sapiens GN=AARS PE=1 SV=2//0
XM_007993650.1	1.0767	4.71E-26	1.25E-24	sp|Q9NUU7|DD19A_HUMAN ATP-dependent RNA helicase DDX19A OS=Homo sapiens GN=DDX19A PE=1 SV=1//0
XM_007993675.1	1.1826	4.81E-64	4.30E-62	sp|Q15393|SF3B3_HUMAN Splicing factor 3B subunit 3 OS=Homo sapiens GN=SF3B3 PE=1 SV=4//0
XM_007993759.1	0.97782	4.52E-32	1.49E-30	sp|P0C7T5|ATX1L_HUMAN Ataxin-1-like OS=Homo sapiens GN=ATXN1L PE=1 SV=1//0
XM_007993773.1	0.85253	6.13E-22	1.35E-20	sp|Q92620|PRP16_HUMAN Pre-mRNA-splicing factor ATP-dependent RNA helicase PRP16 OS=Homo sapiens GN=DHX38 PE=1 SV=2//0
XM_007993787.1	0.7281	8.59E-05	0.0005684	sp|Q02127|PYRD_HUMAN Dihydroorotate dehydrogenase (quinone), mitochondrial OS=Homo sapiens GN=DHODH PE=1 SV=3//0
XM_007993793.1	0.29495	0.0007122	0.0041543	sp|Q3ZBD0|PSMD7_BOVIN 26S proteasome non-ATPase regulatory subunit 7 OS=Bos taurus GN=PSMD7 PE=2 SV=1//0
XM_007993794.1	-1.7814	7.15E-84	9.98E-82	sp|Q5RD31|NQO1_PONAB NAD(P)H dehydrogenase [quinone] 1 OS=Pongo abelii GN=NQO1 PE=2 SV=1//0
XM_007993795.1	1.6239	6.21E-68	6.07E-66	sp|Q4R537|NOB1_MACFA RNA-binding protein NOB1 OS=Macaca fascicularis GN=NOB1 PE=2 SV=1//0
XM_007993814.1	0.8899	0.0057998	0.028523	sp|Q9HBH1|DEFM_HUMAN Peptide deformylase, mitochondrial OS=Homo sapiens GN=PDF PE=1 SV=1//4.48123e-144
XM_007993815.1	1.0343	2.40E-16	3.93E-15	sp|Q96MW5|COG8_HUMAN Conserved oligomeric Golgi complex subunit 8 OS=Homo sapiens GN=COG8 PE=1 SV=2//0
XM_007993816.1	1.9255	1.02E-65	9.51E-64	sp|Q5R9J1|NIP7_PONAB 60S ribosome subunit biogenesis protein NIP7 homolog OS=Pongo abelii GN=NIP7 PE=2 SV=1//1.02812e-125
XM_007993819.1	-1.3397	4.34E-29	1.29E-27	sp|Q13425|SNTB2_HUMAN Beta-2-syntrophin OS=Homo sapiens GN=SNTB2 PE=1 SV=1//0
XM_007993837.1	1.7115	0.0031645	0.016511	sp|P12830|CADH1_HUMAN Cadherin-1 OS=Homo sapiens GN=CDH1 PE=1 SV=3//0
XM_007993860.1	1.4308	6.51E-27	1.78E-25	sp|Q96CW6|S7A6O_HUMAN Probable RNA polymerase II nuclear localization protein SLC7A6OS OS=Homo sapiens GN=SLC7A6OS PE=1 SV=2//2.61259e-151
XM_007993874.1	#NAME?	0.010041	0.047039	sp|Q8NCC3|PAG15_HUMAN Group XV phospholipase A2 OS=Homo sapiens GN=PLA2G15 PE=1 SV=2//0
XM_007993883.1	1.2148	1.14E-10	1.29E-09	sp|Q4R4T6|DDX28_MACFA Probable ATP-dependent RNA helicase DDX28 OS=Macaca fascicularis GN=DDX28 PE=2 SV=1//0
XM_007993901.1	0.72265	3.27E-06	2.54E-05	sp|Q08758|LCAT_PAPAN Phosphatidylcholine-sterol acyltransferase OS=Papio anubis GN=LCAT PE=2 SV=1//0
XM_007993908.1	0.35002	0.0007791	0.0045172	sp|P11801|KPSH1_HUMAN Serine/threonine-protein kinase H1 OS=Homo sapiens GN=PSKH1 PE=1 SV=4//0
XM_007993947.1	-0.4705	2.57E-07	2.21E-06	sp|Q9UI30|TR112_HUMAN Multifunctional methyltransferase subunit TRM112-like protein OS=Homo sapiens GN=TRMT112 PE=1 SV=1//2.12695e-78

XM_007994012.1	0.49666	0.0049042	0.024498	sp|Q9BTX3|TM208_HUMAN Transmembrane protein 208 OS=Homo sapiens GN=TMEM208 PE=1 SV=1//1.96876e-112
XM_007994017.1	-4.2704	0.0015982	0.0088352	sp|Q15628|TRADD_HUMAN Tumor necrosis factor receptor type 1-associated DEATH domain protein OS=Homo sapiens GN=TRADD PE=1 SV=2//2.07851e-174
XM_007994059.1	0.48064	0.0054523	0.026995	sp|Q16254|E2F4_HUMAN Transcription factor E2F4 OS=Homo sapiens GN=E2F4 PE=1 SV=2//0
XM_007994091.1	-1.0836	2.38E-18	4.35E-17	sp|Q96C90|PP14B_HUMAN Protein phosphatase 1 regulatory subunit 14B OS=Homo sapiens GN=PPP1R14B PE=1 SV=3//2.5036e-63
XM_007994094.1	-1.044	0.0001576	0.0010056	sp|Q9GZX3|CHST6_HUMAN Carbohydrate sulfotransferase 6 OS=Homo sapiens GN=CHST6 PE=1 SV=1//0
XM_007994113.1	1.6372	8.34E-55	5.71E-53	sp|Q4R4I0|TE2IP_MACFA Telomeric repeat-binding factor 2-interacting protein 1 OS=Macaca fascicularis GN=TERF2IP PE=2 SV=1//0
XM_007994132.1	1.2303	0.0025367	0.013502	sp|Q8TE60|ATS18_HUMAN A disintegrin and metalloproteinase with thrombospondin motifs 18 OS=Homo sapiens GN=ADAMTS18 PE=1 SV=3//0
XM_007994180.1	-0.76436	3.78E-05	0.0002594	sp|Q9HAY6|BCDO1_HUMAN Beta,beta-carotene 15,15'-monooxygenase OS=Homo sapiens GN=BCO1 PE=1 SV=1//0
XM_007994189.1	0.80851	2.99E-17	5.14E-16	sp|P37059|DHB2_HUMAN Estradiol 17-beta-dehydrogenase 2 OS=Homo sapiens GN=HSD17B2 PE=1 SV=1//0
XM_007994197.1	-0.71503	1.06E-11	1.31E-10	sp|Q5RDI2|HSBP1_PONAB Heat shock factor-binding protein 1 OS=Pongo abelii GN=HSBP1 PE=3 SV=1//1.62402e-44
XM_007994198.1	1.4294	1.63E-18	3.00E-17	sp|O95822|DCMC_HUMAN Malonyl-CoA decarboxylase, mitochondrial OS=Homo sapiens GN=MLYCD PE=1 SV=3//0
XM_007994199.1	2.7359	2.92E-43	1.40E-41	sp|Q9UJX0|OSGI1_HUMAN Oxidative stress-induced growth inhibitor 1 OS=Homo sapiens GN=OSGIN1 PE=1 SV=3//0
XM_007994241.1	0.85587	5.90E-12	7.43E-11	sp|Q9H0B8|CRLD2_HUMAN Cysteine-rich secretory protein LCCL domain-containing 2 OS=Homo sapiens GN=CRISPLD2 PE=2 SV=1//0
XM_007994243.1	0.90924	4.67E-29	1.39E-27	sp|Q9NXF8|ZDHC7_HUMAN Palmitoyltransferase ZDHHC7 OS=Homo sapiens GN=ZDHHC7 PE=1 SV=2//0
XM_007994261.1	0.50514	0.0023461	0.012561	sp|Q9Y248|PSF2_HUMAN DNA replication complex GINS protein PSF2 OS=Homo sapiens GN=GINS2 PE=1 SV=1//1.76342e-125
XM_007994269.1	2.5181	0.0005318	0.0031675	sp|Q02556|IRF8_HUMAN Interferon regulatory factor 8 OS=Homo sapiens GN=IRF8 PE=1 SV=2//0
XM_007994295.1	0.73023	3.74E-18	6.76E-17	sp|Q9GZQ8|MLP3B_HUMAN Microtubule-associated proteins 1A/1B light chain 3B OS=Homo sapiens GN=MAP1LC3B PE=1 SV=3//2.83382e-76
XM_007994297.1	-0.27978	0.0005941	0.0035073	sp|Q8WYQ9|ZCH14_HUMAN Zinc finger CCHC domain-containing protein 14 OS=Homo sapiens GN=ZCCHC14 PE=2 SV=1//2.27787e-77
XM_007994298.1	-0.70219	1.81E-07	1.58E-06	sp|Q8WXH2|JPH3_HUMAN Junctophilin-3 OS=Homo sapiens GN=JPH3 PE=2 SV=2//0
XM_007994301.1	1.2942	3.01E-22	6.77E-21	sp|Q5R8W1|KLDC4_PONAB Kelch domain-containing protein 4 OS=Pongo abelii GN=KLHDC4 PE=2 SV=1//0
XM_007994317.1	2.2792	1.56E-08	1.51E-07	sp|Q8N9N5|BANP_HUMAN Protein BANP OS=Homo sapiens GN=BANP PE=1 SV=3//0
XM_007994326.1	-0.8405	0.0006599	0.0038687	sp|Q8IX07|FOG1_HUMAN Zinc finger protein ZFPM1 OS=Homo sapiens GN=ZFPM1 PE=1 SV=2//0
XM_007994335.1	-1.7458	5.32E-80	6.78E-78	sp|P13498|CY24A_HUMAN Cytochrome b-245 light chain OS=Homo sapiens GN=CYBA PE=1 SV=3//2.32378e-101
XM_007994358.1	-0.76738	4.34E-10	4.76E-09	sp|P34059|GALNS_HUMAN N-acetylgalactosamine-6-sulfatase OS=Homo sapiens GN=GALNS PE=1 SV=1//0
XM_007994379.1	-1.1716	0.0065961	0.032079	sp|A6NKX4|S22AV_HUMAN Putative solute carrier family 22 member 31 OS=Homo sapiens GN=SLC22A31 PE=3 SV=3//1.69592e-169
XM_007994400.1	-0.62951	4.28E-16	6.91E-15	sp|Q96FW1|OTUB1_HUMAN Ubiquitin thioesterase OTUB1 OS=Homo sapiens GN=OTUB1 PE=1 SV=2//0
XM_007994408.1	-0.50259	4.62E-06	3.55E-05	sp|Q5R605|CHM1A_PONAB Charged multivesicular body protein 1a OS=Pongo abelii GN=CHMP1A PE=2 SV=1//8.60747e-112
XM_007994420.1	-0.4606	9.02E-07	7.44E-06	sp|P26373|RL13_HUMAN 60S ribosomal protein L13 OS=Homo sapiens GN=RPL13 PE=1 SV=4//3.50371e-124
XM_007994451.1	-1.5838	1.92E-06	1.53E-05	sp|Q9H9R9|DBND1_HUMAN Dysbindin domain-containing protein 1 OS=Homo sapiens GN=DBNDD1 PE=1 SV=2//2.09067e-94
XM_007994452.1	1.0518	1.91E-12	2.48E-11	sp|Q920A7|AFG31_MOUSE AFG3-like protein 1 OS=Mus musculus GN=Afg3l1 PE=2 SV=2//1.8048e-56
XM_007994457.1	0.80777	4.12E-09	4.14E-08	sp|O95995|GAS8_HUMAN Growth arrest-specific protein 8 OS=Homo sapiens GN=GAS8 PE=1 SV=1//0
XM_007994460.1	-1.9304	0.00423	0.021467	-//-
XM_007994467.1	1.723	3.85E-65	3.51E-63	sp|Q8NDF8|PAPD5_HUMAN Non-canonical poly(A) RNA polymerase PAPD5 OS=Homo sapiens GN=PAPD5 PE=1 SV=2//0
XM_007994470.1	-2.173	0.0001506	0.0009641	-//-
XM_007994475.1	0.57799	2.03E-06	1.61E-05	sp|Q5R9F5|S38A7_PONAB Putative sodium-coupled neutral amino acid transporter 7 OS=Pongo abelii GN=SLC38A7 PE=2 SV=1//0
XM_007994476.1	0.39511	9.10E-06	6.74E-05	sp|Q8NCN5|PDPR_HUMAN Pyruvate dehydrogenase phosphatase regulatory subunit, mitochondrial OS=Homo sapiens GN=PDPR PE=1 SV=2//0
XM_007994478.1	0.53136	0.0062015	0.030341	sp|Q8VE10|NAA40_MOUSE N-alpha-acetyltransferase 40 OS=Mus musculus

				GN=Naa40 PE=2 SV=1//4.85088e-139
XM_007994488.1	-0.53303	5.26E-07	4.41E-06	sp|Q9UQ90|SPG7_HUMAN Paraplegin OS=Homo sapiens GN=SPG7 PE=1 SV=2//0
XM_007994510.1	-0.64329	7.60E-10	8.12E-09	sp|Q3SX64|OD3L2_HUMAN Outer dense fiber protein 3-like protein 2 OS=Homo sapiens GN=ODF3L2 PE=1 SV=2//0
XM_007994513.1	-1.1165	0.010753	0.049998	sp|Q13477|MADCA_HUMAN Mucosal addressin cell adhesion molecule 1 OS=Homo sapiens GN=MADCAM1 PE=1 SV=2//6.25645e-126
XM_007994514.1	-1.6235	4.36E-05	0.000298	sp|Q6ZTW0|TPGS1_HUMAN Tubulin polyglutamylase complex subunit 1 OS=Homo sapiens GN=TPGS1 PE=2 SV=2//9.81816e-176
XM_007994516.1	-0.44509	0.0002199	0.0013728	sp|Q9BV38|WDR18_HUMAN WD repeat-containing protein 18 OS=Homo sapiens GN=WDR18 PE=1 SV=2//0
XM_007994525.1	0.69939	7.47E-14	1.06E-12	sp|Q9Y2X0|MED16_HUMAN Mediator of RNA polymerase II transcription subunit 16 OS=Homo sapiens GN=MED16 PE=1 SV=2//2.46122e-93
XM_007994527.1	-1.2336	1.59E-05	0.0001144	sp|P00746|CFAD_HUMAN Complement factor D OS=Homo sapiens GN=CFD PE=1 SV=5//4.66844e-161
XM_007994551.1	1.6051	1.08E-71	1.14E-69	sp|O95633|FSTL3_HUMAN Follistatin-related protein 3 OS=Homo sapiens GN=FSTL3 PE=1 SV=1//4.93589e-154
XM_007994557.1	-0.75623	5.03E-15	7.60E-14	sp|P49427|UB2R1_HUMAN Ubiquitin-conjugating enzyme E2 R1 OS=Homo sapiens GN=CDC34 PE=1 SV=2//6.22106e-150
XM_007994572.1	-0.4342	0.0028278	0.014918	sp|Q5R587|RPAB1_PONAB DNA-directed RNA polymerases I, II, and III subunit RPABC1 OS=Pongo abelii GN=POLR2E PE=2 SV=1//2.73126e-134
XM_007994574.1	-1.0957	1.78E-51	1.13E-49	sp|Q4AEH2|GPX4_PONPY Phospholipid hydroperoxide glutathione peroxidase, mitochondrial OS=Pongo pygmaeus GN=GPX4 PE=2 SV=2//1.29415e-118
XM_007994582.1	-0.92344	3.45E-23	8.07E-22	sp|P30049|ATPD_HUMAN ATP synthase subunit delta, mitochondrial OS=Homo sapiens GN=ATP5D PE=1 SV=2//4.57919e-66
XM_007994583.1	2.1323	2.81E-35	1.05E-33	sp|Q8N350|DOS_HUMAN Protein Dos OS=Homo sapiens GN=DOS PE=1 SV=2//0
XM_007994585.1	-0.74142	1.30E-05	9.49E-05	sp|Q15831|STK11_HUMAN Serine/threonine-protein kinase STK11 OS=Homo sapiens GN=STK11 PE=1 SV=1//0
XM_007994590.1	0.42849	0.0001037	0.0006792	sp|Q9BVV8|CS024_HUMAN Uncharacterized membrane protein C19orf24 OS=Homo sapiens GN=C19orf24 PE=1 SV=2//1.35138e-57
XM_007994591.1	-2.5988	1.64E-24	4.04E-23	sp|O43921|EFNA2_HUMAN Ephrin-A2 OS=Homo sapiens GN=EFNA2 PE=1 SV=1//1.14912e-123
XM_007994599.1	-0.80755	0.0082277	0.039282	sp|Q2TAK8|MUM1_HUMAN PWWP domain-containing protein MUM1 OS=Homo sapiens GN=MUM1 PE=1 SV=3//2.82292e-144
XM_007994604.1	-1.9308	0.000367	0.0022308	sp|Q14353|GAMT_HUMAN Guanidinoacetate N-methyltransferase OS=Homo sapiens GN=GAMT PE=1 SV=1//2.24976e-159
XM_007994615.1	-0.79314	0.00737	0.035457	sp|Q96EP5|DAZP1_HUMAN DAZ-associated protein 1 OS=Homo sapiens GN=DAZAP1 PE=1 SV=1//4.40944e-15
XM_007994618.1	0.71432	0.000165	0.001051	sp|Q9UFG5|CS025_HUMAN UPF0449 protein C19orf25 OS=Homo sapiens GN=C19orf25 PE=1 SV=2//4.5391e-62
XM_007994622.1	-1.4073	5.42E-06	4.13E-05	sp|Q6UW60|PCSK4_HUMAN Proprotein convertase subtilisin/kexin type 4 OS=Homo sapiens GN=PCSK4 PE=2 SV=2//0
XM_007994633.1	-0.93285	0.0025427	0.013531	sp|Q6ZMM2|ATL5_HUMAN ADAMTS-like protein 5 OS=Homo sapiens GN=ADAMTSL5 PE=1 SV=3//0
XM_007994635.1	-0.63313	1.44E-08	1.40E-07	sp|O14957|QCR10_HUMAN Cytochrome b-c1 complex subunit 10 OS=Homo sapiens GN=UQCR11 PE=3 SV=1//2.26166e-27
XM_007994671.1	-0.40369	3.68E-05	0.0002533	sp|Q969E2|SCAM4_HUMAN Secretory carrier-associated membrane protein 4 OS=Homo sapiens GN=SCAMP4 PE=2 SV=1//5.90854e-127
XM_007994673.1	-0.46908	2.37E-07	2.05E-06	sp|P78368|KC1G2_HUMAN Casein kinase I isoform gamma-2 OS=Homo sapiens GN=CSNK1G2 PE=1 SV=1//0
XM_007994685.1	-1.4581	8.33E-13	1.10E-11	sp|Q9UK45|LSM7_HUMAN U6 snRNA-associated Sm-like protein LSm7 OS=Homo sapiens GN=LSM7 PE=1 SV=1//9.18191e-61
XM_007994690.1	-0.70134	1.18E-19	2.31E-18	sp|P54368|OAZ1_HUMAN Ornithine decarboxylase antizyme 1 OS=Homo sapiens GN=OAZ1 PE=1 SV=3//2.69006e-136
XM_007994699.1	0.83307	0.0002691	0.00166	sp|Q9NW61|PKHJ1_HUMAN Pleckstrin homology domain-containing family J member 1 OS=Homo sapiens GN=PLEKHJ1 PE=2 SV=1//1.39958e-88
XM_007994705.1	0.58468	4.41E-16	7.09E-15	sp|Q865S1|AP3D1_BOVIN AP-3 complex subunit delta-1 OS=Bos taurus GN=AP3D1 PE=1 SV=2//0
XM_007994716.1	3.7002	0	0	sp|O75293|GA45B_HUMAN Growth arrest and DNA damage-inducible protein GADD45 beta OS=Homo sapiens GN=GADD45B PE=1 SV=1//2.20633e-88
XM_007994721.1	-0.70895	3.32E-05	0.0002301	sp|Q9BRY0|S39A3_HUMAN Zinc transporter ZIP3 OS=Homo sapiens GN=SLC39A3 PE=1 SV=2//0
XM_007994725.1	1.3932	1.00E-08	9.82E-08	sp|Q86TJ5|ZN554_HUMAN Zinc finger protein 554 OS=Homo sapiens GN=ZNF554 PE=2 SV=1//0
XM_007994749.1	1.4715	1.10E-16	1.85E-15	sp|Q15935|ZNF77_HUMAN Zinc finger protein 77 OS=Homo sapiens GN=ZNF77 PE=2 SV=2//2.72373e-13
XM_007994750.1	-5.3602	3.59E-07	3.06E-06	sp|P63003|AES_RAT Amino-terminal enhancer of split OS=Rattus norvegicus GN=Aes PE=2 SV=1//7.69804e-101
XM_007994753.1	-0.87018	1.43E-21	3.07E-20	sp|P29992|GNA11_HUMAN Guanine nucleotide-binding protein subunit alpha-11 OS=Homo sapiens GN=GNA11 PE=1 SV=2//0
XM_007994758.1	-3.9292	4.84E-05	0.000329	sp|Q8N6W0|CELF5_HUMAN CUGBP Elav-like family member 5 OS=Homo sapiens

				GN=CELF5 PE=1 SV=1//2.96396e-48
XM_007994759.1	-2.3294	4.23E-06	3.26E-05	sp|Q8N6W0|CELF5_HUMAN CUGBP Elav-like family member 5 OS=Homo sapiens GN=CELF5 PE=1 SV=1//6.44538e-47
XM_007994784.1	-1.1454	0.0040579	0.020656	sp|Q9P0W2|HM20B_HUMAN SWI/SNF-related matrix-associated actin-dependent regulator of chromatin subfamily E member 1-related OS=Homo sapiens GN=HMG20B PE=1 SV=1//1.19943e-145
XM_007994791.1	-1.9323	0.0034033	0.017606	sp|P56486|TA2R_CHLAE Thromboxane A2 receptor OS=Chlorocebus aethiops GN=TBXA2R PE=2 SV=1//0
XM_007994811.1	-0.42814	5.75E-07	4.81E-06	sp|Q5R611|HRSL3_PONAB HRAS-like suppressor 3 OS=Pongo abelii GN=PLA2G16 PE=2 SV=1//2.29684e-82
XM_007994832.1	-0.38192	0.0041743	0.021199	sp|Q99961|SH3G1_HUMAN Endophilin-A2 OS=Homo sapiens GN=SH3GL1 PE=1 SV=1//0
XM_007994833.1	-1.3327	4.73E-08	4.35E-07	sp|Q3TV65|MPND_MOUSE MPN domain-containing protein OS=Mus musculus GN=Mpnd PE=2 SV=2//0
XM_007994863.1	2.0682	0.0048343	0.024187	sp|Q96DT0|LEG12_HUMAN Galectin-12 OS=Homo sapiens GN=LGALS12 PE=1 SV=1//0
XM_007994866.1	-0.81328	1.32E-25	3.46E-24	sp|P36507|MP2K2_HUMAN Dual specificity mitogen-activated protein kinase kinase 2 OS=Homo sapiens GN=MAP2K2 PE=1 SV=1//0
XM_007994881.1	0.3346	0.0002464	0.0015292	sp|Q86TI2|DPP9_HUMAN Dipeptidyl peptidase 9 OS=Homo sapiens GN=DPP9 PE=1 SV=3//0
XM_007994882.1	1.3862	2.04E-44	1.02E-42	sp|Q9BSK4|FEM1A_HUMAN Protein fem-1 homolog A OS=Homo sapiens GN=FEM1A PE=1 SV=1//0
XM_007994883.1	1.9536	8.27E-51	5.18E-49	sp|Q8IUC6|TCAM1_HUMAN TIR domain-containing adapter molecule 1 OS=Homo sapiens GN=TICAM1 PE=1 SV=1//0
XM_007994933.1	-0.6496	3.48E-20	6.96E-19	sp|Q5RAZ9|RL36_PONAB 60S ribosomal protein L36 OS=Pongo abelii GN=RPL36 PE=3 SV=3//2.98786e-61
XM_007994938.1	1.0481	1.83E-19	3.54E-18	sp|Q96G46|DUS3L_HUMAN tRNA-dihydrouridine(47) synthase [NAD(P)(+)]-like OS=Homo sapiens GN=DUS3L PE=1 SV=2//0
XM_007994941.1	-2.4139	4.04E-13	5.44E-12	sp|Q99748|NRTN_HUMAN Neurturin OS=Homo sapiens GN=NRTN PE=1 SV=1//6.75559e-64
XM_007994957.1	-2.1677	5.24E-19	9.89E-18	sp|Q13938|CAYP1_HUMAN Calcyphosin OS=Homo sapiens GN=CAPS PE=1 SV=1//3.63293e-125
XM_007994958.1	-0.76265	1.06E-15	1.67E-14	sp|Q0MQC0|NDUAB_PANTR NADH dehydrogenase [ubiquinone] 1 alpha subcomplex subunit 11 OS=Pan troglodytes GN=NDUFA11 PE=2 SV=3//2.83413e-81
XM_007994971.1	-1.5697	3.42E-16	5.54E-15	sp|Q9BT30|ALKB7_HUMAN Alpha-ketoglutarate-dependent dioxygenase alkB homolog 7, mitochondrial OS=Homo sapiens GN=ALKBH7 PE=1 SV=1//7.09125e-122
XM_007994972.1	-0.48659	6.23E-05	0.0004186	sp|Q16740|CLPP_HUMAN ATP-dependent Clp protease proteolytic subunit, mitochondrial OS=Homo sapiens GN=CLPP PE=1 SV=1//6.95513e-171
XM_007994985.1	-1.5149	4.60E-13	6.17E-12	sp|Q8IV53|DEN1C_HUMAN DENN domain-containing protein 1C OS=Homo sapiens GN=DENND1C PE=1 SV=1//0
XM_007994996.1	-2.1929	2.87E-20	5.78E-19	sp|Q9D6F9|TBB4A_MOUSE Tubulin beta-4A chain OS=Mus musculus GN=Tubb4a PE=1 SV=3//0
XM_007994997.1	-2.8445	1.84E-31	5.96E-30	sp|Q9D6F9|TBB4A_MOUSE Tubulin beta-4A chain OS=Mus musculus GN=Tubb4a PE=1 SV=3//0
XM_007994998.1	-0.56971	0.0019256	0.010463	sp|P41273|TNFL9_HUMAN Tumor necrosis factor ligand superfamily member 9 OS=Homo sapiens GN=TNFSF9 PE=1 SV=1//8.68543e-104
XM_007995014.1	-1.0928	0.0022557	0.012107	sp|P15498|VAV_HUMAN Proto-oncogene vav OS=Homo sapiens GN=VAV1 PE=1 SV=4//0
XM_007995060.1	0.35441	0.0023508	0.012583	sp|Q9HCS7|SYF1_HUMAN Pre-mRNA-splicing factor SYF1 OS=Homo sapiens GN=XAB2 PE=1 SV=2//0
XM_007995061.1	-0.60109	6.54E-07	5.45E-06	sp|Q15833|STXB2_HUMAN Syntaxin-binding protein 2 OS=Homo sapiens GN=STXBP2 PE=1 SV=2//0
XM_007995065.1	-1.8495	3.45E-07	2.94E-06	sp|P0DJ07|PT100_HUMAN Protein PET100 homolog, mitochondrial OS=Homo sapiens GN=PET100 PE=1 SV=1//4.22855e-28
XM_007995087.1	1.3489	4.52E-46	2.46E-44	sp|Q6RFH5|WDR74_HUMAN WD repeat-containing protein 74 OS=Homo sapiens GN=WDR74 PE=1 SV=1//0
XM_007995107.1	0.64285	4.30E-11	5.09E-10	sp|O43615|TIM44_HUMAN Mitochondrial import inner membrane translocase subunit TIM44 OS=Homo sapiens GN=TIMM44 PE=1 SV=2//0
XM_007995108.1	5.4814	8.82E-08	7.92E-07	sp|P08195|4F2_HUMAN 4F2 cell-surface antigen heavy chain OS=Homo sapiens GN=SLC3A2 PE=1 SV=3//0
XM_007995111.1	0.24672	0.0039471	0.02013	sp|Q15717|ELAV1_HUMAN ELAV-like protein 1 OS=Homo sapiens GN=ELAVL1 PE=1 SV=2//0
XM_007995117.1	-0.76057	0.0003105	0.0018995	sp|P62859|RS28_RAT 40S ribosomal protein S28 OS=Rattus norvegicus GN=Rps28 PE=1 SV=1//2.34819e-31
XM_007995124.1	-1.0464	8.77E-25	2.20E-23	sp|O35509|RB11B_RAT Ras-related protein Rab-11B OS=Rattus norvegicus GN=Rab11b PE=1 SV=4//2.77415e-155
XM_007995136.1	#NAME?	0.0045066	0.022734	sp|O08750|NFIL3_MOUSE Nuclear factor interleukin-3-regulated protein OS=Mus musculus GN=Nfil3 PE=1 SV=1//1.09027e-26

XM_007995142.1	0.45151	0.0035115	0.018114	sp|Q96NG5|ZN558_HUMAN Zinc finger protein 558 OS=Homo sapiens GN=ZNF558 PE=2 SV=1//0
XM_007995149.1	1.5491	2.92E-11	3.50E-10	sp|Q96PQ6|ZN317_HUMAN Zinc finger protein 317 OS=Homo sapiens GN=ZNF317 PE=1 SV=2//0
XM_007995151.1	0.66118	3.54E-10	3.90E-09	sp|Q9UBU9|NXF1_HUMAN Nuclear RNA export factor 1 OS=Homo sapiens GN=NXF1 PE=1 SV=1//0
XM_007995161.1	0.38953	0.0091267	0.043123	sp|A0PJW6|TM223_HUMAN Transmembrane protein 223 OS=Homo sapiens GN=TMEM223 PE=1 SV=1//1.35668e-114
XM_007995192.1	-1.1561	2.98E-31	9.57E-30	sp|Q791B0|UBL5_PSAOB Ubiquitin-like protein 5 OS=Psammomys obesus GN=UBL5 PE=3 SV=1//5.28142e-47
XM_007995195.1	-0.52253	0.0003752	0.0022771	sp|Q13526|PIN1_HUMAN Peptidyl-prolyl cis-trans isomerase NIMA-interacting 1 OS=Homo sapiens GN=PIN1 PE=1 SV=1//7.66392e-102
XM_007995200.1	1.8436	9.27E-12	1.15E-10	sp|Q96G91|P2Y11_HUMAN P2Y purinoceptor 11 OS=Homo sapiens GN=P2RY11 PE=2 SV=2//0
XM_007995212.1	-1.6883	6.90E-09	6.82E-08	sp|O95136|S1PR2_HUMAN Sphingosine 1-phosphate receptor 2 OS=Homo sapiens GN=S1PR2 PE=1 SV=2//0
XM_007995219.1	2.498	5.18E-18	9.29E-17	sp|Q5NKV6|ICAM1_MACMU Intercellular adhesion molecule 1 OS=Macaca mulatta GN=ICAM1 PE=2 SV=1//0
XM_007995227.1	-0.57924	7.77E-14	1.10E-12	sp|Q16543|CDC37_HUMAN Hsp90 co-chaperone Cdc37 OS=Homo sapiens GN=CDC37 PE=1 SV=1//0
XM_007995228.1	2.0699	8.14E-05	0.0005411	sp|Q5R774|KEAP1_PONAB Kelch-like ECH-associated protein 1 OS=Pongo abelii GN=KEAP1 PE=2 SV=1//0
XM_007995239.1	1.1123	5.91E-18	1.06E-16	sp|Q86TL0|ATG4D_HUMAN Cysteine protease ATG4D OS=Homo sapiens GN=ATG4D PE=1 SV=1//0
XM_007995248.1	-0.56646	0.001889	0.010277	sp|Q13445|TMED1_HUMAN Transmembrane emp24 domain-containing protein 1 OS=Homo sapiens GN=TMED1 PE=1 SV=1//1.46171e-125
XM_007995254.1	-0.38461	1.25E-05	9.15E-05	sp|Q1KMD3|HNRL2_HUMAN Heterogeneous nuclear ribonucleoprotein U-like protein 2 OS=Homo sapiens GN=HNRNPUL2 PE=1 SV=1//4.63235e-13
XM_007995261.1	-0.67122	7.62E-14	1.08E-12	sp|Q86X55|CARM1_HUMAN Histone-arginine methyltransferase CARM1 OS=Homo sapiens GN=CARM1 PE=1 SV=3//0
XM_007995275.1	1.8648	5.03E-06	3.85E-05	sp|Q6UXH0|BETAT_HUMAN Angiopoietin-like protein 8 OS=Homo sapiens GN=C19orf80 PE=1 SV=1//1.72009e-112
XM_007995288.1	-2.4944	0.006247	0.030544	sp|Q96G97|BSCL2_HUMAN Seipin OS=Homo sapiens GN=BSCL2 PE=1 SV=3//0
XM_007995291.1	-2.0258	5.31E-72	5.65E-70	sp|O95716|RAB3D_HUMAN Ras-related protein Rab-3D OS=Homo sapiens GN=RAB3D PE=1 SV=1//9.1343e-121
XM_007995323.1	-0.37048	0.0047722	0.02392	sp|Q4R5Q4|ECSIT_MACFA Evolutionarily conserved signaling intermediate in Toll pathway, mitochondrial OS=Macaca fascicularis GN=ECSIT PE=2 SV=1//0
XM_007995328.1	1.9412	3.05E-64	2.73E-62	sp|Q7YRL2|CNN1_SHEEP Calponin-1 OS=Ovis aries GN=CNN1 PE=2 SV=1//0
XM_007995340.1	0.80144	9.77E-12	1.21E-10	sp|Q7L945|ZN627_HUMAN Zinc finger protein 627 OS=Homo sapiens GN=ZNF627 PE=1 SV=1//0
XM_007995377.1	3.0714	3.70E-11	4.40E-10	sp|P15621|ZNF44_HUMAN Zinc finger protein 44 OS=Homo sapiens GN=ZNF44 PE=2 SV=3//0
XM_007995381.1	1.1838	5.73E-11	6.70E-10	sp|Q8N972|ZN709_HUMAN Zinc finger protein 709 OS=Homo sapiens GN=ZNF709 PE=2 SV=1//4.82339e-07
XM_007995386.1	-0.60331	1.43E-07	1.26E-06	sp|Q9D937|CK098_MOUSE Uncharacterized protein C11orf98 homolog OS=Mus musculus PE=2 SV=1//7.13957e-48
XM_007995395.1	1.0059	4.02E-11	4.76E-10	sp|Q8TBZ8|ZN564_HUMAN Zinc finger protein 564 OS=Homo sapiens GN=ZNF564 PE=1 SV=1//0
XM_007995402.1	0.84202	2.45E-07	2.12E-06	sp|Q9BRX9|WDR83_HUMAN WD repeat domain-containing protein 83 OS=Homo sapiens GN=WDR83 PE=1 SV=1//0
XM_007995403.1	-0.32768	0.0005576	0.0033085	sp|Q6ZWX0|ASTER_MOUSE Protein Asterix OS=Mus musculus GN=Wdr83os PE=3 SV=1//1.14078e-69
XM_007995409.1	0.92095	0.0007357	0.0042844	sp|Q9BQE6|LBHD1_HUMAN LBH domain-containing protein 1 OS=Homo sapiens GN=LBHD1 PE=1 SV=3//9.98297e-64
XM_007995417.1	-0.72	1.12E-15	1.76E-14	sp|Q9BQ61|CS043_HUMAN Uncharacterized protein C19orf43 OS=Homo sapiens GN=C19orf43 PE=1 SV=1//4.47134e-51
XM_007995418.1	-0.66315	1.93E-12	2.50E-11	sp|O43681|ASNA_HUMAN ATPase ASNA1 OS=Homo sapiens GN=ASNA1 PE=1 SV=2//0
XM_007995423.1	-0.65866	4.63E-08	4.27E-07	sp|Q6UW78|UQCC3_HUMAN Ubiquinol-cytochrome-c reductase complex assembly factor 3 OS=Homo sapiens GN=UQCC3 PE=1 SV=2//1.30345e-26
XM_007995426.1	2.0746	5.01E-70	5.13E-68	sp|P17275|JUNB_HUMAN Transcription factor jun-B OS=Homo sapiens GN=JUNB PE=1 SV=1//9.63791e-166
XM_007995427.1	-1.333	2.53E-54	1.71E-52	sp|Q2PFZ3|PRDX2_MACFA Peroxiredoxin-2 OS=Macaca fascicularis GN=PRDX2 PE=2 SV=3//4.37183e-142
XM_007995428.1	-0.57548	1.11E-06	9.07E-06	sp|O75792|RNH2A_HUMAN Ribonuclease H2 subunit A OS=Homo sapiens GN=RNASEH2A PE=1 SV=2//0
XM_007995434.1	0.98198	5.15E-23	1.19E-21	sp|Q6P9B9|INT5_HUMAN Integrator complex subunit 5 OS=Homo sapiens GN=INTS5 PE=1 SV=1//0
XM_007995448.1	-0.57523	1.57E-17	2.74E-16	sp|Q4R4N7|GANAB_MACFA Neutral alpha-glucosidase AB OS=Macaca fascicularis GN=GANAB PE=2 SV=1//0

XM_007995451.1	1.2301	3.91E-39	1.66E-37	sp|Q5RFA2|SYFA_PONAB Phenylalanine--tRNA ligase alpha subunit OS=Pongo abelii GN=FARSA PE=2 SV=1//0
XM_007995452.1	-0.30522	2.82E-05	0.0001969	sp|P27797|CALR_HUMAN Calreticulin OS=Homo sapiens GN=CALR PE=1 SV=1//0
XM_007995455.1	-0.65465	1.34E-09	1.41E-08	sp|Q8SPE7|G45IP_CHLAE Growth arrest and DNA damage-inducible proteins-interacting protein 1 OS=Chlorocebus aethiops GN=GADD45GIP1 PE=2 SV=1//5.13613e-88
XM_007995471.1	-0.51944	9.76E-09	9.56E-08	sp|Q96AD5|PLPL2_HUMAN Patatin-like phospholipase domain-containing protein 2 OS=Homo sapiens GN=PNPLA2 PE=1 SV=1//0
XM_007995473.1	1.1774	2.52E-24	6.14E-23	sp|Q9NXH9|TRM1_HUMAN tRNA (guanine(26)-N(2))-dimethyltransferase OS=Homo sapiens GN=TRMT1 PE=1 SV=1//0
XM_007995477.1	4.3007	0	0	sp|Q9BTL4|IER2_HUMAN Immediate early response gene 2 protein OS=Homo sapiens GN=IER2 PE=1 SV=1//4.44259e-93
XM_007995482.1	-1.0215	2.23E-26	5.99E-25	sp|Q5REM2|L10K_PONAB Leydig cell tumor 10 kDa protein homolog OS=Pongo abelii PE=3 SV=1//7.25187e-29
XM_007995509.1	-1.56	5.23E-25	1.33E-23	sp|Q6UWB1|I27RA_HUMAN Interleukin-27 receptor subunit alpha OS=Homo sapiens GN=IL27RA PE=2 SV=2//0
XM_007995511.1	-2.4712	0.0001335	0.0008609	sp|Q96FF7|YS003_HUMAN Uncharacterized protein LOC113230 OS=Homo sapiens PE=2 SV=4//5.97806e-48
XM_007995524.1	0.76003	1.25E-21	2.69E-20	sp|Q8VDW0|DX39A_MOUSE ATP-dependent RNA helicase DDX39A OS=Mus musculus GN=Ddx39a PE=2 SV=1//0
XM_007995541.1	-1.5779	4.41E-19	8.38E-18	sp|Q0MQE2|NDUB7_PONPY NADH dehydrogenase [ubiquinone] 1 beta subcomplex subunit 7 OS=Pongo pygmaeus GN=NDUFB7 PE=2 SV=3//1.47978e-69
XM_007995546.1	-1.2284	0.0040911	0.020806	sp|Q03395|ROM1_HUMAN Rod outer segment membrane protein 1 OS=Homo sapiens GN=ROM1 PE=1 SV=2//4.6125e-152
XM_007995558.1	-0.36594	8.73E-09	8.58E-08	sp|Q4R7H5|EF1G_MACFA Elongation factor 1-gamma OS=Macaca fascicularis GN=EEF1G PE=2 SV=1//0
XM_007995571.1	0.92151	1.90E-15	2.94E-14	sp|Q9H6E5|STPAP_HUMAN Speckle targeted PIP5K1A-regulated poly(A) polymerase OS=Homo sapiens GN=TUT1 PE=1 SV=2//0
XM_007995573.1	-2.7136	2.15E-92	3.44E-90	sp|Q9UM47|NOTC3_HUMAN Neurogenic locus notch homolog protein 3 OS=Homo sapiens GN=NOTCH3 PE=1 SV=2//0
XM_007995579.1	0.53979	8.43E-10	8.98E-09	sp|O60885|BRD4_HUMAN Bromodomain-containing protein 4 OS=Homo sapiens GN=BRD4 PE=1 SV=2//0
XM_007995580.1	1.1312	8.01E-08	7.23E-07	sp|O60885|BRD4_HUMAN Bromodomain-containing protein 4 OS=Homo sapiens GN=BRD4 PE=1 SV=2//0
XM_007995581.1	0.90332	3.04E-22	6.81E-21	sp|Q9ULX6|AKP8L_HUMAN A-kinase anchor protein 8-like OS=Homo sapiens GN=AKAP8L PE=1 SV=3//0
XM_007995626.1	-0.53507	2.65E-09	2.71E-08	sp|Q4R7U8|ASGL1_MACFA Isoaspartyl peptidase/L-asparaginase OS=Macaca fascicularis GN=ASRGL1 PE=2 SV=1//0
XM_007995629.1	-1.328	3.33E-11	3.98E-10	sp|P67937|TPM4_PIG Tropomyosin alpha-4 chain OS=Sus scrofa GN=TPM4 PE=2 SV=3//1.23589e-84
XM_007995635.1	-1.2999	2.67E-41	1.21E-39	sp|Q5R9E5|FA32A_PONAB Protein FAM32A OS=Pongo abelii GN=FAM32A PE=3 SV=1//6.69613e-60
XM_007995656.1	2.1503	3.27E-61	2.64E-59	sp|O95402|MED26_HUMAN Mediator of RNA polymerase II transcription subunit 26 OS=Homo sapiens GN=MED26 PE=1 SV=2//0
XM_007995658.1	-0.68745	3.02E-07	2.59E-06	sp|Q9H6F2|TM38A_HUMAN Trimeric intracellular cation channel type A OS=Homo sapiens GN=TMEM38A PE=1 SV=1//1.89012e-167
XM_007995665.1	-2.2579	0.0086019	0.040899	sp|Q8IZJ3|CPMD8_HUMAN C3 and PZP-like alpha-2-macroglobulin domain-containing protein 8 OS=Homo sapiens GN=CPAMD8 PE=1 SV=2//0
XM_007995684.1	-0.43492	0.0035494	0.018289	sp|Q9NZ43|USE1_HUMAN Vesicle transport protein USE1 OS=Homo sapiens GN=USE1 PE=1 SV=2//0
XM_007995689.1	-4.2167	2.40E-08	2.28E-07	-//-
XM_007995690.1	-1.7098	6.83E-06	5.14E-05	sp|P10588|NR2F6_HUMAN Nuclear receptor subfamily 2 group F member 6 OS=Homo sapiens GN=NR2F6 PE=1 SV=2//0
XM_007995711.1	-0.40253	0.0008341	0.004819	sp|Q4R584|ABHD8_MACFA Abhydrolase domain-containing protein 8 OS=Macaca fascicularis GN=ABHD8 PE=2 SV=1//0
XM_007995712.1	-0.36188	0.0038501	0.019699	sp|Q4R319|RM34_MACFA 39S ribosomal protein L34, mitochondrial OS=Macaca fascicularis GN=MRPL34 PE=3 SV=1//4.14367e-54
XM_007995714.1	0.52683	4.63E-08	4.26E-07	sp|Q5RD86|DDA1_PONAB DET1- and DDB1-associated protein 1 OS=Pongo abelii GN=DDA1 PE=3 SV=1//7.83664e-68
XM_007995717.1	0.6689	0.0017999	0.0098462	sp|Q969Y2|GTPB3_HUMAN tRNA modification GTPase GTPBP3, mitochondrial OS=Homo sapiens GN=GTPBP3 PE=1 SV=2//2.39518e-61
XM_007995718.1	-0.6529	2.76E-08	2.60E-07	sp|Q9HCE9|ANO8_HUMAN Anoctamin-8 OS=Homo sapiens GN=ANO8 PE=1 SV=3//0
XM_007995721.1	1.0586	1.53E-06	1.23E-05	sp|Q10589|BST2_HUMAN Bone marrow stromal antigen 2 OS=Homo sapiens GN=BST2 PE=1 SV=1//1.98267e-62
XM_007995722.1	-0.46848	0.0001504	0.0009631	sp|Q96EY5|MB12A_HUMAN Multivesicular body subunit 12A OS=Homo sapiens GN=MVB12A PE=1 SV=1//7.01319e-177
XM_007995727.1	2.5611	0.0079323	0.037954	sp|O95336|6PGL_HUMAN 6-phosphogluconolactonase OS=Homo sapiens GN=PGLS PE=1 SV=2//2.00649e-141
XM_007995729.1	-0.55249	4.63E-10	5.06E-09	sp|Q8NBJ5|GT251_HUMAN Procollagen galactosyltransferase 1 OS=Homo sapiens GN=COLGALT1 PE=1 SV=1//0

XM_007995737.1	0.68383	5.46E-12	6.90E-11	sp|Q66K74|MAP1S_HUMAN Microtubule-associated protein 1S OS=Homo sapiens GN=MAP1S PE=1 SV=2//0
XM_007995747.1	-1.1129	0.0034728	0.017928	sp|Q9Y2A9|B3GN3_HUMAN N-acetyllactosaminide beta-1,3-N-acetylglucosaminyltransferase 3 OS=Homo sapiens GN=B3GNT3 PE=1 SV=2//0
XM_007995754.1	-0.78435	1.28E-13	1.79E-12	sp|Q96CT7|CC124_HUMAN Coiled-coil domain-containing protein 124 OS=Homo sapiens GN=CCDC124 PE=1 SV=1//2.3387e-114
XM_007995778.1	-1.2995	1.76E-52	1.14E-50	sp|P13284|GILT_HUMAN Gamma-interferon-inducible lysosomal thiol reductase OS=Homo sapiens GN=IFI30 PE=1 SV=3//6.97537e-155
XM_007995782.1	-1.9624	0.0040661	0.020688	sp|Q08493|PDE4C_HUMAN cAMP-specific 3',5'-cyclic phosphodiesterase 4C OS=Homo sapiens GN=PDE4C PE=1 SV=2//0
XM_007995793.1	3.2386	0	0	sp|Q99988|GDF15_HUMAN Growth/differentiation factor 15 OS=Homo sapiens GN=GDF15 PE=2 SV=3//1.60394e-162
XM_007995805.1	-1.4439	1.84E-83	2.52E-81	sp|Q4R6E3|INO1_MACFA Inositol-3-phosphate synthase 1 OS=Macaca fascicularis GN=ISYNA1 PE=2 SV=1//0
XM_007995806.1	1.8737	2.59E-71	2.71E-69	sp|P55199|ELL_HUMAN RNA polymerase II elongation factor ELL OS=Homo sapiens GN=ELL PE=1 SV=1//0
XM_007995820.1	-1.0879	8.50E-14	1.20E-12	sp|Q96EN9|CS060_HUMAN Uncharacterized protein C19orf60 OS=Homo sapiens GN=C19orf60 PE=1 SV=1//3.93138e-94
XM_007995825.1	-1.2557	1.03E-05	7.57E-05	sp|Q9UK28|TM59L_HUMAN Transmembrane protein 59-like OS=Homo sapiens GN=TMEM59L PE=2 SV=1//0
XM_007995827.1	2.8874	4.39E-05	0.0002999	sp|Q53HC5|KLH26_HUMAN Kelch-like protein 26 OS=Homo sapiens GN=KLHL26 PE=1 SV=2//0
XM_007995832.1	1.0808	1.76E-24	4.33E-23	sp|Q9Y5Q0|FADS3_HUMAN Fatty acid desaturase 3 OS=Homo sapiens GN=FADS3 PE=2 SV=1//0
XM_007995842.1	1.9635	1.22E-36	4.71E-35	sp|Q9Y6V7|DDX49_HUMAN Probable ATP-dependent RNA helicase DDX49 OS=Homo sapiens GN=DDX49 PE=1 SV=1//0
XM_007995843.1	-0.54234	4.56E-10	4.99E-09	sp|O14579|COPE_HUMAN Coatomer subunit epsilon OS=Homo sapiens GN=COPE PE=1 SV=3//0
XM_007995856.1	-3.0252	1.06E-05	7.81E-05	sp|Q8IX01|SUGP2_HUMAN SURP and G-patch domain-containing protein 2 OS=Homo sapiens GN=SUGP2 PE=1 SV=2//0
XM_007995860.1	-1.4774	0.0021535	0.011604	sp|Q8IX01|SUGP2_HUMAN SURP and G-patch domain-containing protein 2 OS=Homo sapiens GN=SUGP2 PE=1 SV=2//0
XM_007995889.1	1.2301	1.21E-29	3.66E-28	sp|Q4R5U5|FEN1_MACFA Flap endonuclease 1 OS=Macaca fascicularis GN=FEN1 PE=2 SV=1//0
XM_007995895.1	1.198	1.30E-07	1.15E-06	sp|Q4R4C7|ZNF14_MACFA Zinc finger protein 14 OS=Macaca fascicularis GN=ZNF14 PE=2 SV=1//4.04287e-87
XM_007995914.1	-1.0933	0.0031668	0.016519	sp|A4UVI1|FADS1_PAPAN Fatty acid desaturase 1 OS=Papio anubis GN=FADS1 PE=1 SV=1//0
XM_007995925.1	-1.2035	5.86E-30	1.81E-28	sp|Q4R6H1|NDUAD_MACFA NADH dehydrogenase [ubiquinone] 1 alpha subcomplex subunit 13 OS=Macaca fascicularis GN=NDUFA13 PE=2 SV=1//3.52007e-113
XM_007995926.1	0.95184	0.0005885	0.0034791	sp|Q9BXA6|TSSK6_HUMAN Testis-specific serine/threonine-protein kinase 6 OS=Homo sapiens GN=TSSK6 PE=1 SV=1//5.16978e-08
XM_007995938.1	0.37981	0.0038891	0.019871	sp|Q5JVG8|ZN506_HUMAN Zinc finger protein 506 OS=Homo sapiens GN=ZNF506 PE=2 SV=2//1.31382e-24
XM_007995939.1	-1.9364	4.69E-10	5.13E-09	sp|O75346|ZN253_HUMAN Zinc finger protein 253 OS=Homo sapiens GN=ZNF253 PE=2 SV=2//8.19461e-06
XM_007996027.1	-1.3107	0.000131	0.0008459	sp|Q2NL98|VMAC_HUMAN Vimentin-type intermediate filament-associated coiled-coil protein OS=Homo sapiens GN=VMAC PE=2 SV=1//1.69284e-27
XM_007996030.1	0.3852	0.001318	0.0074003	sp|Q8N344|MIER2_HUMAN Mesoderm induction early response protein 2 OS=Homo sapiens GN=MIER2 PE=1 SV=2//0
XM_007996031.1	-1.7544	8.88E-35	3.28E-33	sp|Q9UL51|HCN2_HUMAN Potassium/sodium hyperpolarization-activated cyclic nucleotide-gated channel 2 OS=Homo sapiens GN=HCN2 PE=1 SV=3//0
XM_007996032.1	0.51902	1.60E-07	1.40E-06	sp|O00411|RPOM_HUMAN DNA-directed RNA polymerase, mitochondrial OS=Homo sapiens GN=POLRMT PE=1 SV=2//2.30719e-104
XM_007996036.1	-1.839	9.16E-22	1.99E-20	sp|O95996|APC2_HUMAN Adenomatous polyposis coli protein 2 OS=Homo sapiens GN=APC2 PE=1 SV=1//0
XM_007996037.1	-1.3952	9.51E-58	6.97E-56	sp|Q86XN8|MEX3D_HUMAN RNA-binding protein MEX3D OS=Homo sapiens GN=MEX3D PE=1 SV=3//0
XM_007996039.1	-2.1847	3.88E-10	4.27E-09	sp|Q9BX70|BTBD2_HUMAN BTB/POZ domain-containing protein 2 OS=Homo sapiens GN=BTBD2 PE=1 SV=1//6.10236e-50
XM_007996042.1	-2.3301	7.77E-05	0.0005174	-//-
XM_007996043.1	-1.5897	2.69E-16	4.39E-15	sp|Q04725|TLE2_HUMAN Transducin-like enhancer protein 2 OS=Homo sapiens GN=TLE2 PE=1 SV=2//5.01876e-57
XM_007996044.1	-1.3937	3.92E-09	3.96E-08	sp|Q95049|ZO3_HUMAN Tight junction protein ZO-3 OS=Homo sapiens GN=TJP3 PE=1 SV=3//0
XM_007996045.1	0.8588	2.22E-12	2.88E-11	sp|Q8N2W9|PIAS4_HUMAN E3 SUMO-protein ligase PIAS4 OS=Homo sapiens GN=PIAS4 PE=1 SV=1//0
XM_007996047.1	-0.7067	0.0007373	0.0042923	sp|Q9H3T3|SEM6B_HUMAN Semaphorin-6B OS=Homo sapiens GN=SEMA6B PE=1 SV=4//4.66083e-54

XM_007996050.1	-0.86053	9.40E-20	1.84E-18	sp|Q9H3T3|SEM6B_HUMAN Semaphorin-6B OS=Homo sapiens GN=SEMA6B PE=1 SV=4//1.28988e-95
XM_007996051.1	0.38238	0.0008466	0.0048858	sp|Q969H8|MYDGF_HUMAN Myeloid-derived growth factor OS=Homo sapiens GN=MYDGF PE=1 SV=1//4.0039e-70
XM_007996052.1	-1.0919	1.88E-33	6.58E-32	sp|O60664|PLIN3_HUMAN Perilipin-3 OS=Homo sapiens GN=PLIN3 PE=1 SV=3//3.13751e-67
XM_007996054.1	1.0673	5.26E-24	1.27E-22	sp|Q96T88|UHRF1_HUMAN E3 ubiquitin-protein ligase UHRF1 OS=Homo sapiens GN=UHRF1 PE=1 SV=1//0
XM_007996059.1	-1.6821	2.43E-09	2.49E-08	sp|P01024|CO3_HUMAN Complement C3 OS=Homo sapiens GN=C3 PE=1 SV=2//0
XM_007996066.1	-1.3117	2.60E-08	2.47E-07	sp|Q9P0N8|MARH2_HUMAN E3 ubiquitin-protein ligase MARCH2 OS=Homo sapiens GN=MARCH2 PE=1 SV=1//2.21548e-78
XM_007996068.1	-1.3322	0.0033126	0.01721	sp|Q96QH2|PRAM_HUMAN PML-RARA-regulated adapter molecule 1 OS=Homo sapiens GN=PRAM1 PE=1 SV=2//0
XM_007996079.1	-1.3367	5.96E-10	6.43E-09	sp|P27815|PDE4A_HUMAN cAMP-specific 3',5'-cyclic phosphodiesterase 4A OS=Homo sapiens GN=PDE4A PE=1 SV=3//4.15697e-31
XM_007996080.1	0.78348	1.46E-05	0.0001057	sp|Q9BXR0|TGT_HUMAN Queuine tRNA-ribosyltransferase OS=Homo sapiens GN=QTRT1 PE=1 SV=3//0
XM_007996081.1	-0.4292	0.010401	0.048497	sp|Q9BWQ6|YIPF2_HUMAN Protein YIPF2 OS=Homo sapiens GN=YIPF2 PE=1 SV=1//5.30397e-123
XM_007996082.1	0.92381	2.52E-37	9.99E-36	sp|P01130|LDLR_HUMAN Low-density lipoprotein receptor OS=Homo sapiens GN=LDLR PE=1 SV=1//0
XM_007996085.1	1.5273	2.15E-09	2.22E-08	sp|Q8N8Z8|ZN441_HUMAN Zinc finger protein 441 OS=Homo sapiens GN=ZNF441 PE=2 SV=2//8.32022e-09
XM_007996086.1	2.1033	3.11E-22	6.98E-21	sp|Q5REI6|ZN791_PONAB Zinc finger protein 791 OS=Pongo abelii GN=ZNF791 PE=2 SV=1//3.71291e-97
XM_007996088.1	1.8629	1.94E-19	3.76E-18	sp|O00555|CAC1A_HUMAN Voltage-dependent P/Q-type calcium channel subunit alpha-1A OS=Homo sapiens GN=CACNA1A PE=1 SV=2//3.35814e-06
XM_007996089.1	1.1748	9.46E-16	1.49E-14	sp|P13994|CC130_HUMAN Coiled-coil domain-containing protein 130 OS=Homo sapiens GN=CCDC130 PE=1 SV=2//0
XM_007996090.1	1.284	5.00E-42	2.31E-40	sp|Q8C7B8|ZSWM4_MOUSE Zinc finger SWIM domain-containing protein 4 OS=Mus musculus GN=Zswim4 PE=2 SV=2//0
XM_007996091.1	0.54958	6.42E-06	4.85E-05	sp|P22670|RFX1_HUMAN MHC class II regulatory factor RFX1 OS=Homo sapiens GN=RFX1 PE=1 SV=2//0
XM_007996092.1	-1.2843	1.16E-22	2.63E-21	sp|Q6SPF0|SAMD1_HUMAN Atherin OS=Homo sapiens GN=SAMD1 PE=1 SV=1//1.84966e-104
XM_007996093.1	-1.5799	7.98E-20	1.57E-18	sp|P17612|KAPCA_HUMAN cAMP-dependent protein kinase catalytic subunit alpha OS=Homo sapiens GN=PRKACA PE=1 SV=2//9.33238e-126
XM_007996094.1	-0.33047	0.0052962	0.026306	sp|Q9NVP2|ASF1B_HUMAN Histone chaperone ASF1B OS=Homo sapiens GN=ASF1B PE=1 SV=1//1.48226e-70
XM_007996095.1	-0.48443	2.02E-05	0.0001434	sp|O94910|LPHN1_HUMAN Latrophilin-1 OS=Homo sapiens GN=LPHN1 PE=1 SV=1//0
XM_007996101.1	-2.1017	1.92E-51	1.22E-49	sp|A6NIK2|LR10B_HUMAN Leucine-rich repeat-containing protein 10B OS=Homo sapiens GN=LRRC10B PE=4 SV=2//1.98445e-141
XM_007996102.1	1.7362	5.01E-27	1.38E-25	sp|Q9H6X5|CS044_HUMAN Uncharacterized protein C19orf44 OS=Homo sapiens GN=C19orf44 PE=1 SV=1//0
XM_007996104.1	-1.6973	1.99E-30	6.23E-29	sp|Q6PCB7|S27A1_HUMAN Long-chain fatty acid transport protein 1 OS=Homo sapiens GN=SLC27A1 PE=2 SV=1//0
XM_007996106.1	-1.0919	1.44E-28	4.21E-27	sp|O00459|P85B_HUMAN Phosphatidylinositol 3-kinase regulatory subunit beta OS=Homo sapiens GN=PIK3R2 PE=1 SV=2//2.36818e-10
XM_007996107.1	-1.1964	8.24E-05	0.0005472	sp|Q9NXJ5|PGPI_HUMAN Pyroglutamyl-peptidase 1 OS=Homo sapiens GN=PGPEP1 PE=1 SV=1//3.46153e-128
XM_007996108.1	-0.67193	2.35E-05	0.0001653	sp|P27544|CERS1_HUMAN Ceramide synthase 1 OS=Homo sapiens GN=CERS1 PE=1 SV=1//5.46711e-151
XM_007996118.1	-0.53046	1.28E-11	1.57E-10	sp|Q69BK2|UCRI_THEGE Cytochrome b-c1 complex subunit Rieske, mitochondrial OS=Theropithecus gelada GN=UQCRFS1 PE=3 SV=1//0
XM_007996189.1	-0.76913	8.87E-05	0.0005857	sp|Q63HK5|TSH3_HUMAN Teashirt homolog 3 OS=Homo sapiens GN=TSHZ3 PE=1 SV=2//0
XM_007996233.1	1.0427	5.55E-39	2.34E-37	sp|Q8IUC4|RHPN2_HUMAN Rhophilin-2 OS=Homo sapiens GN=RHPN2 PE=1 SV=1//0
XM_007996237.1	-1.3701	0.0070422	0.034044	sp|O75074|LRP3_HUMAN Low-density lipoprotein receptor-related protein 3 OS=Homo sapiens GN=LRP3 PE=2 SV=2//0
XM_007996240.1	0.3562	0.0022574	0.01211	sp|P49715|CEBPA_HUMAN CCAAT/enhancer-binding protein alpha OS=Homo sapiens GN=CEBPA PE=1 SV=3//7.03509e-108
XM_007996242.1	-0.86062	2.05E-22	4.62E-21	sp|P12955|PEPD_HUMAN Xaa-Pro dipeptidase OS=Homo sapiens GN=PEPD PE=1 SV=3//0
XM_007996256.1	-1.2174	4.19E-41	1.88E-39	sp|Q4R591|G6PI_MACFA Glucose-6-phosphate isomerase OS=Macaca fascicularis GN=GPI PE=2 SV=3//0
XM_007996257.1	1.564	3.87E-18	6.98E-17	sp|Q9BRP1|PDD2L_HUMAN Programmed cell death protein 2-like OS=Homo sapiens GN=PDCD2L PE=1 SV=1//0
XM_007996271.1	0.72786	2.17E-07	1.88E-06	sp|Q2M3W8|ZN181_HUMAN Zinc finger protein 181 OS=Homo sapiens GN=ZNF181 PE=2 SV=1//1.63449e-07

XM_007996276.1	1.0524	3.40E-22	7.58E-21	sp|A6NIX2|WTIP_HUMAN Wilms tumor protein 1-interacting protein OS=Homo sapiens GN=WTIP PE=1 SV=3//4.74755e-138
XM_007996283.1	-0.40197	0.0011568	0.0065507	sp|Q8NBI2|CYAC3_HUMAN Cytochrome b ascorbate-dependent protein 3 OS=Homo sapiens GN=CYB561A3 PE=1 SV=1//7.68393e-136
XM_007996296.1	-1.8394	9.31E-32	3.05E-30	sp|Q4PPC4|SCN1B_CANFA Sodium channel subunit beta-1 OS=Canis familiaris GN=SCN1B PE=2 SV=1//1.89674e-116
XM_007996379.1	-0.8647	0.0017009	0.0093536	sp|P20648|ATP4A_HUMAN Potassium-transporting ATPase alpha chain 1 OS=Homo sapiens GN=ATP4A PE=2 SV=5//0
XM_007996398.1	-1.1782	0.0017986	0.0098411	sp|P62876|RPAB5_MOUSE DNA-directed RNA polymerases I, II, and III subunit RPABC5 OS=Mus musculus GN=Polr2l PE=3 SV=1//4.39846e-41
XM_007996436.1	-1.7529	1.06E-38	4.42E-37	sp|P51693|APLP1_HUMAN Amyloid-like protein 1 OS=Homo sapiens GN=APLP1 PE=1 SV=3//0
XM_007996447.1	-2.0732	4.39E-11	5.19E-10	sp|Q9BTN0|LRFN3_HUMAN Leucine-rich repeat and fibronectin type-III domain-containing protein 3 OS=Homo sapiens GN=LRFN3 PE=2 SV=1//0
XM_007996449.1	1.6626	2.14E-21	4.57E-20	sp|A6NFY7|SDHF1_HUMAN Succinate dehydrogenase assembly factor 1, mitochondrial OS=Homo sapiens GN=SDHAF1 PE=1 SV=1//1.78817e-55
XM_007996470.1	-0.45079	1.31E-05	9.55E-05	sp|Q99426|TBCB_HUMAN Tubulin-folding cofactor B OS=Homo sapiens GN=TBCB PE=1 SV=2//8.41526e-155
XM_007996534.1	-1.4474	8.17E-55	5.61E-53	sp|Q24JP5|T132A_HUMAN Transmembrane protein 132A OS=Homo sapiens GN=TMEM132A PE=2 SV=1//0
XM_007996557.1	0.96616	1.56E-06	1.26E-05	sp|Q8TAQ5|ZN420_HUMAN Zinc finger protein 420 OS=Homo sapiens GN=ZNF420 PE=1 SV=1//6.0433e-11
XM_007996568.1	-0.29296	0.0029275	0.015385	sp|Q9BVC6|TM109_HUMAN Transmembrane protein 109 OS=Homo sapiens GN=TMEM109 PE=1 SV=1//9.19092e-95
XM_007996579.1	0.26834	0.0009916	0.0056677	sp|Q9UMS4|PRP19_HUMAN Pre-mRNA-processing factor 19 OS=Homo sapiens GN=PRPF19 PE=1 SV=1//0
XM_007996586.1	0.89108	4.02E-07	3.41E-06	sp|Q4R6C2|ZN383_MACFA Zinc finger protein 383 OS=Macaca fascicularis GN=ZNF383 PE=2 SV=1//0
XM_007996587.1	1.4854	2.66E-05	0.000186	sp|P10072|HKR1_HUMAN Krueppel-related zinc finger protein 1 OS=Homo sapiens GN=HKR1 PE=2 SV=4//1.36977e-68
XM_007996608.1	2.0778	1.50E-49	9.13E-48	sp|Q9H6F5|CCD86_HUMAN Coiled-coil domain-containing protein 86 OS=Homo sapiens GN=CCDC86 PE=1 SV=1//1.48649e-176
XM_007996659.1	-0.81473	0.0068709	0.033267	sp|Q96A00|PP14A_HUMAN Protein phosphatase 1 regulatory subunit 14A OS=Homo sapiens GN=PPP1R14A PE=1 SV=1//1.34478e-74
XM_007996682.1	-1.1638	0.0009984	0.0057051	sp|Q86UU5|GGN_HUMAN Gametogenetin OS=Homo sapiens GN=GGN PE=1 SV=2//1.37141e-59
XM_007996719.1	-1.2817	1.26E-41	5.73E-40	sp|Q5RFG0|ECH1_PONAB Delta(3,5)-Delta(2,4)-dienoyl-CoA isomerase, mitochondrial OS=Pongo abelii GN=ECH1 PE=2 SV=1//0
XM_007996724.1	1.2688	1.65E-16	2.72E-15	sp|Q15653|IKBB_HUMAN NF-kappa-B inhibitor beta OS=Homo sapiens GN=NFKBIB PE=1 SV=2//1.15831e-173
XM_007996732.1	-4.0012	0.0044538	0.022504	sp|Q8N5U1|M4A15_HUMAN Membrane-spanning 4-domains subfamily A member 15 OS=Homo sapiens GN=MS4A15 PE=2 SV=2//1.33361e-141
XM_007996755.1	-1.9808	0.0001247	0.0008072	sp|Q6ZVX7|FBX50_HUMAN F-box only protein 50 OS=Homo sapiens GN=NCCRP1 PE=1 SV=1//3.74368e-125
XM_007996758.1	Inf	0.0038183	0.01955	sp|Q8IZI9|IFNL3_HUMAN Interferon lambda-3 OS=Homo sapiens GN=IFNL3 PE=1 SV=2//5.01019e-102
XM_007996760.1	5.8374	3.62E-27	1.00E-25	sp|Q8IU54|IFNL1_HUMAN Interferon lambda-1 OS=Homo sapiens GN=IFNL1 PE=1 SV=1//9.13983e-98
XM_007996761.1	-0.46388	0.0020357	0.011007	sp|Q9P244|LRFN1_HUMAN Leucine-rich repeat and fibronectin type III domain-containing protein 1 OS=Homo sapiens GN=LRFN1 PE=1 SV=2//0
XM_007996764.1	2.0766	1.04E-16	1.75E-15	sp|P26651|TTP_HUMAN Tristetraprolin OS=Homo sapiens GN=ZFP36 PE=1 SV=1//7.34305e-126
XM_007996782.1	-0.4038	6.72E-10	7.21E-09	sp|P62250|RS16_RAT 40S ribosomal protein S16 OS=Rattus norvegicus GN=Rps16 PE=1 SV=2//3.3156e-101
XM_007996788.1	-1.6196	2.82E-10	3.14E-09	sp|P59797|SELV_HUMAN Selenoprotein V OS=Homo sapiens GN=SELV PE=1 SV=2//2.22717e-116
XM_007996794.1	2.7708	1.44E-15	2.24E-14	sp|Q9UHV8|PP13_HUMAN Galactoside-binding soluble lectin 13 OS=Homo sapiens GN=LGALS13 PE=1 SV=1//1.37442e-82
XM_007996803.1	-0.31787	6.01E-05	0.0004045	sp|P35550|FBRL_MOUSE rRNA 2'-O-methyltransferase fibrillarin OS=Mus musculus GN=Fbl PE=2 SV=2//2.67982e-177
XM_007996839.1	1.077	2.27E-11	2.75E-10	sp|Q9UHV2|SRTD1_HUMAN SERTA domain-containing protein 1 OS=Homo sapiens GN=SERTAD1 PE=1 SV=2//8.65678e-75
XM_007996844.1	-0.94073	3.89E-19	7.44E-18	sp|Q8TBC3|SHKB1_HUMAN SH3KBP1-binding protein 1 OS=Homo sapiens GN=SHKBP1 PE=1 SV=2//0
XM_007996853.1	2.0614	6.02E-06	4.56E-05	sp|Q96D53|ADCK4_HUMAN AarF domain-containing protein kinase 4 OS=Homo sapiens GN=ADCK4 PE=1 SV=2//0
XM_007996868.1	0.3752	0.0008556	0.0049356	sp|Q5BKX5|CS054_HUMAN UPF0692 protein C19orf54 OS=Homo sapiens GN=C19orf54 PE=1 SV=2//0
XM_007996874.1	-1.0599	2.12E-10	2.38E-09	sp|P61018|RAB4B_HUMAN Ras-related protein Rab-4B OS=Homo sapiens GN=RAB4B PE=1 SV=1//2.36468e-154
XM_007996894.1	-1.6905	7.45E-91	1.17E-88	sp|P01137|TGFB1_HUMAN Transforming growth factor beta-1 OS=Homo sapiens

				GN=TGFB1 PE=1 SV=2//0
XM_007996903.1	-1.0009	5.40E-17	9.17E-16	sp|Q8HXY4|ODBA_MACFA 2-oxoisovalerate dehydrogenase subunit alpha, mitochondrial OS=Macaca fascicularis GN=BCKDHA PE=2 SV=1//0
XM_007996915.1	-2.8765	0.0010578	0.0060212	sp|Q7Z7M8|B3GN8_HUMAN UDP-GlcNAc:betaGal beta-1,3-N-acetylglucosaminyltransferase 8 OS=Homo sapiens GN=B3GNT8 PE=1 SV=1//0
XM_007996943.1	-0.47093	1.75E-12	2.28E-11	sp|Q5R8M9|RS19_PONAB 40S ribosomal protein S19 OS=Pongo abelii GN=RPS19 PE=2 SV=3//4.01126e-102
XM_007996946.1	-1.2848	2.72E-17	4.69E-16	sp|Q9UI14|PRAF1_HUMAN Prenylated Rab acceptor protein 1 OS=Homo sapiens GN=RABAC1 PE=1 SV=1//2.66267e-129
XM_007996971.1	0.64909	1.42E-06	1.15E-05	sp|Q8WXF8|DEDD2_HUMAN DNA-binding death effector domain-containing protein 2 OS=Homo sapiens GN=DEDD2 PE=1 SV=1//3.77033e-133
XM_007996973.1	-0.40432	1.41E-05	0.0001026	sp|P49840|GSK3A_HUMAN Glycogen synthase kinase-3 alpha OS=Homo sapiens GN=GSK3A PE=1 SV=2//0
XM_007996995.1	-0.72567	1.87E-06	1.49E-05	sp|Q7Z7M0|MEGF8_HUMAN Multiple epidermal growth factor-like domains protein 8 OS=Homo sapiens GN=MEGF8 PE=1 SV=2//0
XM_007997022.1	2.3663	0.002801	0.014791	sp|Q6NSJ2|PHLB3_HUMAN Pleckstrin homology-like domain family B member 3 OS=Homo sapiens GN=PHLDB3 PE=2 SV=3//0
XM_007997028.1	-1.3566	0.0044593	0.022511	sp|Q86XF7|ZN575_HUMAN Zinc finger protein 575 OS=Homo sapiens GN=ZNF575 PE=2 SV=1//9.30199e-113
XM_007997043.1	-1.3427	9.00E-25	2.25E-23	sp|Q8NFZ8|CADM4_HUMAN Cell adhesion molecule 4 OS=Homo sapiens GN=CADM4 PE=1 SV=1//0
XM_007997049.1	1.1609	6.23E-10	6.71E-09	sp|Q9GK79|UPAR_CHLAE Urokinase plasminogen activator surface receptor OS=Chlorocebus aethiops GN=PLAUR PE=2 SV=1//9.98018e-164
XM_007997052.1	2.2491	1.30E-09	1.36E-08	sp|Q9H0W8|SMG9_HUMAN Protein SMG9 OS=Homo sapiens GN=SMG9 PE=1 SV=1//0
XM_007997079.1	0.51007	0.0056501	0.027868	sp|Q9UK10|ZN225_HUMAN Zinc finger protein 225 OS=Homo sapiens GN=ZNF225 PE=2 SV=2//9.71766e-118
XM_007997089.1	0.73101	5.24E-05	0.0003549	sp|Q9UK12|ZN222_HUMAN Zinc finger protein 222 OS=Homo sapiens GN=ZNF222 PE=2 SV=2//0
XM_007997111.1	2.4907	2.63E-33	9.11E-32	sp|P32506|PVR_CHLAE Poliovirus receptor homolog OS=Chlorocebus aethiops GN=PVR PE=2 SV=1//0
XM_007997137.1	2.7651	1.38E-23	3.28E-22	sp|Q7Z692|CEA19_HUMAN Carcinoembryonic antigen-related cell adhesion molecule 19 OS=Homo sapiens GN=CEACAM19 PE=1 SV=2//7.78056e-179
XM_007997149.1	-3.646	4.90E-07	4.12E-06	sp|P50895|BCAM_HUMAN Basal cell adhesion molecule OS=Homo sapiens GN=BCAM PE=1 SV=2//0
XM_007997151.1	-0.48217	0.0070715	0.034163	sp|Q92692|PVRL2_HUMAN Nectin-2 OS=Homo sapiens GN=PVRL2 PE=1 SV=1//0
XM_007997153.1	-0.74035	2.27E-09	2.34E-08	sp|Q5R7L3|RM16_PONAB 39S ribosomal protein L16, mitochondrial OS=Pongo abelii GN=MRPL16 PE=2 SV=1//6.75426e-156
XM_007997154.1	0.36421	8.46E-05	0.0005603	sp|O96008|TOM40_HUMAN Mitochondrial import receptor subunit TOM40 homolog OS=Homo sapiens GN=TOMM40 PE=1 SV=1//0
XM_007997175.1	1.1994	2.02E-10	2.27E-09	sp|Q5JPI9|MET10_HUMAN Protein-lysine N-methyltransferase METTL10 OS=Homo sapiens GN=METTL10 PE=1 SV=2//6.24669e-07
XM_007997177.1	1.3351	2.18E-13	3.01E-12	sp|A5A769|BL1S3_PIG Biogenesis of lysosome-related organelles complex 1 subunit 3 OS=Sus scrofa GN=BLOC1S3 PE=2 SV=1//1.09953e-53
XM_007997204.1	Inf	0.000302	0.0018514	sp|P53539|FOSB_HUMAN Protein fosB OS=Homo sapiens GN=FOSB PE=2 SV=1//5.88069e-135
XM_007997242.1	-1.485	2.71E-44	1.35E-42	sp|P62317|SMD2_MOUSE Small nuclear ribonucleoprotein Sm D2 OS=Mus musculus GN=Snrpd2 PE=3 SV=1//1.78456e-57
XM_007997250.1	0.86326	8.56E-23	1.97E-21	sp|Q5R8Q4|PATL1_PONAB Protein PAT1 homolog 1 OS=Pongo abelii GN=PATL1 PE=2 SV=1//0
XM_007997254.1	-1.394	3.48E-25	8.97E-24	sp|Q8N196|SIX5_HUMAN Homeobox protein SIX5 OS=Homo sapiens GN=SIX5 PE=1 SV=3//0
XM_007997262.1	0.62708	4.03E-15	6.10E-14	sp|P22059|OSBP1_HUMAN Oxysterol-binding protein 1 OS=Homo sapiens GN=OSBP PE=1 SV=1//0
XM_007997264.1	0.4952	0.0002133	0.0013364	sp|Q8IU81|I2BP1_HUMAN Interferon regulatory factor 2-binding protein 1 OS=Homo sapiens GN=IRF2BP1 PE=1 SV=1//0
XM_007997270.1	-2.8195	3.99E-19	7.61E-18	sp|Q9UNW9|NOVA2_HUMAN RNA-binding protein Nova-2 OS=Homo sapiens GN=NOVA2 PE=1 SV=1//2.63201e-06
XM_007997279.1	5.7277	1.32E-25	3.47E-24	sp|Q6UW32|IGFL1_HUMAN Insulin growth factor-like family member 1 OS=Homo sapiens GN=IGFL1 PE=1 SV=1//3.56059e-62
XM_007997294.1	-0.54736	4.29E-05	0.0002936	sp|Q9H0W5|CCDC8_HUMAN Coiled-coil domain-containing protein 8 OS=Homo sapiens GN=CCDC8 PE=1 SV=2//0
XM_007997299.1	-2.236	1.65E-126	4.25E-124	sp|P62155|CALM_XENLA Calmodulin OS=Xenopus laevis GN=calm1 PE=1 SV=2//3.80319e-95
XM_007997302.1	-1.1317	0.0010089	0.0057603	sp|Q96B18|DACT3_HUMAN Dapper homolog 3 OS=Homo sapiens GN=DACT3 PE=1 SV=2//1.79169e-180
XM_007997317.1	-0.92909	0.0050244	0.025075	sp|Q86SG4|DPCA2_HUMAN Putative Dresden prostate carcinoma protein 2 OS=Homo sapiens GN=HMGN2P46 PE=5 SV=1//2.73578e-26
XM_007997318.1	-0.60425	0.0001576	0.0010056	sp|P62744|AP2S1_RAT AP-2 complex subunit sigma OS=Rattus norvegicus GN=Ap2s1 PE=1 SV=1//1.33385e-28
XM_007997319.1	-0.30636	0.0010545	0.0060051	sp|Q9NRY4|RHG35_HUMAN Rho GTPase-activating protein 35 OS=Homo sapiens

				GN=ARHGAP35 PE=1 SV=3//0
XM_007997327.1	-1.6802	3.79E-12	4.84E-11	sp|Q9NX00|TM160_HUMAN Transmembrane protein 160 OS=Homo sapiens GN=TMEM160 PE=1 SV=1//4.66279e-93
XM_007997338.1	-1.0426	7.35E-46	3.96E-44	sp|P62856|RS26_RAT 40S ribosomal protein S26 OS=Rattus norvegicus GN=Rps26 PE=3 SV=3//4.45255e-50
XM_007997347.1	-0.59146	0.0017648	0.0096735	sp|C9JVW0|INAM1_HUMAN Putative transmembrane protein INAFM1 OS=Homo sapiens GN=INAFM1 PE=4 SV=1//2.30888e-15
XM_007997363.1	0.37965	8.54E-05	0.0005656	sp|P54920|SNAA_HUMAN Alpha-soluble NSF attachment protein OS=Homo sapiens GN=NAPA PE=1 SV=3//0
XM_007997369.1	-1.3013	3.36E-38	1.37E-36	sp|Q9NZN4|EHD2_HUMAN EH domain-containing protein 2 OS=Homo sapiens GN=EHD2 PE=1 SV=2//0
XM_007997371.1	-0.3189	4.25E-05	0.0002906	sp|Q9NZM5|GSCR2_HUMAN Glioma tumor suppressor candidate region gene 2 protein OS=Homo sapiens GN=GLTSCR2 PE=1 SV=2//0
XM_007997413.1	-0.78768	2.12E-06	1.68E-05	sp|P54852|EMP3_HUMAN Epithelial membrane protein 3 OS=Homo sapiens GN=EMP3 PE=1 SV=1//1.19238e-82
XM_007997422.1	-1.0011	9.78E-34	3.45E-32	sp|P24390|ERD21_HUMAN ER lumen protein-retaining receptor 1 OS=Homo sapiens GN=KDELR1 PE=1 SV=1//5.05255e-129
XM_007997428.1	1.5527	1.02E-34	3.74E-33	sp|Q9BQ67|GRWD1_HUMAN Glutamate-rich WD repeat-containing protein 1 OS=Homo sapiens GN=GRWD1 PE=1 SV=1//0
XM_007997432.1	-1.5563	1.07E-15	1.69E-14	sp|Q96Q04|LMTK3_HUMAN Serine/threonine-protein kinase LMTK3 OS=Homo sapiens GN=LMTK3 PE=1 SV=2//1.56284e-40
XM_007997434.1	0.28897	0.007257	0.034964	sp|Q10586|DBP_HUMAN D site-binding protein OS=Homo sapiens GN=DBP PE=1 SV=1//6.13241e-116
XM_007997451.1	-1.6469	2.78E-20	5.61E-19	sp|Q866X7|CAH11_BOVIN Carbonic anhydrase-related protein 11 OS=Bos taurus GN=CA11 PE=2 SV=1//0
XM_007997465.1	5.445	9.54E-81	1.24E-78	sp|Q9NSA1|FGF21_HUMAN Fibroblast growth factor 21 OS=Homo sapiens GN=FGF21 PE=1 SV=1//7.38743e-120
XM_007997466.1	1.1203	1.34E-09	1.41E-08	sp|Q9BPX1|DHB14_HUMAN 17-beta-hydroxysteroid dehydrogenase 14 OS=Homo sapiens GN=HSD17B14 PE=1 SV=1//1.1783e-132
XM_007997475.1	-1.3637	5.20E-09	5.19E-08	sp|Q7JK39|DHDH_MACFU Trans-1,2-dihydrobenzene-1,2-diol dehydrogenase OS=Macaca fuscata fuscata GN=DHDH PE=1 SV=1//0
XM_007997480.1	-1.2019	9.51E-54	6.37E-52	sp|Q5R538|FRIL_PONAB Ferritin light chain OS=Pongo abelii GN=FTL PE=2 SV=3//9.32697e-114
XM_007997500.1	-2.4373	0.010096	0.047246	sp|P07434|CGHB_PAPAN Choriogonadotropin subunit beta OS=Papio anubis GN=CGB PE=2 SV=1//4.69617e-76
XM_007997546.1	-0.41468	0.0011173	0.006338	sp|Q9P2U7|VGLU1_HUMAN Vesicular glutamate transporter 1 OS=Homo sapiens GN=SLC17A7 PE=2 SV=1//0
XM_007997559.1	-0.49341	0.0005946	0.0035079	sp|Q8IZ83|A16A1_HUMAN Aldehyde dehydrogenase family 16 member A1 OS=Homo sapiens GN=ALDH16A1 PE=1 SV=2//0
XM_007997567.1	-0.57985	0.0021953	0.011804	sp|P62282|RS11_RAT 40S ribosomal protein S11 OS=Rattus norvegicus GN=Rps11 PE=1 SV=3//1.59424e-112
XM_007997568.1	-0.91595	7.89E-06	5.89E-05	sp|Q96D15|RCN3_HUMAN Reticulocalbin-3 OS=Homo sapiens GN=RCN3 PE=1 SV=1//1.26264e-153
XM_007997573.1	-0.68653	4.82E-07	4.06E-06	sp|Q9Y314|NOSIP_HUMAN Nitric oxide synthase-interacting protein OS=Homo sapiens GN=NOSIP PE=1 SV=1//0
XM_007997574.1	-1.5403	5.75E-40	2.50E-38	sp|Q9ULL5|PRR12_HUMAN Proline-rich protein 12 OS=Homo sapiens GN=PRR12 PE=1 SV=2//0
XM_007997575.1	-1.1104	1.58E-14	2.32E-13	sp|P10301|RRAS_HUMAN Ras-related protein R-Ras OS=Homo sapiens GN=RRAS PE=1 SV=1//5.47268e-129
XM_007997603.1	-0.52396	2.64E-10	2.95E-09	sp|Q71SY5|MED25_HUMAN Mediator of RNA polymerase II transcription subunit 25 OS=Homo sapiens GN=MED25 PE=1 SV=2//0
XM_007997604.1	-0.33507	0.0053996	0.026771	sp|Q96T60|PNKP_HUMAN Bifunctional polynucleotide phosphatase/kinase OS=Homo sapiens GN=PNKP PE=1 SV=1//0
XM_007997605.1	-1.4568	4.29E-67	4.14E-65	sp|Q86YD1|PTOV1_HUMAN Prostate tumor-overexpressed gene 1 protein OS=Homo sapiens GN=PTOV1 PE=1 SV=1//4.31739e-56
XM_007997621.1	1.3333	4.29E-31	1.37E-29	sp|P37198|NUP62_HUMAN Nuclear pore glycoprotein p62 OS=Homo sapiens GN=NUP62 PE=1 SV=3//8.83576e-165
XM_007997640.1	-1.5535	4.62E-07	3.89E-06	sp|Q63959|KCNC3_MOUSE Potassium voltage-gated channel subfamily C member 3 OS=Mus musculus GN=Kcnc3 PE=1 SV=2//0
XM_007997642.1	-1.6208	7.30E-21	1.52E-19	sp|O96009|NAPSA_HUMAN Napsin-A OS=Homo sapiens GN=NAPSA PE=1 SV=1//0
XM_007997668.1	Inf	0.0002883	0.0017719	sp|Q969I3|GLYL1_HUMAN Glycine N-acyltransferase-like protein 1 OS=Homo sapiens GN=GLYATL1 PE=1 SV=1//8.10086e-19
XM_007997674.1	-2.0292	0.0002188	0.001367	sp|Q9NT99|LRC4B_HUMAN Leucine-rich repeat-containing protein 4B OS=Homo sapiens GN=LRRC4B PE=2 SV=3//0
XM_007997687.1	-1.4026	0.0001231	0.0007979	sp|Q9Y240|CLC11_HUMAN C-type lectin domain family 11 member A OS=Homo sapiens GN=CLEC11A PE=1 SV=1//2.69415e-167
XM_007997690.1	1.6274	1.77E-22	4.00E-21	sp|P26441|CNTF_HUMAN Ciliary neurotrophic factor OS=Homo sapiens GN=CNTF PE=1 SV=1//1.57013e-123
XM_007997710.1	3.3888	8.18E-12	1.02E-10	sp|Q9Y5K2|KLK4_HUMAN Kallikrein-4 OS=Homo sapiens GN=KLK4 PE=1 SV=2//7.87634e-152

XM_007997770.1	-1.0915	2.55E-26	6.84E-25	sp|Q5RFK0|ETFB_PONAB Electron transfer flavoprotein subunit beta OS=Pongo abelii GN=ETFB PE=2 SV=3//9.77293e-156
XM_007997780.1	2.0778	0.0093976	0.044288	sp|Q96LC7|SIG10_HUMAN Sialic acid-binding Ig-like lectin 10 OS=Homo sapiens GN=SIGLEC10 PE=1 SV=3//0
XM_007997808.1	-2.1326	1.65E-08	1.59E-07	-//-
XM_007997837.1	0.72518	0.0001511	0.000967	sp|Q9GZX5|ZN350_HUMAN Zinc finger protein 350 OS=Homo sapiens GN=ZNF350 PE=1 SV=3//0
XM_007997857.1	-0.76838	2.43E-27	6.80E-26	sp|P30153|2AAA_HUMAN Serine/threonine-protein phosphatase 2A 65 kDa regulatory subunit A alpha isoform OS=Homo sapiens GN=PPP2R1A PE=1 SV=4//0
XM_007997867.1	0.76469	0.0001768	0.0011208	sp|Q8WV37|ZN480_HUMAN Zinc finger protein 480 OS=Homo sapiens GN=ZNF480 PE=1 SV=2//3.98075e-13
XM_007997911.1	1.3035	3.16E-10	3.50E-09	sp|Q96IR2|ZN845_HUMAN Zinc finger protein 845 OS=Homo sapiens GN=ZNF845 PE=2 SV=3//5.22521e-13
XM_007997912.1	1.3338	4.15E-07	3.52E-06	sp|Q96IR2|ZN845_HUMAN Zinc finger protein 845 OS=Homo sapiens GN=ZNF845 PE=2 SV=3//1.26859e-10
XM_007997914.1	-1.4317	0.0016628	0.00916	sp|Q0VGE8|ZN816_HUMAN Zinc finger protein 816 OS=Homo sapiens GN=ZNF816 PE=2 SV=2//5.18372e-31
XM_007997969.1	-2.3991	1.89E-23	4.48E-22	sp|P62957|CCG7_RAT Voltage-dependent calcium channel gamma-7 subunit OS=Rattus norvegicus GN=Cacng7 PE=1 SV=1//2.55949e-52
XM_007997970.1	-2.082	0.0004047	0.0024493	sp|Q8VHW5|CCG8_RAT Voltage-dependent calcium channel gamma-8 subunit OS=Rattus norvegicus GN=Cacng8 PE=1 SV=1//1.50206e-177
XM_007997974.1	-2.3034	0.0012873	0.0072443	sp|Q8VBT3|OSCAR_MOUSE Osteoclast-associated immunoglobulin-like receptor OS=Mus musculus GN=Oscar PE=2 SV=1//6.72565e-130
XM_007997975.1	-1.8779	1.49E-14	2.20E-13	sp|Q0MQ96|NDUA3_PANTR NADH dehydrogenase [ubiquinone] 1 alpha subcomplex subunit 3 OS=Pan troglodytes GN=NDUFA3 PE=3 SV=1//2.44047e-49
XM_007997976.1	-0.75405	1.68E-15	2.60E-14	sp|Q8WWY3|PRP31_HUMAN U4/U6 small nuclear ribonucleoprotein Prp31 OS=Homo sapiens GN=PRPF31 PE=1 SV=2//0
XM_007997979.1	-1.7117	0.000102	0.0006683	sp|Q7Z404|TMC4_HUMAN Transmembrane channel-like protein 4 OS=Homo sapiens GN=TMC4 PE=2 SV=3//1.74566e-160
XM_007998028.1	-0.41736	5.41E-09	5.39E-08	sp|P29314|RS9_RAT 40S ribosomal protein S9 OS=Rattus norvegicus GN=Rps9 PE=1 SV=4//3.34857e-126
XM_007998029.1	-1.0602	2.92E-33	1.01E-31	sp|Q96N66|MBOA7_HUMAN Lysophospholipid acyltransferase 7 OS=Homo sapiens GN=MBOAT7 PE=1 SV=2//0
XM_007998110.1	1.4287	1.38E-30	4.34E-29	sp|Q8NBN7|RDH13_HUMAN Retinol dehydrogenase 13 OS=Homo sapiens GN=RDH13 PE=1 SV=2//0
XM_007998114.1	0.85526	3.04E-17	5.22E-16	sp|Q9BZL4|PP12C_HUMAN Protein phosphatase 1 regulatory subunit 12C OS=Homo sapiens GN=PPP1R12C PE=1 SV=1//0
XM_007998120.1	-1.5932	6.14E-06	4.66E-05	sp|Q8MKD5|TNNI3_CANFA Troponin I, cardiac muscle OS=Canis familiaris GN=TNNI3 PE=2 SV=3//2.59318e-106
XM_007998130.1	3.2451	1.90E-08	1.82E-07	sp|Q9HD43|PTPRH_HUMAN Receptor-type tyrosine-protein phosphatase H OS=Homo sapiens GN=PTPRH PE=1 SV=3//0
XM_007998135.1	0.83451	1.67E-07	1.46E-06	sp|Q8TDC3|BRSK1_HUMAN Serine/threonine-protein kinase BRSK1 OS=Homo sapiens GN=BRSK1 PE=1 SV=2//1.79785e-27
XM_007998147.1	-2.6314	0.0003578	0.0021776	sp|Q86Y97|SV422_HUMAN Histone-lysine N-methyltransferase SUV420H2 OS=Homo sapiens GN=SUV420H2 PE=1 SV=1//8.70191e-132
XM_007998151.1	1.8425	2.52E-13	3.46E-12	sp|Q8WZ59|TM190_HUMAN Transmembrane protein 190 OS=Homo sapiens GN=TMEM190 PE=1 SV=1//8.99741e-71
XM_007998155.1	-1.7171	2.04E-06	1.62E-05	sp|Q6YFQ2|CX6B2_HUMAN Cytochrome c oxidase subunit 6B2 OS=Homo sapiens GN=COX6B2 PE=1 SV=1//7.17835e-55
XM_007998157.1	-0.19416	0.0030692	0.016064	sp|P41105|RL28_MOUSE 60S ribosomal protein L28 OS=Mus musculus GN=Rpl28 PE=1 SV=2//6.62814e-81
XM_007998158.1	0.29033	0.0001748	0.0011098	sp|Q16763|UBE2S_HUMAN Ubiquitin-conjugating enzyme E2 S OS=Homo sapiens GN=UBE2S PE=1 SV=2//1.86647e-130
XM_007998159.1	0.59451	0.0011214	0.00636	sp|Q8IZQ5|SELH_HUMAN Selenoprotein H OS=Homo sapiens GN=SELH PE=1 SV=2//2.68483e-62
XM_007998167.1	0.69592	2.11E-05	0.0001495	sp|Q5EBL2|ZN628_HUMAN Zinc finger protein 628 OS=Homo sapiens GN=ZNF628 PE=2 SV=3//3.53174e-119
XM_007998168.1	-0.70022	2.82E-05	0.0001967	sp|Q8WUY8|NAT14_HUMAN N-acetyltransferase 14 OS=Homo sapiens GN=NAT14 PE=1 SV=1//1.06157e-52
XM_007998169.1	0.46025	7.96E-07	6.59E-06	sp|Q4R5B4|TMX2_MACFA Thioredoxin-related transmembrane protein 2 OS=Macaca fascicularis GN=TMX2 PE=2 SV=1//0
XM_007998182.1	0.84469	2.43E-08	2.31E-07	sp|A0JLT2|MED19_HUMAN Mediator of RNA polymerase II transcription subunit 19 OS=Homo sapiens GN=MED19 PE=1 SV=2//1.71877e-98
XM_007998184.1	-0.6864	0.0020284	0.010975	sp|Q8NCA9|ZN784_HUMAN Zinc finger protein 784 OS=Homo sapiens GN=ZNF784 PE=2 SV=1//7.5117e-166
XM_007998189.1	-1.2172	9.28E-21	1.91E-19	sp|Q9UK33|ZN580_HUMAN Zinc finger protein 580 OS=Homo sapiens GN=ZNF580 PE=1 SV=1//2.42809e-76
XM_007998207.1	0.50047	1.24E-09	1.30E-08	sp|Q2THX1|ZDHC5_PANTR Palmitoyltransferase ZDHHC5 OS=Pan troglodytes GN=ZDHHC5 PE=2 SV=1//0

XM_007998235.1	1.248	9.17E-16	1.45E-14	sp|Q4R7R3|CLP1_MACFA Polyribonucleotide 5'-hydroxyl-kinase Clp1 OS=Macaca fascicularis GN=CLP1 PE=2 SV=1//0
XM_007998245.1	0.82181	4.76E-05	0.0003235	sp|Q96ND8|ZN583_HUMAN Zinc finger protein 583 OS=Homo sapiens GN=ZNF583 PE=2 SV=2//0
XM_007998250.1	0.98484	9.85E-05	0.0006477	sp|Q96NS1|YPEL4_HUMAN Protein yippee-like 4 OS=Homo sapiens GN=YPEL4 PE=2 SV=1//1.17655e-84
XM_007998279.1	0.87468	3.59E-05	0.0002479	sp|O43296|ZN264_HUMAN Zinc finger protein 264 OS=Homo sapiens GN=ZNF264 PE=1 SV=1//0
XM_007998282.1	1.8374	6.06E-27	1.66E-25	sp|Q5CZA5|ZN805_HUMAN Zinc finger protein 805 OS=Homo sapiens GN=ZNF805 PE=2 SV=3//0
XM_007998292.1	1.3882	0.0056095	0.027699	sp|Q9HCX3|ZN304_HUMAN Zinc finger protein 304 OS=Homo sapiens GN=ZNF304 PE=2 SV=2//3.09969e-155
XM_007998323.1	0.98495	6.05E-11	7.06E-10	sp|Q5RBQ3|ZN549_PONAB Zinc finger protein 549 OS=Pongo abelii GN=ZNF549 PE=2 SV=1//0
XM_007998331.1	2.0361	5.50E-67	5.29E-65	sp|P52741|ZN134_HUMAN Zinc finger protein 134 OS=Homo sapiens GN=ZNF134 PE=2 SV=2//1.6224e-66
XM_007998332.1	1.6799	7.38E-11	8.53E-10	sp|Q6P9A1|ZN530_HUMAN Zinc finger protein 530 OS=Homo sapiens GN=ZNF530 PE=2 SV=2//0
XM_007998341.1	0.56508	5.45E-10	5.90E-09	sp|Q9H0E2|TOLIP_HUMAN Toll-interacting protein OS=Homo sapiens GN=TOLLIP PE=1 SV=1//1.19586e-153
XM_007998343.1	1.829	1.36E-23	3.24E-22	sp|Q9BWM5|ZN416_HUMAN Zinc finger protein 416 OS=Homo sapiens GN=ZNF416 PE=2 SV=1//1.72771e-32
XM_007998369.1	1.2289	2.87E-16	4.68E-15	sp|Q96SQ5|ZN587_HUMAN Zinc finger protein 587 OS=Homo sapiens GN=ZNF587 PE=1 SV=1//7.84817e-07
XM_007998373.1	-0.82302	8.34E-05	0.0005532	sp|Q86UN3|R4RL2_HUMAN Reticulon-4 receptor-like 2 OS=Homo sapiens GN=RTN4RL2 PE=1 SV=1//0
XM_007998422.1	0.72195	0.0005601	0.0033221	sp|P17098|ZNF8_HUMAN Zinc finger protein 8 OS=Homo sapiens GN=ZNF8 PE=2 SV=2//0
XM_007998439.1	1.2075	2.13E-07	1.85E-06	sp|Q9NWS9|ZN446_HUMAN Zinc finger protein 446 OS=Homo sapiens GN=ZNF446 PE=1 SV=1//5.22749e-173
XM_007998440.1	1.665	1.25E-21	2.69E-20	sp|O75467|Z324A_HUMAN Zinc finger protein 324A OS=Homo sapiens GN=ZNF324 PE=2 SV=1//0
XM_007998444.1	-0.52667	2.09E-15	3.20E-14	sp|P46782|RS5_HUMAN 40S ribosomal protein S5 OS=Homo sapiens GN=RPS5 PE=1 SV=4//9.19503e-150
XM_007998475.1	-2.1369	0.0002022	0.0012709	-//-
XM_007998476.1	0.63124	2.17E-07	1.88E-06	sp|P24864|CCNE1_HUMAN G1/S-specific cyclin-E1 OS=Homo sapiens GN=CCNE1 PE=1 SV=2//0
XM_007998478.1	0.31319	8.99E-05	0.0005936	sp|Q9C0C2|TB182_HUMAN 182 kDa tankyrase-1-binding protein OS=Homo sapiens GN=TNKS1BP1 PE=1 SV=4//0
XM_007998479.1	2.0444	4.92E-122	1.25E-119	sp|P53567|CEBPG_HUMAN CCAAT/enhancer-binding protein gamma OS=Homo sapiens GN=CEBPG PE=1 SV=1//8.80324e-75
XM_007998480.1	0.37	9.61E-07	7.89E-06	sp|Q8ND56|LS14A_HUMAN Protein LSM14 homolog A OS=Homo sapiens GN=LSM14A PE=1 SV=3//0
XM_007998481.1	-0.3045	0.0020159	0.010911	sp|Q15853|USF2_HUMAN Upstream stimulatory factor 2 OS=Homo sapiens GN=USF2 PE=1 SV=1//0
XM_007998482.1	-2.1919	1.82E-11	2.22E-10	sp|Q9UF12|PROD2_HUMAN Probable proline dehydrogenase 2 OS=Homo sapiens GN=PRODH2 PE=2 SV=1//0
XM_007998484.1	1.0233	8.45E-21	1.75E-19	sp|O43379|WDR62_HUMAN WD repeat-containing protein 62 OS=Homo sapiens GN=WDR62 PE=1 SV=4//0
XM_007998487.1	0.79966	2.43E-23	5.74E-22	sp|O60292|SI1L3_HUMAN Signal-induced proliferation-associated 1-like protein 3 OS=Homo sapiens GN=SIPA1L3 PE=1 SV=3//0
XM_007998488.1	-2.169	0.0004646	0.0027854	sp|P47929|LEG7_HUMAN Galectin-7 OS=Homo sapiens GN=LGALS7 PE=1 SV=2//1.58119e-67
XM_007998491.1	0.39553	2.60E-06	2.04E-05	sp|Q3ZCQ8|TIM50_HUMAN Mitochondrial import inner membrane translocase subunit TIM50 OS=Homo sapiens GN=TIMM50 PE=1 SV=2//0
XM_007998493.1	0.33682	2.77E-06	2.17E-05	sp|Q08945|SSRP1_HUMAN FACT complex subunit SSRP1 OS=Homo sapiens GN=SSRP1 PE=1 SV=1//0
XM_007998495.1	-0.98476	1.38E-09	1.45E-08	sp|Q9Y6R0|NUMBL_HUMAN Numb-like protein OS=Homo sapiens GN=NUMBL PE=1 SV=1//2.66e-106
XM_007998498.1	0.53576	0.0009186	0.0052723	sp|Q96SQ9|CP2S1_HUMAN Cytochrome P450 2S1 OS=Homo sapiens GN=CYP2S1 PE=1 SV=2//1.94697e-57
XM_007998500.1	2.8257	5.53E-83	7.53E-81	sp|P32506|PVR_CHLAE Poliovirus receptor homolog OS=Chlorocebus aethiops GN=PVR PE=2 SV=1//0
XM_007998503.1	-0.6158	2.68E-08	2.53E-07	sp|Q96L34|MARK4_HUMAN MAP/microtubule affinity-regulating kinase 4 OS=Homo sapiens GN=MARK4 PE=1 SV=1//0
XM_007998507.1	1.0802	1.58E-09	1.65E-08	sp|Q4R8W8|AAAT_MACFA Neutral amino acid transporter B(0) OS=Macaca fascicularis GN=SLC1A5 PE=2 SV=1//0
XM_007998508.1	-1.3037	0.003839	0.019647	sp|P62744|AP2S1_RAT AP-2 complex subunit sigma OS=Rattus norvegicus GN=Ap2s1 PE=1 SV=1//3.5014e-30
XM_007998511.1	0.73152	1.25E-08	1.21E-07	sp|Q14147|DHX34_HUMAN Probable ATP-dependent RNA helicase DHX34 OS=Homo sapiens GN=DHX34 PE=1 SV=2//0

XM_007998517.1	4.7674	0	0	sp|Q9H4M7|PKHA4_HUMAN Pleckstrin homology domain-containing family A member 4 OS=Homo sapiens GN=PLEKHA4 PE=1 SV=2//0
XM_007998519.1	-0.9296	4.12E-28	1.19E-26	sp|Q02818|NUCB1_HUMAN Nucleobindin-1 OS=Homo sapiens GN=NUCB1 PE=1 SV=4//1.207e-150
XM_007998522.1	-1.3412	1.07E-16	1.80E-15	sp|Q8TD43|TRPM4_HUMAN Transient receptor potential cation channel subfamily M member 4 OS=Homo sapiens GN=TRPM4 PE=1 SV=1//0
XM_007998523.1	-1.1616	1.15E-14	1.71E-13	sp|Q8SPV9|FCGRN_MACFA IgG receptor FcRn large subunit p51 OS=Macaca fascicularis GN=FCGRT PE=2 SV=1//0
XM_007998525.1	-0.45841	0.0002508	0.0015551	sp|Q9HB09|B2L12_HUMAN Bcl-2-like protein 12 OS=Homo sapiens GN=BCL2L12 PE=1 SV=1//2.67728e-143
XM_007998526.1	0.25534	0.002418	0.012914	sp|Q63009|ANM1_RAT Protein arginine N-methyltransferase 1 OS=Rattus norvegicus GN=Prmt1 PE=1 SV=1//0
XM_007998527.1	-0.90759	1.95E-28	5.68E-27	sp|O95782|AP2A1_HUMAN AP-2 complex subunit alpha-1 OS=Homo sapiens GN=AP2A1 PE=1 SV=3//0
XM_007998528.1	-1.2546	1.11E-32	3.75E-31	sp|Q7Z406|MYH14_HUMAN Myosin-14 OS=Homo sapiens GN=MYH14 PE=1 SV=2//0
XM_007998541.1	3.2985	4.30E-25	1.10E-23	sp|Q8NHL6|LIRB1_HUMAN Leukocyte immunoglobulin-like receptor subfamily B member 1 OS=Homo sapiens GN=LILRB1 PE=1 SV=1//2.00476e-22
XM_007998544.1	Inf	0.0002907	0.0017854	sp|A8MWK0|FS2P1_HUMAN Putative fatty acid desaturase 2-like protein FADS2P1 OS=Homo sapiens GN=FADS2P1 PE=5 SV=2//0
XM_007998545.1	-2.1792	1.21E-34	4.44E-33	sp|A1L4H1|SRCRL_HUMAN Soluble scavenger receptor cysteine-rich domain-containing protein SSC5D OS=Homo sapiens GN=SSC5D PE=1 SV=3//3.74338e-68
XM_007998553.1	1.4487	1.08E-14	1.60E-13	sp|Q96SQ5|ZN587_HUMAN Zinc finger protein 587 OS=Homo sapiens GN=ZNF587 PE=1 SV=1//5.07451e-06
XM_007998556.1	1.3516	2.95E-19	5.68E-18	sp|Q8IVC4|ZN584_HUMAN Zinc finger protein 584 OS=Homo sapiens GN=ZNF584 PE=2 SV=1//4.43806e-15
XM_007998559.1	-2.2471	0.002812	0.014841	sp|Q9TSL6|UDB23_MACFA UDP-glucuronosyltransferase 2B23 OS=Macaca fascicularis GN=UGT2B23 PE=1 SV=1//0
XM_007998600.1	-2.3466	0.0004863	0.0029054	sp|O97827|LPHN3_BOVIN Latrophilin-3 OS=Bos taurus GN=LPHN3 PE=2 SV=1//0
XM_007998602.1	0.26466	4.05E-05	0.0002773	sp|P30876|RPB2_HUMAN DNA-directed RNA polymerase II subunit RPB2 OS=Homo sapiens GN=POLR2B PE=1 SV=1//0
XM_007998603.1	-2.4242	0.0001101	0.0007182	sp|Q4R9D1|UBE2C_MACFA Ubiquitin-conjugating enzyme E2 C OS=Macaca fascicularis GN=UBE2C PE=2 SV=1//1.02661e-95
XM_007998604.1	-0.96365	1.03E-45	5.48E-44	sp|Q16270|IBP7_HUMAN Insulin-like growth factor-binding protein 7 OS=Homo sapiens GN=IGFBP7 PE=1 SV=1//7.57085e-108
XM_007998623.1	-1.2948	2.61E-11	3.15E-10	sp|Q6T311|ARL9_HUMAN ADP-ribosylation factor-like protein 9 OS=Homo sapiens GN=ARL9 PE=2 SV=1//6.9046e-117
XM_007998624.1	0.74624	1.57E-13	2.19E-12	sp|Q06203|PUR1_HUMAN Amidophosphoribosyltransferase OS=Homo sapiens GN=PPAT PE=1 SV=1//0
XM_007998629.1	-0.42621	5.23E-06	4.00E-05	sp|P22234|PUR6_HUMAN Multifunctional protein ADE2 OS=Homo sapiens GN=PAICS PE=1 SV=3//0
XM_007998686.1	-0.84529	1.35E-16	2.23E-15	sp|Q9H8P0|PORED_HUMAN Polyprenol reductase OS=Homo sapiens GN=SRD5A3 PE=1 SV=1//0
XM_007998701.1	2.392	5.77E-73	6.26E-71	sp|Q9UKJ5|CHIC2_HUMAN Cysteine-rich hydrophobic domain-containing protein 2 OS=Homo sapiens GN=CHIC2 PE=1 SV=1//2.81135e-84
XM_007998719.1	1.0663	8.20E-13	1.09E-11	sp|Q9BPW5|RSLBB_HUMAN Ras-like protein family member 11B OS=Homo sapiens GN=RASL11B PE=2 SV=1//1.06326e-159
XM_007998731.1	-0.83945	4.16E-13	5.59E-12	sp|Q5R9U1|SGCB_PONAB Beta-sarcoglycan OS=Pongo abelii GN=SGCB PE=2 SV=1//0
XM_007998743.1	-3.5258	0.0031383	0.016382	sp|O02663|UD2B9_MACFA UDP-glucuronosyltransferase 2B9 OS=Macaca fascicularis GN=UGT2B9 PE=2 SV=1//0
XM_007998747.1	0.60932	1.56E-19	3.03E-18	sp|O76094|SRP72_HUMAN Signal recognition particle subunit SRP72 OS=Homo sapiens GN=SRP72 PE=1 SV=3//0
XM_007998749.1	-0.42308	1.43E-05	0.0001038	sp|P0C864|DANCR_HUMAN Putative uncharacterized protein DANCR OS=Homo sapiens GN=DANCR PE=5 SV=1//1.9707e-35
XM_007998761.1	-1.6427	1.90E-07	1.65E-06	sp|O43704|ST1B1_HUMAN Sulfotransferase family cytosolic 1B member 1 OS=Homo sapiens GN=SULT1B1 PE=1 SV=2//0
XM_007998762.1	-3.2074	6.93E-24	1.67E-22	sp|P49888|ST1E1_HUMAN Estrogen sulfotransferase OS=Homo sapiens GN=SULT1E1 PE=1 SV=1//0
XM_007998798.1	Inf	9.12E-11	1.05E-09	sp|Q9NRM1|ENAM_HUMAN Enamelin OS=Homo sapiens GN=ENAM PE=1 SV=3//0
XM_007998817.1	-2.0648	0.0006416	0.0037703	sp|Q7L099|RUFY3_HUMAN Protein RUFY3 OS=Homo sapiens GN=RUFY3 PE=1 SV=1//0
XM_007998818.1	0.68048	2.27E-08	2.16E-07	sp|Q9NQZ2|SAS10_HUMAN Something about silencing protein 10 OS=Homo sapiens GN=UTP3 PE=1 SV=1//0
XM_007998871.1	5.9391	6.90E-138	1.99E-135	sp|P46653|IL8_CERAT Interleukin-8 OS=Cercocebus atys GN=CXCL8 PE=3 SV=1//1.91344e-45
XM_007998875.1	Inf	0.0002961	0.0018171	sp|P09341|GROA_HUMAN Growth-regulated alpha protein OS=Homo sapiens GN=CXCL1 PE=1 SV=1//5.76234e-42
XM_007998882.1	5.1585	2.52E-41	1.14E-39	sp|P19876|CXCL3_HUMAN C-X-C motif chemokine 3 OS=Homo sapiens GN=CXCL3 PE=1 SV=1//1.03713e-35

XM_007998899.1	4.0614	6.48E-06	4.90E-05	sp|P15514|AREG_HUMAN Amphiregulin OS=Homo sapiens GN=AREG PE=1 SV=2//1.41382e-102
XM_007998939.1	Inf	6.68E-10	7.17E-09	sp|O14625|CXL11_HUMAN C-X-C motif chemokine 11 OS=Homo sapiens GN=CXCL11 PE=1 SV=1//2.81002e-54
XM_007998941.1	8.2657	1.54E-47	8.69E-46	sp|Q8MIZ1|CXL10_MACMU C-X-C motif chemokine 10 OS=Macaca mulatta GN=CXCL10 PE=3 SV=1//5.55659e-58
XM_007998942.1	Inf	0.0084127	0.040069	sp|Q07325|CXCL9_HUMAN C-X-C motif chemokine 9 OS=Homo sapiens GN=CXCL9 PE=1 SV=1//1.04477e-32
XM_007998945.1	-1.0452	3.04E-39	1.30E-37	sp|Q14108|SCRB2_HUMAN Lysosome membrane protein 2 OS=Homo sapiens GN=SCARB2 PE=1 SV=2//0
XM_007998948.1	1.3359	4.78E-28	1.37E-26	sp|O95210|STBD1_HUMAN Starch-binding domain-containing protein 1 OS=Homo sapiens GN=STBD1 PE=1 SV=1//0
XM_007998970.1	-0.76715	0.0034683	0.017909	sp|Q4R555|SEP11_MACFA Septin-11 OS=Macaca fascicularis GN=SEPT11 PE=2 SV=3//0
XM_007998972.1	-0.8558	1.13E-11	1.40E-10	sp|Q9NVA2|SEP11_HUMAN Septin-11 OS=Homo sapiens GN=SEPT11 PE=1 SV=3//0
XM_007998989.1	2.4288	4.18E-65	3.80E-63	sp|P12429|ANXA3_HUMAN Annexin A3 OS=Homo sapiens GN=ANXA3 PE=1 SV=3//0
XM_007998993.1	1.3564	4.25E-15	6.42E-14	sp|Q6TCH7|PAQR3_HUMAN Progestin and adipoQ receptor family member 3 OS=Homo sapiens GN=PAQR3 PE=1 SV=2//0
XM_007999054.1	-4.2732	0.0017353	0.0095284	sp|Q12913|PTPRJ_HUMAN Receptor-type tyrosine-protein phosphatase eta OS=Homo sapiens GN=PTPRJ PE=1 SV=3//0
XM_007999059.1	1.5831	3.50E-16	5.67E-15	sp|Q9H5L6|THAP9_HUMAN DNA transposase THAP9 OS=Homo sapiens GN=THAP9 PE=1 SV=2//0
XM_007999103.1	-2.026	0.0025171	0.013408	sp|Q8N3X1|FNBP4_HUMAN Formin-binding protein 4 OS=Homo sapiens GN=FNBP4 PE=1 SV=3//4.97195e-10
XM_007999132.1	2.9846	1.78E-11	2.17E-10	sp|Q8N264|RHG24_HUMAN Rho GTPase-activating protein 24 OS=Homo sapiens GN=ARHGAP24 PE=1 SV=2//0
XM_007999238.1	0.99992	6.15E-08	5.61E-07	sp|Q4W5G0|TIGD2_HUMAN Tigger transposable element-derived protein 2 OS=Homo sapiens GN=TIGD2 PE=3 SV=1//0
XM_007999348.1	-0.67899	5.59E-08	5.12E-07	sp|Q0MQG7|NDUS3_GORGO NADH dehydrogenase [ubiquinone] iron-sulfur protein 3, mitochondrial OS=Gorilla gorilla gorilla GN=NDUFS3 PE=2 SV=1//0
XM_007999384.1	-1.997	4.63E-14	6.62E-13	sp|Q6YNC8|H2AZ_SHEEP Histone H2A.Z OS=Ovis aries GN=H2AFZ PE=2 SV=3//1.84436e-70
XM_007999404.1	-1.4545	7.86E-08	7.10E-07	sp|Q9C0K1|S39A8_HUMAN Zinc transporter ZIP8 OS=Homo sapiens GN=SLC39A8 PE=2 SV=1//0
XM_007999408.1	0.55225	0.0014322	0.0079861	sp|O00462|MANBA_HUMAN Beta-mannosidase OS=Homo sapiens GN=MANBA PE=2 SV=3//0
XM_007999411.1	-1.2921	0.0099124	0.046484	sp|Q86UD5|SL9B2_HUMAN Mitochondrial sodium/hydrogen exchanger 9B2 OS=Homo sapiens GN=SLC9B2 PE=1 SV=2//0
XM_007999417.1	0.44829	0.0004979	0.0029717	sp|Q8N5K1|CISD2_HUMAN CDGSH iron-sulfur domain-containing protein 2 OS=Homo sapiens GN=CISD2 PE=1 SV=1//1.14577e-69
XM_007999425.1	-0.81947	6.99E-08	6.35E-07	sp|Q9BUT1|BDH2_HUMAN 3-hydroxybutyrate dehydrogenase type 2 OS=Homo sapiens GN=BDH2 PE=1 SV=2//1.19618e-160
XM_007999526.1	-1.6366	3.66E-08	3.40E-07	sp|Q9BZM1|PG12A_HUMAN Group XIIA secretory phospholipase A2 OS=Homo sapiens GN=PLA2G12A PE=1 SV=1//8.64671e-106
XM_007999552.1	2.0013	1.10E-11	1.36E-10	sp|Q8N8J7|CD032_HUMAN Uncharacterized protein C4orf32 OS=Homo sapiens GN=C4orf32 PE=2 SV=2//8.85689e-61
XM_007999570.1	-0.82658	0.0084806	0.040375	sp|Q5R844|MYL6_PONAB Myosin light polypeptide 6 OS=Pongo abelii GN=MYL6 PE=2 SV=3//1.20288e-102
XM_007999648.1	-1.2876	6.70E-11	7.78E-10	sp|Q6YNC8|H2AZ_SHEEP Histone H2A.Z OS=Ovis aries GN=H2AFZ PE=2 SV=3//7.97198e-71
XM_007999653.1	0.94924	3.45E-15	5.24E-14	sp|Q5R5N4|MET14_PONAB N6-adenosine-methyltransferase subunit METTL14 OS=Pongo abelii GN=METTL14 PE=2 SV=1//0
XM_007999662.1	0.94573	3.52E-12	4.50E-11	sp|Q70EK8|UBP53_HUMAN Inactive ubiquitin carboxyl-terminal hydrolase 53 OS=Homo sapiens GN=USP53 PE=2 SV=2//0
XM_007999671.1	-1.1068	7.54E-16	1.20E-14	sp|Q8WVX3|CD003_HUMAN Uncharacterized protein C4orf3 OS=Homo sapiens GN=C4orf3 PE=1 SV=2//9.86712e-07
XM_007999697.1	0.58013	0.0003718	0.0022597	sp|Q06265|EXOS9_HUMAN Exosome complex component RRP45 OS=Homo sapiens GN=EXOSC9 PE=1 SV=3//0
XM_007999729.1	0.86623	1.19E-05	8.70E-05	sp|Q6ZW61|BBS12_HUMAN Bardet-Biedl syndrome 12 protein OS=Homo sapiens GN=BBS12 PE=1 SV=2//0
XM_007999749.1	0.40517	0.0094226	0.044387	sp|Q4R5N9|PPAL_MACFA Lysosomal acid phosphatase OS=Macaca fascicularis GN=ACP2 PE=2 SV=1//0
XM_007999750.1	-1.1691	2.17E-07	1.88E-06	sp|Q6V0I7|FAT4_HUMAN Protocadherin Fat 4 OS=Homo sapiens GN=FAT4 PE=1 SV=2//0
XM_007999757.1	0.42501	0.0010047	0.0057382	sp|O95757|HS74L_HUMAN Heat shock 70 kDa protein 4L OS=Homo sapiens GN=HSPA4L PE=1 SV=3//0
XM_007999785.1	0.65383	3.15E-13	4.29E-12	sp|O15173|PGRC2_HUMAN Membrane-associated progesterone receptor component 2 OS=Homo sapiens GN=PGRMC2 PE=1 SV=1//6.9783e-85
XM_007999807.1	1.7481	9.25E-95	1.56E-92	sp|Q9UPY5|XCT_HUMAN Cystine/glutamate transporter OS=Homo sapiens

				GN=SLC7A11 PE=1 SV=1//0
XM_007999809.1	2.682	1.24E-08	1.20E-07	sp|Q9UK39|NOCT_HUMAN Nocturnin OS=Homo sapiens GN=CCRN4L PE=2 SV=2//0
XM_007999835.1	0.83047	9.25E-16	1.46E-14	sp|Q8WTS6|SETD7_HUMAN Histone-lysine N-methyltransferase SETD7 OS=Homo sapiens GN=SETD7 PE=1 SV=1//0
XM_007999857.1	5.2966	8.21E-07	6.78E-06	sp|P25874|UCP1_HUMAN Mitochondrial brown fat uncoupling protein 1 OS=Homo sapiens GN=UCP1 PE=2 SV=3//0
XM_007999858.1	0.52114	2.76E-08	2.60E-07	sp|Q6ZT07|TBCD9_HUMAN TBC1 domain family member 9 OS=Homo sapiens GN=TBC1D9 PE=2 SV=2//0
XM_007999860.1	-1.0877	0.0002883	0.0017718	sp|Q9ULK6|RN150_HUMAN RING finger protein 150 OS=Homo sapiens GN=RNF150 PE=2 SV=2//0
XM_007999861.1	2.2922	2.16E-95	3.76E-93	sp|Q5R9D9|ZN330_PONAB Zinc finger protein 330 OS=Pongo abelii GN=ZNF330 PE=2 SV=1//0
XM_007999873.1	1.2097	2.33E-33	8.09E-32	sp|Q8NB14|UBP38_HUMAN Ubiquitin carboxyl-terminal hydrolase 38 OS=Homo sapiens GN=USP38 PE=1 SV=2//0
XM_007999905.1	1.0298	0.0066648	0.03237	sp|Q8IVH4|MMAA_HUMAN Methylmalonic aciduria type A protein, mitochondrial OS=Homo sapiens GN=MMAA PE=1 SV=1//0
XM_007999914.1	1.7061	9.69E-46	5.18E-44	sp|Q17R98|ZN827_HUMAN Zinc finger protein 827 OS=Homo sapiens GN=ZNF827 PE=2 SV=1//0
XM_007999916.1	1.1816	8.70E-13	1.15E-11	sp|O75096|LRP4_HUMAN Low-density lipoprotein receptor-related protein 4 OS=Homo sapiens GN=LRP4 PE=1 SV=4//0
XM_007999933.1	0.50408	0.0001157	0.0007525	sp|Q14008|CKAP5_HUMAN Cytoskeleton-associated protein 5 OS=Homo sapiens GN=CKAP5 PE=1 SV=3//0
XM_007999949.1	1.7694	5.44E-40	2.37E-38	sp|Q9H9D4|ZN408_HUMAN Zinc finger protein 408 OS=Homo sapiens GN=ZNF408 PE=1 SV=1//0
XM_007999962.1	-1.8301	0.0001768	0.0011208	sp|D2I3C6|DCLK2_AILME Serine/threonine-protein kinase DCLK2 OS=Ailuropoda melanoleuca GN=DCLK2 PE=3 SV=1//0
XM_008000002.1	3.1424	4.08E-11	4.83E-10	sp|F1MNN4|FBXW7_BOVIN F-box/WD repeat-containing protein 7 OS=Bos taurus GN=FBXW7 PE=1 SV=2//0
XM_008000053.1	0.29356	0.0045301	0.022837	sp|O43660|PLRG1_HUMAN Pleiotropic regulator 1 OS=Homo sapiens GN=PLRG1 PE=1 SV=1//0
XM_008000090.1	-1.184	7.12E-16	1.13E-14	sp|P43234|CATO_HUMAN Cathepsin O OS=Homo sapiens GN=CTSO PE=2 SV=1//0
XM_008000121.1	-0.45252	0.0004293	0.0025876	sp|Q5RDD3|ETFD_PONAB Electron transfer flavoprotein-ubiquinone oxidoreductase, mitochondrial OS=Pongo abelii GN=ETFDH PE=2 SV=1//0
XM_008000152.1	1.745	1.16E-13	1.63E-12	sp|Q96HR8|NAF1_HUMAN H/ACA ribonucleoprotein complex non-core subunit NAF1 OS=Homo sapiens GN=NAF1 PE=1 SV=2//0
XM_008000153.1	1.4785	0.0057916	0.028489	sp|Q96HR8|NAF1_HUMAN H/ACA ribonucleoprotein complex non-core subunit NAF1 OS=Homo sapiens GN=NAF1 PE=1 SV=2//2.11103e-167
XM_008000190.1	Inf	2.63E-05	0.0001841	-//-
XM_008000209.1	-0.8814	6.29E-17	1.07E-15	sp|P16870|CBPE_HUMAN Carboxypeptidase E OS=Homo sapiens GN=CPE PE=1 SV=1//0
XM_008000217.1	-0.76178	1.71E-27	4.80E-26	sp|P05787|K2C8_HUMAN Keratin, type II cytoskeletal 8 OS=Homo sapiens GN=KRT8 PE=1 SV=7//0
XM_008000224.1	2.1951	4.39E-38	1.78E-36	sp|Q8WX93|PALLD_HUMAN Palladin OS=Homo sapiens GN=PALLD PE=1 SV=3//0
XM_008000232.1	-0.98175	9.42E-10	1.00E-08	sp|Q8N4T8|CBR4_HUMAN Carbonyl reductase family member 4 OS=Homo sapiens GN=CBR4 PE=1 SV=3//6.3159e-132
XM_008000256.1	-1.2582	0.0008934	0.0051372	sp|O75121|MFA3L_HUMAN Microfibrillar-associated protein 3-like OS=Homo sapiens GN=MFAP3L PE=2 SV=3//0
XM_008000262.1	-1.3487	3.13E-08	2.93E-07	sp|Q4R3M6|NDUS5_MACFA NADH dehydrogenase [ubiquinone] iron-sulfur protein 5 OS=Macaca fascicularis GN=NDUFS5 PE=3 SV=3//1.76667e-62
XM_008000290.1	2.5126	3.08E-128	8.04E-126	sp|Q8MJY8|PGDH_MACFA 15-hydroxyprostaglandin dehydrogenase [NAD(+)] OS=Macaca fascicularis GN=HPGD PE=2 SV=1//9.42077e-175
XM_008000327.1	0.39663	0.0001012	0.0006638	sp|P61009|SPCS3_HUMAN Signal peptidase complex subunit 3 OS=Homo sapiens GN=SPCS3 PE=1 SV=1//1.40311e-115
XM_008000329.1	2.3963	4.66E-56	3.25E-54	sp|P49767|VEGFC_HUMAN Vascular endothelial growth factor C OS=Homo sapiens GN=VEGFC PE=1 SV=1//0
XM_008000361.1	1.4964	2.00E-06	1.59E-05	sp|Q8N7P3|CLD22_HUMAN Claudin-22 OS=Homo sapiens GN=CLDN22 PE=2 SV=3//7.90506e-89
XM_008000364.1	2.5572	1.67E-59	1.28E-57	sp|Q9NXV6|CARF_HUMAN CDKN2A-interacting protein OS=Homo sapiens GN=CDKN2AIP PE=1 SV=3//8.06536e-87
XM_008000518.1	1.1591	4.64E-09	4.65E-08	sp|P30946|HS90A_RABIT Heat shock protein HSP 90-alpha OS=Oryctolagus cuniculus GN=HSP90AA1 PE=1 SV=2//2.80693e-121
XM_008000523.1	-1.6719	3.62E-05	0.0002497	sp|Q8N3Y3|LARG2_HUMAN Glycosyltransferase-like protein LARGE2 OS=Homo sapiens GN=GYLTL1B PE=1 SV=2//0
XM_008000527.1	0.91892	2.64E-13	3.61E-12	sp|Q6UWZ7|F175A_HUMAN BRCA1-A complex subunit Abraxas OS=Homo sapiens GN=FAM175A PE=1 SV=2//0
XM_008000529.1	-0.99347	3.04E-23	7.12E-22	sp|P11766|ADHX_HUMAN Alcohol dehydrogenase class-3 OS=Homo sapiens GN=ADH5 PE=1 SV=4//0
XM_008000540.1	1.8035	2.69E-27	7.50E-26	sp|Q96NL6|SCLT1_HUMAN Sodium channel and clathrin linker 1 OS=Homo sapiens GN=SCLT1 PE=1 SV=2//0

XM_008000545.1	2.448	7.83E-17	1.32E-15	-//-
XM_008000546.1	2.0758	3.31E-56	2.32E-54	sp|Q49AN0|CRY2_HUMAN Cryptochrome-2 OS=Homo sapiens GN=CRY2 PE=1 SV=2//0
XM_008000561.1	0.84591	9.39E-24	2.25E-22	sp|P49716|CEBPD_HUMAN CCAAT/enhancer-binding protein delta OS=Homo sapiens GN=CEBPD PE=1 SV=2//5.16075e-109
XM_008000567.1	0.51094	3.46E-09	3.50E-08	sp|P33991|MCM4_HUMAN DNA replication licensing factor MCM4 OS=Homo sapiens GN=MCM4 PE=1 SV=5//0
XM_008000574.1	3.4182	4.53E-84	6.41E-82	sp|O43623|SNAI2_HUMAN Zinc finger protein SNAI2 OS=Homo sapiens GN=SNAI2 PE=1 SV=1//1.96065e-170
XM_008000618.1	6.5663	2.43E-21	5.16E-20	sp|Q6UXT8|F150A_HUMAN Protein FAM150A OS=Homo sapiens GN=FAM150A PE=2 SV=1//2.39438e-53
XM_008000633.1	-1.7431	3.89E-21	8.19E-20	sp|A6NMD0|IFM10_HUMAN Interferon-induced transmembrane protein 10 OS=Homo sapiens GN=IFITM10 PE=2 SV=1//1.596e-121
XM_008000656.1	4.5053	5.77E-193	2.69E-190	sp|Q9H6I2|SOX17_HUMAN Transcription factor SOX-17 OS=Homo sapiens GN=SOX17 PE=1 SV=1//0
XM_008000681.1	-0.4561	2.14E-06	1.70E-05	sp|P60868|RS20_RAT 40S ribosomal protein S20 OS=Rattus norvegicus GN=Rps20 PE=3 SV=1//2.19764e-80
XM_008000697.1	-0.52477	2.27E-10	2.54E-09	sp|Q9NX62|IMPA3_HUMAN Inositol monophosphatase 3 OS=Homo sapiens GN=IMPAD1 PE=1 SV=1//0
XM_008000698.1	-0.75836	0.0029352	0.015417	sp|Q9Y6H1|CHCH2_HUMAN Coiled-coil-helix-coiled-coil-helix domain-containing protein 2 OS=Homo sapiens GN=CHCHD2 PE=1 SV=1//5.68339e-39
XM_008000699.1	2.8974	1.90E-77	2.30E-75	sp|Q5R5R3|F110B_PONAB Protein FAM110B OS=Pongo abelii GN=FAM110B PE=2 SV=1//0
XM_008000701.1	-0.44278	0.0001126	0.0007336	sp|Q14CS0|UBX2B_HUMAN UBX domain-containing protein 2B OS=Homo sapiens GN=UBXN2B PE=1 SV=1//0
XM_008000736.1	-1.6029	8.44E-84	1.17E-81	sp|P07339|CATD_HUMAN Cathepsin D OS=Homo sapiens GN=CTSD PE=1 SV=1//0
XM_008000744.1	1.2917	2.36E-05	0.0001664	-//-
XM_008000762.1	0.69729	2.19E-05	0.0001553	sp|Q5RFN3|MTFR1_PONAB Mitochondrial fission regulator 1 OS=Pongo abelii GN=MTFR1 PE=2 SV=1//0
XM_008000775.1	1.5935	7.70E-30	2.36E-28	sp|Q15050|RRS1_HUMAN Ribosome biogenesis regulatory protein homolog OS=Homo sapiens GN=RRS1 PE=1 SV=2//0
XM_008000777.1	4.8319	4.54E-33	1.55E-31	sp|Q8TAG6|CH046_HUMAN Uncharacterized protein C8orf46 OS=Homo sapiens GN=C8orf46 PE=2 SV=2//8.62454e-119
XM_008000780.1	0.9328	1.03E-17	1.82E-16	sp|Q96JH7|VCIP1_HUMAN Deubiquitinating protein VCIP135 OS=Homo sapiens GN=VCPIP1 PE=1 SV=2//0
XM_008000798.1	0.91885	4.17E-13	5.60E-12	sp|Q1MSJ5|CSPP1_HUMAN Centrosome and spindle pole-associated protein 1 OS=Homo sapiens GN=CSPP1 PE=1 SV=4//0
XM_008000843.1	0.97601	9.61E-41	4.27E-39	sp|Q15629|TRAM1_HUMAN Translocating chain-associated membrane protein 1 OS=Homo sapiens GN=TRAM1 PE=1 SV=3//0
XM_008000844.1	2.0423	3.19E-05	0.000221	sp|Q5GH70|XKR9_HUMAN XK-related protein 9 OS=Homo sapiens GN=XKR9 PE=2 SV=1//0
XM_008000846.1	1.1578	3.74E-24	9.06E-23	sp|Q53H82|LACB2_HUMAN Beta-lactamase-like protein 2 OS=Homo sapiens GN=LACTB2 PE=1 SV=2//0
XM_008000872.1	-1.6606	3.25E-18	5.90E-17	sp|P27701|CD82_HUMAN CD82 antigen OS=Homo sapiens GN=CD82 PE=1 SV=1//5.07108e-146
XM_008000873.1	-1.8662	5.61E-48	3.23E-46	sp|Q8IZV5|RDH10_HUMAN Retinol dehydrogenase 10 OS=Homo sapiens GN=RDH10 PE=1 SV=1//0
XM_008000874.1	-0.79908	1.57E-28	4.58E-27	sp|Q5R9R4|RL7_PONAB 60S ribosomal protein L7 OS=Pongo abelii GN=RPL7 PE=2 SV=1//6.50561e-166
XM_008000910.1	2.3791	0.0028354	0.014954	sp|Q9HDC5|JPH1_HUMAN Junctophilin-1 OS=Homo sapiens GN=JPH1 PE=1 SV=2//0
XM_008000945.1	1.8271	1.10E-08	1.08E-07	sp|P13232|IL7_HUMAN Interleukin-7 OS=Homo sapiens GN=IL7 PE=1 SV=1//4.91853e-102
XM_008000950.1	-0.39524	0.0008819	0.0050777	sp|Q32M88|ATHL1_HUMAN Acid trehalase-like protein 1 OS=Homo sapiens GN=ATHL1 PE=1 SV=2//0
XM_008000960.1	1.2526	0.0002344	0.0014601	sp|Q96DT7|ZBT10_HUMAN Zinc finger and BTB domain-containing protein 10 OS=Homo sapiens GN=ZBTB10 PE=1 SV=2//0
XM_008000962.1	1.8285	0.0001452	0.0009314	sp|Q96DT7|ZBT10_HUMAN Zinc finger and BTB domain-containing protein 10 OS=Homo sapiens GN=ZBTB10 PE=1 SV=2//0
XM_008000964.1	-2.0537	4.01E-13	5.40E-12	sp|Q6ZNC4|ZN704_HUMAN Zinc finger protein 704 OS=Homo sapiens GN=ZNF704 PE=1 SV=1//0
XM_008000966.1	1.9356	9.10E-46	4.88E-44	sp|Q9NWQ8|PHAG1_HUMAN Phosphoprotein associated with glycosphingolipid-enriched microdomains 1 OS=Homo sapiens GN=PAG1 PE=1 SV=2//0
XM_008000970.1	-1.288	0.0016217	0.0089495	sp|P15090|FABP4_HUMAN Fatty acid-binding protein, adipocyte OS=Homo sapiens GN=FABP4 PE=1 SV=3//1.10558e-87
XM_008000975.1	1.1545	1.26E-32	4.22E-31	sp|Q5R4X0|IMPA1_PONAB Inositol monophosphatase 1 OS=Pongo abelii GN=IMPA1 PE=2 SV=1//0
XM_008000979.1	1.5637	1.87E-57	1.36E-55	sp|Q8TCF1|ZFAN1_HUMAN AN1-type zinc finger protein 1 OS=Homo sapiens GN=ZFAND1 PE=1 SV=1//0
XM_008000996.1	-0.63229	1.04E-07	9.29E-07	sp|Q8N0T1|CH059_HUMAN Uncharacterized protein C8orf59 OS=Homo sapiens

				GN=C8orf59 PE=1 SV=4//1.37007e-51
XM_008001008.1	-0.89734	5.01E-10	5.46E-09	sp|P00918|CAH2_HUMAN Carbonic anhydrase 2 OS=Homo sapiens GN=CA2 PE=1 SV=2//0
XM_008001026.1	-0.65459	3.11E-13	4.24E-12	sp|O75131|CPNE3_HUMAN Copine-3 OS=Homo sapiens GN=CPNE3 PE=1 SV=1//0
XM_008001037.1	2.9557	3.26E-43	1.55E-41	sp|O43353|RIPK2_HUMAN Receptor-interacting serine/threonine-protein kinase 2 OS=Homo sapiens GN=RIPK2 PE=1 SV=2//0
XM_008001045.1	-0.87668	1.61E-16	2.65E-15	sp|Q16698|DECR_HUMAN 2,4-dienoyl-CoA reductase, mitochondrial OS=Homo sapiens GN=DECR1 PE=1 SV=1//0
XM_008001046.1	-3.018	0.0018481	0.010072	sp|P05937|CALB1_HUMAN Calbindin OS=Homo sapiens GN=CALB1 PE=1 SV=2//5.74667e-179
XM_008001066.1	0.81641	0.0002114	0.0013264	sp|Q06455|MTG8_HUMAN Protein CBFA2T1 OS=Homo sapiens GN=RUNX1T1 PE=1 SV=2//0
XM_008001152.1	-1.5401	8.36E-34	2.97E-32	sp|P14927|QCR7_HUMAN Cytochrome b-c1 complex subunit 7 OS=Homo sapiens GN=UQCRB PE=1 SV=2//5.28997e-64
XM_008001154.1	1.1398	2.00E-11	2.43E-10	sp|Q96E29|MTEF3_HUMAN Transcription termination factor 3, mitochondrial OS=Homo sapiens GN=MTERF3 PE=1 SV=2//0
XM_008001161.1	-1.1667	5.58E-20	1.10E-18	sp|P63159|HMGB1_RAT High mobility group protein B1 OS=Rattus norvegicus GN=Hmgb1 PE=1 SV=2//2.91306e-103
XM_008001167.1	-0.19877	0.0044216	0.022347	sp|Q86VI4|LAP4B_HUMAN Lysosomal-associated transmembrane protein 4B OS=Homo sapiens GN=LAPTM4B PE=1 SV=1//1.5966e-146
XM_008001180.1	-0.68964	2.06E-15	3.16E-14	sp|P52758|UK114_HUMAN Ribonuclease UK114 OS=Homo sapiens GN=HRSP12 PE=1 SV=1//5.24832e-92
XM_008001189.1	-1.3801	0.0002973	0.001824	sp|Q7YRK2|COX6C_MACSL Cytochrome c oxidase subunit 6C OS=Macaca silenus GN=COX6C PE=3 SV=1//1.36489e-44
XM_008001220.1	1.1704	1.15E-32	3.88E-31	sp|Q9NV58|RN19A_HUMAN E3 ubiquitin-protein ligase RNF19A OS=Homo sapiens GN=RNF19A PE=1 SV=3//0
XM_008001255.1	0.65571	1.86E-14	2.73E-13	sp|Q4R741|RIR2B_MACFA Ribonucleoside-diphosphate reductase subunit M2 B OS=Macaca fascicularis GN=RRM2B PE=2 SV=1//0
XM_008001264.1	3.7965	0	0	sp|Q13118|KLF10_HUMAN Krueppel-like factor 10 OS=Homo sapiens GN=KLF10 PE=1 SV=1//0
XM_008001268.1	0.9733	6.52E-42	3.00E-40	sp|Q5RDQ7|VATC1_PONAB V-type proton ATPase subunit C 1 OS=Pongo abelii GN=ATP6V1C1 PE=2 SV=1//0
XM_008001272.1	-1.5738	0.0013713	0.0076799	sp|Q96CG8|CTHR1_HUMAN Collagen triple helix repeat-containing protein 1 OS=Homo sapiens GN=CTHRC1 PE=1 SV=1//4.47979e-162
XM_008001274.1	0.91836	1.78E-26	4.81E-25	sp|Q5R4T8|DCA13_PONAB DDB1- and CUL4-associated factor 13 OS=Pongo abelii GN=DCAF13 PE=2 SV=1//0
XM_008001275.1	1.5597	4.64E-48	2.67E-46	sp|Q95J75|MFTC_MACFA Mitochondrial folate transporter/carrier OS=Macaca fascicularis GN=SLC25A32 PE=2 SV=1//0
XM_008001355.1	0.64198	4.03E-12	5.13E-11	sp|Q15006|EMC2_HUMAN ER membrane protein complex subunit 2 OS=Homo sapiens GN=EMC2 PE=1 SV=1//0
XM_008001359.1	-0.33512	7.88E-05	0.0005242	sp|Q96RS6|NUDC1_HUMAN NudC domain-containing protein 1 OS=Homo sapiens GN=NUDCD1 PE=1 SV=2//0
XM_008001406.1	-0.51384	1.13E-13	1.60E-12	sp|O15372|EIF3H_HUMAN Eukaryotic translation initiation factor 3 subunit H OS=Homo sapiens GN=EIF3H PE=1 SV=1//0
XM_008001407.1	1.0787	2.32E-29	6.98E-28	sp|Q9BRU9|UTP23_HUMAN rRNA-processing protein UTP23 homolog OS=Homo sapiens GN=UTP23 PE=1 SV=2//4.63467e-159
XM_008001418.1	0.17396	0.010748	0.049987	sp|A9X1C8|EXT1_PAPAN Exostosin-1 OS=Papio anubis GN=EXT1 PE=3 SV=1//0
XM_008001451.1	-0.49195	7.55E-08	6.83E-07	sp|Q9BYD1|RM13_HUMAN 39S ribosomal protein L13, mitochondrial OS=Homo sapiens GN=MRPL13 PE=1 SV=1//1.82322e-126
XM_008001454.1	-0.35944	2.09E-05	0.0001484	sp|P22234|PUR6_HUMAN Multifunctional protein ADE2 OS=Homo sapiens GN=PAICS PE=1 SV=3//0
XM_008001480.1	0.62536	9.40E-06	6.95E-05	sp|Q96HA8|NTAQ1_HUMAN Protein N-terminal glutamine amidohydrolase OS=Homo sapiens GN=WDYHV1 PE=1 SV=2//1.49075e-146
XM_008001481.1	1.0967	0.0002444	0.0015178	sp|Q969P5|FBX32_HUMAN F-box only protein 32 OS=Homo sapiens GN=FBXO32 PE=1 SV=1//0
XM_008001482.1	0.77809	6.08E-06	4.61E-05	sp|Q658Y4|F91A1_HUMAN Protein FAM91A1 OS=Homo sapiens GN=FAM91A1 PE=1 SV=3//0
XM_008001491.1	0.87513	9.94E-24	2.38E-22	sp|Q4VAE3|TMM65_MOUSE Transmembrane protein 65 OS=Mus musculus GN=Tmem65 PE=2 SV=1//3.34095e-133
XM_008001492.1	1.2516	1.16E-36	4.48E-35	sp|Q8WU17|RN139_HUMAN E3 ubiquitin-protein ligase RNF139 OS=Homo sapiens GN=RNF139 PE=1 SV=1//0
XM_008001493.1	0.61841	7.57E-06	5.67E-05	sp|Q4R3U8|TYW2_MACFA tRNA wybutosine-synthesizing protein 2 homolog OS=Macaca fascicularis GN=TRMT12 PE=2 SV=2//3.5959e-36
XM_008001494.1	-0.60822	1.17E-12	1.54E-11	sp|Q0MQE9|NDUB9_GORGO NADH dehydrogenase [ubiquinone] 1 beta subcomplex subunit 9 OS=Gorilla gorilla gorilla GN=NDUFB9 PE=2 SV=3//1.78253e-117
XM_008001509.1	-0.61209	6.59E-17	1.12E-15	sp|Q14534|ERG1_HUMAN Squalene monooxygenase OS=Homo sapiens GN=SQLE PE=1 SV=3//0
XM_008001521.1	2.2351	3.76E-112	8.68E-110	sp|Q96RU8|TRIB1_HUMAN Tribbles homolog 1 OS=Homo sapiens GN=TRIB1 PE=1 SV=2//0
XM_008001522.1	0.73773	5.78E-15	8.72E-14	sp|Q96KN1|FA84B_HUMAN Protein FAM84B OS=Homo sapiens GN=FAM84B PE=1

				SV=1//7.97128e-177
XM_008001653.1	-1.1629	7.41E-07	6.15E-06	sp|O75365|TP4A3_HUMAN Protein tyrosine phosphatase type IVA 3 OS=Homo sapiens GN=PTP4A3 PE=1 SV=2//6.67908e-120
XM_008001693.1	-0.72074	2.98E-09	3.04E-08	sp|Q8WUY1|THEM6_HUMAN Protein THEM6 OS=Homo sapiens GN=THEM6 PE=1 SV=2//2.6385e-120
XM_008001702.1	-1.2874	2.58E-13	3.54E-12	sp|Q14210|LY6D_HUMAN Lymphocyte antigen 6D OS=Homo sapiens GN=LY6D PE=1 SV=1//3.6993e-43
XM_008001722.1	1.6023	2.92E-45	1.52E-43	sp|B6CJY4|TRAF6_CERAT TNF receptor-associated factor 6 OS=Cercocebus atys GN=TRAF6 PE=2 SV=1//0
XM_008001735.1	1.2047	5.16E-17	8.78E-16	-//-
XM_008001755.1	-2.7194	0.0002175	0.0013602	sp|Q969P6|TOP1M_HUMAN DNA topoisomerase I, mitochondrial OS=Homo sapiens GN=TOP1MT PE=1 SV=1//0
XM_008001757.1	-2.0834	0.0002613	0.0016153	sp|Q969P6|TOP1M_HUMAN DNA topoisomerase I, mitochondrial OS=Homo sapiens GN=TOP1MT PE=1 SV=1//2.77684e-08
XM_008001782.1	1.7058	4.39E-41	1.97E-39	sp|Q53EQ6|TIGD5_HUMAN Tigger transposable element-derived protein 5 OS=Homo sapiens GN=TIGD5 PE=1 SV=3//0
XM_008001795.1	-0.32411	0.0032737	0.017048	sp|Q4R531|P5CR3_MACFA Pyrroline-5-carboxylate reductase 3 OS=Macaca fascicularis GN=PYCRL PE=2 SV=1//2.69836e-180
XM_008001796.1	-0.34263	0.0004924	0.0029408	sp|Q13630|FCL_HUMAN GDP-L-fucose synthase OS=Homo sapiens GN=TSTA3 PE=1 SV=1//0
XM_008001870.1	-0.88843	5.11E-12	6.47E-11	sp|Q7Z429|LFG1_HUMAN Protein lifeguard 1 OS=Homo sapiens GN=GRINA PE=2 SV=1//2.06148e-138
XM_008001882.1	-1.884	0.01016	0.047472	sp|A6NE52|K1875_HUMAN WD repeat-containing protein KIAA1875 OS=Homo sapiens GN=KIAA1875 PE=2 SV=2//8.04579e-12
XM_008001883.1	-1.21	6.47E-15	9.73E-14	sp|Q9D0U6|MAF1_MOUSE Repressor of RNA polymerase III transcription MAF1 homolog OS=Mus musculus GN=Maf1 PE=2 SV=1//2.54494e-158
XM_008001885.1	-0.51475	9.72E-07	7.97E-06	sp|Q9H0F6|SHRPN_HUMAN Sharpin OS=Homo sapiens GN=SHARPIN PE=1 SV=1//6.20811e-10
XM_008001886.1	-0.38748	2.78E-07	2.39E-06	sp|P08574|CY1_HUMAN Cytochrome c1, heme protein, mitochondrial OS=Homo sapiens GN=CYC1 PE=1 SV=3//3.1879e-169
XM_008001887.1	0.98924	1.97E-14	2.88E-13	sp|Q9BTY7|HGH1_HUMAN Protein HGH1 homolog OS=Homo sapiens GN=HGH1 PE=1 SV=1//1.03054e-176
XM_008001907.1	0.30727	0.0007178	0.0041842	sp|Q14137|BOP1_HUMAN Ribosome biogenesis protein BOP1 OS=Homo sapiens GN=BOP1 PE=1 SV=2//0
XM_008001912.1	-0.89174	3.06E-16	4.96E-15	sp|Q9GMF1|DGAT1_CHLAE Diacylglycerol O-acyltransferase 1 OS=Chlorocebus aethiops GN=DGAT1 PE=2 SV=1//0
XM_008001976.1	-0.85624	7.20E-22	1.58E-20	sp|Q86VR8|FJX1_HUMAN Four-jointed box protein 1 OS=Homo sapiens GN=FJX1 PE=2 SV=1//0
XM_008002031.1	1.196	1.91E-33	6.67E-32	sp|Q9XSR1|ZN252_CANFA Zinc finger protein 252 OS=Canis familiaris GN=ZNF252 PE=2 SV=1//0
XM_008002032.1	0.55985	4.76E-07	4.01E-06	sp|Q5RDQ4|CH033_PONAB UPF0488 protein C8orf33 homolog OS=Pongo abelii PE=2 SV=1//2.63066e-99
XM_008002037.1	2.6643	7.12E-07	5.91E-06	-//-
XM_008002040.1	1.0549	0.0093392	0.044032	-//-
XM_008002050.1	-0.41569	5.24E-05	0.0003552	sp|Q7Z388|D19L4_HUMAN Probable C-mannosyltransferase DPY19L4 OS=Homo sapiens GN=DPY19L4 PE=1 SV=1//0
XM_008002052.1	-2.2385	0.0082695	0.039455	-//-
XM_008002054.1	1.1728	1.64E-61	1.35E-59	sp|Q6PL18|ATAD2_HUMAN ATPase family AAA domain-containing protein 2 OS=Homo sapiens GN=ATAD2 PE=1 SV=1//6.34445e-14
XM_008002056.1	3.2609	3.42E-32	1.14E-30	-//-
XM_008002060.1	-1.5947	0.0005414	0.0032222	sp|A6NE52|K1875_HUMAN WD repeat-containing protein KIAA1875 OS=Homo sapiens GN=KIAA1875 PE=2 SV=2//0
XM_008002088.1	-1.5147	0.0022572	0.01211	-//-
XM_008002114.1	5.4728	0	0	sp|Q99612|KLF6_HUMAN Krueppel-like factor 6 OS=Homo sapiens GN=KLF6 PE=1 SV=3//2.06126e-145
XM_008002130.1	-1.0689	1.80E-21	3.86E-20	sp|Q95JH7|AK1C1_MACFA Aldo-keto reductase family 1 member C1 homolog OS=Macaca fascicularis GN=AKR1C1 PE=1 SV=1//0
XM_008002163.1	1.4432	0.0005748	0.0034041	sp|Q5VWN6|F208B_HUMAN Protein FAM208B OS=Homo sapiens GN=FAM208B PE=1 SV=1//0
XM_008002176.1	1.6124	5.69E-06	4.32E-05	sp|Q6P6B7|ANR16_HUMAN Ankyrin repeat domain-containing protein 16 OS=Homo sapiens GN=ANKRD16 PE=1 SV=1//0
XM_008002195.1	0.62073	4.37E-12	5.56E-11	sp|Q96I25|SPF45_HUMAN Splicing factor 45 OS=Homo sapiens GN=RBM17 PE=1 SV=1//0
XM_008002210.1	0.66886	3.39E-10	3.75E-09	sp|O00330|ODPX_HUMAN Pyruvate dehydrogenase protein X component, mitochondrial OS=Homo sapiens GN=PDHX PE=1 SV=3//0
XM_008002219.1	-0.61805	4.66E-12	5.91E-11	sp|P19823|ITIH2_HUMAN Inter-alpha-trypsin inhibitor heavy chain H2 OS=Homo sapiens GN=ITIH2 PE=1 SV=2//0
XM_008002222.1	1.1828	1.03E-22	2.35E-21	sp|O60870|KIN17_HUMAN DNA/RNA-binding protein KIN17 OS=Homo sapiens GN=KIN PE=1 SV=2//0

XM_008002223.1	0.92634	8.52E-16	1.35E-14	sp|Q5VWG9|TAF3_HUMAN Transcription initiation factor TFIID subunit 3 OS=Homo sapiens GN=TAF3 PE=1 SV=1//1.20559e-55
XM_008002257.1	1.1933	2.12E-19	4.10E-18	sp|Q86WR7|PRSR2_HUMAN Proline and serine-rich protein 2 OS=Homo sapiens GN=PROSER2 PE=1 SV=2//0
XM_008002260.1	0.80602	9.34E-15	1.40E-13	sp|Q9JLR1|S61A2_MOUSE Protein transport protein Sec61 subunit alpha isoform 2 OS=Mus musculus GN=Sec61a2 PE=2 SV=3//0
XM_008002263.1	-0.45549	0.0002321	0.0014463	sp|Q9UKK9|NUDT5_HUMAN ADP-sugar pyrophosphatase OS=Homo sapiens GN=NUDT5 PE=1 SV=1//3.54343e-148
XM_008002289.1	-0.92589	8.43E-14	1.19E-12	sp|O14832|PAHX_HUMAN Phytanoyl-CoA dioxygenase, peroxisomal OS=Homo sapiens GN=PHYH PE=1 SV=1//0
XM_008002340.1	2.2529	7.08E-19	1.33E-17	sp|Q5RE21|HSP7E_PONAB Heat shock 70 kDa protein 14 OS=Pongo abelii GN=HSPA14 PE=2 SV=1//4.75728e-40
XM_008002350.1	-1.537	3.16E-61	2.57E-59	sp|P04040|CATA_HUMAN Catalase OS=Homo sapiens GN=CAT PE=1 SV=3//0
XM_008002365.1	1.4408	1.06E-10	1.21E-09	sp|O60551|NMT2_HUMAN Glycylpeptide N-tetradecanoyltransferase 2 OS=Homo sapiens GN=NMT2 PE=1 SV=1//0
XM_008002369.1	Inf	4.04E-06	3.12E-05	sp|P53708|ITA8_HUMAN Integrin alpha-8 OS=Homo sapiens GN=ITGA8 PE=1 SV=3//0
XM_008002379.1	1.8271	3.07E-101	5.70E-99	sp|Q8N961|ABTB2_HUMAN Ankyrin repeat and BTB/POZ domain-containing protein 2 OS=Homo sapiens GN=ABTB2 PE=2 SV=2//0
XM_008002385.1	1.9297	2.79E-09	2.85E-08	sp|O60494|CUBN_HUMAN Cubilin OS=Homo sapiens GN=CUBN PE=1 SV=5//0
XM_008002386.1	0.72428	0.0032207	0.01678	sp|O14717|TRDMT_HUMAN tRNA (cytosine(38)-C(5))-methyltransferase OS=Homo sapiens GN=TRDMT1 PE=1 SV=1//0
XM_008002391.1	1.6141	6.73E-25	1.70E-23	sp|Q9H0A0|NAT10_HUMAN N-acetyltransferase 10 OS=Homo sapiens GN=NAT10 PE=1 SV=2//0
XM_008002397.1	-0.70688	1.26E-27	3.54E-26	sp|Q4R4X4|VIME_MACFA Vimentin OS=Macaca fascicularis GN=VIM PE=2 SV=3//0
XM_008002400.1	0.22501	0.0013814	0.0077344	sp|Q14444|CAPR1_HUMAN Caprin-1 OS=Homo sapiens GN=CAPRIN1 PE=1 SV=2//0
XM_008002432.1	1.1028	1.72E-36	6.62E-35	sp|Q9D4P0|ARL5B_MOUSE ADP-ribosylation factor-like protein 5B OS=Mus musculus GN=Arl5b PE=2 SV=3//1.98025e-116
XM_008002433.1	1.4489	1.66E-25	4.33E-24	sp|Q8TEA1|NSUN6_HUMAN Putative methyltransferase NSUN6 OS=Homo sapiens GN=NSUN6 PE=1 SV=1//0
XM_008002454.1	1.6218	8.62E-26	2.27E-24	sp|Q5T4H9|CSC10_HUMAN Protein CASC10 OS=Homo sapiens GN=CASC10 PE=2 SV=1//9.02644e-43
XM_008002473.1	1.0173	0.0097847	0.045974	sp|Q96KC8|DNJC1_HUMAN DnaJ homolog subfamily C member 1 OS=Homo sapiens GN=DNAJC1 PE=1 SV=1//0
XM_008002475.1	-0.54625	0.000406	0.002455	sp|Q9UBI1|COMD3_HUMAN COMM domain-containing protein 3 OS=Homo sapiens GN=COMMD3 PE=1 SV=1//4.71813e-139
XM_008002499.1	-1.6316	6.20E-20	1.22E-18	sp|Q9Y3D2|MSRB2_HUMAN Methionine-R-sulfoxide reductase B2, mitochondrial OS=Homo sapiens GN=MSRB2 PE=1 SV=2//1.35142e-97
XM_008002513.1	2.474	2.50E-71	2.62E-69	sp|Q5VV17|OTUD1_HUMAN OTU domain-containing protein 1 OS=Homo sapiens GN=OTUD1 PE=1 SV=1//0
XM_008002522.1	3.5409	0.0044577	0.022511	sp|Q5T5P2|SKT_HUMAN Sickle tail protein homolog OS=Homo sapiens GN=KIAA1217 PE=1 SV=2//0
XM_008002559.1	1.3515	0.0099751	0.046748	sp|Q5T848|GP158_HUMAN Probable G-protein coupled receptor 158 OS=Homo sapiens GN=GPR158 PE=1 SV=1//0
XM_008002595.1	1.4958	5.51E-16	8.82E-15	sp|Q5T2R2|DPS1_HUMAN Decaprenyl-diphosphate synthase subunit 1 OS=Homo sapiens GN=PDSS1 PE=1 SV=1//0
XM_008002666.1	1.3151	2.27E-45	1.18E-43	sp|Q13145|BAMBI_HUMAN BMP and activin membrane-bound inhibitor homolog OS=Homo sapiens GN=BAMBI PE=1 SV=1//1.19852e-173
XM_008002671.1	1.2179	1.35E-59	1.04E-57	sp|O95425|SVIL_HUMAN Supervillin OS=Homo sapiens GN=SVIL PE=1 SV=2//0
XM_008002673.1	-0.32079	0.0004101	0.0024779	sp|Q2KHR3|QSER1_HUMAN Glutamine and serine-rich protein 1 OS=Homo sapiens GN=QSER1 PE=1 SV=3//0
XM_008002723.1	1.0917	5.59E-39	2.35E-37	sp|P33176|KINH_HUMAN Kinesin-1 heavy chain OS=Homo sapiens GN=KIF5B PE=1 SV=1//0
XM_008002752.1	-0.37289	8.99E-09	8.82E-08	sp|P05556|ITB1_HUMAN Integrin beta-1 OS=Homo sapiens GN=ITGB1 PE=1 SV=2//0
XM_008002753.1	0.65719	9.84E-13	1.30E-11	sp|Q7L2H7|EIF3M_HUMAN Eukaryotic translation initiation factor 3 subunit M OS=Homo sapiens GN=EIF3M PE=1 SV=1//0
XM_008002812.1	-0.63545	1.55E-18	2.87E-17	sp|Q15293|RCN1_HUMAN Reticulocalbin-1 OS=Homo sapiens GN=RCN1 PE=1 SV=1//0
XM_008002829.1	0.58305	2.41E-10	2.69E-09	sp|Q92615|LAR4B_HUMAN La-related protein 4B OS=Homo sapiens GN=LARP4B PE=1 SV=3//0
XM_008002831.1	0.39812	1.10E-07	9.82E-07	sp|Q5JRX3|PREP_HUMAN Presequence protease, mitochondrial OS=Homo sapiens GN=PITRM1 PE=1 SV=3//0
XM_008002835.1	-1.6991	2.55E-07	2.20E-06	-//-
XM_008002837.1	1.1623	1.86E-09	1.94E-08	sp|Q5R6Z9|DCR1C_PONAB Protein artemis OS=Pongo abelii GN=DCLRE1C PE=2 SV=1//0
XM_008002839.1	0.5823	1.04E-13	1.47E-12	sp|O88967|YMEL1_MOUSE ATP-dependent zinc metalloprotease YME1L1 OS=Mus musculus GN=Yme1l1 PE=2 SV=1//0

XM_008002866.1	-1.1367	7.15E-11	8.28E-10	sp|Q96QE2|MYCT_HUMAN Proton myo-inositol cotransporter OS=Homo sapiens GN=SLC2A13 PE=1 SV=3//0
XM_008002932.1	0.91244	2.12E-08	2.02E-07	sp|P13164|IFM1_HUMAN Interferon-induced transmembrane protein 1 OS=Homo sapiens GN=IFITM1 PE=1 SV=3//1.0825e-77
XM_008002957.1	0.68353	9.25E-22	2.01E-20	sp|Q68CP9|ARID2_HUMAN AT-rich interactive domain-containing protein 2 OS=Homo sapiens GN=ARID2 PE=1 SV=2//0
XM_008003068.1	1.0963	4.09E-05	0.0002801	sp|Q6P3W2|DJC24_HUMAN DnaJ homolog subfamily C member 24 OS=Homo sapiens GN=DNAJC24 PE=1 SV=1//1.45269e-89
XM_008003079.1	-1.7215	4.28E-05	0.0002923	sp|P54284|CACB3_HUMAN Voltage-dependent L-type calcium channel subunit beta-3 OS=Homo sapiens GN=CACNB3 PE=1 SV=1//0
XM_008003080.1	-1.3757	6.67E-07	5.55E-06	sp|P54284|CACB3_HUMAN Voltage-dependent L-type calcium channel subunit beta-3 OS=Homo sapiens GN=CACNB3 PE=1 SV=1//0
XM_008003082.1	0.2479	0.0006665	0.0039009	sp|Q9BUQ8|DDX23_HUMAN Probable ATP-dependent RNA helicase DDX23 OS=Homo sapiens GN=DDX23 PE=1 SV=3//0
XM_008003083.1	2.7116	2.07E-07	1.80E-06	sp|Q92730|RND1_HUMAN Rho-related GTP-binding protein Rho6 OS=Homo sapiens GN=RND1 PE=1 SV=1//2.08517e-162
XM_008003085.1	-1.9768	0.0082518	0.039383	sp|Q6P3W2|DJC24_HUMAN DnaJ homolog subfamily C member 24 OS=Homo sapiens GN=DNAJC24 PE=1 SV=1//2.23618e-60
XM_008003125.1	-0.73243	1.18E-26	3.20E-25	sp|Q6P9V9|TBA1B_RAT Tubulin alpha-1B chain OS=Rattus norvegicus GN=Tuba1b PE=1 SV=1//0
XM_008003130.1	-1.1853	7.83E-56	5.44E-54	sp|Q9BQE3|TBA1C_HUMAN Tubulin alpha-1C chain OS=Homo sapiens GN=TUBA1C PE=1 SV=1//0
XM_008003131.1	-1.564	1.50E-15	2.33E-14	sp|P41219|PERI_HUMAN Peripherin OS=Homo sapiens GN=PRPH PE=1 SV=2//0
XM_008003143.1	-1.2697	5.94E-11	6.94E-10	sp|Q5RBD7|DJC22_PONAB DnaJ homolog subfamily C member 22 OS=Pongo abelii GN=DNAJC22 PE=2 SV=1//0
XM_008003175.1	-1.2855	1.98E-80	2.56E-78	sp|Q5R7R1|BI1_PONAB Bax inhibitor 1 OS=Pongo abelii GN=TMBIM6 PE=2 SV=2//1.34919e-116
XM_008003181.1	0.89568	2.23E-10	2.50E-09	sp|Q7Z5W3|BN3D2_HUMAN Pre-miRNA 5'-monophosphate methyltransferase OS=Homo sapiens GN=BCDIN3D PE=1 SV=1//0
XM_008003203.1	#NAME?	0.0094269	0.044397	sp|P78348|ASIC1_HUMAN Acid-sensing ion channel 1 OS=Homo sapiens GN=ASIC1 PE=1 SV=3//0
XM_008003234.1	Inf	0.0007767	0.0045054	sp|P18846|ATF1_HUMAN Cyclic AMP-dependent transcription factor ATF-1 OS=Homo sapiens GN=ATF1 PE=1 SV=2//1.11133e-150
XM_008003237.1	-2.2045	4.23E-62	3.55E-60	sp|Q9H8H3|MET7A_HUMAN Methyltransferase-like protein 7A OS=Homo sapiens GN=METTL7A PE=1 SV=1//3.40548e-158
XM_008003248.1	-2.691	0.0024521	0.013081	sp|Q9H175|CSRN2_HUMAN Cysteine/serine-rich nuclear protein 2 OS=Homo sapiens GN=CSRNP2 PE=1 SV=1//0
XM_008003269.1	-0.73223	4.73E-10	5.16E-09	sp|Q0VAQ4|SMAGP_HUMAN Small cell adhesion glycoprotein OS=Homo sapiens GN=SMAGP PE=1 SV=1//7.31476e-55
XM_008003302.1	-2.4976	1.07E-15	1.68E-14	sp|A6NMB9|FIGL2_HUMAN Putative fidgetin-like protein 2 OS=Homo sapiens GN=FIGNL2 PE=5 SV=2//8.85103e-62
XM_008003308.1	-0.38722	7.42E-05	0.0004943	sp|P36896|ACV1B_HUMAN Activin receptor type-1B OS=Homo sapiens GN=ACVR1B PE=1 SV=1//0
XM_008003310.1	-3.0582	2.59E-05	0.0001815	-//-
XM_008003325.1	0.82209	4.19E-34	1.51E-32	sp|A5A6N0|K2C7_PANTR Keratin, type II cytoskeletal 7 OS=Pan troglodytes GN=KRT7 PE=2 SV=1//0
XM_008003326.1	0.83713	0.0039791	0.020279	sp|Q14533|KRT81_HUMAN Keratin, type II cuticular Hb1 OS=Homo sapiens GN=KRT81 PE=1 SV=3//0
XM_008003327.1	0.39828	0.0041706	0.021186	sp|Q14533|KRT81_HUMAN Keratin, type II cuticular Hb1 OS=Homo sapiens GN=KRT81 PE=1 SV=3//0
XM_008003353.1	2.9713	1.42E-05	0.000103	sp|Q8N1N4|K2C78_HUMAN Keratin, type II cytoskeletal 78 OS=Homo sapiens GN=KRT78 PE=2 SV=2//0
XM_008003367.1	0.82817	1.56E-20	3.18E-19	sp|Q8NCJ5|SPRY3_HUMAN SPRY domain-containing protein 3 OS=Homo sapiens GN=SPRYD3 PE=1 SV=2//0
XM_008003408.1	-0.91284	1.80E-26	4.84E-25	sp|Q5RAY0|PFD5_PONAB Prefoldin subunit 5 OS=Pongo abelii GN=PFDN5 PE=2 SV=1//8.30559e-89
XM_008003409.1	-0.31006	0.0027383	0.01447	sp|Q9HB07|MYG1_HUMAN UPF0160 protein MYG1, mitochondrial OS=Homo sapiens GN=C12orf10 PE=1 SV=2//1.50113e-33
XM_008003413.1	-4.9457	1.47E-05	0.000106	sp|Q8TDD2|SP7_HUMAN Transcription factor Sp7 OS=Homo sapiens GN=SP7 PE=1 SV=1//0
XM_008003419.1	0.71597	5.51E-11	6.46E-10	sp|Q8N8R7|AL14E_HUMAN ARL14 effector protein OS=Homo sapiens GN=ARL14EP PE=1 SV=1//1.74922e-170
XM_008003451.1	-1.2276	2.93E-05	0.0002041	sp|Q61990|PCBP2_MOUSE Poly(rC)-binding protein 2 OS=Mus musculus GN=Pcbp2 PE=1 SV=1//0
XM_008003456.1	-0.41737	0.0031678	0.01652	sp|Q9NZ81|PRR13_HUMAN Proline-rich protein 13 OS=Homo sapiens GN=PRR13 PE=1 SV=1//6.5594e-06
XM_008003473.1	-0.65082	1.34E-18	2.49E-17	sp|Q06055|AT5G2_HUMAN ATP synthase F(0) complex subunit C2, mitochondrial OS=Homo sapiens GN=ATP5G2 PE=2 SV=1//1.58937e-69
XM_008003491.1	-2.1935	0.0014033	0.0078491	sp|P32043|HXC5_MOUSE Homeobox protein Hox-C5 OS=Mus musculus GN=Hoxc5 PE=2 SV=3//2.30882e-126

XM_008003492.1	-1.7474	0.0028689	0.015113	sp|P10629|HXC6_MOUSE Homeobox protein Hox-C6 OS=Mus musculus GN=Hoxc6 PE=2 SV=2//7.68722e-139
XM_008003495.1	-1.8807	2.49E-07	2.15E-06	sp|P09025|HXC8_MOUSE Homeobox protein Hox-C8 OS=Mus musculus GN=Hoxc8 PE=2 SV=2//4.36668e-155
XM_008003503.1	1.4397	0.010468	0.048789	sp|P09651|ROA1_HUMAN Heterogeneous nuclear ribonucleoprotein A1 OS=Homo sapiens GN=HNRNPA1 PE=1 SV=5//7.80041e-142
XM_008003516.1	-1.4956	1.56E-18	2.88E-17	sp|P08648|ITA5_HUMAN Integrin alpha-5 OS=Homo sapiens GN=ITGA5 PE=1 SV=2//0
XM_008003560.1	-1.44	1.30E-15	2.03E-14	sp|Q6UX53|MET7B_HUMAN Methyltransferase-like protein 7B OS=Homo sapiens GN=METTL7B PE=1 SV=2//6.97508e-154
XM_008003564.1	-1.2498	3.70E-17	6.32E-16	sp|Q5R7L8|BL1S1_PONAB Biogenesis of lysosome-related organelles complex 1 subunit 1 OS=Pongo abelii GN=BLOC1S1 PE=2 SV=2//3.11176e-75
XM_008003573.1	-1.3549	4.93E-32	1.63E-30	sp|O95390|GDF11_HUMAN Growth/differentiation factor 11 OS=Homo sapiens GN=GDF11 PE=2 SV=1//0
XM_008003578.1	-0.3296	0.0006892	0.0040273	sp|Q6Y2X3|DJC14_HUMAN DnaJ homolog subfamily C member 14 OS=Homo sapiens GN=DNAJC14 PE=2 SV=2//0
XM_008003581.1	-1.1863	1.34E-57	9.74E-56	sp|Q5RBG1|RAB5B_PONAB Ras-related protein Rab-5B OS=Pongo abelii GN=RAB5B PE=2 SV=1//6.73182e-136
XM_008003601.1	-1.2546	7.08E-08	6.43E-07	sp|Q9H2S9|IKZF4_HUMAN Zinc finger protein Eos OS=Homo sapiens GN=IKZF4 PE=1 SV=2//3.72414e-09
XM_008003604.1	2.2245	8.81E-95	1.50E-92	sp|Q8NI77|KI18A_HUMAN Kinesin-like protein KIF18A OS=Homo sapiens GN=KIF18A PE=1 SV=2//0
XM_008003606.1	-0.54371	3.45E-07	2.95E-06	sp|Q64119|MYL6_RAT Myosin light polypeptide 6 OS=Rattus norvegicus GN=Myl6 PE=1 SV=3//7.04173e-105
XM_008003613.1	-0.90462	2.77E-06	2.17E-05	sp|Q96K80|ZC3HA_HUMAN Zinc finger CCCH domain-containing protein 10 OS=Homo sapiens GN=ZC3H10 PE=1 SV=1//0
XM_008003624.1	0.41629	0.0001974	0.0012418	sp|Q5R7T5|RNF41_PONAB E3 ubiquitin-protein ligase NRDP1 OS=Pongo abelii GN=RNF41 PE=2 SV=1//0
XM_008003626.1	-0.62781	5.54E-18	9.90E-17	sp|Q8NB46|ANR52_HUMAN Serine/threonine-protein phosphatase 6 regulatory ankyrin repeat subunit C OS=Homo sapiens GN=ANKRD52 PE=1 SV=3//0
XM_008003651.1	-0.37685	5.17E-05	0.0003508	sp|Q9Y2B0|CNPY2_HUMAN Protein canopy homolog 2 OS=Homo sapiens GN=CNPY2 PE=1 SV=1//3.15232e-111
XM_008003661.1	1.6093	8.93E-19	1.67E-17	sp|P52630|STAT2_HUMAN Signal transducer and activator of transcription 2 OS=Homo sapiens GN=STAT2 PE=1 SV=1//0
XM_008003700.1	-0.49262	5.85E-14	8.34E-13	sp|P06576|ATPB_HUMAN ATP synthase subunit beta, mitochondrial OS=Homo sapiens GN=ATP5B PE=1 SV=3//0
XM_008003705.1	-0.67317	7.86E-18	1.40E-16	sp|Q60817|NACA_MOUSE Nascent polypeptide-associated complex subunit alpha OS=Mus musculus GN=Naca PE=1 SV=1//8.25582e-73
XM_008003731.1	0.29131	0.0005527	0.0032857	sp|Q792I0|LIN7C_RAT Protein lin-7 homolog C OS=Rattus norvegicus GN=Lin7c PE=1 SV=1//5.3365e-114
XM_008003739.1	-0.95773	2.29E-19	4.42E-18	sp|Q07954|LRP1_HUMAN Prolow-density lipoprotein receptor-related protein 1 OS=Homo sapiens GN=LRP1 PE=1 SV=2//0
XM_008003740.1	-1.724	3.81E-21	8.03E-20	sp|Q9Z2N4|NXPH4_RAT Neurexophilin-4 OS=Rattus norvegicus GN=Nxph4 PE=2 SV=1//2.9423e-138
XM_008003745.1	-1.5015	4.87E-81	6.39E-79	sp|Q9BXB1|LGR4_HUMAN Leucine-rich repeat-containing G-protein coupled receptor 4 OS=Homo sapiens GN=LGR4 PE=1 SV=2//0
XM_008003776.1	2.5304	1.16E-23	2.76E-22	sp|P58166|INHBE_HUMAN Inhibin beta E chain OS=Homo sapiens GN=INHBE PE=1 SV=1//0
XM_008003781.1	0.48987	1.30E-07	1.15E-06	sp|P56192|SYMC_HUMAN Methionine--tRNA ligase, cytoplasmic OS=Homo sapiens GN=MARS PE=1 SV=2//0
XM_008003833.1	-0.51353	1.07E-05	7.90E-05	sp|Q12999|TSN31_HUMAN Tetraspanin-31 OS=Homo sapiens GN=TSPAN31 PE=2 SV=1//5.43823e-121
XM_008003840.1	0.71452	9.97E-09	9.76E-08	sp|Q86YJ5|MARH9_HUMAN E3 ubiquitin-protein ligase MARCH9 OS=Homo sapiens GN=MARCH9 PE=1 SV=2//6.86594e-151
XM_008003841.1	2.3283	2.57E-19	4.95E-18	sp|O15528|CP27B_HUMAN 25-hydroxyvitamin D-1 alpha hydroxylase, mitochondrial OS=Homo sapiens GN=CYP27B1 PE=1 SV=1//0
XM_008003843.1	0.82693	1.21E-07	1.08E-06	sp|Q96AZ1|MT21B_HUMAN Protein-lysine methyltransferase METTL21B OS=Homo sapiens GN=METTL21B PE=1 SV=1//1.07288e-154
XM_008003848.1	1.2713	1.01E-14	1.51E-13	sp|Q9UBP6|TRMB_HUMAN tRNA (guanine-N(7)-)-methyltransferase OS=Homo sapiens GN=METTL1 PE=1 SV=1//0
XM_008003857.1	-0.31004	7.01E-05	0.0004692	sp|O14595|CTDS2_HUMAN Carboxy-terminal domain RNA polymerase II polypeptide A small phosphatase 2 OS=Homo sapiens GN=CTDSP2 PE=1 SV=2//0
XM_008003858.1	-0.65781	2.40E-10	2.68E-09	sp|Q9Y6H3|ATP23_HUMAN Mitochondrial inner membrane protease ATP23 homolog OS=Homo sapiens GN=XRCC6BP1 PE=1 SV=3//3.73164e-175
XM_008003862.1	0.93716	5.80E-25	1.47E-23	sp|Q6UXM1|LRIG3_HUMAN Leucine-rich repeats and immunoglobulin-like domains protein 3 OS=Homo sapiens GN=LRIG3 PE=2 SV=1//0
XM_008003873.1	1.1696	0.0020855	0.011259	sp|Q8R5H1|UBP15_MOUSE Ubiquitin carboxyl-terminal hydrolase 15 OS=Mus musculus GN=Usp15 PE=1 SV=1//2.95691e-153
XM_008003890.1	1.9631	1.36E-25	3.56E-24	sp|Q96MD2|CL066_HUMAN UPF0536 protein C12orf66 OS=Homo sapiens GN=C12orf66 PE=2 SV=4//0

XM_008003891.1	1.197	7.00E-25	1.77E-23	sp|Q7Z6B7|SRGP1_HUMAN SLIT-ROBO Rho GTPase-activating protein 1 OS=Homo sapiens GN=SRGAP1 PE=1 SV=1//0
XM_008003897.1	1.1307	0.0062009	0.030341	sp|O43592|XPOT_HUMAN Exportin-T OS=Homo sapiens GN=XPOT PE=1 SV=2//0
XM_008003902.1	-1.3662	5.36E-79	6.67E-77	sp|P15586|GNS_HUMAN N-acetylglucosamine-6-sulfatase OS=Homo sapiens GN=GNS PE=1 SV=3//0
XM_008003910.1	1.0439	7.31E-29	2.16E-27	sp|Q9Y2U8|MAN1_HUMAN Inner nuclear membrane protein Man1 OS=Homo sapiens GN=LEMD3 PE=1 SV=2//0
XM_008003919.1	-1.594	0.0006671	0.0039037	sp|P52926|HMGA2_HUMAN High mobility group protein HMGI-C OS=Homo sapiens GN=HMGA2 PE=1 SV=1//2.86786e-08
XM_008003920.1	-1.3918	4.95E-42	2.29E-40	sp|P52926|HMGA2_HUMAN High mobility group protein HMGI-C OS=Homo sapiens GN=HMGA2 PE=1 SV=1//2.56067e-13
XM_008003924.1	-4.5056	0.00039	0.0023626	sp|P52926|HMGA2_HUMAN High mobility group protein HMGI-C OS=Homo sapiens GN=HMGA2 PE=1 SV=1//3.18691e-08
XM_008003925.1	-0.2611	0.00763	0.036611	sp|Q9BRT6|LLPH_HUMAN Protein LLP homolog OS=Homo sapiens GN=LLPH PE=2 SV=1//1.53241e-51
XM_008003939.1	0.65203	2.98E-21	6.31E-20	sp|Q86VP6|CAND1_HUMAN Cullin-associated NEDD8-dissociated protein 1 OS=Homo sapiens GN=CAND1 PE=1 SV=2//0
XM_008003965.1	0.58523	2.30E-12	2.98E-11	sp|P57740|NU107_HUMAN Nuclear pore complex protein Nup107 OS=Homo sapiens GN=NUP107 PE=1 SV=1//0
XM_008003981.1	-1.492	1.97E-08	1.88E-07	sp|P61634|LYSC_ERYPA Lysozyme C OS=Erythrocebus patas GN=LYZ PE=3 SV=1//2.6132e-102
XM_008003982.1	0.51472	0.0001114	0.0007261	sp|O95619|YETS4_HUMAN YEATS domain-containing protein 4 OS=Homo sapiens GN=YEATS4 PE=1 SV=1//1.83609e-142
XM_008004010.1	-0.9239	7.62E-10	8.15E-09	sp|Q86W47|KCMB4_HUMAN Calcium-activated potassium channel subunit beta-4 OS=Homo sapiens GN=KCNMB4 PE=1 SV=2//7.00667e-136
XM_008004033.1	-1.8111	5.87E-93	9.46E-91	sp|O60293|ZC3H1_HUMAN Zinc finger C3H1 domain-containing protein OS=Homo sapiens GN=ZFC3H1 PE=1 SV=3//0
XM_008004034.1	1.1876	1.23E-44	6.28E-43	sp|Q9UL25|RAB21_HUMAN Ras-related protein Rab-21 OS=Homo sapiens GN=RAB21 PE=1 SV=3//6.49102e-145
XM_008004042.1	-0.62437	0.0075914	0.036442	sp|Q9UKU6|TRHDE_HUMAN Thyrotropin-releasing hormone-degrading ectoenzyme OS=Homo sapiens GN=TRHDE PE=2 SV=1//0
XM_008004043.1	-0.22833	0.0026181	0.013878	sp|Q96GX2|A7L3B_HUMAN Putative ataxin-7-like protein 3B OS=Homo sapiens GN=ATXN7L3B PE=3 SV=2//9.27645e-58
XM_008004057.1	0.93818	8.21E-25	2.06E-23	sp|Q13601|KRR1_HUMAN KRR1 small subunit processome component homolog OS=Homo sapiens GN=KRR1 PE=1 SV=4//0
XM_008004058.1	0.89508	7.70E-05	0.0005126	sp|P48060|GLIP1_HUMAN Glioma pathogenesis-related protein 1 OS=Homo sapiens GN=GLIPR1 PE=1 SV=3//0
XM_008004060.1	0.60599	1.34E-16	2.23E-15	sp|Q8WV24|PHLA1_HUMAN Pleckstrin homology-like domain family A member 1 OS=Homo sapiens GN=PHLDA1 PE=1 SV=4//2.35503e-142
XM_008004073.1	0.71084	1.19E-05	8.69E-05	sp|Q5R8P3|BBS10_PONAB Bardet-Biedl syndrome 10 protein homolog OS=Pongo abelii GN=BBS10 PE=2 SV=1//0
XM_008004113.1	1.4838	6.81E-68	6.64E-66	sp|Q96IZ0|PAWR_HUMAN PRKC apoptosis WT1 regulator protein OS=Homo sapiens GN=PAWR PE=1 SV=1//2.62974e-158
XM_008004119.1	4.3718	1.33E-16	2.21E-15	sp|Q3ZCN5|OTOGL_HUMAN Otogelin-like protein OS=Homo sapiens GN=OTOGL PE=2 SV=5//0
XM_008004120.1	4.4167	0.0006577	0.0038578	sp|Q9UMZ3|PTPRQ_HUMAN Phosphatidylinositol phosphatase PTPRQ OS=Homo sapiens GN=PTPRQ PE=1 SV=2//1.98721e-14
XM_008004125.1	-0.73899	0.0029107	0.015311	sp|Q5REB8|ACSS3_PONAB Acyl-CoA synthetase short-chain family member 3, mitochondrial OS=Pongo abelii GN=ACSS3 PE=2 SV=1//0
XM_008004150.1	-0.45041	1.70E-07	1.49E-06	sp|Q5RFN6|CNN2_PONAB Calponin-2 OS=Pongo abelii GN=CNN2 PE=2 SV=3//0
XM_008004155.1	-0.78977	2.11E-31	6.82E-30	sp|P05783|K1C18_HUMAN Keratin, type I cytoskeletal 18 OS=Homo sapiens GN=KRT18 PE=1 SV=2//0
XM_008004157.1	0.71817	0.0004446	0.0026716	sp|Q5R676|TOM5_PONAB Mitochondrial import receptor subunit TOM5 homolog OS=Pongo abelii GN=TOMM5 PE=3 SV=1//4.72598e-26
XM_008004158.1	1.1625	7.40E-16	1.18E-14	sp|Q9H2J7|S6A15_HUMAN Sodium-dependent neutral amino acid transporter B(0)AT2 OS=Homo sapiens GN=SLC6A15 PE=1 SV=1//0
XM_008004181.1	-1.3705	0.0029601	0.015541	sp|Q75V66|ANO5_HUMAN Anoctamin-5 OS=Homo sapiens GN=ANO5 PE=1 SV=1//0
XM_008004213.1	-0.48137	2.02E-06	1.61E-05	sp|Q16828|DUS6_HUMAN Dual specificity protein phosphatase 6 OS=Homo sapiens GN=DUSP6 PE=1 SV=2//0
XM_008004214.1	-0.88889	3.71E-08	3.44E-07	sp|Q8N4A0|GALT4_HUMAN Polypeptide N-acetylgalactosaminyltransferase 4 OS=Homo sapiens GN=GALNT4 PE=1 SV=2//0
XM_008004234.1	-0.3017	0.0001326	0.0008557	sp|P62325|BTG1_MOUSE Protein BTG1 OS=Mus musculus GN=Btg1 PE=2 SV=1//1.069e-117
XM_008004236.1	2.0221	2.45E-103	4.83E-101	sp|Q15075|EEA1_HUMAN Early endosome antigen 1 OS=Homo sapiens GN=EEA1 PE=1 SV=2//0
XM_008004246.1	0.33798	7.76E-05	0.0005163	sp|Q5R7J6|UBE2N_PONAB Ubiquitin-conjugating enzyme E2 N OS=Pongo abelii GN=UBE2N PE=2 SV=1//1.27342e-102
XM_008004247.1	0.29429	0.010092	0.047245	sp|Q9Y6G3|RM42_HUMAN 39S ribosomal protein L42, mitochondrial OS=Homo sapiens GN=MRPL42 PE=1 SV=1//4.39742e-82
XM_008004276.1	-1.3397	2.99E-23	7.02E-22	sp|Q0MQ87|NDUAC_PANTR NADH dehydrogenase [ubiquinone] 1 alpha

				subcomplex subunit 12 OS=Pan troglodytes GN=NDUFA12 PE=2 SV=1//6.07074e-100
XM_008004290.1	0.37578	1.32E-07	1.17E-06	sp|P50579|MAP2_HUMAN Methionine aminopeptidase 2 OS=Homo sapiens GN=METAP2 PE=1 SV=1//0
XM_008004305.1	-0.41192	2.17E-05	0.0001539	sp|P62321|RUXF_XENLA Small nuclear ribonucleoprotein F OS=Xenopus laevis GN=snrpf PE=3 SV=1//1.32077e-48
XM_008004312.1	0.28263	0.0001296	0.0008372	sp|P41970|ELK3_HUMAN ETS domain-containing protein Elk-3 OS=Homo sapiens GN=ELK3 PE=1 SV=2//0
XM_008004314.1	0.9686	0.0050571	0.025227	sp|Q00537|CDK17_HUMAN Cyclin-dependent kinase 17 OS=Homo sapiens GN=CDK17 PE=1 SV=2//0
XM_008004336.1	0.61048	6.24E-09	6.19E-08	sp|P42166|LAP2A_HUMAN Lamina-associated polypeptide 2, isoform alpha OS=Homo sapiens GN=TMPO PE=1 SV=2//0
XM_008004337.1	-2.7433	0.0086238	0.040985	sp|P42167|LAP2B_HUMAN Lamina-associated polypeptide 2, isoforms beta/gamma OS=Homo sapiens GN=TMPO PE=1 SV=2//0
XM_008004346.1	-0.63242	9.33E-07	7.68E-06	sp|Q5EAJ6|IKIP_RAT Inhibitor of nuclear factor kappa-B kinase-interacting protein OS=Rattus norvegicus GN=Ikbip PE=2 SV=1//5.24421e-170
XM_008004398.1	0.58804	2.36E-08	2.25E-07	sp|P40616|ARL1_HUMAN ADP-ribosylation factor-like protein 1 OS=Homo sapiens GN=ARL1 PE=1 SV=1//1.48552e-117
XM_008004451.1	0.82601	4.84E-09	4.84E-08	sp|Q8WWQ8|STAB2_HUMAN Stabilin-2 OS=Homo sapiens GN=STAB2 PE=1 SV=3//0
XM_008004453.1	0.84769	1.06E-09	1.12E-08	sp|Q4R520|ENPL_MACFA Endoplasmin OS=Macaca fascicularis GN=HSP90B1 PE=2 SV=1//0
XM_008004493.1	1.007	9.31E-07	7.66E-06	sp|Q4R335|S41A2_MACFA Solute carrier family 41 member 2 OS=Macaca fascicularis GN=SLC41A2 PE=2 SV=1//0
XM_008004513.1	-1.1007	7.82E-06	5.84E-05	sp|Q8TAD7|OCC1_HUMAN Overexpressed in colon carcinoma 1 protein OS=Homo sapiens GN=OCC1 PE=1 SV=2//3.43488e-19
XM_008004516.1	0.59855	3.56E-06	2.76E-05	sp|O60285|NUAK1_HUMAN NUAK family SNF1-like kinase 1 OS=Homo sapiens GN=NUAK1 PE=1 SV=1//0
XM_008004518.1	-0.87382	9.90E-21	2.03E-19	sp|Q07065|CKAP4_HUMAN Cytoskeleton-associated protein 4 OS=Homo sapiens GN=CKAP4 PE=1 SV=2//3.11472e-11
XM_008004532.1	1.3971	3.83E-44	1.89E-42	sp|Q9NW08|RPC2_HUMAN DNA-directed RNA polymerase III subunit RPC2 OS=Homo sapiens GN=POLR3B PE=1 SV=2//0
XM_008004538.1	1.3049	3.93E-08	3.64E-07	sp|Q9NVN3|RIC8B_HUMAN Synembryn-B OS=Homo sapiens GN=RIC8B PE=1 SV=2//0
XM_008004554.1	2.0491	7.79E-25	1.96E-23	sp|Q16526|CRY1_HUMAN Cryptochrome-1 OS=Homo sapiens GN=CRY1 PE=1 SV=1//0
XM_008004564.1	1.2756	0.0041374	0.021031	sp|Q9UKN5|PRDM4_HUMAN PR domain zinc finger protein 4 OS=Homo sapiens GN=PRDM4 PE=1 SV=3//0
XM_008004577.1	2.52	1.90E-20	3.84E-19	sp|Q9BVA6|FICD_HUMAN Adenosine monophosphate-protein transferase FICD OS=Homo sapiens GN=FICD PE=1 SV=2//0
XM_008004593.1	0.51059	1.58E-06	1.28E-05	sp|Q8WYL5|SSH1_HUMAN Protein phosphatase Slingshot homolog 1 OS=Homo sapiens GN=SSH1 PE=1 SV=2//0
XM_008004628.1	0.95031	3.68E-09	3.72E-08	sp|Q9H3F6|BACD3_HUMAN BTB/POZ domain-containing adapter for CUL3-mediated RhoA degradation protein 3 OS=Homo sapiens GN=KCTD10 PE=1 SV=1//0
XM_008004652.1	1.3519	5.18E-40	2.26E-38	sp|Q8N2M4|TM86A_HUMAN Lysoplasmalogenase-like protein TMEM86A OS=Homo sapiens GN=TMEM86A PE=2 SV=1//3.87182e-125
XM_008004667.1	-1.21	1.54E-05	0.0001107	sp|Q8N9C0|IGS22_HUMAN Immunoglobulin superfamily member 22 OS=Homo sapiens GN=IGSF22 PE=2 SV=2//2.8296e-09
XM_008004679.1	1.5703	1.18E-66	1.12E-64	sp|Q68D10|SPT2_HUMAN Protein SPT2 homolog OS=Homo sapiens GN=SPTY2D1 PE=1 SV=3//0
XM_008004681.1	1.0422	1.75E-18	3.23E-17	sp|P16615|AT2A2_HUMAN Sarcoplasmic/endoplasmic reticulum calcium ATPase 2 OS=Homo sapiens GN=ATP2A2 PE=1 SV=1//0
XM_008004684.1	0.48641	0.0002001	0.0012579	sp|Q8WUB2|F216A_HUMAN Protein FAM216A OS=Homo sapiens GN=FAM216A PE=2 SV=1//6.16534e-167
XM_008004701.1	1.181	2.43E-27	6.80E-26	sp|Q8NI37|PPTC7_HUMAN Protein phosphatase PTC7 homolog OS=Homo sapiens GN=PPTC7 PE=2 SV=1//0
XM_008004714.1	0.40119	6.67E-05	0.0004473	sp|Q99816|TS101_HUMAN Tumor susceptibility gene 101 protein OS=Homo sapiens GN=TSG101 PE=1 SV=2//0
XM_008004794.1	Inf	5.22E-09	5.20E-08	sp|P00973|OAS1_HUMAN 2'-5'-oligoadenylate synthase 1 OS=Homo sapiens GN=OAS1 PE=1 SV=4//0
XM_008004795.1	3.1465	6.18E-11	7.21E-10	sp|Q9Y6K5|OAS3_HUMAN 2'-5'-oligoadenylate synthase 3 OS=Homo sapiens GN=OAS3 PE=1 SV=3//4.41024e-86
XM_008004796.1	Inf	1.09E-58	8.17E-57	sp|P29728|OAS2_HUMAN 2'-5'-oligoadenylate synthase 2 OS=Homo sapiens GN=OAS2 PE=1 SV=3//0
XM_008004824.1	-0.58984	0.004572	0.023027	sp|Q6J4K2|NCKX6_HUMAN Sodium/potassium/calcium exchanger 6, mitochondrial OS=Homo sapiens GN=SLC8B1 PE=1 SV=2//0
XM_008004832.1	0.70483	3.08E-11	3.68E-10	sp|Q9Y4C8|RBM19_HUMAN Probable RNA-binding protein 19 OS=Homo sapiens GN=RBM19 PE=1 SV=3//0
XM_008004836.1	-1.5369	3.76E-18	6.79E-17	sp|Q9BE24|LDHA_MACFA L-lactate dehydrogenase A chain OS=Macaca fascicularis GN=LDHA PE=2 SV=4//1.26239e-154

XM_008004845.1	-1.4549	0.0005539	0.0032904	sp|Q96EX2|RNFT2_HUMAN RING finger and transmembrane domain-containing protein 2 OS=Homo sapiens GN=RNFT2 PE=2 SV=2//0
XM_008004872.1	0.60605	1.30E-08	1.26E-07	sp|P40937|RFC5_HUMAN Replication factor C subunit 5 OS=Homo sapiens GN=RFC5 PE=1 SV=1//0
XM_008004894.1	2.6703	8.87E-206	4.74E-203	sp|Q5RAB0|HSPB8_PONAB Heat shock protein beta-8 OS=Pongo abelii GN=HSPB8 PE=2 SV=1//2.78996e-121
XM_008004904.1	1.2899	2.19E-17	3.81E-16	sp|Q9Y478|AAKB1_HUMAN 5'-AMP-activated protein kinase subunit beta-1 OS=Homo sapiens GN=PRKAB1 PE=1 SV=4//0
XM_008004912.1	2.7724	3.64E-11	4.34E-10	sp|P32780|TF2H1_HUMAN General transcription factor IIH subunit 1 OS=Homo sapiens GN=GTF2H1 PE=1 SV=1//0
XM_008004921.1	0.37137	2.25E-07	1.95E-06	sp|Q92616|GCN1L_HUMAN Translational activator GCN1 OS=Homo sapiens GN=GCN1L1 PE=1 SV=6//0
XM_008004922.1	-0.68458	7.00E-27	1.91E-25	sp|P05388|RLA0_HUMAN 60S acidic ribosomal protein P0 OS=Homo sapiens GN=RPLP0 PE=1 SV=1//0
XM_008004940.1	-0.78363	4.77E-18	8.57E-17	sp|P12074|CX6A1_HUMAN Cytochrome c oxidase subunit 6A1, mitochondrial OS=Homo sapiens GN=COX6A1 PE=1 SV=4//1.39762e-68
XM_008004941.1	0.5529	9.27E-07	7.64E-06	sp|O43716|GATC_HUMAN Glutamyl-tRNA(Gln) amidotransferase subunit C, mitochondrial OS=Homo sapiens GN=GATC PE=1 SV=1//1.25203e-90
XM_008004943.1	-0.46704	2.78E-07	2.39E-06	sp|Q13242|SRSF9_HUMAN Serine/arginine-rich splicing factor 9 OS=Homo sapiens GN=SRSF9 PE=1 SV=1//4.59869e-117
XM_008004965.1	-0.41269	1.23E-09	1.29E-08	sp|Q14165|MLEC_HUMAN Malectin OS=Homo sapiens GN=MLEC PE=1 SV=1//2.78721e-156
XM_008004966.1	-0.72591	1.67E-16	2.76E-15	sp|A6NIH7|U119B_HUMAN Protein unc-119 homolog B OS=Homo sapiens GN=UNC119B PE=1 SV=1//2.76486e-134
XM_008004970.1	-0.31895	0.0028587	0.015066	sp|Q9CUS9|SPPL3_MOUSE Signal peptide peptidase-like 3 OS=Mus musculus GN=Sppl3 PE=1 SV=3//0
XM_008004971.1	0.79073	1.22E-05	8.92E-05	sp|Q96C57|CL043_HUMAN Uncharacterized protein C12orf43 OS=Homo sapiens GN=C12orf43 PE=1 SV=2//3.04839e-87
XM_008004973.1	Inf	1.93E-18	3.54E-17	sp|Q15646|OASL_HUMAN 2'-5'-oligoadenylate synthase-like protein OS=Homo sapiens GN=OASL PE=1 SV=2//0
XM_008005009.1	-0.45378	2.20E-05	0.0001555	sp|Q9UPS6|SET1B_HUMAN Histone-lysine N-methyltransferase SETD1B OS=Homo sapiens GN=SETD1B PE=1 SV=3//0
XM_008005018.1	0.57262	5.61E-10	6.07E-09	sp|Q9HAP2|MLXIP_HUMAN MLX-interacting protein OS=Homo sapiens GN=MLXIP PE=1 SV=2//0
XM_008005027.1	-1.8812	1.83E-05	0.0001309	sp|Q9C0J1|B3GN4_HUMAN N-acetyllactosaminide beta-1,3-N-acetylglucosaminyltransferase 4 OS=Homo sapiens GN=B3GNT4 PE=1 SV=1//0
XM_008005035.1	3.4196	2.27E-09	2.34E-08	sp|P30622|CLIP1_HUMAN CAP-Gly domain-containing linker protein 1 OS=Homo sapiens GN=CLIP1 PE=1 SV=2//5.49756e-161
XM_008005063.1	0.50467	5.07E-07	4.26E-06	sp|O43583|DENR_HUMAN Density-regulated protein OS=Homo sapiens GN=DENR PE=1 SV=2//1.5268e-106
XM_008005080.1	-0.84028	6.66E-18	1.19E-16	sp|Q66PJ3|AR6P4_HUMAN ADP-ribosylation factor-like protein 6-interacting protein 4 OS=Homo sapiens GN=ARL6IP4 PE=1 SV=2//1.99372e-62
XM_008005115.1	0.80187	8.63E-15	1.29E-13	sp|Q9NQR1|SETD8_HUMAN N-lysine methyltransferase SETD8 OS=Homo sapiens GN=SETD8 PE=1 SV=3//0
XM_008005125.1	0.66218	1.57E-07	1.38E-06	sp|Q5EBL4|RIPL1_HUMAN RILP-like protein 1 OS=Homo sapiens GN=RILPL1 PE=1 SV=1//0
XM_008005137.1	0.48263	0.0018967	0.010311	sp|Q9Y487|VPP2_HUMAN V-type proton ATPase 116 kDa subunit a isoform 2 OS=Homo sapiens GN=ATP6V0A2 PE=1 SV=2//0
XM_008005182.1	0.64908	4.46E-14	6.39E-13	sp|P0CG60|UBB_PONPY Polyubiquitin-B OS=Pongo pygmaeus GN=UBB PE=3 SV=1//1.63657e-160
XM_008005187.1	-0.92912	1.45E-08	1.41E-07	sp|Q8WY22|BRI3B_HUMAN BRI3-binding protein OS=Homo sapiens GN=BRI3BP PE=1 SV=1//1.12662e-105
XM_008005240.1	1.3091	0.0001949	0.0012268	sp|Q12872|SFSWA_HUMAN Splicing factor, suppressor of white-apricot homolog OS=Homo sapiens GN=SFSWAP PE=1 SV=3//1.46168e-17
XM_008005277.1	0.64755	1.37E-06	1.11E-05	sp|Q9BVI4|NOC4L_HUMAN Nucleolar complex protein 4 homolog OS=Homo sapiens GN=NOC4L PE=1 SV=1//0
XM_008005285.1	1.3229	0.0036127	0.018597	sp|Q9GM01|GALT9_MACFA Polypeptide N-acetylgalactosaminyltransferase 9 OS=Macaca fascicularis GN=GALNT9 PE=2 SV=1//7.98537e-174
XM_008005296.1	-1.0671	0.0004244	0.0025604	-//-
XM_008005297.1	-0.40526	6.46E-05	0.0004336	sp|Q9HCM7|FBSL_HUMAN Fibrosin-1-like protein OS=Homo sapiens GN=FBRSL1 PE=1 SV=4//0
XM_008005315.1	1.1434	1.45E-13	2.02E-12	sp|Q86XL3|ANKL2_HUMAN Ankyrin repeat and LEM domain-containing protein 2 OS=Homo sapiens GN=ANKLE2 PE=1 SV=4//0
XM_008005349.1	1.4654	2.53E-07	2.17E-06	sp|P21506|ZNF10_HUMAN Zinc finger protein 10 OS=Homo sapiens GN=ZNF10 PE=1 SV=3//0
XM_008005372.1	1.6879	8.63E-05	0.0005704	-//-
XM_008005376.1	-0.88109	2.55E-40	1.12E-38	sp|P05783|K1C18_HUMAN Keratin, type I cytoskeletal 18 OS=Homo sapiens GN=KRT18 PE=1 SV=2//0
XM_008005377.1	-0.31908	0.0007746	0.0044967	sp|Q9NRG9|AAAS_HUMAN Aladin OS=Homo sapiens GN=AAAS PE=1 SV=1//5.23371e-53

XM_008005379.1	0.82572	3.96E-18	7.13E-17	sp|Q9UQ80|PA2G4_HUMAN Proliferation-associated protein 2G4 OS=Homo sapiens GN=PA2G4 PE=1 SV=3//4.69839e-22
XM_008005381.1	0.66638	1.65E-13	2.29E-12	sp|P56192|SYMC_HUMAN Methionine--tRNA ligase, cytoplasmic OS=Homo sapiens GN=MARS PE=1 SV=2//0
XM_008005395.1	0.65451	7.98E-13	1.06E-11	sp|Q9UJX3|APC7_HUMAN Anaphase-promoting complex subunit 7 OS=Homo sapiens GN=ANAPC7 PE=1 SV=4//0
XM_008005396.1	-1.0137	2.92E-08	2.75E-07	sp|O15145|ARPC3_HUMAN Actin-related protein 2/3 complex subunit 3 OS=Homo sapiens GN=ARPC3 PE=1 SV=3//1.54856e-85
XM_008005398.1	1.0737	1.57E-25	4.10E-24	sp|Q7Z569|BRAP_HUMAN BRCA1-associated protein OS=Homo sapiens GN=BRAP PE=1 SV=2//0
XM_008005399.1	-1.1523	1.22E-29	3.70E-28	sp|Q02878|RL6_HUMAN 60S ribosomal protein L6 OS=Homo sapiens GN=RPL6 PE=1 SV=3//1.01186e-22
XM_008005402.1	-0.42803	0.0008216	0.0047503	sp|Q96RR4|KKCC2_HUMAN Calcium/calmodulin-dependent protein kinase kinase 2 OS=Homo sapiens GN=CAMKK2 PE=1 SV=2//0
XM_008005404.1	1.5538	1.61E-48	9.45E-47	sp|Q6NZY4|ZCHC8_HUMAN Zinc finger CCHC domain-containing protein 8 OS=Homo sapiens GN=ZCCHC8 PE=1 SV=2//0
XM_008005405.1	0.84014	4.30E-17	7.33E-16	sp|Q689Z5|SBNO1_MOUSE Protein strawberry notch homolog 1 OS=Mus musculus GN=Sbno1 PE=1 SV=2//0
XM_008005435.1	1.7173	9.34E-10	9.94E-09	sp|Q8N5I2|ARRD1_HUMAN Arrestin domain-containing protein 1 OS=Homo sapiens GN=ARRDC1 PE=1 SV=1//0
XM_008005437.1	1.3189	8.81E-15	1.32E-13	sp|Q9BTV6|DPH7_HUMAN Diphthine methyltransferase OS=Homo sapiens GN=DPH7 PE=1 SV=2//0
XM_008005440.1	-1.0685	1.79E-13	2.48E-12	sp|Q8IXM3|RM41_HUMAN 39S ribosomal protein L41, mitochondrial OS=Homo sapiens GN=MRPL41 PE=1 SV=1//3.14797e-78
XM_008005475.1	0.37466	0.0038959	0.019897	sp|Q5RFR2|NR3L1_PONAB Natural cytotoxicity triggering receptor 3 ligand 1 OS=Pongo abelii GN=NCR3LG1 PE=2 SV=1//0
XM_008005482.1	1.6047	6.10E-09	6.06E-08	sp|Q91ZA8|NRARP_MOUSE Notch-regulated ankyrin repeat-containing protein OS=Mus musculus GN=Nrarp PE=2 SV=1//7.87837e-72
XM_008005483.1	-0.44796	2.29E-05	0.0001616	sp|Q8WX92|NELFB_HUMAN Negative elongation factor B OS=Homo sapiens GN=NELFB PE=1 SV=1//0
XM_008005484.1	-0.763	0.0042353	0.021489	sp|Q9NXH8|TOR4A_HUMAN Torsin-4A OS=Homo sapiens GN=TOR4A PE=1 SV=2//0
XM_008005489.1	-1.2312	4.44E-36	1.69E-34	sp|P11833|TBB_PARLI Tubulin beta chain OS=Paracentrotus lividus PE=2 SV=1//0
XM_008005501.1	-0.5169	0.0026115	0.013847	sp|Q4KMQ1|TPRN_HUMAN Taperin OS=Homo sapiens GN=TPRN PE=1 SV=2//8.47926e-38
XM_008005502.1	-0.3969	0.0027034	0.014296	sp|Q969S6|TM203_HUMAN Transmembrane protein 203 OS=Homo sapiens GN=TMEM203 PE=2 SV=1//1.06745e-69
XM_008005520.1	-0.87968	2.10E-08	2.00E-07	sp|O43805|SSNA1_HUMAN Sjoegren syndrome nuclear autoantigen 1 OS=Homo sapiens GN=SSNA1 PE=1 SV=2//1.64927e-75
XM_008005523.1	-1.5356	0.0066006	0.032093	sp|Q9UHL4|DPP2_HUMAN Dipeptidyl peptidase 2 OS=Homo sapiens GN=DPP7 PE=1 SV=3//0
XM_008005525.1	-0.75402	2.06E-16	3.39E-15	sp|Q9UKM7|MA1B1_HUMAN Endoplasmic reticulum mannosyl-oligosaccharide 1,2-alpha-mannosidase OS=Homo sapiens GN=MAN1B1 PE=1 SV=2//0
XM_008005547.1	0.68434	0.0006664	0.0039009	sp|Q9BUH6|PAXX_HUMAN Protein PAXX OS=Homo sapiens GN=C9orf142 PE=1 SV=2//5.16131e-90
XM_008005549.1	-2.6487	1.44E-12	1.89E-11	sp|Q8WNM0|PTGDS_PONPY Prostaglandin-H2 D-isomerase OS=Pongo pygmaeus GN=PTGDS PE=2 SV=1//1.26616e-125
XM_008005550.1	-0.59083	2.12E-08	2.02E-07	sp|Q969U6|FBXW5_HUMAN F-box/WD repeat-containing protein 5 OS=Homo sapiens GN=FBXW5 PE=1 SV=1//0
XM_008005561.1	-0.67181	3.84E-19	7.37E-18	sp|P62278|RS13_RAT 40S ribosomal protein S13 OS=Rattus norvegicus GN=Rps13 PE=1 SV=2//7.03803e-105
XM_008005567.1	-1.0373	2.66E-15	4.06E-14	sp|O60869|EDF1_HUMAN Endothelial differentiation-related factor 1 OS=Homo sapiens GN=EDF1 PE=1 SV=1//2.17049e-99
XM_008005573.1	-1.174	7.71E-12	9.62E-11	sp|Q5T5S1|CC183_HUMAN Coiled-coil domain-containing protein 183 OS=Homo sapiens GN=CCDC183 PE=2 SV=3//0
XM_008005574.1	1.2741	1.17E-07	1.04E-06	sp|Q7TQG1|PKHA6_MOUSE Pleckstrin homology domain-containing family A member 6 OS=Mus musculus GN=Plekha6 PE=1 SV=1//9.0752e-11
XM_008005592.1	-1.672	0.0056041	0.027678	sp|Q5VUD6|FA69B_HUMAN Protein FAM69B OS=Homo sapiens GN=FAM69B PE=1 SV=3//0
XM_008005605.1	-1.3466	9.41E-34	3.33E-32	sp|O15120|PLCB_HUMAN 1-acyl-sn-glycerol-3-phosphate acyltransferase beta OS=Homo sapiens GN=AGPAT2 PE=1 SV=1//5.92389e-168
XM_008005658.1	-0.46797	0.0041414	0.021043	sp|H0YL14|CI069_HUMAN Protein C9orf69 OS=Homo sapiens GN=C9orf69 PE=1 SV=2//2.89574e-51
XM_008005662.1	-0.68247	1.71E-12	2.24E-11	sp|Q9BSL1|UBAC1_HUMAN Ubiquitin-associated domain-containing protein 1 OS=Homo sapiens GN=UBAC1 PE=1 SV=1//0
XM_008005742.1	-1.9671	0.0004429	0.0026629	sp|Q15059|BRD3_HUMAN Bromodomain-containing protein 3 OS=Homo sapiens GN=BRD3 PE=1 SV=1//0
XM_008005785.1	1.3052	1.33E-14	1.97E-13	sp|Q15527|SURF2_HUMAN Surfeit locus protein 2 OS=Homo sapiens GN=SURF2 PE=1 SV=3//1.55053e-153
XM_008005786.1	0.25292	0.000613	0.0036133	sp|Q5R705|SURF4_PONAB Surfeit locus protein 4 OS=Pongo abelii GN=SURF4 PE=2 SV=1//1.8841e-162

XM_008005800.1	-4.0058	0.0062827	0.030698	sp|P16442|BGAT_HUMAN Histo-blood group ABO system transferase OS=Homo sapiens GN=ABO PE=1 SV=2//1.60134e-161
XM_008005801.1	#NAME?	0.0030973	0.016199	sp|P16442|BGAT_HUMAN Histo-blood group ABO system transferase OS=Homo sapiens GN=ABO PE=1 SV=2//4.58859e-162
XM_008005861.1	1.8286	2.22E-54	1.51E-52	sp|Q9H8H2|DDX31_HUMAN Probable ATP-dependent RNA helicase DDX31 OS=Homo sapiens GN=DDX31 PE=2 SV=2//0
XM_008005862.1	1.3774	4.64E-49	2.78E-47	sp|Q9UKN8|TF3C4_HUMAN General transcription factor 3C polypeptide 4 OS=Homo sapiens GN=GTF3C4 PE=1 SV=2//0
XM_008005863.1	1.6786	2.86E-44	1.42E-42	sp|Q15361|TTF1_HUMAN Transcription termination factor 1 OS=Homo sapiens GN=TTF1 PE=1 SV=3//0
XM_008005877.1	-4.0413	0.0033013	0.017168	sp|Q96CW9|NTNG2_HUMAN Netrin-G2 OS=Homo sapiens GN=NTNG2 PE=1 SV=2//0
XM_008005905.1	-3.081	5.47E-08	5.01E-07	sp|Q5JUQ0|FA78A_HUMAN Protein FAM78A OS=Homo sapiens GN=FAM78A PE=2 SV=1//0
XM_008005915.1	0.84314	0.0001378	0.0008867	sp|P83859|OX26_HUMAN Orexigenic neuropeptide QRFP OS=Homo sapiens GN=QRFP PE=2 SV=1//2.04451e-49
XM_008005919.1	1.3218	8.78E-34	3.11E-32	sp|Q13868|EXOS2_HUMAN Exosome complex component RRP4 OS=Homo sapiens GN=EXOSC2 PE=1 SV=2//0
XM_008005939.1	0.5809	0.0030284	0.015865	sp|Q96RU3|FNBP1_HUMAN Formin-binding protein 1 OS=Homo sapiens GN=FNBP1 PE=1 SV=2//0
XM_008005955.1	-0.28214	0.0073085	0.035184	sp|Q9NZ63|CI078_HUMAN Uncharacterized protein C9orf78 OS=Homo sapiens GN=C9orf78 PE=1 SV=1//0
XM_008005959.1	-0.61952	4.88E-09	4.88E-08	sp|Q60HG2|TOR1A_MACFA Torsin-1A OS=Macaca fascicularis GN=TOR1A PE=2 SV=1//0
XM_008005960.1	0.4853	0.000912	0.0052375	sp|O14657|TOR1B_HUMAN Torsin-1B OS=Homo sapiens GN=TOR1B PE=1 SV=2//0
XM_008005961.1	-1.3128	0.0040774	0.020741	sp|Q6PWL6|PTGES_MACFA Prostaglandin E synthase OS=Macaca fascicularis GN=PTGES PE=2 SV=1//4.37771e-102
XM_008005968.1	1.8174	7.22E-10	7.72E-09	sp|Q5R4M7|ASB6_PONAB Ankyrin repeat and SOCS box protein 6 OS=Pongo abelii GN=ASB6 PE=2 SV=1//0
XM_008005976.1	-3.9865	0.004549	0.022927	sp|Q8NAJ2|CI106_HUMAN Putative uncharacterized protein C9orf106 OS=Homo sapiens GN=C9orf106 PE=2 SV=1//4.32432e-59
XM_008005998.1	-0.46807	0.0028427	0.014986	sp|Q58CR4|DOLK_BOVIN Dolichol kinase OS=Bos taurus GN=DOLK PE=2 SV=1//0
XM_008006018.1	-1.1153	6.48E-07	5.40E-06	sp|Q14249|NUCG_HUMAN Endonuclease G, mitochondrial OS=Homo sapiens GN=ENDOG PE=1 SV=4//2.68741e-169
XM_008006033.1	-1.4023	1.02E-14	1.52E-13	sp|Q96GR4|ZDH12_HUMAN Probable palmitoyltransferase ZDHHC12 OS=Homo sapiens GN=ZDHHC12 PE=2 SV=2//3.20171e-96
XM_008006042.1	0.40109	0.0001353	0.0008715	sp|Q5RAS2|GLE1_PONAB Nucleoporin GLE1 OS=Pongo abelii GN=GLE1 PE=2 SV=1//0
XM_008006069.1	-0.44883	0.0008624	0.004972	sp|Q1ZZU3|SWI5_HUMAN DNA repair protein SWI5 homolog OS=Homo sapiens GN=SWI5 PE=1 SV=1//6.4033e-131
XM_008006092.1	-1.0567	4.04E-07	3.43E-06	sp|Q9BUW7|CI016_HUMAN UPF0184 protein C9orf16 OS=Homo sapiens GN=C9orf16 PE=1 SV=1//1.66668e-32
XM_008006104.1	Inf	6.79E-05	0.0004546	sp|Q6KCM7|SCMC2_HUMAN Calcium-binding mitochondrial carrier protein SCaMC-2 OS=Homo sapiens GN=SLC25A25 PE=1 SV=1//0
XM_008006105.1	0.38227	0.0011888	0.0067227	sp|Q5T9C2|F102A_HUMAN Protein FAM102A OS=Homo sapiens GN=FAM102A PE=1 SV=2//0
XM_008006130.1	-1.2257	1.26E-24	3.14E-23	sp|P17813|EGLN_HUMAN Endoglin OS=Homo sapiens GN=ENG PE=1 SV=2//0
XM_008006135.1	0.53432	1.62E-06	1.30E-05	sp|P50750|CDK9_HUMAN Cyclin-dependent kinase 9 OS=Homo sapiens GN=CDK9 PE=1 SV=3//0
XM_008006140.1	0.45052	0.0082209	0.039258	sp|Q52V08|CYC_MACSY Cytochrome c OS=Macaca sylvanus GN=CYCS PE=3 SV=3//4.39104e-67
XM_008006155.1	-1.9769	1.05E-13	1.47E-12	sp|Q5JU69|TOR2A_HUMAN Torsin-2A OS=Homo sapiens GN=TOR2A PE=2 SV=1//0
XM_008006159.1	-0.27692	0.00024	0.0014917	sp|Q96TA1|NIBL1_HUMAN Niban-like protein 1 OS=Homo sapiens GN=FAM129B PE=1 SV=3//0
XM_008006166.1	0.21899	0.004064	0.020683	sp|P30050|RL12_HUMAN 60S ribosomal protein L12 OS=Homo sapiens GN=RPL12 PE=1 SV=1//5.40456e-117
XM_008006170.1	1.6749	7.13E-87	1.06E-84	sp|P62071|RRAS2_MOUSE Ras-related protein R-Ras2 OS=Mus musculus GN=Rras2 PE=1 SV=1//2.619e-133
XM_008006197.1	0.3824	2.89E-05	0.0002011	sp|Q5R834|FACR1_PONAB Fatty acyl-CoA reductase 1 OS=Pongo abelii GN=FAR1 PE=2 SV=1//0
XM_008006247.1	0.33675	0.0004669	0.0027975	sp|O00743|PPP6_HUMAN Serine/threonine-protein phosphatase 6 catalytic subunit OS=Homo sapiens GN=PPP6C PE=1 SV=1//0
XM_008006256.1	2.7143	7.88E-37	3.07E-35	sp|P11021|GRP78_HUMAN 78 kDa glucose-regulated protein OS=Homo sapiens GN=HSPA5 PE=1 SV=2//0
XM_008006269.1	-0.58894	2.05E-18	3.75E-17	sp|Q3MHM7|RL35_BOVIN 60S ribosomal protein L35 OS=Bos taurus GN=RPL35 PE=2 SV=3//3.06523e-58
XM_008006270.1	0.89863	7.06E-12	8.83E-11	sp|Q5R4M1|ARP5L_PONAB Actin-related protein 2/3 complex subunit 5-like protein OS=Pongo abelii GN=ARPC5L PE=2 SV=1//3.62992e-92
XM_008006272.1	-2.858	4.33E-24	1.05E-22	sp|Q68BL7|OLM2A_HUMAN Olfactomedin-like protein 2A OS=Homo sapiens GN=OLFML2A PE=2 SV=1//0

XM_008006305.1	0.47498	0.0001431	0.0009183	sp|P50458|LHX2_HUMAN LIM/homeobox protein Lhx2 OS=Homo sapiens GN=LHX2 PE=2 SV=2//0
XM_008006340.1	1.1205	1.49E-12	1.95E-11	sp|Q15916|ZBTB6_HUMAN Zinc finger and BTB domain-containing protein 6 OS=Homo sapiens GN=ZBTB6 PE=1 SV=1//0
XM_008006365.1	-1.0745	5.00E-10	5.45E-09	sp|Q6ZV29|PLPL7_HUMAN Patatin-like phospholipase domain-containing protein 7 OS=Homo sapiens GN=PNPLA7 PE=1 SV=3//1.81501e-111
XM_008006366.1	-2.8534	0.0007417	0.0043161	-//-
XM_008006371.1	1.3105	5.45E-12	6.90E-11	sp|O75683|SURF6_HUMAN Surfeit locus protein 6 OS=Homo sapiens GN=SURF6 PE=1 SV=3//9.06579e-70
XM_008006375.1	1.044	1.28E-17	2.24E-16	sp|Q7L4E1|FA73B_HUMAN Protein FAM73B OS=Homo sapiens GN=FAM73B PE=1 SV=1//0
XM_008006377.1	-0.48918	0.0002128	0.0013342	sp|Q96EX3|WDR34_HUMAN WD repeat-containing protein 34 OS=Homo sapiens GN=WDR34 PE=1 SV=2//0
XM_008006417.1	1.4076	4.86E-45	2.51E-43	sp|Q8WWQ0|PHIP_HUMAN PH-interacting protein OS=Homo sapiens GN=PHIP PE=1 SV=2//0
XM_008006423.1	0.50182	0.0035121	0.018114	sp|Q86VQ0|LCA5_HUMAN Lebercilin OS=Homo sapiens GN=LCA5 PE=1 SV=2//0
XM_008006430.1	0.85351	0.001758	0.0096403	sp|Q3S8M4|ELOV4_MACMU Elongation of very long chain fatty acids protein 4 OS=Macaca mulatta GN=ELOVL4 PE=3 SV=1//0
XM_008006439.1	1.2923	2.11E-15	3.23E-14	sp|A6NK89|RASFA_HUMAN Ras association domain-containing protein 10 OS=Homo sapiens GN=RASSF10 PE=2 SV=3//0
XM_008006440.1	0.89676	4.70E-16	7.54E-15	sp|Q9P2D0|IBTK_HUMAN Inhibitor of Bruton tyrosine kinase OS=Homo sapiens GN=IBTK PE=1 SV=3//0
XM_008006444.1	-2.1976	0.0056516	0.027869	sp|Q4R8Y9|TPBG_MACFA Trophoblast glycoprotein OS=Macaca fascicularis GN=TPBG PE=2 SV=1//0
XM_008006476.1	-1.7369	4.55E-22	1.01E-20	sp|Q1WK24|PRS35_MACMU Inactive serine protease 35 OS=Macaca mulatta GN=PRSS35 PE=2 SV=1//0
XM_008006506.1	0.62428	2.22E-05	0.0001572	sp|Q7L1T6|NB5R4_HUMAN Cytochrome b5 reductase 4 OS=Homo sapiens GN=CYB5R4 PE=1 SV=1//0
XM_008006534.1	-0.26368	0.0032198	0.01678	sp|Q9NVD7|PARVA_HUMAN Alpha-parvin OS=Homo sapiens GN=PARVA PE=1 SV=1//0
XM_008006553.1	2.144	6.82E-43	3.22E-41	sp|Q53H80|AKIR2_HUMAN Akirin-2 OS=Homo sapiens GN=AKIRIN2 PE=1 SV=2//8.04837e-61
XM_008006566.1	2.3185	7.33E-08	6.64E-07	sp|Q5IS73|CNR1_PANTR Cannabinoid receptor 1 OS=Pan troglodytes GN=CNR1 PE=2 SV=1//0
XM_008006583.1	0.61409	1.49E-10	1.69E-09	sp|Q12796|PNRC1_HUMAN Proline-rich nuclear receptor coactivator 1 OS=Homo sapiens GN=PNRC1 PE=1 SV=1//4.44216e-175
XM_008006584.1	-0.87935	0.0093844	0.044235	sp|Q8IYS1|P20D2_HUMAN Peptidase M20 domain-containing protein 2 OS=Homo sapiens GN=PM20D2 PE=1 SV=2//0
XM_008006649.1	-0.42365	4.53E-06	3.48E-05	sp|Q5RD93|MANEA_PONAB Glycoprotein endo-alpha-1,2-mannosidase OS=Pongo abelii GN=MANEA PE=2 SV=1//0
XM_008006659.1	1.5331	4.00E-10	4.40E-09	sp|Q9BZM6|N2DL1_HUMAN NKG2D ligand 1 OS=Homo sapiens GN=ULBP1 PE=1 SV=1//1.27495e-124
XM_008006685.1	1.021	3.55E-14	5.11E-13	sp|Q4R407|KTNA1_MACFA Katanin p60 ATPase-containing subunit A1 OS=Macaca fascicularis GN=KATNA1 PE=2 SV=1//0
XM_008006686.1	-0.96603	2.33E-15	3.57E-14	sp|Q5RBQ2|GINM1_PONAB Glycoprotein integral membrane protein 1 OS=Pongo abelii GN=GINM1 PE=2 SV=1//0
XM_008006687.1	1.4014	1.55E-06	1.25E-05	sp|Q8WUA2|PPIL4_HUMAN Peptidyl-prolyl cis-trans isomerase-like 4 OS=Homo sapiens GN=PPIL4 PE=1 SV=1//0
XM_008006702.1	-0.79907	1.33E-30	4.19E-29	sp|P62425|RL7A_RAT 60S ribosomal protein L7a OS=Rattus norvegicus GN=Rpl7a PE=1 SV=2//1.63463e-157
XM_008006705.1	-0.61064	0.000661	0.0038741	sp|Q9Y2C2|UST_HUMAN Uronyl 2-sulfotransferase OS=Homo sapiens GN=UST PE=2 SV=1//0
XM_008006729.1	1.8463	1.24E-33	4.37E-32	sp|Q13637|RAB32_HUMAN Ras-related protein Rab-32 OS=Homo sapiens GN=RAB32 PE=1 SV=3//1.05313e-146
XM_008006741.1	0.90562	0.0011127	0.0063187	-//-
XM_008006745.1	-0.71661	0.0060119	0.0295	sp|O95278|EPM2A_HUMAN Laforin OS=Homo sapiens GN=EPM2A PE=1 SV=2//0
XM_008006750.1	1.5561	3.90E-22	8.67E-21	sp|Q9NU22|MDN1_HUMAN Midasin OS=Homo sapiens GN=MDN1 PE=1 SV=2//2.39613e-10
XM_008006752.1	0.59588	1.63E-20	3.31E-19	sp|Q5R7J9|IF4G2_PONAB Eukaryotic translation initiation factor 4 gamma 2 OS=Pongo abelii GN=EIF4G2 PE=2 SV=2//0
XM_008006764.1	0.33067	0.0007852	0.0045509	sp|Q6PD62|CTR9_HUMAN RNA polymerase-associated protein CTR9 homolog OS=Homo sapiens GN=CTR9 PE=1 SV=1//0
XM_008006796.1	1.5851	7.26E-48	4.16E-46	sp|Q5R8B2|LTV1_PONAB Protein LTV1 homolog OS=Pongo abelii GN=LTV1 PE=2 SV=1//0
XM_008006807.1	-1.0871	2.40E-18	4.37E-17	sp|Q9BTY2|FUCO2_HUMAN Plasma alpha-L-fucosidase OS=Homo sapiens GN=FUCA2 PE=1 SV=2//0
XM_008006811.1	0.57875	0.0002148	0.0013459	sp|Q60HE1|PEX3_MACFA Peroxisomal biogenesis factor 3 OS=Macaca fascicularis GN=PEX3 PE=2 SV=1//0
XM_008006844.1	2.6807	8.61E-300	8.58E-297	sp|Q99967|CITE2_HUMAN Cbp/p300-interacting transactivator 2 OS=Homo sapiens GN=CITED2 PE=1 SV=2//2.91823e-127

XM_008006872.1	-0.81162	0.0002681	0.0016552	sp|Q9Y5Z4|HEBP2_HUMAN Heme-binding protein 2 OS=Homo sapiens GN=HEBP2 PE=1 SV=1//1.73434e-122
XM_008006875.1	-0.54947	0.0043155	0.021876	sp|Q5TH69|BIG3_HUMAN Brefeldin A-inhibited guanine nucleotide-exchange protein 3 OS=Homo sapiens GN=ARFGEF3 PE=1 SV=3//0
XM_008006876.1	-0.40852	0.000114	0.0007425	sp|Q96FX8|PERP_HUMAN p53 apoptosis effector related to PMP-22 OS=Homo sapiens GN=PERP PE=2 SV=1//2.68304e-111
XM_008006962.1	0.62373	9.72E-08	8.70E-07	sp|Q9Y450|HBS1L_HUMAN HBS1-like protein OS=Homo sapiens GN=HBS1L PE=1 SV=1//0
XM_008006963.1	2.148	7.78E-11	8.98E-10	sp|Q5R6Y0|HBS1L_PONAB HBS1-like protein OS=Pongo abelii GN=HBS1L PE=2 SV=1//8.84218e-87
XM_008006998.1	-0.43531	5.46E-08	5.00E-07	sp|P46405|RS12_PIG 40S ribosomal protein S12 OS=Sus scrofa GN=RPS12 PE=2 SV=2//5.74885e-90
XM_008007024.1	4.1844	2.84E-203	1.48E-200	sp|P29279|CTGF_HUMAN Connective tissue growth factor OS=Homo sapiens GN=CTGF PE=1 SV=2//0
XM_008007025.1	-1.0443	2.10E-08	2.00E-07	sp|P22413|ENPP1_HUMAN Ectonucleotide pyrophosphatase/phosphodiesterase family member 1 OS=Homo sapiens GN=ENPP1 PE=1 SV=2//0
XM_008007140.1	2.5693	1.23E-78	1.52E-76	sp|Q9BXY4|RSPO3_HUMAN R-spondin-3 OS=Homo sapiens GN=RSPO3 PE=1 SV=1//1.28432e-138
XM_008007146.1	1.8417	3.12E-17	5.36E-16	sp|Q7Z4G4|TRM11_HUMAN tRNA (guanine(10)-N2)-methyltransferase homolog OS=Homo sapiens GN=TRMT11 PE=1 SV=1//0
XM_008007159.1	-0.81671	1.51E-19	2.93E-18	sp|Q7Z4H3|HDDC2_HUMAN HD domain-containing protein 2 OS=Homo sapiens GN=HDDC2 PE=1 SV=1//4.02225e-112
XM_008007169.1	-0.83573	5.97E-09	5.93E-08	sp|Q8TC41|RN217_HUMAN Probable E3 ubiquitin-protein ligase RNF217 OS=Homo sapiens GN=RNF217 PE=2 SV=4//0
XM_008007172.1	2.39	0.0078388	0.037556	sp|Q8TC41|RN217_HUMAN Probable E3 ubiquitin-protein ligase RNF217 OS=Homo sapiens GN=RNF217 PE=2 SV=4//2.13436e-138
XM_008007177.1	0.58658	3.09E-09	3.14E-08	sp|Q9UH65|SWP70_HUMAN Switch-associated protein 70 OS=Homo sapiens GN=SWAP70 PE=1 SV=1//0
XM_008007194.1	0.42805	6.56E-06	4.95E-05	sp|Q9NRX5|SERC1_HUMAN Serine incorporator 1 OS=Homo sapiens GN=SERINC1 PE=1 SV=1//0
XM_008007199.1	0.39791	9.14E-08	8.20E-07	sp|Q6TYA9|CXA1_CHLAE Gap junction alpha-1 protein OS=Chlorocebus aethiops GN=GJA1 PE=1 SV=1//0
XM_008007216.1	0.67243	0.0003079	0.0018855	sp|Q3C1E9|ASF1_CHICK Histone chaperone ASF1 OS=Gallus gallus GN=ASF1 PE=1 SV=1//6.80887e-147
XM_008007227.1	0.34754	0.0001693	0.0010763	sp|Q96E22|NGBR_HUMAN Dehydrodolichyl diphosphate syntase complex subunit NUS1 OS=Homo sapiens GN=NUS1 PE=1 SV=1//0
XM_008007231.1	0.48776	0.0029856	0.015667	sp|Q8N8Z6|DCBD1_HUMAN Discoidin, CUB and LCCL domain-containing protein 1 OS=Homo sapiens GN=DCBLD1 PE=1 SV=2//0
XM_008007281.1	0.23496	0.0021849	0.011753	sp|Q92769|HDAC2_HUMAN Histone deacetylase 2 OS=Homo sapiens GN=HDAC2 PE=1 SV=2//0
XM_008007284.1	-0.501	0.0028732	0.015132	sp|P29966|MARCS_HUMAN Myristoylated alanine-rich C-kinase substrate OS=Homo sapiens GN=MARCKS PE=1 SV=4//2.65146e-16
XM_008007293.1	0.59257	1.60E-14	2.35E-13	sp|Q6IQ26|DEN5A_HUMAN DENN domain-containing protein 5A OS=Homo sapiens GN=DENND5A PE=1 SV=2//0
XM_008007313.1	1.3436	1.38E-38	5.70E-37	sp|P17707|DCAM_HUMAN S-adenosylmethionine decarboxylase proenzyme OS=Homo sapiens GN=AMD1 PE=1 SV=2//0
XM_008007322.1	0.59607	1.72E-05	0.0001234	sp|Q5TF39|NAGT1_HUMAN Sodium-dependent glucose transporter 1 OS=Homo sapiens GN=NAGLT1 PE=2 SV=1//0
XM_008007330.1	-0.5678	0.0002509	0.0015556	sp|O14521|DHSD_HUMAN Succinate dehydrogenase [ubiquinone] cytochrome b small subunit, mitochondrial OS=Homo sapiens GN=SDHD PE=1 SV=1//1.17384e-93
XM_008007370.1	-0.65624	1.76E-05	0.0001259	sp|Q8TDZ2|MICA1_HUMAN Protein-methionine sulfoxide oxidase MICAL1 OS=Homo sapiens GN=MICAL1 PE=1 SV=2//0
XM_008007414.1	0.67366	1.11E-10	1.27E-09	sp|Q86WC4|OSTM1_HUMAN Osteopetrosis-associated transmembrane protein 1 OS=Homo sapiens GN=OSTM1 PE=1 SV=1//1.18267e-163
XM_008007436.1	0.94276	0.003293	0.017129	sp|A7XYQ1|SOBP_HUMAN Sine oculis-binding protein homolog OS=Homo sapiens GN=SOBP PE=1 SV=2//0
XM_008007452.1	-0.45009	0.0061978	0.030337	sp|Q8WWV3|RT4I1_HUMAN Reticulon-4-interacting protein 1, mitochondrial OS=Homo sapiens GN=RTN4IP1 PE=1 SV=2//0
XM_008007454.1	0.84624	1.06E-10	1.22E-09	sp|Q4R7R9|GATA_MACFA Glutamyl-tRNA(Gln) amidotransferase subunit A, mitochondrial OS=Macaca fascicularis GN=QRSL1 PE=2 SV=1//0
XM_008007472.1	-0.73285	0.0047604	0.023877	sp|Q8NE79|POPD1_HUMAN Blood vessel epicardial substance OS=Homo sapiens GN=BVES PE=1 SV=1//0
XM_008007492.1	1.6908	2.40E-16	3.93E-15	sp|Q8N3C0|ASCC3_HUMAN Activating signal cointegrator 1 complex subunit 3 OS=Homo sapiens GN=ASCC3 PE=1 SV=3//1.32518e-37
XM_008007518.1	0.76634	1.83E-05	0.0001306	sp|P24863|CCNC_HUMAN Cyclin-C OS=Homo sapiens GN=CCNC PE=1 SV=2//0
XM_008007540.1	1.742	5.07E-26	1.35E-24	sp|Q9P032|NDUF4_HUMAN NADH dehydrogenase [ubiquinone] 1 alpha subcomplex assembly factor 4 OS=Homo sapiens GN=NDUFAF4 PE=1 SV=1//6.47074e-111
XM_008007541.1	1.3719	4.63E-11	5.46E-10	sp|Q9BZJ6|GPR63_HUMAN Probable G-protein coupled receptor 63 OS=Homo sapiens GN=GPR63 PE=2 SV=2//0

XM_008007556.1	-3.3545	1.28E-11	1.57E-10	sp|Q6PHW0|IYD1_HUMAN Iodotyrosine dehalogenase 1 OS=Homo sapiens GN=IYD PE=1 SV=2//3.70107e-168
XM_008007574.1	1.7184	0.0021712	0.011685	sp|Q02952|AKA12_HUMAN A-kinase anchor protein 12 OS=Homo sapiens GN=AKAP12 PE=1 SV=4//0
XM_008007579.1	0.46405	0.000553	0.0032865	sp|Q4R526|ARMT1_MACFA Protein-glutamate O-methyltransferase OS=Macaca fascicularis GN=ARMT1 PE=2 SV=2//0
XM_008007625.1	-0.59176	5.15E-06	3.94E-05	sp|Q4R723|RL27A_MACFA 60S ribosomal protein L27a OS=Macaca fascicularis GN=RPL27A PE=2 SV=1//3.16593e-87
XM_008007652.1	0.76149	2.49E-07	2.15E-06	sp|Q6P9H4|CNKR3_HUMAN Connector enhancer of kinase suppressor of ras 3 OS=Homo sapiens GN=CNKSR3 PE=1 SV=1//0
XM_008007663.1	3.566	2.56E-61	2.09E-59	sp|Q8IVF5|TIAM2_HUMAN T-lymphoma invasion and metastasis-inducing protein 2 OS=Homo sapiens GN=TIAM2 PE=2 SV=4//0
XM_008007664.1	3.3384	1.46E-198	7.27E-196	sp|Q8IVF5|TIAM2_HUMAN T-lymphoma invasion and metastasis-inducing protein 2 OS=Homo sapiens GN=TIAM2 PE=2 SV=4//0
XM_008007701.1	2.3606	3.60E-22	8.00E-21	sp|Q96JX3|SRAC1_HUMAN Protein SERAC1 OS=Homo sapiens GN=SERAC1 PE=1 SV=1//0
XM_008007706.1	0.70644	2.18E-06	1.73E-05	sp|Q9NRJ4|TULP4_HUMAN Tubby-related protein 4 OS=Homo sapiens GN=TULP4 PE=2 SV=2//0
XM_008007707.1	0.71699	5.74E-08	5.25E-07	sp|Q9P2C4|TM181_HUMAN Transmembrane protein 181 OS=Homo sapiens GN=TMEM181 PE=1 SV=2//0
XM_008007713.1	0.54487	4.09E-17	6.97E-16	sp|P15311|EZRI_HUMAN Ezrin OS=Homo sapiens GN=EZR PE=1 SV=4//0
XM_008007728.1	0.33725	3.93E-05	0.0002694	sp|P17987|TCPA_HUMAN T-complex protein 1 subunit alpha OS=Homo sapiens GN=TCP1 PE=1 SV=1//0
XM_008007729.1	-0.89715	4.42E-20	8.79E-19	sp|Q9BWD1|THIC_HUMAN Acetyl-CoA acetyltransferase, cytosolic OS=Homo sapiens GN=ACAT2 PE=1 SV=2//0
XM_008007770.1	1.4388	6.99E-82	9.22E-80	sp|Q5W9D5|QKI_PIG Protein quaking OS=Sus scrofa GN=QKI PE=2 SV=1//8.1931e-07
XM_008007800.1	-1.1401	5.08E-08	4.67E-07	sp|O00584|RNT2_HUMAN Ribonuclease T2 OS=Homo sapiens GN=RNASET2 PE=1 SV=2//2.35187e-166
XM_008007817.1	-0.65622	1.03E-16	1.74E-15	sp|A5A6I3|EIF3F_PANTR Eukaryotic translation initiation factor 3 subunit F OS=Pan troglodytes GN=EIF3F PE=2 SV=1//0
XM_008007862.1	-1.7432	8.15E-08	7.35E-07	sp|A2BDB0|ACTG_XENLA Actin, cytoplasmic 2 OS=Xenopus laevis GN=actg1 PE=2 SV=1//0
XM_008007878.1	-0.69718	3.26E-17	5.59E-16	sp|P35442|TSP2_HUMAN Thrombospondin-2 OS=Homo sapiens GN=THBS2 PE=1 SV=2//0
XM_008007879.1	0.63239	0.0012581	0.0070881	sp|A2RRH5|WDR27_HUMAN WD repeat-containing protein 27 OS=Homo sapiens GN=WDR27 PE=1 SV=3//3.74829e-11
XM_008007880.1	0.36203	0.0005557	0.0032999	sp|Q9Y446|PKP3_HUMAN Plakophilin-3 OS=Homo sapiens GN=PKP3 PE=1 SV=1//0
XM_008007892.1	-0.58675	2.79E-10	3.10E-09	sp|P20618|PSB1_HUMAN Proteasome subunit beta type-1 OS=Homo sapiens GN=PSMB1 PE=1 SV=2//4.47746e-175
XM_008007913.1	0.44384	2.46E-08	2.33E-07	sp|Q9UGP8|SEC63_HUMAN Translocation protein SEC63 homolog OS=Homo sapiens GN=SEC63 PE=1 SV=2//8.39325e-17
XM_008007917.1	0.99167	3.97E-21	8.33E-20	sp|Q6UB35|C1TM_HUMAN Monofunctional C1-tetrahydrofolate synthase, mitochondrial OS=Homo sapiens GN=MTHFD1L PE=1 SV=1//1.51808e-147
XM_008007926.1	1.1803	9.25E-11	1.06E-09	sp|P29037|TBP_MOUSE TATA-box-binding protein OS=Mus musculus GN=Tbp PE=1 SV=1//5.34531e-124
XM_008007931.1	0.33419	6.16E-05	0.0004139	sp|P82675|RT05_HUMAN 28S ribosomal protein S5, mitochondrial OS=Homo sapiens GN=MRPS5 PE=1 SV=2//0
XM_008007934.1	-1.147	0.0001846	0.0011656	sp|Q96NI8|ZN570_HUMAN Zinc finger protein 570 OS=Homo sapiens GN=ZNF570 PE=2 SV=1//5.32901e-86
XM_008007965.1	0.23307	0.001549	0.0085929	sp|Q9NQZ5|STAR7_HUMAN StAR-related lipid transfer protein 7, mitochondrial OS=Homo sapiens GN=STARD7 PE=1 SV=2//0
XM_008007969.1	-0.44728	9.32E-10	9.92E-09	sp|O75643|U520_HUMAN U5 small nuclear ribonucleoprotein 200 kDa helicase OS=Homo sapiens GN=SNRNP200 PE=1 SV=2//0
XM_008007976.1	4.9756	2.66E-65	2.45E-63	sp|Q96EH8|NEUL3_HUMAN E3 ubiquitin-protein ligase NEURL3 OS=Homo sapiens GN=NEURL3 PE=2 SV=2//2.18286e-171
XM_008008033.1	0.90118	4.57E-05	0.0003116	sp|Q6P4Q7|CNNM4_HUMAN Metal transporter CNNM4 OS=Homo sapiens GN=CNNM4 PE=1 SV=3//0
XM_008008035.1	-0.69264	0.0011505	0.0065166	sp|Q8NE01|CNNM3_HUMAN Metal transporter CNNM3 OS=Homo sapiens GN=CNNM3 PE=1 SV=1//0
XM_008008069.1	-1.3576	1.42E-20	2.91E-19	sp|P10606|COX5B_HUMAN Cytochrome c oxidase subunit 5B, mitochondrial OS=Homo sapiens GN=COX5B PE=1 SV=2//4.01303e-67
XM_008008074.1	0.40809	2.83E-05	0.0001974	sp|P42025|ACTY_HUMAN Beta-centractin OS=Homo sapiens GN=ACTR1B PE=1 SV=1//0
XM_008008111.1	-1.1388	4.56E-16	7.34E-15	sp|Q53HI1|UNC50_HUMAN Protein unc-50 homolog OS=Homo sapiens GN=UNC50 PE=1 SV=2//2.19203e-160
XM_008008112.1	-0.71684	5.46E-08	5.01E-07	sp|Q86WW8|COA5_HUMAN Cytochrome c oxidase assembly factor 5 OS=Homo sapiens GN=COA5 PE=1 SV=1//1.98982e-39
XM_008008150.1	0.35187	0.0043286	0.021932	sp|Q8WV92|MITD1_HUMAN MIT domain-containing protein 1 OS=Homo sapiens GN=MITD1 PE=1 SV=1//2.7216e-180

XM_008008151.1	-0.43346	0.0006971	0.0040716	sp|Q4R6U7|RM30_MACFA 39S ribosomal protein L30, mitochondrial OS=Macaca fascicularis GN=MRPL30 PE=2 SV=1//1.61111e-99
XM_008008159.1	0.52085	5.56E-07	4.66E-06	sp|O18883|TXND9_BOVIN Thioredoxin domain-containing protein 9 OS=Bos taurus GN=TXNDC9 PE=2 SV=2//1.01364e-136
XM_008008183.1	-0.3628	0.0002169	0.0013574	sp|Q9H2J4|PDCL3_HUMAN Phosducin-like protein 3 OS=Homo sapiens GN=PDCL3 PE=1 SV=1//6.20248e-130
XM_008008205.1	-0.27845	8.25E-05	0.0005474	sp|Q9UKZ1|CNO11_HUMAN CCR4-NOT transcription complex subunit 11 OS=Homo sapiens GN=CNOT11 PE=1 SV=1//0
XM_008008206.1	1.0397	3.93E-32	1.30E-30	sp|Q8NC42|RN149_HUMAN E3 ubiquitin-protein ligase RNF149 OS=Homo sapiens GN=RNF149 PE=2 SV=2//0
XM_008008207.1	4.0062	0.0001898	0.0011968	sp|Q6ZV50|RFX8_HUMAN DNA-binding protein RFX8 OS=Homo sapiens GN=RFX8 PE=2 SV=2//0
XM_008008238.1	0.92792	0.0002459	0.0015265	sp|Q9UL58|ZN215_HUMAN Zinc finger protein 215 OS=Homo sapiens GN=ZNF215 PE=2 SV=2//2.06119e-17
XM_008008350.1	4.2284	3.61E-92	5.72E-90	sp|Q53LP3|SWAHC_HUMAN Ankyrin repeat domain-containing protein SOWAHC OS=Homo sapiens GN=SOWAHC PE=1 SV=1//0
XM_008008367.1	0.28785	0.0002082	0.0013072	sp|O43683|BUB1_HUMAN Mitotic checkpoint serine/threonine-protein kinase BUB1 OS=Homo sapiens GN=BUB1 PE=1 SV=1//0
XM_008008368.1	0.9254	5.23E-08	4.81E-07	sp|O43521|B2L11_HUMAN Bcl-2-like protein 11 OS=Homo sapiens GN=BCL2L11 PE=1 SV=1//1.33453e-101
XM_008008389.1	1.9259	7.82E-10	8.35E-09	sp|Q8N5P1|ZC3H8_HUMAN Zinc finger CCCH domain-containing protein 8 OS=Homo sapiens GN=ZC3H8 PE=1 SV=2//6.54788e-160
XM_008008391.1	0.92385	0.0033795	0.017498	sp|P61129|ZC3H6_HUMAN Zinc finger CCCH domain-containing protein 6 OS=Homo sapiens GN=ZC3H6 PE=2 SV=2//0
XM_008008400.1	-1.2136	1.14E-07	1.01E-06	sp|Q9BSY4|CHCH5_HUMAN Coiled-coil-helix-coiled-coil-helix domain-containing protein 5 OS=Homo sapiens GN=CHCHD5 PE=1 SV=1//1.12833e-72
XM_008008401.1	2.5677	2.49E-101	4.66E-99	sp|Q5R9L5|S20A1_PONAB Sodium-dependent phosphate transporter 1 OS=Pongo abelii GN=SLC20A1 PE=2 SV=1//0
XM_008008412.1	0.49074	1.18E-06	9.61E-06	sp|Q8IYA6|CKP2L_HUMAN Cytoskeleton-associated protein 2-like OS=Homo sapiens GN=CKAP2L PE=1 SV=4//0
XM_008008414.1	-0.43343	0.0003573	0.0021749	sp|Q9NRX2|RM17_HUMAN 39S ribosomal protein L17, mitochondrial OS=Homo sapiens GN=MRPL17 PE=1 SV=1//1.40971e-104
XM_008008439.1	-0.93331	5.55E-28	1.59E-26	sp|Q60HH1|TPP1_MACFA Tripeptidyl-peptidase 1 OS=Macaca fascicularis GN=TPP1 PE=2 SV=2//0
XM_008008442.1	-1.6115	0.0002893	0.0017773	-//-
XM_008008456.1	4.5833	7.90E-88	1.20E-85	sp|O43159|RRP8_HUMAN Ribosomal RNA-processing protein 8 OS=Homo sapiens GN=RRP8 PE=1 SV=2//0
XM_008008466.1	1.0946	0.0074738	0.035933	sp|P49247|RPIA_HUMAN Ribose-5-phosphate isomerase OS=Homo sapiens GN=RPIA PE=1 SV=3//0
XM_008008480.1	-1.2552	0.0001799	0.0011387	sp|Q5RFE6|THNS2_PONAB Threonine synthase-like 2 OS=Pongo abelii GN=THNSL2 PE=2 SV=1//0
XM_008008493.1	0.26478	0.0063179	0.030849	sp|Q9NPI7|KRCC1_HUMAN Lysine-rich coiled-coil protein 1 OS=Homo sapiens GN=KRCC1 PE=2 SV=1//1.26462e-145
XM_008008507.1	-1.0774	0.0027599	0.014581	sp|Q96GK7|FAH2A_HUMAN Fumarylacetoacetate hydrolase domain-containing protein 2A OS=Homo sapiens GN=FAHD2A PE=1 SV=1//1.18446e-149
XM_008008510.1	-2.0281	1.34E-05	9.76E-05	sp|P20264|PO3F3_HUMAN POU domain, class 3, transcription factor 3 OS=Homo sapiens GN=POU3F3 PE=2 SV=2//0
XM_008008513.1	4.3364	0.0015493	0.0085929	sp|Q86YG4|NT5D4_HUMAN 5'-nucleotidase domain-containing protein 4 OS=Homo sapiens GN=NT5DC4 PE=2 SV=2//0
XM_008008528.1	-0.95132	1.10E-06	9.02E-06	sp|Q5SGD2|PPM1L_HUMAN Protein phosphatase 1L OS=Homo sapiens GN=PPM1L PE=1 SV=1//0
XM_008008531.1	1.0983	2.28E-45	1.19E-43	sp|O00629|IMA3_HUMAN Importin subunit alpha-3 OS=Homo sapiens GN=KPNA4 PE=1 SV=1//0
XM_008008545.1	1.2851	8.10E-08	7.30E-07	sp|F5H4A9|CC080_HUMAN Uncharacterized membrane protein C3orf80 OS=Homo sapiens GN=C3orf80 PE=2 SV=1//8.82184e-66
XM_008008547.1	3.3951	1.67E-15	2.58E-14	sp|P48091|IL12A_MACMU Interleukin-12 subunit alpha OS=Macaca mulatta GN=IL12A PE=2 SV=1//3.91184e-130
XM_008008556.1	Inf	0.0019634	0.010647	sp|Q9P0W5|SCHI1_HUMAN Schwannomin-interacting protein 1 OS=Homo sapiens GN=SCHIP1 PE=1 SV=1//5.65315e-101
XM_008008571.1	-2.0977	1.06E-09	1.13E-08	sp|Q9BS40|LXN_HUMAN Latexin OS=Homo sapiens GN=LXN PE=1 SV=2//2.77979e-154
XM_008008572.1	0.36238	1.88E-05	0.0001342	sp|Q96RP9|EFGM_HUMAN Elongation factor G, mitochondrial OS=Homo sapiens GN=GFM1 PE=1 SV=2//0
XM_008008573.1	0.32516	0.0004056	0.002453	sp|Q5R4T3|MLF1_PONAB Myeloid leukemia factor 1 OS=Pongo abelii GN=MLF1 PE=2 SV=1//1.56163e-163
XM_008008587.1	1.0439	0.0025904	0.013739	sp|P26022|PTX3_HUMAN Pentraxin-related protein PTX3 OS=Homo sapiens GN=PTX3 PE=1 SV=3//0
XM_008008596.1	1.8484	1.15E-40	5.06E-39	sp|Q9UK58|CCNL1_HUMAN Cyclin-L1 OS=Homo sapiens GN=CCNL1 PE=1 SV=1//0
XM_008008598.1	0.70236	8.88E-07	7.33E-06	sp|Q52KE7|CCNL1_MOUSE Cyclin-L1 OS=Mus musculus GN=Ccnl1 PE=1 SV=1//4.4919e-122

XM_008008613.1	2.5169	0.0021325	0.011493	sp|O00370|LORF2_HUMAN LINE-1 retrotransposable element ORF2 protein OS=Homo sapiens PE=1 SV=1//2.5298e-25
XM_008008618.1	0.77634	5.21E-22	1.15E-20	sp|Q9UNL2|SSRG_HUMAN Translocon-associated protein subunit gamma OS=Homo sapiens GN=SSR3 PE=1 SV=1//1.68417e-107
XM_008008660.1	1.1114	1.63E-08	1.57E-07	sp|Q9H2U1|DHX36_HUMAN ATP-dependent RNA helicase DHX36 OS=Homo sapiens GN=DHX36 PE=1 SV=2//0
XM_008008665.1	-1.683	1.37E-13	1.91E-12	sp|P47900|P2RY1_HUMAN P2Y purinoceptor 1 OS=Homo sapiens GN=P2RY1 PE=1 SV=1//0
XM_008008725.1	1.7477	2.82E-43	1.36E-41	sp|O43255|SIAH2_HUMAN E3 ubiquitin-protein ligase SIAH2 OS=Homo sapiens GN=SIAH2 PE=1 SV=1//0
XM_008008730.1	0.42855	5.20E-05	0.0003525	sp|P62341|SELT_HUMAN Selenoprotein T OS=Homo sapiens GN=SELT PE=2 SV=2//2.6199e-132
XM_008008731.1	1.2911	3.93E-37	1.55E-35	sp|Q9R2C1|SERP1_RAT Stress-associated endoplasmic reticulum protein 1 OS=Rattus norvegicus GN=Serp1 PE=1 SV=2//7.33768e-34
XM_008008738.1	2.8753	2.59E-107	5.62E-105	sp|O75157|T22D2_HUMAN TSC22 domain family protein 2 OS=Homo sapiens GN=TSC22D2 PE=1 SV=3//0
XM_008008740.1	0.32757	0.0030281	0.015865	sp|Q5RCV8|RNF13_PONAB E3 ubiquitin-protein ligase RNF13 OS=Pongo abelii GN=RNF13 PE=2 SV=1//0
XM_008008741.1	-0.46743	1.45E-10	1.64E-09	sp|Q5R4E2|PROF2_PONAB Profilin-2 OS=Pongo abelii GN=PFN2 PE=2 SV=3//5.99537e-91
XM_008008742.1	4.2109	0.0057142	0.028139	sp|A6NFN9|ANKUB_HUMAN Protein ANKUB1 OS=Homo sapiens GN=ANKUB1 PE=2 SV=2//0
XM_008008808.1	3.1128	2.52E-05	0.0001768	sp|O15162|PLS1_HUMAN Phospholipid scramblase 1 OS=Homo sapiens GN=PLSCR1 PE=1 SV=1//5.03072e-136
XM_008008832.1	1.4257	1.75E-39	7.52E-38	sp|Q8NDZ4|DIA1_HUMAN Deleted in autism protein 1 OS=Homo sapiens GN=C3orf58 PE=1 SV=1//0
XM_008008880.1	-2.0515	1.66E-05	0.0001194	sp|Q14188|TFDP2_HUMAN Transcription factor Dp-2 OS=Homo sapiens GN=TFDP2 PE=1 SV=2//1.83766e-12
XM_008008923.1	-0.99776	8.29E-10	8.84E-09	sp|P09455|RET1_HUMAN Retinol-binding protein 1 OS=Homo sapiens GN=RBP1 PE=1 SV=2//4.90472e-86
XM_008009044.1	-1.0487	5.49E-16	8.80E-15	sp|P05166|PCCB_HUMAN Propionyl-CoA carboxylase beta chain, mitochondrial OS=Homo sapiens GN=PCCB PE=1 SV=3//0
XM_008009045.1	1.6224	5.34E-63	4.59E-61	sp|Q9HCI7|MSL2_HUMAN E3 ubiquitin-protein ligase MSL2 OS=Homo sapiens GN=MSL2 PE=1 SV=2//0
XM_008009077.1	-0.83368	2.62E-09	2.68E-08	sp|Q92959|SO2A1_HUMAN Solute carrier organic anion transporter family member 2A1 OS=Homo sapiens GN=SLCO2A1 PE=1 SV=2//0
XM_008009081.1	-0.85716	1.83E-10	2.06E-09	sp|P61294|RAB6B_MOUSE Ras-related protein Rab-6B OS=Mus musculus GN=Rab6b PE=1 SV=1//3.46603e-137
XM_008009114.1	-0.40556	0.0033068	0.017189	sp|Q7Z494|NPHP3_HUMAN Nephrocystin-3 OS=Homo sapiens GN=NPHP3 PE=1 SV=1//0
XM_008009126.1	-0.22495	0.0093084	0.043905	sp|P09001|RM03_HUMAN 39S ribosomal protein L3, mitochondrial OS=Homo sapiens GN=MRPL3 PE=1 SV=1//0
XM_008009127.1	-0.82305	8.36E-05	0.0005546	sp|Q96DE0|NUD16_HUMAN U8 snoRNA-decapping enzyme OS=Homo sapiens GN=NUDT16 PE=1 SV=2//5.7709e-88
XM_008009161.1	0.42841	1.11E-06	9.07E-06	sp|Q99570|PI3R4_HUMAN Phosphoinositide 3-kinase regulatory subunit 4 OS=Homo sapiens GN=PIK3R4 PE=1 SV=3//0
XM_008009179.1	1.4652	1.30E-51	8.29E-50	sp|Q5RAM8|EAF1_PONAB ELL-associated factor 1 OS=Pongo abelii GN=EAF1 PE=2 SV=1//1.03221e-117
XM_008009180.1	0.95604	3.56E-05	0.0002461	sp|Q8TCB7|METL6_HUMAN Methyltransferase-like protein 6 OS=Homo sapiens GN=METTL6 PE=2 SV=2//0
XM_008009181.1	-1.4891	1.93E-08	1.85E-07	sp|P23528|COF1_HUMAN Cofilin-1 OS=Homo sapiens GN=CFL1 PE=1 SV=3//2.37961e-52
XM_008009210.1	1.3918	0.0003096	0.0018952	sp|O15084|ANR28_HUMAN Serine/threonine-protein phosphatase 6 regulatory ankyrin repeat subunit A OS=Homo sapiens GN=ANKRD28 PE=1 SV=5//5.33141e-28
XM_008009250.1	0.56518	8.45E-05	0.0005603	sp|Q01826|SATB1_HUMAN DNA-binding protein SATB1 OS=Homo sapiens GN=SATB1 PE=1 SV=1//0
XM_008009261.1	0.9289	1.07E-22	2.44E-21	sp|P61271|RAB5A_MACFA Ras-related protein Rab-5A OS=Macaca fascicularis GN=RAB5A PE=2 SV=1//6.25811e-152
XM_008009290.1	2.7594	4.87E-282	4.45E-279	sp|Q14995|NR1D2_HUMAN Nuclear receptor subfamily 1 group D member 2 OS=Homo sapiens GN=NR1D2 PE=1 SV=3//0
XM_008009292.1	1.5698	7.62E-06	5.71E-05	sp|P10828|THB_HUMAN Thyroid hormone receptor beta OS=Homo sapiens GN=THRB PE=1 SV=2//0
XM_008009299.1	0.66718	1.78E-06	1.42E-05	sp|Q9NWU1|OXSM_HUMAN 3-oxoacyl-[acyl-carrier-protein] synthase, mitochondrial OS=Homo sapiens GN=OXSM PE=1 SV=1//0
XM_008009318.1	-3.9577	6.58E-05	0.0004411	sp|Q6W4X9|MUC6_HUMAN Mucin-6 OS=Homo sapiens GN=MUC6 PE=1 SV=3//7.36463e-06
XM_008009324.1	-0.54689	0.009944	0.046622	sp|Q7Z7K0|COXM1_HUMAN COX assembly mitochondrial protein homolog OS=Homo sapiens GN=CMC1 PE=1 SV=1//1.76138e-63
XM_008009328.1	-3.5782	4.10E-05	0.0002811	sp|P98088|MUC5A_HUMAN Mucin-5AC OS=Homo sapiens GN=MUC5AC PE=1 SV=4//3.89528e-11

XM_008009344.1	-1.736	0.0029131	0.01532	sp|Q9HC84|MUC5B_HUMAN Mucin-5B OS=Homo sapiens GN=MUC5B PE=1 SV=3//2.02139e-06
XM_008009359.1	4.1901	2.67E-157	9.76E-155	-//-
XM_008009369.1	2.4905	4.27E-25	1.10E-23	-//-
XM_008009374.1	-0.95381	1.17E-30	3.69E-29	sp|Q9NX76|CKLF6_HUMAN CKLF-like MARVEL transmembrane domain-containing protein 6 OS=Homo sapiens GN=CMTM6 PE=1 SV=1//1.35995e-102
XM_008009389.1	-1.0015	1.44E-22	3.28E-21	sp|Q60HF6|BGAL_MACFA Beta-galactosidase OS=Macaca fascicularis GN=GLB1 PE=2 SV=1//0
XM_008009390.1	-1.5069	3.43E-61	2.76E-59	sp|O75718|CRTAP_HUMAN Cartilage-associated protein OS=Homo sapiens GN=CRTAP PE=1 SV=1//0
XM_008009428.1	-2.5566	6.75E-12	8.46E-11	-//-
XM_008009506.1	2.1118	1.40E-06	1.14E-05	sp|P41182|BCL6_HUMAN B-cell lymphoma 6 protein OS=Homo sapiens GN=BCL6 PE=1 SV=1//0
XM_008009507.1	2.1293	4.48E-11	5.29E-10	sp|P41182|BCL6_HUMAN B-cell lymphoma 6 protein OS=Homo sapiens GN=BCL6 PE=1 SV=1//0
XM_008009519.1	0.31886	3.49E-07	2.97E-06	-//-
XM_008009525.1	0.84858	0.0012894	0.0072527	sp|P59822|IL1AP_MACMU Interleukin-1 receptor accessory protein OS=Macaca mulatta GN=IL1RAP PE=1 SV=1//0
XM_008009535.1	0.89963	3.25E-08	3.04E-07	sp|P61150|FGF12_RAT Fibroblast growth factor 12 OS=Rattus norvegicus GN=Fgf12 PE=1 SV=1//2.63323e-157
XM_008009537.1	-0.88174	1.57E-13	2.18E-12	sp|Q12851|M4K2_HUMAN Mitogen-activated protein kinase kinase kinase kinase 2 OS=Homo sapiens GN=MAP4K2 PE=1 SV=2//0
XM_008009539.1	2.8213	1.93E-07	1.68E-06	sp|Q8IYB1|M21D2_HUMAN Protein MB21D2 OS=Homo sapiens GN=MB21D2 PE=1 SV=3//0
XM_008009555.1	0.25049	0.0002651	0.001638	sp|P31948|STIP1_HUMAN Stress-induced-phosphoprotein 1 OS=Homo sapiens GN=STIP1 PE=1 SV=1//0
XM_008009556.1	1.519	4.53E-60	3.53E-58	sp|Q14469|HES1_HUMAN Transcription factor HES-1 OS=Homo sapiens GN=HES1 PE=1 SV=1//4.0483e-153
XM_008009560.1	0.67476	0.0005717	0.0033868	sp|Q8TF66|LRC15_HUMAN Leucine-rich repeat-containing protein 15 OS=Homo sapiens GN=LRRC15 PE=2 SV=2//0
XM_008009561.1	-1.1783	9.86E-24	2.36E-22	sp|Q09666|AHNK_HUMAN Neuroblast differentiation-associated protein AHNAK OS=Homo sapiens GN=AHNAK PE=1 SV=2//0
XM_008009574.1	2.0696	1.46E-17	2.55E-16	sp|Q8N2R8|FA43A_HUMAN Protein FAM43A OS=Homo sapiens GN=FAM43A PE=2 SV=2//0
XM_008009576.1	1.7677	6.53E-35	2.42E-33	sp|Q4R8L2|LSG1_MACFA Large subunit GTPase 1 homolog OS=Macaca fascicularis GN=LSG1 PE=2 SV=1//0
XM_008009578.1	1.4871	1.59E-37	6.41E-36	sp|Q8NBI6|XXLT1_HUMAN Xyloside xylosyltransferase 1 OS=Homo sapiens GN=XXYLT1 PE=1 SV=1//0
XM_008009584.1	-0.899	1.31E-13	1.83E-12	sp|P02794|FRIH_HUMAN Ferritin heavy chain OS=Homo sapiens GN=FTH1 PE=1 SV=2//2.13876e-121
XM_008009595.1	-0.38029	0.0052356	0.026023	sp|Q15057|ACAP2_HUMAN Arf-GAP with coiled-coil, ANK repeat and PH domain-containing protein 2 OS=Homo sapiens GN=ACAP2 PE=1 SV=3//0
XM_008009656.1	1.5424	3.08E-27	8.56E-26	sp|P0C2W1|FBSP1_HUMAN F-box/SPRY domain-containing protein 1 OS=Homo sapiens GN=FBXO45 PE=1 SV=1//1.22386e-176
XM_008009658.1	0.4083	0.002097	0.011316	sp|Q8IYW5|RN168_HUMAN E3 ubiquitin-protein ligase RNF168 OS=Homo sapiens GN=RNF168 PE=1 SV=1//0
XM_008009659.1	0.3906	4.51E-05	0.0003074	sp|Q5REY7|UBXN7_PONAB UBX domain-containing protein 7 OS=Pongo abelii GN=UBXN7 PE=2 SV=2//0
XM_008009676.1	-0.65409	2.10E-05	0.0001492	sp|Q86UW1|OSTA_HUMAN Organic solute transporter subunit alpha OS=Homo sapiens GN=SLC51A PE=2 SV=1//0
XM_008009728.1	0.67965	6.36E-08	5.79E-07	sp|Q96CQ1|S2536_HUMAN Solute carrier family 25 member 36 OS=Homo sapiens GN=SLC25A36 PE=1 SV=1//0
XM_008009731.1	-1.2656	0.0086612	0.041154	-//-
XM_008009732.1	1.1151	0.0023738	0.012694	sp|Q9UKY7|CDV3_HUMAN Protein CDV3 homolog OS=Homo sapiens GN=CDV3 PE=1 SV=1//1.25204e-10
XM_008009733.1	1.223	9.70E-12	1.20E-10	sp|Q14699|RFTN1_HUMAN Raftlin OS=Homo sapiens GN=RFTN1 PE=1 SV=4//0
XM_008009734.1	0.63843	2.35E-11	2.85E-10	sp|Q9Y6G9|DC1L1_HUMAN Cytoplasmic dynein 1 light intermediate chain 1 OS=Homo sapiens GN=DYNC1LI1 PE=1 SV=3//0
XM_008009736.1	-4.3723	0.0013614	0.0076299	-//-
XM_008009739.1	-2.0439	7.71E-35	2.85E-33	sp|Q99102|MUC4_HUMAN Mucin-4 OS=Homo sapiens GN=MUC4 PE=1 SV=4//0
XM_008009740.1	-2.8282	1.89E-06	1.51E-05	-//-
XM_008009746.1	0.42818	0.0068211	0.033055	sp|Q12979|ABR_HUMAN Active breakpoint cluster region-related protein OS=Homo sapiens GN=ABR PE=2 SV=2//0
XM_008009747.1	0.96953	2.61E-13	3.57E-12	sp|Q9JKW1|TIM22_RAT Mitochondrial import inner membrane translocase subunit Tim22 OS=Rattus norvegicus GN=Timm22 PE=2 SV=2//2.01685e-82
XM_008009748.1	1.0072	2.75E-34	9.97E-33	sp|Q6DKJ4|NXN_HUMAN Nucleoredoxin OS=Homo sapiens GN=NXN PE=1 SV=2//0
XM_008009749.1	1.3168	6.45E-49	3.84E-47	sp|Q9Z0W3|NU160_MOUSE Nuclear pore complex protein Nup160 OS=Mus musculus GN=Nup160 PE=1 SV=2//0

XM_008009750.1	0.31089	0.0019281	0.010474	sp|Q9CPV4|GLOD4_MOUSE Glyoxalase domain-containing protein 4 OS=Mus musculus GN=Glod4 PE=1 SV=1//0
XM_008009758.1	-0.87492	8.00E-05	0.0005319	sp|Q8CCB4|VPS53_MOUSE Vacuolar protein sorting-associated protein 53 homolog OS=Mus musculus GN=Vps53 PE=2 SV=1//0
XM_008009759.1	-1.5013	3.26E-21	6.88E-20	sp|Q8N5W9|F101B_HUMAN Filamin-interacting protein FAM101B OS=Homo sapiens GN=FAM101B PE=1 SV=1//2.3515e-66
XM_008009766.1	-2.4613	0.0021187	0.011425	sp|Q8IXB3|TUSC5_HUMAN Tumor suppressor candidate 5 OS=Homo sapiens GN=TUSC5 PE=2 SV=2//3.05461e-60
XM_008009785.1	-0.97749	2.30E-06	1.81E-05	sp|A6NGC4|TLCD2_HUMAN TLC domain-containing protein 2 OS=Homo sapiens GN=TLCD2 PE=3 SV=3//3.78359e-121
XM_008009786.1	-0.28846	1.06E-05	7.81E-05	sp|Q6P2Q9|PRP8_HUMAN Pre-mRNA-processing-splicing factor 8 OS=Homo sapiens GN=PRPF8 PE=1 SV=2//0
XM_008009808.1	-2.9687	4.73E-47	2.64E-45	sp|Q86UN2|R4RL1_HUMAN Reticulon-4 receptor-like 1 OS=Homo sapiens GN=RTN4RL1 PE=1 SV=1//0
XM_008009821.1	0.39739	1.52E-07	1.34E-06	sp|Q9H422|HIPK3_HUMAN Homeodomain-interacting protein kinase 3 OS=Homo sapiens GN=HIPK3 PE=1 SV=1//0
XM_008009825.1	1.1394	1.44E-32	4.81E-31	sp|Q2NL82|TSR1_HUMAN Pre-rRNA-processing protein TSR1 homolog OS=Homo sapiens GN=TSR1 PE=1 SV=1//0
XM_008009829.1	0.9645	2.02E-15	3.10E-14	sp|Q99583|MNT_HUMAN Max-binding protein MNT OS=Homo sapiens GN=MNT PE=1 SV=1//0
XM_008009830.1	1.0305	4.33E-20	8.62E-19	sp|Q86W50|MET16_HUMAN Methyltransferase-like protein 16 OS=Homo sapiens GN=METTL16 PE=1 SV=2//0
XM_008009832.1	1.4411	1.16E-17	2.04E-16	sp|Q5RDW9|CSTF3_PONAB Cleavage stimulation factor subunit 3 OS=Pongo abelii GN=CSTF3 PE=2 SV=1//1.44934e-96
XM_008009833.1	1.1019	1.37E-41	6.24E-40	sp|Q8HXX0|LIS1_MACFA Platelet-activating factor acetylhydrolase IB subunit alpha OS=Macaca fascicularis GN=PAFAH1B1 PE=2 SV=3//0
XM_008009875.1	-2.2193	0.0055806	0.027575	sp|Q9DBG9|TX1B3_MOUSE Tax1-binding protein 3 OS=Mus musculus GN=Tax1bp3 PE=1 SV=1//9.45244e-70
XM_008009878.1	1.811	6.39E-36	2.43E-34	sp|Q8TF76|HASP_HUMAN Serine/threonine-protein kinase haspin OS=Homo sapiens GN=GSG2 PE=1 SV=3//0
XM_008009904.1	0.86522	2.24E-24	5.48E-23	sp|O43149|ZZEF1_HUMAN Zinc finger ZZ-type and EF-hand domain-containing protein 1 OS=Homo sapiens GN=ZZEF1 PE=1 SV=6//0
XM_008009916.1	1.3042	5.05E-11	5.92E-10	sp|Q9BQG0|MBB1A_HUMAN Myb-binding protein 1A OS=Homo sapiens GN=MYBBP1A PE=1 SV=2//0
XM_008009918.1	-0.22607	0.0010653	0.0060592	sp|Q8IVW8|SPNS2_HUMAN Protein spinster homolog 2 OS=Homo sapiens GN=SPNS2 PE=1 SV=2//0
XM_008009925.1	-3.0953	5.63E-16	9.01E-15	sp|Q2TAL5|SMTL2_HUMAN Smoothelin-like protein 2 OS=Homo sapiens GN=SMTNL2 PE=2 SV=1//0
XM_008009926.1	-2.4533	0.0001658	0.0010556	sp|Q2TAL5|SMTL2_HUMAN Smoothelin-like protein 2 OS=Homo sapiens GN=SMTNL2 PE=2 SV=1//3.10615e-159
XM_008009928.1	0.77457	8.58E-16	1.36E-14	sp|Q1W1Y5|PELP1_MACMU Proline-, glutamic acid- and leucine-rich protein 1 OS=Macaca mulatta GN=PELP1 PE=2 SV=1//1.02638e-30
XM_008009937.1	-0.76191	0.0055772	0.027564	sp|Q9P086|MED11_HUMAN Mediator of RNA polymerase II transcription subunit 11 OS=Homo sapiens GN=MED11 PE=1 SV=2//6.15902e-76
XM_008009980.1	-0.8627	1.64E-33	5.74E-32	sp|P07737|PROF1_HUMAN Profilin-1 OS=Homo sapiens GN=PFN1 PE=1 SV=2//2.65892e-85
XM_008009981.1	3.9854	1.68E-05	0.0001203	sp|P13929|ENOB_HUMAN Beta-enolase OS=Homo sapiens GN=ENO3 PE=1 SV=5//0
XM_008009984.1	1.1175	3.22E-38	1.31E-36	sp|O00443|P3C2A_HUMAN Phosphatidylinositol 4-phosphate 3-kinase C2 domain-containing subunit alpha OS=Homo sapiens GN=PIK3C2A PE=1 SV=2//0
XM_008009985.1	-1.2715	1.48E-09	1.54E-08	sp|O75391|SPAG7_HUMAN Sperm-associated antigen 7 OS=Homo sapiens GN=SPAG7 PE=1 SV=2//4.1272e-135
XM_008010003.1	0.87504	0.0009149	0.0052523	sp|Q96NJ6|ZFP3_HUMAN Zinc finger protein 3 homolog OS=Homo sapiens GN=ZFP3 PE=2 SV=1//0
XM_008010015.1	0.5106	1.04E-10	1.19E-09	sp|Q9MZE0|C1QBP_CHLAE Complement component 1 Q subcomponent-binding protein, mitochondrial OS=Chlorocebus aethiops GN=C1QBP PE=1 SV=2//0
XM_008010055.1	4.0995	2.67E-17	4.61E-16	sp|Q6GPH4|XAF1_HUMAN XIAP-associated factor 1 OS=Homo sapiens GN=XAF1 PE=1 SV=1//0
XM_008010058.1	4.099	0.0036867	0.018921	sp|Q8N4B4|FBX39_HUMAN F-box only protein 39 OS=Homo sapiens GN=FBXO39 PE=2 SV=1//0
XM_008010059.1	Inf	0.0017583	0.0096403	sp|Q8N4B4|FBX39_HUMAN F-box only protein 39 OS=Homo sapiens GN=FBXO39 PE=2 SV=1//0
XM_008010072.1	-0.80055	0.003953	0.020155	sp|Q7RTY0|MOT13_HUMAN Monocarboxylate transporter 13 OS=Homo sapiens GN=SLC16A13 PE=2 SV=1//0
XM_008010095.1	-0.47011	4.34E-07	3.67E-06	sp|Q8HXY7|ACADV_MACFA Very long-chain specific acyl-CoA dehydrogenase, mitochondrial OS=Macaca fascicularis GN=ACADVL PE=2 SV=1//0
XM_008010101.1	-0.6732	9.36E-18	1.66E-16	sp|P60517|GBRAP_RAT Gamma-aminobutyric acid receptor-associated protein OS=Rattus norvegicus GN=Gabarap PE=1 SV=1//1.72297e-77
XM_008010127.1	1.1291	6.57E-16	1.05E-14	sp|Q693B1|KCD11_HUMAN BTB/POZ domain-containing protein KCTD11 OS=Homo sapiens GN=KCTD11 PE=1 SV=1//9.89201e-152
XM_008010138.1	-1.5157	3.62E-05	0.0002491	sp|Q8NFZ4|NLGN2_HUMAN Neuroligin-2 OS=Homo sapiens GN=NLGN2 PE=1 SV=1//0

XM_008010139.1	-1.5197	2.97E-08	2.79E-07	sp|Q8N2U0|TM256_HUMAN Transmembrane protein 256 OS=Homo sapiens GN=TMEM256 PE=3 SV=1//1.2394e-38
XM_008010141.1	-1.3314	7.42E-29	2.19E-27	sp|Q5R7K4|PCMD2_PONAB Protein-L-isoaspartate O-methyltransferase domain-containing protein 2 OS=Pongo abelii GN=PCMTD2 PE=2 SV=1//0
XM_008010165.1	1.0947	1.18E-17	2.07E-16	sp|P51116|FXR2_HUMAN Fragile X mental retardation syndrome-related protein 2 OS=Homo sapiens GN=FXR2 PE=1 SV=2//0
XM_008010175.1	0.20138	0.0020332	0.010996	sp|P60843|IF4A1_MOUSE Eukaryotic initiation factor 4A-I OS=Mus musculus GN=Eif4a1 PE=1 SV=1//0
XM_008010176.1	0.90112	6.78E-22	1.49E-20	sp|Q9H4L4|SENP3_HUMAN Sentrin-specific protease 3 OS=Homo sapiens GN=SENP3 PE=1 SV=2//0
XM_008010196.1	-3.821	0.000127	0.0008218	sp|Q15768|EFNB3_HUMAN Ephrin-B3 OS=Homo sapiens GN=EFNB3 PE=1 SV=1//2.84972e-168
XM_008010209.1	0.52402	0.0017253	0.0094756	sp|Q6P9G0|CB5D1_HUMAN Cytochrome b5 domain-containing protein 1 OS=Homo sapiens GN=CYB5D1 PE=2 SV=1//5.5252e-156
XM_008010210.1	-1.492	4.29E-29	1.28E-27	sp|Q9BRA0|LSMD1_HUMAN N-alpha-acetyltransferase 38, NatC auxiliary subunit OS=Homo sapiens GN=NAA38 PE=1 SV=1//2.44731e-53
XM_008010211.1	1.9497	0.0001332	0.000859	sp|A6NIN4|YQ014_HUMAN Putative uncharacterized protein FLJ38447 OS=Homo sapiens PE=5 SV=3//1.33196e-24
XM_008010232.1	-1.2842	3.47E-27	9.63E-26	sp|Q9Y5R8|TPPC1_HUMAN Trafficking protein particle complex subunit 1 OS=Homo sapiens GN=TRAPPC1 PE=1 SV=1//2.04016e-103
XM_008010233.1	0.85079	8.38E-08	7.55E-07	sp|Q8N137|CNTRB_HUMAN Centrobin OS=Homo sapiens GN=CNTROB PE=1 SV=1//0
XM_008010236.1	-3.2398	3.40E-08	3.17E-07	sp|Q8TD35|LKAM1_HUMAN Protein LKAAEAR1 OS=Homo sapiens GN=LKAAEAR1 PE=2 SV=3//2.00805e-102
XM_008010246.1	4.8992	0	0	sp|Q9BYJ1|LOXE3_HUMAN Hydroperoxide isomerase ALOXE3 OS=Homo sapiens GN=ALOXE3 PE=1 SV=1//0
XM_008010260.1	#NAME?	0.0045613	0.022984	-//-
XM_008010264.1	-0.3698	0.0034212	0.017686	sp|P63045|VAMP2_RAT Vesicle-associated membrane protein 2 OS=Rattus norvegicus GN=Vamp2 PE=1 SV=2//1.06727e-40
XM_008010274.1	-3.3621	1.55E-06	1.25E-05	sp|P41146|OPRX_HUMAN Nociceptin receptor OS=Homo sapiens GN=OPRL1 PE=1 SV=1//0
XM_008010282.1	0.64751	1.87E-11	2.27E-10	sp|O15067|PUR4_HUMAN Phosphoribosylformylglycinamidine synthase OS=Homo sapiens GN=PFAS PE=1 SV=4//0
XM_008010283.1	-2.8255	0.006406	0.031217	sp|Q53CG4|CX6B1_MACMU Cytochrome c oxidase subunit 6B1 OS=Macaca mulatta GN=COX6B1 PE=3 SV=3//7.50305e-54
XM_008010296.1	-0.55047	3.83E-16	6.20E-15	sp|P61255|RL26_MOUSE 60S ribosomal protein L26 OS=Mus musculus GN=Rpl26 PE=1 SV=1//1.33057e-86
XM_008010298.1	2.0646	8.08E-57	5.77E-55	sp|Q9GZM8|NDEL1_HUMAN Nuclear distribution protein nudE-like 1 OS=Homo sapiens GN=NDEL1 PE=1 SV=1//0
XM_008010326.1	-1.9466	1.64E-13	2.27E-12	sp|P35713|SOX18_HUMAN Transcription factor SOX-18 OS=Homo sapiens GN=SOX18 PE=1 SV=2//2.71018e-08
XM_008010381.1	0.33191	0.0034369	0.017755	sp|O75880|SCO1_HUMAN Protein SCO1 homolog, mitochondrial OS=Homo sapiens GN=SCO1 PE=1 SV=1//0
XM_008010449.1	1.0548	8.60E-06	6.39E-05	sp|O95361|TRI16_HUMAN Tripartite motif-containing protein 16 OS=Homo sapiens GN=TRIM16 PE=1 SV=3//0
XM_008010466.1	-0.70595	6.21E-07	5.19E-06	sp|Q8NAA5|LR75A_HUMAN Leucine-rich repeat-containing protein 75A OS=Homo sapiens GN=LRRC75A PE=2 SV=2//0
XM_008010476.1	0.92311	3.49E-23	8.17E-22	sp|Q96KM6|Z512B_HUMAN Zinc finger protein 512B OS=Homo sapiens GN=ZNF512B PE=1 SV=1//0
XM_008010490.1	-0.29859	0.0004273	0.002576	sp|Q9H3Z4|DNJC5_HUMAN DnaJ homolog subfamily C member 5 OS=Homo sapiens GN=DNAJC5 PE=1 SV=1//1.60339e-109
XM_008010492.1	2.9033	3.84E-138	1.12E-135	sp|P29275|AA2BR_HUMAN Adenosine receptor A2b OS=Homo sapiens GN=ADORA2B PE=2 SV=1//0
XM_008010510.1	1.6339	2.38E-21	5.07E-20	sp|Q8NFG4|FLCN_HUMAN Folliculin OS=Homo sapiens GN=FLCN PE=1 SV=1//0
XM_008010525.1	1.1792	3.08E-21	6.52E-20	sp|Q9NWA0|MED9_HUMAN Mediator of RNA polymerase II transcription subunit 9 OS=Homo sapiens GN=MED9 PE=1 SV=1//3.65809e-53
XM_008010545.1	-0.50493	3.89E-09	3.93E-08	sp|D3ZKD3|ALKB5_RAT RNA demethylase ALKBH5 OS=Rattus norvegicus GN=Alkbh5 PE=3 SV=1//0
XM_008010546.1	0.50363	6.51E-06	4.91E-05	sp|P55039|DRG2_HUMAN Developmentally-regulated GTP-binding protein 2 OS=Homo sapiens GN=DRG2 PE=1 SV=1//0
XM_008010547.1	1.1237	2.11E-21	4.51E-20	sp|Q8IVV7|GID4_HUMAN Glucose-induced degradation protein 4 homolog OS=Homo sapiens GN=GID4 PE=2 SV=1//2.23384e-156
XM_008010553.1	2.7069	4.49E-07	3.79E-06	sp|Q4R550|SYCC_MACFA Cysteine--tRNA ligase, cytoplasmic OS=Macaca fascicularis GN=CARS PE=2 SV=1//1.18127e-11
XM_008010580.1	1.2229	3.80E-31	1.22E-29	sp|Q8TEV9|SMCR8_HUMAN Smith-Magenis syndrome chromosomal region candidate gene 8 protein OS=Homo sapiens GN=SMCR8 PE=1 SV=2//0
XM_008010581.1	1.0891	1.34E-29	4.05E-28	sp|Q13472|TOP3A_HUMAN DNA topoisomerase 3-alpha OS=Homo sapiens GN=TOP3A PE=1 SV=1//0
XM_008010591.1	1.3401	2.72E-17	4.69E-16	sp|A0PJK1|SC5AA_HUMAN Sodium/glucose cotransporter 5 OS=Homo sapiens GN=SLC5A10 PE=1 SV=2//0
XM_008010592.1	1.4395	2.91E-20	5.85E-19	sp|Q5RBA8|KPRB_PONAB Phosphoribosyl pyrophosphate synthase-associated

				protein 2 OS=Pongo abelii GN=PRPSAP2 PE=2 SV=1//0
XM_008010635.1	0.85796	0.0051418	0.025597	sp|Q8IYT8|ULK2_HUMAN Serine/threonine-protein kinase ULK2 OS=Homo sapiens GN=ULK2 PE=1 SV=3//0
XM_008010645.1	3.4288	1.75E-14	2.56E-13	sp|Q5M775|CYTSB_HUMAN Cytospin-B OS=Homo sapiens GN=SPECC1 PE=1 SV=1//0
XM_008010683.1	-1.39	1.85E-11	2.25E-10	sp|Q8N6N6|NATD1_HUMAN Protein NATD1 OS=Homo sapiens GN=NATD1 PE=1 SV=2//5.27036e-69
XM_008010691.1	-0.63951	0.0001525	0.000974	sp|P36955|PEDF_HUMAN Pigment epithelium-derived factor OS=Homo sapiens GN=SERPINF1 PE=1 SV=4//0
XM_008010696.1	1.4986	1.67E-13	2.32E-12	sp|P38570|ITAE_HUMAN Integrin alpha-E OS=Homo sapiens GN=ITGAE PE=1 SV=3//7.42502e-97
XM_008010697.1	0.46572	1.17E-05	8.54E-05	sp|Q53F19|CQ085_HUMAN Uncharacterized protein C17orf85 OS=Homo sapiens GN=C17orf85 PE=1 SV=2//0
XM_008010699.1	0.37591	2.28E-06	1.80E-05	sp|O43896|KIF1C_HUMAN Kinesin-like protein KIF1C OS=Homo sapiens GN=KIF1C PE=1 SV=3//0
XM_008010701.1	-1.7438	3.86E-06	2.99E-05	sp|O95407|TNF6B_HUMAN Tumor necrosis factor receptor superfamily member 6B OS=Homo sapiens GN=TNFRSF6B PE=1 SV=1//7.71696e-171
XM_008010702.1	1.4436	3.05E-23	7.14E-22	sp|Q9H6R0|DHX33_HUMAN Putative ATP-dependent RNA helicase DHX33 OS=Homo sapiens GN=DHX33 PE=1 SV=2//3.42568e-59
XM_008010708.1	-0.33379	0.0011171	0.006338	sp|Q96F10|SAT2_HUMAN Diamine acetyltransferase 2 OS=Homo sapiens GN=SAT2 PE=1 SV=1//7.34409e-98
XM_008010712.1	1.1696	2.84E-37	1.12E-35	sp|Q5RFS2|CSN3_PONAB COP9 signalosome complex subunit 3 OS=Pongo abelii GN=COPS3 PE=2 SV=1//0
XM_008010734.1	1.2556	1.60E-64	1.44E-62	sp|Q9Y6I7|WSB1_HUMAN WD repeat and SOCS box-containing protein 1 OS=Homo sapiens GN=WSB1 PE=1 SV=1//0
XM_008010735.1	-3.0111	3.61E-05	0.0002491	sp|Q9H400|LIME1_HUMAN Lck-interacting transmembrane adapter 1 OS=Homo sapiens GN=LIME1 PE=1 SV=1//2.74109e-138
XM_008010751.1	3.3001	0.004783	0.023968	sp|Q8IVT5|KSR1_HUMAN Kinase suppressor of Ras 1 OS=Homo sapiens GN=KSR1 PE=1 SV=3//0
XM_008010772.1	-0.89019	2.21E-07	1.91E-06	sp|Q6SZW1|SARM1_HUMAN Sterile alpha and TIR motif-containing protein 1 OS=Homo sapiens GN=SARM1 PE=1 SV=1//0
XM_008010788.1	-1.2293	2.06E-07	1.79E-06	sp|Q13432|U119A_HUMAN Protein unc-119 homolog A OS=Homo sapiens GN=UNC119 PE=1 SV=1//6.3024e-134
XM_008010791.1	-0.71555	2.13E-14	3.10E-13	sp|Q96S52|PIGS_HUMAN GPI transamidase component PIG-S OS=Homo sapiens GN=PIGS PE=1 SV=3//0
XM_008010807.1	0.94416	1.12E-22	2.55E-21	sp|Q7KZ85|SPT6H_HUMAN Transcription elongation factor SPT6 OS=Homo sapiens GN=SUPT6H PE=1 SV=2//0
XM_008010827.1	-1.3042	0.0012776	0.0071936	sp|P62752|RL23A_RAT 60S ribosomal protein L23a OS=Rattus norvegicus GN=Rpl23a PE=2 SV=1//1.68862e-83
XM_008010835.1	0.71502	5.61E-13	7.49E-12	sp|Q9BUZ4|TRAF4_HUMAN TNF receptor-associated factor 4 OS=Homo sapiens GN=TRAF4 PE=1 SV=1//0
XM_008010880.1	1.1036	7.30E-45	3.75E-43	sp|Q7Z417|NUFP2_HUMAN Nuclear fragile X mental retardation-interacting protein 2 OS=Homo sapiens GN=NUFIP2 PE=1 SV=1//0
XM_008010892.1	-0.82123	0.0001893	0.0011947	sp|Q6UXT9|ABH15_HUMAN Abhydrolase domain-containing protein 15 OS=Homo sapiens GN=ABHD15 PE=2 SV=2//0
XM_008010905.1	-1.2069	0.0060961	0.029872	sp|Q86YJ7|AN13B_HUMAN Ankyrin repeat domain-containing protein 13B OS=Homo sapiens GN=ANKRD13B PE=1 SV=4//0
XM_008010923.1	-2.7561	0.0018377	0.010025	sp|Q9H3Y6|SRMS_HUMAN Tyrosine-protein kinase Srms OS=Homo sapiens GN=SRMS PE=1 SV=1//0
XM_008010938.1	-1.2952	2.81E-18	5.11E-17	sp|Q9H3Y8|PPDPF_HUMAN Pancreatic progenitor cell differentiation and proliferation factor OS=Homo sapiens GN=PPDPF PE=3 SV=1//1.35872e-50
XM_008010940.1	-1.3171	3.57E-47	1.99E-45	sp|O75976|CBPD_HUMAN Carboxypeptidase D OS=Homo sapiens GN=CPD PE=1 SV=2//0
XM_008010956.1	-3.2365	0.0098111	0.046068	sp|P23515|OMGP_HUMAN Oligodendrocyte-myelin glycoprotein OS=Homo sapiens GN=OMG PE=1 SV=2//0
XM_008010966.1	0.9764	3.31E-29	9.86E-28	sp|Q9NYH9|UTP6_HUMAN U3 small nucleolar RNA-associated protein 6 homolog OS=Homo sapiens GN=UTP6 PE=2 SV=2//0
XM_008010970.1	0.94319	1.28E-06	1.04E-05	sp|Q96QE5|TEFM_HUMAN Transcription elongation factor, mitochondrial OS=Homo sapiens GN=TEFM PE=1 SV=1//0
XM_008010974.1	-0.62285	0.0026425	0.014001	sp|Q9NPF8|ADAP2_HUMAN Arf-GAP with dual PH domain-containing protein 2 OS=Homo sapiens GN=ADAP2 PE=1 SV=1//0
XM_008010993.1	0.22022	0.0053536	0.026561	sp|F1LMZ8|PSD11_RAT 26S proteasome non-ATPase regulatory subunit 11 OS=Rattus norvegicus GN=Psmd11 PE=3 SV=2//0
XM_008010998.1	-0.60713	8.14E-07	6.74E-06	sp|O94832|MYO1D_HUMAN Unconventional myosin-Id OS=Homo sapiens GN=MYO1D PE=1 SV=2//0
XM_008011009.1	1.2413	0.0040064	0.020404	sp|Q5RA36|CCL2_PONAB C-C motif chemokine 2 OS=Pongo abelii GN=CCL2 PE=3 SV=1//2.39719e-49
XM_008011042.1	0.37936	0.0063429	0.03095	sp|Q8WZ73|RFFL_HUMAN E3 ubiquitin-protein ligase rififylin OS=Homo sapiens GN=RFFL PE=1 SV=1//0
XM_008011047.1	1.3951	9.80E-21	2.02E-19	sp|Q9NVX2|NLE1_HUMAN Notchless protein homolog 1 OS=Homo sapiens GN=NLE1 PE=1 SV=4//5.47285e-09

XM_008011065.1	1.0823	3.62E-08	3.37E-07	sp|O00623|PEX12_HUMAN Peroxisome assembly protein 12 OS=Homo sapiens GN=PEX12 PE=1 SV=1//0
XM_008011070.1	-0.7483	3.41E-09	3.45E-08	sp|Q5SSG5|RSLAB_MOUSE Ras-like protein family member 10B OS=Mus musculus GN=Rasl10b PE=1 SV=1//2.37257e-137
XM_008011081.1	3.5523	3.87E-07	3.29E-06	sp|Q8HYQ1|CCL5_MACMU C-C motif chemokine 5 OS=Macaca mulatta GN=CCL5 PE=3 SV=1//8.54918e-45
XM_008011172.1	-0.2477	0.0016444	0.0090631	sp|Q5IS89|LHX1_SAIBB LIM/homeobox protein Lhx1 OS=Saimiri boliviensis boliviensis GN=LHX1 PE=2 SV=1//0
XM_008011173.1	1.0583	4.45E-09	4.47E-08	sp|Q9NY61|AATF_HUMAN Protein AATF OS=Homo sapiens GN=AATF PE=1 SV=1//0
XM_008011178.1	1.6391	1.19E-31	3.88E-30	sp|Q6IN84|MRM1_HUMAN rRNA methyltransferase 1, mitochondrial OS=Homo sapiens GN=MRM1 PE=1 SV=1//0
XM_008011179.1	0.86261	1.16E-07	1.03E-06	sp|Q15649|ZNHI3_HUMAN Zinc finger HIT domain-containing protein 3 OS=Homo sapiens GN=ZNHIT3 PE=1 SV=2//1.41535e-104
XM_008011202.1	1.2657	3.00E-66	2.81E-64	sp|Q92624|APBP2_HUMAN Amyloid protein-binding protein 2 OS=Homo sapiens GN=APPBP2 PE=1 SV=2//0
XM_008011203.1	2.7847	1.216499994 32643e-316	1.48100000 000925e-313	sp|O15297|PPM1D_HUMAN Protein phosphatase 1D OS=Homo sapiens GN=PPM1D PE=1 SV=1//0
XM_008011204.1	-0.669	3.96E-08	3.66E-07	sp|Q13207|TBX2_HUMAN T-box transcription factor TBX2 OS=Homo sapiens GN=TBX2 PE=1 SV=3//0
XM_008011223.1	1.5079	1.30E-44	6.62E-43	sp|Q9BX63|FANCJ_HUMAN Fanconi anemia group J protein OS=Homo sapiens GN=BRIP1 PE=1 SV=1//0
XM_008011228.1	1.6184	3.64E-34	1.31E-32	sp|Q9UHV7|MED13_HUMAN Mediator of RNA polymerase II transcription subunit 13 OS=Homo sapiens GN=MED13 PE=1 SV=3//0
XM_008011242.1	1.5645	0.0022726	0.012182	sp|Q9Y3E5|PTH2_HUMAN Peptidyl-tRNA hydrolase 2, mitochondrial OS=Homo sapiens GN=PTRH2 PE=1 SV=1//5.58731e-124
XM_008011248.1	-0.57098	6.31E-14	8.99E-13	sp|Q8IX18|DHX40_HUMAN Probable ATP-dependent RNA helicase DHX40 OS=Homo sapiens GN=DHX40 PE=1 SV=2//1.56327e-29
XM_008011255.1	0.87691	0.001718	0.0094379	sp|Q8ND04|SMG8_HUMAN Protein SMG8 OS=Homo sapiens GN=SMG8 PE=1 SV=1//0
XM_008011261.1	0.97827	0.0008924	0.0051329	sp|Q8WY54|PPM1E_HUMAN Protein phosphatase 1E OS=Homo sapiens GN=PPM1E PE=1 SV=2//3.17069e-09
XM_008011317.1	0.30742	0.0072027	0.034744	sp|Q4R941|SPT4H_MACFA Transcription elongation factor SPT4 OS=Macaca fascicularis GN=SUPT4H1 PE=3 SV=1//2.57334e-81
XM_008011337.1	0.33752	1.28E-06	1.04E-05	sp|Q5R7H2|SRSF1_PONAB Serine/arginine-rich splicing factor 1 OS=Pongo abelii GN=SRSF1 PE=2 SV=3//3.93985e-85
XM_008011340.1	-1.2143	0.0024379	0.013014	sp|Q14119|VEZF1_HUMAN Vascular endothelial zinc finger 1 OS=Homo sapiens GN=VEZF1 PE=1 SV=2//0
XM_008011341.1	0.82335	3.72E-07	3.17E-06	sp|Q9Y3D9|RT23_HUMAN 28S ribosomal protein S23, mitochondrial OS=Homo sapiens GN=MRPS23 PE=1 SV=2//8.18064e-123
XM_008011364.1	0.93541	4.65E-28	1.34E-26	sp|Q9BYJ9|YTHD1_HUMAN YTH domain-containing family protein 1 OS=Homo sapiens GN=YTHDF1 PE=1 SV=1//0
XM_008011371.1	1.1373	7.59E-29	2.24E-27	sp|P38432|COIL_HUMAN Coilin OS=Homo sapiens GN=COIL PE=1 SV=1//0
XM_008011372.1	2.0087	3.83E-09	3.87E-08	-//-
XM_008011373.1	1.5461	1.42E-49	8.68E-48	sp|Q14258|TRI25_HUMAN E3 ubiquitin/ISG15 ligase TRIM25 OS=Homo sapiens GN=TRIM25 PE=1 SV=2//0
XM_008011393.1	Inf	0.0007758	0.0045022	sp|Q13253|NOGG_HUMAN Noggin OS=Homo sapiens GN=NOG PE=1 SV=1//2.4308e-126
XM_008011412.1	2.7682	2.00E-76	2.35E-74	sp|Q16534|HLF_HUMAN Hepatic leukemia factor OS=Homo sapiens GN=HLF PE=2 SV=1//3.5426e-151
XM_008011415.1	-0.67685	0.002449	0.013071	sp|O75674|TM1L1_HUMAN TOM1-like protein 1 OS=Homo sapiens GN=TOM1L1 PE=1 SV=2//0
XM_008011416.1	0.43743	7.51E-06	5.63E-05	sp|Q9Y6N1|COX11_HUMAN Cytochrome c oxidase assembly protein COX11, mitochondrial OS=Homo sapiens GN=COX11 PE=1 SV=3//2.35194e-177
XM_008011438.1	0.87769	2.93E-13	4.00E-12	sp|Q9Y5J1|UTP18_HUMAN U3 small nucleolar RNA-associated protein 18 homolog OS=Homo sapiens GN=UTP18 PE=1 SV=3//0
XM_008011460.1	-0.59213	0.0001506	0.0009644	sp|P50616|TOB1_HUMAN Protein Tob1 OS=Homo sapiens GN=TOB1 PE=1 SV=1//0
XM_008011474.1	0.86919	2.05E-24	5.04E-23	sp|Q6AI12|ANR40_HUMAN Ankyrin repeat domain-containing protein 40 OS=Homo sapiens GN=ANKRD40 PE=1 SV=2//0
XM_008011507.1	-3.6581	1.24E-08	1.21E-07	sp|O15335|CHAD_HUMAN Chondroadherin OS=Homo sapiens GN=CHAD PE=2 SV=2//0
XM_008011508.1	-1.5651	1.28E-18	2.39E-17	sp|Q4R4Z9|ACSF2_MACFA Acyl-CoA synthetase family member 2, mitochondrial OS=Macaca fascicularis GN=ACSF2 PE=2 SV=1//0
XM_008011515.1	0.74382	1.69E-16	2.79E-15	sp|Q96AG4|LRC59_HUMAN Leucine-rich repeat-containing protein 59 OS=Homo sapiens GN=LRRC59 PE=1 SV=1//8.1575e-115
XM_008011524.1	0.29475	0.0034307	0.017731	sp|Q9NWU2|GID8_HUMAN Glucose-induced degradation protein 8 homolog OS=Homo sapiens GN=GID8 PE=1 SV=1//2.53914e-153
XM_008011530.1	-0.48457	8.32E-09	8.18E-08	sp|Q96SB3|NEB2_HUMAN Neurabin-2 OS=Homo sapiens GN=PPP1R9B PE=1 SV=2//0
XM_008011547.1	-4.5524	0.0002774	0.001709	sp|O60479|DLX3_HUMAN Homeobox protein DLX-3 OS=Homo sapiens GN=DLX3 PE=2 SV=1//1.00549e-169

XM_008011585.1	-1.3051	1.21E-06	9.84E-06	sp|Q14050|CO9A3_HUMAN Collagen alpha-3(IX) chain OS=Homo sapiens GN=COL9A3 PE=1 SV=2//6.03835e-102
XM_008011587.1	-1.4155	0.0006588	0.0038634	sp|Q9H4G8|DPH3B_HUMAN Putative DPH3 homolog B OS=Homo sapiens GN=DPH3P1 PE=5 SV=1//1.90657e-42
XM_008011597.1	0.76481	6.22E-05	0.0004178	sp|Q9UHD8|SEPT9_HUMAN Septin-9 OS=Homo sapiens GN=SEPT9 PE=1 SV=2//0
XM_008011607.1	1.2791	1.37E-66	1.29E-64	sp|Q92503|S14L1_HUMAN SEC14-like protein 1 OS=Homo sapiens GN=SEC14L1 PE=1 SV=2//0
XM_008011618.1	0.85377	1.02E-08	9.99E-08	sp|Q5R6G2|JMJD6_PONAB Bifunctional arginine demethylase and lysyl-hydroxylase JMJD6 OS=Pongo abelii GN=JMJD6 PE=2 SV=1//0
XM_008011627.1	-1.7388	3.61E-07	3.07E-06	sp|P84157|MXRA7_HUMAN Matrix-remodeling-associated protein 7 OS=Homo sapiens GN=MXRA7 PE=1 SV=1//4.91281e-31
XM_008011649.1	1.371	0.008028	0.038387	sp|Q8WWM9|CYGB_HUMAN Cytoglobin OS=Homo sapiens GN=CYGB PE=1 SV=1//1.72658e-112
XM_008011662.1	0.29993	0.0037947	0.019434	sp|Q9NV56|MRGBP_HUMAN MRG/MORF4L-binding protein OS=Homo sapiens GN=MRGBP PE=1 SV=1//3.49753e-89
XM_008011670.1	-0.58677	1.60E-05	0.0001151	sp|Q8IYN6|UBAD2_HUMAN UBA-like domain-containing protein 2 OS=Homo sapiens GN=UBALD2 PE=1 SV=1//2.27152e-100
XM_008011678.1	-1.2462	3.89E-33	1.33E-31	sp|Q92949|FOXJ1_HUMAN Forkhead box protein J1 OS=Homo sapiens GN=FOXJ1 PE=2 SV=3//0
XM_008011697.1	-1.4787	8.97E-06	6.66E-05	sp|Q92817|EVPL_HUMAN Envoplakin OS=Homo sapiens GN=EVPL PE=1 SV=3//0
XM_008011704.1	0.45597	0.003896	0.019897	sp|Q96DV4|RM38_HUMAN 39S ribosomal protein L38, mitochondrial OS=Homo sapiens GN=MRPL38 PE=1 SV=2//1.04118e-95
XM_008011719.1	0.8739	2.01E-07	1.75E-06	sp|Q96LD4|TRI47_HUMAN Tripartite motif-containing protein 47 OS=Homo sapiens GN=TRIM47 PE=1 SV=2//0
XM_008011721.1	0.58732	1.98E-05	0.0001408	sp|Q70J99|UN13D_HUMAN Protein unc-13 homolog D OS=Homo sapiens GN=UNC13D PE=1 SV=1//0
XM_008011728.1	1.0961	1.02E-56	7.27E-55	sp|Q6P823|H33_XENTR Histone H3.3 OS=Xenopus tropicalis GN=TGas113e22.1 PE=1 SV=3//1.85616e-87
XM_008011729.1	-0.69936	1.78E-05	0.0001275	sp|P51570|GALK1_HUMAN Galactokinase OS=Homo sapiens GN=GALK1 PE=1 SV=1//0
XM_008011744.1	0.65318	2.08E-08	1.99E-07	sp|O94762|RECQ5_HUMAN ATP-dependent DNA helicase Q5 OS=Homo sapiens GN=RECQL5 PE=1 SV=2//0
XM_008011746.1	0.72694	0.0006309	0.0037134	sp|Q9UHR5|S30BP_HUMAN SAP30-binding protein OS=Homo sapiens GN=SAP30BP PE=1 SV=1//6.36919e-146
XM_008011770.1	-0.51247	6.71E-09	6.64E-08	sp|P62994|GRB2_RAT Growth factor receptor-bound protein 2 OS=Rattus norvegicus GN=Grb2 PE=1 SV=1//1.2556e-150
XM_008011782.1	0.48094	4.28E-06	3.30E-05	sp|Q9BW27|NUP85_HUMAN Nuclear pore complex protein Nup85 OS=Homo sapiens GN=NUP85 PE=1 SV=1//0
XM_008011783.1	-0.77493	4.52E-25	1.16E-23	sp|P61959|SUMO2_RAT Small ubiquitin-related modifier 2 OS=Rattus norvegicus GN=Sumo2 PE=1 SV=1//1.20321e-60
XM_008011798.1	-1.2124	8.15E-43	3.84E-41	sp|O75947|ATP5H_HUMAN ATP synthase subunit d, mitochondrial OS=Homo sapiens GN=ATP5H PE=1 SV=3//1.21044e-110
XM_008011799.1	0.61342	6.92E-09	6.83E-08	sp|Q14681|KCTD2_HUMAN BTB/POZ domain-containing protein KCTD2 OS=Homo sapiens GN=KCTD2 PE=1 SV=3//2.10811e-122
XM_008011801.1	-0.69509	3.06E-07	2.62E-06	sp|Q9BTV7|CABL2_HUMAN CDK5 and ABL1 enzyme substrate 2 OS=Homo sapiens GN=CABLES2 PE=1 SV=3//0
XM_008011851.1	-0.89401	3.76E-11	4.47E-10	sp|Q4R6G4|NHRF1_MACFA Na(+)/H(+) exchange regulatory cofactor NHE-RF1 OS=Macaca fascicularis GN=SLC9A3R1 PE=2 SV=1//0
XM_008011888.1	-1.165	0.0003294	0.0020122	sp|Q9NQ84|GPC5C_HUMAN G-protein coupled receptor family C group 5 member C OS=Homo sapiens GN=GPRC5C PE=1 SV=2//0
XM_008011890.1	-0.94144	0.0002744	0.0016911	sp|Q9NQ84|GPC5C_HUMAN G-protein coupled receptor family C group 5 member C OS=Homo sapiens GN=GPRC5C PE=1 SV=2//0
XM_008011912.1	Inf	3.10E-10	3.43E-09	-//-
XM_008011933.1	0.48106	0.0001813	0.0011464	sp|Q8WTW3|COG1_HUMAN Conserved oligomeric Golgi complex subunit 1 OS=Homo sapiens GN=COG1 PE=1 SV=1//0
XM_008011956.1	3.2302	3.52E-20	7.05E-19	sp|Q9BG91|SOX9_CALJA Transcription factor SOX-9 OS=Callithrix jacchus GN=SOX9 PE=2 SV=1//0
XM_008011967.1	-3.6335	8.14E-14	1.15E-12	sp|P52564|MP2K6_HUMAN Dual specificity mitogen-activated protein kinase kinase 6 OS=Homo sapiens GN=MAP2K6 PE=1 SV=1//0
XM_008011975.1	-0.81022	1.39E-14	2.05E-13	sp|Q3ZBG0|PSA7_BOVIN Proteasome subunit alpha type-7 OS=Bos taurus GN=PSMA7 PE=1 SV=1//9.74764e-162
XM_008012009.1	0.29243	0.0021956	0.011804	sp|Q12830|BPTF_HUMAN Nucleosome-remodeling factor subunit BPTF OS=Homo sapiens GN=BPTF PE=1 SV=3//0
XM_008012037.1	-1.4969	0.0020447	0.011053	sp|Q9UBN1|CCG4_HUMAN Voltage-dependent calcium channel gamma-4 subunit OS=Homo sapiens GN=CACNG4 PE=2 SV=1//0
XM_008012043.1	-2.2884	2.06E-14	3.00E-13	sp|Q95LB0|APOH_PANTR Beta-2-glycoprotein 1 OS=Pan troglodytes GN=APOH PE=2 SV=1//0
XM_008012068.1	1.5371	2.70E-10	3.01E-09	sp|Q14344|GNA13_HUMAN Guanine nucleotide-binding protein subunit alpha-13 OS=Homo sapiens GN=GNA13 PE=1 SV=2//0
XM_008012070.1	1.1813	0.0083461	0.039769	sp|Q4R684|AMZ2_MACFA Archaemetzincin-2 OS=Macaca fascicularis GN=AMZ2

				PE=2 SV=2//0
XM_008012078.1	0.65712	0.000465	0.002787	sp|Q9UHN1|DPOG2_HUMAN DNA polymerase subunit gamma-2, mitochondrial OS=Homo sapiens GN=POLG2 PE=1 SV=1//0
XM_008012079.1	0.76376	3.32E-31	1.06E-29	sp|P17844|DDX5_HUMAN Probable ATP-dependent RNA helicase DDX5 OS=Homo sapiens GN=DDX5 PE=1 SV=1//0
XM_008012108.1	2.4702	2.18E-13	3.00E-12	sp|P35499|SCN4A_HUMAN Sodium channel protein type 4 subunit alpha OS=Homo sapiens GN=SCN4A PE=1 SV=4//0
XM_008012128.1	-0.43113	4.71E-06	3.61E-05	sp|Q92925|SMRD2_HUMAN SWI/SNF-related matrix-associated actin-dependent regulator of chromatin subfamily D member 2 OS=Homo sapiens GN=SMARCD2 PE=1 SV=3//0
XM_008012156.1	0.50028	4.86E-06	3.72E-05	sp|Q99759|M3K3_HUMAN Mitogen-activated protein kinase kinase kinase 3 OS=Homo sapiens GN=MAP3K3 PE=1 SV=2//0
XM_008012165.1	-1.9563	0.0004625	0.0027746	sp|Q9GLN7|ACE_PANTR Angiotensin-converting enzyme OS=Pan troglodytes GN=ACE PE=3 SV=1//0
XM_008012192.1	-1.2875	8.42E-23	1.94E-21	sp|Q9UBG0|MRC2_HUMAN C-type mannose receptor 2 OS=Homo sapiens GN=MRC2 PE=1 SV=2//0
XM_008012205.1	0.41043	0.0098251	0.046124	sp|Q6P1Q9|MET2B_HUMAN Methyltransferase-like protein 2B OS=Homo sapiens GN=METTL2B PE=1 SV=3//0
XM_008012221.1	0.50889	2.69E-08	2.54E-07	sp|A6NMS7|L37A1_HUMAN Leucine-rich repeat-containing protein 37A OS=Homo sapiens GN=LRRC37A PE=2 SV=3//4.82501e-159
XM_008012225.1	-1.8973	0.0065737	0.031977	sp|O14653|GOSR2_HUMAN Golgi SNAP receptor complex member 2 OS=Homo sapiens GN=GOSR2 PE=1 SV=2//1.69165e-130
XM_008012228.1	-2.952	0.0001332	0.0008592	sp|O14653|GOSR2_HUMAN Golgi SNAP receptor complex member 2 OS=Homo sapiens GN=GOSR2 PE=1 SV=2//3.07112e-116
XM_008012233.1	-1.2195	2.45E-18	4.48E-17	sp|P56703|WNT3_HUMAN Proto-oncogene Wnt-3 OS=Homo sapiens GN=WNT3 PE=1 SV=2//0
XM_008012268.1	2.4384	0.0078635	0.037649	sp|Q7Z3B3|KANL1_HUMAN KAT8 regulatory NSL complex subunit 1 OS=Homo sapiens GN=KANSL1 PE=1 SV=2//0
XM_008012274.1	1.469	9.99E-45	5.13E-43	sp|A6NMS7|L37A1_HUMAN Leucine-rich repeat-containing protein 37A OS=Homo sapiens GN=LRRC37A PE=2 SV=3//0
XM_008012309.1	2.1106	5.16E-42	2.38E-40	sp|Q9Y4G2|PKHM1_HUMAN Pleckstrin homology domain-containing family M member 1 OS=Homo sapiens GN=PLEKHM1 PE=1 SV=3//0
XM_008012343.1	0.25443	0.0015203	0.0084431	sp|O94992|HEXI1_HUMAN Protein HEXIM1 OS=Homo sapiens GN=HEXIM1 PE=1 SV=1//2.08026e-149
XM_008012370.1	-3.9306	0.0088252	0.041833	sp|O88992|C1QRF_MOUSE C1q-related factor OS=Mus musculus GN=C1ql1 PE=1 SV=2//9.12809e-57
XM_008012378.1	-0.68677	0.0021057	0.011357	sp|O95685|PPR3D_HUMAN Protein phosphatase 1 regulatory subunit 3D OS=Homo sapiens GN=PPP1R3D PE=1 SV=1//2.89221e-151
XM_008012393.1	-1.4987	1.05E-13	1.48E-12	sp|Q9NTX9|F217B_HUMAN Protein FAM217B OS=Homo sapiens GN=FAM217B PE=2 SV=1//0
XM_008012398.1	1.0712	2.92E-10	3.24E-09	sp|Q96MW1|CCD43_HUMAN Coiled-coil domain-containing protein 43 OS=Homo sapiens GN=CCDC43 PE=1 SV=2//1.56519e-99
XM_008012400.1	1.3742	6.60E-34	2.35E-32	sp|Q8NFT6|DBF4B_HUMAN Protein DBF4 homolog B OS=Homo sapiens GN=DBF4B PE=1 SV=1//0
XM_008012403.1	-1.2664	3.56E-37	1.40E-35	sp|Q14332|FZD2_HUMAN Frizzled-2 OS=Homo sapiens GN=FZD2 PE=1 SV=1//0
XM_008012417.1	-1.4584	2.03E-22	4.58E-21	sp|A8MVW0|F1712_HUMAN Protein FAM171A2 OS=Homo sapiens GN=FAM171A2 PE=1 SV=1//0
XM_008012432.1	-2.376	1.51E-06	1.22E-05	sp|P17480|UBF1_HUMAN Nucleolar transcription factor 1 OS=Homo sapiens GN=UBTF PE=1 SV=1//0
XM_008012476.1	0.65519	7.71E-13	1.02E-11	sp|Q5RAT5|LSM12_PONAB Protein LSM12 homolog OS=Pongo abelii GN=LSM12 PE=2 SV=1//3.24901e-138
XM_008012479.1	1.3298	0.0051175	0.025505	sp|Q5RFN8|TM101_PONAB Transmembrane protein 101 OS=Pongo abelii GN=TMEM101 PE=2 SV=1//3.51969e-145
XM_008012514.1	1.606	4.52E-108	1.00E-105	sp|Q5RD73|DUS3_PONAB Dual specificity protein phosphatase 3 OS=Pongo abelii GN=DUSP3 PE=2 SV=1//5.37643e-123
XM_008012521.1	0.86236	0.0032745	0.017049	sp|P43268|ETV4_HUMAN ETS translocation variant 4 OS=Homo sapiens GN=ETV4 PE=1 SV=3//0
XM_008012523.1	2.2372	3.15E-09	3.20E-08	sp|Q5REU3|ARL4D_PONAB ADP-ribosylation factor-like protein 4D OS=Pongo abelii GN=ARL4D PE=2 SV=1//9.65898e-131
XM_008012542.1	-1.5645	3.82E-11	4.54E-10	sp|Q99536|VAT1_HUMAN Synaptic vesicle membrane protein VAT-1 homolog OS=Homo sapiens GN=VAT1 PE=1 SV=2//0
XM_008012544.1	-2.1212	1.90E-12	2.46E-11	sp|P52198|RND2_HUMAN Rho-related GTP-binding protein RhoN OS=Homo sapiens GN=RND2 PE=1 SV=2//4.4636e-150
XM_008012545.1	1.0655	2.77E-11	3.34E-10	sp|P80217|IN35_HUMAN Interferon-induced 35 kDa protein OS=Homo sapiens GN=IFI35 PE=1 SV=5//4.5622e-175
XM_008012552.1	-0.69374	8.49E-09	8.35E-08	sp|P61354|RL27_RAT 60S ribosomal protein L27 OS=Rattus norvegicus GN=Rpl27 PE=2 SV=2//1.25644e-83
XM_008012617.1	-0.90784	2.43E-11	2.93E-10	sp|Q86VR2|F134C_HUMAN Protein FAM134C OS=Homo sapiens GN=FAM134C PE=1 SV=1//0
XM_008012624.1	0.95461	3.95E-07	3.36E-06	sp|Q9P2W1|HOP2_HUMAN Homologous-pairing protein 2 homolog OS=Homo

				sapiens GN=PSMC3IP PE=1 SV=1//5.96011e-140
XM_008012630.1	-0.40703	0.0020727	0.011196	sp|P54802|ANAG_HUMAN Alpha-N-acetylglucosaminidase OS=Homo sapiens GN=NAGLU PE=1 SV=2//0
XM_008012631.1	-0.75909	1.22E-22	2.77E-21	sp|Q6NZI2|PTRF_HUMAN Polymerase I and transcript release factor OS=Homo sapiens GN=PTRF PE=1 SV=1//0
XM_008012648.1	-0.51098	0.0005884	0.0034791	sp|P42229|STA5A_HUMAN Signal transducer and activator of transcription 5A OS=Homo sapiens GN=STAT5A PE=1 SV=1//0
XM_008012680.1	-1.516	4.35E-16	7.00E-15	sp|P56381|ATP5E_HUMAN ATP synthase subunit epsilon, mitochondrial OS=Homo sapiens GN=ATP5E PE=1 SV=2//1.05468e-17
XM_008012693.1	-0.38461	0.0003721	0.0022603	sp|Q969T7|5NT3B_HUMAN 7-methylguanosine phosphate-specific 5'-nucleotidase OS=Homo sapiens GN=NT5C3B PE=1 SV=4//0
XM_008012696.1	-0.73319	0.0047027	0.023647	sp|Q92791|SC65_HUMAN Synaptonemal complex protein SC65 OS=Homo sapiens GN=LEPREL4 PE=1 SV=1//0
XM_008012710.1	1.2896	1.14E-64	1.03E-62	sp|Q5RFF4|EIF1_PONAB Eukaryotic translation initiation factor 1 OS=Pongo abelii GN=EIF1 PE=3 SV=1//5.00883e-60
XM_008012712.1	-0.71872	5.18E-19	9.79E-18	sp|Q9UBR2|CATZ_HUMAN Cathepsin Z OS=Homo sapiens GN=CTSZ PE=1 SV=1//0
XM_008012713.1	-2.5641	2.54E-131	6.97E-129	sp|P08727|K1C19_HUMAN Keratin, type I cytoskeletal 19 OS=Homo sapiens GN=KRT19 PE=1 SV=4//0
XM_008012744.1	0.983	0.0037898	0.019413	-//-
XM_008012755.1	1.8663	1.77E-06	1.42E-05	sp|Q07627|KRA11_HUMAN Keratin-associated protein 1-1 OS=Homo sapiens GN=KRTAP1-1 PE=2 SV=1//1.11613e-13
XM_008012758.1	0.54154	0.0026667	0.014122	sp|Q8N816|TMM99_HUMAN Transmembrane protein 99 OS=Homo sapiens GN=TMEM99 PE=2 SV=2//2.31341e-104
XM_008012761.1	1.7074	0.0001068	0.0006977	sp|P35900|K1C20_HUMAN Keratin, type I cytoskeletal 20 OS=Homo sapiens GN=KRT20 PE=1 SV=1//0
XM_008012764.1	1.5389	0.0014472	0.0080615	sp|Q6A163|K1C39_HUMAN Keratin, type I cytoskeletal 39 OS=Homo sapiens GN=KRT39 PE=1 SV=2//0
XM_008012767.1	2.8499	2.03E-12	2.63E-11	sp|Q9BYR6|KRA33_HUMAN Keratin-associated protein 3-3 OS=Homo sapiens GN=KRTAP3-3 PE=1 SV=1//1.23904e-42
XM_008012777.1	2.8699	2.81E-38	1.15E-36	sp|Q7Z3Y7|K1C28_HUMAN Keratin, type I cytoskeletal 28 OS=Homo sapiens GN=KRT28 PE=1 SV=2//0
XM_008012789.1	-0.32491	1.80E-06	1.44E-05	sp|P11388|TOP2A_HUMAN DNA topoisomerase 2-alpha OS=Homo sapiens GN=TOP2A PE=1 SV=3//0
XM_008012793.1	1.9812	3.54E-11	4.22E-10	sp|P10276|RARA_HUMAN Retinoic acid receptor alpha OS=Homo sapiens GN=RARA PE=1 SV=2//0
XM_008012797.1	1.4309	4.80E-06	3.68E-05	sp|Q99741|CDC6_HUMAN Cell division control protein 6 homolog OS=Homo sapiens GN=CDC6 PE=1 SV=1//0
XM_008012802.1	0.67068	1.30E-15	2.03E-14	sp|O15234|CASC3_HUMAN Protein CASC3 OS=Homo sapiens GN=CASC3 PE=1 SV=2//0
XM_008012808.1	4.3561	2.24E-271	1.88E-268	sp|P20393|NR1D1_HUMAN Nuclear receptor subfamily 1 group D member 1 OS=Homo sapiens GN=NR1D1 PE=1 SV=1//0
XM_008012844.1	#NAME?	4.36E-05	0.0002976	sp|Q14451|GRB7_HUMAN Growth factor receptor-bound protein 7 OS=Homo sapiens GN=GRB7 PE=1 SV=2//3.51708e-42
XM_008012845.1	-0.40049	5.87E-07	4.90E-06	sp|P04626|ERBB2_HUMAN Receptor tyrosine-protein kinase erbB-2 OS=Homo sapiens GN=ERBB2 PE=1 SV=1//0
XM_008012846.1	-0.32683	0.0029461	0.015471	sp|Q9BRT3|MIEN1_HUMAN Migration and invasion enhancer 1 OS=Homo sapiens GN=MIEN1 PE=1 SV=1//3.38883e-64
XM_008012877.1	-1.2619	0.0009518	0.0054502	sp|Q6ZMT1|STAC2_HUMAN SH3 and cysteine-rich domain-containing protein 2 OS=Homo sapiens GN=STAC2 PE=1 SV=1//0
XM_008012882.1	-0.66805	5.63E-24	1.36E-22	sp|P84100|RL19_RAT 60S ribosomal protein L19 OS=Rattus norvegicus GN=Rpl19 PE=1 SV=1//7.18395e-87
XM_008012896.1	-0.31526	7.57E-06	5.67E-05	sp|Q5R5W0|LASP1_PONAB LIM and SH3 domain protein 1 OS=Pongo abelii GN=LASP1 PE=2 SV=1//1.82227e-138
XM_008012898.1	-0.43991	1.65E-08	1.59E-07	sp|P62832|RL23_RAT 60S ribosomal protein L23 OS=Rattus norvegicus GN=Rpl23 PE=2 SV=1//1.7651e-88
XM_008012899.1	-1.7125	0.0035311	0.018199	sp|O14662|STX16_HUMAN Syntaxin-16 OS=Homo sapiens GN=STX16 PE=1 SV=3//5.2705e-165
XM_008012900.1	1.7572	5.27E-57	3.79E-55	sp|Q9NXE8|CWC25_HUMAN Pre-mRNA-splicing factor CWC25 homolog OS=Homo sapiens GN=CWC25 PE=1 SV=1//0
XM_008012904.1	-0.48	5.35E-07	4.48E-06	sp|P40112|PSB3_RAT Proteasome subunit beta type-3 OS=Rattus norvegicus GN=Psmb3 PE=1 SV=1//4.23109e-151
XM_008012912.1	-0.9857	6.08E-12	7.65E-11	sp|P55198|AF17_HUMAN Protein AF-17 OS=Homo sapiens GN=MLLT6 PE=1 SV=2//1.2571e-34
XM_008012931.1	1.6624	8.73E-34	3.10E-32	sp|Q9P227|RHG23_HUMAN Rho GTPase-activating protein 23 OS=Homo sapiens GN=ARHGAP23 PE=1 SV=2//0
XM_008012940.1	0.59486	3.07E-09	3.13E-08	sp|O14512|SOCS7_HUMAN Suppressor of cytokine signaling 7 OS=Homo sapiens GN=SOCS7 PE=1 SV=2//0
XM_008012949.1	0.70374	5.41E-25	1.38E-23	sp|Q14974|IMB1_HUMAN Importin subunit beta-1 OS=Homo sapiens GN=KPNB1 PE=1 SV=2//0
XM_008012963.1	-2.7848	0.0088772	0.042043	sp|Q9BZF2|OSBL7_HUMAN Oxysterol-binding protein-related protein 7

				OS=Homo sapiens GN=OSBPL7 PE=2 SV=1//0
XM_008013005.1	-2.143	1.65E-08	1.59E-07	sp|P09067|HXB5_HUMAN Homeobox protein Hox-B5 OS=Homo sapiens GN=HOXB5 PE=1 SV=3//2.44991e-123
XM_008013016.1	-1.1475	2.81E-11	3.37E-10	sp|Q9TT89|HXB7_BOVIN Homeobox protein Hox-B7 OS=Bos taurus GN=HOXB7 PE=2 SV=1//4.77542e-125
XM_008013017.1	4.1426	1.64E-69	1.64E-67	sp|P17482|HXB9_HUMAN Homeobox protein Hox-B9 OS=Homo sapiens GN=HOXB9 PE=1 SV=2//1.60541e-154
XM_008013023.1	-0.42511	0.0073152	0.035209	sp|Q92826|HXB13_HUMAN Homeobox protein Hox-B13 OS=Homo sapiens GN=HOXB13 PE=1 SV=2//0
XM_008013027.1	1.8714	6.40E-22	1.41E-20	sp|Q8N841|TTLL6_HUMAN Tubulin polyglutamylase TTLL6 OS=Homo sapiens GN=TTLL6 PE=1 SV=2//0
XM_008013035.1	-0.53596	9.52E-10	1.01E-08	sp|Q96H20|SNF8_HUMAN Vacuolar-sorting protein SNF8 OS=Homo sapiens GN=SNF8 PE=1 SV=1//8.5717e-174
XM_008013039.1	1.1823	8.81E-12	1.10E-10	sp|Q9NZI8|IF2B1_HUMAN Insulin-like growth factor 2 mRNA-binding protein 1 OS=Homo sapiens GN=IGF2BP1 PE=1 SV=2//0
XM_008013045.1	-0.97052	0.010377	0.048406	sp|Q9P2A4|ABI3_HUMAN ABI gene family member 3 OS=Homo sapiens GN=ABI3 PE=1 SV=2//0
XM_008013059.1	-0.50551	0.0001814	0.0011466	sp|Q9Y2D9|ZN652_HUMAN Zinc finger protein 652 OS=Homo sapiens GN=ZNF652 PE=1 SV=3//0
XM_008013077.1	0.29382	0.0043599	0.02207	sp|O43760|SNG2_HUMAN Synaptogyrin-2 OS=Homo sapiens GN=SYNGR2 PE=1 SV=1//2.54712e-134
XM_008013080.1	-0.87122	0.0051979	0.025847	sp|Q63HM1|KFA_HUMAN Kynurenine formamidase OS=Homo sapiens GN=AFMID PE=2 SV=2//0
XM_008013083.1	-0.5599	1.76E-05	0.000126	sp|Q5RAH9|BIRC5_PONAB Baculoviral IAP repeat-containing protein 5 OS=Pongo abelii GN=BIRC5 PE=2 SV=1//1.3512e-75
XM_008013093.1	0.71362	5.47E-06	4.16E-05	sp|O14543|SOCS3_HUMAN Suppressor of cytokine signaling 3 OS=Homo sapiens GN=SOCS3 PE=1 SV=1//1.87632e-129
XM_008013121.1	-0.90987	9.30E-31	2.95E-29	sp|P16035|TIMP2_HUMAN Metalloproteinase inhibitor 2 OS=Homo sapiens GN=TIMP2 PE=1 SV=2//3.26496e-94
XM_008013156.1	-1.1859	2.08E-14	3.03E-13	sp|Q14781|CBX2_HUMAN Chromobox protein homolog 2 OS=Homo sapiens GN=CBX2 PE=1 SV=2//0
XM_008013159.1	1.2718	7.29E-29	2.16E-27	sp|O00257|CBX4_HUMAN E3 SUMO-protein ligase CBX4 OS=Homo sapiens GN=CBX4 PE=1 SV=3//0
XM_008013160.1	-1.2023	1.34E-05	9.74E-05	-//-
XM_008013207.1	-0.47619	5.10E-05	0.0003464	sp|Q96FZ7|CHMP6_HUMAN Charged multivesicular body protein 6 OS=Homo sapiens GN=CHMP6 PE=1 SV=3//7.47581e-83
XM_008013224.1	-4.2713	1.84E-11	2.25E-10	sp|Q5R5J1|PCKGC_PONAB Phosphoenolpyruvate carboxykinase, cytosolic [GTP] OS=Pongo abelii GN=PCK1 PE=2 SV=1//0
XM_008013242.1	-0.18024	0.0049381	0.02465	sp|A2BDB0|ACTG_XENLA Actin, cytoplasmic 2 OS=Xenopus laevis GN=actg1 PE=2 SV=1//0
XM_008013251.1	-0.76154	0.0047639	0.023884	sp|Q9H1Z9|TSN10_HUMAN Tetraspanin-10 OS=Homo sapiens GN=TSPAN10 PE=2 SV=1//5.82723e-155
XM_008013254.1	-1.7901	0.0006186	0.0036432	sp|Q4R5Y2|NADE_MACFA Glutamine-dependent NAD(+) synthetase OS=Macaca fascicularis GN=NADSYN1 PE=2 SV=1//0
XM_008013257.1	0.68836	0.0001478	0.0009478	sp|Q0P5N6|ARL16_HUMAN ADP-ribosylation factor-like protein 16 OS=Homo sapiens GN=ARL16 PE=1 SV=1//1.07244e-128
XM_008013258.1	0.26368	0.0036397	0.018706	sp|O14964|HGS_HUMAN Hepatocyte growth factor-regulated tyrosine kinase substrate OS=Homo sapiens GN=HGS PE=1 SV=1//0
XM_008013267.1	-1.3739	0.0001772	0.001123	sp|C9JLW8|F195B_HUMAN Protein FAM195B OS=Homo sapiens GN=FAM195B PE=1 SV=1//3.56049e-50
XM_008013285.1	-0.36519	0.0001929	0.0012149	sp|Q86V81|THOC4_HUMAN THO complex subunit 4 OS=Homo sapiens GN=ALYREF PE=1 SV=3//1.20702e-80
XM_008013291.1	2.1975	1.22E-51	7.84E-50	sp|Q9NRC8|SIR7_HUMAN NAD-dependent protein deacetylase sirtuin-7 OS=Homo sapiens GN=SIRT7 PE=1 SV=1//0
XM_008013299.1	-1.669	2.46E-17	4.26E-16	sp|Q6P988|NOTUM_HUMAN Palmitoleoyl-protein carboxylesterase NOTUM OS=Homo sapiens GN=NOTUM PE=1 SV=2//0
XM_008013318.1	-0.64014	2.38E-06	1.88E-05	sp|Q7Z4W1|DCXR_HUMAN L-xylulose reductase OS=Homo sapiens GN=DCXR PE=1 SV=2//2.89878e-157
XM_008013326.1	-2.4141	0.0019466	0.010564	sp|P48900|NU1M_FELCA NADH-ubiquinone oxidoreductase chain 1 OS=Felis catus GN=MT-ND1 PE=3 SV=1//3.78167e-60
XM_008013339.1	1.2191	1.69E-37	6.77E-36	sp|Q5RF99|RAEL1_PONAB mRNA export factor OS=Pongo abelii GN=RAE1 PE=2 SV=1//0
XM_008013373.1	-1.0069	5.34E-05	0.0003613	sp|Q6PK18|OGFD3_HUMAN 2-oxoglutarate and iron-dependent oxygenase domain-containing protein 3 OS=Homo sapiens GN=OGFOD3 PE=1 SV=2//0
XM_008013376.1	0.81038	1.41E-21	3.03E-20	sp|Q01167|FOXK2_HUMAN Forkhead box protein K2 OS=Homo sapiens GN=FOXK2 PE=1 SV=3//0
XM_008013378.1	-0.81453	0.0047053	0.023655	sp|Q12829|RB40B_HUMAN Ras-related protein Rab-40B OS=Homo sapiens GN=RAB40B PE=2 SV=1//0
XM_008013394.1	1.2229	4.85E-23	1.13E-21	sp|Q641Q3|METRL_HUMAN Meteorin-like protein OS=Homo sapiens GN=METRNL PE=2 SV=1//0

XM_008013401.1	2.0118	1.22E-57	8.91E-56	sp|Q9P0J7|KCMF1_HUMAN E3 ubiquitin-protein ligase KCMF1 OS=Homo sapiens GN=KCMF1 PE=1 SV=2//3.10082e-138
XM_008013411.1	0.72712	6.07E-15	9.14E-14	sp|Q2HJF8|MIRO1_BOVIN Mitochondrial Rho GTPase 1 OS=Bos taurus GN=RHOT1 PE=2 SV=1//3.03789e-13
XM_008013416.1	0.32124	0.0014669	0.0081649	sp|Q96H55|MYO19_HUMAN Unconventional myosin-XIX OS=Homo sapiens GN=MYO19 PE=2 SV=2//0
XM_008013431.1	1.1034	6.11E-37	2.38E-35	sp|Q9HAU4|SMUF2_HUMAN E3 ubiquitin-protein ligase SMURF2 OS=Homo sapiens GN=SMURF2 PE=1 SV=1//0
XM_008013433.1	0.62802	4.25E-16	6.87E-15	sp|Q86XP3|DDX42_HUMAN ATP-dependent RNA helicase DDX42 OS=Homo sapiens GN=DDX42 PE=1 SV=1//0
XM_008013442.1	0.48809	1.38E-06	1.12E-05	sp|Q6J6I9|BRCA1_MACMU Breast cancer type 1 susceptibility protein homolog OS=Macaca mulatta GN=BRCA1 PE=3 SV=1//1.27016e-163
XM_008013444.1	1.7739	2.86E-27	7.95E-26	sp|Q96NG3|TTC25_HUMAN Tetratricopeptide repeat protein 25 OS=Homo sapiens GN=TTC25 PE=1 SV=2//0
XM_008013445.1	-0.6094	3.00E-18	5.46E-17	sp|P53396|ACLY_HUMAN ATP-citrate synthase OS=Homo sapiens GN=ACLY PE=1 SV=3//0
XM_008013452.1	0.78628	5.33E-13	7.12E-12	sp|Q8TF74|WIPF2_HUMAN WAS/WASL-interacting protein family member 2 OS=Homo sapiens GN=WIPF2 PE=1 SV=1//1.03326e-162
XM_008013454.1	1.1859	2.59E-53	1.70E-51	sp|Q15648|MED1_HUMAN Mediator of RNA polymerase II transcription subunit 1 OS=Homo sapiens GN=MED1 PE=1 SV=4//2.51624e-154
XM_008013455.1	1.2269	0.0007903	0.004579	sp|A6NH57|ARL5C_HUMAN Putative ADP-ribosylation factor-like protein 5C OS=Homo sapiens GN=ARL5C PE=3 SV=4//5.53017e-08
XM_008013459.1	1.5628	4.05E-22	8.98E-21	sp|Q86WA9|S2611_HUMAN Sodium-independent sulfate anion transporter OS=Homo sapiens GN=SLC26A11 PE=2 SV=2//9.35119e-26
XM_008013463.1	0.60786	1.32E-06	1.07E-05	sp|Q6PK04|CC137_HUMAN Coiled-coil domain-containing protein 137 OS=Homo sapiens GN=CCDC137 PE=1 SV=1//3.7335e-131
XM_008013466.1	-0.8474	3.06E-06	2.39E-05	sp|Q9ER35|FN3K_MOUSE Fructosamine-3-kinase OS=Mus musculus GN=Fn3k PE=2 SV=1//1.99895e-139
XM_008013469.1	-0.64777	6.17E-16	9.86E-15	sp|Q9NV96|CC50A_HUMAN Cell cycle control protein 50A OS=Homo sapiens GN=TMEM30A PE=1 SV=1//0
XM_008013480.1	-1.561	2.47E-09	2.53E-08	sp|Q9NRA2|S17A5_HUMAN Sialin OS=Homo sapiens GN=SLC17A5 PE=1 SV=2//0
XM_008013563.1	-1.1368	8.28E-37	3.22E-35	sp|Q96KR6|F210B_HUMAN Protein FAM210B OS=Homo sapiens GN=FAM210B PE=1 SV=2//3.15706e-60
XM_008013601.1	-0.39587	0.0003441	0.0020998	sp|Q4R5E3|LMBD1_MACFA Probable lysosomal cobalamin transporter OS=Macaca fascicularis GN=LMBRD1 PE=2 SV=1//0
XM_008013608.1	-1.6517	2.99E-05	0.0002078	sp|Q5T1H1|EYS_HUMAN Protein eyes shut homolog OS=Homo sapiens GN=EYS PE=1 SV=5//0
XM_008013614.1	1.6851	1.26E-35	4.74E-34	sp|Q78EG7|TP4A1_RAT Protein tyrosine phosphatase type IVA 1 OS=Rattus norvegicus GN=Ptp4a1 PE=1 SV=1//1.24168e-114
XM_008013615.1	5.0494	0.0024313	0.012982	sp|Q78EG7|TP4A1_RAT Protein tyrosine phosphatase type IVA 1 OS=Rattus norvegicus GN=Ptp4a1 PE=1 SV=1//2.03049e-119
XM_008013618.1	0.81677	6.60E-16	1.05E-14	sp|Q9NQP4|PFD4_HUMAN Prefoldin subunit 4 OS=Homo sapiens GN=PFDN4 PE=1 SV=1//1.30775e-65
XM_008013626.1	1.0918	1.13E-10	1.29E-09	sp|P49643|PRI2_HUMAN DNA primase large subunit OS=Homo sapiens GN=PRIM2 PE=1 SV=2//0
XM_008013635.1	1.4654	6.27E-21	1.31E-19	sp|O95816|BAG2_HUMAN BAG family molecular chaperone regulator 2 OS=Homo sapiens GN=BAG2 PE=1 SV=1//1.99671e-145
XM_008013645.1	1.3365	4.73E-09	4.73E-08	sp|Q9Y4E5|ZN451_HUMAN Zinc finger protein 451 OS=Homo sapiens GN=ZNF451 PE=1 SV=2//3.3151e-28
XM_008013646.1	1.1837	1.92E-15	2.96E-14	sp|Q9HCI6|K1586_HUMAN Uncharacterized protein KIAA1586 OS=Homo sapiens GN=KIAA1586 PE=2 SV=2//0
XM_008013647.1	-0.59562	2.25E-05	0.0001589	sp|Q03001|DYST_HUMAN Dystonin OS=Homo sapiens GN=DST PE=1 SV=4//0
XM_008013686.1	1.1773	4.40E-09	4.42E-08	sp|Q9H8W2|CF155_HUMAN Putative uncharacterized protein encoded by LINC00472 OS=Homo sapiens GN=LINC00472 PE=5 SV=2//6.42329e-08
XM_008013688.1	-1.2795	1.78E-09	1.85E-08	sp|Q9BYG4|PAR6G_HUMAN Partitioning defective 6 homolog gamma OS=Homo sapiens GN=PARD6G PE=1 SV=1//0
XM_008013701.1	-1.1993	5.15E-14	7.35E-13	sp|C9JCN9|HSBPL_HUMAN Heat shock factor-binding protein 1-like protein 1 OS=Homo sapiens GN=HSBP1L1 PE=3 SV=2//3.60871e-42
XM_008013757.1	-0.58613	6.56E-05	0.00044	sp|Q8N4Q0|ZADH2_HUMAN Zinc-binding alcohol dehydrogenase domain-containing protein 2 OS=Homo sapiens GN=ZADH2 PE=1 SV=1//0
XM_008013765.1	0.88166	0.0074393	0.035782	sp|Q9C0G0|ZN407_HUMAN Zinc finger protein 407 OS=Homo sapiens GN=ZNF407 PE=1 SV=2//0
XM_008013780.1	-0.95975	4.17E-14	5.98E-13	sp|P00167|CYB5_HUMAN Cytochrome b5 OS=Homo sapiens GN=CYB5A PE=1 SV=2//4.97149e-89
XM_008013818.1	-1.4414	2.43E-09	2.49E-08	sp|Q6PKX4|DOK6_HUMAN Docking protein 6 OS=Homo sapiens GN=DOK6 PE=1 SV=1//0
XM_008013864.1	0.74878	2.03E-13	2.80E-12	sp|O75351|VPS4B_HUMAN Vacuolar protein sorting-associated protein 4B OS=Homo sapiens GN=VPS4B PE=1 SV=2//0
XM_008013868.1	2.5447	1.25E-143	3.85E-141	sp|O60346|PHLP1_HUMAN PH domain leucine-rich repeat-containing protein phosphatase 1 OS=Homo sapiens GN=PHLPP1 PE=1 SV=3//5.15334e-32

XM_008013869.1	1.5457	1.11E-25	2.93E-24	sp|Q9C0B9|ZCHC2_HUMAN Zinc finger CCHC domain-containing protein 2 OS=Homo sapiens GN=ZCCHC2 PE=1 SV=6//0
XM_008013870.1	-1.0596	0.0047406	0.023797	sp|P62914|RL11_RAT 60S ribosomal protein L11 OS=Rattus norvegicus GN=Rpl11 PE=1 SV=2//2.07477e-124
XM_008013871.1	-1.3948	0.0006175	0.0036377	sp|Q9Y6Q6|TNR11_HUMAN Tumor necrosis factor receptor superfamily member 11A OS=Homo sapiens GN=TNFRSF11A PE=1 SV=1//0
XM_008013902.1	3.6944	3.56E-08	3.31E-07	sp|Q13794|APR_HUMAN Phorbol-12-myristate-13-acetate-induced protein 1 OS=Homo sapiens GN=PMAIP1 PE=1 SV=1//1.672e-14
XM_008013903.1	5.2292	7.48E-279	6.56E-276	sp|Q13794|APR_HUMAN Phorbol-12-myristate-13-acetate-induced protein 1 OS=Homo sapiens GN=PMAIP1 PE=1 SV=1//9.88843e-26
XM_008013913.1	-0.74399	1.95E-11	2.37E-10	sp|Q9TU32|LMAN1_CHLAE Protein ERGIC-53 OS=Chlorocebus aethiops GN=LMAN1 PE=2 SV=1//0
XM_008013927.1	1.5735	1.56E-25	4.07E-24	sp|Q5RC30|SC11C_PONAB Signal peptidase complex catalytic subunit SEC11C OS=Pongo abelii GN=SEC11C PE=2 SV=3//1.4951e-135
XM_008013965.1	1.3508	0.0004832	0.0028876	sp|Q96PU5|NED4L_HUMAN E3 ubiquitin-protein ligase NEDD4-like OS=Homo sapiens GN=NEDD4L PE=1 SV=2//0
XM_008013980.1	0.92058	5.20E-31	1.66E-29	sp|Q4R4Z1|SYNC_MACFA Asparagine--tRNA ligase, cytoplasmic OS=Macaca fascicularis GN=NARS PE=2 SV=1//0
XM_008014031.1	2.1137	1.49E-09	1.55E-08	sp|Q86WA9|S2611_HUMAN Sodium-independent sulfate anion transporter OS=Homo sapiens GN=SLC26A11 PE=2 SV=2//0
XM_008014047.1	-3.7494	7.80E-06	5.83E-05	sp|P61645|SIA8D_PANTR CMP-N-acetylneuraminate-poly-alpha-2,8-sialyltransferase OS=Pan troglodytes GN=ST8SIA4 PE=2 SV=1//0
XM_008014048.1	-1.0603	0.0030937	0.016184	sp|Q6ZQN7|SO4C1_HUMAN Solute carrier organic anion transporter family member 4C1 OS=Homo sapiens GN=SLCO4C1 PE=1 SV=1//0
XM_008014079.1	1.8153	8.03E-20	1.58E-18	sp|Q4R6I1|GIN1_MACFA Gypsy retrotransposon integrase-like protein 1 OS=Macaca fascicularis GN=GIN1 PE=2 SV=1//0
XM_008014080.1	1.2502	2.44E-05	0.0001717	sp|Q96GV9|CE030_HUMAN UNC119-binding protein C5orf30 OS=Homo sapiens GN=C5orf30 PE=1 SV=1//3.35537e-139
XM_008014082.1	1.3483	8.56E-23	1.97E-21	sp|O95396|MOCS3_HUMAN Adenylyltransferase and sulfurtransferase MOCS3 OS=Homo sapiens GN=MOCS3 PE=1 SV=1//0
XM_008014090.1	0.45133	6.72E-06	5.06E-05	sp|O60762|DPM1_HUMAN Dolichol-phosphate mannosyltransferase subunit 1 OS=Homo sapiens GN=DPM1 PE=1 SV=1//2.42824e-177
XM_008014114.1	0.4435	2.66E-07	2.28E-06	sp|Q96AG3|S2546_HUMAN Solute carrier family 25 member 46 OS=Homo sapiens GN=SLC25A46 PE=1 SV=1//0
XM_008014117.1	1.3811	6.80E-41	3.03E-39	sp|Q8NI36|WDR36_HUMAN WD repeat-containing protein 36 OS=Homo sapiens GN=WDR36 PE=1 SV=1//0
XM_008014121.1	-0.67327	2.35E-09	2.41E-08	sp|Q96DR4|STAR4_HUMAN StAR-related lipid transfer protein 4 OS=Homo sapiens GN=STARD4 PE=2 SV=1//6.42851e-151
XM_008014149.1	1.666	2.61E-09	2.67E-08	sp|Q15696|U2AFM_HUMAN U2 small nuclear ribonucleoprotein auxiliary factor 35 kDa subunit-related protein 2 OS=Homo sapiens GN=ZRSR2 PE=1 SV=2//3.78867e-09
XM_008014150.1	-0.33441	0.0001366	0.0008793	sp|Q00765|REEP5_HUMAN Receptor expression-enhancing protein 5 OS=Homo sapiens GN=REEP5 PE=1 SV=3//2.08356e-127
XM_008014176.1	0.67618	1.64E-05	0.0001175	sp|P53609|PGTB1_HUMAN Geranylgeranyl transferase type-1 subunit beta OS=Homo sapiens GN=PGGT1B PE=1 SV=2//0
XM_008014182.1	1.202	2.79E-32	9.27E-31	sp|Q96JP0|FEM1C_HUMAN Protein fem-1 homolog C OS=Homo sapiens GN=FEM1C PE=1 SV=1//0
XM_008014185.1	2.4442	2.17E-10	2.43E-09	sp|Q5RBQ7|CDO1_PONAB Cysteine dioxygenase type 1 OS=Pongo abelii GN=CDO1 PE=2 SV=1//4.33967e-143
XM_008014186.1	1.6551	1.17E-10	1.33E-09	sp|Q5R7W1|ATG12_PONAB Ubiquitin-like protein ATG12 OS=Pongo abelii GN=ATG12 PE=2 SV=1//3.65785e-70
XM_008014202.1	-0.66787	1.10E-12	1.44E-11	sp|Q9H2E6|SEM6A_HUMAN Semaphorin-6A OS=Homo sapiens GN=SEMA6A PE=1 SV=2//0
XM_008014233.1	1.9467	6.91E-46	3.73E-44	sp|Q8NEF9|SRFB1_HUMAN Serum response factor-binding protein 1 OS=Homo sapiens GN=SRFBP1 PE=1 SV=1//0
XM_008014238.1	0.85776	4.93E-33	1.68E-31	sp|P18031|PTN1_HUMAN Tyrosine-protein phosphatase non-receptor type 1 OS=Homo sapiens GN=PTPN1 PE=1 SV=1//0
XM_008014250.1	0.90955	0.0060254	0.029553	sp|Q9Y343|SNX24_HUMAN Sorting nexin-24 OS=Homo sapiens GN=SNX24 PE=1 SV=1//1.40923e-112
XM_008014253.1	-1.7022	2.72E-35	1.02E-33	sp|P45877|PPIC_HUMAN Peptidyl-prolyl cis-trans isomerase C OS=Homo sapiens GN=PPIC PE=1 SV=1//1.3921e-119
XM_008014278.1	1.9762	3.81E-115	9.07E-113	sp|P17676|CEBPB_HUMAN CCAAT/enhancer-binding protein beta OS=Homo sapiens GN=CEBPB PE=1 SV=2//5.03836e-133
XM_008014289.1	-0.37434	2.77E-06	2.17E-05	sp|P20700|LMNB1_HUMAN Lamin-B1 OS=Homo sapiens GN=LMNB1 PE=1 SV=2//0
XM_008014295.1	0.99066	0.0048991	0.024483	sp|Q9CWB7|YD286_MOUSE Glutaredoxin-like protein C5orf63 homolog OS=Mus musculus PE=1 SV=1//2.42924e-53
XM_008014309.1	-0.68541	0.0001135	0.0007396	sp|P35556|FBN2_HUMAN Fibrillin-2 OS=Homo sapiens GN=FBN2 PE=1 SV=3//0
XM_008014314.1	-1.1617	0.010096	0.047246	sp|Q70JA7|CHSS3_HUMAN Chondroitin sulfate synthase 3 OS=Homo sapiens GN=CHSY3 PE=2 SV=3//0
XM_008014321.1	-0.31234	0.0006863	0.0040117	sp|P49773|HINT1_HUMAN Histidine triad nucleotide-binding protein 1

				OS=Homo sapiens GN=HINT1 PE=1 SV=2//1.22775e-86
XM_008014362.1	1.1126	7.33E-09	7.22E-08	sp|Q92878|RAD50_HUMAN DNA repair protein RAD50 OS=Homo sapiens GN=RAD50 PE=1 SV=1//0
XM_008014364.1	0.95486	2.79E-28	8.06E-27	sp|Q9Y508|RN114_HUMAN E3 ubiquitin-protein ligase RNF114 OS=Homo sapiens GN=RNF114 PE=1 SV=1//1.36446e-145
XM_008014372.1	0.80732	1.63E-05	0.0001174	sp|O76082|S22A5_HUMAN Solute carrier family 22 member 5 OS=Homo sapiens GN=SLC22A5 PE=1 SV=1//0
XM_008014415.1	-0.7558	1.18E-16	1.98E-15	sp|Q5R5B8|KCT2_PONAB Keratinocyte-associated transmembrane protein 2 OS=Pongo abelii GN=KCT2 PE=2 SV=1//4.28408e-160
XM_008014437.1	-0.8528	8.43E-21	1.74E-19	sp|Q71U00|SKP1_XENLA S-phase kinase-associated protein 1 OS=Xenopus laevis GN=skp1 PE=1 SV=3//6.6513e-84
XM_008014438.1	0.7815	6.81E-26	1.80E-24	sp|P67777|PP2AA_RABIT Serine/threonine-protein phosphatase 2A catalytic subunit alpha isoform OS=Oryctolagus cuniculus GN=PPP2CA PE=2 SV=1//0
XM_008014462.1	0.26738	0.0022765	0.0122	sp|O43286|B4GT5_HUMAN Beta-1,4-galactosyltransferase 5 OS=Homo sapiens GN=B4GALT5 PE=2 SV=1//0
XM_008014469.1	1.0141	9.67E-23	2.22E-21	sp|O95486|SC24A_HUMAN Protein transport protein Sec24A OS=Homo sapiens GN=SEC24A PE=1 SV=2//4.05196e-172
XM_008014483.1	-2.6961	3.95E-06	3.06E-05	sp|Q86XQ3|CTSR3_HUMAN Cation channel sperm-associated protein 3 OS=Homo sapiens GN=CATSPER3 PE=1 SV=1//0
XM_008014525.1	1.5717	2.32E-55	1.61E-53	sp|Q96GQ7|DDX27_HUMAN Probable ATP-dependent RNA helicase DDX27 OS=Homo sapiens GN=DDX27 PE=1 SV=2//0
XM_008014532.1	-3.1798	5.76E-06	4.38E-05	sp|Q6ZT89|S2548_HUMAN Solute carrier family 25 member 48 OS=Homo sapiens GN=SLC25A48 PE=1 SV=2//0
XM_008014538.1	0.69472	8.89E-16	1.41E-14	sp|Q5R6H7|SMAD5_PONAB Mothers against decapentaplegic homolog 5 OS=Pongo abelii GN=SMAD5 PE=2 SV=1//0
XM_008014543.1	1.9115	1.40E-33	4.91E-32	sp|Q9HCX4|TRPC7_HUMAN Short transient receptor potential channel 7 OS=Homo sapiens GN=TRPC7 PE=1 SV=1//0
XM_008014558.1	2.482	1.61E-06	1.30E-05	sp|O43300|LRRT2_HUMAN Leucine-rich repeat transmembrane neuronal protein 2 OS=Homo sapiens GN=LRRTM2 PE=2 SV=3//0
XM_008014562.1	1.8234	5.92E-74	6.52E-72	sp|Q3ZCH0|GRP75_BOVIN Stress-70 protein, mitochondrial OS=Bos taurus GN=HSPA9 PE=2 SV=1//0
XM_008014563.1	1.1961	8.02E-64	7.11E-62	sp|Q5U2Q7|ERF1_RAT Eukaryotic peptide chain release factor subunit 1 OS=Rattus norvegicus GN=Etf1 PE=2 SV=3//0
XM_008014564.1	7.6943	1.67E-94	2.79E-92	sp|P18146|EGR1_HUMAN Early growth response protein 1 OS=Homo sapiens GN=EGR1 PE=1 SV=1//0
XM_008014582.1	-0.36015	8.51E-06	6.33E-05	sp|O95235|KI20A_HUMAN Kinesin-like protein KIF20A OS=Homo sapiens GN=KIF20A PE=1 SV=1//0
XM_008014624.1	-0.88769	5.04E-23	1.17E-21	sp|Q9BPZ3|PAIP2_HUMAN Polyadenylate-binding protein-interacting protein 2 OS=Homo sapiens GN=PAIP2 PE=1 SV=1//5.69572e-50
XM_008014627.1	-0.72144	0.0056364	0.027813	sp|E7EW31|PROB1_HUMAN Proline-rich basic protein 1 OS=Homo sapiens GN=PROB1 PE=2 SV=2//0
XM_008014636.1	-1.1749	0.0005698	0.0033773	sp|Q86WV6|STING_HUMAN Stimulator of interferon genes protein OS=Homo sapiens GN=TMEM173 PE=1 SV=1//0
XM_008014656.1	-1.9356	1.43E-18	2.64E-17	sp|A6NJ69|IGIP_HUMAN IgA-inducing protein homolog OS=Homo sapiens GN=IGIP PE=3 SV=1//5.29136e-26
XM_008014669.1	4.8874	9.06E-308	9.93E-305	sp|Q09118|HBEGF_CHLAE Proheparin-binding EGF-like growth factor OS=Chlorocebus aethiops GN=HBEGF PE=1 SV=1//3.88941e-95
XM_008014693.1	-0.42467	8.79E-08	7.90E-07	sp|Q66HG8|RED_RAT Protein Red OS=Rattus norvegicus GN=Ik PE=1 SV=1//0
XM_008014694.1	-1.2198	1.36E-16	2.25E-15	sp|Q4R5E2|NDUA2_MACFA NADH dehydrogenase [ubiquinone] 1 alpha subcomplex subunit 2 OS=Macaca fascicularis GN=NDUFA2 PE=3 SV=3//5.94116e-62
XM_008014704.1	-0.5866	8.68E-09	8.54E-08	sp|Q9CPW7|ZMAT2_MOUSE Zinc finger matrin-type protein 2 OS=Mus musculus GN=Zmat2 PE=2 SV=1//1.71339e-111
XM_008014707.1	0.3204	0.0009866	0.005642	sp|P12081|SYHC_HUMAN Histidine--tRNA ligase, cytoplasmic OS=Homo sapiens GN=HARS PE=1 SV=2//0
XM_008014729.1	0.49872	3.50E-10	3.87E-09	sp|Q5R7L9|TAF7_PONAB Transcription initiation factor TFIID subunit 7 OS=Pongo abelii GN=TAF7 PE=2 SV=1//0
XM_008014733.1	-2.7573	0.0001144	0.0007446	sp|Q5DRB6|PCDG4_PANTR Protocadherin gamma-A4 OS=Pan troglodytes GN=PCDHGA4 PE=3 SV=1//0
XM_008014784.1	-0.6727	3.16E-13	4.30E-12	sp|Q9BT67|NFIP1_HUMAN NEDD4 family-interacting protein 1 OS=Homo sapiens GN=NDFIP1 PE=1 SV=1//1.52675e-116
XM_008014922.1	3.9953	3.70E-27	1.02E-25	sp|Q28509|ADRB2_MACMU Beta-2 adrenergic receptor OS=Macaca mulatta GN=ADRB2 PE=2 SV=1//0
XM_008014923.1	0.96283	1.77E-07	1.55E-06	sp|Q8TF17|S3TC2_HUMAN SH3 domain and tetratricopeptide repeat-containing protein 2 OS=Homo sapiens GN=SH3TC2 PE=1 SV=2//0
XM_008014933.1	1.4383	1.94E-08	1.86E-07	sp|Q8TAA5|GRPE2_HUMAN GrpE protein homolog 2, mitochondrial OS=Homo sapiens GN=GRPEL2 PE=1 SV=1//2.08672e-153
XM_008014956.1	-0.4242	0.0088313	0.041853	sp|P16499|PDE6A_HUMAN Rod cGMP-specific 3',5'-cyclic phosphodiesterase subunit alpha OS=Homo sapiens GN=PDE6A PE=1 SV=4//0
XM_008014957.1	-1.2583	9.86E-62	8.18E-60	sp|P50443|S26A2_HUMAN Sulfate transporter OS=Homo sapiens GN=SLC26A2

				PE=1 SV=2//0
XM_008014971.1	-3.69	7.40E-06	5.56E-05	sp|P47902|CDX1_HUMAN Homeobox protein CDX-1 OS=Homo sapiens GN=CDX1 PE=1 SV=2//1.96886e-85
XM_008014981.1	1.6698	2.59E-29	7.78E-28	sp|Q5FYB1|ARSI_HUMAN Arylsulfatase I OS=Homo sapiens GN=ARSI PE=1 SV=1//0
XM_008015007.1	1.1806	2.91E-43	1.39E-41	sp|P52848|NDST1_HUMAN Bifunctional heparan sulfate N-deacetylase/N-sulfotransferase 1 OS=Homo sapiens GN=NDST1 PE=1 SV=1//0
XM_008015013.1	1.2713	1.05E-53	6.99E-52	sp|Q8N3V7|SYNPO_HUMAN Synaptopodin OS=Homo sapiens GN=SYNPO PE=1 SV=2//0
XM_008015014.1	2.8587	0.0002385	0.0014837	sp|Q8N3V7|SYNPO_HUMAN Synaptopodin OS=Homo sapiens GN=SYNPO PE=1 SV=2//0
XM_008015020.1	0.88563	3.33E-22	7.44E-21	sp|Q8BHS3|RBM22_MOUSE Pre-mRNA-splicing factor RBM22 OS=Mus musculus GN=Rbm22 PE=1 SV=1//0
XM_008015039.1	-0.65445	1.12E-10	1.28E-09	sp|P08133|ANXA6_HUMAN Annexin A6 OS=Homo sapiens GN=ANXA6 PE=1 SV=3//0
XM_008015041.1	-2.1274	7.24E-18	1.29E-16	sp|A6NI79|CCD69_HUMAN Coiled-coil domain-containing protein 69 OS=Homo sapiens GN=CCDC69 PE=1 SV=1//1.38314e-147
XM_008015051.1	-2.7549	0.001814	0.009911	sp|Q9NYQ8|FAT2_HUMAN Protocadherin Fat 2 OS=Homo sapiens GN=FAT2 PE=1 SV=2//0
XM_008015060.1	-0.98027	9.29E-50	5.72E-48	sp|Q5R767|SPRC_PONAB SPARC OS=Pongo abelii GN=SPARC PE=2 SV=1//0
XM_008015061.1	1.6922	0.0001237	0.0008017	-//-
XM_008015089.1	-0.84543	2.24E-07	1.94E-06	sp|Q9HAJ7|SP30L_HUMAN Histone deacetylase complex subunit SAP30L OS=Homo sapiens GN=SAP30L PE=1 SV=1//1.25383e-109
XM_008015096.1	0.63121	3.72E-13	5.02E-12	sp|Q6PKG0|LARP1_HUMAN La-related protein 1 OS=Homo sapiens GN=LARP1 PE=1 SV=2//0
XM_008015132.1	-0.23696	0.001176	0.0066574	sp|O46598|HAVR1_CHLAE Hepatitis A virus cellular receptor 1 OS=Chlorocebus aethiops GN=HAVCR1 PE=1 SV=2//5.58668e-81
XM_008015135.1	0.62553	9.60E-05	0.0006322	sp|O43513|MED7_HUMAN Mediator of RNA polymerase II transcription subunit 7 OS=Homo sapiens GN=MED7 PE=1 SV=1//8.00106e-144
XM_008015144.1	1.1477	8.88E-12	1.10E-10	sp|Q96S44|PRPK_HUMAN TP53-regulating kinase OS=Homo sapiens GN=TP53RK PE=1 SV=2//2.38577e-161
XM_008015145.1	-1.911	2.29E-07	1.98E-06	sp|Q9H013|ADA19_HUMAN Disintegrin and metalloproteinase domain-containing protein 19 OS=Homo sapiens GN=ADAM19 PE=1 SV=3//0
XM_008015154.1	-0.74629	0.0063054	0.030795	sp|P83369|LSM11_HUMAN U7 snRNA-associated Sm-like protein LSm11 OS=Homo sapiens GN=LSM11 PE=1 SV=2//5.69987e-132
XM_008015168.1	1.5386	8.15E-07	6.74E-06	sp|Q96MT1|RN145_HUMAN RING finger protein 145 OS=Homo sapiens GN=RNF145 PE=2 SV=2//0
XM_008015173.1	0.86248	1.33E-15	2.07E-14	sp|Q8WVY7|UBCP1_HUMAN Ubiquitin-like domain-containing CTD phosphatase 1 OS=Homo sapiens GN=UBLCP1 PE=1 SV=2//0
XM_008015187.1	3.4966	1.36E-18	2.53E-17	sp|P35368|ADA1B_HUMAN Alpha-1B adrenergic receptor OS=Homo sapiens GN=ADRA1B PE=1 SV=3//0
XM_008015213.1	#NAME?	0.0013187	0.007402	sp|Q4R534|GBRA1_MACFA Gamma-aminobutyric acid receptor subunit alpha-1 OS=Macaca fascicularis GN=GABRA1 PE=2 SV=1//0
XM_008015219.1	0.84435	0.0009332	0.0053505	sp|Q16445|GBRA6_HUMAN Gamma-aminobutyric acid receptor subunit alpha-6 OS=Homo sapiens GN=GABRA6 PE=2 SV=2//0
XM_008015223.1	-0.46398	5.53E-09	5.51E-08	sp|P51959|CCNG1_HUMAN Cyclin-G1 OS=Homo sapiens GN=CCNG1 PE=1 SV=2//0
XM_008015256.1	0.29934	0.0005898	0.0034836	sp|P54136|SYRC_HUMAN Arginine--tRNA ligase, cytoplasmic OS=Homo sapiens GN=RARS PE=1 SV=2//0
XM_008015257.1	-0.28163	0.00068	0.0039757	sp|Q9H999|PANK3_HUMAN Pantothenate kinase 3 OS=Homo sapiens GN=PANK3 PE=1 SV=1//0
XM_008015258.1	-1.7717	2.44E-44	1.22E-42	sp|O75094|SLIT3_HUMAN Slit homolog 3 protein OS=Homo sapiens GN=SLIT3 PE=2 SV=3//0
XM_008015284.1	1.5619	2.53E-05	0.0001777	sp|Q9H2T7|RBP17_HUMAN Ran-binding protein 17 OS=Homo sapiens GN=RANBP17 PE=2 SV=1//0
XM_008015287.1	0.54598	4.84E-11	5.69E-10	sp|P06748|NPM_HUMAN Nucleophosmin OS=Homo sapiens GN=NPM1 PE=1 SV=2//1.48931e-169
XM_008015289.1	1.1392	2.46E-13	3.39E-12	sp|O76093|FGF18_HUMAN Fibroblast growth factor 18 OS=Homo sapiens GN=FGF18 PE=1 SV=1//3.15907e-117
XM_008015291.1	-2.0252	0.0098281	0.046128	sp|Q06732|ZN33B_HUMAN Zinc finger protein 33B OS=Homo sapiens GN=ZNF33B PE=1 SV=2//1.2826e-41
XM_008015300.1	-0.44195	0.0003177	0.0019418	sp|Q8WUN7|UBTD2_HUMAN Ubiquitin domain-containing protein 2 OS=Homo sapiens GN=UBTD2 PE=1 SV=2//2.30813e-164
XM_008015303.1	-1.3035	4.36E-30	1.35E-28	sp|A8MQ27|NEU1B_HUMAN E3 ubiquitin-protein ligase NEURL1B OS=Homo sapiens GN=NEURL1B PE=1 SV=1//0
XM_008015304.1	7.3693	0	0	sp|P28562|DUS1_HUMAN Dual specificity protein phosphatase 1 OS=Homo sapiens GN=DUSP1 PE=1 SV=3//0
XM_008015307.1	-0.28847	0.0058897	0.028939	sp|Q969X5|ERGI1_HUMAN Endoplasmic reticulum-Golgi intermediate compartment protein 1 OS=Homo sapiens GN=ERGIC1 PE=1 SV=1//0
XM_008015308.1	-0.47933	0.0008848	0.0050915	sp|Q9UNX3|RL26L_HUMAN 60S ribosomal protein L26-like 1 OS=Homo sapiens GN=RPL26L1 PE=1 SV=1//3.29968e-82

XM_008015309.1	-0.52492	6.07E-08	5.53E-07	sp|Q5RAV0|VA0E1_PONAB V-type proton ATPase subunit e 1 OS=Pongo abelii GN=ATP6V0E1 PE=3 SV=3//6.55776e-48
XM_008015319.1	1.2084	3.02E-11	3.62E-10	sp|O97561|STC2_MACNE Stanniocalcin-2 OS=Macaca nemestrina GN=STC2 PE=2 SV=1//0
XM_008015357.1	0.4518	3.37E-05	0.0002333	sp|Q96J01|THOC3_HUMAN THO complex subunit 3 OS=Homo sapiens GN=THOC3 PE=1 SV=1//0
XM_008015369.1	-1.2509	3.30E-22	7.39E-21	sp|Q9BW72|HIG2A_HUMAN HIG1 domain family member 2A, mitochondrial OS=Homo sapiens GN=HIGD2A PE=1 SV=1//1.56289e-54
XM_008015370.1	0.63657	0.0004049	0.0024495	sp|Q9Y3C1|NOP16_HUMAN Nucleolar protein 16 OS=Homo sapiens GN=NOP16 PE=1 SV=2//5.40385e-108
XM_008015388.1	-1.1108	0.0083268	0.039694	sp|Q96FV3|TSN17_HUMAN Tetraspanin-17 OS=Homo sapiens GN=TSPAN17 PE=2 SV=2//0
XM_008015421.1	-0.29993	0.0004298	0.0025897	sp|Q12907|LMAN2_HUMAN Vesicular integral-membrane protein VIP36 OS=Homo sapiens GN=LMAN2 PE=1 SV=1//0
XM_008015439.1	-0.63004	1.70E-09	1.78E-08	sp|Q9UJV9|DDX41_HUMAN Probable ATP-dependent RNA helicase DDX41 OS=Homo sapiens GN=DDX41 PE=1 SV=2//0
XM_008015449.1	-2.0133	7.35E-06	5.52E-05	sp|Q9NR12|PDLI7_HUMAN PDZ and LIM domain protein 7 OS=Homo sapiens GN=PDLIM7 PE=1 SV=1//1.73242e-116
XM_008015471.1	-1.0073	4.31E-16	6.95E-15	sp|Q9NX24|NHP2_HUMAN H/ACA ribonucleoprotein complex subunit 2 OS=Homo sapiens GN=NHP2 PE=1 SV=1//4.89757e-103
XM_008015485.1	0.4028	3.42E-05	0.000236	sp|Q99729|ROAA_HUMAN Heterogeneous nuclear ribonucleoprotein A/B OS=Homo sapiens GN=HNRNPAB PE=1 SV=2//8.48234e-07
XM_008015486.1	0.28413	0.0029248	0.015375	sp|Q99729|ROAA_HUMAN Heterogeneous nuclear ribonucleoprotein A/B OS=Homo sapiens GN=HNRNPAB PE=1 SV=2//6.2365e-18
XM_008015492.1	0.69176	1.19E-15	1.86E-14	sp|Q9HCD5|NCOA5_HUMAN Nuclear receptor coactivator 5 OS=Homo sapiens GN=NCOA5 PE=1 SV=2//0
XM_008015515.1	0.21576	0.001066	0.0060613	sp|P31943|HNRH1_HUMAN Heterogeneous nuclear ribonucleoprotein H OS=Homo sapiens GN=HNRNPH1 PE=1 SV=4//0
XM_008015532.1	-1.3049	8.55E-60	6.62E-58	sp|Q9UQ53|MGT4B_HUMAN Alpha-1,3-mannosyl-glycoprotein 4-beta-N-acetylglucosaminyltransferase B OS=Homo sapiens GN=MGAT4B PE=1 SV=1//0
XM_008015549.1	0.69299	9.56E-06	7.06E-05	sp|Q9ULM6|CNOT6_HUMAN CCR4-NOT transcription complex subunit 6 OS=Homo sapiens GN=CNOT6 PE=1 SV=2//0
XM_008015584.1	-3.0312	4.32E-06	3.32E-05	sp|Q9C029|TRIM7_HUMAN Tripartite motif-containing protein 7 OS=Homo sapiens GN=TRIM7 PE=1 SV=2//0
XM_008015592.1	-0.35305	7.11E-08	6.45E-07	sp|P63245|GBLP_RAT Guanine nucleotide-binding protein subunit beta-2-like 1 OS=Rattus norvegicus GN=Gnb2l1 PE=1 SV=3//0
XM_008015595.1	0.81845	0.0079633	0.038094	sp|Q96A61|TRI52_HUMAN Tripartite motif-containing protein 52 OS=Homo sapiens GN=TRIM52 PE=1 SV=1//7.74706e-121
XM_008015600.1	-0.73012	8.83E-08	7.92E-07	-//-
XM_008015603.1	-1.0119	2.53E-30	7.91E-29	sp|P49419|AL7A1_HUMAN Alpha-aminoadipic semialdehyde dehydrogenase OS=Homo sapiens GN=ALDH7A1 PE=1 SV=5//0
XM_008015605.1	0.41932	3.09E-08	2.89E-07	sp|Q5R6D8|DDX46_PONAB Probable ATP-dependent RNA helicase DDX46 OS=Pongo abelii GN=DDX46 PE=2 SV=1//0
XM_008015617.1	-1.0766	5.00E-11	5.87E-10	sp|Q9Y5H3|PCDGA_HUMAN Protocadherin gamma-A10 OS=Homo sapiens GN=PCDHGA10 PE=2 SV=1//1.16134e-58
XM_008015620.1	-1.8248	0.0004674	0.002799	sp|Q8HXX6|SAP3_MACFA Ganglioside GM2 activator OS=Macaca fascicularis GN=GM2A PE=2 SV=2//5.31943e-116
XM_008015627.1	1.8542	3.35E-08	3.13E-07	sp|Q6ZN57|ZFP2_HUMAN Zinc finger protein 2 homolog OS=Homo sapiens GN=ZFP2 PE=1 SV=1//0
XM_008015630.1	-1.723	2.89E-05	0.0002011	-//-
XM_008015631.1	-0.58054	0.0068294	0.033089	sp|O94808|GFPT2_HUMAN Glutamine--fructose-6-phosphate aminotransferase [isomerizing] 2 OS=Homo sapiens GN=GFPT2 PE=1 SV=3//0
XM_008015634.1	4.7996	7.34E-05	0.0004901	sp|Q08379|GOGA2_HUMAN Golgin subfamily A member 2 OS=Homo sapiens GN=GOLGA2 PE=1 SV=3//3.23558e-31
XM_008015659.1	0.99101	8.80E-19	1.65E-17	sp|Q6PCB6|AB17C_HUMAN Alpha/beta hydrolase domain-containing protein 17C OS=Homo sapiens GN=ABHD17C PE=2 SV=2//0
XM_008015665.1	1.9311	3.60E-13	4.86E-12	sp|Q9HBZ2|ARNT2_HUMAN Aryl hydrocarbon receptor nuclear translocator 2 OS=Homo sapiens GN=ARNT2 PE=1 SV=2//0
XM_008015696.1	-4.0524	0.0061708	0.030211	sp|P0C6C1|AN34C_HUMAN Ankyrin repeat domain-containing protein 34C OS=Homo sapiens GN=ANKRD34C PE=3 SV=2//0
XM_008015698.1	-0.80541	9.28E-06	6.87E-05	sp|P09668|CATH_HUMAN Pro-cathepsin H OS=Homo sapiens GN=CTSH PE=1 SV=4//0
XM_008015699.1	-0.25038	0.0011921	0.0067401	sp|P60762|MO4L1_MOUSE Mortality factor 4-like protein 1 OS=Mus musculus GN=Morf4l1 PE=1 SV=2//0
XM_008015704.1	-1.2278	2.17E-06	1.72E-05	sp|Q5IS51|ACHA5_PANTR Neuronal acetylcholine receptor subunit alpha-5 OS=Pan troglodytes GN=CHRNA5 PE=2 SV=1//0
XM_008015718.1	-0.40621	2.46E-07	2.12E-06	sp|P48200|IREB2_HUMAN Iron-responsive element-binding protein 2 OS=Homo sapiens GN=IREB2 PE=1 SV=3//0
XM_008015719.1	-1.464	7.24E-27	1.97E-25	sp|P29762|RABP1_HUMAN Cellular retinoic acid-binding protein 1 OS=Homo sapiens GN=CRABP1 PE=1 SV=2//8.89725e-97

XM_008015728.1	0.7056	3.82E-13	5.16E-12	sp|Q5R678|IDH3A_PONAB Isocitrate dehydrogenase [NAD] subunit alpha, mitochondrial OS=Pongo abelii GN=IDH3A PE=2 SV=1//0
XM_008015729.1	1.3874	1.72E-12	2.25E-11	sp|Q96MP5|ZSWM3_HUMAN Zinc finger SWIM domain-containing protein 3 OS=Homo sapiens GN=ZSWIM3 PE=2 SV=2//0
XM_008015730.1	0.68515	2.18E-11	2.65E-10	sp|Q9UPU7|TBD2B_HUMAN TBC1 domain family member 2B OS=Homo sapiens GN=TBC1D2B PE=1 SV=2//0
XM_008015758.1	-1.2211	1.90E-53	1.26E-51	sp|Q5RE11|TSN3_PONAB Tetraspanin-3 OS=Pongo abelii GN=TSPAN3 PE=2 SV=1//4.5436e-165
XM_008015761.1	-0.35704	0.0003721	0.0022603	sp|Q14257|RCN2_HUMAN Reticulocalbin-2 OS=Homo sapiens GN=RCN2 PE=1 SV=1//0
XM_008015793.1	0.54225	0.0021545	0.011606	sp|Q13136|LIPA1_HUMAN Liprin-alpha-1 OS=Homo sapiens GN=PPFIA1 PE=1 SV=1//0
XM_008015801.1	-0.71971	1.67E-07	1.46E-06	sp|Q9H0A8|COMD4_HUMAN COMM domain-containing protein 4 OS=Homo sapiens GN=COMMD4 PE=1 SV=1//2.98515e-127
XM_008015808.1	1.3204	4.67E-45	2.42E-43	sp|Q9H147|TDIF1_HUMAN Deoxynucleotidyltransferase terminal-interacting protein 1 OS=Homo sapiens GN=DNTTIP1 PE=1 SV=2//0
XM_008015820.1	1.2048	6.65E-11	7.74E-10	sp|Q9NV31|IMP3_HUMAN U3 small nucleolar ribonucleoprotein protein IMP3 OS=Homo sapiens GN=IMP3 PE=1 SV=1//8.65207e-115
XM_008015850.1	-0.90542	1.30E-14	1.92E-13	sp|Q53CF8|COX5A_MACMU Cytochrome c oxidase subunit 5A, mitochondrial OS=Macaca mulatta GN=COX5A PE=2 SV=1//1.68942e-105
XM_008015952.1	-0.6294	1.96E-12	2.54E-11	sp|Q08397|LOXL1_HUMAN Lysyl oxidase homolog 1 OS=Homo sapiens GN=LOXL1 PE=1 SV=2//0
XM_008015987.1	-0.61666	1.96E-14	2.87E-13	sp|Q969N2|PIGT_HUMAN GPI transamidase component PIG-T OS=Homo sapiens GN=PIGT PE=1 SV=1//0
XM_008015994.1	0.62671	2.60E-13	3.56E-12	sp|Q9Z1K5|ARI1_MOUSE E3 ubiquitin-protein ligase ARIH1 OS=Mus musculus GN=Arih1 PE=1 SV=3//0
XM_008016049.1	0.61062	7.16E-05	0.0004786	sp|Q6ZMP0|THSD4_HUMAN Thrombospondin type-1 domain-containing protein 4 OS=Homo sapiens GN=THSD4 PE=2 SV=2//0
XM_008016051.1	0.6293	0.000136	0.0008759	sp|Q9BRS8|LARP6_HUMAN La-related protein 6 OS=Homo sapiens GN=LARP6 PE=1 SV=1//0
XM_008016063.1	0.57234	3.59E-15	5.44E-14	sp|P31431|SDC4_HUMAN Syndecan-4 OS=Homo sapiens GN=SDC4 PE=1 SV=2//2.92104e-82
XM_008016081.1	-0.55489	2.10E-07	1.82E-06	sp|P05386|RLA1_HUMAN 60S acidic ribosomal protein P1 OS=Homo sapiens GN=RPLP1 PE=1 SV=1//4.77512e-42
XM_008016093.1	-1.8171	1.07E-06	8.74E-06	sp|O94923|GLCE_HUMAN D-glucuronyl C5-epimerase OS=Homo sapiens GN=GLCE PE=1 SV=3//0
XM_008016099.1	-0.92555	7.95E-28	2.26E-26	sp|P39687|AN32A_HUMAN Acidic leucine-rich nuclear phosphoprotein 32 family member A OS=Homo sapiens GN=ANP32A PE=1 SV=1//1.64709e-86
XM_008016125.1	0.38897	2.24E-05	0.0001585	sp|O75925|PIAS1_HUMAN E3 SUMO-protein ligase PIAS1 OS=Homo sapiens GN=PIAS1 PE=1 SV=2//0
XM_008016160.1	1.0509	6.71E-06	5.06E-05	sp|P84025|SMAD3_RAT Mothers against decapentaplegic homolog 3 OS=Rattus norvegicus GN=Smad3 PE=1 SV=1//0
XM_008016164.1	1.0695	5.40E-20	1.07E-18	sp|O43541|SMAD6_HUMAN Mothers against decapentaplegic homolog 6 OS=Homo sapiens GN=SMAD6 PE=1 SV=2//0
XM_008016166.1	1.2733	1.71E-06	1.37E-05	sp|Q9H900|ZWILC_HUMAN Protein zwilch homolog OS=Homo sapiens GN=ZWILCH PE=1 SV=2//0
XM_008016180.1	1.4794	1.82E-17	3.17E-16	sp|O75971|SNPC5_HUMAN snRNA-activating protein complex subunit 5 OS=Homo sapiens GN=SNAPC5 PE=1 SV=1//8.03795e-35
XM_008016182.1	-1.6588	0.0018351	0.010014	sp|P19957|ELAF_HUMAN Elafin OS=Homo sapiens GN=PI3 PE=1 SV=3//3.88129e-63
XM_008016207.1	-0.93353	6.47E-20	1.28E-18	sp|P62494|RB11A_RAT Ras-related protein Rab-11A OS=Rattus norvegicus GN=Rab11a PE=1 SV=3//2.88308e-159
XM_008016212.1	1.5293	5.42E-07	4.54E-06	sp|Q7Z401|MYCPP_HUMAN C-myc promoter-binding protein OS=Homo sapiens GN=DENND4A PE=1 SV=2//0
XM_008016254.1	0.8082	1.13E-08	1.11E-07	sp|Q8N8D1|PDCD7_HUMAN Programmed cell death protein 7 OS=Homo sapiens GN=PDCD7 PE=1 SV=1//2.42749e-138
XM_008016281.1	-0.3601	0.0041186	0.020941	sp|Q8TD55|PKHO2_HUMAN Pleckstrin homology domain-containing family O member 2 OS=Homo sapiens GN=PLEKHO2 PE=1 SV=1//0
XM_008016292.1	-0.50716	1.10E-07	9.80E-07	sp|Q5R680|OAZ2_PONAB Ornithine decarboxylase antizyme 2 OS=Pongo abelii GN=OAZ2 PE=2 SV=1//1.58027e-113
XM_008016293.1	0.82926	3.27E-25	8.46E-24	sp|O15014|ZN609_HUMAN Zinc finger protein 609 OS=Homo sapiens GN=ZNF609 PE=1 SV=2//0
XM_008016314.1	-1.1035	0.0013025	0.0073189	sp|Q4R503|SNX1_MACFA Sorting nexin-1 OS=Macaca fascicularis GN=SNX1 PE=2 SV=1//0
XM_008016315.1	-1.063	2.42E-05	0.0001705	sp|Q4R503|SNX1_MACFA Sorting nexin-1 OS=Macaca fascicularis GN=SNX1 PE=2 SV=1//0
XM_008016336.1	0.89579	0.000966	0.005527	sp|Q9Y6I4|UBP3_HUMAN Ubiquitin carboxyl-terminal hydrolase 3 OS=Homo sapiens GN=USP3 PE=1 SV=2//0
XM_008016341.1	0.49965	8.91E-06	6.62E-05	sp|Q92930|RAB8B_HUMAN Ras-related protein Rab-8B OS=Homo sapiens GN=RAB8B PE=1 SV=2//3.48311e-135
XM_008016390.1	-0.80178	1.92E-05	0.0001369	sp|Q9H426|RIMS4_HUMAN Regulating synaptic membrane exocytosis protein 4

				OS=Homo sapiens GN=RIMS4 PE=1 SV=3//0
XM_008016395.1	-0.67016	8.46E-25	2.12E-23	sp|Q5R5A0|ANXA2_PONAB Annexin A2 OS=Pongo abelii GN=ANXA2 PE=2 SV=1//0
XM_008016398.1	0.64053	0.0014718	0.008186	sp|Q12982|BNIP2_HUMAN BCL2/adenovirus E1B 19 kDa protein-interacting protein 2 OS=Homo sapiens GN=BNIP2 PE=1 SV=1//0
XM_008016424.1	1.9227	2.67E-139	7.92E-137	sp|Q12965|MYO1E_HUMAN Unconventional myosin-Ie OS=Homo sapiens GN=MYO1E PE=1 SV=2//0
XM_008016425.1	-0.86441	4.15E-21	8.69E-20	sp|Q4R7A8|CCNB2_MACFA G2/mitotic-specific cyclin-B2 OS=Macaca fascicularis GN=CCNB2 PE=2 SV=1//0
XM_008016455.1	-0.70798	1.76E-21	3.78E-20	sp|O14672|ADA10_HUMAN Disintegrin and metalloproteinase domain-containing protein 10 OS=Homo sapiens GN=ADAM10 PE=1 SV=1//0
XM_008016456.1	3.075	9.15E-07	7.54E-06	sp|O43315|AQP9_HUMAN Aquaporin-9 OS=Homo sapiens GN=AQP9 PE=2 SV=2//2.88319e-176
XM_008016497.1	0.96691	0.0026815	0.014187	sp|Q6N043|Z280D_HUMAN Zinc finger protein 280D OS=Homo sapiens GN=ZNF280D PE=1 SV=3//3.34658e-57
XM_008016508.1	0.50951	3.87E-06	2.99E-05	sp|Q4R7T8|MNS1_MACFA Meiosis-specific nuclear structural protein 1 OS=Macaca fascicularis GN=MNS1 PE=2 SV=2//0
XM_008016512.1	0.80595	0.0027271	0.014414	sp|P46934|NEDD4_HUMAN E3 ubiquitin-protein ligase NEDD4 OS=Homo sapiens GN=NEDD4 PE=1 SV=4//0
XM_008016529.1	#NAME?	0.0067767	0.032877	sp|P00813|ADA_HUMAN Adenosine deaminase OS=Homo sapiens GN=ADA PE=1 SV=3//2.17634e-14
XM_008016539.1	0.47023	1.16E-06	9.48E-06	sp|Q9UHA3|RLP24_HUMAN Probable ribosome biogenesis protein RLP24 OS=Homo sapiens GN=RSL24D1 PE=1 SV=1//1.2446e-109
XM_008016541.1	2.4232	1.39E-11	1.71E-10	sp|Q8NB66|UN13C_HUMAN Protein unc-13 homolog C OS=Homo sapiens GN=UNC13C PE=2 SV=3//0
XM_008016552.1	-0.45388	2.93E-05	0.0002039	sp|Q4R5H8|RL18_MACFA 60S ribosomal protein L18 OS=Macaca fascicularis GN=RPL18 PE=2 SV=1//7.24982e-131
XM_008016588.1	1.0186	2.17E-24	5.32E-23	sp|Q16659|MK06_HUMAN Mitogen-activated protein kinase 6 OS=Homo sapiens GN=MAPK6 PE=1 SV=1//0
XM_008016589.1	-1.7224	0.0002278	0.0014208	sp|Q5R4D6|LEO1_PONAB RNA polymerase-associated protein LEO1 OS=Pongo abelii GN=LEO1 PE=2 SV=1//4.17154e-131
XM_008016608.1	-4.0053	4.80E-05	0.0003264	sp|Q8TDJ6|DMXL2_HUMAN DmX-like protein 2 OS=Homo sapiens GN=DMXL2 PE=1 SV=2//0
XM_008016616.1	0.90585	5.94E-05	0.0003996	sp|Q9UPM8|AP4E1_HUMAN AP-4 complex subunit epsilon-1 OS=Homo sapiens GN=AP4E1 PE=1 SV=2//0
XM_008016680.1	-1.2397	4.54E-43	2.16E-41	sp|Q5RDL6|EID1_PONAB EP300-interacting inhibitor of differentiation 1 OS=Pongo abelii GN=EID1 PE=2 SV=1//1.54466e-71
XM_008016692.1	-0.78573	4.83E-07	4.06E-06	sp|P35555|FBN1_HUMAN Fibrillin-1 OS=Homo sapiens GN=FBN1 PE=1 SV=3//0
XM_008016693.1	-1.2262	0.000648	0.0038039	sp|Q71RS6|NCKX5_HUMAN Sodium/potassium/calcium exchanger 5 OS=Homo sapiens GN=SLC24A5 PE=1 SV=1//0
XM_008016738.1	-0.59727	0.0048016	0.024045	sp|Q8N6M3|FITM2_HUMAN Fat storage-inducing transmembrane protein 2 OS=Homo sapiens GN=FITM2 PE=2 SV=1//4.04712e-178
XM_008016787.1	1.1736	1.52E-06	1.22E-05	sp|Q05D32|CTSL2_HUMAN CTD small phosphatase-like protein 2 OS=Homo sapiens GN=CTDSPL2 PE=1 SV=2//8.04233e-07
XM_008016852.1	1.7298	3.72E-14	5.34E-13	sp|O60294|TYW4_HUMAN tRNA wybutosine-synthesizing protein 4 OS=Homo sapiens GN=LCMT2 PE=1 SV=3//0
XM_008016913.1	#NAME?	0.0015006	0.0083358	sp|Q9HBY8|SGK2_HUMAN Serine/threonine-protein kinase Sgk2 OS=Homo sapiens GN=SGK2 PE=1 SV=1//0
XM_008016927.1	0.75557	2.82E-08	2.66E-07	sp|Q13158|FADD_HUMAN FAS-associated death domain protein OS=Homo sapiens GN=FADD PE=1 SV=1//9.51e-87
XM_008016928.1	1.9161	1.22E-121	3.08E-119	sp|Q9H223|EHD4_HUMAN EH domain-containing protein 4 OS=Homo sapiens GN=EHD4 PE=1 SV=1//0
XM_008016936.1	1.5783	3.32E-05	0.0002301	sp|Q9NRC6|SPTN5_HUMAN Spectrin beta chain, non-erythrocytic 5 OS=Homo sapiens GN=SPTBN5 PE=1 SV=2//0
XM_008016944.1	-0.69006	5.90E-12	7.43E-11	sp|Q5R7F0|CHP1_PONAB Calcineurin B homologous protein 1 OS=Pongo abelii GN=CHP1 PE=2 SV=3//7.11763e-131
XM_008016959.1	-0.64738	2.14E-13	2.95E-12	sp|Q92541|RTF1_HUMAN RNA polymerase-associated protein RTF1 homolog OS=Homo sapiens GN=RTF1 PE=1 SV=4//0
XM_008016967.1	-0.74711	9.85E-08	8.81E-07	sp|Q06418|TYRO3_HUMAN Tyrosine-protein kinase receptor TYRO3 OS=Homo sapiens GN=TYRO3 PE=1 SV=1//0
XM_008016978.1	0.87697	1.15E-33	4.07E-32	sp|Q13247|SRSF6_HUMAN Serine/arginine-rich splicing factor 6 OS=Homo sapiens GN=SRSF6 PE=1 SV=2//2.01362e-93
XM_008016986.1	3.4465	9.29E-25	2.32E-23	sp|Q9BUX1|CHAC1_HUMAN Glutathione-specific gamma-glutamylcyclotransferase 1 OS=Homo sapiens GN=CHAC1 PE=1 SV=2//3.4718e-162
XM_008016988.1	1.1628	1.44E-24	3.56E-23	sp|Q9P253|VPS18_HUMAN Vacuolar protein sorting-associated protein 18 homolog OS=Homo sapiens GN=VPS18 PE=1 SV=2//0
XM_008017007.1	0.6818	0.0087787	0.04164	sp|Q9NVM6|DJC17_HUMAN DnaJ homolog subfamily C member 17 OS=Homo sapiens GN=DNAJC17 PE=1 SV=1//1.76325e-147
XM_008017008.1	0.95209	1.87E-07	1.63E-06	sp|Q96K21|ANCHR_HUMAN Abscission/NoCut checkpoint regulator OS=Homo sapiens GN=ZFYVE19 PE=1 SV=3//0

XM_008017020.1	-0.76581	1.92E-06	1.53E-05	sp|Q8NCH0|CHSTE_HUMAN Carbohydrate sulfotransferase 14 OS=Homo sapiens GN=CHST14 PE=1 SV=2//0
XM_008017042.1	1.623	3.49E-21	7.37E-20	sp|P0DMQ5|INAM2_HUMAN Putative transmembrane protein INAFM2 OS=Homo sapiens GN=INAFM2 PE=2 SV=1//5.35789e-24
XM_008017059.1	-1.3512	0.010116	0.047308	sp|C9JTQ0|ANR63_HUMAN Ankyrin repeat domain-containing protein 63 OS=Homo sapiens GN=ANKRD63 PE=3 SV=1//2.61312e-180
XM_008017078.1	-1.2699	6.90E-30	2.12E-28	sp|Q4R5C7|SRP14_MACFA Signal recognition particle 14 kDa protein OS=Macaca fascicularis GN=SRP14 PE=2 SV=1//3.90561e-68
XM_008017079.1	0.8691	1.55E-11	1.90E-10	sp|Q14439|GP176_HUMAN Probable G-protein coupled receptor 176 OS=Homo sapiens GN=GPR176 PE=2 SV=1//0
XM_008017083.1	2.2007	4.00E-242	2.74E-239	sp|P07996|TSP1_HUMAN Thrombospondin-1 OS=Homo sapiens GN=THBS1 PE=1 SV=2//0
XM_008017098.1	1.1456	2.30E-07	1.98E-06	sp|Q7Z699|SPRE1_HUMAN Sprouty-related, EVH1 domain-containing protein 1 OS=Homo sapiens GN=SPRED1 PE=1 SV=2//0
XM_008017121.1	0.68441	1.08E-14	1.61E-13	sp|Q6IQ21|ZN770_HUMAN Zinc finger protein 770 OS=Homo sapiens GN=ZNF770 PE=2 SV=1//0
XM_008017161.1	1.5509	1.77E-56	1.24E-54	sp|Q68DA7|FMN1_HUMAN Formin-1 OS=Homo sapiens GN=FMN1 PE=1 SV=3//0
XM_008017171.1	0.3098	0.000153	0.0009767	sp|Q6P4F7|RHGBA_HUMAN Rho GTPase-activating protein 11A OS=Homo sapiens GN=ARHGAP11A PE=1 SV=2//0
XM_008017194.1	-0.31482	0.0007792	0.0045172	sp|Q9Y2Y9|KLF13_HUMAN Krueppel-like factor 13 OS=Homo sapiens GN=KLF13 PE=1 SV=1//2.72618e-111
XM_008017204.1	2.0131	2.45E-15	3.75E-14	sp|Q9NXD2|MTMRA_HUMAN Myotubularin-related protein 10 OS=Homo sapiens GN=MTMR10 PE=1 SV=3//0
XM_008017212.1	0.65126	1.24E-07	1.10E-06	sp|Q96MG7|MAGG1_HUMAN Melanoma-associated antigen G1 OS=Homo sapiens GN=NDNL2 PE=1 SV=1//1.01165e-160
XM_008017243.1	-1.787	0.0035658	0.018364	sp|P78325|ADAM8_HUMAN Disintegrin and metalloproteinase domain-containing protein 8 OS=Homo sapiens GN=ADAM8 PE=1 SV=2//0
XM_008017254.1	-0.79759	1.55E-17	2.71E-16	sp|P30084|ECHM_HUMAN Enoyl-CoA hydratase, mitochondrial OS=Homo sapiens GN=ECHS1 PE=1 SV=4//0
XM_008017258.1	-1.0273	9.17E-09	8.99E-08	sp|Q6QHF9|PAOX_HUMAN Peroxisomal N(1)-acetyl-spermine/spermidine oxidase OS=Homo sapiens GN=PAOX PE=1 SV=3//5.38049e-81
XM_008017298.1	-0.48994	2.13E-06	1.69E-05	sp|Q8BHK1|NIPA1_MOUSE Magnesium transporter NIPA1 OS=Mus musculus GN=Nipa1 PE=1 SV=1//3.18498e-171
XM_008017324.1	1.0789	4.38E-50	2.71E-48	sp|Q9WUL0|TOP1_RAT DNA topoisomerase 1 OS=Rattus norvegicus GN=Top1 PE=2 SV=1//0
XM_008017385.1	-2.0518	2.40E-07	2.07E-06	sp|Q6UVK1|CSPG4_HUMAN Chondroitin sulfate proteoglycan 4 OS=Homo sapiens GN=CSPG4 PE=1 SV=2//0
XM_008017390.1	-1.5628	1.21E-18	2.25E-17	sp|Q9UKP4|ATS7_HUMAN A disintegrin and metalloproteinase with thrombospondin motifs 7 OS=Homo sapiens GN=ADAMTS7 PE=1 SV=2//2.22983e-06
XM_008017391.1	-1.1855	4.12E-05	0.0002819	sp|Q9UKP4|ATS7_HUMAN A disintegrin and metalloproteinase with thrombospondin motifs 7 OS=Homo sapiens GN=ADAMTS7 PE=1 SV=2//3.26757e-21
XM_008017392.1	-1.5599	4.80E-09	4.80E-08	sp|Q9UKP4|ATS7_HUMAN A disintegrin and metalloproteinase with thrombospondin motifs 7 OS=Homo sapiens GN=ADAMTS7 PE=1 SV=2//0
XM_008017393.1	1.5333	1.35E-20	2.76E-19	sp|Q9BY12|SCAPE_HUMAN S phase cyclin A-associated protein in the endoplasmic reticulum OS=Homo sapiens GN=SCAPER PE=1 SV=2//0
XM_008017396.1	-1.5851	4.35E-17	7.41E-16	sp|Q8TAC9|SCAM5_HUMAN Secretory carrier-associated membrane protein 5 OS=Homo sapiens GN=SCAMP5 PE=1 SV=1//5.23666e-117
XM_008017401.1	-0.96456	1.98E-48	1.16E-46	sp|Q5NVN0|KPYM_PONAB Pyruvate kinase PKM OS=Pongo abelii GN=PKM PE=2 SV=3//0
XM_008017408.1	1.1492	3.88E-10	4.27E-09	sp|Q08379|GOGA2_HUMAN Golgin subfamily A member 2 OS=Homo sapiens GN=GOLGA2 PE=1 SV=3//1.76982e-06
XM_008017413.1	0.42741	1.18E-06	9.61E-06	sp|Q96JI7|SPTCS_HUMAN Spatacsin OS=Homo sapiens GN=SPG11 PE=1 SV=3//0
XM_008017414.1	0.89728	3.33E-09	3.37E-08	sp|Q5R8D1|EIF3J_PONAB Eukaryotic translation initiation factor 3 subunit J OS=Pongo abelii GN=EIF3J PE=2 SV=1//2.24556e-75
XM_008017416.1	-0.4782	0.0023714	0.012684	sp|Q6PFW1|VIP1_HUMAN Inositol hexakisphosphate and diphosphoinositol-pentakisphosphate kinase 1 OS=Homo sapiens GN=PPIP5K1 PE=1 SV=1//0
XM_008017426.1	4.9464	7.36E-05	0.000491	sp|Q15413|RYR3_HUMAN Ryanodine receptor 3 OS=Homo sapiens GN=RYR3 PE=1 SV=3//1.34061e-19
XM_008017458.1	0.81465	7.22E-19	1.36E-17	sp|Q9P270|SLAI2_HUMAN SLAIN motif-containing protein 2 OS=Homo sapiens GN=SLAIN2 PE=1 SV=2//0
XM_008017461.1	0.70854	7.32E-12	9.14E-11	sp|Q9H4H8|FA83D_HUMAN Protein FAM83D OS=Homo sapiens GN=FAM83D PE=1 SV=3//0
XM_008017466.1	0.90851	5.90E-08	5.39E-07	sp|Q5RDB8|NIPA3_PONAB Magnesium transporter NIPA3 OS=Pongo abelii GN=NIPAL1 PE=2 SV=1//0
XM_008017529.1	-0.23783	0.0083396	0.039746	sp|Q6PML9|ZNT9_HUMAN Zinc transporter 9 OS=Homo sapiens GN=SLC30A9 PE=1 SV=1//0
XM_008017541.1	-0.59792	1.06E-16	1.78E-15	sp|Q02878|RL6_HUMAN 60S ribosomal protein L6 OS=Homo sapiens GN=RPL6 PE=1 SV=3//5.02828e-151

XM_008017554.1	-0.77785	2.43E-24	5.95E-23	sp|Q60HC8|UCHL1_MACFA Ubiquitin carboxyl-terminal hydrolase isozyme L1 OS=Macaca fascicularis GN=UCHL1 PE=2 SV=1//1.37442e-148
XM_008017595.1	-0.46816	0.0016118	0.0088967	sp|Q86UW6|N4BP2_HUMAN NEDD4-binding protein 2 OS=Homo sapiens GN=N4BP2 PE=1 SV=2//0
XM_008017599.1	0.60809	1.43E-19	2.79E-18	sp|Q29RF7|PDS5A_HUMAN Sister chromatid cohesion protein PDS5 homolog A OS=Homo sapiens GN=PDS5A PE=1 SV=1//0
XM_008017615.1	-0.79207	8.46E-32	2.78E-30	sp|P32969|RL9_HUMAN 60S ribosomal protein L9 OS=Homo sapiens GN=RPL9 PE=1 SV=1//1.03586e-127
XM_008017616.1	-3.6328	4.42E-10	4.83E-09	sp|Q86Z14|KLOTB_HUMAN Beta-klotho OS=Homo sapiens GN=KLB PE=1 SV=1//0
XM_008017625.1	-1.3987	2.40E-12	3.09E-11	sp|Q96PQ7|KLHL5_HUMAN Kelch-like protein 5 OS=Homo sapiens GN=KLHL5 PE=2 SV=3//0
XM_008017631.1	-1.9364	4.33E-33	1.49E-31	sp|Q8IWE2|NXP20_HUMAN Protein NOXP20 OS=Homo sapiens GN=FAM114A1 PE=1 SV=2//0
XM_008017651.1	-0.91138	0.0059991	0.02945	sp|P57682|KLF3_HUMAN Krueppel-like factor 3 OS=Homo sapiens GN=KLF3 PE=1 SV=1//0
XM_008017660.1	0.92057	9.82E-19	1.84E-17	sp|Q96G03|PGM2_HUMAN Phosphoglucomutase-2 OS=Homo sapiens GN=PGM2 PE=1 SV=4//0
XM_008017733.1	1.3587	8.34E-28	2.37E-26	sp|Q5RAK7|SPCS_PONAB O-phosphoseryl-tRNA(Sec) selenium transferase OS=Pongo abelii GN=SEPSECS PE=2 SV=1//0
XM_008017735.1	-1.0679	1.16E-05	8.53E-05	sp|Q1EGL1|LGI2_PANTR Leucine-rich repeat LGI family member 2 OS=Pan troglodytes GN=LGI2 PE=2 SV=1//0
XM_008017741.1	-1.2191	5.54E-05	0.0003745	sp|P08294|SODE_HUMAN Extracellular superoxide dismutase [Cu-Zn] OS=Homo sapiens GN=SOD3 PE=1 SV=2//1.18455e-135
XM_008017742.1	0.8273	5.95E-32	1.96E-30	sp|Q5RAZ4|DHX15_PONAB Pre-mRNA-splicing factor ATP-dependent RNA helicase DHX15 OS=Pongo abelii GN=DHX15 PE=2 SV=2//0
XM_008017793.1	0.64457	1.12E-09	1.19E-08	sp|Q9BPX3|CND3_HUMAN Condensin complex subunit 3 OS=Homo sapiens GN=NCAPG PE=1 SV=1//0
XM_008017800.1	0.34551	0.0098677	0.046284	sp|Q8N3X6|LCORL_HUMAN Ligand-dependent nuclear receptor corepressor-like protein OS=Homo sapiens GN=LCORL PE=1 SV=4//0
XM_008017801.1	-1.1415	2.60E-50	1.62E-48	sp|P21980|TGM2_HUMAN Protein-glutamine gamma-glutamyltransferase 2 OS=Homo sapiens GN=TGM2 PE=1 SV=2//0
XM_008017803.1	-1.3016	0.0004624	0.0027745	sp|Q9ULE4|F184B_HUMAN Protein FAM184B OS=Homo sapiens GN=FAM184B PE=2 SV=3//0
XM_008017805.1	-0.72108	1.11E-09	1.17E-08	sp|Q9H204|MED28_HUMAN Mediator of RNA polymerase II transcription subunit 28 OS=Homo sapiens GN=MED28 PE=1 SV=1//4.97306e-88
XM_008017806.1	-0.40128	1.89E-05	0.000135	sp|P28838|AMPL_HUMAN Cytosol aminopeptidase OS=Homo sapiens GN=LAP3 PE=1 SV=3//0
XM_008017808.1	0.50364	2.45E-05	0.0001724	sp|P09417|DHPR_HUMAN Dihydropteridine reductase OS=Homo sapiens GN=QDPR PE=1 SV=2//1.02449e-150
XM_008017842.1	1.3752	9.84E-49	5.83E-47	sp|Q9NQG5|RPR1B_HUMAN Regulation of nuclear pre-mRNA domain-containing protein 1B OS=Homo sapiens GN=RPRD1B PE=1 SV=1//0
XM_008017848.1	-0.40521	0.0001227	0.000796	sp|Q10588|BST1_HUMAN ADP-ribosyl cyclase/cyclic ADP-ribose hydrolase 2 OS=Homo sapiens GN=BST1 PE=1 SV=2//0
XM_008017862.1	-0.27028	0.0035155	0.018127	sp|Q8WYA6|CTBL1_HUMAN Beta-catenin-like protein 1 OS=Homo sapiens GN=CTNNBL1 PE=1 SV=1//0
XM_008017864.1	-1.3487	6.38E-06	4.83E-05	sp|Q9UKA1|FBXL5_HUMAN F-box/LRR-repeat protein 5 OS=Homo sapiens GN=FBXL5 PE=1 SV=2//0
XM_008017869.1	1.4031	1.62E-38	6.69E-37	sp|P0CF97|F200B_HUMAN Protein FAM200B OS=Homo sapiens GN=FAM200B PE=3 SV=1//0
XM_008017884.1	-1.2286	0.0005712	0.003385	sp|P78367|NKX32_HUMAN Homeobox protein Nkx-3.2 OS=Homo sapiens GN=NKX3-2 PE=2 SV=2//8.90494e-119
XM_008017902.1	-0.21235	0.0029945	0.015707	sp|O75083|WDR1_HUMAN WD repeat-containing protein 1 OS=Homo sapiens GN=WDR1 PE=1 SV=4//0
XM_008017913.1	3.2406	0.0071597	0.034544	sp|Q6ZSB9|ZBT49_HUMAN Zinc finger and BTB domain-containing protein 49 OS=Homo sapiens GN=ZBTB49 PE=1 SV=3//0
XM_008017919.1	0.90869	1.24E-20	2.55E-19	sp|Q9P2W9|STX18_HUMAN Syntaxin-18 OS=Homo sapiens GN=STX18 PE=1 SV=1//0
XM_008017924.1	0.81489	1.81E-08	1.74E-07	sp|Q2VL84|MSX1_CALJA Homeobox protein MSX-1 OS=Callithrix jacchus GN=MSX1 PE=3 SV=2//1.32436e-123
XM_008017978.1	0.38703	0.0009118	0.0052375	sp|Q96HT8|MR1L1_HUMAN MORF4 family-associated protein 1-like 1 OS=Homo sapiens GN=MRFAP1L1 PE=1 SV=1//2.0145e-48
XM_008018000.1	0.55182	3.35E-08	3.13E-07	sp|Q5RA81|GRPE1_PONAB GrpE protein homolog 1, mitochondrial OS=Pongo abelii GN=GRPEL1 PE=2 SV=1//8.69912e-111
XM_008018001.1	1.0063	5.05E-20	1.00E-18	sp|Q5RBN9|TAD2B_PONAB Transcriptional adapter 2-beta OS=Pongo abelii GN=TADA2B PE=2 SV=1//0
XM_008018015.1	-1.5944	2.90E-06	2.27E-05	sp|P83110|HTRA3_HUMAN Serine protease HTRA3 OS=Homo sapiens GN=HTRA3 PE=1 SV=2//0
XM_008018017.1	0.94244	5.90E-16	9.42E-15	sp|O15254|ACOX3_HUMAN Peroxisomal acyl-coenzyme A oxidase 3 OS=Homo sapiens GN=ACOX3 PE=1 SV=2//0
XM_008018028.1	0.97332	4.27E-20	8.52E-19	sp|Q9NP08|HMX1_HUMAN Homeobox protein HMX1 OS=Homo sapiens GN=HMX1 PE=2 SV=2//1.37511e-61

XM_008018030.1	-1.8541	2.00E-07	1.74E-06	sp|P18825|ADA2C_HUMAN Alpha-2C adrenergic receptor OS=Homo sapiens GN=ADRA2C PE=2 SV=2//0
XM_008018031.1	-0.57274	5.46E-11	6.40E-10	sp|P30533|AMRP_HUMAN Alpha-2-macroglobulin receptor-associated protein OS=Homo sapiens GN=LRPAP1 PE=1 SV=1//0
XM_008018062.1	0.27969	4.60E-05	0.0003137	sp|P42858|HD_HUMAN Huntingtin OS=Homo sapiens GN=HTT PE=1 SV=2//0
XM_008018064.1	1.7559	1.43E-05	0.0001037	sp|P78316|NOP14_HUMAN Nucleolar protein 14 OS=Homo sapiens GN=NOP14 PE=1 SV=3//0
XM_008018069.1	0.49308	0.0080334	0.038404	sp|P28749|RBL1_HUMAN Retinoblastoma-like protein 1 OS=Homo sapiens GN=RBL1 PE=1 SV=3//1.3534e-80
XM_008018076.1	-1.8823	5.68E-08	5.19E-07	sp|P35611|ADDA_HUMAN Alpha-adducin OS=Homo sapiens GN=ADD1 PE=1 SV=2//0
XM_008018122.1	0.50909	0.0067916	0.032928	-//-
XM_008018123.1	0.59992	8.64E-06	6.43E-05	sp|Q8N9F0|NAT8L_HUMAN N-acetylaspartate synthetase OS=Homo sapiens GN=NAT8L PE=1 SV=3//2.45007e-134
XM_008018125.1	-1.6826	6.87E-19	1.29E-17	sp|Q5BLP8|CD048_HUMAN Neuropeptide-like protein C4orf48 OS=Homo sapiens GN=C4orf48 PE=1 SV=3//7.45532e-33
XM_008018126.1	0.65404	3.54E-11	4.22E-10	sp|Q9H3P2|NELFA_HUMAN Negative elongation factor A OS=Homo sapiens GN=NELFA PE=1 SV=3//0
XM_008018130.1	-0.89014	1.04E-06	8.50E-06	sp|O96028|NSD2_HUMAN Histone-lysine N-methyltransferase NSD2 OS=Homo sapiens GN=WHSC1 PE=1 SV=1//0
XM_008018170.1	1.0104	7.63E-46	4.10E-44	sp|P78504|JAG1_HUMAN Protein jagged-1 OS=Homo sapiens GN=JAG1 PE=1 SV=3//0
XM_008018172.1	-1.4476	3.64E-06	2.83E-05	sp|Q9BUD6|SPON2_HUMAN Spondin-2 OS=Homo sapiens GN=SPON2 PE=1 SV=3//0
XM_008018178.1	-0.5438	0.0022234	0.011939	sp|Q9BSA9|TM175_HUMAN Transmembrane protein 175 OS=Homo sapiens GN=TMEM175 PE=1 SV=1//0
XM_008018191.1	1.0584	0.001718	0.0094379	sp|Q495C1|RN212_HUMAN Probable E3 SUMO-protein ligase RNF212 OS=Homo sapiens GN=RNF212 PE=2 SV=1//2.12476e-14
XM_008018194.1	1.7256	5.21E-136	1.48E-133	sp|P24385|CCND1_HUMAN G1/S-specific cyclin-D1 OS=Homo sapiens GN=CCND1 PE=1 SV=1//2.16194e-167
XM_008018195.1	#NAME?	0.0014075	0.0078681	-//-
XM_008018196.1	-0.27246	0.0015375	0.0085342	sp|Q3KNV8|PCGF3_HUMAN Polycomb group RING finger protein 3 OS=Homo sapiens GN=PCGF3 PE=1 SV=1//3.60387e-164
XM_008018202.1	-1.6335	5.92E-05	0.0003986	sp|Q5RBW2|ATP5I_PONAB ATP synthase subunit e, mitochondrial OS=Pongo abelii GN=ATP5I PE=3 SV=3//5.19268e-28
XM_008018230.1	-0.37885	0.004617	0.023249	sp|Q8N5G0|SMI20_HUMAN Small integral membrane protein 20 OS=Homo sapiens GN=SMIM20 PE=1 SV=2//3.08999e-62
XM_008018237.1	-0.73843	1.26E-08	1.22E-07	sp|Q96PQ0|SORC2_HUMAN VPS10 domain-containing receptor SorCS2 OS=Homo sapiens GN=SORCS2 PE=1 SV=3//0
XM_008018242.1	-1.5271	0.0028963	0.015246	sp|P20171|RASH_RAT GTPase HRas OS=Rattus norvegicus GN=Hras PE=1 SV=2//6.36279e-128
XM_008018244.1	0.96405	5.32E-06	4.06E-05	sp|Q5RCD2|TCPZ_PONAB T-complex protein 1 subunit zeta OS=Pongo abelii GN=CCT6 PE=2 SV=3//3.74599e-58
XM_008018247.1	-0.25899	0.0011138	0.0063233	sp|O77695|BGLR_CHLAE Beta-glucuronidase (Fragment) OS=Chlorocebus aethiops GN=GUSB PE=2 SV=1//0
XM_008018265.1	1.0852	0.0002006	0.0012608	sp|Q9UHL9|GT2D1_HUMAN General transcription factor II-I repeat domain-containing protein 1 OS=Homo sapiens GN=GTF2IRD1 PE=1 SV=1//2.17614e-07
XM_008018280.1	-0.2671	0.0035861	0.018465	sp|Q9NWD8|TM248_HUMAN Transmembrane protein 248 OS=Homo sapiens GN=TMEM248 PE=1 SV=1//0
XM_008018281.1	0.59009	1.36E-08	1.32E-07	sp|Q5RAZ2|SBDS_PONAB Ribosome maturation protein SBDS OS=Pongo abelii GN=SBDS PE=2 SV=1//1.76548e-167
XM_008018305.1	-1.749	2.20E-54	1.50E-52	sp|A6NHX0|GATL2_HUMAN GATS-like protein 2 OS=Homo sapiens GN=GATSL2 PE=2 SV=3//0
XM_008018314.1	-0.38518	2.38E-08	2.26E-07	sp|P78347|GTF2I_HUMAN General transcription factor II-I OS=Homo sapiens GN=GTF2I PE=1 SV=2//0
XM_008018327.1	-2.5242	7.41E-05	0.0004943	sp|P53667|LIMK1_HUMAN LIM domain kinase 1 OS=Homo sapiens GN=LIMK1 PE=1 SV=3//0
XM_008018331.1	2.2587	2.53E-109	5.71E-107	sp|O19005|CLD4_CHLAE Claudin-4 OS=Chlorocebus aethiops GN=CLDN4 PE=2 SV=1//2.21035e-96
XM_008018333.1	-1.6159	1.24E-13	1.75E-12	sp|O15551|CLD3_HUMAN Claudin-3 OS=Homo sapiens GN=CLDN3 PE=1 SV=1//1.75113e-102
XM_008018342.1	0.32637	0.0059488	0.029216	sp|O43709|WBS22_HUMAN Probable 18S rRNA (guanine-N(7))-methyltransferase OS=Homo sapiens GN=WBSCR22 PE=1 SV=2//0
XM_008018344.1	-1.4041	2.36E-16	3.86E-15	sp|Q86XT2|VP37D_HUMAN Vacuolar protein sorting-associated protein 37D OS=Homo sapiens GN=VPS37D PE=1 SV=2//1.32835e-116
XM_008018345.1	1.6659	2.07E-77	2.49E-75	sp|Q9Y4P3|TBL2_HUMAN Transducin beta-like protein 2 OS=Homo sapiens GN=TBL2 PE=1 SV=1//0
XM_008018369.1	1.0781	0.0002153	0.0013484	sp|Q8WV07|ORAV1_HUMAN Oral cancer-overexpressed protein 1 OS=Homo sapiens GN=ORAOV1 PE=1 SV=2//3.6661e-34
XM_008018370.1	0.59052	5.86E-08	5.35E-07	sp|O60488|ACSL4_HUMAN Long-chain-fatty-acid--CoA ligase 4 OS=Homo sapiens GN=ACSL4 PE=1 SV=2//0

XM_008018385.1	-1.7231	0.0045274	0.022828	sp|Q9Y6J8|STYL1_HUMAN Serine/threonine/tyrosine-interacting-like protein 1 OS=Homo sapiens GN=STYXL1 PE=2 SV=1//2.20558e-143
XM_008018394.1	2.876	9.26E-08	8.29E-07	sp|Q9Y6J8|STYL1_HUMAN Serine/threonine/tyrosine-interacting-like protein 1 OS=Homo sapiens GN=STYXL1 PE=2 SV=1//0
XM_008018400.1	-2.0011	0.0063811	0.031123	sp|Q8OWV7|SRRM3_MOUSE Serine/arginine repetitive matrix protein 3 OS=Mus musculus GN=Srrm3 PE=2 SV=1//1.20076e-67
XM_008018401.1	-1.4527	4.66E-14	6.66E-13	sp|Q8OWV7|SRRM3_MOUSE Serine/arginine repetitive matrix protein 3 OS=Mus musculus GN=Srrm3 PE=2 SV=1//1.06714e-66
XM_008018402.1	-1.0681	3.84E-57	2.78E-55	sp|P04792|HSPB1_HUMAN Heat shock protein beta-1 OS=Homo sapiens GN=HSPB1 PE=1 SV=2//2.35529e-90
XM_008018404.1	0.421	1.25E-09	1.32E-08	sp|P61983|1433G_RAT 14-3-3 protein gamma OS=Rattus norvegicus GN=Ywhag PE=1 SV=2//1.55058e-158
XM_008018405.1	-1.0208	0.0010633	0.0060491	sp|Q8WTU2|SRB4D_HUMAN Scavenger receptor cysteine-rich domain-containing group B protein OS=Homo sapiens GN=SSC4D PE=2 SV=1//1.09815e-20
XM_008018416.1	-1.3599	1.49E-35	5.60E-34	sp|Q9H1E5|TMX4_HUMAN Thioredoxin-related transmembrane protein 4 OS=Homo sapiens GN=TMX4 PE=1 SV=1//3.90371e-172
XM_008018432.1	2.4568	6.09E-07	5.08E-06	sp|Q5R5J3|PKRI1_PONAB PRKR-interacting protein 1 OS=Pongo abelii GN=PRKRIP1 PE=2 SV=1//1.20881e-67
XM_008018467.1	-0.90036	2.05E-22	4.62E-21	sp|O43257|ZNHI1_HUMAN Zinc finger HIT domain-containing protein 1 OS=Homo sapiens GN=ZNHIT1 PE=1 SV=1//1.9339e-81
XM_008018469.1	2.6535	0.0001035	0.0006781	sp|Q8N8M0|NAT16_HUMAN Probable N-acetyltransferase 16 OS=Homo sapiens GN=NAT16 PE=2 SV=2//0
XM_008018472.1	3.6203	1.09E-79	1.38E-77	sp|O15240|VGF_HUMAN Neurosecretory protein VGF OS=Homo sapiens GN=VGF PE=1 SV=2//3.19072e-61
XM_008018474.1	Inf	1.90E-10	2.14E-09	sp|O15240|VGF_HUMAN Neurosecretory protein VGF OS=Homo sapiens GN=VGF PE=1 SV=2//2.1468e-61
XM_008018480.1	1.6662	2.60E-73	2.85E-71	sp|Q9BRZ2|TRI56_HUMAN E3 ubiquitin-protein ligase TRIM56 OS=Homo sapiens GN=TRIM56 PE=1 SV=3//0
XM_008018515.1	-0.7698	3.77E-09	3.81E-08	sp|P54760|EPHB4_HUMAN Ephrin type-B receptor 4 OS=Homo sapiens GN=EPHB4 PE=1 SV=2//0
XM_008018524.1	-0.96307	2.03E-34	7.41E-33	sp|P54313|GBB2_RAT Guanine nucleotide-binding protein G(I)/G(S)/G(T) subunit beta-2 OS=Rattus norvegicus GN=Gnb2 PE=1 SV=4//5.85104e-06
XM_008018529.1	3.4222	1.15E-15	1.80E-14	sp|P12643|BMP2_HUMAN Bone morphogenetic protein 2 OS=Homo sapiens GN=BMP2 PE=1 SV=1//0
XM_008018542.1	-1.4602	2.15E-13	2.97E-12	sp|Q15113|PCOC1_HUMAN Procollagen C-endopeptidase enhancer 1 OS=Homo sapiens GN=PCOLCE PE=1 SV=2//0
XM_008018552.1	0.95287	1.01E-28	2.96E-27	sp|Q7L2J0|MEPCE_HUMAN 7SK snRNA methylphosphate capping enzyme OS=Homo sapiens GN=MEPCE PE=1 SV=1//0
XM_008018560.1	-2.0842	1.13E-17	2.00E-16	sp|Q96RP7|G3ST4_HUMAN Galactose-3-O-sulfotransferase 4 OS=Homo sapiens GN=GAL3ST4 PE=1 SV=1//0
XM_008018561.1	-0.76948	6.53E-05	0.0004382	sp|Q8N158|GPC2_HUMAN Glypican-2 OS=Homo sapiens GN=GPC2 PE=2 SV=1//0
XM_008018583.1	-0.98715	7.96E-12	9.93E-11	sp|Q5R483|LTOR4_PONAB Ragulator complex protein LAMTOR4 OS=Pongo abelii GN=LAMTOR4 PE=3 SV=1//1.34602e-63
XM_008018586.1	-1.884	1.54E-28	4.49E-27	sp|Q8N129|CNPY4_HUMAN Protein canopy homolog 4 OS=Homo sapiens GN=CNPY4 PE=2 SV=1//3.28823e-133
XM_008018592.1	-0.43664	7.41E-06	5.56E-05	sp|P49848|TAF6_HUMAN Transcription initiation factor TFIID subunit 6 OS=Homo sapiens GN=TAF6 PE=1 SV=1//0
XM_008018593.1	-0.67281	1.59E-15	2.47E-14	sp|Q5REY0|CSN6_PONAB COP9 signalosome complex subunit 6 OS=Pongo abelii GN=COPS6 PE=2 SV=1//0
XM_008018595.1	1.7424	3.09E-37	1.22E-35	sp|A1YG26|ZSC21_PANPA Zinc finger and SCAN domain-containing protein 21 OS=Pan paniscus GN=ZSCAN21 PE=3 SV=1//0
XM_008018596.1	0.35093	0.0016906	0.0093059	sp|Q5R670|ZKSC1_PONAB Zinc finger protein with KRAB and SCAN domains 1 OS=Pongo abelii GN=ZKSCAN1 PE=2 SV=1//2.10513e-70
XM_008018605.1	-1.5284	1.59E-06	1.28E-05	sp|P25311|ZA2G_HUMAN Zinc-alpha-2-glycoprotein OS=Homo sapiens GN=AZGP1 PE=1 SV=2//0
XM_008018607.1	1.7996	3.78E-85	5.41E-83	sp|Q9C037|TRIM4_HUMAN E3 ubiquitin-protein ligase TRIM4 OS=Homo sapiens GN=TRIM4 PE=1 SV=2//0
XM_008018610.1	8.0956	2.56E-43	1.23E-41	sp|P33268|CP3A8_MACFA Cytochrome P450 3A8 OS=Macaca fascicularis GN=CYP3A8 PE=1 SV=1//0
XM_008018616.1	1.3815	2.61E-06	2.05E-05	sp|P20815|CP3A5_HUMAN Cytochrome P450 3A5 OS=Homo sapiens GN=CYP3A5 PE=1 SV=1//0
XM_008018617.1	2.1728	7.27E-12	9.09E-11	sp|P20815|CP3A5_HUMAN Cytochrome P450 3A5 OS=Homo sapiens GN=CYP3A5 PE=1 SV=1//0
XM_008018648.1	-4.4405	0.0007688	0.0044665	sp|Q5FWF6|ZN789_HUMAN Zinc finger protein 789 OS=Homo sapiens GN=ZNF789 PE=2 SV=3//0
XM_008018651.1	1.6849	4.06E-25	1.05E-23	sp|Q53GI3|ZN394_HUMAN Zinc finger protein 394 OS=Homo sapiens GN=ZNF394 PE=1 SV=2//0
XM_008018653.1	-1.6359	4.30E-21	9.00E-20	sp|Q5R6T5|ATPK_PONAB ATP synthase subunit f, mitochondrial OS=Pongo abelii GN=ATP5J2 PE=3 SV=3//2.31746e-49

XM_008018671.1	-0.39019	2.52E-06	1.99E-05	sp|Q92747|ARC1A_HUMAN Actin-related protein 2/3 complex subunit 1A OS=Homo sapiens GN=ARPC1A PE=1 SV=2//0
XM_008018700.1	3.8866	5.44E-21	1.14E-19	sp|Q7RTS1|BHA15_HUMAN Class A basic helix-loop-helix protein 15 OS=Homo sapiens GN=BHLHA15 PE=1 SV=1//8.85412e-81
XM_008018721.1	-0.76814	0.0030256	0.015858	sp|Q4R8E0|E2AK1_MACFA Eukaryotic translation initiation factor 2-alpha kinase 1 OS=Macaca fascicularis GN=EIF2AK1 PE=2 SV=1//4.68728e-60
XM_008018728.1	0.47518	2.06E-06	1.64E-05	sp|Q4R3X9|F220A_MACFA Protein FAM220A OS=Macaca fascicularis GN=FAM220A PE=2 SV=1//2.11423e-169
XM_008018730.1	-3.8663	0.0095687	0.045007	sp|Q6RUV5|RAC1_RAT Ras-related C3 botulinum toxin substrate 1 OS=Rattus norvegicus GN=Rac1 PE=1 SV=1//2.52144e-116
XM_008018754.1	-0.97592	3.76E-18	6.79E-17	sp|P0CG23|ZN853_HUMAN Zinc finger protein 853 OS=Homo sapiens GN=ZNF853 PE=2 SV=1//6.89373e-21
XM_008018762.1	1.306	1.43E-13	2.00E-12	sp|P17014|ZNF12_HUMAN Zinc finger protein 12 OS=Homo sapiens GN=ZNF12 PE=2 SV=3//0
XM_008018773.1	-0.74458	2.61E-26	6.97E-25	sp|Q16658|FSCN1_HUMAN Fascin OS=Homo sapiens GN=FSCN1 PE=1 SV=3//0
XM_008018774.1	0.67814	1.79E-06	1.44E-05	sp|Q96ME1|FXL18_HUMAN F-box/LRR-repeat protein 18 OS=Homo sapiens GN=FBXL18 PE=1 SV=2//0
XM_008018850.1	-1.1252	2.60E-43	1.25E-41	sp|Q9C0H2|TTYH3_HUMAN Protein tweety homolog 3 OS=Homo sapiens GN=TTYH3 PE=1 SV=3//0
XM_008018851.1	0.53393	8.82E-12	1.10E-10	sp|Q8NES3|LFNG_HUMAN Beta-1,3-N-acetylglucosaminyltransferase lunatic fringe OS=Homo sapiens GN=LFNG PE=1 SV=2//0
XM_008018864.1	-1.1947	4.01E-09	4.04E-08	sp|P36639|8ODP_HUMAN 7,8-dihydro-8-oxoguanine triphosphatase OS=Homo sapiens GN=NUDT1 PE=1 SV=3//1.63054e-112
XM_008018865.1	0.88597	2.14E-17	3.72E-16	sp|Q9Y5X2|SNX8_HUMAN Sorting nexin-8 OS=Homo sapiens GN=SNX8 PE=1 SV=1//0
XM_008018868.1	1.4388	0.0001171	0.0007609	sp|Q9BT73|PSMG3_HUMAN Proteasome assembly chaperone 3 OS=Homo sapiens GN=PSMG3 PE=1 SV=1//1.74393e-77
XM_008018874.1	2.1685	1.43E-10	1.62E-09	sp|O60675|MAFK_HUMAN Transcription factor MafK OS=Homo sapiens GN=MAFK PE=1 SV=1//5.70286e-80
XM_008018885.1	3.7643	2.54E-58	1.90E-56	sp|Q5R966|ZFN2A_PONAB AN1-type zinc finger protein 2A OS=Pongo abelii GN=ZFAND2A PE=3 SV=1//4.32439e-97
XM_008018897.1	-1.5406	6.84E-05	0.0004579	sp|Q8TAV3|CP2W1_HUMAN Cytochrome P450 2W1 OS=Homo sapiens GN=CYP2W1 PE=1 SV=2//0
XM_008018901.1	-0.94262	5.13E-09	5.12E-08	sp|O75689|ADAP1_HUMAN Arf-GAP with dual PH domain-containing protein 1 OS=Homo sapiens GN=ADAP1 PE=1 SV=2//0
XM_008018929.1	-0.69802	1.78E-18	3.27E-17	sp|O95674|CDS2_HUMAN Phosphatidate cytidylyltransferase 2 OS=Homo sapiens GN=CDS2 PE=1 SV=1//0
XM_008018932.1	0.54562	1.69E-10	1.91E-09	sp|Q8IXL6|FA20C_HUMAN Extracellular serine/threonine protein kinase FAM20C OS=Homo sapiens GN=FAM20C PE=1 SV=2//0
XM_008018943.1	1.2842	7.81E-47	4.33E-45	sp|Q9UHL9|GT2D1_HUMAN General transcription factor II-I repeat domain-containing protein 1 OS=Homo sapiens GN=GTF2IRD1 PE=1 SV=1//2.48766e-10
XM_008018956.1	1.0485	1.48E-24	3.67E-23	sp|Q9H0M4|ZCPW1_HUMAN Zinc finger CW-type PWWP domain protein 1 OS=Homo sapiens GN=ZCWPW1 PE=1 SV=2//4.31979e-90
XM_008018957.1	-1.1668	5.41E-49	3.23E-47	sp|O15143|ARC1B_HUMAN Actin-related protein 2/3 complex subunit 1B OS=Homo sapiens GN=ARPC1B PE=1 SV=3//3.58962e-119
XM_008018958.1	1.4529	3.67E-65	3.37E-63	sp|Q9UHR4|BI2L1_HUMAN Brain-specific angiogenesis inhibitor 1-associated protein 2-like protein 1 OS=Homo sapiens GN=BAIAP2L1 PE=1 SV=2//0
XM_008018959.1	-0.49128	1.66E-06	1.33E-05	sp|O95415|BRI3_HUMAN Brain protein I3 OS=Homo sapiens GN=BRI3 PE=2 SV=1//2.4105e-54
XM_008018960.1	0.98352	7.51E-18	1.34E-16	sp|Q96N11|CG026_HUMAN Uncharacterized protein C7orf26 OS=Homo sapiens GN=C7orf26 PE=2 SV=1//0
XM_008018961.1	-1.0358	1.44E-26	3.88E-25	sp|P53505|ACT5_XENLA Actin, cytoplasmic type 5 OS=Xenopus laevis PE=3 SV=1//5.31061e-164
XM_008018962.1	-1.1609	2.09E-20	4.23E-19	sp|P60707|ACTB_TRIVU Actin, cytoplasmic 1 OS=Trichosurus vulpecula GN=ACTB PE=2 SV=1//9.88355e-98
XM_008018966.1	1.313	2.63E-08	2.49E-07	sp|Q9UGH3|S23A2_HUMAN Solute carrier family 23 member 2 OS=Homo sapiens GN=SLC23A2 PE=1 SV=1//0
XM_008018967.1	0.37938	0.0004803	0.0028709	sp|Q6PJG6|BRAT1_HUMAN BRCA1-associated ATM activator 1 OS=Homo sapiens GN=BRAT1 PE=1 SV=2//0
XM_008018968.1	0.41397	2.69E-08	2.54E-07	sp|P55884|EIF3B_HUMAN Eukaryotic translation initiation factor 3 subunit B OS=Homo sapiens GN=EIF3B PE=1 SV=3//0
XM_008019068.1	0.54871	3.68E-10	4.06E-09	sp|P67988|PRIO_CHLAE Major prion protein OS=Chlorocebus aethiops GN=PRNP PE=3 SV=1//2.70449e-94
XM_008019071.1	1.34	1.82E-11	2.22E-10	sp|Q12996|CSTF3_HUMAN Cleavage stimulation factor subunit 3 OS=Homo sapiens GN=CSTF3 PE=1 SV=1//0
XM_008019078.1	-0.8998	9.24E-13	1.22E-11	sp|P11216|PYGB_HUMAN Glycogen phosphorylase, brain form OS=Homo sapiens GN=PYGB PE=1 SV=5//0
XM_008019079.1	0.85284	7.99E-06	5.96E-05	sp|Q8TBE9|NANP_HUMAN N-acylneuraminate-9-phosphatase OS=Homo sapiens GN=NANP PE=1 SV=1//2.74886e-164

XM_008019083.1	0.79788	1.21E-07	1.07E-06	sp|Q9NWM0|SMOX_HUMAN Spermine oxidase OS=Homo sapiens GN=SMOX PE=1 SV=1//4.66137e-80
XM_008019093.1	-1.1014	0.0056601	0.027904	sp|Q9Y2I6|NINL_HUMAN Ninein-like protein OS=Homo sapiens GN=NINL PE=1 SV=2//2.00688e-15
XM_008019102.1	0.75248	1.27E-13	1.77E-12	sp|O75354|ENTP6_HUMAN Ectonucleoside triphosphate diphosphohydrolase 6 OS=Homo sapiens GN=ENTPD6 PE=1 SV=3//0
XM_008019108.1	2.1454	0.0012741	0.0071756	sp|Q13046|PSG7_HUMAN Putative pregnancy-specific beta-1-glycoprotein 7 OS=Homo sapiens GN=PSG7 PE=5 SV=2//0
XM_008019126.1	0.42119	8.42E-08	7.58E-07	sp|Q8NC60|NOA1_HUMAN Nitric oxide-associated protein 1 OS=Homo sapiens GN=NOA1 PE=1 SV=2//0
XM_008019135.1	-1.4835	0.0026446	0.014009	sp|Q76NI1|VKIND_HUMAN Protein very KIND OS=Homo sapiens GN=KNDC1 PE=2 SV=2//0
XM_008019172.1	0.87888	8.64E-08	7.77E-07	sp|Q7L7X3|TAOK1_HUMAN Serine/threonine-protein kinase TAO1 OS=Homo sapiens GN=TAOK1 PE=1 SV=1//3.81062e-107
XM_008019194.1	2.2449	5.40E-15	8.15E-14	sp|Q96EZ4|MYEOV_HUMAN Myeloma-overexpressed gene protein OS=Homo sapiens GN=MYEOV PE=2 SV=2//2.84e-112
XM_008019207.1	-0.33583	0.0016393	0.0090395	sp|Q9BYN8|RT26_HUMAN 28S ribosomal protein S26, mitochondrial OS=Homo sapiens GN=MRPS26 PE=1 SV=1//3.64439e-103
XM_008019208.1	4.1016	2.22E-05	0.0001569	sp|P84239|H3_URECA Histone H3 OS=Urechis caupo PE=1 SV=2//1.47906e-81
XM_008019210.1	1.5437	0.0090137	0.042616	sp|Q64523|H2A2C_MOUSE Histone H2A type 2-C OS=Mus musculus GN=Hist2h2ac PE=1 SV=3//1.8062e-70
XM_008019211.1	3.417	0.004749	0.023825	sp|Q8IUE6|H2A2B_HUMAN Histone H2A type 2-B OS=Homo sapiens GN=HIST2H2AB PE=1 SV=3//2.17808e-68
XM_008019229.1	1.6721	4.79E-34	1.72E-32	sp|Q6GQQ9|OTU7B_HUMAN OTU domain-containing protein 7B OS=Homo sapiens GN=OTUD7B PE=1 SV=1//0
XM_008019235.1	0.9624	4.65E-19	8.81E-18	sp|Q53GL0|PKHO1_HUMAN Pleckstrin homology domain-containing family O member 1 OS=Homo sapiens GN=PLEKHO1 PE=1 SV=2//0
XM_008019241.1	0.57214	1.03E-06	8.48E-06	sp|Q95KD0|FAKD5_MACFA FAST kinase domain-containing protein 5 OS=Macaca fascicularis GN=FASTKD5 PE=2 SV=1//5.55197e-58
XM_008019247.1	-0.69269	1.82E-15	2.81E-14	sp|Q96BI3|APH1A_HUMAN Gamma-secretase subunit APH-1A OS=Homo sapiens GN=APH1A PE=1 SV=1//6.39499e-167
XM_008019256.1	1.5696	4.34E-09	4.36E-08	sp|O43395|PRPF3_HUMAN U4/U6 small nuclear ribonucleoprotein Prp3 OS=Homo sapiens GN=PRPF3 PE=1 SV=2//0
XM_008019267.1	1.6998	3.72E-06	2.88E-05	sp|Q07820|MCL1_HUMAN Induced myeloid leukemia cell differentiation protein Mcl-1 OS=Homo sapiens GN=MCL1 PE=1 SV=3//4.5796e-170
XM_008019283.1	-1.917	8.10E-09	7.98E-08	sp|P61277|CATK_MACMU Cathepsin K OS=Macaca mulatta GN=CTSK PE=1 SV=1//0
XM_008019316.1	0.69169	0.0016498	0.0090908	sp|Q9BUN1|MENT_HUMAN Protein MENT OS=Homo sapiens GN=MENT PE=2 SV=1//5.00363e-163
XM_008019319.1	0.54261	2.06E-11	2.50E-10	sp|Q5VT52|RPRD2_HUMAN Regulation of nuclear pre-mRNA domain-containing protein 2 OS=Homo sapiens GN=RPRD2 PE=1 SV=1//0
XM_008019322.1	0.73157	1.65E-17	2.88E-16	sp|Q15047|SETB1_HUMAN Histone-lysine N-methyltransferase SETDB1 OS=Homo sapiens GN=SETDB1 PE=1 SV=1//0
XM_008019334.1	1.4313	4.52E-36	1.72E-34	sp|O43741|AAKB2_HUMAN 5'-AMP-activated protein kinase subunit beta-2 OS=Homo sapiens GN=PRKAB2 PE=1 SV=1//2.44886e-177
XM_008019359.1	1.311	2.15E-37	8.58E-36	sp|Q4R4U9|SYSC_MACFA Serine--tRNA ligase, cytoplasmic OS=Macaca fascicularis GN=SARS PE=2 SV=3//8.59689e-177
XM_008019365.1	0.44517	0.0025739	0.013664	sp|Q9NRR8|C42S1_HUMAN CDC42 small effector protein 1 OS=Homo sapiens GN=CDC42SE1 PE=1 SV=1//2.86534e-49
XM_008019391.1	-0.88345	2.82E-09	2.88E-08	sp|P12277|KCRB_HUMAN Creatine kinase B-type OS=Homo sapiens GN=CKB PE=1 SV=1//0
XM_008019403.1	-1.247	5.10E-18	9.15E-17	sp|P78552|I13R1_HUMAN Interleukin-13 receptor subunit alpha-1 OS=Homo sapiens GN=IL13RA1 PE=1 SV=1//0
XM_008019405.1	-1.4987	1.80E-07	1.58E-06	sp|P0CG32|ZCC18_HUMAN Zinc finger CCHC domain-containing protein 18 OS=Homo sapiens GN=ZCCHC18 PE=3 SV=1//0
XM_008019413.1	-1.5093	3.34E-79	4.21E-77	sp|Q5RED0|PGRC1_PONAB Membrane-associated progesterone receptor component 1 OS=Pongo abelii GN=PGRMC1 PE=2 SV=3//4.49538e-121
XM_008019417.1	-0.69564	8.92E-05	0.000589	sp|Q8WUT9|S2543_HUMAN Solute carrier family 25 member 43 OS=Homo sapiens GN=SLC25A43 PE=2 SV=2//0
XM_008019418.1	-0.57723	1.03E-11	1.27E-10	sp|P05141|ADT2_HUMAN ADP/ATP translocase 2 OS=Homo sapiens GN=SLC25A5 PE=1 SV=7//0
XM_008019422.1	1.681	1.32E-38	5.48E-37	sp|O15226|NKRF_HUMAN NF-kappa-B-repressing factor OS=Homo sapiens GN=NKRF PE=1 SV=2//0
XM_008019434.1	-1.2702	7.26E-06	5.46E-05	sp|P62893|RL39_RAT 60S ribosomal protein L39 OS=Rattus norvegicus GN=Rpl39 PE=1 SV=2//7.98183e-28
XM_008019438.1	-1.7449	4.03E-19	7.69E-18	sp|Q7JGX4|NDUA1_PANPA NADH dehydrogenase [ubiquinone] 1 alpha subcomplex subunit 1 OS=Pan paniscus GN=NDUFA1 PE=3 SV=1//1.58259e-41
XM_008019454.1	-1.8565	4.33E-11	5.12E-10	sp|P13473|LAMP2_HUMAN Lysosome-associated membrane glycoprotein 2 OS=Homo sapiens GN=LAMP2 PE=1 SV=2//0
XM_008019455.1	-1.3739	0.0067503	0.032763	sp|P13473|LAMP2_HUMAN Lysosome-associated membrane glycoprotein 2 OS=Homo sapiens GN=LAMP2 PE=1 SV=2//0

XM_008019456.1	-1.0737	8.04E-09	7.93E-08	sp|P13473|LAMP2_HUMAN Lysosome-associated membrane glycoprotein 2 OS=Homo sapiens GN=LAMP2 PE=1 SV=2//0
XM_008019509.1	-0.53335	0.0002852	0.0017541	sp|O75882|ATRN_HUMAN Attractin OS=Homo sapiens GN=ATRN PE=1 SV=2//0
XM_008019564.1	-1.5859	0.0037356	0.019153	sp|Q96MM6|HS12B_HUMAN Heat shock 70 kDa protein 12B OS=Homo sapiens GN=HSPA12B PE=1 SV=2//0
XM_008019565.1	-0.61529	0.000776	0.0045022	sp|Q6PDV7|RL10_RAT 60S ribosomal protein L10 OS=Rattus norvegicus GN=Rpl10 PE=1 SV=3//1.48446e-156
XM_008019580.1	-0.71448	1.19E-19	2.32E-18	sp|Q15904|VAS1_HUMAN V-type proton ATPase subunit S1 OS=Homo sapiens GN=ATP6AP1 PE=1 SV=2//0
XM_008019587.1	1.1114	4.61E-06	3.54E-05	sp|P17010|ZFX_HUMAN Zinc finger X-chromosomal protein OS=Homo sapiens GN=ZFX PE=2 SV=2//2.60452e-75
XM_008019598.1	-0.57363	0.0060665	0.029734	sp|Q5NVF5|DYLT3_PONAB Dynein light chain Tctex-type 3 OS=Pongo abelii GN=DYNLT3 PE=3 SV=1//7.5152e-69
XM_008019600.1	0.54249	0.0018918	0.01029	sp|P51811|XK_HUMAN Membrane transport protein XK OS=Homo sapiens GN=XK PE=1 SV=5//0
XM_008019607.1	-1.3761	9.33E-06	6.90E-05	sp|P78539|SRPX_HUMAN Sushi repeat-containing protein SRPX OS=Homo sapiens GN=SRPX PE=1 SV=1//7.74588e-57
XM_008019639.1	-0.28362	1.31E-05	9.52E-05	sp|P39023|RL3_HUMAN 60S ribosomal protein L3 OS=Homo sapiens GN=RPL3 PE=1 SV=2//0
XM_008019641.1	-1.2591	0.0029249	0.015375	sp|Q96N66|MBOA7_HUMAN Lysophospholipid acyltransferase 7 OS=Homo sapiens GN=MBOAT7 PE=1 SV=2//6.60727e-73
XM_008019643.1	0.92095	1.39E-06	1.12E-05	sp|O15014|ZN609_HUMAN Zinc finger protein 609 OS=Homo sapiens GN=ZNF609 PE=1 SV=2//0
XM_008019660.1	-0.43494	1.86E-10	2.10E-09	sp|P12236|ADT3_HUMAN ADP/ATP translocase 3 OS=Homo sapiens GN=SLC25A6 PE=1 SV=4//0
XM_008019664.1	-1.2466	1.85E-12	2.41E-11	sp|O95671|ASML_HUMAN N-acetylserotonin O-methyltransferase-like protein OS=Homo sapiens GN=ASMTL PE=1 SV=3//0
XM_008019666.1	1.5747	2.96E-16	4.82E-15	sp|Q02040|AK17A_HUMAN A-kinase anchor protein 17A OS=Homo sapiens GN=AKAP17A PE=1 SV=2//5.23759e-93
XM_008019699.1	1.098	3.12E-20	6.25E-19	sp|Q5R9I4|TV23B_PONAB Golgi apparatus membrane protein TVP23 homolog B OS=Pongo abelii GN=TVP23B PE=2 SV=1//2.39299e-141
XM_008019702.1	-1.3034	0.0001018	0.0006673	sp|Q9UKP4|ATS7_HUMAN A disintegrin and metalloproteinase with thrombospondin motifs 7 OS=Homo sapiens GN=ADAMTS7 PE=1 SV=2//5.43028e-08
XM_008019703.1	-1.386	2.48E-11	2.99E-10	sp|Q9UKP4|ATS7_HUMAN A disintegrin and metalloproteinase with thrombospondin motifs 7 OS=Homo sapiens GN=ADAMTS7 PE=1 SV=2//4.15159e-17
XM_008019730.1	0.29011	0.010565	0.049207	sp|O15530|PDPK1_HUMAN 3-phosphoinositide-dependent protein kinase 1 OS=Homo sapiens GN=PDPK1 PE=1 SV=1//0
XM_008019736.1	-0.42418	0.0050882	0.025373	sp|Q7Z434|MAVS_HUMAN Mitochondrial antiviral-signaling protein OS=Homo sapiens GN=MAVS PE=1 SV=2//0
XM_008019741.1	1.4677	1.67E-37	6.71E-36	sp|Q9BZ23|PANK2_HUMAN Pantothenate kinase 2, mitochondrial OS=Homo sapiens GN=PANK2 PE=1 SV=3//0
XM_008019760.1	0.43617	6.12E-05	0.0004111	sp|P86791|CCZ1_HUMAN Vacuolar fusion protein CCZ1 homolog OS=Homo sapiens GN=CCZ1 PE=1 SV=1//0
XM_008019770.1	2.3532	0.007692	0.036885	sp|P11465|PSG2_HUMAN Pregnancy-specific beta-1-glycoprotein 2 OS=Homo sapiens GN=PSG2 PE=2 SV=2//2.46606e-146
XM_008019771.1	4.16	0.0036977	0.018968	sp|Q15238|PSG5_HUMAN Pregnancy-specific beta-1-glycoprotein 5 OS=Homo sapiens GN=PSG5 PE=1 SV=3//6.79923e-119
XM_008019788.1	0.57615	1.62E-08	1.56E-07	sp|Q9NUD5|ZCHC3_HUMAN Zinc finger CCHC domain-containing protein 3 OS=Homo sapiens GN=ZCCHC3 PE=1 SV=1//1.06477e-179
XM_008019794.1	-1.3693	3.18E-09	3.23E-08	sp|O60218|AK1BA_HUMAN Aldo-keto reductase family 1 member B10 OS=Homo sapiens GN=AKR1B10 PE=1 SV=2//2.12154e-09
XM_008019798.1	1.0845	1.24E-05	9.07E-05	sp|Q9P2J8|ZN624_HUMAN Zinc finger protein 624 OS=Homo sapiens GN=ZNF624 PE=1 SV=3//4.06531e-115
XM_008019805.1	Inf	6.88E-05	0.0004604	sp|Q13046|PSG7_HUMAN Putative pregnancy-specific beta-1-glycoprotein 7 OS=Homo sapiens GN=PSG7 PE=5 SV=2//5.40625e-64
XM_008019812.1	3.4709	5.90E-37	2.30E-35	sp|P84239|H3_URECA Histone H3 OS=Urechis caupo PE=1 SV=2//3.00721e-89
XM_008019816.1	-1.2173	4.16E-30	1.29E-28	sp|Q04890|SOX12_MOUSE Transcription factor SOX-12 OS=Mus musculus GN=Sox12 PE=2 SV=2//2.6215e-33
XM_008019817.1	2.5621	4.83E-08	4.44E-07	sp|Q13046|PSG7_HUMAN Putative pregnancy-specific beta-1-glycoprotein 7 OS=Homo sapiens GN=PSG7 PE=5 SV=2//0
XM_008019828.1	1.8073	6.42E-07	5.35E-06	sp|Q6PK81|ZN773_HUMAN Zinc finger protein 773 OS=Homo sapiens GN=ZNF773 PE=2 SV=1//1.42591e-35
XM_008019831.1	3.7886	5.20E-232	3.26E-229	sp|Q96RU7|TRIB3_HUMAN Tribbles homolog 3 OS=Homo sapiens GN=TRIB3 PE=1 SV=2//0
XM_008019845.1	1.1826	2.73E-06	2.14E-05	sp|Q9BVS4|RIOK2_HUMAN Serine/threonine-protein kinase RIO2 OS=Homo sapiens GN=RIOK2 PE=1 SV=2//2.33716e-38
XM_008019846.1	2.7091	1.53E-63	1.33E-61	sp|Q96QB1|RHG07_HUMAN Rho GTPase-activating protein 7 OS=Homo sapiens GN=DLC1 PE=1 SV=4//0

XM_008019852.1	-5.1149	1.54E-06	1.24E-05	sp|Q9UKK3|PARP4_HUMAN Poly [ADP-ribose] polymerase 4 OS=Homo sapiens GN=PARP4 PE=1 SV=3//8.36177e-10
XM_008019890.1	-1.1052	1.89E-08	1.81E-07	sp|Q9C030|TRIM6_HUMAN Tripartite motif-containing protein 6 OS=Homo sapiens GN=TRIM6 PE=1 SV=1//0
XM_008019969.1	-1.2595	1.72E-11	2.10E-10	sp|Q9UHJ9|PGAP2_HUMAN Post-GPI attachment to proteins factor 2 OS=Homo sapiens GN=PGAP2 PE=1 SV=2//2.28781e-27
XM_008020015.1	-1.4479	9.18E-27	2.49E-25	sp|P15328|FOLR1_HUMAN Folate receptor alpha OS=Homo sapiens GN=FOLR1 PE=1 SV=3//4.9029e-151
XM_008020022.1	0.70206	1.62E-07	1.42E-06	sp|P41134|ID1_HUMAN DNA-binding protein inhibitor ID-1 OS=Homo sapiens GN=ID1 PE=1 SV=3//1.28359e-77
XM_008020025.1	-0.82435	2.05E-06	1.63E-05	sp|Q9NZE8|RM35_HUMAN 39S ribosomal protein L35, mitochondrial OS=Homo sapiens GN=MRPL35 PE=1 SV=3//7.20331e-117
XM_008020113.1	-0.71193	8.77E-09	8.62E-08	sp|Q96GC5|RM48_HUMAN 39S ribosomal protein L48, mitochondrial OS=Homo sapiens GN=MRPL48 PE=1 SV=2//2.75197e-117
XM_008020122.1	#NAME?	0.0006406	0.0037658	sp|P59910|DJB13_HUMAN DnaJ homolog subfamily B member 13 OS=Homo sapiens GN=DNAJB13 PE=2 SV=1//0
XM_008020123.1	-3.1937	0.0092075	0.043467	sp|P55851|UCP2_HUMAN Mitochondrial uncoupling protein 2 OS=Homo sapiens GN=UCP2 PE=1 SV=1//0
XM_008020140.1	0.75206	4.48E-05	0.0003055	sp|Q15054|DPOD3_HUMAN DNA polymerase delta subunit 3 OS=Homo sapiens GN=POLD3 PE=1 SV=2//0
XM_008020153.1	1.7583	6.46E-44	3.17E-42	sp|Q8NCN4|RN169_HUMAN E3 ubiquitin-protein ligase RNF169 OS=Homo sapiens GN=RNF169 PE=1 SV=2//0
XM_008020170.1	-0.61784	2.89E-17	4.97E-16	sp|Q0Z8U2|RS3_PIG 40S ribosomal protein S3 OS=Sus scrofa GN=RPS3 PE=1 SV=1//1.63136e-175
XM_008020171.1	0.89729	8.42E-26	2.22E-24	sp|Q9NP50|FA60A_HUMAN Protein FAM60A OS=Homo sapiens GN=FAM60A PE=1 SV=1//1.31108e-132
XM_008020186.1	-1.4902	0.0010312	0.0058788	sp|Q96JE9|MAP6_HUMAN Microtubule-associated protein 6 OS=Homo sapiens GN=MAP6 PE=1 SV=2//0
XM_008020188.1	1.8733	1.01E-66	9.61E-65	sp|Q9P2Y5|UVRAG_HUMAN UV radiation resistance-associated gene protein OS=Homo sapiens GN=UVRAG PE=1 SV=1//0
XM_008020195.1	-2.1462	1.23E-15	1.93E-14	sp|O43638|FOXS1_HUMAN Forkhead box protein S1 OS=Homo sapiens GN=FOXS1 PE=2 SV=2//3.96415e-178
XM_008020197.1	-1.1274	0.0023635	0.012645	sp|Q3ZCU0|YK006_HUMAN Putative uncharacterized protein FLJ37770 OS=Homo sapiens PE=5 SV=1//3.15487e-166
XM_008020214.1	1.5554	3.35E-13	4.55E-12	sp|Q9NUG6|PDRG1_HUMAN p53 and DNA damage-regulated protein 1 OS=Homo sapiens GN=PDRG1 PE=1 SV=2//4.35078e-67
XM_008020217.1	-1.524	0.000707	0.0041268	-//-
XM_008020218.1	-1.6695	3.62E-42	1.69E-40	sp|Q8WUA8|TSK_HUMAN Tsukushin OS=Homo sapiens GN=TSKU PE=2 SV=3//0
XM_008020222.1	-0.71362	1.09E-09	1.15E-08	sp|O15484|CAN5_HUMAN Calpain-5 OS=Homo sapiens GN=CAPN5 PE=1 SV=2//0
XM_008020251.1	-0.34551	0.001107	0.0062879	sp|P54105|ICLN_HUMAN Methylosome subunit pICln OS=Homo sapiens GN=CLNS1A PE=1 SV=1//2.68656e-133
XM_008020259.1	0.54802	6.77E-11	7.86E-10	sp|Q96T23|RSF1_HUMAN Remodeling and spacing factor 1 OS=Homo sapiens GN=RSF1 PE=1 SV=2//0
XM_008020261.1	-1.9428	0.0001241	0.0008035	sp|Q92748|THRSP_HUMAN Thyroid hormone-inducible hepatic protein OS=Homo sapiens GN=THRSP PE=1 SV=1//5.7529e-65
XM_008020262.1	-0.84233	1.19E-09	1.25E-08	sp|Q8SPI4|NDUC2_MACFA NADH dehydrogenase [ubiquinone] 1 subunit C2 OS=Macaca fascicularis GN=NDUFC2 PE=3 SV=1//2.52155e-70
XM_008020421.1	0.7456	0.0004639	0.0027822	sp|Q9H489|TSY26_HUMAN Putative testis-specific Y-encoded-like protein 3 OS=Homo sapiens GN=TSPY26P PE=5 SV=1//4.68601e-170
XM_008020433.1	1.1662	2.75E-32	9.16E-31	sp|Q9UPG8|PLAL2_HUMAN Zinc finger protein PLAGL2 OS=Homo sapiens GN=PLAGL2 PE=2 SV=1//0
XM_008020443.1	0.64087	1.88E-12	2.44E-11	sp|Q1WK23|PRS23_MACMU Serine protease 23 OS=Macaca mulatta GN=PRSS23 PE=2 SV=1//0
XM_008020444.1	-0.77903	2.22E-07	1.92E-06	sp|Q9ULV1|FZD4_HUMAN Frizzled-4 OS=Homo sapiens GN=FZD4 PE=1 SV=2//0
XM_008020456.1	-1.8978	1.12E-46	6.19E-45	sp|Q60HG6|CATC_MACFA Dipeptidyl peptidase 1 OS=Macaca fascicularis GN=CTSC PE=2 SV=1//0
XM_008020457.1	-1.8311	1.21E-05	8.82E-05	sp|Q60HG6|CATC_MACFA Dipeptidyl peptidase 1 OS=Macaca fascicularis GN=CTSC PE=2 SV=1//3.89572e-56
XM_008020466.1	1.0918	0.0011849	0.0067063	sp|Q5R5C5|NOX4_PONAB NADPH oxidase 4 OS=Pongo abelii GN=NOX4 PE=2 SV=2//0
XM_008020478.1	-0.66859	5.54E-08	5.07E-07	sp|Q7Z2W9|RM21_HUMAN 39S ribosomal protein L21, mitochondrial OS=Homo sapiens GN=MRPL21 PE=1 SV=2//1.0649e-121
XM_008020481.1	-1.112	0.001438	0.0080144	sp|Q8HXQ0|SODC_MACMU Superoxide dismutase [Cu-Zn] OS=Macaca mulatta GN=SOD1 PE=2 SV=3//3.37374e-105
XM_008020490.1	1.1283	2.24E-21	4.77E-20	sp|Q4R7U2|CHRD1_MACFA Cysteine and histidine-rich domain-containing protein 1 OS=Macaca fascicularis GN=CHORDC1 PE=2 SV=1//1.66989e-14
XM_008020544.1	1.4343	1.21E-13	1.70E-12	sp|Q9NVC6|MED17_HUMAN Mediator of RNA polymerase II transcription subunit 17 OS=Homo sapiens GN=MED17 PE=1 SV=2//0
XM_008020554.1	1.1654	3.46E-06	2.69E-05	sp|Q96RD7|PANX1_HUMAN Pannexin-1 OS=Homo sapiens GN=PANX1 PE=1 SV=4//0

XM_008020579.1	1.5341	2.58E-08	2.45E-07	sp|Q659K9|FUT4_PANTR Alpha-(1,3)-fucosyltransferase 4 OS=Pan troglodytes GN=FUT4 PE=2 SV=2//0
XM_008020594.1	1.2473	1.89E-32	6.29E-31	sp|Q9BRL6|SRSF8_HUMAN Serine/arginine-rich splicing factor 8 OS=Homo sapiens GN=SRSF8 PE=1 SV=1//1.7711e-61
XM_008020597.1	-1.3408	1.97E-06	1.57E-05	sp|P58005|SESN3_HUMAN Sestrin-3 OS=Homo sapiens GN=SESN3 PE=2 SV=2//0
XM_008020604.1	0.76029	6.40E-07	5.34E-06	sp|Q5HYJ3|FA76B_HUMAN Protein FAM76B OS=Homo sapiens GN=FAM76B PE=1 SV=3//0
XM_008020652.1	#NAME?	0.0004412	0.0026529	sp|Q9UBC3|DNM3B_HUMAN DNA (cytosine-5)-methyltransferase 3B OS=Homo sapiens GN=DNMT3B PE=1 SV=1//0
XM_008020653.1	2.2633	1.92E-07	1.67E-06	sp|Q13489|BIRC3_HUMAN Baculoviral IAP repeat-containing protein 3 OS=Homo sapiens GN=BIRC3 PE=1 SV=2//0
XM_008020665.1	-1.1022	5.27E-22	1.16E-20	sp|P09237|MMP7_HUMAN Matrilysin OS=Homo sapiens GN=MMP7 PE=1 SV=1//0
XM_008020707.1	1.3289	1.68E-36	6.46E-35	sp|P38935|SMBP2_HUMAN DNA-binding protein SMUBP-2 OS=Homo sapiens GN=IGHMBP2 PE=1 SV=3//0
XM_008020716.1	1.035	2.08E-18	3.82E-17	sp|Q9NRN7|ADPPT_HUMAN L-aminoadipate-semialdehyde dehydrogenase-phosphopantetheinyl transferase OS=Homo sapiens GN=AASDHPPT PE=1 SV=2//0
XM_008020723.1	-0.92109	3.52E-09	3.56E-08	-//-
XM_008020740.1	2.4419	0.0028036	0.014801	sp|Q8IXU6|S35F2_HUMAN Solute carrier family 35 member F2 OS=Homo sapiens GN=SLC35F2 PE=1 SV=1//0
XM_008020745.1	0.67326	3.99E-11	4.74E-10	sp|Q93034|CUL5_HUMAN Cullin-5 OS=Homo sapiens GN=CUL5 PE=1 SV=4//0
XM_008020746.1	-0.75211	7.45E-13	9.90E-12	sp|P24752|THIL_HUMAN Acetyl-CoA acetyltransferase, mitochondrial OS=Homo sapiens GN=ACAT1 PE=1 SV=1//0
XM_008020754.1	0.58065	5.27E-05	0.0003566	sp|Q14207|NPAT_HUMAN Protein NPAT OS=Homo sapiens GN=NPAT PE=1 SV=3//0
XM_008020770.1	1.8187	4.65E-72	4.97E-70	sp|Q13206|DDX10_HUMAN Probable ATP-dependent RNA helicase DDX10 OS=Homo sapiens GN=DDX10 PE=1 SV=2//0
XM_008020776.1	1.6953	1.21E-06	9.81E-06	sp|Q9C0D7|ZC12C_HUMAN Probable ribonuclease ZC3H12C OS=Homo sapiens GN=ZC3H12C PE=1 SV=2//0
XM_008020817.1	1.5418	3.91E-05	0.0002683	sp|P30154|2AAB_HUMAN Serine/threonine-protein phosphatase 2A 65 kDa regulatory subunit A beta isoform OS=Homo sapiens GN=PPP2R1B PE=1 SV=3//8.14987e-15
XM_008020827.1	-0.82238	0.010269	0.047942	sp|Q9H6U8|ALG9_HUMAN Alpha-1,2-mannosyltransferase ALG9 OS=Homo sapiens GN=ALG9 PE=1 SV=2//0
XM_008020886.1	-1.2381	1.10E-15	1.72E-14	sp|P13591|NCAM1_HUMAN Neural cell adhesion molecule 1 OS=Homo sapiens GN=NCAM1 PE=1 SV=3//0
XM_008020970.1	-1.418	0.0005819	0.0034438	sp|Q6Q788|APOA5_HUMAN Apolipoprotein A-V OS=Homo sapiens GN=APOA5 PE=1 SV=1//0
XM_008020973.1	0.99368	2.65E-06	2.08E-05	sp|A9CB27|ZPR1_PAPAN Zinc finger protein ZPR1 OS=Papio anubis GN=ZNF259 PE=3 SV=1//0
XM_008020996.1	-0.88747	2.00E-09	2.07E-08	sp|P56817|BACE1_HUMAN Beta-secretase 1 OS=Homo sapiens GN=BACE1 PE=1 SV=2//0
XM_008021005.1	0.79087	4.72E-05	0.0003212	sp|Q01995|TAGL_HUMAN Transgelin OS=Homo sapiens GN=TAGLN PE=1 SV=4//4.96801e-145
XM_008021023.1	-3.6629	2.81E-16	4.58E-15	sp|P54710|ATNG_HUMAN Sodium/potassium-transporting ATPase subunit gamma OS=Homo sapiens GN=FXYD2 PE=1 SV=3//4.22426e-28
XM_008021036.1	-3.7851	3.99E-06	3.09E-05	sp|Q8IWT1|SCN4B_HUMAN Sodium channel subunit beta-4 OS=Homo sapiens GN=SCN4B PE=1 SV=1//1.31597e-108
XM_008021045.1	-1.0226	0.0047199	0.023707	sp|O60487|MPZL2_HUMAN Myelin protein zero-like protein 2 OS=Homo sapiens GN=MPZL2 PE=1 SV=1//1.27369e-147
XM_008021063.1	-0.71533	1.11E-08	1.09E-07	sp|O75964|ATP5L_HUMAN ATP synthase subunit g, mitochondrial OS=Homo sapiens GN=ATP5L PE=1 SV=3//8.50356e-68
XM_008021116.1	-0.94059	0.000589	0.0034799	sp|Q86UU1|PHLB1_HUMAN Pleckstrin homology-like domain family B member 1 OS=Homo sapiens GN=PHLDB1 PE=1 SV=1//0
XM_008021122.1	0.53348	0.0001105	0.0007209	sp|Q01094|E2F1_HUMAN Transcription factor E2F1 OS=Homo sapiens GN=E2F1 PE=1 SV=1//0
XM_008021129.1	-1.1715	3.22E-12	4.12E-11	sp|Q6Q311|RS25_SHEEP 40S ribosomal protein S25 OS=Ovis aries GN=RPS25 PE=2 SV=1//9.04604e-45
XM_008021190.1	1.447	3.58E-22	7.98E-21	sp|P43121|MUC18_HUMAN Cell surface glycoprotein MUC18 OS=Homo sapiens GN=MCAM PE=1 SV=2//0
XM_008021193.1	-0.8048	3.09E-13	4.21E-12	sp|Q9BY78|RNF26_HUMAN RING finger protein 26 OS=Homo sapiens GN=RNF26 PE=2 SV=1//0
XM_008021205.1	2.8535	2.03E-11	2.47E-10	sp|O75604|UBP2_HUMAN Ubiquitin carboxyl-terminal hydrolase 2 OS=Homo sapiens GN=USP2 PE=1 SV=2//0
XM_008021206.1	-0.59192	7.87E-13	1.05E-11	sp|Q9H444|CHM4B_HUMAN Charged multivesicular body protein 4b OS=Homo sapiens GN=CHMP4B PE=1 SV=1//4.65068e-94
XM_008021207.1	-1.7523	1.25E-05	9.15E-05	sp|Q15223|PVRL1_HUMAN Nectin-1 OS=Homo sapiens GN=PVRL1 PE=1 SV=3//0
XM_008021209.1	-0.86695	2.07E-05	0.0001465	sp|Q86UD1|OAF_HUMAN Out at first protein homolog OS=Homo sapiens GN=OAF PE=2 SV=1//2.21435e-165
XM_008021238.1	-0.47134	4.87E-06	3.73E-05	sp|O75845|SC5D_HUMAN Lathosterol oxidase OS=Homo sapiens GN=SC5D PE=1

				SV=2//0
XM_008021240.1	-1.9945	4.02E-09	4.05E-08	sp|Q92673|SORL_HUMAN Sortilin-related receptor OS=Homo sapiens GN=SORL1 PE=1 SV=2//0
XM_008021336.1	0.53465	0.0012815	0.0072137	sp|Q3YBR2|TBRG1_HUMAN Transforming growth factor beta regulator 1 OS=Homo sapiens GN=TBRG1 PE=1 SV=1//0
XM_008021337.1	-1.7942	0.0018133	0.0099094	sp|Q9HAT2|SIAE_HUMAN Sialate O-acetylesterase OS=Homo sapiens GN=SIAE PE=1 SV=1//0
XM_008021390.1	0.36052	4.28E-06	3.30E-05	sp|Q5RCE2|STT3A_PONAB Dolichyl-diphosphooligosaccharide--protein glycosyltransferase subunit STT3A OS=Pongo abelii GN=STT3A PE=2 SV=1//0
XM_008021429.1	1.5176	0.0001792	0.0011346	sp|Q96CM3|RUSD4_HUMAN RNA pseudouridylate synthase domain-containing protein 4 OS=Homo sapiens GN=RPUSD4 PE=2 SV=1//0
XM_008021443.1	0.44705	0.0008835	0.0050858	sp|Q96C86|DCPS_HUMAN m7GpppX diphosphatase OS=Homo sapiens GN=DCPS PE=1 SV=2//0
XM_008021529.1	2.6666	2.41E-12	3.11E-11	sp|Q9Y5Y6|ST14_HUMAN Suppressor of tumorigenicity 14 protein OS=Homo sapiens GN=ST14 PE=1 SV=2//0
XM_008021532.1	2.8384	1.61E-146	5.20E-144	sp|Q8TE58|ATS15_HUMAN A disintegrin and metalloproteinase with thrombospondin motifs 15 OS=Homo sapiens GN=ADAMTS15 PE=2 SV=1//0
XM_008021535.1	0.40838	6.56E-06	4.95E-05	sp|Q92543|SNX19_HUMAN Sorting nexin-19 OS=Homo sapiens GN=SNX19 PE=1 SV=2//0
XM_008021574.1	-1.4908	3.87E-16	6.26E-15	sp|Q9BX67|JAM3_HUMAN Junctional adhesion molecule C OS=Homo sapiens GN=JAM3 PE=1 SV=1//0
XM_008021580.1	-0.93975	5.93E-11	6.93E-10	sp|Q9P016|THYN1_HUMAN Thymocyte nuclear protein 1 OS=Homo sapiens GN=THYN1 PE=1 SV=1//8.77226e-164
XM_008021581.1	-0.49836	3.05E-05	0.0002117	sp|Q4G0F5|VP26B_HUMAN Vacuolar protein sorting-associated protein 26B OS=Homo sapiens GN=VPS26B PE=1 SV=2//0
XM_008021608.1	0.64096	7.21E-05	0.0004815	sp|Q9R283|TRPC2_RAT Short transient receptor potential channel 2 OS=Rattus norvegicus GN=Trpc2 PE=2 SV=2//4.54606e-78
XM_008021610.1	0.96038	3.23E-22	7.22E-21	sp|Q4AC94|C2CD3_HUMAN C2 domain-containing protein 3 OS=Homo sapiens GN=C2CD3 PE=1 SV=4//0
XM_008021611.1	1.546	3.16E-30	9.86E-29	sp|Q96I59|SYNM_HUMAN Probable asparagine--tRNA ligase, mitochondrial OS=Homo sapiens GN=NARS2 PE=1 SV=3//0
XM_008021622.1	0.77031	2.79E-12	3.58E-11	sp|O43264|ZW10_HUMAN Centromere/kinetochore protein zw10 homolog OS=Homo sapiens GN=ZW10 PE=1 SV=3//0
XM_008021635.1	-0.48456	1.05E-07	9.35E-07	sp|Q5RDT5|SAP18_PONAB Histone deacetylase complex subunit SAP18 OS=Pongo abelii GN=SAP18 PE=2 SV=1//9.35496e-95
XM_008021636.1	2.7075	3.46E-238	2.30E-235	sp|Q9NRM7|LATS2_HUMAN Serine/threonine-protein kinase LATS2 OS=Homo sapiens GN=LATS2 PE=1 SV=2//0
XM_008021685.1	0.44506	1.01E-08	9.83E-08	sp|O94906|PRP6_HUMAN Pre-mRNA-processing factor 6 OS=Homo sapiens GN=PRPF6 PE=1 SV=1//0
XM_008021691.1	-1.9215	0.0001595	0.001017	sp|Q4R6N0|TPTE2_MACFA Phosphatidylinositol 3,4,5-trisphosphate 3-phosphatase TPTE2 OS=Macaca fascicularis GN=TPTE2 PE=2 SV=1//0
XM_008021708.1	2.1876	0.0009463	0.0054228	sp|P31371|FGF9_HUMAN Fibroblast growth factor 9 OS=Homo sapiens GN=FGF9 PE=1 SV=3//5.5323e-127
XM_008021709.1	0.58585	2.59E-07	2.22E-06	sp|Q5RE34|RTEL1_PONAB Regulator of telomere elongation helicase 1 OS=Pongo abelii GN=RTEL1 PE=2 SV=1//0
XM_008021748.1	-2.5396	0.0058475	0.028744	sp|Q9BXT8|RNF17_HUMAN RING finger protein 17 OS=Homo sapiens GN=RNF17 PE=1 SV=3//0
XM_008021792.1	1.4531	1.03E-56	7.31E-55	sp|O75317|UBP12_HUMAN Ubiquitin carboxyl-terminal hydrolase 12 OS=Homo sapiens GN=USP12 PE=1 SV=2//0
XM_008021796.1	0.35673	0.0007714	0.0044801	sp|Q6T310|RSLBA_HUMAN Ras-like protein family member 11A OS=Homo sapiens GN=RASL11A PE=2 SV=1//3.06489e-158
XM_008021797.1	2.5791	4.62E-75	5.22E-73	sp|Q9P2E3|ZNFX1_HUMAN NFX1-type zinc finger-containing protein 1 OS=Homo sapiens GN=ZNFX1 PE=2 SV=2//0
XM_008021801.1	0.72737	2.99E-07	2.56E-06	sp|Q92664|TF3A_HUMAN Transcription factor IIIA OS=Homo sapiens GN=GTF3A PE=1 SV=3//0
XM_008021802.1	1.1669	2.81E-21	5.95E-20	sp|Q8N448|LNX2_HUMAN Ligand of Numb protein X 2 OS=Homo sapiens GN=LNX2 PE=1 SV=1//0
XR_489486.1	2.2741	5.27E-10	5.72E-09	-//-
XR_489517.1	#NAME?	0.0046296	0.023301	-//-
XR_489608.1	3.9918	0.0001232	0.0007986	-//-
XR_489616.1	1.2459	0.0051927	0.025827	sp|Q6UX73|CP089_HUMAN UPF0764 protein C16orf89 OS=Homo sapiens GN=C16orf89 PE=1 SV=2//9.53473e-06
XR_489808.1	3.7061	2.70E-08	2.55E-07	-//-
XR_489809.1	2.0712	7.48E-08	6.77E-07	-//-
XR_489852.1	-4.3829	0.000545	0.0032427	-//-
XR_489890.1	-1.8506	2.42E-09	2.49E-08	-//-
XR_489934.1	-2.3919	0.0002207	0.0013775	-//-
XR_490208.1	-0.90816	2.12E-07	1.84E-06	sp|P00390|GSHR_HUMAN Glutathione reductase, mitochondrial OS=Homo

				sapiens GN=GSR PE=1 SV=2//4.53879e-29
XR_490233.1	4.1911	6.39E-06	4.83E-05	-//-
XR_490236.1	1.0546	1.51E-11	1.86E-10	-//-
XR_490251.1	1.8888	8.41E-05	0.0005573	-//-
XR_490301.1	-2.5235	9.50E-05	0.0006259	-//-
XR_490344.1	1.1051	9.30E-07	7.66E-06	sp|Q14692|BMS1_HUMAN Ribosome biogenesis protein BMS1 homolog OS=Homo sapiens GN=BMS1 PE=1 SV=1//1.28965e-06
XR_490359.1	-0.85317	3.09E-05	0.0002145	sp|Q5I0H3|SUMO1_RAT Small ubiquitin-related modifier 1 OS=Rattus norvegicus GN=Sumo1 PE=1 SV=1//1.46738e-52
XR_490362.1	2.1731	0.0098587	0.046256	-//-
XR_490376.1	-0.63916	0.0001108	0.0007224	sp|Q9HDC9|APMAP_HUMAN Adipocyte plasma membrane-associated protein OS=Homo sapiens GN=APMAP PE=1 SV=2//3.24007e-30
XR_490377.1	-2.1596	3.18E-09	3.23E-08	-//-
XR_490385.1	-1.4605	0.0013841	0.0077454	-//-
XR_490428.1	0.79042	0.0002682	0.0016553	sp|P16260|GDC_HUMAN Graves disease carrier protein OS=Homo sapiens GN=SLC25A16 PE=1 SV=3//1.90425e-28
XR_490441.1	-1.7854	6.59E-06	4.97E-05	-//-
XR_490451.1	-3.8293	3.40E-08	3.17E-07	-//-
XR_490492.1	4.5656	0.0004145	0.0025035	-//-
XR_490604.1	-4.095	0.0043531	0.022046	-//-
XR_490633.1	1.3811	0.0025583	0.013591	-//-
XR_490634.1	-0.63389	3.63E-08	3.38E-07	-//-
XR_490635.1	-0.95734	2.67E-09	2.73E-08	-//-
XR_490662.1	-1.7186	4.68E-05	0.0003187	-//-
XR_490765.1	-0.45369	0.0014283	0.0079702	-//-
XR_490791.1	Inf	0.0051904	0.025821	-//-
XR_490828.1	Inf	0.0051343	0.025565	-//-
XR_490887.1	-0.75805	0.0033386	0.01732	-//-
XR_490927.1	#NAME?	0.010303	0.04809	-//-
XR_490929.1	-3.6635	1.73E-07	1.52E-06	-//-
XR_490964.1	-0.84452	9.70E-06	7.16E-05	-//-
XR_491014.1	-2.2809	4.29E-11	5.07E-10	sp|Q9P1C3|YN010_HUMAN Putative uncharacterized protein PRO2829 OS=Homo sapiens GN=PRO2829 PE=5 SV=1//1.72198e-11
XR_491129.1	4.2129	0.0057018	0.028085	-//-
XR_491248.1	-1.3799	0.0047438	0.023805	-//-
XR_491289.1	2.5685	4.39E-14	6.29E-13	-//-
XR_491332.1	1.1506	0.0002573	0.0015926	sp|P52742|ZN135_HUMAN Zinc finger protein 135 OS=Homo sapiens GN=ZNF135 PE=2 SV=3//7.7392e-06
XR_491361.1	6.5898	2.84E-43	1.36E-41	-//-
XR_491363.1	2.5935	0.0092243	0.043527	-//-
XR_491366.1	#NAME?	0.0006664	0.0039009	-//-
XR_491397.1	#NAME?	0.010116	0.047308	-//-
XR_491403.1	-0.79219	7.64E-11	8.83E-10	-//-
XR_491419.1	0.76222	2.90E-09	2.95E-08	-//-
XR_491434.1	3.3256	0.0071055	0.034305	-//-
XR_491453.1	-1.3468	0.0036367	0.018694	-//-
XR_491516.1	-2.0465	2.54E-06	2.00E-05	-//-
XR_491526.1	-4.4848	0.0005538	0.0032904	-//-
XR_491556.1	-4.0788	0.0031165	0.016288	-//-
XR_491595.1	2.4187	1.56E-15	2.42E-14	-//-
XR_491709.1	-2.1089	1.46E-05	0.0001053	-//-
XR_491795.1	1.0352	0.0009025	0.0051867	-//-
XR_491800.1	-1.2403	0.0054149	0.026835	-//-
XR_491895.1	-1.6083	1.97E-05	0.0001404	-//-
XR_491928.1	-1.4842	1.80E-07	1.57E-06	-//-
XR_492025.1	-1.949	0.000299	0.0018336	-//-
XR_492136.1	-1.0236	0.0003556	0.0021652	-//-

XR_492144.1	1.368	2.11E-14	3.07E-13	sp|O60729|CC14B_HUMAN Dual specificity protein phosphatase CDC14B OS=Homo sapiens GN=CDC14B PE=1 SV=1//0
XR_492153.1	-2.3485	0.000469	0.0028072	-//-
XR_492186.1	-1.2475	0.0034107	0.017636	-//-
XR_492273.1	3.45	2.92E-10	3.24E-09	-//-
XR_492299.1	1.2301	0.0010955	0.006224	sp|Q9NW38|FANCL_HUMAN E3 ubiquitin-protein ligase FANCL OS=Homo sapiens GN=FANCL PE=1 SV=2//4.25131e-07
XR_492311.1	5.0155	4.61E-05	0.000314	-//-
XR_492312.1	2.8011	0.0077865	0.037329	-//-
XR_492363.1	1.9395	2.61E-05	0.0001831	-//-
XR_492399.1	-1.8829	0.0083017	0.0396	-//-
XR_492408.1	-1.762	9.36E-12	1.16E-10	-//-
XR_492422.1	-2.8639	1.68E-08	1.62E-07	-//-
XR_492451.1	3.0965	3.26E-09	3.31E-08	-//-
XR_492457.1	2.6844	2.06E-06	1.64E-05	-//-
XR_492479.1	3.9815	2.94E-16	4.78E-15	-//-
XR_492527.1	-3.3007	0.0048044	0.024054	-//-
XR_492562.1	0.63912	1.49E-05	0.0001078	sp|Q9C0D9|EPT1_HUMAN Ethanolaminephosphotransferase 1 OS=Homo sapiens GN=EPT1 PE=1 SV=3//0
XR_492863.1	1.1485	7.11E-11	8.24E-10	sp|Q9BQQ3|GORS1_HUMAN Golgi reassembly-stacking protein 1 OS=Homo sapiens GN=GORASP1 PE=1 SV=3//0
XR_492875.1	-0.85193	0.0088655	0.041997	sp|O15527|OGG1_HUMAN N-glycosylase/DNA lyase OS=Homo sapiens GN=OGG1 PE=1 SV=2//7.3557e-130
XR_493050.1	-2.9455	2.76E-12	3.55E-11	-//-
XR_493066.1	-0.72866	2.84E-08	2.67E-07	-//-
XR_493083.1	2.0545	4.87E-07	4.10E-06	sp|P22105|TENX_HUMAN Tenascin-X OS=Homo sapiens GN=TNXB PE=1 SV=4//1.69224e-06
XR_493088.1	1.059	1.96E-05	0.0001391	sp|P30685|1B35_HUMAN HLA class I histocompatibility antigen, B-35 alpha chain OS=Homo sapiens GN=HLA-B PE=1 SV=1//0
XR_493140.1	1.1475	4.09E-09	4.12E-08	-//-
XR_493142.1	0.80138	0.00012	0.0007786	-//-
XR_493151.1	-1.4835	4.89E-07	4.11E-06	-//-
XR_493212.1	2.1765	1.23E-09	1.30E-08	-//-
XR_493223.1	4.1014	0.008822	0.041827	-//-
XR_493229.1	5.0686	7.85E-06	5.86E-05	-//-
XR_493321.1	-3.9458	0.0090303	0.042686	-//-
XR_493329.1	0.91188	4.93E-38	2.00E-36	sp|Q9UBA6|G8_HUMAN Protein G8 OS=Homo sapiens GN=C6orf48 PE=4 SV=2//3.36519e-33
XR_493331.1	-3.4514	0.0017751	0.0097227	sp|Q5R8Z6|MCFD2_PONAB Multiple coagulation factor deficiency protein 2 homolog OS=Pongo abelii GN=MCFD2 PE=2 SV=1//3.01846e-50
XR_493419.1	-1.2727	0.0075458	0.036255	-//-
XR_493499.1	-3.2798	2.14E-06	1.70E-05	-//-
XR_493580.1	-2.0349	2.49E-05	0.0001748	-//-
XR_493595.1	-2.3989	2.03E-05	0.000144	-//-
XR_493596.1	-3.0547	0.0018716	0.010195	-//-
XR_493598.1	-1.9196	6.95E-06	5.23E-05	-//-
XR_493599.1	-1.5884	0.0006505	0.0038175	-//-
XR_493604.1	-0.87945	0.001577	0.0087283	-//-
XR_493608.1	-1.1768	0.0066949	0.032509	-//-
XR_493619.1	3.2258	1.26E-41	5.72E-40	-//-
XR_493624.1	Inf	0.0084468	0.040222	sp|Q8N402|YV020_HUMAN Putative uncharacterized protein LOC388882 OS=Homo sapiens PE=2 SV=1//1.72766e-08
XR_493671.1	2.3868	0.0002732	0.0016848	-//-
XR_493677.1	3.0982	3.23E-06	2.52E-05	-//-
XR_493690.1	3.7204	4.06E-25	1.05E-23	-//-
XR_493702.1	-2.7454	0.0087286	0.041429	-//-
XR_493755.1	Inf	0.0007638	0.0044384	sp|Q2KIX7|HP251_BOVIN Protein HP-25 homolog 1 OS=Bos taurus PE=1 SV=1//1.07084e-06
XR_493799.1	-1.4716	0.0065072	0.031667	sp|Q29488|CP2DH_MACFA Cytochrome P450 2D17 OS=Macaca fascicularis GN=CYP2D17 PE=2 SV=1//1.18928e-30

XR_493802.1	-1.5393	0.0009527	0.0054536	-//-
XR_493803.1	1.5956	0.0008689	0.0050081	sp|Q9H4I8|SEHL2_HUMAN Serine hydrolase-like protein 2 OS=Homo sapiens GN=SERHL2 PE=2 SV=1//4.60949e-29
XR_493829.1	1.9825	1.23E-23	2.94E-22	-//-
XR_493830.1	-0.9977	3.06E-14	4.42E-13	-//-
XR_493876.1	Inf	0.000469	0.0028072	-//-
XR_493882.1	-3.8371	0.0089918	0.04254	-//-
XR_493913.1	1.1726	4.37E-06	3.36E-05	-//-
XR_493941.1	1.8808	0.0017457	0.009578	-//-
XR_493943.1	Inf	0.0002915	0.00179	-//-
XR_493956.1	-4.0634	0.0071548	0.034527	-//-
XR_493991.1	-0.70516	0.0003509	0.0021388	sp|Q02878|RL6_HUMAN 60S ribosomal protein L6 OS=Homo sapiens GN=RPL6 PE=1 SV=3//6.98216e-118
XR_494025.1	-0.52068	4.48E-12	5.69E-11	sp|Q53CG2|COX7C_MACSL Cytochrome c oxidase subunit 7C, mitochondrial OS=Macaca silenus GN=COX7C PE=3 SV=1//3.74618e-36
XR_494062.1	-2.4354	0.0001738	0.0011037	-//-
XR_494063.1	2.9326	5.74E-26	1.52E-24	-//-
XR_494099.1	2.0006	2.73E-05	0.0001909	-//-
XR_494128.1	0.86168	9.47E-07	7.78E-06	-//-
XR_494132.1	#NAME?	0.0001904	0.0011998	-//-
XR_494207.1	Inf	1.23E-07	1.10E-06	sp|Q8TCB0|IFI44_HUMAN Interferon-induced protein 44 OS=Homo sapiens GN=IFI44 PE=2 SV=2//3.27286e-08
XR_494300.1	-3.0479	0.0002184	0.0013653	-//-
XR_494325.1	-3.7093	1.08E-05	7.92E-05	-//-
XR_494327.1	-0.82212	0.0026759	0.014168	-//-
XR_494330.1	-1.2318	0.0070692	0.03416	-//-
XR_494334.1	2.3979	0.0078074	0.037422	-//-
XR_494385.1	-3.1278	1.89E-05	0.0001346	-//-
XR_494401.1	2.5504	0.0080076	0.038298	sp|P23246|SFPQ_HUMAN Splicing factor, proline- and glutamine-rich OS=Homo sapiens GN=SFPQ PE=1 SV=2//0
XR_494424.1	-0.78879	0.0014259	0.0079592	-//-
XR_494457.1	-4.6662	0.0001478	0.0009478	-//-
XR_494462.1	3.7245	0.0006438	0.0037814	-//-
XR_494465.1	2.3563	0.0001438	0.0009229	-//-
XR_494468.1	1.8531	7.80E-08	7.05E-07	sp|A2T715|SCND1_PANTR SCAN domain-containing protein 1 OS=Pan troglodytes GN=SCAND1 PE=3 SV=1//1.15969e-13
XR_494478.1	-1.9037	0.0034519	0.017828	-//-
XR_494567.1	-1.4428	1.60E-08	1.55E-07	-//-
XR_494574.1	-2.1051	4.02E-10	4.42E-09	-//-
XR_494584.1	-1.9956	0.0051035	0.025441	-//-
XR_494595.1	-2.2543	3.64E-19	6.99E-18	-//-
XR_494597.1	-2.6964	1.09E-05	8.00E-05	-//-
XR_494657.1	1.041	0.0039886	0.020322	-//-
XR_494661.1	2.0825	4.76E-12	6.03E-11	-//-
XR_494678.1	3.4341	0.0021205	0.011431	-//-
XR_494680.1	Inf	2.97E-10	3.29E-09	-//-
XR_494756.1	-2.1802	0.0073038	0.035169	sp|P31271|HXA13_HUMAN Homeobox protein Hox-A13 OS=Homo sapiens GN=HOXA13 PE=1 SV=3//1.76347e-09
XR_494760.1	-3.1603	2.91E-25	7.55E-24	sp|P31269|HXA9_HUMAN Homeobox protein Hox-A9 OS=Homo sapiens GN=HOXA9 PE=1 SV=4//5.30004e-10
XR_494809.1	-1.0074	0.003947	0.02013	-//-
XR_494912.1	-1.4682	1.66E-07	1.46E-06	sp|Q6B7M7|COF1_SHEEP Cofilin-1 OS=Ovis aries GN=CFL1 PE=2 SV=3//6.72274e-37
XR_494925.1	2.1709	2.47E-11	2.98E-10	-//-
XR_494940.1	-1.2883	0.00024	0.0014917	-//-
XR_494945.1	-0.93298	0.0017167	0.0094356	-//-
XR_494955.1	-2.792	9.88E-10	1.05E-08	-//-
XR_494970.1	2.3701	0.000141	0.0009064	-//-
XR_494992.1	-0.44226	0.0084021	0.040027	sp|Q13148|TADBP_HUMAN TAR DNA-binding protein 43 OS=Homo sapiens

				GN=TARDBP PE=1 SV=1//0
XR_495003.1	-1.7822	0.0069309	0.033536	-//-
XR_495029.1	2.114	7.77E-10	8.30E-09	-//-
XR_495035.1	-2.0742	9.05E-05	0.0005974	-//-
XR_495071.1	0.90747	1.33E-07	1.18E-06	-//-
XR_495129.1	3.0091	6.38E-11	7.43E-10	-//-
XR_495198.1	-3.9806	0.0068183	0.03305	-//-
XR_495201.1	-1.712	2.34E-23	5.52E-22	-//-
XR_495206.1	0.46309	0.0005037	0.0030042	sp|O95376|ARI2_HUMAN E3 ubiquitin-protein ligase ARIH2 OS=Homo sapiens GN=ARIH2 PE=1 SV=1//0
XR_495223.1	-0.99558	0.0001284	0.0008302	-//-
XR_495269.1	-4.5014	0.0009329	0.0053501	-//-
XR_495330.1	-1.1707	0.0074713	0.035928	-//-
XR_495343.1	-0.71948	2.77E-05	0.0001935	sp|Q9NY33|DPP3_HUMAN Dipeptidyl peptidase 3 OS=Homo sapiens GN=DPP3 PE=1 SV=2//0
XR_495351.1	3.3376	3.86E-19	7.38E-18	-//-
XR_495354.1	-1.6291	0.0001001	0.000657	sp|Q60HD6|PMM2_MACFA Phosphomannomutase 2 OS=Macaca fascicularis GN=PMM2 PE=2 SV=1//2.55963e-171
XR_495368.1	0.51667	0.0028906	0.01522	-//-
XR_495375.1	-1.8619	2.65E-05	0.0001858	sp|Q9HBG6|IF122_HUMAN Intraflagellar transport protein 122 homolog OS=Homo sapiens GN=IFT122 PE=1 SV=2//3.22494e-10
XR_495427.1	-0.53654	1.15E-14	1.72E-13	sp|Q5R9Y1|FSTL1_PONAB Follistatin-related protein 1 OS=Pongo abelii GN=FSTL1 PE=2 SV=2//0
XR_495574.1	5.0282	3.44E-25	8.90E-24	-//-
XR_495575.1	5.0416	5.19E-71	5.36E-69	-//-
XR_495599.1	-1.0268	1.90E-07	1.66E-06	sp|Q02878|RL6_HUMAN 60S ribosomal protein L6 OS=Homo sapiens GN=RPL6 PE=1 SV=3//9.9556e-149
XR_495691.1	-0.68689	0.0004078	0.0024654	sp|Q5RFN6|CNN2_PONAB Calponin-2 OS=Pongo abelii GN=CNN2 PE=2 SV=3//1.10489e-144
XR_495872.1	2.9744	1.63E-31	5.30E-30	-//-
XR_495886.1	1.197	1.75E-09	1.82E-08	-//-
XR_495903.1	4.1213	5.20E-20	1.03E-18	-//-
XR_495915.1	1.3612	3.95E-21	8.30E-20	sp|Q9Y5J7|TIM9_HUMAN Mitochondrial import inner membrane translocase subunit Tim9 OS=Homo sapiens GN=TIMM9 PE=1 SV=1//9.5675e-48
XR_495919.1	8.1073	1.73E-77	2.11E-75	-//-
XR_495938.1	-1.6736	0.0069372	0.033559	-//-
XR_495991.1	0.65949	0.0061672	0.030201	-//-
XR_496002.1	-3.9144	0.0090069	0.042593	-//-
XR_496086.1	-1.0127	4.39E-05	0.0002995	-//-
XR_496326.1	-1.4653	0.0001517	0.0009698	sp|Q8IVF2|AHNK2_HUMAN Protein AHNAK2 OS=Homo sapiens GN=AHNAK2 PE=1 SV=2//1.28869e-24
XR_496353.1	-3.1452	8.79E-05	0.0005809	-//-
XR_496357.1	-3.0795	0.0012997	0.0073046	-//-
XR_496404.1	1.4965	6.21E-16	9.90E-15	-//-
XR_496418.1	4.721	3.73E-08	3.46E-07	-//-
XR_496453.1	1.1428	0.0003929	0.0023798	-//-
XR_496474.1	3.6457	0.0009876	0.0056462	-//-
XR_496477.1	2.8143	2.13E-14	3.10E-13	-//-
XR_496518.1	2.9441	0.0054249	0.026878	-//-
XR_496529.1	1.4491	0.0010577	0.0060212	-//-
XR_496716.1	-1.5436	3.57E-05	0.0002462	-//-
XR_496796.1	-1.3925	0.0072891	0.035106	-//-
XR_496875.1	-1.2687	0.0072513	0.034947	-//-
XR_496912.1	2.7601	4.21E-28	1.21E-26	-//-
XR_497060.1	-0.88923	8.82E-11	1.02E-09	sp|Q5RDV1|YX011_PONAB Putative uncharacterized protein LOC550643 homolog OS=Pongo abelii PE=5 SV=1//2.89625e-24
XR_497065.1	0.46659	4.30E-07	3.64E-06	sp|Q6NVR8|LS14A_XENTR Protein LSM14 homolog A OS=Xenopus tropicalis GN=lsm14a PE=2 SV=1//1.90584e-120
XR_497085.1	-1.3281	1.19E-06	9.67E-06	-//-
XR_497098.1	#NAME?	0.0030559	0.016002	-//-

XR_497167.1	-1.0656	4.99E-06	3.81E-05	sp|P63219|GBG5_RAT Guanine nucleotide-binding protein G(I)/G(S)/G(O) subunit gamma-5 OS=Rattus norvegicus GN=Gng5 PE=3 SV=1//7.51283e-30
XR_497239.1	-1.9693	1.24E-06	1.01E-05	-//-
XR_497278.1	-1.5018	0.0011984	0.0067739	-//-
XR_497401.1	Inf	2.06E-09	2.13E-08	-//-
XR_497432.1	-1.9758	0.0002354	0.0014661	-//-
XR_497445.1	0.95464	2.26E-05	0.0001595	sp|P21733|YCR2_BACTK Uncharacterized 29.1 kDa protein in cryB1 5'region OS=Bacillus thuringiensis subsp. kurstaki PE=4 SV=1//1.3224e-08
XR_497446.1	-0.9346	0.0099666	0.046718	-//-
XR_497458.1	1.561	1.03E-10	1.18E-09	-//-
XR_497466.1	1.3336	0.0001535	0.0009797	sp|Q5RF83|CIRBP_PONAB Cold-inducible RNA-binding protein OS=Pongo abelii GN=CIRBP PE=2 SV=1//3.81574e-54
XR_497468.1	#NAME?	0.0020326	0.010995	-//-
XR_497500.1	3.0202	1.47E-140	4.42E-138	-//-
XR_497520.1	-0.5426	2.66E-08	2.52E-07	sp|Q8IWA5|CTL2_HUMAN Choline transporter-like protein 2 OS=Homo sapiens GN=SLC44A2 PE=1 SV=3//8.30056e-23
XR_497626.1	-5.125	2.14E-06	1.70E-05	-//-
XR_497631.1	-2.2706	1.54E-06	1.24E-05	sp|Q2M3W8|ZN181_HUMAN Zinc finger protein 181 OS=Homo sapiens GN=ZNF181 PE=2 SV=1//2.27938e-10
XR_497637.1	-1.6135	2.16E-37	8.60E-36	sp|Q53CG4|CX6B1_MACMU Cytochrome c oxidase subunit 6B1 OS=Macaca mulatta GN=COX6B1 PE=3 SV=3//2.38647e-31
XR_497638.1	-2.0809	4.81E-18	8.64E-17	-//-
XR_497682.1	1.4506	1.62E-07	1.43E-06	sp|Q96KS0|EGLN2_HUMAN Egl nine homolog 2 OS=Homo sapiens GN=EGLN2 PE=1 SV=1//0
XR_497708.1	2.0995	2.56E-21	5.43E-20	-//-
XR_497730.1	-2.5011	0.010192	0.047612	-//-
XR_497740.1	3.881	0.0096134	0.045198	-//-
XR_497745.1	1.4317	8.49E-08	7.64E-07	sp|Q9H4M7|PKHA4_HUMAN Pleckstrin homology domain-containing family A member 4 OS=Homo sapiens GN=PLEKHA4 PE=1 SV=2//1.84357e-18
XR_497754.1	-1.4388	1.91E-05	0.000136	-//-
XR_497764.1	-1.9065	0.0053155	0.026396	-//-
XR_497767.1	-1.6414	0.0036528	0.018764	sp|Q9BQG1|SYT3_HUMAN Synaptotagmin-3 OS=Homo sapiens GN=SYT3 PE=2 SV=1//0
XR_497775.1	1.2764	3.42E-06	2.66E-05	-//-
XR_497807.1	5.3606	7.33E-108	1.61E-105	sp|Q8N5Q1|F71E2_HUMAN Protein FAM71E2 OS=Homo sapiens GN=FAM71E2 PE=2 SV=3//1.36009e-10
XR_497808.1	1.0389	0.0053691	0.026632	sp|Q8N5Q1|F71E2_HUMAN Protein FAM71E2 OS=Homo sapiens GN=FAM71E2 PE=2 SV=3//1.56559e-10
XR_497810.1	-1.1757	3.20E-09	3.25E-08	-//-
XR_497855.1	-3.8212	0.0037582	0.019256	-//-
XR_497895.1	-2.1921	3.92E-05	0.0002693	-//-
XR_497938.1	2.2093	1.29E-17	2.26E-16	-//-
XR_498017.1	-1.1985	0.0033555	0.0174	sp|P07919|QCR6_HUMAN Cytochrome b-c1 complex subunit 6, mitochondrial OS=Homo sapiens GN=UQCRH PE=1 SV=2//1.19566e-15
XR_498076.1	1.5705	3.01E-08	2.82E-07	sp|Q8NDZ0|BEND2_HUMAN BEN domain-containing protein 2 OS=Homo sapiens GN=BEND2 PE=2 SV=2//6.17039e-07
XR_498129.1	Inf	5.03E-10	5.48E-09	-//-
XR_498144.1	1.3465	3.37E-08	3.15E-07	sp|Q9H082|RB33B_HUMAN Ras-related protein Rab-33B OS=Homo sapiens GN=RAB33B PE=1 SV=1//6.19563e-140
XR_498184.1	Inf	1.98E-09	2.06E-08	-//-
XR_498197.1	3.4366	4.34E-10	4.76E-09	-//-
XR_498354.1	-0.74602	0.0001483	0.0009504	sp|Q5RAQ8|RS24_PONAB 40S ribosomal protein S24 OS=Pongo abelii GN=RPS24 PE=2 SV=1//3.88828e-63
XR_498369.1	4.1467	2.36E-05	0.000166	sp|P04211|LV001_HUMAN Ig lambda chain V region 4A OS=Homo sapiens PE=4 SV=1//1.68263e-21
XR_498372.1	-1.6646	9.23E-08	8.27E-07	-//-
XR_498454.1	-1.6285	7.25E-08	6.57E-07	sp|Q0VCQ8|P5I11_BOVIN Tumor protein p53-inducible protein 11 OS=Bos taurus GN=TP53I11 PE=2 SV=1//1.15543e-98
XR_498455.1	0.52925	4.25E-06	3.28E-05	-//-
XR_498468.1	-3.3997	0.0001509	0.0009658	-//-
XR_498521.1	-4.1841	0.0016229	0.0089539	-//-
XR_498531.1	-2.894	0.0033237	0.017252	-//-

XR_498593.1	0.48455	9.10E-06	6.74E-05	-//-
XR_498602.1	2.8576	2.98E-07	2.55E-06	-//-
XR_498603.1	2.7066	3.43E-09	3.48E-08	-//-
XR_498676.1	2.4114	1.49E-10	1.68E-09	-//-
XR_498692.1	-0.89516	0.0034948	0.018033	-//-
XR_498705.1	2.4227	3.61E-14	5.20E-13	-//-
XR_498712.1	0.6963	0.000253	0.0015676	-//-
XR_498757.1	1.2361	1.81E-30	5.66E-29	sp|Q96KN1|FA84B_HUMAN Protein FAM84B OS=Homo sapiens GN=FAM84B PE=1 SV=1//0
XR_498788.1	#NAME?	0.006465	0.031497	-//-
XR_498836.1	-2.2087	0.000379	0.002298	-//-
XR_498847.1	-1.1269	1.54E-05	0.0001107	-//-
XR_498866.1	0.36325	7.81E-05	0.0005194	-//-
XR_498872.1	-2.0834	0.0031234	0.016316	-//-
XR_498882.1	-1.4347	0.0004708	0.0028172	-//-
XR_498883.1	-1.3001	9.89E-05	0.0006497	-//-
XR_498886.1	-3.4203	4.36E-07	3.69E-06	-//-
XR_498891.1	1.5983	0.0012454	0.0070233	-//-
XR_498910.1	1.049	2.80E-08	2.64E-07	sp|Q8N769|CN178_HUMAN Uncharacterized protein C14orf178 OS=Homo sapiens GN=C14orf178 PE=2 SV=1//4.92885e-08
XR_498914.1	1.9304	0.00144	0.0080237	-//-
XR_498921.1	4.074	2.68E-05	0.0001876	-//-
XR_498922.1	3.3219	1.43E-09	1.50E-08	-//-
XR_498956.1	1.3024	1.53E-05	0.0001102	-//-
XR_499065.1	2.5076	2.73E-29	8.16E-28	sp|Q3KNS1|PTHD3_HUMAN Patched domain-containing protein 3 OS=Homo sapiens GN=PTCHD3 PE=1 SV=3//7.77158e-09
XR_499119.1	-2.887	0.0048269	0.024155	sp|Q8IY57|YAF2_HUMAN YY1-associated factor 2 OS=Homo sapiens GN=YAF2 PE=1 SV=3//9.10691e-31
XR_499151.1	-4.311	0.0015773	0.0087283	sp|Q4R7G7|CCD65_MACFA Coiled-coil domain-containing protein 65 OS=Macaca fascicularis GN=CCDC65 PE=2 SV=1//9.92266e-80
XR_499153.1	-4.7165	5.17E-05	0.0003507	-//-
XR_499166.1	3.0235	0.0001171	0.0007609	-//-
XR_499183.1	-1.2892	0.0075621	0.036322	-//-
XR_499184.1	-1.8204	3.64E-17	6.22E-16	-//-
XR_499188.1	-2.5102	0.0003176	0.0019418	-//-
XR_499189.1	-2.1807	0.0036911	0.018939	-//-
XR_499190.1	-1.3458	0.0034053	0.017613	-//-
XR_499201.1	0.7981	6.80E-06	5.12E-05	-//-
XR_499211.1	-1.7443	4.38E-07	3.71E-06	-//-
XR_499216.1	-1.804	0.010662	0.049627	-//-
XR_499235.1	-3.0881	0.0018402	0.010036	-//-
XR_499257.1	1.106	7.33E-05	0.0004895	sp|Q8TC05|MDM1_HUMAN Nuclear protein MDM1 OS=Homo sapiens GN=MDM1 PE=1 SV=2//5.82111e-09
XR_499269.1	-1.7293	9.42E-09	9.23E-08	-//-
XR_499328.1	-2.3673	1.86E-08	1.78E-07	-//-
XR_499382.1	2.9923	0.0004399	0.0026459	-//-
XR_499384.1	3.6327	2.92E-06	2.28E-05	-//-
XR_499428.1	-1.4032	7.22E-08	6.55E-07	sp|Q6QMZ4|RL6_CHILA 60S ribosomal protein L6 OS=Chinchilla lanigera GN=RPL6 PE=2 SV=3//2.76693e-30
XR_499465.1	-0.75552	0.0001776	0.0011249	-//-
XR_499476.1	-1.7286	0.0022628	0.012136	sp|P63170|DYL1_RAT Dynein light chain 1, cytoplasmic OS=Rattus norvegicus GN=Dynll1 PE=1 SV=1//2.11548e-45
XR_499482.1	-3.0849	8.02E-55	5.53E-53	-//-
XR_499490.1	1.8301	3.70E-48	2.14E-46	sp|Q5R8J6|RSRC2_PONAB Arginine/serine-rich coiled-coil protein 2 OS=Pongo abelii GN=RSRC2 PE=2 SV=1//1.74315e-56
XR_499603.1	-1.0626	0.0014716	0.008186	-//-
XR_499606.1	-0.87752	4.14E-05	0.0002833	-//-
XR_499632.1	-1.0302	1.26E-10	1.43E-09	-//-
XR_499641.1	1.1791	0.0004541	0.0027272	sp|O75683|SURF6_HUMAN Surfeit locus protein 6 OS=Homo sapiens GN=SURF6

				PE=1 SV=3//6.24326e-37
XR_499644.1	-0.97157	1.35E-06	1.09E-05	sp|Q96BR6|ZN669_HUMAN Zinc finger protein 669 OS=Homo sapiens GN=ZNF669 PE=2 SV=2//2.28668e-26
XR_499671.1	-1.6518	0.0087153	0.041375	-//-
XR_499692.1	4.217	0.0056771	0.027976	-//-
XR_499769.1	0.63419	0.000583	0.0034484	-//-
XR_499770.1	0.93669	0.0003479	0.0021221	-//-
XR_499771.1	-0.93697	2.39E-17	4.14E-16	sp|P05787|K2C8_HUMAN Keratin, type II cytoskeletal 8 OS=Homo sapiens GN=KRT8 PE=1 SV=7//9.00574e-09
XR_499941.1	-1.2682	1.25E-05	9.14E-05	-//-
XR_499947.1	-0.84085	0.010236	0.047795	sp|O43491|E41L2_HUMAN Band 4.1-like protein 2 OS=Homo sapiens GN=EPB41L2 PE=1 SV=1//0
XR_500007.1	2.7699	0.0003442	0.0021001	-//-
XR_500033.1	3.5588	0.0021646	0.011657	-//-
XR_500159.1	0.57512	0.0022903	0.012271	-//-
XR_500192.1	1.8979	5.53E-09	5.51E-08	-//-
XR_500284.1	-3.453	7.43E-25	1.87E-23	-//-
XR_500345.1	1.7923	0.001873	0.0102	sp|Q8N769|CN178_HUMAN Uncharacterized protein C14orf178 OS=Homo sapiens GN=C14orf178 PE=2 SV=1//7.36482e-07
XR_500379.1	3.1373	0.010396	0.048484	-//-
XR_500380.1	1.2046	1.08E-30	3.41E-29	-//-
XR_500381.1	3.0405	1.44E-20	2.94E-19	-//-
XR_500415.1	Inf	0.0031927	0.016643	-//-
XR_500416.1	2.9971	1.46E-05	0.0001052	sp|P18621|RL17_HUMAN 60S ribosomal protein L17 OS=Homo sapiens GN=RPL17 PE=1 SV=3//6.57569e-121
XR_500521.1	1.7856	2.37E-06	1.87E-05	-//-
XR_500522.1	1.1277	0.0001285	0.0008309	-//-
XR_500550.1	-3.8416	0.0089414	0.04232	-//-
XR_500594.1	-4.6135	0.0001901	0.0011985	-//-
XR_500607.1	6.5708	1.10E-16	1.85E-15	-//-
XR_500639.1	2.9393	0.0004272	0.002576	-//-
XR_500644.1	1.8695	0.0008364	0.0048296	-//-
XR_500645.1	4.311	0.0047624	0.023882	sp|Q2T9K0|TMM44_HUMAN Transmembrane protein 44 OS=Homo sapiens GN=TMEM44 PE=2 SV=3//0
XR_500652.1	2.933	0.0002393	0.0014884	-//-
XR_500666.1	-1.8632	9.73E-05	0.0006403	-//-
XR_500669.1	1.8599	0.0001349	0.0008695	-//-
XR_500679.1	0.81226	5.74E-05	0.000387	-//-
XR_500694.1	-1.0848	0.0015401	0.0085462	-//-
XR_500698.1	-2.1507	5.52E-08	5.05E-07	sp|Q6AY62|NEUFC_RAT Neuferricin OS=Rattus norvegicus GN=Cyb5d2 PE=2 SV=1//7.44581e-07
XR_500702.1	0.59296	0.0075805	0.036398	-//-
XR_500727.1	-2.7693	0.0087827	0.04165	-//-
XR_500732.1	5.5477	3.62E-08	3.37E-07	-//-
XR_500765.1	2.6877	6.64E-73	7.17E-71	sp|Q8N1F1|C1AS1_HUMAN Putative uncharacterized protein LRRC75A-AS1, mitochondrial OS=Homo sapiens GN=LRRC75A-AS1 PE=5 SV=1//1.10097e-06
XR_500885.1	-1.1448	1.24E-14	1.83E-13	-//-
XR_500904.1	Inf	0.0043949	0.022242	-//-
XR_500905.1	1.5841	0.0001164	0.0007572	-//-
XR_500914.1	-4.0871	0.0056768	0.027976	-//-
XR_500941.1	3.4387	9.75E-41	4.32E-39	-//-
XR_500945.1	-2.1968	1.44E-07	1.28E-06	-//-
XR_500962.1	-2.4808	9.07E-06	6.73E-05	-//-
XR_501053.1	-2.4663	0.010232	0.047787	sp|Q8N2A0|CX062_HUMAN Putative uncharacterized protein encoded by LINC00269 OS=Homo sapiens GN=LINC00269 PE=5 SV=1//4.53457e-23
XR_501063.1	-2.1951	1.70E-13	2.35E-12	-//-
XR_501092.1	0.29868	0.0046821	0.02356	sp|Q01628|IFM3_HUMAN Interferon-induced transmembrane protein 3 OS=Homo sapiens GN=IFITM3 PE=1 SV=2//1.75246e-77
XR_501097.1	-1.3892	9.13E-06	6.76E-05	-//-

XR_501111.1	-4.599	0.0002502	0.0015519	-//-
XR_501123.1	-1.1031	0.0007638	0.0044384	-//-
XR_501141.1	-2.922	5.64E-16	9.02E-15	-//-
XR_501206.1	-2.9049	0.0048676	0.024337	-//-
XR_501223.1	1.1815	0.0037458	0.019201	sp|Q32MQ0|ZN750_HUMAN Zinc finger protein 750 OS=Homo sapiens GN=ZNF750 PE=1 SV=1//1.23921e-07
XR_501235.1	-1.6344	1.03E-13	1.46E-12	-//-
XR_501458.1	4.8905	0.0001139	0.0007419	-//-
XR_501478.1	-2.1904	1.04E-05	7.65E-05	sp|Q8N769|CN178_HUMAN Uncharacterized protein C14orf178 OS=Homo sapiens GN=C14orf178 PE=2 SV=1//3.28864e-07
XR_501479.1	-2.277	0.0001299	0.000839	-//-
XR_501484.1	1.9536	9.12E-23	2.09E-21	sp|O18973|RABX5_BOVIN Rab5 GDP/GTP exchange factor OS=Bos taurus GN=RABGEF1 PE=1 SV=1//0
XR_501514.1	3.7525	7.13E-09	7.03E-08	-//-
XR_501532.1	2.1557	7.48E-30	2.29E-28	-//-
XR_501578.1	-1.2406	0.0033575	0.017406	sp|P15880|RS2_HUMAN 40S ribosomal protein S2 OS=Homo sapiens GN=RPS2 PE=1 SV=2//2.64988e-21
XR_501703.1	-1.1402	1.41E-07	1.25E-06	sp|P04406|G3P_HUMAN Glyceraldehyde-3-phosphate dehydrogenase OS=Homo sapiens GN=GAPDH PE=1 SV=3//0
XR_501745.1	-4.2698	0.0016	0.0088428	-//-
XR_501746.1	-1.2028	3.56E-13	4.81E-12	sp|O00244|ATOX1_HUMAN Copper transport protein ATOX1 OS=Homo sapiens GN=ATOX1 PE=1 SV=1//8.546e-29
XR_501813.1	1.5885	0.0067601	0.032803	-//-
XR_501861.1	-1.626	0.0002253	0.0014056	-//-
XR_501923.1	2.2596	0.0001742	0.0011059	-//-
XR_501935.1	-1.2213	0.0005822	0.0034447	-//-
XR_501964.1	0.85717	2.62E-08	2.48E-07	sp|O15014|ZN609_HUMAN Zinc finger protein 609 OS=Homo sapiens GN=ZNF609 PE=1 SV=2//2.57385e-118
XR_501976.1	1.1217	5.20E-06	3.97E-05	-//-
XR_502056.1	-0.82174	0.0024571	0.013104	-//-
XR_502167.1	-2.8293	0.0066244	0.032202	-//-
XR_502168.1	-1.5936	0.0019066	0.010362	sp|P63174|RL38_RAT 60S ribosomal protein L38 OS=Rattus norvegicus GN=Rpl38 PE=1 SV=2//3.06947e-21
XR_502175.1	Inf	0.0012513	0.0070544	-//-
XR_502194.1	-4.5454	0.0003585	0.002181	-//-
XR_502253.1	-0.99377	5.77E-05	0.0003886	-//-
XR_502309.1	2.9539	1.14E-20	2.33E-19	-//-
XR_502409.1	-0.87803	0.0011451	0.0064876	-//-
XR_502412.1	Inf	4.40E-05	0.0003002	-//-
XR_502429.1	-0.71815	1.58E-12	2.07E-11	sp|P29314|RS9_RAT 40S ribosomal protein S9 OS=Rattus norvegicus GN=Rps9 PE=1 SV=4//2.02661e-113
XR_502435.1	-2.7702	0.0017566	0.0096358	-//-
XR_502556.1	3.8303	1.80E-07	1.57E-06	-//-
XR_502561.1	1.8766	0.000102	0.0006684	-//-
XR_502595.1	-1.2788	0.0003172	0.00194	-//-
XR_502597.1	0.65509	1.22E-11	1.50E-10	sp|Q4R766|ABD12_MACFA Monoacylglycerol lipase ABHD12 OS=Macaca fascicularis GN=ABHD12 PE=2 SV=1//2.59663e-44
XR_502599.1	1.9746	2.82E-07	2.42E-06	sp|P81126|SNAB_BOVIN Beta-soluble NSF attachment protein OS=Bos taurus GN=NAPB PE=1 SV=1//6.39774e-09
XR_502601.1	1.0649	2.59E-06	2.04E-05	sp|Q9BVI0|PHF20_HUMAN PHD finger protein 20 OS=Homo sapiens GN=PHF20 PE=1 SV=2//2.3431e-54
XR_502603.1	1.212	9.67E-48	5.50E-46	-//-
XR_502632.1	-2.7316	1.78E-10	2.00E-09	-//-
XR_502633.1	-2.4308	0.001432	0.0079861	-//-
XR_502635.1	-2.3344	0.0067918	0.032928	-//-
XR_502638.1	-4.2031	0.0047896	0.023996	-//-
XR_502744.1	-0.93032	0.0019539	0.010598	-//-
XR_502749.1	2.7438	6.47E-20	1.28E-18	-//-
XR_502773.1	0.58218	8.50E-06	6.33E-05	sp|Q92834|RPGR_HUMAN X-linked retinitis pigmentosa GTPase regulator OS=Homo sapiens GN=RPGR PE=1 SV=2//2.58787e-84
XR_502777.1	-1.2767	8.79E-07	7.26E-06	sp|Q5R8H3|BAP31_PONAB B-cell receptor-associated protein 31 OS=Pongo

				abelii GN=BCAP31 PE=2 SV=3//1.02445e-13
XR_502842.1	1.9898	0.0040394	0.020567	-//-
XR_502976.1	2.6684	0.0056958	0.028062	sp|Q6ZU15|SEP14_HUMAN Septin-14 OS=Homo sapiens GN=SEPT14 PE=1 SV=2//8.06143e-18
XR_503030.1	1.0723	4.08E-08	3.77E-07	sp|P28749|RBL1_HUMAN Retinoblastoma-like protein 1 OS=Homo sapiens GN=RBL1 PE=1 SV=3//2.53615e-43
XR_503049.1	#NAME?	0.0006445	0.0037847	sp|Q9ULZ0|T53G3_HUMAN TP53-target gene 3 protein OS=Homo sapiens GN=TP53TG3 PE=2 SV=1//5.32221e-13
XR_503134.1	-3.0624	3.34E-05	0.0002314	sp|Q9NUG6|PDRG1_HUMAN p53 and DNA damage-regulated protein 1 OS=Homo sapiens GN=PDRG1 PE=1 SV=2//1.60941e-08
XR_503146.1	-2.2099	0.007145	0.034488	sp|Q9GZT6|CC90B_HUMAN Coiled-coil domain-containing protein 90B, mitochondrial OS=Homo sapiens GN=CCDC90B PE=1 SV=2//3.97736e-153
XR_503150.1	1.8542	0.0002553	0.0015807	-//-
XR_503260.1	#NAME?	0.0042562	0.02159	-//-
XR_503341.1	4.6019	7.50E-07	6.22E-06	-//-
XR_503367.1	-2.654	2.20E-06	1.74E-05	-//-
XR_503481.1	-0.37762	3.46E-05	0.0002392	-//-
XR_503569.1	-1.3543	6.84E-29	2.03E-27	sp|P04406|G3P_HUMAN Glyceraldehyde-3-phosphate dehydrogenase OS=Homo sapiens GN=GAPDH PE=1 SV=3//0

Table S2 Lists of the significantly regulated DEGs in 85-7 infected MARC-145 cells.

Gene_id	log2FoldChange	pval	padj	Blast swiss prot
XM_007960048.1	0.80779	1.37E-21	5.87E-20	sp|O95164|UBL3_HUMAN Ubiquitin-like protein 3 OS=Homo sapiens GN=UBL3 PE=1 SV=1//8.38869e-74
XM_007960060.1	2.6238	6.29E-06	6.74E-05	-//-
XM_007960071.1	-4.1575	0.003245	0.021225	sp|Q8WXD0|RXFP2_HUMAN Relaxin receptor 2 OS=Homo sapiens GN=RXFP2 PE=1 SV=1//0
XM_007960082.1	1.0237	0.004851	0.030561	-//-
XM_007960106.1	0.61653	1.80E-05	0.000179	sp|P40938|RFC3_HUMAN Replication factor C subunit 3 OS=Homo sapiens GN=RFC3 PE=1 SV=2//0
XM_007960123.1	-0.83392	0.00016	0.001369	sp|Q9JLM8|DCLK1_MOUSE Serine/threonine-protein kinase DCLK1 OS=Mus musculus GN=Dclk1 PE=1 SV=1//0
XM_007960150.1	0.9366	3.35E-10	6.03E-09	sp|O00287|RFXAP_HUMAN Regulatory factor X-associated protein OS=Homo sapiens GN=RFXAP PE=1 SV=1//6.34309e-93
XM_007960193.1	0.40135	0.000234	0.001954	sp|Q5R4N5|UFM1_PONAB Ubiquitin-fold modifier 1 OS=Pongo abelii GN=UFM1 PE=3 SV=1//2.058e-49
XM_007960198.1	0.53979	0.000246	0.002045	sp|Q5JS37|NHLC3_HUMAN NHL repeat-containing protein 3 OS=Homo sapiens GN=NHLRC3 PE=2 SV=1//0
XM_007960206.1	1.7808	5.76E-46	7.04E-44	sp|Q12778|FOXO1_HUMAN Forkhead box protein O1 OS=Homo sapiens GN=FOXO1 PE=1 SV=2//0
XM_007960218.1	0.92312	1.17E-11	2.42E-10	sp|O75554|WBP4_HUMAN WW domain-binding protein 4 OS=Homo sapiens GN=WBP4 PE=1 SV=1//0
XM_007960222.1	0.39416	0.004171	0.026638	sp|Q86V97|KBTB6_HUMAN Kelch repeat and BTB domain-containing protein 6 OS=Homo sapiens GN=KBTBD6 PE=1 SV=1//0
XM_007960315.1	0.50125	0.000192	0.001617	sp|Q4R4H5|T22D1_MACFA TSC22 domain family protein 1 OS=Macaca fascicularis GN=TSC22D1 PE=2 SV=1//3.23385e-71
XM_007960317.1	1.9131	6.98E-30	4.63E-28	sp|Q9UHK0|NUFP1_HUMAN Nuclear fragile X mental retardation-interacting protein 1 OS=Homo sapiens GN=NUFIP1 PE=1 SV=2//0
XM_007960326.1	-0.2805	0.001705	0.011935	sp|P61288|TCTP_PIG Translationally-controlled tumor protein OS=Sus scrofa GN=TPT1 PE=2 SV=1//1.93386e-121
XM_007960331.1	0.79207	8.26E-12	1.73E-10	sp|Q96JB2|COG3_HUMAN Conserved oligomeric Golgi complex subunit 3 OS=Homo sapiens GN=COG3 PE=1 SV=3//0
XM_007960377.1	-0.48564	0.001029	0.00758	sp|Q9NPJ6|MED4_HUMAN Mediator of RNA polymerase II transcription subunit 4 OS=Homo sapiens GN=MED4 PE=1 SV=1//5.21117e-156
XM_007960379.1	-0.55046	5.29E-13	1.24E-11	sp|Q5R876|ITM2B_PONAB Integral membrane protein 2B OS=Pongo abelii GN=ITM2B PE=2 SV=1//3.23778e-155
XM_007960381.1	0.35448	0.000242	0.002012	sp|P06400|RB_HUMAN Retinoblastoma-associated protein OS=Homo sapiens GN=RB1 PE=1 SV=2//0
XM_007960389.1	1.1975	7.39E-06	7.80E-05	sp|Q4R683|CDAC1_MACFA Cytidine and dCMP deaminase domain-containing protein 1 OS=Macaca fascicularis GN=CDADC1 PE=2 SV=1//0
XM_007960420.1	-1.2336	9.64E-06	9.97E-05	sp|Q8N5I3|KCNRG_HUMAN Potassium channel regulatory protein OS=Homo sapiens GN=KCNRG PE=1 SV=1//3.2983e-161
XM_007960440.1	0.98567	1.27E-07	1.69E-06	sp|Q9UL03|INT6_HUMAN Integrator complex subunit 6 OS=Homo sapiens GN=INTS6 PE=1 SV=1//0
XM_007960444.1	1.4121	1.92E-06	2.22E-05	sp|Q5RA50|F124A_PONAB Protein FAM124A OS=Pongo abelii GN=FAM124A PE=2 SV=1//0
XM_007960449.1	0.6956	2.63E-10	4.78E-09	sp|Q9H501|ESF1_HUMAN ESF1 homolog OS=Homo sapiens GN=ESF1 PE=1 SV=1//0
XM_007960511.1	0.96818	3.29E-09	5.32E-08	sp|Q9NZU0|FLRT3_HUMAN Leucine-rich repeat transmembrane protein FLRT3 OS=Homo sapiens GN=FLRT3 PE=1 SV=1//0
XM_007960515.1	0.73451	2.15E-08	3.14E-07	sp|O14917|PCD17_HUMAN Protocadherin-17 OS=Homo sapiens GN=PCDH17 PE=2 SV=2//0
XM_007960530.1	0.31742	0.003689	0.023824	sp|Q8WXF1|PSPC1_HUMAN Paraspeckle component 1 OS=Homo sapiens GN=PSPC1 PE=1 SV=1//4.28468e-115
XM_007960538.1	-1.0235	3.40E-05	0.000325	sp|P63159|HMGB1_RAT High mobility group protein B1 OS=Rattus norvegicus GN=Hmgb1 PE=1 SV=2//8.18615e-82
XM_007960539.1	-0.70122	2.16E-12	4.78E-11	sp|P07156|HMGB1_CRIGR High mobility group protein B1 (Fragment) OS=Cricetulus griseus GN=HMGB1 PE=1 SV=1//3.87754e-27
XM_007960540.1	0.49285	0.000142	0.001226	sp|Q92665|RT31_HUMAN 28S ribosomal protein S31, mitochondrial OS=Homo sapiens GN=MRPS31 PE=1 SV=3//1.03497e-155
XM_007960564.1	1.3827	7.50E-13	1.73E-11	sp|Q08AG7|MZT1_HUMAN Mitotic-spindle organizing protein 1 OS=Homo sapiens GN=MZT1 PE=1 SV=2//3.75449e-32
XM_007960571.1	3.3486	6.49E-14	1.64E-12	sp|Q13887|KLF5_HUMAN Krueppel-like factor 5 OS=Homo sapiens GN=KLF5 PE=1 SV=2//0
XM_007960578.1	-0.80005	1.95E-05	0.000193	sp|Q7Z4G1|COMD6_HUMAN COMM domain-containing protein 6 OS=Homo sapiens GN=COMMD6 PE=1 SV=1//8.57457e-56
XM_007960664.1	0.46033	4.62E-08	6.45E-07	sp|Q9NV92|NFIP2_HUMAN NEDD4 family-interacting protein 2 OS=Homo sapiens GN=NDFIP2 PE=1 SV=2//9.73781e-155
XM_007960665.1	2.1218	1.54E-09	2.58E-08	sp|Q866R9|SPY2_CHLAE Protein sprouty homolog 2 OS=Chlorocebus aethiops

				GN=SPRY2 PE=2 SV=1//0
XM_007960691.1	1.0197	1.06E-12	2.41E-11	sp|Q86V85|GP180_HUMAN Integral membrane protein GPR180 OS=Homo sapiens GN=GPR180 PE=2 SV=1//0
XM_007960704.1	0.54438	4.01E-05	0.00038	sp|Q13217|DNJC3_HUMAN DnaJ homolog subfamily C member 3 OS=Homo sapiens GN=DNAJC3 PE=1 SV=1//0
XM_007960755.1	-0.48758	6.20E-10	1.08E-08	sp|P60982|DEST_PIG Destrin OS=Sus scrofa GN=DSTN PE=1 SV=3//2.47196e-110
XM_007960854.1	1.2977	2.07E-52	3.36E-50	sp|P52799|EFNB2_HUMAN Ephrin-B2 OS=Homo sapiens GN=EFNB2 PE=1 SV=1//0
XM_007960855.1	-0.27128	0.007352	0.043914	sp|Q9NWB6|ARGL1_HUMAN Arginine and glutamate-rich protein 1 OS=Homo sapiens GN=ARGLU1 PE=1 SV=1//1.70791e-18
XM_007960867.1	0.80421	3.09E-09	5.03E-08	sp|Q7L211|ABHDD_HUMAN Alpha/beta hydrolase domain-containing protein 13 OS=Homo sapiens GN=ABHD13 PE=2 SV=1//0
XM_007960879.1	2.7252	########	########	sp|Q9Y4H2|IRS2_HUMAN Insulin receptor substrate 2 OS=Homo sapiens GN=IRS2 PE=1 SV=2//0
XM_007960880.1	1.9622	6.55E-76	1.91E-73	sp|Q9NX57|RAB20_HUMAN Ras-related protein Rab-20 OS=Homo sapiens GN=RAB20 PE=1 SV=1//1.19818e-164
XM_007960882.1	-0.23911	0.001399	0.009997	sp|P08572|CO4A2_HUMAN Collagen alpha-2(IV) chain OS=Homo sapiens GN=COL4A2 PE=1 SV=4//8.79501e-06
XM_007960883.1	-1.0267	1.11E-26	6.37E-25	sp|Q7SIB2|CO4A1_BOVIN Collagen alpha-1(IV) chain (Fragment) OS=Bos taurus GN=COL4A1 PE=1 SV=1//1.71235e-151
XM_007960891.1	0.6848	1.17E-06	1.40E-05	sp|Q9HA77|SYCM_HUMAN Probable cysteine--tRNA ligase, mitochondrial OS=Homo sapiens GN=CARS2 PE=1 SV=1//0
XM_007960902.1	-1.1463	0.002227	0.015163	sp|Q9NXR5|ANR10_HUMAN Ankyrin repeat domain-containing protein 10 OS=Homo sapiens GN=ANKRD10 PE=1 SV=2//4.02628e-104
XM_007960922.1	-0.72751	3.00E-20	1.19E-18	sp|O75197|LRP5_HUMAN Low-density lipoprotein receptor-related protein 5 OS=Homo sapiens GN=LRP5 PE=1 SV=2//7.2502e-117
XM_007960937.1	0.75768	0.003023	0.019898	sp|Q96CW5|GCP3_HUMAN Gamma-tubulin complex component 3 OS=Homo sapiens GN=TUBGCP3 PE=1 SV=2//0
XM_007960989.1	-0.38277	1.11E-06	1.33E-05	sp|P11279|LAMP1_HUMAN Lysosome-associated membrane glycoprotein 1 OS=Homo sapiens GN=LAMP1 PE=1 SV=3//0
XM_007961011.1	-0.83863	9.18E-15	2.49E-13	sp|Q14393|GAS6_HUMAN Growth arrest-specific protein 6 OS=Homo sapiens GN=GAS6 PE=1 SV=2//0
XM_007961040.1	-0.66051	0.001855	0.012837	sp|Q15319|PO4F3_HUMAN POU domain, class 4, transcription factor 3 OS=Homo sapiens GN=POU4F3 PE=1 SV=1//1.11342e-122
XM_007961092.1	0.3502	0.006671	0.040433	sp|Q15645|PCH2_HUMAN Pachytene checkpoint protein 2 homolog OS=Homo sapiens GN=TRIP13 PE=1 SV=2//0
XM_007961098.1	0.71815	0.005364	0.033351	sp|Q9H1D9|RPC6_HUMAN DNA-directed RNA polymerase III subunit RPC6 OS=Homo sapiens GN=POLR3F PE=1 SV=1//0
XM_007961146.1	-1.1491	1.94E-06	2.25E-05	sp|Q4R5X8|NDUS6_MACFA NADH dehydrogenase [ubiquinone] iron-sulfur protein 6, mitochondrial OS=Macaca fascicularis GN=NDUFS6 PE=2 SV=1//6.99203e-87
XM_007961166.1	0.56014	2.09E-05	0.000206	sp|Q4R6W4|MED10_MACFA Mediator of RNA polymerase II transcription subunit 10 OS=Macaca fascicularis GN=MED10 PE=2 SV=1//2.86225e-91
XM_007961215.1	0.7132	7.58E-07	9.30E-06	sp|A6NCL7|AN33B_HUMAN Ankyrin repeat domain-containing protein 33B OS=Homo sapiens GN=ANKRD33B PE=3 SV=1//0
XM_007961216.1	-0.62314	3.73E-08	5.28E-07	sp|P51397|DAP1_HUMAN Death-associated protein 1 OS=Homo sapiens GN=DAP PE=1 SV=3//4.15896e-54
XM_007961243.1	0.89128	2.14E-22	9.74E-21	sp|Q96BN8|OTUL_HUMAN Ubiquitin thioesterase otulin OS=Homo sapiens GN=OTULIN PE=1 SV=3//0
XM_007961244.1	0.38344	3.09E-05	0.000297	sp|Q9HCJ1|ANKH_HUMAN Progressive ankylosis protein homolog OS=Homo sapiens GN=ANKH PE=1 SV=2//0
XM_007961248.1	1.3028	4.91E-50	7.16E-48	sp|Q9HD67|MYO10_HUMAN Unconventional myosin-X OS=Homo sapiens GN=MYO10 PE=1 SV=3//0
XM_007961251.1	0.61264	5.17E-09	8.19E-08	sp|Q969S3|ZN622_HUMAN Zinc finger protein 622 OS=Homo sapiens GN=ZNF622 PE=1 SV=1//0
XM_007961271.1	-0.49552	1.53E-11	3.14E-10	sp|P55285|CADH6_HUMAN Cadherin-6 OS=Homo sapiens GN=CDH6 PE=1 SV=1//0
XM_007961272.1	-1.023	3.70E-08	5.25E-07	sp|P55285|CADH6_HUMAN Cadherin-6 OS=Homo sapiens GN=CDH6 PE=1 SV=1//0
XM_007961302.1	-0.5784	5.84E-10	1.02E-08	sp|Q5R6D0|TCP4_PONAB Activated RNA polymerase II transcriptional coactivator p15 OS=Pongo abelii GN=SUB1 PE=2 SV=1//4.69895e-50
XM_007961310.1	1.8831	0.007885	0.04673	sp|P58397|ATS12_HUMAN A disintegrin and metalloproteinase with thrombospondin motifs 12 OS=Homo sapiens GN=ADAMTS12 PE=1 SV=2//0
XM_007961333.1	0.64668	0.000125	0.001094	sp|Q8TDN6|BRX1_HUMAN Ribosome biogenesis protein BRX1 homolog OS=Homo sapiens GN=BRIX1 PE=1 SV=2//0
XM_007961334.1	0.63053	9.43E-05	0.000843	sp|Q5R7X9|RAD1_PONAB Cell cycle checkpoint protein RAD1 OS=Pongo abelii GN=RAD1 PE=2 SV=1//0
XM_007961351.1	-0.35166	0.001309	0.009399	sp|P61600|NAA20_MOUSE N-alpha-acetyltransferase 20 OS=Mus musculus GN=Naa20 PE=2 SV=1//6.71712e-127
XM_007961363.1	4.4693	0.00036	0.00291	sp|Q38IC7|IL7RA_MACFA Interleukin-7 receptor subunit alpha OS=Macaca fascicularis GN=IL7R PE=2 SV=1//0
XM_007961378.1	0.43293	0.000661	0.005078	sp|Q68DH5|LMBD2_HUMAN LMBR1 domain-containing protein 2 OS=Homo sapiens GN=LMBRD2 PE=2 SV=1//0

XM_007961392.1	1.4847	5.45E-40	5.40E-38	sp|P43003|EAA1_HUMAN Excitatory amino acid transporter 1 OS=Homo sapiens GN=SLC1A3 PE=1 SV=1//0
XM_007961398.1	1.615	0.007365	0.043961	sp|Q6KC79|NIPBL_HUMAN Nipped-B-like protein OS=Homo sapiens GN=NIPBL PE=1 SV=2//1.32289e-08
XM_007961415.1	0.60518	6.47E-11	1.25E-09	sp|O75694|NU155_HUMAN Nuclear pore complex protein Nup155 OS=Homo sapiens GN=NUP155 PE=1 SV=1//0
XM_007961444.1	0.80303	9.44E-07	1.15E-05	sp|Q99650|OSMR_HUMAN Oncostatin-M-specific receptor subunit beta OS=Homo sapiens GN=OSMR PE=1 SV=1//0
XM_007961465.1	0.29996	0.001685	0.011799	sp|Q2PPJ7|RGPA2_HUMAN Ral GTPase-activating protein subunit alpha-2 OS=Homo sapiens GN=RALGAPA2 PE=1 SV=2//0
XM_007961467.1	0.67124	5.77E-09	9.06E-08	sp|Q95KZ0|PE2R4_PANTR Prostaglandin E2 receptor EP4 subtype OS=Pan troglodytes GN=PTGER4 PE=2 SV=1//0
XM_007961482.1	0.48766	1.94E-06	2.25E-05	sp|A6NDU8|CE051_HUMAN UPF0600 protein C5orf51 OS=Homo sapiens GN=C5orf51 PE=1 SV=1//1.95727e-168
XM_007961534.1	0.9018	3.62E-12	7.82E-11	sp|Q9NP92|RT30_HUMAN 28S ribosomal protein S30, mitochondrial OS=Homo sapiens GN=MRPS30 PE=1 SV=2//0
XM_007961547.1	0.62886	0.000774	0.005842	-//-
XM_007961625.1	-2.1092	0.00069	0.005276	sp|A8MXJ8|F90A5_HUMAN Putative protein FAM90A5P OS=Homo sapiens GN=FAM90A5P PE=5 SV=1//4.63236e-148
XM_007961633.1	-0.63903	4.89E-10	8.64E-09	sp|Q4R5M2|CATB_MACFA Cathepsin B OS=Macaca fascicularis GN=CTSB PE=2 SV=1//0
XM_007961657.1	1.4858	8.09E-09	1.24E-07	sp|Q96BK5|PINX1_HUMAN PIN2/TERF1-interacting telomerase inhibitor 1 OS=Homo sapiens GN=PINX1 PE=1 SV=2//0
XM_007961676.1	0.29369	0.005211	0.032497	sp|O95271|TNKS1_HUMAN Tankyrase-1 OS=Homo sapiens GN=TNKS PE=1 SV=2//0
XM_007961691.1	1.4979	3.42E-09	5.51E-08	sp|Q86YV5|SG223_HUMAN Tyrosine-protein kinase SgK223 OS=Homo sapiens GN=SGK223 PE=1 SV=4//0
XM_007961732.1	0.6986	3.49E-10	6.26E-09	sp|Q8NEZ2|VP37A_HUMAN Vacuolar protein sorting-associated protein 37A OS=Homo sapiens GN=VPS37A PE=1 SV=1//0
XM_007961733.1	0.39813	2.84E-05	0.000276	sp|Q9UIV1|CNOT7_HUMAN CCR4-NOT transcription complex subunit 7 OS=Homo sapiens GN=CNOT7 PE=1 SV=3//0
XM_007961748.1	1.2232	4.14E-12	8.90E-11	sp|Q2PFX1|PGFRL_MACFA Platelet-derived growth factor receptor-like protein OS=Macaca fascicularis GN=PDGFRL PE=2 SV=1//0
XM_007961775.1	0.93883	1.10E-07	1.48E-06	sp|Q9UKK6|NXT1_HUMAN NTF2-related export protein 1 OS=Homo sapiens GN=NXT1 PE=1 SV=1//7.58403e-98
XM_007961814.1	0.71785	3.09E-17	1.01E-15	sp|P21281|VATB2_HUMAN V-type proton ATPase subunit B, brain isoform OS=Homo sapiens GN=ATP6V1B2 PE=1 SV=3//0
XM_007961873.1	-0.9492	4.18E-08	5.89E-07	sp|P13497|BMP1_HUMAN Bone morphogenetic protein 1 OS=Homo sapiens GN=BMP1 PE=1 SV=2//0
XM_007961900.1	1.1759	4.60E-24	2.33E-22	sp|P48454|PP2BC_HUMAN Serine/threonine-protein phosphatase 2B catalytic subunit gamma isoform OS=Homo sapiens GN=PPP3CC PE=1 SV=3//0
XM_007961907.1	0.66207	1.84E-06	2.14E-05	sp|Q9NQY0|BIN3_HUMAN Bridging integrator 3 OS=Homo sapiens GN=BIN3 PE=1 SV=1//4.50806e-178
XM_007961908.1	-0.80326	1.39E-29	9.19E-28	sp|O19092|CYTC_MACMU Cystatin-C OS=Macaca mulatta GN=CST3 PE=2 SV=1//1.26632e-78
XM_007961930.1	2.2451	3.36E-34	2.71E-32	sp|Q9UBN6|TR10D_HUMAN Tumor necrosis factor receptor superfamily member 10D OS=Homo sapiens GN=TNFRSF10D PE=1 SV=1//3.73838e-23
XM_007961931.1	2.5329	1.93E-36	1.70E-34	sp|Q9UBN6|TR10D_HUMAN Tumor necrosis factor receptor superfamily member 10D OS=Homo sapiens GN=TNFRSF10D PE=1 SV=1//7.12558e-10
XM_007961932.1	1.4737	1.42E-19	5.45E-18	sp|O00220|TR10A_HUMAN Tumor necrosis factor receptor superfamily member 10A OS=Homo sapiens GN=TNFRSF10A PE=1 SV=3//0
XM_007961936.1	-0.46412	5.43E-10	9.53E-09	sp|Q9Y4K0|LOXL2_HUMAN Lysyl oxidase homolog 2 OS=Homo sapiens GN=LOXL2 PE=1 SV=1//0
XM_007961944.1	0.77687	0.000198	0.00167	sp|A6NCS4|NKX26_HUMAN Homeobox protein Nkx-2.6 OS=Homo sapiens GN=NKX2-6 PE=1 SV=1//3.2728e-153
XM_007961968.1	1.0332	1.13E-21	4.86E-20	sp|Q7L273|KCTD9_HUMAN BTB/POZ domain-containing protein KCTD9 OS=Homo sapiens GN=KCTD9 PE=1 SV=1//0
XM_007961971.1	0.97843	2.74E-21	1.16E-19	sp|P63150|2ABA_RABIT Serine/threonine-protein phosphatase 2A 55 kDa regulatory subunit B alpha isoform OS=Oryctolagus cuniculus GN=PPP2R2A PE=2 SV=1//0
XM_007961978.1	-1.832	0.000473	0.003736	sp|Q5R9Y6|DPYL2_PONAB Dihydropyrimidinase-related protein 2 OS=Pongo abelii GN=DPYSL2 PE=2 SV=1//0
XM_007961987.1	0.82632	1.74E-06	2.02E-05	sp|Q9UPQ4|TRI35_HUMAN Tripartite motif-containing protein 35 OS=Homo sapiens GN=TRIM35 PE=1 SV=2//0
XM_007962030.1	-3.8873	0.007557	0.044943	sp|Q9H8N7|ZN395_HUMAN Zinc finger protein 395 OS=Homo sapiens GN=ZNF395 PE=1 SV=2//0
XM_007962035.1	-0.59209	2.30E-06	2.63E-05	sp|Q9H8N7|ZN395_HUMAN Zinc finger protein 395 OS=Homo sapiens GN=ZNF395 PE=1 SV=2//0
XM_007962075.1	3.2286	8.30E-50	1.20E-47	sp|Q13115|DUS4_HUMAN Dual specificity protein phosphatase 4 OS=Homo sapiens GN=DUSP4 PE=1 SV=1//0
XM_007962079.1	0.79763	6.31E-09	9.84E-08	sp|Q5RDE9|LERL1_PONAB Leptin receptor overlapping transcript-like 1

				OS=Pongo abelii GN=LEPROTL1 PE=2 SV=1//4.26377e-81
XM_007962099.1	-0.51851	8.12E-09	1.25E-07	sp|P00390|GSHR_HUMAN Glutathione reductase, mitochondrial OS=Homo sapiens GN=GSR PE=1 SV=2//0
XM_007962105.1	0.31517	0.00245	0.016457	sp|P62716|PP2AB_RAT Serine/threonine-protein phosphatase 2A catalytic subunit beta isoform OS=Rattus norvegicus GN=Ppp2cb PE=2 SV=1//0
XM_007962122.1	-1.4601	9.00E-05	0.000807	sp|Q9UJV8|PURG_HUMAN Purine-rich element-binding protein gamma OS=Homo sapiens GN=PURG PE=2 SV=1//0
XM_007962138.1	-1.0178	2.93E-28	1.82E-26	sp|Q96SM3|CPXM1_HUMAN Probable carboxypeptidase X1 OS=Homo sapiens GN=CPXM1 PE=2 SV=2//0
XM_007962141.1	4.2494	0.003545	0.022974	sp|P43322|NRG1_RAT Pro-neuregulin-1, membrane-bound isoform OS=Rattus norvegicus GN=Nrg1 PE=1 SV=1//2.77845e-102
XM_007962173.1	2.4298	8.48E-44	9.51E-42	sp|Q9HAW0|BRF2_HUMAN Transcription factor IIIB 50 kDa subunit OS=Homo sapiens GN=BRF2 PE=1 SV=1//0
XM_007962176.1	1.2783	1.27E-10	2.40E-09	sp|Q6WKZ4|RFIP1_HUMAN Rab11 family-interacting protein 1 OS=Homo sapiens GN=RAB11FIP1 PE=1 SV=3//0
XM_007962183.1	0.66695	1.10E-08	1.67E-07	sp|Q9UBL3|ASH2L_HUMAN Set1/Ash2 histone methyltransferase complex subunit ASH2 OS=Homo sapiens GN=ASH2L PE=1 SV=1//0
XM_007962207.1	0.94732	9.04E-06	9.41E-05	sp|Q9BZ95|NSD3_HUMAN Histone-lysine N-methyltransferase NSD3 OS=Homo sapiens GN=WHSC1L1 PE=1 SV=1//0
XM_007962231.1	2.1532	0.000143	0.001237	sp|O75410|TACC1_HUMAN Transforming acidic coiled-coil-containing protein 1 OS=Homo sapiens GN=TACC1 PE=1 SV=2//0
XM_007962242.1	0.4792	3.45E-10	6.20E-09	sp|Q13443|ADAM9_HUMAN Disintegrin and metalloproteinase domain-containing protein 9 OS=Homo sapiens GN=ADAM9 PE=1 SV=1//0
XM_007962277.1	-1.1088	3.64E-09	5.86E-08	sp|Q8N474|SFRP1_HUMAN Secreted frizzled-related protein 1 OS=Homo sapiens GN=SFRP1 PE=1 SV=1//1.05807e-157
XM_007962363.1	-4.0937	0.003348	0.021846	sp|Q95JJ0|SG196_MACFA Protein O-mannose kinase OS=Macaca fascicularis GN=POMK PE=2 SV=2//0
XM_007962367.1	-0.29542	0.005726	0.035336	sp|Q68CP4|HGNAT_HUMAN Heparan-alpha-glucosaminide N-acetyltransferase OS=Homo sapiens GN=HGSNAT PE=1 SV=2//0
XM_007962371.1	2.3812	########	########	sp|Q96QB1|RHG07_HUMAN Rho GTPase-activating protein 7 OS=Homo sapiens GN=DLC1 PE=1 SV=4//1.70896e-164
XM_007962376.1	1.3444	5.17E-10	9.11E-09	sp|Q9BXY0|MAK16_HUMAN Protein MAK16 homolog OS=Homo sapiens GN=MAK16 PE=1 SV=2//1.66831e-13
XM_007962413.1	0.96893	1.17E-07	1.56E-06	sp|P17030|ZNF25_HUMAN Zinc finger protein 25 OS=Homo sapiens GN=ZNF25 PE=2 SV=2//0
XM_007962434.1	-1.582	0.005912	0.036295	sp|P07949|RET_HUMAN Proto-oncogene tyrosine-protein kinase receptor Ret OS=Homo sapiens GN=RET PE=1 SV=3//0
XM_007962479.1	-2.085	0.001526	0.010826	sp|P17041|ZNF32_HUMAN Zinc finger protein 32 OS=Homo sapiens GN=ZNF32 PE=1 SV=2//1.52308e-06
XM_007962484.1	0.33586	0.000123	0.001072	sp|P48061|SDF1_HUMAN Stromal cell-derived factor 1 OS=Homo sapiens GN=CXCL12 PE=1 SV=1//9.5587e-44
XM_007962485.1	0.36329	0.002304	0.015631	sp|P48061|SDF1_HUMAN Stromal cell-derived factor 1 OS=Homo sapiens GN=CXCL12 PE=1 SV=1//6.44916e-43
XM_007962486.1	-0.84406	0.004505	0.028518	sp|A0PK05|TMM72_HUMAN Transmembrane protein 72 OS=Homo sapiens GN=TMEM72 PE=2 SV=1//8.16914e-164
XM_007962493.1	1.9755	2.24E-20	8.98E-19	sp|Q5RBE4|DEPP_PONAB Protein DEPP OS=Pongo abelii GN=DEPP PE=2 SV=1//7.1583e-109
XM_007962499.1	4.0739	2.67E-22	1.21E-20	sp|Q9BXX2|AN30B_HUMAN Ankyrin repeat domain-containing protein 30B OS=Homo sapiens GN=ANKRD30B PE=2 SV=3//5.0165e-08
XM_007962559.1	0.27298	0.005288	0.032942	sp|O14925|TIM23_HUMAN Mitochondrial import inner membrane translocase subunit Tim23 OS=Homo sapiens GN=TIMM23 PE=1 SV=1//2.67714e-108
XM_007962667.1	1.2497	0.001033	0.0076	sp|P00367|DHE3_HUMAN Glutamate dehydrogenase 1, mitochondrial OS=Homo sapiens GN=GLUD1 PE=1 SV=2//4.73513e-175
XM_007962669.1	0.66801	2.47E-05	0.000242	sp|A6NIR3|AGAP5_HUMAN Arf-GAP with GTPase, ANK repeat and PH domain-containing protein 5 OS=Homo sapiens GN=AGAP5 PE=2 SV=2//0
XM_007962672.1	-0.6235	3.53E-11	7.02E-10	sp|P00367|DHE3_HUMAN Glutamate dehydrogenase 1, mitochondrial OS=Homo sapiens GN=GLUD1 PE=1 SV=2//0
XM_007962684.1	0.55786	1.17E-05	0.00012	sp|O60269|GRIN2_HUMAN G protein-regulated inducer of neurite outgrowth 2 OS=Homo sapiens GN=GPRIN2 PE=1 SV=2//0
XM_007962701.1	0.41818	0.000152	0.001304	sp|P36894|BMR1A_HUMAN Bone morphogenetic protein receptor type-1A OS=Homo sapiens GN=BMPR1A PE=1 SV=2//0
XM_007962818.1	-0.39397	0.002497	0.016735	sp|Q8N2G6|ZCH24_HUMAN Zinc finger CCHC domain-containing protein 24 OS=Homo sapiens GN=ZCCHC24 PE=1 SV=1//3.57453e-165
XM_007962819.1	1.0701	1.43E-20	5.78E-19	sp|P30405|PPIF_HUMAN Peptidyl-prolyl cis-trans isomerase F, mitochondrial OS=Homo sapiens GN=PPIF PE=1 SV=1//5.67137e-119
XM_007962847.1	1.0393	3.69E-27	2.17E-25	sp|O14802|RPC1_HUMAN DNA-directed RNA polymerase III subunit RPC1 OS=Homo sapiens GN=POLR3A PE=1 SV=2//0
XM_007962905.1	1.5606	6.38E-35	5.35E-33	sp|Q96F45|ZN503_HUMAN Zinc finger protein 503 OS=Homo sapiens GN=ZNF503 PE=1 SV=1//0
XM_007962987.1	-1.6009	0.000313	0.002557	sp|Q13555|KCC2G_HUMAN Calcium/calmodulin-dependent protein kinase type II subunit gamma OS=Homo sapiens GN=CAMK2G PE=1 SV=3//0

XM_007963014.1	-0.55452	0.000629	0.004843	sp|Q96BP2|CHCH1_HUMAN Coiled-coil-helix-coiled-coil-helix domain-containing protein 1 OS=Homo sapiens GN=CHCHD1 PE=1 SV=1//1.97474e-62
XM_007963021.1	-1.5465	0.004491	0.028463	sp|Q495W5|FUT11_HUMAN Alpha-(1,3)-fucosyltransferase 11 OS=Homo sapiens GN=FUT11 PE=1 SV=1//0
XM_007963072.1	0.58936	1.08E-09	1.84E-08	sp|Q8WXX5|DNJC9_HUMAN DnaJ homolog subfamily C member 9 OS=Homo sapiens GN=DNAJC9 PE=1 SV=1//7.7745e-152
XM_007963114.1	0.59518	0.005066	0.031687	sp|Q08E62|DDIT4_BOVIN DNA damage-inducible transcript 4 protein OS=Bos taurus GN=DDIT4 PE=2 SV=1//3.1536e-121
XM_007963125.1	0.46192	0.00343	0.022331	sp|Q7LGC8|CHST3_HUMAN Carbohydrate sulfotransferase 3 OS=Homo sapiens GN=CHST3 PE=1 SV=3//0
XM_007963136.1	0.81489	3.55E-10	6.36E-09	sp|Q9H7M9|GI24_HUMAN Platelet receptor Gi24 OS=Homo sapiens GN=C10orf54 PE=1 SV=3//0
XM_007963148.1	-0.60849	5.78E-06	6.23E-05	sp|O95470|SGPL1_HUMAN Sphingosine-1-phosphate lyase 1 OS=Homo sapiens GN=SGPL1 PE=1 SV=3//0
XM_007963167.1	-1.2921	1.23E-52	2.01E-50	sp|Q13542|4EBP2_HUMAN Eukaryotic translation initiation factor 4E-binding protein 2 OS=Homo sapiens GN=EIF4EBP2 PE=1 SV=1//2.44207e-67
XM_007963218.1	-0.46051	0.000101	0.000897	sp|O95858|TSN15_HUMAN Tetraspanin-15 OS=Homo sapiens GN=TSPAN15 PE=1 SV=1//0
XM_007963229.1	1.3504	2.67E-49	3.81E-47	sp|Q8IYB8|SUV3_HUMAN ATP-dependent RNA helicase SUPV3L1, mitochondrial OS=Homo sapiens GN=SUPV3L1 PE=1 SV=1//0
XM_007963235.1	1.8761	7.00E-84	2.51E-81	sp|Q9NR30|DDX21_HUMAN Nucleolar RNA helicase 2 OS=Homo sapiens GN=DDX21 PE=1 SV=5//0
XM_007963255.1	-1.7501	1.91E-19	7.22E-18	sp|Q8NFU7|TET1_HUMAN Methylcytosine dioxygenase TET1 OS=Homo sapiens GN=TET1 PE=1 SV=2//0
XM_007963295.1	1.1629	1.15E-31	8.17E-30	sp|Q96EB6|SIR1_HUMAN NAD-dependent protein deacetylase sirtuin-1 OS=Homo sapiens GN=SIRT1 PE=1 SV=2//0
XM_007963312.1	-1.0088	1.22E-21	5.26E-20	sp|Q6NUK4|REEP3_HUMAN Receptor expression-enhancing protein 3 OS=Homo sapiens GN=REEP3 PE=1 SV=1//7.16123e-147
XM_007963314.1	0.58863	4.36E-09	6.95E-08	sp|Q15652|JHD2C_HUMAN Probable JmjC domain-containing histone demethylation protein 2C OS=Homo sapiens GN=JMJD1C PE=1 SV=2//0
XM_007963324.1	1.4882	1.04E-44	1.23E-42	sp|Q96SZ5|AEDO_HUMAN 2-aminoethanethiol dioxygenase OS=Homo sapiens GN=ADO PE=1 SV=2//6.75486e-143
XM_007963400.1	0.82062	2.72E-16	8.17E-15	sp|Q4HOT5|TFAM_TRACR Transcription factor A, mitochondrial OS=Trachypithecus cristatus GN=TFAM PE=2 SV=1//8.35155e-146
XM_007963444.1	2.2695	3.20E-24	1.62E-22	sp|O94907|DKK1_HUMAN Dickkopf-related protein 1 OS=Homo sapiens GN=DKK1 PE=1 SV=1//9.39e-148
XM_007963445.1	-1.5455	0.006473	0.039424	sp|P40617|ARL4A_HUMAN ADP-ribosylation factor-like protein 4A OS=Homo sapiens GN=ARL4A PE=1 SV=2//1.89065e-136
XM_007963447.1	0.59048	3.54E-06	3.95E-05	sp|Q9H0L4|CSTFT_HUMAN Cleavage stimulation factor subunit 2 tau variant OS=Homo sapiens GN=CSTF2T PE=1 SV=1//0
XM_007963486.1	-0.66636	2.87E-17	9.43E-16	sp|Q9H1C4|UN93B_HUMAN Protein unc-93 homolog B1 OS=Homo sapiens GN=UNC93B1 PE=1 SV=2//0
XM_007963496.1	2.1355	6.65E-46	8.08E-44	sp|P09913|IFIT2_HUMAN Interferon-induced protein with tetratricopeptide repeats 2 OS=Homo sapiens GN=IFIT2 PE=1 SV=1//0
XM_007963503.1	1.1967	7.96E-06	8.36E-05	sp|Q13325|IFIT5_HUMAN Interferon-induced protein with tetratricopeptide repeats 5 OS=Homo sapiens GN=IFIT5 PE=1 SV=1//0
XM_007963523.1	3.5182	0	0	sp|Q15327|ANKR1_HUMAN Ankyrin repeat domain-containing protein 1 OS=Homo sapiens GN=ANKRD1 PE=1 SV=2//0
XM_007963529.1	1.0879	8.46E-09	1.30E-07	sp|Q5RD78|HECD2_PONAB Probable E3 ubiquitin-protein ligase HECTD2 OS=Pongo abelii GN=HECTD2 PE=2 SV=1//0
XM_007963533.1	3.0583	3.32E-93	1.37E-90	sp|Q9UQK1|PPR3C_HUMAN Protein phosphatase 1 regulatory subunit 3C OS=Homo sapiens GN=PPP1R3C PE=1 SV=2//0
XM_007963534.1	0.82872	1.17E-25	6.34E-24	sp|Q9H2K2|TNKS2_HUMAN Tankyrase-2 OS=Homo sapiens GN=TNKS2 PE=1 SV=1//0
XM_007963544.1	0.53264	3.40E-07	4.32E-06	sp|Q3KNM2|MARH5_MOUSE E3 ubiquitin-protein ligase MARCH5 OS=Mus musculus GN=March5 PE=2 SV=1//0
XM_007963564.1	0.35701	1.77E-05	0.000176	sp|P52732|KIF11_HUMAN Kinesin-like protein KIF11 OS=Homo sapiens GN=KIF11 PE=1 SV=2//0
XM_007963586.1	0.4072	7.77E-09	1.20E-07	sp|Q9NZM1|MYOF_HUMAN Myoferlin OS=Homo sapiens GN=MYOF PE=1 SV=1//0
XM_007963598.1	-2.0458	0.000428	0.003412	sp|P51160|PDE6C_HUMAN Cone cGMP-specific 3',5'-cyclic phosphodiesterase subunit alpha' OS=Homo sapiens GN=PDE6C PE=1 SV=2//0
XM_007963611.1	1.4773	3.14E-09	5.11E-08	sp|Q8WTT2|NOC3L_HUMAN Nucleolar complex protein 3 homolog OS=Homo sapiens GN=NOC3L PE=1 SV=1//0
XM_007963695.1	-0.34852	4.60E-06	5.05E-05	sp|Q9HD45|TM9S3_HUMAN Transmembrane 9 superfamily member 3 OS=Homo sapiens GN=TM9SF3 PE=1 SV=2//0
XM_007963697.1	-0.65234	4.61E-09	7.33E-08	sp|A2T7M0|SCND1_PONPY SCAN domain-containing protein 1 OS=Pongo pygmaeus GN=SCAND1 PE=3 SV=1//2.48117e-67
XM_007963731.1	1.0162	2.37E-06	2.71E-05	sp|Q92837|FRAT1_HUMAN Proto-oncogene FRAT1 OS=Homo sapiens GN=FRAT1 PE=1 SV=3//7.13653e-128
XM_007963732.1	0.79873	0.000212	0.001776	sp|O75474|FRAT2_HUMAN GSK-3-binding protein FRAT2 OS=Homo sapiens GN=FRAT2

				PE=2 SV=3//4.19043e-84
XM_007963747.1	0.45253	0.000574	0.004457	sp|Q9Y3B2|EXOS1_HUMAN Exosome complex component CSL4 OS=Homo sapiens GN=EXOSC1 PE=1 SV=1//2.57887e-140
XM_007963748.1	-0.83491	8.63E-11	1.66E-09	sp|P18669|PGAM1_HUMAN Phosphoglycerate mutase 1 OS=Homo sapiens GN=PGAM1 PE=1 SV=2//1.22234e-87
XM_007963753.1	-0.77338	2.46E-15	6.89E-14	sp|Q86XE5|HOGA1_HUMAN 4-hydroxy-2-oxoglutarate aldolase, mitochondrial OS=Homo sapiens GN=HOGA1 PE=1 SV=1//0
XM_007963755.1	0.63021	0.001001	0.007391	sp|Q5R578|MORN4_PONAB MORN repeat-containing protein 4 OS=Pongo abelii GN=MORN4 PE=2 SV=1//1.37906e-77
XM_007963758.1	1.5187	9.25E-28	5.60E-26	sp|Q5R8D4|AVPI1_PONAB Arginine vasopressin-induced protein 1 OS=Pongo abelii GN=AVPI1 PE=2 SV=1//8.10411e-82
XM_007963759.1	0.2994	0.000771	0.005824	sp|Q9BTU6|P4K2A_HUMAN Phosphatidylinositol 4-kinase type 2-alpha OS=Homo sapiens GN=PI4K2A PE=1 SV=1//0
XM_007963762.1	-0.72736	2.47E-12	5.43E-11	sp|Q9BSK0|MALD1_HUMAN MARVEL domain-containing protein 1 OS=Homo sapiens GN=MARVELD1 PE=1 SV=1//1.34911e-72
XM_007963814.1	0.59055	5.66E-06	6.12E-05	sp|Q92887|MRP2_HUMAN Canalicular multispecific organic anion transporter 1 OS=Homo sapiens GN=ABCC2 PE=1 SV=3//0
XM_007963821.1	0.5805	1.26E-06	1.50E-05	sp|Q9NQZ7|ENTP7_HUMAN Ectonucleoside triphosphate diphosphohydrolase 7 OS=Homo sapiens GN=ENTPD7 PE=2 SV=1//0
XM_007963822.1	1.5869	1.17E-15	3.38E-14	sp|Q6XZF7|DNMBP_HUMAN Dynamin-binding protein OS=Homo sapiens GN=DNMBP PE=1 SV=1//0
XM_007963823.1	1.5583	1.05E-33	8.26E-32	sp|Q6XZF7|DNMBP_HUMAN Dynamin-binding protein OS=Homo sapiens GN=DNMBP PE=1 SV=1//0
XM_007963830.1	0.71323	0.001205	0.008732	sp|O15111|IKKA_HUMAN Inhibitor of nuclear factor kappa-B kinase subunit alpha OS=Homo sapiens GN=CHUK PE=1 SV=2//0
XM_007963838.1	0.62461	1.80E-07	2.36E-06	sp|Q6QNY1|BL1S2_HUMAN Biogenesis of lysosome-related organelles complex 1 subunit 2 OS=Homo sapiens GN=BLOC1S2 PE=1 SV=1//4.86722e-59
XM_007963839.1	-0.60219	1.34E-18	4.79E-17	sp|O00767|ACOD_HUMAN Acyl-CoA desaturase OS=Homo sapiens GN=SCD PE=1 SV=2//0
XM_007963849.1	-0.74813	1.82E-14	4.79E-13	sp|O95169|NDUB8_HUMAN NADH dehydrogenase [ubiquinone] 1 beta subcomplex subunit 8, mitochondrial OS=Homo sapiens GN=NDUFB8 PE=1 SV=1//3.72823e-124
XM_007963850.1	0.53366	5.28E-07	6.59E-06	sp|Q9NWT6|HIF1N_HUMAN Hypoxia-inducible factor 1-alpha inhibitor OS=Homo sapiens GN=HIF1AN PE=1 SV=2//0
XM_007963860.1	0.92863	0.00103	0.007583	sp|Q96RR1|PEO1_HUMAN Twinkle protein, mitochondrial OS=Homo sapiens GN=PEO1 PE=1 SV=1//0
XM_007963876.1	-0.35529	0.001783	0.012405	sp|Q9BWM7|SFXN3_HUMAN Sideroflexin-3 OS=Homo sapiens GN=SFXN3 PE=1 SV=2//0
XM_007963878.1	-2.3662	6.99E-05	0.00064	sp|Q9BRK4|LZTS2_HUMAN Leucine zipper putative tumor suppressor 2 OS=Homo sapiens GN=LZTS2 PE=1 SV=2//0
XM_007963884.1	-1.599	0.001504	0.010685	-//-
XM_007963934.1	-2.9445	0.003116	0.020448	sp|Q8WN03|KCIP2_MUSPF Kv channel-interacting protein 2 OS=Mustela putorius furo GN=Kcnip2 PE=2 SV=1//3.97147e-131
XM_007963935.1	-3.6674	0.001716	0.011995	sp|Q8WN03|KCIP2_MUSPF Kv channel-interacting protein 2 OS=Mustela putorius furo GN=Kcnip2 PE=2 SV=1//2.36957e-130
XM_007963939.1	0.90485	1.40E-10	2.64E-09	sp|Q86YV9|HPS6_HUMAN Hermansky-Pudlak syndrome 6 protein OS=Homo sapiens GN=HPS6 PE=1 SV=1//0
XM_007963975.1	0.33169	0.000514	0.004027	sp|P85515|ACTZ_RAT Alpha-centractin OS=Rattus norvegicus GN=Actr1a PE=1 SV=1//0
XM_007963982.1	0.28803	0.000173	0.001475	sp|Q9BZR9|TRIM8_HUMAN Probable E3 ubiquitin-protein ligase TRIM8 OS=Homo sapiens GN=TRIM8 PE=1 SV=2//0
XM_007963995.1	-0.51185	0.003387	0.022086	sp|Q96B45|CJ032_HUMAN UPF0693 protein C10orf32 OS=Homo sapiens GN=C10orf32 PE=1 SV=1//4.57544e-63
XM_007964001.1	-0.99336	0.005167	0.032278	sp|Q9ESN1|DOC2G_MOUSE Double C2-like domain-containing protein gamma OS=Mus musculus GN=Doc2g PE=2 SV=1//0
XM_007964007.1	1.0484	2.45E-15	6.89E-14	sp|Q9BYE7|PCGF6_HUMAN Polycomb group RING finger protein 6 OS=Homo sapiens GN=PCGF6 PE=1 SV=2//0
XM_007964010.1	0.80303	4.76E-06	5.21E-05	sp|Q15542|TAF5_HUMAN Transcription initiation factor TFIID subunit 5 OS=Homo sapiens GN=TAF5 PE=1 SV=3//0
XM_007964038.1	-0.71701	0.000724	0.005506	sp|Q8NDM7|CFA43_HUMAN Cilia- and flagella-associated protein 43 OS=Homo sapiens GN=CFAP43 PE=2 SV=3//0
XM_007964039.1	-0.68117	0.000225	0.001879	sp|P78417|GSTO1_HUMAN Glutathione S-transferase omega-1 OS=Homo sapiens GN=GSTO1 PE=1 SV=2//1.24102e-169
XM_007964043.1	1.7588	2.14E-22	9.75E-21	sp|Q8IWB1|IPRI_HUMAN Inositol 1,4,5-trisphosphate receptor-interacting protein OS=Homo sapiens GN=ITPRIP PE=1 SV=1//0
XM_007964056.1	-0.96055	1.10E-09	1.88E-08	sp|Q5RBA4|MYL9_PONAB Myosin regulatory light polypeptide 9 OS=Pongo abelii GN=MYL9 PE=2 SV=3//1.71856e-106
XM_007964059.1	-0.71412	4.23E-19	1.56E-17	sp|Q6PDV7|RL10_RAT 60S ribosomal protein L10 OS=Rattus norvegicus GN=Rpl10 PE=1 SV=3//3.07116e-157
XM_007964077.1	1.9342	3.32E-39	3.23E-37	sp|Q16690|DUS5_HUMAN Dual specificity protein phosphatase 5 OS=Homo sapiens GN=DUSP5 PE=1 SV=2//0
XM_007964078.1	0.31079	0.004868	0.030648	sp|Q5R4K5|SMC3_PONAB Structural maintenance of chromosomes protein 3

				OS=Pongo abelii GN=SMC3 PE=2 SV=1//0
XM_007964094.1	-0.55548	0.000277	0.00228	sp|Q8WV74|NUDT8_HUMAN Nucleoside diphosphate-linked moiety X motif 8, mitochondrial OS=Homo sapiens GN=NUDT8 PE=2 SV=2//3.99056e-143
XM_007964096.1	0.75487	1.08E-20	4.44E-19	sp|Q9HCL2|GPAT1_HUMAN Glycerol-3-phosphate acyltransferase 1, mitochondrial OS=Homo sapiens GN=GPAM PE=1 SV=3//0
XM_007964114.1	-0.49963	3.54E-06	3.95E-05	sp|Q86VF7|NRAP_HUMAN Nebulin-related-anchoring protein OS=Homo sapiens GN=NRAP PE=2 SV=2//0
XM_007964115.1	-0.48408	1.31E-07	1.75E-06	sp|Q14520|HABP2_HUMAN Hyaluronan-binding protein 2 OS=Homo sapiens GN=HABP2 PE=1 SV=1//0
XM_007964116.1	-0.99802	1.81E-06	2.11E-05	sp|Q14520|HABP2_HUMAN Hyaluronan-binding protein 2 OS=Homo sapiens GN=HABP2 PE=1 SV=1//0
XM_007964121.1	-0.52076	5.73E-08	7.93E-07	sp|O94964|SOGA1_HUMAN Protein SOGA1 OS=Homo sapiens GN=SOGA1 PE=1 SV=2//0
XM_007964123.1	0.94504	5.54E-14	1.40E-12	sp|Q8NBF2|NHLC2_HUMAN NHL repeat-containing protein 2 OS=Homo sapiens GN=NHLRC2 PE=1 SV=1//0
XM_007964128.1	1.9858	0.000229	0.001908	sp|P47899|ADRB1_MACMU Beta-1 adrenergic receptor OS=Macaca mulatta GN=ADRB1 PE=3 SV=1//0
XM_007964129.1	0.55666	0.000904	0.006724	sp|Q7Z3E2|CC186_HUMAN Coiled-coil domain-containing protein 186 OS=Homo sapiens GN=CCDC186 PE=1 SV=2//0
XM_007964169.1	1.433	5.33E-52	8.39E-50	sp|Q5W0V3|F16B1_HUMAN Protein FAM160B1 OS=Homo sapiens GN=FAM160B1 PE=1 SV=1//0
XM_007964170.1	0.59875	2.52E-11	5.07E-10	sp|Q8WWH5|TRUB1_HUMAN Probable tRNA pseudouridine synthase 1 OS=Homo sapiens GN=TRUB1 PE=1 SV=1//0
XM_007964205.1	-0.60915	1.64E-10	3.06E-09	sp|Q8HXQ9|NDUV1_MACFA NADH dehydrogenase [ubiquinone] flavoprotein 1, mitochondrial OS=Macaca fascicularis GN=NDUFV1 PE=2 SV=1//0
XM_007964222.1	0.96001	1.66E-19	6.37E-18	sp|Q05940|VMAT2_HUMAN Synaptic vesicular amine transporter OS=Homo sapiens GN=SLC18A2 PE=1 SV=2//0
XM_007964223.1	1.1483	5.22E-52	8.28E-50	sp|Q8NEN9|PDZD8_HUMAN PDZ domain-containing protein 8 OS=Homo sapiens GN=PDZD8 PE=1 SV=1//0
XM_007964236.1	0.50047	3.88E-11	7.69E-10	sp|Q86Y37|CACL1_HUMAN CDK2-associated and cullin domain-containing protein 1 OS=Homo sapiens GN=CACUL1 PE=1 SV=1//0
XM_007964241.1	-1.7405	0.005316	0.033092	sp|Q6P4A7|SFXN4_HUMAN Sideroflexin-4 OS=Homo sapiens GN=SFXN4 PE=1 SV=1//3.8655e-107
XM_007964245.1	-0.97507	4.49E-30	3.03E-28	sp|Q5REY3|PRDX3_PONAB Thioredoxin-dependent peroxide reductase, mitochondrial OS=Pongo abelii GN=PRDX3 PE=2 SV=1//0
XM_007964272.1	0.25451	0.005205	0.032472	sp|Q9BZH6|WDR11_HUMAN WD repeat-containing protein 11 OS=Homo sapiens GN=WDR11 PE=1 SV=1//0
XM_007964287.1	0.48749	3.82E-06	4.23E-05	sp|O95359|TACC2_HUMAN Transforming acidic coiled-coil-containing protein 2 OS=Homo sapiens GN=TACC2 PE=1 SV=3//0
XM_007964297.1	-0.24735	0.001013	0.007469	sp|Q92743|HTRA1_HUMAN Serine protease HTRA1 OS=Homo sapiens GN=HTRA1 PE=1 SV=1//0
XM_007964306.1	-0.98373	2.36E-32	1.73E-30	sp|P48047|ATPO_HUMAN ATP synthase subunit O, mitochondrial OS=Homo sapiens GN=ATP5O PE=1 SV=1//6.19186e-149
XM_007964318.1	1.6732	4.59E-36	3.99E-34	sp|Q9H8K7|CJ088_HUMAN Uncharacterized protein C10orf88 OS=Homo sapiens GN=C10orf88 PE=1 SV=2//0
XM_007964320.1	-0.53521	2.68E-08	3.86E-07	sp|Q5RF40|ACDSB_PONAB Short/branched chain specific acyl-CoA dehydrogenase, mitochondrial OS=Pongo abelii GN=ACADSB PE=2 SV=1//0
XM_007964322.1	-0.44887	3.15E-05	0.000302	sp|Q9WVA3|BUB3_MOUSE Mitotic checkpoint protein BUB3 OS=Mus musculus GN=Bub3 PE=2 SV=2//0
XM_007964340.1	-1.1498	5.37E-13	1.26E-11	sp|Q9H008|LHPP_HUMAN Phospholysine phosphohistidine inorganic pyrophosphate phosphatase OS=Homo sapiens GN=LHPP PE=1 SV=2//2.11948e-170
XM_007964344.1	0.31128	0.001135	0.008279	sp|Q9UD57|NKX12_HUMAN NK1 transcription factor-related protein 2 OS=Homo sapiens GN=NKX1-2 PE=2 SV=3//1.84517e-101
XM_007964386.1	-2.4628	0.003016	0.019858	sp|Q9P287|BCCIP_HUMAN BRCA2 and CDKN1A-interacting protein OS=Homo sapiens GN=BCCIP PE=1 SV=1//1.68298e-109
XM_007964389.1	-1.02	0.007763	0.046052	sp|Q9P287|BCCIP_HUMAN BRCA2 and CDKN1A-interacting protein OS=Homo sapiens GN=BCCIP PE=1 SV=1//9.07358e-159
XM_007964392.1	1.9558	3.48E-31	2.43E-29	sp|O43184|ADA12_HUMAN Disintegrin and metalloproteinase domain-containing protein 12 OS=Homo sapiens GN=ADAM12 PE=1 SV=3//0
XM_007964402.1	-1.3624	0.005907	0.036275	sp|P18583|SON_HUMAN Protein SON OS=Homo sapiens GN=SON PE=1 SV=4//2.29547e-126
XM_007964422.1	0.40778	0.004946	0.031031	sp|P18583|SON_HUMAN Protein SON OS=Homo sapiens GN=SON PE=1 SV=4//3.69309e-07
XM_007964452.1	-1.4097	1.70E-09	2.83E-08	sp|Q12983|BNIP3_HUMAN BCL2/adenovirus E1B 19 kDa protein-interacting protein 3 OS=Homo sapiens GN=BNIP3 PE=1 SV=2//4.40385e-95
XM_007964496.1	-0.66743	3.35E-05	0.000321	sp|Q8IYW2|CFA46_HUMAN Cilia- and flagella-associated protein 46 OS=Homo sapiens GN=CFAP46 PE=2 SV=3//0
XM_007964500.1	6.9609	8.31E-62	1.73E-59	sp|Q5JTH9|RRP12_HUMAN RRP12-like protein OS=Homo sapiens GN=RRP12 PE=1 SV=2//1.4013e-46
XM_007964514.1	0.90677	0.000252	0.002092	sp|P16260|GDC_HUMAN Graves disease carrier protein OS=Homo sapiens GN=SLC25A16 PE=1 SV=3//1.57818e-08

XM_007964519.1	0.33294	0.000102	0.000902	sp|Q53EZ4|CEP55_HUMAN Centrosomal protein of 55 kDa OS=Homo sapiens GN=CEP55 PE=1 SV=3//0
XM_007964523.1	-0.52567	2.82E-08	4.06E-07	sp|P18669|PGAM1_HUMAN Phosphoglycerate mutase 1 OS=Homo sapiens GN=PGAM1 PE=1 SV=2//2.58919e-73
XM_007964525.1	0.41125	0.001184	0.0086	sp|Q5T2E6|CJ076_HUMAN UPF0668 protein C10orf76 OS=Homo sapiens GN=C10orf76 PE=2 SV=1//0
XM_007964533.1	0.54339	3.42E-08	4.87E-07	sp|Q9NQB0|TF7L2_HUMAN Transcription factor 7-like 2 OS=Homo sapiens GN=TCF7L2 PE=1 SV=2//4.1998e-20
XM_007964535.1	-0.34186	0.000921	0.006831	sp|Q8TCE6|FA45A_HUMAN Protein FAM45A OS=Homo sapiens GN=FAM45A PE=2 SV=1//0
XM_007964537.1	0.48691	1.92E-07	2.51E-06	sp|Q2PFX0|ATE1_MACFA Arginyl-tRNA--protein transferase 1 OS=Macaca fascicularis GN=ATE1 PE=2 SV=1//0
XM_007964539.1	3.5797	3.97E-08	5.62E-07	sp|P0C7Q2|ARMS2_HUMAN Age-related maculopathy susceptibility protein 2 OS=Homo sapiens GN=ARMS2 PE=2 SV=1//9.58638e-33
XM_007964542.1	0.99005	1.69E-17	5.66E-16	sp|Q15018|F175B_HUMAN BRISC complex subunit Abro1 OS=Homo sapiens GN=FAM175B PE=1 SV=2//0
XM_007964553.1	-0.35377	0.000108	0.000954	sp|Q96NT0|CC115_HUMAN Coiled-coil domain-containing protein 115 OS=Homo sapiens GN=CCDC115 PE=2 SV=1//4.84147e-98
XM_007964559.1	0.39333	3.80E-06	4.21E-05	sp|Q9NXE4|NSMA3_HUMAN Sphingomyelin phosphodiesterase 4 OS=Homo sapiens GN=SMPD4 PE=1 SV=2//0
XM_007964571.1	0.96621	3.37E-40	3.39E-38	sp|O60243|H6ST1_HUMAN Heparan-sulfate 6-O-sulfotransferase 1 OS=Homo sapiens GN=HS6ST1 PE=1 SV=5//0
XM_007964593.1	0.83293	6.66E-16	1.95E-14	sp|Q9D7M8|RPB4_MOUSE DNA-directed RNA polymerase II subunit RPB4 OS=Mus musculus GN=Polr2d PE=2 SV=2//7.30188e-91
XM_007964595.1	-1.2454	0.005193	0.032405	sp|P38484|INGR2_HUMAN Interferon gamma receptor 2 OS=Homo sapiens GN=IFNGR2 PE=1 SV=2//0
XM_007964598.1	1.3968	3.42E-10	6.15E-09	sp|Q587I9|SFT2C_HUMAN Vesicle transport protein SFT2C OS=Homo sapiens GN=SFT2D3 PE=2 SV=1//2.03802e-20
XM_007964615.1	0.77564	1.60E-23	7.80E-22	sp|Q9Y2U5|M3K2_HUMAN Mitogen-activated protein kinase kinase kinase 2 OS=Homo sapiens GN=MAP3K2 PE=1 SV=2//0
XM_007964616.1	0.45934	6.98E-07	8.60E-06	sp|Q60HG1|ERCC3_MACFA TFIIH basal transcription factor complex helicase XPB subunit OS=Macaca fascicularis GN=ERCC3 PE=2 SV=1//0
XM_007964650.1	1.0718	7.99E-14	2.00E-12	sp|Q9BYG3|MK67I_HUMAN MKI67 FHA domain-interacting nucleolar phosphoprotein OS=Homo sapiens GN=NIFK PE=1 SV=1//2.22376e-169
XM_007964687.1	-0.54056	6.83E-05	0.000627	sp|Q9NZI6|TF2L1_HUMAN Transcription factor CP2-like protein 1 OS=Homo sapiens GN=TFCP2L1 PE=2 SV=1//0
XM_007964700.1	0.83547	1.61E-18	5.72E-17	sp|Q9H7F4|T185B_HUMAN Transmembrane protein 185B OS=Homo sapiens GN=TMEM185B PE=1 SV=2//0
XM_007964707.1	2.2429	1.12E-06	1.34E-05	sp|Q13516|OLIG2_HUMAN Oligodendrocyte transcription factor 2 OS=Homo sapiens GN=OLIG2 PE=2 SV=2//8.71027e-18
XM_007964708.1	1.4044	1.94E-06	2.24E-05	sp|Q9MYU8|E41L5_CANFA Band 4.1-like protein 5 OS=Canis familiaris GN=EPB41L5 PE=2 SV=1//0
XM_007964711.1	0.37397	8.71E-05	0.000784	sp|P29074|PTN4_HUMAN Tyrosine-protein phosphatase non-receptor type 4 OS=Homo sapiens GN=PTPN4 PE=1 SV=1//5.50534e-13
XM_007964731.1	-2.0546	0.007805	0.04628	sp|P07108|ACBP_HUMAN Acyl-CoA-binding protein OS=Homo sapiens GN=DBI PE=1 SV=2//3.56402e-35
XM_007964751.1	0.57259	3.87E-12	8.36E-11	sp|Q9NVP1|DDX18_HUMAN ATP-dependent RNA helicase DDX18 OS=Homo sapiens GN=DDX18 PE=1 SV=2//0
XM_007964756.1	0.61844	2.78E-05	0.00027	sp|Q5R8D1|EIF3J_PONAB Eukaryotic translation initiation factor 3 subunit J OS=Pongo abelii GN=EIF3J PE=2 SV=1//1.87164e-49
XM_007964757.1	0.53971	0.000754	0.005708	sp|Q96JI7|SPTCS_HUMAN Spatacsin OS=Homo sapiens GN=SPG11 PE=1 SV=3//1.57961e-177
XM_007964758.1	-1.2352	0.002436	0.01638	sp|Q9Y5B6|PAXB1_HUMAN PAX3- and PAX7-binding protein 1 OS=Homo sapiens GN=PAXBP1 PE=1 SV=2//0
XM_007964765.1	0.39398	0.004764	0.030064	sp|P57076|CU059_HUMAN UPF0769 protein C21orf59 OS=Homo sapiens GN=C21orf59 PE=1 SV=1//0
XM_007964768.1	2.4967	0.003488	0.022628	sp|O43194|GPR39_HUMAN G-protein coupled receptor 39 OS=Homo sapiens GN=GPR39 PE=1 SV=1//4.59297e-101
XM_007964809.1	-0.9587	0.000106	0.000934	sp|O60583|CCNT2_HUMAN Cyclin-T2 OS=Homo sapiens GN=CCNT2 PE=1 SV=2//0
XM_007964853.1	0.46027	5.27E-09	8.34E-08	sp|Q92575|UBXN4_HUMAN UBX domain-containing protein 4 OS=Homo sapiens GN=UBXN4 PE=1 SV=2//0
XM_007964855.1	2.3364	0.003625	0.023427	sp|P09848|LPH_HUMAN Lactase-phlorizin hydrolase OS=Homo sapiens GN=LCT PE=1 SV=3//0
XM_007964863.1	-0.7858	9.64E-27	5.55E-25	sp|P14618|KPYM_HUMAN Pyruvate kinase PKM OS=Homo sapiens GN=PKM PE=1 SV=4//0
XM_007964950.1	-1.1806	5.73E-17	1.84E-15	sp|Q9BE24|LDHA_MACFA L-lactate dehydrogenase A chain OS=Macaca fascicularis GN=LDHA PE=2 SV=4//0
XM_007964951.1	0.36983	0.001035	0.007608	sp|Q9H3L0|MMAD_HUMAN Methylmalonic aciduria and homocystinuria type D protein, mitochondrial OS=Homo sapiens GN=MMADHC PE=1 SV=2//0
XM_007964953.1	2.7188	5.46E-97	2.49E-94	sp|Q6SA80|RND3_RAT Rho-related GTP-binding protein RhoE OS=Rattus norvegicus GN=Rnd3 PE=2 SV=1//1.32127e-162

XM_007965011.1	0.67493	8.39E-06	8.78E-05	sp|Q9NYP9|MS18A_HUMAN Protein Mis18-alpha OS=Homo sapiens GN=MIS18A PE=1 SV=1//1.99579e-133
XM_007965027.1	1.3539	3.01E-16	9.01E-15	sp|Q68UT7|HUNK_PANTR Hormonally up-regulated neu tumor-associated kinase OS=Pan troglodytes GN=HUNK PE=3 SV=1//0
XM_007965029.1	2.1037	0.001472	0.010477	sp|Q99569|PKP4_HUMAN Plakophilin-4 OS=Homo sapiens GN=PKP4 PE=1 SV=2//0
XM_007965040.1	1.4546	0.000417	0.003328	-//-
XM_007965134.1	-0.9709	1.06E-23	5.25E-22	sp|Q8HXQ0|SODC_MACMU Superoxide dismutase [Cu-Zn] OS=Macaca mulatta GN=SOD1 PE=2 SV=3//2.94506e-105
XM_007965214.1	-0.96064	0.002412	0.01625	sp|Q99250|SCN2A_HUMAN Sodium channel protein type 2 subunit alpha OS=Homo sapiens GN=SCN2A PE=1 SV=3//0
XM_007965224.1	-0.47579	1.27E-06	1.51E-05	sp|Q7Z4L5|TT21B_HUMAN Tetratricopeptide repeat protein 21B OS=Homo sapiens GN=TTC21B PE=1 SV=2//0
XM_007965236.1	-0.497	0.000907	0.00674	sp|Q15858|SCN9A_HUMAN Sodium channel protein type 9 subunit alpha OS=Homo sapiens GN=SCN9A PE=1 SV=3//0
XM_007965248.1	-0.51724	0.000521	0.004079	sp|A2BDB0|ACTG_XENLA Actin, cytoplasmic 2 OS=Xenopus laevis GN=actg1 PE=2 SV=1//0
XM_007965302.1	1.2991	2.86E-10	5.18E-09	sp|Q0VFZ6|CC173_HUMAN Coiled-coil domain-containing protein 173 OS=Homo sapiens GN=CCDC173 PE=2 SV=2//0
XM_007965316.1	0.38717	1.29E-05	0.00013	sp|Q6ZT12|UBR3_HUMAN E3 ubiquitin-protein ligase UBR3 OS=Homo sapiens GN=UBR3 PE=2 SV=2//0
XM_007965327.1	0.49864	9.59E-10	1.64E-08	sp|Q9H8Y8|GORS2_HUMAN Golgi reassembly-stacking protein 2 OS=Homo sapiens GN=GORASP2 PE=1 SV=3//0
XM_007965335.1	-1.2989	4.04E-09	6.49E-08	sp|Q53TN4|CYBR1_HUMAN Cytochrome b reductase 1 OS=Homo sapiens GN=CYBRD1 PE=1 SV=1//1.13681e-162
XM_007965360.1	1.4	1.46E-05	0.000147	sp|Q07687|DLX2_HUMAN Homeobox protein DLX-2 OS=Homo sapiens GN=DLX2 PE=1 SV=2//2.63312e-145
XM_007965380.1	1.5175	6.21E-09	9.71E-08	sp|Q9NYL2|MLTK_HUMAN Mitogen-activated protein kinase kinase kinase MLT OS=Homo sapiens GN=ZAK PE=1 SV=3//0
XM_007965420.1	-0.75692	4.42E-18	1.53E-16	sp|Q71S46|AT5G3_RAT ATP synthase F(0) complex subunit C3, mitochondrial OS=Rattus norvegicus GN=Atp5g3 PE=2 SV=1//5.87815e-74
XM_007965425.1	1.072	8.60E-24	4.29E-22	sp|P35453|HXD13_HUMAN Homeobox protein Hox-D13 OS=Homo sapiens GN=HOXD13 PE=1 SV=3//7.16944e-162
XM_007965426.1	-0.71682	0.003222	0.021107	sp|P28358|HXD10_HUMAN Homeobox protein Hox-D10 OS=Homo sapiens GN=HOXD10 PE=1 SV=2//0
XM_007965434.1	-1.1314	2.74E-07	3.52E-06	sp|A2D5I1|HXD4_LAGLA Homeobox protein Hox-D4 OS=Lagothrix lagotricha GN=HOXD4 PE=3 SV=1//2.14997e-110
XM_007965440.1	-0.45078	4.98E-10	8.79E-09	sp|P51991|ROA3_HUMAN Heterogeneous nuclear ribonucleoprotein A3 OS=Homo sapiens GN=HNRNPA3 PE=1 SV=2//9.89502e-123
XM_007965471.1	1.2134	5.14E-26	2.84E-24	sp|Q9HB20|PKHA3_HUMAN Pleckstrin homology domain-containing family A member 3 OS=Homo sapiens GN=PLEKHA3 PE=1 SV=2//6.93045e-178
XM_007965569.1	1.4424	3.16E-33	2.44E-31	sp|Q8NFH5|NUP53_HUMAN Nucleoporin NUP53 OS=Homo sapiens GN=NUP35 PE=1 SV=1//0
XM_007965572.1	0.39889	3.67E-05	0.000349	sp|Q8WU90|ZC3HF_HUMAN Zinc finger CCCH domain-containing protein 15 OS=Homo sapiens GN=ZC3H15 PE=1 SV=1//0
XM_007965573.1	0.27911	0.000848	0.006348	sp|P06756|ITAV_HUMAN Integrin alpha-V OS=Homo sapiens GN=ITGAV PE=1 SV=2//0
XM_007965594.1	-1.5812	0.001762	0.012278	sp|Q9UBP9|GULP1_HUMAN PTB domain-containing engulfment adapter protein 1 OS=Homo sapiens GN=GULP1 PE=1 SV=1//0
XM_007965604.1	-0.47164	6.43E-09	1.00E-07	sp|P05997|CO5A2_HUMAN Collagen alpha-2(V) chain OS=Homo sapiens GN=COL5A2 PE=1 SV=3//4.87664e-175
XM_007965606.1	0.62009	1.04E-06	1.26E-05	sp|Q8IWA0|WDR75_HUMAN WD repeat-containing protein 75 OS=Homo sapiens GN=WDR75 PE=1 SV=1//0
XM_007965656.1	1.4685	1.11E-09	1.89E-08	sp|A6NFY4|NEMP2_HUMAN Nuclear envelope integral membrane protein 2 OS=Homo sapiens GN=NEMP2 PE=2 SV=3//0
XM_007965665.1	-0.33081	0.000819	0.006158	sp|O94925|GLSK_HUMAN Glutaminase kidney isoform, mitochondrial OS=Homo sapiens GN=GLS PE=1 SV=1//0
XM_007965668.1	-0.87628	0.001567	0.011091	sp|O94925|GLSK_HUMAN Glutaminase kidney isoform, mitochondrial OS=Homo sapiens GN=GLS PE=1 SV=1//0
XM_007965714.1	1.5505	3.81E-54	6.41E-52	sp|O94768|ST17B_HUMAN Serine/threonine-protein kinase 17B OS=Homo sapiens GN=STK17B PE=1 SV=1//0
XM_007965727.1	0.52665	5.16E-06	5.61E-05	sp|Q9Y5Q9|TF3C3_HUMAN General transcription factor 3C polypeptide 3 OS=Homo sapiens GN=GTF3C3 PE=1 SV=1//0
XM_007965729.1	-0.63185	1.21E-09	2.04E-08	sp|Q75T13|PGAP1_HUMAN GPI inositol-deacylase OS=Homo sapiens GN=PGAP1 PE=1 SV=1//0
XM_007965741.1	1.0689	5.63E-08	7.80E-07	sp|Q9H8M1|CQ10B_HUMAN Coenzyme Q-binding protein COQ10 homolog B, mitochondrial OS=Homo sapiens GN=COQ10B PE=2 SV=1//4.90297e-160
XM_007965748.1	-1.3003	0.001216	0.008798	sp|P26772|CH10_RAT 10 kDa heat shock protein, mitochondrial OS=Rattus norvegicus GN=Hspe1 PE=1 SV=3//1.75372e-56
XM_007965770.1	1.0016	2.99E-08	4.28E-07	sp|A2RUC4|TYW5_HUMAN tRNA wybutosine-synthesizing protein 5 OS=Homo sapiens GN=TYW5 PE=1 SV=1//0

XM_007965798.1	1.5009	2.65E-55	4.67E-53	sp|P49759|CLK1_HUMAN Dual specificity protein kinase CLK1 OS=Homo sapiens GN=CLK1 PE=1 SV=2//0
XM_007965813.1	0.9875	0.000209	0.001758	sp|Q5R977|F126B_PONAB Protein FAM126B OS=Pongo abelii GN=FAM126B PE=2 SV=1//0
XM_007965857.1	0.58178	0.000237	0.001974	sp|Q96Q45|TM237_HUMAN Transmembrane protein 237 OS=Homo sapiens GN=TMEM237 PE=1 SV=2//0
XM_007965883.1	-0.501	8.23E-06	8.62E-05	sp|Q5I0H3|SUMO1_RAT Small ubiquitin-related modifier 1 OS=Rattus norvegicus GN=Sumo1 PE=1 SV=1//3.96523e-64
XM_007965911.1	-0.6277	0.001579	0.011157	sp|Q6UW02|CP20A_HUMAN Cytochrome P450 20A1 OS=Homo sapiens GN=CYP20A1 PE=1 SV=1//0
XM_007965938.1	1.7746	5.00E-88	1.95E-85	sp|Q9UHI8|ATS1_HUMAN A disintegrin and metalloproteinase with thrombospondin motifs 1 OS=Homo sapiens GN=ADAMTS1 PE=1 SV=4//0
XM_007966033.1	0.76444	4.74E-06	5.19E-05	sp|Q13467|FZD5_HUMAN Frizzled-5 OS=Homo sapiens GN=FZD5 PE=1 SV=2//0
XM_007966037.1	1.2962	8.55E-12	1.79E-10	sp|Q6ZWE6|PKHM3_HUMAN Pleckstrin homology domain-containing family M member 3 OS=Homo sapiens GN=PLEKHM3 PE=2 SV=2//0
XM_007966041.1	-2.0765	0.003422	0.022289	sp|P07320|CRGD_HUMAN Gamma-crystallin D OS=Homo sapiens GN=CRYGD PE=1 SV=3//9.00174e-118
XM_007966192.1	-0.48291	0.000962	0.00712	sp|Q9BY49|PECR_HUMAN Peroxisomal trans-2-enoyl-CoA reductase OS=Homo sapiens GN=PECR PE=1 SV=2//0
XM_007966202.1	-0.29597	0.001011	0.007461	sp|P13010|XRCC5_HUMAN X-ray repair cross-complementing protein 5 OS=Homo sapiens GN=XRCC5 PE=1 SV=3//0
XM_007966212.1	-0.63449	6.56E-06	6.98E-05	sp|P61515|RL37P_RAT Putative 60S ribosomal protein L37a OS=Rattus norvegicus GN=Rpl37a-ps1 PE=5 SV=2//2.86886e-60
XM_007966218.1	-0.91294	3.00E-17	9.83E-16	sp|P18065|IBP2_HUMAN Insulin-like growth factor-binding protein 2 OS=Homo sapiens GN=IGFBP2 PE=1 SV=2//0
XM_007966279.1	0.72162	8.53E-10	1.46E-08	sp|Q5R6Z6|RCD1_PONAB Cell differentiation protein RCD1 homolog OS=Pongo abelii GN=RQCD1 PE=2 SV=2//0
XM_007966307.1	0.71091	0.000411	0.003283	sp|Q96BH1|RNF25_HUMAN E3 ubiquitin-protein ligase RNF25 OS=Homo sapiens GN=RNF25 PE=1 SV=1//0
XM_007966314.1	-0.73886	1.80E-19	6.85E-18	sp|Q02318|CP27A_HUMAN Sterol 26-hydroxylase, mitochondrial OS=Homo sapiens GN=CYP27A1 PE=1 SV=1//0
XM_007966398.1	#NAME?	0.003819	0.024619	sp|Q15772|SPEG_HUMAN Striated muscle preferentially expressed protein kinase OS=Homo sapiens GN=SPEG PE=1 SV=4//5.65376e-06
XM_007966414.1	-0.77127	5.14E-09	8.15E-08	sp|Q8IZ52|CHSS2_HUMAN Chondroitin sulfate synthase 2 OS=Homo sapiens GN=CHPF PE=1 SV=2//0
XM_007966441.1	0.70306	1.73E-07	2.27E-06	sp|Q9NSD9|SYFB_HUMAN Phenylalanine--tRNA ligase beta subunit OS=Homo sapiens GN=FARSB PE=1 SV=3//0
XM_007966443.1	0.57198	1.82E-13	4.44E-12	sp|O95573|ACSL3_HUMAN Long-chain-fatty-acid--CoA ligase 3 OS=Homo sapiens GN=ACSL3 PE=1 SV=3//0
XM_007966452.1	1.0159	7.81E-38	7.24E-36	sp|Q8IWB7|WDFY1_HUMAN WD repeat and FYVE domain-containing protein 1 OS=Homo sapiens GN=WDFY1 PE=1 SV=1//0
XM_007966472.1	2.4743	########	########	sp|Q28224|IRS1_CHLAE Insulin receptor substrate 1 OS=Chlorocebus aethiops GN=IRS1 PE=2 SV=1//0
XM_007966529.1	2.0543	1.45E-77	4.67E-75	sp|Q3UBG2|PCLI1_MOUSE PTB-containing, cubilin and LRP1-interacting protein OS=Mus musculus GN=Pid1 PE=1 SV=2//9.31419e-139
XM_007966566.1	1.0989	8.58E-09	1.31E-07	sp|Q9H930|SP14L_HUMAN Nuclear body protein SP140-like protein OS=Homo sapiens GN=SP140L PE=2 SV=3//0
XM_007966597.1	0.93249	9.25E-29	5.92E-27	sp|Q9Y376|CAB39_HUMAN Calcium-binding protein 39 OS=Homo sapiens GN=CAB39 PE=1 SV=1//0
XM_007966598.1	-1.0088	7.75E-21	3.21E-19	sp|Q9NQX7|ITM2C_HUMAN Integral membrane protein 2C OS=Homo sapiens GN=ITM2C PE=1 SV=1//6.37035e-149
XM_007966665.1	-0.54964	0.000689	0.005268	sp|Q9BUP0|EFHD1_HUMAN EF-hand domain-containing protein D1 OS=Homo sapiens GN=EFHD1 PE=1 SV=1//5.78786e-94
XM_007966730.1	1.7271	3.37E-74	9.46E-72	sp|P61208|ARL4C_MOUSE ADP-ribosylation factor-like protein 4C OS=Mus musculus GN=Arl4c PE=2 SV=1//3.21315e-118
XM_007966731.1	2.1971	########	########	sp|Q9P0V3|SH3B4_HUMAN SH3 domain-binding protein 4 OS=Homo sapiens GN=SH3BP4 PE=1 SV=1//0
XM_007966780.1	0.57015	1.80E-09	2.99E-08	sp|Q5RBX2|BET1L_PONAB BET1-like protein OS=Pongo abelii GN=BET1L PE=3 SV=1//1.6261e-65
XM_007966844.1	0.54302	0.000155	0.001329	sp|Q9H0C8|ILKAP_HUMAN Integrin-linked kinase-associated serine/threonine phosphatase 2C OS=Homo sapiens GN=ILKAP PE=1 SV=1//0
XM_007966854.1	0.54528	0.000114	0.000999	sp|Q9Y576|ASB1_HUMAN Ankyrin repeat and SOCS box protein 1 OS=Homo sapiens GN=ASB1 PE=1 SV=1//0
XM_007966885.1	-0.6843	1.92E-09	3.17E-08	sp|Q8WXC6|MYOV2_HUMAN Myeloma-overexpressed gene 2 protein OS=Homo sapiens GN=MYEOV2 PE=1 SV=3//1.5029e-19
XM_007966904.1	-1.0021	4.62E-21	1.93E-19	sp|P35052|GPC1_HUMAN Glypican-1 OS=Homo sapiens GN=GPC1 PE=1 SV=2//0
XM_007966946.1	0.70167	1.25E-20	5.10E-19	sp|P48552|NRIP1_HUMAN Nuclear receptor-interacting protein 1 OS=Homo sapiens GN=NRIP1 PE=1 SV=2//0
XM_007966984.1	-0.79583	0.000695	0.005307	sp|Q6IWH7|ANO7_HUMAN Anoctamin-7 OS=Homo sapiens GN=ANO7 PE=1 SV=2//0
XM_007967047.1	0.43136	0.001358	0.009725	sp|Q5H9S7|DCA17_HUMAN DDB1- and CUL4-associated factor 17 OS=Homo sapiens

				GN=DCAF17 PE=1 SV=1//0
XM_007967054.1	1.2107	0.000837	0.006277	sp|E5RQL4|FONG_HUMAN Formiminotransferase N-terminal subdomain-containing protein OS=Homo sapiens GN=FTCDNL1 PE=2 SV=1//1.38094e-70
XM_007967063.1	-0.30302	0.001103	0.008072	sp|Q5R5S4|PSMD1_PONAB 26S proteasome non-ATPase regulatory subunit 1 OS=Pongo abelii GN=PSMD1 PE=2 SV=1//0
XM_007967098.1	0.73586	5.95E-20	2.32E-18	sp|P29375|KDM5A_HUMAN Lysine-specific demethylase 5A OS=Homo sapiens GN=KDM5A PE=1 SV=3//0
XM_007967158.1	0.49646	2.79E-06	3.16E-05	sp|Q8BID8|FXL14_MOUSE F-box/LRR-repeat protein 14 OS=Mus musculus GN=Fbxl14 PE=2 SV=1//0
XM_007967273.1	0.36572	0.007366	0.043961	sp|Q9NQ88|TIGAR_HUMAN Fructose-2,6-bisphosphatase TIGAR OS=Homo sapiens GN=TIGAR PE=1 SV=1//0
XM_007967276.1	1.2123	0.001273	0.009172	sp|Q5RES7|RCAN1_PONAB Calcipressin-1 OS=Pongo abelii GN=RCAN1 PE=2 SV=1//2.36554e-90
XM_007967277.1	0.31402	0.002316	0.015691	sp|Q5RD58|CL004_PONAB Protein C12orf4 homolog OS=Pongo abelii PE=2 SV=1//0
XM_007967301.1	-0.61439	6.40E-12	1.35E-10	sp|Q0MQB3|NDUA9_GORGO NADH dehydrogenase [ubiquinone] 1 alpha subcomplex subunit 9, mitochondrial OS=Gorilla gorilla gorilla GN=NDUFA9 PE=2 SV=2//0
XM_007967339.1	-1.8545	3.45E-18	1.20E-16	sp|P23763|VAMP1_HUMAN Vesicle-associated membrane protein 1 OS=Homo sapiens GN=VAMP1 PE=1 SV=1//1.3118e-49
XM_007967342.1	-0.84302	6.26E-35	5.27E-33	sp|P04406|G3P_HUMAN Glyceraldehyde-3-phosphate dehydrogenase OS=Homo sapiens GN=GAPDH PE=1 SV=3//0
XM_007967346.1	-0.52936	5.45E-08	7.57E-07	sp|Q4U2R6|RM51_HUMAN 39S ribosomal protein L51, mitochondrial OS=Homo sapiens GN=MRPL51 PE=1 SV=1//1.3486e-82
XM_007967389.1	2.0364	1.54E-05	0.000155	-//-
XM_007967404.1	-1.0716	3.02E-47	3.94E-45	-//-
XM_007967411.1	-1.4976	1.69E-05	0.000169	sp|Q16538|GP162_HUMAN Probable G-protein coupled receptor 162 OS=Homo sapiens GN=GPR162 PE=2 SV=1//0
XM_007967423.1	-0.95576	1.18E-40	1.21E-38	sp|P60174|TPIS_HUMAN Triosephosphate isomerase OS=Homo sapiens GN=TPI1 PE=1 SV=3//0
XM_007967439.1	-2.2943	5.60E-15	1.54E-13	sp|Q53EV4|LRC23_HUMAN Leucine-rich repeat-containing protein 23 OS=Homo sapiens GN=LRRC23 PE=2 SV=2//5.4757e-138
XM_007967442.1	-1.0288	1.61E-16	4.96E-15	sp|O35127|C10_MOUSE Protein C10 OS=Mus musculus GN=Grcc10 PE=2 SV=1//5.38351e-67
XM_007967446.1	-1.2097	0.001474	0.010485	sp|P29350|PTN6_HUMAN Tyrosine-protein phosphatase non-receptor type 6 OS=Homo sapiens GN=PTPN6 PE=1 SV=1//0
XM_007967448.1	3.3572	1.04E-58	2.09E-56	sp|Q92979|NEP1_HUMAN Ribosomal RNA small subunit methyltransferase NEP1 OS=Homo sapiens GN=EMG1 PE=1 SV=4//8.94042e-176
XM_007967449.1	-0.67825	3.91E-17	1.27E-15	sp|Q6P1A2|MBOA5_HUMAN Lysophospholipid acyltransferase 5 OS=Homo sapiens GN=LPCAT3 PE=1 SV=1//0
XM_007967451.1	-0.60128	0.00154	0.010919	sp|Q9NZP8|C1RL_HUMAN Complement C1r subcomponent-like protein OS=Homo sapiens GN=C1RL PE=1 SV=2//0
XM_007967455.1	-2.6069	0.004938	0.031002	sp|Q5RBM7|RET5_PONAB Retinol-binding protein 5 OS=Pongo abelii GN=RBP5 PE=2 SV=3//5.76943e-94
XM_007967486.1	1.281	1.18E-58	2.35E-56	sp|P11169|GTR3_HUMAN Solute carrier family 2, facilitated glucose transporter member 3 OS=Homo sapiens GN=SLC2A3 PE=2 SV=1//0
XM_007967487.1	0.68972	6.08E-10	1.06E-08	sp|Q9P0K8|FOXJ2_HUMAN Forkhead box protein J2 OS=Homo sapiens GN=FOXJ2 PE=1 SV=1//0
XM_007967489.1	1.5515	0.000617	0.004758	sp|Q6TAC8|C3AR_MACFA C3a anaphylatoxin chemotactic receptor OS=Macaca fascicularis GN=C3AR1 PE=2 SV=1//0
XM_007967490.1	1.0864	9.80E-25	5.10E-23	sp|Q5R630|NECP1_PONAB Adaptin ear-binding coat-associated protein 1 OS=Pongo abelii GN=NECAP1 PE=2 SV=1//1.38771e-147
XM_007967499.1	-0.67262	6.05E-13	1.41E-11	sp|Q8MI29|CBR1_MACFA Carbonyl reductase [NADPH] 1 OS=Macaca fascicularis GN=CBR1 PE=2 SV=1//0
XM_007967604.1	-0.56165	9.62E-06	9.96E-05	sp|Q15370|ELOB_HUMAN Transcription elongation factor B polypeptide 2 OS=Homo sapiens GN=TCEB2 PE=1 SV=1//2.13345e-80
XM_007967608.1	-1.0607	4.46E-06	4.90E-05	sp|Q4KMG9|TM52B_HUMAN Transmembrane protein 52B OS=Homo sapiens GN=TMEM52B PE=2 SV=1//1.6793e-85
XM_007967609.1	1.7104	3.03E-77	9.20E-75	sp|Q5BIZ2|GBRL1_XENTR Gamma-aminobutyric acid receptor-associated protein-like 1 OS=Xenopus tropicalis GN=gabarapl1 PE=3 SV=1//5.79418e-74
XM_007967630.1	0.80196	4.55E-17	1.47E-15	sp|Q13112|CAF1B_HUMAN Chromatin assembly factor 1 subunit B OS=Homo sapiens GN=CHAF1B PE=1 SV=1//0
XM_007967696.1	-1.2763	4.65E-11	9.14E-10	sp|Q95KG7|MANS1_MACFA MANSC domain-containing protein 1 OS=Macaca fascicularis GN=MANSC1 PE=2 SV=1//0
XM_007967699.1	1.2919	0.001272	0.009172	sp|Q9BY84|DUS16_HUMAN Dual specificity protein phosphatase 16 OS=Homo sapiens GN=DUSP16 PE=1 SV=1//0
XM_007967702.1	0.48653	0.000301	0.002463	sp|O60519|CRBL2_HUMAN cAMP-responsive element-binding protein-like 2 OS=Homo sapiens GN=CREBL2 PE=1 SV=1//1.66853e-59
XM_007967704.1	-0.80974	0.001717	0.012	sp|P46527|CDN1B_HUMAN Cyclin-dependent kinase inhibitor 1B OS=Homo sapiens GN=CDKN1B PE=1 SV=1//4.10182e-133
XM_007967707.1	-4.6652	0.000149	0.001284	sp|Q96LR9|APLD1_HUMAN Apolipoprotein L domain-containing protein 1 OS=Homo sapiens GN=APOLD1 PE=2 SV=2//5.68245e-99

XM_007967718.1	-0.74398	2.33E-07	3.02E-06	sp|Q9NRV9|HEBP1_HUMAN Heme-binding protein 1 OS=Homo sapiens GN=HEBP1 PE=1 SV=1//1.3155e-133
XM_007967719.1	1.8831	5.48E-29	3.53E-27	sp|A2RU67|K1467_HUMAN Uncharacterized protein KIAA1467 OS=Homo sapiens GN=KIAA1467 PE=1 SV=1//0
XM_007967725.1	4.4165	3.63E-40	3.63E-38	sp|Q5RCY3|EMP1_PONAB Epithelial membrane protein 1 OS=Pongo abelii GN=EMP1 PE=2 SV=1//2.05711e-71
XM_007967740.1	1.2312	0.007365	0.043961	sp|Q28DR4|H4_XENTR Histone H4 OS=Xenopus tropicalis GN=TGas006m08.1 PE=3 SV=1//4.09329e-46
XM_007967741.1	-1.1306	1.58E-35	1.36E-33	sp|Q4R3X5|H2AJ_MACFA Histone H2A.J OS=Macaca fascicularis GN=H2AFJ PE=2 SV=1//6.40341e-85
XM_007967772.1	-0.37196	4.80E-05	0.00045	sp|P53804|TTC3_HUMAN E3 ubiquitin-protein ligase TTC3 OS=Homo sapiens GN=TTC3 PE=1 SV=2//0
XM_007967773.1	0.4108	1.72E-07	2.27E-06	sp|Q9Y3F4|STRAP_HUMAN Serine-threonine kinase receptor-associated protein OS=Homo sapiens GN=STRAP PE=1 SV=1//0
XM_007967774.1	-0.29812	0.001719	0.012009	sp|Q9Y315|DEOC_HUMAN Deoxyribose-phosphate aldolase OS=Homo sapiens GN=DERA PE=1 SV=2//0
XM_007967875.1	-0.48885	1.61E-11	3.30E-10	sp|Q4R5B6|LDHB_MACFA L-lactate dehydrogenase B chain OS=Macaca fascicularis GN=LDHB PE=2 SV=3//0
XM_007967876.1	1.1901	6.72E-07	8.30E-06	sp|Q15842|KCNJ8_HUMAN ATP-sensitive inward rectifier potassium channel 8 OS=Homo sapiens GN=KCNJ8 PE=1 SV=1//0
XM_007967903.1	-3.9422	0.000138	0.001192	sp|Q86YS7|C2CD5_HUMAN C2 domain-containing protein 5 OS=Homo sapiens GN=C2CD5 PE=1 SV=1//0
XM_007967905.1	0.28454	0.000427	0.003401	sp|Q9HBU6|EKI1_HUMAN Ethanolamine kinase 1 OS=Homo sapiens GN=ETNK1 PE=1 SV=1//0
XM_007967990.1	3.0888	6.88E-07	8.48E-06	sp|Q9C0J9|BHE41_HUMAN Class E basic helix-loop-helix protein 41 OS=Homo sapiens GN=BHLHE41 PE=1 SV=1//2.8825e-44
XM_007967994.1	-1.5842	0.003306	0.021581	sp|Q9NVK5|FGOP2_HUMAN FGFR1 oncogene partner 2 OS=Homo sapiens GN=FGFR1OP2 PE=1 SV=1//5.41099e-172
XM_007967996.1	0.86924	1.72E-21	7.38E-20	sp|Q9NVM9|ASUN_HUMAN Protein asunder homolog OS=Homo sapiens GN=ASUN PE=1 SV=2//0
XM_007967999.1	0.51413	1.65E-06	1.93E-05	sp|Q4R6N3|MED21_MACFA Mediator of RNA polymerase II transcription subunit 21 OS=Macaca fascicularis GN=MED21 PE=2 SV=1//5.90935e-94
XM_007968037.1	0.88837	1.24E-16	3.85E-15	sp|Q9P2K6|KLH42_HUMAN Kelch-like protein 42 OS=Homo sapiens GN=KLHL42 PE=1 SV=2//0
XM_007968089.1	-0.33962	0.00016	0.001372	sp|O15397|IPO8_HUMAN Importin-8 OS=Homo sapiens GN=IPO8 PE=1 SV=2//0
XM_007968100.1	1.1671	9.57E-33	7.15E-31	sp|Q6ZUT9|DEN5B_HUMAN DENN domain-containing protein 5B OS=Homo sapiens GN=DENND5B PE=1 SV=2//0
XM_007968116.1	#NAME?	0.005864	0.036071	sp|Q96G01|BICD1_HUMAN Protein bicaudal D homolog 1 OS=Homo sapiens GN=BICD1 PE=1 SV=3//0
XM_007968132.1	0.93077	1.11E-27	6.66E-26	sp|Q99959|PKP2_HUMAN Plakophilin-2 OS=Homo sapiens GN=PKP2 PE=1 SV=2//0
XM_007968133.1	2.3043	########	4.03E-97	sp|P15036|ETS2_HUMAN Protein C-ets-2 OS=Homo sapiens GN=ETS2 PE=1 SV=1//0
XM_007968140.1	0.38214	5.36E-05	0.000499	sp|Q9DC53|CPNE8_MOUSE Copine-8 OS=Mus musculus GN=Cpne8 PE=2 SV=3//0
XM_007968144.1	-1.4655	8.03E-17	2.53E-15	sp|Q4R577|C1R_MACFA Complement C1r subcomponent OS=Macaca fascicularis GN=C1R PE=2 SV=1//0
XM_007968155.1	0.75355	5.31E-18	1.83E-16	sp|Q9NP50|FA60A_HUMAN Protein FAM60A OS=Homo sapiens GN=FAM60A PE=1 SV=1//4.64056e-106
XM_007968156.1	1.0283	7.03E-23	3.31E-21	sp|Q96M96|FGD4_HUMAN FYVE, RhoGEF and PH domain-containing protein 4 OS=Homo sapiens GN=FGD4 PE=1 SV=2//0
XM_007968161.1	1.7095	1.73E-18	6.09E-17	sp|Q5R6W3|RBM18_PONAB Probable RNA-binding protein 18 OS=Pongo abelii GN=RBM18 PE=2 SV=1//1.54264e-130
XM_007968177.1	-0.67179	1.63E-10	3.06E-09	sp|P0CB92|NDUA8_PONPY NADH dehydrogenase [ubiquinone] 1 alpha subcomplex subunit 8 OS=Pongo pygmaeus GN=NDUFA8 PE=2 SV=1//2.89381e-102
XM_007968180.1	1.653	1.34E-07	1.78E-06	sp|A4Q9F4|TTL11_MOUSE Tubulin polyglutamylase TTLL11 OS=Mus musculus GN=Ttll11 PE=2 SV=1//0
XM_007968201.1	-1.389	1.48E-09	2.48E-08	sp|Q9NSI6|BRWD1_HUMAN Bromodomain and WD repeat-containing protein 1 OS=Homo sapiens GN=BRWD1 PE=1 SV=4//0
XM_007968212.1	-0.44799	5.18E-08	7.22E-07	sp|P05114|HMGN1_HUMAN Non-histone chromosomal protein HMG-14 OS=Homo sapiens GN=HMGN1 PE=1 SV=3//7.62395e-31
XM_007968213.1	-0.98132	7.17E-10	1.24E-08	sp|O15145|ARPC3_HUMAN Actin-related protein 2/3 complex subunit 3 OS=Homo sapiens GN=ARPC3 PE=1 SV=3//1.32394e-120
XM_007968245.1	-0.64167	3.71E-16	1.11E-14	sp|P62138|PP1A_RAT Serine/threonine-protein phosphatase PP1-alpha catalytic subunit OS=Rattus norvegicus GN=Ppp1ca PE=1 SV=1//0
XM_007968260.1	1.8929	0.000127	0.001108	sp|Q13219|PAPP1_HUMAN Pappalysin-1 OS=Homo sapiens GN=PAPPA PE=1 SV=3//0
XM_007968284.1	2.6019	4.24E-10	7.56E-09	sp|O95150|TNF15_HUMAN Tumor necrosis factor ligand superfamily member 15 OS=Homo sapiens GN=TNFSF15 PE=1 SV=2//1.44153e-176
XM_007968323.1	-0.78397	0.000386	0.003102	sp|P02760|AMBP_HUMAN Protein AMBP OS=Homo sapiens GN=AMBP PE=1 SV=1//0
XM_007968345.1	0.68804	0.000541	0.004216	sp|P49796|RGS3_HUMAN Regulator of G-protein signaling 3 OS=Homo sapiens GN=RGS3 PE=1 SV=2//0
XM_007968351.1	0.69538	1.39E-07	1.84E-06	sp|Q5R4W3|DPOE3_PONAB DNA polymerase epsilon subunit 3 OS=Pongo abelii

				GN=POLE3 PE=2 SV=1//2.32697e-60
XM_007968369.1	-0.30701	0.00547	0.03394	sp|Q5RAS6|COPT1_PONAB High affinity copper uptake protein 1 OS=Pongo abelii GN=SLC31A1 PE=2 SV=1//2.74474e-104
XM_007968393.1	0.7062	3.07E-17	1.01E-15	sp|Q5VWJ9|SNX30_HUMAN Sorting nexin-30 OS=Homo sapiens GN=SNX30 PE=1 SV=1//0
XM_007968416.1	1.6884	########	########	sp|Q16739|CEGT_HUMAN Ceramide glucosyltransferase OS=Homo sapiens GN=UGCG PE=1 SV=1//0
XM_007968424.1	1.7568	9.42E-39	9.01E-37	sp|Q9H1X3|DJC25_HUMAN DnaJ homolog subfamily C member 25 OS=Homo sapiens GN=DNAJC25 PE=1 SV=1//2.81765e-180
XM_007968434.1	-1.5365	5.24E-05	0.000489	sp|Q8TF39|ZN483_HUMAN Zinc finger protein 483 OS=Homo sapiens GN=ZNF483 PE=1 SV=3//0
XM_007968459.1	-0.77656	7.89E-22	3.45E-20	sp|P29451|THIO_MACMU Thioredoxin OS=Macaca mulatta GN=TXN PE=3 SV=2//8.10551e-69
XM_007968476.1	0.5359	1.36E-06	1.60E-05	sp|B2RYE5|E41LB_RAT Band 4.1-like protein 4B OS=Rattus norvegicus GN=Epb41l4b PE=2 SV=1//0
XM_007968490.1	0.29838	0.000101	0.000899	sp|P54727|RD23B_HUMAN UV excision repair protein RAD23 homolog B OS=Homo sapiens GN=RAD23B PE=1 SV=1//0
XM_007968515.1	0.8058	2.55E-07	3.30E-06	sp|Q9NVV0|TM38B_HUMAN Trimeric intracellular cation channel type B OS=Homo sapiens GN=TMEM38B PE=1 SV=1//0
XM_007968516.1	0.46742	0.00067	0.005135	sp|Q9BXM9|FSD1L_HUMAN FSD1-like protein OS=Homo sapiens GN=FSD1L PE=1 SV=2//0
XM_007968532.1	-0.62375	0.00052	0.004074	sp|Q5RAA9|NPS3A_PONAB Protein NipSnap homolog 3A OS=Pongo abelii GN=NIPSNAP3A PE=2 SV=1//1.99594e-165
XM_007968594.1	0.51346	0.000395	0.003168	sp|P56962|STX17_HUMAN Syntaxin-17 OS=Homo sapiens GN=STX17 PE=1 SV=2//1.83031e-160
XM_007968599.1	-0.46935	6.34E-05	0.000584	sp|Q5RB31|SC61B_PONAB Protein transport protein Sec61 subunit beta OS=Pongo abelii GN=SEC61B PE=3 SV=3//9.9777e-39
XM_007968600.1	-0.4938	0.000193	0.001631	sp|Q9H553|ALG2_HUMAN Alpha-1,3/1,6-mannosyltransferase ALG2 OS=Homo sapiens GN=ALG2 PE=1 SV=1//0
XM_007968609.1	-1.6348	0.000346	0.002802	sp|Q8IXK2|GLT12_HUMAN Polypeptide N-acetylgalactosaminyltransferase 12 OS=Homo sapiens GN=GALNT12 PE=1 SV=3//0
XM_007968653.1	0.79209	1.02E-10	1.94E-09	sp|Q5RCP1|TSTD2_PONAB Thiosulfate sulfurtransferase/rhodanese-like domain-containing protein 2 OS=Pongo abelii GN=TSTD2 PE=2 SV=1//0
XM_007968663.1	0.83352	8.66E-24	4.31E-22	sp|Q15464|SHB_HUMAN SH2 domain-containing adapter protein B OS=Homo sapiens GN=SHB PE=1 SV=2//0
XM_007968704.1	0.63004	1.98E-06	2.28E-05	sp|Q9GZS1|RPA49_HUMAN DNA-directed RNA polymerase I subunit RPA49 OS=Homo sapiens GN=POLR1E PE=1 SV=2//0
XM_007968709.1	-0.6729	0.005778	0.035632	sp|Q9UBQ7|GRHPR_HUMAN Glyoxylate reductase/hydroxypyruvate reductase OS=Homo sapiens GN=GRHPR PE=1 SV=1//0
XM_007968770.1	0.45836	0.008049	0.047596	sp|Q6UXD1|HRCT1_HUMAN Histidine-rich carboxyl terminus protein 1 OS=Homo sapiens GN=HRCT1 PE=4 SV=1//9.83161e-19
XM_007968778.1	0.56479	1.03E-05	0.000107	sp|Q9HCG7|GBA2_HUMAN Non-lysosomal glucosylceramidase OS=Homo sapiens GN=GBA2 PE=1 SV=2//0
XM_007968795.1	2.1265	2.27E-57	4.22E-55	sp|P57078|RIPK4_HUMAN Receptor-interacting serine/threonine-protein kinase 4 OS=Homo sapiens GN=RIPK4 PE=1 SV=1//0
XM_007968871.1	-0.87277	0.007159	0.042957	sp|Q01853|TERA_MOUSE Transitional endoplasmic reticulum ATPase OS=Mus musculus GN=Vcp PE=1 SV=4//0
XM_007968912.1	-1.0865	0.005175	0.032324	sp|A6NKF2|ARI3C_HUMAN AT-rich interactive domain-containing protein 3C OS=Homo sapiens GN=ARID3C PE=3 SV=1//9.7366e-157
XM_007968941.1	-1.2576	1.13E-05	0.000116	sp|Q8K1D8|ENHO_MOUSE Adropin OS=Mus musculus GN=Enho PE=2 SV=1//1.86156e-12
XM_007968994.1	1.026	0.000236	0.001963	sp|Q12986|NFX1_HUMAN Transcriptional repressor NF-X1 OS=Homo sapiens GN=NFX1 PE=1 SV=2//0
XM_007968996.1	1.3713	3.74E-10	6.69E-09	sp|Q12986|NFX1_HUMAN Transcriptional repressor NF-X1 OS=Homo sapiens GN=NFX1 PE=1 SV=2//0
XM_007969002.1	0.98163	3.01E-13	7.25E-12	sp|Q99933|BAG1_HUMAN BAG family molecular chaperone regulator 1 OS=Homo sapiens GN=BAG1 PE=1 SV=4//0
XM_007969024.1	-0.5532	6.48E-10	1.13E-08	sp|P31689|DNJA1_HUMAN DnaJ homolog subfamily A member 1 OS=Homo sapiens GN=DNAJA1 PE=1 SV=2//0
XM_007969028.1	-1.2415	9.25E-23	4.32E-21	sp|P0CB94|NDUB6_PONPY NADH dehydrogenase [ubiquinone] 1 beta subcomplex subunit 6 OS=Pongo pygmaeus GN=NDUFB6 PE=2 SV=1//2.87259e-65
XM_007969031.1	-0.66327	0.001284	0.009239	sp|P21399|ACOC_HUMAN Cytoplasmic aconitate hydratase OS=Homo sapiens GN=ACO1 PE=1 SV=3//0
XM_007969032.1	2.7995	8.83E-88	3.39E-85	sp|O95786|DDX58_HUMAN Probable ATP-dependent RNA helicase DDX58 OS=Homo sapiens GN=DDX58 PE=1 SV=2//0
XM_007969038.1	-1.0529	1.41E-50	2.12E-48	sp|P60707|ACTB_TRIVU Actin, cytoplasmic 1 OS=Trichosurus vulpecula GN=ACTB PE=2 SV=1//0
XM_007969043.1	-0.72809	0.001622	0.011429	sp|Q86TA1|MOB3B_HUMAN MOB kinase activator 3B OS=Homo sapiens GN=MOB3B PE=1 SV=2//5.28267e-146
XM_007969127.1	1.2944	9.15E-19	3.29E-17	sp|Q9UI40|NCKX2_HUMAN Sodium/potassium/calcium exchanger 2 OS=Homo sapiens GN=SLC24A2 PE=1 SV=1//0

XM_007969132.1	-0.39563	2.18E-08	3.19E-07	sp|P62755|RS6_RAT 40S ribosomal protein S6 OS=Rattus norvegicus GN=Rps6 PE=1 SV=1//6.92248e-149
XM_007969147.1	-0.40964	1.80E-05	0.000179	sp|Q63486|RRAGA_RAT Ras-related GTP-binding protein A OS=Rattus norvegicus GN=Rraga PE=1 SV=1//0
XM_007969206.1	-0.62742	0.00046	0.003641	sp|Q5RB84|ZDH21_PONAB Probable palmitoyltransferase ZDHHC21 OS=Pongo abelii GN=ZDHHC21 PE=2 SV=1//3.36393e-162
XM_007969242.1	2.9428	4.48E-77	1.34E-74	sp|Q8IV03|LUR1L_HUMAN Leucine rich adaptor protein 1-like OS=Homo sapiens GN=LURAP1L PE=1 SV=2//5.07407e-91
XM_007969343.1	1.5291	2.44E-05	0.000239	sp|Q9Y2P8|RCL1_HUMAN RNA 3'-terminal phosphate cyclase-like protein OS=Homo sapiens GN=RCL1 PE=1 SV=3//0
XM_007969349.1	1.1615	4.41E-19	1.62E-17	sp|Q7L3B6|CD37L_HUMAN Hsp90 co-chaperone Cdc37-like 1 OS=Homo sapiens GN=CDC37L1 PE=1 SV=1//0
XM_007969350.1	0.75027	1.43E-06	1.68E-05	sp|Q8IY26|PPAC2_HUMAN Presqualene diphosphate phosphatase OS=Homo sapiens GN=PPAPDC2 PE=1 SV=3//4.94127e-154
XM_007969360.1	1.2426	9.13E-38	8.43E-36	sp|Q8NEA6|GLIS3_HUMAN Zinc finger protein GLIS3 OS=Homo sapiens GN=GLIS3 PE=2 SV=5//0
XM_007969370.1	1.1939	6.94E-08	9.52E-07	sp|P55347|PKNX1_HUMAN Homeobox protein PKNOX1 OS=Homo sapiens GN=PKNOX1 PE=1 SV=3//3.23211e-33
XM_007969373.1	0.73411	8.97E-12	1.88E-10	sp|Q15397|K0020_HUMAN Pumilio domain-containing protein KIAA0020 OS=Homo sapiens GN=KIAA0020 PE=1 SV=3//0
XM_007969408.1	-0.88731	3.83E-13	9.08E-12	sp|P02488|CRYAA_MACMU Alpha-crystallin A chain OS=Macaca mulatta GN=CRYAA PE=1 SV=2//4.76494e-111
XM_007969428.1	0.94367	6.34E-12	1.34E-10	sp|Q96E09|F122A_HUMAN Protein FAM122A OS=Homo sapiens GN=FAM122A PE=1 SV=1//3.31632e-148
XM_007969442.1	0.43511	9.99E-05	0.000888	sp|Q7Z6K3|PTAR1_HUMAN Protein prenyltransferase alpha subunit repeat-containing protein 1 OS=Homo sapiens GN=PTAR1 PE=1 SV=2//0
XM_007969452.1	1.8352	2.09E-48	2.84E-46	sp|P57059|SIK1_HUMAN Serine/threonine-protein kinase SIK1 OS=Homo sapiens GN=SIK1 PE=1 SV=2//0
XM_007969455.1	0.99	5.61E-06	6.07E-05	sp|Q8IY18|SMC5_HUMAN Structural maintenance of chromosomes protein 5 OS=Homo sapiens GN=SMC5 PE=1 SV=2//0
XM_007969466.1	2.8596	2.52E-58	4.92E-56	sp|Q14684|RRP1B_HUMAN Ribosomal RNA processing protein 1 homolog B OS=Homo sapiens GN=RRP1B PE=1 SV=3//0
XM_007969484.1	0.61799	2.73E-09	4.45E-08	sp|P79288|KLF9_PIG Krueppel-like factor 9 OS=Sus scrofa GN=KLF9 PE=2 SV=3//3.67805e-147
XM_007969485.1	-0.44654	2.36E-09	3.88E-08	sp|Q9UHN6|TMEM2_HUMAN Transmembrane protein 2 OS=Homo sapiens GN=TMEM2 PE=1 SV=1//0
XM_007969487.1	0.86924	8.45E-10	1.45E-08	sp|Q5VST6|AB17B_HUMAN Alpha/beta hydrolase domain-containing protein 17B OS=Homo sapiens GN=ABHD17B PE=2 SV=1//0
XM_007969496.1	1.1898	7.65E-55	1.33E-52	sp|O76080|ZFAN5_HUMAN AN1-type zinc finger protein 5 OS=Homo sapiens GN=ZFAND5 PE=1 SV=1//8.19673e-119
XM_007969498.1	3.3163	1.47E-11	3.04E-10	sp|Q6JZS3|CP1A1_ORYLA Cytochrome P450 1A1 OS=Oryzias latipes GN=cyp1a1 PE=3 SV=1//1.62499e-166
XM_007969503.1	0.72584	3.90E-17	1.27E-15	sp|P04083|ANXA1_HUMAN Annexin A1 OS=Homo sapiens GN=ANXA1 PE=1 SV=2//0
XM_007969505.1	2.1062	3.69E-06	4.10E-05	sp|O75031|HSF2B_HUMAN Heat shock factor 2-binding protein OS=Homo sapiens GN=HSF2BP PE=1 SV=1//0
XM_007969525.1	-0.45171	0.001793	0.01247	sp|Q92882|OSTF1_HUMAN Osteoclast-stimulating factor 1 OS=Homo sapiens GN=OSTF1 PE=1 SV=2//1.99736e-140
XM_007969536.1	1.3861	1.40E-39	1.37E-37	sp|Q969G6|RIFK_HUMAN Riboflavin kinase OS=Homo sapiens GN=RFK PE=1 SV=2//5.50339e-103
XM_007969549.1	0.94656	2.09E-31	1.47E-29	sp|Q2PKF4|GNAQ_PIG Guanine nucleotide-binding protein G(q) subunit alpha OS=Sus scrofa GN=GNAQ PE=2 SV=3//0
XM_007969560.1	0.88355	3.86E-21	1.62E-19	sp|Q9Y617|SERC_HUMAN Phosphoserine aminotransferase OS=Homo sapiens GN=PSAT1 PE=1 SV=2//0
XM_007969562.1	-0.8523	2.15E-06	2.48E-05	sp|Q862Z5|CYTB_MACFU Cystatin-B OS=Macaca fuscata fuscata GN=CSTB PE=3 SV=1//2.68964e-63
XM_007969575.1	0.99258	4.39E-07	5.51E-06	sp|Q04724|TLE1_HUMAN Transducin-like enhancer protein 1 OS=Homo sapiens GN=TLE1 PE=1 SV=2//0
XM_007969583.1	1.8679	1.86E-13	4.54E-12	sp|Q8IZ41|RASEF_HUMAN Ras and EF-hand domain-containing protein OS=Homo sapiens GN=RASEF PE=1 SV=1//0
XM_007969593.1	-1.607	0.006804	0.041132	sp|Q5T6J7|GNTK_HUMAN Probable gluconokinase OS=Homo sapiens GN=IDNK PE=1 SV=1//3.51585e-113
XM_007969613.1	-0.21215	0.002907	0.019176	sp|Q5R5H8|HNRPK_PONAB Heterogeneous nuclear ribonucleoprotein K OS=Pongo abelii GN=HNRNPK PE=2 SV=1//0
XM_007969666.1	1.2328	1.02E-18	3.65E-17	sp|P54826|GAS1_HUMAN Growth arrest-specific protein 1 OS=Homo sapiens GN=GAS1 PE=2 SV=2//1.47819e-116
XM_007969698.1	1.2674	6.06E-06	6.51E-05	sp|Q99500|S1PR3_HUMAN Sphingosine 1-phosphate receptor 3 OS=Homo sapiens GN=S1PR3 PE=1 SV=2//0
XM_007969731.1	0.83413	1.43E-14	3.80E-13	sp|Q01974|ROR2_HUMAN Tyrosine-protein kinase transmembrane receptor ROR2 OS=Homo sapiens GN=ROR2 PE=1 SV=2//0
XM_007969734.1	0.61791	1.46E-13	3.59E-12	sp|Q60HD1|SPTC1_MACFA Serine palmitoyltransferase 1 OS=Macaca fascicularis GN=SPTLC1 PE=2 SV=1//0

XM_007969741.1	1.836	6.34E-52	9.84E-50	sp|Q9H8X2|IPPK_HUMAN Inositol-pentakisphosphate 2-kinase OS=Homo sapiens GN=IPPK PE=1 SV=1//0
XM_007969748.1	-0.73504	0.000211	0.001772	sp|O75144|ICOSL_HUMAN ICOS ligand OS=Homo sapiens GN=ICOSLG PE=1 SV=2//3.91242e-163
XM_007969795.1	0.50911	1.56E-06	1.82E-05	sp|Q5T036|F120S_HUMAN Putative FAM120A opposite strand protein OS=Homo sapiens GN=FAM120AOS PE=5 SV=1//2.4357e-31
XM_007969799.1	0.51069	0.000868	0.006484	sp|O75151|PHF2_HUMAN Lysine-specific demethylase PHF2 OS=Homo sapiens GN=PHF2 PE=1 SV=4//7.3962e-35
XM_007969812.1	0.29845	0.002873	0.018984	sp|Q9Y520|PRC2C_HUMAN Protein PRRC2C OS=Homo sapiens GN=PRRC2C PE=1 SV=4//0
XM_007969814.1	1.2762	6.58E-05	0.000605	sp|Q5VZR2|NTM2G_HUMAN NUT family member 2G OS=Homo sapiens GN=NUTM2G PE=3 SV=2//0
XM_007969821.1	-0.76599	3.22E-16	9.59E-15	sp|Q5SR56|HIAL1_HUMAN Hippocampus abundant transcript-like protein 1 OS=Homo sapiens GN=HIATL1 PE=2 SV=3//0
XM_007969835.1	-0.37158	0.008372	0.049334	sp|Q9HOH9|C4F30_HUMAN Putative cytochrome P450 family member 4F30 OS=Homo sapiens GN=CYP4F30P PE=5 SV=1//7.87401e-49
XM_007969848.1	-0.8318	2.38E-25	1.27E-23	sp|P17858|PFKAL_HUMAN ATP-dependent 6-phosphofructokinase, liver type OS=Homo sapiens GN=PFKL PE=1 SV=6//0
XM_007969894.1	2.8857	########	########	sp|Q7RTV3|ZN367_HUMAN Zinc finger protein 367 OS=Homo sapiens GN=ZNF367 PE=1 SV=1//6.53365e-170
XM_007969902.1	0.63233	1.69E-06	1.97E-05	sp|Q5T890|ER6L2_HUMAN DNA excision repair protein ERCC-6-like 2 OS=Homo sapiens GN=ERCC6L2 PE=1 SV=2//0
XM_007969921.1	-1.0167	3.73E-06	4.14E-05	sp|Q8N6M6|AMPO_HUMAN Aminopeptidase O OS=Homo sapiens GN=AOPEP PE=1 SV=2//3.89139e-47
XM_007969924.1	-1.0683	0.001068	0.007833	sp|Q8N6M6|AMPO_HUMAN Aminopeptidase O OS=Homo sapiens GN=AOPEP PE=1 SV=2//0
XM_007969944.1	1.1868	1.37E-28	8.69E-27	sp|Q5NVD0|PRP4_PONAB U4/U6 small nuclear ribonucleoprotein Prp4 OS=Pongo abelii GN=PRPF4 PE=2 SV=1//0
XM_007969945.1	-0.44575	0.002457	0.016495	-//-
XM_007969955.1	-0.6668	5.67E-05	0.000525	sp|Q5ZME8|SMU1_CHICK WD40 repeat-containing protein SMU1 OS=Gallus gallus GN=SMU1 PE=2 SV=1//2.91822e-58
XM_007969957.1	-1.0052	2.17E-05	0.000214	-//-
XM_007969958.1	1.8031	2.94E-06	3.32E-05	-//-
XM_007969969.1	-2.6825	0.002665	0.01773	sp|Q6ZUB1|S31E1_HUMAN Spermatogenesis-associated protein 31E1 OS=Homo sapiens GN=SPATA31E1 PE=2 SV=2//0
XM_007969976.1	1.1654	2.01E-15	5.70E-14	sp|Q9BZ76|CNTP3_HUMAN Contactin-associated protein-like 3 OS=Homo sapiens GN=CNTNAP3 PE=2 SV=3//0
XM_007969980.1	0.84638	1.89E-12	4.20E-11	sp|O00237|RN103_HUMAN E3 ubiquitin-protein ligase RNF103 OS=Homo sapiens GN=RNF103 PE=1 SV=1//0
XM_007969990.1	-0.70424	0.003113	0.020437	sp|Q96EY7|PTCD3_HUMAN Pentatricopeptide repeat domain-containing protein 3, mitochondrial OS=Homo sapiens GN=PTCD3 PE=1 SV=3//0
XM_007969992.1	0.38493	1.91E-05	0.000189	sp|O95602|RPA1_HUMAN DNA-directed RNA polymerase I subunit RPA1 OS=Homo sapiens GN=POLR1A PE=1 SV=2//0
XM_007970001.1	0.43641	0.004607	0.029129	sp|Q96SQ7|ATOH8_HUMAN Protein atonal homolog 8 OS=Homo sapiens GN=ATOH8 PE=2 SV=2//1.43354e-148
XM_007970019.1	-0.50549	0.002081	0.01424	sp|O95183|VAMP5_HUMAN Vesicle-associated membrane protein 5 OS=Homo sapiens GN=VAMP5 PE=1 SV=1//4.07092e-40
XM_007970020.1	-0.77556	2.63E-07	3.38E-06	sp|Q5REQ5|VAMP8_PONAB Vesicle-associated membrane protein 8 OS=Pongo abelii GN=VAMP8 PE=3 SV=1//2.46237e-47
XM_007970026.1	-0.64925	2.28E-15	6.43E-14	sp|Q5R5H1|METK2_PONAB S-adenosylmethionine synthase isoform type-2 OS=Pongo abelii GN=MAT2A PE=2 SV=1//0
XM_007970033.1	1.0515	1.25E-15	3.60E-14	sp|Q96FG2|ELMD3_HUMAN ELMO domain-containing protein 3 OS=Homo sapiens GN=ELMOD3 PE=1 SV=2//0
XM_007970038.1	-0.94764	0.000262	0.002166	sp|P63312|TYB10_RAT Thymosin beta-10 OS=Rattus norvegicus GN=Tmsb10 PE=2 SV=2//7.46476e-22
XM_007970051.1	-0.46397	5.85E-07	7.25E-06	sp|P53597|SUCA_HUMAN Succinyl-CoA ligase [ADP/GDP-forming] subunit alpha, mitochondrial OS=Homo sapiens GN=SUCLG1 PE=1 SV=4//1.06582e-164
XM_007970063.1	0.29444	0.001313	0.009419	sp|Q5RF84|UB2G2_PONAB Ubiquitin-conjugating enzyme E2 G2 OS=Pongo abelii GN=UBE2G2 PE=2 SV=1//5.24071e-111
XM_007970131.1	0.64431	0.000758	0.005741	sp|Q9BSM1|PCGF1_HUMAN Polycomb group RING finger protein 1 OS=Homo sapiens GN=PCGF1 PE=1 SV=2//7.33732e-177
XM_007970141.1	-0.41187	9.91E-05	0.000882	sp|Q13724|MOGS_HUMAN Mannosyl-oligosaccharide glucosidase OS=Homo sapiens GN=MOGS PE=1 SV=5//0
XM_007970145.1	-0.57836	2.80E-08	4.02E-07	sp|Q96G27|WBP1_HUMAN WW domain-binding protein 1 OS=Homo sapiens GN=WBP1 PE=1 SV=1//5.74574e-130
XM_007970185.1	1.8602	8.07E-26	4.40E-24	sp|P13995|MTDC_HUMAN Bifunctional methylenetetrahydrofolate dehydrogenase/cyclohydrolase, mitochondrial OS=Homo sapiens GN=MTHFD2 PE=1 SV=2//0
XM_007970198.1	1.5998	0.000211	0.001767	sp|P63269|ACTH_RAT Actin, gamma-enteric smooth muscle OS=Rattus norvegicus GN=Actg2 PE=2 SV=1//0

XM_007970204.1	0.89772	3.32E-19	1.24E-17	sp|O75319|DUS11_HUMAN RNA/RNP complex-1-interacting phosphatase OS=Homo sapiens GN=DUSP11 PE=1 SV=1//0
XM_007970229.1	0.34988	0.001136	0.00828	sp|Q6GMV2|SMYD5_HUMAN SET and MYND domain-containing protein 5 OS=Homo sapiens GN=SMYD5 PE=1 SV=2//0
XM_007970253.1	-0.39388	0.000141	0.001217	sp|P35270|SPRE_HUMAN Sepiapterin reductase OS=Homo sapiens GN=SPR PE=1 SV=1//9.23089e-148
XM_007970290.1	0.93343	1.32E-07	1.75E-06	sp|O00566|MPP10_HUMAN U3 small nucleolar ribonucleoprotein protein MPP10 OS=Homo sapiens GN=MPHOSPH10 PE=1 SV=2//0
XM_007970291.1	-0.5905	0.000838	0.006277	sp|Q9UJ70|NAGK_HUMAN N-acetyl-D-glucosamine kinase OS=Homo sapiens GN=NAGK PE=1 SV=4//0
XM_007970318.1	0.30734	0.006424	0.039151	sp|Q96C01|F136A_HUMAN Protein FAM136A OS=Homo sapiens GN=FAM136A PE=1 SV=1//5.22334e-88
XM_007970319.1	-0.77998	2.91E-18	1.02E-16	sp|Q95KC9|PCYOX_MACFA Prenylcysteine oxidase OS=Macaca fascicularis GN=PCYOX1 PE=2 SV=1//0
XM_007970343.1	1.5966	6.96E-30	4.63E-28	sp|Q05195|MAD1_HUMAN Max dimerization protein 1 OS=Homo sapiens GN=MXD1 PE=1 SV=1//3.95037e-102
XM_007970348.1	-0.93723	8.34E-08	1.13E-06	sp|P09525|ANXA4_HUMAN Annexin A4 OS=Homo sapiens GN=ANXA4 PE=1 SV=4//0
XM_007970412.1	1.3652	4.05E-36	3.53E-34	sp|Q9NRX1|PNO1_HUMAN RNA-binding protein PNO1 OS=Homo sapiens GN=PNO1 PE=1 SV=1//1.10674e-167
XM_007970415.1	0.75264	7.87E-05	0.000714	sp|Q96MX6|WDR92_HUMAN WD repeat-containing protein 92 OS=Homo sapiens GN=WDR92 PE=1 SV=1//0
XM_007970431.1	-0.20051	0.007503	0.044644	sp|P61161|ARP2_MOUSE Actin-related protein 2 OS=Mus musculus GN=Actr2 PE=1 SV=1//0
XM_007970438.1	2.1253	2.88E-74	8.19E-72	sp|P43007|SATT_HUMAN Neutral amino acid transporter A OS=Homo sapiens GN=SLC1A4 PE=1 SV=1//0
XM_007970450.1	1.45	4.76E-54	7.96E-52	sp|Q96FA3|PELI1_HUMAN E3 ubiquitin-protein ligase pellino homolog 1 OS=Homo sapiens GN=PELI1 PE=1 SV=2//0
XM_007970472.1	-0.45793	9.98E-08	1.35E-06	sp|P40925|MDHC_HUMAN Malate dehydrogenase, cytoplasmic OS=Homo sapiens GN=MDH1 PE=1 SV=4//0
XM_007970496.1	-1.2409	3.51E-11	7.00E-10	-//-
XM_007970499.1	1.1791	6.84E-08	9.39E-07	sp|O14980|XPO1_HUMAN Exportin-1 OS=Homo sapiens GN=XPO1 PE=1 SV=1//0
XM_007970524.1	0.93392	0.001239	0.008946	sp|Q3MIT2|PUS10_HUMAN Putative tRNA pseudouridine synthase Pus10 OS=Homo sapiens GN=PUS10 PE=1 SV=1//0
XM_007970527.1	0.37467	0.000379	0.003053	sp|Q92968|PEX13_HUMAN Peroxisomal membrane protein PEX13 OS=Homo sapiens GN=PEX13 PE=1 SV=2//0
XM_007970528.1	1.4861	1.27E-08	1.91E-07	sp|Q04864|REL_HUMAN Proto-oncogene c-Rel OS=Homo sapiens GN=REL PE=1 SV=1//0
XM_007970606.1	-0.54946	7.19E-08	9.85E-07	sp|P62979|RS27A_HUMAN Ubiquitin-40S ribosomal protein S27a OS=Homo sapiens GN=RPS27A PE=1 SV=2//2.87543e-83
XM_007970631.1	0.52041	1.52E-06	1.78E-05	sp|Q01082|SPTB2_HUMAN Spectrin beta chain, non-erythrocytic 1 OS=Homo sapiens GN=SPTBN1 PE=1 SV=2//0
XM_007970653.1	-1.3185	7.09E-14	1.78E-12	sp|O95800|GPR75_HUMAN Probable G-protein coupled receptor 75 OS=Homo sapiens GN=GPR75 PE=1 SV=1//0
XM_007970689.1	-0.8379	7.68E-13	1.76E-11	sp|P12110|CO6A2_HUMAN Collagen alpha-2(VI) chain OS=Homo sapiens GN=COL6A2 PE=1 SV=4//0
XM_007970695.1	-0.84863	1.13E-05	0.000116	sp|P12110|CO6A2_HUMAN Collagen alpha-2(VI) chain OS=Homo sapiens GN=COL6A2 PE=1 SV=4//1.8553e-152
XM_007970713.1	-0.81349	4.63E-10	8.20E-09	sp|Q1WER1|EPCAM_MACMU Epithelial cell adhesion molecule OS=Macaca mulatta GN=TACSTD1 PE=2 SV=1//0
XM_007970714.1	0.30946	3.41E-05	0.000326	sp|P62155|CALM_XENLA Calmodulin OS=Xenopus laevis GN=calm1 PE=1 SV=2//8.23041e-102
XM_007970736.1	3.1902	1.91E-08	2.80E-07	sp|O75159|SOCS5_HUMAN Suppressor of cytokine signaling 5 OS=Homo sapiens GN=SOCS5 PE=1 SV=1//0
XM_007970745.1	1.1373	0.005589	0.034647	sp|Q99814|EPAS1_HUMAN Endothelial PAS domain-containing protein 1 OS=Homo sapiens GN=EPAS1 PE=1 SV=3//0
XM_007970759.1	3.3625	7.27E-71	1.89E-68	sp|Q02156|KPCE_HUMAN Protein kinase C epsilon type OS=Homo sapiens GN=PRKCE PE=1 SV=1//0
XM_007970760.1	0.5155	2.36E-08	3.43E-07	sp|Q5RDI0|SRBD1_PONAB S1 RNA-binding domain-containing protein 1 OS=Pongo abelii GN=SRBD1 PE=2 SV=1//0
XM_007970782.1	1.112	1.68E-18	5.93E-17	sp|O75688|PPM1B_HUMAN Protein phosphatase 1B OS=Homo sapiens GN=PPM1B PE=1 SV=1//0
XM_007970783.1	0.84685	2.07E-08	3.03E-07	sp|O60318|GANP_HUMAN Germinal-center associated nuclear protein OS=Homo sapiens GN=MCM3AP PE=1 SV=2//2.05536e-70
XM_007970796.1	0.55708	5.19E-13	1.22E-11	sp|Q8IVE3|PKHH2_HUMAN Pleckstrin homology domain-containing family H member 2 OS=Homo sapiens GN=PLEKHH2 PE=1 SV=2//0
XM_007970809.1	-3.3602	0.005849	0.036016	sp|O60318|GANP_HUMAN Germinal-center associated nuclear protein OS=Homo sapiens GN=MCM3AP PE=1 SV=2//0
XM_007970812.1	-1.1384	0.007163	0.04297	sp|Q8TDS5|OXER1_HUMAN Oxoeicosanoid receptor 1 OS=Homo sapiens GN=OXER1 PE=2 SV=1//0
XM_007970831.1	0.34702	0.001134	0.00827	sp|Q504Y2|PKDCC_HUMAN Extracellular tyrosine-protein kinase PKDCC OS=Homo

				sapiens GN=PKDCC PE=2 SV=2//0
XM_007970859.1	1.1822	1.69E-11	3.45E-10	sp|Q9BTF0|THUM2_HUMAN THUMP domain-containing protein 2 OS=Homo sapiens GN=THUMPD2 PE=2 SV=2//0
XM_007970929.1	1.762	4.22E-05	0.000399	sp|Q9UKI2|BORG2_HUMAN Cdc42 effector protein 3 OS=Homo sapiens GN=CDC42EP3 PE=1 SV=1//1.11192e-118
XM_007970942.1	0.5249	9.73E-09	1.48E-07	sp|Q03701|CEBPZ_HUMAN CCAAT/enhancer-binding protein zeta OS=Homo sapiens GN=CEBPZ PE=1 SV=3//0
XM_007970945.1	0.87537	1.76E-14	4.66E-13	sp|P19525|E2AK2_HUMAN Interferon-induced, double-stranded RNA-activated protein kinase OS=Homo sapiens GN=EIF2AK2 PE=1 SV=2//0
XM_007970951.1	0.53713	0.006601	0.040084	sp|Q8N954|GPT11_HUMAN G patch domain-containing protein 11 OS=Homo sapiens GN=GPATCH11 PE=1 SV=3//5.27554e-154
XM_007970961.1	0.73745	1.16E-14	3.13E-13	sp|Q9NZV1|CRIM1_HUMAN Cysteine-rich motor neuron 1 protein OS=Homo sapiens GN=CRIM1 PE=1 SV=1//0
XM_007970975.1	0.61287	4.03E-05	0.000382	sp|Q6P3X3|TTC27_HUMAN Tetratricopeptide repeat protein 27 OS=Homo sapiens GN=TTC27 PE=1 SV=1//0
XM_007970994.1	1.6002	1.02E-19	3.93E-18	sp|Q8IV61|GRP3_HUMAN Ras guanyl-releasing protein 3 OS=Homo sapiens GN=RASGRP3 PE=1 SV=1//0
XM_007970996.1	0.38596	3.82E-05	0.000363	sp|Q5R679|FA98A_PONAB Protein FAM98A OS=Pongo abelii GN=FAM98A PE=2 SV=1//0
XM_007971026.1	0.38538	0.003127	0.020513	sp|Q4R6D9|MEMO1_MACFA Protein MEMO1 OS=Macaca fascicularis GN=MEMO1 PE=2 SV=1//0
XM_007971055.1	-2.597	1.09E-24	5.64E-23	sp|Q9UM73|ALK_HUMAN ALK tyrosine kinase receptor OS=Homo sapiens GN=ALK PE=1 SV=3//0
XM_007971056.1	-0.32771	0.004072	0.026088	sp|Q5RDU7|YPEL5_PONAB Protein yippee-like 5 OS=Pongo abelii GN=YPEL5 PE=2 SV=1//3.69467e-78
XM_007971062.1	-0.49466	0.002646	0.017638	sp|Q6ZUX3|F179A_HUMAN Protein FAM179A OS=Homo sapiens GN=FAM179A PE=2 SV=2//0
XM_007971063.1	1.4036	1.50E-57	2.80E-55	sp|Q15061|WDR43_HUMAN WD repeat-containing protein 43 OS=Homo sapiens GN=WDR43 PE=1 SV=3//0
XM_007971068.1	0.21791	0.002878	0.019007	sp|P62142|PP1B_RAT Serine/threonine-protein phosphatase PP1-beta catalytic subunit OS=Rattus norvegicus GN=Ppp1cb PE=1 SV=3//0
XM_007971077.1	2.5573	2.95E-82	1.04E-79	sp|P15408|FOSL2_HUMAN Fos-related antigen 2 OS=Homo sapiens GN=FOSL2 PE=1 SV=1//3.00885e-162
XM_007971121.1	-0.858	3.62E-09	5.84E-08	sp|Q9H6D8|FNDC4_HUMAN Fibronectin type III domain-containing protein 4 OS=Homo sapiens GN=FNDC4 PE=2 SV=1//2.43551e-150
XM_007971125.1	0.27437	0.00066	0.00507	sp|O15355|PPM1G_HUMAN Protein phosphatase 1G OS=Homo sapiens GN=PPM1G PE=1 SV=1//0
XM_007971138.1	0.27836	0.002254	0.015321	sp|Q15036|SNX17_HUMAN Sorting nexin-17 OS=Homo sapiens GN=SNX17 PE=1 SV=1//0
XM_007971162.1	0.86486	1.07E-15	3.11E-14	sp|P27708|PYR1_HUMAN CAD protein OS=Homo sapiens GN=CAD PE=1 SV=3//0
XM_007971186.1	-1.2729	5.89E-05	0.000545	sp|B0BLS0|OST4_RAT Dolichyl-diphosphooligosaccharide--protein glycosyltransferase subunit 4 OS=Rattus norvegicus GN=Ost4 PE=3 SV=1//1.30606e-17
XM_007971254.1	0.4272	2.94E-08	4.22E-07	sp|Q5R5U1|RAB10_PONAB Ras-related protein Rab-10 OS=Pongo abelii GN=RAB10 PE=2 SV=1//1.79965e-133
XM_007971255.1	0.31502	0.000108	0.000955	sp|O14782|KIF3C_HUMAN Kinesin-like protein KIF3C OS=Homo sapiens GN=KIF3C PE=1 SV=3//0
XM_007971271.1	-0.9893	1.41E-05	0.000143	sp|Q9Y2G0|EFR3B_HUMAN Protein EFR3 homolog B OS=Homo sapiens GN=EFR3B PE=1 SV=2//0
XM_007971305.1	-0.34945	0.002058	0.014099	sp|P0C875|F228B_HUMAN Protein FAM228B OS=Homo sapiens GN=FAM228B PE=2 SV=1//2.00639e-169
XM_007971320.1	-0.75942	6.58E-11	1.27E-09	sp|P59708|SF3B6_MOUSE Splicing factor 3B subunit 6 OS=Mus musculus GN=Sf3b6 PE=1 SV=1//4.65163e-75
XM_007971321.1	-0.60193	0.000495	0.003895	sp|Q53FA7|QORX_HUMAN Quinone oxidoreductase PIG3 OS=Homo sapiens GN=TP53I3 PE=1 SV=2//0
XM_007971326.1	-2.0637	1.53E-06	1.80E-05	sp|P55345|ANM2_HUMAN Protein arginine N-methyltransferase 2 OS=Homo sapiens GN=PRMT2 PE=1 SV=1//7.944e-136
XM_007971328.1	0.76149	0.000761	0.005761	sp|P68543|UBX2A_HUMAN UBX domain-containing protein 2A OS=Homo sapiens GN=UBXN2A PE=2 SV=1//7.68367e-153
XM_007971334.1	-2.6162	0.000123	0.001074	sp|P55345|ANM2_HUMAN Protein arginine N-methyltransferase 2 OS=Homo sapiens GN=PRMT2 PE=1 SV=1//4.49522e-137
XM_007971340.1	-1.5914	1.70E-05	0.00017	sp|P55345|ANM2_HUMAN Protein arginine N-methyltransferase 2 OS=Homo sapiens GN=PRMT2 PE=1 SV=1//4.46129e-30
XM_007971360.1	3.1858	0	0	sp|P62747|RHOB_RAT Rho-related GTP-binding protein RhoB OS=Rattus norvegicus GN=Rhob PE=1 SV=1//2.34133e-135
XM_007971370.1	-0.7156	1.99E-13	4.84E-12	sp|P18827|SDC1_HUMAN Syndecan-1 OS=Homo sapiens GN=SDC1 PE=1 SV=3//8.29271e-141
XM_007971371.1	-0.45635	1.02E-08	1.55E-07	sp|Q4R4I5|LAP4A_MACFA Lysosomal-associated transmembrane protein 4A OS=Macaca fascicularis GN=LAPTM4A PE=2 SV=1//4.25911e-134
XM_007971381.1	0.68402	1.20E-05	0.000122	sp|Q9HBH5|RDH14_HUMAN Retinol dehydrogenase 14 OS=Homo sapiens GN=RDH14

				PE=1 SV=1//0
XM_007971431.1	-0.91251	3.31E-10	5.96E-09	sp|Q5R669|TRIB2_PONAB Tribbles homolog 2 OS=Pongo abelii GN=TRIB2 PE=2 SV=1//0
XM_007971447.1	1.8994	8.02E-11	1.54E-09	sp|O75461|E2F6_HUMAN Transcription factor E2F6 OS=Homo sapiens GN=E2F6 PE=1 SV=1//7.25383e-176
XM_007971448.1	1.6473	0.006064	0.03714	sp|O75461|E2F6_HUMAN Transcription factor E2F6 OS=Homo sapiens GN=E2F6 PE=1 SV=1//3.40612e-176
XM_007971472.1	0.69531	1.12E-16	3.49E-15	sp|O75116|ROCK2_HUMAN Rho-associated protein kinase 2 OS=Homo sapiens GN=ROCK2 PE=1 SV=4//0
XM_007971497.1	1.1682	2.72E-47	3.56E-45	sp|P11926|DCOR_HUMAN Ornithine decarboxylase OS=Homo sapiens GN=ODC1 PE=1 SV=2//0
XM_007971516.1	-0.60259	2.74E-17	9.04E-16	sp|Q5RFJ2|1433T_PONAB 14-3-3 protein theta OS=Pongo abelii GN=YWHAQ PE=2 SV=2//9.36134e-161
XM_007971537.1	2.2807	3.17E-30	2.16E-28	sp|P28663|SNAB_MOUSE Beta-soluble NSF attachment protein OS=Mus musculus GN=Napb PE=1 SV=2//3.09162e-93
XM_007971563.1	1.157	2.23E-15	6.29E-14	sp|Q5RCH7|ID2_PONAB DNA-binding protein inhibitor ID-2 OS=Pongo abelii GN=ID2 PE=2 SV=1//2.0151e-88
XM_007971596.1	-0.48792	0.003963	0.025447	sp|Q9NUR3|TM74B_HUMAN Transmembrane protein 74B OS=Homo sapiens GN=TMEM74B PE=2 SV=1//1.18463e-134
XM_007971606.1	-0.41224	0.001439	0.010261	sp|P62083|RS7_RAT 40S ribosomal protein S7 OS=Rattus norvegicus GN=Rps7 PE=1 SV=1//7.42642e-136
XM_007971607.1	0.45259	2.20E-05	0.000216	sp|O60930|RNH1_HUMAN Ribonuclease H1 OS=Homo sapiens GN=RNASEH1 PE=1 SV=2//0
XM_007971611.1	-0.43836	6.95E-06	7.37E-05	sp|Q9CQE7|ERGI3_MOUSE Endoplasmic reticulum-Golgi intermediate compartment protein 3 OS=Mus musculus GN=Ergic3 PE=2 SV=1//8.99835e-121
XM_007971619.1	1.0689	1.28E-34	1.05E-32	sp|Q5RC80|RBM39_PONAB RNA-binding protein 39 OS=Pongo abelii GN=RBM39 PE=2 SV=1//0
XM_007971623.1	0.85883	5.20E-28	3.20E-26	sp|Q9BVI0|PHF20_HUMAN PHD finger protein 20 OS=Homo sapiens GN=PHF20 PE=1 SV=2//0
XM_007971653.1	0.67428	9.81E-08	1.33E-06	sp|Q9H410|DSN1_HUMAN Kinetochore-associated protein DSN1 homolog OS=Homo sapiens GN=DSN1 PE=1 SV=2//0
XM_007971674.1	1.3158	2.99E-27	1.78E-25	sp|O60287|NPA1P_HUMAN Nucleolar pre-ribosomal-associated protein 1 OS=Homo sapiens GN=URB1 PE=1 SV=4//0
XM_007971687.1	1.0578	1.89E-05	0.000187	-//-
XM_007971691.1	0.74082	8.49E-18	2.89E-16	sp|Q76L83|ASXL2_HUMAN Putative Polycomb group protein ASXL2 OS=Homo sapiens GN=ASXL2 PE=1 SV=1//0
XM_007971694.1	0.34877	0.003	0.019764	-//-
XM_007971704.1	1.0951	5.72E-10	1.00E-08	sp|Q96DW6|S2538_HUMAN Solute carrier family 25 member 38 OS=Homo sapiens GN=SLC25A38 PE=1 SV=1//0
XM_007971741.1	-0.76677	0.008159	0.048153	sp|Q9Y2C4|EXOG_HUMAN Nuclease EXOG, mitochondrial OS=Homo sapiens GN=EXOG PE=1 SV=2//0
XM_007971751.1	0.63491	2.27E-08	3.30E-07	sp|B3Y681|MYD88_PONPY Myeloid differentiation primary response protein MyD88 OS=Pongo pygmaeus GN=MYD88 PE=2 SV=1//0
XM_007971802.1	-0.34326	0.001651	0.011597	sp|Q7L775|EPMIP_HUMAN EPM2A-interacting protein 1 OS=Homo sapiens GN=EPM2AIP1 PE=1 SV=1//0
XM_007971822.1	0.3915	2.92E-06	3.30E-05	sp|Q5RAJ6|DJB11_PONAB DnaJ homolog subfamily B member 11 OS=Pongo abelii GN=DNAJB11 PE=2 SV=1//0
XM_007971836.1	1.7406	2.52E-15	7.07E-14	sp|P41161|ETV5_HUMAN ETS translocation variant 5 OS=Homo sapiens GN=ETV5 PE=1 SV=1//0
XM_007971851.1	0.95572	1.03E-07	1.39E-06	sp|Q96HV5|TM41A_HUMAN Transmembrane protein 41A OS=Homo sapiens GN=TMEM41A PE=1 SV=1//1.02912e-168
XM_007971862.1	0.51458	5.39E-06	5.85E-05	sp|Q5R5M8|ECHP_PONAB Peroxisomal bifunctional enzyme OS=Pongo abelii GN=EHHADH PE=2 SV=1//0
XM_007971873.1	-0.73419	2.39E-05	0.000234	sp|P54753|EPHB3_HUMAN Ephrin type-B receptor 3 OS=Homo sapiens GN=EPHB3 PE=1 SV=2//0
XM_007971905.1	-0.47389	1.04E-10	1.99E-09	sp|Q13200|PSMD2_HUMAN 26S proteasome non-ATPase regulatory subunit 2 OS=Homo sapiens GN=PSMD2 PE=1 SV=3//0
XM_007971913.1	-1.0701	7.77E-10	1.34E-08	sp|P59089|CU086_HUMAN Putative uncharacterized protein encoded by LINC00205 OS=Homo sapiens GN=LINC00205 PE=5 SV=1//1.06207e-74
XM_007971929.1	0.38684	0.000245	0.002034	sp|Q9NUQ8|ABCF3_HUMAN ATP-binding cassette sub-family F member 3 OS=Homo sapiens GN=ABCF3 PE=1 SV=2//0
XM_007971937.1	-0.66952	1.00E-21	4.34E-20	sp|P39060|COIA1_HUMAN Collagen alpha-1(XVIII) chain OS=Homo sapiens GN=COL18A1 PE=1 SV=5//0
XM_007971961.1	-0.90452	1.99E-30	1.36E-28	sp|P12109|CO6A1_HUMAN Collagen alpha-1(VI) chain OS=Homo sapiens GN=COL6A1 PE=1 SV=3//1.41523e-07
XM_007971962.1	0.83385	2.52E-05	0.000246	sp|Q9BYG0|B3GN5_HUMAN Lactosylceramide 1,3-N-acetyl-beta-D-glucosaminyltransferase OS=Homo sapiens GN=B3GNT5 PE=1 SV=1//0
XM_007971975.1	-1.8003	0.003243	0.021218	sp|Q6TFL4|KLH24_HUMAN Kelch-like protein 24 OS=Homo sapiens GN=KLHL24 PE=2 SV=1//0
XM_007971976.1	0.24912	0.001956	0.013442	sp|Q9ULM3|YETS2_HUMAN YEATS domain-containing protein 2 OS=Homo sapiens

				GN=YEATS2 PE=1 SV=2//0
XM_007971991.1	-0.92517	2.10E-06	2.42E-05	sp|Q6PCB8|EMB_HUMAN Embigin OS=Homo sapiens GN=EMB PE=1 SV=1//0
XM_007972005.1	1.5235	0.000282	0.002319	sp|P48431|SOX2_HUMAN Transcription factor SOX-2 OS=Homo sapiens GN=SOX2 PE=1 SV=1//1.83781e-170
XM_007972009.1	-1.5583	1.02E-05	0.000105	sp|Q9UFE4|CCD39_HUMAN Coiled-coil domain-containing protein 39 OS=Homo sapiens GN=CCDC39 PE=2 SV=3//0
XM_007972018.1	0.41975	7.36E-05	0.000672	sp|Q92995|UBP13_HUMAN Ubiquitin carboxyl-terminal hydrolase 13 OS=Homo sapiens GN=USP13 PE=1 SV=2//0
XM_007972091.1	0.58309	3.43E-05	0.000328	sp|Q6PIU2|NCEH1_HUMAN Neutral cholesterol ester hydrolase 1 OS=Homo sapiens GN=NCEH1 PE=1 SV=3//0
XM_007972124.1	0.2948	0.00122	0.008823	sp|Q8NDX5|PHC3_HUMAN Polyhomeotic-like protein 3 OS=Homo sapiens GN=PHC3 PE=1 SV=1//0
XM_007972137.1	1.2703	3.61E-51	5.56E-49	sp|Q9BRX2|PELO_HUMAN Protein pelota homolog OS=Homo sapiens GN=PELO PE=1 SV=2//0
XM_007972153.1	1.5987	0.001118	0.008175	sp|Q9NPC7|MYNN_HUMAN Myoneurin OS=Homo sapiens GN=MYNN PE=1 SV=1//0
XM_007972171.1	-1.1594	1.69E-08	2.50E-07	sp|O00461|GOLI4_HUMAN Golgi integral membrane protein 4 OS=Homo sapiens GN=GOLIM4 PE=1 SV=1//0
XM_007972202.1	0.57581	0.001661	0.01165	sp|O75683|SURF6_HUMAN Surfeit locus protein 6 OS=Homo sapiens GN=SURF6 PE=1 SV=3//4.0834e-128
XM_007972213.1	1.4144	2.99E-14	7.71E-13	sp|Q9UID6|ZN639_HUMAN Zinc finger protein 639 OS=Homo sapiens GN=ZNF639 PE=1 SV=1//0
XM_007972214.1	-0.39444	0.007147	0.04291	sp|Q4R979|RBM4_MACFA RNA-binding protein 4 OS=Macaca fascicularis GN=RBM4 PE=2 SV=1//1.04838e-10
XM_007972215.1	1.4105	0.003815	0.024601	-//-
XM_007972216.1	0.40264	8.52E-05	0.000769	sp|Q5R4K9|KPCI_PONAB Protein kinase C iota type OS=Pongo abelii GN=PRKCI PE=2 SV=2//0
XM_007972221.1	0.45698	0.00424	0.027004	sp|Q9BTT6|LRRC1_HUMAN Leucine-rich repeat-containing protein 1 OS=Homo sapiens GN=LRRC1 PE=1 SV=1//0
XM_007972240.1	0.54855	0.000435	0.003457	sp|Q9UPZ9|ICK_HUMAN Serine/threonine-protein kinase ICK OS=Homo sapiens GN=ICK PE=1 SV=1//0
XM_007972250.1	-0.63283	8.33E-05	0.000753	sp|P0CB96|NDUS4_PONPY NADH dehydrogenase [ubiquinone] iron-sulfur protein 4, mitochondrial OS=Pongo pygmaeus GN=NDUFS4 PE=2 SV=1//3.31282e-100
XM_007972274.1	-0.69799	1.16E-07	1.55E-06	sp|P08F94|PKHD1_HUMAN Fibrocystin OS=Homo sapiens GN=PKHD1 PE=1 SV=1//0
XM_007972310.1	2.1942	1.65E-79	5.64E-77	sp|Q91ZR2|SNX18_MOUSE Sorting nexin-18 OS=Mus musculus GN=Snx18 PE=1 SV=1//0
XM_007972324.1	0.37572	0.001961	0.013471	sp|Q5T601|AGRF1_HUMAN Adhesion G-protein coupled receptor F1 OS=Homo sapiens GN=ADGRF1 PE=1 SV=2//0
XM_007972363.1	0.81191	0.000606	0.004681	sp|Q13950|RUNX2_HUMAN Runt-related transcription factor 2 OS=Homo sapiens GN=RUNX2 PE=1 SV=2//0
XM_007972367.1	1.4405	1.65E-09	2.75E-08	-//-
XM_007972376.1	0.41174	9.89E-07	1.20E-05	sp|Q99459|CDC5L_HUMAN Cell division cycle 5-like protein OS=Homo sapiens GN=CDC5L PE=1 SV=2//0
XM_007972394.1	1.5535	6.79E-16	1.98E-14	sp|O00221|IKBE_HUMAN NF-kappa-B inhibitor epsilon OS=Homo sapiens GN=NFKBIE PE=1 SV=3//0
XM_007972436.1	0.50178	0.000461	0.003654	sp|Q15013|MD2BP_HUMAN MAD2L1-binding protein OS=Homo sapiens GN=MAD2L1BP PE=1 SV=1//0
XM_007972478.1	0.80776	4.33E-09	6.91E-08	sp|O15160|RPAC1_HUMAN DNA-directed RNA polymerases I and III subunit RPAC1 OS=Homo sapiens GN=POLR1C PE=1 SV=1//0
XM_007972487.1	0.2677	0.004044	0.025941	sp|Q5VUA4|ZN318_HUMAN Zinc finger protein 318 OS=Homo sapiens GN=ZNF318 PE=1 SV=2//0
XM_007972496.1	0.59786	6.73E-09	1.04E-07	sp|P11831|SRF_HUMAN Serum response factor OS=Homo sapiens GN=SRF PE=1 SV=1//0
XM_007972522.1	4.6475	########	########	sp|Q96EU6|RRP36_HUMAN Ribosomal RNA processing protein 36 homolog OS=Homo sapiens GN=RRP36 PE=1 SV=1//1.89929e-112
XM_007972523.1	-0.48989	1.44E-08	2.14E-07	sp|Q14738|2A5D_HUMAN Serine/threonine-protein phosphatase 2A 56 kDa regulatory subunit delta isoform OS=Homo sapiens GN=PPP2R5D PE=1 SV=1//0
XM_007972536.1	-0.69375	4.58E-10	8.12E-09	sp|Q9BT09|CNPY3_HUMAN Protein canopy homolog 3 OS=Homo sapiens GN=CNPY3 PE=1 SV=1//5.56997e-155
XM_007972538.1	0.60446	6.27E-07	7.76E-06	sp|Q6DKI1|RL7L_HUMAN 60S ribosomal protein L7-like 1 OS=Homo sapiens GN=RPL7L1 PE=1 SV=1//7.26649e-152
XM_007972540.1	1.5371	1.07E-11	2.22E-10	sp|D6RGH6|MCIN_HUMAN Multicilin OS=Homo sapiens GN=MCIDAS PE=1 SV=1//0
XM_007972590.1	1.7687	1.37E-47	1.82E-45	sp|Q13895|BYST_HUMAN Bystin OS=Homo sapiens GN=BYSL PE=1 SV=3//0
XM_007972602.1	-0.38411	6.16E-05	0.000569	sp|Q96B49|TOM6_HUMAN Mitochondrial import receptor subunit TOM6 homolog OS=Homo sapiens GN=TOMM6 PE=1 SV=1//8.35011e-35
XM_007972644.1	-2.8038	0.000317	0.002591	sp|Q9Y235|ABEC2_HUMAN Probable C->U-editing enzyme APOBEC-2 OS=Homo sapiens GN=APOBEC2 PE=1 SV=1//2.14358e-129
XM_007972691.1	-1.5253	0.000177	0.001507	sp|Q3Y452|TDRG1_HUMAN Testis development-related protein 1 OS=Homo sapiens GN=TDRG1 PE=1 SV=1//1.17139e-45

XM_007972719.1	1.473	5.01E-26	2.78E-24	sp|O95279|KCNK5_HUMAN Potassium channel subfamily K member 5 OS=Homo sapiens GN=KCNK5 PE=1 SV=1//0
XM_007972730.1	-0.57773	4.48E-08	6.27E-07	sp|Q4R5F2|LGUL_MACFA Lactoylglutathione lyase OS=Macaca fascicularis GN=GLO1 PE=2 SV=3//8.30326e-128
XM_007972738.1	0.47224	6.31E-09	9.84E-08	sp|Q9H8U3|ZFAN3_HUMAN AN1-type zinc finger protein 3 OS=Homo sapiens GN=ZFAND3 PE=1 SV=1//3.16195e-121
XM_007972742.1	-0.68144	4.06E-07	5.12E-06	sp|Q9P0B6|CC167_HUMAN Coiled-coil domain-containing protein 167 OS=Homo sapiens GN=CCDC167 PE=1 SV=2//1.01559e-59
XM_007972744.1	0.68965	2.25E-12	4.95E-11	sp|Q5R981|CMTR1_PONAB Cap-specific mRNA (nucleoside-2'-O-)-methyltransferase 1 OS=Pongo abelii GN=CMTR1 PE=2 SV=1//0
XM_007972753.1	1.3212	6.00E-29	3.85E-27	sp|Q95LL3|TB22B_MACFA TBC1 domain family member 22B OS=Macaca fascicularis GN=TBC1D22B PE=2 SV=1//0
XM_007972754.1	2.7332	########	########	sp|P11309|PIM1_HUMAN Serine/threonine-protein kinase pim-1 OS=Homo sapiens GN=PIM1 PE=1 SV=3//0
XM_007972781.1	1.0834	5.01E-47	6.44E-45	sp|P84104|SRSF3_MOUSE Serine/arginine-rich splicing factor 3 OS=Mus musculus GN=Srsf3 PE=1 SV=1//3.62012e-51
XM_007972798.1	2.3135	2.42E-05	0.000238	sp|P0C671|CF222_HUMAN Uncharacterized protein C6orf222 OS=Homo sapiens GN=C6orf222 PE=1 SV=1//0
XM_007972832.1	#NAME?	0.001031	0.007588	-//-
XM_007972852.1	0.54631	1.16E-09	1.97E-08	sp|Q6BDS2|URFB1_HUMAN UHRF1-binding protein 1 OS=Homo sapiens GN=UHRF1BP1 PE=1 SV=1//0
XM_007972856.1	0.2293	0.003994	0.02563	sp|P40189|IL6RB_HUMAN Interleukin-6 receptor subunit beta OS=Homo sapiens GN=IL6ST PE=1 SV=2//0
XM_007972899.1	-0.95235	0.004312	0.027427	-//-
XM_007972911.1	0.67855	0.000559	0.004344	sp|Q8NC56|LEMD2_HUMAN LEM domain-containing protein 2 OS=Homo sapiens GN=LEMD2 PE=1 SV=1//4.69758e-09
XM_007972913.1	2.9138	########	########	sp|Q96PC2|IP6K3_HUMAN Inositol hexakisphosphate kinase 3 OS=Homo sapiens GN=IP6K3 PE=1 SV=2//0
XM_007972915.1	0.64713	2.33E-06	2.67E-05	sp|Q16611|BAK_HUMAN Bcl-2 homologous antagonist/killer OS=Homo sapiens GN=BAK1 PE=1 SV=1//1.47138e-110
XM_007972918.1	-0.23327	0.001832	0.012687	sp|Q14573|ITPR3_HUMAN Inositol 1,4,5-trisphosphate receptor type 3 OS=Homo sapiens GN=ITPR3 PE=1 SV=2//0
XM_007972937.1	0.57917	0.004059	0.026012	sp|O43189|PHF1_HUMAN PHD finger protein 1 OS=Homo sapiens GN=PHF1 PE=1 SV=3//0
XM_007972957.1	-0.48377	2.46E-11	4.97E-10	sp|P62271|RS18_RAT 40S ribosomal protein S18 OS=Rattus norvegicus GN=Rps18 PE=1 SV=3//4.33385e-93
XM_007972986.1	-1.5113	2.79E-05	0.000271	sp|P28068|DMB_HUMAN HLA class II histocompatibility antigen, DM beta chain OS=Homo sapiens GN=HLA-DMB PE=1 SV=1//5.91629e-148
XM_007972992.1	-0.43696	0.001456	0.010375	sp|Q03519|TAP2_HUMAN Antigen peptide transporter 2 OS=Homo sapiens GN=TAP2 PE=1 SV=1//0
XM_007973048.1	1.052	9.01E-13	2.05E-11	sp|O77932|DXO_HUMAN Decapping and exoribonuclease protein OS=Homo sapiens GN=DXO PE=2 SV=2//0
XM_007973076.1	-0.46124	0.005366	0.033355	sp|Q9Y330|ZBT12_HUMAN Zinc finger and BTB domain-containing protein 12 OS=Homo sapiens GN=ZBTB12 PE=1 SV=1//0
XM_007973077.1	-0.63115	0.004757	0.030027	sp|Q5RAF4|NEUR1_PONAB Sialidase-1 OS=Pongo abelii GN=NEU1 PE=2 SV=1//0
XM_007973079.1	-0.50323	0.001371	0.009815	sp|Q5R7D3|HSP71_PONAB Heat shock 70 kDa protein 1 OS=Pongo abelii GN=HSPA1 PE=2 SV=1//0
XM_007973083.1	-1.1077	1.70E-05	0.00017	sp|Q9Y334|VWA7_HUMAN von Willebrand factor A domain-containing protein 7 OS=Homo sapiens GN=VWA7 PE=2 SV=4//0
XM_007973086.1	-0.44938	5.83E-05	0.00054	sp|O35900|LSM2_MOUSE U6 snRNA-associated Sm-like protein LSm2 OS=Mus musculus GN=Lsm2 PE=3 SV=1//9.5542e-58
XM_007973129.1	-1.0169	0.000462	0.003658	sp|Q8NDX9|LY65B_HUMAN Lymphocyte antigen 6 complex locus protein G5b OS=Homo sapiens GN=LY6G5B PE=1 SV=1//3.56555e-117
XM_007973144.1	-0.83436	1.17E-12	2.65E-11	sp|Q31612|1B73_HUMAN HLA class I histocompatibility antigen, B-73 alpha chain OS=Homo sapiens GN=HLA-B PE=1 SV=1//4.38819e-180
XM_007973201.1	1.8067	1.04E-99	5.04E-97	sp|Q7YR42|IEX1_PANTR Radiation-inducible immediate-early gene IEX-1 OS=Pan troglodytes GN=IER3 PE=3 SV=1//1.23464e-83
XM_007973204.1	-0.77985	1.76E-29	1.15E-27	sp|P09244|TBB7_CHICK Tubulin beta-7 chain OS=Gallus gallus PE=2 SV=1//0
XM_007973210.1	2.607	########	########	sp|Q5R4L1|PLK2_PONAB Serine/threonine-protein kinase PLK2 OS=Pongo abelii GN=PLK2 PE=3 SV=1//0
XM_007973241.1	0.66571	3.65E-05	0.000348	sp|Q5TM62|RT18B_MACMU 28S ribosomal protein S18b, mitochondrial OS=Macaca mulatta GN=MRPS18B PE=3 SV=1//5.42683e-167
XM_007973321.1	1.7802	0.008433	0.049653	sp|Q01063|PDE4D_MOUSE cAMP-specific 3',5'-cyclic phosphodiesterase 4D OS=Mus musculus GN=Pde4d PE=1 SV=2//0
XM_007973330.1	2.7006	1.33E-10	2.51E-09	sp|Q08499|PDE4D_HUMAN cAMP-specific 3',5'-cyclic phosphodiesterase 4D OS=Homo sapiens GN=PDE4D PE=1 SV=2//0
XM_007973341.1	1.1028	2.22E-06	2.54E-05	sp|Q6R2W3|SCND3_HUMAN SCAN domain-containing protein 3 OS=Homo sapiens GN=ZBED9 PE=2 SV=1//0
XM_007973356.1	0.92317	3.72E-16	1.11E-14	sp|Q16670|ZSC26_HUMAN Zinc finger and SCAN domain-containing protein 26 OS=Homo sapiens GN=ZSCAN26 PE=1 SV=2//0

XM_007973386.1	1.9853	0.003447	0.02243	sp|Q08499|PDE4D_HUMAN cAMP-specific 3',5'-cyclic phosphodiesterase 4D OS=Homo sapiens GN=PDE4D PE=1 SV=2//0
XM_007973397.1	1.4963	0.00549	0.034058	sp|P02262|H2A1_RAT Histone H2A type 1 OS=Rattus norvegicus PE=1 SV=2//2.1685e-69
XM_007973401.1	-1.087	0.006838	0.041271	sp|Q28DR4|H4_XENTR Histone H4 OS=Xenopus tropicalis GN=TGas006m08.1 PE=3 SV=1//1.21708e-50
XM_007973405.1	2.7866	9.92E-05	0.000883	sp|P16401|H15_HUMAN Histone H1.5 OS=Homo sapiens GN=HIST1H1B PE=1 SV=3//9.85065e-30
XM_007973411.1	-0.67836	4.08E-05	0.000386	sp|Q9UJN7|ZN391_HUMAN Zinc finger protein 391 OS=Homo sapiens GN=ZNF391 PE=2 SV=2//0
XM_007973415.1	1.977	0.000776	0.005857	sp|Q5R5U3|ZN271_PONAB Zinc finger protein 271 OS=Pongo abelii GN=ZNF271 PE=2 SV=1//6.43128e-06
XM_007973425.1	2.6958	2.78E-06	3.15E-05	sp|P02262|H2A1_RAT Histone H2A type 1 OS=Rattus norvegicus PE=1 SV=2//7.85131e-67
XM_007973428.1	-0.74933	0.000233	0.001946	sp|O14618|CCS_HUMAN Copper chaperone for superoxide dismutase OS=Homo sapiens GN=CCS PE=1 SV=1//0
XM_007973434.1	0.72585	3.62E-08	5.13E-07	sp|Q9ULW3|ABT1_HUMAN Activator of basal transcription 1 OS=Homo sapiens GN=ABT1 PE=1 SV=1//1.46079e-168
XM_007973435.1	-0.28792	0.006966	0.041914	sp|Q86SG4|DPCA2_HUMAN Putative Dresden prostate carcinoma protein 2 OS=Homo sapiens GN=HMGN2P46 PE=5 SV=1//5.17345e-13
XM_007973449.1	0.4433	0.00088	0.006565	sp|Q5R4D8|SIM15_PONAB Small integral membrane protein 15 OS=Pongo abelii GN=SMIM15 PE=3 SV=1//1.49422e-28
XM_007973474.1	2.5143	0.001019	0.007512	sp|P16402|H13_HUMAN Histone H1.3 OS=Homo sapiens GN=HIST1H1D PE=1 SV=2//4.72919e-28
XM_007973479.1	-1.0074	0.000185	0.00157	sp|P10854|H2B1M_MOUSE Histone H2B type 1-M OS=Mus musculus GN=Hist1h2bm PE=1 SV=2//5.2168e-58
XM_007973482.1	4.3843	3.95E-26	2.21E-24	sp|P02252|H14_RABIT Histone H1.4 OS=Oryctolagus cuniculus GN=HIST1H1E PE=1 SV=2//5.65308e-32
XM_007973483.1	1.8428	3.53E-27	2.08E-25	sp|P0C169|H2A1C_RAT Histone H2A type 1-C OS=Rattus norvegicus PE=1 SV=2//9.37257e-69
XM_007973487.1	Inf	5.54E-09	8.73E-08	sp|Q6LED0|H31_RAT Histone H3.1 OS=Rattus norvegicus PE=1 SV=3//1.09076e-91
XM_007973488.1	1.5655	2.05E-05	0.000203	sp|Q6LED0|H31_RAT Histone H3.1 OS=Rattus norvegicus PE=1 SV=3//6.33056e-71
XM_007973508.1	-1.0538	0.007439	0.044351	sp|Q9Y2C5|S17A4_HUMAN Probable small intestine urate exporter OS=Homo sapiens GN=SLC17A4 PE=2 SV=1//0
XM_007973545.1	0.4588	0.000677	0.005184	sp|O95551|TYDP2_HUMAN Tyrosyl-DNA phosphodiesterase 2 OS=Homo sapiens GN=TDP2 PE=1 SV=1//0
XM_007973546.1	-0.49948	0.000281	0.002315	sp|Q5R833|ACO13_PONAB Acyl-coenzyme A thioesterase 13 OS=Pongo abelii GN=ACOT13 PE=2 SV=1//1.01889e-77
XM_007973548.1	0.89382	3.52E-07	4.46E-06	sp|Q9HD23|MRS2_HUMAN Magnesium transporter MRS2 homolog, mitochondrial OS=Homo sapiens GN=MRS2 PE=1 SV=1//0
XM_007973572.1	1.2257	5.31E-34	4.24E-32	sp|Q9HCJ5|ZSWM6_HUMAN Zinc finger SWIM domain-containing protein 6 OS=Homo sapiens GN=ZSWIM6 PE=1 SV=2//4.94718e-35
XM_007973573.1	0.89897	1.35E-17	4.52E-16	sp|Q06945|SOX4_HUMAN Transcription factor SOX-4 OS=Homo sapiens GN=SOX4 PE=1 SV=1//2.01529e-119
XM_007973575.1	0.87746	0.001857	0.012843	sp|Q5VV42|CDKAL_HUMAN Threonylcarbamoyladenosine tRNA methylthiotransferase OS=Homo sapiens GN=CDKAL1 PE=1 SV=1//0
XM_007973581.1	0.90818	1.02E-09	1.74E-08	sp|Q6ZNC8|MBOA1_HUMAN Lysophospholipid acyltransferase 1 OS=Homo sapiens GN=MBOAT1 PE=1 SV=1//0
XM_007973587.1	2.1198	3.15E-07	4.01E-06	sp|Q7Z419|R144B_HUMAN E3 ubiquitin-protein ligase RNF144B OS=Homo sapiens GN=RNF144B PE=1 SV=1//0
XM_007973590.1	0.50958	4.18E-06	4.60E-05	sp|P35659|DEK_HUMAN Protein DEK OS=Homo sapiens GN=DEK PE=1 SV=1//1.83456e-144
XM_007973595.1	-0.84682	0.000104	0.00092	sp|Q3BCR4|TPMT_CHLAE Thiopurine S-methyltransferase OS=Chlorocebus aethiops GN=TPMT PE=2 SV=1//1.21611e-161
XM_007973607.1	1.2659	2.98E-55	5.22E-53	sp|P49790|NU153_HUMAN Nuclear pore complex protein Nup153 OS=Homo sapiens GN=NUP153 PE=1 SV=2//0
XM_007973608.1	0.29296	0.002674	0.017779	sp|Q9UBU6|FA8A1_HUMAN Protein FAM8A1 OS=Homo sapiens GN=FAM8A1 PE=1 SV=1//0
XM_007973621.1	-0.57666	0.001136	0.008282	sp|P36959|GMPR1_HUMAN GMP reductase 1 OS=Homo sapiens GN=GMPR PE=1 SV=1//0
XM_007973652.1	0.75713	4.91E-17	1.58E-15	sp|Q96S59|RANB9_HUMAN Ran-binding protein 9 OS=Homo sapiens GN=RANBP9 PE=1 SV=1//0
XM_007973675.1	0.86448	7.34E-13	1.69E-11	sp|Q9UI26|IPO11_HUMAN Importin-11 OS=Homo sapiens GN=IPO11 PE=1 SV=1//0
XM_007973676.1	1.9226	4.62E-67	1.10E-64	sp|P05305|EDN1_HUMAN Endothelin-1 OS=Homo sapiens GN=EDN1 PE=1 SV=1//6.40237e-115
XM_007973690.1	0.41392	0.001255	0.009056	sp|P0DJ93|SIM13_HUMAN Small integral membrane protein 13 OS=Homo sapiens GN=SMIM13 PE=3 SV=1//4.71481e-40
XM_007973718.1	1.2345	1.81E-18	6.38E-17	sp|Q8N0V5|GNT2A_HUMAN N-acetyllactosaminide beta-1,6-N-acetylglucosaminyl-transferase, isoform A OS=Homo sapiens GN=GCNT2 PE=2 SV=1//0
XM_007973743.1	0.7841	0.000793	0.005974	sp|O43324|MCA3_HUMAN Eukaryotic translation elongation factor 1 epsilon-1 OS=Homo sapiens GN=EEF1E1 PE=1 SV=1//4.09762e-108

XM_007973750.1	-0.35981	1.72E-05	0.000171	sp|Q8NBS9|TXND5_HUMAN Thioredoxin domain-containing protein 5 OS=Homo sapiens GN=TXNDC5 PE=1 SV=2//0
XM_007973771.1	1.3888	3.57E-08	5.07E-07	sp|Q9BRS2|RIOK1_HUMAN Serine/threonine-protein kinase RIO1 OS=Homo sapiens GN=RIOK1 PE=1 SV=2//0
XM_007973784.1	-2.0234	0.001563	0.011062	sp|Q6UX98|ZDH24_HUMAN Probable palmitoyltransferase ZDHHC24 OS=Homo sapiens GN=ZDHHC24 PE=1 SV=1//5.3689e-58
XM_007973802.1	0.7554	2.14E-07	2.79E-06	sp|O75818|RPP40_HUMAN Ribonuclease P protein subunit p40 OS=Homo sapiens GN=RPP40 PE=1 SV=3//0
XM_007973803.1	0.85207	6.29E-17	2.00E-15	sp|Q9Y232|CDYL1_HUMAN Chromodomain Y-like protein OS=Homo sapiens GN=CDYL PE=1 SV=2//0
XM_007973818.1	2.1525	3.52E-95	1.54E-92	sp|Q5TGL8|PXDC1_HUMAN PX domain-containing protein 1 OS=Homo sapiens GN=PXDC1 PE=2 SV=3//4.46806e-161
XM_007973825.1	-0.63888	0.000494	0.003895	sp|Q6UX98|ZDH24_HUMAN Probable palmitoyltransferase ZDHHC24 OS=Homo sapiens GN=ZDHHC24 PE=1 SV=1//5.86274e-121
XM_007973849.1	-0.6624	2.93E-08	4.20E-07	sp|P50453|SPB9_HUMAN Serpin B9 OS=Homo sapiens GN=SERPINB9 PE=1 SV=1//0
XM_007973863.1	1.2945	1.39E-14	3.71E-13	sp|Q12948|FOXC1_HUMAN Forkhead box protein C1 OS=Homo sapiens GN=FOXC1 PE=1 SV=3//0
XM_007973864.1	0.44793	0.000933	0.006917	sp|Q12947|FOXF2_HUMAN Forkhead box protein F2 OS=Homo sapiens GN=FOXF2 PE=1 SV=2//1.76971e-160
XM_007973869.1	1.5522	1.08E-09	1.84E-08	sp|Q9UKP5|ATS6_HUMAN A disintegrin and metalloproteinase with thrombospondin motifs 6 OS=Homo sapiens GN=ADAMTS6 PE=2 SV=2//0
XM_007973919.1	0.77939	0.000501	0.003936	sp|P36406|TRI23_HUMAN E3 ubiquitin-protein ligase TRIM23 OS=Homo sapiens GN=TRIM23 PE=1 SV=1//0
XM_007973920.1	0.97294	4.89E-07	6.12E-06	sp|A2T6E3|ZSC12_MACNE Zinc finger and SCAN domain-containing protein 12 OS=Macaca nemestrina GN=ZSCAN12 PE=3 SV=1//5.0922e-06
XM_007973925.1	2.5662	4.95E-19	1.81E-17	sp|Q6LED0|H31_RAT Histone H3.1 OS=Rattus norvegicus PE=1 SV=3//1.52001e-74
XM_007973926.1	0.52519	0.001271	0.00917	sp|Q5VV43|K0319_HUMAN Dyslexia-associated protein KIAA0319 OS=Homo sapiens GN=KIAA0319 PE=1 SV=1//8.33806e-40
XM_007973935.1	-0.97936	1.00E-06	1.21E-05	-//-
XM_007973946.1	0.75865	2.71E-08	3.90E-07	sp|Q96EQ0|SGTB_HUMAN Small glutamine-rich tetratricopeptide repeat-containing protein beta OS=Homo sapiens GN=SGTB PE=1 SV=1//0
XM_007973967.1	0.28674	0.004164	0.026608	sp|Q9UBB5|MBD2_HUMAN Methyl-CpG-binding domain protein 2 OS=Homo sapiens GN=MBD2 PE=1 SV=1//3.72589e-09
XM_007974053.1	-0.49088	1.92E-06	2.22E-05	sp|P42765|THIM_HUMAN 3-ketoacyl-CoA thiolase, mitochondrial OS=Homo sapiens GN=ACAA2 PE=1 SV=2//0
XM_007974059.1	-0.50781	5.54E-11	1.08E-09	sp|P18621|RL17_HUMAN 60S ribosomal protein L17 OS=Homo sapiens GN=RPL17 PE=1 SV=3//3.32433e-123
XM_007974093.1	-0.65038	2.16E-14	5.64E-13	sp|Q9UBX1|CATF_HUMAN Cathepsin F OS=Homo sapiens GN=CTSF PE=1 SV=1//0
XM_007974106.1	0.73372	0.000582	0.004512	sp|Q5R4B4|HDHD2_PONAB Haloacid dehalogenase-like hydrolase domain-containing protein 2 OS=Pongo abelii GN=HDHD2 PE=2 SV=1//7.22211e-179
XM_007974150.1	-0.72333	6.82E-22	3.00E-20	sp|A5A6H5|ATPA_PANTR ATP synthase subunit alpha, mitochondrial OS=Pan troglodytes GN=ATP5A1 PE=2 SV=1//0
XM_007974189.1	-0.5887	5.26E-10	9.24E-09	sp|Q9NY33|DPP3_HUMAN Dipeptidyl peptidase 3 OS=Homo sapiens GN=DPP3 PE=1 SV=2//0
XM_007974254.1	0.79841	5.10E-05	0.000476	sp|Q5R8Y3|RPR1A_PONAB Regulation of nuclear pre-mRNA domain-containing protein 1A OS=Pongo abelii GN=RPRD1A PE=2 SV=1//0
XM_007974265.1	0.41797	0.002759	0.018298	sp|Q5RAE6|ZNF24_PONAB Zinc finger protein 24 OS=Pongo abelii GN=ZNF24 PE=2 SV=1//0
XM_007974346.1	-0.50616	0.000533	0.004165	sp|Q9P2G3|KLH14_HUMAN Kelch-like protein 14 OS=Homo sapiens GN=KLHL14 PE=1 SV=2//0
XM_007974381.1	-0.9215	0.000501	0.003936	sp|Q5R9X1|CADH2_PONAB Cadherin-2 OS=Pongo abelii GN=CDH2 PE=2 SV=1//0
XM_007974383.1	0.89398	0.005671	0.03504	sp|Q5R685|P85A_PONAB Phosphatidylinositol 3-kinase regulatory subunit alpha OS=Pongo abelii GN=PIK3R1 PE=2 SV=1//0
XM_007974399.1	1.1379	2.03E-08	2.98E-07	sp|Q92750|TAF4B_HUMAN Transcription initiation factor TFIID subunit 4B OS=Homo sapiens GN=TAF4B PE=1 SV=2//0
XM_007974473.1	1.1889	1.75E-10	3.27E-09	sp|Q92908|GATA6_HUMAN Transcription factor GATA-6 OS=Homo sapiens GN=GATA6 PE=1 SV=2//0
XM_007974477.1	-0.31986	0.007574	0.04502	sp|P62315|SMD1_MOUSE Small nuclear ribonucleoprotein Sm D1 OS=Mus musculus GN=Snrpd1 PE=3 SV=1//2.59192e-61
XM_007974489.1	0.47349	2.28E-06	2.61E-05	sp|Q96FV9|THOC1_HUMAN THO complex subunit 1 OS=Homo sapiens GN=THOC1 PE=1 SV=1//0
XM_007974497.1	-0.52566	0.007488	0.04459	sp|Q5RAT4|ENOF1_PONAB Mitochondrial enolase superfamily member 1 OS=Pongo abelii GN=ENOSF1 PE=2 SV=1//0
XM_007974506.1	-1.9376	0.000937	0.00694	sp|Q8N3J2|METL4_HUMAN Methyltransferase-like protein 4 OS=Homo sapiens GN=METTL4 PE=2 SV=3//0
XM_007974515.1	2.3673	0.000102	0.000905	sp|Q92539|LPIN2_HUMAN Phosphatidate phosphatase LPIN2 OS=Homo sapiens GN=LPIN2 PE=1 SV=1//0
XM_007974520.1	1.2751	4.64E-05	0.000435	sp|Q9BXX0|EMIL2_HUMAN EMILIN-2 OS=Homo sapiens GN=EMILIN2 PE=1 SV=3//1.63525e-117

XM_007974531.1	-0.5117	8.31E-10	1.43E-08	sp|Q3THE2|ML12B_MOUSE Myosin regulatory light chain 12B OS=Mus musculus GN=Myl12b PE=1 SV=2//8.65065e-109
XM_007974534.1	1.0338	1.10E-24	5.66E-23	sp|Q5IS58|TGIF1_PANTR Homeobox protein TGIF1 OS=Pan troglodytes GN=TGIF1 PE=2 SV=1//0
XM_007974535.1	1.1628	1.21E-08	1.82E-07	-//-
XM_007974636.1	0.5727	1.54E-08	2.29E-07	sp|Q6IQ22|RAB12_HUMAN Ras-related protein Rab-12 OS=Homo sapiens GN=RAB12 PE=1 SV=3//1.45149e-128
XM_007974644.1	0.56018	1.35E-07	1.80E-06	sp|Q16594|TAF9_HUMAN Transcription initiation factor TFIID subunit 9 OS=Homo sapiens GN=TAF9 PE=1 SV=1//1.74173e-176
XM_007974657.1	-0.54318	1.05E-09	1.79E-08	sp|Q9GZX9|TWSG1_HUMAN Twisted gastrulation protein homolog 1 OS=Homo sapiens GN=TWSG1 PE=1 SV=1//1.67903e-150
XM_007974664.1	0.6606	4.24E-16	1.26E-14	sp|Q13636|RAB31_HUMAN Ras-related protein Rab-31 OS=Homo sapiens GN=RAB31 PE=1 SV=1//1.75162e-126
XM_007974676.1	-1.0117	1.13E-31	8.09E-30	sp|O14732|IMPA2_HUMAN Inositol monophosphatase 2 OS=Homo sapiens GN=IMPA2 PE=1 SV=1//0
XM_007974683.1	0.37682	1.70E-06	1.98E-05	sp|Q7LBR1|CHM1B_HUMAN Charged multivesicular body protein 1b OS=Homo sapiens GN=CHMP1B PE=1 SV=1//3.02954e-101
XM_007974712.1	0.40378	2.16E-07	2.81E-06	sp|Q9Y4W6|AFG32_HUMAN AFG3-like protein 2 OS=Homo sapiens GN=AFG3L2 PE=1 SV=2//0
XM_007974714.1	-0.43262	9.44E-05	0.000843	sp|Q9BUF5|TBB6_HUMAN Tubulin beta-6 chain OS=Homo sapiens GN=TUBB6 PE=1 SV=1//0
XM_007974715.1	1.3809	1.79E-14	4.73E-13	sp|Q96N28|SLMO1_HUMAN Protein slowmo homolog 1 OS=Homo sapiens GN=SLMO1 PE=2 SV=1//1.55032e-72
XM_007974718.1	0.80845	1.28E-06	1.52E-05	sp|Q8TAP6|CEP76_HUMAN Centrosomal protein of 76 kDa OS=Homo sapiens GN=CEP76 PE=1 SV=1//0
XM_007974721.1	-1.4629	0.004691	0.029625	sp|Q08AE8|SPIR1_HUMAN Protein spire homolog 1 OS=Homo sapiens GN=SPIRE1 PE=1 SV=3//1.71086e-102
XM_007974762.1	0.63612	7.19E-12	1.51E-10	sp|Q4R7K1|MCES_MACFA mRNA cap guanine-N7 methyltransferase OS=Macaca fascicularis GN=RNMT PE=2 SV=1//0
XM_007974769.1	-1.0194	0.000125	0.001092	sp|Q5R7L8|BL1S1_PONAB Biogenesis of lysosome-related organelles complex 1 subunit 1 OS=Pongo abelii GN=BLOC1S1 PE=2 SV=2//1.34024e-76
XM_007974774.1	1.5014	1.25E-64	2.77E-62	sp|O15118|NPC1_HUMAN Niemann-Pick C1 protein OS=Homo sapiens GN=NPC1 PE=1 SV=2//0
XM_007974779.1	-0.64107	1.78E-10	3.32E-09	sp|Q9Y3B7|RM11_HUMAN 39S ribosomal protein L11, mitochondrial OS=Homo sapiens GN=MRPL11 PE=1 SV=1//6.91001e-114
XM_007974781.1	-1.9728	0.003569	0.02311	-//-
XM_007974830.1	-2.385	0.005443	0.033813	sp|Q8N1L1|CV037_HUMAN Putative uncharacterized protein encoded by LINC00528 OS=Homo sapiens GN=LINC00528 PE=5 SV=1//7.59091e-38
XM_007974845.1	1.739	3.33E-07	4.23E-06	sp|Q7RTP6|MICA3_HUMAN Protein-methionine sulfoxide oxidase MICAL3 OS=Homo sapiens GN=MICAL3 PE=1 SV=2//0
XM_007974860.1	5.0902	2.57E-26	1.44E-24	sp|Q9UMW8|UBP18_HUMAN Ubl carboxyl-terminal hydrolase 18 OS=Homo sapiens GN=USP18 PE=1 SV=1//3.80731e-144
XM_007974862.1	4.7982	4.86E-26	2.70E-24	sp|Q5RE63|UBP18_PONAB Ubl carboxyl-terminal hydrolase 18 OS=Pongo abelii GN=USP18 PE=2 SV=1//9.27867e-22
XM_007974863.1	-0.52724	2.93E-06	3.31E-05	sp|Q9HCC0|MCCB_HUMAN Methylcrotonoyl-CoA carboxylase beta chain, mitochondrial OS=Homo sapiens GN=MCCC2 PE=1 SV=1//0
XM_007974868.1	0.70013	0.00047	0.003718	sp|Q14129|DGCR6_HUMAN Protein DGCR6 OS=Homo sapiens GN=DGCR6 PE=1 SV=3//9.63848e-125
XM_007974893.1	-0.90888	0.001489	0.010577	sp|P46821|MAP1B_HUMAN Microtubule-associated protein 1B OS=Homo sapiens GN=MAP1B PE=1 SV=2//0
XM_007974910.1	-0.75087	3.44E-14	8.83E-13	sp|P49593|PPM1F_HUMAN Protein phosphatase 1F OS=Homo sapiens GN=PPM1F PE=1 SV=3//0
XM_007974912.1	-1.0081	0.000474	0.003744	sp|O60688|YPEL1_HUMAN Protein yippee-like 1 OS=Homo sapiens GN=YPEL1 PE=3 SV=1//8.42994e-81
XM_007974921.1	1.1772	6.47E-10	1.13E-08	sp|A8MPS7|YDJC_HUMAN Carbohydrate deacetylase OS=Homo sapiens GN=YDJC PE=1 SV=1//0
XM_007974934.1	0.30106	0.000716	0.005446	sp|P42356|PI4KA_HUMAN Phosphatidylinositol 4-kinase alpha OS=Homo sapiens GN=PI4KA PE=1 SV=3//0
XM_007974987.1	1.3643	4.46E-06	4.89E-05	sp|Q16587|ZNF74_HUMAN Zinc finger protein 74 OS=Homo sapiens GN=ZNF74 PE=2 SV=3//0
XM_007974990.1	-0.74843	7.15E-06	7.56E-05	sp|Q9BZR6|RTN4R_HUMAN Reticulon-4 receptor OS=Homo sapiens GN=RTN4R PE=1 SV=1//0
XM_007974997.1	0.40342	0.000298	0.002441	sp|Q8WYQ5|DGCR8_HUMAN Microprocessor complex subunit DGCR8 OS=Homo sapiens GN=DGCR8 PE=1 SV=1//0
XM_007974998.1	0.71486	2.74E-09	4.46E-08	sp|Q8IZ69|TRM2A_HUMAN tRNA (uracil-5-)-methyltransferase homolog A OS=Homo sapiens GN=TRMT2A PE=1 SV=2//0
XM_007975031.1	0.71868	7.76E-05	0.000705	sp|Q7L3V2|BOP_HUMAN Protein Bop OS=Homo sapiens GN=BOP PE=1 SV=1//0
XM_007975035.1	-0.86286	0.000172	0.001467	sp|P13224|GP1BB_HUMAN Platelet glycoprotein Ib beta chain OS=Homo sapiens GN=GP1BB PE=1 SV=1//5.11009e-67
XM_007975038.1	-2.4349	0.001763	0.01228	sp|Q99719|SEPT5_HUMAN Septin-5 OS=Homo sapiens GN=SEPT5 PE=1 SV=1//0

XM_007975046.1	-0.5216	2.02E-06	2.34E-05	sp|Q9NQ50|RM40_HUMAN 39S ribosomal protein L40, mitochondrial OS=Homo sapiens GN=MRPL40 PE=1 SV=1//9.49874e-96
XM_007975089.1	-0.72235	1.98E-07	2.58E-06	sp|Q8WYQ3|CHC10_HUMAN Coiled-coil-helix-coiled-coil-helix domain-containing protein 10, mitochondrial OS=Homo sapiens GN=CHCHD10 PE=1 SV=1//1.57138e-34
XM_007975099.1	-1.5155	5.79E-26	3.17E-24	sp|P24347|MMP11_HUMAN Stromelysin-3 OS=Homo sapiens GN=MMP11 PE=1 SV=3//0
XM_007975114.1	-1.1939	5.10E-56	9.22E-54	sp|Q6DN04|MIF_MACMU Macrophage migration inhibitory factor OS=Macaca mulatta GN=MIF PE=3 SV=4//2.48942e-80
XM_007975125.1	0.54246	0.005066	0.031687	sp|Q16676|FOXD1_HUMAN Forkhead box protein D1 OS=Homo sapiens GN=FOXD1 PE=2 SV=1//1.83822e-69
XM_007975140.1	-1.3105	2.09E-32	1.53E-30	sp|P30046|DOPD_HUMAN D-dopachrome decarboxylase OS=Homo sapiens GN=DDT PE=1 SV=3//2.30482e-77
XM_007975143.1	-1.4969	1.06E-10	2.02E-09	sp|P30711|GSTT1_HUMAN Glutathione S-transferase theta-1 OS=Homo sapiens GN=GSTT1 PE=1 SV=4//4.79598e-168
XM_007975170.1	-3.376	9.82E-07	1.19E-05	sp|P19440|GGT1_HUMAN Gamma-glutamyltranspeptidase 1 OS=Homo sapiens GN=GGT1 PE=1 SV=2//0
XM_007975174.1	-1.6488	9.55E-33	7.15E-31	sp|Q2VPJ9|LR75B_HUMAN Leucine-rich repeat-containing protein 75B OS=Homo sapiens GN=LRRC75B PE=2 SV=1//4.60015e-131
XM_007975177.1	2.1119	1.49E-66	3.46E-64	sp|Q8TED0|UTP15_HUMAN U3 small nucleolar RNA-associated protein 15 homolog OS=Homo sapiens GN=UTP15 PE=1 SV=3//0
XM_007975184.1	-0.62407	1.61E-05	0.000161	sp|P62323|SMD3_XENLA Small nuclear ribonucleoprotein Sm D3 OS=Xenopus laevis GN=snrpd3 PE=2 SV=1//3.7429e-78
XM_007975199.1	0.93577	2.75E-22	1.24E-20	sp|Q69YQ0|CYTSA_HUMAN Cytospin-A OS=Homo sapiens GN=SPECC1L PE=1 SV=2//2.8643e-51
XM_007975200.1	1.092	8.96E-19	3.23E-17	sp|Q69YQ0|CYTSA_HUMAN Cytospin-A OS=Homo sapiens GN=SPECC1L PE=1 SV=2//2.64069e-150
XM_007975208.1	0.67186	6.00E-09	9.41E-08	sp|Q9BY89|K1671_HUMAN Uncharacterized protein KIAA1671 OS=Homo sapiens GN=KIAA1671 PE=1 SV=2//0
XM_007975223.1	2.3334	5.18E-08	7.22E-07	sp|Q8N1W1|ARG28_HUMAN Rho guanine nucleotide exchange factor 28 OS=Homo sapiens GN=ARHGEF28 PE=1 SV=3//0
XM_007975233.1	0.87459	3.18E-07	4.04E-06	sp|Q9UH36|SRR1L_HUMAN SRR1-like protein OS=Homo sapiens GN=SRRD PE=2 SV=1//0
XM_007975254.1	0.56503	7.10E-05	0.000649	sp|Q96AY4|TTC28_HUMAN Tetratricopeptide repeat protein 28 OS=Homo sapiens GN=TTC28 PE=1 SV=4//0
XM_007975271.1	3.1525	1.24800982139499e-320	3.41409983687687e-317	sp|O14682|ENC1_HUMAN Ectoderm-neural cortex protein 1 OS=Homo sapiens GN=ENC1 PE=1 SV=2//0
XM_007975273.1	1.4095	8.91E-49	1.24E-46	sp|P17861|XBP1_HUMAN X-box-binding protein 1 OS=Homo sapiens GN=XBP1 PE=1 SV=2//1.72407e-87
XM_007975306.1	-1.1081	5.40E-06	5.86E-05	sp|Q01844|EWS_HUMAN RNA-binding protein EWS OS=Homo sapiens GN=EWSR1 PE=1 SV=1//1.06888e-11
XM_007975350.1	-2.1862	8.11E-18	2.77E-16	sp|Q66H96|CABP7_RAT Calcium-binding protein 7 OS=Rattus norvegicus GN=Cabp7 PE=1 SV=1//1.54371e-148
XM_007975375.1	-0.3502	0.000138	0.001192	sp|P07686|HEXB_HUMAN Beta-hexosaminidase subunit beta OS=Homo sapiens GN=HEXB PE=1 SV=3//0
XM_007975381.1	1.653	2.59E-20	1.03E-18	sp|P15018|LIF_HUMAN Leukemia inhibitory factor OS=Homo sapiens GN=LIF PE=1 SV=1//2.04343e-110
XM_007975387.1	1.2101	2.04E-15	5.76E-14	sp|Q9BXI6|TB10A_HUMAN TBC1 domain family member 10A OS=Homo sapiens GN=TBC1D10A PE=1 SV=1//0
XM_007975394.1	0.33107	7.84E-05	0.000712	sp|A2VDN6|SF3A1_BOVIN Splicing factor 3A subunit 1 OS=Bos taurus GN=SF3A1 PE=2 SV=1//0
XM_007975418.1	0.33686	0.000209	0.001753	sp|O00541|PESC_HUMAN Pescadillo homolog OS=Homo sapiens GN=PES1 PE=1 SV=1//0
XM_007975426.1	-0.45873	0.001281	0.009218	sp|Q8NEJ0|DUS18_HUMAN Dual specificity protein phosphatase 18 OS=Homo sapiens GN=DUSP18 PE=1 SV=1//9.91058e-128
XM_007975427.1	0.62593	9.00E-12	1.88E-10	sp|Q9Y6X9|MORC2_HUMAN MORC family CW-type zinc finger protein 2 OS=Homo sapiens GN=MORC2 PE=1 SV=2//0
XM_007975443.1	-1.0285	8.53E-06	8.92E-05	sp|Q8WWX9|SELM_HUMAN Selenoprotein M OS=Homo sapiens GN=SELM PE=1 SV=3//2.82296e-80
XM_007975461.1	1.4238	1.57E-07	2.08E-06	sp|P53671|LIMK2_HUMAN LIM domain kinase 2 OS=Homo sapiens GN=LIMK2 PE=1 SV=1//0
XM_007975468.1	1.0514	1.15E-07	1.55E-06	sp|Q9HBE1|PATZ1_HUMAN POZ-, AT hook-, and zinc finger-containing protein 1 OS=Homo sapiens GN=PATZ1 PE=1 SV=1//1.77224e-07
XM_007975514.1	0.40583	5.09E-05	0.000475	sp|Q9Y295|DRG1_HUMAN Developmentally-regulated GTP-binding protein 1 OS=Homo sapiens GN=DRG1 PE=1 SV=1//0
XM_007975576.1	0.34937	0.000132	0.001142	sp|A9CB42|RTCB_PAPAN tRNA-splicing ligase RtcB homolog OS=Papio anubis GN=RTCB PE=3 SV=1//0
XM_007975578.1	1.0746	1.16E-29	7.65E-28	sp|Q5PXZ9|TIMP3_MACMU Metalloproteinase inhibitor 3 OS=Macaca mulatta GN=TIMP3 PE=2 SV=1//2.1731e-138
XM_007975601.1	0.53156	2.08E-10	3.82E-09	sp|P33992|MCM5_HUMAN DNA replication licensing factor MCM5 OS=Homo sapiens GN=MCM5 PE=1 SV=5//0

XM_007975611.1	0.61936	5.39E-05	0.000501	sp|Q9BWW9|APOL5_HUMAN Apolipoprotein L5 OS=Homo sapiens GN=APOL5 PE=2 SV=1//0
XM_007975644.1	2.6031	0.000901	0.006709	sp|Q9BQE5|APOL2_HUMAN Apolipoprotein L2 OS=Homo sapiens GN=APOL2 PE=1 SV=1//2.52521e-154
XM_007975651.1	-0.42807	1.13E-05	0.000115	sp|Q8IWF2|FXRD2_HUMAN FAD-dependent oxidoreductase domain-containing protein 2 OS=Homo sapiens GN=FOXRED2 PE=1 SV=1//0
XM_007975661.1	-2.811	0.005896	0.036215	sp|Q9BW83|IFT27_HUMAN Intraflagellar transport protein 27 homolog OS=Homo sapiens GN=IFT27 PE=1 SV=1//1.22709e-100
XM_007975692.1	-1.1559	2.25E-21	9.58E-20	sp|Q9BXI9|C1QT6_HUMAN Complement C1q tumor necrosis factor-related protein 6 OS=Homo sapiens GN=C1QTNF6 PE=1 SV=3//4.57964e-162
XM_007975700.1	0.874	2.07E-26	1.18E-24	sp|Q5R3F8|PPR29_HUMAN Protein phosphatase 1 regulatory subunit 29 OS=Homo sapiens GN=ELFN2 PE=1 SV=1//0
XM_007975702.1	2.5638	########	########	sp|Q00587|BORG5_HUMAN Cdc42 effector protein 1 OS=Homo sapiens GN=CDC42EP1 PE=1 SV=1//6.94607e-33
XM_007975707.1	1.0996	1.51E-11	3.10E-10	sp|Q96GD0|PLPP_HUMAN Pyridoxal phosphate phosphatase OS=Homo sapiens GN=PDXP PE=1 SV=2//8.40915e-155
XM_007975708.1	-0.62563	6.75E-06	7.17E-05	sp|Q9Y3L3|3BP1_HUMAN SH3 domain-binding protein 1 OS=Homo sapiens GN=SH3BP1 PE=1 SV=3//0
XM_007975711.1	-1.0477	3.88E-46	4.79E-44	sp|P09382|LEG1_HUMAN Galectin-1 OS=Homo sapiens GN=LGALS1 PE=1 SV=2//5.66732e-94
XM_007975718.1	1.4004	3.79E-67	9.11E-65	sp|P07305|H10_HUMAN Histone H1.0 OS=Homo sapiens GN=H1F0 PE=1 SV=3//1.08053e-49
XM_007975725.1	-1.0373	6.05E-11	1.18E-09	sp|Q5R592|RPAB2_PONAB DNA-directed RNA polymerases I, II, and III subunit RPABC2 OS=Pongo abelii GN=POLR2F PE=2 SV=1//4.44082e-58
XM_007975742.1	2.7665	1.30E-40	1.33E-38	sp|Q9ULX9|MAFF_HUMAN Transcription factor MafF OS=Homo sapiens GN=MAFF PE=1 SV=2//8.71737e-73
XM_007975762.1	-0.8372	9.25E-24	4.59E-22	sp|O43731|ERD23_HUMAN ER lumen protein-retaining receptor 3 OS=Homo sapiens GN=KDELR3 PE=2 SV=1//2.52367e-119
XM_007975766.1	-0.71765	8.43E-09	1.29E-07	sp|Q5R4S2|B4GA1_PONAB Beta-1,4-glucuronyltransferase 1 OS=Pongo abelii GN=B4GAT1 PE=2 SV=1//0
XM_007975776.1	-0.28297	0.004912	0.030875	sp|Q4R3C7|TOM22_MACFA Mitochondrial import receptor subunit TOM22 homolog OS=Macaca fascicularis GN=TOMM22 PE=2 SV=3//1.99154e-57
XM_007975786.1	-1.6785	5.79E-23	2.74E-21	sp|O95502|NPTXR_HUMAN Neuronal pentraxin receptor OS=Homo sapiens GN=NPTXR PE=3 SV=2//0
XM_007975790.1	-1.1965	7.48E-07	9.20E-06	sp|Q694C5|ABC3G_ERYPA DNA dC->dU-editing enzyme APOBEC-3G OS=Erythrocebus patas GN=APOBEC3G PE=3 SV=1//7.30418e-30
XM_007975813.1	-0.75263	2.48E-05	0.000242	sp|Q9NRW3|ABC3C_HUMAN DNA dC->dU-editing enzyme APOBEC-3C OS=Homo sapiens GN=APOBEC3C PE=1 SV=2//3.07047e-113
XM_007975814.1	-0.59473	2.49E-13	6.02E-12	sp|Q7YR25|ABC3G_CHLAE DNA dC->dU-editing enzyme APOBEC-3G (Fragment) OS=Chlorocebus aethiops GN=APOBEC3G PE=1 SV=1//1.96898e-25
XM_007975827.1	0.80491	0.00039	0.003133	sp|P01127|PDGFB_HUMAN Platelet-derived growth factor subunit B OS=Homo sapiens GN=PDGFB PE=1 SV=1//2.61176e-151
XM_007975829.1	-1.0508	4.46E-12	9.55E-11	sp|Q5R703|SNG1_PONAB Synaptogyrin-1 OS=Pongo abelii GN=SYNGR1 PE=2 SV=1//1.89329e-123
XM_007975831.1	-2.7634	0.00045	0.003566	sp|O43759|SNG1_HUMAN Synaptogyrin-1 OS=Homo sapiens GN=SYNGR1 PE=1 SV=2//1.76375e-80
XM_007975834.1	0.61415	4.49E-08	6.28E-07	sp|Q15750|TAB1_HUMAN TGF-beta-activated kinase 1 and MAP3K7-binding protein 1 OS=Homo sapiens GN=TAB1 PE=1 SV=1//0
XM_007975835.1	2.1669	3.25E-28	2.01E-26	sp|O00254|PAR3_HUMAN Proteinase-activated receptor 3 OS=Homo sapiens GN=F2RL2 PE=1 SV=1//0
XM_007975836.1	-1.297	0.002317	0.015691	sp|Q09327|MGAT3_HUMAN Beta-1,4-mannosyl-glycoprotein 4-beta-N-acetylglucosaminyltransferase OS=Homo sapiens GN=MGAT3 PE=2 SV=3//0
XM_007975840.1	0.43388	7.72E-06	8.13E-05	sp|Q52MA5|MID51_XENTR Mitochondrial dynamics protein MID51 OS=Xenopus tropicalis GN=mief1 PE=2 SV=1//0
XM_007975841.1	1.7927	########	########	sp|P18848|ATF4_HUMAN Cyclic AMP-dependent transcription factor ATF-4 OS=Homo sapiens GN=ATF4 PE=1 SV=3//0
XM_007975842.1	1.1574	5.36E-16	1.58E-14	sp|Q86WX3|AROS_HUMAN Active regulator of SIRT1 OS=Homo sapiens GN=RPS19BP1 PE=1 SV=1//4.50914e-66
XM_007975883.1	1.5124	2.72E-74	7.82E-72	sp|P56488|PAR1_PAPHA Proteinase-activated receptor 1 OS=Papio hamadryas GN=F2R PE=2 SV=1//0
XM_007975885.1	-0.64504	0.000253	0.002098	sp|Q5R9W8|XPP3_PONAB Probable Xaa-Pro aminopeptidase 3 OS=Pongo abelii GN=XPNPEP3 PE=2 SV=1//0
XM_007975887.1	-0.33343	4.88E-06	5.32E-05	sp|P50502|F10A1_HUMAN Hsc70-interacting protein OS=Homo sapiens GN=ST13 PE=1 SV=2//0
XM_007975893.1	1.1764	1.71E-36	1.51E-34	sp|P55085|PAR2_HUMAN Proteinase-activated receptor 2 OS=Homo sapiens GN=F2RL1 PE=1 SV=1//0
XM_007975919.1	-0.44816	7.65E-10	1.32E-08	sp|P12956|XRCC6_HUMAN X-ray repair cross-complementing protein 6 OS=Homo sapiens GN=XRCC6 PE=1 SV=2//0
XM_007975920.1	0.49332	2.34E-06	2.67E-05	sp|Q6ICB0|DESI1_HUMAN Desumoylating isopeptidase 1 OS=Homo sapiens GN=DESI1 PE=1 SV=1//2.0409e-98
XM_007975932.1	0.65212	5.63E-06	6.09E-05	sp|Q8N302|AGGF1_HUMAN Angiogenic factor with G patch and FHA domains 1

				OS=Homo sapiens GN=AGGF1 PE=1 SV=2//0
XM_007975955.1	-0.53927	0.0002	0.001684	sp|Q9H4I9|EMRE_HUMAN Essential MCU regulator, mitochondrial OS=Homo sapiens GN=SMDT1 PE=1 SV=1//8.28066e-53
XM_007975960.1	-0.59541	4.64E-05	0.000436	sp|P56556|NDUA6_HUMAN NADH dehydrogenase [ubiquinone] 1 alpha subcomplex subunit 6 OS=Homo sapiens GN=NDUFA6 PE=1 SV=3//1.01382e-100
XM_007975983.1	3.4798	1.55E-80	5.40E-78	sp|Q9Y3A4|RRP7A_HUMAN Ribosomal RNA-processing protein 7 homolog A OS=Homo sapiens GN=RRP7A PE=1 SV=2//3.29512e-167
XM_007976001.1	1.0339	2.56E-05	0.000249	sp|Q8IVS2|FABD_HUMAN Malonyl-CoA-acyl carrier protein transacylase, mitochondrial OS=Homo sapiens GN=MCAT PE=1 SV=2//0
XM_007976008.1	-0.83843	1.48E-15	4.22E-14	sp|P30536|TSPOA_HUMAN Translocator protein OS=Homo sapiens GN=TSPO PE=1 SV=3//1.28558e-92
XM_007976046.1	0.44132	1.02E-06	1.23E-05	sp|Q6ICC9|LDOCL_HUMAN Protein LDOC1L OS=Homo sapiens GN=LDOC1L PE=2 SV=1//5.30236e-136
XM_007976057.1	0.54934	2.09E-10	3.85E-09	sp|Q9UKX7|NUP50_HUMAN Nuclear pore complex protein Nup50 OS=Homo sapiens GN=NUP50 PE=1 SV=2//0
XM_007976078.1	-0.82934	0.000604	0.004668	sp|P23142|FBLN1_HUMAN Fibulin-1 OS=Homo sapiens GN=FBLN1 PE=1 SV=4//0
XM_007976086.1	-1.1044	0.00043	0.00342	-//-
XM_007976095.1	-0.56792	0.002446	0.016433	sp|O75347|TBCA_HUMAN Tubulin-specific chaperone A OS=Homo sapiens GN=TBCA PE=1 SV=3//4.38572e-43
XM_007976134.1	1.8317	0.001237	0.008939	sp|O00203|AP3B1_HUMAN AP-3 complex subunit beta-1 OS=Homo sapiens GN=AP3B1 PE=1 SV=3//0
XM_007976152.1	2.5939	8.07E-97	3.60E-94	sp|Q86V86|PIM3_HUMAN Serine/threonine-protein kinase pim-3 OS=Homo sapiens GN=PIM3 PE=1 SV=3//0
XM_007976180.1	-1.2837	8.60E-21	3.54E-19	sp|Q96RD6|PANX2_HUMAN Pannexin-2 OS=Homo sapiens GN=PANX2 PE=2 SV=2//0
XM_007976198.1	0.87039	7.86E-16	2.29E-14	sp|Q6ZUX7|LHPL2_HUMAN Lipoma HMGIC fusion partner-like 2 protein OS=Homo sapiens GN=LHFPL2 PE=2 SV=2//1.46502e-120
XM_007976237.1	-1.1804	1.48E-16	4.58E-15	sp|P15848|ARSB_HUMAN Arylsulfatase B OS=Homo sapiens GN=ARSB PE=1 SV=1//0
XM_007976282.1	1.0459	1.45E-32	1.08E-30	sp|Q8N9B5|JMY_HUMAN Junction-mediating and -regulatory protein OS=Homo sapiens GN=JMY PE=1 SV=2//0
XM_007976305.1	0.46136	3.68E-06	4.09E-05	sp|Q13356|PPIL2_HUMAN Peptidyl-prolyl cis-trans isomerase-like 2 OS=Homo sapiens GN=PPIL2 PE=1 SV=1//0
XM_007976306.1	0.6793	0.006529	0.039691	sp|O15547|P2RX6_HUMAN P2X purinoceptor 6 OS=Homo sapiens GN=P2RX6 PE=1 SV=2//0
XM_007976324.1	1.03	1.91E-35	1.63E-33	sp|Q9BWT7|CAR10_HUMAN Caspase recruitment domain-containing protein 10 OS=Homo sapiens GN=CARD10 PE=2 SV=2//7.56851e-123
XM_007976327.1	-0.33159	1.36E-05	0.000138	sp|Q9Y262|EIF3L_HUMAN Eukaryotic translation initiation factor 3 subunit L OS=Homo sapiens GN=EIF3L PE=1 SV=1//0
XM_007976328.1	-0.31896	0.000236	0.001966	sp|Q9UPQ9|TNR6B_HUMAN Trinucleotide repeat-containing gene 6B protein OS=Homo sapiens GN=TNRC6B PE=1 SV=4//0
XM_007976329.1	0.43122	0.000209	0.001756	sp|Q8HXY5|PUR8_MACFA Adenylosuccinate lyase OS=Macaca fascicularis GN=ADSL PE=2 SV=1//6.59884e-81
XM_007976331.1	-2.8495	0.006032	0.036968	-//-
XM_007976333.1	-0.72847	8.09E-18	2.76E-16	sp|Q60HG4|NB5R3_MACFA NADH-cytochrome b5 reductase 3 OS=Macaca fascicularis GN=CYB5R3 PE=2 SV=3//2.29325e-162
XM_007976338.1	-0.28489	0.002108	0.014412	sp|Q5RFF7|TTC38_PONAB Tetratricopeptide repeat protein 38 OS=Pongo abelii GN=TTC38 PE=2 SV=1//0
XM_007976342.1	-0.61824	1.57E-05	0.000157	sp|Q9BYB0|SHAN3_HUMAN SH3 and multiple ankyrin repeat domains protein 3 OS=Homo sapiens GN=SHANK3 PE=1 SV=3//0
XM_007976392.1	0.57133	0.002184	0.014893	sp|P17066|HSP76_HUMAN Heat shock 70 kDa protein 6 OS=Homo sapiens GN=HSPA6 PE=1 SV=2//0
XM_007976410.1	-0.45079	5.02E-07	6.27E-06	sp|Q0MQG5|NDUS2_PANTR NADH dehydrogenase [ubiquinone] iron-sulfur protein 2, mitochondrial OS=Pan troglodytes GN=NDUFS2 PE=2 SV=1//0
XM_007976458.1	-1.1689	5.34E-25	2.82E-23	sp|Q9Y624|JAM1_HUMAN Junctional adhesion molecule A OS=Homo sapiens GN=F11R PE=1 SV=1//1.59897e-173
XM_007976489.1	-1.1808	4.70E-16	1.39E-14	sp|P84889|VANG2_RAT Vang-like protein 2 OS=Rattus norvegicus GN=Vangl2 PE=2 SV=1//0
XM_007976518.1	1.22	1.29E-34	1.05E-32	sp|Q5U318|PEA15_RAT Astrocytic phosphoprotein PEA-15 OS=Rattus norvegicus GN=Pea15 PE=1 SV=1//1.35353e-75
XM_007976523.1	-0.80065	2.14E-06	2.46E-05	sp|Q969P0|IGSF8_HUMAN Immunoglobulin superfamily member 8 OS=Homo sapiens GN=IGSF8 PE=1 SV=1//0
XM_007976560.1	-0.7413	0.005864	0.036071	sp|Q9BVJ7|DUS23_HUMAN Dual specificity protein phosphatase 23 OS=Homo sapiens GN=DUSP23 PE=1 SV=1//1.08195e-91
XM_007976589.1	1.1566	2.56E-28	1.60E-26	sp|Q16666|IF16_HUMAN Gamma-interferon-inducible protein 16 OS=Homo sapiens GN=IFI16 PE=1 SV=3//0
XM_007976640.1	-0.54403	1.35E-06	1.59E-05	sp|Q4R6L9|SERC5_MACFA Serine incorporator 5 OS=Macaca fascicularis GN=SERINC5 PE=2 SV=1//0
XM_007976711.1	0.27567	0.004363	0.027716	sp|Q92733|PRCC_HUMAN Proline-rich protein PRCC OS=Homo sapiens GN=PRCC PE=1 SV=1//5.30503e-180
XM_007976713.1	-0.88651	0.005875	0.036126	sp|P51858|HDGF_HUMAN Hepatoma-derived growth factor OS=Homo sapiens

				GN=HDGF PE=1 SV=1//2.51533e-119
XM_007976732.1	-0.67671	5.96E-12	1.27E-10	sp|P48681|NEST_HUMAN Nestin OS=Homo sapiens GN=NES PE=1 SV=2//0
XM_007976742.1	-0.20128	0.004203	0.026811	sp|P02545|LMNA_HUMAN Prelamin-A/C OS=Homo sapiens GN=LMNA PE=1 SV=1//0
XM_007976749.1	0.74408	2.43E-13	5.87E-12	sp|Q5RD67|S2544_PONAB Solute carrier family 25 member 44 OS=Pongo abelii GN=SLC25A44 PE=2 SV=2//0
XM_007976754.1	-1.3134	0.008314	0.049016	sp|A2T6K4|OSTCN_MACNE Osteocalcin OS=Macaca nemestrina GN=BGLAP PE=3 SV=1//6.30847e-52
XM_007976757.1	0.4014	3.97E-06	4.39E-05	sp|Q9UPR3|SMG5_HUMAN Protein SMG5 OS=Homo sapiens GN=SMG5 PE=1 SV=3//0
XM_007976768.1	-0.20471	0.007065	0.042433	sp|Q4R963|TCPG_MACFA T-complex protein 1 subunit gamma OS=Macaca fascicularis GN=CCT3 PE=2 SV=1//0
XM_007976793.1	0.31641	0.001197	0.008679	sp|A1L020|MEX3A_HUMAN RNA-binding protein MEX3A OS=Homo sapiens GN=MEX3A PE=1 SV=1//0
XM_007976795.1	-0.98686	1.81E-12	4.02E-11	sp|Q9Y2Q5|LTOR2_HUMAN Ragulator complex protein LAMTOR2 OS=Homo sapiens GN=LAMTOR2 PE=1 SV=1//1.87719e-85
XM_007976800.1	1.1567	6.46E-41	6.70E-39	sp|Q92974|ARHG2_HUMAN Rho guanine nucleotide exchange factor 2 OS=Homo sapiens GN=ARHGEF2 PE=1 SV=4//0
XM_007976806.1	0.8525	0.004377	0.027787	sp|Q92963|RIT1_HUMAN GTP-binding protein Rit1 OS=Homo sapiens GN=RIT1 PE=1 SV=1//1.06838e-149
XM_007976844.1	-2.7228	0.000609	0.004706	sp|P14324|FPPS_HUMAN Farnesyl pyrophosphate synthase OS=Homo sapiens GN=FDPS PE=1 SV=4//0
XM_007976889.1	-0.68496	2.43E-09	3.98E-08	sp|Q8N6L1|KTAP2_HUMAN Keratinocyte-associated protein 2 OS=Homo sapiens GN=KRTCAP2 PE=1 SV=2//2.49303e-63
XM_007976893.1	-0.9556	1.20E-09	2.02E-08	sp|Q9P2X0|DPM3_HUMAN Dolichol-phosphate mannosyltransferase subunit 3 OS=Homo sapiens GN=DPM3 PE=1 SV=2//3.30685e-42
XM_007976898.1	1.6472	1.94E-38	1.82E-36	sp|P20827|EFNA1_HUMAN Ephrin-A1 OS=Homo sapiens GN=EFNA1 PE=1 SV=2//1.44764e-139
XM_007976911.1	-4.2215	0.002238	0.015223	sp|Q9Y5L5|LENEP_HUMAN Lens epithelial cell protein LEP503 OS=Homo sapiens GN=LENEP PE=2 SV=1//1.01185e-32
XM_007976966.1	1.0676	3.09E-13	7.41E-12	sp|P08887|IL6RA_HUMAN Interleukin-6 receptor subunit alpha OS=Homo sapiens GN=IL6R PE=1 SV=1//0
XM_007976976.1	0.70603	2.36E-15	6.64E-14	sp|Q7Z7E8|UB2Q1_HUMAN Ubiquitin-conjugating enzyme E2 Q1 OS=Homo sapiens GN=UBE2Q1 PE=1 SV=1//0
XM_007977016.1	-0.69406	1.84E-13	4.49E-12	sp|P51153|RAB13_HUMAN Ras-related protein Rab-13 OS=Homo sapiens GN=RAB13 PE=1 SV=1//5.70574e-145
XM_007977017.1	-0.46092	0.000209	0.001756	sp|O76095|JTB_HUMAN Protein JTB OS=Homo sapiens GN=JTB PE=1 SV=1//5.77004e-88
XM_007977018.1	-0.4028	0.002107	0.01441	sp|Q71TY3|RS27_RAT 40S ribosomal protein S27 OS=Rattus norvegicus GN=Rps27 PE=2 SV=3//4.40983e-45
XM_007977084.1	-1.5182	3.80E-64	8.23E-62	sp|P26447|S10A4_HUMAN Protein S100-A4 OS=Homo sapiens GN=S100A4 PE=1 SV=1//1.96395e-68
XM_007977085.1	-1.0645	1.05E-30	7.22E-29	sp|P06703|S10A6_HUMAN Protein S100-A6 OS=Homo sapiens GN=S100A6 PE=1 SV=1//1.0985e-46
XM_007977141.1	-2.575	0.001627	0.011449	sp|Q07283|TRHY_HUMAN Trichohyalin OS=Homo sapiens GN=TCHH PE=1 SV=2//2.21233e-06
XM_007977146.1	-1.1281	6.57E-30	4.38E-28	sp|P31949|S10AB_HUMAN Protein S100-A11 OS=Homo sapiens GN=S100A11 PE=1 SV=2//5.9555e-71
XM_007977182.1	-1.2445	0.005751	0.035473	sp|Q52LC2|VAS1L_HUMAN V-type proton ATPase subunit S1-like protein OS=Homo sapiens GN=ATP6AP1L PE=2 SV=1//5.56713e-103
XM_007977187.1	-1.3208	0.000154	0.001322	sp|Q96L92|SNX27_HUMAN Sorting nexin-27 OS=Homo sapiens GN=SNX27 PE=1 SV=2//0
XM_007977193.1	1.5891	1.08E-70	2.78E-68	sp|A9X1A5|CING_PAPAN Cingulin OS=Papio anubis GN=CGN PE=3 SV=1//0
XM_007977194.1	-0.62899	1.06E-06	1.28E-05	sp|P62268|RS23_RAT 40S ribosomal protein S23 OS=Rattus norvegicus GN=Rps23 PE=1 SV=3//4.69482e-99
XM_007977202.1	-0.66488	2.06E-14	5.38E-13	sp|P28070|PSB4_HUMAN Proteasome subunit beta type-4 OS=Homo sapiens GN=PSMB4 PE=1 SV=4//0
XM_007977215.1	0.89114	5.27E-19	1.93E-17	sp|Q8N1G0|ZN687_HUMAN Zinc finger protein 687 OS=Homo sapiens GN=ZNF687 PE=1 SV=1//0
XM_007977226.1	-0.41637	4.42E-06	4.86E-05	sp|P55036|PSMD4_HUMAN 26S proteasome non-ATPase regulatory subunit 4 OS=Homo sapiens GN=PSMD4 PE=1 SV=1//0
XM_007977281.1	1.3105	4.61E-72	1.25E-69	sp|Q9H3M7|TXNIP_HUMAN Thioredoxin-interacting protein OS=Homo sapiens GN=TXNIP PE=1 SV=1//0
XM_007977284.1	-0.95311	3.75E-13	8.93E-12	sp|Q8IVB5|LIX1L_HUMAN LIX1-like protein OS=Homo sapiens GN=LIX1L PE=2 SV=1//0
XM_007977333.1	1.0693	3.18E-40	3.21E-38	sp|Q04721|NOTC2_HUMAN Neurogenic locus notch homolog protein 2 OS=Homo sapiens GN=NOTCH2 PE=1 SV=3//0
XM_007977337.1	0.32418	0.003957	0.025415	sp|Q5VU43|MYOME_HUMAN Myomegalin OS=Homo sapiens GN=PDE4DIP PE=1 SV=1//1.17059e-16
XM_007977338.1	0.78123	1.73E-06	2.01E-05	sp|Q9H094|NBPF3_HUMAN Neuroblastoma breakpoint family member 3 OS=Homo sapiens GN=NBPF3 PE=2 SV=1//1.9622e-16

XM_007977352.1	0.93611	0.008093	0.047819	sp|Q6P3W6|NBPFA_HUMAN Neuroblastoma breakpoint family member 10 OS=Homo sapiens GN=NBPF10 PE=2 SV=2//2.24276e-89
XM_007977354.1	0.93555	0.004185	0.026709	sp|Q3BBV0|NBPF1_HUMAN Neuroblastoma breakpoint family member 1 OS=Homo sapiens GN=NBPF1 PE=2 SV=1//1.29656e-83
XM_007977392.1	-0.99175	3.05E-15	8.49E-14	sp|P13611|CSPG2_HUMAN Versican core protein OS=Homo sapiens GN=VCAN PE=1 SV=3//0
XM_007977424.1	-1.0581	1.24E-33	9.75E-32	sp|Q9P2B2|FPRP_HUMAN Prostaglandin F2 receptor negative regulator OS=Homo sapiens GN=PTGFRN PE=1 SV=2//0
XM_007977481.1	0.32777	0.004861	0.030612	sp|Q9D287|SPF27_MOUSE Pre-mRNA-splicing factor SPF27 OS=Mus musculus GN=Bcas2 PE=2 SV=1//1.82666e-156
XM_007977492.1	-0.82964	4.90E-10	8.65E-09	sp|Q9NRN5|OLFL3_HUMAN Olfactomedin-like protein 3 OS=Homo sapiens GN=OLFML3 PE=2 SV=1//0
XM_007977504.1	0.91011	3.46E-10	6.22E-09	sp|Q9H816|DCR1B_HUMAN 5' exonuclease Apollo OS=Homo sapiens GN=DCLRE1B PE=1 SV=1//0
XM_007977505.1	0.68921	9.46E-12	1.97E-10	sp|Q5VWQ0|RSBN1_HUMAN Round spermatid basic protein 1 OS=Homo sapiens GN=RSBN1 PE=1 SV=2//0
XM_007977585.1	0.83074	1.07E-11	2.23E-10	sp|Q9BQA1|MEP50_HUMAN Methylosome protein 50 OS=Homo sapiens GN=WDR77 PE=1 SV=1//0
XM_007977679.1	-1.9016	0.004394	0.027882	sp|Q01433|AMPD2_HUMAN AMP deaminase 2 OS=Homo sapiens GN=AMPD2 PE=1 SV=2//0
XM_007977682.1	-0.23527	0.006844	0.041294	sp|P08754|GNAI3_HUMAN Guanine nucleotide-binding protein G(k) subunit alpha OS=Homo sapiens GN=GNAI3 PE=1 SV=3//0
XM_007977719.1	-0.57109	3.85E-14	9.86E-13	sp|Q9HCU4|CELR2_HUMAN Cadherin EGF LAG seven-pass G-type receptor 2 OS=Homo sapiens GN=CELSR2 PE=1 SV=1//0
XM_007977722.1	0.59521	0.00018	0.001526	sp|Q8NDZ6|T161B_HUMAN Transmembrane protein 161B OS=Homo sapiens GN=TMEM161B PE=2 SV=1//0
XM_007977725.1	0.88569	9.81E-18	3.32E-16	sp|Q5RAU6|KISHB_PONAB Protein kish-B OS=Pongo abelii GN=TMEM167B PE=3 SV=1//1.04849e-39
XM_007977728.1	0.97484	0.000893	0.006653	sp|Q5R9W6|TAF13_PONAB Transcription initiation factor TFIID subunit 13 OS=Pongo abelii GN=TAF13 PE=2 SV=1//6.87922e-65
XM_007977757.1	1.4333	3.13E-67	7.61E-65	sp|Q5VTL8|PR38B_HUMAN Pre-mRNA-splicing factor 38B OS=Homo sapiens GN=PRPF38B PE=1 SV=1//9.76558e-10
XM_007977769.1	0.81621	1.05E-22	4.90E-21	sp|Q6NUK1|SCMC1_HUMAN Calcium-binding mitochondrial carrier protein SCaMC-1 OS=Homo sapiens GN=SLC25A24 PE=1 SV=2//0
XM_007977770.1	-1.9693	2.43E-06	2.77E-05	sp|Q9UKW4|VAV3_HUMAN Guanine nucleotide exchange factor VAV3 OS=Homo sapiens GN=VAV3 PE=1 SV=1//0
XM_007977771.1	-0.75508	1.09E-21	4.70E-20	sp|Q9UKW4|VAV3_HUMAN Guanine nucleotide exchange factor VAV3 OS=Homo sapiens GN=VAV3 PE=1 SV=1//0
XM_007977793.1	-1.3026	0.000591	0.004577	sp|P12107|COBA1_HUMAN Collagen alpha-1(XI) chain OS=Homo sapiens GN=COL11A1 PE=1 SV=4//4.43011e-06
XM_007977794.1	-1.4171	2.32E-06	2.65E-05	sp|P12107|COBA1_HUMAN Collagen alpha-1(XI) chain OS=Homo sapiens GN=COL11A1 PE=1 SV=4//4.53176e-06
XM_007977823.1	0.8723	4.37E-08	6.14E-07	sp|Q9NUP7|TRM13_HUMAN tRNA:m(4)X modification enzyme TRM13 homolog OS=Homo sapiens GN=TRMT13 PE=1 SV=2//0
XM_007977824.1	1.7092	0.000427	0.003405	sp|Q96DD0|LRC39_HUMAN Leucine-rich repeat-containing protein 39 OS=Homo sapiens GN=LRRC39 PE=2 SV=1//0
XM_007977839.1	-0.79542	4.27E-10	7.62E-09	sp|P70187|HIAT1_MOUSE Hippocampus abundant transcript 1 protein OS=Mus musculus GN=Hiat1 PE=2 SV=3//0
XM_007977851.1	-2.682	0.002666	0.017735	sp|Q32ZL2|LPPR5_HUMAN Lipid phosphate phosphatase-related protein type 5 OS=Homo sapiens GN=LPPR5 PE=2 SV=2//0
XM_007977882.1	-0.86705	5.64E-09	8.86E-08	sp|Q96MV1|TMM56_HUMAN Transmembrane protein 56 OS=Homo sapiens GN=TMEM56 PE=1 SV=1//1.64554e-164
XM_007977899.1	1.6764	6.31E-17	2.01E-15	sp|P13726|TF_HUMAN Tissue factor OS=Homo sapiens GN=F3 PE=1 SV=1//6.79728e-170
XM_007977907.1	0.43958	3.25E-07	4.13E-06	sp|P78363|ABCA4_HUMAN Retinal-specific ATP-binding cassette transporter OS=Homo sapiens GN=ABCA4 PE=1 SV=3//0
XM_007977925.1	0.43425	2.86E-07	3.66E-06	sp|Q91WV0|NC2B_MOUSE Protein Dr1 OS=Mus musculus GN=Dr1 PE=2 SV=1//2.96231e-71
XM_007978020.1	0.90894	2.56E-16	7.75E-15	sp|Q5RCA4|ZN326_PONAB DBIRD complex subunit ZNF326 OS=Pongo abelii GN=ZNF326 PE=2 SV=1//0
XM_007978024.1	1.2883	0.006102	0.037341	sp|Q7L1W4|LRC8D_HUMAN Volume-regulated anion channel subunit LRRC8D OS=Homo sapiens GN=LRRC8D PE=1 SV=1//0
XM_007978033.1	0.55689	0.000177	0.001507	sp|Q7Z3D4|LYSM3_HUMAN LysM and putative peptidoglycan-binding domain-containing protein 3 OS=Homo sapiens GN=LYSMD3 PE=1 SV=2//0
XM_007978040.1	0.84525	0.001664	0.011664	sp|P32456|GBP2_HUMAN Interferon-induced guanylate-binding protein 2 OS=Homo sapiens GN=GBP2 PE=1 SV=3//0
XM_007978049.1	0.29731	0.004144	0.026498	sp|Q96E39|RMXL1_HUMAN RNA binding motif protein, X-linked-like-1 OS=Homo sapiens GN=RBMXL1 PE=1 SV=1//4.40365e-115
XM_007978050.1	-0.49049	0.001469	0.010459	sp|Q5R886|TF2B_PONAB Transcription initiation factor IIB OS=Pongo abelii GN=GTF2B PE=2 SV=1//0
XM_007978058.1	-0.97326	8.74E-06	9.12E-05	-//-

XM_007978062.1	-0.45166	2.64E-05	0.000257	sp|Q7LGA3|HS2ST_HUMAN Heparan sulfate 2-O-sulfotransferase 1 OS=Homo sapiens GN=HS2ST1 PE=1 SV=1//0
XM_007978072.1	1.2712	3.80E-15	1.05E-13	sp|Q68D91|MBLC2_HUMAN Metallo-beta-lactamase domain-containing protein 2 OS=Homo sapiens GN=MBLAC2 PE=2 SV=3//1.24894e-178
XM_007978090.1	1.6879	3.26E-20	1.28E-18	sp|Q9NWK9|BCD1_HUMAN Box C/D snoRNA protein 1 OS=Homo sapiens GN=ZNHIT6 PE=1 SV=1//0
XM_007978100.1	2.6843	########	########	sp|O00622|CYR61_HUMAN Protein CYR61 OS=Homo sapiens GN=CYR61 PE=1 SV=1//0
XM_007978101.1	0.83649	5.17E-12	1.10E-10	sp|O95999|BCL10_HUMAN B-cell lymphoma/leukemia 10 OS=Homo sapiens GN=BCL10 PE=1 SV=1//1.65938e-112
XM_007978102.1	0.96889	1.20E-11	2.49E-10	sp|Q8N6N3|CA052_HUMAN UPF0690 protein C1orf52 OS=Homo sapiens GN=C1orf52 PE=1 SV=1//2.83126e-97
XM_007978104.1	0.95417	2.31E-06	2.64E-05	sp|Q5VT97|SYDE2_HUMAN Rho GTPase-activating protein SYDE2 OS=Homo sapiens GN=SYDE2 PE=1 SV=2//0
XM_007978165.1	0.67463	0.004525	0.028635	sp|Q6ZT98|TTLL7_HUMAN Tubulin polyglutamylase TTLL7 OS=Homo sapiens GN=TTLL7 PE=2 SV=2//0
XM_007978169.1	-0.92885	0.003273	0.02139	sp|O97817|AGRL2_BOVIN Adhesion G protein-coupled receptor L2 OS=Bos taurus GN=ADGRL2 PE=2 SV=1//0
XM_007978199.1	Inf	8.01E-07	9.80E-06	sp|Q53G44|IF44L_HUMAN Interferon-induced protein 44-like OS=Homo sapiens GN=IFI44L PE=2 SV=3//0
XM_007978205.1	0.54359	1.91E-07	2.49E-06	sp|Q9UDY4|DNJB4_HUMAN DnaJ homolog subfamily B member 4 OS=Homo sapiens GN=DNAJB4 PE=1 SV=1//0
XM_007978225.1	0.75999	6.90E-06	7.32E-05	sp|Q8NAN2|FA73A_HUMAN Protein FAM73A OS=Homo sapiens GN=FAM73A PE=1 SV=1//0
XM_007978263.1	1.2745	4.19E-32	3.03E-30	sp|Q96B67|ARRD3_HUMAN Arrestin domain-containing protein 3 OS=Homo sapiens GN=ARRDC3 PE=1 SV=1//0
XM_007978289.1	-0.41311	0.00163	0.011466	sp|O14772|FPGT_HUMAN Fucose-1-phosphate guanylyltransferase OS=Homo sapiens GN=FPGT PE=1 SV=2//0
XM_007978338.1	2.7511	2.31E-08	3.36E-07	sp|Q16518|RPE65_HUMAN Retinoid isomerohydrolase OS=Homo sapiens GN=RPE65 PE=1 SV=3//0
XM_007978345.1	0.36867	0.000207	0.001744	sp|Q5RBQ0|GBG12_PONAB Guanine nucleotide-binding protein G(I)/G(S)/G(O) subunit gamma-12 OS=Pongo abelii GN=GNG12 PE=3 SV=3//1.03202e-39
XM_007978352.1	3.2653	########	########	sp|P24522|GA45A_HUMAN Growth arrest and DNA damage-inducible protein GADD45 alpha OS=Homo sapiens GN=GADD45A PE=1 SV=1//1.26262e-113
XM_007978374.1	0.56476	0.00479	0.030208	sp|Q5RDC9|S35D2_PONAB UDP-N-acetylglucosamine/UDP-glucose/GDP-mannose transporter OS=Pongo abelii GN=SLC35D2 PE=2 SV=1//0
XM_007978453.1	-0.31517	0.000195	0.001646	sp|P23458|JAK1_HUMAN Tyrosine-protein kinase JAK1 OS=Homo sapiens GN=JAK1 PE=1 SV=2//0
XM_007978456.1	-1.6454	2.09E-16	6.35E-15	sp|P10589|COT1_HUMAN COUP transcription factor 1 OS=Homo sapiens GN=NR2F1 PE=1 SV=1//0
XM_007978538.1	0.85707	1.18E-14	3.17E-13	sp|Q9UJC3|HOOK1_HUMAN Protein Hook homolog 1 OS=Homo sapiens GN=HOOK1 PE=1 SV=2//0
XM_007978554.1	3.9612	########	########	sp|P05627|JUN_MOUSE Transcription factor AP-1 OS=Mus musculus GN=Jun PE=1 SV=3//2.61244e-159
XM_007978593.1	0.90789	9.48E-18	3.21E-16	sp|P54646|AAPK2_HUMAN 5'-AMP-activated protein kinase catalytic subunit alpha-2 OS=Homo sapiens GN=PRKAA2 PE=1 SV=2//0
XM_007978594.1	0.77169	2.70E-17	8.92E-16	sp|O14495|LPP3_HUMAN Lipid phosphate phosphohydrolase 3 OS=Homo sapiens GN=PPAP2B PE=1 SV=1//0
XM_007978596.1	-0.99544	6.50E-44	7.34E-42	sp|Q6B7M7|COF1_SHEEP Cofilin-1 OS=Ovis aries GN=CFL1 PE=2 SV=3//9.43706e-112
XM_007978628.1	1.474	3.64E-34	2.91E-32	sp|O95801|TTC4_HUMAN Tetratricopeptide repeat protein 4 OS=Homo sapiens GN=TTC4 PE=1 SV=3//0
XM_007978662.1	-2.2128	0.002486	0.016669	sp|Q5VXM1|CDCP2_HUMAN CUB domain-containing protein 2 OS=Homo sapiens GN=CDCP2 PE=2 SV=1//0
XM_007978665.1	0.48274	0.002638	0.017596	sp|Q96MN5|TEAN2_HUMAN Transcription elongation factor A N-terminal and central domain-containing protein 2 OS=Homo sapiens GN=TCEANC2 PE=1 SV=1//1.34808e-140
XM_007978686.1	-0.40984	0.006389	0.038982	sp|P49895|IOD1_HUMAN Type I iodothyronine deiodinase OS=Homo sapiens GN=DIO1 PE=2 SV=3//9.10533e-152
XM_007978735.1	0.36651	0.00242	0.016287	sp|Q96BR5|COA7_HUMAN Cytochrome c oxidase assembly factor 7 OS=Homo sapiens GN=COA7 PE=1 SV=2//1.34971e-162
XM_007978752.1	0.29478	0.001607	0.011333	sp|Q5RDD2|PR38A_PONAB Pre-mRNA-splicing factor 38A OS=Pongo abelii GN=PRPF38A PE=2 SV=1//3.22955e-136
XM_007978755.1	0.84904	4.90E-05	0.000458	sp|Q13415|ORC1_HUMAN Origin recognition complex subunit 1 OS=Homo sapiens GN=ORC1 PE=1 SV=2//0
XM_007978758.1	0.84416	1.17E-08	1.77E-07	sp|Q96EK9|KTI12_HUMAN Protein KTI12 homolog OS=Homo sapiens GN=KTI12 PE=1 SV=1//0
XM_007978759.1	-0.4086	0.000323	0.002631	sp|O95881|TXD12_HUMAN Thioredoxin domain-containing protein 12 OS=Homo sapiens GN=TXNDC12 PE=1 SV=1//7.72207e-117
XM_007978763.1	-1.2809	0.000562	0.00437	sp|P20337|RAB3B_HUMAN Ras-related protein Rab-3B OS=Homo sapiens GN=RAB3B PE=1 SV=2//3.80658e-159

XM_007978764.1	-3.7066	0.000379	0.003052	sp|O43847|NRDC_HUMAN Nardilysin OS=Homo sapiens GN=NRD1 PE=1 SV=2//6.7452e-71
XM_007978781.1	0.67084	5.63E-15	1.54E-13	sp|Q9Y3C5|RNF11_HUMAN RING finger protein 11 OS=Homo sapiens GN=RNF11 PE=1 SV=1//6.45029e-83
XM_007978784.1	-0.729	1.60E-12	3.58E-11	sp|P42773|CDN2C_HUMAN Cyclin-dependent kinase 4 inhibitor C OS=Homo sapiens GN=CDKN2C PE=1 SV=1//6.39344e-98
XM_007978832.1	-1.2645	9.77E-15	2.64E-13	sp|A6NFA1|TIKI2_HUMAN Metalloprotease TIKI2 OS=Homo sapiens GN=TRABD2B PE=1 SV=2//0
XM_007978833.1	-0.89245	1.15E-06	1.38E-05	sp|A7XV07|SKIT8_MOUSE Selection and upkeep of intraepithelial T-cells protein 8 OS=Mus musculus GN=Skint8 PE=2 SV=2//3.59903e-75
XM_007978837.1	-0.25798	0.006167	0.037687	sp|P30085|KCY_HUMAN UMP-CMP kinase OS=Homo sapiens GN=CMPK1 PE=1 SV=3//5.44429e-132
XM_007978851.1	-0.94739	3.52E-35	2.99E-33	sp|P62752|RL23A_RAT 60S ribosomal protein L23a OS=Rattus norvegicus GN=Rpl23a PE=2 SV=1//5.39372e-85
XM_007978869.1	0.89207	0.000496	0.003901	sp|Q70IA8|MOB3C_HUMAN MOB kinase activator 3C OS=Homo sapiens GN=MOB3C PE=1 SV=1//5.11148e-152
XM_007978923.1	0.75059	9.10E-18	3.09E-16	sp|O94955|RHBT3_HUMAN Rho-related BTB domain-containing protein 3 OS=Homo sapiens GN=RHOBTB3 PE=1 SV=2//0
XM_007978940.1	0.47715	1.16E-05	0.000118	sp|Q5SWH9|TMM69_HUMAN Transmembrane protein 69 OS=Homo sapiens GN=TMEM69 PE=2 SV=1//8.46759e-160
XM_007978941.1	0.39289	5.05E-06	5.50E-05	sp|Q5R9C3|GPBL1_PONAB Vasculin-like protein 1 OS=Pongo abelii GN=GPBP1L1 PE=2 SV=1//0
XM_007978955.1	-0.77	4.94E-18	1.71E-16	sp|Q06830|PRDX1_HUMAN Peroxiredoxin-1 OS=Homo sapiens GN=PRDX1 PE=1 SV=1//1.04714e-142
XM_007978973.1	2.3156	0.000806	0.006059	sp|O00472|ELL2_HUMAN RNA polymerase II elongation factor ELL2 OS=Homo sapiens GN=ELL2 PE=1 SV=2//0
XM_007978981.1	1.9828	6.81E-13	1.58E-11	sp|Q9H4B4|PLK3_HUMAN Serine/threonine-protein kinase PLK3 OS=Homo sapiens GN=PLK3 PE=1 SV=2//0
XM_007979043.1	1.1905	0.003248	0.021234	sp|Q5RE82|DPH2_PONAB Diphthamide biosynthesis protein 2 OS=Pongo abelii GN=DPH2 PE=2 SV=1//0
XM_007979050.1	1.6077	9.85E-11	1.88E-09	sp|Q6P179|ERAP2_HUMAN Endoplasmic reticulum aminopeptidase 2 OS=Homo sapiens GN=ERAP2 PE=1 SV=2//0
XM_007979100.1	-0.41317	9.78E-07	1.19E-05	sp|Q12834|CDC20_HUMAN Cell division cycle protein 20 homolog OS=Homo sapiens GN=CDC20 PE=1 SV=2//0
XM_007979144.1	1.0513	1.55E-06	1.82E-05	sp|Q9BV19|CA050_HUMAN Uncharacterized protein C1orf50 OS=Homo sapiens GN=C1orf50 PE=1 SV=2//3.31945e-130
XM_007979207.1	1.2759	5.31E-14	1.35E-12	sp|P20800|EDN2_HUMAN Endothelin-2 OS=Homo sapiens GN=EDN2 PE=1 SV=2//4.68184e-105
XM_007979209.1	0.54308	3.87E-05	0.000367	sp|Q70KY4|FOXO6_MOUSE Forkhead box protein O6 OS=Mus musculus GN=Foxo6 PE=1 SV=1//5.35517e-18
XM_007979228.1	2.4216	0.000321	0.002618	sp|P17812|PYRG1_HUMAN CTP synthase 1 OS=Homo sapiens GN=CTPS1 PE=1 SV=2//0
XM_007979229.1	2.9016	7.23E-05	0.00066	sp|P17812|PYRG1_HUMAN CTP synthase 1 OS=Homo sapiens GN=CTPS1 PE=1 SV=2//0
XM_007979281.1	-0.4666	4.25E-08	5.99E-07	sp|Q8HXW6|PPT1_MACFA Palmitoyl-protein thioesterase 1 OS=Macaca fascicularis GN=PPT1 PE=2 SV=1//0
XM_007979289.1	1.6402	1.81E-15	5.15E-14	sp|Q8NA29|NLS1_HUMAN Sodium-dependent lysophosphatidylcholine symporter 1 OS=Homo sapiens GN=MFSD2A PE=1 SV=1//0
XM_007979292.1	0.71452	2.85E-07	3.65E-06	sp|Q9H3H1|MOD5_HUMAN tRNA dimethylallyltransferase, mitochondrial OS=Homo sapiens GN=TRIT1 PE=1 SV=1//0
XM_007979294.1	-1.4143	0.002157	0.014725	sp|P34820|BMP8B_HUMAN Bone morphogenetic protein 8B OS=Homo sapiens GN=BMP8B PE=2 SV=2//0
XM_007979295.1	0.82015	1.18E-12	2.67E-11	sp|Q9BVS4|RIOK2_HUMAN Serine/threonine-protein kinase RIO2 OS=Homo sapiens GN=RIOK2 PE=1 SV=2//0
XM_007979302.1	-1.5855	0.000549	0.004277	sp|Q9BXI3|5NT1A_HUMAN Cytosolic 5'-nucleotidase 1A OS=Homo sapiens GN=NT5C1A PE=2 SV=1//0
XM_007979312.1	0.71221	1.08E-11	2.24E-10	sp|Q9HB90|RRAGC_HUMAN Ras-related GTP-binding protein C OS=Homo sapiens GN=RRAGC PE=1 SV=1//0
XM_007979313.1	-0.56221	7.79E-05	0.000708	sp|Q99417|MYCBP_HUMAN C-Myc-binding protein OS=Homo sapiens GN=MYCBP PE=1 SV=3//9.93098e-55
XM_007979320.1	0.62549	0.001861	0.012864	sp|Q9Y3A2|UTP11_HUMAN Probable U3 small nucleolar RNA-associated protein 11 OS=Homo sapiens GN=UTP11L PE=1 SV=2//5.64567e-166
XM_007979334.1	0.95388	2.16E-10	3.96E-09	sp|Q14872|MTF1_HUMAN Metal regulatory transcription factor 1 OS=Homo sapiens GN=MTF1 PE=1 SV=2//0
XM_007979361.1	0.61316	1.35E-08	2.03E-07	sp|Q5RBS5|BOREA_PONAB Borealin OS=Pongo abelii GN=CDCA8 PE=2 SV=1//0
XM_007979369.1	0.27901	0.00427	0.027182	sp|Q13823|NOG2_HUMAN Nucleolar GTP-binding protein 2 OS=Homo sapiens GN=GNL2 PE=1 SV=1//0
XM_007979370.1	0.86225	0.000179	0.001522	sp|Q8TAD8|SNIP1_HUMAN Smad nuclear-interacting protein 1 OS=Homo sapiens GN=SNIP1 PE=1 SV=1//3.86142e-153
XM_007979382.1	-0.55134	1.33E-09	2.24E-08	sp|P82914|RT15_HUMAN 28S ribosomal protein S15, mitochondrial OS=Homo sapiens GN=MRPS15 PE=1 SV=1//2.53259e-118
XM_007979390.1	-0.8321	3.10E-14	7.96E-13	sp|Q9NVM1|EVA1B_HUMAN Protein eva-1 homolog B OS=Homo sapiens GN=EVA1B

				PE=1 SV=1//6.31906e-49
XM_007979432.1	0.68416	0.001378	0.009855	sp|Q9HCK5|AGO4_HUMAN Protein argonaute-4 OS=Homo sapiens GN=AGO4 PE=1 SV=2//0
XM_007979458.1	-0.33808	0.000534	0.00417	sp|P23246|SFPQ_HUMAN Splicing factor, proline- and glutamine-rich OS=Homo sapiens GN=SFPQ PE=1 SV=2//0
XM_007979540.1	1.4078	7.22E-62	1.52E-59	sp|P54577|SYYC_HUMAN Tyrosine--tRNA ligase, cytoplasmic OS=Homo sapiens GN=YARS PE=1 SV=4//0
XM_007979564.1	-0.52667	2.77E-05	0.000269	sp|Q8IWT0|ARCH_HUMAN Protein archease OS=Homo sapiens GN=ZBTB8OS PE=1 SV=2//6.24004e-118
XM_007979602.1	-0.4766	2.25E-08	3.27E-07	sp|Q13347|EIF3I_HUMAN Eukaryotic translation initiation factor 3 subunit I OS=Homo sapiens GN=EIF3I PE=1 SV=1//0
XM_007979612.1	-0.33603	9.17E-06	9.53E-05	sp|Q07666|KHDR1_HUMAN KH domain-containing, RNA-binding, signal transduction-associated protein 1 OS=Homo sapiens GN=KHDRBS1 PE=1 SV=1//5.69318e-175
XM_007979615.1	-1.0991	3.17E-18	1.11E-16	sp|Q9BV90|SNR25_HUMAN U11/U12 small nuclear ribonucleoprotein 25 kDa protein OS=Homo sapiens GN=SNRNP25 PE=1 SV=1//3.3363e-86
XM_007979663.1	-0.68346	1.62E-06	1.90E-05	sp|P05413|FABPH_HUMAN Fatty acid-binding protein, heart OS=Homo sapiens GN=FABP3 PE=1 SV=4//2.11854e-88
XM_007979717.1	0.48268	7.28E-09	1.12E-07	sp|Q8VE97|SRSF4_MOUSE Serine/arginine-rich splicing factor 4 OS=Mus musculus GN=Srsf4 PE=2 SV=1//4.40387e-92
XM_007979750.1	0.79487	5.89E-19	2.15E-17	sp|Q9Y5A9|YTHD2_HUMAN YTH domain-containing family protein 2 OS=Homo sapiens GN=YTHDF2 PE=1 SV=2//0
XM_007979760.1	-0.37565	3.11E-06	3.50E-05	sp|Q9H0X4|ITFG3_HUMAN Protein ITFG3 OS=Homo sapiens GN=ITFG3 PE=1 SV=1//0
XM_007979784.1	2.3271	########	########	sp|P58004|SESN2_HUMAN Sestrin-2 OS=Homo sapiens GN=SESN2 PE=1 SV=1//0
XM_007979785.1	-1.0765	6.55E-08	9.00E-07	sp|Q5RFJ9|ATIF1_PONAB ATPase inhibitor, mitochondrial OS=Pongo abelii GN=ATPIF1 PE=3 SV=1//3.23986e-46
XM_007979803.1	1.603	7.13E-42	7.51E-40	sp|P15927|RFA2_HUMAN Replication protein A 32 kDa subunit OS=Homo sapiens GN=RPA2 PE=1 SV=1//5.38929e-177
XM_007979809.1	0.44303	0.001191	0.008645	sp|Q86Y82|STX12_HUMAN Syntaxin-12 OS=Homo sapiens GN=STX12 PE=1 SV=1//9.77769e-150
XM_007979812.1	2.0335	4.61E-42	4.92E-40	sp|Q28808|IFI6_PANTR Interferon alpha-inducible protein 6 OS=Pan troglodytes GN=IFI6 PE=2 SV=1//1.49884e-54
XM_007979819.1	-1.3852	0.000499	0.003922	sp|Q9NQ29|LUC7L_HUMAN Putative RNA-binding protein Luc7-like 1 OS=Homo sapiens GN=LUC7L PE=1 SV=1//2.7248e-10
XM_007979853.1	1.7959	1.13E-44	1.31E-42	sp|Q96A09|FA46B_HUMAN Protein FAM46B OS=Homo sapiens GN=FAM46B PE=1 SV=2//0
XM_007979865.1	0.71581	0.000529	0.004134	sp|Q96I76|GPTC3_HUMAN G patch domain-containing protein 3 OS=Homo sapiens GN=GPATCH3 PE=2 SV=1//0
XM_007979902.1	0.5624	0.005451	0.03385	sp|O00488|ZN593_HUMAN Zinc finger protein 593 OS=Homo sapiens GN=ZNF593 PE=1 SV=2//1.1837e-73
XM_007979921.1	-1.1082	2.02E-09	3.32E-08	sp|Q9H299|SH3L3_HUMAN SH3 domain-binding glutamic acid-rich-like protein 3 OS=Homo sapiens GN=SH3BGRL3 PE=1 SV=1//7.02492e-57
XM_007979940.1	-0.54729	2.49E-09	4.07E-08	sp|Q86SG4|DPCA2_HUMAN Putative Dresden prostate carcinoma protein 2 OS=Homo sapiens GN=HMGN2P46 PE=5 SV=1//2.33391e-26
XM_007979974.1	-1.2438	3.56E-27	2.10E-25	sp|Q9NZV5|SELN_HUMAN Selenoprotein N OS=Homo sapiens GN=SEPN1 PE=1 SV=5//0
XM_007979979.1	-2.1082	9.11E-31	6.27E-29	sp|Q4R626|RSRP1_MACFA Arginine/serine-rich protein 1 OS=Macaca fascicularis GN=RSRP1 PE=2 SV=1//1.38076e-68
XM_007980024.1	-0.44135	8.62E-07	1.05E-05	sp|O43290|SNUT1_HUMAN U4/U6.U5 tri-snRNP-associated protein 1 OS=Homo sapiens GN=SART1 PE=1 SV=1//0
XM_007980049.1	1.2688	0.002079	0.014229	sp|Q9R0U0|SRS10_MOUSE Serine/arginine-rich splicing factor 10 OS=Mus musculus GN=Srsf10 PE=1 SV=2//3.43239e-36
XM_007980051.1	1.7117	3.93E-44	4.51E-42	sp|Q9NPJ4|PNRC2_HUMAN Proline-rich nuclear receptor coactivator 2 OS=Homo sapiens GN=PNRC2 PE=1 SV=1//2.93317e-75
XM_007980070.1	0.43939	8.44E-05	0.000762	sp|Q14241|ELOA1_HUMAN Transcription elongation factor B polypeptide 3 OS=Homo sapiens GN=TCEB3 PE=1 SV=2//0
XM_007980071.1	1.5942	1.35E-20	5.51E-19	sp|Q9GZP4|PITH1_HUMAN PITH domain-containing protein 1 OS=Homo sapiens GN=PITHD1 PE=1 SV=1//1.47958e-143
XM_007980073.1	-0.67485	1.53E-10	2.88E-09	sp|P62914|RL11_RAT 60S ribosomal protein L11 OS=Rattus norvegicus GN=Rpl11 PE=1 SV=2//1.86101e-128
XM_007980074.1	2.0499	########	########	sp|Q02535|ID3_HUMAN DNA-binding protein inhibitor ID-3 OS=Homo sapiens GN=ID3 PE=1 SV=2//2.03109e-65
XM_007980075.1	0.82424	0.000221	0.00185	sp|Q14209|E2F2_HUMAN Transcription factor E2F2 OS=Homo sapiens GN=E2F2 PE=1 SV=1//0
XM_007980091.1	0.47012	0.00013	0.001125	sp|Q5R5Y7|ZN436_PONAB Zinc finger protein 436 OS=Pongo abelii GN=ZNF436 PE=2 SV=1//0
XM_007980092.1	1.1507	3.01E-05	0.00029	sp|P28221|5HT1D_HUMAN 5-hydroxytryptamine receptor 1D OS=Homo sapiens GN=HTR1D PE=1 SV=1//0
XM_007980093.1	-0.80799	0.001839	0.012733	sp|O00746|NDKM_HUMAN Nucleoside diphosphate kinase, mitochondrial OS=Homo sapiens GN=NME4 PE=1 SV=1//8.27531e-124
XM_007980094.1	1.0666	3.80E-36	3.33E-34	sp|Q86V48|LUZP1_HUMAN Leucine zipper protein 1 OS=Homo sapiens GN=LUZP1

				PE=1 SV=2//0
XM_007980113.1	0.86796	2.95E-08	4.22E-07	sp|Q9NUA8|ZBT40_HUMAN Zinc finger and BTB domain-containing protein 40 OS=Homo sapiens GN=ZBTB40 PE=1 SV=4//0
XM_007980209.1	1.1292	6.55E-17	2.08E-15	sp|Q969V5|MUL1_HUMAN Mitochondrial ubiquitin ligase activator of NFKB 1 OS=Homo sapiens GN=MUL1 PE=1 SV=1//0
XM_007980211.1	-1.0886	0.000698	0.005323	sp|Q6ZT52|FA43B_HUMAN Protein FAM43B OS=Homo sapiens GN=FAM43B PE=2 SV=1//9.14206e-163
XM_007980213.1	-0.99879	1.06E-14	2.85E-13	sp|Q7Z7J9|CK2N1_HUMAN Calcium/calmodulin-dependent protein kinase II inhibitor 1 OS=Homo sapiens GN=CAMK2N1 PE=1 SV=1//3.26965e-35
XM_007980270.1	2.2674	0.002682	0.017827	sp|Q8NHP1|ARK74_HUMAN Aflatoxin B1 aldehyde reductase member 4 OS=Homo sapiens GN=AKR7L PE=2 SV=6//0
XM_007980272.1	0.75175	1.87E-07	2.44E-06	sp|Q9UKD2|MRT4_HUMAN mRNA turnover protein 4 homolog OS=Homo sapiens GN=MRTO4 PE=1 SV=2//1.08996e-149
XM_007980292.1	0.65234	4.89E-07	6.12E-06	sp|Q9QYT7|PIGQ_MOUSE Phosphatidylinositol N-acetylglucosaminyltransferase subunit Q OS=Mus musculus GN=Pigq PE=2 SV=3//0
XM_007980293.1	1.034	0.000556	0.004322	sp|Q5VTJ3|KLD7A_HUMAN Kelch domain-containing protein 7A OS=Homo sapiens GN=KLHDC7A PE=1 SV=5//0
XM_007980323.1	-0.57064	1.55E-06	1.82E-05	sp|P21912|SDHB_HUMAN Succinate dehydrogenase [ubiquinone] iron-sulfur subunit, mitochondrial OS=Homo sapiens GN=SDHB PE=1 SV=3//0
XM_007980358.1	-0.67684	1.79E-12	3.97E-11	sp|Q14919|NC2A_HUMAN Dr1-associated corepressor OS=Homo sapiens GN=DRAP1 PE=1 SV=3//3.11204e-72
XM_007980431.1	1.571	3.00E-32	2.19E-30	sp|Q92681|RSCA1_HUMAN Regulatory solute carrier protein family 1 member 1 OS=Homo sapiens GN=RSC1A1 PE=2 SV=1//0
XM_007980444.1	0.78534	5.68E-17	1.82E-15	sp|Q96C19|EFHD2_HUMAN EF-hand domain-containing protein D2 OS=Homo sapiens GN=EFHD2 PE=1 SV=1//3.73486e-113
XM_007980506.1	1.3983	0.006524	0.039683	sp|P20333|TNR1B_HUMAN Tumor necrosis factor receptor superfamily member 1B OS=Homo sapiens GN=TNFRSF1B PE=1 SV=3//0
XM_007980528.1	3.1703	3.73E-21	1.57E-19	sp|P16860|ANFB_HUMAN Natriuretic peptides B OS=Homo sapiens GN=NPPB PE=1 SV=1//6.36805e-71
XM_007980533.1	0.83539	4.49E-10	7.98E-09	sp|P51797|CLCN6_HUMAN Chloride transport protein 6 OS=Homo sapiens GN=CLCN6 PE=1 SV=2//0
XM_007980558.1	-1.2288	8.79E-07	1.07E-05	sp|Q9UK22|FBX2_HUMAN F-box only protein 2 OS=Homo sapiens GN=FBXO2 PE=1 SV=2//7.57174e-155
XM_007980567.1	0.89555	4.47E-13	1.06E-11	sp|Q8CFJ9|WDR24_MOUSE WD repeat-containing protein 24 OS=Mus musculus GN=Wdr24 PE=2 SV=1//0
XM_007980571.1	1.1864	1.36E-05	0.000137	sp|Q9Y5Z9|UBIA1_HUMAN UbiA prenyltransferase domain-containing protein 1 OS=Homo sapiens GN=UBIAD1 PE=1 SV=1//0
XM_007980584.1	-1.3592	1.08E-08	1.64E-07	sp|O00187|MASP2_HUMAN Mannan-binding lectin serine protease 2 OS=Homo sapiens GN=MASP2 PE=1 SV=4//0
XM_007980585.1	-0.6184	7.08E-08	9.71E-07	sp|Q13148|TADBP_HUMAN TAR DNA-binding protein 43 OS=Homo sapiens GN=TARDBP PE=1 SV=1//0
XM_007980615.1	0.43221	0.000838	0.006279	sp|O43896|KIF1C_HUMAN Kinesin-like protein KIF1C OS=Homo sapiens GN=KIF1C PE=1 SV=3//0
XM_007980639.1	1.752	9.47E-15	2.57E-13	sp|Q96BD6|SPSB1_HUMAN SPRY domain-containing SOCS box protein 1 OS=Homo sapiens GN=SPSB1 PE=1 SV=1//0
XM_007980643.1	0.54124	0.000157	0.001349	sp|Q5SNT2|TM201_HUMAN Transmembrane protein 201 OS=Homo sapiens GN=TMEM201 PE=1 SV=1//0
XM_007980646.1	-1.7534	7.64E-23	3.59E-21	sp|O95479|G6PE_HUMAN GDH/6PGL endoplasmic bifunctional protein OS=Homo sapiens GN=H6PD PE=1 SV=2//0
XM_007980661.1	-0.78723	1.68E-29	1.10E-27	sp|P06733|ENOA_HUMAN Alpha-enolase OS=Homo sapiens GN=ENO1 PE=1 SV=2//0
XM_007980677.1	2.19	1.32E-68	3.33E-66	sp|Q9UJM3|ERRFI_HUMAN ERBB receptor feedback inhibitor 1 OS=Homo sapiens GN=ERRFI1 PE=1 SV=1//0
XM_007980702.1	-0.43678	1.82E-05	0.00018	sp|Q15836|VAMP3_HUMAN Vesicle-associated membrane protein 3 OS=Homo sapiens GN=VAMP3 PE=1 SV=3//3.78777e-46
XM_007980735.1	1.2555	4.72E-21	1.97E-19	sp|Q9UJP4|KLH21_HUMAN Kelch-like protein 21 OS=Homo sapiens GN=KLHL21 PE=1 SV=4//0
XM_007980769.1	-2.2311	0.005672	0.03504	sp|Q13421|MSLN_HUMAN Mesothelin OS=Homo sapiens GN=MSLN PE=1 SV=2//0
XM_007980773.1	0.99714	1.02E-08	1.55E-07	sp|Q9Y543|HES2_HUMAN Transcription factor HES-2 OS=Homo sapiens GN=HES2 PE=2 SV=1//4.23579e-78
XM_007980781.1	-1.2451	1.06E-36	9.40E-35	sp|Q6NV75|GP153_HUMAN Probable G-protein coupled receptor 153 OS=Homo sapiens GN=GPR153 PE=2 SV=2//0
XM_007980797.1	-0.57239	3.85E-06	4.26E-05	sp|Q6ZRF8|RN207_HUMAN RING finger protein 207 OS=Homo sapiens GN=RNF207 PE=2 SV=2//0
XM_007980886.1	-0.40611	3.11E-06	3.50E-05	sp|O46431|FIBP_CHLAE Acidic fibroblast growth factor intracellular-binding protein OS=Chlorocebus aethiops GN=FIBP PE=2 SV=2//0
XM_007980991.1	-0.8482	1.69E-14	4.50E-13	sp|Q6PCB0|VWA1_HUMAN von Willebrand factor A domain-containing protein 1 OS=Homo sapiens GN=VWA1 PE=2 SV=1//0
XM_007981002.1	-1.1248	1.72E-08	2.54E-07	sp|Q96S94|CCNL2_HUMAN Cyclin-L2 OS=Homo sapiens GN=CCNL2 PE=1 SV=1//1.24115e-128
XM_007981053.1	0.86756	1.25E-09	2.11E-08	sp|Q96L58|B3GT6_HUMAN Beta-1,3-galactosyltransferase 6 OS=Homo sapiens

				GN=B3GALT6 PE=1 SV=2//8.13911e-152
XM_007981089.1	2.038	2.07E-22	9.45E-21	sp|P05161|ISG15_HUMAN Ubiquitin-like protein ISG15 OS=Homo sapiens GN=ISG15 PE=1 SV=5//9.73105e-88
XM_007981094.1	-0.47607	1.86E-07	2.44E-06	sp|Q96NU1|SAM11_HUMAN Sterile alpha motif domain-containing protein 11 OS=Homo sapiens GN=SAMD11 PE=2 SV=3//0
XM_007981106.1	-1.7479	0.002362	0.015962	-//-
XM_007981120.1	-4.5638	0.000321	0.002614	sp|Q5VU13|VSIG8_HUMAN V-set and immunoglobulin domain-containing protein 8 OS=Homo sapiens GN=VSIG8 PE=2 SV=1//0
XM_007981125.1	-0.34293	0.000327	0.002663	sp|P49760|CLK2_HUMAN Dual specificity protein kinase CLK2 OS=Homo sapiens GN=CLK2 PE=1 SV=1//0
XM_007981136.1	0.92848	3.54E-22	1.59E-20	sp|Q4R4U9|SYSC_MACFA Serine--tRNA ligase, cytoplasmic OS=Macaca fascicularis GN=SARS PE=2 SV=3//6.7033e-22
XM_007981137.1	1.4514	1.07E-08	1.62E-07	sp|Q6UXG2|K1324_HUMAN UPF0577 protein KIAA1324 OS=Homo sapiens GN=KIAA1324 PE=2 SV=2//0
XM_007981144.1	-0.88209	9.75E-05	0.000869	sp|P70187|HIAT1_MOUSE Hippocampus abundant transcript 1 protein OS=Mus musculus GN=Hiat1 PE=2 SV=3//3.06517e-16
XM_007981174.1	0.94485	1.49E-16	4.60E-15	sp|O95789|ZMYM6_HUMAN Zinc finger MYM-type protein 6 OS=Homo sapiens GN=ZMYM6 PE=2 SV=2//0
XM_007981176.1	1.3157	6.03E-23	2.85E-21	sp|Q6ZMZ0|RN19B_HUMAN E3 ubiquitin-protein ligase RNF19B OS=Homo sapiens GN=RNF19B PE=1 SV=2//4.75993e-13
XM_007981177.1	-0.97913	1.95E-25	1.05E-23	-//-
XM_007981182.1	0.97366	6.36E-06	6.79E-05	sp|Q96I76|GPTC3_HUMAN G patch domain-containing protein 3 OS=Homo sapiens GN=GPATCH3 PE=2 SV=1//6.32097e-161
XM_007981183.1	0.60819	8.47E-06	8.86E-05	sp|Q86SQ9|DHDDS_HUMAN Dehydrodolichyl diphosphate syntase complex subunit DHDDS OS=Homo sapiens GN=DHDDS PE=1 SV=3//3.48949e-139
XM_007981188.1	-0.64926	1.43E-05	0.000144	sp|Q60HF8|FUCO_MACFA Tissue alpha-L-fucosidase OS=Macaca fascicularis GN=FUCA1 PE=2 SV=1//2.84271e-85
XM_007981198.1	1.1256	1.17E-05	0.00012	sp|O95154|ARK73_HUMAN Aflatoxin B1 aldehyde reductase member 3 OS=Homo sapiens GN=AKR7A3 PE=1 SV=2//0
XM_007981205.1	-0.61978	3.54E-06	3.95E-05	sp|Q5R9N3|PLOD1_PONAB Procollagen-lysine,2-oxoglutarate 5-dioxygenase 1 OS=Pongo abelii GN=PLOD1 PE=2 SV=1//1.16006e-25
XM_007981206.1	-0.39734	7.27E-06	7.68E-05	sp|P52209|6PGD_HUMAN 6-phosphogluconate dehydrogenase, decarboxylating OS=Homo sapiens GN=PGD PE=1 SV=3//0
XM_007981208.1	1.2009	0.000128	0.001113	sp|Q9HAN9|NMNA1_HUMAN Nicotinamide/nicotinic acid mononucleotide adenylyltransferase 1 OS=Homo sapiens GN=NMNAT1 PE=1 SV=1//3.98731e-132
XM_007981210.1	-0.33703	7.45E-05	0.000679	sp|Q28CQ4|UBC9_XENTR SUMO-conjugating enzyme UBC9 OS=Xenopus tropicalis GN=ube2i PE=2 SV=1//3.20078e-105
XM_007981214.1	0.6762	1.33E-07	1.77E-06	sp|Q5T9A4|ATD3B_HUMAN ATPase family AAA domain-containing protein 3B OS=Homo sapiens GN=ATAD3B PE=1 SV=1//0
XM_007981236.1	-0.83896	0.004202	0.026811	sp|Q9UII5|ZN107_HUMAN Zinc finger protein 107 OS=Homo sapiens GN=ZNF107 PE=2 SV=1//6.22407e-06
XM_007981250.1	-0.49207	1.66E-06	1.94E-05	sp|Q9Y6H1|CHCH2_HUMAN Coiled-coil-helix-coiled-coil-helix domain-containing protein 2 OS=Homo sapiens GN=CHCHD2 PE=1 SV=1//6.99256e-38
XM_007981280.1	2.8654	########	########	sp|P55245|EGFR_MACMU Epidermal growth factor receptor OS=Macaca mulatta GN=EGFR PE=2 SV=2//0
XM_007981281.1	-0.99606	3.79E-06	4.21E-05	sp|P60060|SC61G_MOUSE Protein transport protein Sec61 subunit gamma OS=Mus musculus GN=Sec61g PE=3 SV=1//1.28834e-18
XM_007981337.1	0.49795	0.002716	0.018041	sp|Q4G0I0|CSMT1_HUMAN Protein CCSMST1 OS=Homo sapiens GN=CCSMST1 PE=2 SV=1//2.44823e-41
XM_007981388.1	0.67125	4.60E-15	1.27E-13	sp|P17936|IBP3_HUMAN Insulin-like growth factor-binding protein 3 OS=Homo sapiens GN=IGFBP3 PE=1 SV=2//2.31542e-170
XM_007981498.1	0.43161	0.000183	0.001554	sp|Q96EL2|RT24_HUMAN 28S ribosomal protein S24, mitochondrial OS=Homo sapiens GN=MRPS24 PE=1 SV=1//4.19765e-111
XM_007981526.1	-0.3993	9.65E-05	0.000861	sp|P25787|PSA2_HUMAN Proteasome subunit alpha type-2 OS=Homo sapiens GN=PSMA2 PE=1 SV=2//6.93194e-173
XM_007981538.1	4.7515	0	0	sp|P08476|INHBA_HUMAN Inhibin beta A chain OS=Homo sapiens GN=INHBA PE=1 SV=2//0
XM_007981541.1	-0.3381	0.002294	0.015583	sp|Q3UKJ7|SMU1_MOUSE WD40 repeat-containing protein SMU1 OS=Mus musculus GN=Smu1 PE=2 SV=2//0
XM_007981551.1	0.54866	9.54E-07	1.16E-05	sp|P11233|RALA_HUMAN Ras-related protein Ral-A OS=Homo sapiens GN=RALA PE=1 SV=1//9.74395e-127
XM_007981553.1	-0.58302	1.09E-06	1.31E-05	sp|P02794|FRIH_HUMAN Ferritin heavy chain OS=Homo sapiens GN=FTH1 PE=1 SV=2//6.13994e-125
XM_007981572.1	-0.35854	0.00019	0.001607	sp|Q9N0C7|EPDR1_MACFA Mammalian ependymin-related protein 1 OS=Macaca fascicularis GN=EPDR1 PE=2 SV=3//2.49682e-157
XM_007981573.1	2.2479	2.52E-05	0.000246	sp|Q7YRN1|SFRP4_MACMU Secreted frizzled-related protein 4 OS=Macaca mulatta GN=SFRP4 PE=2 SV=1//0
XM_007981618.1	1.6717	4.13E-27	2.41E-25	sp|Q7L9B9|EEPD1_HUMAN Endonuclease/exonuclease/phosphatase family domain-containing protein 1 OS=Homo sapiens GN=EEPD1 PE=1 SV=2//0
XM_007981653.1	0.80307	9.16E-05	0.00082	sp|Q8TA86|RP9_HUMAN Retinitis pigmentosa 9 protein OS=Homo sapiens GN=RP9

				PE=1 SV=2//8.26701e-90
XM_007981654.1	-1.6681	2.71E-15	7.59E-14	sp|O95302|FKBP9_HUMAN Peptidyl-prolyl cis-trans isomerase FKBP9 OS=Homo sapiens GN=FKBP9 PE=1 SV=2//0
XM_007981657.1	1.0217	1.55E-09	2.59E-08	sp|Q8IY47|KBTB2_HUMAN Kelch repeat and BTB domain-containing protein 2 OS=Homo sapiens GN=KBTBD2 PE=1 SV=2//0
XM_007981701.1	-1.3649	0.000323	0.00263	sp|Q9UPT6|JIP3_HUMAN C-Jun-amino-terminal kinase-interacting protein 3 OS=Homo sapiens GN=MAPK8IP3 PE=1 SV=3//0
XM_007981709.1	-1.9603	1.21E-05	0.000123	sp|Q9UPT6|JIP3_HUMAN C-Jun-amino-terminal kinase-interacting protein 3 OS=Homo sapiens GN=MAPK8IP3 PE=1 SV=3//0
XM_007981718.1	1.1995	1.51E-47	1.99E-45	sp|Q5RBL1|SYG_PONAB Glycine--tRNA ligase OS=Pongo abelii GN=GARS PE=2 SV=1//0
XM_007981734.1	-0.97232	2.23E-08	3.26E-07	sp|Q8N3F0|MTURN_HUMAN Maturin OS=Homo sapiens GN=MTURN PE=2 SV=2//1.11228e-77
XM_007981790.1	-0.56758	7.03E-10	1.22E-08	sp|P31937|3HIDH_HUMAN 3-hydroxyisobutyrate dehydrogenase, mitochondrial OS=Homo sapiens GN=HIBADH PE=1 SV=2//0
XM_007981805.1	-0.65346	3.31E-06	3.71E-05	sp|Q0VCS4|HXA2_BOVIN Homeobox protein Hox-A2 OS=Bos taurus GN=HOXA2 PE=2 SV=1//0
XM_007981807.1	-0.81325	5.43E-07	6.77E-06	sp|O43365|HXA3_HUMAN Homeobox protein Hox-A3 OS=Homo sapiens GN=HOXA3 PE=1 SV=1//2.87628e-39
XM_007981808.1	-2.5644	0.005059	0.03167	sp|O43365|HXA3_HUMAN Homeobox protein Hox-A3 OS=Homo sapiens GN=HOXA3 PE=1 SV=1//3.60339e-39
XM_007981817.1	-0.31447	0.001279	0.009206	sp|P31269|HXA9_HUMAN Homeobox protein Hox-A9 OS=Homo sapiens GN=HOXA9 PE=1 SV=4//9.69208e-146
XM_007981818.1	-1.0284	0.007286	0.043577	-//-
XM_007981819.1	-1.2155	2.31E-26	1.31E-24	sp|P31260|HXA10_HUMAN Homeobox protein Hox-A10 OS=Homo sapiens GN=HOXA10 PE=1 SV=3//2.3236e-161
XM_007981837.1	-0.68935	1.13E-07	1.52E-06	sp|Q9Y4A8|NF2L3_HUMAN Nuclear factor erythroid 2-related factor 3 OS=Homo sapiens GN=NFE2L3 PE=1 SV=1//0
XM_007981880.1	0.92948	6.88E-07	8.48E-06	sp|Q96EE4|CC126_HUMAN Coiled-coil domain-containing protein 126 OS=Homo sapiens GN=CCDC126 PE=2 SV=2//6.83323e-63
XM_007981890.1	1.6085	1.98E-20	7.96E-19	sp|O15504|NUPL2_HUMAN Nucleoporin-like protein 2 OS=Homo sapiens GN=NUPL2 PE=1 SV=1//0
XM_007981896.1	0.42738	0.001832	0.012687	sp|Q9BYI3|HYCCI_HUMAN Hyccin OS=Homo sapiens GN=FAM126A PE=1 SV=2//0
XM_007981920.1	1.2218	1.39E-05	0.00014	sp|Q02446|SP4_HUMAN Transcription factor Sp4 OS=Homo sapiens GN=SP4 PE=1 SV=2//0
XM_007981926.1	1.0275	1.94E-18	6.84E-17	sp|P26012|ITB8_HUMAN Integrin beta-8 OS=Homo sapiens GN=ITGB8 PE=2 SV=1//0
XM_007981935.1	0.89342	1.94E-14	5.09E-13	sp|Q3B726|RPA43_HUMAN DNA-directed RNA polymerase I subunit RPA43 OS=Homo sapiens GN=TWISTNB PE=1 SV=1//0
XM_007981960.1	-0.40083	0.003473	0.022564	sp|Q5R869|MSRB1_PONAB Methionine-R-sulfoxide reductase B1 OS=Pongo abelii GN=MSRB1 PE=3 SV=2//3.45901e-73
XM_007981965.1	-1.0203	9.18E-22	3.99E-20	sp|Q02373|NDUBA_BOVIN NADH dehydrogenase [ubiquinone] 1 beta subcomplex subunit 10 OS=Bos taurus GN=NDUFB10 PE=1 SV=2//1.01484e-92
XM_007981966.1	1.6799	5.31E-46	6.53E-44	sp|P35869|AHR_HUMAN Aryl hydrocarbon receptor OS=Homo sapiens GN=AHR PE=1 SV=2//0
XM_007981979.1	0.48604	0.000306	0.002504	sp|Q8IV38|ANKY2_HUMAN Ankyrin repeat and MYND domain-containing protein 2 OS=Homo sapiens GN=ANKMY2 PE=1 SV=1//0
XM_007981989.1	-0.43479	0.004476	0.028378	sp|P36578|RL4_HUMAN 60S ribosomal protein L4 OS=Homo sapiens GN=RPL4 PE=1 SV=5//0
XM_007982004.1	0.42279	0.000117	0.001026	sp|Q12788|TBL3_HUMAN Transducin beta-like protein 3 OS=Homo sapiens GN=TBL3 PE=1 SV=2//0
XM_007982014.1	-0.41452	0.004142	0.02649	sp|Q9Y6U3|ADSV_HUMAN Adseverin OS=Homo sapiens GN=SCIN PE=1 SV=4//0
XM_007982044.1	-0.95575	1.05E-24	5.45E-23	sp|Q3YAJ5|NDUA4_MACMU Cytochrome c oxidase subunit NDUFA4 OS=Macaca mulatta GN=NDUFA4 PE=3 SV=1//6.02684e-51
XM_007982062.1	0.93313	1.46E-06	1.72E-05	sp|P55789|ALR_HUMAN FAD-linked sulfhydryl oxidase ALR OS=Homo sapiens GN=GFER PE=1 SV=2//3.63948e-120
XM_007982083.1	0.85173	5.98E-15	1.64E-13	sp|Q9NS00|C1GLT_HUMAN Glycoprotein-N-acetylgalactosamine 3-beta-galactosyltransferase 1 OS=Homo sapiens GN=C1GALT1 PE=1 SV=1//0
XM_007982098.1	-0.98854	1.54E-05	0.000155	sp|P60897|DSS1_MOUSE 26S proteasome complex subunit DSS1 OS=Mus musculus GN=Shfm1 PE=3 SV=1//5.00447e-20
XM_007982107.1	3.4744	6.24E-05	0.000576	sp|O88485|DC1I1_MOUSE Cytoplasmic dynein 1 intermediate chain 1 OS=Mus musculus GN=Dync1i1 PE=1 SV=2//0
XM_007982113.1	-0.67385	1.89E-11	3.84E-10	sp|Q15165|PON2_HUMAN Serum paraoxonase/arylesterase 2 OS=Homo sapiens GN=PON2 PE=1 SV=3//0
XM_007982123.1	-1.0963	2.56E-55	4.55E-53	sp|Q86TG7|PEG10_HUMAN Retrotransposon-derived protein PEG10 OS=Homo sapiens GN=PEG10 PE=1 SV=2//0
XM_007982139.1	0.36646	0.000222	0.001859	sp|O15155|BET1_HUMAN BET1 homolog OS=Homo sapiens GN=BET1 PE=1 SV=1//5.2628e-62
XM_007982141.1	-0.45791	0.001545	0.010949	sp|P61954|GBG11_RAT Guanine nucleotide-binding protein G(I)/G(S)/G(O) subunit gamma-11 OS=Rattus norvegicus GN=Gng11 PE=3 SV=1//3.67032e-42

XM_007982173.1	0.77381	3.47E-05	0.000331	sp|Q5RL73|RBM48_HUMAN RNA-binding protein 48 OS=Homo sapiens GN=RBM48 PE=2 SV=1//0
XM_007982175.1	0.35227	0.003221	0.021103	sp|Q8WUU5|GATD1_HUMAN GATA zinc finger domain-containing protein 1 OS=Homo sapiens GN=GATAD1 PE=1 SV=1//1.58699e-155
XM_007982216.1	-0.29557	0.002971	0.019592	sp|Q9UP38|FZD1_HUMAN Frizzled-1 OS=Homo sapiens GN=FZD1 PE=1 SV=2//0
XM_007982234.1	0.69746	8.97E-05	0.000804	sp|A4D1E9|GTPBA_HUMAN GTP-binding protein 10 OS=Homo sapiens GN=GTPBP10 PE=1 SV=1//0
XM_007982286.1	1.4208	0.003203	0.020992	sp|Q9UBU7|DBF4A_HUMAN Protein DBF4 homolog A OS=Homo sapiens GN=DBF4 PE=1 SV=1//0
XM_007982309.1	0.64381	0.000765	0.005785	sp|Q9UKG9|OCTC_HUMAN Peroxisomal carnitine O-octanoyltransferase OS=Homo sapiens GN=CROT PE=1 SV=2//0
XM_007982331.1	-0.28887	0.007495	0.04462	sp|O95025|SEM3D_HUMAN Semaphorin-3D OS=Homo sapiens GN=SEMA3D PE=2 SV=2//0
XM_007982333.1	0.87332	2.91E-13	7.01E-12	sp|Q14563|SEM3A_HUMAN Semaphorin-3A OS=Homo sapiens GN=SEMA3A PE=1 SV=1//0
XM_007982361.1	1.1988	1.23E-45	1.48E-43	sp|Q5RE75|SEM3C_PONAB Semaphorin-3C OS=Pongo abelii GN=SEMA3C PE=2 SV=1//0
XM_007982374.1	0.78357	0.000122	0.001065	sp|B2RSH2|GNAI1_MOUSE Guanine nucleotide-binding protein G(i) subunit alpha-1 OS=Mus musculus GN=Gnai1 PE=2 SV=1//0
XM_007982377.1	0.87309	0.003468	0.022542	sp|Q8N0U4|F185A_HUMAN Protein FAM185A OS=Homo sapiens GN=FAM185A PE=2 SV=3//0
XM_007982459.1	0.82283	8.87E-06	9.24E-05	sp|Q6IQ20|NAPEP_HUMAN N-acyl-phosphatidylethanolamine-hydrolyzing phospholipase D OS=Homo sapiens GN=NAPEPLD PE=1 SV=2//0
XM_007982480.1	0.95646	1.77E-10	3.30E-09	sp|O43913|ORC5_HUMAN Origin recognition complex subunit 5 OS=Homo sapiens GN=ORC5 PE=1 SV=1//0
XM_007982502.1	0.85083	1.45E-09	2.44E-08	sp|Q96PZ0|PUS7_HUMAN Pseudouridylate synthase 7 homolog OS=Homo sapiens GN=PUS7 PE=1 SV=2//0
XM_007982516.1	0.47143	4.07E-07	5.13E-06	sp|Q52I78|NAMPT_PIG Nicotinamide phosphoribosyltransferase OS=Sus scrofa GN=NAMPT PE=2 SV=2//0
XM_007982517.1	0.95535	2.10E-08	3.07E-07	sp|Q8N9Z2|CC71L_HUMAN Coiled-coil domain-containing protein 71L OS=Homo sapiens GN=CCDC71L PE=2 SV=2//1.76959e-80
XM_007982562.1	-0.47288	1.25E-05	0.000127	sp|P09622|DLDH_HUMAN Dihydrolipoyl dehydrogenase, mitochondrial OS=Homo sapiens GN=DLD PE=1 SV=2//0
XM_007982563.1	0.22269	0.002305	0.015633	sp|P07942|LAMB1_HUMAN Laminin subunit beta-1 OS=Homo sapiens GN=LAMB1 PE=1 SV=2//0
XM_007982593.1	-0.6526	7.12E-07	8.76E-06	sp|P42126|ECI1_HUMAN Enoyl-CoA delta isomerase 1, mitochondrial OS=Homo sapiens GN=ECI1 PE=1 SV=1//1.81272e-179
XM_007982598.1	2.1477	1.85E-44	2.13E-42	sp|Q9QYI6|DNJB9_MOUSE DnaJ homolog subfamily B member 9 OS=Mus musculus GN=Dnajb9 PE=1 SV=2//1.37108e-133
XM_007982626.1	2.521	3.44E-06	3.85E-05	sp|O00458|IFRD1_HUMAN Interferon-related developmental regulator 1 OS=Homo sapiens GN=IFRD1 PE=1 SV=4//0
XM_007982630.1	0.49047	2.03E-05	0.0002	sp|Q1RMZ1|BMT2_HUMAN Probable methyltransferase BTM2 homolog OS=Homo sapiens GN=C7orf60 PE=2 SV=1//0
XM_007982660.1	0.87988	1.92E-08	2.82E-07	sp|Q9P1T7|MDFIC_HUMAN MyoD family inhibitor domain-containing protein OS=Homo sapiens GN=MDFIC PE=1 SV=2//7.00653e-139
XM_007982678.1	1.0307	2.36E-38	2.21E-36	sp|Q2IBA6|MET_CHLAE Hepatocyte growth factor receptor OS=Chlorocebus aethiops GN=MET PE=3 SV=1//0
XM_007982703.1	-0.82979	6.71E-05	0.000616	sp|Q5RCP3|LSM8_PONAB U6 snRNA-associated Sm-like protein LSm8 OS=Pongo abelii GN=LSM8 PE=3 SV=3//1.04535e-61
XM_007982787.1	0.36448	3.41E-06	3.81E-05	sp|O00401|WASL_HUMAN Neural Wiskott-Aldrich syndrome protein OS=Homo sapiens GN=WASL PE=1 SV=2//1.57687e-47
XM_007982790.1	-0.28707	0.000868	0.006484	sp|Q99758|ABCA3_HUMAN ATP-binding cassette sub-family A member 3 OS=Homo sapiens GN=ABCA3 PE=1 SV=2//0
XM_007982793.1	3.2338	########	########	sp|O15354|GPR37_HUMAN Prosaposin receptor GPR37 OS=Homo sapiens GN=GPR37 PE=1 SV=2//0
XM_007982811.1	-2.1453	6.44E-05	0.000593	sp|O00634|NET3_HUMAN Netrin-3 OS=Homo sapiens GN=NTN3 PE=1 SV=1//0
XM_007982816.1	-0.55615	2.17E-08	3.17E-07	sp|P84083|ARF5_RAT ADP-ribosylation factor 5 OS=Rattus norvegicus GN=Arf5 PE=1 SV=2//7.52311e-129
XM_007982817.1	1.3197	1.83E-28	1.15E-26	sp|Q96CN9|GCC1_HUMAN GRIP and coiled-coil domain-containing protein 1 OS=Homo sapiens GN=GCC1 PE=1 SV=1//0
XM_007982820.1	-0.72631	3.28E-18	1.14E-16	sp|P27449|VATL_HUMAN V-type proton ATPase 16 kDa proteolipid subunit OS=Homo sapiens GN=ATP6V0C PE=1 SV=1//1.12101e-79
XM_007982822.1	-0.26149	0.001661	0.01165	sp|Q5REU4|SND1_PONAB Staphylococcal nuclease domain-containing protein 1 OS=Pongo abelii GN=SND1 PE=2 SV=1//0
XM_007982825.1	0.73345	4.61E-11	9.08E-10	sp|Q9NW13|RBM28_HUMAN RNA-binding protein 28 OS=Homo sapiens GN=RBM28 PE=1 SV=3//0
XM_007982826.1	0.33601	0.007968	0.047165	sp|Q9Y303|NAGA_HUMAN Putative N-acetylglucosamine-6-phosphate deacetylase OS=Homo sapiens GN=AMDHD2 PE=1 SV=2//0
XM_007982856.1	-0.4455	5.89E-06	6.34E-05	sp|Q16864|VATF_HUMAN V-type proton ATPase subunit F OS=Homo sapiens GN=ATP6V1F PE=1 SV=2//2.33458e-79
XM_007982877.1	-0.57051	3.47E-10	6.22E-09	sp|Q99835|SMO_HUMAN Smoothened homolog OS=Homo sapiens GN=SMO PE=1 SV=1//0
XM_007982923.1	0.73336	1.11E-08	1.68E-07	sp|Q2VPB7|AP5B1_HUMAN AP-5 complex subunit beta-1 OS=Homo sapiens GN=AP5B1

				PE=1 SV=4//0
XM_007982958.1	-2.189	0.000171	0.001462	sp|Q7RTY9|PRS41_HUMAN Putative serine protease 41 OS=Homo sapiens GN=PRSS41 PE=5 SV=1//2.05898e-91
XM_007982975.1	-0.22201	0.001966	0.013497	sp|P15121|ALDR_HUMAN Aldose reductase OS=Homo sapiens GN=AKR1B1 PE=1 SV=3//0
XM_007982978.1	-1.028	0.000601	0.004645	sp|O60218|AK1BA_HUMAN Aldo-keto reductase family 1 member B10 OS=Homo sapiens GN=AKR1B10 PE=1 SV=2//0
XM_007983009.1	2.0271	2.32E-33	1.79E-31	sp|Q9NV12|TM140_HUMAN Transmembrane protein 140 OS=Homo sapiens GN=TMEM140 PE=1 SV=2//2.9118e-51
XM_007983012.1	-0.51951	3.02E-07	3.85E-06	sp|A4D1P6|WDR91_HUMAN WD repeat-containing protein 91 OS=Homo sapiens GN=WDR91 PE=1 SV=2//0
XM_007983032.1	-1.8733	0.000705	0.005369	sp|Q9UQ35|SRRM2_HUMAN Serine/arginine repetitive matrix protein 2 OS=Homo sapiens GN=SRRM2 PE=1 SV=2//6.27353e-44
XM_007983037.1	-0.90076	9.52E-07	1.16E-05	sp|P68303|MT2_MACFA Metallothionein-2 OS=Macaca fascicularis GN=MT2 PE=3 SV=1//2.14723e-09
XM_007983039.1	-0.82517	9.18E-16	2.67E-14	sp|Q15370|ELOB_HUMAN Transcription elongation factor B polypeptide 2 OS=Homo sapiens GN=TCEB2 PE=1 SV=1//8.26059e-81
XM_007983065.1	0.47831	0.000111	0.000977	sp|Q70SY1|CR3L2_HUMAN Cyclic AMP-responsive element-binding protein 3-like protein 2 OS=Homo sapiens GN=CREB3L2 PE=1 SV=3//0
XM_007983088.1	-1.1976	4.29E-19	1.58E-17	sp|Q96H79|ZCCHL_HUMAN Zinc finger CCCH-type antiviral protein 1-like OS=Homo sapiens GN=ZC3HAV1L PE=1 SV=2//3.07233e-170
XM_007983111.1	-0.89788	3.01E-05	0.00029	sp|Q4G012|CG055_RAT UPF0562 protein C7orf55 homolog OS=Rattus norvegicus PE=3 SV=1//6.62146e-74
XM_007983137.1	0.88393	1.76E-14	4.66E-13	sp|Q9H0J9|PAR12_HUMAN Poly [ADP-ribose] polymerase 12 OS=Homo sapiens GN=PARP12 PE=1 SV=1//0
XM_007983158.1	-0.60587	0.002078	0.014226	sp|Q9ULE3|DEN2A_HUMAN DENN domain-containing protein 2A OS=Homo sapiens GN=DENND2A PE=2 SV=4//0
XM_007983167.1	0.6933	0.000158	0.001358	sp|P15056|BRAF_HUMAN Serine/threonine-protein kinase B-raf OS=Homo sapiens GN=BRAF PE=1 SV=4//0
XM_007983170.1	0.51674	0.004167	0.026618	sp|Q0MQC8|NDUB2_GORGO NADH dehydrogenase [ubiquinone] 1 beta subcomplex subunit 2, mitochondrial OS=Gorilla gorilla gorilla GN=NDUFB2 PE=3 SV=1//4.41306e-48
XM_007983187.1	0.43818	0.002222	0.015137	sp|Q99640|PMYT1_HUMAN Membrane-associated tyrosine- and threonine-specific cdc2-inhibitory kinase OS=Homo sapiens GN=PKMYT1 PE=1 SV=1//0
XM_007983231.1	2.5078	########	########	sp|Q9NP84|TNR12_HUMAN Tumor necrosis factor receptor superfamily member 12A OS=Homo sapiens GN=TNFRSF12A PE=1 SV=1//1.83127e-65
XM_007983267.1	-0.59094	7.57E-07	9.29E-06	sp|Q9Y2Q3|GSTK1_HUMAN Glutathione S-transferase kappa 1 OS=Homo sapiens GN=GSTK1 PE=1 SV=3//5.52691e-151
XM_007983322.1	0.78434	5.31E-13	1.24E-11	sp|Q12774|ARHG5_HUMAN Rho guanine nucleotide exchange factor 5 OS=Homo sapiens GN=ARHGEF5 PE=1 SV=3//0
XM_007983335.1	0.44	5.56E-08	7.72E-07	sp|Q9UHC6|CNTP2_HUMAN Contactin-associated protein-like 2 OS=Homo sapiens GN=CNTNAP2 PE=1 SV=1//0
XM_007983344.1	0.59925	1.26E-12	2.83E-11	sp|Q5R4G6|CUL1_PONAB Cullin-1 OS=Pongo abelii GN=CUL1 PE=2 SV=1//0
XM_007983354.1	-0.90903	1.76E-32	1.30E-30	sp|P13667|PDIA4_HUMAN Protein disulfide-isomerase A4 OS=Homo sapiens GN=PDIA4 PE=1 SV=2//0
XM_007983355.1	1.6069	6.30E-06	6.74E-05	sp|Q8N393|ZN786_HUMAN Zinc finger protein 786 OS=Homo sapiens GN=ZNF786 PE=2 SV=2//0
XM_007983356.1	0.56465	0.00105	0.007718	sp|Q9N003|ZN425_MACFA Zinc finger protein 425 (Fragment) OS=Macaca fascicularis GN=ZNF425 PE=2 SV=2//0
XM_007983357.1	1.9414	4.33E-87	1.63E-84	sp|Q8TD17|ZN398_HUMAN Zinc finger protein 398 OS=Homo sapiens GN=ZNF398 PE=1 SV=1//0
XM_007983358.1	0.92991	6.14E-19	2.23E-17	sp|Q9UDV7|ZN282_HUMAN Zinc finger protein 282 OS=Homo sapiens GN=ZNF282 PE=2 SV=3//0
XM_007983408.1	-0.65631	1.21E-05	0.000124	sp|Q5EB76|VA0E2_RAT V-type proton ATPase subunit e 2 OS=Rattus norvegicus GN=Atp6v0e2 PE=3 SV=1//3.56265e-46
XM_007983460.1	-0.79658	0.000244	0.00203	sp|Q8TDP1|RNH2C_HUMAN Ribonuclease H2 subunit C OS=Homo sapiens GN=RNASEH2C PE=1 SV=1//1.78393e-101
XM_007983508.1	0.49651	6.05E-06	6.50E-05	sp|Q9UG63|ABCF2_HUMAN ATP-binding cassette sub-family F member 2 OS=Homo sapiens GN=ABCF2 PE=1 SV=2//0
XM_007983536.1	6.3669	3.30E-11	6.60E-10	sp|Q9UGJ0|AAKG2_HUMAN 5'-AMP-activated protein kinase subunit gamma-2 OS=Homo sapiens GN=PRKAG2 PE=1 SV=1//0
XM_007983560.1	1.6393	6.04E-16	1.77E-14	sp|O43543|XRCC2_HUMAN DNA repair protein XRCC2 OS=Homo sapiens GN=XRCC2 PE=1 SV=1//4.04143e-173
XM_007983586.1	1.0332	2.84E-21	1.20E-19	sp|O15503|INSI1_HUMAN Insulin-induced gene 1 protein OS=Homo sapiens GN=INSIG1 PE=1 SV=3//2.55543e-133
XM_007983604.1	-0.59526	7.55E-13	1.74E-11	-//-
XM_007983655.1	0.50054	1.30E-07	1.73E-06	sp|Q86XI2|CNDG2_HUMAN Condensin-2 complex subunit G2 OS=Homo sapiens GN=NCAPG2 PE=1 SV=1//0
XM_007983686.1	-0.77189	1.40E-06	1.65E-05	sp|Q9UHC9|NPCL1_HUMAN Niemann-Pick C1-like protein 1 OS=Homo sapiens GN=NPC1L1 PE=1 SV=2//5.5276e-55

XM_007983704.1	0.39063	0.000741	0.005625	sp|A4D0T7|YG055_HUMAN Putative transmembrane protein LINC00998 OS=Homo sapiens GN=LINC00998 PE=5 SV=2//2.19456e-13
XM_007983705.1	0.24194	0.004273	0.027194	sp|Q04206|TF65_HUMAN Transcription factor p65 OS=Homo sapiens GN=RELA PE=1 SV=2//0
XM_007983707.1	0.56537	1.62E-10	3.03E-09	sp|Q92621|NU205_HUMAN Nuclear pore complex protein Nup205 OS=Homo sapiens GN=NUP205 PE=1 SV=3//0
XM_007983717.1	0.67685	8.80E-05	0.000791	sp|P42575|CASP2_HUMAN Caspase-2 OS=Homo sapiens GN=CASP2 PE=1 SV=2//0
XM_007983888.1	1.4183	0.000109	0.000962	sp|Q96LX8|ZN597_HUMAN Zinc finger protein 597 OS=Homo sapiens GN=ZNF597 PE=2 SV=1//0
XM_007983905.1	-1.0394	2.91E-07	3.72E-06	sp|Q96AT1|K1143_HUMAN Uncharacterized protein KIAA1143 OS=Homo sapiens GN=KIAA1143 PE=1 SV=2//1.17633e-72
XM_007983922.1	-2.1292	0.000684	0.005233	sp|Q8WZ71|TM158_HUMAN Transmembrane protein 158 OS=Homo sapiens GN=TMEM158 PE=2 SV=2//2.18709e-90
XM_007983940.1	0.30785	0.000347	0.002808	sp|Q9BQS8|FYCO1_HUMAN FYVE and coiled-coil domain-containing protein 1 OS=Homo sapiens GN=FYCO1 PE=1 SV=3//0
XM_007983991.1	-1.7928	0.000114	0.001	sp|Q7Z5A4|PRS42_HUMAN Serine protease 42 OS=Homo sapiens GN=PRSS42 PE=2 SV=1//6.70191e-143
XM_007984086.1	-0.52354	6.75E-05	0.00062	sp|P27816|MAP4_HUMAN Microtubule-associated protein 4 OS=Homo sapiens GN=MAP4 PE=1 SV=3//2.13609e-142
XM_007984136.1	-0.42741	2.06E-08	3.03E-07	sp|P31930|QCR1_HUMAN Cytochrome b-c1 complex subunit 1, mitochondrial OS=Homo sapiens GN=UQCRC1 PE=1 SV=3//0
XM_007984178.1	-0.37612	0.00223	0.015172	sp|Q9BU61|NDUF3_HUMAN NADH dehydrogenase [ubiquinone] 1 alpha subcomplex assembly factor 3 OS=Homo sapiens GN=NDUFAF3 PE=1 SV=1//4.66044e-117
XM_007984206.1	0.70912	1.11E-06	1.34E-05	sp|Q9BZE0|GLIS2_HUMAN Zinc finger protein GLIS2 OS=Homo sapiens GN=GLIS2 PE=1 SV=2//4.88973e-34
XM_007984221.1	-0.28279	0.003919	0.02519	sp|Q865R2|GPX1_MACFU Glutathione peroxidase 1 OS=Macaca fuscata fuscata GN=GPX1 PE=2 SV=3//2.69867e-128
XM_007984222.1	-0.50111	6.39E-12	1.35E-10	sp|Q5REY6|RHOA_PONAB Transforming protein RhoA OS=Pongo abelii GN=RHOA PE=2 SV=2//5.92947e-135
XM_007984226.1	-0.482	0.000253	0.002098	sp|Q9BSH3|NICN1_HUMAN Nicolin-1 OS=Homo sapiens GN=NICN1 PE=2 SV=1//1.28449e-149
XM_007984231.1	0.84395	4.03E-25	2.14E-23	sp|Q6EMK4|VASN_HUMAN Vasorin OS=Homo sapiens GN=VASN PE=1 SV=1//0
XM_007984245.1	-0.3931	0.000875	0.006526	sp|P13798|ACPH_HUMAN Acylamino-acid-releasing enzyme OS=Homo sapiens GN=APEH PE=1 SV=4//0
XM_007984297.1	-0.76402	1.91E-20	7.71E-19	sp|P04899|GNAI2_HUMAN Guanine nucleotide-binding protein G(i) subunit alpha-2 OS=Homo sapiens GN=GNAI2 PE=1 SV=3//0
XM_007984339.1	0.67604	7.29E-08	9.97E-07	sp|Q9WVF8|TUSC2_MOUSE Tumor suppressor candidate 2 OS=Mus musculus GN=Tusc2 PE=1 SV=3//2.56179e-55
XM_007984348.1	0.56857	5.82E-08	8.04E-07	sp|Q12893|TM115_HUMAN Transmembrane protein 115 OS=Homo sapiens GN=TMEM115 PE=1 SV=1//3.06062e-173
XM_007984395.1	0.34284	0.001456	0.010374	sp|Q8NDT2|RB15B_HUMAN Putative RNA-binding protein 15B OS=Homo sapiens GN=RBM15B PE=1 SV=3//0
XM_007984399.1	0.89805	1.74E-08	2.57E-07	sp|Q9Y4B4|ARIP4_HUMAN Helicase ARIP4 OS=Homo sapiens GN=RAD54L2 PE=1 SV=4//0
XM_007984429.1	4.2213	1.85E-66	4.26E-64	sp|O43818|U3IP2_HUMAN U3 small nucleolar RNA-interacting protein 2 OS=Homo sapiens GN=RRP9 PE=1 SV=1//0
XM_007984497.1	-0.89662	3.11E-08	4.45E-07	sp|P19123|TNNC1_MOUSE Troponin C, slow skeletal and cardiac muscles OS=Mus musculus GN=Tnnc1 PE=2 SV=1//2.57866e-100
XM_007984501.1	-1.667	0.002604	0.017395	sp|Q6Q0N3|NT5D2_RAT 5'-nucleotidase domain-containing protein 2 OS=Rattus norvegicus GN=Nt5dc2 PE=2 SV=2//0
XM_007984508.1	-0.91039	0.001155	0.008403	sp|Q8WVI0|SMIM4_HUMAN Small integral membrane protein 4 OS=Homo sapiens GN=SMIM4 PE=1 SV=2//9.32747e-42
XM_007984512.1	0.99549	1.41E-21	6.07E-20	sp|Q9BVP2|GNL3_HUMAN Guanine nucleotide-binding protein-like 3 OS=Homo sapiens GN=GNL3 PE=1 SV=2//0
XM_007984573.1	1.3718	5.44E-10	9.53E-09	sp|O60304|ZN500_HUMAN Zinc finger protein 500 OS=Homo sapiens GN=ZNF500 PE=2 SV=2//0
XM_007984574.1	-0.87727	4.00E-35	3.38E-33	sp|Q60HC7|TKT_MACFA Transketolase OS=Macaca fascicularis GN=TKT PE=2 SV=1//0
XM_007984575.1	1.0904	7.51E-35	6.28E-33	sp|Q9NPI6|DCP1A_HUMAN mRNA-decapping enzyme 1A OS=Homo sapiens GN=DCP1A PE=1 SV=2//0
XM_007984610.1	0.44655	7.64E-08	1.04E-06	sp|Q49A26|GLYR1_HUMAN Putative oxidoreductase GLYR1 OS=Homo sapiens GN=GLYR1 PE=1 SV=3//0
XM_007984657.1	-0.66176	0.000285	0.002342	sp|Q9UK61|F208A_HUMAN Protein FAM208A OS=Homo sapiens GN=FAM208A PE=1 SV=3//1.96886e-96
XM_007984737.1	0.5999	1.40E-08	2.09E-07	sp|Q6L8Q7|PDE12_HUMAN 2',5'-phosphodiesterase 12 OS=Homo sapiens GN=PDE12 PE=1 SV=2//0
XM_007984741.1	-0.32517	0.004503	0.028514	sp|Q8IWF6|DEN6A_HUMAN Protein DENND6A OS=Homo sapiens GN=DENND6A PE=1 SV=1//0
XM_007984768.1	-4.4456	0.00073	0.005551	sp|O75369|FLNB_HUMAN Filamin-B OS=Homo sapiens GN=FLNB PE=1 SV=2//0
XM_007984772.1	-0.63451	0.00016	0.001372	sp|Q9UK23|NAGPA_HUMAN N-acetylglucosamine-1-phosphodiester alpha-N-

				acetylglucosaminidase OS=Homo sapiens GN=NAGPA PE=1 SV=2//0
XM_007984776.1	0.63093	0.0019	0.013105	sp|P86397|HTD2_HUMAN Hydroxyacyl-thioester dehydratase type 2, mitochondrial OS=Homo sapiens GN=RPP14 PE=1 SV=1//1.49624e-116
XM_007984787.1	-0.649	6.14E-11	1.20E-09	sp|P11177|ODPB_HUMAN Pyruvate dehydrogenase E1 component subunit beta, mitochondrial OS=Homo sapiens GN=PDHB PE=1 SV=3//0
XM_007984890.1	2.3256	1.17E-25	6.34E-24	sp|Q7Z3G6|PRIC2_HUMAN Prickle-like protein 2 OS=Homo sapiens GN=PRICKLE2 PE=1 SV=2//0
XM_007984939.1	-0.23396	0.004055	0.025998	sp|Q4R4R4|PRAF3_MACFA PRA1 family protein 3 OS=Macaca fascicularis GN=ARL6IP5 PE=2 SV=1//7.82827e-118
XM_007984949.1	-1.456	2.45E-07	3.17E-06	-//-
XM_007984982.1	-0.58461	0.003741	0.024144	sp|Q9NS67|GPR27_HUMAN Probable G-protein coupled receptor 27 OS=Homo sapiens GN=GPR27 PE=2 SV=1//1.88231e-142
XM_007984997.1	2.188	########	########	sp|Q8N488|RYBP_HUMAN RING1 and YY1-binding protein OS=Homo sapiens GN=RYBP PE=1 SV=2//1.96396e-76
XM_007984998.1	0.54481	0.00059	0.004569	sp|Q6PI26|SHQ1_HUMAN Protein SHQ1 homolog OS=Homo sapiens GN=SHQ1 PE=1 SV=2//0
XM_007984999.1	-1.9164	4.95E-15	1.36E-13	sp|A0PJZ3|GXLT2_HUMAN Glucoside xylosyltransferase 2 OS=Homo sapiens GN=GXYLT2 PE=2 SV=2//0
XM_007985051.1	-1.026	7.03E-06	7.45E-05	sp|Q6UXK5|LRRN1_HUMAN Leucine-rich repeat neuronal protein 1 OS=Homo sapiens GN=LRRN1 PE=1 SV=1//0
XM_007985061.1	1.947	3.01E-58	5.82E-56	sp|O14503|BHE40_HUMAN Class E basic helix-loop-helix protein 40 OS=Homo sapiens GN=BHLHE40 PE=1 SV=1//0
XM_007985075.1	0.52316	1.04E-09	1.78E-08	sp|Q66HA6|ARL8B_RAT ADP-ribosylation factor-like protein 8B OS=Rattus norvegicus GN=Arl8b PE=2 SV=1//9.07202e-121
XM_007985091.1	2.2691	1.23E-65	2.78E-63	sp|Q9NZU5|LMCD1_HUMAN LIM and cysteine-rich domains protein 1 OS=Homo sapiens GN=LMCD1 PE=1 SV=1//0
XM_007985101.1	1.0682	2.19E-19	8.24E-18	sp|Q9NS91|RAD18_HUMAN E3 ubiquitin-protein ligase RAD18 OS=Homo sapiens GN=RAD18 PE=1 SV=2//0
XM_007985119.1	-2.0009	4.33E-06	4.77E-05	-//-
XM_007985159.1	-0.91753	2.89E-12	6.31E-11	sp|Q9Y4R7|TTLL3_HUMAN Tubulin monoglycylase TTLL3 OS=Homo sapiens GN=TTLL3 PE=1 SV=2//0
XM_007985164.1	-0.24736	0.006624	0.04019	sp|O75528|TADA3_HUMAN Transcriptional adapter 3 OS=Homo sapiens GN=TADA3 PE=1 SV=1//0
XM_007985165.1	0.75933	1.34E-09	2.26E-08	sp|Q8N5M9|JAGN1_HUMAN Protein jagunal homolog 1 OS=Homo sapiens GN=JAGN1 PE=1 SV=1//1.94007e-114
XM_007985209.1	-0.34582	0.000585	0.004533	sp|Q8WUW1|BRK1_HUMAN Protein BRICK1 OS=Homo sapiens GN=BRK1 PE=1 SV=1//3.008e-46
XM_007985212.1	0.66702	5.03E-05	0.00047	sp|Q93075|TATD2_HUMAN Putative deoxyribonuclease TATDN2 OS=Homo sapiens GN=TATDN2 PE=2 SV=2//0
XM_007985224.1	0.9896	2.65E-09	4.32E-08	sp|Q96B77|TM186_HUMAN Transmembrane protein 186 OS=Homo sapiens GN=TMEM186 PE=2 SV=1//2.88403e-133
XM_007985235.1	1.2313	5.22E-05	0.000487	sp|Q14135|VGLL4_HUMAN Transcription cofactor vestigial-like protein 4 OS=Homo sapiens GN=VGLL4 PE=1 SV=4//4.54253e-165
XM_007985236.1	2.1469	7.99E-05	0.000724	sp|Q14135|VGLL4_HUMAN Transcription cofactor vestigial-like protein 4 OS=Homo sapiens GN=VGLL4 PE=1 SV=4//0
XM_007985263.1	1.613	2.49E-26	1.41E-24	sp|Q8NCE0|SEN2_HUMAN tRNA-splicing endonuclease subunit Sen2 OS=Homo sapiens GN=TSEN2 PE=1 SV=2//0
XM_007985266.1	0.37265	0.000394	0.003156	sp|Q9H000|MKRN2_HUMAN Probable E3 ubiquitin-protein ligase makorin-2 OS=Homo sapiens GN=MKRN2 PE=1 SV=2//0
XM_007985280.1	-0.65094	1.27E-18	4.56E-17	sp|P62912|RL32_RAT 60S ribosomal protein L32 OS=Rattus norvegicus GN=Rpl32 PE=1 SV=2//8.74144e-85
XM_007985310.1	0.73045	1.33E-13	3.28E-12	sp|Q9BTV4|TMM43_HUMAN Transmembrane protein 43 OS=Homo sapiens GN=TMEM43 PE=1 SV=1//0
XM_007985313.1	-0.8349	3.16E-09	5.12E-08	sp|P62311|LSM3_MOUSE U6 snRNA-associated Sm-like protein LSm3 OS=Mus musculus GN=Lsm3 PE=3 SV=2//9.64876e-49
XM_007985325.1	0.86721	7.16E-15	1.96E-13	sp|Q6PII3|CC174_HUMAN Coiled-coil domain-containing protein 174 OS=Homo sapiens GN=CCDC174 PE=2 SV=3//0
XM_007985334.1	1.5331	6.94E-06	7.37E-05	sp|Q6ZNL6|FGD5_HUMAN FYVE, RhoGEF and PH domain-containing protein 5 OS=Homo sapiens GN=FGD5 PE=1 SV=3//0
XM_007985335.1	1.2722	5.46E-48	7.37E-46	sp|Q28HY5|CP072_XENTR UPF0472 protein C16orf72 homolog OS=Xenopus tropicalis GN=TEgg029f10.1 PE=2 SV=1//1.43904e-144
XM_007985336.1	-0.63096	2.83E-06	3.20E-05	sp|P82663|RT25_HUMAN 28S ribosomal protein S25, mitochondrial OS=Homo sapiens GN=MRPS25 PE=1 SV=1//4.63029e-103
XM_007985371.1	2.6883	5.20E-08	7.25E-07	sp|O94876|TMCC1_HUMAN Transmembrane and coiled-coil domains protein 1 OS=Homo sapiens GN=TMCC1 PE=1 SV=3//0
XM_007985375.1	-0.54671	0.001087	0.007967	sp|Q9Y4D7|PLXD1_HUMAN Plexin-D1 OS=Homo sapiens GN=PLXND1 PE=1 SV=3//0
XM_007985407.1	-0.36786	0.000408	0.003266	sp|Q86YS6|RAB43_HUMAN Ras-related protein Rab-43 OS=Homo sapiens GN=RAB43 PE=1 SV=1//9.67522e-124
XM_007985441.1	-0.25245	0.00086	0.006428	sp|P18067|RAB7A_CANFA Ras-related protein Rab-7a OS=Canis familiaris GN=RAB7A PE=2 SV=1//3.35015e-139

XM_007985442.1	-0.22491	0.001868	0.012906	sp|Q4R4T0|RPN1_MACFA Dolichyl-diphosphooligosaccharide--protein glycosyltransferase subunit 1 OS=Macaca fascicularis GN=RPN1 PE=2 SV=1//0
XM_007985512.1	0.77217	0.00522	0.032546	sp|Q2QGD7|ZXDC_HUMAN Zinc finger protein ZXDC OS=Homo sapiens GN=ZXDC PE=1 SV=2//0
XM_007985522.1	-0.64428	2.20E-06	2.53E-05	sp|Q5RFM9|AL1L1_PONAB Cytosolic 10-formyltetrahydrofolate dehydrogenase OS=Pongo abelii GN=ALDH1L1 PE=2 SV=1//0
XM_007985535.1	0.53684	1.20E-10	2.28E-09	sp|O95219|SNX4_HUMAN Sorting nexin-4 OS=Homo sapiens GN=SNX4 PE=1 SV=1//0
XM_007985536.1	1.4692	1.16E-06	1.39E-05	sp|Q9BXB4|OSB11_HUMAN Oxysterol-binding protein-related protein 11 OS=Homo sapiens GN=OSBPL11 PE=1 SV=2//0
XM_007985546.1	-0.2668	0.001175	0.008539	sp|P18084|ITB5_HUMAN Integrin beta-5 OS=Homo sapiens GN=ITGB5 PE=1 SV=1//0
XM_007985548.1	0.78678	2.04E-10	3.77E-09	sp|P11172|UMPS_HUMAN Uridine 5'-monophosphate synthase OS=Homo sapiens GN=UMPS PE=1 SV=1//0
XM_007985573.1	0.50075	4.80E-09	7.62E-08	sp|Q49A88|CCD14_HUMAN Coiled-coil domain-containing protein 14 OS=Homo sapiens GN=CCDC14 PE=1 SV=3//0
XM_007985597.1	0.61667	5.05E-06	5.50E-05	sp|Q96SL1|DIRC2_HUMAN Disrupted in renal carcinoma protein 2 OS=Homo sapiens GN=DIRC2 PE=1 SV=1//0
XM_007985598.1	0.68268	6.67E-09	1.03E-07	sp|Q460N5|PAR14_HUMAN Poly [ADP-ribose] polymerase 14 OS=Homo sapiens GN=PARP14 PE=1 SV=3//0
XM_007985606.1	0.65083	1.31E-13	3.25E-12	sp|Q5R909|IMA5_PONAB Importin subunit alpha-5 OS=Pongo abelii GN=KPNA1 PE=2 SV=1//0
XM_007985612.1	0.52328	0.002472	0.016589	sp|Q5RE95|WDR5B_PONAB WD repeat-containing protein 5B OS=Pongo abelii PE=2 SV=1//0
XM_007985637.1	0.70515	6.69E-07	8.28E-06	sp|Q15051|IQCB1_HUMAN IQ calmodulin-binding motif-containing protein 1 OS=Homo sapiens GN=IQCB1 PE=1 SV=1//0
XM_007985662.1	1.1485	6.35E-09	9.89E-08	sp|Q9Y2K9|STB5L_HUMAN Syntaxin-binding protein 5-like OS=Homo sapiens GN=STXBP5L PE=1 SV=2//0
XM_007985665.1	0.77741	7.59E-17	2.39E-15	sp|Q5R8H5|T2EA_PONAB General transcription factor IIE subunit 1 OS=Pongo abelii GN=GTF2E1 PE=2 SV=1//0
XM_007985667.1	-0.47193	0.001728	0.012071	sp|P0CB71|NDUB4_PONPY NADH dehydrogenase [ubiquinone] 1 beta subcomplex subunit 4 OS=Pongo pygmaeus GN=NDUFB4 PE=2 SV=1//5.35727e-72
XM_007985672.1	0.70275	4.01E-07	5.05E-06	sp|Q96CX6|LRC58_HUMAN Leucine-rich repeat-containing protein 58 OS=Homo sapiens GN=LRRC58 PE=1 SV=2//7.56676e-173
XM_007985692.1	-0.96377	2.94E-06	3.31E-05	sp|Q14061|COX17_HUMAN Cytochrome c oxidase copper chaperone OS=Homo sapiens GN=COX17 PE=1 SV=2//1.01055e-25
XM_007985707.1	1.1348	2.44E-19	9.08E-18	sp|Q9NPL8|TIDC1_HUMAN Complex I assembly factor TIMMDC1, mitochondrial OS=Homo sapiens GN=TIMMDC1 PE=1 SV=2//2.31404e-173
XM_007985762.1	1.8934	7.55E-39	7.25E-37	sp|Q9H974|QTRD1_HUMAN Queuine tRNA-ribosyltransferase subunit QTRTD1 OS=Homo sapiens GN=QTRTD1 PE=1 SV=1//0
XM_007985778.1	1.9145	1.77E-05	0.000176	sp|O15524|SOCS1_HUMAN Suppressor of cytokine signaling 1 OS=Homo sapiens GN=SOCS1 PE=1 SV=1//5.19402e-114
XM_007985802.1	0.79485	3.00E-05	0.000289	sp|Q6NW34|CC017_HUMAN Uncharacterized protein C3orf17 OS=Homo sapiens GN=C3orf17 PE=1 SV=3//0
XM_007985819.1	0.9174	1.96E-16	5.99E-15	sp|Q9NT62|ATG3_HUMAN Ubiquitin-like-conjugating enzyme ATG3 OS=Homo sapiens GN=ATG3 PE=1 SV=1//0
XM_007985845.1	3.3129	########	########	sp|Q0VAA5|PLCX2_HUMAN PI-PLC X domain-containing protein 2 OS=Homo sapiens GN=PLCXD2 PE=2 SV=1//0
XM_007985937.1	0.7327	9.63E-13	2.19E-11	sp|Q6PKC3|TXD11_HUMAN Thioredoxin domain-containing protein 11 OS=Homo sapiens GN=TXNDC11 PE=1 SV=2//0
XM_007985958.1	0.69991	2.66E-12	5.84E-11	sp|Q5RCE6|RL1D1_PONAB Ribosomal L1 domain-containing protein 1 OS=Pongo abelii GN=RSL1D1 PE=2 SV=2//0
XM_007985959.1	-0.4766	0.000321	0.002618	sp|Q5RCA5|NXPE3_PONAB NXPE family member 3 OS=Pongo abelii GN=NXPE3 PE=2 SV=1//0
XM_007985964.1	-0.65302	1.64E-11	3.36E-10	sp|P83732|RL24_RAT 60S ribosomal protein L24 OS=Rattus norvegicus GN=Rpl24 PE=2 SV=1//4.6018e-76
XM_007985968.1	0.87782	1.03E-06	1.25E-05	sp|Q7L0Y3|MRRP1_HUMAN Mitochondrial ribonuclease P protein 1 OS=Homo sapiens GN=TRMT10C PE=1 SV=2//0
XM_007985969.1	0.48875	0.000794	0.005979	sp|Q5RCE6|RL1D1_PONAB Ribosomal L1 domain-containing protein 1 OS=Pongo abelii GN=RSL1D1 PE=2 SV=2//0
XM_007986053.1	0.79855	2.24E-12	4.93E-11	sp|O94826|TOM70_HUMAN Mitochondrial import receptor subunit TOM70 OS=Homo sapiens GN=TOMM70A PE=1 SV=1//0
XM_007986140.1	1.1126	4.43E-06	4.87E-05	sp|Q9H649|NSUN3_HUMAN Putative methyltransferase NSUN3 OS=Homo sapiens GN=NSUN3 PE=2 SV=1//0
XM_007986148.1	-0.68085	6.52E-06	6.95E-05	sp|Q28520|PROS_MACMU Vitamin K-dependent protein S (Fragment) OS=Macaca mulatta GN=PROS1 PE=2 SV=2//0
XM_007986169.1	0.42341	0.002691	0.01788	sp|Q8BJF9|CHM2B_MOUSE Charged multivesicular body protein 2b OS=Mus musculus GN=Chmp2b PE=1 SV=1//3.01732e-105
XM_007986199.1	0.98111	1.88E-09	3.12E-08	sp|Q92889|XPF_HUMAN DNA repair endonuclease XPF OS=Homo sapiens GN=ERCC4 PE=1 SV=3//0
XM_007986203.1	-0.92522	8.02E-05	0.000726	-//-
XM_007986205.1	0.26461	0.000414	0.003306	sp|Q92922|SMRC1_HUMAN SWI/SNF complex subunit SMARCC1 OS=Homo sapiens

				GN=SMARCC1 PE=1 SV=3//1.57833e-08
XM_007986207.1	2.2701	8.10E-98	3.77E-95	sp|P30304|MPIP1_HUMAN M-phase inducer phosphatase 1 OS=Homo sapiens GN=CDC25A PE=1 SV=2//0
XM_007986209.1	0.54528	1.40E-08	2.09E-07	sp|P78332|RBM6_HUMAN RNA-binding protein 6 OS=Homo sapiens GN=RBM6 PE=1 SV=5//0
XM_007986210.1	-0.32891	0.001948	0.013402	sp|P55145|MANF_HUMAN Mesencephalic astrocyte-derived neurotrophic factor OS=Homo sapiens GN=MANF PE=1 SV=3//3.24138e-125
XM_007986217.1	0.5212	8.83E-08	1.20E-06	sp|Q9BXW9|FACD2_HUMAN Fanconi anemia group D2 protein OS=Homo sapiens GN=FANCD2 PE=1 SV=2//0
XM_007986218.1	1.0136	6.36E-11	1.24E-09	sp|O43187|IRAK2_HUMAN Interleukin-1 receptor-associated kinase-like 2 OS=Homo sapiens GN=IRAK2 PE=1 SV=2//2.31674e-86
XM_007986224.1	-1.459	0.001192	0.008649	-//-
XM_007986225.1	-0.45841	1.42E-08	2.12E-07	sp|Q9Y678|COPG1_HUMAN Coatomer subunit gamma-1 OS=Homo sapiens GN=COPG1 PE=1 SV=1//0
XM_007986226.1	-0.73613	1.42E-12	3.19E-11	sp|Q9NZ53|PDXL2_HUMAN Podocalyxin-like protein 2 OS=Homo sapiens GN=PODXL2 PE=1 SV=1//0
XM_007986233.1	1.4387	1.30E-47	1.74E-45	sp|O75417|DPOLQ_HUMAN DNA polymerase theta OS=Homo sapiens GN=POLQ PE=1 SV=2//0
XM_007986290.1	-0.7111	1.18E-14	3.17E-13	sp|P58238|PSME1_MACFA Proteasome activator complex subunit 1 OS=Macaca fascicularis GN=PSME1 PE=2 SV=1//1.20644e-154
XM_007986294.1	0.68941	1.31E-12	2.95E-11	sp|Q96EP0|RNF31_HUMAN E3 ubiquitin-protein ligase RNF31 OS=Homo sapiens GN=RNF31 PE=1 SV=1//0
XM_007986295.1	-0.55052	1.79E-05	0.000178	sp|Q9UL46|PSME2_HUMAN Proteasome activator complex subunit 2 OS=Homo sapiens GN=PSME2 PE=1 SV=4//1.53974e-157
XM_007986303.1	-0.56754	0.00013	0.001125	sp|Q9BY43|CHM4A_HUMAN Charged multivesicular body protein 4a OS=Homo sapiens GN=CHMP4A PE=1 SV=3//1.31554e-72
XM_007986315.1	-0.58158	4.46E-07	5.60E-06	sp|Q8N5H3|FA89B_HUMAN Protein FAM89B OS=Homo sapiens GN=FAM89B PE=1 SV=2//3.96718e-62
XM_007986317.1	-0.82938	6.34E-14	1.60E-12	sp|Q71UE8|NEDD8_RAT NEDD8 OS=Rattus norvegicus GN=Nedd8 PE=1 SV=1//1.47225e-26
XM_007986324.1	0.30646	0.000874	0.006519	sp|Q9NZS9|BFAR_HUMAN Bifunctional apoptosis regulator OS=Homo sapiens GN=BFAR PE=1 SV=1//0
XM_007986327.1	-3.272	0.004942	0.031015	sp|Q8NFM4|ADCY4_HUMAN Adenylate cyclase type 4 OS=Homo sapiens GN=ADCY4 PE=1 SV=1//0
XM_007986336.1	0.31405	0.000822	0.006173	sp|O95453|PARN_HUMAN Poly(A)-specific ribonuclease PARN OS=Homo sapiens GN=PARN PE=1 SV=1//0
XM_007986342.1	-0.89188	4.44E-08	6.23E-07	sp|Q9P2P1|NYNRI_HUMAN Protein NYNRIN OS=Homo sapiens GN=NYNRIN PE=2 SV=3//0
XM_007986365.1	1.5244	2.98E-50	4.40E-48	sp|Q9NYV6|RRN3_HUMAN RNA polymerase I-specific transcription initiation factor RRN3 OS=Homo sapiens GN=RRN3 PE=1 SV=1//0
XM_007986429.1	0.54392	0.0028	0.018537	sp|Q96CQ1|S2536_HUMAN Solute carrier family 25 member 36 OS=Homo sapiens GN=SLC25A36 PE=1 SV=1//0
XM_007986466.1	-1.1487	0.001625	0.011441	sp|Q4R3M6|NDUS5_MACFA NADH dehydrogenase [ubiquinone] iron-sulfur protein 5 OS=Macaca fascicularis GN=NDUFS5 PE=3 SV=3//3.09591e-67
XM_007986467.1	0.8581	1.12E-17	3.77E-16	sp|Q9NRL2|BAZ1A_HUMAN Bromodomain adjacent to zinc finger domain protein 1A OS=Homo sapiens GN=BAZ1A PE=1 SV=2//0
XM_007986481.1	1.6406	4.78E-07	5.98E-06	sp|Q969Q6|P2R3C_HUMAN Serine/threonine-protein phosphatase 2A regulatory subunit B'' subunit gamma OS=Homo sapiens GN=PPP2R3C PE=1 SV=1//0
XM_007986523.1	1.1177	2.92E-16	8.75E-15	sp|P55317|FOXA1_HUMAN Hepatocyte nuclear factor 3-alpha OS=Homo sapiens GN=FOXA1 PE=1 SV=2//0
XM_007986545.1	-0.48532	0.001895	0.013072	sp|Q15436|SC23A_HUMAN Protein transport protein Sec23A OS=Homo sapiens GN=SEC23A PE=1 SV=2//0
XM_007986552.1	-0.64769	2.75E-12	6.01E-11	sp|Q5R5X0|PININ_PONAB Pinin OS=Pongo abelii GN=PNN PE=2 SV=1//9.70803e-22
XM_007986562.1	0.99211	4.07E-11	8.07E-10	sp|Q7Z6M2|FBX33_HUMAN F-box only protein 33 OS=Homo sapiens GN=FBXO33 PE=1 SV=1//0
XM_007986569.1	-0.69238	2.91E-06	3.30E-05	sp|O95255|MRP6_HUMAN Multidrug resistance-associated protein 6 OS=Homo sapiens GN=ABCC6 PE=1 SV=2//0
XM_007986593.1	-0.75649	1.40E-12	3.15E-11	sp|Q00688|FKBP3_HUMAN Peptidyl-prolyl cis-trans isomerase FKBP3 OS=Homo sapiens GN=FKBP3 PE=1 SV=1//1.51962e-160
XM_007986612.1	-0.89889	5.46E-14	1.38E-12	sp|P83883|RL36A_RAT 60S ribosomal protein L36a OS=Rattus norvegicus GN=Rpl36a PE=1 SV=2//1.50064e-70
XM_007986613.1	0.43002	1.71E-05	0.00017	sp|Q10469|MGAT2_HUMAN Alpha-1,6-mannosyl-glycoprotein 2-beta-N-acetylglucosaminyltransferase OS=Homo sapiens GN=MGAT2 PE=1 SV=1//0
XM_007986624.1	0.34089	2.48E-05	0.000243	sp|P62332|ARF6_RAT ADP-ribosylation factor 6 OS=Rattus norvegicus GN=Arf6 PE=1 SV=2//8.9906e-116
XM_007986656.1	0.63507	4.84E-07	6.06E-06	sp|Q9H4B6|SAV1_HUMAN Protein salvador homolog 1 OS=Homo sapiens GN=SAV1 PE=1 SV=2//0
XM_007986670.1	-0.38349	6.16E-05	0.000568	sp|Q9H3N1|TMX1_HUMAN Thioredoxin-related transmembrane protein 1 OS=Homo sapiens GN=TMX1 PE=1 SV=1//1.36946e-156

XM_007986672.1	-1.1175	6.23E-28	3.81E-26	sp|Q15041|AR6P1_HUMAN ADP-ribosylation factor-like protein 6-interacting protein 1 OS=Homo sapiens GN=ARL6IP1 PE=1 SV=2//1.24226e-116
XM_007986690.1	-0.42323	2.04E-06	2.35E-05	sp|Q5R8I2|CN166_PONAB UPF0568 protein C14orf166 homolog OS=Pongo abelii PE=2 SV=1//6.06591e-167
XM_007986697.1	-0.68033	3.90E-06	4.31E-05	sp|Q96HE7|ERO1A_HUMAN ERO1-like protein alpha OS=Homo sapiens GN=ERO1L PE=1 SV=2//0
XM_007986725.1	-0.95475	6.98E-13	1.62E-11	sp|Q16667|CDKN3_HUMAN Cyclin-dependent kinase inhibitor 3 OS=Homo sapiens GN=CDKN3 PE=1 SV=1//2.21065e-143
XM_007986736.1	2.3117	0.00356	0.023054	sp|Q9UPU9|SMAG1_HUMAN Protein Smaug homolog 1 OS=Homo sapiens GN=SAMD4A PE=1 SV=3//0
XM_007986742.1	1.1669	4.54E-37	4.08E-35	sp|Q8WXH5|SOCS4_HUMAN Suppressor of cytokine signaling 4 OS=Homo sapiens GN=SOCS4 PE=1 SV=1//0
XM_007986749.1	-0.51475	3.98E-07	5.02E-06	sp|P17931|LEG3_HUMAN Galectin-3 OS=Homo sapiens GN=LGALS3 PE=1 SV=5//4.04386e-90
XM_007986753.1	0.72692	1.66E-05	0.000166	sp|Q6ZNE5|BAKOR_HUMAN Beclin 1-associated autophagy-related key regulator OS=Homo sapiens GN=ATG14 PE=1 SV=1//0
XM_007986770.1	#NAME?	0.000191	0.001614	sp|Q9HAT8|PELI2_HUMAN E3 ubiquitin-protein ligase pellino homolog 2 OS=Homo sapiens GN=PELI2 PE=1 SV=1//0
XM_007986787.1	0.85771	4.76E-11	9.35E-10	sp|Q147X3|NAA30_HUMAN N-alpha-acetyltransferase 30 OS=Homo sapiens GN=NAA30 PE=1 SV=1//4.96209e-167
XM_007986802.1	-1.819	4.98E-07	6.22E-06	sp|Q86TY3|CN037_HUMAN Uncharacterized protein C14orf37 OS=Homo sapiens GN=C14orf37 PE=1 SV=1//0
XM_007986803.1	1.1503	3.95E-27	2.31E-25	sp|Q9BVV6|TALD3_HUMAN Protein TALPID3 OS=Homo sapiens GN=KIAA0586 PE=1 SV=4//0
XM_007986823.1	0.78548	0.001588	0.011217	sp|Q96EM0|T3HPD_HUMAN Trans-3-hydroxy-L-proline dehydratase OS=Homo sapiens GN=L3HYPDH PE=1 SV=2//0
XM_007986825.1	-0.29097	0.002296	0.015587	sp|Q9BZ67|FRMD8_HUMAN FERM domain-containing protein 8 OS=Homo sapiens GN=FRMD8 PE=1 SV=1//0
XM_007986886.1	0.45863	4.00E-05	0.000379	sp|P51948|MAT1_HUMAN CDK-activating kinase assembly factor MAT1 OS=Homo sapiens GN=MNAT1 PE=1 SV=1//0
XM_007986893.1	0.62478	1.08E-06	1.31E-05	sp|F7GSQ4|TRM5_MACMU tRNA (guanine(37)-N1)-methyltransferase OS=Macaca mulatta GN=TRMT5 PE=3 SV=1//0
XM_007986908.1	0.96178	3.43E-10	6.17E-09	sp|Q4R6W9|SNPC1_MACFA snRNA-activating protein complex subunit 1 OS=Macaca fascicularis GN=SNAPC1 PE=2 SV=1//0
XM_007986913.1	4.1174	0.003498	0.02269	sp|Q8NCM2|KCNH5_HUMAN Potassium voltage-gated channel subfamily H member 5 OS=Homo sapiens GN=KCNH5 PE=1 SV=3//0
XM_007986966.1	-0.90135	6.37E-10	1.11E-08	sp|P54652|HSP72_HUMAN Heat shock-related 70 kDa protein 2 OS=Homo sapiens GN=HSPA2 PE=1 SV=1//0
XM_007986974.1	1.2953	6.40E-48	8.59E-46	sp|A1L390|PKHG3_HUMAN Pleckstrin homology domain-containing family G member 3 OS=Homo sapiens GN=PLEKHG3 PE=1 SV=1//0
XM_007986976.1	-1.6916	0.002828	0.018701	sp|Q4AEH9|GPX2_HYLLA Glutathione peroxidase 2 OS=Hylobates lar GN=GPX2 PE=2 SV=2//2.97456e-134
XM_007987080.1	0.30974	0.000912	0.006776	sp|Q8TC12|RDH11_HUMAN Retinol dehydrogenase 11 OS=Homo sapiens GN=RDH11 PE=1 SV=2//0
XM_007987084.1	1.9893	6.18E-06	6.63E-05	sp|Q68DK2|ZFY26_HUMAN Zinc finger FYVE domain-containing protein 26 OS=Homo sapiens GN=ZFYVE26 PE=1 SV=3//0
XM_007987093.1	0.85976	1.06E-18	3.79E-17	sp|Q07352|TISB_HUMAN Zinc finger protein 36, C3H1 type-like 1 OS=Homo sapiens GN=ZFP36L1 PE=1 SV=1//3.83506e-174
XM_007987114.1	-0.78212	1.28E-08	1.93E-07	sp|P84089|ERH_MOUSE Enhancer of rudimentary homolog OS=Mus musculus GN=Erh PE=1 SV=1//5.82762e-72
XM_007987118.1	0.34278	0.005935	0.036424	sp|Q92537|SUSD6_HUMAN Sushi domain-containing protein 6 OS=Homo sapiens GN=SUSD6 PE=1 SV=1//2.12246e-176
XM_007987156.1	0.5763	1.98E-05	0.000195	sp|P57105|SYJ2B_HUMAN Synaptojanin-2-binding protein OS=Homo sapiens GN=SYNJ2BP PE=1 SV=2//5.70457e-96
XM_007987162.1	0.64254	1.01E-16	3.16E-15	sp|Q96RV3|PCX1_HUMAN Pecanex-like protein 1 OS=Homo sapiens GN=PCNX PE=1 SV=2//0
XM_007987193.1	0.85878	1.19E-07	1.59E-06	sp|Q9HBF4|ZFYV1_HUMAN Zinc finger FYVE domain-containing protein 1 OS=Homo sapiens GN=ZFYVE1 PE=1 SV=1//0
XM_007987220.1	-0.38766	0.00437	0.027754	sp|P49753|ACOT2_HUMAN Acyl-coenzyme A thioesterase 2, mitochondrial OS=Homo sapiens GN=ACOT2 PE=1 SV=6//0
XM_007987247.1	-0.78835	1.08E-08	1.64E-07	sp|Q02252|MMSA_HUMAN Methylmalonate-semialdehyde dehydrogenase [acylating], mitochondrial OS=Homo sapiens GN=ALDH6A1 PE=1 SV=2//0
XM_007987262.1	0.88298	0.000235	0.001957	sp|Q96MY7|F161B_HUMAN Protein FAM161B OS=Homo sapiens GN=FAM161B PE=1 SV=2//0
XM_007987272.1	-0.80402	5.31E-14	1.35E-12	sp|P61917|NPC2_PANTR Epididymal secretory protein E1 OS=Pan troglodytes GN=NPC2 PE=2 SV=1//4.5412e-92
XM_007987279.1	-0.92331	0.006121	0.037448	sp|Q3B8N5|PROX2_HUMAN Prospero homeobox protein 2 OS=Homo sapiens GN=PROX2 PE=2 SV=3//0
XM_007987281.1	-1.8886	0.000411	0.003285	sp|P49750|YLPM1_HUMAN YLP motif-containing protein 1 OS=Homo sapiens GN=YLPM1 PE=1 SV=3//8.08264e-39
XM_007987294.1	0.40951	0.000693	0.005296	sp|P49770|EI2BB_HUMAN Translation initiation factor eIF-2B subunit beta

				OS=Homo sapiens GN=EIF2B2 PE=1 SV=3//0
XM_007987321.1	5.8866	5.20E-24	2.62E-22	sp|P01100|FOS_HUMAN Proto-oncogene c-Fos OS=Homo sapiens GN=FOS PE=1 SV=1//0
XM_007987322.1	1.982	4.04E-56	7.37E-54	sp|Q8WYK2|JDP2_HUMAN Jun dimerization protein 2 OS=Homo sapiens GN=JDP2 PE=1 SV=1//7.25098e-75
XM_007987327.1	0.54381	0.001097	0.008037	sp|Q9UPI3|FLVC2_HUMAN Feline leukemia virus subgroup C receptor-related protein 2 OS=Homo sapiens GN=FLVCR2 PE=1 SV=1//0
XM_007987350.1	0.67826	4.44E-08	6.23E-07	sp|P10600|TGFB3_HUMAN Transforming growth factor beta-3 OS=Homo sapiens GN=TGFB3 PE=1 SV=1//0
XM_007987359.1	0.39949	0.002499	0.016746	sp|Q7L8A9|VASH1_HUMAN Vasohibin-1 OS=Homo sapiens GN=VASH1 PE=1 SV=1//0
XM_007987378.1	1.2862	1.12E-44	1.31E-42	sp|Q8K3X4|I2BPL_MOUSE Interferon regulatory factor 2-binding protein-like OS=Mus musculus GN=Irf2bpl PE=1 SV=1//1.86156e-71
XM_007987397.1	0.43744	3.83E-05	0.000364	sp|Q9UKY4|POMT2_HUMAN Protein O-mannosyl-transferase 2 OS=Homo sapiens GN=POMT2 PE=1 SV=2//0
XM_007987398.1	-1.02	0.001219	0.008816	sp|Q95JY5|SAM15_MACFA Sterile alpha motif domain-containing protein 15 OS=Macaca fascicularis GN=SAMD15 PE=2 SV=2//0
XM_007987404.1	0.64243	1.37E-05	0.000138	sp|Q9NXG2|THUM1_HUMAN THUMP domain-containing protein 1 OS=Homo sapiens GN=THUMPD1 PE=1 SV=2//0
XM_007987406.1	-1.023	7.27E-19	2.63E-17	sp|O95433|AHSA1_HUMAN Activator of 90 kDa heat shock protein ATPase homolog 1 OS=Homo sapiens GN=AHSA1 PE=1 SV=1//0
XM_007987415.1	-1.0972	1.11E-05	0.000114	sp|Q9GZT3|SLIRP_HUMAN SRA stem-loop-interacting RNA-binding protein, mitochondrial OS=Homo sapiens GN=SLIRP PE=1 SV=1//6.96244e-44
XM_007987495.1	-0.90898	3.12E-06	3.50E-05	sp|Q8WXE9|STON2_HUMAN Stonin-2 OS=Homo sapiens GN=STON2 PE=1 SV=1//0
XM_007987496.1	-0.99021	5.76E-28	3.53E-26	sp|Q9UBV2|SE1L1_HUMAN Protein sel-1 homolog 1 OS=Homo sapiens GN=SEL1L PE=1 SV=3//0
XM_007987570.1	-0.46866	3.45E-07	4.37E-06	sp|P62193|PRS4_RAT 26S protease regulatory subunit 4 OS=Rattus norvegicus GN=Psmc1 PE=2 SV=1//0
XM_007987572.1	-0.84862	1.34E-24	6.87E-23	sp|P62155|CALM_XENLA Calmodulin OS=Xenopus laevis GN=calm1 PE=1 SV=2//1.78506e-10
XM_007987583.1	1.1969	9.47E-06	9.81E-05	sp|Q6B0B8|TIGD3_HUMAN Tigger transposable element-derived protein 3 OS=Homo sapiens GN=TIGD3 PE=2 SV=1//0
XM_007987603.1	1.0897	7.62E-06	8.03E-05	sp|Q6IN85|P4R3A_HUMAN Serine/threonine-protein phosphatase 4 regulatory subunit 3A OS=Homo sapiens GN=SMEK1 PE=1 SV=1//0
XM_007987625.1	0.77436	2.99E-05	0.000288	sp|Q15643|TRIPB_HUMAN Thyroid receptor-interacting protein 11 OS=Homo sapiens GN=TRIP11 PE=1 SV=3//0
XM_007987629.1	-0.54008	3.88E-11	7.70E-10	sp|P22695|QCR2_HUMAN Cytochrome b-c1 complex subunit 2, mitochondrial OS=Homo sapiens GN=UQCRC2 PE=1 SV=3//0
XM_007987631.1	-1.4883	0.003554	0.023025	sp|Q0MQC6|NDUB1_PANTR NADH dehydrogenase [ubiquinone] 1 beta subcomplex subunit 1 OS=Pan troglodytes GN=NDUFB1 PE=3 SV=1//8.5776e-33
XM_007987641.1	-0.26844	0.000494	0.003892	sp|Q4R4T8|LGMN_MACFA Legumain OS=Macaca fascicularis GN=LGMN PE=2 SV=1//0
XM_007987652.1	0.98154	8.11E-13	1.86E-11	sp|Q7SZ07|CP52B_XENLA Uncharacterized protein C16orf52 homolog B OS=Xenopus laevis PE=2 SV=1//3.80311e-104
XM_007987696.1	0.68654	2.16E-18	7.59E-17	sp|P58268|REQU_CHICK Zinc finger protein ubi-d4 OS=Gallus gallus GN=REQ PE=2 SV=1//0
XM_007987697.1	-1.2807	2.71E-11	5.44E-10	sp|Q5R9E3|CBG_PONAB Corticosteroid-binding globulin OS=Pongo abelii GN=SERPINA6 PE=2 SV=1//0
XM_007987748.1	0.40916	1.19E-05	0.000122	sp|Q01850|CDR2_HUMAN Cerebellar degeneration-related protein 2 OS=Homo sapiens GN=CDR2 PE=1 SV=2//0
XM_007987761.1	3.5114	1.19E-07	1.59E-06	sp|P30411|BKRB2_HUMAN B2 bradykinin receptor OS=Homo sapiens GN=BDKRB2 PE=1 SV=2//0
XM_007987765.1	-1.3763	0.007152	0.04293	sp|O95976|IGSF6_HUMAN Immunoglobulin superfamily member 6 OS=Homo sapiens GN=IGSF6 PE=2 SV=2//9.20101e-161
XM_007987766.1	0.49185	0.000304	0.002488	sp|Q96BY7|ATG2B_HUMAN Autophagy-related protein 2 homolog B OS=Homo sapiens GN=ATG2B PE=1 SV=5//0
XM_007987788.1	-1.2498	2.45E-09	4.01E-08	sp|Q9H1A3|METL9_HUMAN Methyltransferase-like protein 9 OS=Homo sapiens GN=METTL9 PE=2 SV=1//5.88189e-158
XM_007987790.1	1.2416	7.18E-22	3.14E-20	sp|O14613|BORG1_HUMAN Cdc42 effector protein 2 OS=Homo sapiens GN=CDC42EP2 PE=1 SV=1//2.30146e-113
XM_007987798.1	0.41779	0.000554	0.004311	sp|O75909|CCNK_HUMAN Cyclin-K OS=Homo sapiens GN=CCNK PE=1 SV=2//0
XM_007987802.1	Inf	0.000709	0.005396	sp|Q7RTW8|OTOAN_HUMAN Otoancorin OS=Homo sapiens GN=OTOA PE=1 SV=1//0
XM_007987815.1	0.52256	0.001598	0.011277	sp|Q9H9Y2|RPF1_HUMAN Ribosome production factor 1 OS=Homo sapiens GN=RPF1 PE=1 SV=2//0
XM_007987831.1	-1.5493	0.000305	0.002493	sp|Q9UI08|EVL_HUMAN Ena/VASP-like protein OS=Homo sapiens GN=EVL PE=1 SV=2//0
XM_007987832.1	-1.5781	1.46E-08	2.17E-07	sp|Q5R896|EVL_PONAB Ena/VASP-like protein OS=Pongo abelii GN=EVL PE=2 SV=1//0
XM_007987837.1	-1.4842	1.23E-08	1.85E-07	sp|Q8N8R3|MCATL_HUMAN Mitochondrial basic amino acids transporter OS=Homo sapiens GN=SLC25A29 PE=2 SV=2//2.08137e-12
XM_007987866.1	0.42671	0.000768	0.005806	sp|Q70CQ4|UBP31_HUMAN Ubiquitin carboxyl-terminal hydrolase 31 OS=Homo

				sapiens GN=USP31 PE=2 SV=2//1.27349e-85
XM_007987883.1	1.201	7.89E-06	8.29E-05	sp|Q8TBZ3|WDR20_HUMAN WD repeat-containing protein 20 OS=Homo sapiens GN=WDR20 PE=1 SV=2//0
XM_007987898.1	0.98416	3.44E-06	3.84E-05	sp|A8K0R7|ZN839_HUMAN Zinc finger protein 839 OS=Homo sapiens GN=ZNF839 PE=2 SV=1//0
XM_007987937.1	-0.49397	1.29E-06	1.53E-05	sp|Q78JW9|UBFD1_MOUSE Ubiquitin domain-containing protein UBFD1 OS=Mus musculus GN=Ubfd1 PE=1 SV=2//0
XM_007987993.1	-1.5985	0.002864	0.018932	sp|Q8N142|PURA1_HUMAN Adenylosuccinate synthetase isozyme 1 OS=Homo sapiens GN=ADSSL1 PE=1 SV=1//0
XM_007988015.1	-1.3704	1.33E-25	7.21E-24	sp|Q8IVF2|AHNK2_HUMAN Protein AHNAK2 OS=Homo sapiens GN=AHNAK2 PE=1 SV=2//0
XM_007988016.1	1.1932	0.000295	0.002419	sp|Q96F83|CN079_HUMAN Uncharacterized protein C14orf79 OS=Homo sapiens GN=C14orf79 PE=2 SV=2//0
XM_007988057.1	0.29213	0.006538	0.039736	sp|Q9BTE1|DCTN5_HUMAN Dynactin subunit 5 OS=Homo sapiens GN=DCTN5 PE=1 SV=1//1.2172e-111
XM_007988059.1	-0.84168	5.41E-05	0.000502	sp|P50238|CRIP1_HUMAN Cysteine-rich protein 1 OS=Homo sapiens GN=CRIP1 PE=1 SV=3//9.09902e-50
XM_007988093.1	1.1379	0.003485	0.022618	sp|Q8N7A1|KLDC1_HUMAN Kelch domain-containing protein 1 OS=Homo sapiens GN=KLHDC1 PE=2 SV=2//0
XM_007988101.1	-1.2951	1.00E-41	1.05E-39	sp|Q15398|DLGP5_HUMAN Disks large-associated protein 5 OS=Homo sapiens GN=DLGAP5 PE=1 SV=2//1.18523e-85
XM_007988105.1	1.5195	1.77E-33	1.37E-31	sp|Q92623|TTC9A_HUMAN Tetratricopeptide repeat protein 9A OS=Homo sapiens GN=TTC9 PE=2 SV=3//2.76421e-105
XM_007988107.1	0.90373	8.85E-05	0.000795	sp|Q8CD94|LIN52_MOUSE Protein lin-52 homolog OS=Mus musculus GN=Lin52 PE=3 SV=1//9.15183e-47
XM_007988108.1	-0.51689	9.93E-06	0.000103	sp|O15033|AREL1_HUMAN Apoptosis-resistant E3 ubiquitin protein ligase 1 OS=Homo sapiens GN=AREL1 PE=1 SV=3//0
XM_007988109.1	0.86541	2.24E-06	2.57E-05	sp|Q9UHC1|MLH3_HUMAN DNA mismatch repair protein Mlh3 OS=Homo sapiens GN=MLH3 PE=1 SV=3//0
XM_007988110.1	0.53188	0.001216	0.008798	sp|Q8NDV7|TNR6A_HUMAN Trinucleotide repeat-containing gene 6A protein OS=Homo sapiens GN=TNRC6A PE=1 SV=2//0
XM_007988111.1	1.1374	4.17E-39	4.02E-37	sp|O15270|SPTC2_HUMAN Serine palmitoyltransferase 2 OS=Homo sapiens GN=SPTLC2 PE=1 SV=1//0
XM_007988112.1	2.4844	6.03E-43	6.60E-41	sp|Q13686|ALKB1_HUMAN Alkylated DNA repair protein alkB homolog 1 OS=Homo sapiens GN=ALKBH1 PE=1 SV=2//0
XM_007988123.1	-0.55175	0.004127	0.026407	sp|P12277|KCRB_HUMAN Creatine kinase B-type OS=Homo sapiens GN=CKB PE=1 SV=1//4.18481e-111
XM_007988124.1	0.49005	2.04E-08	3.00E-07	sp|Q5R581|KLC1_PONAB Kinesin light chain 1 OS=Pongo abelii GN=KLC1 PE=2 SV=3//0
XM_007988143.1	-1.4264	0.001933	0.013308	sp|Q9Y577|TRI17_HUMAN E3 ubiquitin-protein ligase TRIM17 OS=Homo sapiens GN=TRIM17 PE=1 SV=1//0
XM_007988144.1	-2.693	0.002588	0.017306	sp|Q16695|H31T_HUMAN Histone H3.1t OS=Homo sapiens GN=HIST3H3 PE=1 SV=3//1.94661e-83
XM_007988206.1	0.87249	4.33E-12	9.28E-11	sp|Q9BU76|MMTA2_HUMAN Multiple myeloma tumor-associated protein 2 OS=Homo sapiens GN=MMTAG2 PE=1 SV=1//5.13316e-109
XM_007988211.1	-1.4321	0.000259	0.002139	sp|P56704|WNT3A_HUMAN Protein Wnt-3a OS=Homo sapiens GN=WNT3A PE=1 SV=2//3.69413e-164
XM_007988274.1	1.1473	9.81E-28	5.93E-26	sp|Q6IQ49|SDE2_HUMAN Protein SDE2 homolog OS=Homo sapiens GN=SDE2 PE=1 SV=1//0
XM_007988280.1	-0.84479	0.007413	0.044214	sp|O00292|LFTY2_HUMAN Left-right determination factor 2 OS=Homo sapiens GN=LEFTY2 PE=1 SV=2//0
XM_007988315.1	1.1443	1.76E-13	4.30E-12	sp|Q63HK3|ZKSC2_HUMAN Zinc finger protein with KRAB and SCAN domains 2 OS=Homo sapiens GN=ZKSCAN2 PE=1 SV=2//0
XM_007988342.1	1.1709	2.15E-20	8.65E-19	sp|Q9NVF7|FBX28_HUMAN F-box only protein 28 OS=Homo sapiens GN=FBXO28 PE=1 SV=1//0
XM_007988365.1	-2.5444	0.00685	0.04132	sp|Q96F81|DISP1_HUMAN Protein dispatched homolog 1 OS=Homo sapiens GN=DISP1 PE=1 SV=3//0
XM_007988369.1	-0.35705	0.000752	0.005699	sp|Q5RAV3|AIDA_PONAB Axin interactor, dorsalization-associated protein OS=Pongo abelii GN=AIDA PE=2 SV=1//0
XM_007988390.1	1.782	1.20E-15	3.46E-14	sp|Q14774|HLX_HUMAN H2.0-like homeobox protein OS=Homo sapiens GN=HLX PE=1 SV=3//0
XM_007988392.1	-0.54584	4.62E-06	5.06E-05	sp|Q9H7X2|CA115_HUMAN Uncharacterized protein C1orf115 OS=Homo sapiens GN=C1orf115 PE=2 SV=1//7.61034e-32
XM_007988398.1	0.68318	0.003906	0.025121	sp|Q8N371|KDM8_HUMAN Lysine-specific demethylase 8 OS=Homo sapiens GN=KDM8 PE=1 SV=1//0
XM_007988420.1	4.5954	1.27E-46	1.59E-44	sp|Q9Y3B9|RRP15_HUMAN RRP15-like protein OS=Homo sapiens GN=RRP15 PE=1 SV=2//8.3571e-158
XM_007988457.1	0.65061	1.45E-15	4.16E-14	sp|Q15678|PTN14_HUMAN Tyrosine-protein phosphatase non-receptor type 14 OS=Homo sapiens GN=PTPN14 PE=1 SV=2//0
XM_007988480.1	-0.46412	0.005857	0.036048	sp|Q86V25|VASH2_HUMAN Vasohibin-2 OS=Homo sapiens GN=VASH2 PE=1 SV=2//0

XM_007988481.1	1.4641	7.68E-09	1.18E-07	sp|Q5VTE6|ANGE2_HUMAN Protein angel homolog 2 OS=Homo sapiens GN=ANGEL2 PE=2 SV=1//0
XM_007988489.1	0.53009	0.003163	0.020736	sp|Q9Y5Y0|FLVC1_HUMAN Feline leukemia virus subgroup C receptor-related protein 1 OS=Homo sapiens GN=FLVCR1 PE=1 SV=1//0
XM_007988501.1	6.1397	0	0	sp|P18847|ATF3_HUMAN Cyclic AMP-dependent transcription factor ATF-3 OS=Homo sapiens GN=ATF3 PE=1 SV=2//3.77845e-94
XM_007988502.1	-0.66515	6.54E-07	8.09E-06	sp|Q9UMX5|NENF_HUMAN Neudesin OS=Homo sapiens GN=NENF PE=1 SV=1//1.51082e-97
XM_007988506.1	0.80936	6.58E-16	1.93E-14	sp|Q15172|2A5A_HUMAN Serine/threonine-protein phosphatase 2A 56 kDa regulatory subunit alpha isoform OS=Homo sapiens GN=PPP2R5A PE=1 SV=1//0
XM_007988509.1	2.189	2.05E-77	6.31E-75	sp|Q9NZJ0|DTL_HUMAN Denticleless protein homolog OS=Homo sapiens GN=DTL PE=1 SV=3//0
XM_007988514.1	-0.53998	0.000107	0.000945	sp|Q92604|LGAT1_HUMAN Acyl-CoA:lysophosphatidylglycerol acyltransferase 1 OS=Homo sapiens GN=LPGAT1 PE=1 SV=1//0
XM_007988520.1	1.7533	3.97E-79	1.34E-76	sp|Q4R6K2|ZNT1_MACFA Zinc transporter 1 OS=Macaca fascicularis GN=SLC30A1 PE=2 SV=1//0
XM_007988578.1	3.3009	0.004888	0.030746	sp|Q68CQ4|DIEXF_HUMAN Digestive organ expansion factor homolog OS=Homo sapiens GN=DIEXF PE=1 SV=2//0
XM_007988590.1	1.4019	1.62E-08	2.40E-07	sp|Q5REJ0|CA074_PONAB UPF0739 protein C1orf74 homolog OS=Pongo abelii PE=2 SV=1//2.61276e-157
XM_007988650.1	1.6615	5.09E-32	3.68E-30	sp|Q5VVQ6|OTU1_HUMAN Ubiquitin thioesterase OTU1 OS=Homo sapiens GN=YOD1 PE=1 SV=1//0
XM_007988707.1	0.71804	1.71E-11	3.49E-10	sp|Q5R839|S41A1_PONAB Solute carrier family 41 member 1 OS=Pongo abelii GN=SLC41A1 PE=2 SV=1//0
XM_007988717.1	-1.0823	9.80E-44	1.09E-41	sp|Q9H1E3|NUCKS_HUMAN Nuclear ubiquitous casein and cyclin-dependent kinase substrate 1 OS=Homo sapiens GN=NUCKS1 PE=1 SV=1//8.81528e-41
XM_007988731.1	-0.76001	6.62E-06	7.04E-05	sp|Q5RD01|CDK18_PONAB Cyclin-dependent kinase 18 OS=Pongo abelii GN=CDK18 PE=2 SV=2//0
XM_007988753.1	2.664	2.02E-48	2.76E-46	sp|Q9H093|NUAK2_HUMAN NUAK family SNF1-like kinase 2 OS=Homo sapiens GN=NUAK2 PE=1 SV=1//0
XM_007988754.1	1.5926	0.003475	0.022572	sp|O75069|TMCC2_HUMAN Transmembrane and coiled-coil domains protein 2 OS=Homo sapiens GN=TMCC2 PE=1 SV=3//0
XM_007988806.1	1.2721	8.66E-47	1.10E-44	sp|Q5SWA1|PR15B_HUMAN Protein phosphatase 1 regulatory subunit 15B OS=Homo sapiens GN=PPP1R15B PE=1 SV=1//0
XM_007988885.1	0.71191	7.55E-07	9.27E-06	sp|P78543|BTG2_HUMAN Protein BTG2 OS=Homo sapiens GN=BTG2 PE=1 SV=1//1.79864e-90
XM_007988912.1	0.8086	1.11E-16	3.46E-15	sp|Q53G59|KLH12_HUMAN Kelch-like protein 12 OS=Homo sapiens GN=KLHL12 PE=1 SV=2//0
XM_007988917.1	0.33869	0.006059	0.03712	sp|Q9UGL1|KDM5B_HUMAN Lysine-specific demethylase 5B OS=Homo sapiens GN=KDM5B PE=1 SV=3//0
XM_007988928.1	-1.159	0.006074	0.03719	sp|Q9NRF2|SH2B1_HUMAN SH2B adapter protein 1 OS=Homo sapiens GN=SH2B1 PE=1 SV=3//1.17734e-20
XM_007988954.1	-0.87574	3.97E-16	1.18E-14	sp|Q9H4A4|AMPB_HUMAN Aminopeptidase B OS=Homo sapiens GN=RNPEP PE=1 SV=2//0
XM_007988970.1	0.33657	0.004585	0.028998	sp|Q9Y5J5|PHLA3_HUMAN Pleckstrin homology-like domain family A member 3 OS=Homo sapiens GN=PHLDA3 PE=1 SV=1//3.07047e-75
XM_007989026.1	-0.34075	0.008025	0.047466	sp|Q15058|KIF14_HUMAN Kinesin-like protein KIF14 OS=Homo sapiens GN=KIF14 PE=1 SV=1//0
XM_007989035.1	-0.7053	7.07E-22	3.10E-20	sp|P18669|PGAM1_HUMAN Phosphoglycerate mutase 1 OS=Homo sapiens GN=PGAM1 PE=1 SV=2//7.75524e-176
XM_007989042.1	-1.4149	1.45E-10	2.72E-09	sp|Q6P823|H33_XENTR Histone H3.3 OS=Xenopus tropicalis GN=TGas113e22.1 PE=1 SV=3//2.65897e-90
XM_007989057.1	1.6541	0.002787	0.018457	sp|Q8WWM7|ATX2L_HUMAN Ataxin-2-like protein OS=Homo sapiens GN=ATXN2L PE=1 SV=2//0
XM_007989180.1	-0.56516	9.44E-10	1.62E-08	sp|P23284|PPIB_HUMAN Peptidyl-prolyl cis-trans isomerase B OS=Homo sapiens GN=PPIB PE=1 SV=2//1.32285e-139
XM_007989207.1	-0.4662	0.00766	0.045456	sp|Q9H246|CA021_HUMAN Uncharacterized protein C1orf21 OS=Homo sapiens GN=C1orf21 PE=1 SV=1//7.67373e-76
XM_007989213.1	0.373	0.001225	0.008855	sp|Q8WW01|SEN15_HUMAN tRNA-splicing endonuclease subunit Sen15 OS=Homo sapiens GN=TSEN15 PE=1 SV=1//1.78594e-101
XM_007989219.1	-0.56284	7.33E-10	1.27E-08	sp|Q9CPW4|ARPC5_MOUSE Actin-related protein 2/3 complex subunit 5 OS=Mus musculus GN=Arpc5 PE=2 SV=3//2.10523e-99
XM_007989224.1	-0.51391	0.007641	0.04537	sp|Q9H3K6|BOLA2_HUMAN BolA-like protein 2 OS=Homo sapiens GN=BOLA2 PE=1 SV=1//2.47013e-52
XM_007989239.1	-1.0508	4.32E-05	0.000407	sp|Q9BQ83|SLX1_HUMAN Structure-specific endonuclease subunit SLX1 OS=Homo sapiens GN=SLX1A PE=1 SV=1//3.26007e-171
XM_007989248.1	0.52475	1.78E-07	2.34E-06	sp|Q5R874|DHX9_PONAB ATP-dependent RNA helicase A OS=Pongo abelii GN=DHX9 PE=2 SV=1//0
XM_007989250.1	-1.065	0.007256	0.043423	sp|P0DMN0|ST1A4_HUMAN Sulfotransferase 1A4 OS=Homo sapiens GN=SULT1A4 PE=1 SV=1//2.24107e-174
XM_007989298.1	1.3981	9.60E-24	4.74E-22	sp|Q5VY09|IER5_HUMAN Immediate early response gene 5 protein OS=Homo

				sapiens GN=IER5 PE=2 SV=3//4.46601e-101
XM_007989303.1	0.87313	1.22E-08	1.83E-07	sp|Q5R6Q2|STX6_PONAB Syntaxin-6 OS=Pongo abelii GN=STX6 PE=2 SV=1//1.89076e-149
XM_007989311.1	-1.2983	0.000313	0.002557	sp|O00391|QSOX1_HUMAN Sulfhydryl oxidase 1 OS=Homo sapiens GN=QSOX1 PE=1 SV=3//0
XM_007989327.1	-0.78149	2.64E-10	4.80E-09	sp|Q8NFQ8|TOIP2_HUMAN Torsin-1A-interacting protein 2 OS=Homo sapiens GN=TOR1AIP2 PE=1 SV=1//0
XM_007989375.1	1.4338	1.46E-32	1.08E-30	-//-
XM_007989419.1	-0.83057	4.14E-09	6.63E-08	sp|O60783|RT14_HUMAN 28S ribosomal protein S14, mitochondrial OS=Homo sapiens GN=MRPS14 PE=1 SV=1//4.07001e-86
XM_007989487.1	-0.38727	6.41E-06	6.83E-05	sp|Q2PFL9|PRDX6_MACFA Peroxiredoxin-6 OS=Macaca fascicularis GN=PRDX6 PE=2 SV=3//1.26771e-155
XM_007989488.1	1.1308	0.000962	0.00712	sp|Q9UNG2|TNF18_HUMAN Tumor necrosis factor ligand superfamily member 18 OS=Homo sapiens GN=TNFSF18 PE=1 SV=2//7.08579e-133
XM_007989504.1	#NAME?	0.002474	0.016596	sp|P04075|ALDOA_HUMAN Fructose-bisphosphate aldolase A OS=Homo sapiens GN=ALDOA PE=1 SV=2//0
XM_007989545.1	1.0559	2.27E-11	4.59E-10	sp|Q5T7V8|GORAB_HUMAN RAB6-interacting golgin OS=Homo sapiens GN=GORAB PE=1 SV=1//0
XM_007989591.1	0.2957	0.000278	0.002284	sp|Q4R4V5|AT1B1_MACFA Sodium/potassium-transporting ATPase subunit beta-1 OS=Macaca fascicularis GN=ATP1B1 PE=2 SV=1//0
XM_007989601.1	1.0573	1.28E-15	3.66E-14	sp|Q9H2G9|GO45_HUMAN Golgin-45 OS=Homo sapiens GN=BLZF1 PE=1 SV=2//0
XM_007989602.1	3.3272	0.007195	0.04313	sp|Q07507|DERM_HUMAN Dermatopontin OS=Homo sapiens GN=DPT PE=1 SV=2//6.98328e-117
XM_007989617.1	0.4382	4.74E-05	0.000444	sp|O75663|TIPRL_HUMAN TIP41-like protein OS=Homo sapiens GN=TIPRL PE=1 SV=2//1.59744e-173
XM_007989643.1	-1.1535	1.65E-10	3.08E-09	-//-
XM_007989649.1	-0.60623	0.003833	0.024695	sp|Q9UL54|TAOK2_HUMAN Serine/threonine-protein kinase TAO2 OS=Homo sapiens GN=TAOK2 PE=1 SV=2//0
XM_007989675.1	1.6159	2.45E-06	2.79E-05	sp|Q71H61|ILDR2_HUMAN Immunoglobulin-like domain-containing receptor 2 OS=Homo sapiens GN=ILDR2 PE=2 SV=1//0
XM_007989684.1	0.88572	3.35E-15	9.31E-14	sp|Q5RDB9|TADA1_PONAB Transcriptional adapter 1 OS=Pongo abelii GN=TADA1 PE=2 SV=1//0
XM_007989692.1	0.9849	2.50E-17	8.27E-16	sp|Q9BZX2|UCK2_HUMAN Uridine-cytidine kinase 2 OS=Homo sapiens GN=UCK2 PE=1 SV=1//3.42755e-174
XM_007989696.1	-1.279	9.56E-20	3.69E-18	sp|O14880|MGST3_HUMAN Microsomal glutathione S-transferase 3 OS=Homo sapiens GN=MGST3 PE=1 SV=1//6.28297e-104
XM_007989722.1	-0.91329	2.41E-17	8.02E-16	sp|O15539|RGS5_HUMAN Regulator of G-protein signaling 5 OS=Homo sapiens GN=RGS5 PE=1 SV=1//1.28657e-99
XM_007989723.1	2.2384	8.70E-07	1.06E-05	sp|Q4R525|RGS4_MACFA Regulator of G-protein signaling 4 OS=Macaca fascicularis GN=RGS4 PE=2 SV=1//4.16904e-133
XM_007989735.1	1.3686	6.25E-17	1.99E-15	sp|Q7L0Q8|RHOU_HUMAN Rho-related GTP-binding protein RhoU OS=Homo sapiens GN=RHOU PE=1 SV=1//3.2577e-142
XM_007989740.1	0.53779	0.000262	0.002164	sp|Q6IQ19|CCSAP_HUMAN Centriole, cilia and spindle-associated protein OS=Homo sapiens GN=CCSAP PE=1 SV=2//5.93844e-138
XM_007989747.1	-1.2105	0.000204	0.001715	sp|Q8WZ19|BACD1_HUMAN BTB/POZ domain-containing adapter for CUL3-mediated RhoA degradation protein 1 OS=Homo sapiens GN=KCTD13 PE=1 SV=1//0
XM_007989766.1	0.78013	6.36E-11	1.24E-09	sp|Q14746|COG2_HUMAN Conserved oligomeric Golgi complex subunit 2 OS=Homo sapiens GN=COG2 PE=1 SV=1//0
XM_007989769.1	0.45332	0.001294	0.0093	sp|Q9H425|CA198_HUMAN Uncharacterized protein C1orf198 OS=Homo sapiens GN=C1orf198 PE=1 SV=1//0
XM_007989770.1	-0.72476	0.000154	0.001321	sp|Q9H2C2|ARV1_HUMAN Protein ARV1 OS=Homo sapiens GN=ARV1 PE=2 SV=1//9.11548e-173
XM_007989792.1	-0.5101	1.63E-08	2.42E-07	sp|Q14764|MVP_HUMAN Major vault protein OS=Homo sapiens GN=MVP PE=1 SV=4//0
XM_007989794.1	0.54777	6.50E-05	0.000598	sp|Q8IYI6|EXOC8_HUMAN Exocyst complex component 8 OS=Homo sapiens GN=EXOC8 PE=1 SV=2//0
XM_007989798.1	0.66297	1.09E-07	1.46E-06	sp|Q4R599|TSNAX_MACFA Translin-associated protein X OS=Macaca fascicularis GN=TSNAX PE=2 SV=1//0
XM_007989818.1	-0.49435	1.98E-11	4.01E-10	sp|P55812|UBIM_PIG Ubiquitin-like protein FUBI OS=Sus scrofa GN=FAU PE=3 SV=1//3.18591e-43
XM_007989849.1	0.38391	0.000486	0.003837	sp|O95749|GGPPS_HUMAN Geranylgeranyl pyrophosphate synthase OS=Homo sapiens GN=GGPS1 PE=1 SV=1//0
XM_007989850.1	0.33321	0.003664	0.023675	sp|Q8NCR0|B3GL2_HUMAN UDP-GalNAc:beta-1,3-N-acetylgalactosaminyltransferase 2 OS=Homo sapiens GN=B3GALNT2 PE=1 SV=1//0
XM_007989871.1	0.94782	0.000737	0.005599	sp|Q99698|LYST_HUMAN Lysosomal-trafficking regulator OS=Homo sapiens GN=LYST PE=1 SV=3//0
XM_007989874.1	-1.239	6.87E-58	1.32E-55	sp|P14543|NID1_HUMAN Nidogen-1 OS=Homo sapiens GN=NID1 PE=1 SV=3//0
XM_007989918.1	0.53133	0.001053	0.007732	sp|Q9UHR6|ZNHI2_HUMAN Zinc finger HIT domain-containing protein 2 OS=Homo sapiens GN=ZNHIT2 PE=1 SV=1//0

XM_007989956.1	0.29094	0.004687	0.02961	sp|Q4KMP7|TB10B_HUMAN TBC1 domain family member 10B OS=Homo sapiens GN=TBC1D10B PE=1 SV=3//0
XM_007990065.1	0.75233	4.53E-15	1.25E-13	sp|Q99611|SPS2_HUMAN Selenide, water dikinase 2 OS=Homo sapiens GN=SEPHS2 PE=1 SV=3//0
XM_007990077.1	0.64135	2.13E-05	0.00021	sp|Q9H5H4|ZN768_HUMAN Zinc finger protein 768 OS=Homo sapiens GN=ZNF768 PE=1 SV=2//0
XM_007990088.1	1.3709	0.001757	0.012258	sp|Q8NG06|TRI58_HUMAN E3 ubiquitin-protein ligase TRIM58 OS=Homo sapiens GN=TRIM58 PE=2 SV=2//0
XM_007990107.1	1.5972	1.03E-34	8.51E-33	sp|Q96CS4|ZN689_HUMAN Zinc finger protein 689 OS=Homo sapiens GN=ZNF689 PE=2 SV=1//0
XM_007990155.1	-1.209	9.86E-25	5.11E-23	sp|Q6P823|H33_XENTR Histone H3.3 OS=Xenopus tropicalis GN=TGas113e22.1 PE=1 SV=3//1.70347e-75
XM_007990157.1	-0.72245	1.62E-11	3.32E-10	sp|Q14739|LBR_HUMAN Lamin-B receptor OS=Homo sapiens GN=LBR PE=1 SV=2//0
XM_007990158.1	0.29023	0.000314	0.002565	sp|Q9H7D7|WDR26_HUMAN WD repeat-containing protein 26 OS=Homo sapiens GN=WDR26 PE=1 SV=3//0
XM_007990168.1	-1.4424	0.005621	0.0348	-//-
XM_007990169.1	-1.2924	9.24E-08	1.25E-06	sp|P70289|PTPRV_MOUSE Receptor-type tyrosine-protein phosphatase V OS=Mus musculus GN=Ptprv PE=2 SV=1//3.40742e-06
XM_007990176.1	0.26772	0.000218	0.001822	sp|Q9Y520|PRC2C_HUMAN Protein PRRC2C OS=Homo sapiens GN=PRRC2C PE=1 SV=4//3.30855e-36
XM_007990181.1	0.95419	4.21E-11	8.34E-10	sp|Q8WYP5|ELYS_HUMAN Protein ELYS OS=Homo sapiens GN=AHCTF1 PE=1 SV=3//0
XM_007990227.1	0.75049	1.09E-13	2.71E-12	sp|Q9H0C5|BTBD1_HUMAN BTB/POZ domain-containing protein 1 OS=Homo sapiens GN=BTBD1 PE=1 SV=1//0
XM_007990230.1	1.0956	1.98E-06	2.29E-05	sp|Q8WUR7|CO040_HUMAN UPF0235 protein C15orf40 OS=Homo sapiens GN=C15orf40 PE=1 SV=2//6.5578e-76
XM_007990241.1	1.8973	1.62E-37	1.48E-35	sp|Q8TF30|WHAMM_HUMAN WASP homolog-associated protein with actin, membranes and microtubules OS=Homo sapiens GN=WHAMM PE=1 SV=2//0
XM_007990265.1	0.84716	7.52E-10	1.30E-08	sp|Q92610|ZN592_HUMAN Zinc finger protein 592 OS=Homo sapiens GN=ZNF592 PE=1 SV=2//0
XM_007990279.1	1.135	3.95E-13	9.36E-12	sp|Q6P4I2|WDR73_HUMAN WD repeat-containing protein 73 OS=Homo sapiens GN=WDR73 PE=1 SV=1//0
XM_007990297.1	-0.87511	1.03E-13	2.57E-12	sp|P62425|RL7A_RAT 60S ribosomal protein L7a OS=Rattus norvegicus GN=Rpl7a PE=1 SV=2//6.01933e-161
XM_007990326.1	3.2178	1.73E-44	2.00E-42	sp|Q5REE2|AEN_PONAB Apoptosis-enhancing nuclease OS=Pongo abelii GN=AEN PE=2 SV=1//0
XM_007990341.1	-0.53395	0.001001	0.00739	sp|Q4R2Y9|ABHD2_MACFA Abhydrolase domain-containing protein 2 OS=Macaca fascicularis GN=ABHD2 PE=2 SV=1//0
XM_007990386.1	0.65623	4.29E-05	0.000405	sp|Q5RDP9|AP3S2_PONAB AP-3 complex subunit sigma-2 OS=Pongo abelii GN=AP3S2 PE=2 SV=1//3.01873e-130
XM_007990400.1	-0.54201	0.000109	0.000964	sp|Q99828|CIB1_HUMAN Calcium and integrin-binding protein 1 OS=Homo sapiens GN=CIB1 PE=1 SV=4//6.57534e-115
XM_007990401.1	0.57463	4.09E-07	5.15E-06	sp|Q5R7E7|NGRN_PONAB Neugrin OS=Pongo abelii GN=NGRN PE=2 SV=2//8.54488e-170
XM_007990473.1	1.2721	6.79E-07	8.38E-06	sp|Q6NX45|ZN774_HUMAN Zinc finger protein 774 OS=Homo sapiens GN=ZNF774 PE=1 SV=2//0
XM_007990493.1	1.2608	1.15E-10	2.17E-09	-//-
XM_007990512.1	0.67136	5.50E-09	8.66E-08	sp|Q96FF9|CDCA5_HUMAN Sororin OS=Homo sapiens GN=CDCA5 PE=1 SV=1//3.10624e-134
XM_007990524.1	2.1847	6.67E-42	7.06E-40	sp|Q8NCT1|ARRD4_HUMAN Arrestin domain-containing protein 4 OS=Homo sapiens GN=ARRDC4 PE=2 SV=3//0
XM_007990538.1	0.64669	6.55E-09	1.02E-07	sp|O15015|ZN646_HUMAN Zinc finger protein 646 OS=Homo sapiens GN=ZNF646 PE=1 SV=1//0
XM_007990571.1	0.95749	1.23E-09	2.08E-08	sp|Q9H672|ASB7_HUMAN Ankyrin repeat and SOCS box protein 7 OS=Homo sapiens GN=ASB7 PE=1 SV=2//0
XM_007990584.1	0.65198	0.000126	0.001096	sp|Q9BQE4|SELS_HUMAN Selenoprotein S OS=Homo sapiens GN=VIMP PE=1 SV=3//1.33152e-79
XM_007990586.1	-0.55304	6.59E-09	1.02E-07	sp|O14874|BCKD_HUMAN [3-methyl-2-oxobutanoate dehydrogenase [lipoamide]] kinase, mitochondrial OS=Homo sapiens GN=BCKDK PE=1 SV=2//0
XM_007990632.1	0.47908	0.001812	0.012578	sp|Q8N0Z6|TTC5_HUMAN Tetratricopeptide repeat protein 5 OS=Homo sapiens GN=TTC5 PE=1 SV=2//0
XM_007990665.1	0.6995	0.000148	0.001273	sp|P00491|PNPH_HUMAN Purine nucleoside phosphorylase OS=Homo sapiens GN=PNP PE=1 SV=2//0
XM_007990694.1	0.48885	0.000107	0.000945	sp|Q9H7H0|MET17_HUMAN Methyltransferase-like protein 17, mitochondrial OS=Homo sapiens GN=METTL17 PE=1 SV=1//0
XM_007990748.1	0.3586	3.07E-06	3.46E-05	sp|Q9Y5B9|SP16H_HUMAN FACT complex subunit SPT16 OS=Homo sapiens GN=SUPT16H PE=1 SV=1//0
XM_007990753.1	0.63146	3.30E-10	5.96E-09	sp|Q5R6A9|TOX4_PONAB TOX high mobility group box family member 4 OS=Pongo abelii GN=TOX4 PE=2 SV=2//0
XM_007990771.1	0.59698	1.83E-10	3.41E-09	sp|Q8VD66|ABHD4_MOUSE Abhydrolase domain-containing protein 4 OS=Mus musculus GN=Abhd4 PE=2 SV=1//0

XM_007990777.1	-0.27234	0.008053	0.047608	sp|Q15070|OXA1L_HUMAN Mitochondrial inner membrane protein OXA1L OS=Homo sapiens GN=OXA1L PE=1 SV=3//0
XM_007990785.1	-0.53082	1.17E-07	1.56E-06	sp|P50281|MMP14_HUMAN Matrix metalloproteinase-14 OS=Homo sapiens GN=MMP14 PE=1 SV=3//0
XM_007990786.1	-0.47374	1.15E-06	1.38E-05	sp|Q7Z4F1|LRP10_HUMAN Low-density lipoprotein receptor-related protein 10 OS=Homo sapiens GN=LRP10 PE=1 SV=2//0
XM_007990792.1	0.91599	6.40E-09	9.97E-08	sp|Q86U06|RBM23_HUMAN Probable RNA-binding protein 23 OS=Homo sapiens GN=RBM23 PE=1 SV=1//3.97272e-13
XM_007990794.1	-0.56429	0.005878	0.036132	sp|O43294|TGFI1_HUMAN Transforming growth factor beta-1-induced transcript 1 protein OS=Homo sapiens GN=TGFB1I1 PE=1 SV=2//0
XM_007990812.1	1.6637	4.20E-89	1.67E-86	sp|Q96IF1|AJUBA_HUMAN LIM domain-containing protein ajuba OS=Homo sapiens GN=AJUBA PE=1 SV=1//0
XM_007990849.1	-0.51476	3.83E-09	6.15E-08	sp|Q28165|PABP2_BOVIN Polyadenylate-binding protein 2 OS=Bos taurus GN=PABPN1 PE=1 SV=3//1.22761e-101
XM_007990881.1	0.55083	0.002238	0.015223	sp|Q8NEJ9|NGDN_HUMAN Neuroguidin OS=Homo sapiens GN=NGDN PE=1 SV=1//8.65552e-151
XM_007990909.1	4.9873	2.53E-29	1.64E-27	sp|Q13268|DHRS2_HUMAN Dehydrogenase/reductase SDR family member 2, mitochondrial OS=Homo sapiens GN=DHRS2 PE=1 SV=4//2.75726e-160
XM_007990919.1	-1.2598	1.65E-05	0.000165	sp|A6NEY3|GG6L3_HUMAN Putative golgin subfamily A member 6-like protein 3 OS=Homo sapiens GN=GOLGA6L3 PE=5 SV=3//5.09112e-56
XM_007990921.1	1.7156	9.38E-49	1.30E-46	sp|Q7Z2Z1|TICRR_HUMAN Treslin OS=Homo sapiens GN=TICRR PE=1 SV=2//0
XM_007990993.1	0.63946	2.06E-13	4.99E-12	sp|Q13796|SHRM2_HUMAN Protein Shroom2 OS=Homo sapiens GN=SHROOM2 PE=1 SV=1//0
XM_007990999.1	1.0655	6.98E-12	1.47E-10	sp|Q9ULE0|WWC3_HUMAN Protein WWC3 OS=Homo sapiens GN=WWC3 PE=1 SV=3//0
XM_007991001.1	1.5437	5.65E-34	4.50E-32	sp|P51793|CLCN4_HUMAN H(+)/Cl(-) exchange transporter 4 OS=Homo sapiens GN=CLCN4 PE=1 SV=2//0
XM_007991023.1	0.39665	1.43E-05	0.000144	sp|P80723|BASP1_HUMAN Brain acid soluble protein 1 OS=Homo sapiens GN=BASP1 PE=1 SV=2//7.30213e-09
XM_007991039.1	-1.2752	4.35E-22	1.94E-20	sp|P20065|TYB4_MOUSE Thymosin beta-4 OS=Mus musculus GN=Tmsb4x PE=1 SV=1//4.97794e-22
XM_007991057.1	1.1261	8.25E-19	2.98E-17	sp|O75665|OFD1_HUMAN Oral-facial-digital syndrome 1 protein OS=Homo sapiens GN=OFD1 PE=1 SV=1//0
XM_007991116.1	1.3127	2.07E-10	3.81E-09	sp|Q9HBJ8|TMM27_HUMAN Collectrin OS=Homo sapiens GN=TMEM27 PE=1 SV=1//7.6521e-127
XM_007991136.1	-0.70316	6.50E-10	1.13E-08	sp|Q96A49|SYAP1_HUMAN Synapse-associated protein 1 OS=Homo sapiens GN=SYAP1 PE=1 SV=1//0
XM_007991257.1	-0.91824	2.37E-10	4.34E-09	sp|P51812|KS6A3_HUMAN Ribosomal protein S6 kinase alpha-3 OS=Homo sapiens GN=RPS6KA3 PE=1 SV=1//0
XM_007991281.1	0.61009	4.47E-08	6.26E-07	sp|O43462|MBTP2_HUMAN Membrane-bound transcription factor site-2 protease OS=Homo sapiens GN=MBTPS2 PE=1 SV=1//0
XM_007991284.1	-0.6398	0.000662	0.005085	sp|Q6PRD7|CEMP1_HUMAN Cementoblastoma-derived protein 1 OS=Homo sapiens GN=CEMP1 PE=2 SV=1//8.3979e-131
XM_007991288.1	0.72619	5.82E-10	1.02E-08	sp|Q9NXV2|KCTD5_HUMAN BTB/POZ domain-containing protein KCTD5 OS=Homo sapiens GN=KCTD5 PE=1 SV=1//4.04663e-27
XM_007991301.1	0.34015	7.97E-06	8.37E-05	sp|P21673|SAT1_HUMAN Diamine acetyltransferase 1 OS=Homo sapiens GN=SAT1 PE=1 SV=1//5.68647e-121
XM_007991322.1	0.39928	0.000167	0.001423	sp|P09884|DPOLA_HUMAN DNA polymerase alpha catalytic subunit OS=Homo sapiens GN=POLA1 PE=1 SV=2//0
XM_007991366.1	0.34172	0.004125	0.026401	sp|Q92793|CBP_HUMAN CREB-binding protein OS=Homo sapiens GN=CREBBP PE=1 SV=3//6.94862e-46
XM_007991374.1	0.54478	2.01E-09	3.31E-08	sp|Q96EY1|DNJA3_HUMAN DnaJ homolog subfamily A member 3, mitochondrial OS=Homo sapiens GN=DNAJA3 PE=1 SV=2//1.24163e-16
XM_007991402.1	-0.91659	1.80E-07	2.36E-06	sp|P41732|TSN7_HUMAN Tetraspanin-7 OS=Homo sapiens GN=TSPAN7 PE=1 SV=2//2.68481e-179
XM_007991403.1	1.3235	3.48E-34	2.80E-32	sp|Q9NPA3|M1IP1_HUMAN Mid1-interacting protein 1 OS=Homo sapiens GN=MID1IP1 PE=1 SV=1//1.92059e-122
XM_007991420.1	-0.50227	2.23E-06	2.56E-05	sp|Q5R563|RENR_PONAB Renin receptor OS=Pongo abelii GN=ATP6AP2 PE=2 SV=1//0
XM_007991421.1	1.1538	6.79E-36	5.85E-34	sp|Q8IWR0|Z3H7A_HUMAN Zinc finger CCCH domain-containing protein 7A OS=Homo sapiens GN=ZC3H7A PE=1 SV=1//0
XM_007991428.1	0.32413	0.001505	0.010686	sp|O60244|MED14_HUMAN Mediator of RNA polymerase II transcription subunit 14 OS=Homo sapiens GN=MED14 PE=1 SV=2//0
XM_007991433.1	0.79559	0.006865	0.041397	sp|O00571|DDX3X_HUMAN ATP-dependent RNA helicase DDX3X OS=Homo sapiens GN=DDX3X PE=1 SV=3//0
XM_007991453.1	1.9098	0.000415	0.003315	sp|Q5RE60|AOFA_PONAB Amine oxidase [flavin-containing] A OS=Pongo abelii GN=MAOA PE=2 SV=1//0
XM_007991495.1	0.6111	0.00055	0.004282	sp|Q86YC2|PALB2_HUMAN Partner and localizer of BRCA2 OS=Homo sapiens GN=PALB2 PE=1 SV=1//0
XM_007991509.1	-0.79137	4.22E-11	8.35E-10	sp|Q0MQJ3|NDUBB_PONPY NADH dehydrogenase [ubiquinone] 1 beta subcomplex subunit 11, mitochondrial OS=Pongo pygmaeus GN=NDUFB11 PE=2

				SV=1//3.91675e-89
XM_007991533.1	-1.842	1.19E-05	0.000122	sp|P52846|ST1A1_MACFA Sulfotransferase 1A1 OS=Macaca fascicularis GN=SULT1A1 PE=2 SV=1//3.07225e-32
XM_007991541.1	-0.48729	5.21E-05	0.000486	sp|P49061|TIMP1_PAPCY Metalloproteinase inhibitor 1 OS=Papio cynocephalus GN=TIMP1 PE=2 SV=1//1.43394e-136
XM_007991549.1	-0.64761	9.71E-10	1.66E-08	sp|Q9UBK9|UXT_HUMAN Protein UXT OS=Homo sapiens GN=UXT PE=1 SV=1//1.37874e-85
XM_007991553.1	0.71608	0.001121	0.008192	sp|P17025|ZN182_HUMAN Zinc finger protein 182 OS=Homo sapiens GN=ZNF182 PE=2 SV=2//0
XM_007991559.1	0.89543	3.42E-07	4.34E-06	sp|P51508|ZNF81_HUMAN Zinc finger protein 81 OS=Homo sapiens GN=ZNF81 PE=1 SV=3//0
XM_007991575.1	0.48486	0.008005	0.047373	-//-
XM_007991578.1	5.2931	5.82E-86	2.16E-83	sp|Q9UET6|TRM7_HUMAN Putative tRNA (cytidine(32)/guanosine(34)-2'-O)-methyltransferase OS=Homo sapiens GN=FTSJ1 PE=1 SV=2//0
XM_007991579.1	-0.47436	9.79E-05	0.000872	sp|Q9NRS6|SNX15_HUMAN Sorting nexin-15 OS=Homo sapiens GN=SNX15 PE=1 SV=1//1.43323e-165
XM_007991593.1	0.91632	3.40E-13	8.10E-12	sp|Q3MII6|TBC25_HUMAN TBC1 domain family member 25 OS=Homo sapiens GN=TBC1D25 PE=1 SV=2//0
XM_007991663.1	-0.69239	2.55E-07	3.30E-06	sp|Q4R4I9|PRAF2_MACFA PRA1 family protein 2 OS=Macaca fascicularis GN=PRAF2 PE=2 SV=1//1.19157e-64
XM_007991676.1	-0.69382	1.84E-14	4.84E-13	sp|Q04941|PLP2_HUMAN Proteolipid protein 2 OS=Homo sapiens GN=PLP2 PE=1 SV=1//1.59557e-102
XM_007991776.1	-1.0409	0.001331	0.009547	-//-
XM_007991781.1	-0.63914	1.75E-11	3.57E-10	sp|Q99714|HCD2_HUMAN 3-hydroxyacyl-CoA dehydrogenase type-2 OS=Homo sapiens GN=HSD17B10 PE=1 SV=3//6.45257e-180
XM_007991793.1	0.95888	1.02E-10	1.95E-09	sp|Q9NX05|F120C_HUMAN Constitutive coactivator of PPAR-gamma-like protein 2 OS=Homo sapiens GN=FAM120C PE=2 SV=3//4.41023e-09
XM_007991862.1	-0.49093	1.05E-06	1.27E-05	sp|Q9UHD9|UBQL2_HUMAN Ubiquilin-2 OS=Homo sapiens GN=UBQLN2 PE=1 SV=2//0
XM_007991871.1	-0.87444	8.48E-21	3.49E-19	sp|Q6P823|H33_XENTR Histone H3.3 OS=Xenopus tropicalis GN=TGas113e22.1 PE=1 SV=3//2.05964e-81
XM_007991884.1	-1.3349	2.21E-07	2.87E-06	sp|Q2TA37|ARL2_BOVIN ADP-ribosylation factor-like protein 2 OS=Bos taurus GN=ARL2 PE=1 SV=1//2.83742e-126
XM_007991899.1	0.6052	0.00153	0.01085	sp|Q5JTC6|AMER1_HUMAN APC membrane recruitment protein 1 OS=Homo sapiens GN=AMER1 PE=1 SV=2//0
XM_007991957.1	-1.0844	2.49E-35	2.12E-33	sp|P98172|EFNB1_HUMAN Ephrin-B1 OS=Homo sapiens GN=EFNB1 PE=1 SV=1//0
XM_007992006.1	-0.43431	0.006798	0.04112	sp|P98177|FOXO4_HUMAN Forkhead box protein O4 OS=Homo sapiens GN=FOXO4 PE=1 SV=5//0
XM_007992032.1	-0.37075	8.79E-06	9.16E-05	sp|Q9UKP3|ITBP2_HUMAN Integrin beta-1-binding protein 2 OS=Homo sapiens GN=ITGB1BP2 PE=1 SV=1//0
XM_007992033.1	0.57124	3.19E-09	5.16E-08	sp|P21675|TAF1_HUMAN Transcription initiation factor TFIID subunit 1 OS=Homo sapiens GN=TAF1 PE=1 SV=2//0
XM_007992050.1	-0.48159	0.003695	0.023854	sp|Q5HYW3|RGAG4_HUMAN Retrotransposon gag domain-containing protein 4 OS=Homo sapiens GN=RGAG4 PE=2 SV=1//2.92536e-49
XM_007992066.1	-1.5519	4.90E-09	7.78E-08	sp|Q9Y237|PIN4_HUMAN Peptidyl-prolyl cis-trans isomerase NIMA-interacting 4 OS=Homo sapiens GN=PIN4 PE=1 SV=1//3.77548e-67
XM_007992068.1	-0.60193	1.17E-06	1.40E-05	sp|P79103|RS4_BOVIN 40S ribosomal protein S4 OS=Bos taurus GN=RPS4 PE=2 SV=3//0
XM_007992094.1	-1.1703	3.78E-08	5.35E-07	sp|P36021|MOT8_HUMAN Monocarboxylate transporter 8 OS=Homo sapiens GN=SLC16A2 PE=1 SV=2//0
XM_007992119.1	-0.97425	2.60E-07	3.34E-06	sp|Q9BE18|MAGE1_MACFA Melanoma-associated antigen E1 OS=Macaca fascicularis GN=MAGEE1 PE=2 SV=1//0
XM_007992133.1	-0.4292	0.000287	0.002358	sp|Q9H0U3|MAGT1_HUMAN Magnesium transporter protein 1 OS=Homo sapiens GN=MAGT1 PE=1 SV=1//0
XM_007992134.1	-0.59222	4.86E-06	5.31E-05	sp|Q9H0U3|MAGT1_HUMAN Magnesium transporter protein 1 OS=Homo sapiens GN=MAGT1 PE=1 SV=1//5.23909e-14
XM_007992135.1	-1.199	5.35E-33	4.05E-31	sp|Q5R9K2|COX7B_PONAB Cytochrome c oxidase subunit 7B, mitochondrial OS=Pongo abelii GN=COX7B PE=3 SV=1//5.17038e-53
XM_007992136.1	-0.40342	0.003328	0.02172	sp|Q04656|ATP7A_HUMAN Copper-transporting ATPase 1 OS=Homo sapiens GN=ATP7A PE=1 SV=3//0
XM_007992139.1	-0.90245	2.24E-18	7.86E-17	sp|A5A6K4|PGK1_PANTR Phosphoglycerate kinase 1 OS=Pan troglodytes GN=PGK1 PE=2 SV=1//0
XM_007992154.1	0.42383	2.20E-06	2.53E-05	sp|Q2HJ94|DNJA2_BOVIN DnaJ homolog subfamily A member 2 OS=Bos taurus GN=DNAJA2 PE=2 SV=1//0
XM_007992160.1	-0.67578	0.007486	0.04459	sp|Q6RI45|BRWD3_HUMAN Bromodomain and WD repeat-containing protein 3 OS=Homo sapiens GN=BRWD3 PE=1 SV=2//3.92199e-37
XM_007992162.1	-0.80504	6.69E-11	1.29E-09	sp|Q6P823|H33_XENTR Histone H3.3 OS=Xenopus tropicalis GN=TGas113e22.1 PE=1 SV=3//2.76909e-89
XM_007992163.1	-0.9564	1.36E-18	4.83E-17	sp|Q5RFN7|SH3L1_PONAB SH3 domain-binding glutamic acid-rich-like protein OS=Pongo abelii GN=SH3BGRL PE=3 SV=1//2.34435e-70

XM_007992185.1	-0.72973	1.07E-13	2.67E-12	sp|Q8TB96|TIP_HUMAN T-cell immunomodulatory protein OS=Homo sapiens GN=ITFG1 PE=1 SV=1//0
XM_007992195.1	0.5152	8.19E-09	1.26E-07	sp|P24386|RAE1_HUMAN Rab proteins geranylgeranyltransferase component A 1 OS=Homo sapiens GN=CHM PE=1 SV=3//0
XM_007992331.1	-0.96144	0.001138	0.008287	sp|Q4R7Y2|RL10_MACFA 60S ribosomal protein L10 OS=Macaca fascicularis GN=RPL10 PE=2 SV=1//1.26979e-140
XM_007992366.1	0.62781	6.83E-09	1.05E-07	sp|Q2PG52|BEX1_MACFA Protein BEX1 OS=Macaca fascicularis GN=BEX1 PE=2 SV=1//1.23873e-63
XM_007992444.1	-0.36046	0.001657	0.01163	sp|Q6PEV8|F199X_HUMAN Protein FAM199X OS=Homo sapiens GN=FAM199X PE=1 SV=1//0
XM_007992468.1	-1.5556	0.003414	0.02225	sp|Q8IUQ4|SIAH1_HUMAN E3 ubiquitin-protein ligase SIAH1 OS=Homo sapiens GN=SIAH1 PE=1 SV=2//0
XM_007992469.1	-0.95551	5.97E-08	8.24E-07	sp|Q5RF74|RN128_PONAB E3 ubiquitin-protein ligase RNF128 OS=Pongo abelii GN=RNF128 PE=2 SV=1//0
XM_007992474.1	-0.86551	5.94E-25	3.12E-23	sp|P57739|CLD2_HUMAN Claudin-2 OS=Homo sapiens GN=CLDN2 PE=1 SV=1//8.22603e-112
XM_007992506.1	0.507	0.000406	0.003251	sp|P60892|PRPS1_RAT Ribose-phosphate pyrophosphokinase 1 OS=Rattus norvegicus GN=Prps1 PE=1 SV=2//0
XM_007992509.1	1.0632	2.52E-05	0.000246	sp|Q5RED5|T22D3_PONAB TSC22 domain family protein 3 OS=Pongo abelii GN=TSC22D3 PE=2 SV=1//9.2867e-79
XM_007992590.1	1.6751	2.80E-70	7.13E-68	sp|O75113|N4BP1_HUMAN NEDD4-binding protein 1 OS=Homo sapiens GN=N4BP1 PE=1 SV=4//0
XM_007992640.1	-0.70832	1.43E-06	1.69E-05	sp|Q02819|NUCB1_MOUSE Nucleobindin-1 OS=Mus musculus GN=Nucb1 PE=1 SV=2//1.06024e-55
XM_007992660.1	1.1459	0.000215	0.001797	sp|Q9BVJ6|UT14A_HUMAN U3 small nucleolar RNA-associated protein 14 homolog A OS=Homo sapiens GN=UTP14A PE=1 SV=1//0
XM_007992686.1	0.43879	0.005612	0.034751	sp|Q9Y388|RBMX2_HUMAN RNA-binding motif protein, X-linked 2 OS=Homo sapiens GN=RBMX2 PE=1 SV=2//3.55892e-142
XM_007992736.1	0.36876	1.30E-05	0.000132	sp|O75487|GPC4_HUMAN Glypican-4 OS=Homo sapiens GN=GPC4 PE=1 SV=4//0
XM_007992776.1	-0.89401	0.002785	0.018455	sp|A6ZKI3|F127A_HUMAN Protein FAM127A OS=Homo sapiens GN=FAM127A PE=1 SV=1//3.85805e-73
XM_007992777.1	-1.098	3.46E-12	7.51E-11	sp|Q17RB0|F127C_HUMAN Protein FAM127C OS=Homo sapiens GN=FAM127C PE=1 SV=1//8.28803e-61
XM_007992822.1	-0.49074	2.69E-05	0.000261	sp|Q5RB63|HTSF1_PONAB HIV Tat-specific factor 1 homolog OS=Pongo abelii GN=HTATSF1 PE=2 SV=1//1.47024e-17
XM_007992827.1	0.67345	1.57E-10	2.95E-09	sp|Q7Z4Q2|HEAT3_HUMAN HEAT repeat-containing protein 3 OS=Homo sapiens GN=HEATR3 PE=1 SV=2//0
XM_007992832.1	-1.4386	0.001771	0.012338	sp|O60481|ZIC3_HUMAN Zinc finger protein ZIC 3 OS=Homo sapiens GN=ZIC3 PE=1 SV=1//0
XM_007992835.1	1.0346	3.15E-05	0.000302	sp|Q6PJ61|FBX46_HUMAN F-box only protein 46 OS=Homo sapiens GN=FBXO46 PE=1 SV=3//0
XM_007992922.1	-0.36399	6.45E-06	6.88E-05	sp|P22304|IDS_HUMAN Iduronate 2-sulfatase OS=Homo sapiens GN=IDS PE=1 SV=1//0
XM_007992924.1	-1.3155	0.000495	0.003895	sp|P22304|IDS_HUMAN Iduronate 2-sulfatase OS=Homo sapiens GN=IDS PE=1 SV=1//2.07223e-48
XM_007993047.1	-0.90962	5.28E-05	0.000492	sp|P21810|PGS1_HUMAN Biglycan OS=Homo sapiens GN=BGN PE=1 SV=2//0
XM_007993075.1	-0.31422	5.00E-05	0.000468	sp|P28570|SC6A8_RAT Sodium- and chloride-dependent creatine transporter 1 OS=Rattus norvegicus GN=Slc6a8 PE=2 SV=1//0
XM_007993086.1	-0.86684	7.47E-14	1.88E-12	sp|Q5REH6|SSRD_PONAB Translocon-associated protein subunit delta OS=Pongo abelii GN=SSR4 PE=2 SV=2//2.2526e-104
XM_007993126.1	-0.625	6.73E-11	1.30E-09	sp|Q95LG8|MECP2_MACFA Methyl-CpG-binding protein 2 OS=Macaca fascicularis GN=MECP2 PE=2 SV=1//5.80766e-31
XM_007993136.1	0.40204	0.00017	0.001448	sp|P50402|EMD_HUMAN Emerin OS=Homo sapiens GN=EMD PE=1 SV=1//2.54696e-125
XM_007993146.1	-0.55027	1.21E-06	1.44E-05	sp|Q14657|LAGE3_HUMAN EKC/KEOPS complex subunit LAGE3 OS=Homo sapiens GN=LAGE3 PE=1 SV=2//3.68707e-53
XM_007993147.1	0.44791	4.41E-05	0.000414	sp|P11441|UBL4A_HUMAN Ubiquitin-like protein 4A OS=Homo sapiens GN=UBL4A PE=1 SV=1//7.1022e-87
XM_007993180.1	1.0415	2.22E-29	1.44E-27	sp|O60832|DKC1_HUMAN H/ACA ribonucleoprotein complex subunit 4 OS=Homo sapiens GN=DKC1 PE=1 SV=3//0
XM_007993188.1	-0.33179	0.006935	0.041765	sp|Q7YRC0|FUND2_MACMU FUN14 domain-containing protein 2 OS=Macaca mulatta GN=FUNDC2 PE=2 SV=1//5.1924e-124
XM_007993189.1	0.28229	0.007473	0.044525	sp|P23610|F8I2_HUMAN Factor VIII intron 22 protein OS=Homo sapiens GN=F8A1 PE=1 SV=2//1.91874e-129
XM_007993237.1	-0.5356	1.09E-06	1.31E-05	sp|P14209|CD99_HUMAN CD99 antigen OS=Homo sapiens GN=CD99 PE=1 SV=1//1.26536e-07
XM_007993252.1	0.5536	6.30E-06	6.74E-05	sp|P23607|ZFA_MOUSE Zinc finger autosomal protein OS=Mus musculus GN=Zfa PE=3 SV=1//1.36813e-10
XM_007993262.1	1.206	3.55E-06	3.96E-05	sp|Q2M3X9|ZN674_HUMAN Zinc finger protein 674 OS=Homo sapiens GN=ZNF674 PE=2 SV=1//0
XM_007993278.1	-0.40503	2.72E-08	3.92E-07	sp|Q5RFL9|NONO_PONAB Non-POU domain-containing octamer-binding protein

				OS=Pongo abelii GN=NONO PE=2 SV=3//4.04231e-175
XM_007993299.1	-0.49182	0.000102	0.000905	sp|O15347|HMGB3_HUMAN High mobility group protein B3 OS=Homo sapiens GN=HMGB3 PE=1 SV=4//3.02051e-53
XM_007993302.1	-0.46663	1.40E-06	1.65E-05	sp|P51572|BAP31_HUMAN B-cell receptor-associated protein 31 OS=Homo sapiens GN=BCAP31 PE=1 SV=3//4.69752e-112
XM_007993306.1	0.72948	0.000362	0.002924	sp|Q8VGS3|O1019_MOUSE Olfactory receptor 1019 OS=Mus musculus GN=Olfr1019 PE=3 SV=1//1.14506e-81
XM_007993327.1	-0.26708	0.001429	0.010201	sp|Q5RBP4|EHD1_PONAB EH domain-containing protein 1 OS=Pongo abelii GN=EHD1 PE=2 SV=1//0
XM_007993330.1	-0.78262	9.56E-05	0.000853	sp|Q7L5N7|PCAT2_HUMAN Lysophosphatidylcholine acyltransferase 2 OS=Homo sapiens GN=LPCAT2 PE=1 SV=1//0
XM_007993346.1	-0.2632	0.001208	0.008752	sp|Q9UKV5|AMFR_HUMAN E3 ubiquitin-protein ligase AMFR OS=Homo sapiens GN=AMFR PE=1 SV=2//0
XM_007993347.1	-0.39914	0.000284	0.002331	sp|Q5RAI8|CPSF5_PONAB Cleavage and polyadenylation specificity factor subunit 5 OS=Pongo abelii GN=NUDT21 PE=2 SV=1//2.90573e-161
XM_007993350.1	-0.47645	2.24E-05	0.00022	sp|Q9BXC9|BBS2_HUMAN Bardet-Biedl syndrome 2 protein OS=Homo sapiens GN=BBS2 PE=1 SV=1//0
XM_007993354.1	-0.90319	1.41E-12	3.15E-11	sp|P68303|MT2_MACFA Metallothionein-2 OS=Macaca fascicularis GN=MT2 PE=3 SV=1//3.03936e-09
XM_007993356.1	-0.24837	0.00255	0.017067	-//-
XM_007993357.1	-0.84223	1.94E-10	3.59E-09	-//-
XM_007993398.1	1.7308	6.03E-21	2.50E-19	sp|Q15011|HERP1_HUMAN Homocysteine-responsive endoplasmic reticulum-resident ubiquitin-like domain member 1 protein OS=Homo sapiens GN=HERPUD1 PE=1 SV=1//0
XM_007993433.1	-0.65362	8.64E-05	0.000779	sp|P78423|X3CL1_HUMAN Fractalkine OS=Homo sapiens GN=CX3CL1 PE=1 SV=1//0
XM_007993437.1	0.3281	0.001883	0.012993	sp|Q6FI81|CPIN1_HUMAN Anamorsin OS=Homo sapiens GN=CIAPIN1 PE=1 SV=2//0
XM_007993442.1	0.44254	2.77E-05	0.000269	sp|P19387|RPB3_HUMAN DNA-directed RNA polymerase II subunit RPB3 OS=Homo sapiens GN=POLR2C PE=1 SV=2//0
XM_007993495.1	-1.1815	4.40E-16	1.30E-14	sp|P51511|MMP15_HUMAN Matrix metalloproteinase-15 OS=Homo sapiens GN=MMP15 PE=1 SV=1//0
XM_007993543.1	-0.37488	4.86E-06	5.31E-05	sp|Q4R559|AATM_MACFA Aspartate aminotransferase, mitochondrial OS=Macaca fascicularis GN=GOT2 PE=2 SV=1//0
XM_007993562.1	-0.51772	2.16E-10	3.96E-09	sp|P37837|TALDO_HUMAN Transaldolase OS=Homo sapiens GN=TALDO1 PE=1 SV=2//0
XM_007993580.1	-1.4596	0.006405	0.039057	sp|Q96MX0|CKLF3_HUMAN CKLF-like MARVEL transmembrane domain-containing protein 3 OS=Homo sapiens GN=CMTM3 PE=1 SV=1//2.55055e-82
XM_007993618.1	1.1335	2.88E-05	0.000279	sp|O00748|EST2_HUMAN Cocaine esterase OS=Homo sapiens GN=CES2 PE=1 SV=1//0
XM_007993619.1	1.688	1.59E-23	7.77E-22	sp|O00748|EST2_HUMAN Cocaine esterase OS=Homo sapiens GN=CES2 PE=1 SV=1//0
XM_007993641.1	0.80262	5.31E-21	2.21E-19	sp|Q6PCD5|RFWD3_HUMAN E3 ubiquitin-protein ligase RFWD3 OS=Homo sapiens GN=RFWD3 PE=1 SV=3//0
XM_007993642.1	-0.7574	2.03E-17	6.77E-16	sp|Q92896|GSLG1_HUMAN Golgi apparatus protein 1 OS=Homo sapiens GN=GLG1 PE=1 SV=2//1.80317e-12
XM_007993648.1	0.79526	1.99E-07	2.60E-06	sp|Q5RKV6|EXOS6_HUMAN Exosome complex component MTR3 OS=Homo sapiens GN=EXOSC6 PE=1 SV=1//4.26302e-87
XM_007993649.1	1.2648	1.13E-55	2.03E-53	sp|P49588|SYAC_HUMAN Alanine--tRNA ligase, cytoplasmic OS=Homo sapiens GN=AARS PE=1 SV=2//0
XM_007993650.1	0.54779	4.39E-07	5.51E-06	sp|Q9NUU7|DD19A_HUMAN ATP-dependent RNA helicase DDX19A OS=Homo sapiens GN=DDX19A PE=1 SV=1//0
XM_007993675.1	0.69565	2.28E-20	9.10E-19	sp|Q15393|SF3B3_HUMAN Splicing factor 3B subunit 3 OS=Homo sapiens GN=SF3B3 PE=1 SV=4//0
XM_007993759.1	0.9483	2.44E-28	1.53E-26	sp|P0C7T5|ATX1L_HUMAN Ataxin-1-like OS=Homo sapiens GN=ATXN1L PE=1 SV=1//0
XM_007993773.1	0.48195	2.82E-07	3.61E-06	sp|Q92620|PRP16_HUMAN Pre-mRNA-splicing factor ATP-dependent RNA helicase PRP16 OS=Homo sapiens GN=DHX38 PE=1 SV=2//0
XM_007993794.1	-1.0702	8.07E-34	6.40E-32	sp|Q5RD31|NQO1_PONAB NAD(P)H dehydrogenase [quinone] 1 OS=Pongo abelii GN=NQO1 PE=2 SV=1//0
XM_007993795.1	1.3306	3.85E-42	4.13E-40	sp|Q4R537|NOB1_MACFA RNA-binding protein NOB1 OS=Macaca fascicularis GN=NOB1 PE=2 SV=1//0
XM_007993815.1	0.88379	3.09E-08	4.43E-07	sp|Q96MW5|COG8_HUMAN Conserved oligomeric Golgi complex subunit 8 OS=Homo sapiens GN=COG8 PE=1 SV=2//0
XM_007993816.1	1.2901	2.52E-26	1.42E-24	sp|Q5R9J1|NIP7_PONAB 60S ribosome subunit biogenesis protein NIP7 homolog OS=Pongo abelii GN=NIP7 PE=2 SV=1//1.02812e-125
XM_007993819.1	-0.76048	7.41E-12	1.56E-10	sp|Q13425|SNTB2_HUMAN Beta-2-syntrophin OS=Homo sapiens GN=SNTB2 PE=1 SV=1//0
XM_007993839.1	0.88065	0.002827	0.018698	sp|P22223|CADH3_HUMAN Cadherin-3 OS=Homo sapiens GN=CDH3 PE=1 SV=2//0
XM_007993860.1	1.323	1.38E-19	5.31E-18	sp|Q96CW6|S7A6O_HUMAN Probable RNA polymerase II nuclear localization protein SLC7A6OS OS=Homo sapiens GN=SLC7A6OS PE=1 SV=2//2.61259e-151
XM_007993874.1	#NAME?	0.00597	0.03662	sp|Q8NCC3|PAG15_HUMAN Group XV phospholipase A2 OS=Homo sapiens GN=PLA2G15 PE=1 SV=2//0
XM_007993875.1	-2.6494	0.0036	0.023275	sp|Q8NCC3|PAG15_HUMAN Group XV phospholipase A2 OS=Homo sapiens GN=PLA2G15

				PE=1 SV=2//0
XM_007993883.1	1.2669	3.07E-12	6.69E-11	sp|Q4R4T6|DDX28_MACFA Probable ATP-dependent RNA helicase DDX28 OS=Macaca fascicularis GN=DDX28 PE=2 SV=1//0
XM_007993908.1	0.44456	1.70E-05	0.000169	sp|P11801|KPSH1_HUMAN Serine/threonine-protein kinase H1 OS=Homo sapiens GN=PSKH1 PE=1 SV=4//0
XM_007993943.1	0.60547	0.000117	0.001027	sp|Q96AP0|ACD_HUMAN Adrenocortical dysplasia protein homolog OS=Homo sapiens GN=ACD PE=1 SV=3//0
XM_007993947.1	-0.40663	1.10E-05	0.000113	sp|Q9UI30|TR112_HUMAN Multifunctional methyltransferase subunit TRM112-like protein OS=Homo sapiens GN=TRMT112 PE=1 SV=1//2.12695e-78
XM_007994012.1	0.48343	0.008086	0.04779	sp|Q9BTX3|TM208_HUMAN Transmembrane protein 208 OS=Homo sapiens GN=TMEM208 PE=1 SV=1//1.96876e-112
XM_007994091.1	-0.61583	1.09E-06	1.32E-05	sp|Q96C90|PP14B_HUMAN Protein phosphatase 1 regulatory subunit 14B OS=Homo sapiens GN=PPP1R14B PE=1 SV=3//2.5036e-63
XM_007994113.1	1.3844	4.44E-37	4.00E-35	sp|Q4R4I0|TE2IP_MACFA Telomeric repeat-binding factor 2-interacting protein 1 OS=Macaca fascicularis GN=TERF2IP PE=2 SV=1//0
XM_007994132.1	1.9752	5.50E-09	8.66E-08	sp|Q8TE60|ATS18_HUMAN A disintegrin and metalloproteinase with thrombospondin motifs 18 OS=Homo sapiens GN=ADAMTS18 PE=1 SV=3//0
XM_007994189.1	0.91838	3.24E-23	1.57E-21	sp|P37059|DHB2_HUMAN Estradiol 17-beta-dehydrogenase 2 OS=Homo sapiens GN=HSD17B2 PE=1 SV=1//0
XM_007994197.1	-0.51827	5.78E-07	7.18E-06	sp|Q5RDI2|HSBP1_PONAB Heat shock factor-binding protein 1 OS=Pongo abelii GN=HSBP1 PE=3 SV=1//1.62402e-44
XM_007994198.1	1.2679	7.14E-18	2.44E-16	sp|O95822|DCMC_HUMAN Malonyl-CoA decarboxylase, mitochondrial OS=Homo sapiens GN=MLYCD PE=1 SV=3//0
XM_007994199.1	1.9376	4.54E-18	1.57E-16	sp|Q9UJX0|OSGI1_HUMAN Oxidative stress-induced growth inhibitor 1 OS=Homo sapiens GN=OSGIN1 PE=1 SV=3//0
XM_007994241.1	1.0831	2.06E-19	7.77E-18	sp|Q9H0B8|CRLD2_HUMAN Cysteine-rich secretory protein LCCL domain-containing 2 OS=Homo sapiens GN=CRISPLD2 PE=2 SV=1//0
XM_007994243.1	0.7638	2.11E-19	7.96E-18	sp|Q9NXF8|ZDHC7_HUMAN Palmitoyltransferase ZDHHC7 OS=Homo sapiens GN=ZDHHC7 PE=1 SV=2//0
XM_007994261.1	0.85252	2.60E-08	3.76E-07	sp|Q9Y248|PSF2_HUMAN DNA replication complex GINS protein PSF2 OS=Homo sapiens GN=GINS2 PE=1 SV=1//1.76342e-125
XM_007994295.1	0.36217	4.30E-05	0.000406	sp|Q9GZQ8|MLP3B_HUMAN Microtubule-associated proteins 1A/1B light chain 3B OS=Homo sapiens GN=MAP1LC3B PE=1 SV=3//2.83382e-76
XM_007994301.1	0.79893	1.88E-08	2.77E-07	sp|Q5R8W1|KLDC4_PONAB Kelch domain-containing protein 4 OS=Pongo abelii GN=KLHDC4 PE=2 SV=1//0
XM_007994317.1	2.1673	6.49E-08	8.94E-07	sp|Q8N9N5|BANP_HUMAN Protein BANP OS=Homo sapiens GN=BANP PE=1 SV=3//0
XM_007994335.1	-1.1476	9.37E-38	8.62E-36	sp|P13498|CY24A_HUMAN Cytochrome b-245 light chain OS=Homo sapiens GN=CYBA PE=1 SV=3//2.32378e-101
XM_007994351.1	0.54936	0.000131	0.001134	sp|Q9H211|CDT1_HUMAN DNA replication factor Cdt1 OS=Homo sapiens GN=CDT1 PE=1 SV=3//0
XM_007994358.1	-0.53808	1.06E-06	1.28E-05	sp|P34059|GALNS_HUMAN N-acetylgalactosamine-6-sulfatase OS=Homo sapiens GN=GALNS PE=1 SV=1//0
XM_007994400.1	-0.35457	7.56E-06	7.97E-05	sp|Q96FW1|OTUB1_HUMAN Ubiquitin thioesterase OTUB1 OS=Homo sapiens GN=OTUB1 PE=1 SV=2//0
XM_007994408.1	-0.51759	1.91E-06	2.21E-05	sp|Q5R605|CHM1A_PONAB Charged multivesicular body protein 1a OS=Pongo abelii GN=CHMP1A PE=2 SV=1//8.60747e-112
XM_007994420.1	-0.53607	9.78E-12	2.04E-10	sp|P26373|RL13_HUMAN 60S ribosomal protein L13 OS=Homo sapiens GN=RPL13 PE=1 SV=4//3.50371e-124
XM_007994451.1	-1.4726	2.38E-06	2.71E-05	sp|Q9H9R9|DBND1_HUMAN Dysbindin domain-containing protein 1 OS=Homo sapiens GN=DBNDD1 PE=1 SV=2//2.09067e-94
XM_007994452.1	0.73417	1.88E-06	2.19E-05	sp|Q920A7|AFG31_MOUSE AFG3-like protein 1 OS=Mus musculus GN=Afg3l1 PE=2 SV=2//1.8048e-56
XM_007994457.1	0.52679	0.000179	0.001525	sp|O95995|GAS8_HUMAN Growth arrest-specific protein 8 OS=Homo sapiens GN=GAS8 PE=1 SV=1//0
XM_007994460.1	-1.6673	0.007007	0.042107	-//-
XM_007994467.1	1.6691	4.46E-59	9.04E-57	sp|Q8NDF8|PAPD5_HUMAN Non-canonical poly(A) RNA polymerase PAPD5 OS=Homo sapiens GN=PAPD5 PE=1 SV=2//0
XM_007994475.1	0.69895	4.20E-09	6.72E-08	sp|Q5R9F5|S38A7_PONAB Putative sodium-coupled neutral amino acid transporter 7 OS=Pongo abelii GN=SLC38A7 PE=2 SV=1//0
XM_007994476.1	0.36743	6.30E-05	0.000581	sp|Q8NCN5|PDPR_HUMAN Pyruvate dehydrogenase phosphatase regulatory subunit, mitochondrial OS=Homo sapiens GN=PDPR PE=1 SV=2//0
XM_007994514.1	-1.3132	0.001183	0.008595	sp|Q6ZTW0|TPGS1_HUMAN Tubulin polyglutamylase complex subunit 1 OS=Homo sapiens GN=TPGS1 PE=2 SV=2//9.81816e-176
XM_007994525.1	0.5846	1.37E-09	2.31E-08	sp|Q9Y2X0|MED16_HUMAN Mediator of RNA polymerase II transcription subunit 16 OS=Homo sapiens GN=MED16 PE=1 SV=2//2.46122e-93
XM_007994527.1	-0.71771	0.001592	0.011237	sp|P00746|CFAD_HUMAN Complement factor D OS=Homo sapiens GN=CFD PE=1 SV=5//4.66844e-161
XM_007994551.1	1.2885	1.22E-26	6.99E-25	sp|O95633|FSTL3_HUMAN Follistatin-related protein 3 OS=Homo sapiens GN=FSTL3 PE=1 SV=1//4.93589e-154
XM_007994572.1	-0.38231	0.007264	0.043457	sp|Q5R587|RPAB1_PONAB DNA-directed RNA polymerases I, II, and III subunit

				RPABC1 OS=Pongo abelii GN=POLR2E PE=2 SV=1//2.73126e-134
XM_007994574.1	-0.72706	4.55E-22	2.02E-20	sp|Q4AEH2|GPX4_PONPY Phospholipid hydroperoxide glutathione peroxidase, mitochondrial OS=Pongo pygmaeus GN=GPX4 PE=2 SV=2//1.29415e-118
XM_007994582.1	-0.66298	8.23E-13	1.88E-11	sp|P30049|ATPD_HUMAN ATP synthase subunit delta, mitochondrial OS=Homo sapiens GN=ATP5D PE=1 SV=2//4.57919e-66
XM_007994583.1	1.5883	5.35E-14	1.36E-12	sp|Q8N350|DOS_HUMAN Protein Dos OS=Homo sapiens GN=DOS PE=1 SV=2//0
XM_007994591.1	-1.8188	1.24E-16	3.85E-15	sp|O43921|EFNA2_HUMAN Ephrin-A2 OS=Homo sapiens GN=EFNA2 PE=1 SV=1//1.14912e-123
XM_007994599.1	-0.96269	0.000873	0.006519	sp|Q2TAK8|MUM1_HUMAN PWWP domain-containing protein MUM1 OS=Homo sapiens GN=MUM1 PE=1 SV=3//2.82292e-144
XM_007994615.1	-0.90204	0.005601	0.034693	sp|Q96EP5|DAZP1_HUMAN DAZ-associated protein 1 OS=Homo sapiens GN=DAZAP1 PE=1 SV=1//4.40944e-15
XM_007994618.1	0.55353	0.001907	0.013139	sp|Q9UFG5|CS025_HUMAN UPF0449 protein C19orf25 OS=Homo sapiens GN=C19orf25 PE=1 SV=2//4.5391e-62
XM_007994622.1	-0.82291	0.002618	0.017476	sp|Q6UW60|PCSK4_HUMAN Proprotein convertase subtilisin/kexin type 4 OS=Homo sapiens GN=PCSK4 PE=2 SV=2//0
XM_007994672.1	-4.7309	0.002503	0.016766	sp|Q8NCQ2|CNAS1_HUMAN Uncharacterized protein CSNK1G2-AS1 OS=Homo sapiens GN=CSNK1G2-AS1 PE=2 SV=2//2.60359e-40
XM_007994685.1	-1.2076	4.57E-11	9.02E-10	sp|Q9UK45|LSM7_HUMAN U6 snRNA-associated Sm-like protein LSm7 OS=Homo sapiens GN=LSM7 PE=1 SV=1//9.18191e-61
XM_007994690.1	-0.49491	5.69E-10	9.98E-09	sp|P54368|OAZ1_HUMAN Ornithine decarboxylase antizyme 1 OS=Homo sapiens GN=OAZ1 PE=1 SV=3//2.69006e-136
XM_007994699.1	0.61387	0.000854	0.006386	sp|Q9NW61|PKHJ1_HUMAN Pleckstrin homology domain-containing family J member 1 OS=Homo sapiens GN=PLEKHJ1 PE=2 SV=1//1.39958e-88
XM_007994705.1	0.23886	0.002183	0.014892	sp|Q865S1|AP3D1_BOVIN AP-3 complex subunit delta-1 OS=Bos taurus GN=AP3D1 PE=1 SV=2//0
XM_007994716.1	3.4429	0	0	sp|O75293|GA45B_HUMAN Growth arrest and DNA damage-inducible protein GADD45 beta OS=Homo sapiens GN=GADD45B PE=1 SV=1//2.20633e-88
XM_007994725.1	1.3414	3.99E-07	5.03E-06	sp|Q86TJ5|ZN554_HUMAN Zinc finger protein 554 OS=Homo sapiens GN=ZNF554 PE=2 SV=1//0
XM_007994749.1	1.0691	6.55E-09	1.02E-07	sp|Q15935|ZNF77_HUMAN Zinc finger protein 77 OS=Homo sapiens GN=ZNF77 PE=2 SV=2//2.72373e-13
XM_007994750.1	-1.8497	0.006942	0.041794	sp|P63003|AES_RAT Amino-terminal enhancer of split OS=Rattus norvegicus GN=Aes PE=2 SV=1//7.69804e-101
XM_007994753.1	-0.55754	7.89E-10	1.36E-08	sp|P29992|GNA11_HUMAN Guanine nucleotide-binding protein subunit alpha-11 OS=Homo sapiens GN=GNA11 PE=1 SV=2//0
XM_007994811.1	-0.35999	4.14E-05	0.000391	sp|Q5R611|HRSL3_PONAB HRAS-like suppressor 3 OS=Pongo abelii GN=PLA2G16 PE=2 SV=1//2.29684e-82
XM_007994833.1	-1.2458	7.09E-08	9.71E-07	sp|Q3TV65|MPND_MOUSE MPN domain-containing protein OS=Mus musculus GN=Mpnd PE=2 SV=2//0
XM_007994863.1	2.0375	0.007227	0.043259	sp|Q96DT0|LEG12_HUMAN Galectin-12 OS=Homo sapiens GN=LGALS12 PE=1 SV=1//0
XM_007994866.1	-0.55346	3.15E-09	5.12E-08	sp|P36507|MP2K2_HUMAN Dual specificity mitogen-activated protein kinase kinase 2 OS=Homo sapiens GN=MAP2K2 PE=1 SV=1//0
XM_007994869.1	-0.25187	0.000224	0.001875	sp|Q5R8Z3|EF2_PONAB Elongation factor 2 OS=Pongo abelii GN=EEF2 PE=2 SV=3//0
XM_007994881.1	0.25377	0.007375	0.044005	sp|Q86TI2|DPP9_HUMAN Dipeptidyl peptidase 9 OS=Homo sapiens GN=DPP9 PE=1 SV=3//0
XM_007994882.1	0.86955	1.91E-16	5.85E-15	sp|Q9BSK4|FEM1A_HUMAN Protein fem-1 homolog A OS=Homo sapiens GN=FEM1A PE=1 SV=1//0
XM_007994883.1	1.4387	2.49E-11	5.02E-10	sp|Q8IUC6|TCAM1_HUMAN TIR domain-containing adapter molecule 1 OS=Homo sapiens GN=TICAM1 PE=1 SV=1//0
XM_007994925.1	-1.9476	0.002663	0.017729	sp|Q2ACH7|MIC13_MACFA MICOS complex subunit MIC13 OS=Macaca fascicularis GN=MIC13 PE=3 SV=1//2.97055e-78
XM_007994933.1	-0.52956	1.19E-12	2.69E-11	sp|Q5RAZ9|RL36_PONAB 60S ribosomal protein L36 OS=Pongo abelii GN=RPL36 PE=3 SV=3//2.98786e-61
XM_007994938.1	0.6776	2.36E-08	3.43E-07	sp|Q96G46|DUS3L_HUMAN tRNA-dihydrouridine(47) synthase [NAD(P)(+)]-like OS=Homo sapiens GN=DUS3L PE=1 SV=2//0
XM_007994941.1	-1.2189	5.06E-06	5.51E-05	sp|Q99748|NRTN_HUMAN Neurturin OS=Homo sapiens GN=NRTN PE=1 SV=1//6.75559e-64
XM_007994957.1	-1.3818	4.43E-11	8.76E-10	sp|Q13938|CAYP1_HUMAN Calcyphosin OS=Homo sapiens GN=CAPS PE=1 SV=1//3.63293e-125
XM_007994958.1	-0.64319	2.69E-10	4.88E-09	sp|Q0MQC0|NDUAB_PANTR NADH dehydrogenase [ubiquinone] 1 alpha subcomplex subunit 11 OS=Pan troglodytes GN=NDUFA11 PE=2 SV=3//2.83413e-81
XM_007994971.1	-1.1032	2.84E-10	5.14E-09	sp|Q9BT30|ALKB7_HUMAN Alpha-ketoglutarate-dependent dioxygenase alkB homolog 7, mitochondrial OS=Homo sapiens GN=ALKBH7 PE=1 SV=1//7.09125e-122
XM_007994985.1	-0.67312	0.000181	0.001539	sp|Q8IV53|DEN1C_HUMAN DENN domain-containing protein 1C OS=Homo sapiens GN=DENND1C PE=1 SV=1//0
XM_007994996.1	-1.1949	1.85E-09	3.06E-08	sp|Q9D6F9|TBB4A_MOUSE Tubulin beta-4A chain OS=Mus musculus GN=Tubb4a PE=1 SV=3//0
XM_007994997.1	-1.4688	1.23E-13	3.04E-12	sp|Q9D6F9|TBB4A_MOUSE Tubulin beta-4A chain OS=Mus musculus GN=Tubb4a PE=1

				SV=3//0
XM_007995061.1	-0.49868	2.62E-05	0.000255	sp|Q15833|STXB2_HUMAN Syntaxin-binding protein 2 OS=Homo sapiens GN=STXBP2 PE=1 SV=2//0
XM_007995065.1	-1.402	5.84E-05	0.00054	sp|P0DJ07|PT100_HUMAN Protein PET100 homolog, mitochondrial OS=Homo sapiens GN=PET100 PE=1 SV=1//4.22855e-28
XM_007995087.1	0.92293	2.98E-20	1.18E-18	sp|Q6RFH5|WDR74_HUMAN WD repeat-containing protein 74 OS=Homo sapiens GN=WDR74 PE=1 SV=1//0
XM_007995093.1	#NAME?	0.001603	0.011302	sp|Q9H6K5|PRR36_HUMAN Proline-rich protein 36 OS=Homo sapiens GN=PRR36 PE=2 SV=2//4.51905e-85
XM_007995107.1	0.35248	0.000502	0.003943	sp|O43615|TIM44_HUMAN Mitochondrial import inner membrane translocase subunit TIM44 OS=Homo sapiens GN=TIMM44 PE=1 SV=2//0
XM_007995108.1	4.5447	0.007795	0.046232	sp|P08195|4F2_HUMAN 4F2 cell-surface antigen heavy chain OS=Homo sapiens GN=SLC3A2 PE=1 SV=3//0
XM_007995117.1	-0.72124	0.00074	0.005616	sp|P62859|RS28_RAT 40S ribosomal protein S28 OS=Rattus norvegicus GN=Rps28 PE=1 SV=1//2.34819e-31
XM_007995124.1	-0.66865	1.19E-11	2.48E-10	sp|O35509|RB11B_RAT Ras-related protein Rab-11B OS=Rattus norvegicus GN=Rab11b PE=1 SV=4//2.77415e-155
XM_007995142.1	0.6383	9.35E-05	0.000836	sp|Q96NG5|ZN558_HUMAN Zinc finger protein 558 OS=Homo sapiens GN=ZNF558 PE=2 SV=1//0
XM_007995149.1	1.6113	7.03E-13	1.63E-11	sp|Q96PQ6|ZN317_HUMAN Zinc finger protein 317 OS=Homo sapiens GN=ZNF317 PE=1 SV=2//0
XM_007995151.1	0.30295	0.000905	0.006735	sp|Q9UBU9|NXF1_HUMAN Nuclear RNA export factor 1 OS=Homo sapiens GN=NXF1 PE=1 SV=1//0
XM_007995192.1	-0.95032	5.17E-22	2.30E-20	sp|Q791B0|UBL5_PSAOB Ubiquitin-like protein 5 OS=Psammomys obesus GN=UBL5 PE=3 SV=1//5.28142e-47
XM_007995199.1	-0.24752	0.003095	0.020336	sp|O75821|EIF3G_HUMAN Eukaryotic translation initiation factor 3 subunit G OS=Homo sapiens GN=EIF3G PE=1 SV=2//0
XM_007995200.1	1.2134	4.25E-05	0.000402	sp|Q96G91|P2Y11_HUMAN P2Y purinoceptor 11 OS=Homo sapiens GN=P2RY11 PE=2 SV=2//0
XM_007995219.1	1.3215	0.00016	0.001368	sp|Q5NKV6|ICAM1_MACMU Intercellular adhesion molecule 1 OS=Macaca mulatta GN=ICAM1 PE=2 SV=1//0
XM_007995220.1	-0.63624	0.000361	0.002915	sp|Q9UMF0|ICAM5_HUMAN Intercellular adhesion molecule 5 OS=Homo sapiens GN=ICAM5 PE=1 SV=3//0
XM_007995227.1	-0.51264	1.70E-10	3.18E-09	sp|Q16543|CDC37_HUMAN Hsp90 co-chaperone Cdc37 OS=Homo sapiens GN=CDC37 PE=1 SV=1//0
XM_007995228.1	1.5935	0.0048	0.030253	sp|Q5R774|KEAP1_PONAB Kelch-like ECH-associated protein 1 OS=Pongo abelii GN=KEAP1 PE=2 SV=1//0
XM_007995239.1	0.85267	1.26E-10	2.38E-09	sp|Q86TL0|ATG4D_HUMAN Cysteine protease ATG4D OS=Homo sapiens GN=ATG4D PE=1 SV=1//0
XM_007995248.1	-0.62261	0.000509	0.003999	sp|Q13445|TMED1_HUMAN Transmembrane emp24 domain-containing protein 1 OS=Homo sapiens GN=TMED1 PE=1 SV=1//1.46171e-125
XM_007995258.1	0.38589	0.006133	0.0375	sp|Q9BSF4|CS052_HUMAN Uncharacterized protein C19orf52 OS=Homo sapiens GN=C19orf52 PE=1 SV=2//1.5667e-113
XM_007995261.1	-0.41536	3.53E-06	3.94E-05	sp|Q86X55|CARM1_HUMAN Histone-arginine methyltransferase CARM1 OS=Homo sapiens GN=CARM1 PE=1 SV=3//0
XM_007995275.1	2.0322	1.04E-07	1.40E-06	sp|Q6UXH0|BETAT_HUMAN Angiopoietin-like protein 8 OS=Homo sapiens GN=C19orf80 PE=1 SV=1//1.72009e-112
XM_007995291.1	-1.4213	2.45E-41	2.55E-39	sp|O95716|RAB3D_HUMAN Ras-related protein Rab-3D OS=Homo sapiens GN=RAB3D PE=1 SV=1//9.1343e-121
XM_007995323.1	-0.40178	0.000769	0.005811	sp|Q4R5Q4|ECSIT_MACFA Evolutionarily conserved signaling intermediate in Toll pathway, mitochondrial OS=Macaca fascicularis GN=ECSIT PE=2 SV=1//0
XM_007995328.1	0.8524	2.15E-12	4.75E-11	sp|Q7YRL2|CNN1_SHEEP Calponin-1 OS=Ovis aries GN=CNN1 PE=2 SV=1//0
XM_007995340.1	0.94953	1.49E-12	3.34E-11	sp|Q7L945|ZN627_HUMAN Zinc finger protein 627 OS=Homo sapiens GN=ZNF627 PE=1 SV=1//0
XM_007995377.1	2.7904	5.37E-09	8.48E-08	sp|P15621|ZNF44_HUMAN Zinc finger protein 44 OS=Homo sapiens GN=ZNF44 PE=2 SV=3//0
XM_007995386.1	-0.45129	5.75E-05	0.000533	sp|Q9D937|CK098_MOUSE Uncharacterized protein C11orf98 homolog OS=Mus musculus PE=2 SV=1//7.13957e-48
XM_007995395.1	0.6882	1.27E-05	0.000129	sp|Q8TBZ8|ZN564_HUMAN Zinc finger protein 564 OS=Homo sapiens GN=ZNF564 PE=1 SV=1//0
XM_007995402.1	0.76548	2.35E-06	2.68E-05	sp|Q9BRX9|WDR83_HUMAN WD repeat domain-containing protein 83 OS=Homo sapiens GN=WDR83 PE=1 SV=1//0
XM_007995403.1	-0.4172	1.64E-05	0.000165	sp|Q6ZWX0|ASTER_MOUSE Protein Asterix OS=Mus musculus GN=Wdr83os PE=3 SV=1//1.14078e-69
XM_007995417.1	-0.46689	2.06E-07	2.69E-06	sp|Q9BQ61|CS043_HUMAN Uncharacterized protein C19orf43 OS=Homo sapiens GN=C19orf43 PE=1 SV=1//4.47134e-51
XM_007995418.1	-0.38969	2.93E-05	0.000283	sp|O43681|ASNA_HUMAN ATPase ASNA1 OS=Homo sapiens GN=ASNA1 PE=1 SV=2//0
XM_007995426.1	2.3139	2.36E-62	5.01E-60	sp|P17275|JUNB_HUMAN Transcription factor jun-B OS=Homo sapiens GN=JUNB PE=1 SV=1//9.63791e-166
XM_007995427.1	-0.94167	1.89E-29	1.23E-27	sp|Q2PFZ3|PRDX2_MACFA Peroxiredoxin-2 OS=Macaca fascicularis GN=PRDX2 PE=2

				SV=3//4.37183e-142
XM_007995428.1	-0.49091	2.47E-05	0.000242	sp|O75792|RNH2A_HUMAN Ribonuclease H2 subunit A OS=Homo sapiens GN=RNASEH2A PE=1 SV=2//0
XM_007995434.1	0.73935	6.62E-11	1.28E-09	sp|Q6P9B9|INT5_HUMAN Integrator complex subunit 5 OS=Homo sapiens GN=INTS5 PE=1 SV=1//0
XM_007995448.1	-0.46375	1.02E-10	1.95E-09	sp|Q4R4N7|GANAB_MACFA Neutral alpha-glucosidase AB OS=Macaca fascicularis GN=GANAB PE=2 SV=1//0
XM_007995451.1	0.79091	2.38E-15	6.69E-14	sp|Q5RFA2|SYFA_PONAB Phenylalanine--tRNA ligase alpha subunit OS=Pongo abelii GN=FARSA PE=2 SV=1//0
XM_007995452.1	-0.86086	8.83E-35	7.32E-33	sp|P27797|CALR_HUMAN Calreticulin OS=Homo sapiens GN=CALR PE=1 SV=1//0
XM_007995455.1	-0.41812	8.65E-05	0.000779	sp|Q8SPE7|G45IP_CHLAE Growth arrest and DNA damage-inducible proteins-interacting protein 1 OS=Chlorocebus aethiops GN=GADD45GIP1 PE=2 SV=1//5.13613e-88
XM_007995473.1	0.712	4.30E-09	6.87E-08	sp|Q9NXH9|TRM1_HUMAN tRNA (guanine(26)-N(2))-dimethyltransferase OS=Homo sapiens GN=TRMT1 PE=1 SV=1//0
XM_007995477.1	3.3579	2.94E-99	1.40E-96	sp|Q9BTL4|IER2_HUMAN Immediate early response gene 2 protein OS=Homo sapiens GN=IER2 PE=1 SV=1//4.44259e-93
XM_007995482.1	-0.67185	3.57E-12	7.73E-11	sp|Q5REM2|L10K_PONAB Leydig cell tumor 10 kDa protein homolog OS=Pongo abelii PE=3 SV=1//7.25187e-29
XM_007995507.1	0.24646	0.001731	0.012084	sp|O94776|MTA2_HUMAN Metastasis-associated protein MTA2 OS=Homo sapiens GN=MTA2 PE=1 SV=1//0
XM_007995509.1	-0.95981	2.75E-12	6.02E-11	sp|Q6UWB1|I27RA_HUMAN Interleukin-27 receptor subunit alpha OS=Homo sapiens GN=IL27RA PE=2 SV=2//0
XM_007995524.1	0.32041	0.000158	0.001358	sp|Q8VDW0|DX39A_MOUSE ATP-dependent RNA helicase DDX39A OS=Mus musculus GN=Ddx39a PE=2 SV=1//0
XM_007995541.1	-0.94492	4.37E-10	7.78E-09	sp|Q0MQE2|NDUB7_PONPY NADH dehydrogenase [ubiquinone] 1 beta subcomplex subunit 7 OS=Pongo pygmaeus GN=NDUFB7 PE=2 SV=3//1.47978e-69
XM_007995558.1	-0.53277	1.33E-14	3.56E-13	sp|Q4R7H5|EF1G_MACFA Elongation factor 1-gamma OS=Macaca fascicularis GN=EEF1G PE=2 SV=1//0
XM_007995571.1	0.75455	2.01E-10	3.71E-09	sp|Q9H6E5|STPAP_HUMAN Speckle targeted PIP5K1A-regulated poly(A) polymerase OS=Homo sapiens GN=TUT1 PE=1 SV=2//0
XM_007995573.1	-1.417	9.30E-44	1.04E-41	sp|Q9UM47|NOTC3_HUMAN Neurogenic locus notch homolog protein 3 OS=Homo sapiens GN=NOTCH3 PE=1 SV=2//0
XM_007995580.1	0.64849	0.00182	0.012626	sp|O60885|BRD4_HUMAN Bromodomain-containing protein 4 OS=Homo sapiens GN=BRD4 PE=1 SV=2//0
XM_007995629.1	-0.63114	0.000363	0.00293	sp|P67937|TPM4_PIG Tropomyosin alpha-4 chain OS=Sus scrofa GN=TPM4 PE=2 SV=3//1.23589e-84
XM_007995635.1	-1.2287	1.21E-36	1.07E-34	sp|Q5R9E5|FA32A_PONAB Protein FAM32A OS=Pongo abelii GN=FAM32A PE=3 SV=1//6.69613e-60
XM_007995656.1	2.0192	8.34E-53	1.38E-50	sp|O95402|MED26_HUMAN Mediator of RNA polymerase II transcription subunit 26 OS=Homo sapiens GN=MED26 PE=1 SV=2//0
XM_007995658.1	-0.42778	0.000894	0.006662	sp|Q9H6F2|TM38A_HUMAN Trimeric intracellular cation channel type A OS=Homo sapiens GN=TMEM38A PE=1 SV=1//1.89012e-167
XM_007995691.1	-4.1262	0.003106	0.020396	sp|P10588|NR2F6_HUMAN Nuclear receptor subfamily 2 group F member 6 OS=Homo sapiens GN=NR2F6 PE=1 SV=2//3.78586e-149
XM_007995717.1	0.63024	0.002651	0.017658	sp|Q969Y2|GTPB3_HUMAN tRNA modification GTPase GTPBP3, mitochondrial OS=Homo sapiens GN=GTPBP3 PE=1 SV=2//2.39518e-61
XM_007995718.1	-0.47716	2.81E-05	0.000273	sp|Q9HCE9|ANO8_HUMAN Anoctamin-8 OS=Homo sapiens GN=ANO8 PE=1 SV=3//0
XM_007995722.1	-0.37704	0.00204	0.01398	sp|Q96EY5|MB12A_HUMAN Multivesicular body subunit 12A OS=Homo sapiens GN=MVB12A PE=1 SV=1//7.01319e-177
XM_007995729.1	-0.33565	0.000179	0.001521	sp|Q8NBJ5|GT251_HUMAN Procollagen galactosyltransferase 1 OS=Homo sapiens GN=COLGALT1 PE=1 SV=1//0
XM_007995737.1	0.61866	2.01E-05	0.000199	sp|Q66K74|MAP1S_HUMAN Microtubule-associated protein 1S OS=Homo sapiens GN=MAP1S PE=1 SV=2//0
XM_007995754.1	-0.56273	6.67E-08	9.17E-07	sp|Q96CT7|CC124_HUMAN Coiled-coil domain-containing protein 124 OS=Homo sapiens GN=CCDC124 PE=1 SV=1//2.3387e-114
XM_007995778.1	-0.97664	3.49E-30	2.37E-28	sp|P13284|GILT_HUMAN Gamma-interferon-inducible lysosomal thiol reductase OS=Homo sapiens GN=IFI30 PE=1 SV=3//6.97537e-155
XM_007995793.1	3.3304	1.31E-76	3.89E-74	sp|Q99988|GDF15_HUMAN Growth/differentiation factor 15 OS=Homo sapiens GN=GDF15 PE=2 SV=3//1.60394e-162
XM_007995805.1	-0.83396	4.75E-28	2.93E-26	sp|Q4R6E3|INO1_MACFA Inositol-3-phosphate synthase 1 OS=Macaca fascicularis GN=ISYNA1 PE=2 SV=1//0
XM_007995806.1	1.5964	1.76E-48	2.42E-46	sp|P55199|ELL_HUMAN RNA polymerase II elongation factor ELL OS=Homo sapiens GN=ELL PE=1 SV=1//0
XM_007995820.1	-0.54668	0.000105	0.000932	sp|Q96EN9|CS060_HUMAN Uncharacterized protein C19orf60 OS=Homo sapiens GN=C19orf60 PE=1 SV=1//3.93138e-94
XM_007995825.1	-0.98253	0.00019	0.001609	sp|Q9UK28|TM59L_HUMAN Transmembrane protein 59-like OS=Homo sapiens GN=TMEM59L PE=2 SV=1//0
XM_007995827.1	2.3375	0.002596	0.017349	sp|Q53HC5|KLH26_HUMAN Kelch-like protein 26 OS=Homo sapiens GN=KLHL26 PE=1 SV=2//0

XM_007995832.1	0.57675	2.83E-07	3.63E-06	sp|Q9Y5Q0|FADS3_HUMAN Fatty acid desaturase 3 OS=Homo sapiens GN=FADS3 PE=2 SV=1//0
XM_007995842.1	1.3457	1.58E-15	4.51E-14	sp|Q9Y6V7|DDX49_HUMAN Probable ATP-dependent RNA helicase DDX49 OS=Homo sapiens GN=DDX49 PE=1 SV=1//0
XM_007995843.1	-0.66206	1.73E-13	4.25E-12	sp|O14579|COPE_HUMAN Coatomer subunit epsilon OS=Homo sapiens GN=COPE PE=1 SV=3//0
XM_007995856.1	-2.0123	0.000266	0.002192	sp|Q8IX01|SUGP2_HUMAN SURP and G-patch domain-containing protein 2 OS=Homo sapiens GN=SUGP2 PE=1 SV=2//0
XM_007995860.1	-2.2634	9.58E-06	9.92E-05	sp|Q8IX01|SUGP2_HUMAN SURP and G-patch domain-containing protein 2 OS=Homo sapiens GN=SUGP2 PE=1 SV=2//0
XM_007995889.1	0.94334	7.16E-13	1.65E-11	sp|Q4R5U5|FEN1_MACFA Flap endonuclease 1 OS=Macaca fascicularis GN=FEN1 PE=2 SV=1//0
XM_007995925.1	-0.83905	2.84E-16	8.53E-15	sp|Q4R6H1|NDUAD_MACFA NADH dehydrogenase [ubiquinone] 1 alpha subcomplex subunit 13 OS=Macaca fascicularis GN=NDUFA13 PE=2 SV=1//3.52007e-113
XM_007995926.1	1.5603	6.31E-11	1.23E-09	sp|Q9BXA6|TSSK6_HUMAN Testis-specific serine/threonine-protein kinase 6 OS=Homo sapiens GN=TSSK6 PE=1 SV=1//5.16978e-08
XM_007995939.1	-1.6902	4.04E-09	6.49E-08	sp|O75346|ZN253_HUMAN Zinc finger protein 253 OS=Homo sapiens GN=ZNF253 PE=2 SV=2//8.19461e-06
XM_007995994.1	-0.57977	0.000585	0.004535	sp|Q9H8G1|ZN430_HUMAN Zinc finger protein 430 OS=Homo sapiens GN=ZNF430 PE=1 SV=3//2.95844e-102
XM_007996014.1	-1.0018	3.59E-05	0.000342	sp|Q8TD23|ZN675_HUMAN Zinc finger protein 675 OS=Homo sapiens GN=ZNF675 PE=1 SV=3//2.43643e-140
XM_007996031.1	-1.0651	4.87E-20	1.91E-18	sp|Q9UL51|HCN2_HUMAN Potassium/sodium hyperpolarization-activated cyclic nucleotide-gated channel 2 OS=Homo sapiens GN=HCN2 PE=1 SV=3//0
XM_007996032.1	0.41418	3.32E-06	3.72E-05	sp|O00411|RPOM_HUMAN DNA-directed RNA polymerase, mitochondrial OS=Homo sapiens GN=POLRMT PE=1 SV=2//2.30719e-104
XM_007996036.1	-1.1437	8.40E-11	1.61E-09	sp|O95996|APC2_HUMAN Adenomatous polyposis coli protein 2 OS=Homo sapiens GN=APC2 PE=1 SV=1//0
XM_007996037.1	-0.97219	1.17E-25	6.34E-24	sp|Q86XN8|MEX3D_HUMAN RNA-binding protein MEX3D OS=Homo sapiens GN=MEX3D PE=1 SV=3//0
XM_007996039.1	-1.0396	0.000596	0.004609	sp|Q9BX70|BTBD2_HUMAN BTB/POZ domain-containing protein 2 OS=Homo sapiens GN=BTBD2 PE=1 SV=1//6.10236e-50
XM_007996043.1	-0.86797	3.52E-07	4.46E-06	sp|Q04725|TLE2_HUMAN Transducin-like enhancer protein 2 OS=Homo sapiens GN=TLE2 PE=1 SV=2//5.01876e-57
XM_007996044.1	-0.50246	0.006476	0.039432	sp|O95049|ZO3_HUMAN Tight junction protein ZO-3 OS=Homo sapiens GN=TJP3 PE=1 SV=3//0
XM_007996045.1	0.84658	1.17E-06	1.40E-05	sp|Q8N2W9|PIAS4_HUMAN E3 SUMO-protein ligase PIAS4 OS=Homo sapiens GN=PIAS4 PE=1 SV=1//0
XM_007996050.1	-0.40642	1.10E-05	0.000113	sp|Q9H3T3|SEM6B_HUMAN Semaphorin-6B OS=Homo sapiens GN=SEMA6B PE=1 SV=4//1.28988e-95
XM_007996051.1	-0.38345	0.002126	0.014519	sp|Q969H8|MYDGF_HUMAN Myeloid-derived growth factor OS=Homo sapiens GN=MYDGF PE=1 SV=1//4.0039e-70
XM_007996052.1	-0.79217	1.47E-18	5.22E-17	sp|O60664|PLIN3_HUMAN Perilipin-3 OS=Homo sapiens GN=PLIN3 PE=1 SV=3//3.13751e-67
XM_007996054.1	1.0896	1.37E-20	5.58E-19	sp|Q96T88|UHRF1_HUMAN E3 ubiquitin-protein ligase UHRF1 OS=Homo sapiens GN=UHRF1 PE=1 SV=1//0
XM_007996059.1	-0.97529	6.02E-05	0.000556	sp|P01024|CO3_HUMAN Complement C3 OS=Homo sapiens GN=C3 PE=1 SV=2//0
XM_007996064.1	0.50296	1.47E-07	1.95E-06	sp|P06213|INSR_HUMAN Insulin receptor OS=Homo sapiens GN=INSR PE=1 SV=4//0
XM_007996066.1	-0.69218	0.000823	0.006181	sp|Q9P0N8|MARH2_HUMAN E3 ubiquitin-protein ligase MARCH2 OS=Homo sapiens GN=MARCH2 PE=1 SV=1//2.21548e-78
XM_007996067.1	-0.26842	0.000448	0.003556	sp|Q62826|HNRPM_RAT Heterogeneous nuclear ribonucleoprotein M OS=Rattus norvegicus GN=Hnrnpm PE=1 SV=4//0
XM_007996068.1	-1.3676	0.001655	0.011621	sp|Q96QH2|PRAM_HUMAN PML-RARA-regulated adapter molecule 1 OS=Homo sapiens GN=PRAM1 PE=1 SV=2//0
XM_007996079.1	-0.69713	0.000232	0.001938	sp|P27815|PDE4A_HUMAN cAMP-specific 3',5'-cyclic phosphodiesterase 4A OS=Homo sapiens GN=PDE4A PE=1 SV=3//4.15697e-31
XM_007996080.1	0.46407	0.006378	0.038933	sp|Q9BXR0|TGT_HUMAN Queuine tRNA-ribosyltransferase OS=Homo sapiens GN=QTRT1 PE=1 SV=3//0
XM_007996082.1	1.1867	8.43E-55	1.45E-52	sp|P01130|LDLR_HUMAN Low-density lipoprotein receptor OS=Homo sapiens GN=LDLR PE=1 SV=1//0
XM_007996085.1	0.80416	7.84E-06	8.24E-05	sp|Q8N8Z8|ZN441_HUMAN Zinc finger protein 441 OS=Homo sapiens GN=ZNF441 PE=2 SV=2//8.32022e-09
XM_007996086.1	1.1709	4.39E-10	7.82E-09	sp|Q5REI6|ZN791_PONAB Zinc finger protein 791 OS=Pongo abelii GN=ZNF791 PE=2 SV=1//3.71291e-97
XM_007996088.1	0.97347	0.000749	0.005679	sp|O00555|CAC1A_HUMAN Voltage-dependent P/Q-type calcium channel subunit alpha-1A OS=Homo sapiens GN=CACNA1A PE=1 SV=2//3.35814e-06
XM_007996089.1	0.94641	2.19E-10	4.01E-09	sp|P13994|CC130_HUMAN Coiled-coil domain-containing protein 130 OS=Homo sapiens GN=CCDC130 PE=1 SV=2//0
XM_007996090.1	1.1118	5.78E-30	3.87E-28	sp|Q8C7B8|ZSWM4_MOUSE Zinc finger SWIM domain-containing protein 4 OS=Mus musculus GN=Zswim4 PE=2 SV=2//0

XM_007996092.1	-0.55236	6.91E-07	8.51E-06	sp|Q6SPF0|SAMD1_HUMAN Atherin OS=Homo sapiens GN=SAMD1 PE=1 SV=1//1.84966e-104
XM_007996093.1	-1.138	3.24E-13	7.74E-12	sp|P17612|KAPCA_HUMAN cAMP-dependent protein kinase catalytic subunit alpha OS=Homo sapiens GN=PRKACA PE=1 SV=2//9.33238e-126
XM_007996095.1	-0.50396	9.44E-06	9.79E-05	sp|O94910|LPHN1_HUMAN Latrophilin-1 OS=Homo sapiens GN=LPHN1 PE=1 SV=1//0
XM_007996101.1	-1.1846	6.01E-22	2.65E-20	sp|A6NIK2|LR10B_HUMAN Leucine-rich repeat-containing protein 10B OS=Homo sapiens GN=LRRC10B PE=4 SV=2//1.98445e-141
XM_007996102.1	1.5747	4.10E-22	1.83E-20	sp|Q9H6X5|CS044_HUMAN Uncharacterized protein C19orf44 OS=Homo sapiens GN=C19orf44 PE=1 SV=1//0
XM_007996104.1	-0.82852	1.79E-10	3.33E-09	sp|Q6PCB7|S27A1_HUMAN Long-chain fatty acid transport protein 1 OS=Homo sapiens GN=SLC27A1 PE=2 SV=1//0
XM_007996106.1	-0.48902	2.05E-07	2.68E-06	sp|O00459|P85B_HUMAN Phosphatidylinositol 3-kinase regulatory subunit beta OS=Homo sapiens GN=PIK3R2 PE=1 SV=2//2.36818e-10
XM_007996107.1	-0.69937	0.006911	0.041667	sp|Q9NXJ5|PGPI_HUMAN Pyroglutamyl-peptidase 1 OS=Homo sapiens GN=PGPEP1 PE=1 SV=1//3.46153e-128
XM_007996118.1	-0.46936	8.26E-09	1.27E-07	sp|Q69BK2|UCRI_THEGE Cytochrome b-c1 complex subunit Rieske, mitochondrial OS=Theropithecus gelada GN=UQCRFS1 PE=3 SV=1//0
XM_007996233.1	1.2257	3.78E-50	5.55E-48	sp|Q8IUC4|RHPN2_HUMAN Rhophilin-2 OS=Homo sapiens GN=RHPN2 PE=1 SV=1//0
XM_007996240.1	0.52786	1.08E-07	1.46E-06	sp|P49715|CEBPA_HUMAN CCAAT/enhancer-binding protein alpha OS=Homo sapiens GN=CEBPA PE=1 SV=3//7.03509e-108
XM_007996242.1	-0.64697	3.27E-13	7.81E-12	sp|P12955|PEPD_HUMAN Xaa-Pro dipeptidase OS=Homo sapiens GN=PEPD PE=1 SV=3//0
XM_007996256.1	-0.96023	1.19E-33	9.39E-32	sp|Q4R591|G6PI_MACFA Glucose-6-phosphate isomerase OS=Macaca fascicularis GN=GPI PE=2 SV=3//0
XM_007996257.1	1.1564	7.61E-10	1.32E-08	sp|Q9BRP1|PDD2L_HUMAN Programmed cell death protein 2-like OS=Homo sapiens GN=PDCD2L PE=1 SV=1//0
XM_007996276.1	0.64918	2.71E-07	3.48E-06	sp|A6NIX2|WTIP_HUMAN Wilms tumor protein 1-interacting protein OS=Homo sapiens GN=WTIP PE=1 SV=3//4.74755e-138
XM_007996296.1	-1.0498	4.20E-14	1.07E-12	sp|Q4PPC4|SCN1B_CANFA Sodium channel subunit beta-1 OS=Canis familiaris GN=SCN1B PE=2 SV=1//1.89674e-116
XM_007996398.1	-0.99667	0.004656	0.029436	sp|P62876|RPAB5_MOUSE DNA-directed RNA polymerases I, II, and III subunit RPABC5 OS=Mus musculus GN=Polr2l PE=3 SV=1//4.39846e-41
XM_007996436.1	-0.96574	7.24E-17	2.29E-15	sp|P51693|APLP1_HUMAN Amyloid-like protein 1 OS=Homo sapiens GN=APLP1 PE=1 SV=3//0
XM_007996447.1	-0.86637	0.000212	0.001776	sp|Q9BTN0|LRFN3_HUMAN Leucine-rich repeat and fibronectin type-III domain-containing protein 3 OS=Homo sapiens GN=LRFN3 PE=2 SV=1//0
XM_007996449.1	1.2643	5.24E-12	1.12E-10	sp|A6NFY7|SDHF1_HUMAN Succinate dehydrogenase assembly factor 1, mitochondrial OS=Homo sapiens GN=SDHAF1 PE=1 SV=1//1.78817e-55
XM_007996534.1	-0.96619	1.86E-26	1.06E-24	sp|Q24JP5|T132A_HUMAN Transmembrane protein 132A OS=Homo sapiens GN=TMEM132A PE=2 SV=1//0
XM_007996586.1	0.56143	0.001758	0.012258	sp|Q4R6C2|ZN383_MACFA Zinc finger protein 383 OS=Macaca fascicularis GN=ZNF383 PE=2 SV=1//0
XM_007996587.1	1.4298	7.53E-06	7.94E-05	sp|P10072|HKR1_HUMAN Krueppel-related zinc finger protein 1 OS=Homo sapiens GN=HKR1 PE=2 SV=4//1.36977e-68
XM_007996608.1	1.4881	2.61E-16	7.87E-15	sp|Q9H6F5|CCD86_HUMAN Coiled-coil domain-containing protein 86 OS=Homo sapiens GN=CCDC86 PE=1 SV=1//1.48649e-176
XM_007996682.1	-1.149	0.000666	0.005108	sp|Q86UU5|GGN_HUMAN Gametogenetin OS=Homo sapiens GN=GGN PE=1 SV=2//1.37141e-59
XM_007996719.1	-0.96768	3.67E-25	1.96E-23	sp|Q5RFG0|ECH1_PONAB Delta(3,5)-Delta(2,4)-dienoyl-CoA isomerase, mitochondrial OS=Pongo abelii GN=ECH1 PE=2 SV=1//0
XM_007996724.1	0.9021	1.66E-08	2.46E-07	sp|Q15653|IKBB_HUMAN NF-kappa-B inhibitor beta OS=Homo sapiens GN=NFKBIB PE=1 SV=2//1.15831e-173
XM_007996733.1	-0.35633	0.002667	0.017735	sp|O15235|RT12_HUMAN 28S ribosomal protein S12, mitochondrial OS=Homo sapiens GN=MRPS12 PE=1 SV=1//2.43367e-75
XM_007996782.1	-0.43221	8.68E-10	1.49E-08	sp|P62250|RS16_RAT 40S ribosomal protein S16 OS=Rattus norvegicus GN=Rps16 PE=1 SV=2//3.3156e-101
XM_007996788.1	-0.79841	0.000275	0.002263	sp|P59797|SELV_HUMAN Selenoprotein V OS=Homo sapiens GN=SELV PE=1 SV=2//2.22717e-116
XM_007996794.1	2.0695	7.97E-08	1.08E-06	sp|Q9UHV8|PP13_HUMAN Galactoside-binding soluble lectin 13 OS=Homo sapiens GN=LGALS13 PE=1 SV=1//1.37442e-82
XM_007996803.1	-0.31302	0.000145	0.001247	sp|P35550|FBRL_MOUSE rRNA 2'-O-methyltransferase fibrillarin OS=Mus musculus GN=Fbl PE=2 SV=2//2.67982e-177
XM_007996839.1	0.81955	5.77E-07	7.17E-06	sp|Q9UHV2|SRTD1_HUMAN SERTA domain-containing protein 1 OS=Homo sapiens GN=SERTAD1 PE=1 SV=2//8.65678e-75
XM_007996844.1	-0.40244	5.52E-05	0.000512	sp|Q8TBC3|SHKB1_HUMAN SH3KBP1-binding protein 1 OS=Homo sapiens GN=SHKBP1 PE=1 SV=2//0
XM_007996853.1	1.6334	0.004178	0.02667	sp|Q96D53|ADCK4_HUMAN AarF domain-containing protein kinase 4 OS=Homo sapiens GN=ADCK4 PE=1 SV=2//0
XM_007996874.1	-0.88007	3.21E-08	4.59E-07	sp|P61018|RAB4B_HUMAN Ras-related protein Rab-4B OS=Homo sapiens GN=RAB4B PE=1 SV=1//2.36468e-154

XM_007996893.1	0.28795	0.001623	0.01143	sp|Q96F63|CCD97_HUMAN Coiled-coil domain-containing protein 97 OS=Homo sapiens GN=CCDC97 PE=1 SV=1//1.58774e-146
XM_007996894.1	-0.88788	1.53E-27	9.20E-26	sp|P01137|TGFB1_HUMAN Transforming growth factor beta-1 OS=Homo sapiens GN=TGFB1 PE=1 SV=2//0
XM_007996903.1	-0.60385	9.75E-08	1.32E-06	sp|Q8HXY4|ODBA_MACFA 2-oxoisovalerate dehydrogenase subunit alpha, mitochondrial OS=Macaca fascicularis GN=BCKDHA PE=2 SV=1//0
XM_007996906.1	-2.2249	0.005738	0.035403	sp|A6NGS2|ERIC4_HUMAN Glutamate-rich protein 4 OS=Homo sapiens GN=ERICH4 PE=4 SV=2//3.2739e-34
XM_007996943.1	-0.47679	3.29E-11	6.58E-10	sp|Q5R8M9|RS19_PONAB 40S ribosomal protein S19 OS=Pongo abelii GN=RPS19 PE=2 SV=3//4.01126e-102
XM_007996946.1	-0.9969	4.23E-12	9.08E-11	sp|Q9UI14|PRAF1_HUMAN Prenylated Rab acceptor protein 1 OS=Homo sapiens GN=RABAC1 PE=1 SV=1//2.66267e-129
XM_007996971.1	0.68492	2.28E-07	2.96E-06	sp|Q8WXF8|DEDD2_HUMAN DNA-binding death effector domain-containing protein 2 OS=Homo sapiens GN=DEDD2 PE=1 SV=1//3.77033e-133
XM_007996995.1	-0.51398	0.003587	0.023209	sp|Q7Z7M0|MEGF8_HUMAN Multiple epidermal growth factor-like domains protein 8 OS=Homo sapiens GN=MEGF8 PE=1 SV=2//0
XM_007997043.1	-0.95208	5.50E-12	1.17E-10	sp|Q8NFZ8|CADM4_HUMAN Cell adhesion molecule 4 OS=Homo sapiens GN=CADM4 PE=1 SV=1//0
XM_007997049.1	0.6524	0.001189	0.008631	sp|Q9GK79|UPAR_CHLAE Urokinase plasminogen activator surface receptor OS=Chlorocebus aethiops GN=PLAUR PE=2 SV=1//9.98018e-164
XM_007997052.1	1.8092	3.79E-06	4.21E-05	sp|Q9H0W8|SMG9_HUMAN Protein SMG9 OS=Homo sapiens GN=SMG9 PE=1 SV=1//0
XM_007997076.1	-0.93094	0.006404	0.039057	sp|Q9UK13|ZN221_HUMAN Zinc finger protein 221 OS=Homo sapiens GN=ZNF221 PE=2 SV=3//1.82261e-24
XM_007997111.1	1.642	7.19E-28	4.37E-26	sp|P32506|PVR_CHLAE Poliovirus receptor homolog OS=Chlorocebus aethiops GN=PVR PE=2 SV=1//0
XM_007997137.1	1.7664	5.57E-08	7.72E-07	sp|Q7Z692|CEA19_HUMAN Carcinoembryonic antigen-related cell adhesion molecule 19 OS=Homo sapiens GN=CEACAM19 PE=1 SV=2//7.78056e-179
XM_007997141.1	0.58671	0.000339	0.00275	sp|P20749|BCL3_HUMAN B-cell lymphoma 3 protein OS=Homo sapiens GN=BCL3 PE=1 SV=2//0
XM_007997149.1	-2.7739	1.06E-05	0.000109	sp|P50895|BCAM_HUMAN Basal cell adhesion molecule OS=Homo sapiens GN=BCAM PE=1 SV=2//0
XM_007997151.1	-0.56468	0.000193	0.001626	sp|Q92692|PVRL2_HUMAN Nectin-2 OS=Homo sapiens GN=PVRL2 PE=1 SV=1//0
XM_007997153.1	-0.54241	6.04E-06	6.49E-05	sp|Q5R7L3|RM16_PONAB 39S ribosomal protein L16, mitochondrial OS=Pongo abelii GN=MRPL16 PE=2 SV=1//6.75426e-156
XM_007997175.1	0.85179	1.44E-05	0.000145	sp|Q5JPI9|MET10_HUMAN Protein-lysine N-methyltransferase METTL10 OS=Homo sapiens GN=METTL10 PE=1 SV=2//6.24669e-07
XM_007997177.1	0.92707	1.08E-06	1.30E-05	sp|A5A769|BL1S3_PIG Biogenesis of lysosome-related organelles complex 1 subunit 3 OS=Sus scrofa GN=BLOC1S3 PE=2 SV=1//1.09953e-53
XM_007997204.1	Inf	0.006804	0.041132	sp|P53539|FOSB_HUMAN Protein fosB OS=Homo sapiens GN=FOSB PE=2 SV=1//5.88069e-135
XM_007997242.1	-1.3722	1.43E-38	1.35E-36	sp|P62317|SMD2_MOUSE Small nuclear ribonucleoprotein Sm D2 OS=Mus musculus GN=Snrpd2 PE=3 SV=1//1.78456e-57
XM_007997250.1	0.59718	9.02E-11	1.73E-09	sp|Q5R8Q4|PATL1_PONAB Protein PAT1 homolog 1 OS=Pongo abelii GN=PATL1 PE=2 SV=1//0
XM_007997254.1	-0.59322	8.93E-07	1.09E-05	sp|Q8N196|SIX5_HUMAN Homeobox protein SIX5 OS=Homo sapiens GN=SIX5 PE=1 SV=3//0
XM_007997264.1	0.49498	0.000168	0.001436	sp|Q8IU81|I2BP1_HUMAN Interferon regulatory factor 2-binding protein 1 OS=Homo sapiens GN=IRF2BP1 PE=1 SV=1//0
XM_007997266.1	#NAME?	0.005641	0.034883	sp|Q86VE0|MYPOP_HUMAN Myb-related transcription factor, partner of profilin OS=Homo sapiens GN=MYPOP PE=2 SV=2//3.56237e-100
XM_007997270.1	-1.7215	1.98E-11	4.02E-10	sp|Q9UNW9|NOVA2_HUMAN RNA-binding protein Nova-2 OS=Homo sapiens GN=NOVA2 PE=1 SV=1//2.63201e-06
XM_007997279.1	4.8278	9.80E-14	2.44E-12	sp|Q6UW32|IGFL1_HUMAN Insulin growth factor-like family member 1 OS=Homo sapiens GN=IGFL1 PE=1 SV=1//3.56059e-62
XM_007997299.1	-1.5029	1.25E-63	2.67E-61	sp|P62155|CALM_XENLA Calmodulin OS=Xenopus laevis GN=calm1 PE=1 SV=2//3.80319e-95
XM_007997317.1	-0.97113	0.003418	0.02227	sp|Q86SG4|DPCA2_HUMAN Putative Dresden prostate carcinoma protein 2 OS=Homo sapiens GN=HMGN2P46 PE=5 SV=1//2.73578e-26
XM_007997318.1	-0.68632	1.36E-05	0.000137	sp|P62744|AP2S1_RAT AP-2 complex subunit sigma OS=Rattus norvegicus GN=Ap2s1 PE=1 SV=1//1.33385e-28
XM_007997319.1	0.25762	0.00546	0.033898	sp|Q9NRY4|RHG35_HUMAN Rho GTPase-activating protein 35 OS=Homo sapiens GN=ARHGAP35 PE=1 SV=3//0
XM_007997327.1	-0.99256	2.92E-06	3.30E-05	sp|Q9NX00|TM160_HUMAN Transmembrane protein 160 OS=Homo sapiens GN=TMEM160 PE=1 SV=1//4.66279e-93
XM_007997338.1	-0.76612	2.48E-20	9.92E-19	sp|P62856|RS26_RAT 40S ribosomal protein S26 OS=Rattus norvegicus GN=Rps26 PE=3 SV=3//4.45255e-50
XM_007997347.1	-0.48427	0.007318	0.043736	sp|C9JVW0|INAM1_HUMAN Putative transmembrane protein INAFM1 OS=Homo sapiens GN=INAFM1 PE=4 SV=1//2.30888e-15
XM_007997369.1	-0.87212	2.40E-19	8.95E-18	sp|Q9NZN4|EHD2_HUMAN EH domain-containing protein 2 OS=Homo sapiens GN=EHD2 PE=1 SV=2//0

XM_007997371.1	-0.31715	8.95E-05	0.000802	sp|Q9NZM5|GSCR2_HUMAN Glioma tumor suppressor candidate region gene 2 protein OS=Homo sapiens GN=GLTSCR2 PE=1 SV=2//0
XM_007997413.1	-0.80436	0.000172	0.001466	sp|P54852|EMP3_HUMAN Epithelial membrane protein 3 OS=Homo sapiens GN=EMP3 PE=1 SV=1//1.19238e-82
XM_007997422.1	-0.79836	2.43E-21	1.03E-19	sp|P24390|ERD21_HUMAN ER lumen protein-retaining receptor 1 OS=Homo sapiens GN=KDELR1 PE=1 SV=1//5.05255e-129
XM_007997428.1	0.82053	3.16E-09	5.12E-08	sp|Q9BQ67|GRWD1_HUMAN Glutamate-rich WD repeat-containing protein 1 OS=Homo sapiens GN=GRWD1 PE=1 SV=1//0
XM_007997432.1	-1.2717	2.19E-12	4.82E-11	sp|Q96Q04|LMTK3_HUMAN Serine/threonine-protein kinase LMTK3 OS=Homo sapiens GN=LMTK3 PE=1 SV=2//1.56284e-40
XM_007997451.1	-0.94361	2.95E-11	5.93E-10	sp|Q866X7|CAH11_BOVIN Carbonic anhydrase-related protein 11 OS=Bos taurus GN=CA11 PE=2 SV=1//0
XM_007997465.1	4.1885	9.31E-32	6.68E-30	sp|Q9NSA1|FGF21_HUMAN Fibroblast growth factor 21 OS=Homo sapiens GN=FGF21 PE=1 SV=1//7.38743e-120
XM_007997466.1	1.0965	6.09E-11	1.19E-09	sp|Q9BPX1|DHB14_HUMAN 17-beta-hydroxysteroid dehydrogenase 14 OS=Homo sapiens GN=HSD17B14 PE=1 SV=1//1.1783e-132
XM_007997475.1	-0.88019	2.51E-05	0.000245	sp|Q7JK39|DHDH_MACFU Trans-1,2-dihydrobenzene-1,2-diol dehydrogenase OS=Macaca fuscata fuscata GN=DHDH PE=1 SV=1//0
XM_007997480.1	-0.93113	1.43E-38	1.35E-36	sp|Q5R538|FRIL_PONAB Ferritin light chain OS=Pongo abelii GN=FTL PE=2 SV=3//9.32697e-114
XM_007997568.1	-0.59415	0.005468	0.033939	sp|Q96D15|RCN3_HUMAN Reticulocalbin-3 OS=Homo sapiens GN=RCN3 PE=1 SV=1//1.26264e-153
XM_007997573.1	-0.40605	0.00187	0.012911	sp|Q9Y314|NOSIP_HUMAN Nitric oxide synthase-interacting protein OS=Homo sapiens GN=NOSIP PE=1 SV=1//0
XM_007997574.1	-0.94006	8.80E-20	3.41E-18	sp|Q9ULL5|PRR12_HUMAN Proline-rich protein 12 OS=Homo sapiens GN=PRR12 PE=1 SV=2//0
XM_007997575.1	-0.84618	6.59E-10	1.14E-08	sp|P10301|RRAS_HUMAN Ras-related protein R-Ras OS=Homo sapiens GN=RRAS PE=1 SV=1//5.47268e-129
XM_007997603.1	-0.33156	8.27E-05	0.000748	sp|Q71SY5|MED25_HUMAN Mediator of RNA polymerase II transcription subunit 25 OS=Homo sapiens GN=MED25 PE=1 SV=2//0
XM_007997604.1	-0.46811	0.000127	0.001103	sp|Q96T60|PNKP_HUMAN Bifunctional polynucleotide phosphatase/kinase OS=Homo sapiens GN=PNKP PE=1 SV=1//0
XM_007997605.1	-1.004	6.58E-33	4.96E-31	sp|Q86YD1|PTOV1_HUMAN Prostate tumor-overexpressed gene 1 protein OS=Homo sapiens GN=PTOV1 PE=1 SV=1//4.31739e-56
XM_007997621.1	0.92957	1.41E-14	3.76E-13	sp|P37198|NUP62_HUMAN Nuclear pore glycoprotein p62 OS=Homo sapiens GN=NUP62 PE=1 SV=3//8.83576e-165
XM_007997640.1	-1.0073	0.000847	0.006342	sp|Q63959|KCNC3_MOUSE Potassium voltage-gated channel subfamily C member 3 OS=Mus musculus GN=Kcnc3 PE=1 SV=2//0
XM_007997642.1	-1.0303	3.18E-11	6.37E-10	sp|O96009|NAPSA_HUMAN Napsin-A OS=Homo sapiens GN=NAPSA PE=1 SV=1//0
XM_007997668.1	Inf	0.001121	0.008193	sp|Q969I3|GLYL1_HUMAN Glycine N-acyltransferase-like protein 1 OS=Homo sapiens GN=GLYATL1 PE=1 SV=1//8.10086e-19
XM_007997674.1	-1.2352	0.008096	0.047819	sp|Q9NT99|LRC4B_HUMAN Leucine-rich repeat-containing protein 4B OS=Homo sapiens GN=LRRC4B PE=2 SV=3//0
XM_007997690.1	1.0942	3.67E-06	4.08E-05	sp|P26441|CNTF_HUMAN Ciliary neurotrophic factor OS=Homo sapiens GN=CNTF PE=1 SV=1//1.57013e-123
XM_007997710.1	2.1143	0.000813	0.006112	sp|Q9Y5K2|KLK4_HUMAN Kallikrein-4 OS=Homo sapiens GN=KLK4 PE=1 SV=2//7.87634e-152
XM_007997770.1	-0.78664	2.85E-15	7.94E-14	sp|Q5RFK0|ETFB_PONAB Electron transfer flavoprotein subunit beta OS=Pongo abelii GN=ETFB PE=2 SV=3//9.77293e-156
XM_007997808.1	-1.9388	3.60E-08	5.12E-07	-//-
XM_007997857.1	-0.59135	1.90E-15	5.40E-14	sp|P30153|2AAA_HUMAN Serine/threonine-protein phosphatase 2A 65 kDa regulatory subunit A alpha isoform OS=Homo sapiens GN=PPP2R1A PE=1 SV=4//0
XM_007997911.1	1.1726	1.47E-08	2.19E-07	sp|Q96IR2|ZN845_HUMAN Zinc finger protein 845 OS=Homo sapiens GN=ZNF845 PE=2 SV=3//5.22521e-13
XM_007997912.1	1.1932	5.09E-06	5.54E-05	sp|Q96IR2|ZN845_HUMAN Zinc finger protein 845 OS=Homo sapiens GN=ZNF845 PE=2 SV=3//1.26859e-10
XM_007997914.1	-1.1176	0.006712	0.040643	sp|Q0VGE8|ZN816_HUMAN Zinc finger protein 816 OS=Homo sapiens GN=ZNF816 PE=2 SV=2//5.18372e-31
XM_007997969.1	-1.5726	1.22E-15	3.51E-14	sp|P62957|CCG7_RAT Voltage-dependent calcium channel gamma-7 subunit OS=Rattus norvegicus GN=Cacng7 PE=1 SV=1//2.55949e-52
XM_007997975.1	-1.2815	1.26E-07	1.68E-06	sp|Q0MQ96|NDUA3_PANTR NADH dehydrogenase [ubiquinone] 1 alpha subcomplex subunit 3 OS=Pan troglodytes GN=NDUFA3 PE=3 SV=1//2.44047e-49
XM_007997976.1	-0.74077	1.18E-14	3.18E-13	sp|Q8WWY3|PRP31_HUMAN U4/U6 small nuclear ribonucleoprotein Prp31 OS=Homo sapiens GN=PRPF31 PE=1 SV=2//0
XM_007997979.1	-1.4687	0.000288	0.002363	sp|Q7Z404|TMC4_HUMAN Transmembrane channel-like protein 4 OS=Homo sapiens GN=TMC4 PE=2 SV=3//1.74566e-160
XM_007998028.1	-0.58003	2.22E-14	5.78E-13	sp|P29314|RS9_RAT 40S ribosomal protein S9 OS=Rattus norvegicus GN=Rps9 PE=1 SV=4//3.34857e-126
XM_007998029.1	-0.58652	1.43E-11	2.95E-10	sp|Q96N66|MBOA7_HUMAN Lysophospholipid acyltransferase 7 OS=Homo sapiens GN=MBOAT7 PE=1 SV=2//0

XM_007998110.1	1.0364	4.47E-17	1.45E-15	sp|Q8NBN7|RDH13_HUMAN Retinol dehydrogenase 13 OS=Homo sapiens GN=RDH13 PE=1 SV=2//0
XM_007998114.1	0.65796	3.02E-10	5.46E-09	sp|Q9BZL4|PP12C_HUMAN Protein phosphatase 1 regulatory subunit 12C OS=Homo sapiens GN=PPP1R12C PE=1 SV=1//0
XM_007998120.1	-1.636	1.66E-06	1.94E-05	sp|Q8MKD5|TNNI3_CANFA Troponin I, cardiac muscle OS=Canis familiaris GN=TNNI3 PE=2 SV=3//2.59318e-106
XM_007998129.1	-2.1887	0.005849	0.036016	sp|Q8N661|TM86B_HUMAN Lysoplasmalogenase OS=Homo sapiens GN=TMEM86B PE=1 SV=2//6.98656e-128
XM_007998135.1	0.72301	5.44E-06	5.90E-05	sp|Q8TDC3|BRSK1_HUMAN Serine/threonine-protein kinase BRSK1 OS=Homo sapiens GN=BRSK1 PE=1 SV=2//1.79785e-27
XM_007998147.1	-2.1989	0.000868	0.006484	sp|Q86Y97|SV422_HUMAN Histone-lysine N-methyltransferase SUV420H2 OS=Homo sapiens GN=SUV420H2 PE=1 SV=1//8.70191e-132
XM_007998155.1	-1.2257	0.000138	0.001192	sp|Q6YFQ2|CX6B2_HUMAN Cytochrome c oxidase subunit 6B2 OS=Homo sapiens GN=COX6B2 PE=1 SV=1//7.17835e-55
XM_007998157.1	-0.33876	1.55E-06	1.82E-05	sp|P41105|RL28_MOUSE 60S ribosomal protein L28 OS=Mus musculus GN=Rpl28 PE=1 SV=2//6.62814e-81
XM_007998169.1	0.35595	0.000189	0.001602	sp|Q4R5B4|TMX2_MACFA Thioredoxin-related transmembrane protein 2 OS=Macaca fascicularis GN=TMX2 PE=2 SV=1//0
XM_007998189.1	-0.70248	5.57E-09	8.76E-08	sp|Q9UK33|ZN580_HUMAN Zinc finger protein 580 OS=Homo sapiens GN=ZNF580 PE=1 SV=1//2.42809e-76
XM_007998207.1	0.43649	3.40E-07	4.31E-06	sp|Q2THX1|ZDHC5_PANTR Palmitoyltransferase ZDHHC5 OS=Pan troglodytes GN=ZDHHC5 PE=2 SV=1//0
XM_007998235.1	1.0017	2.51E-10	4.58E-09	sp|Q4R7R3|CLP1_MACFA Polyribonucleotide 5'-hydroxyl-kinase Clp1 OS=Macaca fascicularis GN=CLP1 PE=2 SV=1//0
XM_007998282.1	1.3238	5.29E-13	1.24E-11	sp|Q5CZA5|ZN805_HUMAN Zinc finger protein 805 OS=Homo sapiens GN=ZNF805 PE=2 SV=3//0
XM_007998323.1	0.67681	2.20E-05	0.000216	sp|Q5RBQ3|ZN549_PONAB Zinc finger protein 549 OS=Pongo abelii GN=ZNF549 PE=2 SV=1//0
XM_007998331.1	1.0706	1.05E-15	3.05E-14	sp|P52741|ZN134_HUMAN Zinc finger protein 134 OS=Homo sapiens GN=ZNF134 PE=2 SV=2//1.6224e-66
XM_007998332.1	1.6051	2.34E-10	4.29E-09	sp|Q6P9A1|ZN530_HUMAN Zinc finger protein 530 OS=Homo sapiens GN=ZNF530 PE=2 SV=2//0
XM_007998341.1	0.54388	5.24E-09	8.29E-08	sp|Q9H0E2|TOLIP_HUMAN Toll-interacting protein OS=Homo sapiens GN=TOLLIP PE=1 SV=1//1.19586e-153
XM_007998343.1	1.5677	4.25E-17	1.37E-15	sp|Q9BWM5|ZN416_HUMAN Zinc finger protein 416 OS=Homo sapiens GN=ZNF416 PE=2 SV=1//1.72771e-32
XM_007998369.1	0.56521	0.000544	0.004245	sp|Q96SQ5|ZN587_HUMAN Zinc finger protein 587 OS=Homo sapiens GN=ZNF587 PE=1 SV=1//7.84817e-07
XM_007998439.1	1.2519	2.60E-08	3.76E-07	sp|Q9NWS9|ZN446_HUMAN Zinc finger protein 446 OS=Homo sapiens GN=ZNF446 PE=1 SV=1//5.22749e-173
XM_007998440.1	1.6309	2.42E-21	1.03E-19	sp|O75467|Z324A_HUMAN Zinc finger protein 324A OS=Homo sapiens GN=ZNF324 PE=2 SV=1//0
XM_007998444.1	-0.54394	2.27E-14	5.90E-13	sp|P46782|RS5_HUMAN 40S ribosomal protein S5 OS=Homo sapiens GN=RPS5 PE=1 SV=4//9.19503e-150
XM_007998476.1	0.78628	1.62E-11	3.32E-10	sp|P24864|CCNE1_HUMAN G1/S-specific cyclin-E1 OS=Homo sapiens GN=CCNE1 PE=1 SV=2//0
XM_007998478.1	0.29295	0.000459	0.003637	sp|Q9C0C2|TB182_HUMAN 182 kDa tankyrase-1-binding protein OS=Homo sapiens GN=TNKS1BP1 PE=1 SV=4//0
XM_007998479.1	1.8665	1.12E-71	3.00E-69	sp|P53567|CEBPG_HUMAN CCAAT/enhancer-binding protein gamma OS=Homo sapiens GN=CEBPG PE=1 SV=1//8.80324e-75
XM_007998480.1	0.3009	0.000154	0.001322	sp|Q8ND56|LS14A_HUMAN Protein LSM14 homolog A OS=Homo sapiens GN=LSM14A PE=1 SV=3//0
XM_007998482.1	-1.1106	3.30E-05	0.000316	sp|Q9UF12|PROD2_HUMAN Probable proline dehydrogenase 2 OS=Homo sapiens GN=PRODH2 PE=2 SV=1//0
XM_007998484.1	0.87835	4.05E-15	1.12E-13	sp|O43379|WDR62_HUMAN WD repeat-containing protein 62 OS=Homo sapiens GN=WDR62 PE=1 SV=4//0
XM_007998487.1	0.59341	1.19E-05	0.000122	sp|O60292|SI1L3_HUMAN Signal-induced proliferation-associated 1-like protein 3 OS=Homo sapiens GN=SIPA1L3 PE=1 SV=3//0
XM_007998488.1	-1.7301	0.001808	0.012552	sp|P47929|LEG7_HUMAN Galectin-7 OS=Homo sapiens GN=LGALS7 PE=1 SV=2//1.58119e-67
XM_007998491.1	0.27884	0.004422	0.028045	sp|Q3ZCQ8|TIM50_HUMAN Mitochondrial import inner membrane translocase subunit TIM50 OS=Homo sapiens GN=TIMM50 PE=1 SV=2//0
XM_007998493.1	0.28336	0.000188	0.001592	sp|Q08945|SSRP1_HUMAN FACT complex subunit SSRP1 OS=Homo sapiens GN=SSRP1 PE=1 SV=1//0
XM_007998500.1	2.2328	5.97E-45	7.10E-43	sp|P32506|PVR_CHLAE Poliovirus receptor homolog OS=Chlorocebus aethiops GN=PVR PE=2 SV=1//0
XM_007998507.1	1.4333	3.62E-18	1.26E-16	sp|Q4R8W8|AAAT_MACFA Neutral amino acid transporter B(0) OS=Macaca fascicularis GN=SLC1A5 PE=2 SV=1//0
XM_007998511.1	0.62844	1.27E-06	1.50E-05	sp|Q14147|DHX34_HUMAN Probable ATP-dependent RNA helicase DHX34 OS=Homo sapiens GN=DHX34 PE=1 SV=2//0
XM_007998517.1	3.8937	0	0	sp|Q9H4M7|PKHA4_HUMAN Pleckstrin homology domain-containing family A

				member 4 OS=Homo sapiens GN=PLEKHA4 PE=1 SV=2//0
XM_007998519.1	-0.67921	1.77E-15	5.03E-14	sp|Q02818|NUCB1_HUMAN Nucleobindin-1 OS=Homo sapiens GN=NUCB1 PE=1 SV=4//1.207e-150
XM_007998522.1	-1.176	2.62E-14	6.79E-13	sp|Q8TD43|TRPM4_HUMAN Transient receptor potential cation channel subfamily M member 4 OS=Homo sapiens GN=TRPM4 PE=1 SV=1//0
XM_007998523.1	-0.7962	6.45E-09	1.00E-07	sp|Q8SPV9|FCGRN_MACFA IgG receptor FcRn large subunit p51 OS=Macaca fascicularis GN=FCGRT PE=2 SV=1//0
XM_007998527.1	-0.55685	1.54E-11	3.16E-10	sp|O95782|AP2A1_HUMAN AP-2 complex subunit alpha-1 OS=Homo sapiens GN=AP2A1 PE=1 SV=3//0
XM_007998528.1	-0.77143	2.04E-14	5.34E-13	sp|Q7Z406|MYH14_HUMAN Myosin-14 OS=Homo sapiens GN=MYH14 PE=1 SV=2//0
XM_007998541.1	1.6334	7.66E-05	0.000698	sp|Q8NHL6|LIRB1_HUMAN Leukocyte immunoglobulin-like receptor subfamily B member 1 OS=Homo sapiens GN=LILRB1 PE=1 SV=1//2.00476e-22
XM_007998545.1	-1.4631	8.35E-21	3.45E-19	sp|A1L4H1|SRCRL_HUMAN Soluble scavenger receptor cysteine-rich domain-containing protein SSC5D OS=Homo sapiens GN=SSC5D PE=1 SV=3//3.74338e-68
XM_007998553.1	0.49258	0.005186	0.032382	sp|Q96SQ5|ZN587_HUMAN Zinc finger protein 587 OS=Homo sapiens GN=ZNF587 PE=1 SV=1//5.07451e-06
XM_007998556.1	0.93915	9.50E-08	1.29E-06	sp|Q8IVC4|ZN584_HUMAN Zinc finger protein 584 OS=Homo sapiens GN=ZNF584 PE=2 SV=1//4.43806e-15
XM_007998603.1	-2.9273	4.90E-06	5.34E-05	sp|Q4R9D1|UBE2C_MACFA Ubiquitin-conjugating enzyme E2 C OS=Macaca fascicularis GN=UBE2C PE=2 SV=1//1.02661e-95
XM_007998604.1	-0.64158	4.50E-19	1.66E-17	sp|Q16270|IBP7_HUMAN Insulin-like growth factor-binding protein 7 OS=Homo sapiens GN=IGFBP7 PE=1 SV=1//7.57085e-108
XM_007998623.1	-0.88353	7.36E-07	9.05E-06	sp|Q6T311|ARL9_HUMAN ADP-ribosylation factor-like protein 9 OS=Homo sapiens GN=ARL9 PE=2 SV=1//6.9046e-117
XM_007998624.1	0.6813	1.00E-16	3.14E-15	sp|Q06203|PUR1_HUMAN Amidophosphoribosyltransferase OS=Homo sapiens GN=PPAT PE=1 SV=1//0
XM_007998686.1	-0.42618	1.68E-05	0.000168	sp|Q9H8P0|PORED_HUMAN Polyprenol reductase OS=Homo sapiens GN=SRD5A3 PE=1 SV=1//0
XM_007998701.1	1.7525	2.75E-52	4.43E-50	sp|Q9UKJ5|CHIC2_HUMAN Cysteine-rich hydrophobic domain-containing protein 2 OS=Homo sapiens GN=CHIC2 PE=1 SV=1//2.81135e-84
XM_007998719.1	1.0236	7.15E-12	1.51E-10	sp|Q9BPW5|RSLBB_HUMAN Ras-like protein family member 11B OS=Homo sapiens GN=RASL11B PE=2 SV=1//1.06326e-159
XM_007998731.1	-0.49187	9.55E-06	9.89E-05	sp|Q5R9U1|SGCB_PONAB Beta-sarcoglycan OS=Pongo abelii GN=SGCB PE=2 SV=1//0
XM_007998747.1	0.28215	8.91E-05	0.000799	sp|O76094|SRP72_HUMAN Signal recognition particle subunit SRP72 OS=Homo sapiens GN=SRP72 PE=1 SV=3//0
XM_007998749.1	-0.27292	0.005193	0.032405	sp|P0C864|DANCR_HUMAN Putative uncharacterized protein DANCR OS=Homo sapiens GN=DANCR PE=5 SV=1//1.9707e-35
XM_007998761.1	-0.91107	0.000743	0.005633	sp|O43704|ST1B1_HUMAN Sulfotransferase family cytosolic 1B member 1 OS=Homo sapiens GN=SULT1B1 PE=1 SV=2//0
XM_007998762.1	-1.7353	3.95E-12	8.50E-11	sp|P49888|ST1E1_HUMAN Estrogen sulfotransferase OS=Homo sapiens GN=SULT1E1 PE=1 SV=1//0
XM_007998798.1	Inf	2.95E-05	0.000286	sp|Q9NRM1|ENAM_HUMAN Enamelin OS=Homo sapiens GN=ENAM PE=1 SV=3//0
XM_007998871.1	4.0188	6.43E-23	3.03E-21	sp|P46653|IL8_CERAT Interleukin-8 OS=Cercocebus atys GN=CXCL8 PE=3 SV=1//1.91344e-45
XM_007998882.1	3.4662	5.85E-11	1.14E-09	sp|P19876|CXCL3_HUMAN C-X-C motif chemokine 3 OS=Homo sapiens GN=CXCL3 PE=1 SV=1//1.03713e-35
XM_007998939.1	Inf	0.001133	0.00827	sp|O14625|CXL11_HUMAN C-X-C motif chemokine 11 OS=Homo sapiens GN=CXCL11 PE=1 SV=1//2.81002e-54
XM_007998941.1	5.4121	4.01E-08	5.67E-07	sp|Q8MIZ1|CXL10_MACMU C-X-C motif chemokine 10 OS=Macaca mulatta GN=CXCL10 PE=3 SV=1//5.55659e-58
XM_007998945.1	-0.65453	4.65E-13	1.10E-11	sp|Q14108|SCRB2_HUMAN Lysosome membrane protein 2 OS=Homo sapiens GN=SCARB2 PE=1 SV=2//0
XM_007998948.1	1.4819	2.44E-26	1.38E-24	sp|O95210|STBD1_HUMAN Starch-binding domain-containing protein 1 OS=Homo sapiens GN=STBD1 PE=1 SV=1//0
XM_007998972.1	-0.5721	2.13E-06	2.45E-05	sp|Q9NVA2|SEP11_HUMAN Septin-11 OS=Homo sapiens GN=SEPT11 PE=1 SV=3//0
XM_007998989.1	1.4364	1.38E-18	4.90E-17	sp|P12429|ANXA3_HUMAN Annexin A3 OS=Homo sapiens GN=ANXA3 PE=1 SV=3//0
XM_007998993.1	0.81996	9.64E-06	9.98E-05	sp|Q6TCH7|PAQR3_HUMAN Progestin and adipoQ receptor family member 3 OS=Homo sapiens GN=PAQR3 PE=1 SV=2//0
XM_007999054.1	-4.3406	0.001202	0.008712	sp|Q12913|PTPRJ_HUMAN Receptor-type tyrosine-protein phosphatase eta OS=Homo sapiens GN=PTPRJ PE=1 SV=3//0
XM_007999059.1	1.0349	6.52E-11	1.26E-09	sp|Q9H5L6|THAP9_HUMAN DNA transposase THAP9 OS=Homo sapiens GN=THAP9 PE=1 SV=2//0
XM_007999103.1	-3.0365	4.42E-05	0.000415	sp|Q8N3X1|FNBP4_HUMAN Formin-binding protein 4 OS=Homo sapiens GN=FNBP4 PE=1 SV=3//4.97195e-10
XM_007999132.1	2.4565	3.07E-07	3.92E-06	sp|Q8N264|RHG24_HUMAN Rho GTPase-activating protein 24 OS=Homo sapiens GN=ARHGAP24 PE=1 SV=2//0
XM_007999135.1	2.4426	0.000569	0.004421	sp|Q8N264|RHG24_HUMAN Rho GTPase-activating protein 24 OS=Homo sapiens GN=ARHGAP24 PE=1 SV=2//0
XM_007999238.1	0.65144	0.000593	0.004591	sp|Q4W5G0|TIGD2_HUMAN Tigger transposable element-derived protein 2

				OS=Homo sapiens GN=TIGD2 PE=3 SV=1//0
XM_007999348.1	-0.67196	5.63E-08	7.80E-07	sp|Q0MQG7|NDUS3_GORGO NADH dehydrogenase [ubiquinone] iron-sulfur protein 3, mitochondrial OS=Gorilla gorilla gorilla GN=NDUFS3 PE=2 SV=1//0
XM_007999384.1	-1.1339	5.33E-07	6.64E-06	sp|Q6YNC8|H2AZ_SHEEP Histone H2A.Z OS=Ovis aries GN=H2AFZ PE=2 SV=3//1.84436e-70
XM_007999404.1	-0.89353	0.000214	0.001792	sp|Q9C0K1|S39A8_HUMAN Zinc transporter ZIP8 OS=Homo sapiens GN=SLC39A8 PE=2 SV=1//0
XM_007999425.1	-0.94469	2.48E-10	4.54E-09	sp|Q9BUT1|BDH2_HUMAN 3-hydroxybutyrate dehydrogenase type 2 OS=Homo sapiens GN=BDH2 PE=1 SV=2//1.19618e-160
XM_007999458.1	#NAME?	0.005889	0.036194	-//-
XM_007999526.1	-0.98442	5.31E-05	0.000494	sp|Q9BZM1|PG12A_HUMAN Group XIIA secretory phospholipase A2 OS=Homo sapiens GN=PLA2G12A PE=1 SV=1//8.64671e-106
XM_007999552.1	1.7127	1.18E-08	1.78E-07	sp|Q8N8J7|CD032_HUMAN Uncharacterized protein C4orf32 OS=Homo sapiens GN=C4orf32 PE=2 SV=2//8.85689e-61
XM_007999570.1	-0.89449	0.004884	0.03073	sp|Q5R844|MYL6_PONAB Myosin light polypeptide 6 OS=Pongo abelii GN=MYL6 PE=2 SV=3//1.20288e-102
XM_007999653.1	0.5413	1.68E-05	0.000168	sp|Q5R5N4|MET14_PONAB N6-adenosine-methyltransferase subunit METTL14 OS=Pongo abelii GN=METTL14 PE=2 SV=1//0
XM_007999662.1	0.73439	1.25E-12	2.81E-11	sp|Q70EK8|UBP53_HUMAN Inactive ubiquitin carboxyl-terminal hydrolase 53 OS=Homo sapiens GN=USP53 PE=2 SV=2//0
XM_007999671.1	-0.88027	2.65E-07	3.40E-06	sp|Q8WVX3|CD003_HUMAN Uncharacterized protein C4orf3 OS=Homo sapiens GN=C4orf3 PE=1 SV=2//9.86712e-07
XM_007999729.1	0.90834	0.000371	0.002994	sp|Q6ZW61|BBS12_HUMAN Bardet-Biedl syndrome 12 protein OS=Homo sapiens GN=BBS12 PE=1 SV=2//0
XM_007999748.1	-0.34331	0.005066	0.031687	sp|Q9ULJ7|ANR50_HUMAN Ankyrin repeat domain-containing protein 50 OS=Homo sapiens GN=ANKRD50 PE=1 SV=4//0
XM_007999750.1	-0.94268	6.95E-06	7.37E-05	sp|Q6V0I7|FAT4_HUMAN Protocadherin Fat 4 OS=Homo sapiens GN=FAT4 PE=1 SV=2//0
XM_007999757.1	0.30859	0.001645	0.011561	sp|O95757|HS74L_HUMAN Heat shock 70 kDa protein 4L OS=Homo sapiens GN=HSPA4L PE=1 SV=3//0
XM_007999785.1	0.42875	3.98E-06	4.40E-05	sp|O15173|PGRC2_HUMAN Membrane-associated progesterone receptor component 2 OS=Homo sapiens GN=PGRMC2 PE=1 SV=1//6.9783e-85
XM_007999802.1	0.51323	0.00669	0.040521	sp|P0CB38|PAB4L_HUMAN Polyadenylate-binding protein 4-like OS=Homo sapiens GN=PABPC4L PE=2 SV=1//1.1603e-33
XM_007999807.1	1.5419	9.58E-68	2.35E-65	sp|Q9UPY5|XCT_HUMAN Cystine/glutamate transporter OS=Homo sapiens GN=SLC7A11 PE=1 SV=1//0
XM_007999809.1	1.8226	0.000897	0.006683	sp|Q9UK39|NOCT_HUMAN Nocturnin OS=Homo sapiens GN=CCRN4L PE=2 SV=2//0
XM_007999835.1	0.59682	4.16E-09	6.65E-08	sp|Q8WTS6|SETD7_HUMAN Histone-lysine N-methyltransferase SETD7 OS=Homo sapiens GN=SETD7 PE=1 SV=1//0
XM_007999857.1	4.3377	0.006817	0.041181	sp|P25874|UCP1_HUMAN Mitochondrial brown fat uncoupling protein 1 OS=Homo sapiens GN=UCP1 PE=2 SV=3//0
XM_007999858.1	0.26452	0.006919	0.041705	sp|Q6ZT07|TBCD9_HUMAN TBC1 domain family member 9 OS=Homo sapiens GN=TBC1D9 PE=2 SV=2//0
XM_007999861.1	1.6452	6.79E-43	7.40E-41	sp|Q5R9D9|ZN330_PONAB Zinc finger protein 330 OS=Pongo abelii GN=ZNF330 PE=2 SV=1//0
XM_007999873.1	0.98154	1.88E-19	7.14E-18	sp|Q8NB14|UBP38_HUMAN Ubiquitin carboxyl-terminal hydrolase 38 OS=Homo sapiens GN=USP38 PE=1 SV=2//0
XM_007999914.1	1.3527	4.98E-27	2.90E-25	sp|Q17R98|ZN827_HUMAN Zinc finger protein 827 OS=Homo sapiens GN=ZNF827 PE=2 SV=1//0
XM_007999916.1	0.83627	1.13E-06	1.35E-05	sp|O75096|LRP4_HUMAN Low-density lipoprotein receptor-related protein 4 OS=Homo sapiens GN=LRP4 PE=1 SV=4//0
XM_007999949.1	1.6088	5.58E-18	1.92E-16	sp|Q9H9D4|ZN408_HUMAN Zinc finger protein 408 OS=Homo sapiens GN=ZNF408 PE=1 SV=1//0
XM_007999962.1	-1.8837	5.44E-05	0.000505	sp|D2I3C6|DCLK2_AILME Serine/threonine-protein kinase DCLK2 OS=Ailuropoda melanoleuca GN=DCLK2 PE=3 SV=1//0
XM_008000002.1	2.2422	7.41E-05	0.000676	sp|F1MNN4|FBXW7_BOVIN F-box/WD repeat-containing protein 7 OS=Bos taurus GN=FBXW7 PE=1 SV=2//0
XM_008000090.1	-0.77636	1.64E-08	2.43E-07	sp|P43234|CATO_HUMAN Cathepsin O OS=Homo sapiens GN=CTSO PE=2 SV=1//0
XM_008000121.1	-0.40791	0.001289	0.009273	sp|Q5RDD3|ETFD_PONAB Electron transfer flavoprotein-ubiquinone oxidoreductase, mitochondrial OS=Pongo abelii GN=ETFDH PE=2 SV=1//0
XM_008000152.1	1.2119	2.23E-05	0.000219	sp|Q96HR8|NAF1_HUMAN H/ACA ribonucleoprotein complex non-core subunit NAF1 OS=Homo sapiens GN=NAF1 PE=1 SV=2//0
XM_008000153.1	1.422	0.003227	0.021133	sp|Q96HR8|NAF1_HUMAN H/ACA ribonucleoprotein complex non-core subunit NAF1 OS=Homo sapiens GN=NAF1 PE=1 SV=2//2.11103e-167
XM_008000209.1	-0.61853	5.39E-08	7.50E-07	sp|P16870|CBPE_HUMAN Carboxypeptidase E OS=Homo sapiens GN=CPE PE=1 SV=1//0
XM_008000217.1	-0.4518	9.55E-10	1.63E-08	sp|P05787|K2C8_HUMAN Keratin, type II cytoskeletal 8 OS=Homo sapiens GN=KRT8 PE=1 SV=7//0
XM_008000224.1	1.7917	1.21E-23	5.93E-22	sp|Q8WX93|PALLD_HUMAN Palladin OS=Homo sapiens GN=PALLD PE=1 SV=3//0

XM_008000256.1	-0.97473	0.005407	0.033597	sp|O75121|MFA3L_HUMAN Microfibrillar-associated protein 3-like OS=Homo sapiens GN=MFAP3L PE=2 SV=3//0
XM_008000262.1	-0.83178	0.000127	0.001103	sp|Q4R3M6|NDUS5_MACFA NADH dehydrogenase [ubiquinone] iron-sulfur protein 5 OS=Macaca fascicularis GN=NDUFS5 PE=3 SV=3//1.76667e-62
XM_008000290.1	2.059	1.68E-77	5.26E-75	sp|Q8MJY8|PGDH_MACFA 15-hydroxyprostaglandin dehydrogenase [NAD(+)] OS=Macaca fascicularis GN=HPGD PE=2 SV=1//9.42077e-175
XM_008000329.1	1.7893	2.25E-27	1.34E-25	sp|P49767|VEGFC_HUMAN Vascular endothelial growth factor C OS=Homo sapiens GN=VEGFC PE=1 SV=1//0
XM_008000361.1	1.5655	1.73E-07	2.27E-06	sp|Q8N7P3|CLD22_HUMAN Claudin-22 OS=Homo sapiens GN=CLDN22 PE=2 SV=3//7.90506e-89
XM_008000364.1	2.3474	5.55E-44	6.29E-42	sp|Q9NXV6|CARF_HUMAN CDKN2A-interacting protein OS=Homo sapiens GN=CDKN2AIP PE=1 SV=3//8.06536e-87
XM_008000518.1	1.3015	2.42E-10	4.43E-09	sp|P30946|HS90A_RABIT Heat shock protein HSP 90-alpha OS=Oryctolagus cuniculus GN=HSP90AA1 PE=1 SV=2//2.80693e-121
XM_008000523.1	-1.1207	0.001774	0.012351	sp|Q8N3Y3|LARG2_HUMAN Glycosyltransferase-like protein LARGE2 OS=Homo sapiens GN=GYLTL1B PE=1 SV=2//0
XM_008000527.1	0.75077	6.10E-09	9.53E-08	sp|Q6UWZ7|F175A_HUMAN BRCA1-A complex subunit Abraxas OS=Homo sapiens GN=FAM175A PE=1 SV=2//0
XM_008000529.1	-0.65994	1.80E-11	3.67E-10	sp|P11766|ADHX_HUMAN Alcohol dehydrogenase class-3 OS=Homo sapiens GN=ADH5 PE=1 SV=4//0
XM_008000540.1	1.4803	7.53E-27	4.36E-25	sp|Q96NL6|SCLT1_HUMAN Sodium channel and clathrin linker 1 OS=Homo sapiens GN=SCLT1 PE=1 SV=2//0
XM_008000545.1	1.7784	0.000123	0.001075	-//-
XM_008000546.1	2.0623	1.55E-78	5.06E-76	sp|Q49AN0|CRY2_HUMAN Cryptochrome-2 OS=Homo sapiens GN=CRY2 PE=1 SV=2//0
XM_008000561.1	1.4502	7.97E-31	5.52E-29	sp|P49716|CEBPD_HUMAN CCAAT/enhancer-binding protein delta OS=Homo sapiens GN=CEBPD PE=1 SV=2//5.16075e-109
XM_008000567.1	0.57769	6.25E-11	1.22E-09	sp|P33991|MCM4_HUMAN DNA replication licensing factor MCM4 OS=Homo sapiens GN=MCM4 PE=1 SV=5//0
XM_008000574.1	3.0498	1.09E-43	1.21E-41	sp|O43623|SNAI2_HUMAN Zinc finger protein SNAI2 OS=Homo sapiens GN=SNAI2 PE=1 SV=1//1.96065e-170
XM_008000618.1	3.8656	0.000151	0.001297	sp|Q6UXT8|F150A_HUMAN Protein FAM150A OS=Homo sapiens GN=FAM150A PE=2 SV=1//2.39438e-53
XM_008000633.1	-1.1933	5.78E-13	1.35E-11	sp|A6NMD0|IFM10_HUMAN Interferon-induced transmembrane protein 10 OS=Homo sapiens GN=IFITM10 PE=2 SV=1//1.596e-121
XM_008000656.1	3.2385	1.43E-64	3.13E-62	sp|Q9H6I2|SOX17_HUMAN Transcription factor SOX-17 OS=Homo sapiens GN=SOX17 PE=1 SV=1//0
XM_008000681.1	-0.36988	0.000365	0.002948	sp|P60868|RS20_RAT 40S ribosomal protein S20 OS=Rattus norvegicus GN=Rps20 PE=3 SV=1//2.19764e-80
XM_008000697.1	-0.41488	9.55E-07	1.16E-05	sp|Q9NX62|IMPA3_HUMAN Inositol monophosphatase 3 OS=Homo sapiens GN=IMPAD1 PE=1 SV=1//0
XM_008000698.1	-0.80557	0.001166	0.008479	sp|Q9Y6H1|CHCH2_HUMAN Coiled-coil-helix-coiled-coil-helix domain-containing protein 2 OS=Homo sapiens GN=CHCHD2 PE=1 SV=1//5.68339e-39
XM_008000699.1	2.73	6.39E-67	1.50E-64	sp|Q5R5R3|F110B_PONAB Protein FAM110B OS=Pongo abelii GN=FAM110B PE=2 SV=1//0
XM_008000736.1	-0.90663	3.47E-39	3.36E-37	sp|P07339|CATD_HUMAN Cathepsin D OS=Homo sapiens GN=CTSD PE=1 SV=1//0
XM_008000744.1	0.86543	0.007206	0.043181	-//-
XM_008000762.1	0.50613	0.002222	0.015137	sp|Q5RFN3|MTFR1_PONAB Mitochondrial fission regulator 1 OS=Pongo abelii GN=MTFR1 PE=2 SV=1//0
XM_008000775.1	1.3284	3.48E-20	1.37E-18	sp|Q15050|RRS1_HUMAN Ribosome biogenesis regulatory protein homolog OS=Homo sapiens GN=RRS1 PE=1 SV=2//0
XM_008000777.1	3.6421	3.22E-13	7.70E-12	sp|Q8TAG6|CH046_HUMAN Uncharacterized protein C8orf46 OS=Homo sapiens GN=C8orf46 PE=2 SV=2//8.62454e-119
XM_008000798.1	0.60073	4.34E-06	4.77E-05	sp|Q1MSJ5|CSPP1_HUMAN Centrosome and spindle pole-associated protein 1 OS=Homo sapiens GN=CSPP1 PE=1 SV=4//0
XM_008000843.1	0.53901	4.70E-12	1.01E-10	sp|Q15629|TRAM1_HUMAN Translocating chain-associated membrane protein 1 OS=Homo sapiens GN=TRAM1 PE=1 SV=3//0
XM_008000844.1	1.602	0.00213	0.014547	sp|Q5GH70|XKR9_HUMAN XK-related protein 9 OS=Homo sapiens GN=XKR9 PE=2 SV=1//0
XM_008000846.1	0.92812	2.60E-12	5.70E-11	sp|Q53H82|LACB2_HUMAN Beta-lactamase-like protein 2 OS=Homo sapiens GN=LACTB2 PE=1 SV=2//0
XM_008000872.1	-0.94132	2.31E-08	3.36E-07	sp|P27701|CD82_HUMAN CD82 antigen OS=Homo sapiens GN=CD82 PE=1 SV=1//5.07108e-146
XM_008000873.1	-1.3568	4.95E-30	3.33E-28	sp|Q8IZV5|RDH10_HUMAN Retinol dehydrogenase 10 OS=Homo sapiens GN=RDH10 PE=1 SV=1//0
XM_008000874.1	-0.77463	3.11E-24	1.59E-22	sp|Q5R9R4|RL7_PONAB 60S ribosomal protein L7 OS=Pongo abelii GN=RPL7 PE=2 SV=1//6.50561e-166
XM_008000945.1	1.2427	3.40E-07	4.31E-06	sp|P13232|IL7_HUMAN Interleukin-7 OS=Homo sapiens GN=IL7 PE=1 SV=1//4.91853e-102
XM_008000964.1	-1.1348	1.75E-06	2.03E-05	sp|Q6ZNC4|ZN704_HUMAN Zinc finger protein 704 OS=Homo sapiens GN=ZNF704 PE=1 SV=1//0

XM_008000966.1	1.5589	3.40E-27	2.01E-25	sp|Q9NWQ8|PHAG1_HUMAN Phosphoprotein associated with glycosphingolipid-enriched microdomains 1 OS=Homo sapiens GN=PAG1 PE=1 SV=2//0
XM_008000970.1	-1.1447	0.002884	0.01904	sp|P15090|FABP4_HUMAN Fatty acid-binding protein, adipocyte OS=Homo sapiens GN=FABP4 PE=1 SV=3//1.10558e-87
XM_008000975.1	1.0814	1.30E-27	7.80E-26	sp|Q5R4X0|IMPA1_PONAB Inositol monophosphatase 1 OS=Pongo abelii GN=IMPA1 PE=2 SV=1//0
XM_008000979.1	1.1586	1.28E-18	4.58E-17	sp|Q8TCF1|ZFAN1_HUMAN AN1-type zinc finger protein 1 OS=Homo sapiens GN=ZFAND1 PE=1 SV=1//0
XM_008000996.1	-0.65597	0.000251	0.002083	sp|Q8N0T1|CH059_HUMAN Uncharacterized protein C8orf59 OS=Homo sapiens GN=C8orf59 PE=1 SV=4//1.37007e-51
XM_008001008.1	-1.1919	2.62E-17	8.67E-16	sp|P00918|CAH2_HUMAN Carbonic anhydrase 2 OS=Homo sapiens GN=CA2 PE=1 SV=2//0
XM_008001026.1	-0.5473	1.75E-09	2.90E-08	sp|O75131|CPNE3_HUMAN Copine-3 OS=Homo sapiens GN=CPNE3 PE=1 SV=1//0
XM_008001037.1	2.2288	1.13E-20	4.63E-19	sp|O43353|RIPK2_HUMAN Receptor-interacting serine/threonine-protein kinase 2 OS=Homo sapiens GN=RIPK2 PE=1 SV=2//0
XM_008001045.1	-0.5311	2.70E-07	3.47E-06	sp|Q16698|DECR_HUMAN 2,4-dienoyl-CoA reductase, mitochondrial OS=Homo sapiens GN=DECR1 PE=1 SV=1//0
XM_008001046.1	-2.7653	0.00186	0.012859	sp|P05937|CALB1_HUMAN Calbindin OS=Homo sapiens GN=CALB1 PE=1 SV=2//5.74667e-179
XM_008001152.1	-1.3181	2.66E-22	1.21E-20	sp|P14927|QCR7_HUMAN Cytochrome b-c1 complex subunit 7 OS=Homo sapiens GN=UQCRB PE=1 SV=2//5.28997e-64
XM_008001154.1	0.65222	0.00029	0.002377	sp|Q96E29|MTEF3_HUMAN Transcription termination factor 3, mitochondrial OS=Homo sapiens GN=MTERF3 PE=1 SV=2//0
XM_008001161.1	-0.99907	1.47E-17	4.93E-16	sp|P63159|HMGB1_RAT High mobility group protein B1 OS=Rattus norvegicus GN=Hmgb1 PE=1 SV=2//2.91306e-103
XM_008001180.1	-0.36181	0.005632	0.034845	sp|P52758|UK114_HUMAN Ribonuclease UK114 OS=Homo sapiens GN=HRSP12 PE=1 SV=1//5.24832e-92
XM_008001189.1	-0.90567	0.00757	0.045009	sp|Q7YRK2|COX6C_MACSL Cytochrome c oxidase subunit 6C OS=Macaca silenus GN=COX6C PE=3 SV=1//1.36489e-44
XM_008001215.1	0.48777	0.001108	0.008105	sp|Q4G163|FBX43_HUMAN F-box only protein 43 OS=Homo sapiens GN=FBXO43 PE=1 SV=3//0
XM_008001220.1	0.92636	1.10E-19	4.22E-18	sp|Q9NV58|RN19A_HUMAN E3 ubiquitin-protein ligase RNF19A OS=Homo sapiens GN=RNF19A PE=1 SV=3//0
XM_008001230.1	-0.25287	0.000164	0.001402	sp|P11940|PABP1_HUMAN Polyadenylate-binding protein 1 OS=Homo sapiens GN=PABPC1 PE=1 SV=2//1.58394e-51
XM_008001255.1	0.50625	1.36E-08	2.04E-07	sp|Q4R741|RIR2B_MACFA Ribonucleoside-diphosphate reductase subunit M2 B OS=Macaca fascicularis GN=RRM2B PE=2 SV=1//0
XM_008001264.1	3.1451	8.79683882420339e-320	2.13909999975461e-316	sp|Q13118|KLF10_HUMAN Krueppel-like factor 10 OS=Homo sapiens GN=KLF10 PE=1 SV=1//0
XM_008001268.1	0.91197	1.18E-22	5.43E-21	sp|Q5RDQ7|VATC1_PONAB V-type proton ATPase subunit C 1 OS=Pongo abelii GN=ATP6V1C1 PE=2 SV=1//0
XM_008001274.1	0.45625	6.85E-07	8.45E-06	sp|Q5R4T8|DCA13_PONAB DDB1- and CUL4-associated factor 13 OS=Pongo abelii GN=DCAF13 PE=2 SV=1//0
XM_008001275.1	1.1993	9.63E-27	5.55E-25	sp|Q95J75|MFTC_MACFA Mitochondrial folate transporter/carrier OS=Macaca fascicularis GN=SLC25A32 PE=2 SV=1//0
XM_008001406.1	-0.53292	3.97E-13	9.41E-12	sp|O15372|EIF3H_HUMAN Eukaryotic translation initiation factor 3 subunit H OS=Homo sapiens GN=EIF3H PE=1 SV=1//0
XM_008001407.1	0.5122	5.97E-07	7.40E-06	sp|Q9BRU9|UTP23_HUMAN rRNA-processing protein UTP23 homolog OS=Homo sapiens GN=UTP23 PE=1 SV=2//4.63467e-159
XM_008001451.1	-0.41757	7.17E-06	7.58E-05	sp|Q9BYD1|RM13_HUMAN 39S ribosomal protein L13, mitochondrial OS=Homo sapiens GN=MRPL13 PE=1 SV=1//1.82322e-126
XM_008001452.1	0.6457	1.08E-05	0.000111	sp|Q13884|SNTB1_HUMAN Beta-1-syntrophin OS=Homo sapiens GN=SNTB1 PE=1 SV=3//0
XM_008001480.1	0.80735	2.58E-09	4.22E-08	sp|Q96HA8|NTAQ1_HUMAN Protein N-terminal glutamine amidohydrolase OS=Homo sapiens GN=WDYHV1 PE=1 SV=2//1.49075e-146
XM_008001482.1	1.07	7.87E-05	0.000714	sp|Q658Y4|F91A1_HUMAN Protein FAM91A1 OS=Homo sapiens GN=FAM91A1 PE=1 SV=3//0
XM_008001491.1	0.74259	1.91E-16	5.85E-15	sp|Q4VAE3|TMM65_MOUSE Transmembrane protein 65 OS=Mus musculus GN=Tmem65 PE=2 SV=1//3.34095e-133
XM_008001492.1	1.0698	5.53E-32	3.98E-30	sp|Q8WU17|RN139_HUMAN E3 ubiquitin-protein ligase RNF139 OS=Homo sapiens GN=RNF139 PE=1 SV=1//0
XM_008001494.1	-0.51665	2.78E-09	4.53E-08	sp|Q0MQE9|NDUB9_GORGO NADH dehydrogenase [ubiquinone] 1 beta subcomplex subunit 9 OS=Gorilla gorilla gorilla GN=NDUFB9 PE=2 SV=3//1.78253e-117
XM_008001521.1	1.8983	########	########	sp|Q96RU8|TRIB1_HUMAN Tribbles homolog 1 OS=Homo sapiens GN=TRIB1 PE=1 SV=2//0
XM_008001522.1	0.94828	9.52E-24	4.71E-22	sp|Q96KN1|FA84B_HUMAN Protein FAM84B OS=Homo sapiens GN=FAM84B PE=1 SV=1//7.97128e-177
XM_008001702.1	-0.98767	1.89E-09	3.13E-08	sp|Q14210|LY6D_HUMAN Lymphocyte antigen 6D OS=Homo sapiens GN=LY6D PE=1 SV=1//3.6993e-43

XM_008001722.1	1.4004	3.29E-33	2.52E-31	sp|B6CJY4|TRAF6_CERAT TNF receptor-associated factor 6 OS=Cercocebus atys GN=TRAF6 PE=2 SV=1//0
XM_008001735.1	1.1115	8.70E-15	2.37E-13	-//-
XM_008001755.1	-2.3088	0.000165	0.001408	sp|Q969P6|TOP1M_HUMAN DNA topoisomerase I, mitochondrial OS=Homo sapiens GN=TOP1MT PE=1 SV=1//0
XM_008001757.1	-1.3579	0.005673	0.03504	sp|Q969P6|TOP1M_HUMAN DNA topoisomerase I, mitochondrial OS=Homo sapiens GN=TOP1MT PE=1 SV=1//2.77684e-08
XM_008001758.1	-1.4416	0.001901	0.013106	sp|Q969P6|TOP1M_HUMAN DNA topoisomerase I, mitochondrial OS=Homo sapiens GN=TOP1MT PE=1 SV=1//0
XM_008001782.1	1.6629	5.19E-13	1.22E-11	sp|Q53EQ6|TIGD5_HUMAN Tigger transposable element-derived protein 5 OS=Homo sapiens GN=TIGD5 PE=1 SV=3//0
XM_008001786.1	-2.3252	0.007437	0.044349	sp|Q4R3D4|EF1D_MACFA Elongation factor 1-delta OS=Macaca fascicularis GN=EEF1D PE=2 SV=2//1.5434e-165
XM_008001796.1	-0.34588	0.000496	0.003898	sp|Q13630|FCL_HUMAN GDP-L-fucose synthase OS=Homo sapiens GN=TSTA3 PE=1 SV=1//0
XM_008001870.1	-0.5845	2.72E-05	0.000264	sp|Q7Z429|LFG1_HUMAN Protein lifeguard 1 OS=Homo sapiens GN=GRINA PE=2 SV=1//2.06148e-138
XM_008001883.1	-0.77596	5.64E-07	7.01E-06	sp|Q9D0U6|MAF1_MOUSE Repressor of RNA polymerase III transcription MAF1 homolog OS=Mus musculus GN=Maf1 PE=2 SV=1//2.54494e-158
XM_008001886.1	-0.23855	0.002435	0.01638	sp|P08574|CY1_HUMAN Cytochrome c1, heme protein, mitochondrial OS=Homo sapiens GN=CYC1 PE=1 SV=3//3.1879e-169
XM_008001887.1	1.0525	1.09E-16	3.39E-15	sp|Q9BTY7|HGH1_HUMAN Protein HGH1 homolog OS=Homo sapiens GN=HGH1 PE=1 SV=1//1.03054e-176
XM_008001912.1	-0.38033	0.000221	0.001846	sp|Q9GMF1|DGAT1_CHLAE Diacylglycerol O-acyltransferase 1 OS=Chlorocebus aethiops GN=DGAT1 PE=2 SV=1//0
XM_008001976.1	-0.51648	5.82E-09	9.13E-08	sp|Q86VR8|FJX1_HUMAN Four-jointed box protein 1 OS=Homo sapiens GN=FJX1 PE=2 SV=1//0
XM_008002024.1	0.80494	0.001832	0.012687	sp|P15622|ZN250_HUMAN Zinc finger protein 250 OS=Homo sapiens GN=ZNF250 PE=1 SV=3//0
XM_008002031.1	0.80116	1.93E-14	5.08E-13	sp|Q9XSR1|ZN252_CANFA Zinc finger protein 252 OS=Canis familiaris GN=ZNF252 PE=2 SV=1//0
XM_008002032.1	0.50234	7.78E-06	8.18E-05	sp|Q5RDQ4|CH033_PONAB UPF0488 protein C8orf33 homolog OS=Pongo abelii PE=2 SV=1//2.63066e-99
XM_008002050.1	-0.28988	0.004998	0.031323	sp|Q7Z388|D19L4_HUMAN Probable C-mannosyltransferase DPY19L4 OS=Homo sapiens GN=DPY19L4 PE=1 SV=1//0
XM_008002054.1	1.0972	1.72E-19	6.57E-18	sp|Q6PL18|ATAD2_HUMAN ATPase family AAA domain-containing protein 2 OS=Homo sapiens GN=ATAD2 PE=1 SV=1//6.34445e-14
XM_008002056.1	2.4076	2.36E-14	6.13E-13	-//-
XM_008002057.1	0.2111	0.005357	0.033314	sp|Q05397|FAK1_HUMAN Focal adhesion kinase 1 OS=Homo sapiens GN=PTK2 PE=1 SV=2//0
XM_008002059.1	-2.0358	4.15E-06	4.58E-05	sp|P58107|EPIPL_HUMAN Epiplakin OS=Homo sapiens GN=EPPK1 PE=1 SV=2//1.97113e-19
XM_008002060.1	-1.3287	0.001557	0.011023	sp|A6NE52|K1875_HUMAN WD repeat-containing protein KIAA1875 OS=Homo sapiens GN=KIAA1875 PE=2 SV=2//0
XM_008002088.1	-2.463	7.37E-06	7.78E-05	-//-
XM_008002114.1	4.3187	########	########	sp|Q99612|KLF6_HUMAN Krueppel-like factor 6 OS=Homo sapiens GN=KLF6 PE=1 SV=3//2.06126e-145
XM_008002130.1	-0.7185	2.05E-11	4.16E-10	sp|Q95JH7|AK1C1_MACFA Aldo-keto reductase family 1 member C1 homolog OS=Macaca fascicularis GN=AKR1C1 PE=1 SV=1//0
XM_008002176.1	1.6634	3.42E-06	3.83E-05	sp|Q6P6B7|ANR16_HUMAN Ankyrin repeat domain-containing protein 16 OS=Homo sapiens GN=ANKRD16 PE=1 SV=1//0
XM_008002195.1	0.31709	0.000774	0.005842	sp|Q96I25|SPF45_HUMAN Splicing factor 45 OS=Homo sapiens GN=RBM17 PE=1 SV=1//0
XM_008002210.1	0.5194	1.73E-06	2.01E-05	sp|O00330|ODPX_HUMAN Pyruvate dehydrogenase protein X component, mitochondrial OS=Homo sapiens GN=PDHX PE=1 SV=3//0
XM_008002218.1	Inf	0.006828	0.041231	sp|Q86UX2|ITIH5_HUMAN Inter-alpha-trypsin inhibitor heavy chain H5 OS=Homo sapiens GN=ITIH5 PE=2 SV=2//0
XM_008002219.1	-0.24722	0.00571	0.03525	sp|P19823|ITIH2_HUMAN Inter-alpha-trypsin inhibitor heavy chain H2 OS=Homo sapiens GN=ITIH2 PE=1 SV=2//0
XM_008002222.1	0.62874	1.15E-06	1.38E-05	sp|O60870|KIN17_HUMAN DNA/RNA-binding protein KIN17 OS=Homo sapiens GN=KIN PE=1 SV=2//0
XM_008002223.1	0.96382	7.47E-11	1.44E-09	sp|Q5VWG9|TAF3_HUMAN Transcription initiation factor TFIID subunit 3 OS=Homo sapiens GN=TAF3 PE=1 SV=1//1.20559e-55
XM_008002257.1	1.2676	5.23E-10	9.21E-09	sp|Q86WR7|PRSR2_HUMAN Proline and serine-rich protein 2 OS=Homo sapiens GN=PROSER2 PE=1 SV=2//0
XM_008002260.1	0.60298	1.58E-08	2.35E-07	sp|Q9JLR1|S61A2_MOUSE Protein transport protein Sec61 subunit alpha isoform 2 OS=Mus musculus GN=Sec61a2 PE=2 SV=3//0
XM_008002263.1	-0.32777	0.006814	0.041181	sp|Q9UKK9|NUDT5_HUMAN ADP-sugar pyrophosphatase OS=Homo sapiens GN=NUDT5 PE=1 SV=1//3.54343e-148
XM_008002289.1	-0.64213	7.32E-08	1.00E-06	sp|O14832|PAHX_HUMAN Phytanoyl-CoA dioxygenase, peroxisomal OS=Homo

				sapiens GN=PHYH PE=1 SV=1//0
XM_008002332.1	0.83561	3.02E-10	5.46E-09	sp|Q9P2Q2|FRM4A_HUMAN FERM domain-containing protein 4A OS=Homo sapiens GN=FRMD4A PE=1 SV=3//0
XM_008002340.1	1.6134	3.19E-09	5.16E-08	sp|Q5RE21|HSP7E_PONAB Heat shock 70 kDa protein 14 OS=Pongo abelii GN=HSPA14 PE=2 SV=1//4.75728e-40
XM_008002350.1	-0.74903	3.78E-17	1.23E-15	sp|P04040|CATA_HUMAN Catalase OS=Homo sapiens GN=CAT PE=1 SV=3//0
XM_008002365.1	0.83308	0.001964	0.013488	sp|O60551|NMT2_HUMAN Glycylpeptide N-tetradecanoyltransferase 2 OS=Homo sapiens GN=NMT2 PE=1 SV=1//0
XM_008002369.1	Inf	0.002754	0.018267	sp|P53708|ITA8_HUMAN Integrin alpha-8 OS=Homo sapiens GN=ITGA8 PE=1 SV=3//0
XM_008002379.1	1.6818	4.32E-79	1.43E-76	sp|Q8N961|ABTB2_HUMAN Ankyrin repeat and BTB/POZ domain-containing protein 2 OS=Homo sapiens GN=ABTB2 PE=2 SV=2//0
XM_008002385.1	1.2585	0.000479	0.003782	sp|O60494|CUBN_HUMAN Cubilin OS=Homo sapiens GN=CUBN PE=1 SV=5//0
XM_008002391.1	1.0813	7.71E-25	4.02E-23	sp|Q9H0A0|NAT10_HUMAN N-acetyltransferase 10 OS=Homo sapiens GN=NAT10 PE=1 SV=2//0
XM_008002397.1	-0.52081	6.57E-14	1.66E-12	sp|Q4R4X4|VIME_MACFA Vimentin OS=Macaca fascicularis GN=VIM PE=2 SV=3//0
XM_008002400.1	0.28311	0.000147	0.001268	sp|Q14444|CAPR1_HUMAN Caprin-1 OS=Homo sapiens GN=CAPRIN1 PE=1 SV=2//0
XM_008002432.1	0.83362	6.44E-17	2.05E-15	sp|Q9D4P0|ARL5B_MOUSE ADP-ribosylation factor-like protein 5B OS=Mus musculus GN=Arl5b PE=2 SV=3//1.98025e-116
XM_008002433.1	1.0156	3.07E-12	6.70E-11	sp|Q8TEA1|NSUN6_HUMAN Putative methyltransferase NSUN6 OS=Homo sapiens GN=NSUN6 PE=1 SV=1//0
XM_008002454.1	1.4059	3.86E-19	1.43E-17	sp|Q5T4H9|CSC10_HUMAN Protein CASC10 OS=Homo sapiens GN=CASC10 PE=2 SV=1//9.02644e-43
XM_008002475.1	-0.48065	0.001413	0.010094	sp|Q9UBI1|COMD3_HUMAN COMM domain-containing protein 3 OS=Homo sapiens GN=COMMD3 PE=1 SV=1//4.71813e-139
XM_008002499.1	-0.44768	0.002647	0.017638	sp|Q9Y3D2|MSRB2_HUMAN Methionine-R-sulfoxide reductase B2, mitochondrial OS=Homo sapiens GN=MSRB2 PE=1 SV=2//1.35142e-97
XM_008002513.1	1.7696	2.55E-31	1.79E-29	sp|Q5VV17|OTUD1_HUMAN OTU domain-containing protein 1 OS=Homo sapiens GN=OTUD1 PE=1 SV=1//0
XM_008002595.1	0.89321	8.64E-06	9.03E-05	sp|Q5T2R2|DPS1_HUMAN Decaprenyl-diphosphate synthase subunit 1 OS=Homo sapiens GN=PDSS1 PE=1 SV=1//0
XM_008002666.1	1.3359	2.86E-49	4.07E-47	sp|Q13145|BAMBI_HUMAN BMP and activin membrane-bound inhibitor homolog OS=Homo sapiens GN=BAMBI PE=1 SV=1//1.19852e-173
XM_008002671.1	1.1948	5.84E-52	9.12E-50	sp|O95425|SVIL_HUMAN Supervillin OS=Homo sapiens GN=SVIL PE=1 SV=2//0
XM_008002673.1	-0.49433	2.16E-06	2.48E-05	sp|Q2KHR3|QSER1_HUMAN Glutamine and serine-rich protein 1 OS=Homo sapiens GN=QSER1 PE=1 SV=3//0
XM_008002678.1	-1.6427	0.001274	0.009174	sp|Q08379|GOGA2_HUMAN Golgin subfamily A member 2 OS=Homo sapiens GN=GOLGA2 PE=1 SV=3//9.69499e-11
XM_008002723.1	0.65898	1.27E-14	3.42E-13	sp|P33176|KINH_HUMAN Kinesin-1 heavy chain OS=Homo sapiens GN=KIF5B PE=1 SV=1//0
XM_008002752.1	-0.33704	1.42E-06	1.67E-05	sp|P05556|ITB1_HUMAN Integrin beta-1 OS=Homo sapiens GN=ITGB1 PE=1 SV=2//0
XM_008002753.1	0.29486	0.000926	0.006864	sp|Q7L2H7|EIF3M_HUMAN Eukaryotic translation initiation factor 3 subunit M OS=Homo sapiens GN=EIF3M PE=1 SV=1//0
XM_008002812.1	-0.40731	7.88E-08	1.07E-06	sp|Q15293|RCN1_HUMAN Reticulocalbin-1 OS=Homo sapiens GN=RCN1 PE=1 SV=1//0
XM_008002829.1	0.39114	0.000471	0.003721	sp|Q92615|LAR4B_HUMAN La-related protein 4B OS=Homo sapiens GN=LARP4B PE=1 SV=3//0
XM_008002837.1	1.3248	6.16E-13	1.44E-11	sp|Q5R6Z9|DCR1C_PONAB Protein artemis OS=Pongo abelii GN=DCLRE1C PE=2 SV=1//0
XM_008002957.1	0.73454	1.97E-22	9.02E-21	sp|Q68CP9|ARID2_HUMAN AT-rich interactive domain-containing protein 2 OS=Homo sapiens GN=ARID2 PE=1 SV=2//0
XM_008003068.1	1.1514	2.08E-07	2.71E-06	sp|Q6P3W2|DJC24_HUMAN DnaJ homolog subfamily C member 24 OS=Homo sapiens GN=DNAJC24 PE=1 SV=1//1.45269e-89
XM_008003079.1	-1.8129	8.96E-06	9.33E-05	sp|P54284|CACB3_HUMAN Voltage-dependent L-type calcium channel subunit beta-3 OS=Homo sapiens GN=CACNB3 PE=1 SV=1//0
XM_008003080.1	-1.1387	0.000191	0.001615	sp|P54284|CACB3_HUMAN Voltage-dependent L-type calcium channel subunit beta-3 OS=Homo sapiens GN=CACNB3 PE=1 SV=1//0
XM_008003082.1	0.21497	0.005054	0.031648	sp|Q9BUQ8|DDX23_HUMAN Probable ATP-dependent RNA helicase DDX23 OS=Homo sapiens GN=DDX23 PE=1 SV=3//0
XM_008003083.1	2.0814	0.000338	0.002746	sp|Q92730|RND1_HUMAN Rho-related GTP-binding protein Rho6 OS=Homo sapiens GN=RND1 PE=1 SV=1//2.08517e-162
XM_008003124.1	0.41372	0.002419	0.016287	sp|Q4R7X9|LMBRL_MACFA Protein LMBR1L OS=Macaca fascicularis GN=LMBR1L PE=2 SV=1//0
XM_008003125.1	-0.31348	5.66E-06	6.12E-05	sp|Q6P9V9|TBA1B_RAT Tubulin alpha-1B chain OS=Rattus norvegicus GN=Tuba1b PE=1 SV=1//0
XM_008003130.1	-0.68326	5.46E-19	1.99E-17	sp|Q9BQE3|TBA1C_HUMAN Tubulin alpha-1C chain OS=Homo sapiens GN=TUBA1C PE=1 SV=1//0
XM_008003131.1	-0.6731	6.33E-05	0.000583	sp|P41219|PERI_HUMAN Peripherin OS=Homo sapiens GN=PRPH PE=1 SV=2//0
XM_008003143.1	-0.52404	0.001979	0.01358	sp|Q5RBD7|DJC22_PONAB DnaJ homolog subfamily C member 22 OS=Pongo abelii

				GN=DNAJC22 PE=2 SV=1//0
XM_008003175.1	-0.95492	1.75E-40	1.78E-38	sp|Q5R7R1|BI1_PONAB Bax inhibitor 1 OS=Pongo abelii GN=TMBIM6 PE=2 SV=2//1.34919e-116
XM_008003181.1	0.82063	1.58E-13	3.88E-12	sp|Q7Z5W3|BN3D2_HUMAN Pre-miRNA 5'-monophosphate methyltransferase OS=Homo sapiens GN=BCDIN3D PE=1 SV=1//0
XM_008003234.1	Inf	0.002739	0.018182	sp|P18846|ATF1_HUMAN Cyclic AMP-dependent transcription factor ATF-1 OS=Homo sapiens GN=ATF1 PE=1 SV=2//1.11133e-150
XM_008003237.1	-1.4437	1.51E-33	1.18E-31	sp|Q9H8H3|MET7A_HUMAN Methyltransferase-like protein 7A OS=Homo sapiens GN=METTL7A PE=1 SV=1//3.40548e-158
XM_008003248.1	-4.8765	2.99E-05	0.000289	sp|Q9H175|CSRN2_HUMAN Cysteine/serine-rich nuclear protein 2 OS=Homo sapiens GN=CSRNP2 PE=1 SV=1//0
XM_008003302.1	-1.0895	6.74E-05	0.000619	sp|A6NMB9|FIGL2_HUMAN Putative fidgetin-like protein 2 OS=Homo sapiens GN=FIGNL2 PE=5 SV=2//8.85103e-62
XM_008003310.1	-2.9404	1.50E-05	0.000151	-//-
XM_008003325.1	0.61305	2.48E-17	8.23E-16	sp|A5A6N0|K2C7_PANTR Keratin, type II cytoskeletal 7 OS=Pan troglodytes GN=KRT7 PE=2 SV=1//0
XM_008003327.1	0.34269	0.008289	0.048897	sp|Q14533|KRT81_HUMAN Keratin, type II cuticular Hb1 OS=Homo sapiens GN=KRT81 PE=1 SV=3//0
XM_008003367.1	0.6404	5.37E-12	1.14E-10	sp|Q8NCJ5|SPRY3_HUMAN SPRY domain-containing protein 3 OS=Homo sapiens GN=SPRYD3 PE=1 SV=2//0
XM_008003408.1	-0.63098	3.08E-13	7.39E-12	sp|Q5RAY0|PFD5_PONAB Prefoldin subunit 5 OS=Pongo abelii GN=PFDN5 PE=2 SV=1//8.30559e-89
XM_008003413.1	-2.4786	0.000959	0.007097	sp|Q8TDD2|SP7_HUMAN Transcription factor Sp7 OS=Homo sapiens GN=SP7 PE=1 SV=1//0
XM_008003419.1	0.32794	0.004041	0.025927	sp|Q8N8R7|AL14E_HUMAN ARL14 effector protein OS=Homo sapiens GN=ARL14EP PE=1 SV=1//1.74922e-170
XM_008003451.1	-1.0941	6.99E-05	0.000639	sp|Q61990|PCBP2_MOUSE Poly(rC)-binding protein 2 OS=Mus musculus GN=Pcbp2 PE=1 SV=1//0
XM_008003473.1	-0.59618	1.40E-14	3.75E-13	sp|Q06055|AT5G2_HUMAN ATP synthase F(0) complex subunit C2, mitochondrial OS=Homo sapiens GN=ATP5G2 PE=2 SV=1//1.58937e-69
XM_008003478.1	-3.1852	0.004213	0.026867	sp|Q9P1Z2|CACO1_HUMAN Calcium-binding and coiled-coil domain-containing protein 1 OS=Homo sapiens GN=CALCOCO1 PE=1 SV=2//0
XM_008003480.1	0.75782	0.000128	0.001113	sp|P31276|HXC13_HUMAN Homeobox protein Hox-C13 OS=Homo sapiens GN=HOXC13 PE=1 SV=3//9.505e-157
XM_008003495.1	-1.8428	9.83E-08	1.33E-06	sp|P09025|HXC8_MOUSE Homeobox protein Hox-C8 OS=Mus musculus GN=Hoxc8 PE=2 SV=2//4.36668e-155
XM_008003516.1	-1.2096	2.94E-14	7.59E-13	sp|P08648|ITA5_HUMAN Integrin alpha-5 OS=Homo sapiens GN=ITGA5 PE=1 SV=2//0
XM_008003560.1	-0.91101	2.50E-08	3.62E-07	sp|Q6UX53|MET7B_HUMAN Methyltransferase-like protein 7B OS=Homo sapiens GN=METTL7B PE=1 SV=2//6.97508e-154
XM_008003564.1	-0.6541	1.33E-06	1.57E-05	sp|Q5R7L8|BL1S1_PONAB Biogenesis of lysosome-related organelles complex 1 subunit 1 OS=Pongo abelii GN=BLOC1S1 PE=2 SV=2//3.11176e-75
XM_008003573.1	-0.77822	4.27E-13	1.01E-11	sp|O95390|GDF11_HUMAN Growth/differentiation factor 11 OS=Homo sapiens GN=GDF11 PE=2 SV=1//0
XM_008003578.1	-0.28524	0.003405	0.022199	sp|Q6Y2X3|DJC14_HUMAN DnaJ homolog subfamily C member 14 OS=Homo sapiens GN=DNAJC14 PE=2 SV=2//0
XM_008003581.1	-0.89707	1.33E-31	9.45E-30	sp|Q5RBG1|RAB5B_PONAB Ras-related protein Rab-5B OS=Pongo abelii GN=RAB5B PE=2 SV=1//6.73182e-136
XM_008003601.1	-0.72549	0.000513	0.004025	sp|Q9H2S9|IKZF4_HUMAN Zinc finger protein Eos OS=Homo sapiens GN=IKZF4 PE=1 SV=2//3.72414e-09
XM_008003604.1	1.5905	1.35E-39	1.33E-37	sp|Q8NI77|KI18A_HUMAN Kinesin-like protein KIF18A OS=Homo sapiens GN=KIF18A PE=1 SV=2//0
XM_008003606.1	-0.90002	4.22E-26	2.36E-24	sp|Q64119|MYL6_RAT Myosin light polypeptide 6 OS=Rattus norvegicus GN=Myl6 PE=1 SV=3//7.04173e-105
XM_008003613.1	-0.56223	0.002098	0.014354	sp|Q96K80|ZC3HA_HUMAN Zinc finger CCCH domain-containing protein 10 OS=Homo sapiens GN=ZC3H10 PE=1 SV=1//0
XM_008003624.1	0.49915	5.90E-06	6.34E-05	sp|Q5R7T5|RNF41_PONAB E3 ubiquitin-protein ligase NRDP1 OS=Pongo abelii GN=RNF41 PE=2 SV=1//0
XM_008003626.1	-0.32901	1.15E-05	0.000118	sp|Q8NB46|ANR52_HUMAN Serine/threonine-protein phosphatase 6 regulatory ankyrin repeat subunit C OS=Homo sapiens GN=ANKRD52 PE=1 SV=3//0
XM_008003651.1	-0.28596	0.002428	0.016339	sp|Q9Y2B0|CNPY2_HUMAN Protein canopy homolog 2 OS=Homo sapiens GN=CNPY2 PE=1 SV=1//3.15232e-111
XM_008003700.1	-0.38923	3.52E-08	5.01E-07	sp|P06576|ATPB_HUMAN ATP synthase subunit beta, mitochondrial OS=Homo sapiens GN=ATP5B PE=1 SV=3//0
XM_008003705.1	-0.65324	1.02E-15	2.95E-14	sp|Q60817|NACA_MOUSE Nascent polypeptide-associated complex subunit alpha OS=Mus musculus GN=Naca PE=1 SV=1//8.25582e-73
XM_008003715.1	0.61148	8.07E-09	1.24E-07	sp|O15060|ZBT39_HUMAN Zinc finger and BTB domain-containing protein 39 OS=Homo sapiens GN=ZBTB39 PE=2 SV=1//0
XM_008003739.1	-0.73368	6.23E-12	1.32E-10	sp|Q07954|LRP1_HUMAN Prolow-density lipoprotein receptor-related protein 1 OS=Homo sapiens GN=LRP1 PE=1 SV=2//0

XM_008003740.1	-0.91795	6.36E-09	9.91E-08	sp|Q9Z2N4|NXPH4_RAT Neurexophilin-4 OS=Rattus norvegicus GN=Nxph4 PE=2 SV=1//2.9423e-138
XM_008003745.1	-0.65593	7.27E-17	2.29E-15	sp|Q9BXB1|LGR4_HUMAN Leucine-rich repeat-containing G-protein coupled receptor 4 OS=Homo sapiens GN=LGR4 PE=1 SV=2//0
XM_008003758.1	0.3189	0.003137	0.02057	sp|Q96HJ3|CCD34_HUMAN Coiled-coil domain-containing protein 34 OS=Homo sapiens GN=CCDC34 PE=2 SV=2//3.61474e-147
XM_008003776.1	2.9615	1.30E-38	1.23E-36	sp|P58166|INHBE_HUMAN Inhibin beta E chain OS=Homo sapiens GN=INHBE PE=1 SV=1//0
XM_008003781.1	0.55815	2.69E-09	4.38E-08	sp|P56192|SYMC_HUMAN Methionine--tRNA ligase, cytoplasmic OS=Homo sapiens GN=MARS PE=1 SV=2//0
XM_008003833.1	-0.54342	1.66E-05	0.000167	sp|Q12999|TSN31_HUMAN Tetraspanin-31 OS=Homo sapiens GN=TSPAN31 PE=2 SV=1//5.43823e-121
XM_008003840.1	0.83213	7.45E-12	1.57E-10	sp|Q86YJ5|MARH9_HUMAN E3 ubiquitin-protein ligase MARCH9 OS=Homo sapiens GN=MARCH9 PE=1 SV=2//6.86594e-151
XM_008003841.1	1.318	3.63E-08	5.15E-07	sp|O15528|CP27B_HUMAN 25-hydroxyvitamin D-1 alpha hydroxylase, mitochondrial OS=Homo sapiens GN=CYP27B1 PE=1 SV=1//0
XM_008003843.1	0.7766	4.65E-07	5.84E-06	sp|Q96AZ1|MT21B_HUMAN Protein-lysine methyltransferase METTL21B OS=Homo sapiens GN=METTL21B PE=1 SV=1//1.07288e-154
XM_008003848.1	0.66403	0.000177	0.001508	sp|Q9UBP6|TRMB_HUMAN tRNA (guanine-N(7)-)-methyltransferase OS=Homo sapiens GN=METTL1 PE=1 SV=1//0
XM_008003858.1	-0.56066	6.90E-08	9.47E-07	sp|Q9Y6H3|ATP23_HUMAN Mitochondrial inner membrane protease ATP23 homolog OS=Homo sapiens GN=XRCC6BP1 PE=1 SV=3//3.73164e-175
XM_008003862.1	0.92038	4.05E-23	1.95E-21	sp|Q6UXM1|LRIG3_HUMAN Leucine-rich repeats and immunoglobulin-like domains protein 3 OS=Homo sapiens GN=LRIG3 PE=2 SV=1//0
XM_008003884.1	0.52585	0.008317	0.049024	sp|Q9Y2B1|TMEM5_HUMAN Transmembrane protein 5 OS=Homo sapiens GN=TMEM5 PE=1 SV=1//0
XM_008003890.1	2.0477	2.38E-34	1.93E-32	sp|Q96MD2|CL066_HUMAN UPF0536 protein C12orf66 OS=Homo sapiens GN=C12orf66 PE=2 SV=4//0
XM_008003891.1	1.2002	7.91E-33	5.95E-31	sp|Q7Z6B7|SRGP1_HUMAN SLIT-ROBO Rho GTPase-activating protein 1 OS=Homo sapiens GN=SRGAP1 PE=1 SV=1//0
XM_008003897.1	1.3001	0.000637	0.004898	sp|O43592|XPOT_HUMAN Exportin-T OS=Homo sapiens GN=XPOT PE=1 SV=2//0
XM_008003902.1	-1.0795	4.24E-49	5.95E-47	sp|P15586|GNS_HUMAN N-acetylglucosamine-6-sulfatase OS=Homo sapiens GN=GNS PE=1 SV=3//0
XM_008003910.1	0.87226	1.85E-19	7.02E-18	sp|Q9Y2U8|MAN1_HUMAN Inner nuclear membrane protein Man1 OS=Homo sapiens GN=LEMD3 PE=1 SV=2//0
XM_008003925.1	-0.65641	1.29E-10	2.44E-09	sp|Q9BRT6|LLPH_HUMAN Protein LLP homolog OS=Homo sapiens GN=LLPH PE=2 SV=1//1.53241e-51
XM_008003939.1	0.44964	1.05E-09	1.80E-08	sp|Q86VP6|CAND1_HUMAN Cullin-associated NEDD8-dissociated protein 1 OS=Homo sapiens GN=CAND1 PE=1 SV=2//0
XM_008003965.1	0.50082	7.06E-09	1.09E-07	sp|P57740|NU107_HUMAN Nuclear pore complex protein Nup107 OS=Homo sapiens GN=NUP107 PE=1 SV=1//0
XM_008003981.1	-0.79419	0.000573	0.004447	sp|P61634|LYSC_ERYPA Lysozyme C OS=Erythrocebus patas GN=LYZ PE=3 SV=1//2.6132e-102
XM_008003982.1	0.42707	0.001354	0.009701	sp|O95619|YETS4_HUMAN YEATS domain-containing protein 4 OS=Homo sapiens GN=YEATS4 PE=1 SV=1//1.83609e-142
XM_008004010.1	-0.5373	0.000114	0.000999	sp|Q86W47|KCMB4_HUMAN Calcium-activated potassium channel subunit beta-4 OS=Homo sapiens GN=KCNMB4 PE=1 SV=2//7.00667e-136
XM_008004033.1	-1.3017	2.30E-50	3.43E-48	sp|O60293|ZC3H1_HUMAN Zinc finger C3H1 domain-containing protein OS=Homo sapiens GN=ZFC3H1 PE=1 SV=3//0
XM_008004034.1	0.88254	4.88E-23	2.32E-21	sp|Q9UL25|RAB21_HUMAN Ras-related protein Rab-21 OS=Homo sapiens GN=RAB21 PE=1 SV=3//6.49102e-145
XM_008004057.1	0.69498	1.63E-12	3.64E-11	sp|Q13601|KRR1_HUMAN KRR1 small subunit processome component homolog OS=Homo sapiens GN=KRR1 PE=1 SV=4//0
XM_008004058.1	0.74403	0.000264	0.002179	sp|P48060|GLIP1_HUMAN Glioma pathogenesis-related protein 1 OS=Homo sapiens GN=GLIPR1 PE=1 SV=3//0
XM_008004060.1	0.7242	4.53E-21	1.89E-19	sp|Q8WV24|PHLA1_HUMAN Pleckstrin homology-like domain family A member 1 OS=Homo sapiens GN=PHLDA1 PE=1 SV=4//2.35503e-142
XM_008004073.1	0.5336	8.16E-05	0.000738	sp|Q5R8P3|BBS10_PONAB Bardet-Biedl syndrome 10 protein homolog OS=Pongo abelii GN=BBS10 PE=2 SV=1//0
XM_008004113.1	1.2761	1.66E-46	2.07E-44	sp|Q96IZ0|PAWR_HUMAN PRKC apoptosis WT1 regulator protein OS=Homo sapiens GN=PAWR PE=1 SV=1//2.62974e-158
XM_008004119.1	2.8872	1.69E-07	2.23E-06	sp|Q3ZCN5|OTOGL_HUMAN Otogelin-like protein OS=Homo sapiens GN=OTOGL PE=2 SV=5//0
XM_008004120.1	4.0354	0.003534	0.022909	sp|Q9UMZ3|PTPRQ_HUMAN Phosphatidylinositol phosphatase PTPRQ OS=Homo sapiens GN=PTPRQ PE=1 SV=2//1.98721e-14
XM_008004124.1	1.0992	0.000693	0.005296	sp|Q8JZS0|LIN7A_MOUSE Protein lin-7 homolog A OS=Mus musculus GN=Lin7a PE=1 SV=2//1.59913e-132
XM_008004150.1	-0.46038	1.97E-07	2.57E-06	sp|Q5RFN6|CNN2_PONAB Calponin-2 OS=Pongo abelii GN=CNN2 PE=2 SV=3//0
XM_008004155.1	-0.45655	1.84E-10	3.42E-09	sp|P05783|K1C18_HUMAN Keratin, type I cytoskeletal 18 OS=Homo sapiens GN=KRT18 PE=1 SV=2//0

XM_008004158.1	1.1742	4.91E-19	1.80E-17	sp|Q9H2J7|S6A15_HUMAN Sodium-dependent neutral amino acid transporter B(0)AT2 OS=Homo sapiens GN=SLC6A15 PE=1 SV=1//0
XM_008004213.1	0.28598	0.001095	0.008025	sp|Q16828|DUS6_HUMAN Dual specificity protein phosphatase 6 OS=Homo sapiens GN=DUSP6 PE=1 SV=2//0
XM_008004214.1	-0.60686	3.55E-05	0.000338	sp|Q8N4A0|GALT4_HUMAN Polypeptide N-acetylgalactosaminyltransferase 4 OS=Homo sapiens GN=GALNT4 PE=1 SV=2//0
XM_008004236.1	1.5702	1.02E-57	1.93E-55	sp|Q15075|EEA1_HUMAN Early endosome antigen 1 OS=Homo sapiens GN=EEA1 PE=1 SV=2//0
XM_008004276.1	-0.94121	1.67E-14	4.44E-13	sp|Q0MQ87|NDUAC_PANTR NADH dehydrogenase [ubiquinone] 1 alpha subcomplex subunit 12 OS=Pan troglodytes GN=NDUFA12 PE=2 SV=1//6.07074e-100
XM_008004289.1	0.41422	0.000466	0.003685	sp|Q6ZV73|FGD6_HUMAN FYVE, RhoGEF and PH domain-containing protein 6 OS=Homo sapiens GN=FGD6 PE=1 SV=2//0
XM_008004305.1	-0.65584	2.92E-05	0.000282	sp|P62321|RUXF_XENLA Small nuclear ribonucleoprotein F OS=Xenopus laevis GN=snrpf PE=3 SV=1//1.32077e-48
XM_008004312.1	0.38855	5.21E-07	6.50E-06	sp|P41970|ELK3_HUMAN ETS domain-containing protein Elk-3 OS=Homo sapiens GN=ELK3 PE=1 SV=2//0
XM_008004314.1	1.2747	4.27E-05	0.000403	sp|Q00537|CDK17_HUMAN Cyclin-dependent kinase 17 OS=Homo sapiens GN=CDK17 PE=1 SV=2//0
XM_008004336.1	0.74712	8.81E-13	2.01E-11	sp|P42166|LAP2A_HUMAN Lamina-associated polypeptide 2, isoform alpha OS=Homo sapiens GN=TMPO PE=1 SV=2//0
XM_008004337.1	-2.8989	0.004278	0.027218	sp|P42167|LAP2B_HUMAN Lamina-associated polypeptide 2, isoforms beta/gamma OS=Homo sapiens GN=TMPO PE=1 SV=2//0
XM_008004346.1	-0.51977	3.47E-05	0.000331	sp|Q5EAJ6|IKIP_RAT Inhibitor of nuclear factor kappa-B kinase-interacting protein OS=Rattus norvegicus GN=Ikbip PE=2 SV=1//5.24421e-170
XM_008004398.1	0.38942	0.00029	0.002382	sp|P40616|ARL1_HUMAN ADP-ribosylation factor-like protein 1 OS=Homo sapiens GN=ARL1 PE=1 SV=1//1.48552e-117
XM_008004453.1	-0.40991	1.84E-07	2.41E-06	sp|Q4R520|ENPL_MACFA Endoplasmin OS=Macaca fascicularis GN=HSP90B1 PE=2 SV=1//0
XM_008004472.1	0.72709	0.004407	0.027954	sp|P25208|NFYB_HUMAN Nuclear transcription factor Y subunit beta OS=Homo sapiens GN=NFYB PE=1 SV=2//1.0441e-140
XM_008004493.1	0.93242	3.85E-06	4.26E-05	sp|Q4R335|S41A2_MACFA Solute carrier family 41 member 2 OS=Macaca fascicularis GN=SLC41A2 PE=2 SV=1//0
XM_008004513.1	-0.745	0.000881	0.00657	sp|Q8TAD7|OCC1_HUMAN Overexpressed in colon carcinoma 1 protein OS=Homo sapiens GN=OCC1 PE=1 SV=2//3.43488e-19
XM_008004516.1	0.62135	1.02E-06	1.23E-05	sp|O60285|NUAK1_HUMAN NUAK family SNF1-like kinase 1 OS=Homo sapiens GN=NUAK1 PE=1 SV=1//0
XM_008004518.1	-0.74589	6.05E-08	8.34E-07	sp|Q07065|CKAP4_HUMAN Cytoskeleton-associated protein 4 OS=Homo sapiens GN=CKAP4 PE=1 SV=2//3.11472e-11
XM_008004532.1	1.1856	1.62E-14	4.30E-13	sp|Q9NW08|RPC2_HUMAN DNA-directed RNA polymerase III subunit RPC2 OS=Homo sapiens GN=POLR3B PE=1 SV=2//0
XM_008004535.1	-1.582	0.004393	0.027882	sp|Q9NVN3|RIC8B_HUMAN Synembryn-B OS=Homo sapiens GN=RIC8B PE=1 SV=2//0
XM_008004538.1	1.0089	5.67E-06	6.13E-05	sp|Q9NVN3|RIC8B_HUMAN Synembryn-B OS=Homo sapiens GN=RIC8B PE=1 SV=2//0
XM_008004554.1	1.5844	5.86E-14	1.48E-12	sp|Q16526|CRY1_HUMAN Cryptochrome-1 OS=Homo sapiens GN=CRY1 PE=1 SV=1//0
XM_008004564.1	1.2696	0.002733	0.018147	sp|Q9UKN5|PRDM4_HUMAN PR domain zinc finger protein 4 OS=Homo sapiens GN=PRDM4 PE=1 SV=3//0
XM_008004577.1	1.0727	0.002302	0.015624	sp|Q9BVA6|FICD_HUMAN Adenosine monophosphate-protein transferase FICD OS=Homo sapiens GN=FICD PE=1 SV=2//0
XM_008004593.1	0.52794	7.65E-07	9.38E-06	sp|Q8WYL5|SSH1_HUMAN Protein phosphatase Slingshot homolog 1 OS=Homo sapiens GN=SSH1 PE=1 SV=2//0
XM_008004628.1	0.65591	7.70E-05	0.000701	sp|Q9H3F6|BACD3_HUMAN BTB/POZ domain-containing adapter for CUL3-mediated RhoA degradation protein 3 OS=Homo sapiens GN=KCTD10 PE=1 SV=1//0
XM_008004652.1	1.4669	8.53E-47	1.09E-44	sp|Q8N2M4|TM86A_HUMAN Lysoplasmalogenase-like protein TMEM86A OS=Homo sapiens GN=TMEM86A PE=2 SV=1//3.87182e-125
XM_008004679.1	0.98603	5.15E-24	2.60E-22	sp|Q68D10|SPT2_HUMAN Protein SPT2 homolog OS=Homo sapiens GN=SPTY2D1 PE=1 SV=3//0
XM_008004681.1	0.51829	4.27E-05	0.000403	sp|P16615|AT2A2_HUMAN Sarcoplasmic/endoplasmic reticulum calcium ATPase 2 OS=Homo sapiens GN=ATP2A2 PE=1 SV=1//0
XM_008004701.1	0.91744	4.21E-16	1.25E-14	sp|Q8NI37|PPTC7_HUMAN Protein phosphatase PTC7 homolog OS=Homo sapiens GN=PPTC7 PE=2 SV=1//0
XM_008004714.1	0.39729	9.09E-05	0.000814	sp|Q99816|TS101_HUMAN Tumor susceptibility gene 101 protein OS=Homo sapiens GN=TSG101 PE=1 SV=2//0
XM_008004794.1	Inf	0.001749	0.012211	sp|P00973|OAS1_HUMAN 2'-5'-oligoadenylate synthase 1 OS=Homo sapiens GN=OAS1 PE=1 SV=4//0
XM_008004796.1	Inf	1.72E-12	3.84E-11	sp|P29728|OAS2_HUMAN 2'-5'-oligoadenylate synthase 2 OS=Homo sapiens GN=OAS2 PE=1 SV=3//0
XM_008004832.1	0.55176	1.77E-07	2.32E-06	sp|Q9Y4C8|RBM19_HUMAN Probable RNA-binding protein 19 OS=Homo sapiens GN=RBM19 PE=1 SV=3//0
XM_008004836.1	-1.1309	3.39E-11	6.76E-10	sp|Q9BE24|LDHA_MACFA L-lactate dehydrogenase A chain OS=Macaca fascicularis GN=LDHA PE=2 SV=4//1.26239e-154
XM_008004872.1	0.61249	1.15E-08	1.75E-07	sp|P40937|RFC5_HUMAN Replication factor C subunit 5 OS=Homo sapiens

				GN=RFC5 PE=1 SV=1//0
XM_008004894.1	1.5624	1.83E-58	3.61E-56	sp|Q5RAB0|HSPB8_PONAB Heat shock protein beta-8 OS=Pongo abelii GN=HSPB8 PE=2 SV=1//2.78996e-121
XM_008004904.1	1.2362	2.59E-16	7.82E-15	sp|Q9Y478|AAKB1_HUMAN 5'-AMP-activated protein kinase subunit beta-1 OS=Homo sapiens GN=PRKAB1 PE=1 SV=4//0
XM_008004912.1	2.5529	1.60E-09	2.66E-08	sp|P32780|TF2H1_HUMAN General transcription factor IIH subunit 1 OS=Homo sapiens GN=GTF2H1 PE=1 SV=1//0
XM_008004919.1	0.43941	0.001292	0.009286	sp|Q5U316|RAB35_RAT Ras-related protein Rab-35 OS=Rattus norvegicus GN=Rab35 PE=1 SV=1//6.4557e-117
XM_008004921.1	0.2177	0.004495	0.028482	sp|Q92616|GCN1L_HUMAN Translational activator GCN1 OS=Homo sapiens GN=GCN1L1 PE=1 SV=6//0
XM_008004922.1	-0.50591	2.02E-13	4.91E-12	sp|P05388|RLA0_HUMAN 60S acidic ribosomal protein P0 OS=Homo sapiens GN=RPLP0 PE=1 SV=1//0
XM_008004940.1	-0.58834	9.15E-11	1.75E-09	sp|P12074|CX6A1_HUMAN Cytochrome c oxidase subunit 6A1, mitochondrial OS=Homo sapiens GN=COX6A1 PE=1 SV=4//1.39762e-68
XM_008004941.1	0.54786	1.21E-06	1.44E-05	sp|O43716|GATC_HUMAN Glutamyl-tRNA(Gln) amidotransferase subunit C, mitochondrial OS=Homo sapiens GN=GATC PE=1 SV=1//1.25203e-90
XM_008004943.1	-0.38303	3.02E-05	0.000291	sp|Q13242|SRSF9_HUMAN Serine/arginine-rich splicing factor 9 OS=Homo sapiens GN=SRSF9 PE=1 SV=1//4.59869e-117
XM_008004965.1	-0.37485	2.18E-07	2.84E-06	sp|Q14165|MLEC_HUMAN Malectin OS=Homo sapiens GN=MLEC PE=1 SV=1//2.78721e-156
XM_008004966.1	-0.38666	1.05E-05	0.000108	sp|A6NIH7|U119B_HUMAN Protein unc-119 homolog B OS=Homo sapiens GN=UNC119B PE=1 SV=1//2.76486e-134
XM_008004971.1	0.7756	2.95E-06	3.33E-05	sp|Q96C57|CL043_HUMAN Uncharacterized protein C12orf43 OS=Homo sapiens GN=C12orf43 PE=1 SV=2//3.04839e-87
XM_008004973.1	Inf	2.46E-10	4.49E-09	sp|Q15646|OASL_HUMAN 2'-5'-oligoadenylate synthase-like protein OS=Homo sapiens GN=OASL PE=1 SV=2//0
XM_008005009.1	-0.27865	0.007209	0.043186	sp|Q9UPS6|SET1B_HUMAN Histone-lysine N-methyltransferase SETD1B OS=Homo sapiens GN=SETD1B PE=1 SV=3//0
XM_008005018.1	0.47813	1.22E-05	0.000125	sp|Q9HAP2|MLXIP_HUMAN MLX-interacting protein OS=Homo sapiens GN=MLXIP PE=1 SV=2//0
XM_008005027.1	-1.5879	6.97E-05	0.000638	sp|Q9C0J1|B3GN4_HUMAN N-acetyllactosaminide beta-1,3-N-acetylglucosaminyltransferase 4 OS=Homo sapiens GN=B3GNT4 PE=1 SV=1//0
XM_008005035.1	2.1919	0.000336	0.002732	sp|P30622|CLIP1_HUMAN CAP-Gly domain-containing linker protein 1 OS=Homo sapiens GN=CLIP1 PE=1 SV=2//5.49756e-161
XM_008005080.1	-0.63933	5.07E-11	9.96E-10	sp|Q66PJ3|AR6P4_HUMAN ADP-ribosylation factor-like protein 6-interacting protein 4 OS=Homo sapiens GN=ARL6IP4 PE=1 SV=2//1.99372e-62
XM_008005115.1	0.58544	2.13E-08	3.11E-07	sp|Q9NQR1|SETD8_HUMAN N-lysine methyltransferase SETD8 OS=Homo sapiens GN=SETD8 PE=1 SV=3//0
XM_008005125.1	0.5746	5.69E-06	6.14E-05	sp|Q5EBL4|RIPL1_HUMAN RILP-like protein 1 OS=Homo sapiens GN=RILPL1 PE=1 SV=1//0
XM_008005126.1	-0.32555	3.95E-05	0.000375	sp|Q9R0Q3|TMED2_MOUSE Transmembrane emp24 domain-containing protein 2 OS=Mus musculus GN=Tmed2 PE=1 SV=1//4.74527e-128
XM_008005137.1	0.60394	6.55E-06	6.97E-05	sp|Q9Y487|VPP2_HUMAN V-type proton ATPase 116 kDa subunit a isoform 2 OS=Homo sapiens GN=ATP6V0A2 PE=1 SV=2//0
XM_008005187.1	-0.54838	0.000339	0.002753	sp|Q8WY22|BRI3B_HUMAN BRI3-binding protein OS=Homo sapiens GN=BRI3BP PE=1 SV=1//1.12662e-105
XM_008005285.1	1.3819	0.001424	0.010169	sp|Q9GM01|GALT9_MACFA Polypeptide N-acetylgalactosaminyltransferase 9 OS=Macaca fascicularis GN=GALNT9 PE=2 SV=1//7.98537e-174
XM_008005296.1	-0.84331	0.001942	0.013363	-//-
XM_008005315.1	0.81243	3.65E-07	4.61E-06	sp|Q86XL3|ANKL2_HUMAN Ankyrin repeat and LEM domain-containing protein 2 OS=Homo sapiens GN=ANKLE2 PE=1 SV=4//0
XM_008005365.1	0.84723	0.002321	0.015715	-//-
XM_008005369.1	-1.8546	0.001484	0.010549	-//-
XM_008005376.1	-0.55259	3.83E-15	1.06E-13	sp|P05783|K1C18_HUMAN Keratin, type I cytoskeletal 18 OS=Homo sapiens GN=KRT18 PE=1 SV=2//0
XM_008005379.1	0.3509	9.41E-06	9.77E-05	sp|Q9UQ80|PA2G4_HUMAN Proliferation-associated protein 2G4 OS=Homo sapiens GN=PA2G4 PE=1 SV=3//4.69839e-22
XM_008005381.1	0.61257	3.38E-11	6.74E-10	sp|P56192|SYMC_HUMAN Methionine--tRNA ligase, cytoplasmic OS=Homo sapiens GN=MARS PE=1 SV=2//0
XM_008005395.1	0.36529	0.000124	0.001078	sp|Q9UJX3|APC7_HUMAN Anaphase-promoting complex subunit 7 OS=Homo sapiens GN=ANAPC7 PE=1 SV=4//0
XM_008005396.1	-0.79247	7.10E-06	7.51E-05	sp|O15145|ARPC3_HUMAN Actin-related protein 2/3 complex subunit 3 OS=Homo sapiens GN=ARPC3 PE=1 SV=3//1.54856e-85
XM_008005398.1	0.89061	3.81E-17	1.24E-15	sp|Q7Z569|BRAP_HUMAN BRCA1-associated protein OS=Homo sapiens GN=BRAP PE=1 SV=2//0
XM_008005399.1	-0.85735	4.01E-19	1.48E-17	sp|Q02878|RL6_HUMAN 60S ribosomal protein L6 OS=Homo sapiens GN=RPL6 PE=1 SV=3//1.01186e-22
XM_008005404.1	1.2372	3.93E-29	2.54E-27	sp|Q6NZY4|ZCHC8_HUMAN Zinc finger CCHC domain-containing protein 8 OS=Homo sapiens GN=ZCCHC8 PE=1 SV=2//0

XM_008005405.1	0.73799	2.26E-18	7.93E-17	sp|Q689Z5|SBNO1_MOUSE Protein strawberry notch homolog 1 OS=Mus musculus GN=Sbno1 PE=1 SV=2//0
XM_008005435.1	1.2299	4.01E-05	0.00038	sp|Q8N5I2|ARRD1_HUMAN Arrestin domain-containing protein 1 OS=Homo sapiens GN=ARRDC1 PE=1 SV=1//0
XM_008005437.1	1.191	2.64E-12	5.79E-11	sp|Q9BTV6|DPH7_HUMAN Diphthine methyltransferase OS=Homo sapiens GN=DPH7 PE=1 SV=2//0
XM_008005440.1	-0.93846	2.14E-11	4.32E-10	sp|Q8IXM3|RM41_HUMAN 39S ribosomal protein L41, mitochondrial OS=Homo sapiens GN=MRPL41 PE=1 SV=1//3.14797e-78
XM_008005475.1	0.52725	2.86E-05	0.000277	sp|Q5RFR2|NR3L1_PONAB Natural cytotoxicity triggering receptor 3 ligand 1 OS=Pongo abelii GN=NCR3LG1 PE=2 SV=1//0
XM_008005482.1	1.8353	9.79E-13	2.22E-11	sp|Q91ZA8|NRARP_MOUSE Notch-regulated ankyrin repeat-containing protein OS=Mus musculus GN=Nrarp PE=2 SV=1//7.87837e-72
XM_008005483.1	-0.30056	0.004091	0.026204	sp|Q8WX92|NELFB_HUMAN Negative elongation factor B OS=Homo sapiens GN=NELFB PE=1 SV=1//0
XM_008005489.1	-0.7376	5.41E-17	1.74E-15	sp|P11833|TBB_PARLI Tubulin beta chain OS=Paracentrotus lividus PE=2 SV=1//0
XM_008005520.1	-0.41691	0.003911	0.025146	sp|O43805|SSNA1_HUMAN Sjoegren syndrome nuclear autoantigen 1 OS=Homo sapiens GN=SSNA1 PE=1 SV=2//1.64927e-75
XM_008005523.1	-1.4363	0.007502	0.044644	sp|Q9UHL4|DPP2_HUMAN Dipeptidyl peptidase 2 OS=Homo sapiens GN=DPP7 PE=1 SV=3//0
XM_008005525.1	-0.41915	3.90E-06	4.31E-05	sp|Q9UKM7|MA1B1_HUMAN Endoplasmic reticulum mannosyl-oligosaccharide 1,2-alpha-mannosidase OS=Homo sapiens GN=MAN1B1 PE=1 SV=2//0
XM_008005549.1	-1.5674	2.59E-07	3.34E-06	sp|Q8WNM0|PTGDS_PONPY Prostaglandin-H2 D-isomerase OS=Pongo pygmaeus GN=PTGDS PE=2 SV=1//1.26616e-125
XM_008005561.1	-0.64485	2.25E-16	6.84E-15	sp|P62278|RS13_RAT 40S ribosomal protein S13 OS=Rattus norvegicus GN=Rps13 PE=1 SV=2//7.03803e-105
XM_008005567.1	-0.79385	1.88E-11	3.82E-10	sp|O60869|EDF1_HUMAN Endothelial differentiation-related factor 1 OS=Homo sapiens GN=EDF1 PE=1 SV=1//2.17049e-99
XM_008005573.1	-0.61151	8.65E-05	0.000779	sp|Q5T5S1|CC183_HUMAN Coiled-coil domain-containing protein 183 OS=Homo sapiens GN=CCDC183 PE=2 SV=3//0
XM_008005592.1	-1.8035	0.001952	0.013427	sp|Q5VUD6|FA69B_HUMAN Protein FAM69B OS=Homo sapiens GN=FAM69B PE=1 SV=3//0
XM_008005605.1	-0.67679	6.33E-11	1.23E-09	sp|O15120|PLCB_HUMAN 1-acyl-sn-glycerol-3-phosphate acyltransferase beta OS=Homo sapiens GN=AGPAT2 PE=1 SV=1//5.92389e-168
XM_008005662.1	-0.28135	0.002985	0.019678	sp|Q9BSL1|UBAC1_HUMAN Ubiquitin-associated domain-containing protein 1 OS=Homo sapiens GN=UBAC1 PE=1 SV=1//0
XM_008005742.1	-2.2301	5.93E-05	0.000548	sp|Q15059|BRD3_HUMAN Bromodomain-containing protein 3 OS=Homo sapiens GN=BRD3 PE=1 SV=1//0
XM_008005785.1	0.99788	9.37E-09	1.43E-07	sp|Q15527|SURF2_HUMAN Surfeit locus protein 2 OS=Homo sapiens GN=SURF2 PE=1 SV=3//1.55053e-153
XM_008005801.1	#NAME?	0.001632	0.011471	sp|P16442|BGAT_HUMAN Histo-blood group ABO system transferase OS=Homo sapiens GN=ABO PE=1 SV=2//4.58859e-162
XM_008005861.1	1.4001	1.41E-29	9.28E-28	sp|Q9H8H2|DDX31_HUMAN Probable ATP-dependent RNA helicase DDX31 OS=Homo sapiens GN=DDX31 PE=2 SV=2//0
XM_008005862.1	0.9065	2.33E-21	9.92E-20	sp|Q9UKN8|TF3C4_HUMAN General transcription factor 3C polypeptide 4 OS=Homo sapiens GN=GTF3C4 PE=1 SV=2//0
XM_008005863.1	0.99686	1.04E-17	3.52E-16	sp|Q15361|TTF1_HUMAN Transcription termination factor 1 OS=Homo sapiens GN=TTF1 PE=1 SV=3//0
XM_008005905.1	-1.8251	4.36E-05	0.00041	sp|Q5JUQ0|FA78A_HUMAN Protein FAM78A OS=Homo sapiens GN=FAM78A PE=2 SV=1//0
XM_008005919.1	0.85846	2.62E-06	2.98E-05	sp|Q13868|EXOS2_HUMAN Exosome complex component RRP4 OS=Homo sapiens GN=EXOSC2 PE=1 SV=2//0
XM_008005961.1	-1.1576	0.007175	0.04303	sp|Q6PWL6|PTGES_MACFA Prostaglandin E synthase OS=Macaca fascicularis GN=PTGES PE=2 SV=1//4.37771e-102
XM_008005968.1	1.4712	3.14E-08	4.48E-07	sp|Q5R4M7|ASB6_PONAB Ankyrin repeat and SOCS box protein 6 OS=Pongo abelii GN=ASB6 PE=2 SV=1//0
XM_008005978.1	0.64242	0.000697	0.005321	sp|Q5PQP0|IER5L_RAT Immediate early response gene 5-like protein OS=Rattus norvegicus GN=Ier5l PE=2 SV=1//1.71544e-42
XM_008006018.1	-0.77196	0.000349	0.002823	sp|Q14249|NUCG_HUMAN Endonuclease G, mitochondrial OS=Homo sapiens GN=ENDOG PE=1 SV=4//2.68741e-169
XM_008006033.1	-0.67128	2.68E-05	0.000261	sp|Q96GR4|ZDH12_HUMAN Probable palmitoyltransferase ZDHHC12 OS=Homo sapiens GN=ZDHHC12 PE=2 SV=2//3.20171e-96
XM_008006042.1	0.62162	1.59E-09	2.65E-08	sp|Q5RAS2|GLE1_PONAB Nucleoporin GLE1 OS=Pongo abelii GN=GLE1 PE=2 SV=1//0
XM_008006069.1	-0.43631	0.001126	0.008219	sp|Q1ZZU3|SWI5_HUMAN DNA repair protein SWI5 homolog OS=Homo sapiens GN=SWI5 PE=1 SV=1//6.4033e-131
XM_008006092.1	-0.75598	9.79E-05	0.000872	sp|Q9BUW7|CI016_HUMAN UPF0184 protein C9orf16 OS=Homo sapiens GN=C9orf16 PE=1 SV=1//1.66668e-32
XM_008006104.1	Inf	0.006924	0.041711	sp|Q6KCM7|SCMC2_HUMAN Calcium-binding mitochondrial carrier protein SCaMC-2 OS=Homo sapiens GN=SLC25A25 PE=1 SV=1//0
XM_008006105.1	0.69208	1.09E-09	1.85E-08	sp|Q5T9C2|F102A_HUMAN Protein FAM102A OS=Homo sapiens GN=FAM102A PE=1 SV=2//0

XM_008006130.1	-0.7536	1.81E-11	3.68E-10	sp|P17813|EGLN_HUMAN Endoglin OS=Homo sapiens GN=ENG PE=1 SV=2//0
XM_008006135.1	0.45305	4.90E-05	0.000458	sp|P50750|CDK9_HUMAN Cyclin-dependent kinase 9 OS=Homo sapiens GN=CDK9 PE=1 SV=3//0
XM_008006155.1	-0.99154	8.13E-06	8.52E-05	sp|Q5JU69|TOR2A_HUMAN Torsin-2A OS=Homo sapiens GN=TOR2A PE=2 SV=1//0
XM_008006170.1	1.2504	5.53E-44	6.29E-42	sp|P62071|RRAS2_MOUSE Ras-related protein R-Ras2 OS=Mus musculus GN=Rras2 PE=1 SV=1//2.619e-133
XM_008006256.1	0.76601	1.23E-26	7.02E-25	sp|P11021|GRP78_HUMAN 78 kDa glucose-regulated protein OS=Homo sapiens GN=HSPA5 PE=1 SV=2//0
XM_008006269.1	-0.48173	1.91E-11	3.88E-10	sp|Q3MHM7|RL35_BOVIN 60S ribosomal protein L35 OS=Bos taurus GN=RPL35 PE=2 SV=3//3.06523e-58
XM_008006270.1	0.77487	4.25E-09	6.79E-08	sp|Q5R4M1|ARP5L_PONAB Actin-related protein 2/3 complex subunit 5-like protein OS=Pongo abelii GN=ARPC5L PE=2 SV=1//3.62992e-92
XM_008006272.1	-1.8899	1.04E-15	3.02E-14	sp|Q68BL7|OLM2A_HUMAN Olfactomedin-like protein 2A OS=Homo sapiens GN=OLFML2A PE=2 SV=1//0
XM_008006305.1	0.36397	0.003589	0.023218	sp|P50458|LHX2_HUMAN LIM/homeobox protein Lhx2 OS=Homo sapiens GN=LHX2 PE=2 SV=2//0
XM_008006340.1	0.80835	8.16E-07	9.96E-06	sp|Q15916|ZBTB6_HUMAN Zinc finger and BTB domain-containing protein 6 OS=Homo sapiens GN=ZBTB6 PE=1 SV=1//0
XM_008006365.1	-0.463	0.002749	0.018243	sp|Q6ZV29|PLPL7_HUMAN Patatin-like phospholipase domain-containing protein 7 OS=Homo sapiens GN=PNPLA7 PE=1 SV=3//1.81501e-111
XM_008006375.1	0.88852	8.83E-13	2.02E-11	sp|Q7L4E1|FA73B_HUMAN Protein FAM73B OS=Homo sapiens GN=FAM73B PE=1 SV=1//0
XM_008006417.1	0.99751	1.84E-37	1.67E-35	sp|Q8WWQ0|PHIP_HUMAN PH-interacting protein OS=Homo sapiens GN=PHIP PE=1 SV=2//0
XM_008006423.1	0.64703	0.006642	0.040286	sp|Q86VQ0|LCA5_HUMAN Lebercilin OS=Homo sapiens GN=LCA5 PE=1 SV=2//0
XM_008006430.1	0.76416	0.000241	0.002006	sp|Q3S8M4|ELOV4_MACMU Elongation of very long chain fatty acids protein 4 OS=Macaca mulatta GN=ELOVL4 PE=3 SV=1//0
XM_008006439.1	1.5766	9.00E-15	2.44E-13	sp|A6NK89|RASFA_HUMAN Ras association domain-containing protein 10 OS=Homo sapiens GN=RASSF10 PE=2 SV=3//0
XM_008006440.1	0.45488	3.72E-07	4.70E-06	sp|Q9P2D0|IBTK_HUMAN Inhibitor of Bruton tyrosine kinase OS=Homo sapiens GN=IBTK PE=1 SV=3//0
XM_008006476.1	-1.1921	3.04E-13	7.32E-12	sp|Q1WK24|PRS35_MACMU Inactive serine protease 35 OS=Macaca mulatta GN=PRSS35 PE=2 SV=1//0
XM_008006506.1	0.46652	0.001599	0.011282	sp|Q7L1T6|NB5R4_HUMAN Cytochrome b5 reductase 4 OS=Homo sapiens GN=CYB5R4 PE=1 SV=1//0
XM_008006553.1	1.6834	3.43E-33	2.62E-31	sp|Q53H80|AKIR2_HUMAN Akirin-2 OS=Homo sapiens GN=AKIRIN2 PE=1 SV=2//8.04837e-61
XM_008006566.1	1.5783	0.001247	0.009001	sp|Q5IS73|CNR1_PANTR Cannabinoid receptor 1 OS=Pan troglodytes GN=CNR1 PE=2 SV=1//0
XM_008006577.1	#NAME?	0.003845	0.024766	sp|Q8N8Z3|PRR26_HUMAN Proline-rich protein 26 OS=Homo sapiens GN=PRR26 PE=2 SV=1//2.53342e-27
XM_008006583.1	0.70475	3.38E-13	8.07E-12	sp|Q12796|PNRC1_HUMAN Proline-rich nuclear receptor coactivator 1 OS=Homo sapiens GN=PNRC1 PE=1 SV=1//4.44216e-175
XM_008006659.1	1.2662	3.72E-07	4.70E-06	sp|Q9BZM6|N2DL1_HUMAN NKG2D ligand 1 OS=Homo sapiens GN=ULBP1 PE=1 SV=1//1.27495e-124
XM_008006685.1	0.95031	3.50E-12	7.59E-11	sp|Q4R407|KTNA1_MACFA Katanin p60 ATPase-containing subunit A1 OS=Macaca fascicularis GN=KATNA1 PE=2 SV=1//0
XM_008006686.1	-0.81402	7.48E-12	1.57E-10	sp|Q5RBQ2|GINM1_PONAB Glycoprotein integral membrane protein 1 OS=Pongo abelii GN=GINM1 PE=2 SV=1//0
XM_008006687.1	0.84746	0.006833	0.041254	sp|Q8WUA2|PPIL4_HUMAN Peptidyl-prolyl cis-trans isomerase-like 4 OS=Homo sapiens GN=PPIL4 PE=1 SV=1//0
XM_008006702.1	-0.65385	8.74E-19	3.16E-17	sp|P62425|RL7A_RAT 60S ribosomal protein L7a OS=Rattus norvegicus GN=Rpl7a PE=1 SV=2//1.63463e-157
XM_008006729.1	0.66414	0.000257	0.002127	sp|Q13637|RAB32_HUMAN Ras-related protein Rab-32 OS=Homo sapiens GN=RAB32 PE=1 SV=3//1.05313e-146
XM_008006750.1	1.1858	6.77E-41	6.98E-39	sp|Q9NU22|MDN1_HUMAN Midasin OS=Homo sapiens GN=MDN1 PE=1 SV=2//2.39613e-10
XM_008006752.1	0.37824	4.31E-08	6.06E-07	sp|Q5R7J9|IF4G2_PONAB Eukaryotic translation initiation factor 4 gamma 2 OS=Pongo abelii GN=EIF4G2 PE=2 SV=2//0
XM_008006764.1	0.27703	0.002436	0.016381	sp|Q6PD62|CTR9_HUMAN RNA polymerase-associated protein CTR9 homolog OS=Homo sapiens GN=CTR9 PE=1 SV=1//0
XM_008006796.1	0.94862	3.40E-12	7.39E-11	sp|Q5R8B2|LTV1_PONAB Protein LTV1 homolog OS=Pongo abelii GN=LTV1 PE=2 SV=1//0
XM_008006807.1	-0.73009	2.01E-10	3.71E-09	sp|Q9BTY2|FUCO2_HUMAN Plasma alpha-L-fucosidase OS=Homo sapiens GN=FUCA2 PE=1 SV=2//0
XM_008006844.1	2.1252	1.81E-68	4.50E-66	sp|Q99967|CITE2_HUMAN Cbp/p300-interacting transactivator 2 OS=Homo sapiens GN=CITED2 PE=1 SV=2//2.91823e-127
XM_008006872.1	-0.60465	0.006456	0.039331	sp|Q9Y5Z4|HEBP2_HUMAN Heme-binding protein 2 OS=Homo sapiens GN=HEBP2 PE=1 SV=1//1.73434e-122
XM_008006875.1	-0.55599	0.000196	0.001656	sp|Q5TH69|BIG3_HUMAN Brefeldin A-inhibited guanine nucleotide-exchange

				protein 3 OS=Homo sapiens GN=ARFGEF3 PE=1 SV=3//0
XM_008006962.1	0.4236	0.00036	0.00291	sp|Q9Y450|HBS1L_HUMAN HBS1-like protein OS=Homo sapiens GN=HBS1L PE=1 SV=1//0
XM_008006963.1	1.4964	0.000991	0.007328	sp|Q5R6Y0|HBS1L_PONAB HBS1-like protein OS=Pongo abelii GN=HBS1L PE=2 SV=1//8.84218e-87
XM_008006998.1	-0.58865	9.84E-10	1.68E-08	sp|P46405|RS12_PIG 40S ribosomal protein S12 OS=Sus scrofa GN=RPS12 PE=2 SV=2//5.74885e-90
XM_008007024.1	2.9449	1.58E-45	1.88E-43	sp|P29279|CTGF_HUMAN Connective tissue growth factor OS=Homo sapiens GN=CTGF PE=1 SV=2//0
XM_008007025.1	-0.75483	1.04E-05	0.000107	sp|P22413|ENPP1_HUMAN Ectonucleotide pyrophosphatase/phosphodiesterase family member 1 OS=Homo sapiens GN=ENPP1 PE=1 SV=2//0
XM_008007140.1	1.3209	4.91E-16	1.45E-14	sp|Q9BXY4|RSPO3_HUMAN R-spondin-3 OS=Homo sapiens GN=RSPO3 PE=1 SV=1//1.28432e-138
XM_008007146.1	1.0784	9.14E-06	9.51E-05	sp|Q7Z4G4|TRM11_HUMAN tRNA (guanine(10)-N2)-methyltransferase homolog OS=Homo sapiens GN=TRMT11 PE=1 SV=1//0
XM_008007156.1	-3.1365	0.008117	0.047932	sp|Q8NI08|NCOA7_HUMAN Nuclear receptor coactivator 7 OS=Homo sapiens GN=NCOA7 PE=1 SV=2//2.24041e-110
XM_008007159.1	-0.56994	3.20E-10	5.77E-09	sp|Q7Z4H3|HDDC2_HUMAN HD domain-containing protein 2 OS=Homo sapiens GN=HDDC2 PE=1 SV=1//4.02225e-112
XM_008007177.1	0.58919	4.05E-09	6.49E-08	sp|Q9UH65|SWP70_HUMAN Switch-associated protein 70 OS=Homo sapiens GN=SWAP70 PE=1 SV=1//0
XM_008007194.1	0.27259	0.005084	0.031788	sp|Q9NRX5|SERC1_HUMAN Serine incorporator 1 OS=Homo sapiens GN=SERINC1 PE=1 SV=1//0
XM_008007199.1	0.33923	1.36E-05	0.000138	sp|Q6TYA9|CXA1_CHLAE Gap junction alpha-1 protein OS=Chlorocebus aethiops GN=GJA1 PE=1 SV=1//0
XM_008007231.1	0.67237	1.32E-05	0.000134	sp|Q8N8Z6|DCBD1_HUMAN Discoidin, CUB and LCCL domain-containing protein 1 OS=Homo sapiens GN=DCBLD1 PE=1 SV=2//0
XM_008007293.1	0.49241	1.16E-09	1.96E-08	sp|Q6IQ26|DEN5A_HUMAN DENN domain-containing protein 5A OS=Homo sapiens GN=DENND5A PE=1 SV=2//0
XM_008007313.1	0.93206	5.17E-26	2.85E-24	sp|P17707|DCAM_HUMAN S-adenosylmethionine decarboxylase proenzyme OS=Homo sapiens GN=AMD1 PE=1 SV=2//0
XM_008007414.1	0.34815	0.002824	0.018682	sp|Q86WC4|OSTM1_HUMAN Osteopetrosis-associated transmembrane protein 1 OS=Homo sapiens GN=OSTM1 PE=1 SV=1//1.18267e-163
XM_008007452.1	-0.5095	0.008025	0.047466	sp|Q8WWV3|RT4I1_HUMAN Reticulon-4-interacting protein 1, mitochondrial OS=Homo sapiens GN=RTN4IP1 PE=1 SV=2//0
XM_008007454.1	0.57963	1.61E-05	0.000161	sp|Q4R7R9|GATA_MACFA Glutamyl-tRNA(Gln) amidotransferase subunit A, mitochondrial OS=Macaca fascicularis GN=QRSL1 PE=2 SV=1//0
XM_008007492.1	0.96804	2.19E-05	0.000216	sp|Q8N3C0|ASCC3_HUMAN Activating signal cointegrator 1 complex subunit 3 OS=Homo sapiens GN=ASCC3 PE=1 SV=3//1.32518e-37
XM_008007518.1	0.42094	0.001713	0.011984	sp|P24863|CCNC_HUMAN Cyclin-C OS=Homo sapiens GN=CCNC PE=1 SV=2//0
XM_008007526.1	0.90231	1.48E-05	0.000149	sp|Q9UKA2|FBXL4_HUMAN F-box/LRR-repeat protein 4 OS=Homo sapiens GN=FBXL4 PE=1 SV=2//0
XM_008007540.1	1.2218	2.64E-11	5.32E-10	sp|Q9P032|NDUF4_HUMAN NADH dehydrogenase [ubiquinone] 1 alpha subcomplex assembly factor 4 OS=Homo sapiens GN=NDUFAF4 PE=1 SV=1//6.47074e-111
XM_008007541.1	1.0465	1.24E-06	1.48E-05	sp|Q9BZJ6|GPR63_HUMAN Probable G-protein coupled receptor 63 OS=Homo sapiens GN=GPR63 PE=2 SV=2//0
XM_008007556.1	-2.2585	2.91E-08	4.18E-07	sp|Q6PHW0|IYD1_HUMAN Iodotyrosine dehalogenase 1 OS=Homo sapiens GN=IYD PE=1 SV=2//3.70107e-168
XM_008007579.1	0.53823	0.001051	0.007726	sp|Q4R526|ARMT1_MACFA Protein-glutamate O-methyltransferase OS=Macaca fascicularis GN=ARMT1 PE=2 SV=2//0
XM_008007625.1	-0.43129	0.000704	0.005368	sp|Q4R723|RL27A_MACFA 60S ribosomal protein L27a OS=Macaca fascicularis GN=RPL27A PE=2 SV=1//3.16593e-87
XM_008007652.1	0.81718	1.48E-08	2.20E-07	sp|Q6P9H4|CNKR3_HUMAN Connector enhancer of kinase suppressor of ras 3 OS=Homo sapiens GN=CNKSR3 PE=1 SV=1//0
XM_008007663.1	2.9209	7.57E-35	6.30E-33	sp|Q8IVF5|TIAM2_HUMAN T-lymphoma invasion and metastasis-inducing protein 2 OS=Homo sapiens GN=TIAM2 PE=2 SV=4//0
XM_008007664.1	2.59	########	7.84E-98	sp|Q8IVF5|TIAM2_HUMAN T-lymphoma invasion and metastasis-inducing protein 2 OS=Homo sapiens GN=TIAM2 PE=2 SV=4//0
XM_008007701.1	1.4212	0.000113	0.000997	sp|Q96JX3|SRAC1_HUMAN Protein SERAC1 OS=Homo sapiens GN=SERAC1 PE=1 SV=1//0
XM_008007706.1	0.77641	9.46E-08	1.28E-06	sp|Q9NRJ4|TULP4_HUMAN Tubby-related protein 4 OS=Homo sapiens GN=TULP4 PE=2 SV=2//0
XM_008007707.1	0.4964	0.000246	0.00204	sp|Q9P2C4|TM181_HUMAN Transmembrane protein 181 OS=Homo sapiens GN=TMEM181 PE=1 SV=2//0
XM_008007713.1	0.42123	1.35E-09	2.28E-08	sp|P15311|EZRI_HUMAN Ezrin OS=Homo sapiens GN=EZR PE=1 SV=4//0
XM_008007729.1	-0.55904	6.07E-09	9.50E-08	sp|Q9BWD1|THIC_HUMAN Acetyl-CoA acetyltransferase, cytosolic OS=Homo sapiens GN=ACAT2 PE=1 SV=2//0
XM_008007740.1	-0.33668	5.35E-05	0.000498	sp|P11717|MPRI_HUMAN Cation-independent mannose-6-phosphate receptor OS=Homo sapiens GN=IGF2R PE=1 SV=3//0
XM_008007770.1	1.0617	3.04E-40	3.08E-38	sp|Q5W9D5|QKI_PIG Protein quaking OS=Sus scrofa GN=QKI PE=2 SV=1//8.1931e-

				07
XM_008007800.1	-0.83035	1.78E-05	0.000177	sp|O00584|RNT2_HUMAN Ribonuclease T2 OS=Homo sapiens GN=RNASET2 PE=1 SV=2//2.35187e-166
XM_008007817.1	-0.55035	1.41E-11	2.90E-10	sp|A5A6I3|EIF3F_PANTR Eukaryotic translation initiation factor 3 subunit F OS=Pan troglodytes GN=EIF3F PE=2 SV=1//0
XM_008007862.1	-1.4131	1.21E-07	1.61E-06	sp|A2BDB0|ACTG_XENLA Actin, cytoplasmic 2 OS=Xenopus laevis GN=actg1 PE=2 SV=1//0
XM_008007879.1	0.65249	0.000624	0.004816	sp|A2RRH5|WDR27_HUMAN WD repeat-containing protein 27 OS=Homo sapiens GN=WDR27 PE=1 SV=3//3.74829e-11
XM_008007880.1	0.30228	0.00423	0.026956	sp|Q9Y446|PKP3_HUMAN Plakophilin-3 OS=Homo sapiens GN=PKP3 PE=1 SV=1//0
XM_008007884.1	0.56252	9.94E-05	0.000884	sp|Q9BGQ6|CF120_MACFA UPF0669 protein C6orf120 homolog OS=Macaca fascicularis GN=QflA-14362 PE=2 SV=2//2.06156e-123
XM_008007892.1	-0.50114	2.76E-07	3.54E-06	sp|P20618|PSB1_HUMAN Proteasome subunit beta type-1 OS=Homo sapiens GN=PSMB1 PE=1 SV=2//4.47746e-175
XM_008007917.1	0.81054	6.70E-14	1.69E-12	sp|Q6UB35|C1TM_HUMAN Monofunctional C1-tetrahydrofolate synthase, mitochondrial OS=Homo sapiens GN=MTHFD1L PE=1 SV=1//1.51808e-147
XM_008007926.1	0.82537	8.52E-08	1.16E-06	sp|P29037|TBP_MOUSE TATA-box-binding protein OS=Mus musculus GN=Tbp PE=1 SV=1//5.34531e-124
XM_008007961.1	-3.9623	0.007642	0.04537	sp|P18089|ADA2B_HUMAN Alpha-2B adrenergic receptor OS=Homo sapiens GN=ADRA2B PE=1 SV=3//0
XM_008007965.1	0.25588	0.00098	0.007248	sp|Q9NQZ5|STAR7_HUMAN StAR-related lipid transfer protein 7, mitochondrial OS=Homo sapiens GN=STARD7 PE=1 SV=2//0
XM_008007969.1	-0.3307	3.59E-06	4.00E-05	sp|O75643|U520_HUMAN U5 small nuclear ribonucleoprotein 200 kDa helicase OS=Homo sapiens GN=SNRNP200 PE=1 SV=2//0
XM_008007976.1	4.0386	6.28E-45	7.43E-43	sp|Q96EH8|NEUL3_HUMAN E3 ubiquitin-protein ligase NEURL3 OS=Homo sapiens GN=NEURL3 PE=2 SV=2//2.18286e-171
XM_008007980.1	-4.1058	0.003477	0.022579	-//-
XM_008008033.1	1.023	1.22E-06	1.45E-05	sp|Q6P4Q7|CNNM4_HUMAN Metal transporter CNNM4 OS=Homo sapiens GN=CNNM4 PE=1 SV=3//0
XM_008008069.1	-0.86162	4.51E-11	8.91E-10	sp|P10606|COX5B_HUMAN Cytochrome c oxidase subunit 5B, mitochondrial OS=Homo sapiens GN=COX5B PE=1 SV=2//4.01303e-67
XM_008008111.1	-0.82631	4.28E-10	7.64E-09	sp|Q53HI1|UNC50_HUMAN Protein unc-50 homolog OS=Homo sapiens GN=UNC50 PE=1 SV=2//2.19203e-160
XM_008008112.1	-0.50941	5.70E-05	0.000528	sp|Q86WW8|COA5_HUMAN Cytochrome c oxidase assembly factor 5 OS=Homo sapiens GN=COA5 PE=1 SV=1//1.98982e-39
XM_008008159.1	0.45273	2.06E-05	0.000204	sp|O18883|TXND9_BOVIN Thioredoxin domain-containing protein 9 OS=Bos taurus GN=TXNDC9 PE=2 SV=2//1.01364e-136
XM_008008183.1	-0.25754	0.008429	0.049642	sp|Q9H2J4|PDCL3_HUMAN Phosducin-like protein 3 OS=Homo sapiens GN=PDCL3 PE=1 SV=1//6.20248e-130
XM_008008206.1	0.9394	1.57E-28	9.96E-27	sp|Q8NC42|RN149_HUMAN E3 ubiquitin-protein ligase RNF149 OS=Homo sapiens GN=RNF149 PE=2 SV=2//0
XM_008008207.1	4.3213	0.002386	0.016085	sp|Q6ZV50|RFX8_HUMAN DNA-binding protein RFX8 OS=Homo sapiens GN=RFX8 PE=2 SV=2//0
XM_008008314.1	-3.3275	0.006489	0.039492	sp|O75897|ST1C4_HUMAN Sulfotransferase 1C4 OS=Homo sapiens GN=SULT1C4 PE=1 SV=2//0
XM_008008350.1	3.3317	########	########	sp|Q53LP3|SWAHC_HUMAN Ankyrin repeat domain-containing protein SOWAHC OS=Homo sapiens GN=SOWAHC PE=1 SV=1//0
XM_008008368.1	0.83287	0.005347	0.033261	sp|O43521|B2L11_HUMAN Bcl-2-like protein 11 OS=Homo sapiens GN=BCL2L11 PE=1 SV=1//1.33453e-101
XM_008008389.1	1.631	3.70E-07	4.68E-06	sp|Q8N5P1|ZC3H8_HUMAN Zinc finger CCCH domain-containing protein 8 OS=Homo sapiens GN=ZC3H8 PE=1 SV=2//6.54788e-160
XM_008008391.1	0.98459	0.001099	0.008046	sp|P61129|ZC3H6_HUMAN Zinc finger CCCH domain-containing protein 6 OS=Homo sapiens GN=ZC3H6 PE=2 SV=2//0
XM_008008400.1	-0.769	0.000229	0.001912	sp|Q9BSY4|CHCH5_HUMAN Coiled-coil-helix-coiled-coil-helix domain-containing protein 5 OS=Homo sapiens GN=CHCHD5 PE=1 SV=1//1.12833e-72
XM_008008401.1	1.9088	########	########	sp|Q5R9L5|S20A1_PONAB Sodium-dependent phosphate transporter 1 OS=Pongo abelii GN=SLC20A1 PE=2 SV=1//0
XM_008008439.1	-0.46123	4.48E-08	6.28E-07	sp|Q60HH1|TPP1_MACFA Tripeptidyl-peptidase 1 OS=Macaca fascicularis GN=TPP1 PE=2 SV=2//0
XM_008008442.1	-1.467	0.000373	0.003009	-//-
XM_008008456.1	4.1678	4.33E-65	9.68E-63	sp|O43159|RRP8_HUMAN Ribosomal RNA-processing protein 8 OS=Homo sapiens GN=RRP8 PE=1 SV=2//0
XM_008008480.1	-0.98462	0.001479	0.010514	sp|Q5RFE6|THNS2_PONAB Threonine synthase-like 2 OS=Pongo abelii GN=THNSL2 PE=2 SV=1//0
XM_008008493.1	0.58362	0.000112	0.000986	sp|Q9NPI7|KRCC1_HUMAN Lysine-rich coiled-coil protein 1 OS=Homo sapiens GN=KRCC1 PE=2 SV=1//1.26462e-145
XM_008008507.1	-1.1654	0.000906	0.006737	sp|Q96GK7|FAH2A_HUMAN Fumarylacetoacetate hydrolase domain-containing protein 2A OS=Homo sapiens GN=FAHD2A PE=1 SV=1//1.18446e-149
XM_008008512.1	0.91823	1.60E-05	0.00016	sp|Q12866|MERTK_HUMAN Tyrosine-protein kinase Mer OS=Homo sapiens GN=MERTK PE=1 SV=2//0

XM_008008531.1	0.76967	8.27E-21	3.42E-19	sp|O00629|IMA3_HUMAN Importin subunit alpha-3 OS=Homo sapiens GN=KPNA4 PE=1 SV=1//0
XM_008008545.1	1.6262	9.13E-14	2.28E-12	sp|F5H4A9|CC080_HUMAN Uncharacterized membrane protein C3orf80 OS=Homo sapiens GN=C3orf80 PE=2 SV=1//8.82184e-66
XM_008008571.1	-1.7562	2.24E-08	3.27E-07	sp|Q9BS40|LXN_HUMAN Latexin OS=Homo sapiens GN=LXN PE=1 SV=2//2.77979e-154
XM_008008596.1	0.83462	1.62E-07	2.14E-06	sp|Q9UK58|CCNL1_HUMAN Cyclin-L1 OS=Homo sapiens GN=CCNL1 PE=1 SV=1//0
XM_008008660.1	0.60326	8.16E-05	0.000739	sp|Q9H2U1|DHX36_HUMAN ATP-dependent RNA helicase DHX36 OS=Homo sapiens GN=DHX36 PE=1 SV=2//0
XM_008008665.1	-1.284	6.41E-10	1.12E-08	sp|P47900|P2RY1_HUMAN P2Y purinoceptor 1 OS=Homo sapiens GN=P2RY1 PE=1 SV=1//0
XM_008008725.1	1.6445	1.04E-37	9.52E-36	sp|O43255|SIAH2_HUMAN E3 ubiquitin-protein ligase SIAH2 OS=Homo sapiens GN=SIAH2 PE=1 SV=1//0
XM_008008730.1	0.33154	0.001592	0.011237	sp|P62341|SELT_HUMAN Selenoprotein T OS=Homo sapiens GN=SELT PE=2 SV=2//2.6199e-132
XM_008008731.1	0.5362	4.30E-08	6.05E-07	sp|Q9R2C1|SERP1_RAT Stress-associated endoplasmic reticulum protein 1 OS=Rattus norvegicus GN=Serp1 PE=1 SV=2//7.33768e-34
XM_008008738.1	2.4915	3.48E-32	2.53E-30	sp|O75157|T22D2_HUMAN TSC22 domain family protein 2 OS=Homo sapiens GN=TSC22D2 PE=1 SV=3//0
XM_008008740.1	0.56521	1.55E-07	2.05E-06	sp|Q5RCV8|RNF13_PONAB E3 ubiquitin-protein ligase RNF13 OS=Pongo abelii GN=RNF13 PE=2 SV=1//0
XM_008008741.1	-0.23143	0.002592	0.017323	sp|Q5R4E2|PROF2_PONAB Profilin-2 OS=Pongo abelii GN=PFN2 PE=2 SV=3//5.99537e-91
XM_008008745.1	0.27557	0.004662	0.029461	sp|Q86X83|COMD2_HUMAN COMM domain-containing protein 2 OS=Homo sapiens GN=COMMD2 PE=1 SV=2//3.40029e-112
XM_008008832.1	1.2316	2.13E-28	1.34E-26	sp|Q8NDZ4|DIA1_HUMAN Deleted in autism protein 1 OS=Homo sapiens GN=C3orf58 PE=1 SV=1//0
XM_008008880.1	-1.15	0.003672	0.023719	sp|Q14188|TFDP2_HUMAN Transcription factor Dp-2 OS=Homo sapiens GN=TFDP2 PE=1 SV=2//1.83766e-12
XM_008008913.1	0.78985	7.48E-13	1.72E-11	sp|Q96A44|SPSB4_HUMAN SPRY domain-containing SOCS box protein 4 OS=Homo sapiens GN=SPSB4 PE=1 SV=1//0
XM_008008923.1	-0.69394	6.63E-06	7.05E-05	sp|P09455|RET1_HUMAN Retinol-binding protein 1 OS=Homo sapiens GN=RBP1 PE=1 SV=2//4.90472e-86
XM_008009044.1	-0.75141	1.33E-09	2.24E-08	sp|P05166|PCCB_HUMAN Propionyl-CoA carboxylase beta chain, mitochondrial OS=Homo sapiens GN=PCCB PE=1 SV=3//0
XM_008009045.1	1.1932	2.79E-31	1.95E-29	sp|Q9HCI7|MSL2_HUMAN E3 ubiquitin-protein ligase MSL2 OS=Homo sapiens GN=MSL2 PE=1 SV=2//0
XM_008009081.1	-0.33593	0.007591	0.04511	sp|P61294|RAB6B_MOUSE Ras-related protein Rab-6B OS=Mus musculus GN=Rab6b PE=1 SV=1//3.46603e-137
XM_008009127.1	-0.65136	0.000921	0.006831	sp|Q96DE0|NUD16_HUMAN U8 snoRNA-decapping enzyme OS=Homo sapiens GN=NUDT16 PE=1 SV=2//5.7709e-88
XM_008009161.1	0.28305	0.001803	0.012527	sp|Q99570|PI3R4_HUMAN Phosphoinositide 3-kinase regulatory subunit 4 OS=Homo sapiens GN=PIK3R4 PE=1 SV=3//0
XM_008009179.1	1.2088	5.02E-33	3.81E-31	sp|Q5RAM8|EAF1_PONAB ELL-associated factor 1 OS=Pongo abelii GN=EAF1 PE=2 SV=1//1.03221e-117
XM_008009180.1	0.82398	0.000374	0.003016	sp|Q8TCB7|METL6_HUMAN Methyltransferase-like protein 6 OS=Homo sapiens GN=METTL6 PE=2 SV=2//0
XM_008009181.1	-0.68369	0.002621	0.017495	sp|P23528|COF1_HUMAN Cofilin-1 OS=Homo sapiens GN=CFL1 PE=1 SV=3//2.37961e-52
XM_008009210.1	1.2464	0.004335	0.027564	sp|O15084|ANR28_HUMAN Serine/threonine-protein phosphatase 6 regulatory ankyrin repeat subunit A OS=Homo sapiens GN=ANKRD28 PE=1 SV=5//5.33141e-28
XM_008009261.1	0.63736	1.08E-10	2.05E-09	sp|P61271|RAB5A_MACFA Ras-related protein Rab-5A OS=Macaca fascicularis GN=RAB5A PE=2 SV=1//6.25811e-152
XM_008009290.1	2.4602	########	########	sp|Q14995|NR1D2_HUMAN Nuclear receptor subfamily 1 group D member 2 OS=Homo sapiens GN=NR1D2 PE=1 SV=3//0
XM_008009292.1	1.7187	1.78E-07	2.34E-06	sp|P10828|THB_HUMAN Thyroid hormone receptor beta OS=Homo sapiens GN=THRB PE=1 SV=2//0
XM_008009318.1	-2.0738	0.003459	0.022497	sp|Q6W4X9|MUC6_HUMAN Mucin-6 OS=Homo sapiens GN=MUC6 PE=1 SV=3//7.36463e-06
XM_008009328.1	-2.9381	8.58E-05	0.000773	sp|P98088|MUC5A_HUMAN Mucin-5AC OS=Homo sapiens GN=MUC5AC PE=1 SV=4//3.89528e-11
XM_008009344.1	-1.4959	0.005291	0.03295	sp|Q9HC84|MUC5B_HUMAN Mucin-5B OS=Homo sapiens GN=MUC5B PE=1 SV=3//2.02139e-06
XM_008009359.1	3.6556	########	########	-//-
XM_008009369.1	1.4987	8.07E-07	9.86E-06	-//-
XM_008009374.1	-0.6625	1.34E-14	3.58E-13	sp|Q9NX76|CKLF6_HUMAN CKLF-like MARVEL transmembrane domain-containing protein 6 OS=Homo sapiens GN=CMTM6 PE=1 SV=1//1.35995e-102
XM_008009383.1	1.174	0.003942	0.025326	sp|Q2Q1W2|LIN41_HUMAN E3 ubiquitin-protein ligase TRIM71 OS=Homo sapiens GN=TRIM71 PE=1 SV=1//1.81048e-40
XM_008009389.1	-0.71792	8.78E-13	2.01E-11	sp|Q60HF6|BGAL_MACFA Beta-galactosidase OS=Macaca fascicularis GN=GLB1 PE=2 SV=1//0

XM_008009390.1	-1.0351	5.43E-31	3.77E-29	sp|O75718|CRTAP_HUMAN Cartilage-associated protein OS=Homo sapiens GN=CRTAP PE=1 SV=1//0
XM_008009428.1	-1.3757	4.91E-06	5.35E-05	-//-
XM_008009507.1	1.0648	1.33E-06	1.58E-05	sp|P41182|BCL6_HUMAN B-cell lymphoma 6 protein OS=Homo sapiens GN=BCL6 PE=1 SV=1//0
XM_008009525.1	0.72693	0.0039	0.025091	sp|P59822|IL1AP_MACMU Interleukin-1 receptor accessory protein OS=Macaca mulatta GN=IL1RAP PE=1 SV=1//0
XM_008009535.1	0.64875	9.43E-05	0.000843	sp|P61150|FGF12_RAT Fibroblast growth factor 12 OS=Rattus norvegicus GN=Fgf12 PE=1 SV=1//2.63323e-157
XM_008009537.1	-0.38803	0.000535	0.004178	sp|Q12851|M4K2_HUMAN Mitogen-activated protein kinase kinase kinase kinase 2 OS=Homo sapiens GN=MAP4K2 PE=1 SV=2//0
XM_008009539.1	2.3675	4.37E-05	0.000412	sp|Q8IYB1|M21D2_HUMAN Protein MB21D2 OS=Homo sapiens GN=MB21D2 PE=1 SV=3//0
XM_008009556.1	1.0697	3.14E-30	2.14E-28	sp|Q14469|HES1_HUMAN Transcription factor HES-1 OS=Homo sapiens GN=HES1 PE=1 SV=1//4.0483e-153
XM_008009561.1	-0.74656	1.87E-13	4.57E-12	sp|Q09666|AHNK_HUMAN Neuroblast differentiation-associated protein AHNAK OS=Homo sapiens GN=AHNAK PE=1 SV=2//0
XM_008009574.1	2.2038	5.99E-17	1.92E-15	sp|Q8N2R8|FA43A_HUMAN Protein FAM43A OS=Homo sapiens GN=FAM43A PE=2 SV=2//0
XM_008009576.1	1.4147	2.98E-38	2.78E-36	sp|Q4R8L2|LSG1_MACFA Large subunit GTPase 1 homolog OS=Macaca fascicularis GN=LSG1 PE=2 SV=1//0
XM_008009578.1	1.1625	1.35E-17	4.54E-16	sp|Q8NBI6|XXLT1_HUMAN Xyloside xylosyltransferase 1 OS=Homo sapiens GN=XXYLT1 PE=1 SV=1//0
XM_008009584.1	-0.62056	3.55E-08	5.04E-07	sp|P02794|FRIH_HUMAN Ferritin heavy chain OS=Homo sapiens GN=FTH1 PE=1 SV=2//2.13876e-121
XM_008009656.1	1.5358	1.85E-27	1.11E-25	sp|P0C2W1|FBSP1_HUMAN F-box/SPRY domain-containing protein 1 OS=Homo sapiens GN=FBXO45 PE=1 SV=1//1.22386e-176
XM_008009658.1	0.37987	0.004159	0.02658	sp|Q8IYW5|RN168_HUMAN E3 ubiquitin-protein ligase RNF168 OS=Homo sapiens GN=RNF168 PE=1 SV=1//0
XM_008009676.1	-0.54204	0.000253	0.002095	sp|Q86UW1|OSTA_HUMAN Organic solute transporter subunit alpha OS=Homo sapiens GN=SLC51A PE=2 SV=1//0
XM_008009728.1	0.57425	5.79E-06	6.24E-05	sp|Q96CQ1|S2536_HUMAN Solute carrier family 25 member 36 OS=Homo sapiens GN=SLC25A36 PE=1 SV=1//0
XM_008009733.1	1.2691	1.70E-16	5.24E-15	sp|Q14699|RFTN1_HUMAN Raftlin OS=Homo sapiens GN=RFTN1 PE=1 SV=4//0
XM_008009734.1	0.57746	3.22E-09	5.21E-08	sp|Q9Y6G9|DC1L1_HUMAN Cytoplasmic dynein 1 light intermediate chain 1 OS=Homo sapiens GN=DYNC1LI1 PE=1 SV=3//0
XM_008009739.1	-1.1965	1.17E-16	3.65E-15	sp|Q99102|MUC4_HUMAN Mucin-4 OS=Homo sapiens GN=MUC4 PE=1 SV=4//0
XM_008009740.1	-2.9713	2.63E-07	3.39E-06	-//-
XM_008009746.1	0.6089	5.37E-05	0.0005	sp|Q12979|ABR_HUMAN Active breakpoint cluster region-related protein OS=Homo sapiens GN=ABR PE=2 SV=2//0
XM_008009747.1	0.6685	1.10E-06	1.32E-05	sp|Q9JKW1|TIM22_RAT Mitochondrial import inner membrane translocase subunit Tim22 OS=Rattus norvegicus GN=Timm22 PE=2 SV=2//2.01685e-82
XM_008009748.1	0.75002	1.39E-13	3.42E-12	sp|Q6DKJ4|NXN_HUMAN Nucleoredoxin OS=Homo sapiens GN=NXN PE=1 SV=2//0
XM_008009749.1	0.95956	3.03E-24	1.55E-22	sp|Q9Z0W3|NU160_MOUSE Nuclear pore complex protein Nup160 OS=Mus musculus GN=Nup160 PE=1 SV=2//0
XM_008009758.1	-0.77795	0.000214	0.001795	sp|Q8CCB4|VPS53_MOUSE Vacuolar protein sorting-associated protein 53 homolog OS=Mus musculus GN=Vps53 PE=2 SV=1//0
XM_008009759.1	-0.85418	5.24E-10	9.21E-09	sp|Q8N5W9|F101B_HUMAN Filamin-interacting protein FAM101B OS=Homo sapiens GN=FAM101B PE=1 SV=1//2.3515e-66
XM_008009785.1	-0.52241	0.006383	0.038953	sp|A6NGC4|TLCD2_HUMAN TLC domain-containing protein 2 OS=Homo sapiens GN=TLCD2 PE=3 SV=3//3.78359e-121
XM_008009786.1	-0.28579	4.38E-05	0.000412	sp|Q6P2Q9|PRP8_HUMAN Pre-mRNA-processing-splicing factor 8 OS=Homo sapiens GN=PRPF8 PE=1 SV=2//0
XM_008009808.1	-1.3546	3.71E-17	1.21E-15	sp|Q86UN2|R4RL1_HUMAN Reticulon-4 receptor-like 1 OS=Homo sapiens GN=RTN4RL1 PE=1 SV=1//0
XM_008009821.1	0.49875	2.51E-10	4.58E-09	sp|Q9H422|HIPK3_HUMAN Homeodomain-interacting protein kinase 3 OS=Homo sapiens GN=HIPK3 PE=1 SV=1//0
XM_008009825.1	0.60047	4.66E-09	7.40E-08	sp|Q2NL82|TSR1_HUMAN Pre-rRNA-processing protein TSR1 homolog OS=Homo sapiens GN=TSR1 PE=1 SV=1//0
XM_008009829.1	0.9499	1.18E-07	1.57E-06	sp|Q99583|MNT_HUMAN Max-binding protein MNT OS=Homo sapiens GN=MNT PE=1 SV=1//0
XM_008009830.1	0.70715	1.41E-09	2.37E-08	sp|Q86W50|MET16_HUMAN Methyltransferase-like protein 16 OS=Homo sapiens GN=METTL16 PE=1 SV=2//0
XM_008009832.1	1.4101	1.86E-17	6.21E-16	sp|Q5RDW9|CSTF3_PONAB Cleavage stimulation factor subunit 3 OS=Pongo abelii GN=CSTF3 PE=2 SV=1//1.44934e-96
XM_008009833.1	0.65831	3.01E-14	7.75E-13	sp|Q8HXX0|LIS1_MACFA Platelet-activating factor acetylhydrolase IB subunit alpha OS=Macaca fascicularis GN=PAFAH1B1 PE=2 SV=3//0
XM_008009878.1	1.5895	6.29E-27	3.65E-25	sp|Q8TF76|HASP_HUMAN Serine/threonine-protein kinase haspin OS=Homo sapiens GN=GSG2 PE=1 SV=3//0

XM_008009904.1	0.7107	7.00E-13	1.62E-11	sp|O43149|ZZEF1_HUMAN Zinc finger ZZ-type and EF-hand domain-containing protein 1 OS=Homo sapiens GN=ZZEF1 PE=1 SV=6//0
XM_008009916.1	1.1282	1.48E-08	2.20E-07	sp|Q9BQG0|MBB1A_HUMAN Myb-binding protein 1A OS=Homo sapiens GN=MYBBP1A PE=1 SV=2//0
XM_008009925.1	-1.0804	7.66E-05	0.000698	sp|Q2TAL5|SMTL2_HUMAN Smoothelin-like protein 2 OS=Homo sapiens GN=SMTNL2 PE=2 SV=1//0
XM_008009926.1	-1.6981	0.002307	0.015641	sp|Q2TAL5|SMTL2_HUMAN Smoothelin-like protein 2 OS=Homo sapiens GN=SMTNL2 PE=2 SV=1//3.10615e-159
XM_008009928.1	0.46646	0.000433	0.003443	sp|Q1W1Y5|PELP1_MACMU Proline-, glutamic acid- and leucine-rich protein 1 OS=Macaca mulatta GN=PELP1 PE=2 SV=1//1.02638e-30
XM_008009980.1	-0.57845	7.61E-15	2.07E-13	sp|P07737|PROF1_HUMAN Profilin-1 OS=Homo sapiens GN=PFN1 PE=1 SV=2//2.65892e-85
XM_008009981.1	3.0345	8.53E-05	0.000769	sp|P13929|ENOB_HUMAN Beta-enolase OS=Homo sapiens GN=ENO3 PE=1 SV=5//0
XM_008009984.1	0.96432	1.72E-20	6.96E-19	sp|O00443|P3C2A_HUMAN Phosphatidylinositol 4-phosphate 3-kinase C2 domain-containing subunit alpha OS=Homo sapiens GN=PIK3C2A PE=1 SV=2//0
XM_008009985.1	-0.74361	7.99E-05	0.000724	sp|O75391|SPAG7_HUMAN Sperm-associated antigen 7 OS=Homo sapiens GN=SPAG7 PE=1 SV=2//4.1272e-135
XM_008010005.1	-0.67059	0.001115	0.008152	sp|Q96JF6|ZN594_HUMAN Zinc finger protein 594 OS=Homo sapiens GN=ZNF594 PE=2 SV=3//0
XM_008010015.1	0.2767	0.000864	0.006461	sp|Q9MZE0|C1QBP_CHLAE Complement component 1 Q subcomponent-binding protein, mitochondrial OS=Chlorocebus aethiops GN=C1QBP PE=1 SV=2//0
XM_008010041.1	0.60287	0.000276	0.002277	sp|Q9BZ71|PITM3_HUMAN Membrane-associated phosphatidylinositol transfer protein 3 OS=Homo sapiens GN=PITPNM3 PE=1 SV=2//0
XM_008010055.1	2.8497	3.44E-06	3.84E-05	sp|Q6GPH4|XAF1_HUMAN XIAP-associated factor 1 OS=Homo sapiens GN=XAF1 PE=1 SV=1//0
XM_008010095.1	-0.51684	5.40E-08	7.51E-07	sp|Q8HXY7|ACADV_MACFA Very long-chain specific acyl-CoA dehydrogenase, mitochondrial OS=Macaca fascicularis GN=ACADVL PE=2 SV=1//0
XM_008010101.1	-0.45492	1.80E-08	2.65E-07	sp|P60517|GBRAP_RAT Gamma-aminobutyric acid receptor-associated protein OS=Rattus norvegicus GN=Gabarap PE=1 SV=1//1.72297e-77
XM_008010127.1	0.86114	1.98E-09	3.27E-08	sp|Q693B1|KCD11_HUMAN BTB/POZ domain-containing protein KCTD11 OS=Homo sapiens GN=KCTD11 PE=1 SV=1//9.89201e-152
XM_008010138.1	-1.3452	1.87E-05	0.000185	sp|Q8NFZ4|NLGN2_HUMAN Neuroligin-2 OS=Homo sapiens GN=NLGN2 PE=1 SV=1//0
XM_008010139.1	-0.91485	0.001574	0.011126	sp|Q8N2U0|TM256_HUMAN Transmembrane protein 256 OS=Homo sapiens GN=TMEM256 PE=3 SV=1//1.2394e-38
XM_008010141.1	-0.79193	1.68E-12	3.74E-11	sp|Q5R7K4|PCMD2_PONAB Protein-L-isoaspartate O-methyltransferase domain-containing protein 2 OS=Pongo abelii GN=PCMTD2 PE=2 SV=1//0
XM_008010165.1	0.92206	1.25E-20	5.10E-19	sp|P51116|FXR2_HUMAN Fragile X mental retardation syndrome-related protein 2 OS=Homo sapiens GN=FXR2 PE=1 SV=2//0
XM_008010174.1	-0.55099	0.002987	0.019684	sp|P34810|CD68_HUMAN Macrosialin OS=Homo sapiens GN=CD68 PE=1 SV=2//1.84566e-147
XM_008010176.1	0.79346	1.49E-07	1.97E-06	sp|Q9H4L4|SENP3_HUMAN Sentrin-specific protease 3 OS=Homo sapiens GN=SENP3 PE=1 SV=2//0
XM_008010210.1	-0.90357	1.74E-13	4.27E-12	sp|Q9BRA0|LSMD1_HUMAN N-alpha-acetyltransferase 38, NatC auxiliary subunit OS=Homo sapiens GN=NAA38 PE=1 SV=1//2.44731e-53
XM_008010232.1	-0.88208	7.30E-15	1.99E-13	sp|Q9Y5R8|TPPC1_HUMAN Trafficking protein particle complex subunit 1 OS=Homo sapiens GN=TRAPPC1 PE=1 SV=1//2.04016e-103
XM_008010233.1	0.72677	5.18E-06	5.63E-05	sp|Q8N137|CNTRB_HUMAN Centrobin OS=Homo sapiens GN=CNTROB PE=1 SV=1//0
XM_008010236.1	-1.7824	7.22E-05	0.000659	sp|Q8TD35|LKAM1_HUMAN Protein LKAAEAR1 OS=Homo sapiens GN=LKAAEAR1 PE=2 SV=3//2.00805e-102
XM_008010246.1	3.1322	1.10E-93	4.64E-91	sp|Q9BYJ1|LOXE3_HUMAN Hydroperoxide isomerase ALOXE3 OS=Homo sapiens GN=ALOXE3 PE=1 SV=1//0
XM_008010264.1	-0.70524	7.89E-08	1.08E-06	sp|P63045|VAMP2_RAT Vesicle-associated membrane protein 2 OS=Rattus norvegicus GN=Vamp2 PE=1 SV=2//1.06727e-40
XM_008010274.1	-2.0762	0.000142	0.001224	sp|P41146|OPRX_HUMAN Nociceptin receptor OS=Homo sapiens GN=OPRL1 PE=1 SV=1//0
XM_008010282.1	0.28594	0.004938	0.031002	sp|O15067|PUR4_HUMAN Phosphoribosylformylglycinamidine synthase OS=Homo sapiens GN=PFAS PE=1 SV=4//0
XM_008010296.1	-0.51793	2.75E-12	6.01E-11	sp|P61255|RL26_MOUSE 60S ribosomal protein L26 OS=Mus musculus GN=Rpl26 PE=1 SV=1//1.33057e-86
XM_008010298.1	1.6461	1.07E-45	1.29E-43	sp|Q9GZM8|NDEL1_HUMAN Nuclear distribution protein nudE-like 1 OS=Homo sapiens GN=NDEL1 PE=1 SV=1//0
XM_008010326.1	-0.62239	0.000446	0.00354	sp|P35713|SOX18_HUMAN Transcription factor SOX-18 OS=Homo sapiens GN=SOX18 PE=1 SV=2//2.71018e-08
XM_008010476.1	0.87091	2.68E-16	8.08E-15	sp|Q96KM6|Z512B_HUMAN Zinc finger protein 512B OS=Homo sapiens GN=ZNF512B PE=1 SV=1//0
XM_008010492.1	2.2716	5.92E-73	1.62E-70	sp|P29275|AA2BR_HUMAN Adenosine receptor A2b OS=Homo sapiens GN=ADORA2B PE=2 SV=1//0
XM_008010510.1	1.077	6.48E-09	1.01E-07	sp|Q8NFG4|FLCN_HUMAN Folliculin OS=Homo sapiens GN=FLCN PE=1 SV=1//0
XM_008010525.1	1.0797	8.72E-18	2.96E-16	sp|Q9NWA0|MED9_HUMAN Mediator of RNA polymerase II transcription subunit 9 OS=Homo sapiens GN=MED9 PE=1 SV=1//3.65809e-53

XM_008010545.1	-0.4943	2.78E-06	3.15E-05	sp|D3ZKD3|ALKB5_RAT RNA demethylase ALKBH5 OS=Rattus norvegicus GN=Alkbh5 PE=3 SV=1//0
XM_008010547.1	1.3433	1.73E-31	1.22E-29	sp|Q8IVV7|GID4_HUMAN Glucose-induced degradation protein 4 homolog OS=Homo sapiens GN=GID4 PE=2 SV=1//2.23384e-156
XM_008010553.1	2.1448	0.000108	0.000955	sp|Q4R550|SYCC_MACFA Cysteine--tRNA ligase, cytoplasmic OS=Macaca fascicularis GN=CARS PE=2 SV=1//1.18127e-11
XM_008010580.1	1.2952	1.56E-34	1.27E-32	sp|Q8TEV9|SMCR8_HUMAN Smith-Magenis syndrome chromosomal region candidate gene 8 protein OS=Homo sapiens GN=SMCR8 PE=1 SV=2//0
XM_008010581.1	0.90032	1.67E-19	6.38E-18	sp|Q13472|TOP3A_HUMAN DNA topoisomerase 3-alpha OS=Homo sapiens GN=TOP3A PE=1 SV=1//0
XM_008010591.1	1.1523	6.78E-13	1.58E-11	sp|A0PJK1|SC5AA_HUMAN Sodium/glucose cotransporter 5 OS=Homo sapiens GN=SLC5A10 PE=1 SV=2//0
XM_008010592.1	1.112	2.35E-16	7.14E-15	sp|Q5RBA8|KPRB_PONAB Phosphoribosyl pyrophosphate synthase-associated protein 2 OS=Pongo abelii GN=PRPSAP2 PE=2 SV=1//0
XM_008010593.1	1.0471	0.002065	0.014146	sp|Q13588|GRAP_HUMAN GRB2-related adapter protein OS=Homo sapiens GN=GRAP PE=1 SV=1//4.36596e-152
XM_008010635.1	1.0481	8.69E-06	9.08E-05	sp|Q8IYT8|ULK2_HUMAN Serine/threonine-protein kinase ULK2 OS=Homo sapiens GN=ULK2 PE=1 SV=3//0
XM_008010645.1	2.2359	5.94E-05	0.000549	sp|Q5M775|CYTSB_HUMAN Cytospin-B OS=Homo sapiens GN=SPECC1 PE=1 SV=1//0
XM_008010683.1	-0.72846	5.76E-05	0.000533	sp|Q8N6N6|NATD1_HUMAN Protein NATD1 OS=Homo sapiens GN=NATD1 PE=1 SV=2//5.27036e-69
XM_008010691.1	-0.57827	0.000396	0.003171	sp|P36955|PEDF_HUMAN Pigment epithelium-derived factor OS=Homo sapiens GN=SERPINF1 PE=1 SV=4//0
XM_008010696.1	0.86485	0.001277	0.009196	sp|P38570|ITAE_HUMAN Integrin alpha-E OS=Homo sapiens GN=ITGAE PE=1 SV=3//7.42502e-97
XM_008010697.1	0.29812	0.006244	0.038148	sp|Q53F19|CQ085_HUMAN Uncharacterized protein C17orf85 OS=Homo sapiens GN=C17orf85 PE=1 SV=2//0
XM_008010702.1	1.2145	3.17E-16	9.47E-15	sp|Q9H6R0|DHX33_HUMAN Putative ATP-dependent RNA helicase DHX33 OS=Homo sapiens GN=DHX33 PE=1 SV=2//3.42568e-59
XM_008010712.1	0.88402	3.09E-20	1.22E-18	sp|Q5RFS2|CSN3_PONAB COP9 signalosome complex subunit 3 OS=Pongo abelii GN=COPS3 PE=2 SV=1//0
XM_008010734.1	0.87168	2.92E-28	1.82E-26	sp|Q9Y6I7|WSB1_HUMAN WD repeat and SOCS box-containing protein 1 OS=Homo sapiens GN=WSB1 PE=1 SV=1//0
XM_008010735.1	-1.8028	0.00198	0.01358	sp|Q9H400|LIME1_HUMAN Lck-interacting transmembrane adapter 1 OS=Homo sapiens GN=LIME1 PE=1 SV=1//2.74109e-138
XM_008010788.1	-0.75865	0.000383	0.003079	sp|Q13432|U119A_HUMAN Protein unc-119 homolog A OS=Homo sapiens GN=UNC119 PE=1 SV=1//6.3024e-134
XM_008010791.1	-0.46663	5.47E-07	6.81E-06	sp|Q96S52|PIGS_HUMAN GPI transamidase component PIG-S OS=Homo sapiens GN=PIGS PE=1 SV=3//0
XM_008010794.1	-1.7805	2.41E-05	0.000236	sp|Q96LW2|SG494_HUMAN Uncharacterized serine/threonine-protein kinase SgK494 OS=Homo sapiens GN=SGK494 PE=2 SV=1//3.8685e-157
XM_008010807.1	0.60027	3.02E-13	7.27E-12	sp|Q7KZ85|SPT6H_HUMAN Transcription elongation factor SPT6 OS=Homo sapiens GN=SUPT6H PE=1 SV=2//0
XM_008010827.1	-0.99846	0.00705	0.04235	sp|P62752|RL23A_RAT 60S ribosomal protein L23a OS=Rattus norvegicus GN=Rpl23a PE=2 SV=1//1.68862e-83
XM_008010835.1	0.66298	4.81E-11	9.46E-10	sp|Q9BUZ4|TRAF4_HUMAN TNF receptor-associated factor 4 OS=Homo sapiens GN=TRAF4 PE=1 SV=1//0
XM_008010880.1	0.67013	2.17E-19	8.15E-18	sp|Q7Z417|NUFP2_HUMAN Nuclear fragile X mental retardation-interacting protein 2 OS=Homo sapiens GN=NUFIP2 PE=1 SV=1//0
XM_008010938.1	-0.88124	4.64E-09	7.38E-08	sp|Q9H3Y8|PPDPF_HUMAN Pancreatic progenitor cell differentiation and proliferation factor OS=Homo sapiens GN=PPDPF PE=3 SV=1//1.35872e-50
XM_008010940.1	-0.77467	7.29E-16	2.13E-14	sp|O75976|CBPD_HUMAN Carboxypeptidase D OS=Homo sapiens GN=CPD PE=1 SV=2//0
XM_008010956.1	-4.3023	0.002118	0.01447	sp|P23515|OMGP_HUMAN Oligodendrocyte-myelin glycoprotein OS=Homo sapiens GN=OMG PE=1 SV=2//0
XM_008010966.1	0.53773	5.30E-09	8.38E-08	sp|Q9NYH9|UTP6_HUMAN U3 small nucleolar RNA-associated protein 6 homolog OS=Homo sapiens GN=UTP6 PE=2 SV=2//0
XM_008010970.1	0.55365	0.000258	0.002135	sp|Q96QE5|TEFM_HUMAN Transcription elongation factor, mitochondrial OS=Homo sapiens GN=TEFM PE=1 SV=1//0
XM_008010998.1	-0.35529	0.001867	0.012901	sp|O94832|MYO1D_HUMAN Unconventional myosin-Id OS=Homo sapiens GN=MYO1D PE=1 SV=2//0
XM_008011042.1	0.3732	0.002114	0.014448	sp|Q8WZ73|RFFL_HUMAN E3 ubiquitin-protein ligase rififylin OS=Homo sapiens GN=RFFL PE=1 SV=1//0
XM_008011047.1	0.87228	4.88E-08	6.81E-07	sp|Q9NVX2|NLE1_HUMAN Notchless protein homolog 1 OS=Homo sapiens GN=NLE1 PE=1 SV=4//5.47285e-09
XM_008011065.1	1.0838	4.60E-14	1.17E-12	sp|O00623|PEX12_HUMAN Peroxisome assembly protein 12 OS=Homo sapiens GN=PEX12 PE=1 SV=1//0
XM_008011070.1	-0.41559	0.000553	0.004304	sp|Q5SSG5|RSLAB_MOUSE Ras-like protein family member 10B OS=Mus musculus GN=Rasl10b PE=1 SV=1//2.37257e-137
XM_008011173.1	0.78462	2.12E-05	0.000208	sp|Q9NY61|AATF_HUMAN Protein AATF OS=Homo sapiens GN=AATF PE=1 SV=1//0

XM_008011178.1	1.4172	3.88E-23	1.87E-21	sp|Q6IN84|MRM1_HUMAN rRNA methyltransferase 1, mitochondrial OS=Homo sapiens GN=MRM1 PE=1 SV=1//0
XM_008011179.1	0.66308	0.001827	0.012664	sp|Q15649|ZNHI3_HUMAN Zinc finger HIT domain-containing protein 3 OS=Homo sapiens GN=ZNHIT3 PE=1 SV=2//1.41535e-104
XM_008011180.1	0.56735	5.92E-06	6.36E-05	sp|Q7Z7B1|PIGW_HUMAN Phosphatidylinositol-glycan biosynthesis class W protein OS=Homo sapiens GN=PIGW PE=1 SV=1//0
XM_008011202.1	0.77209	1.19E-22	5.47E-21	sp|Q92624|APBP2_HUMAN Amyloid protein-binding protein 2 OS=Homo sapiens GN=APPBP2 PE=1 SV=2//0
XM_008011203.1	2.147	########	########	sp|O15297|PPM1D_HUMAN Protein phosphatase 1D OS=Homo sapiens GN=PPM1D PE=1 SV=1//0
XM_008011223.1	1.0609	6.78E-17	2.15E-15	sp|Q9BX63|FANCJ_HUMAN Fanconi anemia group J protein OS=Homo sapiens GN=BRIP1 PE=1 SV=1//0
XM_008011228.1	1.3371	5.53E-22	2.45E-20	sp|Q9UHV7|MED13_HUMAN Mediator of RNA polymerase II transcription subunit 13 OS=Homo sapiens GN=MED13 PE=1 SV=3//0
XM_008011248.1	-0.2154	0.008441	0.049684	sp|Q8IX18|DHX40_HUMAN Probable ATP-dependent RNA helicase DHX40 OS=Homo sapiens GN=DHX40 PE=1 SV=2//1.56327e-29
XM_008011254.1	0.76503	0.002895	0.019105	sp|Q8N9F7|GDPD1_HUMAN Glycerophosphodiester phosphodiesterase domain-containing protein 1 OS=Homo sapiens GN=GDPD1 PE=1 SV=2//9.22834e-173
XM_008011261.1	1.0739	0.000116	0.00102	sp|Q8WY54|PPM1E_HUMAN Protein phosphatase 1E OS=Homo sapiens GN=PPM1E PE=1 SV=2//3.17069e-09
XM_008011317.1	0.38765	0.00066	0.005073	sp|Q4R941|SPT4H_MACFA Transcription elongation factor SPT4 OS=Macaca fascicularis GN=SUPT4H1 PE=3 SV=1//2.57334e-81
XM_008011318.1	0.74653	1.41E-05	0.000142	sp|Q68DV7|RNF43_HUMAN E3 ubiquitin-protein ligase RNF43 OS=Homo sapiens GN=RNF43 PE=1 SV=1//0
XM_008011341.1	0.49072	0.003453	0.022462	sp|Q9Y3D9|RT23_HUMAN 28S ribosomal protein S23, mitochondrial OS=Homo sapiens GN=MRPS23 PE=1 SV=2//8.18064e-123
XM_008011364.1	0.48176	0.001126	0.008219	sp|Q9BYJ9|YTHD1_HUMAN YTH domain-containing family protein 1 OS=Homo sapiens GN=YTHDF1 PE=1 SV=1//0
XM_008011371.1	0.81491	9.32E-13	2.12E-11	sp|P38432|COIL_HUMAN Coilin OS=Homo sapiens GN=COIL PE=1 SV=1//0
XM_008011373.1	0.55778	7.14E-12	1.50E-10	sp|Q14258|TRI25_HUMAN E3 ubiquitin/ISG15 ligase TRIM25 OS=Homo sapiens GN=TRIM25 PE=1 SV=2//0
XM_008011393.1	Inf	0.001762	0.012278	sp|Q13253|NOGG_HUMAN Noggin OS=Homo sapiens GN=NOG PE=1 SV=1//2.4308e-126
XM_008011412.1	2.4769	1.39E-57	2.62E-55	sp|Q16534|HLF_HUMAN Hepatic leukemia factor OS=Homo sapiens GN=HLF PE=2 SV=1//3.5426e-151
XM_008011438.1	0.37553	0.00308	0.020261	sp|Q9Y5J1|UTP18_HUMAN U3 small nucleolar RNA-associated protein 18 homolog OS=Homo sapiens GN=UTP18 PE=1 SV=3//0
XM_008011460.1	-0.8328	1.08E-07	1.46E-06	sp|P50616|TOB1_HUMAN Protein Tob1 OS=Homo sapiens GN=TOB1 PE=1 SV=1//0
XM_008011474.1	0.91463	1.75E-25	9.44E-24	sp|Q6AI12|ANR40_HUMAN Ankyrin repeat domain-containing protein 40 OS=Homo sapiens GN=ANKRD40 PE=1 SV=2//0
XM_008011507.1	-2.3121	4.14E-06	4.56E-05	sp|O15335|CHAD_HUMAN Chondroadherin OS=Homo sapiens GN=CHAD PE=2 SV=2//0
XM_008011508.1	-0.97393	1.94E-10	3.59E-09	sp|Q4R4Z9|ACSF2_MACFA Acyl-CoA synthetase family member 2, mitochondrial OS=Macaca fascicularis GN=ACSF2 PE=2 SV=1//0
XM_008011585.1	-0.86917	0.000382	0.00307	sp|Q14050|CO9A3_HUMAN Collagen alpha-3(IX) chain OS=Homo sapiens GN=COL9A3 PE=1 SV=2//6.03835e-102
XM_008011607.1	0.78688	4.81E-23	2.30E-21	sp|Q92503|S14L1_HUMAN SEC14-like protein 1 OS=Homo sapiens GN=SEC14L1 PE=1 SV=2//0
XM_008011618.1	0.44881	0.00083	0.006227	sp|Q5R6G2|JMJD6_PONAB Bifunctional arginine demethylase and lysyl-hydroxylase JMJD6 OS=Pongo abelii GN=JMJD6 PE=2 SV=1//0
XM_008011627.1	-2.0386	2.28E-09	3.75E-08	sp|P84157|MXRA7_HUMAN Matrix-remodeling-associated protein 7 OS=Homo sapiens GN=MXRA7 PE=1 SV=1//4.91281e-31
XM_008011662.1	0.32899	0.001398	0.009993	sp|Q9NV56|MRGBP_HUMAN MRG/MORF4L-binding protein OS=Homo sapiens GN=MRGBP PE=1 SV=1//3.49753e-89
XM_008011678.1	-0.57681	3.66E-09	5.89E-08	sp|Q92949|FOXJ1_HUMAN Forkhead box protein J1 OS=Homo sapiens GN=FOXJ1 PE=2 SV=3//0
XM_008011719.1	1.1029	4.19E-13	9.91E-12	sp|Q96LD4|TRI47_HUMAN Tripartite motif-containing protein 47 OS=Homo sapiens GN=TRIM47 PE=1 SV=2//0
XM_008011721.1	0.38404	0.006479	0.039441	sp|Q70J99|UN13D_HUMAN Protein unc-13 homolog D OS=Homo sapiens GN=UNC13D PE=1 SV=1//0
XM_008011728.1	0.71447	3.77E-22	1.69E-20	sp|Q6P823|H33_XENTR Histone H3.3 OS=Xenopus tropicalis GN=TGas113e22.1 PE=1 SV=3//1.85616e-87
XM_008011729.1	-0.58382	2.17E-05	0.000214	sp|P51570|GALK1_HUMAN Galactokinase OS=Homo sapiens GN=GALK1 PE=1 SV=1//0
XM_008011744.1	0.70735	9.10E-10	1.56E-08	sp|O94762|RECQ5_HUMAN ATP-dependent DNA helicase Q5 OS=Homo sapiens GN=RECQL5 PE=1 SV=2//0
XM_008011754.1	0.47261	4.52E-06	4.96E-05	sp|Q8WXE0|CSKI2_HUMAN Caskin-2 OS=Homo sapiens GN=CASKIN2 PE=1 SV=2//0
XM_008011783.1	-0.61216	3.50E-15	9.72E-14	sp|P61959|SUMO2_RAT Small ubiquitin-related modifier 2 OS=Rattus norvegicus GN=Sumo2 PE=1 SV=1//1.20321e-60
XM_008011798.1	-0.97593	4.16E-28	2.57E-26	sp|O75947|ATP5H_HUMAN ATP synthase subunit d, mitochondrial OS=Homo sapiens GN=ATP5H PE=1 SV=3//1.21044e-110
XM_008011799.1	0.90529	8.43E-14	2.11E-12	sp|Q14681|KCTD2_HUMAN BTB/POZ domain-containing protein KCTD2 OS=Homo

				sapiens GN=KCTD2 PE=1 SV=3//2.10811e-122
XM_008011851.1	-0.36195	0.003822	0.024632	sp|Q4R6G4|NHRF1_MACFA Na(+)/H(+) exchange regulatory cofactor NHE-RF1 OS=Macaca fascicularis GN=SLC9A3R1 PE=2 SV=1//0
XM_008011912.1	Inf	0.002798	0.018527	-//-
XM_008011933.1	0.45536	0.000526	0.00411	sp|Q8WTW3|COG1_HUMAN Conserved oligomeric Golgi complex subunit 1 OS=Homo sapiens GN=COG1 PE=1 SV=1//0
XM_008011956.1	2.716	6.83E-13	1.59E-11	sp|Q9BG91|SOX9_CALJA Transcription factor SOX-9 OS=Callithrix jacchus GN=SOX9 PE=2 SV=1//0
XM_008011967.1	-2.1106	1.39E-08	2.09E-07	sp|P52564|MP2K6_HUMAN Dual specificity mitogen-activated protein kinase kinase 6 OS=Homo sapiens GN=MAP2K6 PE=1 SV=1//0
XM_008011975.1	-0.66795	1.61E-13	3.96E-12	sp|Q3ZBG0|PSA7_BOVIN Proteasome subunit alpha type-7 OS=Bos taurus GN=PSMA7 PE=1 SV=1//9.74764e-162
XM_008012007.1	-0.30131	0.000199	0.001677	sp|P52292|IMA1_HUMAN Importin subunit alpha-1 OS=Homo sapiens GN=KPNA2 PE=1 SV=1//0
XM_008012009.1	0.34193	9.33E-06	9.68E-05	sp|Q12830|BPTF_HUMAN Nucleosome-remodeling factor subunit BPTF OS=Homo sapiens GN=BPTF PE=1 SV=3//0
XM_008012033.1	-0.34695	0.006619	0.040171	sp|P42694|HELZ_HUMAN Probable helicase with zinc finger domain OS=Homo sapiens GN=HELZ PE=1 SV=2//0
XM_008012043.1	-1.2777	2.81E-07	3.61E-06	sp|Q95LB0|APOH_PANTR Beta-2-glycoprotein 1 OS=Pan troglodytes GN=APOH PE=2 SV=1//0
XM_008012068.1	0.83624	0.001905	0.013129	sp|Q14344|GNA13_HUMAN Guanine nucleotide-binding protein subunit alpha-13 OS=Homo sapiens GN=GNA13 PE=1 SV=2//0
XM_008012079.1	0.41512	3.78E-09	6.08E-08	sp|P17844|DDX5_HUMAN Probable ATP-dependent RNA helicase DDX5 OS=Homo sapiens GN=DDX5 PE=1 SV=1//0
XM_008012156.1	0.87726	6.92E-17	2.19E-15	sp|Q99759|M3K3_HUMAN Mitogen-activated protein kinase kinase kinase 3 OS=Homo sapiens GN=MAP3K3 PE=1 SV=2//0
XM_008012192.1	-0.68639	9.66E-09	1.47E-07	sp|Q9UBG0|MRC2_HUMAN C-type mannose receptor 2 OS=Homo sapiens GN=MRC2 PE=1 SV=2//0
XM_008012205.1	0.42971	0.002439	0.016395	sp|Q6P1Q9|MET2B_HUMAN Methyltransferase-like protein 2B OS=Homo sapiens GN=METTL2B PE=1 SV=3//0
XM_008012221.1	0.39491	3.07E-05	0.000295	sp|A6NMS7|L37A1_HUMAN Leucine-rich repeat-containing protein 37A OS=Homo sapiens GN=LRRC37A PE=2 SV=3//4.82501e-159
XM_008012228.1	-2.2343	0.000613	0.004735	sp|O14653|GOSR2_HUMAN Golgi SNAP receptor complex member 2 OS=Homo sapiens GN=GOSR2 PE=1 SV=2//3.07112e-116
XM_008012233.1	-1.0613	4.23E-15	1.17E-13	sp|P56703|WNT3_HUMAN Proto-oncogene Wnt-3 OS=Homo sapiens GN=WNT3 PE=1 SV=2//0
XM_008012274.1	1.1997	4.77E-36	4.13E-34	sp|A6NMS7|L37A1_HUMAN Leucine-rich repeat-containing protein 37A OS=Homo sapiens GN=LRRC37A PE=2 SV=3//0
XM_008012309.1	1.6217	1.08E-22	5.03E-21	sp|Q9Y4G2|PKHM1_HUMAN Pleckstrin homology domain-containing family M member 1 OS=Homo sapiens GN=PLEKHM1 PE=1 SV=3//0
XM_008012343.1	-0.25018	0.002578	0.017242	sp|O94992|HEXI1_HUMAN Protein HEXIM1 OS=Homo sapiens GN=HEXIM1 PE=1 SV=1//2.08026e-149
XM_008012364.1	-0.26494	0.002203	0.015019	sp|P30419|NMT1_HUMAN Glycylpeptide N-tetradecanoyltransferase 1 OS=Homo sapiens GN=NMT1 PE=1 SV=2//0
XM_008012393.1	-0.83749	2.27E-06	2.60E-05	sp|Q9NTX9|F217B_HUMAN Protein FAM217B OS=Homo sapiens GN=FAM217B PE=2 SV=1//0
XM_008012398.1	0.69492	8.77E-05	0.000789	sp|Q96MW1|CCD43_HUMAN Coiled-coil domain-containing protein 43 OS=Homo sapiens GN=CCDC43 PE=1 SV=2//1.56519e-99
XM_008012400.1	0.91853	2.45E-14	6.35E-13	sp|Q8NFT6|DBF4B_HUMAN Protein DBF4 homolog B OS=Homo sapiens GN=DBF4B PE=1 SV=1//0
XM_008012403.1	-0.64006	2.11E-11	4.27E-10	sp|Q14332|FZD2_HUMAN Frizzled-2 OS=Homo sapiens GN=FZD2 PE=1 SV=1//0
XM_008012417.1	-0.72941	4.14E-08	5.85E-07	sp|A8MVW0|F1712_HUMAN Protein FAM171A2 OS=Homo sapiens GN=FAM171A2 PE=1 SV=1//0
XM_008012432.1	-1.0367	0.007222	0.043252	sp|P17480|UBF1_HUMAN Nucleolar transcription factor 1 OS=Homo sapiens GN=UBTF PE=1 SV=1//0
XM_008012435.1	-2.9598	0.00051	0.004004	sp|P17480|UBF1_HUMAN Nucleolar transcription factor 1 OS=Homo sapiens GN=UBTF PE=1 SV=1//0
XM_008012476.1	0.38902	5.00E-05	0.000467	sp|Q5RAT5|LSM12_PONAB Protein LSM12 homolog OS=Pongo abelii GN=LSM12 PE=2 SV=1//3.24901e-138
XM_008012479.1	1.2386	0.008178	0.048255	sp|Q5RFN8|TM101_PONAB Transmembrane protein 101 OS=Pongo abelii GN=TMEM101 PE=2 SV=1//3.51969e-145
XM_008012514.1	1.0734	3.17E-43	3.49E-41	sp|Q5RD73|DUS3_PONAB Dual specificity protein phosphatase 3 OS=Pongo abelii GN=DUSP3 PE=2 SV=1//5.37643e-123
XM_008012521.1	1.0645	0.0004	0.003207	sp|P43268|ETV4_HUMAN ETS translocation variant 4 OS=Homo sapiens GN=ETV4 PE=1 SV=3//0
XM_008012523.1	2.0149	1.22E-08	1.83E-07	sp|Q5REU3|ARL4D_PONAB ADP-ribosylation factor-like protein 4D OS=Pongo abelii GN=ARL4D PE=2 SV=1//9.65898e-131
XM_008012542.1	-0.94591	7.29E-06	7.70E-05	sp|Q99536|VAT1_HUMAN Synaptic vesicle membrane protein VAT-1 homolog OS=Homo sapiens GN=VAT1 PE=1 SV=2//0
XM_008012544.1	-1.136	6.32E-06	6.76E-05	sp|P52198|RND2_HUMAN Rho-related GTP-binding protein RhoN OS=Homo sapiens

				GN=RND2 PE=1 SV=2//4.4636e-150
XM_008012552.1	-0.67309	2.53E-09	4.15E-08	sp|P61354|RL27_RAT 60S ribosomal protein L27 OS=Rattus norvegicus GN=Rpl27 PE=2 SV=2//1.25644e-83
XM_008012617.1	-0.77383	4.08E-09	6.53E-08	sp|Q86VR2|F134C_HUMAN Protein FAM134C OS=Homo sapiens GN=FAM134C PE=1 SV=1//0
XM_008012624.1	0.98073	7.45E-08	1.02E-06	sp|Q9P2W1|HOP2_HUMAN Homologous-pairing protein 2 homolog OS=Homo sapiens GN=PSMC3IP PE=1 SV=1//5.96011e-140
XM_008012631.1	-0.61567	1.35E-14	3.61E-13	sp|Q6NZI2|PTRF_HUMAN Polymerase I and transcript release factor OS=Homo sapiens GN=PTRF PE=1 SV=1//0
XM_008012680.1	-1.1535	6.94E-11	1.34E-09	sp|P56381|ATP5E_HUMAN ATP synthase subunit epsilon, mitochondrial OS=Homo sapiens GN=ATP5E PE=1 SV=2//1.05468e-17
XM_008012693.1	-0.35709	0.000976	0.007217	sp|Q969T7|5NT3B_HUMAN 7-methylguanosine phosphate-specific 5'-nucleotidase OS=Homo sapiens GN=NT5C3B PE=1 SV=4//0
XM_008012710.1	0.83719	5.25E-25	2.78E-23	sp|Q5RFF4|EIF1_PONAB Eukaryotic translation initiation factor 1 OS=Pongo abelii GN=EIF1 PE=3 SV=1//5.00883e-60
XM_008012712.1	-0.47962	5.91E-09	9.27E-08	sp|Q9UBR2|CATZ_HUMAN Cathepsin Z OS=Homo sapiens GN=CTSZ PE=1 SV=1//0
XM_008012713.1	-1.4216	7.89E-51	1.20E-48	sp|P08727|K1C19_HUMAN Keratin, type I cytoskeletal 19 OS=Homo sapiens GN=KRT19 PE=1 SV=4//0
XM_008012761.1	1.248	0.00745	0.044399	sp|P35900|K1C20_HUMAN Keratin, type I cytoskeletal 20 OS=Homo sapiens GN=KRT20 PE=1 SV=1//0
XM_008012764.1	1.0755	0.002368	0.015984	sp|Q6A163|K1C39_HUMAN Keratin, type I cytoskeletal 39 OS=Homo sapiens GN=KRT39 PE=1 SV=2//0
XM_008012767.1	2.217	6.84E-05	0.000627	sp|Q9BYR6|KRA33_HUMAN Keratin-associated protein 3-3 OS=Homo sapiens GN=KRTAP3-3 PE=1 SV=1//1.23904e-42
XM_008012777.1	2.1609	3.29E-21	1.38E-19	sp|Q7Z3Y7|K1C28_HUMAN Keratin, type I cytoskeletal 28 OS=Homo sapiens GN=KRT28 PE=1 SV=2//0
XM_008012788.1	-1.2739	0.001993	0.013667	sp|Q8N144|CXD3_HUMAN Gap junction delta-3 protein OS=Homo sapiens GN=GJD3 PE=1 SV=1//2.35992e-132
XM_008012789.1	-0.29237	0.000665	0.005105	sp|P11388|TOP2A_HUMAN DNA topoisomerase 2-alpha OS=Homo sapiens GN=TOP2A PE=1 SV=3//0
XM_008012793.1	1.8178	7.86E-08	1.07E-06	sp|P10276|RARA_HUMAN Retinoic acid receptor alpha OS=Homo sapiens GN=RARA PE=1 SV=2//0
XM_008012802.1	0.30637	0.000586	0.004537	sp|O15234|CASC3_HUMAN Protein CASC3 OS=Homo sapiens GN=CASC3 PE=1 SV=2//0
XM_008012808.1	3.3127	2.78E-57	5.10E-55	sp|P20393|NR1D1_HUMAN Nuclear receptor subfamily 1 group D member 1 OS=Homo sapiens GN=NR1D1 PE=1 SV=1//0
XM_008012844.1	-4.6891	0.000103	0.000909	sp|Q14451|GRB7_HUMAN Growth factor receptor-bound protein 7 OS=Homo sapiens GN=GRB7 PE=1 SV=2//3.51708e-42
XM_008012882.1	-0.677	1.76E-21	7.54E-20	sp|P84100|RL19_RAT 60S ribosomal protein L19 OS=Rattus norvegicus GN=Rpl19 PE=1 SV=1//7.18395e-87
XM_008012898.1	-0.56135	2.86E-07	3.65E-06	sp|P62832|RL23_RAT 60S ribosomal protein L23 OS=Rattus norvegicus GN=Rpl23 PE=2 SV=1//1.7651e-88
XM_008012899.1	-1.8384	0.001123	0.008201	sp|O14662|STX16_HUMAN Syntaxin-16 OS=Homo sapiens GN=STX16 PE=1 SV=3//5.2705e-165
XM_008012900.1	1.1	3.86E-20	1.52E-18	sp|Q9NXE8|CWC25_HUMAN Pre-mRNA-splicing factor CWC25 homolog OS=Homo sapiens GN=CWC25 PE=1 SV=1//0
XM_008012904.1	-0.54338	2.69E-08	3.88E-07	sp|P40112|PSB3_RAT Proteasome subunit beta type-3 OS=Rattus norvegicus GN=Psmb3 PE=1 SV=1//4.23109e-151
XM_008012912.1	-0.44659	0.000646	0.004968	sp|P55198|AF17_HUMAN Protein AF-17 OS=Homo sapiens GN=MLLT6 PE=1 SV=2//1.2571e-34
XM_008012931.1	1.4587	4.71E-42	5.01E-40	sp|Q9P227|RHG23_HUMAN Rho GTPase-activating protein 23 OS=Homo sapiens GN=ARHGAP23 PE=1 SV=2//0
XM_008012940.1	0.85778	3.27E-18	1.14E-16	sp|O14512|SOCS7_HUMAN Suppressor of cytokine signaling 7 OS=Homo sapiens GN=SOCS7 PE=1 SV=2//0
XM_008012949.1	0.41452	1.36E-08	2.04E-07	sp|Q14974|IMB1_HUMAN Importin subunit beta-1 OS=Homo sapiens GN=KPNB1 PE=1 SV=2//0
XM_008013005.1	-0.97968	0.001295	0.009306	sp|P09067|HXB5_HUMAN Homeobox protein Hox-B5 OS=Homo sapiens GN=HOXB5 PE=1 SV=3//2.44991e-123
XM_008013016.1	-0.73467	3.68E-06	4.10E-05	sp|Q9TT89|HXB7_BOVIN Homeobox protein Hox-B7 OS=Bos taurus GN=HOXB7 PE=2 SV=1//4.77542e-125
XM_008013017.1	2.6221	7.07E-18	2.42E-16	sp|P17482|HXB9_HUMAN Homeobox protein Hox-B9 OS=Homo sapiens GN=HOXB9 PE=1 SV=2//1.60541e-154
XM_008013027.1	1.6422	1.06E-16	3.33E-15	sp|Q8N841|TTLL6_HUMAN Tubulin polyglutamylase TTLL6 OS=Homo sapiens GN=TTLL6 PE=1 SV=2//0
XM_008013035.1	-0.55475	6.72E-10	1.17E-08	sp|Q96H20|SNF8_HUMAN Vacuolar-sorting protein SNF8 OS=Homo sapiens GN=SNF8 PE=1 SV=1//8.5717e-174
XM_008013039.1	1.1761	4.06E-12	8.72E-11	sp|Q9NZI8|IF2B1_HUMAN Insulin-like growth factor 2 mRNA-binding protein 1 OS=Homo sapiens GN=IGF2BP1 PE=1 SV=2//0
XM_008013045.1	-0.93875	0.007947	0.047054	sp|Q9P2A4|ABI3_HUMAN ABI gene family member 3 OS=Homo sapiens GN=ABI3 PE=1 SV=2//0
XM_008013059.1	-0.54587	3.84E-05	0.000364	sp|Q9Y2D9|ZN652_HUMAN Zinc finger protein 652 OS=Homo sapiens GN=ZNF652

				PE=1 SV=3//0
XM_008013080.1	-1.4903	6.40E-06	6.83E-05	sp|Q63HM1|KFA_HUMAN Kynurenine formamidase OS=Homo sapiens GN=AFMID PE=2 SV=2//0
XM_008013083.1	-0.6701	7.63E-07	9.36E-06	sp|Q5RAH9|BIRC5_PONAB Baculoviral IAP repeat-containing protein 5 OS=Pongo abelii GN=BIRC5 PE=2 SV=1//1.3512e-75
XM_008013093.1	0.91698	6.39E-08	8.81E-07	sp|O14543|SOCS3_HUMAN Suppressor of cytokine signaling 3 OS=Homo sapiens GN=SOCS3 PE=1 SV=1//1.87632e-129
XM_008013121.1	-0.63697	2.33E-19	8.71E-18	sp|P16035|TIMP2_HUMAN Metalloproteinase inhibitor 2 OS=Homo sapiens GN=TIMP2 PE=1 SV=2//3.26496e-94
XM_008013156.1	-0.51484	0.000221	0.001851	sp|Q14781|CBX2_HUMAN Chromobox protein homolog 2 OS=Homo sapiens GN=CBX2 PE=1 SV=2//0
XM_008013159.1	1.0727	5.38E-20	2.10E-18	sp|O00257|CBX4_HUMAN E3 SUMO-protein ligase CBX4 OS=Homo sapiens GN=CBX4 PE=1 SV=3//0
XM_008013160.1	-0.73013	0.003298	0.021542	-//-
XM_008013224.1	-1.5942	0.000106	0.000935	sp|Q5R5J1|PCKGC_PONAB Phosphoenolpyruvate carboxykinase, cytosolic [GTP] OS=Pongo abelii GN=PCK1 PE=2 SV=1//0
XM_008013242.1	-0.49792	7.92E-13	1.82E-11	sp|A2BDB0|ACTG_XENLA Actin, cytoplasmic 2 OS=Xenopus laevis GN=actg1 PE=2 SV=1//0
XM_008013258.1	0.24902	0.006945	0.041799	sp|O14964|HGS_HUMAN Hepatocyte growth factor-regulated tyrosine kinase substrate OS=Homo sapiens GN=HGS PE=1 SV=1//0
XM_008013267.1	-2.1333	5.73E-08	7.93E-07	sp|C9JLW8|F195B_HUMAN Protein FAM195B OS=Homo sapiens GN=FAM195B PE=1 SV=1//3.56049e-50
XM_008013291.1	1.9184	2.04E-17	6.78E-16	sp|Q9NRC8|SIR7_HUMAN NAD-dependent protein deacetylase sirtuin-7 OS=Homo sapiens GN=SIRT7 PE=1 SV=1//0
XM_008013299.1	-0.86011	1.64E-08	2.43E-07	sp|Q6P988|NOTUM_HUMAN Palmitoleoyl-protein carboxylesterase NOTUM OS=Homo sapiens GN=NOTUM PE=1 SV=2//0
XM_008013318.1	-0.44396	0.000625	0.004819	sp|Q7Z4W1|DCXR_HUMAN L-xylulose reductase OS=Homo sapiens GN=DCXR PE=1 SV=2//2.89878e-157
XM_008013339.1	0.74291	5.49E-11	1.08E-09	sp|Q5RF99|RAEL1_PONAB mRNA export factor OS=Pongo abelii GN=RAE1 PE=2 SV=1//0
XM_008013373.1	-0.63122	0.005797	0.035739	sp|Q6PK18|OGFD3_HUMAN 2-oxoglutarate and iron-dependent oxygenase domain-containing protein 3 OS=Homo sapiens GN=OGFOD3 PE=1 SV=2//0
XM_008013376.1	0.73794	6.24E-17	1.99E-15	sp|Q01167|FOXK2_HUMAN Forkhead box protein K2 OS=Homo sapiens GN=FOXK2 PE=1 SV=3//0
XM_008013394.1	1.1413	5.31E-20	2.08E-18	sp|Q641Q3|METRL_HUMAN Meteorin-like protein OS=Homo sapiens GN=METRNL PE=2 SV=1//0
XM_008013401.1	1.6689	4.66E-26	2.60E-24	sp|Q9P0J7|KCMF1_HUMAN E3 ubiquitin-protein ligase KCMF1 OS=Homo sapiens GN=KCMF1 PE=1 SV=2//3.10082e-138
XM_008013411.1	0.74834	8.28E-11	1.59E-09	sp|Q2HJF8|MIRO1_BOVIN Mitochondrial Rho GTPase 1 OS=Bos taurus GN=RHOT1 PE=2 SV=1//3.03789e-13
XM_008013416.1	0.30167	0.003243	0.021218	sp|Q96H55|MYO19_HUMAN Unconventional myosin-XIX OS=Homo sapiens GN=MYO19 PE=2 SV=2//0
XM_008013431.1	0.8911	8.48E-23	3.97E-21	sp|Q9HAU4|SMUF2_HUMAN E3 ubiquitin-protein ligase SMURF2 OS=Homo sapiens GN=SMURF2 PE=1 SV=1//0
XM_008013433.1	0.40854	5.41E-07	6.74E-06	sp|Q86XP3|DDX42_HUMAN ATP-dependent RNA helicase DDX42 OS=Homo sapiens GN=DDX42 PE=1 SV=1//0
XM_008013442.1	0.64112	1.61E-09	2.68E-08	sp|Q6J6I9|BRCA1_MACMU Breast cancer type 1 susceptibility protein homolog OS=Macaca mulatta GN=BRCA1 PE=3 SV=1//1.27016e-163
XM_008013444.1	1.5397	2.92E-20	1.16E-18	sp|Q96NG3|TTC25_HUMAN Tetratricopeptide repeat protein 25 OS=Homo sapiens GN=TTC25 PE=1 SV=2//0
XM_008013445.1	-0.41275	2.20E-08	3.21E-07	sp|P53396|ACLY_HUMAN ATP-citrate synthase OS=Homo sapiens GN=ACLY PE=1 SV=3//0
XM_008013449.1	-2.0467	0.000323	0.00263	-//-
XM_008013451.1	1.2982	0.001144	0.00833	sp|Q8IZW8|TENS4_HUMAN Tensin-4 OS=Homo sapiens GN=TNS4 PE=1 SV=3//0
XM_008013452.1	0.61581	2.45E-07	3.17E-06	sp|Q8TF74|WIPF2_HUMAN WAS/WASL-interacting protein family member 2 OS=Homo sapiens GN=WIPF2 PE=1 SV=1//1.03326e-162
XM_008013454.1	0.90819	1.11E-28	7.07E-27	sp|Q15648|MED1_HUMAN Mediator of RNA polymerase II transcription subunit 1 OS=Homo sapiens GN=MED1 PE=1 SV=4//2.51624e-154
XM_008013455.1	1.5869	1.21E-06	1.45E-05	sp|A6NH57|ARL5C_HUMAN Putative ADP-ribosylation factor-like protein 5C OS=Homo sapiens GN=ARL5C PE=3 SV=4//5.53017e-08
XM_008013459.1	1.6204	6.71E-25	3.52E-23	sp|Q86WA9|S2611_HUMAN Sodium-independent sulfate anion transporter OS=Homo sapiens GN=SLC26A11 PE=2 SV=2//9.35119e-26
XM_008013463.1	0.5544	0.000381	0.00306	sp|Q6PK04|CC137_HUMAN Coiled-coil domain-containing protein 137 OS=Homo sapiens GN=CCDC137 PE=1 SV=1//3.7335e-131
XM_008013466.1	-0.4671	0.003013	0.019842	sp|Q9ER35|FN3K_MOUSE Fructosamine-3-kinase OS=Mus musculus GN=Fn3k PE=2 SV=1//1.99895e-139
XM_008013469.1	-0.39846	1.17E-06	1.40E-05	sp|Q9NV96|CC50A_HUMAN Cell cycle control protein 50A OS=Homo sapiens GN=TMEM30A PE=1 SV=1//0
XM_008013480.1	-0.72623	0.001223	0.008841	sp|Q9NRA2|S17A5_HUMAN Sialin OS=Homo sapiens GN=SLC17A5 PE=1 SV=2//0

XM_008013563.1	-0.72632	2.29E-16	6.94E-15	sp|Q96KR6|F210B_HUMAN Protein FAM210B OS=Homo sapiens GN=FAM210B PE=1 SV=2//3.15706e-60
XM_008013601.1	-0.32077	0.003577	0.023153	sp|Q4R5E3|LMBD1_MACFA Probable lysosomal cobalamin transporter OS=Macaca fascicularis GN=LMBRD1 PE=2 SV=1//0
XM_008013608.1	-1.1043	0.001313	0.009419	sp|Q5T1H1|EYS_HUMAN Protein eyes shut homolog OS=Homo sapiens GN=EYS PE=1 SV=5//0
XM_008013614.1	0.85479	9.63E-12	2.01E-10	sp|Q78EG7|TP4A1_RAT Protein tyrosine phosphatase type IVA 1 OS=Rattus norvegicus GN=Ptp4a1 PE=1 SV=1//1.24168e-114
XM_008013618.1	0.57229	0.000463	0.003664	sp|Q9NQP4|PFD4_HUMAN Prefoldin subunit 4 OS=Homo sapiens GN=PFDN4 PE=1 SV=1//1.30775e-65
XM_008013626.1	0.73206	0.000802	0.006032	sp|P49643|PRI2_HUMAN DNA primase large subunit OS=Homo sapiens GN=PRIM2 PE=1 SV=2//0
XM_008013635.1	0.66507	9.29E-06	9.65E-05	sp|O95816|BAG2_HUMAN BAG family molecular chaperone regulator 2 OS=Homo sapiens GN=BAG2 PE=1 SV=1//1.99671e-145
XM_008013645.1	0.68824	0.006015	0.036873	sp|Q9Y4E5|ZN451_HUMAN Zinc finger protein 451 OS=Homo sapiens GN=ZNF451 PE=1 SV=2//3.3151e-28
XM_008013646.1	0.61412	0.00166	0.01165	sp|Q9HCI6|K1586_HUMAN Uncharacterized protein KIAA1586 OS=Homo sapiens GN=KIAA1586 PE=2 SV=2//0
XM_008013686.1	0.88366	1.74E-05	0.000174	sp|Q9H8W2|CF155_HUMAN Putative uncharacterized protein encoded by LINC00472 OS=Homo sapiens GN=LINC00472 PE=5 SV=2//6.42329e-08
XM_008013688.1	-0.73277	0.000123	0.001077	sp|Q9BYG4|PAR6G_HUMAN Partitioning defective 6 homolog gamma OS=Homo sapiens GN=PARD6G PE=1 SV=1//0
XM_008013701.1	-0.63544	1.10E-05	0.000113	sp|C9JCN9|HSBPL_HUMAN Heat shock factor-binding protein 1-like protein 1 OS=Homo sapiens GN=HSBP1L1 PE=3 SV=2//3.60871e-42
XM_008013780.1	-0.54312	6.18E-06	6.63E-05	sp|P00167|CYB5_HUMAN Cytochrome b5 OS=Homo sapiens GN=CYB5A PE=1 SV=2//4.97149e-89
XM_008013818.1	-0.65489	0.001669	0.011691	sp|Q6PKX4|DOK6_HUMAN Docking protein 6 OS=Homo sapiens GN=DOK6 PE=1 SV=1//0
XM_008013864.1	0.63062	1.39E-09	2.34E-08	sp|O75351|VPS4B_HUMAN Vacuolar protein sorting-associated protein 4B OS=Homo sapiens GN=VPS4B PE=1 SV=2//0
XM_008013868.1	2.1253	4.63E-73	1.28E-70	sp|O60346|PHLP1_HUMAN PH domain leucine-rich repeat-containing protein phosphatase 1 OS=Homo sapiens GN=PHLPP1 PE=1 SV=3//5.15334e-32
XM_008013869.1	1.5308	1.25E-34	1.03E-32	sp|Q9C0B9|ZCHC2_HUMAN Zinc finger CCHC domain-containing protein 2 OS=Homo sapiens GN=ZCCHC2 PE=1 SV=6//0
XM_008013871.1	-1.5677	0.000687	0.005253	sp|Q9Y6Q6|TNR11_HUMAN Tumor necrosis factor receptor superfamily member 11A OS=Homo sapiens GN=TNFRSF11A PE=1 SV=1//0
XM_008013902.1	2.648	0.001307	0.009384	sp|Q13794|APR_HUMAN Phorbol-12-myristate-13-acetate-induced protein 1 OS=Homo sapiens GN=PMAIP1 PE=1 SV=1//1.672e-14
XM_008013903.1	3.6993	1.45E-92	5.88E-90	sp|Q13794|APR_HUMAN Phorbol-12-myristate-13-acetate-induced protein 1 OS=Homo sapiens GN=PMAIP1 PE=1 SV=1//9.88843e-26
XM_008013913.1	-1.0404	8.49E-20	3.29E-18	sp|Q9TU32|LMAN1_CHLAE Protein ERGIC-53 OS=Chlorocebus aethiops GN=LMAN1 PE=2 SV=1//0
XM_008013927.1	0.63679	0.000113	0.000997	sp|Q5RC30|SC11C_PONAB Signal peptidase complex catalytic subunit SEC11C OS=Pongo abelii GN=SEC11C PE=2 SV=3//1.4951e-135
XM_008013953.1	0.78592	1.80E-08	2.66E-07	sp|Q86TB3|ALPK2_HUMAN Alpha-protein kinase 2 OS=Homo sapiens GN=ALPK2 PE=2 SV=3//0
XM_008013965.1	1.144	0.003507	0.022741	sp|Q96PU5|NED4L_HUMAN E3 ubiquitin-protein ligase NEDD4-like OS=Homo sapiens GN=NEDD4L PE=1 SV=2//0
XM_008013980.1	0.60108	7.55E-13	1.74E-11	sp|Q4R4Z1|SYNC_MACFA Asparagine--tRNA ligase, cytoplasmic OS=Macaca fascicularis GN=NARS PE=2 SV=1//0
XM_008014031.1	2.2022	3.38E-11	6.74E-10	sp|Q86WA9|S2611_HUMAN Sodium-independent sulfate anion transporter OS=Homo sapiens GN=SLC26A11 PE=2 SV=2//0
XM_008014048.1	-0.96208	0.00424	0.027004	sp|Q6ZQN7|SO4C1_HUMAN Solute carrier organic anion transporter family member 4C1 OS=Homo sapiens GN=SLCO4C1 PE=1 SV=1//0
XM_008014079.1	1.3468	1.21E-11	2.50E-10	sp|Q4R6I1|GIN1_MACFA Gypsy retrotransposon integrase-like protein 1 OS=Macaca fascicularis GN=GIN1 PE=2 SV=1//0
XM_008014080.1	1.3094	1.73E-05	0.000172	sp|Q96GV9|CE030_HUMAN UNC119-binding protein C5orf30 OS=Homo sapiens GN=C5orf30 PE=1 SV=1//3.35537e-139
XM_008014082.1	0.81234	3.33E-08	4.75E-07	sp|O95396|MOCS3_HUMAN Adenylyltransferase and sulfurtransferase MOCS3 OS=Homo sapiens GN=MOCS3 PE=1 SV=1//0
XM_008014090.1	0.32552	0.001475	0.010492	sp|O60762|DPM1_HUMAN Dolichol-phosphate mannosyltransferase subunit 1 OS=Homo sapiens GN=DPM1 PE=1 SV=1//2.42824e-177
XM_008014094.1	#NAME?	0.000703	0.005363	sp|Q9UF56|FXL17_HUMAN F-box/LRR-repeat protein 17 OS=Homo sapiens GN=FBXL17 PE=2 SV=3//0
XM_008014110.1	1.9701	0.000582	0.004512	sp|C9JQI7|TM232_HUMAN Transmembrane protein 232 OS=Homo sapiens GN=TMEM232 PE=2 SV=2//0
XM_008014114.1	0.35174	0.000318	0.002598	sp|Q96AG3|S2546_HUMAN Solute carrier family 25 member 46 OS=Homo sapiens GN=SLC25A46 PE=1 SV=1//0
XM_008014117.1	0.91554	4.45E-22	1.98E-20	sp|Q8NI36|WDR36_HUMAN WD repeat-containing protein 36 OS=Homo sapiens GN=WDR36 PE=1 SV=1//0
XM_008014121.1	-0.79373	3.36E-12	7.32E-11	sp|Q96DR4|STAR4_HUMAN StAR-related lipid transfer protein 4 OS=Homo

				sapiens GN=STARD4 PE=2 SV=1//6.42851e-151
XM_008014149.1	1.4179	6.74E-07	8.33E-06	sp|Q15696|U2AFM_HUMAN U2 small nuclear ribonucleoprotein auxiliary factor 35 kDa subunit-related protein 2 OS=Homo sapiens GN=ZRSR2 PE=1 SV=2//3.78867e-09
XM_008014150.1	-0.39686	9.99E-06	0.000103	sp|Q00765|REEP5_HUMAN Receptor expression-enhancing protein 5 OS=Homo sapiens GN=REEP5 PE=1 SV=3//2.08356e-127
XM_008014176.1	0.62881	4.89E-05	0.000458	sp|P53609|PGTB1_HUMAN Geranylgeranyl transferase type-1 subunit beta OS=Homo sapiens GN=PGGT1B PE=1 SV=2//0
XM_008014182.1	0.86671	3.17E-16	9.47E-15	sp|Q96JP0|FEM1C_HUMAN Protein fem-1 homolog C OS=Homo sapiens GN=FEM1C PE=1 SV=1//0
XM_008014183.1	-0.49758	6.69E-09	1.03E-07	sp|Q9Y3B3|TMED7_HUMAN Transmembrane emp24 domain-containing protein 7 OS=Homo sapiens GN=TMED7 PE=1 SV=2//9.34712e-127
XM_008014185.1	2.0955	1.83E-07	2.39E-06	sp|Q5RBQ7|CDO1_PONAB Cysteine dioxygenase type 1 OS=Pongo abelii GN=CDO1 PE=2 SV=1//4.33967e-143
XM_008014186.1	1.3919	1.08E-07	1.45E-06	sp|Q5R7W1|ATG12_PONAB Ubiquitin-like protein ATG12 OS=Pongo abelii GN=ATG12 PE=2 SV=1//3.65785e-70
XM_008014205.1	0.38807	0.001629	0.011459	sp|Q4R7M4|DTWD2_MACFA DTW domain-containing protein 2 OS=Macaca fascicularis GN=DTWD2 PE=2 SV=1//0
XM_008014219.1	-0.28061	0.005316	0.033092	sp|P51659|DHB4_HUMAN Peroxisomal multifunctional enzyme type 2 OS=Homo sapiens GN=HSD17B4 PE=1 SV=3//0
XM_008014233.1	1.3295	2.22E-19	8.33E-18	sp|Q8NEF9|SRFB1_HUMAN Serum response factor-binding protein 1 OS=Homo sapiens GN=SRFBP1 PE=1 SV=1//0
XM_008014238.1	0.8395	1.65E-28	1.04E-26	sp|P18031|PTN1_HUMAN Tyrosine-protein phosphatase non-receptor type 1 OS=Homo sapiens GN=PTPN1 PE=1 SV=1//0
XM_008014250.1	1.0987	0.000387	0.00311	sp|Q9Y343|SNX24_HUMAN Sorting nexin-24 OS=Homo sapiens GN=SNX24 PE=1 SV=1//1.40923e-112
XM_008014253.1	-1.4388	6.34E-28	3.87E-26	sp|P45877|PPIC_HUMAN Peptidyl-prolyl cis-trans isomerase C OS=Homo sapiens GN=PPIC PE=1 SV=1//1.3921e-119
XM_008014278.1	1.8125	1.67E-09	2.77E-08	sp|P17676|CEBPB_HUMAN CCAAT/enhancer-binding protein beta OS=Homo sapiens GN=CEBPB PE=1 SV=2//5.03836e-133
XM_008014289.1	-0.32603	7.69E-05	0.0007	sp|P20700|LMNB1_HUMAN Lamin-B1 OS=Homo sapiens GN=LMNB1 PE=1 SV=2//0
XM_008014300.1	-0.46331	3.40E-06	3.81E-05	sp|Q96M27|PRRC1_HUMAN Protein PRRC1 OS=Homo sapiens GN=PRRC1 PE=1 SV=1//0
XM_008014309.1	-0.63907	0.000129	0.001125	sp|P35556|FBN2_HUMAN Fibrillin-2 OS=Homo sapiens GN=FBN2 PE=1 SV=3//0
XM_008014345.1	#NAME?	0.003965	0.025454	sp|Q8TEU7|RPGF6_HUMAN Rap guanine nucleotide exchange factor 6 OS=Homo sapiens GN=RAPGEF6 PE=1 SV=2//0
XM_008014362.1	0.9706	4.68E-07	5.87E-06	sp|Q92878|RAD50_HUMAN DNA repair protein RAD50 OS=Homo sapiens GN=RAD50 PE=1 SV=1//0
XM_008014364.1	0.69432	1.99E-14	5.21E-13	sp|Q9Y508|RN114_HUMAN E3 ubiquitin-protein ligase RNF114 OS=Homo sapiens GN=RNF114 PE=1 SV=1//1.36446e-145
XM_008014372.1	0.44315	0.002818	0.018647	sp|O76082|S22A5_HUMAN Solute carrier family 22 member 5 OS=Homo sapiens GN=SLC22A5 PE=1 SV=1//0
XM_008014378.1	-2.0855	0.00346	0.022497	sp|Q4R628|KIF3A_MACFA Kinesin-like protein KIF3A OS=Macaca fascicularis GN=KIF3A PE=2 SV=1//1.18565e-139
XM_008014415.1	-0.57159	4.43E-10	7.88E-09	sp|Q5R5B8|KCT2_PONAB Keratinocyte-associated transmembrane protein 2 OS=Pongo abelii GN=KCT2 PE=2 SV=1//4.28408e-160
XM_008014437.1	-0.78059	1.89E-20	7.63E-19	sp|Q71U00|SKP1_XENLA S-phase kinase-associated protein 1 OS=Xenopus laevis GN=skp1 PE=1 SV=3//6.6513e-84
XM_008014438.1	0.43757	3.20E-05	0.000307	sp|P67777|PP2AA_RABIT Serine/threonine-protein phosphatase 2A catalytic subunit alpha isoform OS=Oryctolagus cuniculus GN=PPP2CA PE=2 SV=1//0
XM_008014462.1	0.33128	0.000788	0.005946	sp|O43286|B4GT5_HUMAN Beta-1,4-galactosyltransferase 5 OS=Homo sapiens GN=B4GALT5 PE=2 SV=1//0
XM_008014469.1	0.30609	0.006291	0.038428	sp|O95486|SC24A_HUMAN Protein transport protein Sec24A OS=Homo sapiens GN=SEC24A PE=1 SV=2//4.05196e-172
XM_008014483.1	-2.2948	1.14E-05	0.000117	sp|Q86XQ3|CTSR3_HUMAN Cation channel sperm-associated protein 3 OS=Homo sapiens GN=CATSPER3 PE=1 SV=1//0
XM_008014525.1	0.9474	6.72E-25	3.52E-23	sp|Q96GQ7|DDX27_HUMAN Probable ATP-dependent RNA helicase DDX27 OS=Homo sapiens GN=DDX27 PE=1 SV=2//0
XM_008014532.1	-3.0937	2.98E-06	3.36E-05	sp|Q6ZT89|S2548_HUMAN Solute carrier family 25 member 48 OS=Homo sapiens GN=SLC25A48 PE=1 SV=2//0
XM_008014538.1	0.54008	1.72E-09	2.85E-08	sp|Q5R6H7|SMAD5_PONAB Mothers against decapentaplegic homolog 5 OS=Pongo abelii GN=SMAD5 PE=2 SV=1//0
XM_008014543.1	1.5797	5.89E-22	2.60E-20	sp|Q9HCX4|TRPC7_HUMAN Short transient receptor potential channel 7 OS=Homo sapiens GN=TRPC7 PE=1 SV=1//0
XM_008014562.1	1.1771	1.39E-60	2.88E-58	sp|Q3ZCH0|GRP75_BOVIN Stress-70 protein, mitochondrial OS=Bos taurus GN=HSPA9 PE=2 SV=1//0
XM_008014563.1	0.63701	5.50E-17	1.77E-15	sp|Q5U2Q7|ERF1_RAT Eukaryotic peptide chain release factor subunit 1 OS=Rattus norvegicus GN=Etf1 PE=2 SV=3//0
XM_008014564.1	4.7157	2.64E-24	1.35E-22	sp|P18146|EGR1_HUMAN Early growth response protein 1 OS=Homo sapiens GN=EGR1 PE=1 SV=1//0
XM_008014582.1	-0.31978	0.000136	0.00118	sp|O95235|KI20A_HUMAN Kinesin-like protein KIF20A OS=Homo sapiens

				GN=KIF20A PE=1 SV=1//0
XM_008014624.1	-0.91184	3.47E-23	1.69E-21	sp|Q9BPZ3|PAIP2_HUMAN Polyadenylate-binding protein-interacting protein 2 OS=Homo sapiens GN=PAIP2 PE=1 SV=1//5.69572e-50
XM_008014637.1	0.46281	0.007888	0.046732	sp|P62840|UB2D2_XENLA Ubiquitin-conjugating enzyme E2 D2 OS=Xenopus laevis GN=ube2d2 PE=1 SV=1//6.716e-99
XM_008014656.1	-0.78583	3.20E-05	0.000307	sp|A6NJ69|IGIP_HUMAN IgA-inducing protein homolog OS=Homo sapiens GN=IGIP PE=3 SV=1//5.29136e-26
XM_008014669.1	3.4157	########	########	sp|Q09118|HBEGF_CHLAE Proheparin-binding EGF-like growth factor OS=Chlorocebus aethiops GN=HBEGF PE=1 SV=1//3.88941e-95
XM_008014693.1	-0.37316	5.78E-06	6.23E-05	sp|Q66HG8|RED_RAT Protein Red OS=Rattus norvegicus GN=Ik PE=1 SV=1//0
XM_008014694.1	-0.92297	1.77E-10	3.30E-09	sp|Q4R5E2|NDUA2_MACFA NADH dehydrogenase [ubiquinone] 1 alpha subcomplex subunit 2 OS=Macaca fascicularis GN=NDUFA2 PE=3 SV=3//5.94116e-62
XM_008014703.1	-0.4166	0.003766	0.024296	sp|Q5R9T6|WDR55_PONAB WD repeat-containing protein 55 OS=Pongo abelii GN=WDR55 PE=2 SV=1//0
XM_008014704.1	-0.49575	1.28E-06	1.52E-05	sp|Q9CPW7|ZMAT2_MOUSE Zinc finger matrin-type protein 2 OS=Mus musculus GN=Zmat2 PE=2 SV=1//1.71339e-111
XM_008014729.1	0.29085	0.000472	0.003729	sp|Q5R7L9|TAF7_PONAB Transcription initiation factor TFIID subunit 7 OS=Pongo abelii GN=TAF7 PE=2 SV=1//0
XM_008014784.1	-0.51856	2.75E-08	3.96E-07	sp|Q9BT67|NFIP1_HUMAN NEDD4 family-interacting protein 1 OS=Homo sapiens GN=NDFIP1 PE=1 SV=1//1.52675e-116
XM_008014912.1	#NAME?	0.003893	0.025054	sp|P58062|ISK7_HUMAN Serine protease inhibitor Kazal-type 7 OS=Homo sapiens GN=SPINK7 PE=1 SV=1//1.9515e-43
XM_008014922.1	3.082	1.08E-12	2.44E-11	sp|Q28509|ADRB2_MACMU Beta-2 adrenergic receptor OS=Macaca mulatta GN=ADRB2 PE=2 SV=1//0
XM_008014923.1	0.81304	9.91E-06	0.000102	sp|Q8TF17|S3TC2_HUMAN SH3 domain and tetratricopeptide repeat-containing protein 2 OS=Homo sapiens GN=SH3TC2 PE=1 SV=2//0
XM_008014933.1	1.2898	0.00079	0.005957	sp|Q8TAA5|GRPE2_HUMAN GrpE protein homolog 2, mitochondrial OS=Homo sapiens GN=GRPEL2 PE=1 SV=1//2.08672e-153
XM_008014957.1	-0.67244	2.97E-20	1.18E-18	sp|P50443|S26A2_HUMAN Sulfate transporter OS=Homo sapiens GN=SLC26A2 PE=1 SV=2//0
XM_008014981.1	1.4184	4.26E-16	1.26E-14	sp|Q5FYB1|ARSI_HUMAN Arylsulfatase I OS=Homo sapiens GN=ARSI PE=1 SV=1//0
XM_008015007.1	0.98822	5.36E-23	2.54E-21	sp|P52848|NDST1_HUMAN Bifunctional heparan sulfate N-deacetylase/N-sulfotransferase 1 OS=Homo sapiens GN=NDST1 PE=1 SV=1//0
XM_008015013.1	1.2419	1.07E-19	4.13E-18	sp|Q8N3V7|SYNPO_HUMAN Synaptopodin OS=Homo sapiens GN=SYNPO PE=1 SV=2//0
XM_008015014.1	2.4828	0.002486	0.016669	sp|Q8N3V7|SYNPO_HUMAN Synaptopodin OS=Homo sapiens GN=SYNPO PE=1 SV=2//0
XM_008015020.1	0.67501	1.20E-12	2.71E-11	sp|Q8BHS3|RBM22_MOUSE Pre-mRNA-splicing factor RBM22 OS=Mus musculus GN=Rbm22 PE=1 SV=1//0
XM_008015039.1	-0.63987	3.63E-10	6.49E-09	sp|P08133|ANXA6_HUMAN Annexin A6 OS=Homo sapiens GN=ANXA6 PE=1 SV=3//0
XM_008015041.1	-1.0991	8.75E-08	1.19E-06	sp|A6NI79|CCD69_HUMAN Coiled-coil domain-containing protein 69 OS=Homo sapiens GN=CCDC69 PE=1 SV=1//1.38314e-147
XM_008015060.1	-0.70439	2.03E-23	9.91E-22	sp|Q5R767|SPRC_PONAB SPARC OS=Pongo abelii GN=SPARC PE=2 SV=1//0
XM_008015061.1	1.3999	0.001954	0.013431	-//-
XM_008015096.1	0.3808	0.008127	0.047979	sp|Q6PKG0|LARP1_HUMAN La-related protein 1 OS=Homo sapiens GN=LARP1 PE=1 SV=2//0
XM_008015132.1	-0.37945	2.98E-05	0.000288	sp|O46598|HAVR1_CHLAE Hepatitis A virus cellular receptor 1 OS=Chlorocebus aethiops GN=HAVCR1 PE=1 SV=2//5.58668e-81
XM_008015144.1	0.90432	3.70E-11	7.35E-10	sp|Q96S44|PRPK_HUMAN TP53-regulating kinase OS=Homo sapiens GN=TP53RK PE=1 SV=2//2.38577e-161
XM_008015145.1	-0.91223	0.002664	0.017729	sp|Q9H013|ADA19_HUMAN Disintegrin and metalloproteinase domain-containing protein 19 OS=Homo sapiens GN=ADAM19 PE=1 SV=3//0
XM_008015168.1	1.0918	0.000917	0.006806	sp|Q96MT1|RN145_HUMAN RING finger protein 145 OS=Homo sapiens GN=RNF145 PE=2 SV=2//0
XM_008015173.1	0.55396	7.73E-07	9.46E-06	sp|Q8WVY7|UBCP1_HUMAN Ubiquitin-like domain-containing CTD phosphatase 1 OS=Homo sapiens GN=UBLCP1 PE=1 SV=2//0
XM_008015187.1	2.6038	1.90E-08	2.80E-07	sp|P35368|ADA1B_HUMAN Alpha-1B adrenergic receptor OS=Homo sapiens GN=ADRA1B PE=1 SV=3//0
XM_008015257.1	-0.31557	0.000246	0.002045	sp|Q9H999|PANK3_HUMAN Pantothenate kinase 3 OS=Homo sapiens GN=PANK3 PE=1 SV=1//0
XM_008015258.1	-1.1564	3.83E-23	1.85E-21	sp|O75094|SLIT3_HUMAN Slit homolog 3 protein OS=Homo sapiens GN=SLIT3 PE=2 SV=3//0
XM_008015284.1	1.2738	0.005894	0.036212	sp|Q9H2T7|RBP17_HUMAN Ran-binding protein 17 OS=Homo sapiens GN=RANBP17 PE=2 SV=1//0
XM_008015287.1	0.33927	0.000795	0.005984	sp|P06748|NPM_HUMAN Nucleophosmin OS=Homo sapiens GN=NPM1 PE=1 SV=2//1.48931e-169
XM_008015289.1	1.5513	3.33E-27	1.97E-25	sp|O76093|FGF18_HUMAN Fibroblast growth factor 18 OS=Homo sapiens GN=FGF18 PE=1 SV=1//3.15907e-117
XM_008015300.1	-0.3772	0.001464	0.010423	sp|Q8WUN7|UBTD2_HUMAN Ubiquitin domain-containing protein 2 OS=Homo sapiens GN=UBTD2 PE=1 SV=2//2.30813e-164

XM_008015303.1	-0.67955	1.81E-10	3.36E-09	sp|A8MQ27|NEU1B_HUMAN E3 ubiquitin-protein ligase NEURL1B OS=Homo sapiens GN=NEURL1B PE=1 SV=1//0
XM_008015304.1	6.1474	0	0	sp|P28562|DUS1_HUMAN Dual specificity protein phosphatase 1 OS=Homo sapiens GN=DUSP1 PE=1 SV=3//0
XM_008015308.1	-0.89535	2.25E-09	3.70E-08	sp|Q9UNX3|RL26L_HUMAN 60S ribosomal protein L26-like 1 OS=Homo sapiens GN=RPL26L1 PE=1 SV=1//3.29968e-82
XM_008015309.1	-0.45998	2.43E-06	2.77E-05	sp|Q5RAV0|VA0E1_PONAB V-type proton ATPase subunit e 1 OS=Pongo abelii GN=ATP6V0E1 PE=3 SV=3//6.55776e-48
XM_008015319.1	1.3009	1.40E-13	3.46E-12	sp|O97561|STC2_MACNE Stanniocalcin-2 OS=Macaca nemestrina GN=STC2 PE=2 SV=1//0
XM_008015357.1	0.48043	1.05E-05	0.000108	sp|Q96J01|THOC3_HUMAN THO complex subunit 3 OS=Homo sapiens GN=THOC3 PE=1 SV=1//0
XM_008015369.1	-0.79037	5.16E-11	1.01E-09	sp|Q9BW72|HIG2A_HUMAN HIG1 domain family member 2A, mitochondrial OS=Homo sapiens GN=HIGD2A PE=1 SV=1//1.56289e-54
XM_008015370.1	0.48715	0.000253	0.002098	sp|Q9Y3C1|NOP16_HUMAN Nucleolar protein 16 OS=Homo sapiens GN=NOP16 PE=1 SV=2//5.40385e-108
XM_008015421.1	-0.42473	1.26E-06	1.50E-05	sp|Q12907|LMAN2_HUMAN Vesicular integral-membrane protein VIP36 OS=Homo sapiens GN=LMAN2 PE=1 SV=1//0
XM_008015439.1	-0.55315	1.20E-07	1.60E-06	sp|Q9UJV9|DDX41_HUMAN Probable ATP-dependent RNA helicase DDX41 OS=Homo sapiens GN=DDX41 PE=1 SV=2//0
XM_008015449.1	-2.0225	2.66E-06	3.02E-05	sp|Q9NR12|PDLI7_HUMAN PDZ and LIM domain protein 7 OS=Homo sapiens GN=PDLIM7 PE=1 SV=1//1.73242e-116
XM_008015465.1	-0.53875	4.18E-06	4.61E-05	sp|Q9BVK6|TMED9_HUMAN Transmembrane emp24 domain-containing protein 9 OS=Homo sapiens GN=TMED9 PE=1 SV=2//1.97553e-148
XM_008015470.1	0.86275	0.007194	0.04313	sp|O15049|N4BP3_HUMAN NEDD4-binding protein 3 OS=Homo sapiens GN=N4BP3 PE=1 SV=3//0
XM_008015471.1	-0.77753	1.16E-10	2.20E-09	sp|Q9NX24|NHP2_HUMAN H/ACA ribonucleoprotein complex subunit 2 OS=Homo sapiens GN=NHP2 PE=1 SV=1//4.89757e-103
XM_008015485.1	0.30036	0.002372	0.01601	sp|Q99729|ROAA_HUMAN Heterogeneous nuclear ribonucleoprotein A/B OS=Homo sapiens GN=HNRNPAB PE=1 SV=2//8.48234e-07
XM_008015486.1	0.29433	0.000745	0.005651	sp|Q99729|ROAA_HUMAN Heterogeneous nuclear ribonucleoprotein A/B OS=Homo sapiens GN=HNRNPAB PE=1 SV=2//6.2365e-18
XM_008015492.1	0.53223	3.61E-09	5.82E-08	sp|Q9HCD5|NCOA5_HUMAN Nuclear receptor coactivator 5 OS=Homo sapiens GN=NCOA5 PE=1 SV=2//0
XM_008015515.1	-0.20329	0.006098	0.037328	sp|P31943|HNRH1_HUMAN Heterogeneous nuclear ribonucleoprotein H OS=Homo sapiens GN=HNRNPH1 PE=1 SV=4//0
XM_008015532.1	-0.81529	4.16E-24	2.11E-22	sp|Q9UQ53|MGT4B_HUMAN Alpha-1,3-mannosyl-glycoprotein 4-beta-N-acetylglucosaminyltransferase B OS=Homo sapiens GN=MGAT4B PE=1 SV=1//0
XM_008015549.1	0.59068	0.000148	0.001274	sp|Q9ULM6|CNOT6_HUMAN CCR4-NOT transcription complex subunit 6 OS=Homo sapiens GN=CNOT6 PE=1 SV=2//0
XM_008015584.1	-1.3222	0.006988	0.042039	sp|Q9C029|TRIM7_HUMAN Tripartite motif-containing protein 7 OS=Homo sapiens GN=TRIM7 PE=1 SV=2//0
XM_008015592.1	-0.39412	2.46E-08	3.56E-07	sp|P63245|GBLP_RAT Guanine nucleotide-binding protein subunit beta-2-like 1 OS=Rattus norvegicus GN=Gnb2l1 PE=1 SV=3//0
XM_008015600.1	-0.74044	3.27E-08	4.67E-07	-//-
XM_008015603.1	-0.6324	6.59E-13	1.54E-11	sp|P49419|AL7A1_HUMAN Alpha-aminoadipic semialdehyde dehydrogenase OS=Homo sapiens GN=ALDH7A1 PE=1 SV=5//0
XM_008015617.1	-0.82231	9.08E-08	1.23E-06	sp|Q9Y5H3|PCDGA_HUMAN Protocadherin gamma-A10 OS=Homo sapiens GN=PCDHGA10 PE=2 SV=1//1.16134e-58
XM_008015627.1	1.421	6.36E-05	0.000586	sp|Q6ZN57|ZFP2_HUMAN Zinc finger protein 2 homolog OS=Homo sapiens GN=ZFP2 PE=1 SV=1//0
XM_008015630.1	-1.3244	0.00038	0.003053	-//-
XM_008015659.1	0.86618	3.05E-14	7.85E-13	sp|Q6PCB6|AB17C_HUMAN Alpha/beta hydrolase domain-containing protein 17C OS=Homo sapiens GN=ABHD17C PE=2 SV=2//0
XM_008015665.1	1.7575	1.28E-08	1.93E-07	sp|Q9HBZ2|ARNT2_HUMAN Aryl hydrocarbon receptor nuclear translocator 2 OS=Homo sapiens GN=ARNT2 PE=1 SV=2//0
XM_008015698.1	-0.50466	0.00255	0.017067	sp|P09668|CATH_HUMAN Pro-cathepsin H OS=Homo sapiens GN=CTSH PE=1 SV=4//0
XM_008015699.1	-0.26308	0.001174	0.008534	sp|P60762|MO4L1_MOUSE Mortality factor 4-like protein 1 OS=Mus musculus GN=Morf4l1 PE=1 SV=2//0
XM_008015704.1	-0.80605	0.000556	0.004327	sp|Q5IS51|ACHA5_PANTR Neuronal acetylcholine receptor subunit alpha-5 OS=Pan troglodytes GN=CHRNA5 PE=2 SV=1//0
XM_008015718.1	-0.52678	1.43E-10	2.70E-09	sp|P48200|IREB2_HUMAN Iron-responsive element-binding protein 2 OS=Homo sapiens GN=IREB2 PE=1 SV=3//0
XM_008015719.1	-1.0151	1.36E-15	3.89E-14	sp|P29762|RABP1_HUMAN Cellular retinoic acid-binding protein 1 OS=Homo sapiens GN=CRABP1 PE=1 SV=2//8.89725e-97
XM_008015728.1	0.50219	2.86E-06	3.24E-05	sp|Q5R678|IDH3A_PONAB Isocitrate dehydrogenase [NAD] subunit alpha, mitochondrial OS=Pongo abelii GN=IDH3A PE=2 SV=1//0
XM_008015729.1	1.1097	3.48E-08	4.96E-07	sp|Q96MP5|ZSWM3_HUMAN Zinc finger SWIM domain-containing protein 3 OS=Homo sapiens GN=ZSWIM3 PE=2 SV=2//0
XM_008015730.1	0.77003	5.81E-16	1.71E-14	sp|Q9UPU7|TBD2B_HUMAN TBC1 domain family member 2B OS=Homo sapiens

				GN=TBC1D2B PE=1 SV=2//0
XM_008015758.1	-0.82209	1.20E-20	4.91E-19	sp|Q5RE11|TSN3_PONAB Tetraspanin-3 OS=Pongo abelii GN=TSPAN3 PE=2 SV=1//4.5436e-165
XM_008015761.1	-0.30173	0.003087	0.020299	sp|Q14257|RCN2_HUMAN Reticulocalbin-2 OS=Homo sapiens GN=RCN2 PE=1 SV=1//0
XM_008015801.1	-0.46692	0.000392	0.003145	sp|Q9H0A8|COMD4_HUMAN COMM domain-containing protein 4 OS=Homo sapiens GN=COMMD4 PE=1 SV=1//2.98515e-127
XM_008015808.1	0.84692	1.68E-17	5.62E-16	sp|Q9H147|TDIF1_HUMAN Deoxynucleotidyltransferase terminal-interacting protein 1 OS=Homo sapiens GN=DNTTIP1 PE=1 SV=2//0
XM_008015814.1	0.30405	0.008375	0.049335	sp|P43378|PTN9_HUMAN Tyrosine-protein phosphatase non-receptor type 9 OS=Homo sapiens GN=PTPN9 PE=1 SV=1//0
XM_008015820.1	1.1044	3.07E-10	5.55E-09	sp|Q9NV31|IMP3_HUMAN U3 small nucleolar ribonucleoprotein protein IMP3 OS=Homo sapiens GN=IMP3 PE=1 SV=1//8.65207e-115
XM_008015850.1	-0.61596	1.10E-07	1.47E-06	sp|Q53CF8|COX5A_MACMU Cytochrome c oxidase subunit 5A, mitochondrial OS=Macaca mulatta GN=COX5A PE=2 SV=1//1.68942e-105
XM_008015952.1	-0.30998	0.00051	0.004001	sp|Q08397|LOXL1_HUMAN Lysyl oxidase homolog 1 OS=Homo sapiens GN=LOXL1 PE=1 SV=2//0
XM_008015987.1	-0.35805	1.15E-05	0.000118	sp|Q969N2|PIGT_HUMAN GPI transamidase component PIG-T OS=Homo sapiens GN=PIGT PE=1 SV=1//0
XM_008015994.1	0.41615	3.31E-06	3.72E-05	sp|Q9Z1K5|ARI1_MOUSE E3 ubiquitin-protein ligase ARIH1 OS=Mus musculus GN=Arih1 PE=1 SV=3//0
XM_008016049.1	0.70622	1.89E-06	2.19E-05	sp|Q6ZMP0|THSD4_HUMAN Thrombospondin type-1 domain-containing protein 4 OS=Homo sapiens GN=THSD4 PE=2 SV=2//0
XM_008016063.1	0.35448	4.82E-06	5.26E-05	sp|P31431|SDC4_HUMAN Syndecan-4 OS=Homo sapiens GN=SDC4 PE=1 SV=2//2.92104e-82
XM_008016081.1	-0.54968	2.57E-07	3.32E-06	sp|P05386|RLA1_HUMAN 60S acidic ribosomal protein P1 OS=Homo sapiens GN=RPLP1 PE=1 SV=1//4.77512e-42
XM_008016093.1	-0.89438	0.005578	0.03459	sp|O94923|GLCE_HUMAN D-glucuronyl C5-epimerase OS=Homo sapiens GN=GLCE PE=1 SV=3//0
XM_008016099.1	-0.64335	2.40E-13	5.80E-12	sp|P39687|AN32A_HUMAN Acidic leucine-rich nuclear phosphoprotein 32 family member A OS=Homo sapiens GN=ANP32A PE=1 SV=1//1.64709e-86
XM_008016125.1	0.43603	1.33E-06	1.58E-05	sp|O75925|PIAS1_HUMAN E3 SUMO-protein ligase PIAS1 OS=Homo sapiens GN=PIAS1 PE=1 SV=2//0
XM_008016160.1	1.2634	6.84E-06	7.26E-05	sp|P84025|SMAD3_RAT Mothers against decapentaplegic homolog 3 OS=Rattus norvegicus GN=Smad3 PE=1 SV=1//0
XM_008016164.1	1.5305	1.25E-59	2.55E-57	sp|O43541|SMAD6_HUMAN Mothers against decapentaplegic homolog 6 OS=Homo sapiens GN=SMAD6 PE=1 SV=2//0
XM_008016165.1	-0.27522	0.000495	0.003895	sp|P36578|RL4_HUMAN 60S ribosomal protein L4 OS=Homo sapiens GN=RPL4 PE=1 SV=5//0
XM_008016166.1	1.0523	8.72E-05	0.000785	sp|Q9H900|ZWILC_HUMAN Protein zwilch homolog OS=Homo sapiens GN=ZWILCH PE=1 SV=2//0
XM_008016180.1	0.92154	7.67E-07	9.40E-06	sp|O75971|SNPC5_HUMAN snRNA-activating protein complex subunit 5 OS=Homo sapiens GN=SNAPC5 PE=1 SV=1//8.03795e-35
XM_008016182.1	-1.5031	0.002776	0.018401	sp|P19957|ELAF_HUMAN Elafin OS=Homo sapiens GN=PI3 PE=1 SV=3//3.88129e-63
XM_008016207.1	-0.65293	6.52E-11	1.26E-09	sp|P62494|RB11A_RAT Ras-related protein Rab-11A OS=Rattus norvegicus GN=Rab11a PE=1 SV=3//2.88308e-159
XM_008016212.1	1.0394	0.00145	0.010336	sp|Q7Z401|MYCPP_HUMAN C-myc promoter-binding protein OS=Homo sapiens GN=DENND4A PE=1 SV=2//0
XM_008016240.1	-2.8916	0.000824	0.006187	sp|Q8IVU1|IGDC3_HUMAN Immunoglobulin superfamily DCC subclass member 3 OS=Homo sapiens GN=IGDCC3 PE=2 SV=2//4.13347e-10
XM_008016247.1	0.56411	0.001376	0.009846	sp|Q8N5Y8|PAR16_HUMAN Mono [ADP-ribose] polymerase PARP16 OS=Homo sapiens GN=PARP16 PE=1 SV=2//0
XM_008016254.1	0.81411	5.78E-09	9.08E-08	sp|Q8N8D1|PDCD7_HUMAN Programmed cell death protein 7 OS=Homo sapiens GN=PDCD7 PE=1 SV=1//2.42749e-138
XM_008016292.1	-0.57267	2.45E-07	3.17E-06	sp|Q5R680|OAZ2_PONAB Ornithine decarboxylase antizyme 2 OS=Pongo abelii GN=OAZ2 PE=2 SV=1//1.58027e-113
XM_008016293.1	0.81731	1.15E-22	5.33E-21	sp|O15014|ZN609_HUMAN Zinc finger protein 609 OS=Homo sapiens GN=ZNF609 PE=1 SV=2//0
XM_008016314.1	-0.89684	0.00496	0.031102	sp|Q4R503|SNX1_MACFA Sorting nexin-1 OS=Macaca fascicularis GN=SNX1 PE=2 SV=1//0
XM_008016315.1	-0.62388	0.006499	0.039542	sp|Q4R503|SNX1_MACFA Sorting nexin-1 OS=Macaca fascicularis GN=SNX1 PE=2 SV=1//0
XM_008016336.1	1.032	5.48E-05	0.000509	sp|Q9Y6I4|UBP3_HUMAN Ubiquitin carboxyl-terminal hydrolase 3 OS=Homo sapiens GN=USP3 PE=1 SV=2//0
XM_008016341.1	0.3306	0.004216	0.026879	sp|Q92930|RAB8B_HUMAN Ras-related protein Rab-8B OS=Homo sapiens GN=RAB8B PE=1 SV=2//3.48311e-135
XM_008016390.1	-0.51188	0.003104	0.020388	sp|Q9H426|RIMS4_HUMAN Regulating synaptic membrane exocytosis protein 4 OS=Homo sapiens GN=RIMS4 PE=1 SV=3//0
XM_008016395.1	-0.5202	1.23E-13	3.04E-12	sp|Q5R5A0|ANXA2_PONAB Annexin A2 OS=Pongo abelii GN=ANXA2 PE=2 SV=1//0
XM_008016424.1	1.7268	########	3.96E-98	sp|Q12965|MYO1E_HUMAN Unconventional myosin-Ie OS=Homo sapiens GN=MYO1E PE=1 SV=2//0

XM_008016425.1	-0.70736	1.86E-14	4.88E-13	sp|Q4R7A8|CCNB2_MACFA G2/mitotic-specific cyclin-B2 OS=Macaca fascicularis GN=CCNB2 PE=2 SV=1//0
XM_008016455.1	-0.4208	5.77E-08	7.97E-07	sp|O14672|ADA10_HUMAN Disintegrin and metalloproteinase domain-containing protein 10 OS=Homo sapiens GN=ADAM10 PE=1 SV=1//0
XM_008016456.1	2.7214	3.02E-05	0.000291	sp|O43315|AQP9_HUMAN Aquaporin-9 OS=Homo sapiens GN=AQP9 PE=2 SV=2//2.88319e-176
XM_008016508.1	0.35131	0.001689	0.011825	sp|Q4R7T8|MNS1_MACFA Meiosis-specific nuclear structural protein 1 OS=Macaca fascicularis GN=MNS1 PE=2 SV=2//0
XM_008016512.1	0.90137	0.000416	0.00332	sp|P46934|NEDD4_HUMAN E3 ubiquitin-protein ligase NEDD4 OS=Homo sapiens GN=NEDD4 PE=1 SV=4//0
XM_008016541.1	1.6183	7.14E-05	0.000652	sp|Q8NB66|UN13C_HUMAN Protein unc-13 homolog C OS=Homo sapiens GN=UNC13C PE=2 SV=3//0
XM_008016552.1	-0.51895	1.48E-07	1.96E-06	sp|Q4R5H8|RL18_MACFA 60S ribosomal protein L18 OS=Macaca fascicularis GN=RPL18 PE=2 SV=1//7.24982e-131
XM_008016588.1	0.68993	5.68E-06	6.13E-05	sp|Q16659|MK06_HUMAN Mitogen-activated protein kinase 6 OS=Homo sapiens GN=MAPK6 PE=1 SV=1//0
XM_008016589.1	-1.4529	0.000794	0.005979	sp|Q5R4D6|LEO1_PONAB RNA polymerase-associated protein LEO1 OS=Pongo abelii GN=LEO1 PE=2 SV=1//4.17154e-131
XM_008016608.1	-2.1087	0.002651	0.017658	sp|Q8TDJ6|DMXL2_HUMAN DmX-like protein 2 OS=Homo sapiens GN=DMXL2 PE=1 SV=2//0
XM_008016616.1	0.66369	0.003854	0.024814	sp|Q9UPM8|AP4E1_HUMAN AP-4 complex subunit epsilon-1 OS=Homo sapiens GN=AP4E1 PE=1 SV=2//0
XM_008016680.1	-1.0346	4.84E-23	2.31E-21	sp|Q5RDL6|EID1_PONAB EP300-interacting inhibitor of differentiation 1 OS=Pongo abelii GN=EID1 PE=2 SV=1//1.54466e-71
XM_008016692.1	-0.44398	0.001358	0.009725	sp|P35555|FBN1_HUMAN Fibrillin-1 OS=Homo sapiens GN=FBN1 PE=1 SV=3//0
XM_008016693.1	-1.4198	7.08E-05	0.000648	sp|Q71RS6|NCKX5_HUMAN Sodium/potassium/calcium exchanger 5 OS=Homo sapiens GN=SLC24A5 PE=1 SV=1//0
XM_008016787.1	1.1288	2.29E-06	2.62E-05	sp|Q05D32|CTSL2_HUMAN CTD small phosphatase-like protein 2 OS=Homo sapiens GN=CTDSPL2 PE=1 SV=2//8.04233e-07
XM_008016820.1	-0.50154	2.20E-10	4.03E-09	sp|Q4VIT4|PDIA3_CHLAE Protein disulfide-isomerase A3 OS=Chlorocebus aethiops GN=PDIA3 PE=2 SV=1//0
XM_008016852.1	1.5969	3.52E-10	6.31E-09	sp|O60294|TYW4_HUMAN tRNA wybutosine-synthesizing protein 4 OS=Homo sapiens GN=LCMT2 PE=1 SV=3//0
XM_008016927.1	0.92898	9.38E-14	2.34E-12	sp|Q13158|FADD_HUMAN FAS-associated death domain protein OS=Homo sapiens GN=FADD PE=1 SV=1//9.51e-87
XM_008016928.1	1.5471	1.69E-71	4.47E-69	sp|Q9H223|EHD4_HUMAN EH domain-containing protein 4 OS=Homo sapiens GN=EHD4 PE=1 SV=1//0
XM_008016933.1	-3.4551	9.62E-05	0.000858	sp|Q9HBY8|SGK2_HUMAN Serine/threonine-protein kinase Sgk2 OS=Homo sapiens GN=SGK2 PE=1 SV=1//0
XM_008016944.1	-0.63832	1.96E-10	3.63E-09	sp|Q5R7F0|CHP1_PONAB Calcineurin B homologous protein 1 OS=Pongo abelii GN=CHP1 PE=2 SV=3//7.11763e-131
XM_008016959.1	-0.30761	0.000487	0.003843	sp|Q92541|RTF1_HUMAN RNA polymerase-associated protein RTF1 homolog OS=Homo sapiens GN=RTF1 PE=1 SV=4//0
XM_008016978.1	0.43068	2.80E-08	4.02E-07	sp|Q13247|SRSF6_HUMAN Serine/arginine-rich splicing factor 6 OS=Homo sapiens GN=SRSF6 PE=1 SV=2//2.01362e-93
XM_008016986.1	3.8631	1.96E-37	1.77E-35	sp|Q9BUX1|CHAC1_HUMAN Glutathione-specific gamma-glutamylcyclotransferase 1 OS=Homo sapiens GN=CHAC1 PE=1 SV=2//3.4718e-162
XM_008016988.1	1.1016	8.10E-22	3.52E-20	sp|Q9P253|VPS18_HUMAN Vacuolar protein sorting-associated protein 18 homolog OS=Homo sapiens GN=VPS18 PE=1 SV=2//0
XM_008017008.1	0.75421	4.24E-05	0.0004	sp|Q96K21|ANCHR_HUMAN Abscission/NoCut checkpoint regulator OS=Homo sapiens GN=ZFYVE19 PE=1 SV=3//0
XM_008017042.1	1.5964	2.84E-17	9.35E-16	sp|P0DMQ5|INAM2_HUMAN Putative transmembrane protein INAFM2 OS=Homo sapiens GN=INAFM2 PE=2 SV=1//5.35789e-24
XM_008017073.1	#NAME?	0.005857	0.036048	sp|Q96LC9|BMF_HUMAN Bcl-2-modifying factor OS=Homo sapiens GN=BMF PE=1 SV=1//2.52172e-104
XM_008017078.1	-1.1088	7.98E-22	3.48E-20	sp|Q4R5C7|SRP14_MACFA Signal recognition particle 14 kDa protein OS=Macaca fascicularis GN=SRP14 PE=2 SV=1//3.90561e-68
XM_008017083.1	1.3774	3.56E-85	1.30E-82	sp|P07996|TSP1_HUMAN Thrombospondin-1 OS=Homo sapiens GN=THBS1 PE=1 SV=2//0
XM_008017098.1	0.80061	0.000443	0.003516	sp|Q7Z699|SPRE1_HUMAN Sprouty-related, EVH1 domain-containing protein 1 OS=Homo sapiens GN=SPRED1 PE=1 SV=2//0
XM_008017121.1	0.64947	1.03E-12	2.35E-11	sp|Q6IQ21|ZN770_HUMAN Zinc finger protein 770 OS=Homo sapiens GN=ZNF770 PE=2 SV=1//0
XM_008017148.1	1.0183	0.006677	0.040454	sp|Q96DM1|PGBD4_HUMAN PiggyBac transposable element-derived protein 4 OS=Homo sapiens GN=PGBD4 PE=2 SV=3//0
XM_008017161.1	1.3498	9.18E-43	9.95E-41	sp|Q68DA7|FMN1_HUMAN Formin-1 OS=Homo sapiens GN=FMN1 PE=1 SV=3//0
XM_008017204.1	1.5128	2.40E-08	3.49E-07	sp|Q9NXD2|MTMRA_HUMAN Myotubularin-related protein 10 OS=Homo sapiens GN=MTMR10 PE=1 SV=3//0
XM_008017212.1	0.53777	1.29E-05	0.000131	sp|Q96MG7|MAGG1_HUMAN Melanoma-associated antigen G1 OS=Homo sapiens GN=NDNL2 PE=1 SV=1//1.01165e-160

XM_008017254.1	-0.51757	2.43E-08	3.52E-07	sp|P30084|ECHM_HUMAN Enoyl-CoA hydratase, mitochondrial OS=Homo sapiens GN=ECHS1 PE=1 SV=4//0
XM_008017258.1	-0.62359	0.000188	0.001588	sp|Q6QHF9|PAOX_HUMAN Peroxisomal N(1)-acetyl-spermine/spermidine oxidase OS=Homo sapiens GN=PAOX PE=1 SV=3//5.38049e-81
XM_008017324.1	0.66079	4.01E-12	8.64E-11	sp|Q9WUL0|TOP1_RAT DNA topoisomerase 1 OS=Rattus norvegicus GN=Top1 PE=2 SV=1//0
XM_008017385.1	-1.5025	1.82E-05	0.00018	sp|Q6UVK1|CSPG4_HUMAN Chondroitin sulfate proteoglycan 4 OS=Homo sapiens GN=CSPG4 PE=1 SV=2//0
XM_008017390.1	-0.75961	8.03E-07	9.82E-06	sp|Q9UKP4|ATS7_HUMAN A disintegrin and metalloproteinase with thrombospondin motifs 7 OS=Homo sapiens GN=ADAMTS7 PE=1 SV=2//2.22983e-06
XM_008017392.1	-1.0586	7.77E-06	8.17E-05	sp|Q9UKP4|ATS7_HUMAN A disintegrin and metalloproteinase with thrombospondin motifs 7 OS=Homo sapiens GN=ADAMTS7 PE=1 SV=2//0
XM_008017393.1	1.2598	7.17E-09	1.11E-07	sp|Q9BY12|SCAPE_HUMAN S phase cyclin A-associated protein in the endoplasmic reticulum OS=Homo sapiens GN=SCAPER PE=1 SV=2//0
XM_008017396.1	-0.95235	1.71E-08	2.53E-07	sp|Q8TAC9|SCAM5_HUMAN Secretory carrier-associated membrane protein 5 OS=Homo sapiens GN=SCAMP5 PE=1 SV=1//5.23666e-117
XM_008017401.1	-0.58926	4.15E-17	1.34E-15	sp|Q5NVN0|KPYM_PONAB Pyruvate kinase PKM OS=Pongo abelii GN=PKM PE=2 SV=3//0
XM_008017406.1	-0.72295	1.38E-12	3.10E-11	sp|P23284|PPIB_HUMAN Peptidyl-prolyl cis-trans isomerase B OS=Homo sapiens GN=PPIB PE=1 SV=2//1.34814e-89
XM_008017408.1	1.4603	9.92E-18	3.35E-16	sp|Q08379|GOGA2_HUMAN Golgin subfamily A member 2 OS=Homo sapiens GN=GOLGA2 PE=1 SV=3//1.76982e-06
XM_008017413.1	0.44995	1.49E-06	1.75E-05	sp|Q96JI7|SPTCS_HUMAN Spatacsin OS=Homo sapiens GN=SPG11 PE=1 SV=3//0
XM_008017416.1	-0.4498	0.003286	0.021464	sp|Q6PFW1|VIP1_HUMAN Inositol hexakisphosphate and diphosphoinositol-pentakisphosphate kinase 1 OS=Homo sapiens GN=PPIP5K1 PE=1 SV=1//0
XM_008017422.1	-0.35717	0.001752	0.012225	sp|Q8NG31|CASC5_HUMAN Protein CASC5 OS=Homo sapiens GN=CASC5 PE=1 SV=3//0
XM_008017458.1	0.67264	1.54E-12	3.45E-11	sp|Q9P270|SLAI2_HUMAN SLAIN motif-containing protein 2 OS=Homo sapiens GN=SLAIN2 PE=1 SV=2//0
XM_008017461.1	0.5186	1.03E-06	1.25E-05	sp|Q9H4H8|FA83D_HUMAN Protein FAM83D OS=Homo sapiens GN=FAM83D PE=1 SV=3//0
XM_008017527.1	-2.7912	0.001	0.00739	sp|Q3SXM0|DC4L1_HUMAN DDB1- and CUL4-associated factor 4-like protein 1 OS=Homo sapiens GN=DCAF4L1 PE=2 SV=1//0
XM_008017541.1	-0.5236	6.50E-08	8.94E-07	sp|Q02878|RL6_HUMAN 60S ribosomal protein L6 OS=Homo sapiens GN=RPL6 PE=1 SV=3//5.02828e-151
XM_008017554.1	-0.50513	1.36E-10	2.57E-09	sp|Q60HC8|UCHL1_MACFA Ubiquitin carboxyl-terminal hydrolase isozyme L1 OS=Macaca fascicularis GN=UCHL1 PE=2 SV=1//1.37442e-148
XM_008017599.1	0.65065	6.86E-12	1.45E-10	sp|Q29RF7|PDS5A_HUMAN Sister chromatid cohesion protein PDS5 homolog A OS=Homo sapiens GN=PDS5A PE=1 SV=1//0
XM_008017615.1	-0.64014	3.93E-19	1.46E-17	sp|P32969|RL9_HUMAN 60S ribosomal protein L9 OS=Homo sapiens GN=RPL9 PE=1 SV=1//1.03586e-127
XM_008017616.1	-2.2948	5.58E-07	6.94E-06	sp|Q86Z14|KLOTB_HUMAN Beta-klotho OS=Homo sapiens GN=KLB PE=1 SV=1//0
XM_008017625.1	-0.58871	0.000672	0.005149	sp|Q96PQ7|KLHL5_HUMAN Kelch-like protein 5 OS=Homo sapiens GN=KLHL5 PE=2 SV=3//0
XM_008017631.1	-1.1758	1.86E-16	5.70E-15	sp|Q8IWE2|NXP20_HUMAN Protein NOXP20 OS=Homo sapiens GN=FAM114A1 PE=1 SV=2//0
XM_008017660.1	0.39996	1.22E-05	0.000125	sp|Q96G03|PGM2_HUMAN Phosphoglucomutase-2 OS=Homo sapiens GN=PGM2 PE=1 SV=4//0
XM_008017733.1	1.3139	6.39E-26	3.49E-24	sp|Q5RAK7|SPCS_PONAB O-phosphoseryl-tRNA(Sec) selenium transferase OS=Pongo abelii GN=SEPSECS PE=2 SV=1//0
XM_008017735.1	-0.60724	0.006418	0.039124	sp|Q1EGL1|LGI2_PANTR Leucine-rich repeat LGI family member 2 OS=Pan troglodytes GN=LGI2 PE=2 SV=1//0
XM_008017741.1	-0.75022	0.002247	0.015277	sp|P08294|SODE_HUMAN Extracellular superoxide dismutase [Cu-Zn] OS=Homo sapiens GN=SOD3 PE=1 SV=2//1.18455e-135
XM_008017742.1	0.51713	1.19E-06	1.42E-05	sp|Q5RAZ4|DHX15_PONAB Pre-mRNA-splicing factor ATP-dependent RNA helicase DHX15 OS=Pongo abelii GN=DHX15 PE=2 SV=2//0
XM_008017793.1	0.569	0.001019	0.007512	sp|Q9BPX3|CND3_HUMAN Condensin complex subunit 3 OS=Homo sapiens GN=NCAPG PE=1 SV=1//0
XM_008017801.1	-0.40482	1.24E-07	1.65E-06	sp|P21980|TGM2_HUMAN Protein-glutamine gamma-glutamyltransferase 2 OS=Homo sapiens GN=TGM2 PE=1 SV=2//0
XM_008017803.1	-1.0711	0.001822	0.012637	sp|Q9ULE4|F184B_HUMAN Protein FAM184B OS=Homo sapiens GN=FAM184B PE=2 SV=3//0
XM_008017805.1	-0.66867	1.98E-06	2.29E-05	sp|Q9H204|MED28_HUMAN Mediator of RNA polymerase II transcription subunit 28 OS=Homo sapiens GN=MED28 PE=1 SV=1//4.97306e-88
XM_008017806.1	-0.31271	0.001011	0.007462	sp|P28838|AMPL_HUMAN Cytosol aminopeptidase OS=Homo sapiens GN=LAP3 PE=1 SV=3//0
XM_008017808.1	0.39786	0.00091	0.006759	sp|P09417|DHPR_HUMAN Dihydropteridine reductase OS=Homo sapiens GN=QDPR PE=1 SV=2//1.02449e-150
XM_008017842.1	1.0169	7.64E-25	3.99E-23	sp|Q9NQG5|RPR1B_HUMAN Regulation of nuclear pre-mRNA domain-containing protein 1B OS=Homo sapiens GN=RPRD1B PE=1 SV=1//0
XM_008017848.1	-0.33014	0.001796	0.012486	sp|Q10588|BST1_HUMAN ADP-ribosyl cyclase/cyclic ADP-ribose hydrolase 2

				OS=Homo sapiens GN=BST1 PE=1 SV=2//0
XM_008017862.1	-0.3402	0.000328	0.002665	sp|Q8WYA6|CTBL1_HUMAN Beta-catenin-like protein 1 OS=Homo sapiens GN=CTNNBL1 PE=1 SV=1//0
XM_008017864.1	-1.2411	1.10E-05	0.000113	sp|Q9UKA1|FBXL5_HUMAN F-box/LRR-repeat protein 5 OS=Homo sapiens GN=FBXL5 PE=1 SV=2//0
XM_008017869.1	0.80092	5.92E-12	1.26E-10	sp|P0CF97|F200B_HUMAN Protein FAM200B OS=Homo sapiens GN=FAM200B PE=3 SV=1//0
XM_008017886.1	0.53212	0.002348	0.015876	sp|Q5RFI2|RAB28_PONAB Ras-related protein Rab-28 OS=Pongo abelii GN=RAB28 PE=2 SV=1//2.33399e-158
XM_008017902.1	-0.38561	3.91E-07	4.94E-06	sp|O75083|WDR1_HUMAN WD repeat-containing protein 1 OS=Homo sapiens GN=WDR1 PE=1 SV=4//0
XM_008017919.1	0.64286	2.50E-10	4.57E-09	sp|Q9P2W9|STX18_HUMAN Syntaxin-18 OS=Homo sapiens GN=STX18 PE=1 SV=1//0
XM_008017924.1	0.60433	3.45E-05	0.000329	sp|Q2VL84|MSX1_CALJA Homeobox protein MSX-1 OS=Callithrix jacchus GN=MSX1 PE=3 SV=2//1.32436e-123
XM_008018001.1	0.85727	2.06E-14	5.38E-13	sp|Q5RBN9|TAD2B_PONAB Transcriptional adapter 2-beta OS=Pongo abelii GN=TADA2B PE=2 SV=1//0
XM_008018015.1	-1.0101	0.000705	0.005373	sp|P83110|HTRA3_HUMAN Serine protease HTRA3 OS=Homo sapiens GN=HTRA3 PE=1 SV=2//0
XM_008018017.1	0.66035	4.17E-08	5.89E-07	sp|O15254|ACOX3_HUMAN Peroxisomal acyl-coenzyme A oxidase 3 OS=Homo sapiens GN=ACOX3 PE=1 SV=2//0
XM_008018028.1	0.67676	5.04E-05	0.000471	sp|Q9NP08|HMX1_HUMAN Homeobox protein HMX1 OS=Homo sapiens GN=HMX1 PE=2 SV=2//1.37511e-61
XM_008018030.1	-0.93087	0.001788	0.012437	sp|P18825|ADA2C_HUMAN Alpha-2C adrenergic receptor OS=Homo sapiens GN=ADRA2C PE=2 SV=2//0
XM_008018031.1	-0.34287	0.000792	0.005972	sp|P30533|AMRP_HUMAN Alpha-2-macroglobulin receptor-associated protein OS=Homo sapiens GN=LRPAP1 PE=1 SV=1//0
XM_008018038.1	-2.1859	0.005945	0.036473	sp|Q04756|HGFA_HUMAN Hepatocyte growth factor activator OS=Homo sapiens GN=HGFAC PE=1 SV=1//0
XM_008018064.1	1.24	0.004498	0.028493	sp|P78316|NOP14_HUMAN Nucleolar protein 14 OS=Homo sapiens GN=NOP14 PE=1 SV=3//0
XM_008018069.1	0.49142	0.006737	0.040773	sp|P28749|RBL1_HUMAN Retinoblastoma-like protein 1 OS=Homo sapiens GN=RBL1 PE=1 SV=3//1.3534e-80
XM_008018076.1	-1.8029	4.54E-08	6.34E-07	sp|P35611|ADDA_HUMAN Alpha-adducin OS=Homo sapiens GN=ADD1 PE=1 SV=2//0
XM_008018122.1	0.68893	0.000939	0.006955	-//-
XM_008018123.1	0.57305	0.000328	0.002664	sp|Q8N9F0|NAT8L_HUMAN N-acetylaspartate synthetase OS=Homo sapiens GN=NAT8L PE=1 SV=3//2.45007e-134
XM_008018125.1	-0.9489	1.02E-08	1.55E-07	sp|Q5BLP8|CD048_HUMAN Neuropeptide-like protein C4orf48 OS=Homo sapiens GN=C4orf48 PE=1 SV=3//7.45532e-33
XM_008018126.1	0.64825	1.08E-10	2.05E-09	sp|Q9H3P2|NELFA_HUMAN Negative elongation factor A OS=Homo sapiens GN=NELFA PE=1 SV=3//0
XM_008018130.1	-0.61029	0.000376	0.003028	sp|O96028|NSD2_HUMAN Histone-lysine N-methyltransferase NSD2 OS=Homo sapiens GN=WHSC1 PE=1 SV=1//0
XM_008018160.1	2.5657	0.000525	0.00411	sp|Q5VYV7|SLX4I_HUMAN Protein SLX4IP OS=Homo sapiens GN=SLX4IP PE=1 SV=1//0
XM_008018170.1	1.0734	3.19E-46	3.96E-44	sp|P78504|JAG1_HUMAN Protein jagged-1 OS=Homo sapiens GN=JAG1 PE=1 SV=3//0
XM_008018172.1	-1.141	7.72E-05	0.000703	sp|Q9BUD6|SPON2_HUMAN Spondin-2 OS=Homo sapiens GN=SPON2 PE=1 SV=3//0
XM_008018185.1	-0.47801	0.000139	0.001205	sp|P35475|IDUA_HUMAN Alpha-L-iduronidase OS=Homo sapiens GN=IDUA PE=1 SV=2//0
XM_008018194.1	1.1823	6.13E-51	9.38E-49	sp|P24385|CCND1_HUMAN G1/S-specific cyclin-D1 OS=Homo sapiens GN=CCND1 PE=1 SV=1//2.16194e-167
XM_008018195.1	#NAME?	0.000685	0.005241	-//-
XM_008018202.1	-1.3974	0.00034	0.002759	sp|Q5RBW2|ATP5I_PONAB ATP synthase subunit e, mitochondrial OS=Pongo abelii GN=ATP5I PE=3 SV=3//5.19268e-28
XM_008018265.1	1.0655	0.000157	0.001349	sp|Q9UHL9|GT2D1_HUMAN General transcription factor II-I repeat domain-containing protein 1 OS=Homo sapiens GN=GTF2IRD1 PE=1 SV=1//2.17614e-07
XM_008018280.1	-0.34035	0.000271	0.002233	sp|Q9NWD8|TM248_HUMAN Transmembrane protein 248 OS=Homo sapiens GN=TMEM248 PE=1 SV=1//0
XM_008018305.1	-1.5922	3.77E-47	4.88E-45	sp|A6NHX0|GATL2_HUMAN GATS-like protein 2 OS=Homo sapiens GN=GATSL2 PE=2 SV=3//0
XM_008018314.1	-0.22847	0.00182	0.012626	sp|P78347|GTF2I_HUMAN General transcription factor II-I OS=Homo sapiens GN=GTF2I PE=1 SV=2//0
XM_008018331.1	1.5243	6.23E-66	1.42E-63	sp|O19005|CLD4_CHLAE Claudin-4 OS=Chlorocebus aethiops GN=CLDN4 PE=2 SV=1//2.21035e-96
XM_008018333.1	-0.91919	1.38E-06	1.63E-05	sp|O15551|CLD3_HUMAN Claudin-3 OS=Homo sapiens GN=CLDN3 PE=1 SV=1//1.75113e-102
XM_008018338.1	-0.85821	0.002606	0.017404	sp|Q16623|STX1A_HUMAN Syntaxin-1A OS=Homo sapiens GN=STX1A PE=1 SV=1//2.88596e-124
XM_008018345.1	1.1371	1.64E-32	1.21E-30	sp|Q9Y4P3|TBL2_HUMAN Transducin beta-like protein 2 OS=Homo sapiens GN=TBL2 PE=1 SV=1//0

XM_008018369.1	1.1102	0.000183	0.001554	sp|Q8WV07|ORAV1_HUMAN Oral cancer-overexpressed protein 1 OS=Homo sapiens GN=ORAOV1 PE=1 SV=2//3.6661e-34
XM_008018370.1	0.38571	0.000522	0.004088	sp|O60488|ACSL4_HUMAN Long-chain-fatty-acid--CoA ligase 4 OS=Homo sapiens GN=ACSL4 PE=1 SV=2//0
XM_008018385.1	-2.1513	0.000533	0.004165	sp|Q9Y6J8|STYL1_HUMAN Serine/threonine/tyrosine-interacting-like protein 1 OS=Homo sapiens GN=STYXL1 PE=2 SV=1//2.20558e-143
XM_008018394.1	2.0388	0.001082	0.007938	sp|Q9Y6J8|STYL1_HUMAN Serine/threonine/tyrosine-interacting-like protein 1 OS=Homo sapiens GN=STYXL1 PE=2 SV=1//0
XM_008018400.1	-1.8035	0.007342	0.043864	sp|Q8OWV7|SRRM3_MOUSE Serine/arginine repetitive matrix protein 3 OS=Mus musculus GN=Srrm3 PE=2 SV=1//1.20076e-67
XM_008018401.1	-0.89098	2.51E-07	3.25E-06	sp|Q8OWV7|SRRM3_MOUSE Serine/arginine repetitive matrix protein 3 OS=Mus musculus GN=Srrm3 PE=2 SV=1//1.06714e-66
XM_008018402.1	-0.69091	1.83E-22	8.39E-21	sp|P04792|HSPB1_HUMAN Heat shock protein beta-1 OS=Homo sapiens GN=HSPB1 PE=1 SV=2//2.35529e-90
XM_008018416.1	-0.78212	2.87E-14	7.43E-13	sp|Q9H1E5|TMX4_HUMAN Thioredoxin-related transmembrane protein 4 OS=Homo sapiens GN=TMX4 PE=1 SV=1//3.90371e-172
XM_008018432.1	2.1731	1.47E-05	0.000148	sp|Q5R5J3|PKRI1_PONAB PRKR-interacting protein 1 OS=Pongo abelii GN=PRKRIP1 PE=2 SV=1//1.20881e-67
XM_008018467.1	-0.70391	3.49E-14	8.94E-13	sp|O43257|ZNHI1_HUMAN Zinc finger HIT domain-containing protein 1 OS=Homo sapiens GN=ZNHIT1 PE=1 SV=1//1.9339e-81
XM_008018472.1	2.2822	3.40E-13	8.10E-12	sp|O15240|VGF_HUMAN Neurosecretory protein VGF OS=Homo sapiens GN=VGF PE=1 SV=2//3.19072e-61
XM_008018474.1	Inf	0.002773	0.018386	sp|O15240|VGF_HUMAN Neurosecretory protein VGF OS=Homo sapiens GN=VGF PE=1 SV=2//2.1468e-61
XM_008018480.1	1.1056	1.63E-22	7.49E-21	sp|Q9BRZ2|TRI56_HUMAN E3 ubiquitin-protein ligase TRIM56 OS=Homo sapiens GN=TRIM56 PE=1 SV=3//0
XM_008018501.1	0.56144	0.007907	0.046835	sp|Q9CZP0|UFSP1_MOUSE Ufm1-specific protease 1 OS=Mus musculus GN=Ufsp1 PE=1 SV=1//9.06102e-101
XM_008018524.1	-0.63257	3.43E-15	9.52E-14	sp|P54313|GBB2_RAT Guanine nucleotide-binding protein G(I)/G(S)/G(T) subunit beta-2 OS=Rattus norvegicus GN=Gnb2 PE=1 SV=4//5.85104e-06
XM_008018529.1	3.2775	1.73E-14	4.59E-13	sp|P12643|BMP2_HUMAN Bone morphogenetic protein 2 OS=Homo sapiens GN=BMP2 PE=1 SV=1//0
XM_008018542.1	-0.97728	5.54E-08	7.69E-07	sp|Q15113|PCOC1_HUMAN Procollagen C-endopeptidase enhancer 1 OS=Homo sapiens GN=PCOLCE PE=1 SV=2//0
XM_008018552.1	0.86033	4.01E-22	1.80E-20	sp|Q7L2J0|MEPCE_HUMAN 7SK snRNA methylphosphate capping enzyme OS=Homo sapiens GN=MEPCE PE=1 SV=1//0
XM_008018556.1	-1.0307	0.000258	0.00213	sp|Q6DKI7|PVRIG_HUMAN Transmembrane protein PVRIG OS=Homo sapiens GN=PVRIG PE=2 SV=1//1.17906e-176
XM_008018557.1	1.1909	0.007226	0.043259	sp|Q8WVR3|CG043_HUMAN Uncharacterized protein C7orf43 OS=Homo sapiens GN=C7orf43 PE=1 SV=2//0
XM_008018560.1	-1.4883	3.87E-12	8.36E-11	sp|Q96RP7|G3ST4_HUMAN Galactose-3-O-sulfotransferase 4 OS=Homo sapiens GN=GAL3ST4 PE=1 SV=1//0
XM_008018583.1	-0.65453	4.53E-08	6.34E-07	sp|Q5R483|LTOR4_PONAB Ragulator complex protein LAMTOR4 OS=Pongo abelii GN=LAMTOR4 PE=3 SV=1//1.34602e-63
XM_008018586.1	-1.2184	9.08E-16	2.64E-14	sp|Q8N129|CNPY4_HUMAN Protein canopy homolog 4 OS=Homo sapiens GN=CNPY4 PE=2 SV=1//3.28823e-133
XM_008018593.1	-0.62449	7.05E-13	1.63E-11	sp|Q5REY0|CSN6_PONAB COP9 signalosome complex subunit 6 OS=Pongo abelii GN=COPS6 PE=2 SV=1//0
XM_008018595.1	1.2041	1.40E-16	4.35E-15	sp|A1YG26|ZSC21_PANPA Zinc finger and SCAN domain-containing protein 21 OS=Pan paniscus GN=ZSCAN21 PE=3 SV=1//0
XM_008018605.1	-0.98451	0.000934	0.006923	sp|P25311|ZA2G_HUMAN Zinc-alpha-2-glycoprotein OS=Homo sapiens GN=AZGP1 PE=1 SV=2//0
XM_008018607.1	1.4762	1.07E-52	1.76E-50	sp|Q9C037|TRIM4_HUMAN E3 ubiquitin-protein ligase TRIM4 OS=Homo sapiens GN=TRIM4 PE=1 SV=2//0
XM_008018610.1	6.8327	5.55E-19	2.02E-17	sp|P33268|CP3A8_MACFA Cytochrome P450 3A8 OS=Macaca fascicularis GN=CYP3A8 PE=1 SV=1//0
XM_008018617.1	1.77	9.45E-08	1.28E-06	sp|P20815|CP3A5_HUMAN Cytochrome P450 3A5 OS=Homo sapiens GN=CYP3A5 PE=1 SV=1//0
XM_008018648.1	-2.956	0.003069	0.020191	sp|Q5FWF6|ZN789_HUMAN Zinc finger protein 789 OS=Homo sapiens GN=ZNF789 PE=2 SV=3//0
XM_008018651.1	0.93869	3.20E-13	7.67E-12	sp|Q53GI3|ZN394_HUMAN Zinc finger protein 394 OS=Homo sapiens GN=ZNF394 PE=1 SV=2//0
XM_008018653.1	-1.31	2.78E-15	7.77E-14	sp|Q5R6T5|ATPK_PONAB ATP synthase subunit f, mitochondrial OS=Pongo abelii GN=ATP5J2 PE=3 SV=3//2.31746e-49
XM_008018666.1	-0.28273	0.000616	0.004757	sp|Q13442|HAP28_HUMAN 28 kDa heat- and acid-stable phosphoprotein OS=Homo sapiens GN=PDAP1 PE=1 SV=1//8.2052e-54
XM_008018671.1	-0.32517	0.00013	0.00113	sp|Q92747|ARC1A_HUMAN Actin-related protein 2/3 complex subunit 1A OS=Homo sapiens GN=ARPC1A PE=1 SV=2//0
XM_008018728.1	0.42116	2.47E-05	0.000241	sp|Q4R3X9|F220A_MACFA Protein FAM220A OS=Macaca fascicularis GN=FAM220A PE=2 SV=1//2.11423e-169
XM_008018754.1	-0.33884	0.001351	0.009682	sp|P0CG23|ZN853_HUMAN Zinc finger protein 853 OS=Homo sapiens GN=ZNF853

				PE=2 SV=1//6.89373e-21
XM_008018762.1	1.3362	4.49E-10	7.98E-09	sp|P17014|ZNF12_HUMAN Zinc finger protein 12 OS=Homo sapiens GN=ZNF12 PE=2 SV=3//0
XM_008018773.1	-0.33527	5.24E-06	5.68E-05	sp|Q16658|FSCN1_HUMAN Fascin OS=Homo sapiens GN=FSCN1 PE=1 SV=3//0
XM_008018774.1	0.39804	0.006527	0.039687	sp|Q96ME1|FXL18_HUMAN F-box/LRR-repeat protein 18 OS=Homo sapiens GN=FBXL18 PE=1 SV=2//0
XM_008018850.1	-0.72369	1.31E-18	4.67E-17	sp|Q9C0H2|TTYH3_HUMAN Protein tweety homolog 3 OS=Homo sapiens GN=TTYH3 PE=1 SV=3//0
XM_008018851.1	0.93661	2.84E-10	5.14E-09	sp|Q8NES3|LFNG_HUMAN Beta-1,3-N-acetylglucosaminyltransferase lunatic fringe OS=Homo sapiens GN=LFNG PE=1 SV=2//0
XM_008018864.1	-0.85411	5.88E-06	6.33E-05	sp|P36639|8ODP_HUMAN 7,8-dihydro-8-oxoguanine triphosphatase OS=Homo sapiens GN=NUDT1 PE=1 SV=3//1.63054e-112
XM_008018865.1	1.0029	3.23E-33	2.48E-31	sp|Q9Y5X2|SNX8_HUMAN Sorting nexin-8 OS=Homo sapiens GN=SNX8 PE=1 SV=1//0
XM_008018868.1	1.224	0.000825	0.006189	sp|Q9BT73|PSMG3_HUMAN Proteasome assembly chaperone 3 OS=Homo sapiens GN=PSMG3 PE=1 SV=1//1.74393e-77
XM_008018874.1	1.1987	0.002451	0.016458	sp|O60675|MAFK_HUMAN Transcription factor MafK OS=Homo sapiens GN=MAFK PE=1 SV=1//5.70286e-80
XM_008018885.1	2.695	1.01E-42	1.09E-40	sp|Q5R966|ZFN2A_PONAB AN1-type zinc finger protein 2A OS=Pongo abelii GN=ZFAND2A PE=3 SV=1//4.32439e-97
XM_008018929.1	-0.46982	9.07E-09	1.39E-07	sp|O95674|CDS2_HUMAN Phosphatidate cytidylyltransferase 2 OS=Homo sapiens GN=CDS2 PE=1 SV=1//0
XM_008018932.1	0.45491	3.08E-07	3.92E-06	sp|Q8IXL6|FA20C_HUMAN Extracellular serine/threonine protein kinase FAM20C OS=Homo sapiens GN=FAM20C PE=1 SV=2//0
XM_008018943.1	1.3465	3.86E-49	5.45E-47	sp|Q9UHL9|GT2D1_HUMAN General transcription factor II-I repeat domain-containing protein 1 OS=Homo sapiens GN=GTF2IRD1 PE=1 SV=1//2.48766e-10
XM_008018956.1	0.86478	2.02E-16	6.16E-15	sp|Q9H0M4|ZCPW1_HUMAN Zinc finger CW-type PWWP domain protein 1 OS=Homo sapiens GN=ZCWPW1 PE=1 SV=2//4.31979e-90
XM_008018957.1	-0.6327	3.00E-15	8.37E-14	sp|O15143|ARC1B_HUMAN Actin-related protein 2/3 complex subunit 1B OS=Homo sapiens GN=ARPC1B PE=1 SV=3//3.58962e-119
XM_008018958.1	1.2771	9.82E-47	1.24E-44	sp|Q9UHR4|BI2L1_HUMAN Brain-specific angiogenesis inhibitor 1-associated protein 2-like protein 1 OS=Homo sapiens GN=BAIAP2L1 PE=1 SV=2//0
XM_008018960.1	0.91674	1.94E-15	5.49E-14	sp|Q96N11|CG026_HUMAN Uncharacterized protein C7orf26 OS=Homo sapiens GN=C7orf26 PE=2 SV=1//0
XM_008018961.1	-1.0198	3.84E-23	1.86E-21	sp|P53505|ACT5_XENLA Actin, cytoplasmic type 5 OS=Xenopus laevis PE=3 SV=1//5.31061e-164
XM_008018962.1	-1.106	1.93E-16	5.90E-15	sp|P60707|ACTB_TRIVU Actin, cytoplasmic 1 OS=Trichosurus vulpecula GN=ACTB PE=2 SV=1//9.88355e-98
XM_008018966.1	0.86106	0.000697	0.005321	sp|Q9UGH3|S23A2_HUMAN Solute carrier family 23 member 2 OS=Homo sapiens GN=SLC23A2 PE=1 SV=1//0
XM_008018967.1	0.34232	0.001797	0.012488	sp|Q6PJG6|BRAT1_HUMAN BRCA1-associated ATM activator 1 OS=Homo sapiens GN=BRAT1 PE=1 SV=2//0
XM_008019068.1	0.28472	0.001875	0.012947	sp|P67988|PRIO_CHLAE Major prion protein OS=Chlorocebus aethiops GN=PRNP PE=3 SV=1//2.70449e-94
XM_008019071.1	1.3516	2.65E-14	6.86E-13	sp|Q12996|CSTF3_HUMAN Cleavage stimulation factor subunit 3 OS=Homo sapiens GN=CSTF3 PE=1 SV=1//0
XM_008019078.1	-0.38571	0.00115	0.008368	sp|P11216|PYGB_HUMAN Glycogen phosphorylase, brain form OS=Homo sapiens GN=PYGB PE=1 SV=5//0
XM_008019083.1	0.61781	5.41E-05	0.000502	sp|Q9NWM0|SMOX_HUMAN Spermine oxidase OS=Homo sapiens GN=SMOX PE=1 SV=1//4.66137e-80
XM_008019094.1	-0.37178	0.000844	0.006318	sp|Q9UGV2|NDRG3_HUMAN Protein NDRG3 OS=Homo sapiens GN=NDRG3 PE=1 SV=2//6.78484e-28
XM_008019102.1	0.53029	4.38E-07	5.50E-06	sp|O75354|ENTP6_HUMAN Ectonucleoside triphosphate diphosphohydrolase 6 OS=Homo sapiens GN=ENTPD6 PE=1 SV=3//0
XM_008019126.1	0.45933	2.03E-08	2.98E-07	sp|Q8NC60|NOA1_HUMAN Nitric oxide-associated protein 1 OS=Homo sapiens GN=NOA1 PE=1 SV=2//0
XM_008019172.1	0.64791	6.85E-05	0.000627	sp|Q7L7X3|TAOK1_HUMAN Serine/threonine-protein kinase TAO1 OS=Homo sapiens GN=TAOK1 PE=1 SV=1//3.81062e-107
XM_008019194.1	2.2601	5.51E-13	1.29E-11	sp|Q96EZ4|MYEOV_HUMAN Myeloma-overexpressed gene protein OS=Homo sapiens GN=MYEOV PE=2 SV=2//2.84e-112
XM_008019208.1	3.574	0.00072	0.005476	sp|P84239|H3_URECA Histone H3 OS=Urechis caupo PE=1 SV=2//1.47906e-81
XM_008019209.1	-0.88819	1.69E-05	0.000169	sp|P0C1H3|H2B1_CHICK Histone H2B 1/2/3/4/6 OS=Gallus gallus GN=H2B-I PE=1 SV=2//1.44895e-54
XM_008019211.1	3.3337	0.004931	0.030982	sp|Q8IUE6|H2A2B_HUMAN Histone H2A type 2-B OS=Homo sapiens GN=HIST2H2AB PE=1 SV=3//2.17808e-68
XM_008019229.1	1.3923	9.12E-23	4.27E-21	sp|Q6GQQ9|OTU7B_HUMAN OTU domain-containing protein 7B OS=Homo sapiens GN=OTUD7B PE=1 SV=1//0
XM_008019235.1	0.63752	1.51E-08	2.25E-07	sp|Q53GL0|PKHO1_HUMAN Pleckstrin homology domain-containing family O member 1 OS=Homo sapiens GN=PLEKHO1 PE=1 SV=2//0
XM_008019241.1	0.50738	0.001628	0.011459	sp|Q95KD0|FAKD5_MACFA FAST kinase domain-containing protein 5 OS=Macaca fascicularis GN=FASTKD5 PE=2 SV=1//5.55197e-58

XM_008019247.1	-0.57327	9.44E-11	1.81E-09	sp|Q96BI3|APH1A_HUMAN Gamma-secretase subunit APH-1A OS=Homo sapiens GN=APH1A PE=1 SV=1//6.39499e-167
XM_008019256.1	1.276	3.27E-06	3.68E-05	sp|O43395|PRPF3_HUMAN U4/U6 small nuclear ribonucleoprotein Prp3 OS=Homo sapiens GN=PRPF3 PE=1 SV=2//0
XM_008019283.1	-1.7087	2.62E-08	3.78E-07	sp|P61277|CATK_MACMU Cathepsin K OS=Macaca mulatta GN=CTSK PE=1 SV=1//0
XM_008019316.1	0.75029	0.000359	0.0029	sp|Q9BUN1|MENT_HUMAN Protein MENT OS=Homo sapiens GN=MENT PE=2 SV=1//5.00363e-163
XM_008019319.1	0.56858	1.00E-11	2.09E-10	sp|Q5VT52|RPRD2_HUMAN Regulation of nuclear pre-mRNA domain-containing protein 2 OS=Homo sapiens GN=RPRD2 PE=1 SV=1//0
XM_008019322.1	0.52009	6.66E-09	1.03E-07	sp|Q15047|SETB1_HUMAN Histone-lysine N-methyltransferase SETDB1 OS=Homo sapiens GN=SETDB1 PE=1 SV=1//0
XM_008019334.1	1.1795	8.47E-24	4.23E-22	sp|O43741|AAKB2_HUMAN 5'-AMP-activated protein kinase subunit beta-2 OS=Homo sapiens GN=PRKAB2 PE=1 SV=1//2.44886e-177
XM_008019359.1	1.2244	1.30E-31	9.21E-30	sp|Q4R4U9|SYSC_MACFA Serine--tRNA ligase, cytoplasmic OS=Macaca fascicularis GN=SARS PE=2 SV=3//8.59689e-177
XM_008019365.1	0.45045	0.001709	0.011956	sp|Q9NRR8|C42S1_HUMAN CDC42 small effector protein 1 OS=Homo sapiens GN=CDC42SE1 PE=1 SV=1//2.86534e-49
XM_008019391.1	-0.65601	3.15E-06	3.54E-05	sp|P12277|KCRB_HUMAN Creatine kinase B-type OS=Homo sapiens GN=CKB PE=1 SV=1//0
XM_008019403.1	-0.77001	2.25E-09	3.70E-08	sp|P78552|I13R1_HUMAN Interleukin-13 receptor subunit alpha-1 OS=Homo sapiens GN=IL13RA1 PE=1 SV=1//0
XM_008019405.1	-0.72538	0.002363	0.015962	sp|P0CG32|ZCC18_HUMAN Zinc finger CCHC domain-containing protein 18 OS=Homo sapiens GN=ZCCHC18 PE=3 SV=1//0
XM_008019413.1	-0.78863	4.63E-23	2.22E-21	sp|Q5RED0|PGRC1_PONAB Membrane-associated progesterone receptor component 1 OS=Pongo abelii GN=PGRMC1 PE=2 SV=3//4.49538e-121
XM_008019418.1	-0.49345	1.39E-08	2.08E-07	sp|P05141|ADT2_HUMAN ADP/ATP translocase 2 OS=Homo sapiens GN=SLC25A5 PE=1 SV=7//0
XM_008019422.1	1.1824	2.04E-19	7.73E-18	sp|O15226|NKRF_HUMAN NF-kappa-B-repressing factor OS=Homo sapiens GN=NKRF PE=1 SV=2//0
XM_008019434.1	-0.87733	0.000836	0.006271	sp|P62893|RL39_RAT 60S ribosomal protein L39 OS=Rattus norvegicus GN=Rpl39 PE=1 SV=2//7.98183e-28
XM_008019438.1	-1.2176	3.90E-12	8.42E-11	sp|Q7JGX4|NDUA1_PANPA NADH dehydrogenase [ubiquinone] 1 alpha subcomplex subunit 1 OS=Pan paniscus GN=NDUFA1 PE=3 SV=1//1.58259e-41
XM_008019454.1	-1.1897	1.12E-06	1.34E-05	sp|P13473|LAMP2_HUMAN Lysosome-associated membrane glycoprotein 2 OS=Homo sapiens GN=LAMP2 PE=1 SV=2//0
XM_008019456.1	-0.79657	4.76E-06	5.20E-05	sp|P13473|LAMP2_HUMAN Lysosome-associated membrane glycoprotein 2 OS=Homo sapiens GN=LAMP2 PE=1 SV=2//0
XM_008019469.1	-1.1483	8.76E-06	9.13E-05	sp|Q64119|MYL6_RAT Myosin light polypeptide 6 OS=Rattus norvegicus GN=Myl6 PE=1 SV=3//2.27848e-104
XM_008019579.1	-0.21866	0.006652	0.040336	sp|Q8HXX7|GDIA_MACFA Rab GDP dissociation inhibitor alpha OS=Macaca fascicularis GN=GDI1 PE=2 SV=1//0
XM_008019580.1	-0.63875	3.94E-15	1.09E-13	sp|Q15904|VAS1_HUMAN V-type proton ATPase subunit S1 OS=Homo sapiens GN=ATP6AP1 PE=1 SV=2//0
XM_008019587.1	0.68938	0.006783	0.041043	sp|P17010|ZFX_HUMAN Zinc finger X-chromosomal protein OS=Homo sapiens GN=ZFX PE=2 SV=2//2.60452e-75
XM_008019607.1	-1.015	0.000357	0.002888	sp|P78539|SRPX_HUMAN Sushi repeat-containing protein SRPX OS=Homo sapiens GN=SRPX PE=1 SV=1//7.74588e-57
XM_008019639.1	-0.24664	0.000441	0.003506	sp|P39023|RL3_HUMAN 60S ribosomal protein L3 OS=Homo sapiens GN=RPL3 PE=1 SV=2//0
XM_008019643.1	1.0052	4.18E-08	5.89E-07	sp|O15014|ZN609_HUMAN Zinc finger protein 609 OS=Homo sapiens GN=ZNF609 PE=1 SV=2//0
XM_008019660.1	-0.42641	4.79E-09	7.61E-08	sp|P12236|ADT3_HUMAN ADP/ATP translocase 3 OS=Homo sapiens GN=SLC25A6 PE=1 SV=4//0
XM_008019664.1	-0.78401	1.30E-06	1.54E-05	sp|O95671|ASML_HUMAN N-acetylserotonin O-methyltransferase-like protein OS=Homo sapiens GN=ASMTL PE=1 SV=3//0
XM_008019666.1	0.74973	0.000553	0.004308	sp|Q02040|AK17A_HUMAN A-kinase anchor protein 17A OS=Homo sapiens GN=AKAP17A PE=1 SV=2//5.23759e-93
XM_008019670.1	-3.8342	0.00013	0.00113	-//-
XM_008019677.1	0.27635	0.002364	0.015962	sp|P07199|CENPB_HUMAN Major centromere autoantigen B OS=Homo sapiens GN=CENPB PE=1 SV=2//1.51633e-45
XM_008019699.1	0.44471	0.000525	0.004107	sp|Q5R9I4|TV23B_PONAB Golgi apparatus membrane protein TVP23 homolog B OS=Pongo abelii GN=TVP23B PE=2 SV=1//2.39299e-141
XM_008019702.1	-0.88266	0.003446	0.02243	sp|Q9UKP4|ATS7_HUMAN A disintegrin and metalloproteinase with thrombospondin motifs 7 OS=Homo sapiens GN=ADAMTS7 PE=1 SV=2//5.43028e-08
XM_008019703.1	-0.76945	4.44E-06	4.88E-05	sp|Q9UKP4|ATS7_HUMAN A disintegrin and metalloproteinase with thrombospondin motifs 7 OS=Homo sapiens GN=ADAMTS7 PE=1 SV=2//4.15159e-17
XM_008019730.1	0.35333	0.001926	0.013264	sp|O15530|PDPK1_HUMAN 3-phosphoinositide-dependent protein kinase 1 OS=Homo sapiens GN=PDPK1 PE=1 SV=1//0
XM_008019736.1	-0.64059	3.07E-05	0.000295	sp|Q7Z434|MAVS_HUMAN Mitochondrial antiviral-signaling protein OS=Homo sapiens GN=MAVS PE=1 SV=2//0

XM_008019741.1	1.0426	6.85E-18	2.35E-16	sp|Q9BZ23|PANK2_HUMAN Pantothenate kinase 2, mitochondrial OS=Homo sapiens GN=PANK2 PE=1 SV=3//0
XM_008019788.1	0.45618	1.30E-05	0.000132	sp|Q9NUD5|ZCHC3_HUMAN Zinc finger CCHC domain-containing protein 3 OS=Homo sapiens GN=ZCCHC3 PE=1 SV=1//1.06477e-179
XM_008019794.1	-0.92356	7.86E-05	0.000714	sp|O60218|AK1BA_HUMAN Aldo-keto reductase family 1 member B10 OS=Homo sapiens GN=AKR1B10 PE=1 SV=2//2.12154e-09
XM_008019798.1	0.83943	1.50E-06	1.76E-05	sp|Q9P2J8|ZN624_HUMAN Zinc finger protein 624 OS=Homo sapiens GN=ZNF624 PE=1 SV=3//4.06531e-115
XM_008019805.1	Inf	0.000447	0.003549	sp|Q13046|PSG7_HUMAN Putative pregnancy-specific beta-1-glycoprotein 7 OS=Homo sapiens GN=PSG7 PE=5 SV=2//5.40625e-64
XM_008019811.1	-0.86787	1.51E-06	1.77E-05	sp|P0CC09|H2A2A_RAT Histone H2A type 2-A OS=Rattus norvegicus GN=Hist2h2aa3 PE=1 SV=1//8.2361e-70
XM_008019812.1	1.8726	2.69E-07	3.46E-06	sp|P84239|H3_URECA Histone H3 OS=Urechis caupo PE=1 SV=2//3.00721e-89
XM_008019816.1	-0.45185	3.98E-06	4.40E-05	sp|Q04890|SOX12_MOUSE Transcription factor SOX-12 OS=Mus musculus GN=Sox12 PE=2 SV=2//2.6215e-33
XM_008019817.1	2.2013	6.37E-06	6.80E-05	sp|Q13046|PSG7_HUMAN Putative pregnancy-specific beta-1-glycoprotein 7 OS=Homo sapiens GN=PSG7 PE=5 SV=2//0
XM_008019828.1	1.417	0.000182	0.001543	sp|Q6PK81|ZN773_HUMAN Zinc finger protein 773 OS=Homo sapiens GN=ZNF773 PE=2 SV=1//1.42591e-35
XM_008019831.1	3.5726	1.64E-77	5.22E-75	sp|Q96RU7|TRIB3_HUMAN Tribbles homolog 3 OS=Homo sapiens GN=TRIB3 PE=1 SV=2//0
XM_008019845.1	0.72698	0.006149	0.037589	sp|Q9BVS4|RIOK2_HUMAN Serine/threonine-protein kinase RIO2 OS=Homo sapiens GN=RIOK2 PE=1 SV=2//2.33716e-38
XM_008019846.1	2.4473	2.12E-49	3.05E-47	sp|Q96QB1|RHG07_HUMAN Rho GTPase-activating protein 7 OS=Homo sapiens GN=DLC1 PE=1 SV=4//0
XM_008019852.1	-2.1576	0.001138	0.008287	sp|Q9UKK3|PARP4_HUMAN Poly [ADP-ribose] polymerase 4 OS=Homo sapiens GN=PARP4 PE=1 SV=3//8.36177e-10
XM_008019890.1	-0.92168	7.14E-07	8.79E-06	sp|Q9C030|TRIM6_HUMAN Tripartite motif-containing protein 6 OS=Homo sapiens GN=TRIM6 PE=1 SV=1//0
XM_008019969.1	-0.49768	0.002417	0.016278	sp|Q9UHJ9|PGAP2_HUMAN Post-GPI attachment to proteins factor 2 OS=Homo sapiens GN=PGAP2 PE=1 SV=2//2.28781e-27
XM_008020015.1	-0.58188	5.69E-05	0.000527	sp|P15328|FOLR1_HUMAN Folate receptor alpha OS=Homo sapiens GN=FOLR1 PE=1 SV=3//4.9029e-151
XM_008020022.1	1.2512	5.63E-30	3.78E-28	sp|P41134|ID1_HUMAN DNA-binding protein inhibitor ID-1 OS=Homo sapiens GN=ID1 PE=1 SV=3//1.28359e-77
XM_008020025.1	-0.59899	8.85E-05	0.000795	sp|Q9NZE8|RM35_HUMAN 39S ribosomal protein L35, mitochondrial OS=Homo sapiens GN=MRPL35 PE=1 SV=3//7.20331e-117
XM_008020113.1	-0.41827	0.000441	0.003506	sp|Q96GC5|RM48_HUMAN 39S ribosomal protein L48, mitochondrial OS=Homo sapiens GN=MRPL48 PE=1 SV=2//2.75197e-117
XM_008020134.1	0.97958	0.001779	0.012382	sp|A6NK58|LIPT2_HUMAN Putative lipoyltransferase 2, mitochondrial OS=Homo sapiens GN=LIPT2 PE=3 SV=1//1.52052e-142
XM_008020140.1	0.79061	1.47E-08	2.19E-07	sp|Q15054|DPOD3_HUMAN DNA polymerase delta subunit 3 OS=Homo sapiens GN=POLD3 PE=1 SV=2//0
XM_008020153.1	1.3696	6.22E-40	6.13E-38	sp|Q8NCN4|RN169_HUMAN E3 ubiquitin-protein ligase RNF169 OS=Homo sapiens GN=RNF169 PE=1 SV=2//0
XM_008020170.1	-0.56317	2.22E-13	5.38E-12	sp|Q0Z8U2|RS3_PIG 40S ribosomal protein S3 OS=Sus scrofa GN=RPS3 PE=1 SV=1//1.63136e-175
XM_008020171.1	0.91027	4.10E-25	2.17E-23	sp|Q9NP50|FA60A_HUMAN Protein FAM60A OS=Homo sapiens GN=FAM60A PE=1 SV=1//1.31108e-132
XM_008020188.1	1.6962	3.06E-52	4.89E-50	sp|Q9P2Y5|UVRAG_HUMAN UV radiation resistance-associated gene protein OS=Homo sapiens GN=UVRAG PE=1 SV=1//0
XM_008020195.1	-1.0753	1.09E-06	1.32E-05	sp|O43638|FOXS1_HUMAN Forkhead box protein S1 OS=Homo sapiens GN=FOXS1 PE=2 SV=2//3.96415e-178
XM_008020214.1	1.0604	2.63E-06	2.99E-05	sp|Q9NUG6|PDRG1_HUMAN p53 and DNA damage-regulated protein 1 OS=Homo sapiens GN=PDRG1 PE=1 SV=2//4.35078e-67
XM_008020217.1	-1.1006	0.006599	0.040082	-//-
XM_008020218.1	-0.29022	0.005337	0.033211	sp|Q8WUA8|TSK_HUMAN Tsukushin OS=Homo sapiens GN=TSKU PE=2 SV=3//0
XM_008020259.1	0.46989	4.07E-08	5.76E-07	sp|Q96T23|RSF1_HUMAN Remodeling and spacing factor 1 OS=Homo sapiens GN=RSF1 PE=1 SV=2//0
XM_008020261.1	-1.927	6.82E-05	0.000625	sp|Q92748|THRSP_HUMAN Thyroid hormone-inducible hepatic protein OS=Homo sapiens GN=THRSP PE=1 SV=1//5.7529e-65
XM_008020262.1	-0.5912	8.18E-06	8.57E-05	sp|Q8SPI4|NDUC2_MACFA NADH dehydrogenase [ubiquinone] 1 subunit C2 OS=Macaca fascicularis GN=NDUFC2 PE=3 SV=1//2.52155e-70
XM_008020421.1	0.88025	1.26E-05	0.000128	sp|Q9H489|TSY26_HUMAN Putative testis-specific Y-encoded-like protein 3 OS=Homo sapiens GN=TSPY26P PE=5 SV=1//4.68601e-170
XM_008020433.1	1.1309	9.55E-37	8.53E-35	sp|Q9UPG8|PLAL2_HUMAN Zinc finger protein PLAGL2 OS=Homo sapiens GN=PLAGL2 PE=2 SV=1//0
XM_008020443.1	0.27556	0.004356	0.027687	sp|Q1WK23|PRS23_MACMU Serine protease 23 OS=Macaca mulatta GN=PRSS23 PE=2 SV=1//0
XM_008020444.1	-0.5137	0.000114	0.000999	sp|Q9ULV1|FZD4_HUMAN Frizzled-4 OS=Homo sapiens GN=FZD4 PE=1 SV=2//0

XM_008020456.1	-1.0493	6.16E-19	2.24E-17	sp|Q60HG6|CATC_MACFA Dipeptidyl peptidase 1 OS=Macaca fascicularis GN=CTSC PE=2 SV=1//0
XM_008020466.1	1.2268	0.000107	0.000943	sp|Q5R5C5|NOX4_PONAB NADPH oxidase 4 OS=Pongo abelii GN=NOX4 PE=2 SV=2//0
XM_008020478.1	-0.56681	0.00012	0.001049	sp|Q7Z2W9|RM21_HUMAN 39S ribosomal protein L21, mitochondrial OS=Homo sapiens GN=MRPL21 PE=1 SV=2//1.0649e-121
XM_008020490.1	0.64655	2.91E-05	0.000281	sp|Q4R7U2|CHRD1_MACFA Cysteine and histidine-rich domain-containing protein 1 OS=Macaca fascicularis GN=CHORDC1 PE=2 SV=1//1.66989e-14
XM_008020544.1	0.72849	0.000635	0.004888	sp|Q9NVC6|MED17_HUMAN Mediator of RNA polymerase II transcription subunit 17 OS=Homo sapiens GN=MED17 PE=1 SV=2//0
XM_008020579.1	1.2519	8.43E-06	8.82E-05	sp|Q659K9|FUT4_PANTR Alpha-(1,3)-fucosyltransferase 4 OS=Pan troglodytes GN=FUT4 PE=2 SV=2//0
XM_008020594.1	1.0597	1.15E-22	5.33E-21	sp|Q9BRL6|SRSF8_HUMAN Serine/arginine-rich splicing factor 8 OS=Homo sapiens GN=SRSF8 PE=1 SV=1//1.7711e-61
XM_008020597.1	-0.76843	0.002302	0.015624	sp|P58005|SESN3_HUMAN Sestrin-3 OS=Homo sapiens GN=SESN3 PE=2 SV=2//0
XM_008020604.1	0.58709	6.45E-06	6.87E-05	sp|Q5HYJ3|FA76B_HUMAN Protein FAM76B OS=Homo sapiens GN=FAM76B PE=1 SV=3//0
XM_008020653.1	1.5612	1.51E-07	2.00E-06	sp|Q13489|BIRC3_HUMAN Baculoviral IAP repeat-containing protein 3 OS=Homo sapiens GN=BIRC3 PE=1 SV=2//0
XM_008020656.1	0.72697	0.003094	0.020332	sp|P03956|MMP1_HUMAN Interstitial collagenase OS=Homo sapiens GN=MMP1 PE=1 SV=3//0
XM_008020707.1	1.1342	1.51E-25	8.13E-24	sp|P38935|SMBP2_HUMAN DNA-binding protein SMUBP-2 OS=Homo sapiens GN=IGHMBP2 PE=1 SV=3//0
XM_008020716.1	0.81963	1.87E-15	5.30E-14	sp|Q9NRN7|ADPPT_HUMAN L-aminoadipate-semialdehyde dehydrogenase-phosphopantetheinyl transferase OS=Homo sapiens GN=AASDHPPT PE=1 SV=2//0
XM_008020723.1	-0.69753	4.82E-06	5.27E-05	-//-
XM_008020745.1	0.57009	7.11E-08	9.74E-07	sp|Q93034|CUL5_HUMAN Cullin-5 OS=Homo sapiens GN=CUL5 PE=1 SV=4//0
XM_008020746.1	-0.53698	1.98E-07	2.58E-06	sp|P24752|THIL_HUMAN Acetyl-CoA acetyltransferase, mitochondrial OS=Homo sapiens GN=ACAT1 PE=1 SV=1//0
XM_008020754.1	0.61372	4.82E-10	8.53E-09	sp|Q14207|NPAT_HUMAN Protein NPAT OS=Homo sapiens GN=NPAT PE=1 SV=3//0
XM_008020770.1	1.1115	5.79E-24	2.91E-22	sp|Q13206|DDX10_HUMAN Probable ATP-dependent RNA helicase DDX10 OS=Homo sapiens GN=DDX10 PE=1 SV=2//0
XM_008020817.1	1.2233	1.36E-09	2.29E-08	sp|P30154|2AAB_HUMAN Serine/threonine-protein phosphatase 2A 65 kDa regulatory subunit A beta isoform OS=Homo sapiens GN=PPP2R1B PE=1 SV=3//8.14987e-15
XM_008020886.1	-0.84064	4.36E-09	6.95E-08	sp|P13591|NCAM1_HUMAN Neural cell adhesion molecule 1 OS=Homo sapiens GN=NCAM1 PE=1 SV=3//0
XM_008020996.1	-0.52721	0.000138	0.001192	sp|P56817|BACE1_HUMAN Beta-secretase 1 OS=Homo sapiens GN=BACE1 PE=1 SV=2//0
XM_008021023.1	-2.1364	4.83E-10	8.55E-09	sp|P54710|ATNG_HUMAN Sodium/potassium-transporting ATPase subunit gamma OS=Homo sapiens GN=FXYD2 PE=1 SV=3//4.22426e-28
XM_008021063.1	-0.72755	1.56E-05	0.000157	sp|O75964|ATP5L_HUMAN ATP synthase subunit g, mitochondrial OS=Homo sapiens GN=ATP5L PE=1 SV=3//8.50356e-68
XM_008021122.1	0.78317	8.01E-14	2.01E-12	sp|Q01094|E2F1_HUMAN Transcription factor E2F1 OS=Homo sapiens GN=E2F1 PE=1 SV=1//0
XM_008021129.1	-0.98075	1.21E-08	1.82E-07	sp|Q6Q311|RS25_SHEEP 40S ribosomal protein S25 OS=Ovis aries GN=RPS25 PE=2 SV=1//9.04604e-45
XM_008021190.1	0.67027	0.00012	0.001047	sp|P43121|MUC18_HUMAN Cell surface glycoprotein MUC18 OS=Homo sapiens GN=MCAM PE=1 SV=2//0
XM_008021193.1	-0.69437	2.01E-10	3.71E-09	sp|Q9BY78|RNF26_HUMAN RING finger protein 26 OS=Homo sapiens GN=RNF26 PE=2 SV=1//0
XM_008021205.1	2.4571	9.21E-08	1.25E-06	sp|O75604|UBP2_HUMAN Ubiquitin carboxyl-terminal hydrolase 2 OS=Homo sapiens GN=USP2 PE=1 SV=2//0
XM_008021206.1	-0.55494	7.72E-11	1.49E-09	sp|Q9H444|CHM4B_HUMAN Charged multivesicular body protein 4b OS=Homo sapiens GN=CHMP4B PE=1 SV=1//4.65068e-94
XM_008021209.1	-0.64402	0.000665	0.005104	sp|Q86UD1|OAF_HUMAN Out at first protein homolog OS=Homo sapiens GN=OAF PE=2 SV=1//2.21435e-165
XM_008021240.1	-1.9397	1.59E-09	2.66E-08	sp|Q92673|SORL_HUMAN Sortilin-related receptor OS=Homo sapiens GN=SORL1 PE=1 SV=2//0
XM_008021336.1	0.56078	0.000488	0.003849	sp|Q3YBR2|TBRG1_HUMAN Transforming growth factor beta regulator 1 OS=Homo sapiens GN=TBRG1 PE=1 SV=1//0
XM_008021390.1	-0.26836	0.001526	0.010826	sp|Q5RCE2|STT3A_PONAB Dolichyl-diphosphooligosaccharide--protein glycosyltransferase subunit STT3A OS=Pongo abelii GN=STT3A PE=2 SV=1//0
XM_008021399.1	-0.28584	0.001209	0.008753	sp|Q9NP97|DLRB1_HUMAN Dynein light chain roadblock-type 1 OS=Homo sapiens GN=DYNLRB1 PE=1 SV=3//4.89169e-62
XM_008021429.1	1.3441	0.000453	0.003591	sp|Q96CM3|RUSD4_HUMAN RNA pseudouridylate synthase domain-containing protein 4 OS=Homo sapiens GN=RPUSD4 PE=2 SV=1//0
XM_008021443.1	0.38221	0.004712	0.029753	sp|Q96C86|DCPS_HUMAN m7GpppX diphosphatase OS=Homo sapiens GN=DCPS PE=1 SV=2//0
XM_008021529.1	1.1285	0.005652	0.034945	sp|Q9Y5Y6|ST14_HUMAN Suppressor of tumorigenicity 14 protein OS=Homo sapiens GN=ST14 PE=1 SV=2//0

XM_008021532.1	2.4104	4.49E-95	1.93E-92	sp|Q8TE58|ATS15_HUMAN A disintegrin and metalloproteinase with thrombospondin motifs 15 OS=Homo sapiens GN=ADAMTS15 PE=2 SV=1//0
XM_008021535.1	0.3108	0.000912	0.006776	sp|Q92543|SNX19_HUMAN Sorting nexin-19 OS=Homo sapiens GN=SNX19 PE=1 SV=2//0
XM_008021574.1	-0.87331	1.06E-07	1.42E-06	sp|Q9BX67|JAM3_HUMAN Junctional adhesion molecule C OS=Homo sapiens GN=JAM3 PE=1 SV=1//0
XM_008021580.1	-0.73742	3.12E-07	3.98E-06	sp|Q9P016|THYN1_HUMAN Thymocyte nuclear protein 1 OS=Homo sapiens GN=THYN1 PE=1 SV=1//8.77226e-164
XM_008021608.1	0.70848	5.79E-06	6.23E-05	sp|Q9R283|TRPC2_RAT Short transient receptor potential channel 2 OS=Rattus norvegicus GN=Trpc2 PE=2 SV=2//4.54606e-78
XM_008021610.1	0.97235	3.25E-22	1.47E-20	sp|Q4AC94|C2CD3_HUMAN C2 domain-containing protein 3 OS=Homo sapiens GN=C2CD3 PE=1 SV=4//0
XM_008021611.1	1.0136	2.68E-12	5.87E-11	sp|Q96I59|SYNM_HUMAN Probable asparagine--tRNA ligase, mitochondrial OS=Homo sapiens GN=NARS2 PE=1 SV=3//0
XM_008021635.1	-0.43783	2.68E-06	3.04E-05	sp|Q5RDT5|SAP18_PONAB Histone deacetylase complex subunit SAP18 OS=Pongo abelii GN=SAP18 PE=2 SV=1//9.35496e-95
XM_008021636.1	2.1582	########	########	sp|Q9NRM7|LATS2_HUMAN Serine/threonine-protein kinase LATS2 OS=Homo sapiens GN=LATS2 PE=1 SV=2//0
XM_008021685.1	0.28067	0.000573	0.004447	sp|O94906|PRP6_HUMAN Pre-mRNA-processing factor 6 OS=Homo sapiens GN=PRPF6 PE=1 SV=1//0
XM_008021709.1	0.57137	5.78E-07	7.18E-06	sp|Q5RE34|RTEL1_PONAB Regulator of telomere elongation helicase 1 OS=Pongo abelii GN=RTEL1 PE=2 SV=1//0
XM_008021748.1	-2.6831	0.002643	0.017621	sp|Q9BXT8|RNF17_HUMAN RING finger protein 17 OS=Homo sapiens GN=RNF17 PE=1 SV=3//0
XM_008021752.1	0.31043	0.008294	0.048912	sp|O00268|TAF4_HUMAN Transcription initiation factor TFIID subunit 4 OS=Homo sapiens GN=TAF4 PE=1 SV=2//0
XM_008021792.1	1.4607	9.85E-55	1.68E-52	sp|O75317|UBP12_HUMAN Ubiquitin carboxyl-terminal hydrolase 12 OS=Homo sapiens GN=USP12 PE=1 SV=2//0
XM_008021796.1	0.72182	1.66E-12	3.71E-11	sp|Q6T310|RSLBA_HUMAN Ras-like protein family member 11A OS=Homo sapiens GN=RASL11A PE=2 SV=1//3.06489e-158
XM_008021797.1	1.0149	1.51E-24	7.76E-23	sp|Q9P2E3|ZNFX1_HUMAN NFX1-type zinc finger-containing protein 1 OS=Homo sapiens GN=ZNFX1 PE=2 SV=2//0
XM_008021802.1	1.2073	4.63E-23	2.22E-21	sp|Q8N448|LNX2_HUMAN Ligand of Numb protein X 2 OS=Homo sapiens GN=LNX2 PE=1 SV=1//0
XR_489608.1	4.0377	9.31E-05	0.000833	-//-
XR_489808.1	3.0564	0.000149	0.001284	-//-
XR_489809.1	1.697	2.17E-05	0.000213	-//-
XR_489828.1	0.46663	0.001192	0.008649	-//-
XR_489858.1	-0.72156	7.76E-05	0.000705	-//-
XR_489890.1	-1.3828	9.43E-09	1.44E-07	-//-
XR_490208.1	-0.46312	0.001653	0.011612	sp|P00390|GSHR_HUMAN Glutathione reductase, mitochondrial OS=Homo sapiens GN=GSR PE=1 SV=2//4.53879e-29
XR_490233.1	3.195	0.004869	0.030649	-//-
XR_490236.1	1.2321	1.49E-16	4.59E-15	-//-
XR_490301.1	-1.7585	0.001416	0.010111	-//-
XR_490344.1	0.67611	0.004249	0.027054	sp|Q14692|BMS1_HUMAN Ribosome biogenesis protein BMS1 homolog OS=Homo sapiens GN=BMS1 PE=1 SV=1//1.28965e-06
XR_490359.1	-0.66299	0.000625	0.004819	sp|Q5I0H3|SUMO1_RAT Small ubiquitin-related modifier 1 OS=Rattus norvegicus GN=Sumo1 PE=1 SV=1//1.46738e-52
XR_490377.1	-1.3687	8.79E-06	9.16E-05	-//-
XR_490385.1	-2.372	1.72E-06	2.01E-05	-//-
XR_490428.1	0.77064	0.000275	0.002264	sp|P16260|GDC_HUMAN Graves disease carrier protein OS=Homo sapiens GN=SLC25A16 PE=1 SV=3//1.90425e-28
XR_490441.1	-1.9352	6.20E-07	7.69E-06	-//-
XR_490451.1	-4.3933	8.39E-10	1.44E-08	-//-
XR_490492.1	4.0271	0.005272	0.032863	-//-
XR_490633.1	1.3305	0.001807	0.01255	-//-
XR_490662.1	-1.476	0.000155	0.001332	-//-
XR_490765.1	-0.39695	0.004128	0.026409	-//-
XR_490791.1	Inf	0.006816	0.041181	-//-
XR_490887.1	-0.68113	0.006371	0.038902	-//-
XR_490927.1	#NAME?	0.00613	0.037494	-//-
XR_490929.1	-1.8745	0.000315	0.002573	-//-
XR_490964.1	-0.49594	0.005155	0.032218	-//-

XR_491014.1	-1.5401	2.37E-07	3.07E-06	sp|Q9P1C3|YN010_HUMAN Putative uncharacterized protein PRO2829 OS=Homo sapiens GN=PRO2829 PE=5 SV=1//1.72198e-11
XR_491289.1	1.2306	0.001022	0.007532	-//-
XR_491361.1	4.9271	8.32E-15	2.27E-13	-//-
XR_491397.1	#NAME?	0.006004	0.036814	-//-
XR_491403.1	-0.71342	1.88E-09	3.12E-08	-//-
XR_491419.1	0.44843	0.000753	0.005703	-//-
XR_491516.1	-1.8665	3.47E-06	3.88E-05	-//-
XR_491526.1	-2.986	0.002229	0.015171	-//-
XR_491556.1	-3.2513	0.004813	0.030331	-//-
XR_491650.1	-2.8078	7.24E-05	0.000661	-//-
XR_491709.1	-1.1346	0.005151	0.032199	-//-
XR_491735.1	0.81182	0.000183	0.001549	-//-
XR_491795.1	0.93644	0.002376	0.016026	-//-
XR_491895.1	-1.3529	4.60E-05	0.000432	-//-
XR_491928.1	-0.965	0.000138	0.001193	-//-
XR_492053.1	-3.1817	0.007003	0.042104	-//-
XR_492136.1	-1.2016	1.89E-05	0.000187	-//-
XR_492144.1	1.2536	2.10E-12	4.65E-11	sp|O60729|CC14B_HUMAN Dual specificity protein phosphatase CDC14B OS=Homo sapiens GN=CDC14B PE=1 SV=1//0
XR_492273.1	2.5923	4.73E-05	0.000444	-//-
XR_492313.1	-2.0498	0.002551	0.017069	-//-
XR_492422.1	-2.139	2.63E-06	2.99E-05	-//-
XR_492457.1	2.3837	4.36E-05	0.00041	-//-
XR_492479.1	3.0947	5.40E-08	7.51E-07	-//-
XR_492612.1	2.0173	6.22E-06	6.67E-05	-//-
XR_492863.1	0.87116	1.26E-06	1.50E-05	sp|Q9BQQ3|GORS1_HUMAN Golgi reassembly-stacking protein 1 OS=Homo sapiens GN=GORASP1 PE=1 SV=3//0
XR_493050.1	-1.671	9.52E-05	0.000849	-//-
XR_493066.1	-0.5245	3.13E-05	0.000301	-//-
XR_493081.1	-1.1323	0.005594	0.034673	-//-
XR_493090.1	-0.54861	0.000633	0.004878	sp|P30484|1B46_HUMAN HLA class I histocompatibility antigen, B-46 alpha chain OS=Homo sapiens GN=HLA-B PE=1 SV=1//0
XR_493091.1	-0.5884	3.60E-12	7.80E-11	sp|P30381|1B03_GORGO Class I histocompatibility antigen, Gogo-B*0103 alpha chain OS=Gorilla gorilla gorilla PE=2 SV=1//5.18137e-165
XR_493140.1	0.78592	9.08E-05	0.000813	-//-
XR_493142.1	-0.80713	0.002382	0.016061	-//-
XR_493212.1	1.4986	0.000167	0.001426	-//-
XR_493223.1	4.406	0.001572	0.011114	-//-
XR_493329.1	0.50786	1.77E-11	3.61E-10	sp|Q9UBA6|G8_HUMAN Protein G8 OS=Homo sapiens GN=C6orf48 PE=4 SV=2//3.36519e-33
XR_493419.1	-1.3595	0.003248	0.021234	-//-
XR_493499.1	-1.9487	0.000324	0.002638	-//-
XR_493580.1	-1.5115	0.000406	0.003249	-//-
XR_493595.1	-1.9917	0.00018	0.001531	-//-
XR_493598.1	-1.9934	1.27E-06	1.51E-05	-//-
XR_493599.1	-1.9384	3.26E-05	0.000312	-//-
XR_493608.1	-1.2093	0.00323	0.021144	-//-
XR_493619.1	1.7738	1.62E-07	2.14E-06	-//-
XR_493690.1	2.7999	3.13E-11	6.28E-10	-//-
XR_493702.1	-3.4809	0.001663	0.011658	-//-
XR_493802.1	-1.1527	0.006561	0.039864	-//-
XR_493803.1	1.338	0.00017	0.00145	sp|Q9H4I8|SEHL2_HUMAN Serine hydrolase-like protein 2 OS=Homo sapiens GN=SERHL2 PE=2 SV=1//4.60949e-29
XR_493829.1	1.9987	3.91E-25	2.08E-23	-//-
XR_493830.1	-0.53875	1.05E-05	0.000109	-//-
XR_493872.1	-1.5734	0.000679	0.005198	-//-
XR_493882.1	-4.0101	0.004938	0.031002	-//-

XR_493883.1	-1.0096	2.48E-08	3.59E-07	-//-
XR_494025.1	-0.60145	3.44E-14	8.83E-13	sp|Q53CG2|COX7C_MACSL Cytochrome c oxidase subunit 7C, mitochondrial OS=Macaca silenus GN=COX7C PE=3 SV=1//3.74618e-36
XR_494062.1	-1.4093	0.00765	0.045405	-//-
XR_494063.1	1.7403	3.32E-07	4.23E-06	-//-
XR_494080.1	-4.0157	0.006996	0.042073	sp|Q96LT9|RBM40_HUMAN RNA-binding protein 40 OS=Homo sapiens GN=RNPC3 PE=1 SV=1//0
XR_494099.1	1.7083	0.000428	0.003409	-//-
XR_494325.1	-3.4302	7.95E-06	8.35E-05	-//-
XR_494401.1	3.0615	0.000199	0.001678	sp|P23246|SFPQ_HUMAN Splicing factor, proline- and glutamine-rich OS=Homo sapiens GN=SFPQ PE=1 SV=2//0
XR_494424.1	-0.6612	0.004771	0.030096	-//-
XR_494457.1	-2.5709	0.004897	0.030795	-//-
XR_494465.1	2.218	0.00034	0.002759	-//-
XR_494468.1	1.8464	6.12E-08	8.44E-07	sp|A2T715|SCND1_PANTR SCAN domain-containing protein 1 OS=Pan troglodytes GN=SCAND1 PE=3 SV=1//1.15969e-13
XR_494567.1	-0.81098	0.000282	0.002318	-//-
XR_494574.1	-1.0354	0.000121	0.001056	-//-
XR_494584.1	-2.1302	0.001982	0.013593	-//-
XR_494595.1	-1.4348	2.48E-11	5.00E-10	-//-
XR_494597.1	-2.5176	9.23E-06	9.59E-05	-//-
XR_494661.1	1.7086	1.53E-08	2.27E-07	-//-
XR_494680.1	Inf	0.006924	0.041711	-//-
XR_494760.1	-2.607	5.35E-22	2.37E-20	sp|P31269|HXA9_HUMAN Homeobox protein Hox-A9 OS=Homo sapiens GN=HOXA9 PE=1 SV=4//5.30004e-10
XR_494912.1	-0.94065	0.000202	0.001702	sp|Q6B7M7|COF1_SHEEP Cofilin-1 OS=Ovis aries GN=CFL1 PE=2 SV=3//6.72274e-37
XR_494925.1	2.3151	4.66E-14	1.19E-12	-//-
XR_494940.1	-0.82037	0.007007	0.042107	-//-
XR_494945.1	-1.3553	8.08E-06	8.48E-05	-//-
XR_494955.1	-1.5934	1.15E-05	0.000117	-//-
XR_494970.1	2.0517	0.001441	0.010277	-//-
XR_494992.1	-0.54257	0.001098	0.008042	sp|Q13148|TADBP_HUMAN TAR DNA-binding protein 43 OS=Homo sapiens GN=TARDBP PE=1 SV=1//0
XR_495012.1	-1.2652	0.001849	0.012796	-//-
XR_495029.1	1.1364	0.0056	0.034693	-//-
XR_495035.1	-1.6875	0.00037	0.002984	-//-
XR_495067.1	0.7604	0.005815	0.035825	-//-
XR_495071.1	0.77862	6.28E-06	6.73E-05	-//-
XR_495129.1	1.9142	0.000732	0.005561	-//-
XR_495176.1	-2.042	0.007825	0.046384	-//-
XR_495198.1	-4.086	0.005042	0.031582	-//-
XR_495201.1	-1.3053	2.50E-16	7.58E-15	-//-
XR_495206.1	0.40185	0.002354	0.015915	sp|O95376|ARI2_HUMAN E3 ubiquitin-protein ligase ARIH2 OS=Homo sapiens GN=ARIH2 PE=1 SV=1//0
XR_495236.1	-2.2174	0.005813	0.035823	-//-
XR_495351.1	2.9143	3.87E-11	7.69E-10	-//-
XR_495375.1	-1.0036	0.006727	0.040722	sp|Q9HBG6|IF122_HUMAN Intraflagellar transport protein 122 homolog OS=Homo sapiens GN=IFT122 PE=1 SV=2//3.22494e-10
XR_495427.1	-0.46713	2.51E-10	4.58E-09	sp|Q5R9Y1|FSTL1_PONAB Follistatin-related protein 1 OS=Pongo abelii GN=FSTL1 PE=2 SV=2//0
XR_495545.1	1.2149	0.006666	0.040414	-//-
XR_495574.1	3.9312	5.03E-12	1.07E-10	-//-
XR_495575.1	3.9403	2.19E-50	3.28E-48	-//-
XR_495599.1	-0.90414	2.32E-06	2.65E-05	sp|Q02878|RL6_HUMAN 60S ribosomal protein L6 OS=Homo sapiens GN=RPL6 PE=1 SV=3//9.9556e-149
XR_495691.1	-0.6529	0.002375	0.016024	sp|Q5RFN6|CNN2_PONAB Calponin-2 OS=Pongo abelii GN=CNN2 PE=2 SV=3//1.10489e-144
XR_495872.1	2.1975	6.05E-09	9.47E-08	-//-
XR_495894.1	2.9759	0.002878	0.019007	-//-

XR_495903.1	3.3234	1.26E-10	2.38E-09	-//-
XR_495915.1	0.62263	8.79E-05	0.00079	sp|Q9Y5J7|TIM9_HUMAN Mitochondrial import inner membrane translocase subunit Tim9 OS=Homo sapiens GN=TIMM9 PE=1 SV=1//9.5675e-48
XR_495919.1	6.1315	6.27E-24	3.14E-22	-//-
XR_495921.1	3.5824	0.00106	0.007778	-//-
XR_495991.1	0.7067	0.002341	0.015838	-//-
XR_496002.1	-4.0144	0.004912	0.030875	-//-
XR_496086.1	-0.64681	0.004362	0.027716	-//-
XR_496326.1	-1.745	5.74E-06	6.19E-05	sp|Q8IVF2|AHNK2_HUMAN Protein AHNAK2 OS=Homo sapiens GN=AHNAK2 PE=1 SV=2//1.28869e-24
XR_496353.1	-2.4908	0.00032	0.002613	-//-
XR_496357.1	-2.3977	0.005634	0.034847	-//-
XR_496404.1	0.76567	0.00143	0.010206	-//-
XR_496477.1	1.5234	0.001056	0.007754	-//-
XR_496716.1	-1.7323	2.45E-06	2.79E-05	-//-
XR_496796.1	-2.2042	7.86E-05	0.000713	-//-
XR_496912.1	1.9331	1.42E-11	2.93E-10	-//-
XR_497060.1	-0.54594	2.52E-05	0.000246	sp|Q5RDV1|YX011_PONAB Putative uncharacterized protein LOC550643 homolog OS=Pongo abelii PE=5 SV=1//2.89625e-24
XR_497062.1	-4.1551	0.002324	0.01573	-//-
XR_497065.1	0.37814	6.81E-05	0.000625	sp|Q6NVR8|LS14A_XENTR Protein LSM14 homolog A OS=Xenopus tropicalis GN=lsm14a PE=2 SV=1//1.90584e-120
XR_497085.1	-1.4005	1.98E-12	4.38E-11	-//-
XR_497098.1	#NAME?	0.001611	0.011358	-//-
XR_497167.1	-0.83552	0.000139	0.001204	sp|P63219|GBG5_RAT Guanine nucleotide-binding protein G(I)/G(S)/G(O) subunit gamma-5 OS=Rattus norvegicus GN=Gng5 PE=3 SV=1//7.51283e-30
XR_497239.1	-1.4903	8.91E-05	0.000799	-//-
XR_497401.1	Inf	0.000115	0.001007	-//-
XR_497431.1	1.0108	0.003093	0.020332	sp|Q9UL33|TPC2L_HUMAN Trafficking protein particle complex subunit 2-like protein OS=Homo sapiens GN=TRAPPC2L PE=1 SV=1//1.01181e-96
XR_497432.1	-1.2246	0.00751	0.044676	-//-
XR_497445.1	0.74847	2.96E-05	0.000286	sp|P21733|YCR2_BACTK Uncharacterized 29.1 kDa protein in cryB1 5'region OS=Bacillus thuringiensis subsp. kurstaki PE=4 SV=1//1.3224e-08
XR_497446.1	-1.1277	0.001375	0.009837	-//-
XR_497458.1	1.2004	1.69E-06	1.97E-05	-//-
XR_497472.1	-3.0511	0.001547	0.010959	-//-
XR_497500.1	2.297	########	########	-//-
XR_497626.1	-3.787	3.38E-05	0.000324	-//-
XR_497631.1	-1.715	3.73E-05	0.000355	sp|Q2M3W8|ZN181_HUMAN Zinc finger protein 181 OS=Homo sapiens GN=ZNF181 PE=2 SV=1//2.27938e-10
XR_497637.1	-1.1938	1.29E-23	6.31E-22	sp|Q53CG4|CX6B1_MACMU Cytochrome c oxidase subunit 6B1 OS=Macaca mulatta GN=COX6B1 PE=3 SV=3//2.38647e-31
XR_497638.1	-1.6705	5.47E-14	1.39E-12	-//-
XR_497682.1	1.2809	3.87E-06	4.29E-05	sp|Q96KS0|EGLN2_HUMAN Egl nine homolog 2 OS=Homo sapiens GN=EGLN2 PE=1 SV=1//0
XR_497708.1	1.4087	9.94E-08	1.34E-06	-//-
XR_497764.1	-2.9058	0.000123	0.001074	-//-
XR_497775.1	0.95419	0.000414	0.003309	-//-
XR_497807.1	3.8877	1.85E-54	3.14E-52	sp|Q8N5Q1|F71E2_HUMAN Protein FAM71E2 OS=Homo sapiens GN=FAM71E2 PE=2 SV=3//1.36009e-10
XR_497810.1	-0.59388	0.000806	0.006059	-//-
XR_497895.1	-2.0226	4.09E-05	0.000386	-//-
XR_497938.1	2.0353	6.10E-20	2.37E-18	-//-
XR_498076.1	1.4804	1.40E-08	2.09E-07	sp|Q8NDZ0|BEND2_HUMAN BEN domain-containing protein 2 OS=Homo sapiens GN=BEND2 PE=2 SV=2//6.17039e-07
XR_498129.1	Inf	0.000116	0.001017	-//-
XR_498144.1	0.98685	3.28E-05	0.000314	sp|Q9H082|RB33B_HUMAN Ras-related protein Rab-33B OS=Homo sapiens GN=RAB33B PE=1 SV=1//6.19563e-140
XR_498184.1	Inf	0.002293	0.015581	-//-
XR_498197.1	2.9271	8.34E-05	0.000754	-//-

XR_498354.1	-0.75916	1.24E-05	0.000126	sp|Q5RAQ8|RS24_PONAB 40S ribosomal protein S24 OS=Pongo abelii GN=RPS24 PE=2 SV=1//3.88828e-63
XR_498369.1	3.9695	0.000103	0.000916	sp|P04211|LV001_HUMAN Ig lambda chain V region 4A OS=Homo sapiens PE=4 SV=1//1.68263e-21
XR_498372.1	-1.1558	1.01E-05	0.000104	-//-
XR_498468.1	-2.7965	0.000322	0.002621	-//-
XR_498593.1	0.47931	1.10E-05	0.000113	-//-
XR_498603.1	2.0637	4.57E-05	0.00043	-//-
XR_498645.1	-3.3182	0.004793	0.030218	-//-
XR_498676.1	2.6451	3.08E-14	7.93E-13	-//-
XR_498705.1	1.5574	2.06E-05	0.000204	-//-
XR_498712.1	0.81624	7.25E-06	7.67E-05	-//-
XR_498757.1	1.2488	8.20E-31	5.66E-29	sp|Q96KN1|FA84B_HUMAN Protein FAM84B OS=Homo sapiens GN=FAM84B PE=1 SV=1//0
XR_498836.1	-2.3521	0.000253	0.002098	-//-
XR_498847.1	-0.68035	0.003872	0.024926	-//-
XR_498872.1	-1.7673	0.005676	0.035048	-//-
XR_498882.1	-1.3862	0.000336	0.002732	-//-
XR_498883.1	-1.3182	3.60E-05	0.000343	-//-
XR_498886.1	-2.8816	1.27E-06	1.51E-05	-//-
XR_498887.1	-3.8516	0.000123	0.001073	-//-
XR_498910.1	0.71074	0.000283	0.002322	sp|Q8N769|CN178_HUMAN Uncharacterized protein C14orf178 OS=Homo sapiens GN=C14orf178 PE=2 SV=1//4.92885e-08
XR_498922.1	2.2158	0.000341	0.002761	-//-
XR_498947.1	0.5987	0.00125	0.009022	sp|Q5R7C9|AK1C3_PONAB Aldo-keto reductase family 1 member C3 homolog OS=Pongo abelii GN=AKR1C3 PE=2 SV=1//9.14154e-15
XR_498956.1	1.0497	0.000603	0.004664	-//-
XR_499065.1	2.0465	6.24E-18	2.14E-16	sp|Q3KNS1|PTHD3_HUMAN Patched domain-containing protein 3 OS=Homo sapiens GN=PTCHD3 PE=1 SV=3//7.77158e-09
XR_499151.1	#NAME?	0.00012	0.001051	sp|Q4R7G7|CCD65_MACFA Coiled-coil domain-containing protein 65 OS=Macaca fascicularis GN=CCDC65 PE=2 SV=1//9.92266e-80
XR_499184.1	-1.4317	3.79E-13	8.99E-12	-//-
XR_499188.1	-1.5594	0.007308	0.043699	-//-
XR_499201.1	0.61161	3.75E-05	0.000356	-//-
XR_499205.1	-1.8422	0.000663	0.00509	sp|P30742|RS26_CRICR 40S ribosomal protein S26 OS=Cricetus cricetus GN=RPS26 PE=3 SV=3//4.29645e-12
XR_499211.1	-1.45	4.55E-06	4.99E-05	-//-
XR_499226.1	-0.73441	0.002315	0.015691	-//-
XR_499257.1	1.094	0.000838	0.006277	sp|Q8TC05|MDM1_HUMAN Nuclear protein MDM1 OS=Homo sapiens GN=MDM1 PE=1 SV=2//5.82111e-09
XR_499269.1	-1.2193	5.50E-06	5.96E-05	-//-
XR_499328.1	-1.9607	2.42E-07	3.13E-06	-//-
XR_499378.1	0.68138	0.003799	0.024502	-//-
XR_499428.1	-0.92064	8.41E-05	0.00076	sp|Q6QMZ4|RL6_CHILA 60S ribosomal protein L6 OS=Chinchilla lanigera GN=RPL6 PE=2 SV=3//2.76693e-30
XR_499460.1	-1.7153	0.00103	0.007583	-//-
XR_499465.1	-0.49273	0.000727	0.00553	-//-
XR_499476.1	-1.6026	0.002624	0.017506	sp|P63170|DYL1_RAT Dynein light chain 1, cytoplasmic OS=Rattus norvegicus GN=Dynll1 PE=1 SV=1//2.11548e-45
XR_499482.1	-2.0033	1.61E-33	1.26E-31	-//-
XR_499483.1	0.655	0.004058	0.026012	-//-
XR_499490.1	0.81234	9.98E-05	0.000887	sp|Q5R8J6|RSRC2_PONAB Arginine/serine-rich coiled-coil protein 2 OS=Pongo abelii GN=RSRC2 PE=2 SV=1//1.74315e-56
XR_499606.1	-0.48291	0.002913	0.019213	-//-
XR_499632.1	-0.466	0.001273	0.009172	-//-
XR_499644.1	-0.8053	2.47E-05	0.000242	sp|Q96BR6|ZN669_HUMAN Zinc finger protein 669 OS=Homo sapiens GN=ZNF669 PE=2 SV=2//2.28668e-26
XR_499741.1	3.9487	0.000103	0.000912	sp|Q4R4D3|RL10L_MACFA 60S ribosomal protein L10-like OS=Macaca fascicularis GN=RPL10L PE=2 SV=1//5.61636e-34
XR_499771.1	-0.45327	2.16E-05	0.000212	sp|P05787|K2C8_HUMAN Keratin, type II cytoskeletal 8 OS=Homo sapiens GN=KRT8 PE=1 SV=7//9.00574e-09

XR_499795.1	1.5917	0.001506	0.010692	sp|Q9Y2E4|DIP2C_HUMAN Disco-interacting protein 2 homolog C OS=Homo sapiens GN=DIP2C PE=1 SV=2//1.03343e-14
XR_499941.1	-0.90072	0.000651	0.005009	-//-
XR_499947.1	-1.0251	0.001313	0.009419	sp|O43491|E41L2_HUMAN Band 4.1-like protein 2 OS=Homo sapiens GN=EPB41L2 PE=1 SV=1//0
XR_500192.1	1.2852	3.37E-09	5.44E-08	-//-
XR_500275.1	-2.0931	0.007632	0.045337	-//-
XR_500284.1	-3.3268	5.22E-26	2.87E-24	-//-
XR_500380.1	0.64094	1.01E-08	1.54E-07	-//-
XR_500381.1	2.2388	1.60E-09	2.67E-08	-//-
XR_500416.1	2.3795	0.001906	0.013136	sp|P18621|RL17_HUMAN 60S ribosomal protein L17 OS=Homo sapiens GN=RPL17 PE=1 SV=3//6.57569e-121
XR_500521.1	1.389	0.000188	0.001592	-//-
XR_500522.1	0.83954	0.005282	0.032917	-//-
XR_500594.1	-2.4326	0.004108	0.026304	-//-
XR_500607.1	4.6124	0.005629	0.034838	-//-
XR_500653.1	-3.2457	0.000537	0.004193	-//-
XR_500666.1	-2.3713	5.17E-06	5.62E-05	-//-
XR_500694.1	-1.1739	0.000438	0.003486	-//-
XR_500698.1	-1.292	0.00011	0.000966	sp|Q6AY62|NEUFC_RAT Neuferricin OS=Rattus norvegicus GN=Cyb5d2 PE=2 SV=1//7.44581e-07
XR_500732.1	4.7206	0.000761	0.005756	-//-
XR_500765.1	1.6069	5.28E-40	5.25E-38	sp|Q8N1F1|C1AS1_HUMAN Putative uncharacterized protein LRRC75A-AS1, mitochondrial OS=Homo sapiens GN=LRRC75A-AS1 PE=5 SV=1//1.10097e-06
XR_500885.1	-1.2061	1.39E-16	4.32E-15	-//-
XR_500897.1	-3.3007	3.15E-05	0.000302	sp|Q0P5P2|CQ067_HUMAN Uncharacterized protein C17orf67 OS=Homo sapiens GN=C17orf67 PE=1 SV=2//3.67964e-06
XR_500941.1	2.2211	3.43E-05	0.000328	-//-
XR_500945.1	-1.2585	0.000254	0.002105	-//-
XR_500962.1	-1.3918	0.002207	0.015041	-//-
XR_501053.1	-3.0024	0.002178	0.014862	sp|Q8N2A0|CX062_HUMAN Putative uncharacterized protein encoded by LINC00269 OS=Homo sapiens GN=LINC00269 PE=5 SV=1//4.53457e-23
XR_501063.1	-1.8543	6.37E-12	1.35E-10	-//-
XR_501074.1	-3.7462	7.30E-06	7.71E-05	sp|Q14596|NBR1_HUMAN Next to BRCA1 gene 1 protein OS=Homo sapiens GN=NBR1 PE=1 SV=3//1.34878e-14
XR_501092.1	-0.4456	5.33E-09	8.42E-08	sp|Q01628|IFM3_HUMAN Interferon-induced transmembrane protein 3 OS=Homo sapiens GN=IFITM3 PE=1 SV=2//1.75246e-77
XR_501097.1	-1.2855	1.59E-05	0.000159	-//-
XR_501111.1	-3.1266	0.00106	0.007778	-//-
XR_501123.1	-0.82281	0.006619	0.040171	-//-
XR_501141.1	-2.3234	2.05E-13	4.98E-12	-//-
XR_501206.1	-2.5674	0.005008	0.031379	-//-
XR_501235.1	-1.1881	5.22E-10	9.19E-09	-//-
XR_501333.1	0.79087	0.003482	0.022606	-//-
XR_501461.1	-3.0208	0.004948	0.031039	-//-
XR_501478.1	-1.2882	0.001964	0.013488	sp|Q8N769|CN178_HUMAN Uncharacterized protein C14orf178 OS=Homo sapiens GN=C14orf178 PE=2 SV=1//3.28864e-07
XR_501479.1	-2.7435	6.34E-06	6.78E-05	-//-
XR_501484.1	1.2987	2.55E-09	4.18E-08	sp|O18973|RABX5_BOVIN Rab5 GDP/GTP exchange factor OS=Bos taurus GN=RABGEF1 PE=1 SV=1//0
XR_501514.1	3.0591	2.91E-05	0.000282	-//-
XR_501532.1	1.2966	1.53E-09	2.56E-08	-//-
XR_501703.1	-0.83759	1.88E-05	0.000186	sp|P04406|G3P_HUMAN Glyceraldehyde-3-phosphate dehydrogenase OS=Homo sapiens GN=GAPDH PE=1 SV=3//0
XR_501746.1	-0.93148	1.92E-09	3.17E-08	sp|O00244|ATOX1_HUMAN Copper transport protein ATOX1 OS=Homo sapiens GN=ATOX1 PE=1 SV=1//8.546e-29
XR_501861.1	-1.781	3.88E-05	0.000368	-//-
XR_501935.1	-1.0891	0.00157	0.011104	-//-
XR_501964.1	0.86443	3.15E-09	5.12E-08	sp|O15014|ZN609_HUMAN Zinc finger protein 609 OS=Homo sapiens GN=ZNF609 PE=1 SV=2//2.57385e-118
XR_501976.1	1.2782	4.34E-08	6.09E-07	-//-

XR_502168.1	-1.442	0.002504	0.016766	sp|P63174|RL38_RAT 60S ribosomal protein L38 OS=Rattus norvegicus GN=Rpl38 PE=1 SV=2//3.06947e-21
XR_502194.1	-4.6435	0.000138	0.001192	-//-
XR_502253.1	-0.69708	0.002356	0.015922	-//-
XR_502309.1	2.617	1.74E-15	4.95E-14	-//-
XR_502409.1	-1.2824	3.11E-06	3.50E-05	-//-
XR_502429.1	-0.77167	4.95E-10	8.74E-09	sp|P29314|RS9_RAT 40S ribosomal protein S9 OS=Rattus norvegicus GN=Rps9 PE=1 SV=4//2.02661e-113
XR_502435.1	-2.5967	0.001544	0.010945	-//-
XR_502451.1	-1.7172	0.00036	0.00291	-//-
XR_502595.1	-0.8595	0.00731	0.043699	-//-
XR_502597.1	0.46063	4.07E-06	4.49E-05	sp|Q4R766|ABD12_MACFA Monoacylglycerol lipase ABHD12 OS=Macaca fascicularis GN=ABHD12 PE=2 SV=1//2.59663e-44
XR_502599.1	1.6106	6.30E-05	0.000581	sp|P81126|SNAB_BOVIN Beta-soluble NSF attachment protein OS=Bos taurus GN=NAPB PE=1 SV=1//6.39774e-09
XR_502601.1	0.81075	0.000425	0.003388	sp|Q9BVI0|PHF20_HUMAN PHD finger protein 20 OS=Homo sapiens GN=PHF20 PE=1 SV=2//2.3431e-54
XR_502603.1	0.80978	5.51E-20	2.15E-18	-//-
XR_502632.1	-2.4226	6.11E-10	1.07E-08	-//-
XR_502633.1	-1.9837	0.003385	0.02208	-//-
XR_502638.1	-4.3617	0.003596	0.023254	-//-
XR_502744.1	-1.0257	0.000406	0.003249	-//-
XR_502749.1	2.2796	1.05E-13	2.60E-12	-//-
XR_502777.1	-0.81036	0.000525	0.004107	sp|Q5R8H3|BAP31_PONAB B-cell receptor-associated protein 31 OS=Pongo abelii GN=BCAP31 PE=2 SV=3//1.02445e-13
XR_502976.1	2.6568	0.004563	0.028869	sp|Q6ZU15|SEP14_HUMAN Septin-14 OS=Homo sapiens GN=SEPT14 PE=1 SV=2//8.06143e-18
XR_503030.1	0.93098	1.98E-06	2.28E-05	sp|P28749|RBL1_HUMAN Retinoblastoma-like protein 1 OS=Homo sapiens GN=RBL1 PE=1 SV=3//2.53615e-43
XR_503046.1	4.6187	0.000454	0.0036	sp|Q9UQ74|PSG8_HUMAN Pregnancy-specific beta-1-glycoprotein 8 OS=Homo sapiens GN=PSG8 PE=2 SV=2//2.76019e-35
XR_503101.1	-3.2847	0.004995	0.031311	-//-
XR_503150.1	1.7068	0.000715	0.005441	-//-
XR_503260.1	#NAME?	0.002335	0.0158	-//-
XR_503367.1	-1.8584	8.84E-05	0.000795	-//-
XR_503569.1	-1.0463	2.70E-21	1.14E-19	sp|P04406|G3P_HUMAN Glyceraldehyde-3-phosphate dehydrogenase OS=Homo sapiens GN=GAPDH PE=1 SV=3//0

Table S3 The difference of the genome and encoding proteins between the PEDV 85-7 and variant 85-7^{C40} strain.

	Gene			Protein		
	85-7 strain	85-7^{C40} strain	Location [1]	85-7 strain	85-7^{C40} strain	Location [2]
**Nsp16**	G	A	20139	E	K	2518
**S**	A	G	23303	R	G	895
	T	C	24530	F	L	1304
	G	C	24572	V	L	1318
**E**	TACT…CTTT[3]	15nt deleted	25434-25448	LWLFV	——[4]	16-20
	T	C	25460	L	P	25

Note: 1 represents the location in the whole genome, 2 represents the location in the corresponding coding protein,

3 represents the 46-60 nt in the E gene, 4 represents the corresponding amino acids were deleted.

Table S4 Primers used in this study

PCR or RT-qPCR	Forward (F) or reverse (R) primers	Sequence (5' to 3')	Description
Primers for recombinant vector construction			
N1-IFIT2	F	ctaccggactcagatctcgagATGAGTGAGAACAATAAGAATTCC	XhoI
	R	cggtggatcccgggcccgcggTTCCCCATTCCAGCTTGATGC	SacII
N1-IFIT3	F	ctaccggactcagatctcgagATGAGTGAGGTCACCAAGAATTCC	XhoI
	R	cggtggatcccgggcccgcggCTCTGAGTTAGAGACGAGCTCTCTAG	SacII
N1-ISG15	F	ctaccggactcagatctcgagATGAGCTGGGACCTGAAGGTG	XhoI
	R	cggtggatcccgggcccgcggGCTCTGCCCGCCAGGCTCTGTG	SacII
N1-IFI16	F	ctaccggactcagatctcgagATGGGAAAAAAATACAAGAACAT	XhoI
	R	cggtggatcccgggcccgcggGAAGAAAAAGCCTGGTGAAGT	SacII
N1-OASL	F	ctaccggactcagatctcgagATGGCACTGCTGCAGGAACTGT	XhoI
	R	cggtggatcccgggcccgcggACTGGATGGAAACAGAGCCTC	SacII
N1-USP18	F	ctaccggactcagatctcgagATGATCCGGGTGAAGGACT	XhoI
	R	cggtggatcccgggcccgcggGCATTCCATCTTCATGTAAACCAG	SacII
N1-RSAD2	F	ctaccggactcagatctcgagATGTGGGTACTCACGCCTGCT	XhoI
	R	cggtggatcccgggcccgcggCCAATCCAGCTTCAGATCAG	SacII
N1-STAT2	F	ctaccggactcagatctcgagATGGCACAGTGGGAAATGCT	XhoI
	R	cggtggatcccgggcccgcggGAAGTCAGAAGGCATCAAAGGT	SacII
N1-IFI35	F	ctaccggactcagatctcgagATGTCAGCCCCGCTGAATGCC	XhoI
	R	cggtggatcccgggcccgcggCCCTGACTCAGAGGTGAAGACTG	SacII

N1-TRIM16	F	ctaccggactcagatctcgagATGGCTGAATTGGATCTGATGG	XhoI
	R	cggtggatcccgggcccgcggGGGAATAGTCTCCACCAAGGATG	SacII
N1-IFI44	F	ctaccggactcagatctcgagATGGCAGTGACAACTCATT	XhoI
	R	cggtggatcccgggcccgcggATTTTCTTCCCTGTGCACACT	SacII
Primers for RT-qPCR			
IFIT2	F	CTGGTCACCTGGGGAAACTA	This study
	R	GAGCCTTCTCAAAGCACACC	
IFIT3	F	AGGAAGGGTGGACACAACTG	This study
	R	GAGCCTTTTCCAAGGCTTCT	
ISG15	F	CTGGGACCTGAAGGTGAAGA	This study
	R	TCAGAGGTTCATCGCACTTG	
IFI16	F	TTTGTGAATGGGGTGTTTGA	This study
	R	TTTTTCCTGGCCTTGATGAC	
OASL	F	AAAGAGAGGCCCATCATCCT	This study
	R	ATCTGGGTAACCCCTCTGCT	
USP18	F	CAGACCCTGACAATCCACCT	This study
	R	ATTTTCCATCCACAGCGTTC	
RSAD2	F	TCTCTGTGGAGGAGCCTTGT	This study
	R	TGGCTGTGTGGAAACAGAAG	
STAT2	F	CCTGTAATGGAGCCCACACT	This study
	R	GTAAGCCTCATCCACGGTGT	
IFI35	F	GAGGGTGTTGGTCAGTGGAT	This study

	R	GCTGCAACCTGATCTCAACA	
TRIM16	F	AGACTTGGAGCGGAAACTCA	This study
	R	TTCTTCTCCATCTCGGCACT	
IFI44	F	TTCCAAGGGCATGTAACACA	This study
	R	CACAGGCCACCTTCTTTCTC	
IFN-β	F	CGGCTCTTTCCATGAGCTAC	This study
	R	CGGCTGCTTAATTTCCTCCTCAG	
β-Actin	F	CGGGAAATCGTGCGTGAC	This study
	R	ATGCCCAGGAGGAAGGTTG	
PEDV Genome	F	TTTTGCTGTCGTCTTTC	This study
	R	AAGTGATGTAATGGTCGC	
